# AS-CMC: a pan-cancer database of alternative splicing for molecular classification of cancer

**DOI:** 10.1038/s41598-022-25584-6

**Published:** 2022-12-06

**Authors:** Jiyeon Park, Jin-Ok Lee, Minho Lee, Yeun-Jun Chung

**Affiliations:** 1 Precision Medicine Research Center, College of Medicine, The Catholic University of Korea, 222 Banpo-daero, Seocho-Gu, Seoul, 06591 Republic of Korea; 2 Integrated Research Center for Genome Polymorphism, The Catholic University of Korea, Seoul, Republic of Korea; 3 Department of Biomedicine and Health Sciences, Graduate School, The Catholic University of Korea, Seoul, Republic of Korea; 4 Department of Life Science, Dongguk University-Seoul, Goyang, 10326 Republic of Korea; 5 Department of Microbiology, College of Medicine, The Catholic University of Korea, Seoul, Republic of Korea

**Keywords:** Bioinformatics, Genomic analysis

## Abstract

Alternative splicing (AS) is a post-transcriptional regulation that leads to the complexity of the transcriptome. Despite the growing importance of AS in cancer research, the role of AS has not been systematically studied, especially in understanding cancer molecular classification. Herein, we analyzed the molecular subtype-specific regulation of AS using The Cancer Genome Atlas data and constructed a web-based database, named Alternative Splicing for Cancer Molecular Classification (AS-CMC). Our system harbors three analysis modules for exploring subtype-specific AS events, evaluating their phenotype association, and performing pan-cancer comparison. The number of subtype-specific AS events was found to be diverse across cancer types, and some differentially regulated AS events were recurrently found in multiple cancer types. We analyzed a subtype-specific AS in exon 11 of mitogen-activated protein kinase kinase 7 (*MAP3K7*) as an example of a pan-cancer AS biomarker. This AS marker showed significant association with the survival of patients with stomach adenocarcinoma. Our analysis revealed AS as an important determinant for cancer molecular classification. AS-CMC is the first web-based resource that provides a comprehensive tool to explore the biological implications of AS events, facilitating the discovery of novel AS biomarkers.

## Introduction

Integrated clustering of The Cancer Genome Atlas (TCGA) has identified distinct molecular subtypes of diverse human cancers^1^. The molecular subtypes are known to be associated with distinct drug responses and prognosis of cancers^2–4^. Therefore, precise molecular classification is important for better understanding of tumorigenesis as well as improving treatment outcomes for patients with cancer. The molecular subtypes have been mainly established based on DNA methylation, copy number alteration, mRNA, and microRNA genomics data. However, the role of post-transcriptional regulation in these molecular classifications has not been extensively studied due to the technical constraints encountered during the analysis and interpretation of post-transcriptional processes.

Alternative splicing (AS) is an important post-transcriptional process that generates RNA isoforms, leading to transcriptome diversity. AS refers to alternative usage of exons and introns, thus consequently altering the protein structure or abundance^5^. The role of AS in organ development and tumorigenesis has been increasingly realized with the recent advances in RNA-sequencing^6,7^. Previous AS-related studies in cancer have identified cancer-specific and clinically relevant AS events^8–10^. Indeed, a number of AS events have been reported to be associated with the prognosis of diverse cancers^11–14^, suggesting their clinical implications. Recently, cancer-specific AS events have emerged as a source of neoantigens, thus raising the possibility of using AS in clinical settings^15,16^. Studies have shown that pharmacological splicing modulation can generate neoantigens, which can be effectively employed in immunotherapy^17,18^.

With the growing interest in the role of AS in cancer, several splicing-related databases have been developed using TCGA RNA-sequencing data. TCGASpliceSeq has provided percent spliced in (PSI) values for AS events, which has allowed users to compare changes in AS across cancer types^19^. ExonSkipDB database can be used to understand AS events in the context of mutation and epigenetic background^20^. The recently updated OncoSplicing database focuses on identifying clinically relevant AS events in cancer development by conducting survival and differential analyses^21^. ASPN, a splicing-derived neoepitope database, has compiled a list of potential AS-derived neoepitopes found in 16 types of cancers^22^. However, these databases have focused on identifying cancer-specific alterations in AS, and the role of AS in cancer classification has not been systematically explored to date.

To expand the molecular determinants for cancer classification, herein we have analyzed differentially regulated AS events in molecular subtypes of cancer using TCGA data. To promote the discovery of AS events as biomarkers, we have developed a pan-cancer database named AS-CMC (Alternative Splicing for Cancer Molecular Classification). This is a novel database for allowing users to browse subtype-specific changes in AS along with phenotypic associations for each cancer type as well as compare the regulation pattern across diverse cancer types. As an example, we have provided a potential AS event as a pan-cancer biomarker that can distinguish among molecular subtypes.

## Results

### AS-CMC analysis modules

**Figure 1:**
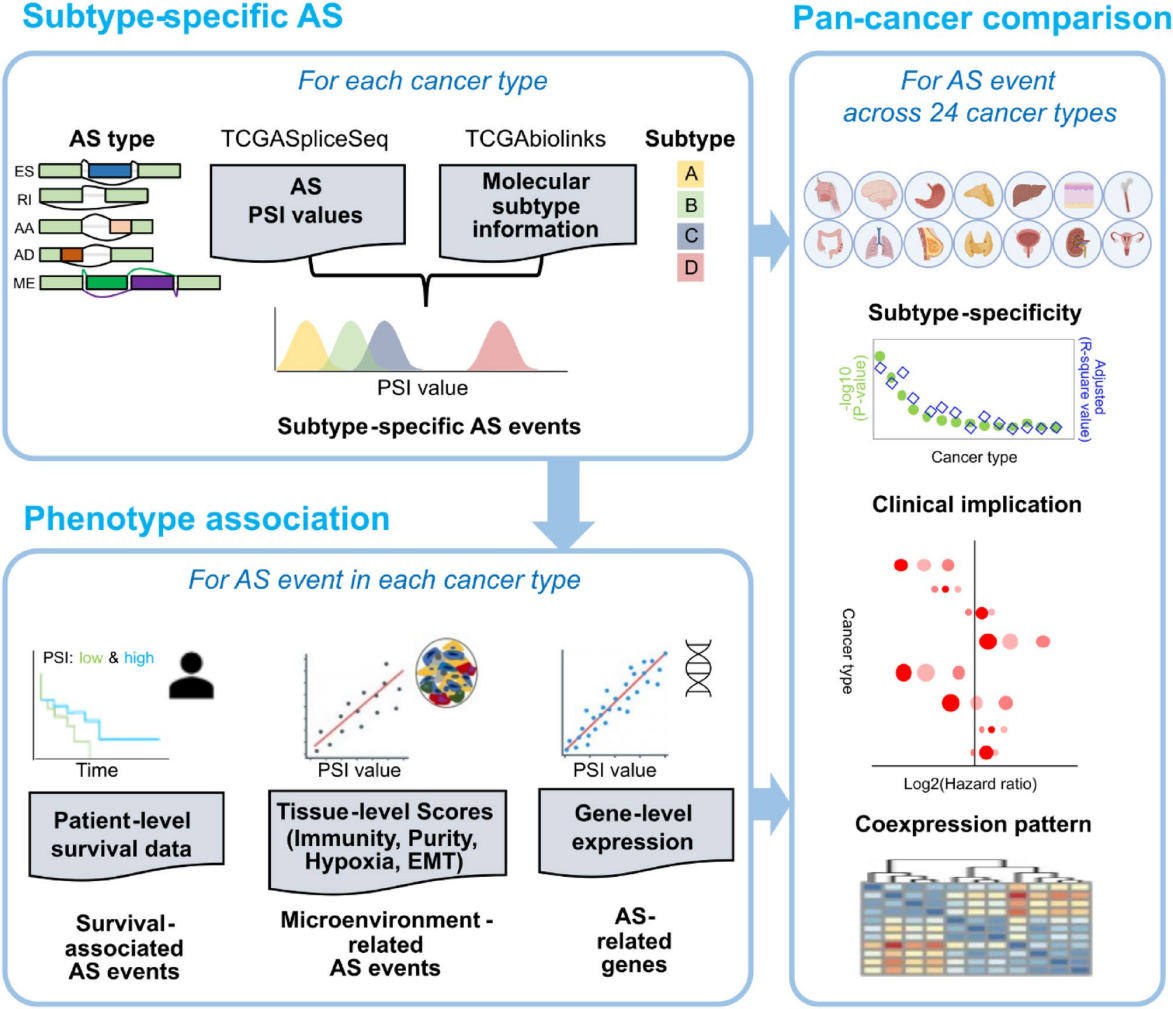
AS-CMC workflow. AS-CMC provides three analysis modules. In the “Subtype-specific AS” module (top left), differential regulation of AS PSI values was tested among molecular subtypes provided by TCGAbiolinks. We used AS events in five splice types (ES, RI, AA, AD, and ME). In the “Phenotype association” module (bottom left), each AS event was evaluated in association with patient-level (clinical outcomes), tissue-level (microenvironment), and gene-level (gene-expression) data. In the “Pan-cancer comparison” module (right), the analyzed data pertaining to each AS event is displayed in a panoramic view across cancer types. In the pan-cancer view, users can access plots summarizing subtype-specificity, clinical implications, and correlations with gene expression. The figure was created with BioRender.com.

AS-CMC is comprised of the following three analysis modules: “Subtype-specific AS,” “Phenotype association,” and “Pan-cancer comparison” (Fig. 1). The “Subtype-specific AS” module allows users to identify AS events with significant differences between molecular subtypes in each cancer type. The “Phenotype association” module enables prioritization of AS events by relevance in terms of clinical outcomes (patient-level), cancer microenvironment scores (tissue-level), and gene expression levels (gene-level). The “Pan-cancer comparison” module can be used to compare the statistical significance of an AS event across cancer types.

For constructing the “Subtype-specific AS” module, we used PSI values of AS events based on TCGASpliceSeq^19^ and molecular subtype information provided by TCGA marker papers (Table S1) through the TCGAbiolinks R package^23^. We performed analysis of variance (ANOVA) and selected subtype-specific AS events if they had *p* < 0.001 and could explain at least 10% of the variation (adjusted R^2^). AS events were categorized into the following five types: exon skip (ES), retained intron (RI), alternate acceptor sites (AA), alternate donor sites (AD), and mutually exclusive exons (ME).

For the “Phenotype association” module, we utilized patient clinical data, molecular scores reflecting the cancer microenvironment, and gene-level expression data. For assessing the association with patient survival, users can compare patient survival rates between high- and low-PSI groups for a given AS. In the cancer microenvironment part, users can investigate the correlations between the changes in AS and predefined scores indicating the status of immune and stromal cells, epithelial-to-mesenchymal transition (EMT), and hypoxia^24–27^. Users can also examine the correlations of each AS event with expression levels of all genes. The gene expression levels correlated with AS PSI values enables determination of biological pathways underlying AS changes. The relationship of the host gene with AS helps in assessing the dependency of the AS event on transcriptional regulation. If the AS is independent of gene expression, it is likely regulated solely by splicing machinery.

“Pan-cancer comparison” module can be used to determine whether the subtype-specific AS regulation is cancer-specific. The module provides panoramic views summarizing the analysis results across cancer types. Users can check whether the biological and clinical association of an AS event is observed in multiple cancer types, which can promote the discovery of pan-cancer AS markers.

### Landscape of subtype-specific AS in TCGA

**Figure 2:**
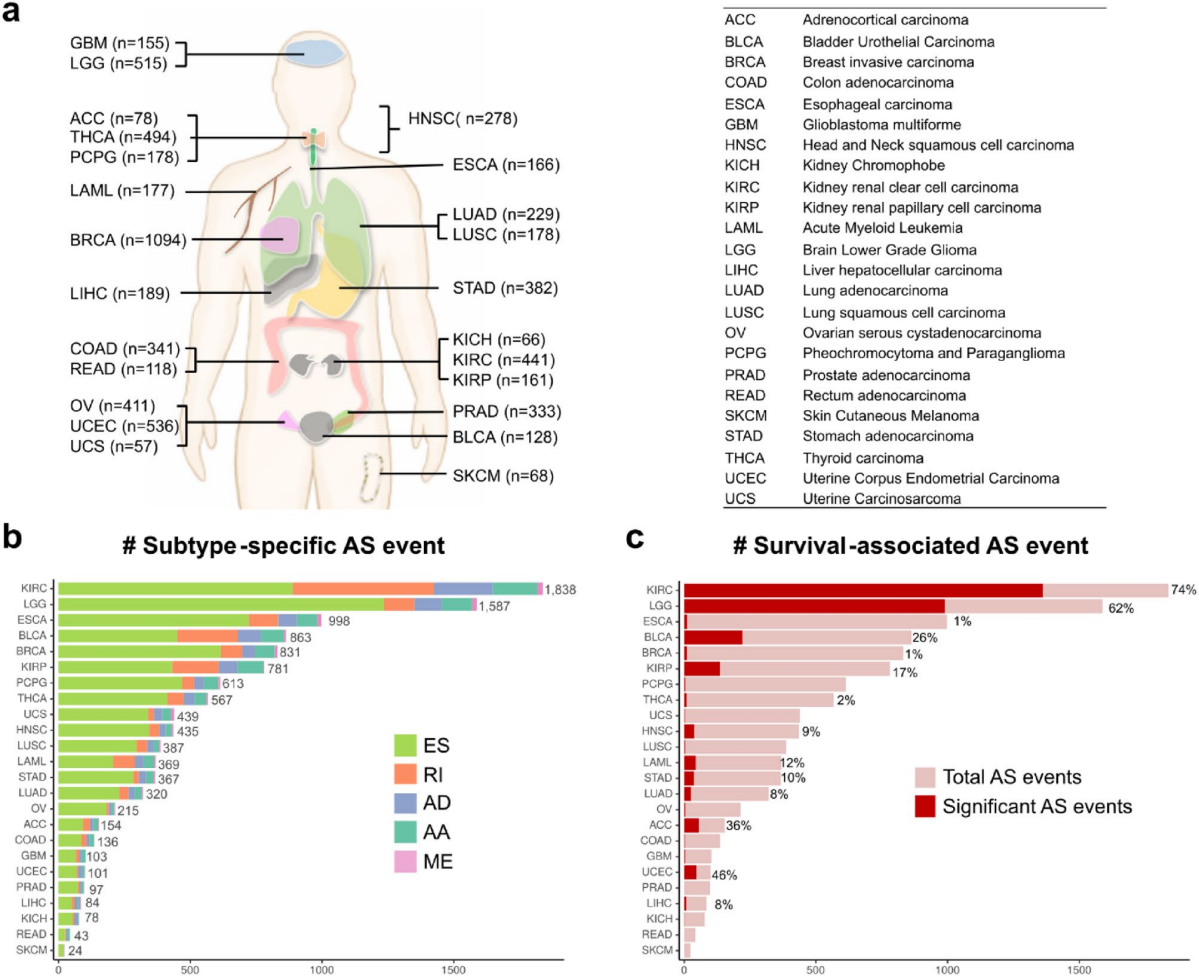
Landscape of subtype-specific AS in TCGA. **(a)** The 24 cancer types included in AS-CMC and number of samples in each cancer type. The TCGA abbreviations and full names are displayed on the right side. **(b)** The number of subtype-specific AS events by splice types. The subtype-specific AS events were selected based on the ANOVA test (*p* < 0.001 and adjusted R^2^ > 0.1). **(c)** The fraction of survival-associated AS events among subtype-specific AS events. The fraction is marked by dark red color and is also shown as percentage on the right side of each bar. X-axis indicates the number of AS events.

We focused on 24 TCGA cancer types with available molecular subtype information among 33 types (Fig. 2a). The sample size for each cancer type varied from 57 to 1094. Details of the molecular subtypes of the selected 24 cancers are listed in Table S2. Of the five AS types, ES was observed to be the most common event, followed by RI (Fig. 2b). We found that the number of subtype-specific AS events are diverse across cancers. Kidney renal clear cell carcinoma (KIRC) was observed to have the highest number of subtype-specific AS events (n = 1838), and 74% of them were found to be associated with patient survival (Fig. 2b,c; Tables S3 and S4; average *p*-value of three survival tests < 0.05). Skin cutaneous melanoma (SKCM) showed the lowest number (n = 24) of subtype-specific AS events, and none of them were found to be associated with survival. Since the difference in sample size can affect the selection of subtype-specific AS event, we checked the relationship between the number of samples and the number of significant AS events (Supplementary Fig. 1). The degree of correlation was generally not high but varies in cancer types or splicing types. Users should be aware that some cancer types with small sample size may have fewer AS events than they actually do.

### AS-CMC user interface

**Figure 3:**
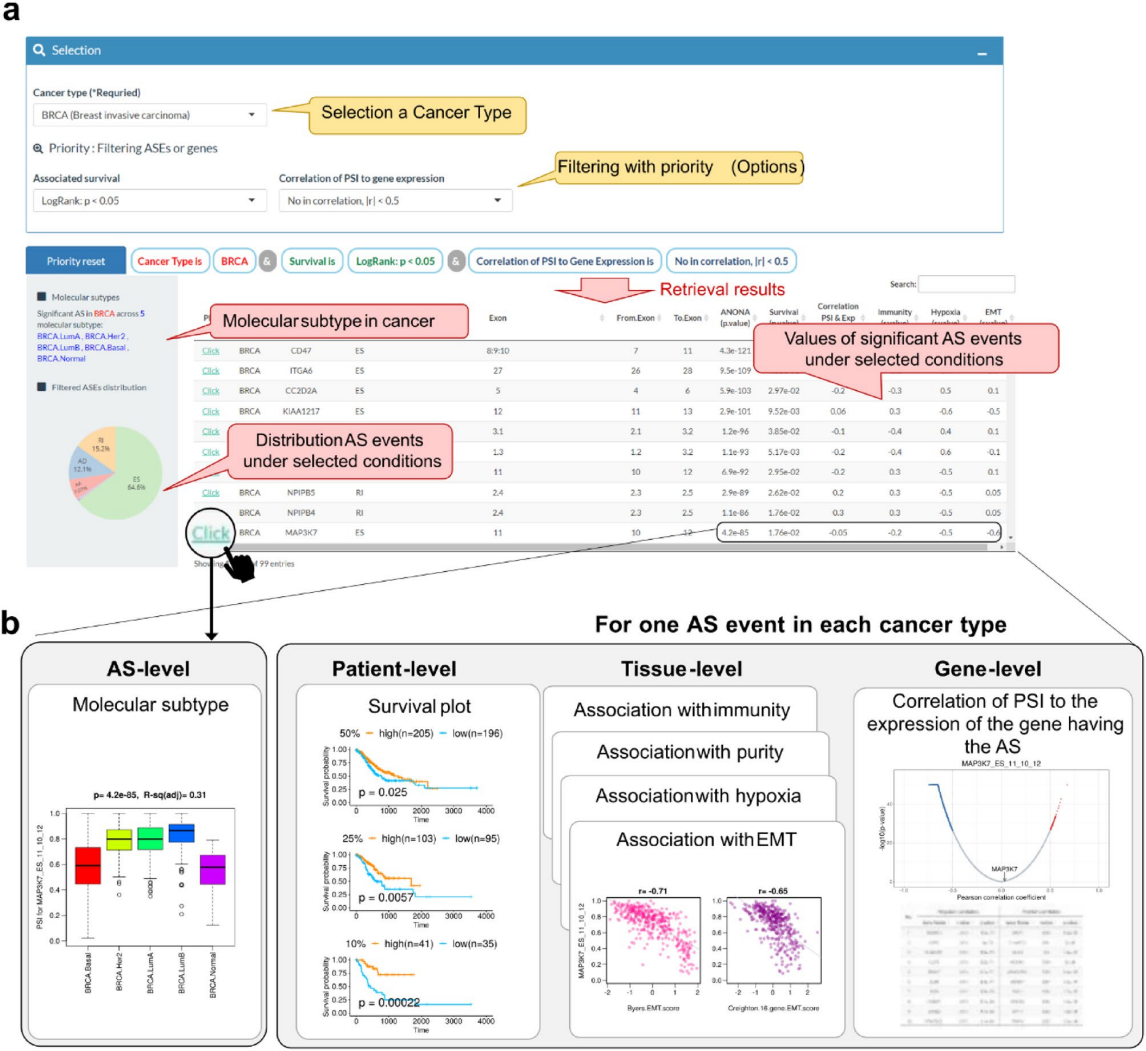
An example of “Single-cancer AS” part in AS-CMC. **(a)** List of subtype-specific AS events for a selected cancer type. Once a cancer type is selected, the subtype-specific AS events are shown with relevant statistics displayed in a tabular form. Users can filter the results using the defined cut-off in survival and correlation with the expression level of genes with the AS. **(b)** Visualization panel showing the analysis results of an AS event. Once an AS event is selected, a window with various plots pops up.

AS-CMC has a user-friendly interface that allows researchers to explore AS events in TCGA molecular subtypes. The brief introduction and summary statistics are shown on the home page (https://www.pmrc.re.kr/ASCMC/). Our web service consists of two parts, viz., “Single-cancer AS” and “Pan-cancer AS.” In the “Single-cancer AS” part, users can browse subtype-specific AS events for each cancer type (Fig. 3a). Users can get basic statistics from ANOVA and correlation analysis in tabular form. The subtype-specific regulation and relationship with phenotypes can be visualized in a pop-up window (Fig. 3b). In the survival plot section, AS-CMC provides three plots representing the survival difference between the groups for an AS event according to the following PSI cut-offs: 50% (upper 50% vs. lower 50%), 25% (upper 25% vs. lower 25%), and 10% (upper 10% vs. lower 10%); for example, the survival difference between the groups with an upper 25% PSI value and the ones with a lower 25% PSI value is provided. The log rank *p*-value is shown for each plot. At the tissue level, AS-CMC provides the following plots showing the relevance of the cancer microenvironment: association with immune infiltration, hypoxia, and EMT scores. AS-CMC provides the distribution plot of AS PSI values and gene expression levels among molecular subtypes, and the correlation coefficient plot of the AS event with all genes in a cancer type is also provided.

**Figure 4:**
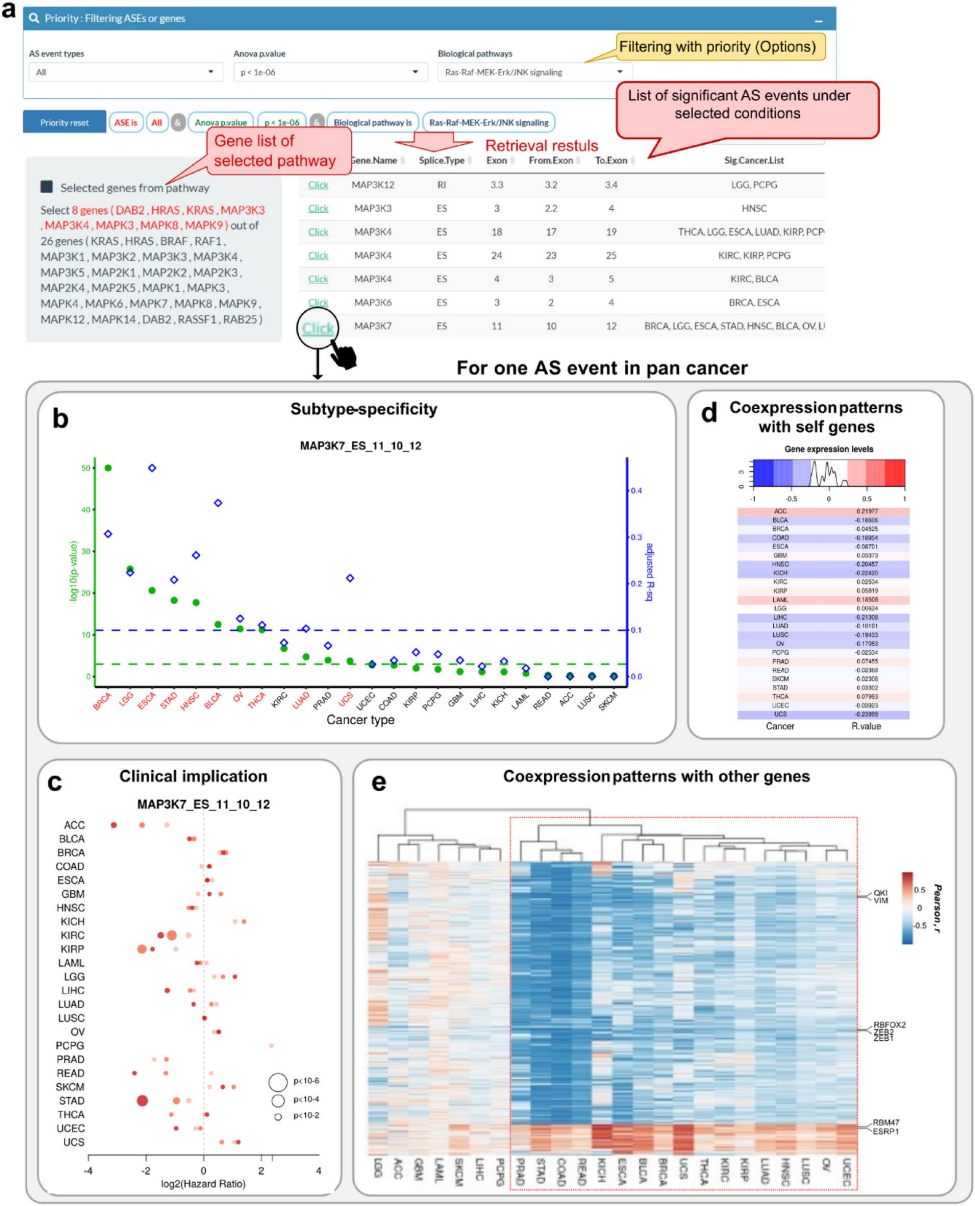
“Pan-cancer comparison” using AS-CMC. **(a)** Selection panel for AS events. Users are directed to the pan-cancer views upon clicking on an AS event. **(b–e)** Pan-cancer information pertaining to *MAP3K7* exon 11 as an example. **(b)** Comparison of subtype-specificity across cancer types. ANOVA results are displayed using two different y-axes: − log10 (*p*-value) and adjusted R^2^ values on the left (green) and right (blue), respectively. The two cut-offs to select subtype-specific AS events are marked by two dotted lines: − log10 (*p*-value) > 3 and adjusted R^2^ > 0.1. The significant cancer types are marked by red on the x-axis. **(c)** Pan-cancer comparison of hazard ratios and log rank *p*-values obtained in survival analysis. The dot size indicates *p*-values from log rank test. Each cancer type has three dots, which match with three survival tests based on PSI cut-offs. The darkest red color indicates the comparison between two patient groups (group with PSI values higher than 10% and one with that lower than 10%). The medium red color indicates 25% cut-off and the lightest red color indicates 50% cut-off used for generating two groups. **(d)** Heatmap showing correlation coefficients with the gene harboring the AS event. The color key on the top shows the distribution of correlation coefficients across cancer types. **(e)** Heatmap showing correlation pattern for a given AS event with gene expression levels. Each cell contains Pearson’s correlation coefficients (r) between *MAP3K7* AS PSI values and expression level of each gene for a given cancer type. Only those genes showing |r|> 0.5 in any cancer type are included. The cancer types and genes are arranged by hierarchical clustering (Euclidean distance and complete linkage method). EMT-related genes are shown on the right, and the cancer types with similar correlation pattern are marked by a red dotted box, on this plot in AS-CMC.

The other part, viz., “Pan-cancer AS”, provides pan-cancer views for a selected AS event (Fig. 4a). For the queried AS event, users can compare subtype-specificity, clinical association, and correlation with genes in a heatmap. Users can prioritize AS events by choosing the AS event type, the minimum *p*-value across cancer types, or relevant biological pathways. Each AS event is displayed with cancer types showing subtype-specificity. Pan-cancer plot for subtype-specificity shows both ANOVA *p*-values and adjusted R^2^ values across cancer types (Fig. 4b). In the pan-cancer survival plot, each cancer type is allotted three data points, which are derived from three survival tests based on PSI cutoff in creating patient groups (Fig. 4c). The correlations between the AS event and host gene expression can be compared in a heatmap (Fig. 4d). The correlations between the AS event and all genes can also be visualized using a heatmap (Fig. 4e). Users can download the analyzed data.

### Case study: an AS event in exon 11 of MAP3K7 gene

Using AS-CMC, we selected a notable subtype-specific AS event that can serve as a pan-cancer AS biomarker as an example. An ES event in exon 11 of *MAP3K7* (mitogen-activated protein kinase kinase kinase 7) gene (Supplementary Fig. 2a) was chosen as this marker showed significant subtype-specificity in 10 cancer types (marked by red on x-axis in Fig. 4b). In the survival analysis, the skipping of the exon was found to be strongly associated with poor clinical outcome in stomach adenocarcinoma (STAD), which was the most significant across all cancer types (log-rank *p* = 0.0002 between upper 10% and lower 10% groups) (marked by the darkest red in Fig. 4c). The survival difference was found to be the largest in the most stringent cut-off (upper 10% vs lower 10%) (Supplementary Fig. 2d). This AS was observed to be likely independent of the transcriptional regulation based on the correlation with the *MAP3K7* gene (|r|< 0.25) (Fig. 4d). Notably, the PSI values were correlated with the expression levels of many genes encoding EMT markers (VIM, ZEB1, and ZEB2) and EMT-related RNA binding proteins (QKI, RBFOX2, RBM47, and ESRP1) (Fig. 4e). Intriguingly, 17 out of 24 cancer types shared the correlation pattern, indicating that they have a common mechanism underlying the AS of *MAP3K7*.

*MAP3K7* AS was analyzed in depth in STAD due to its significant association with survival (Fig. 4c). Among the five molecular subtypes of STAD, only GI.GS subtype showed significant distribution of PSI values compared to the other subtypes (Supplementary Fig. 2b). The PSI values were correlated with the molecular scores related to EMT scores (r = − 0.71) (Supplementary Fig. 2c). Taken together, these data support that an ES event in exon 11 of *MAP3K7* may play a role in regulating subtypes across diverse cancers, and that this event may particularly play a crucial role in STAD, where its function has not been reported earlier.

## Discussion

Molecular classification of cancer is important for precision medicine as it represents the pathophysiology of cancers and can have prognostic value. In recent years, large-scale genomic studies have aided the molecular classification of cancers. Since 2011, the TCGA network has published marker papers providing molecular subtypes for 24 cancer types based on the integrated analysis of the multi-omics data^23^. However, the role of AS in defining molecular subtypes and predicting progression of cancers has not been extensively studied.

AS enhances proteomic diversity by altering protein composition or abundance, which can provide a basis for studying underlying mechanisms of human diseases^5^. In this study, we focused on AS events involved in the molecular classification of cancer that can serve as cancer biomarkers for understanding the pathophysiology of cancers and prediction of the prognosis. Through a comprehensive analysis of AS events in 24 TCGA cancer types, we developed AS-CMC platform to evaluate each AS in association with clinical outcomes, cancer microenvironment, and gene expression.

Compared to other AS databases^20,21^, AS-CMC has distinct advantages. First, it enables the evaluation of the functionality of AS events by incorporating various molecular scores reflecting cancer microenvironment. Cancer microenvironment changes dynamically and contributes to the dynamic nature of events associated with tumor progression such as angiogenesis and metastasis. We collected various molecular scores from published data^24–27^. Extensive immunogenomic analyses provided cellular fraction estimates important for exploring immunogenicity^24,25^. EMT, a biological process that promotes cancer metastasis, was measured in a cancer sample using gene expression profiles^26^. Recent studies have developed hypoxia-associated signature to infer the level of molecular oxygen in tumor tissues^27^.

Another advantage of AS-CMC is that it can be used to assess the utility of pan-cancer AS biomarkers by providing a pan-cancer view of their biological and clinical associations. It allows testing whether the subtype-specific regulation is recurrent across diverse cancers. The ES event in *MAP3K7* gene (exon 11) was represented in this study as a potential pan-cancer AS biomarker capable of discriminating molecular subtypes in 10 cancer types. Exon 11 is 81 bp in size, and encodes 27 amino acids between the kinase and regulatory domains^28^. The function of this AS has been mainly reported in breast cancer^29^. Our study has suggested a potential role of *MAP3K7* AS in STAD due to its significant association with patient survival. The protein expression of *MAP3K7* gene was reported to be involved in gastric cancer progression^30^; however, the role of AS is unknown. We also suggest a potential function of *MAP3K7* AS in EMT based on the coexpression pattern of genes with *MAP3K7* AS. EMT marker genes were found to be consistently correlated with *MAP3K7* AS across 17 cancer types (Fig. 4e), indicating that the regulation mechanism is common beyond breast cancer.

Despite the growing interest in AS in association with cancer, the functional investigation of AS events for biomarker development has not been completely explored due to insufficient data and analysis tools. AS-CMC can prove to be a valuable resource to identify and prioritize AS events for cancer classification. In the future, we continue to update the AS-CMC by including more phenotype association data and using other AS tools to calculate PSI values differently.

## Methods

### Data

For TCGA data analysis, the information pertaining to cancer molecular subtypes was obtained from the TCGAbiolinks R package^23^. We used the most prominent subtype classification for a given tumor, which was found in the column called “Selected subtype” (Table S1). TCGAbiolinks provided gene expression and patient survival data as well. For AS in TCGA samples, we used the per-cent-spliced-in index (PSI) value from the TCGASpliceSeq database^19^. Even though seven AS types exist in the database, we selected the following five AS types: ES, RI, AA, AD, and ME. Alternative promoter and alternative termination types were excluded because they are regulated by transcription initiation and termination in addition to splicing.

### Collection of tissue-level molecular scores using TCGA data

We used various predefined scores to estimate the phenotype of cancer tissues in TCGA data. For immune cell infiltration, the fraction of leukocytes and CIBERSORT immune cells were downloaded from an immunogenomics analysis (https://gdc.cancer.gov/about-data/publications/panimmune)^24^. We also used molecular scores indicating immune and stemness signatures based on the expression levels of their marker genes^25^. For EMT, we used two scores based on the mRNA expression of EMT marker genes^31,32^, which were obtained from a previous report^26^. For the level of molecular oxygen in tumor samples, we used 8 types of hypoxia scores (Buffa, Winter, Ragnum, West, Sorensen, Elvidge, Hu, and Seigneuric) in 19 cancer types in TCGA data^27^.

### Statistical analysis

We selected subtype-specific AS events by analysis of variance (ANOVA) (*p* < 0.001 and adjusted R^2^ > 0.1) for each cancer type. To evaluate whether AS is related with transcriptional regulation, we calculated the correlation coefficient of the PSI values with the expression level of the host gene containing an AS event. For clinical association, we performed survival analysis between high- and low-PSI groups. The threshold to separate the two groups was defined as 10%, 25%, and 50% in the PSI value distribution. The significance of differential survival rates was evaluated by log rank test. Hazard ratio was calculated based on the Cox regression test. For each AS event, correlation analysis for various molecular scores was performed by calculating Spearman’s rank correlation coefficient in R.

### Construction of web-based database

The web interface was written in the R programming language using the shiny package. The Plotly R graphing library was used to generate interactive visualization. Our analyzed data can be accessed in two ways: “Single-cancer AS” and “Pan-cancer AS.” In the “Single-cancer AS,” users can select the cancer type first and get the list of AS events with the statistical analysis results. In the “Pan-cancer AS,” users can obtain pan-cancer views for a selected AS event. The result table can be sorted by clicking the column names of the table and can be filtered by selecting one of the predefined criteria.

## Supplementary Information

Supplementary Information.

## Data Availability

AS-CMC is available at http://www.pmrc.re.kr/ASCMC/.

## References

[1] Hoadley KA (2018). Cell-of-origin patterns dominate the molecular classification of 10,000 Tumors from 33 Types of Cancer. Cell.

[2] Cancer Genome Atlas Research N (2015). Comprehensive, Integrative genomic analysis of diffuse lower-grade gliomas. N. Engl. J. Med..

[3] Cance rGenome Atlas Research N (2016). Comprehensive molecular characterization of papillary renal-cell carcinoma. N. Engl. J. Med..

[4] Guinney J (2015). The consensus molecular subtypes of colorectal cancer. Nat. Med..

[5] Liu Y (2017). Impact of alternative splicing on the human proteome. Cell Rep..

[6] Mazin PV, Khaitovich P, Cardoso-Moreira M, Kaessmann H (2021). Alternative splicing during mammalian organ development. Nat. Genet..

[7] Cherry S, Lynch KW (2020). Alternative splicing and cancer: Insights, opportunities, and challenges from an expanding view of the transcriptome. Genes Dev..

[8] Kahles A (2018). Comprehensive analysis of alternative splicing across tumors from 8705 patients. Cancer Cell.

[9] Zhang Y (2019). Pan-cancer analysis of clinical relevance of alternative splicing events in 31 human cancers. Oncogene.

[10] Sebestyen E (2016). Large-scale analysis of genome and transcriptome alterations in multiple tumors unveils novel cancer-relevant splicing networks. Genome Res..

[11] Wu S (2020). The functional impact of alternative splicing on the survival prognosis of triple-negative breast cancer. Front Genet..

[12] Jia K, Wu Y, Huang J, Wu H (2019). Survival-associated alternative splicing events in pan-renal cell carcinoma. Front Oncol..

[13] Liu Y, Jia W, Li J, Zhu H, Yu J (2020). Identification of survival-associated alternative splicing signatures in lung squamous cell carcinoma. Front Oncol..

[14] Han B (2021). Systematic analysis of survival-associated alternative splicing signatures in thyroid carcinoma. Front Oncol..

[15] Park J, Chung YJ (2019). Identification of neoantigens derived from alternative splicing and RNA modification. Genomics Inform..

[16] Cheng R (2022). Identification of alternative splicing-derived cancer neoantigens for mRNA vaccine development. Brief Bioinform..

[17] Bowling EA (2021). Spliceosome-targeted therapies trigger an antiviral immune response in triple-negative breast cancer. Cell.

[18] Lu SX (2021). Pharmacologic modulation of RNA splicing enhances anti-tumor immunity. Cell.

[19] Ryan M (2016). TCGASpliceSeq a compendium of alternative mRNA splicing in cancer. Nucleic Acids Res..

[20] Kim P, Yang M, Yiya K, Zhao W, Zhou X (2020). ExonSkipDB: Functional annotation of exon skipping event in human. Nucleic Acids Res..

[21] Zhang Y (2022). OncoSplicing: An updated database for clinically relevant alternative splicing in 33 human cancers. Nucleic Acids Res..

[22] Cheng R (2021). A pan-cancer analysis of alternative splicing of splicing factors in 6904 patients. Oncogene.

[23] Mounir M (2019). New functionalities in the TCGAbiolinks package for the study and integration of cancer data from GDC and GTEx. PLoS Comput. Biol..

[24] Thorsson V (2018). The Immune landscape of cancer. Immunity.

[25] Chen Z, Chen C, Li L, Zhang T, Wang X (2021). The spliceosome pathway activity correlates with reduced anti-tumor immunity and immunotherapy response, and unfavorable clinical outcomes in pan-cancer. Comput. Struct. Biotechnol. J..

[26] Gibbons DL, Creighton CJ (2018). Pan-cancer survey of epithelial-mesenchymal transition markers across the Cancer Genome Atlas. Dev. Dyn..

[27] Bhandari V (2019). Molecular landmarks of tumor hypoxia across cancer types. Nat. Genet..

[28] Venables JP, Vignal E, Baghdiguian S, Fort P, Tazi J (2012). Tissue-specific alternative splicing of Tak1 is conserved in deuterostomes. Mol. Biol. Evol..

[29] Qiu Y, Lyu J, Dunlap M, Harvey SE, Cheng C (2020). A combinatorially regulated RNA splicing signature predicts breast cancer EMT states and patient survival. RNA.

[30] Yang Y (2017). Expression and function of transforming growth factorbetaactivated protein kinase 1 in gastric cancer. Mol. Med. Rep..

[31] Byers LA (2013). An epithelial-mesenchymal transition gene signature predicts resistance to EGFR and PI3K inhibitors and identifies Axl as a therapeutic target for overcoming EGFR inhibitor resistance. Clin. Cancer Res..

[32] Creighton CJ, Gibbons DL, Kurie JM (2013). The role of epithelial-mesenchymal transition programming in invasion and metastasis: A clinical perspective. Cancer Manag. Res..

