# Supplementary material for: AS-CMC: a pan-cancer database of alternative splicing for molecular classification of cancer
Source: Sci Rep. 2022 Dec 6;12:21074. doi: 10.1038/s41598-022-25584-6 (PMC9726986; doi:10.1038/s41598-022-25584-6)
Supplement: Supplementary file 1 — Supplementary Information. [file 41598_2022_25584_MOESM1_ESM.pdf]

AS-CMC: A Pan-cancer Database of Alternative Splicing for Molecular Classification of Cancer

Jiyeon Park[1,2,3,#] Jin-Ok Lee[3,#], Minho Lee[4], Yeun-Jun Chung[1,2,5*]

[1]Precision Medicine Research Center, College of Medicine, the Catholic University of Korea, Seoul, Republic of Korea.

[2]Integrated Research Center for Genome Polymorphism, the Catholic University of Korea, Seoul, Republic of Korea

[3]Department of Biomedicine and Health Sciences, Graduate School, the Catholic University of Korea, Seoul, Republic of Korea

[4]Department of Life Science, Dongguk University-Seoul, Goyang 10326, Republic of Korea

[5]Department of Microbiology, College of Medicine, the Catholic University of Korea, Seoul, Republic of Korea

# Both authors contributed equally to this paper

Supplementary Information

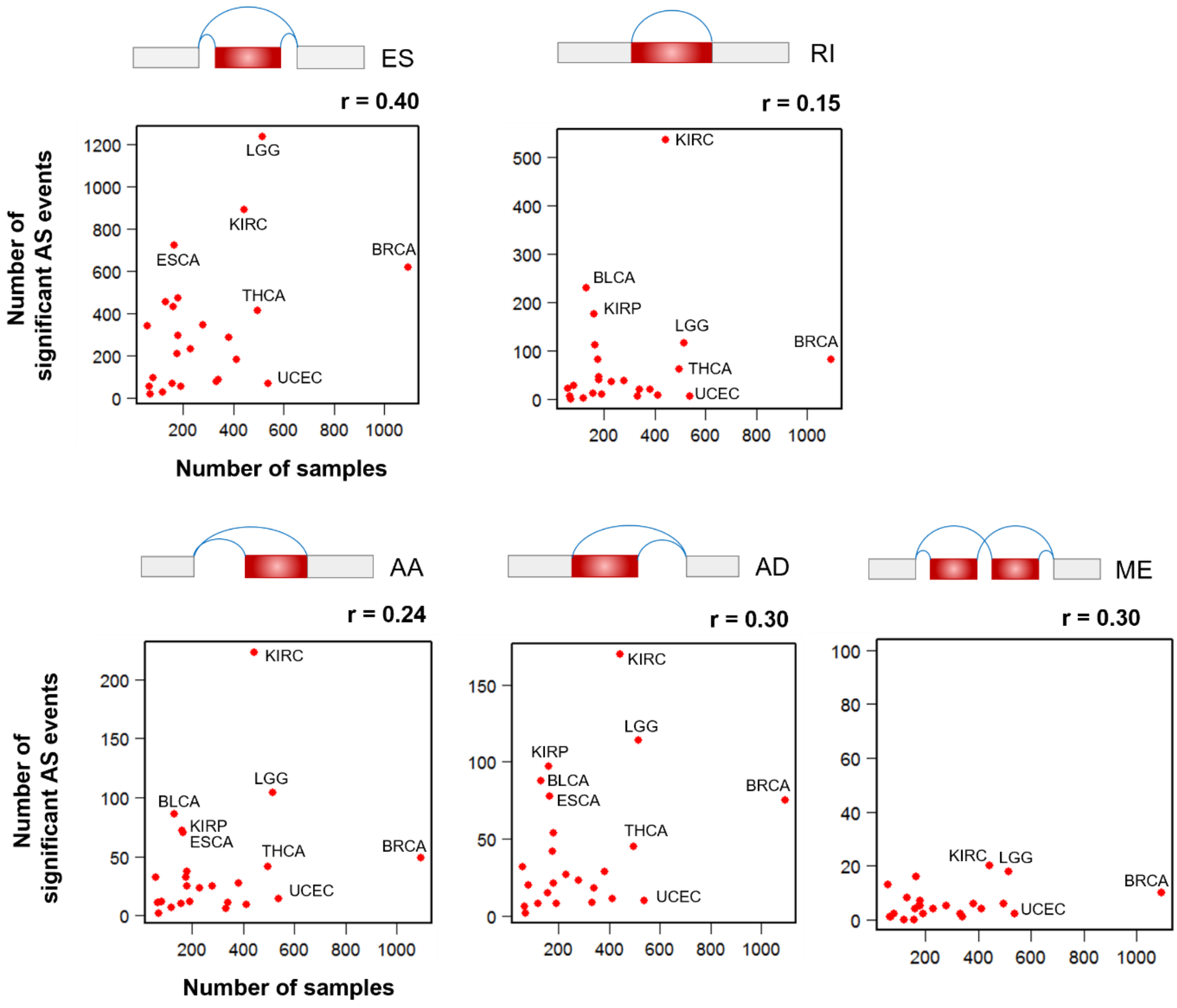

Supplementary Figure 1. Effect of sample size on the number of subtype-specific AS events for each AS type. Pearson correlation coefficient is shown on the top right for each plot. AS events were categorized into following five types: exon skip (ES), retained intron (RI), alternate acceptor sites (AA), alternate donor sites (AD), and mutually exclusive exons (ME).

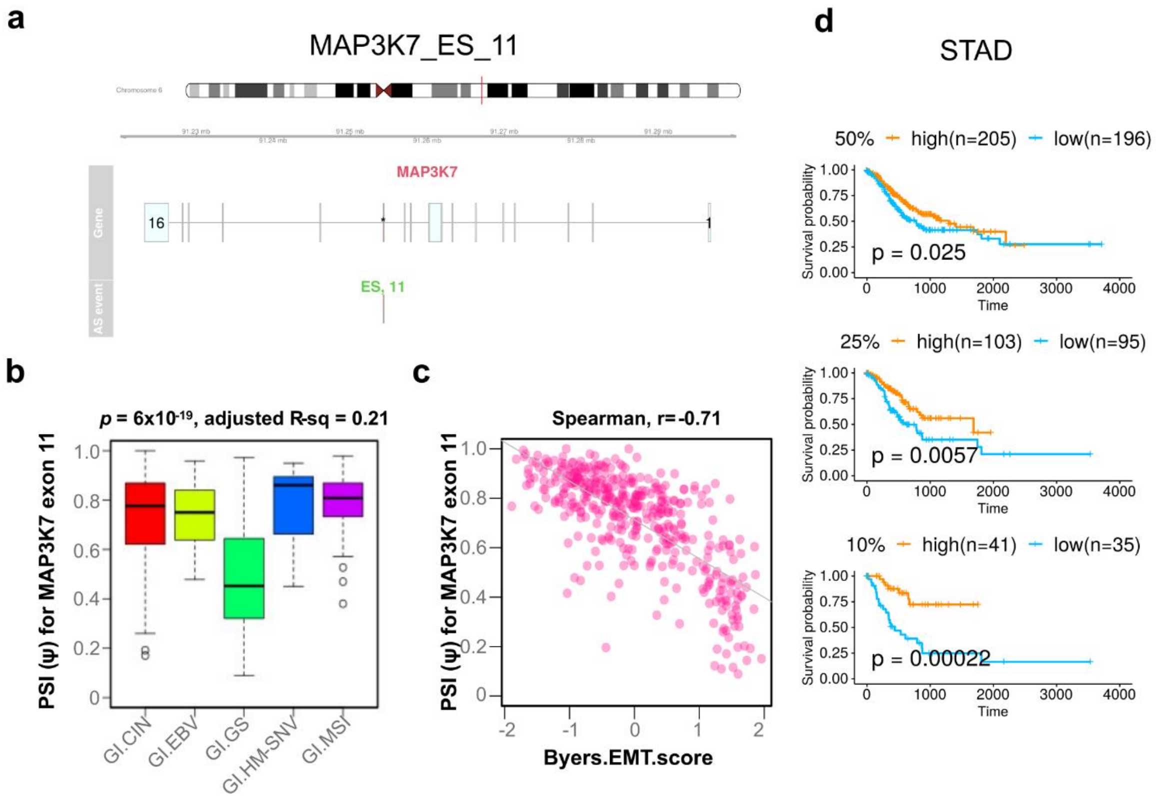

Supplementary Figure 2. An example of a potential pan-cancer AS biomarker. (a) Location of *MAP3K7* exon with AS on the corresponding gene and chromosome. **(b)** Distribution of PSI values for five molecular subtypes in STAD. **(c)** Correlations between the AS PSIs and EMT scores in STAD. Spearman's correlation coefficient is shown on the top. Each dot represents an individual patient sample. **(d)** Survival plots comparing survival rates between two patient groups with high- and low-PSI values in STAD. In the survival plots, AS-CMC provides three plots representing the survival difference between the groups for an AS event according to the following PSI cut-offs: 50% (upper 50% vs lower 50%), 25% (upper 25% vs lower 25%), and 10% (upper 10% vs lower 10%). The significance of differential survival rates was evaluated using log rank test.

Table S1. Molecular classification for cacner type.

The table derives from the following site: https://www.bioconductor.org/packages/devel/bioc/vignettes/TCGAbiolinks/inst/doc/subtypes.html.

Cancer type	All available molecular data based-subtype	Selected subtype	Number of samples	Reference	link to paper
ACC	mRNA, DNAmeth, protein, miRNA, CNA, COC, C1A.C1B	DNAmeth	91	Cancer Cell 2016	Link
LAML	mRNA and miRNA	mRNA	187	NEJM 2013	Link
BLCA	mRNA subtypes	mRNA	129	Nature 2014	Link
BRCA	PAM50 (mRNA)	PAM50	1,218	Nature 2012	Link
GBM/LGG*	mRNA, DNAmeth, protein, Supervised_DNAmeth	Supervised_DNAmeth	1,122	Cell 2016	Link
Pan-GI (preliminary) ESCA/STAD/COAD/READ	Molecular_Subtype	Molecular_Subtype	1,011	Cancer Cell 2018	Link
HNSC	mRNA, DNAmeth, RPPA, miRNA, CNA, Paradigm	mRNA	279	Nature 2015	Link
KICH	Eosinophilic	Eosinophilic	66	Cancer Cell 2014	Link
KIRC	mRNA, miRNA	mRNA	442	Nature 2013	Link
KIRP	mRNA, DNAmeth, protein, miRNA, CNA, COC	COC	161	NEJM 2015	Link
LIHC (preliminary)	mRNA, DNAmeth, protein, miRNA, CNA, Paradigma, iCluster	iCluster	196	not published	
LUAD	DNAmeth, iCluster	iCluster	230	Nature 2014	Link
LUSC	mRNA	mRNA	178	Nature 2012	Link
OV	mRNA	mRNA	489	Nature 2011	Link
PCPG	mRNA, DNAmeth, protein, miRNA, CNA	mRNA	178	Cancer Cell 2017	Link
PRAD	mRNA, DNAmeth, protein, miRNA, CNA, icluster, mutation/fusion	mutation/fusion	333	Cell 2015	Link
SKCM	mRNA, DNAmeth, protein, miRNA, mutation	mutation	331	Cell 2015	Link
THCA	mRNA, DNAmeth, protein, miRNA, CNA, histology	mRNA	496	Cell 2014	Link
UCEC	iCluster, MSI, CNA, mRNA	iCluster - updated according to Pan-Gyne/Pathways groups	538	Nature 2013	Link
UCS (preliminary)	mRNA	mRNA	57	not published	

The column “Selected subtype” was selected as most prominent subtype classification (from the other columns)

Table S2. Number of samples for cancer type and subtypes.
The subtype information derived from PLoS Comput Biol 2019 Vol. 15 Issue 3 Pages e1006701.

Cancer type	Full cancer name	n.sample	subtypes	n.subtype
BRCA	Breast invasive carcinoma	1,094	BRCA.Normal(40), BRCA.Her2(82), BRCA.Basal(190), BRCA.LumB(217), BRCA.LumA(565)	5
UCEC	Uterine Corpus Endometrial Carcinoma	536	UCEC.NA(29), UCEC.POLE(79), UCEC.MSI(124), UCEC.CN_LOW(144), UCEC.CN_HIGH(160)	5
LGG	Brain Lower Grade Glioma	515	GBM_LGG.NA(3), GBM_LGG.G-CIMP-low(12), GBM_LGG.Classic-like(22), GBM_LGG.PA-like(26), GBM_LGG.Mesenchymal-like(45), GBM_LGG.Codel(173), GBM_LGG.G-CIMP-high(234)	7
THCA	Thyroid carcinoma	494	THCA.NA(12), THCA.2(67), THCA.3(82), THCA.5(93), THCA.4(106), THCA.1(134)	6
KIRC	Kidney renal clear cell carcinoma	441	KIRC.NA(24), KIRC.4(86), KIRC.2(90), KIRC.3(94), KIRC.1(147)	5
OV	Ovarian serous cystadenocarcinoma	411	OVCA.Immunoreactive(89), OVCA.Mesenchymal(98), OVCA.Differentiated(108), OVCA.Proliferative(116)	4
STAD	Stomach adenocarcinoma	382	GI.HM-SNV(7), GI.EBV(30), GI.GS(50), GI.MSI(73), GI.CIN(222)	5
COAD	Colon adenocarcinoma	341	GI.HM-SNV(6), GI.GS(49), GI.MSI(60), GI.CIN(226)	4
PRAD	Prostate adenocarcinoma	333	PRAD.7-IDH1(3), PRAD.4-FLI1(4), PRAD.6-FOXA1(9), PRAD.3-ETV4(14), PRAD.2-ETV1(28), PRAD.5-SPOP(37), PRAD.8-other(86), PRAD.1-ERG(152)	8
HNSC	Head and Neck squamous cell carcinoma	278	HNSC.Classical(49), HNSC.Atypical(68), HNSC.Mesenchymal(75), HNSC.Basal(86)	4
LUAD	Lung adenocarcinoma	229	LUAD.1(22), LUAD.2(32), LUAD.4(32), LUAD.6(41), LUAD.3(51), LUAD.5(51)	6
LIHC	Liver hepatocellular carcinoma	189	LIHC.NA(6), LIHC.iCluster:2(55), LIHC.iCluster:3(63), LIHC.iCluster:1(65)	4
LUSC	Lung squamous cell carcinoma	178	LUSC.primitive(27), LUSC.basal(43), LUSC.secretory(43), LUSC.classical(65)	4
PCPG	Pheochromocytoma and Paraganglioma	178	PCPG.NA(5), PCPG.Cortical admixture(22), PCPG.Wnt-altered(22), PCPG.Pseudohypoxia(61), PCPG.Kinase signaling(68)	5
LAML	Acute Myeloid Leukemia	177	AML.1(14), AML.3(18), AML.7(20), AML.2(21), AML.5(33), AML.6(35), AML.4(36)	7
ESCA	Esophageal carcinoma	165	GI.GS(1), GI.HM-SNV(2), GI.MSI(2), GI.CIN(74), GI.ESCC(87)	5
KIRP	Kidney renal papillary cell carcinoma	161	KIRP.C2c - CIMP(9), KIRP.C2b(22), KIRP.C2a(35), KIRP.C1(95)	4
GBM	Glioblastoma multiforme	155	GBM_LGG.G-CIMP-high(2), GBM_LGG.G-CIMP-low(5), GBM_LGG.LGm6-GBM(13), GBM_LGG.NA(36), GBM_LGG.Classic-like(46), GBM_LGG.Mesenchymal-like(53)	6
BLCA	Bladder Urothelial Carcinoma	128	BLCA.4(15), BLCA.3(30), BLCA.1(41), BLCA.2(42)	4
READ	Rectum adenocarcinoma	118	GI.MSI(3), GI.HM-SNV(4), GI.GS(9), GI.CIN(102)	4
ACC	Adrenocortical carcinoma	78	ACC.CIMP-high(19), ACC.CIMP-intermediate(27), ACC.CIMP-low(32)	3
SKCM	Skin Cutaneous Melanoma	68	SKCM.NF1_Any_Mutants(4), SKCM.Triple_WT(10), SKCM.-(11), SKCM.RAS_Hotspot_Mutants(11), SKCM.BRAF_Hotspot_Mutants(32)	5
KICH	Kidney Chromophobe	66	KICH.Eosin.1(19), KICH.Eosin.0(47)	2
UCS	Uterine Carcinosarcoma	57	UCS.1(17), UCS.2(40)	2

Table S3. Number of subtype-specific AS events ($p < 0.001$ and adjusted $R^2 > 0.1$)

ES: exon skip, RI: retained intron, AA: alternate acceptor sites, AD: alternate donor sites, ME: mutually exclusive exons

Cancer type	ES	RI	AA	AD	ME	total
KIRC	889	536	223	170	20	1,838
LGG	1,235	116	104	114	18	1,587
ESCA	722	112	70	78	16	998
BLCA	451	230	86	88	8	863
BRCA	615	82	49	75	10	831
KIRP	432	176	72	97	4	781
PCPG	469	46	37	54	7	613
THCA	413	62	41	45	6	567
UCS	340	22	32	32	13	439
HNSC	344	38	25	23	5	435
LUSC	296	40	25	21	5	387
LAML	207	83	32	42	5	369
STAD	284	21	27	29	6	367
LUAD	230	36	23	27	4	320
OV	182	9	9	11	4	215
ACC	92	28	12	20	2	154
COAD	86	20	11	18	1	136
GBM	65	13	10	15	-	103
UCEC	69	6	14	10	2	101
PRAD	74	6	6	9	2	97
LIHC	51	11	12	8	2	84
KICH	53	7	11	6	1	78
READ	25	3	7	8	-	43
SKCM	18	1	2	2	1	24

Table S4. Subtype-specific AS events and the phenotype associations

							ANOVA		correlation to the host gene	Survival analysis		
cancer type	id	Gene Symbol	splice_type	Exon	From.Exon	To.Exon	anova.p	adj.r2	r	p.50	p.25	p.10
ACC	ACAD9_AD_1.2_1.1_2.1	ACAD9	AD	1.2	1.1	2.1	3.6E-04	0.192	-0.174	5.6E-01	9.1E-01	8.9E-01
ACC	ACOT9_ES_6_5.1_7.1	ACOT9	ES	6	5.1	7.1	7.3E-04	0.175	0.124	9.8E-01	8.6E-01	2.6E-01
ACC	ACP1_ES_4.1_3_4.4	ACP1	ES	4.1	3	4.4	7.2E-04	0.155	-0.340	8.3E-01	5.6E-01	4.7E-01
ACC	AKAP17A_ES_4.3_4.1_5	AKAP17A	ES	4.3	4.1	5	3.1E-04	0.191	-0.111	1.9E-01	1.5E-01	6.6E-03
ACC	ALAS1_ES_2_1_3.1	ALAS1	ES	2	1	3.1	4.6E-04	0.168	0.115	4.0E-01	1.1E-01	2.9E-02
ACC	AMHR2_ES_7:8:9:10.2_6_11	AMHR2	ES	7:8:9:10.2	6	11	1.0E-06	0.362	0.592	1.2E-02	8.2E-03	1.9E-01
ACC	ANKS3_ES_4_3_5.1	ANKS3	ES	4	3	5.1	7.0E-05	0.223	0.154	8.8E-05	8.8E-04	1.2E-02
ACC	APH1A_RI_6.2_6.1_6.3	APH1A	RI	6.2	6.1	6.3	5.7E-04	0.159	0.079	3.1E-02	3.5E-02	1.9E-01
ACC	ATXN2_ES_25_23_26	ATXN2	ES	25	23	26	1.0E-04	0.201	0.211	1.7E-03	1.1E-02	6.7E-02
ACC	AVL9_ES_14_13_15	AVL9	ES	14	13	15	1.5E-05	0.242	0.023	2.4E-02	7.5E-03	4.2E-02
ACC	BCL2L1_AA_2.2_1_2.3	BCL2L1	AA	2.2	1	2.3	1.0E-05	0.262	-0.102	9.9E-01	7.9E-01	2.4E-01
ACC	BRD8_ES_22_21_23	BRD8	ES	22	21	23	1.3E-06	0.285	0.141	4.5E-03	2.3E-03	9.8E-03
ACC	BTN3A2_AA_4.1_3.2_4.2	BTN3A2	AA	4.1	3.2	4.2	8.9E-04	0.175	-0.239	7.7E-01	3.6E-01	1.5E-01
ACC	C16orf58_RI_8.3_8.2_8.4	C16orf58	RI	8.3	8.2	8.4	1.7E-04	0.190	-0.024	2.7E-01	6.3E-01	9.7E-01
ACC	C16orf91_RI_4.2_4.1_4.3	C16orf91	RI	4.2	4.1	4.3	3.2E-06	0.277	0.305	5.2E-03	1.2E-02	2.9E-03
ACC	C21orf2_AD_6.3_6.2_7	C21orf2	AD	6.3	6.2	7	5.9E-06	0.255	-0.267	2.4E-03	3.3E-04	9.4E-02
ACC	C9orf117_RI_5.4_5.3_5.5	C9orf117	RI	5.4	5.3	5.5	2.5E-04	0.230	-0.603	3.6E-03	1.5E-02	8.8E-02
ACC	CALHM2_AA_4.1_3.2_4.2	CALHM2	AA	4.1	3.2	4.2	4.4E-05	0.217	-0.649	1.9E-02	3.0E-02	1.2E-02
ACC	CAPRIN2_AA_15.2_14_15.3	CAPRIN2	AA	15.2	14	15.3	2.6E-04	0.200	0.158	9.5E-03	2.9E-01	3.0E-01
ACC	CCNL2_ES_6.1:7.1_5_7.2	CCNL2	ES	6.1:7.1	5	7.2	7.8E-04	0.162	0.271	3.7E-01	6.4E-01	4.3E-01
ACC	CD320_ES_2.2:3.2_1_4	CD320	ES	2.2:3.2	1	4	2.8E-04	0.220	0.332	1.6E-02	2.0E-01	2.8E-02
ACC	CDC42SE1_ES_5_4_6	CDC42SE1	ES	5	4	6	7.7E-04	0.158	-0.127	5.8E-02	1.0E-01	2.8E-01
ACC	CIRBP_RI_9.9:9.10_9.8_9.11	CIRBP	RI	9.9:9.10	9.8	9.11	4.4E-04	0.165	-0.395	2.3E-01	1.7E-01	2.8E-02
ACC	COPS4_ES_10:11_9_12	COPS4	ES	10:11	9	12	9.4E-05	0.225	-0.235	5.8E-02	1.0E-01	6.7E-01
ACC	COQ4_RI_2.2_2.1_2.3	COQ4	RI	2.2	2.1	2.3	2.3E-04	0.181	0.029	2.1E-01	3.7E-01	8.3E-01
ACC	COX4I1_AA_5.1:5.2:5.3_4.1_5.4	COX4I1	AA	5.1:5.2:5.3	4.1	5.4	4.4E-05	0.214	-0.464	2.0E-03	1.2E-02	2.7E-02
ACC	CPT1B_RI_22.2_22.1_22.3	CPT1B	RI	22.2	22.1	22.3	5.4E-04	0.160	-0.158	1.1E-01	4.1E-01	8.6E-01
ACC	CTNNB1_RI_18.2_18.1_18.3	CTNNB1	RI	18.2	18.1	18.3	4.5E-04	0.164	-0.328	2.6E-03	1.2E-04	1.9E-03
ACC	CUTC_ES_7_6_8	CUTC	ES	7	6	8	1.4E-04	0.194	0.334	2.2E-02	3.4E-01	8.2E-01
ACC	DAP3_AD_2.3_2.2_3	DAP3	AD	2.3	2.2	3	2.7E-04	0.180	0.162	2.0E-02	1.5E-03	1.8E-02
ACC	DDX41_AA_3.1_2_3.2	DDX41	AA	3.1	2	3.2	3.3E-04	0.175	0.487	2.6E-02	3.5E-02	3.2E-01
ACC	DMAP1_RI_1.2_1.1_1.3	DMAP1	RI	1.2	1.1	1.3	1.7E-04	0.190	0.300	3.9E-01	5.4E-01	5.7E-02
ACC	DNAJC5_ES_5_4_6	DNAJC5	ES	5	4	6	5.7E-05	0.209	-0.176	1.2E-01	1.1E-02	3.6E-01
ACC	DYNLL1_RI_4.2_4.1_4.3	DYNLL1	RI	4.2	4.1	4.3	2.9E-05	0.226	-0.186	2.5E-04	4.7E-05	2.7E-02
ACC	DYRK1B_AA_10.1:10.2_9_10.3	DYRK1B	AA	10.1:10.2	9	10.3	3.4E-05	0.222	-0.119	4.3E-03	1.2E-03	5.5E-03
ACC	EHBP1_ES_18_17_19	EHBP1	ES	18	17	19	2.0E-04	0.192	0.095	7.7E-01	5.1E-01	5.0E-01

cancer type	id	Gene Symbol	splice_type	Exon	From.Exon	To.Exon	anova.p	adj.r2	r	p.50	p.25	p.10
ACC	EPOR_RI_9.2_9.1_9.3	EPOR	RI	9.2	9.1	9.3	1.5E-04	0.191	-0.562	2.5E-02	8.9E-05	1.0E-02
ACC	FAM156B_RI_2.3:2.4_2.2_2.5	FAM156B	RI	2.3:2.4	2.2	2.5	5.7E-04	0.161	0.286	1.1E-03	4.0E-04	1.1E-01
ACC	FGFR1_ES_6_4_8.2	FGFR1	ES	6	4	8.2	8.3E-04	0.169	0.128	2.0E-02	1.6E-01	2.5E-01
ACC	FLNB_ES_32.1_31_33	FLNB	ES	32.1	31	33	6.0E-04	0.196	0.160	1.5E-01	9.9E-02	7.5E-02
ACC	GLRX3_ES_11.1_10_12.1	GLRX3	ES	11.1	10	12.1	1.3E-06	0.313	0.322	5.2E-01	5.9E-01	8.0E-01
ACC	GNAS_ES_6_5_8.1	GNAS	ES	6	5	8.1	1.8E-04	0.184	-0.184	1.0E-01	5.8E-02	4.1E-02
ACC	HBP1_AD_1.2_1.1_4	HBP1	AD	1.2	1.1	4	2.9E-04	0.185	0.092	6.1E-04	4.5E-04	2.0E-01
ACC	HHLA3_ES_4_3_5	HHLA3	ES	4	3	5	6.0E-04	0.158	0.138	3.9E-02	4.9E-02	9.6E-01
ACC	HM13_ES_11:12.1_10_13	HM13	ES	11:12.1	10	13	5.3E-06	0.267	-0.076	4.2E-05	4.8E-05	2.2E-02
ACC	HM13_ES_11:12.1:12.2_10_13	HM13	ES	11:12.1:12.2	10	13	1.2E-08	0.376	-0.107	6.1E-06	4.2E-06	1.1E-04
ACC	HMGN3_AD_5.2_5.1_6	HMGN3	AD	5.2	5.1	6	3.8E-04	0.168	0.404	2.9E-01	5.5E-01	2.2E-01
ACC	HNRNPA1_ES_6.2:7.1:7.2:8:9.1_6.1_9.2	HNRNPA1	ES	6.2:7.1:7.2:8:9.1	6.1	9.2	1.5E-04	0.188	0.212	7.9E-04	1.1E-04	5.7E-03
ACC	HNRNPA2B1_ES_12.2_11_12.4	HNRNPA2B1	ES	12.2	11	12.4	3.1E-05	0.221	-0.018	8.3E-02	3.7E-02	1.2E-01
ACC	HNRNPDL_ES_8_7_9	HNRNPDL	ES	8	7	9	6.2E-04	0.157	-0.215	3.1E-01	7.6E-01	9.6E-01
ACC	HSF2_ES_11_10_12	HSF2	ES	11	10	12	8.7E-05	0.205	-0.039	4.6E-02	1.3E-02	2.7E-01
ACC	ICAM3_RI_3.2_3.1_3.3	ICAM3	RI	3.2	3.1	3.3	3.8E-04	0.174	-0.105	9.1E-02	1.1E-02	3.5E-01
ACC	IKBIP_ES_2_1_4	IKBIP	ES	2	1	4	7.0E-04	0.160	0.208	5.1E-01	2.2E-02	5.3E-01
ACC	IL18BP_RI_1.7_1.6_1.8	IL18BP	RI	1.7	1.6	1.8	1.1E-05	0.277	-0.432	4.2E-02	1.7E-02	1.6E-01
ACC	IL32_AA_1.3:1.4:1.5:1.6:1.7:1.8_1.1_1.9	IL32	AA	3:1.4:1.5:1.6:1.7:[illegible]	1.1	1.9	1.1E-05	0.282	0.427	5.6E-02	1.4E-04	4.2E-03
ACC	IL32_ES_1.3:1.4:1.5_1.1_1.9	IL32	ES	1.3:1.4:1.5	1.1	1.9	1.6E-04	0.215	0.349	1.4E-01	4.0E-02	4.9E-02
ACC	INO80E_ES_6.1:6.2:6.3_5_11	INO80E	ES	6.1:6.2:6.3	5	11	5.9E-05	0.208	-0.055	7.9E-05	4.8E-06	1.1E-03
ACC	INO80E_ES_6.2:6.3_5_11	INO80E	ES	6.2:6.3	5	11	7.4E-04	0.153	0.168	9.4E-06	1.1E-05	1.5E-03
ACC	INSR_ES_11_10_12	INSR	ES	11	10	12	2.5E-05	0.290	-0.170	4.8E-01	3.2E-01	4.0E-02
ACC	KDM1A_ES_3_2_4	KDM1A	ES	3	2	4	5.8E-04	0.197	0.016	7.3E-01	2.0E-01	3.4E-01
ACC	LDB2_ES_9.2:10_9.1_11.2	LDB2	ES	9.2:10	9.1	11.2	8.0E-04	0.172	-0.510	4.8E-03	6.5E-03	2.2E-02
ACC	LEF1_ES_7_6.1_8	LEF1	ES	7	6.1	8	3.4E-05	0.274	-0.260	5.4E-03	4.4E-05	1.3E-03
ACC	LGALS9_ES_5_4_6	LGALS9	ES	5	4	6	4.7E-04	0.171	-0.373	1.2E-02	1.6E-01	4.1E-01
ACC	LMBR1L_ES_4.1:4.2_3_5	LMBR1L	ES	4.1:4.2	3	5	4.4E-04	0.169	-0.075	1.1E-02	1.5E-02	3.3E-02
ACC	MACF1_ES_103_102_104	MACF1	ES	103	102	104	4.7E-07	0.307	0.109	5.8E-02	2.0E-02	9.7E-02
ACC	MACF1_ES_107_106_108	MACF1	ES	107	106	108	6.2E-07	0.299	0.101	9.7E-03	3.6E-02	7.0E-02
ACC	MAPK9_ES_3_2_4	MAPK9	ES	3	2	4	1.7E-04	0.199	-0.222	7.5E-03	4.7E-03	1.1E-01
ACC	MBNL2_ES_10_8_11	MBNL2	ES	10	8	11	8.8E-04	0.194	-0.072	3.4E-01	4.8E-02	8.1E-01
ACC	MDH2_ES_4_1_5	MDH2	ES	4	1	5	3.2E-05	0.254	-0.149	1.5E-04	2.0E-03	2.2E-03
ACC	MELK_ES_8_7_9	MELK	ES	8	7	9	2.8E-04	0.220	-0.634	2.1E-03	2.9E-03	2.3E-02
ACC	METTL5_AD_1.2_1.1_1.4	METTL5	AD	1.2	1.1	1.4	1.7E-05	0.236	0.130	1.3E-02	4.9E-03	2.6E-02
ACC	METTL5_RI_1.2:1.3_1.1_1.4	METTL5	RI	1.2:1.3	1.1	1.4	9.2E-04	0.150	0.108	9.1E-02	1.5E-01	4.6E-01
ACC	MGRN1_ES_17.1_16_17.3	MGRN1	ES	17.1	16	17.3	3.9E-04	0.176	0.213	9.4E-01	1.9E-01	7.1E-01
ACC	MLH3_ES_7_6_8	MLH3	ES	7	6	8	2.4E-04	0.180	-0.645	3.7E-01	1.1E-01	1.0E-01
ACC	MPV17_AA_6.1:6.2_3.2_6.3	MPV17	AA	6.1:6.2	3.2	6.3	4.7E-04	0.191	-0.074	4.7E-01	4.5E-01	6.3E-01
ACC	MRPL55_AD_2.3:2.4:2.5:2.6_2.2_2.9	MRPL55	AD	2.3:2.4:2.5:2.6	2.2	2.9	6.8E-05	0.205	-0.311	6.1E-01	4.7E-01	3.1E-01

cancer type	id	Gene Symbol	splice_type	Exon	From.Exon	To.Exon	anova.p	adj.r2	r	p.50	p.25	p.10
ACC	MTCH1_AD_8.2_8.1_9	MTCH1	AD	8.2	8.1	9	1.1E-04	0.195	-0.235	3.2E-02	4.3E-02	3.7E-02
ACC	MYL6_ES_2.1_1.4_3.1	MYL6	ES	2.1	1.4	3.1	1.8E-05	0.235	-0.154	1.4E-05	2.6E-05	2.4E-04
ACC	MYL6_RI_1.2_1.1_1.3	MYL6	RI	1.2	1.1	1.3	2.3E-04	0.181	0.071	3.0E-01	1.4E-01	9.8E-02
ACC	NCOR2_AD_46.2_46.1_47	NCOR2	AD	46.2	46.1	47	3.9E-04	0.167	-0.152	1.9E-01	2.2E-01	3.8E-01
ACC	NFE2L1_ES_6_5.2_7	NFE2L1	ES	6	5.2	7	3.5E-06	0.266	0.247	1.1E-04	6.8E-05	8.5E-04
ACC	NUMA1_ES_18_17_19	NUMA1	ES	18	17	19	2.4E-06	0.276	0.117	2.1E-02	4.6E-03	1.1E-01
ACC	OBSL1_ES_13_12_14.1	OBSL1	ES	13	12	14.1	2.4E-04	0.182	0.217	5.4E-02	1.6E-03	1.1E-02
ACC	PAXBP1_AD_8.2_8.1_9	PAXBP1	AD	8.2	8.1	9	5.5E-04	0.185	0.633	8.1E-01	6.9E-01	1.8E-01
ACC	PCBP2_ES_15_14.1_16.2	PCBP2	ES	15	14.1	16.2	2.9E-04	0.178	0.106	8.3E-01	9.8E-01	7.0E-01
ACC	PDCD10_ES_4.2_1.1_5	PDCD10	ES	4.2	1.1	5	7.0E-04	0.160	0.118	3.7E-01	3.7E-01	3.7E-01
ACC	PEX5_ES_9_8_10	PEX5	ES	9	8	10	7.4E-04	0.159	-0.042	1.1E-01	1.6E-01	1.1E-01
ACC	PFDN5_ES_2_1_5	PFDN5	ES	2	1	5	1.8E-05	0.232	-0.001	5.0E-02	1.7E-03	1.4E-01
ACC	PI4KB_ES_5_4_6	PI4KB	ES	5	4	6	3.8E-05	0.246	0.133	3.8E-03	1.4E-01	4.7E-02
ACC	PILRB_AA_12.1_11_12.2	PILRB	AA	12.1	11	12.2	3.6E-04	0.169	0.075	9.8E-02	5.4E-03	2.8E-03
ACC	PILRB_RI_8.4_8.3_8.5	PILRB	RI	8.4	8.3	8.5	7.3E-04	0.153	0.202	8.7E-06	2.8E-06	3.4E-03
ACC	PKMYT1_AA_9.1:9.2_8_9.3	PKMYT1	AA	9.1:9.2	8	9.3	4.5E-04	0.166	-0.655	1.6E-03	1.1E-02	1.6E-02
ACC	POLL_RI_1.2:1.3:1.4_1.1_1.5	POLL	RI	1.2:1.3:1.4	1.1	1.5	6.5E-04	0.160	0.033	6.9E-02	1.5E-01	1.3E-01
ACC	POMT1_AD_8.2_8.1_9	POMT1	AD	8.2	8.1	9	3.8E-04	0.193	0.204	3.8E-01	2.6E-01	8.5E-02
ACC	PPAP2A_ES_2_1_4	PPAP2A	ES	2	1	4	2.0E-04	0.236	0.014	8.3E-03	9.0E-03	1.8E-02
ACC	PQLC2_RI_1.2_1.1_1.3	PQLC2	RI	1.2	1.1	1.3	6.2E-04	0.161	-0.041	2.2E-03	8.2E-03	4.4E-02
ACC	PRKDC_ES_81_80_82	PRKDC	ES	81	80	82	1.2E-05	0.254	-0.386	9.5E-03	8.3E-03	2.9E-01
ACC	PSTPIP1_ES_3_2.2_5	PSTPIP1	ES	3	2.2	5	1.7E-04	0.203	-0.139	4.3E-01	8.0E-02	1.2E-01
ACC	PTOV1_RI_13.2_13.1_13.3	PTOV1	RI	13.2	13.1	13.3	7.5E-07	0.295	-0.222	1.2E-01	1.7E-01	3.4E-02
ACC	R3HDM2_ES_12:13_11_15	R3HDM2	ES	12:13	11	15	5.4E-08	0.358	0.006	3.3E-01	5.0E-01	4.3E-01
ACC	RAB1A_ES_5_4_7	RAB1A	ES	5	4	7	1.2E-05	0.287	-0.165	9.2E-03	4.3E-02	4.0E-01
ACC	RAB40C_ES_6_5_7	RAB40C	ES	6	5	7	6.9E-04	0.169	-0.060	1.1E-02	1.6E-01	1.8E-01
ACC	RBMS1_ES_13_12_14	RBMS1	ES	13	12	14	2.3E-04	0.218	-0.109	9.6E-01	4.9E-01	8.5E-01
ACC	RHOT1_ES_19.3_19.1_20	RHOT1	ES	19.3	19.1	20	4.4E-04	0.177	-0.051	5.3E-02	9.1E-03	9.6E-01
ACC	RNF220_AD_9.2_9.1_10.1	RNF220	AD	9.2	9.1	10.1	4.2E-05	0.215	-0.197	2.1E-02	3.3E-01	1.6E-01
ACC	RNPS1_ES_3_1.1_4	RNPS1	ES	3	1.1	4	9.0E-04	0.148	-0.047	9.1E-04	4.3E-04	4.9E-03
ACC	ROMO1_RI_1.2:1.3:1.4_1.1_1.5	ROMO1	RI	1.2:1.3:1.4	1.1	1.5	1.2E-08	0.368	0.066	1.3E-02	3.4E-02	8.1E-02
ACC	RPAIN_ES_4:5:6.1_3_7	RPAIN	ES	05:06.1	3	7	1.4E-04	0.190	-0.221	1.3E-01	1.2E-01	4.4E-01
ACC	RPAIN_ES_6.1_5_7	RPAIN	ES	6.1	5	7	3.7E-06	0.264	-0.289	6.6E-01	9.4E-01	4.8E-01
ACC	RPL35_AD_4.2_4.1_5	RPL35	AD	4.2	4.1	5	4.5E-04	0.166	0.132	3.0E-01	4.5E-01	4.0E-01
ACC	RTN2_ES_5_4.1_6	RTN2	ES	5	4.1	6	6.3E-04	0.176	0.123	1.5E-01	6.7E-02	1.8E-01
ACC	SCP2_ES_12_11_13	SCP2	ES	12	11	13	8.6E-04	0.150	-0.404	1.8E-04	1.4E-03	3.5E-03
ACC	SEC61G_AD_2.2:2.3_2.1_3	SEC61G	AD	2.2:2.3	2.1	3	2.7E-05	0.227	0.402	1.3E-01	4.2E-03	5.6E-02
ACC	SLC15A4_RI_3.3_3.2_3.4	SLC15A4	RI	3.3	3.2	3.4	4.3E-04	0.188	0.101	1.3E-01	5.0E-01	9.5E-01
ACC	SLC9B2_ES_16_15_17.1	SLC9B2	ES	16	15	17.1	5.7E-04	0.181	-0.043	7.9E-01	7.3E-01	8.1E-01
ACC	SMARCC2_ES_18_17_19	SMARCC2	ES	18	17	19	8.3E-04	0.152	0.326	2.4E-02	5.3E-02	7.8E-02

cancer type	id	Gene Symbol	splice_type	Exon	From.Exon	To.Exon	anova.p	adj.r2	r	p.50	p.25	p.10
ACC	SMUG1_ES_2.2:2.3:3_1.1_4.1	SMUG1	ES	2.2:2.3:3	1.1	4.1	9.8E-04	0.146	-0.157	1.5E-02	1.0E-02	2.0E-02
ACC	SMUG1_ES_2.3:3_2.2_4.1	SMUG1	ES	2.3:3	2.2	4.1	8.7E-06	0.266	-0.150	4.0E-05	6.5E-04	5.8E-03
ACC	SREK1_ES_4_3.2_5	SREK1	ES	4	3.2	5	9.5E-05	0.213	0.475	8.9E-01	6.8E-01	3.9E-01
ACC	STAR_ES_5_4_7	STAR	ES	5	4	7	4.2E-08	0.355	-0.442	2.3E-03	1.3E-05	2.5E-02
ACC	STRADA_ES_3_2.2_6	STRADA	ES	3	2.2	6	1.7E-04	0.236	-0.210	2.2E-01	3.3E-01	5.9E-03
ACC	SUOX_ES_3_2_5.2	SUOX	ES	3	2	5.2	8.9E-05	0.202	-0.514	4.7E-03	2.6E-04	2.0E-02
ACC	SYNE1_ES_151_150_152	SYNE1	ES	151	150	152	5.1E-04	0.174	-0.029	9.9E-01	4.4E-01	7.8E-01
ACC	TANK_ES_2.2:3_2.1_4	TANK	ES	2.2:3	2.1	4	2.3E-04	0.206	-0.333	1.0E-03	5.1E-04	7.9E-04
ACC	TANK_ES_3_2.2_4	TANK	ES	3	2.2	4	7.9E-05	0.215	-0.403	8.3E-05	6.5E-05	7.3E-04
ACC	TCERG1_ES_22_21_23	TCERG1	ES	22	21	23	2.4E-04	0.178	0.216	1.1E-03	4.6E-04	8.5E-03
ACC	THNSL2_ES_9_8_11	THNSL2	ES	9	8	11	1.8E-07	0.399	0.249	4.9E-04	3.4E-04	6.4E-03
ACC	THNSL2_ME_9|10_8_11	THNSL2	ME	9|10	8	11	6.7E-05	0.261	0.372	4.6E-06	8.0E-05	1.7E-03
ACC	THYN1_RI_1.2:1.3_1.1_1.4	THYN1	RI	1.2:1.3	1.1	1.4	3.8E-04	0.170	-0.217	5.4E-03	1.1E-01	1.2E-02
ACC	TLK2_ES_10_9_11	TLK2	ES	10	9	11	5.2E-04	0.189	0.074	3.0E-01	3.6E-01	5.6E-01
ACC	TM2D3_ES_1.3_1.1_2.2	TM2D3	ES	1.3	1.1	2.2	7.3E-04	0.163	-0.217	5.2E-02	2.1E-02	2.6E-01
ACC	TMBIM6_AD_2.3_2.2_3.2	TMBIM6	AD	2.3	2.2	3.2	1.2E-04	0.222	-0.098	6.5E-02	5.8E-02	6.9E-02
ACC	TMEM144_RI_1.2_1.1_1.3	TMEM144	RI	1.2	1.1	1.3	4.0E-04	0.190	-0.110	5.6E-01	8.0E-01	3.7E-01
ACC	TMEM150A_ES_6_5_7	TMEM150A	ES	6	5	7	2.6E-04	0.181	0.057	5.5E-01	9.5E-01	4.3E-01
ACC	TMEM175_ES_2:3_1_5.1	TMEM175	ES	2:03	1	5.1	6.5E-05	0.211	-0.060	4.4E-01	7.7E-02	3.8E-01
ACC	TPD52L1_ES_8:9.1_6_10	TPD52L1	ES	08:09.1	6	10	1.1E-04	0.195	-0.020	8.1E-01	7.5E-01	9.8E-01
ACC	TRAFD1_AD_1.2_1.1_2	TRAFD1	AD	1.2	1.1	2	2.1E-04	0.218	-0.104	9.9E-07	8.8E-05	1.4E-04
ACC	TRAPPC6A_AD_1.2_1.1_2	TRAPPC6A	AD	1.2	1.1	2	1.3E-04	0.198	0.082	2.8E-03	2.9E-03	2.2E-02
ACC	TRAPPC6A_ES_1.2:2_1.1_3	TRAPPC6A	ES	1.2:2	1.1	3	4.8E-04	0.169	0.241	4.4E-04	5.2E-05	6.1E-04
ACC	TTC31_RI_5.2_5.1_5.3	TTC31	RI	5.2	5.1	5.3	3.4E-04	0.184	0.097	1.3E-01	9.5E-01	9.5E-01
ACC	U2AF1L4_ES_8_7_9.1	U2AF1L4	ES	8	7	9.1	8.2E-04	0.150	-0.077	1.6E-01	2.8E-01	1.9E-01
ACC	UBL7_AD_1.2_1.1_3	UBL7	AD	1.2	1.1	3	3.5E-05	0.230	-0.020	5.2E-06	1.7E-03	3.0E-02
ACC	VCPKMT_ES_5_4_6	VCPKMT	ES	5	4	6	7.6E-05	0.240	-0.257	5.3E-02	3.0E-01	9.9E-01
ACC	VPS29_ES_3.1_1_5	VPS29	ES	3.1	1	5	5.5E-04	0.160	0.235	1.8E-01	2.7E-02	2.3E-01
ACC	WRAP73_RI_11.2_11.1_11.3	WRAP73	RI	11.2	11.1	11.3	9.4E-04	0.147	0.072	2.1E-01	7.6E-01	1.6E-01
ACC	ZDHHC4_AA_2.1_1.1_2.2	ZDHHC4	AA	2.1	1.1	2.2	8.0E-04	0.163	0.193	1.7E-01	4.2E-01	2.8E-01
ACC	ZDHHC4_AD_1.2:1.3_1.1_2.2	ZDHHC4	AD	1.2:1.3	1.1	2.2	7.9E-04	0.161	-0.074	4.2E-01	1.5E-01	7.6E-02
ACC	ZFP2_ME_6|7_3_8	ZFP2	ME	6|7	3	8	3.3E-04	0.200	0.412	5.1E-02	3.4E-01	1.6E-01
ACC	ZFYVE19_ES_8_7_9	ZFYVE19	ES	8	7	9	8.0E-04	0.151	-0.263	2.6E-01	9.5E-02	4.3E-01
ACC	ZNF10_AD_1.2_1.1_2.2	ZNF10	AD	1.2	1.1	2.2	7.0E-06	0.288	-0.099	3.7E-01	2.4E-01	5.7E-01
ACC	ZNF189_ES_2_1_3.2	ZNF189	ES	2	1	3.2	3.8E-04	0.214	-0.121	4.7E-01	2.5E-01	1.3E-01
ACC	ZNF576_RI_1.4:1.5_1.3_1.6	ZNF576	RI	1.4:1.5	1.3	1.6	3.8E-04	0.199	0.356	4.8E-02	3.2E-01	8.6E-01
ACC	ZNF91_ES_3_2_4	ZNF91	ES	3	2	4	4.3E-04	0.178	-0.178	7.0E-01	1.0E-01	1.3E-01
ACC	ZSWIM7_RI_7.6_7.5_7.7	ZSWIM7	RI	7.6	7.5	7.7	3.0E-04	0.175	0.070	8.9E-02	9.8E-02	8.8E-02
BLCA	ABCB8_RI_6.2_6.1_6.3	ABCB8	RI	6.2	6.1	6.3	1.4E-04	0.131	-0.045	5.5E-03	1.1E-01	1.1E-02
BLCA	ABCC5_RI_7.5_7.4_7.6	ABCC5	RI	7.5	7.4	7.6	6.2E-04	0.109	0.217	9.9E-02	3.5E-01	9.7E-02

cancer type	id	Gene Symbol	splice_type	Exon	From.Exon	To.Exon	anova.p	adj.r2	r	p.50	p.25	p.10
BLCA	ABCE1_ES_14_13_15	ABCE1	ES	14	13	15	1.5E-05	0.162	-0.562	4.4E-02	6.0E-03	3.1E-02
BLCA	ABI1_ES_5_4_7	ABI1	ES	5	4	7	9.3E-10	0.290	-0.304	7.6E-01	1.0E+00	9.1E-01
BLCA	ACAA1_AD_9.2:9.3_9.1_10	ACAA1	AD	9.2:9.3	9.1	10	9.9E-08	0.229	0.289	1.8E-02	8.7E-03	1.9E-03
BLCA	ACAD9_AD_1.2_1.1_2.1	ACAD9	AD	1.2	1.1	2.1	6.7E-04	0.108	-0.094	4.5E-02	3.3E-01	1.4E-01
BLCA	ACOT9_ES_6_5.1_7.1	ACOT9	ES	6	5.1	7.1	2.7E-07	0.233	0.227	1.0E-01	5.6E-02	2.0E-01
BLCA	ACSL1_ME_16|17_15_18.1	ACSL1	ME	16|17	15	18.1	2.2E-05	0.158	-0.221	1.2E-01	1.3E-01	2.5E-02
BLCA	ACTG1_RI_1.3:1.4_1.2_1.5	ACTG1	RI	1.3:1.4	1.2	1.5	5.9E-05	0.143	-0.256	1.4E-01	5.2E-03	4.0E-03
BLCA	ACTN1_ES_20_19_21	ACTN1	ES	20	19	21	7.2E-05	0.149	0.128	2.1E-01	3.6E-01	3.1E-01
BLCA	ACTR10_ES_3_2_4	ACTR10	ES	3	2	4	6.3E-04	0.108	-0.415	4.0E-03	1.4E-03	1.4E-02
BLCA	ACY1_ES_8.1:8.2:9:10:11:12_7.2_15	ACY1	ES	3.1:8.2:9:10:11:1	7.2	15	5.9E-04	0.141	-0.337	3.9E-01	2.7E-02	1.4E-01
BLCA	ADAM15_AD_22.2_22.1_23	ADAM15	AD	22.2	22.1	23	3.3E-04	0.119	-0.266	5.5E-01	4.2E-01	3.9E-01
BLCA	ADAM15_ES_21.1:21.2:22.1_20_23	ADAM15	ES	21.1:21.2:22.1	20	23	4.4E-04	0.115	0.218	8.3E-01	4.0E-01	7.3E-01
BLCA	ADAM15_ES_21.2:22.1_20_23	ADAM15	ES	21.2:22.1	20	23	7.4E-05	0.141	0.213	9.6E-01	4.1E-01	6.6E-01
BLCA	ADAM15_ES_22.1_20_23	ADAM15	ES	22.1	20	23	1.8E-04	0.128	0.322	5.2E-01	4.4E-01	8.2E-01
BLCA	ADCK5_RI_9.2_9.1_9.3	ADCK5	RI	9.2	9.1	9.3	3.3E-04	0.120	0.290	2.9E-02	7.2E-03	1.2E-02
BLCA	ADD3_ES_15_14_16	ADD3	ES	15	14	16	5.9E-07	0.208	0.137	1.9E-01	1.2E-01	3.1E-01
BLCA	AFMID_ES_7:8:9:10:11.1:11.2_6_12	AFMID	ES	7:8:9:10:11.1:11.	6	12	9.3E-04	0.102	0.114	2.7E-03	5.0E-03	2.0E-01
BLCA	AFMID_ES_7:8:9:10:11.1:11.2:12_6_13	AFMID	ES	8:9:10:11.1:11.2:	6	13	1.0E-05	0.204	0.131	2.6E-03	2.2E-02	8.4E-01
BLCA	AFMID_ES_7:8:9:10:11.1:12_6_13	AFMID	ES	7:8:9:10:11.1:12	6	13	1.9E-04	0.154	0.077	2.4E-03	3.7E-01	6.6E-01
BLCA	AGPAT4_RI_5.2_5.1_5.3	AGPAT4	RI	5.2	5.1	5.3	5.6E-04	0.125	-0.243	5.0E-01	7.9E-02	4.0E-01
BLCA	AKAP8L_ES_10_9_11	AKAP8L	ES	10	9	11	2.0E-05	0.159	0.330	7.2E-02	1.4E-04	3.3E-03
BLCA	AKR1E2_ES_5:6:7_4_8	AKR1E2	ES	5:06:07	4	8	9.0E-04	0.142	-0.128	2.4E-01	1.9E-01	2.5E-01
BLCA	ALS2CL_RI_18.5_18.4_18.6	ALS2CL	RI	18.5	18.4	18.6	1.9E-04	0.126	0.249	9.6E-03	4.0E-02	3.4E-01
BLCA	AMIGO2_ES_2.1_1_3.1	AMIGO2	ES	2.1	1	3.1	1.1E-05	0.188	0.176	3.2E-01	9.4E-01	1.9E-01
BLCA	AMIGO2_ES_2.1:2.2_1_3.1	AMIGO2	ES	2.1:2.2	1	3.1	4.5E-05	0.150	0.218	2.2E-02	6.3E-02	3.3E-02
BLCA	ANKLE2_RI_1.2_1.1_1.3	ANKLE2	RI	1.2	1.1	1.3	4.8E-04	0.112	-0.205	2.5E-01	2.6E-01	1.0E-01
BLCA	ANKRD49_AD_2.3:2.4:2.5:2.6_2.2_2.8	ANKRD49	AD	2.3:2.4:2.5:2.6	2.2	2.8	1.9E-04	0.127	0.349	9.1E-01	5.2E-01	9.2E-02
BLCA	ANKRD49_RI_2.3:2.4:2.5:2.6:2.7_2.2_2.	ANKRD49	RI	2.3:2.4:2.5:2.6:2.	2.2	2.8	3.8E-05	0.154	0.422	1.6E-01	1.6E-01	1.1E-03
BLCA	ANXA6_ES_22_21_23	ANXA6	ES	22	21	23	7.2E-05	0.140	0.112	3.7E-01	1.1E-01	1.5E-01
BLCA	AP1B1_ES_24_23_25	AP1B1	ES	24	23	25	1.4E-17	0.468	-0.338	2.2E-03	2.0E-01	2.5E-01
BLCA	AP1G2_AD_1.2_1.1_1.4	AP1G2	AD	1.2	1.1	1.4	1.6E-04	0.130	0.237	2.2E-02	5.1E-01	5.4E-01
BLCA	AP1G2_RI_1.3_1.2_1.4	AP1G2	RI	1.3	1.2	1.4	4.7E-06	0.184	-0.377	9.6E-01	9.9E-01	6.7E-01
BLCA	AP2M1_ES_7_6_8	AP2M1	ES	7	6	8	1.8E-08	0.251	-0.170	3.9E-01	5.9E-01	2.0E-01
BLCA	ARFGAP1_ES_13_12_14.1	ARFGAP1	ES	13	12	14.1	1.2E-06	0.198	0.238	2.9E-02	2.3E-02	2.2E-02
BLCA	ARFIP1_ES_4_3_5	ARFIP1	ES	4	3	5	9.0E-06	0.171	0.243	5.6E-02	2.3E-01	2.8E-01
BLCA	ARFIP2_ES_6_5.2_7	ARFIP2	ES	6	5.2	7	4.0E-05	0.149	-0.399	1.6E-03	3.5E-03	1.6E-02
BLCA	ARGLU1_ES_4_3_5	ARGLU1	ES	4	3	5	1.8E-06	0.192	0.701	1.1E-02	2.4E-03	1.6E-01
BLCA	ARHGAP17_ES_18_17_19	ARHGAP17	ES	18	17	19	1.1E-04	0.135	0.245	3.6E-01	5.0E-03	1.7E-01
BLCA	ARHGEF1_ES_15_14_16	ARHGEF1	ES	15	14	16	4.6E-04	0.113	0.280	4.2E-04	1.3E-06	2.9E-02
BLCA	ARHGEF10L_ES_18_17_19	ARHGEF10L	ES	18	17	19	1.9E-22	0.566	0.462	2.1E-01	1.8E-01	2.7E-01

cancer type	id	Gene Symbol	splice_type	Exon	From.Exon	To.Exon	anova.p	adj.r2	r	p.50	p.25	p.10
BLCA	ARHGEF11_ES_39_38_40	ARHGEF11	ES	39	38	40	6.2E-10	0.295	-0.213	6.1E-01	7.4E-01	8.9E-01
BLCA	ARIH2_ES_5_4.2_6	ARIH2	ES	5	4.2	6	5.6E-04	0.111	0.186	1.4E-01	2.9E-01	4.4E-01
BLCA	ARL16_ES_3:4_2.3_5	ARL16	ES	3:04	2.3	5	8.4E-05	0.138	0.221	1.2E-02	3.2E-03	3.1E-01
BLCA	ARL16_ES_4_3_5	ARL16	ES	4	3	5	2.8E-07	0.216	0.269	1.4E-02	1.6E-02	3.4E-04
BLCA	ARMC5_RI_5.2_5.1_5.3	ARMC5	RI	5.2	5.1	5.3	1.2E-04	0.133	-0.376	3.6E-02	1.8E-01	1.7E-02
BLCA	ARVCF_ES_20_19_21	ARVCF	ES	20	19	21	9.6E-07	0.219	0.356	1.4E-01	5.5E-02	3.1E-01
BLCA	ASCC2_ES_6:7_5_8	ASCC2	ES	6:07	5	8	4.4E-04	0.116	0.265	4.8E-01	4.1E-01	9.5E-01
BLCA	ASPHD1_RI_1.2_1.1_1.3	ASPHD1	RI	1.2	1.1	1.3	1.8E-05	0.173	-0.185	7.5E-01	9.4E-01	9.9E-01
BLCA	ATAD3A_AA_4.1_3_4.2	ATAD3A	AA	4.1	3	4.2	2.7E-04	0.121	-0.293	5.1E-04	2.4E-03	1.1E-04
BLCA	ATF3_AA_5.1:5.2_4_5.3	ATF3	AA	5.1:5.2	4	5.3	5.4E-04	0.110	-0.046	4.8E-01	7.8E-01	7.1E-01
BLCA	ATG4B_AD_14.2:14.3_14.1_15	ATG4B	AD	14.2:14.3	14.1	15	5.9E-04	0.109	0.253	1.1E-02	4.3E-04	5.2E-03
BLCA	ATG4D_ES_4.1:5_3.2_6	ATG4D	ES	4.1:5	3.2	6	2.4E-05	0.186	0.170	4.7E-02	3.2E-02	4.0E-02
BLCA	ATL2_ES_3_1_5	ATL2	ES	3	1	5	7.2E-05	0.140	-0.492	2.7E-01	4.6E-01	1.6E-01
BLCA	ATP11C_ES_32_29_33	ATP11C	ES	32	29	33	2.9E-09	0.275	0.026	1.4E-02	2.7E-01	9.2E-01
BLCA	ATP2B4_ES_21_20_22	ATP2B4	ES	21	20	22	5.3E-07	0.211	-0.082	6.9E-01	9.0E-01	1.4E-01
BLCA	ATP5J_AD_1.3_1.2_3	ATP5J	AD	1.3	1.2	3	7.5E-06	0.172	-0.060	4.1E-01	5.7E-01	7.6E-01
BLCA	ATP5J_AD_1.3:1.4_1.2_3	ATP5J	AD	1.3:1.4	1.2	3	4.3E-04	0.115	-0.078	7.3E-01	6.7E-01	9.9E-01
BLCA	ATP5J_AD_1.3:1.4:1.5_1.2_3	ATP5J	AD	1.3:1.4:1.5	1.2	3	4.4E-08	0.240	0.019	8.3E-01	7.7E-01	2.0E-01
BLCA	ATP6V0B_ES_2.1:2.2_1.1_3.2	ATP6V0B	ES	2.1:2.2	1.1	3.2	1.4E-05	0.164	-0.135	1.5E-04	8.7E-03	4.9E-03
BLCA	ATXN2_ES_21_20_22	ATXN2	ES	21	20	22	5.9E-04	0.109	-0.043	6.4E-01	8.6E-01	3.8E-01
BLCA	B4GALT2_AD_2.2_2.1_3	B4GALT2	AD	2.2	2.1	3	1.7E-04	0.128	-0.257	2.5E-01	1.9E-01	1.9E-01
BLCA	BAIAP2_ES_17.1_16.1_18.1	BAIAP2	ES	17.1	16.1	18.1	8.0E-05	0.146	0.210	5.7E-01	5.5E-01	5.3E-01
BLCA	BAIAP2_ES_17.1:17.2_16.1_18.1	BAIAP2	ES	17.1:17.2	16.1	18.1	6.4E-07	0.208	0.216	9.6E-01	7.9E-01	5.0E-01
BLCA	BAIAP2_RI_16.2_16.1_16.3	BAIAP2	RI	16.2	16.1	16.3	6.5E-04	0.111	-0.316	1.6E-01	7.1E-02	8.0E-01
BLCA	BCS1L_AD_1.2:1.3:1.4:1.5:1.6:1.7:1.8_1.	BCS1L	AD	1.3:1.4:1.5:1.6:1.	1.1	2	3.1E-04	0.119	0.157	2.4E-03	6.5E-04	4.8E-02
BLCA	BCS1L_AD_1.5:1.6:1.7:1.8_1.4_2	BCS1L	AD	1.5:1.6:1.7:1.8	1.4	2	4.6E-04	0.114	0.023	6.1E-02	3.9E-01	2.4E-01
BLCA	BICD2_RI_7.2_7.1_7.3	BICD2	RI	7.2	7.1	7.3	3.1E-04	0.119	-0.270	5.5E-01	8.4E-02	9.8E-01
BLCA	BMP1_ES_17.1:17.2_16_18.1	BMP1	ES	17.1:17.2	16	18.1	5.1E-16	0.439	-0.482	1.1E-03	2.4E-02	7.2E-01
BLCA	BMP1_ES_17.1:17.2:18.1_16_18.2	BMP1	ES	17.1:17.2:18.1	16	18.2	3.9E-08	0.241	-0.262	3.4E-02	2.7E-02	3.9E-02
BLCA	BOP1_RI_4.2_4.1_4.3	BOP1	RI	4.2	4.1	4.3	1.8E-06	0.192	-0.412	5.6E-02	6.5E-03	2.4E-02
BLCA	BTAF1_ES_25:26.1_24_26.2	BTAF1	ES	25:26.1	24	26.2	1.3E-04	0.144	0.141	3.6E-03	1.3E-02	3.9E-02
BLCA	BTBD3_AA_2.1:2.2_1_2.3	BTBD3	AA	2.1:2.2	1	2.3	3.7E-05	0.158	0.218	3.3E-01	8.6E-02	6.3E-02
BLCA	BTBD3_RI_2.2_2.1_2.3	BTBD3	RI	2.2	2.1	2.3	5.6E-04	0.118	0.088	3.3E-01	2.1E-01	3.1E-01
BLCA	BTN2A1_AA_9.1_8_9.2	BTN2A1	AA	9.1	8	9.2	4.0E-07	0.212	0.123	8.3E-02	1.1E-01	1.5E-01
BLCA	BTN2A1_ES_4_3_5	BTN2A1	ES	4	3	5	3.1E-05	0.152	-0.094	9.6E-02	5.1E-03	2.1E-01
BLCA	BTN3A1_AA_10.1:10.2_9_10.3	BTN3A1	AA	10.1:10.2	9	10.3	2.5E-05	0.158	-0.141	7.2E-01	5.1E-01	6.9E-01
BLCA	C12orf43_ES_2_1_3.1	C12orf43	ES	2	1	3.1	2.7E-04	0.121	-0.338	2.8E-01	5.7E-02	1.0E-01
BLCA	C14orf80_ES_9_8_10	C14orf80	ES	9	8	10	8.2E-04	0.104	-0.186	3.8E-03	1.4E-01	2.1E-01
BLCA	C16orf58_RI_8.3_8.2_8.4	C16orf58	RI	8.3	8.2	8.4	1.3E-05	0.165	0.209	1.2E-03	1.1E-02	1.3E-01
BLCA	C19orf48_RI_5.2_5.1_5.3	C19orf48	RI	5.2	5.1	5.3	5.8E-05	0.143	-0.010	6.7E-01	2.6E-01	2.7E-02

cancer type	id	Gene Symbol	splice_type	Exon	From.Exon	To.Exon	anova.p	adj.r2	r	p.50	p.25	p.10
BLCA	C1orf159_AD_11.2:11.3_11.1_12	C1orf159	AD	11.2:11.3	11.1	12	2.0E-06	0.190	0.411	3.6E-03	8.7E-03	1.1E-04
BLCA	C1orf159_RI_11.2_11.1_11.3	C1orf159	RI	11.2	11.1	11.3	4.7E-04	0.113	0.026	1.2E-01	9.8E-01	5.6E-01
BLCA	C1orf162_AA_5.1_4_5.2	C1orf162	AA	5.1	4	5.2	3.8E-05	0.165	-0.316	8.7E-01	9.3E-01	8.6E-01
BLCA	C1orf63_ES_5.1:5.2:5.3_4.2_6.2	C1orf63	ES	5.1:5.2:5.3	4.2	6.2	7.5E-05	0.140	-0.119	3.7E-01	4.9E-01	3.3E-01
BLCA	C1orf63_ES_5.1:5.2:6.1_4.2_6.2	C1orf63	ES	5.1:5.2:6.1	4.2	6.2	3.0E-05	0.153	-0.123	6.3E-01	7.8E-01	3.3E-01
BLCA	C1RL_AA_5.1_4.2_5.2	C1RL	AA	5.1	4.2	5.2	9.6E-05	0.137	-0.085	8.8E-01	1.7E-02	2.0E-01
BLCA	C5orf45_RI_7.2_7.1_7.3	C5orf45	RI	7.2	7.1	7.3	2.1E-04	0.124	-0.103	3.0E-01	6.7E-03	1.9E-02
BLCA	C7orf49_AD_1.2_1.1_2.1	C7orf49	AD	1.2	1.1	2.1	7.0E-04	0.107	-0.106	2.1E-03	1.3E-02	2.3E-01
BLCA	CA12_ES_9_8_10	CA12	ES	9	8	10	3.4E-10	0.327	0.434	9.5E-02	2.5E-01	6.2E-01
BLCA	CADPS2_ES_25_24.1_27	CADPS2	ES	25	24.1	27	1.8E-09	0.320	-0.135	7.3E-01	2.4E-01	5.4E-01
BLCA	CALCOCO1_RI_14.3_14.2_14.4	CALCOCO1	RI	14.3	14.2	14.4	1.1E-06	0.199	0.064	7.7E-02	8.3E-03	8.4E-02
BLCA	CALCOCO2_ES_3_2_6	CALCOCO2	ES	3	2	6	9.2E-04	0.102	-0.546	1.1E-01	5.5E-03	7.3E-02
BLCA	CALD1_ES_8.3:9_8.2_10	CALD1	ES	8.3:9	8.2	10	6.5E-07	0.205	0.400	5.7E-01	3.5E-01	1.6E-01
BLCA	CAMKK2_ES_15_14_16	CAMKK2	ES	15	14	16	2.5E-04	0.122	0.004	3.4E-01	8.5E-01	3.4E-01
BLCA	CAMKK2_ES_17_16_19.1	CAMKK2	ES	17	16	19.1	4.8E-04	0.112	-0.030	8.5E-01	9.1E-01	8.0E-01
BLCA	CARD16_ES_4_3_5	CARD16	ES	4	3	5	8.1E-04	0.108	0.013	2.9E-01	7.3E-02	2.5E-02
BLCA	CARKD_RI_7.2_7.1_7.3	CARKD	RI	7.2	7.1	7.3	9.4E-04	0.102	0.141	7.2E-02	5.0E-02	5.1E-01
BLCA	CARKD_RI_7.4_7.3_7.5	CARKD	RI	7.4	7.3	7.5	2.9E-06	0.185	0.362	8.4E-03	1.1E-03	9.9E-03
BLCA	CARM1_RI_16.2_16.1_16.3	CARM1	RI	16.2	16.1	16.3	4.6E-04	0.115	0.097	3.0E-05	3.0E-03	3.5E-02
BLCA	CASK_ES_21_19.1_22	CASK	ES	21	19.1	22	1.0E-05	0.189	-0.010	7.4E-01	6.2E-01	2.4E-01
BLCA	CASP1_RI_10.2_10.1_10.3	CASP1	RI	10.2	10.1	10.3	2.6E-04	0.121	-0.205	1.0E-01	7.3E-01	9.1E-01
BLCA	CAST_ES_8.2_7.1_9	CAST	ES	8.2	7.1	9	1.5E-05	0.162	-0.319	3.1E-01	4.4E-01	9.9E-02
BLCA	CC2D2A_ES_5_4_6	CC2D2A	ES	5	4	6	3.1E-07	0.231	0.041	7.1E-01	4.2E-01	6.9E-02
BLCA	CCDC14_AD_2.7:2.8:2.9_2.6_3	CCDC14	AD	2.7:2.8:2.9	2.6	3	1.3E-04	0.142	0.251	4.3E-01	4.7E-01	2.2E-01
BLCA	CCDC14_RI_2.3_2.2_2.4	CCDC14	RI	2.3	2.2	2.4	1.1E-04	0.139	0.485	1.9E-01	4.7E-04	5.4E-01
BLCA	CCDC14_RI_2.5_2.4_2.6	CCDC14	RI	2.5	2.4	2.6	5.3E-06	0.185	0.484	3.7E-01	1.0E-02	1.7E-02
BLCA	CCDC50_ES_6_5_7	CCDC50	ES	6	5	7	2.0E-10	0.305	-0.098	8.1E-02	4.2E-01	8.3E-02
BLCA	CCDC51_AA_3.1_2.2_3.2	CCDC51	AA	3.1	2.2	3.2	9.8E-04	0.103	0.359	3.4E-02	5.7E-02	9.3E-01
BLCA	CCDC53_ME_4|5_2_6	CCDC53	ME	4|5	2	6	1.9E-04	0.126	0.153	7.0E-01	9.7E-02	1.1E-01
BLCA	CCNL1_ES_4.1_3_5	CCNL1	ES	4.1	3	5	4.9E-07	0.209	0.351	2.7E-02	8.6E-03	1.6E-01
BLCA	CCSER2_ES_11_10_12	CCSER2	ES	11	10	12	3.4E-05	0.152	0.100	9.6E-01	1.4E-01	4.0E-01
BLCA	CCT3_ES_3_1_4	CCT3	ES	3	1	4	5.7E-04	0.110	-0.185	6.1E-02	2.0E-02	4.6E-03
BLCA	CD44_ES_10:11:12.1:13:14_5_15	CD44	ES	10:11:12.1:13:14	5	15	2.7E-15	0.502	0.231	8.6E-01	6.2E-01	4.8E-01
BLCA	CD44_ES_12.1:13:14_5_15	CD44	ES	12.1:13:14	5	15	1.5E-06	0.197	0.276	3.9E-01	5.7E-01	3.2E-01
BLCA	CD44_ES_3.1:3.2:4:5:6:7:8:9.1:9.2:10:11	CD44	ES	9.2:10:11:12.1:13	2.1	17.2	7.8E-11	0.344	-0.173	5.8E-01	9.0E-01	2.6E-01
BLCA	CD44_ES_3.1:3.2:4:5:6:7:8:9.2:10:11:12.	CD44	ES	2:10:11:12.1:13:1	2.1	17.2	5.7E-14	0.437	-0.147	9.4E-01	7.0E-01	3.7E-01
BLCA	CD44_ES_3.1:3.2:4:5:7:8:9.1:9.2:10:11:1	CD44	ES	.2:10:11:12.1:13:	2.1	17.2	1.7E-05	0.174	-0.043	9.7E-01	8.2E-01	5.5E-01
BLCA	CD44_ES_3.1:3.2:4:5:7:8:9.2:10:11:12.1:	CD44	ES	:10:11:12.1:13:14	2.1	17.2	5.6E-05	0.157	-0.022	7.1E-01	5.6E-01	9.7E-01
BLCA	CD44_ES_6:7:8:9.1:9.2:10:11_5_12.1	CD44	ES	6:7:8:9.1:9.2:10:1	5	12.1	1.0E-05	0.179	-0.102	6.7E-01	1.2E-01	4.8E-01
BLCA	CD44_ES_6:7:8:9.1:9.2:10:11:12.1:13_5_	CD44	ES	:9.1:9.2:10:11:12	5	14	9.2E-09	0.280	-0.095	7.5E-01	9.3E-01	7.3E-01

cancer type	id	Gene Symbol	splice_type	Exon	From.Exon	To.Exon	anova.p	adj.r2	r	p.50	p.25	p.10
BLCA	CD44_ES_6:7:8:9.1:9.2:10:11:12.1:13:14	CD44	ES	.1:9.2:10:11:12.1	5	15	2.3E-11	0.333	0.279	6.8E-01	6.9E-01	1.3E-01
BLCA	CD44_ES_6:7:8:9.2:10:11_5_12.1	CD44	ES	6:7:8:9.2:10:11	5	12.1	9.5E-06	0.182	-0.099	5.9E-01	1.5E-01	4.2E-01
BLCA	CD44_ES_6:7:8:9.2:10:11:12.1:13_5_14	CD44	ES	:8:9.2:10:11:12.1	5	14	4.7E-08	0.263	-0.108	7.4E-01	9.6E-01	9.7E-01
BLCA	CD44_ES_6:7:8:9.2:10:11:12.1:13:14_5_	CD44	ES	:9.2:10:11:12.1:1	5	15	2.3E-10	0.307	0.298	5.3E-01	4.7E-01	4.3E-02
BLCA	CD44_ES_7:8:9.1:9.2:10:11_5_12.1	CD44	ES	7:8:9.1:9.2:10:11	5	12.1	1.3E-04	0.139	0.039	2.0E-01	2.2E-01	5.1E-01
BLCA	CD44_ES_7:8:9.1:9.2:10:11:12.1:13_5_1	CD44	ES	9.1:9.2:10:11:12.	5	14	2.3E-05	0.168	-0.039	4.8E-01	6.8E-01	4.7E-01
BLCA	CD44_ES_7:8:9.1:9.2:10:11:12.1:13:14_5	CD44	ES	1:9.2:10:11:12.1:	5	15	3.8E-14	0.398	0.391	9.1E-01	5.4E-01	1.4E-01
BLCA	CD44_ES_7:8:9.2:10:11_5_12.1	CD44	ES	7:8:9.2:10:11	5	12.1	3.2E-05	0.163	0.076	1.4E-01	2.4E-01	2.7E-01
BLCA	CD44_ES_7:8:9.2:10:11:12.1:13_5_14	CD44	ES	8:9.2:10:11:12.1:	5	14	3.3E-05	0.165	-0.048	7.1E-01	3.1E-01	9.2E-01
BLCA	CD44_ES_7:8:9.2:10:11:12.1:13:14_5_15	CD44	ES	9.2:10:11:12.1:13	5	15	7.4E-14	0.393	0.422	7.3E-01	5.9E-01	1.7E-02
BLCA	CD46_ES_13_12_14	CD46	ES	13	12	14	1.2E-06	0.197	-0.285	8.6E-01	7.3E-01	9.7E-01
BLCA	CD47_ES_8:9:10_7_11	CD47	ES	8:09:10	7	11	2.8E-07	0.236	-0.234	4.8E-01	1.7E-01	5.8E-01
BLCA	CD47_ES_9:10_8_11	CD47	ES	9:10	8	11	4.4E-08	0.240	-0.141	1.9E-01	3.4E-02	4.7E-02
BLCA	CD97_ES_5:6_4_7	CD97	ES	5:06	4	7	3.1E-04	0.121	-0.005	8.6E-01	4.1E-02	6.7E-02
BLCA	CDC37_RI_4.2_4.1_4.3	CDC37	RI	4.2	4.1	4.3	2.5E-06	0.187	0.361	2.5E-03	2.4E-03	1.9E-04
BLCA	CDC42SE1_ES_5_4_6	CDC42SE1	ES	5	4	6	2.0E-05	0.158	-0.010	3.0E-01	7.4E-02	3.0E-02
BLCA	CDC42SE2_RI_5.2_5.1_5.3	CDC42SE2	RI	5.2	5.1	5.3	4.1E-06	0.180	-0.331	6.4E-01	7.2E-01	4.9E-02
BLCA	CDCA7_ES_6_5_7	CDCA7	ES	6	5	7	5.7E-04	0.126	-0.316	1.7E-01	1.1E-01	3.9E-01
BLCA	CDK10_RI_10.2_10.1_10.3	CDK10	RI	10.2	10.1	10.3	6.6E-06	0.174	0.273	2.3E-02	5.2E-02	2.4E-01
BLCA	CDK13_AA_13.1_12_13.2	CDK13	AA	13.1	12	13.2	4.6E-04	0.113	0.071	7.0E-01	4.6E-01	4.6E-01
BLCA	CDK18_RI_2.4_2.3_2.5	CDK18	RI	2.4	2.3	2.5	3.2E-05	0.178	0.212	5.4E-01	4.7E-02	1.7E-01
BLCA	CDK2_RI_1.2_1.1_1.3	CDK2	RI	1.2	1.1	1.3	2.5E-05	0.155	-0.193	7.2E-04	2.4E-04	3.6E-02
BLCA	CDK5RAP3_RI_2.2:2.3:2.4_2.1_2.5	CDK5RAP3	RI	2.2:2.3:2.4	2.1	2.5	7.3E-04	0.106	0.438	1.6E-01	3.8E-03	4.9E-02
BLCA	CEACAM1_ES_5_4_8	CEACAM1	ES	5	4	8	5.6E-04	0.112	0.112	4.0E-01	3.5E-01	8.1E-01
BLCA	CEACAM1_ES_9_8_10	CEACAM1	ES	9	8	10	3.3E-07	0.231	-0.506	4.4E-01	9.2E-01	4.2E-01
BLCA	CEP57_RI_10.2_10.1_10.3	CEP57	RI	10.2	10.1	10.3	4.3E-05	0.148	-0.323	2.3E-01	6.7E-01	9.6E-01
BLCA	CEP95_AD_13.2_13.1_14	CEP95	AD	13.2	13.1	14	5.5E-05	0.144	0.275	6.6E-02	1.0E-01	2.0E-01
BLCA	CERCAM_RI_3.2_3.1_3.3	CERCAM	RI	3.2	3.1	3.3	9.6E-12	0.338	-0.440	1.3E-03	9.5E-03	5.6E-02
BLCA	CERS5_ES_6.1:6.2_1_8	CERS5	ES	6.1:6.2	1	8	8.2E-05	0.138	0.060	4.6E-03	3.8E-03	6.9E-02
BLCA	CES2_AD_1.2:1.3_1.1_2	CES2	AD	1.2:1.3	1.1	2	5.7E-07	0.207	-0.339	2.4E-01	5.5E-02	5.7E-01
BLCA	CFLAR_ES_10_8.1_11	CFLAR	ES	10	8.1	11	1.0E-04	0.135	-0.582	8.0E-01	5.4E-01	1.9E-01
BLCA	CGGBP1_AA_5.2:5.3_4_5.4	CGGBP1	AA	5.2:5.3	4	5.4	1.5E-04	0.130	0.180	1.8E-02	6.6E-06	4.0E-03
BLCA	CGGBP1_RI_5.3_5.2_5.4	CGGBP1	RI	5.3	5.2	5.4	2.7E-04	0.121	0.039	2.8E-02	7.4E-04	9.5E-02
BLCA	CHEK1_RI_13.2_13.1_13.3	CHEK1	RI	13.2	13.1	13.3	4.4E-04	0.114	-0.150	4.3E-01	5.3E-01	2.8E-01
BLCA	CHMP7_ES_4_3_5	CHMP7	ES	4	3	5	1.2E-04	0.133	-0.166	5.0E-03	8.0E-03	7.6E-02
BLCA	CHTF8_AA_4.1:4.2_3_4.3	CHTF8	AA	4.1:4.2	3	4.3	1.0E-04	0.138	-0.295	8.8E-02	7.5E-02	1.4E-02
BLCA	CKMT1B_AA_5.1_4_5.2	CKMT1B	AA	5.1	4	5.2	3.1E-04	0.123	-0.175	1.3E-01	8.4E-01	3.5E-01
BLCA	CKMT1B_AA_8.1_7.1_8.2	CKMT1B	AA	8.1	7.1	8.2	1.4E-04	0.133	0.233	5.8E-02	5.4E-03	2.1E-01
BLCA	CLASRP_AD_15.2_15.1_16	CLASRP	AD	15.2	15.1	16	6.8E-04	0.108	0.481	7.2E-01	3.0E-01	1.3E-03
BLCA	CLCN6_ES_13_12_14	CLCN6	ES	13	12	14	4.0E-06	0.195	-0.079	7.6E-03	1.6E-02	7.0E-02

cancer type	id	Gene Symbol	splice_type	Exon	From.Exon	To.Exon	anova.p	adj.r2	r	p.50	p.25	p.10
BLCA	CLEC1A_ES_2_1_3	CLEC1A	ES	2	1	3	1.9E-05	0.188	-0.661	1.2E-01	9.3E-02	2.8E-02
BLCA	CLN3_RI_3.3_3.2_3.4	CLN3	RI	3.3	3.2	3.4	4.3E-05	0.148	-0.009	1.0E-01	6.8E-01	2.1E-01
BLCA	CLSTN1_ES_11_10_12	CLSTN1	ES	11	10	12	6.5E-06	0.176	-0.037	3.9E-01	8.7E-01	8.0E-01
BLCA	CLSTN1_ES_3_2_4	CLSTN1	ES	3	2	4	1.5E-08	0.262	-0.209	8.7E-01	6.1E-01	7.1E-01
BLCA	CNGA1_ES_5_4.2_6	CNGA1	ES	5	4.2	6	3.1E-06	0.219	-0.737	3.5E-02	2.4E-01	1.0E+00
BLCA	COG4_ES_9_8_10	COG4	ES	9	8	10	2.8E-06	0.185	0.144	3.9E-03	3.0E-04	8.7E-03
BLCA	COL1A1_ES_18:19:20:21:22:23:24:25:26	COL1A1	ES	25:26:27:28:29:3	17	40	1.5E-04	0.154	0.116	6.7E-02	5.7E-01	4.4E-02
BLCA	COL1A1_ES_19:20:21:22:23:24:25:26:27	COL1A1	ES	:25:26:27:28:29:	18	38	2.1E-06	0.229	0.234	3.6E-02	6.6E-02	3.6E-01
BLCA	COL1A1_ES_24:25:26:27:28:29:30:31:32	COL1A1	ES	:30:31:32:33:34:	23	43	9.6E-04	0.115	0.087	6.4E-01	1.4E-01	2.1E-01
BLCA	COL4A5_ES_42:43_41_44	COL4A5	ES	42:43:00	41	44	7.3E-09	0.292	0.007	4.1E-01	8.2E-01	8.2E-01
BLCA	COMMD5_AD_2.2_2.1_3	COMMD5	AD	2.2	2.1	3	4.3E-08	0.240	0.073	6.3E-01	5.8E-01	8.0E-01
BLCA	COMMD7_RI_9.2_9.1_9.3	COMMD7	RI	9.2	9.1	9.3	5.3E-04	0.111	0.315	3.0E-01	1.8E-03	9.5E-02
BLCA	CORO7_AD_18.3:18.4_18.2_19	CORO7	AD	18.3:18.4	18.2	19	3.0E-04	0.127	-0.142	1.4E-02	9.0E-05	1.9E-03
BLCA	COX11_RI_3.2_3.1_3.3	COX11	RI	3.2	3.1	3.3	1.4E-05	0.164	-0.194	2.4E-02	8.6E-02	5.3E-02
BLCA	COX20_ES_2:3_1_4	COX20	ES	2:03	1	4	6.8E-04	0.109	-0.149	2.8E-01	1.9E-01	9.4E-01
BLCA	COX4I1_AA_5.1:5.2:5.3_4.1_5.4	COX4I1	AA	5.1:5.2:5.3	4.1	5.4	5.3E-04	0.118	-0.065	8.8E-02	2.1E-02	3.9E-02
BLCA	COX4I1_RI_5.2_5.1_5.3	COX4I1	RI	5.2	5.1	5.3	3.4E-04	0.118	-0.045	4.8E-01	9.8E-01	4.8E-01
BLCA	CPSF3L_AA_11.1_10_11.2	CPSF3L	AA	11.1	10	11.2	1.8E-04	0.127	0.129	2.5E-02	3.7E-02	7.1E-02
BLCA	CSF1_AA_6.1:6.2_5_6.3	CSF1	AA	6.1:6.2	5	6.3	9.6E-07	0.200	0.065	8.3E-03	2.7E-02	6.3E-02
BLCA	CSF2RA_ES_13_12_14	CSF2RA	ES	13	12	14	4.8E-08	0.239	-0.840	2.3E-01	4.9E-01	3.0E-01
BLCA	CSNK1G3_ES_13_12_14	CSNK1G3	ES	13	12	14	1.2E-07	0.231	-0.130	6.7E-01	2.2E-01	8.0E-01
BLCA	CTNNB1_RI_18.2_18.1_18.3	CTNNB1	RI	18.2	18.1	18.3	1.1E-05	0.166	-0.090	6.4E-01	1.7E-01	4.2E-01
BLCA	CTNND1_ES_21_20_22.1	CTNND1	ES	21	20	22.1	1.6E-04	0.129	0.194	8.8E-02	1.0E-02	4.6E-01
BLCA	CTNND1_ES_21:22.1_20_22.2	CTNND1	ES	21:22.1	20	22.2	5.3E-08	0.237	0.120	1.0E-01	1.1E-02	7.3E-01
BLCA	CTNND1_ES_3:4.1:4.2:4.3_2.1_5	CTNND1	ES	3:4.1:4.2:4.3	2.1	5	3.3E-08	0.245	-0.412	9.1E-01	1.8E-01	1.9E-01
BLCA	CTNND1_ES_3:4.1:4.2:4.3:5_2.1_6	CTNND1	ES	3:4.1:4.2:4.3:5	2.1	6	5.3E-05	0.149	-0.316	8.6E-01	6.5E-01	5.0E-01
BLCA	CTTN_ES_11_10_13	CTTN	ES	11	10	13	2.8E-04	0.121	-0.097	7.1E-01	8.7E-01	7.9E-01
BLCA	CWC25_AD_6.2_6.1_7	CWC25	AD	6.2	6.1	7	5.1E-09	0.266	0.253	1.5E-03	2.4E-03	6.1E-02
BLCA	CYB561A3_AA_6.6_6.4_6.7	CYB561A3	AA	6.6	6.4	6.7	8.2E-16	0.432	0.213	7.2E-02	5.7E-01	7.3E-01
BLCA	CYB561A3_RI_6.5_6.4_6.6	CYB561A3	RI	6.5	6.4	6.6	1.2E-05	0.168	-0.260	2.9E-01	4.6E-02	1.2E-02
BLCA	CYP4F12_RI_9.2_9.1_9.3	CYP4F12	RI	9.2	9.1	9.3	2.3E-08	0.285	-0.697	1.5E-01	6.5E-01	2.1E-01
BLCA	CYTH1_ES_11.2:13.1_11.1_13.2	CYTH1	ES	11.2:13.1	11.1	13.2	1.3E-08	0.260	0.417	6.5E-01	2.1E-01	1.5E-01
BLCA	CYTH1_ES_12_11.1_13.2	CYTH1	ES	12	11.1	13.2	3.1E-10	0.307	0.415	4.2E-01	4.4E-01	4.3E-01
BLCA	DAGLB_AD_12.3_12.2_13	DAGLB	AD	12.3	12.2	13	3.9E-04	0.116	-0.083	1.0E-02	2.1E-02	6.3E-01
BLCA	DALRD3_RI_11.3_11.2_11.4	DALRD3	RI	11.3	11.2	11.4	6.2E-05	0.143	0.001	4.6E-02	7.6E-02	1.2E-01
BLCA	DAW1_ES_12_11_13	DAW1	ES	12	11	13	4.7E-05	0.178	-0.813	7.5E-01	7.5E-01	9.9E-01
BLCA	DCAF11_ES_1.2:2.2:2.3_1.1_2.4	DCAF11	ES	1.2:2.2:2.3	1.1	2.4	2.7E-04	0.121	-0.045	7.8E-02	9.5E-02	5.1E-03
BLCA	DCTN3_AA_3.3:3.4_3.1_3.5	DCTN3	AA	3.3:3.4	3.1	3.5	4.7E-05	0.147	-0.336	1.4E-01	7.6E-02	6.9E-01
BLCA	DCTN3_AD_3.2:3.3_3.1_3.5	DCTN3	AD	3.2:3.3	3.1	3.5	5.1E-04	0.115	-0.382	3.2E-01	2.9E-01	4.4E-01
BLCA	DCTN3_RI_3.6_3.5_3.7	DCTN3	RI	3.6	3.5	3.7	6.6E-04	0.116	-0.370	2.0E-03	1.9E-01	3.2E-02

cancer type	id	Gene Symbol	splice_type	Exon	From.Exon	To.Exon	anova.p	adj.r2	r	p.50	p.25	p.10
BLCA	DCUN1D4_AD_9.2:9.3_9.1_10	DCUN1D4	AD	9.2:9.3	9.1	10	4.8E-07	0.209	0.218	4.1E-03	9.4E-03	1.6E-02
BLCA	DDX11_AA_12.1_11.1_12.2	DDX11	AA	12.1	11.1	12.2	1.5E-04	0.134	0.063	4.1E-02	1.3E-02	5.8E-02
BLCA	DDX20_RI_5.2_5.1_5.3	DDX20	RI	5.2	5.1	5.3	5.3E-04	0.120	0.031	3.4E-01	1.1E-01	2.8E-01
BLCA	DDX20_RI_5.6_5.5_5.7	DDX20	RI	5.6	5.5	5.7	3.3E-04	0.118	-0.007	1.9E-01	9.8E-05	8.0E-05
BLCA	DDX39A_AA_7.1_6_7.2	DDX39A	AA	7.1	6	7.2	3.1E-04	0.119	-0.307	1.3E-01	2.2E-02	1.1E-02
BLCA	DDX41_AA_7.1_6_7.2	DDX41	AA	7.1	6	7.2	1.1E-04	0.134	0.031	4.8E-03	4.4E-04	1.0E-04
BLCA	DEDD2_ES_3_2_4.1	DEDD2	ES	3	2	4.1	1.1E-04	0.142	0.076	4.7E-02	3.0E-01	6.0E-01
BLCA	DERL2_ES_5.2_4.3_6	DERL2	ES	5.2	4.3	6	1.7E-04	0.132	0.215	6.0E-01	5.2E-01	6.6E-01
BLCA	DGUOK_ES_3_1_4	DGUOK	ES	3	1	4	3.2E-06	0.184	-0.027	4.1E-01	5.2E-01	9.5E-01
BLCA	DGUOK_ES_4:5_1_7	DGUOK	ES	4:05	1	7	4.4E-05	0.154	-0.172	5.1E-01	1.9E-01	6.2E-01
BLCA	DGUOK_ES_4:5:6_1_7	DGUOK	ES	4:05:06	1	7	1.5E-06	0.195	-0.099	4.7E-01	8.6E-01	9.0E-01
BLCA	DHRS1_RI_6.3_6.2_6.4	DHRS1	RI	6.3	6.2	6.4	6.7E-04	0.107	-0.519	2.1E-01	2.5E-03	4.5E-03
BLCA	DIS3L2_RI_24.2:24.3_24.1_24.4	DIS3L2	RI	24.2:24.3	24.1	24.4	7.8E-04	0.106	-0.021	3.0E-04	3.3E-02	5.6E-02
BLCA	DMPK_ES_13:14.1:14.2_12_15	DMPK	ES	13:14.1:14.2	12	15	7.0E-06	0.173	-0.233	1.9E-02	1.0E+00	9.3E-01
BLCA	DMPK_ES_13:14.2_12_15	DMPK	ES	13:14.2	12	15	1.2E-04	0.135	-0.133	1.2E-01	4.4E-02	2.4E-01
BLCA	DMTF1_AA_13.2_12_13.3	DMTF1	AA	13.2	12	13.3	5.0E-04	0.113	0.321	3.0E-01	3.0E-01	9.6E-01
BLCA	DNAJA3_ES_2_1_3	DNAJA3	ES	2	1	3	7.7E-07	0.203	-0.395	1.2E-01	1.9E-02	2.1E-02
BLCA	DNAJC12_ES_2_1_3	DNAJC12	ES	2	1	3	2.8E-04	0.124	-0.774	7.6E-02	5.3E-01	8.8E-01
BLCA	DNAJC2_ES_5_4_6	DNAJC2	ES	5	4	6	1.2E-06	0.197	0.046	1.5E-02	1.2E-04	6.4E-02
BLCA	DNM2_ES_15_14_17	DNM2	ES	15	14	17	7.1E-04	0.106	0.222	8.7E-01	3.6E-01	5.7E-02
BLCA	DNMT3B_ES_22:23_21_24	DNMT3B	ES	22:23	21	24	1.6E-10	0.315	0.247	3.7E-01	5.6E-01	1.7E-01
BLCA	DOCK6_ES_23_22_24	DOCK6	ES	23	22	24	1.9E-04	0.128	-0.106	2.5E-02	4.0E-02	6.7E-02
BLCA	DPH2_AA_3.1_2.1_3.2	DPH2	AA	3.1	2.1	3.2	7.9E-06	0.177	-0.204	1.0E-02	1.5E-02	3.4E-02
BLCA	DPH2_AD_2.2_2.1_3.2	DPH2	AD	2.2	2.1	3.2	5.7E-06	0.180	-0.177	7.2E-04	2.2E-02	8.6E-02
BLCA	DPP9_ES_5_3_6	DPP9	ES	5	3	6	1.0E-04	0.137	0.241	1.0E-01	3.5E-02	8.0E-02
BLCA	DRG2_RI_6.3_6.2_6.4	DRG2	RI	6.3	6.2	6.4	6.4E-06	0.174	0.084	1.2E-03	4.4E-04	1.0E-02
BLCA	DST_ES_104_103_105	DST	ES	104	103	105	1.1E-05	0.166	0.135	3.8E-02	1.6E-01	8.5E-01
BLCA	DTNA_ES_31:32.1_30_32.2	DTNA	ES	31:32.1	30	32.2	1.5E-07	0.261	-0.344	1.9E-01	1.0E+00	7.1E-01
BLCA	DTX3_AD_1.2:1.3_1.1_1.5	DTX3	AD	1.2:1.3	1.1	1.5	1.2E-04	0.134	0.103	4.6E-02	5.8E-03	1.5E-01
BLCA	DUSP22_RI_7.4_7.3_7.5	DUSP22	RI	7.4	7.3	7.5	1.5E-05	0.163	-0.183	1.8E-01	6.0E-01	9.7E-01
BLCA	DVL1_AD_11.2_11.1_12	DVL1	AD	11.2	11.1	12	1.2E-05	0.166	0.122	2.3E-01	3.1E-01	2.7E-01
BLCA	EDF1_RI_4.2_4.1_4.3	EDF1	RI	4.2	4.1	4.3	1.4E-04	0.131	-0.007	7.6E-04	3.6E-04	3.8E-03
BLCA	EED_ES_8:9:10_7_11.2	EED	ES	8:09:10	7	11.2	3.0E-08	0.248	0.108	7.8E-01	4.1E-01	9.7E-01
BLCA	EFHC1_RI_5.2_5.1_5.3	EFHC1	RI	5.2	5.1	5.3	3.9E-04	0.116	0.267	2.7E-01	1.5E-02	8.7E-03
BLCA	EIF2A_ES_3_2_4	EIF2A	ES	3	2	4	1.3E-04	0.132	-0.266	1.5E-03	5.4E-03	1.7E-02
BLCA	ELMOD3_RI_2.4:2.5:2.6:2.7:2.8_2.3_2.9	ELMOD3	RI	2.4:2.5:2.6:2.7:2.8	2.3	2.9	2.3E-04	0.123	0.247	4.9E-01	6.4E-01	1.9E-01
BLCA	EMC10_ES_7_6_8.1	EMC10	ES	7	6	8.1	2.3E-08	0.249	0.156	2.1E-03	8.0E-04	2.7E-03
BLCA	EMC9_AA_3.2_2_3.3	EMC9	AA	3.2	2	3.3	5.5E-05	0.145	-0.256	9.8E-02	1.0E-01	1.1E-01
BLCA	ENAH_AA_7.1_6_7.2	ENAH	AA	7.1	6	7.2	4.9E-04	0.113	-0.299	4.6E-01	7.5E-01	7.2E-01
BLCA	ENAH_ES_13_12_14	ENAH	ES	13	12	14	2.7E-06	0.187	-0.070	1.7E-01	1.8E-01	3.8E-03

cancer type	id	Gene Symbol	splice_type	Exon	From.Exon	To.Exon	anova.p	adj.r2	r	p.50	p.25	p.10
BLCA	ENPP2_ES_24_23_25	ENPP2	ES	24	23	25	1.0E-04	0.161	0.007	1.5E-01	1.2E-01	9.2E-01
BLCA	ENY2_AD_1.2:1.3_1.1_2.1	ENY2	AD	1.2:1.3	1.1	2.1	2.2E-05	0.157	0.052	3.2E-01	8.5E-01	4.7E-01
BLCA	EPB41_ES_18_15_19.1	EPB41	ES	18	15	19.1	1.6E-06	0.205	0.166	7.6E-01	8.6E-01	9.3E-01
BLCA	EPS8L2_RI_4.2_4.1_4.3	EPS8L2	RI	4.2	4.1	4.3	1.9E-06	0.191	0.164	8.4E-05	2.3E-02	1.5E-02
BLCA	ERCC5_RI_11.3_11.2_11.4	ERCC5	RI	11.3	11.2	11.4	6.2E-04	0.108	0.246	1.3E-02	4.1E-02	3.6E-02
BLCA	ERRFI1_RI_3.3_3.2_3.4	ERRFI1	RI	3.3	3.2	3.4	5.0E-05	0.145	-0.002	3.3E-03	2.4E-02	3.5E-02
BLCA	ESRP1_AD_12.2_12.1_13	ESRP1	AD	12.2	12.1	13	3.6E-06	0.186	-0.411	4.1E-01	9.5E-02	7.1E-01
BLCA	EVI5L_ES_12_11_13	EVI5L	ES	12	11	13	9.8E-11	0.327	-0.085	6.1E-02	9.7E-02	4.4E-01
BLCA	EXOC1_ES_11_10_12	EXOC1	ES	11	10	12	2.2E-14	0.401	0.385	2.9E-01	2.1E-01	4.2E-01
BLCA	EXOC7_AA_8.1_7_8.2	EXOC7	AA	8.1	7	8.2	6.1E-10	0.315	0.356	9.7E-01	1.5E-01	3.7E-02
BLCA	EXOC7_ES_7_6_8.2	EXOC7	ES	7	6	8.2	7.6E-10	0.301	0.041	1.5E-01	9.9E-02	1.1E-01
BLCA	EXOC7_ES_7:8.1:8.2_6_9	EXOC7	ES	7:8.1:8.2	6	9	6.4E-07	0.205	0.443	1.4E-02	1.4E-02	1.4E-04
BLCA	EXOC7_ES_8.1:8.2_7_9	EXOC7	ES	8.1:8.2	7	9	2.5E-08	0.263	0.269	9.7E-03	1.5E-01	5.0E-02
BLCA	EXOC7_ES_8.2_6_9	EXOC7	ES	8.2	6	9	4.1E-04	0.115	0.111	7.5E-01	9.2E-01	7.1E-01
BLCA	EXOSC9_ES_10.3_10.1_10.5	EXOSC9	ES	10.3	10.1	10.5	3.4E-04	0.118	0.111	9.2E-01	1.6E-01	9.4E-02
BLCA	EXOSC9_RI_10.2_10.1_10.3	EXOSC9	RI	10.2	10.1	10.3	1.7E-04	0.127	-0.230	9.6E-01	1.7E-01	2.7E-02
BLCA	EZH2_RI_14.2_14.1_14.3	EZH2	RI	14.2	14.1	14.3	2.9E-04	0.121	-0.218	1.4E-02	6.3E-04	2.8E-01
BLCA	EZH2_RI_14.4_14.3_14.5	EZH2	RI	14.4	14.3	14.5	3.6E-04	0.117	-0.150	9.2E-03	5.9E-03	5.4E-02
BLCA	FADS3_RI_9.2_9.1_9.3	FADS3	RI	9.2	9.1	9.3	2.3E-07	0.220	-0.219	7.7E-03	6.2E-04	2.7E-03
BLCA	FAM111A_RI_3.2_3.1_3.3	FAM111A	RI	3.2	3.1	3.3	5.4E-05	0.149	0.134	7.3E-02	1.1E-01	1.7E-01
BLCA	FAM129C_AA_14.1_13_14.2	FAM129C	AA	14.1	13	14.2	1.2E-06	0.197	-0.798	2.9E-01	2.3E-01	8.9E-01
BLCA	FAM136A_ES_1.3_1.1_2	FAM136A	ES	1.3	1.1	2	8.4E-04	0.104	-0.075	1.4E-01	3.0E-02	3.2E-01
BLCA	FAM13B_ES_19_18_20	FAM13B	ES	19	18	20	6.3E-04	0.113	-0.038	6.8E-03	1.6E-01	7.4E-01
BLCA	FAM195A_ES_3_2_4	FAM195A	ES	3	2	4	6.9E-06	0.173	0.172	8.8E-03	4.0E-03	5.5E-03
BLCA	FAM211B_ES_4_3_5	FAM211B	ES	4	3	5	2.1E-07	0.221	-0.026	4.8E-02	2.8E-02	4.2E-02
BLCA	FAM219B_ES_3.1:3.2_2_4	FAM219B	ES	3.1:3.2	2	4	4.6E-05	0.147	0.353	8.0E-01	4.9E-02	2.2E-03
BLCA	FAM219B_RI_5.3_5.2_5.4	FAM219B	RI	5.3	5.2	5.4	3.3E-04	0.118	-0.284	3.2E-01	2.4E-03	2.0E-01
BLCA	FAM49B_ES_7.1:9_6_10	FAM49B	ES	7.1:9	6	10	2.8E-04	0.142	0.065	8.5E-01	9.7E-01	7.7E-01
BLCA	FAM73B_AD_15.3_15.2_16	FAM73B	AD	15.3	15.2	16	6.5E-06	0.177	0.310	4.3E-03	6.0E-04	3.9E-03
BLCA	FAM76B_ES_9_8_10.1	FAM76B	ES	9	8	10.1	7.5E-06	0.175	0.075	5.3E-01	6.8E-01	4.0E-02
BLCA	FASTK_RI_5.4_5.3_5.5	FASTK	RI	5.4	5.3	5.5	8.7E-04	0.103	0.232	4.3E-02	7.5E-04	1.4E-04
BLCA	FASTK_RI_5.8:5.9:5.10_5.7_5.11	FASTK	RI	5.8:5.9:5.10	5.7	5.11	5.1E-04	0.132	0.267	2.1E-01	5.2E-02	2.7E-01
BLCA	FBLN5_ES_7_5_8	FBLN5	ES	7	5	8	7.6E-06	0.172	-0.655	2.1E-02	3.3E-03	4.5E-02
BLCA	FBXO44_ES_3.1:3.2_2_4	FBXO44	ES	3.1:3.2	2	4	4.0E-04	0.119	0.039	4.0E-01	1.8E-01	1.5E-02
BLCA	FBXO44_ES_5.2:6_5.1_7	FBXO44	ES	5.2:6	5.1	7	4.1E-04	0.116	0.120	1.8E-01	9.8E-02	4.3E-02
BLCA	FGF11_RI_3.2:3.3_3.1_3.4	FGF11	RI	3.2:3.3	3.1	3.4	7.8E-04	0.117	-0.084	5.9E-01	5.9E-01	5.6E-02
BLCA	FLAD1_ES_2.1:2.2:3:4.1:4.2:4.3_1.3_6.1	FLAD1	ES	.1:2.2:3:4.1:4.2:4	1.3	6.1	1.5E-05	0.198	0.088	4.6E-02	7.2E-02	2.2E-01
BLCA	FLAD1_RI_4.2_4.1_4.3	FLAD1	RI	4.2	4.1	4.3	9.5E-06	0.169	0.113	1.5E-02	1.1E-02	6.1E-03
BLCA	FLNA_ES_30_29_31	FLNA	ES	30	29	31	5.1E-09	0.266	-0.208	4.9E-01	3.2E-01	6.0E-02
BLCA	FLNB_ES_32.1_31_33	FLNB	ES	32.1	31	33	4.6E-12	0.353	0.257	4.3E-03	4.0E-04	1.2E-02

cancer type	id	Gene Symbol	splice_type	Exon	From.Exon	To.Exon	anova.p	adj.r2	r	p.50	p.25	p.10
BLCA	FMNL3_ES_26_25_27	FMNL3	ES	26	25	27	3.5E-07	0.215	-0.247	3.5E-01	2.1E-02	5.3E-02
BLCA	FMNL3_ES_6_5_7	FMNL3	ES	6	5	7	3.6E-04	0.124	-0.236	7.4E-01	7.8E-01	6.0E-01
BLCA	FN1_ES_25_24_26	FN1	ES	25	24	26	3.9E-04	0.116	-0.333	4.0E-01	6.2E-01	6.1E-01
BLCA	FN1_ES_40.2_39_40.4	FN1	ES	40.2	39	40.4	9.2E-04	0.103	0.073	8.2E-01	8.5E-01	5.9E-01
BLCA	FNBP1L_RI_16.2_16.1_16.3	FNBP1L	RI	16.2	16.1	16.3	7.5E-06	0.172	0.307	5.0E-01	8.1E-02	4.8E-02
BLCA	FYN_ME_11|12_10_13	FYN	ME	11|12	10	13	1.9E-04	0.126	-0.029	1.3E-01	3.3E-02	2.0E-02
BLCA	GABARAP_ES_1.2:1.5_1.1_2.1	GABARAP	ES	1.2:1.5	1.1	2.1	8.1E-04	0.122	-0.040	1.5E-01	2.6E-01	7.4E-02
BLCA	GAS8_AA_4.1:4.2_3_4.3	GAS8	AA	4.1:4.2	3	4.3	5.9E-04	0.113	0.145	8.1E-01	6.8E-02	5.7E-01
BLCA	GBA2_RI_14.3_14.2_14.4	GBA2	RI	14.3	14.2	14.4	1.4E-04	0.131	0.000	6.7E-01	9.0E-01	7.4E-01
BLCA	GBA2_RI_14.5_14.4_14.6	GBA2	RI	14.5	14.4	14.6	6.6E-04	0.108	0.015	9.6E-01	8.1E-01	4.1E-01
BLCA	GBA2_RI_15.2:15.3_15.1_15.4	GBA2	RI	15.2:15.3	15.1	15.4	9.5E-05	0.136	-0.042	3.1E-01	7.7E-01	7.8E-01
BLCA	GEMIN2_ES_5_4_6	GEMIN2	ES	5	4	6	4.5E-04	0.113	-0.593	7.9E-03	4.8E-03	1.7E-01
BLCA	GGA2_AA_4.1_3.1_4.2	GGA2	AA	4.1	3.1	4.2	4.7E-05	0.147	0.143	1.3E-02	9.4E-03	3.6E-02
BLCA	GGA3_RI_14.2_14.1_14.3	GGA3	RI	14.2	14.1	14.3	2.1E-04	0.125	0.343	9.1E-04	5.2E-03	1.1E-02
BLCA	GMPPA_RI_9.2:9.3_9.1_9.4	GMPPA	RI	9.2:9.3	9.1	9.4	8.3E-04	0.104	-0.220	2.2E-01	1.2E-02	6.7E-02
BLCA	GNPDA1_ES_2.4_1_2.6	GNPDA1	ES	2.4	1	2.6	2.9E-04	0.124	0.087	4.0E-01	2.3E-01	2.7E-01
BLCA	GORASP1_AA_4.1_3_4.2	GORASP1	AA	4.1	3	4.2	5.1E-04	0.114	0.207	7.0E-02	9.4E-02	1.1E-01
BLCA	GPBP1L1_ES_2_1_3	GPBP1L1	ES	2	1	3	7.5E-04	0.107	-0.111	7.4E-01	7.0E-01	4.2E-01
BLCA	GPS1_RI_1.2:1.3:1.4_1.1_1.5	GPS1	RI	1.2:1.3:1.4	1.1	1.5	9.5E-05	0.136	-0.030	3.8E-01	1.5E-01	2.7E-01
BLCA	GRK6_AA_17.1_15_17.2	GRK6	AA	17.1	15	17.2	7.0E-08	0.237	0.188	8.3E-01	5.7E-01	5.0E-01
BLCA	GSS_RI_5.2_5.1_5.3	GSS	RI	5.2	5.1	5.3	3.2E-07	0.214	0.195	2.2E-03	4.0E-03	6.3E-03
BLCA	GSTK1_AD_4.2:4.3_4.1_5	GSTK1	AD	4.2:4.3	4.1	5	2.5E-05	0.155	0.361	1.8E-01	3.9E-01	9.5E-01
BLCA	GSTK1_RI_4.2_4.1_4.3	GSTK1	RI	4.2	4.1	4.3	1.4E-05	0.163	0.048	1.7E-02	1.8E-02	1.3E-01
BLCA	GTPBP3_RI_6.2_6.1_6.3	GTPBP3	RI	6.2	6.1	6.3	6.6E-05	0.141	0.181	1.4E-02	1.2E-02	5.6E-02
BLCA	H2AFY_ME_7|8_6.3_9	H2AFY	ME	7|8	6.3	9	1.4E-04	0.130	0.340	2.8E-01	5.9E-02	2.8E-01
BLCA	HAPLN3_ES_5_4_6	HAPLN3	ES	5	4	6	3.3E-05	0.155	-0.407	5.9E-02	9.1E-03	4.9E-02
BLCA	HARS2_RI_6.2_6.1_6.3	HARS2	RI	6.2	6.1	6.3	2.0E-04	0.125	0.036	1.0E-01	3.2E-03	1.5E-01
BLCA	HAUS5_RI_15.2_15.1_15.3	HAUS5	RI	15.2	15.1	15.3	1.7E-04	0.128	0.206	1.3E-02	5.4E-03	4.9E-02
BLCA	HDAC10_ES_14_13_15	HDAC10	ES	14	13	15	5.8E-04	0.110	-0.099	2.3E-01	5.9E-02	1.6E-01
BLCA	HDAC10_ES_3_2_4	HDAC10	ES	3	2	4	1.9E-04	0.126	-0.247	1.0E-01	1.3E-01	4.5E-01
BLCA	HERC2_ES_52_51_53	HERC2	ES	52	51	53	5.5E-06	0.179	0.399	3.2E-02	1.4E-01	2.7E-01
BLCA	HLX_RI_2.2_2.1_2.3	HLX	RI	2.2	2.1	2.3	2.4E-06	0.188	-0.523	3.1E-02	2.4E-02	1.1E-01
BLCA	HMBS_RI_12.2_12.1_12.3	HMBS	RI	12.2	12.1	12.3	2.5E-07	0.218	-0.226	7.5E-03	3.9E-03	8.4E-02
BLCA	HN1L_ES_3.1_1.2_4	HN1L	ES	3.1	1.2	4	7.2E-04	0.107	0.155	2.6E-01	6.7E-01	8.8E-01
BLCA	HOOK2_AD_4.2_4.1_5	HOOK2	AD	4.2	4.1	5	2.3E-06	0.190	0.262	2.8E-02	1.1E-01	2.3E-01
BLCA	HOPX_RI_4.3:4.4:4.5_4.2_4.6	HOPX	RI	4.3:4.4:4.5	4.2	4.6	5.6E-06	0.203	0.102	1.9E-02	4.9E-04	3.0E-02
BLCA	HPS4_RI_10.2_10.1_10.3	HPS4	RI	10.2	10.1	10.3	2.5E-08	0.263	0.120	7.3E-01	5.9E-02	1.7E-02
BLCA	HPS4_RI_10.2:10.3:10.4_10.1_10.5	HPS4	RI	10.2:10.3:10.4	10.1	10.5	1.5E-07	0.225	0.247	7.4E-02	2.1E-01	6.0E-01
BLCA	HPS4_RI_10.4_10.3_10.5	HPS4	RI	10.4	10.3	10.5	3.3E-06	0.185	0.070	1.7E-01	1.2E-02	4.0E-02
BLCA	HRAS_ES_6_5_7.1	HRAS	ES	6	5	7.1	1.2E-07	0.227	-0.152	2.4E-01	2.7E-03	3.8E-02

cancer type	id	Gene Symbol	splice_type	Exon	From.Exon	To.Exon	anova.p	adj.r2	r	p.50	p.25	p.10
BLCA	HSBP1L1_ES_2.2:3.1_2.1_3.2	HSBP1L1	ES	2.2:3.1	2.1	3.2	1.3E-04	0.132	-0.341	2.9E-01	6.4E-01	4.1E-01
BLCA	HTATIP2_RI_1.4_1.3_1.5	HTATIP2	RI	1.4	1.3	1.5	1.1E-04	0.135	0.023	8.1E-01	4.5E-01	6.2E-01
BLCA	ICAM3_RI_3.2_3.1_3.3	ICAM3	RI	3.2	3.1	3.3	1.0E-08	0.258	-0.510	4.9E-01	2.0E-01	2.1E-01
BLCA	IGFLR1_ES_3:4.1:4.2:4.3_2.2_5	IGFLR1	ES	3:4.1:4.2:4.3	2.2	5	1.0E-09	0.287	-0.455	9.6E-01	6.5E-01	7.7E-01
BLCA	IL18BP_RI_1.2_1.1_1.3	IL18BP	RI	1.2	1.1	1.3	3.4E-05	0.176	-0.395	9.2E-01	7.5E-01	6.9E-01
BLCA	IL18BP_RI_1.2:1.3:1.4:1.5:1.6:1.7_1.1_1.	IL18BP	RI	2:1.3:1.4:1.5:1.6:	1.1	1.8	6.9E-10	0.318	-0.477	5.3E-01	6.9E-01	8.1E-01
BLCA	IL18BP_RI_1.7_1.6_1.8	IL18BP	RI	1.7	1.6	1.8	5.9E-06	0.179	-0.479	9.5E-01	8.4E-01	2.6E-01
BLCA	ILF3_RI_17.2:17.3_17.1_17.4	ILF3	RI	17.2:17.3	17.1	17.4	9.1E-06	0.176	0.086	3.7E-01	5.2E-01	7.9E-01
BLCA	ILK_RI_1.2_1.1_1.3	ILK	RI	1.2	1.1	1.3	6.3E-05	0.146	0.162	4.3E-01	7.3E-01	6.2E-01
BLCA	IMMP1L_ES_4_1_7	IMMP1L	ES	4	1	7	1.0E-03	0.107	-0.136	4.8E-01	5.2E-02	8.1E-01
BLCA	IMPA1_ES_7_6.2_8	IMPA1	ES	7	6.2	8	8.2E-04	0.108	0.003	3.6E-02	1.5E-01	1.2E-03
BLCA	ING3_RI_4.2_4.1_4.3	ING3	RI	4.2	4.1	4.3	2.6E-07	0.222	0.052	5.4E-03	1.4E-03	5.5E-03
BLCA	ING4_ES_2:3_1_4	ING4	ES	2:03	1	4	5.5E-04	0.115	-0.113	2.5E-01	2.5E-01	3.2E-01
BLCA	INO80E_AA_6.1_5_6.2	INO80E	AA	6.1	5	6.2	5.1E-04	0.111	0.015	2.3E-02	1.2E-01	3.5E-01
BLCA	IP6K2_RI_11.3_11.2_11.4	IP6K2	RI	11.3	11.2	11.4	2.4E-04	0.122	0.312	2.6E-01	1.5E-01	8.8E-02
BLCA	IP6K2_RI_11.6:11.7:11.8_11.5_11.9	IP6K2	RI	11.6:11.7:11.8	11.5	11.9	1.8E-04	0.128	0.303	2.0E-01	3.2E-02	1.4E-02
BLCA	IRF3_RI_1.2:1.3_1.1_1.4	IRF3	RI	1.2:1.3	1.1	1.4	1.2E-06	0.197	0.266	2.7E-02	3.6E-02	3.2E-02
BLCA	IRF3_RI_1.2:1.3:1.4_1.1_1.5	IRF3	RI	1.2:1.3:1.4	1.1	1.5	9.2E-05	0.142	0.096	1.8E-01	4.6E-01	2.8E-01
BLCA	ISG20L2_RI_1.2_1.1_1.3	ISG20L2	RI	1.2	1.1	1.3	8.8E-04	0.104	0.082	2.2E-01	1.2E-01	9.3E-01
BLCA	IST1_ES_12:13_11_14.1	IST1	ES	12:13	11	14.1	6.1E-06	0.176	0.187	7.2E-02	1.2E-01	1.5E-01
BLCA	IST1_ES_13_12_14.1	IST1	ES	13	12	14.1	4.1E-05	0.148	0.256	5.4E-03	2.6E-02	7.8E-02
BLCA	ITGB1BP1_AA_8.1:8.2_7_8.3	ITGB1BP1	AA	8.1:8.2	7	8.3	7.6E-05	0.140	-0.225	5.6E-01	2.8E-01	2.0E-02
BLCA	ITGB3BP_ES_2_1_3	ITGB3BP	ES	2	1	3	3.8E-04	0.117	-0.028	3.3E-01	9.9E-01	6.0E-01
BLCA	IVNS1ABP_ES_9_8_10	IVNS1ABP	ES	9	8	10	6.4E-04	0.108	-0.051	4.1E-01	4.9E-01	5.7E-01
BLCA	KCTD7_ES_13_12_14	KCTD7	ES	13	12	14	4.4E-06	0.180	-0.040	6.0E-01	2.9E-01	4.8E-01
BLCA	KIAA0907_RI_13.2_13.1_13.3	KIAA0907	RI	13.2	13.1	13.3	1.2E-04	0.133	0.498	3.6E-02	1.0E-02	1.5E-01
BLCA	KIAA1217_ES_12_11_13	KIAA1217	ES	12	11	13	3.5E-05	0.155	0.053	9.5E-01	5.5E-01	2.2E-01
BLCA	KIAA1217_ES_23:24_22_25.1	KIAA1217	ES	23:24	22	25.1	2.7E-10	0.301	0.376	4.3E-02	2.4E-02	4.2E-03
BLCA	KIAA1468_ME_24|25_23_26	KIAA1468	ME	24|25	23	26	2.6E-05	0.155	-0.346	7.0E-01	8.2E-01	6.0E-01
BLCA	KIF13A_ES_40_39_41.1	KIF13A	ES	40	39	41.1	2.5E-11	0.327	0.002	6.9E-02	1.0E-01	2.7E-01
BLCA	KIFC3_ES_7_5_13	KIFC3	ES	7	5	13	1.5E-05	0.167	-0.239	1.7E-01	6.8E-01	7.7E-01
BLCA	KLC1_ES_15_13.3_18	KLC1	ES	15	13.3	18	1.6E-07	0.225	0.127	5.5E-01	6.2E-01	1.6E-01
BLCA	KLC1_ES_16_15_18	KLC1	ES	16	15	18	1.2E-06	0.199	-0.242	8.5E-01	7.9E-01	5.0E-01
BLCA	KLHDC2_RI_7.2_7.1_7.3	KLHDC2	RI	7.2	7.1	7.3	6.3E-08	0.235	0.222	8.8E-04	7.2E-03	3.5E-03
BLCA	KLHL21_RI_4.2:4.3_4.1_4.4	KLHL21	RI	4.2:4.3	4.1	4.4	5.1E-07	0.208	-0.523	2.8E-02	2.2E-03	8.9E-03
BLCA	KPNA1_ES_6_5_7	KPNA1	ES	6	5	7	4.4E-06	0.180	-0.086	1.0E-01	1.1E-02	1.8E-02
BLCA	KRAS_ES_6_5_7	KRAS	ES	6	5	7	2.2E-04	0.126	-0.001	4.7E-01	5.8E-01	2.3E-01
BLCA	KRT15_ES_2_1.7_3	KRT15	ES	2	1.7	3	4.9E-04	0.123	-0.318	3.0E-02	1.1E-02	2.0E-01
BLCA	KRT15_RI_7.4_7.3_7.5	KRT15	RI	7.4	7.3	7.5	1.7E-04	0.133	-0.393	5.9E-02	3.2E-01	4.5E-01
BLCA	KTN1_ES_2_1_3.2	KTN1	ES	2	1	3.2	3.3E-04	0.118	-0.034	6.3E-01	2.8E-02	1.9E-01

cancer type	id	Gene Symbol	splice_type	Exon	From.Exon	To.Exon	anova.p	adj.r2	r	p.50	p.25	p.10
BLCA	LBH_ES_4_3.1_5	LBH	ES	4	3.1	5	1.9E-04	0.126	-0.428	1.1E-01	1.6E-02	5.8E-03
BLCA	LDHA_ES_3_2.4_4	LDHA	ES	3	2.4	4	3.7E-04	0.116	-0.537	2.8E-03	2.3E-02	3.1E-02
BLCA	LDLRAD3_ES_5_4_6	LDLRAD3	ES	5	4	6	3.7E-10	0.297	-0.710	3.5E-03	1.0E-02	2.7E-02
BLCA	LENG8_RI_15.2:15.3:15.4_15.1_15.5	LENG8	RI	15.2:15.3:15.4	15.1	15.5	2.9E-04	0.120	0.748	2.6E-03	1.8E-02	1.1E-01
BLCA	LEPRE1_RI_14.2:14.3_14.1_14.4	LEPRE1	RI	14.2:14.3	14.1	14.4	2.4E-10	0.302	-0.394	2.7E-02	2.0E-03	1.7E-04
BLCA	LEPRE1_RI_14.3_14.2_14.4	LEPRE1	RI	14.3	14.2	14.4	1.6E-06	0.193	-0.311	3.7E-02	4.1E-03	2.0E-02
BLCA	LGALS8_ES_11_10.1_12	LGALS8	ES	11	10.1	12	1.5E-04	0.129	0.134	8.3E-01	5.3E-01	7.1E-01
BLCA	LGALS8_RI_3.2_3.1_3.3	LGALS8	RI	3.2	3.1	3.3	3.9E-04	0.130	-0.213	9.6E-01	1.2E-01	4.2E-01
BLCA	LGALS9_ES_5_4_6	LGALS9	ES	5	4	6	4.5E-09	0.271	0.205	2.1E-02	1.3E-01	7.5E-02
BLCA	LIMCH1_ES_28_27_29	LIMCH1	ES	28	27	29	1.3E-04	0.146	0.201	2.1E-01	2.1E-01	1.4E-01
BLCA	LMBR1L_ES_4.1:4.2_3_5	LMBR1L	ES	4.1:4.2	3	5	4.3E-04	0.116	0.332	4.0E-02	1.4E-02	3.6E-01
BLCA	LMO7_ES_12_9_13	LMO7	ES	12	9	13	3.1E-05	0.173	-0.222	1.1E-01	1.6E-01	4.3E-01
BLCA	LRRFIP1_ES_15_14_16	LRRFIP1	ES	15	14	16	5.3E-08	0.239	-0.063	1.2E-01	8.1E-02	4.5E-02
BLCA	LRRFIP2_ES_20_19_21	LRRFIP2	ES	20	19	21	2.4E-11	0.330	0.239	3.1E-04	2.6E-02	1.9E-01
BLCA	LTB4R2_RI_1.2:1.3_1.1_1.4	LTB4R2	RI	1.2:1.3	1.1	1.4	1.8E-05	0.166	-0.478	4.1E-01	3.8E-01	8.5E-01
BLCA	LTBP3_ES_25_24_26	LTBP3	ES	25	24	26	5.7E-05	0.146	0.367	1.3E-01	4.3E-01	1.0E+00
BLCA	LUC7L_AD_1.2:1.3:1.4_1.1_2.2	LUC7L	AD	1.2:1.3:1.4	1.1	2.2	5.6E-05	0.144	0.413	8.5E-02	4.1E-02	4.7E-03
BLCA	LUC7L_ES_1.3:1.4_1.1_2.2	LUC7L	ES	1.3:1.4	1.1	2.2	2.7E-04	0.122	0.376	9.8E-03	1.8E-01	1.8E-02
BLCA	MACF1_ES_107_106_108	MACF1	ES	107	106	108	3.2E-05	0.152	-0.127	5.1E-01	2.5E-01	1.1E-01
BLCA	MAGIX_AA_4.1_3_4.2	MAGIX	AA	4.1	3	4.2	1.7E-04	0.148	0.049	7.6E-01	3.7E-01	4.7E-01
BLCA	MAN2A2_AA_17.1_16_17.2	MAN2A2	AA	17.1	16	17.2	3.7E-04	0.121	0.163	9.3E-02	5.4E-03	1.6E-02
BLCA	MAN2C1_AA_19.1_18.2_19.2	MAN2C1	AA	19.1	18.2	19.2	6.3E-05	0.142	0.272	4.5E-01	1.2E-01	2.8E-01
BLCA	MANBAL_ES_3_1_4.2	MANBAL	ES	3	1	4.2	2.7E-06	0.186	-0.178	2.6E-01	8.4E-01	5.3E-01
BLCA	MAP2K7_ES_2_1_3	MAP2K7	ES	2	1	3	1.8E-05	0.170	-0.163	4.9E-02	1.6E-01	9.9E-01
BLCA	MAP3K4_ES_4_3_5	MAP3K4	ES	4	3	5	4.6E-05	0.155	-0.453	1.6E-01	2.1E-01	1.5E-01
BLCA	MAP3K7_ES_11_10_12	MAP3K7	ES	11	10	12	3.2E-13	0.374	-0.186	6.1E-02	2.8E-01	3.5E-01
BLCA	MAP4K1_ES_31_30_32	MAP4K1	ES	31	30	32	8.6E-06	0.175	-0.665	3.4E-01	6.5E-01	9.7E-01
BLCA	MAP7_ES_9_8_10	MAP7	ES	9	8	10	1.5E-09	0.283	-0.288	2.0E-01	3.1E-01	6.1E-01
BLCA	MARK2_ES_16.1:16.2_15.2_17	MARK2	ES	16.1:16.2	15.2	17	2.5E-04	0.122	0.197	9.4E-01	7.2E-01	2.3E-01
BLCA	MARK3_ES_17_16_19	MARK3	ES	17	16	19	9.9E-08	0.229	-0.129	2.2E-02	1.2E-01	1.0E-01
BLCA	MARK3_ES_17:18_16_19	MARK3	ES	17:18	16	19	2.7E-06	0.186	-0.198	4.3E-02	1.2E-01	4.9E-01
BLCA	MBNL1_ES_10_9_11	MBNL1	ES	10	9	11	6.5E-11	0.328	0.200	7.5E-01	9.5E-01	4.9E-01
BLCA	MBNL1_ES_10:11_9_13	MBNL1	ES	10:11	9	13	3.6E-04	0.120	-0.090	1.2E-01	4.2E-01	8.7E-01
BLCA	MBNL1_ES_8_7_9	MBNL1	ES	8	7	9	5.8E-12	0.348	-0.518	1.2E-01	1.7E-01	8.1E-01
BLCA	MBNL2_ES_7_6.3_8	MBNL2	ES	7	6.3	8	8.1E-08	0.241	-0.107	6.3E-01	3.8E-01	6.9E-01
BLCA	MCFD2_ES_5_3_6	MCFD2	ES	5	3	6	7.4E-04	0.106	-0.605	1.9E-02	5.7E-02	1.8E-01
BLCA	MCM7_RI_5.2_5.1_5.3	MCM7	RI	5.2	5.1	5.3	6.3E-07	0.205	-0.188	3.5E-02	1.7E-02	2.6E-03
BLCA	MCM8_ME_11|12_10.2_13	MCM8	ME	11|12	10.2	13	4.8E-04	0.112	0.629	1.3E-02	2.8E-02	5.0E-02
BLCA	MDH2_ES_4_1_5	MDH2	ES	4	1	5	8.2E-04	0.120	-0.016	5.6E-01	6.9E-01	1.0E+00
BLCA	ME3_ES_10_8_11	ME3	ES	10	8	11	2.6E-05	0.166	0.323	7.3E-03	1.0E-02	1.7E-03

cancer type	id	Gene Symbol	splice_type	Exon	From.Exon	To.Exon	anova.p	adj.r2	r	p.50	p.25	p.10
BLCA	MEFV_ES_6_5_7	MEFV	ES	6	5	7	1.7E-10	0.308	-0.723	2.2E-01	7.7E-01	7.4E-01
BLCA	MEIS1_RI_12.4_12.3_12.5	MEIS1	RI	12.4	12.3	12.5	3.0E-04	0.119	0.123	4.8E-02	2.8E-03	7.2E-03
BLCA	MELK_ES_8_7_9	MELK	ES	8	7	9	1.1E-05	0.176	-0.477	1.3E-01	3.2E-01	8.2E-02
BLCA	METTL17_AA_4.1_3_4.2	METTL17	AA	4.1	3	4.2	3.9E-05	0.149	0.196	1.2E-03	9.8E-03	1.5E-01
BLCA	METTL17_RI_10.2_10.1_10.3	METTL17	RI	10.2	10.1	10.3	4.3E-08	0.240	0.253	2.1E-02	6.8E-03	5.4E-03
BLCA	METTL22_ES_4_3.4_5	METTL22	ES	4	3.4	5	5.1E-10	0.299	-0.031	6.3E-02	1.5E-01	3.7E-02
BLCA	METTL3_RI_8.2_8.1_8.3	METTL3	RI	8.2	8.1	8.3	4.4E-05	0.147	0.292	2.5E-03	1.0E-03	5.6E-03
BLCA	METTL3_RI_8.4_8.3_8.5	METTL3	RI	8.4	8.3	8.5	5.2E-04	0.111	0.285	2.9E-04	1.2E-05	5.3E-02
BLCA	MIF4GD_ES_3.1:3.2_2_4	MIF4GD	ES	3.1:3.2	2	4	1.7E-04	0.128	0.015	4.6E-02	4.4E-01	9.8E-01
BLCA	MIS18BP1_ES_8_7_9	MIS18BP1	ES	8	7	9	1.0E-04	0.135	-0.609	8.0E-02	1.7E-03	1.4E-04
BLCA	MLPH_ES_10_9_11	MLPH	ES	10	9	11	5.9E-05	0.170	-0.343	1.7E-03	1.1E-01	2.5E-01
BLCA	MMP23B_RI_3.4_3.3_3.5	MMP23B	RI	3.4	3.3	3.5	1.4E-05	0.180	-0.481	2.3E-01	1.1E-01	6.4E-01
BLCA	MOK_RI_15.2:15.3:15.4_15.1_15.5	MOK	RI	15.2:15.3:15.4	15.1	15.5	2.5E-04	0.124	-0.070	2.9E-01	9.1E-02	2.5E-01
BLCA	MOK_RI_15.2:15.3:15.4:15.5_15.1_15.6	MOK	RI	5.2:15.3:15.4:15.	15.1	15.6	5.1E-04	0.115	-0.197	6.3E-01	6.1E-01	7.0E-01
BLCA	MORC4_AD_17.2_17.1_18	MORC4	AD	17.2	17.1	18	6.2E-05	0.143	-0.205	1.6E-01	4.1E-01	3.1E-01
BLCA	MORF4L2_AA_6.1_3.2_6.2	MORF4L2	AA	6.1	3.2	6.2	1.0E-04	0.152	0.097	9.7E-01	2.6E-01	2.5E-01
BLCA	MPPE1_ES_12_11_13	MPPE1	ES	12	11	13	2.4E-04	0.122	-0.173	6.8E-01	9.5E-01	7.9E-01
BLCA	MRPL10_ES_2.2_1.1_3	MRPL10	ES	2.2	1.1	3	1.7E-04	0.131	-0.065	4.4E-01	3.4E-01	6.0E-01
BLCA	MRPL33_ES_3_2_4	MRPL33	ES	3	2	4	6.1E-05	0.143	0.200	6.6E-02	6.8E-03	9.4E-02
BLCA	MRPL55_AA_2.2:2.3:2.4:2.5:2.6:2.7:2.8_	MRPL55	AA	2.3:2.4:2.5:2.6:2.	1.1	2.9	3.0E-04	0.128	-0.029	1.1E-01	9.4E-03	7.3E-03
BLCA	MRPL55_ES_2.2:2.3:2.4:2.5:2.6_1.2_2.9	MRPL55	ES	2.2:2.3:2.4:2.5:2.	1.2	2.9	6.3E-04	0.108	-0.090	2.4E-01	6.1E-02	4.8E-01
BLCA	MRPL55_RI_2.3:2.4:2.5:2.6:2.7_2.2_2.8	MRPL55	RI	2.3:2.4:2.5:2.6:2.	2.2	2.8	2.7E-04	0.122	-0.185	5.8E-02	4.6E-03	1.6E-02
BLCA	MRPL55_RI_2.3:2.4:2.5:2.6:2.7:2.8_2.2_	MRPL55	RI	3:2.4:2.5:2.6:2.7:	2.2	2.9	2.3E-04	0.125	-0.108	2.7E-02	3.5E-03	1.6E-02
BLCA	MRPL55_RI_2.7:2.8_2.6_2.9	MRPL55	RI	2.7:2.8	2.6	2.9	7.5E-04	0.106	-0.069	5.2E-01	5.8E-03	3.8E-03
BLCA	MRRF_ES_5:6_4_7	MRRF	ES	5:06	4	7	1.8E-06	0.192	-0.009	3.4E-02	2.3E-02	3.6E-04
BLCA	MSTO1_RI_18.2_18.1_18.3	MSTO1	RI	18.2	18.1	18.3	5.7E-07	0.207	0.118	5.1E-03	1.4E-02	3.0E-03
BLCA	MTERFD3_RI_1.2_1.1_1.3	MTERFD3	RI	1.2	1.1	1.3	1.4E-04	0.131	0.378	1.0E-01	9.6E-02	3.4E-01
BLCA	MTFR1L_AA_7.1_4.2_7.2	MTFR1L	AA	7.1	4.2	7.2	5.6E-04	0.111	0.315	5.9E-06	5.5E-06	5.4E-05
BLCA	MTFR1L_ES_5_4.2_7.2	MTFR1L	ES	5	4.2	7.2	1.6E-04	0.128	0.298	3.4E-03	1.7E-03	8.9E-03
BLCA	MTIF2_ES_4_3_5	MTIF2	ES	4	3	5	6.4E-04	0.108	-0.367	9.8E-02	2.1E-02	4.1E-02
BLCA	MTIF3_ES_5_4.2_6	MTIF3	ES	5	4.2	6	8.0E-04	0.105	0.140	8.4E-01	2.8E-01	3.1E-01
BLCA	MTMR3_ES_20_19_21	MTMR3	ES	20	19	21	3.2E-10	0.301	-0.285	4.1E-04	2.1E-03	8.9E-01
BLCA	MUTYH_ES_6.2:6.3:6.4:6.5:7:8:9_5_10	MUTYH	ES	.2:6.3:6.4:6.5:7:8	5	10	9.0E-04	0.128	0.068	9.3E-01	8.7E-01	2.8E-01
BLCA	MUTYH_ES_6.5:7:8:9_5_10	MUTYH	ES	6.5:7:8:9	5	10	2.7E-04	0.155	0.040	9.6E-01	8.4E-01	9.3E-01
BLCA	MYH11_ES_42_41_43	MYH11	ES	42	41	43	1.5E-11	0.351	0.769	7.9E-03	4.3E-04	2.6E-02
BLCA	MYL5_AD_1.4_1.3_2	MYL5	AD	1.4	1.3	2	2.5E-04	0.124	-0.005	1.1E-01	8.6E-01	3.6E-01
BLCA	MYL6B_RI_7.2_7.1_7.3	MYL6B	RI	7.2	7.1	7.3	7.8E-05	0.140	-0.336	3.7E-02	1.3E-02	4.5E-02
BLCA	MYO19_AA_21.1_20_21.2	MYO19	AA	21.1	20	21.2	4.6E-05	0.147	-0.410	1.7E-02	7.2E-04	4.3E-02
BLCA	MYO19_ES_11.2:12_11.1_13	MYO19	ES	11.2:12	11.1	13	4.5E-06	0.179	-0.258	1.6E-01	7.7E-02	8.7E-02
BLCA	MYO1B_ES_23:24_22_25	MYO1B	ES	23:24	22	25	9.2E-06	0.171	-0.175	3.4E-01	1.2E-01	1.9E-02

cancer type	id	Gene Symbol	splice_type	Exon	From.Exon	To.Exon	anova.p	adj.r2	r	p.50	p.25	p.10
BLCA	MYO1B_ES_24_22_25	MYO1B	ES	24	22	25	1.4E-05	0.165	-0.407	4.1E-01	6.7E-02	1.3E-03
BLCA	MYO9B_ES_37_36_38.1	MYO9B	ES	37	36	38.1	1.3E-12	0.381	-0.249	5.5E-01	8.8E-01	3.1E-01
BLCA	MYOF_ES_17_16_18	MYOF	ES	17	16	18	4.3E-05	0.150	-0.220	1.0E-01	8.5E-02	2.4E-01
BLCA	NAA25_ES_17_16_18	NAA25	ES	17	16	18	4.3E-05	0.151	0.014	8.2E-01	2.9E-01	8.5E-01
BLCA	NAA40_AA_4.1_3_4.2	NAA40	AA	4.1	3	4.2	1.6E-05	0.161	0.325	2.6E-02	2.1E-05	1.4E-02
BLCA	NAB1_ES_2:3_1_4.2	NAB1	ES	2:03	1	4.2	3.1E-04	0.122	-0.314	2.2E-01	4.0E-02	2.9E-01
BLCA	NABP1_ES_4:5.1_3_5.2	NABP1	ES	04:05.1	3	5.2	1.2E-05	0.169	-0.141	3.6E-01	4.5E-01	5.8E-02
BLCA	NBPF1_ES_10:11:12:13:14:15_9_16	NBPF1	ES	L0:11:12:13:14:1!	9	16	1.4E-05	0.163	0.252	9.2E-01	7.6E-01	2.9E-01
BLCA	NBPF10_ES_17:18_16_19	NBPF10	ES	17:18	16	19	2.9E-07	0.216	0.325	1.8E-02	4.0E-02	7.9E-01
BLCA	NCKAP5_ES_14:15_13_16	NCKAP5	ES	14:15	13	16	9.3E-04	0.108	-0.038	4.0E-01	4.0E-01	4.0E-01
BLCA	NCOR2_AD_46.2_46.1_47	NCOR2	AD	46.2	46.1	47	2.0E-09	0.278	-0.308	1.1E-01	1.8E-01	5.4E-01
BLCA	NCOR2_ES_46.1:46.2_45_47	NCOR2	ES	46.1:46.2	45	47	1.8E-05	0.163	-0.284	1.2E-03	4.8E-03	3.2E-01
BLCA	NDUFAF5_ES_9_8_10	NDUFAF5	ES	9	8	10	4.7E-06	0.179	0.090	1.5E-01	7.4E-02	4.9E-01
BLCA	NDUFAF6_ES_12_11_14	NDUFAF6	ES	12	11	14	8.0E-04	0.105	-0.041	9.9E-02	3.1E-01	3.1E-01
BLCA	NDUFB10_RI_3.2_3.1_3.3	NDUFB10	RI	3.2	3.1	3.3	3.2E-04	0.118	-0.226	3.1E-01	8.2E-01	5.4E-01
BLCA	NDUFS7_RI_9.2_9.1_9.3	NDUFS7	RI	9.2	9.1	9.3	5.1E-05	0.145	0.022	1.1E-01	2.5E-01	5.6E-01
BLCA	NDUFV3_ES_3_2_4	NDUFV3	ES	3	2	4	4.0E-05	0.149	-0.399	5.0E-01	8.5E-01	5.6E-01
BLCA	NEDD4L_ES_18_17_19	NEDD4L	ES	18	17	19	2.9E-11	0.332	0.352	6.9E-02	1.0E-01	5.1E-01
BLCA	NF2_ES_16.1_15_17	NF2	ES	16.1	15	17	5.4E-06	0.178	0.007	3.3E-01	6.6E-01	5.8E-01
BLCA	NFIX_ES_10_9_11	NFIX	ES	10	9	11	1.3E-07	0.236	-0.131	1.8E-01	2.9E-01	6.8E-01
BLCA	NFS1_ES_7_5_8	NFS1	ES	7	5	8	5.0E-04	0.112	-0.104	3.1E-02	2.0E-02	3.5E-03
BLCA	NFYA_ES_3_2_4	NFYA	ES	3	2	4	3.5E-10	0.306	-0.326	3.6E-01	2.7E-02	2.7E-02
BLCA	NLN_ES_2_1_3	NLN	ES	2	1	3	1.0E-06	0.210	-0.674	2.1E-02	2.1E-02	3.8E-03
BLCA	NME6_AA_5.1_4_5.2	NME6	AA	5.1	4	5.2	7.7E-04	0.106	-0.316	2.6E-01	1.8E-01	8.4E-01
BLCA	NMRAL1_AD_2.4_2.3_2.6	NMRAL1	AD	2.4	2.3	2.6	2.1E-04	0.128	0.010	4.9E-02	1.0E-01	3.4E-01
BLCA	NMRK1_ES_3_2_4	NMRK1	ES	3	2	4	2.4E-04	0.124	0.094	1.2E-02	1.1E-03	8.8E-04
BLCA	NOP2_ES_5_4.5_6	NOP2	ES	5	4.5	6	9.2E-05	0.137	-0.264	3.1E-01	1.9E-02	1.8E-01
BLCA	NOP2_RI_4.3_4.2_4.4	NOP2	RI	4.3	4.2	4.4	2.1E-06	0.190	0.013	5.2E-01	9.1E-02	1.4E-01
BLCA	NOP2_RI_4.3:4.4_4.2_4.5	NOP2	RI	4.3:4.4	4.2	4.5	1.0E-06	0.199	-0.103	5.1E-01	2.9E-01	1.9E-01
BLCA	NPEPPS_AD_9.2_9.1_10	NPEPPS	AD	9.2	9.1	10	1.4E-07	0.225	0.003	2.6E-04	4.2E-04	9.1E-03
BLCA	NR1H3_ES_4_3.5_5.2	NR1H3	ES	4	3.5	5.2	3.2E-04	0.123	-0.055	1.6E-03	8.2E-03	2.5E-02
BLCA	NRBP2_RI_5.2_5.1_5.3	NRBP2	RI	5.2	5.1	5.3	2.6E-06	0.203	0.274	5.3E-02	2.1E-02	2.0E-01
BLCA	NSFL1C_AA_5.1_4_5.2	NSFL1C	AA	5.1	4	5.2	4.4E-04	0.121	-0.106	9.8E-03	9.2E-02	6.3E-01
BLCA	NSFL1C_ES_5.2:7.2_4_7.3	NSFL1C	ES	5.2:7.2	4	7.3	3.0E-08	0.250	-0.063	3.4E-03	4.3E-02	6.7E-02
BLCA	NSUN5_RI_9.2_9.1_9.3	NSUN5	RI	9.2	9.1	9.3	7.4E-05	0.140	-0.325	1.4E-01	1.8E-01	1.7E-01
BLCA	NT5C_AD_3.3_3.2_4.1	NT5C	AD	3.3	3.2	4.1	1.7E-04	0.127	-0.046	2.5E-01	1.2E-01	4.9E-02
BLCA	NT5C3B_ES_3_2_4	NT5C3B	ES	3	2	4	1.8E-06	0.195	-0.059	3.6E-02	1.5E-02	4.3E-03
BLCA	NUDT16L1_AD_2.2_2.1_2.4	NUDT16L1	AD	2.2	2.1	2.4	1.5E-04	0.133	0.131	4.1E-01	2.8E-01	3.4E-01
BLCA	NUMB_ES_13_12_14	NUMB	ES	13	12	14	1.4E-10	0.308	0.056	6.2E-01	5.4E-01	9.0E-01
BLCA	NUMB_ES_7_6_8.2	NUMB	ES	7	6	8.2	8.7E-06	0.171	-0.021	4.0E-01	6.9E-01	7.9E-01

cancer type	id	Gene Symbol	splice_type	Exon	From.Exon	To.Exon	anova.p	adj.r2	r	p.50	p.25	p.10
BLCA	NUP62_AD_1.3:1.4:1.5_1.2_2.1	NUP62	AD	1.3:1.4:1.5	1.2	2.1	3.1E-04	0.119	-0.243	5.9E-02	1.2E-01	7.1E-02
BLCA	NXF1_RI_11.2_11.1_11.3	NXF1	RI	11.2	11.1	11.3	3.3E-07	0.214	0.597	2.1E-02	3.0E-03	6.4E-02
BLCA	OARD1_AD_2.2:2.3_2.1_3.2	OARD1	AD	2.2:2.3	2.1	3.2	2.4E-06	0.189	-0.137	5.9E-01	6.5E-01	5.0E-01
BLCA	OARD1_AD_2.3_2.2_3.2	OARD1	AD	2.3	2.2	3.2	8.3E-07	0.203	-0.291	9.4E-01	4.7E-01	2.2E-01
BLCA	OGFOD2_ES_6.3:8.1:8.2_6.2_8.3	OGFOD2	ES	6.3:8.1:8.2	6.2	8.3	2.6E-05	0.155	0.083	4.6E-01	2.9E-02	1.8E-02
BLCA	OGFOD2_ES_7_6.2_8.2	OGFOD2	ES	7	6.2	8.2	9.5E-05	0.144	0.092	8.4E-02	1.7E-01	3.1E-02
BLCA	OGFOD2_ES_7:8.2_6.2_8.3	OGFOD2	ES	07:08.2	6.2	8.3	4.2E-06	0.181	0.216	1.0E-01	1.3E-01	3.3E-02
BLCA	OPA1_ES_5_4_6	OPA1	ES	5	4	6	3.1E-05	0.157	0.024	6.9E-01	4.3E-01	3.3E-01
BLCA	ORAOV1_RI_5.2_5.1_5.3	ORAOV1	RI	5.2	5.1	5.3	6.6E-05	0.142	0.152	2.6E-01	1.1E-01	2.1E-01
BLCA	ORMDL1_RI_2.3_2.2_2.4	ORMDL1	RI	2.3	2.2	2.4	1.3E-06	0.196	0.422	1.8E-02	4.4E-03	4.1E-03
BLCA	OS9_ES_5.1:5.2:5.3:6:7.1:7.2:7.3:7.4:8:9	OS9	ES	5.3:6:7.1:7.2:7.3:7	4	9.2	2.6E-05	0.160	-0.264	1.5E-01	1.3E-02	2.4E-02
BLCA	OS9_RI_7.2_7.1_7.3	OS9	RI	7.2	7.1	7.3	1.4E-04	0.130	-0.353	1.9E-02	4.8E-03	3.6E-03
BLCA	OSBPL3_ES_9_8_10	OSBPL3	ES	9	8	10	6.9E-06	0.186	-0.255	5.2E-01	5.9E-01	8.2E-01
BLCA	OSGEP_AD_4.5_4.4_5	OSGEP	AD	4.5	4.4	5	1.6E-04	0.129	-0.181	3.5E-02	1.0E-01	5.7E-01
BLCA	OSGEP_RI_4.3_4.2_4.4	OSGEP	RI	4.3	4.2	4.4	6.1E-07	0.206	0.054	1.1E-01	1.2E-03	6.3E-03
BLCA	P4HA1_ME_10|11_9_12.1	P4HA1	ME	10|11	9	12.1	2.6E-04	0.122	-0.126	6.2E-01	6.7E-01	3.6E-01
BLCA	P4HTM_ES_6.2:7.1_6.1_7.2	P4HTM	ES	6.2:7.1	6.1	7.2	6.2E-04	0.109	0.311	5.3E-05	1.8E-06	4.6E-05
BLCA	PABPC1L_ES_12_11_13	PABPC1L	ES	12	11	13	6.3E-04	0.109	0.098	6.1E-01	6.4E-01	9.6E-01
BLCA	PAM_ES_14_13_15	PAM	ES	14	13	15	1.8E-09	0.279	0.005	4.2E-01	6.3E-01	5.2E-01
BLCA	PAPOLA_RI_9.2_9.1_9.3	PAPOLA	RI	9.2	9.1	9.3	7.7E-04	0.105	-0.483	3.7E-01	2.4E-01	4.1E-02
BLCA	PARP2_RI_15.2_15.1_15.3	PARP2	RI	15.2	15.1	15.3	2.8E-04	0.120	-0.083	3.3E-04	1.2E-04	2.2E-02
BLCA	PARPBP_ES_3_2_4.1	PARPBP	ES	3	2	4.1	2.0E-04	0.139	-0.308	1.2E-01	8.8E-02	7.4E-02
BLCA	PAXBP1_AD_8.2_8.1_9	PAXBP1	AD	8.2	8.1	9	7.9E-04	0.106	0.492	8.7E-02	1.0E-01	3.2E-01
BLCA	PBRM1_ES_28:29_27_30	PBRM1	ES	28:29:00	27	30	4.2E-04	0.118	0.127	1.6E-01	4.5E-02	3.1E-01
BLCA	PBRM1_ES_29_27_30	PBRM1	ES	29	27	30	3.0E-04	0.121	0.145	1.7E-01	9.1E-01	6.2E-01
BLCA	PCGF3_AA_5.3_5.1_5.4	PCGF3	AA	5.3	5.1	5.4	1.2E-06	0.197	0.546	1.5E-04	3.7E-03	1.1E-02
BLCA	PCGF3_RI_5.2:5.3_5.1_5.4	PCGF3	RI	5.2:5.3	5.1	5.4	8.9E-04	0.103	0.262	1.5E-02	9.4E-03	8.8E-02
BLCA	PCNP_AA_2.1_1_2.2	PCNP	AA	2.1	1	2.2	1.5E-04	0.130	-0.011	5.2E-02	4.5E-02	2.9E-01
BLCA	PCNP_AA_2.2_1_2.3	PCNP	AA	2.2	1	2.3	1.3E-04	0.132	0.043	8.9E-01	2.3E-01	1.7E-02
BLCA	PCSK7_RI_8.2_8.1_8.3	PCSK7	RI	8.2	8.1	8.3	2.8E-05	0.154	-0.064	1.5E-02	2.5E-02	3.0E-01
BLCA	PCYT1A_AA_7.1_6_7.2	PCYT1A	AA	7.1	6	7.2	4.5E-05	0.148	-0.255	3.5E-01	3.7E-01	4.8E-01
BLCA	PCYT2_AA_3.1_2_3.2	PCYT2	AA	3.1	2	3.2	1.3E-07	0.226	0.422	1.8E-02	1.9E-03	1.5E-03
BLCA	PCYT2_ES_7_6_8	PCYT2	ES	7	6	8	6.0E-07	0.206	0.394	9.6E-01	1.1E-02	6.5E-01
BLCA	PDGFA_ES_6_5_7	PDGFA	ES	6	5	7	6.2E-04	0.109	0.222	3.5E-01	1.1E-01	2.3E-02
BLCA	PDLIM7_AA_10.1_8_10.2	PDLIM7	AA	10.1	8	10.2	1.3E-05	0.164	-0.391	2.6E-02	5.6E-02	4.2E-02
BLCA	PFDN5_ES_2:3:5:6.1_1_6.2	PFDN5	ES	2:3:5:6.1	1	6.2	3.4E-05	0.204	-0.051	8.8E-04	2.2E-02	1.4E-01
BLCA	PFKM_ES_8_7.3_9	PFKM	ES	8	7.3	9	1.0E-05	0.168	-0.587	1.5E-02	4.8E-03	3.1E-02
BLCA	PFKM_RI_16.2_16.1_16.3	PFKM	RI	16.2	16.1	16.3	1.0E-04	0.136	-0.295	9.4E-02	1.1E-02	3.1E-02
BLCA	PGAP2_AD_11.2:11.3_11.1_12	PGAP2	AD	11.2:11.3	11.1	12	5.8E-04	0.111	0.071	1.5E-02	2.2E-01	5.6E-02
BLCA	PGAP2_AD_11.3_11.2_12	PGAP2	AD	11.3	11.2	12	2.3E-04	0.125	0.064	2.0E-01	7.8E-01	5.9E-01

cancer type	id	Gene Symbol	splice_type	Exon	From.Exon	To.Exon	anova.p	adj.r2	r	p.50	p.25	p.10
BLCA	PHF12_RI_12.6_12.5_12.7	PHF12	RI	12.6	12.5	12.7	5.0E-05	0.146	0.296	2.1E-01	1.7E-01	9.1E-01
BLCA	PHLDB1_ES_18.3_18.1_19	PHLDB1	ES	18.3	18.1	19	1.4E-07	0.231	0.202	6.5E-02	2.2E-02	1.1E-02
BLCA	PHYKPL_ES_2_1_3	PHYKPL	ES	2	1	3	8.5E-05	0.147	0.244	4.1E-02	5.2E-03	2.8E-01
BLCA	PI4KA_ES_37:38:39:40:41:42:43_36_44	PI4KA	ES	[illegible]:38:39:40:41:42:[illegible]	36	44	2.9E-04	0.134	-0.018	7.6E-01	8.7E-01	8.6E-01
BLCA	PI4KB_ES_5_4_6	PI4KB	ES	5	4	6	7.4E-04	0.107	0.086	4.5E-02	1.3E-01	9.7E-01
BLCA	PIAS3_RI_7.2_7.1_7.3	PIAS3	RI	7.2	7.1	7.3	6.2E-06	0.176	0.078	8.0E-03	1.5E-02	1.7E-01
BLCA	PIDD_AA_3.1_2.2_3.2	PIDD	AA	3.1	2.2	3.2	1.3E-05	0.173	0.130	7.7E-02	2.2E-02	2.5E-02
BLCA	PIGO_RI_9.2_9.1_9.3	PIGO	RI	9.2	9.1	9.3	3.5E-04	0.117	0.068	9.9E-03	5.0E-01	4.4E-01
BLCA	PLA2G15_ES_2_1_3	PLA2G15	ES	2	1	3	5.0E-04	0.112	-0.117	1.6E-02	1.5E-02	4.1E-02
BLCA	PLA2G6_ES_14_13_15	PLA2G6	ES	14	13	15	2.4E-06	0.207	0.345	3.1E-02	3.2E-02	2.7E-02
BLCA	PLAGL1_RI_5.4:5.5:5.6_5.3_5.7	PLAGL1	RI	5.4:5.5:5.6	5.3	5.7	6.1E-06	0.203	-0.198	1.6E-01	9.5E-02	6.0E-01
BLCA	PLB1_ES_42:43:44_41_45	PLB1	ES	42:43:44	41	45	5.2E-07	0.247	0.261	5.7E-01	7.9E-01	7.5E-01
BLCA	PLCD4_ES_13_12_14	PLCD4	ES	13	12	14	4.0E-04	0.146	-0.617	1.1E-02	3.1E-02	2.3E-02
BLCA	PLD2_RI_18.2_18.1_18.3	PLD2	RI	18.2	18.1	18.3	5.5E-04	0.110	-0.100	1.4E-02	1.2E-03	1.4E-04
BLCA	PLEKHA1_ES_15.1_14_16	PLEKHA1	ES	15.1	14	16	2.7E-11	0.329	-0.337	4.4E-01	2.6E-01	4.0E-01
BLCA	PLEKHG3_AD_15.2_15.1_16	PLEKHG3	AD	15.2	15.1	16	6.5E-04	0.112	-0.381	2.6E-01	6.3E-01	7.6E-01
BLCA	PLEKHH3_AD_11.2_11.1_12.2	PLEKHH3	AD	11.2	11.1	12.2	5.0E-04	0.114	-0.278	6.7E-01	6.2E-01	8.0E-01
BLCA	PLOD2_ES_15_14_16	PLOD2	ES	15	14	16	3.3E-14	0.407	-0.234	3.9E-01	8.5E-01	6.9E-01
BLCA	PLS3_ES_5_3_6	PLS3	ES	5	3	6	8.9E-06	0.170	-0.717	1.3E-03	1.3E-03	4.9E-05
BLCA	PLXDC1_ES_13:14.1_12_14.2	PLXDC1	ES	13:14.1	12	14.2	3.6E-08	0.242	-0.538	1.4E-02	1.7E-01	1.8E-01
BLCA	PML_RI_6.5:6.6_6.4_6.7	PML	RI	6.5:6.6	6.4	6.7	1.9E-04	0.126	-0.118	3.2E-01	3.3E-01	6.6E-01
BLCA	PMM1_ES_2_1_3	PMM1	ES	2	1	3	4.0E-04	0.117	-0.129	2.0E-02	3.3E-01	1.2E-02
BLCA	PMPCB_RI_12.2_12.1_12.3	PMPCB	RI	12.2	12.1	12.3	5.6E-05	0.144	0.096	3.5E-03	1.1E-03	2.8E-04
BLCA	POFUT2_AA_8.3:8.4_8.1_8.5	POFUT2	AA	8.3:8.4	8.1	8.5	3.7E-04	0.117	0.317	1.3E-02	1.4E-02	4.3E-01
BLCA	POFUT2_AD_8.2:8.3_8.1_8.5	POFUT2	AD	8.2:8.3	8.1	8.5	3.9E-05	0.149	0.273	3.3E-02	2.2E-03	1.6E-01
BLCA	POGLUT1_ES_5:6.1_4_6.2	POGLUT1	ES	05:06.1	4	6.2	3.1E-04	0.125	0.128	7.6E-01	4.2E-01	7.6E-01
BLCA	POLR2H_ES_2.1:2.2_1_3	POLR2H	ES	2.1:2.2	1	3	1.9E-04	0.127	0.070	4.2E-02	2.6E-01	1.2E-01
BLCA	POLR2J3_ES_2_1_4.3	POLR2J3	ES	2	1	4.3	2.7E-05	0.175	0.044	7.0E-01	3.2E-01	9.6E-01
BLCA	POLR2J3_ES_6:7_4.3_8	POLR2J3	ES	6:07	4.3	8	2.3E-05	0.157	0.687	6.6E-01	7.4E-02	6.0E-01
BLCA	POMT1_AD_8.2_8.1_9	POMT1	AD	8.2	8.1	9	1.5E-05	0.175	0.067	2.2E-02	3.4E-01	3.7E-01
BLCA	POR_RI_16.3_16.2_16.4	POR	RI	16.3	16.2	16.4	4.9E-05	0.150	-0.126	2.4E-01	4.1E-01	7.6E-01
BLCA	PPCS_AD_2.2_2.1_3.1	PPCS	AD	2.2	2.1	3.1	6.6E-04	0.108	-0.138	9.9E-02	5.2E-01	1.7E-01
BLCA	PPIE_AD_7.2_7.1_8	PPIE	AD	7.2	7.1	8	4.4E-05	0.147	0.004	7.1E-01	1.3E-01	4.3E-01
BLCA	PPIE_ES_11_9.1_12	PPIE	ES	11	9.1	12	8.3E-04	0.104	-0.001	3.0E-01	1.1E-01	6.0E-01
BLCA	PPIL3_AD_1.2_1.1_2.2	PPIL3	AD	1.2	1.1	2.2	2.0E-04	0.127	-0.152	4.4E-01	5.5E-01	9.4E-01
BLCA	PPM1M_RI_10.2_10.1_10.3	PPM1M	RI	10.2	10.1	10.3	7.8E-04	0.105	-0.069	2.9E-02	6.8E-04	1.3E-02
BLCA	PPP4C_ES_3.1:3.2_2.2_4	PPP4C	ES	3.1:3.2	2.2	4	3.2E-04	0.119	-0.123	2.5E-01	1.2E-01	7.3E-01
BLCA	PPP4R1_ES_15_14_16	PPP4R1	ES	15	14	16	2.9E-06	0.185	-0.191	7.5E-02	2.6E-01	9.1E-02
BLCA	PPP4R1_ES_5_4.2_6	PPP4R1	ES	5	4.2	6	4.7E-05	0.147	-0.322	1.3E-01	1.1E-01	1.8E-02
BLCA	PQLC1_ES_4.1:4.2_2_5	PQLC1	ES	4.1:4.2	2	5	2.3E-04	0.123	-0.352	1.5E-03	2.0E-03	3.3E-02

cancer type	id	Gene Symbol	splice_type	Exon	From.Exon	To.Exon	anova.p	adj.r2	r	p.50	p.25	p.10
BLCA	PRKRIP1_ES_5_4_6	PRKRIP1	ES	5	4	6	1.1E-04	0.138	-0.405	2.9E-01	2.9E-02	2.9E-03
BLCA	PRMT2_RI_1.2_1.1_1.3	PRMT2	RI	1.2	1.1	1.3	1.1E-04	0.134	0.030	6.9E-02	2.1E-02	3.5E-02
BLCA	PRMT7_AA_17.1_16_17.2	PRMT7	AA	17.1	16	17.2	9.6E-04	0.102	0.092	1.9E-01	7.6E-03	1.5E-01
BLCA	PRPF3_ES_4_3_5	PRPF3	ES	4	3	5	2.9E-04	0.121	-0.167	3.5E-02	8.0E-03	8.2E-02
BLCA	PRPF39_ES_4.1:4.2:4.3_3_5	PRPF39	ES	4.1:4.2:4.3	3	5	1.4E-05	0.163	0.565	3.7E-02	2.3E-01	4.3E-01
BLCA	PRRC2C_ES_34_33_35	PRRC2C	ES	34	33	35	5.2E-04	0.111	0.130	6.8E-01	4.4E-01	8.7E-02
BLCA	PSMC3IP_AD_2.2_2.1_4.1	PSMC3IP	AD	2.2	2.1	4.1	2.8E-04	0.122	-0.367	4.8E-02	8.4E-02	6.5E-01
BLCA	PSMC3IP_AD_4.2:4.3_4.1_5.1	PSMC3IP	AD	4.2:4.3	4.1	5.1	6.1E-09	0.288	-0.259	1.7E-02	2.5E-01	4.1E-01
BLCA	PSMC5_AA_2.1:2.2_1_2.3	PSMC5	AA	2.1:2.2	1	2.3	7.5E-05	0.140	0.008	2.2E-03	5.3E-06	8.9E-05
BLCA	PSMC5_RI_2.2_2.1_2.3	PSMC5	RI	2.2	2.1	2.3	7.5E-07	0.203	0.002	1.4E-02	2.6E-04	1.4E-02
BLCA	PSRC1_RI_5.2:5.3_5.1_5.4	PSRC1	RI	5.2:5.3	5.1	5.4	1.8E-04	0.140	-0.132	7.1E-02	4.3E-02	3.6E-03
BLCA	PSTPIP1_ES_3_2.2_5	PSTPIP1	ES	3	2.2	5	3.3E-09	0.272	-0.704	1.6E-01	1.1E-01	6.8E-02
BLCA	PTCD2_AD_1.2_1.1_2	PTCD2	AD	1.2	1.1	2	2.6E-05	0.155	-0.566	4.0E-02	5.2E-03	1.2E-02
BLCA	PTK2B_ES_28_27_29	PTK2B	ES	28	27	29	1.1E-13	0.385	-0.100	8.7E-01	5.9E-01	1.7E-02
BLCA	PTK2B_RI_13.2_13.1_13.3	PTK2B	RI	13.2	13.1	13.3	4.1E-04	0.122	-0.151	7.7E-02	4.4E-01	6.0E-01
BLCA	QKI_RI_8.3:8.4_8.2_8.5	QKI	RI	8.3:8.4	8.2	8.5	2.8E-05	0.160	-0.385	8.2E-01	6.6E-01	2.4E-01
BLCA	RAB3GAP1_ES_4:5_3.1_6	RAB3GAP1	ES	4:05	3.1	6	6.7E-04	0.107	-0.644	5.6E-01	8.3E-02	5.1E-01
BLCA	RAC1_ES_4_3_5	RAC1	ES	4	3	5	7.7E-04	0.105	0.025	7.2E-01	2.7E-01	4.2E-01
BLCA	RAD51C_ES_5_4_6	RAD51C	ES	5	4	6	1.9E-06	0.192	-0.273	7.7E-01	4.9E-01	7.4E-01
BLCA	RAI14_ES_18_17_19	RAI14	ES	18	17	19	6.7E-07	0.205	0.115	9.7E-02	1.8E-02	8.9E-02
BLCA	RALGPS2_ES_15_14_16	RALGPS2	ES	15	14	16	1.9E-09	0.282	0.025	8.2E-01	9.5E-01	5.3E-01
BLCA	RANBP3_AA_10.1_8_10.2	RANBP3	AA	10.1	8	10.2	2.2E-04	0.124	0.290	2.8E-03	1.2E-03	3.1E-03
BLCA	RANGRF_AD_3.2:3.3_3.1_3.5	RANGRF	AD	3.2:3.3	3.1	3.5	5.8E-06	0.178	-0.104	7.0E-01	3.6E-01	8.0E-02
BLCA	RANGRF_RI_3.2:3.3:3.4_3.1_3.5	RANGRF	RI	3.2:3.3:3.4	3.1	3.5	1.1E-05	0.172	-0.168	9.1E-01	4.0E-01	1.6E-01
BLCA	RANGRF_RI_3.4_3.3_3.5	RANGRF	RI	3.4	3.3	3.5	9.1E-04	0.103	-0.196	9.3E-01	3.4E-01	3.8E-01
BLCA	RBM3_RI_3.2_3.1_3.3	RBM3	RI	3.2	3.1	3.3	6.6E-05	0.142	-0.390	9.0E-04	2.6E-03	3.3E-02
BLCA	RBM39_ES_4_3_6	RBM39	ES	4	3	6	3.7E-09	0.270	0.382	1.5E-01	4.1E-01	8.9E-01
BLCA	RBM48_RI_4.2_4.1_4.3	RBM48	RI	4.2	4.1	4.3	6.3E-04	0.108	0.241	2.4E-01	1.2E-02	2.7E-05
BLCA	RBM6_ES_3.2:4:5_2_7	RBM6	ES	3.2:4:5	2	7	3.0E-05	0.156	0.362	1.5E-02	1.9E-01	8.2E-01
BLCA	RBM6_ES_4:5_2_7	RBM6	ES	4:05	2	7	3.4E-04	0.137	0.367	6.8E-02	3.6E-01	4.5E-01
BLCA	RBM6_ES_6_5_7	RBM6	ES	6	5	7	9.2E-05	0.138	-0.465	4.0E-02	7.0E-02	2.3E-01
BLCA	RBM6_RI_14.2_14.1_14.3	RBM6	RI	14.2	14.1	14.3	2.9E-05	0.153	0.684	1.7E-02	3.1E-03	1.4E-04
BLCA	RBM7_RI_4.3_4.2_4.4	RBM7	RI	4.3	4.2	4.4	2.4E-05	0.156	-0.645	1.8E-01	3.8E-02	7.8E-02
BLCA	RBMX_RI_8.2:8.3_8.1_8.4	RBMX	RI	8.2:8.3	8.1	8.4	1.2E-04	0.133	-0.018	5.4E-01	2.1E-01	3.7E-01
BLCA	RCOR3_ES_5_4_6	RCOR3	ES	5	4	6	1.3E-08	0.264	0.125	7.1E-02	1.2E-01	8.7E-01
BLCA	REEP5_ES_4:5_3_6	REEP5	ES	4:05	3	6	4.6E-04	0.113	-0.238	7.3E-02	1.5E-02	1.5E-01
BLCA	RFX5_ES_7_6.2_8	RFX5	ES	7	6.2	8	6.6E-05	0.141	-0.301	2.2E-01	5.7E-02	3.0E-03
BLCA	RGS12_ES_19_18_20.1	RGS12	ES	19	18	20.1	1.8E-04	0.127	-0.090	6.7E-01	9.9E-01	7.3E-01
BLCA	RHBDF1_RI_11.2_11.1_11.3	RHBDF1	RI	11.2	11.1	11.3	3.3E-04	0.124	0.205	2.9E-01	1.7E-01	8.6E-02
BLCA	RHOT2_RI_9.4_9.3_9.5	RHOT2	RI	9.4	9.3	9.5	1.8E-07	0.222	0.552	4.3E-05	2.3E-03	5.8E-03

cancer type	id	Gene Symbol	splice_type	Exon	From.Exon	To.Exon	anova.p	adj.r2	r	p.50	p.25	p.10
BLCA	RIC8B_ES_13_11_17	RIC8B	ES	13	11	17	9.1E-04	0.108	-0.013	8.3E-01	3.9E-01	1.1E-01
BLCA	RIPK3_AD_5.2_5.1_6	RIPK3	AD	5.2	5.1	6	6.1E-04	0.125	-0.002	6.1E-03	6.0E-02	2.1E-02
BLCA	RMND5B_AD_4.2_4.1_5.1	RMND5B	AD	4.2	4.1	5.1	8.6E-04	0.105	-0.026	3.2E-01	4.4E-01	3.0E-01
BLCA	RNF123_AD_27.2_27.1_28	RNF123	AD	27.2	27.1	28	2.9E-05	0.153	0.140	1.6E-02	3.1E-04	2.2E-01
BLCA	RNF14_AD_2.2_2.1_3	RNF14	AD	2.2	2.1	3	2.1E-04	0.138	-0.135	6.2E-01	5.9E-01	9.2E-01
BLCA	RNF220_AD_9.2_9.1_10.1	RNF220	AD	9.2	9.1	10.1	7.8E-05	0.139	-0.109	2.2E-02	4.6E-02	1.3E-01
BLCA	RNPC3_AA_15.1_14_15.2	RNPC3	AA	15.1	14	15.2	4.1E-05	0.148	0.382	2.3E-02	3.5E-01	5.1E-01
BLCA	ROGDI_AD_4.2_4.1_5	ROGDI	AD	4.2	4.1	5	1.1E-07	0.228	0.078	4.7E-02	5.5E-02	1.2E-02
BLCA	RPL10_RI_2.2_2.1_2.3	RPL10	RI	2.2	2.1	2.3	6.2E-06	0.175	0.011	1.8E-01	7.3E-01	9.6E-01
BLCA	RPL22L1_AA_3.1_2.2_3.2	RPL22L1	AA	3.1	2.2	3.2	4.5E-04	0.113	-0.623	7.0E-01	4.8E-01	9.5E-01
BLCA	RPS20_RI_1.3_1.2_1.4	RPS20	RI	1.3	1.2	1.4	1.2E-04	0.136	-0.079	5.2E-02	3.8E-02	4.0E-02
BLCA	RPS21_AA_3.3:3.4_3.1_3.5	RPS21	AA	3.3:3.4	3.1	3.5	4.0E-07	0.228	-0.109	8.7E-01	8.4E-01	4.3E-01
BLCA	RPS21_AD_3.2:3.3_3.1_3.5	RPS21	AD	3.2:3.3	3.1	3.5	1.1E-06	0.208	-0.129	3.1E-01	3.4E-01	7.5E-01
BLCA	RPS24_AA_5.1_4_5.2	RPS24	AA	5.1	4	5.2	3.9E-11	0.323	-0.020	1.6E-01	9.5E-03	1.0E-02
BLCA	RPS24_ES_5.1:5.2_4_6	RPS24	ES	5.1:5.2	4	6	1.0E-11	0.338	0.021	3.7E-02	1.1E-02	5.1E-02
BLCA	RPS24_ES_5.2_4_6	RPS24	ES	5.2	4	6	8.6E-08	0.231	0.002	2.1E-01	1.7E-01	6.5E-01
BLCA	RQCD1_ES_7_6_8	RQCD1	ES	7	6	8	7.5E-05	0.140	-0.758	5.6E-02	1.4E-01	5.5E-02
BLCA	RRM2B_ES_2_1_3.1	RRM2B	ES	2	1	3.1	7.9E-04	0.105	-0.459	4.7E-02	4.1E-02	2.1E-02
BLCA	RRNAD1_RI_2.2_2.1_2.3	RRNAD1	RI	2.2	2.1	2.3	6.9E-06	0.173	0.036	2.8E-02	6.1E-02	1.6E-01
BLCA	RSAD1_AD_8.2_8.1_9	RSAD1	AD	8.2	8.1	9	4.3E-05	0.148	0.339	1.1E-01	3.2E-02	2.4E-02
BLCA	RSRC2_ES_4:5.1_3_5.2	RSRC2	ES	04:05.1	3	5.2	8.4E-05	0.138	0.196	6.4E-01	8.5E-01	2.1E-01
BLCA	RWDD1_ES_3_1_4	RWDD1	ES	3	1	4	3.8E-04	0.116	-0.216	6.8E-02	2.8E-01	3.7E-01
BLCA	RWDD2A_RI_2.3_2.2_2.4	RWDD2A	RI	2.3	2.2	2.4	2.2E-04	0.126	0.130	3.8E-02	3.1E-01	6.0E-02
BLCA	S100A1_AA_4.1_3_4.2	S100A1	AA	4.1	3	4.2	7.9E-07	0.205	-0.231	2.8E-02	2.8E-02	2.0E-02
BLCA	S100A6_AA_3.1_2_3.2	S100A6	AA	3.1	2	3.2	1.0E-03	0.116	-0.150	7.3E-01	7.6E-01	5.4E-01
BLCA	SBDS_ES_2.1_1_3	SBDS	ES	2.1	1	3	5.1E-04	0.114	-0.137	1.4E-01	1.3E-01	3.6E-01
BLCA	SCP2_ES_12_11_13	SCP2	ES	12	11	13	2.4E-07	0.218	-0.688	5.0E-01	5.0E-01	9.2E-01
BLCA	SCPEP1_ES_10.1:10.2_9_11	SCPEP1	ES	10.1:10.2	9	11	9.7E-04	0.119	0.143	2.1E-02	5.6E-01	1.7E-01
BLCA	SCRIB_ES_17_16_18	SCRIB	ES	17	16	18	3.0E-06	0.188	-0.390	3.6E-01	6.9E-01	6.0E-01
BLCA	SCRN2_RI_7.2_7.1_7.3	SCRN2	RI	7.2	7.1	7.3	2.4E-04	0.123	-0.143	4.4E-01	4.1E-01	4.3E-01
BLCA	SDC3_RI_2.2_2.1_2.3	SDC3	RI	2.2	2.1	2.3	5.7E-04	0.115	-0.296	3.3E-01	1.8E-02	7.4E-02
BLCA	SDCCAG3_ES_3_2_4	SDCCAG3	ES	3	2	4	2.3E-05	0.163	-0.283	2.5E-01	6.4E-01	7.6E-01
BLCA	SDR39U1_AA_8.1_7_8.2	SDR39U1	AA	8.1	7	8.2	1.5E-04	0.130	-0.321	7.4E-01	2.0E-01	8.6E-02
BLCA	SEC16A_ES_25_24_26	SEC16A	ES	25	24	26	9.7E-06	0.169	0.009	1.2E-02	3.8E-01	3.5E-01
BLCA	SEC31A_AD_10.2_10.1_11	SEC31A	AD	10.2	10.1	11	1.4E-04	0.130	-0.595	1.9E-01	4.4E-02	2.0E-02
BLCA	SEC31A_ES_26.1_25.1_28	SEC31A	ES	26.1	25.1	28	6.5E-07	0.205	-0.161	1.6E-02	1.1E-02	3.6E-02
BLCA	SEC31A_ES_26.1:26.2_25.1_28	SEC31A	ES	26.1:26.2	25.1	28	1.3E-07	0.226	-0.233	1.0E-01	5.4E-03	2.5E-01
BLCA	SEC31A_ES_26.1:26.2:27_25.1_28	SEC31A	ES	26.1:26.2:27	25.1	28	2.4E-05	0.156	-0.057	1.7E-01	8.2E-04	2.6E-02
BLCA	SEMA3F_ES_7_6_8	SEMA3F	ES	7	6	8	8.1E-08	0.232	0.487	1.0E-01	1.7E-01	5.8E-02
BLCA	SERPINF1_AA_4.1_3_4.2	SERPINF1	AA	4.1	3	4.2	1.7E-10	0.312	-0.377	1.6E-02	5.7E-05	1.9E-04

cancer type	id	Gene Symbol	splice_type	Exon	From.Exon	To.Exon	anova.p	adj.r2	r	p.50	p.25	p.10
BLCA	SF3B1_AD_4.2:4.3_4.1_5	SF3B1	AD	4.2:4.3	4.1	5	1.2E-05	0.166	0.285	1.3E-01	7.5E-01	8.2E-01
BLCA	SGSM2_RI_5.2_5.1_5.3	SGSM2	RI	5.2	5.1	5.3	7.5E-07	0.206	0.353	3.2E-01	1.7E-01	4.7E-01
BLCA	SGSM3_RI_19.2_19.1_19.3	SGSM3	RI	19.2	19.1	19.3	1.1E-04	0.134	0.313	2.7E-02	7.0E-05	9.6E-02
BLCA	SH2B1_RI_3.3:3.4:3.5_3.2_3.6	SH2B1	RI	3.3:3.4:3.5	3.2	3.6	9.8E-04	0.102	0.283	1.8E-01	6.4E-01	3.5E-01
BLCA	SH2B1_RI_3.4:3.5_3.3_3.6	SH2B1	RI	3.4:3.5	3.3	3.6	4.8E-05	0.146	0.333	2.3E-01	3.8E-01	2.7E-01
BLCA	SH3GL3_ES_2:3:4_1_5	SH3GL3	ES	2:03:04	1	5	3.3E-08	0.243	-0.771	3.5E-02	1.8E-02	1.5E-02
BLCA	SHROOM1_AA_3.1:3.2_2_3.3	SHROOM1	AA	3.1:3.2	2	3.3	2.3E-04	0.145	0.309	2.5E-01	1.1E-02	1.6E-01
BLCA	SHROOM1_ES_3.1_2_3.3	SHROOM1	ES	3.1	2	3.3	1.6E-06	0.215	0.345	1.0E-02	1.5E-01	6.1E-03
BLCA	SIDT2_RI_25.2_25.1_25.3	SIDT2	RI	25.2	25.1	25.3	1.4E-05	0.164	-0.305	2.6E-02	3.9E-02	2.5E-01
BLCA	SIGIRR_AD_1.2_1.1_3	SIGIRR	AD	1.2	1.1	3	9.0E-04	0.118	-0.045	2.6E-02	1.0E-01	8.4E-01
BLCA	SIMC1_ES_2:3_1_4	SIMC1	ES	2:03	1	4	2.2E-04	0.126	-0.485	3.2E-01	9.0E-01	8.1E-01
BLCA	SIN3B_ES_10_9_11.2	SIN3B	ES	10	9	11.2	7.7E-05	0.139	0.108	1.4E-01	2.1E-01	5.6E-02
BLCA	SKA2_AD_1.2_1.1_5	SKA2	AD	1.2	1.1	5	4.0E-04	0.122	0.086	9.2E-02	4.3E-01	8.3E-02
BLCA	SKA2_ES_1.2:2_1.1_5	SKA2	ES	1.2:2	1.1	5	4.0E-05	0.156	0.203	1.0E-01	3.4E-01	1.0E+00
BLCA	SKA2_ES_1.2:2:4.1_1.1_5	SKA2	ES	1.2:2:4.1	1.1	5	9.6E-05	0.143	0.189	5.5E-02	1.7E-02	7.8E-01
BLCA	SLC10A7_ES_13_12_14	SLC10A7	ES	13	12	14	8.5E-04	0.107	0.031	9.1E-01	9.9E-01	8.7E-01
BLCA	SLC15A4_RI_3.3_3.2_3.4	SLC15A4	RI	3.3	3.2	3.4	3.2E-04	0.122	-0.175	2.6E-02	8.3E-04	1.0E-02
BLCA	SLC25A10_ES_7_6_8.1	SLC25A10	ES	7	6	8.1	4.7E-05	0.147	0.191	3.6E-01	6.4E-01	8.0E-01
BLCA	SLC25A14_RI_9.2_9.1_9.3	SLC25A14	RI	9.2	9.1	9.3	1.9E-06	0.191	0.194	2.2E-02	3.4E-03	4.7E-02
BLCA	SLC25A3_AD_2.2_2.1_4.1	SLC25A3	AD	2.2	2.1	4.1	1.0E-04	0.135	-0.351	3.5E-02	5.4E-03	2.4E-01
BLCA	SLC25A36_ES_6.1:6.2_4.1_7	SLC25A36	ES	6.1:6.2	4.1	7	8.4E-05	0.143	0.157	9.4E-02	1.1E-01	2.6E-01
BLCA	SLC27A1_RI_4.2:4.3_4.1_4.4	SLC27A1	RI	4.2:4.3	4.1	4.4	1.5E-04	0.158	-0.050	2.6E-01	2.9E-01	3.6E-02
BLCA	SLC37A2_ES_12_11_13	SLC37A2	ES	12	11	13	3.3E-04	0.121	0.010	6.0E-01	5.9E-01	9.0E-02
BLCA	SLC37A2_ES_18_17_19	SLC37A2	ES	18	17	19	1.3E-08	0.274	0.409	1.9E-01	1.8E-01	2.6E-01
BLCA	SLC37A3_ES_4_3_5	SLC37A3	ES	4	3	5	6.0E-05	0.143	-0.300	1.5E-02	5.7E-05	8.1E-05
BLCA	SLC39A13_RI_6.2_6.1_6.3	SLC39A13	RI	6.2	6.1	6.3	4.9E-06	0.178	-0.207	5.9E-02	6.3E-02	1.8E-01
BLCA	SLK_ES_13_12_14	SLK	ES	13	12	14	3.6E-15	0.418	0.358	8.2E-02	4.9E-03	3.0E-02
BLCA	SLMAP_ES_25_24_26	SLMAP	ES	25	24	26	2.1E-08	0.249	-0.002	2.5E-02	4.4E-02	8.6E-03
BLCA	SMARCD3_RI_11.2_11.1_11.3	SMARCD3	RI	11.2	11.1	11.3	3.9E-06	0.182	-0.250	1.1E-02	4.0E-04	8.8E-05
BLCA	SMC4_RI_1.2_1.1_1.3	SMC4	RI	1.2	1.1	1.3	1.7E-07	0.223	-0.075	8.8E-02	3.7E-02	9.7E-02
BLCA	SMG7_ES_20_19_21	SMG7	ES	20	19	21	1.3E-04	0.132	-0.039	6.9E-01	3.6E-01	6.2E-01
BLCA	SMPD4_ES_12_11_14	SMPD4	ES	12	11	14	4.1E-07	0.213	0.095	4.9E-02	4.7E-03	5.9E-03
BLCA	SMPD4_ES_13_11_14	SMPD4	ES	13	11	14	2.5E-06	0.190	0.167	9.2E-03	1.1E-02	1.9E-03
BLCA	SMURF2_ES_4_3_5	SMURF2	ES	4	3	5	1.3E-05	0.167	-0.534	6.4E-02	7.2E-02	3.6E-01
BLCA	SNAP23_AA_5.1:5.2_4_5.3	SNAP23	AA	5.1:5.2	4	5.3	5.0E-04	0.112	0.160	1.5E-01	5.1E-02	1.0E-02
BLCA	SNRNP70_ES_8.1:8.2:8.3_7_9	SNRNP70	ES	8.1:8.2:8.3	7	9	1.9E-05	0.160	0.414	6.0E-02	5.6E-03	6.4E-02
BLCA	SNX11_ES_5_4_6	SNX11	ES	5	4	6	1.1E-04	0.134	-0.193	3.6E-01	1.9E-01	5.7E-02
BLCA	SNX29_ES_5_4_6	SNX29	ES	5	4	6	8.3E-05	0.144	-0.503	2.3E-03	4.7E-02	4.0E-01
BLCA	SON_ES_11_10_12	SON	ES	11	10	12	1.2E-05	0.165	0.097	1.0E-02	2.0E-03	3.7E-02
BLCA	SP140L_RI_16.2_16.1_16.3	SP140L	RI	16.2	16.1	16.3	9.3E-07	0.203	0.000	2.8E-02	2.7E-02	3.6E-02

cancer type	id	Gene Symbol	splice_type	Exon	From.Exon	To.Exon	anova.p	adj.r2	r	p.50	p.25	p.10
BLCA	SP140L_RI_16.4_16.3_16.5	SP140L	RI	16.4	16.3	16.5	5.7E-04	0.112	-0.039	4.0E-04	9.3E-02	1.1E-02
BLCA	SPAG9_ES_30_29_31	SPAG9	ES	30	29	31	1.7E-10	0.306	0.032	2.4E-01	3.2E-01	1.5E-01
BLCA	SPATA20_ES_5_4.2_6	SPATA20	ES	5	4.2	6	4.1E-04	0.117	-0.211	1.6E-01	2.0E-01	1.1E-01
BLCA	SPATA20_RI_11.2_11.1_11.3	SPATA20	RI	11.2	11.1	11.3	3.3E-06	0.185	0.329	4.4E-02	1.6E-02	7.7E-03
BLCA	SPATS2L_AD_1.3_1.2_4	SPATS2L	AD	1.3	1.2	4	9.3E-04	0.109	-0.118	1.7E-01	3.8E-01	2.2E-01
BLCA	SPIN1_ES_2:3:4_1_5	SPIN1	ES	2:03:04	1	5	3.0E-05	0.153	-0.400	2.3E-01	4.4E-02	5.9E-02
BLCA	SPTAN1_ES_23_22_24	SPTAN1	ES	23	22	24	1.0E-05	0.189	0.311	6.3E-01	3.9E-01	8.7E-01
BLCA	SREBF1_ES_2_1_3.2	SREBF1	ES	2	1	3.2	4.5E-04	0.115	0.657	7.1E-01	5.9E-01	3.8E-01
BLCA	SREK1_ES_4_3.2_5	SREK1	ES	4	3.2	5	1.1E-04	0.136	0.491	9.5E-02	2.8E-01	4.2E-03
BLCA	SRSF1_RI_3.2_3.1_3.3	SRSF1	RI	3.2	3.1	3.3	1.1E-05	0.167	0.073	2.1E-01	8.6E-04	5.5E-02
BLCA	SRSF11_AA_6.1:6.2:6.3:6.4_4.2_6.5	SRSF11	AA	6.1:6.2:6.3:6.4	4.2	6.5	4.8E-06	0.178	0.429	5.8E-01	9.6E-01	3.3E-01
BLCA	SRSF11_ES_5:6.1:6.2:6.3_4.2_6.5	SRSF11	ES	5:6.1:6.2:6.3	4.2	6.5	2.3E-04	0.124	0.643	1.6E-01	5.6E-02	1.2E-01
BLCA	SRSF11_ES_5:6.1:6.2:6.3:6.4_4.2_6.5	SRSF11	ES	5:6.1:6.2:6.3:6.4	4.2	6.5	8.9E-07	0.201	0.666	5.5E-01	3.7E-02	6.6E-03
BLCA	SRSF2_AA_2.3:2.4_2.1_2.5	SRSF2	AA	2.3:2.4	2.1	2.5	6.5E-08	0.235	0.247	3.5E-02	5.3E-03	8.6E-02
BLCA	SRSF2_AD_2.2:2.3_2.1_2.5	SRSF2	AD	2.2:2.3	2.1	2.5	2.6E-07	0.217	0.396	3.2E-02	1.7E-03	6.7E-03
BLCA	SRSF2_RI_2.2:2.3:2.4_2.1_2.5	SRSF2	RI	2.2:2.3:2.4	2.1	2.5	4.4E-06	0.180	0.413	4.4E-02	6.2E-04	1.1E-02
BLCA	SRSF4_ES_2:3:4_1_5	SRSF4	ES	2:03:04	1	5	8.5E-05	0.138	0.096	3.7E-02	9.6E-02	4.0E-02
BLCA	SRSF6_ES_3_2_4	SRSF6	ES	3	2	4	6.3E-04	0.108	0.445	3.3E-02	8.2E-02	2.3E-02
BLCA	SRSF7_AA_4.3:4.4:4.5_4.1_4.6	SRSF7	AA	4.3:4.4:4.5	4.1	4.6	1.6E-06	0.193	0.093	1.7E-03	7.5E-03	4.9E-03
BLCA	SRSF7_RI_4.2_4.1_4.3	SRSF7	RI	4.2	4.1	4.3	1.2E-05	0.167	0.121	5.3E-01	1.3E-02	9.0E-03
BLCA	SRSF7_RI_4.2:4.3:4.4:4.5_4.1_4.6	SRSF7	RI	4.2:4.3:4.4:4.5	4.1	4.6	7.4E-08	0.233	0.151	9.3E-02	1.1E-03	2.4E-04
BLCA	SRSF7_RI_4.5_4.4_4.6	SRSF7	RI	4.5	4.4	4.6	2.8E-05	0.154	0.178	6.4E-02	9.2E-03	2.1E-03
BLCA	ST7_ES_10_9_11.1	ST7	ES	10	9	11.1	4.1E-04	0.115	0.076	8.1E-02	8.0E-02	2.9E-02
BLCA	STEAP3_ES_3_1.1_5.3	STEAP3	ES	3	1.1	5.3	6.4E-04	0.109	-0.476	7.1E-01	6.9E-01	3.7E-01
BLCA	STRA13_ES_3.1:3.2_2_4.1	STRA13	ES	3.1:3.2	2	4.1	2.5E-04	0.122	-0.083	9.3E-03	1.9E-02	7.2E-02
BLCA	STRA6_AD_18.2_18.1_19	STRA6	AD	18.2	18.1	19	5.4E-08	0.237	-0.780	5.3E-02	7.2E-02	2.4E-02
BLCA	STRADB_AA_12.1_11_12.2	STRADB	AA	12.1	11	12.2	7.3E-05	0.144	-0.197	1.4E-02	2.8E-02	1.3E-01
BLCA	STX17_ES_6_5_7	STX17	ES	6	5	7	7.7E-08	0.236	-0.302	3.5E-02	1.3E-02	3.4E-03
BLCA	SULF2_ES_20_19.2_21	SULF2	ES	20	19.2	21	7.5E-06	0.175	-0.247	3.2E-01	6.5E-01	9.7E-01
BLCA	SULT1A2_AA_5.1_4_5.2	SULT1A2	AA	5.1	4	5.2	6.5E-04	0.116	0.289	2.4E-01	1.4E-01	6.4E-01
BLCA	SUPT20H_AA_21.1_20_21.2	SUPT20H	AA	21.1	20	21.2	1.3E-04	0.132	0.132	9.8E-02	5.7E-02	9.5E-02
BLCA	SUPT4H1_AD_2.4_2.3_3.2	SUPT4H1	AD	2.4	2.3	3.2	6.8E-06	0.177	-0.027	4.7E-01	7.7E-01	1.3E-01
BLCA	SUPT7L_RI_2.2_2.1_2.3	SUPT7L	RI	2.2	2.1	2.3	8.8E-05	0.138	0.281	3.7E-01	9.4E-03	2.5E-02
BLCA	SUPT7L_RI_2.2:2.3_2.1_2.4	SUPT7L	RI	2.2:2.3	2.1	2.4	2.7E-06	0.186	0.391	4.4E-03	3.6E-02	6.1E-03
BLCA	SUV420H1_ES_6_5_7	SUV420H1	ES	6	5	7	1.5E-05	0.164	0.073	4.1E-03	4.2E-02	8.4E-03
BLCA	SVIL_ES_10:11:12_9_13	SVIL	ES	10:11:12	9	13	1.2E-09	0.293	0.155	9.7E-01	7.4E-01	7.8E-01
BLCA	SVIL_ES_21_20_22	SVIL	ES	21	20	22	6.4E-07	0.218	0.343	7.7E-01	9.8E-01	6.0E-01
BLCA	SYK_ES_9_8_10	SYK	ES	9	8	10	2.1E-07	0.235	-0.160	7.0E-01	4.2E-01	8.9E-01
BLCA	SYTL2_AD_8.3_8.2_9	SYTL2	AD	8.3	8.2	9	1.4E-04	0.132	0.127	9.3E-01	1.2E-01	1.4E-01
BLCA	SYTL2_ES_11.2_10.3_12.2	SYTL2	ES	11.2	10.3	12.2	1.4E-05	0.190	-0.423	4.4E-02	1.1E-01	4.7E-01

cancer type	id	Gene Symbol	splice_type	Exon	From.Exon	To.Exon	anova.p	adj.r2	r	p.50	p.25	p.10
BLCA	SYTL2_ES_13_12.2_14	SYTL2	ES	13	12.2	14	1.6E-09	0.290	0.388	1.3E-01	2.7E-02	2.7E-01
BLCA	TAF1D_RI_12.4_12.3_12.5	TAF1D	RI	12.4	12.3	12.5	1.1E-05	0.167	0.002	3.3E-04	3.0E-03	9.5E-03
BLCA	TAGLN_RI_1.2_1.1_1.3	TAGLN	RI	1.2	1.1	1.3	2.0E-05	0.167	-0.562	1.0E-01	5.6E-01	2.7E-01
BLCA	TAMM41_ES_5_4_7	TAMM41	ES	5	4	7	7.6E-04	0.106	-0.069	4.1E-01	1.1E-01	6.9E-01
BLCA	TARBP2_AD_4.2_4.1_5.1	TARBP2	AD	4.2	4.1	5.1	4.5E-08	0.239	0.144	7.4E-02	8.4E-02	7.3E-02
BLCA	TARBP2_ES_4.2:5.1_4.1_5.2	TARBP2	ES	4.2:5.1	4.1	5.2	5.3E-04	0.116	0.013	3.3E-01	2.5E-01	6.6E-01
BLCA	TAZ_AD_3.2:3.3_3.1_4	TAZ	AD	3.2:3.3	3.1	4	8.5E-07	0.205	0.093	1.2E-01	4.5E-01	3.6E-02
BLCA	TBC1D10A_ES_11_10_12	TBC1D10A	ES	11	10	12	2.8E-04	0.120	-0.181	5.2E-03	1.4E-04	2.9E-04
BLCA	TBC1D20_RI_8.2_8.1_8.3	TBC1D20	RI	8.2	8.1	8.3	1.1E-05	0.167	0.251	7.7E-02	2.2E-01	9.0E-01
BLCA	TBC1D23_ES_15_14_16	TBC1D23	ES	15	14	16	9.3E-11	0.319	-0.037	9.5E-03	6.5E-02	1.7E-01
BLCA	TBC1D25_ES_3_2_4	TBC1D25	ES	3	2	4	5.1E-05	0.148	-0.121	7.6E-01	5.7E-01	1.9E-01
BLCA	TBL2_AA_4.1:4.2_1_4.3	TBL2	AA	4.1:4.2	1	4.3	2.0E-04	0.126	-0.256	9.6E-03	1.2E-01	3.6E-01
BLCA	TBXAS1_ES_8_7.1_9	TBXAS1	ES	8	7.1	9	8.8E-10	0.319	-0.743	5.4E-02	8.3E-02	4.7E-02
BLCA	TCERG1_ES_22_21_23	TCERG1	ES	22	21	23	2.9E-05	0.153	0.232	9.0E-03	2.7E-02	7.9E-02
BLCA	TCF3_ES_17.3_17.1_18.1	TCF3	ES	17.3	17.1	18.1	2.0E-04	0.136	-0.132	9.4E-01	2.6E-01	7.5E-02
BLCA	TCF3_ES_17.3:18.1_17.1_18.2	TCF3	ES	17.3:18.1	17.1	18.2	3.0E-06	0.199	-0.161	9.3E-01	3.4E-01	4.2E-02
BLCA	TCIRG1_RI_5.2_5.1_5.3	TCIRG1	RI	5.2	5.1	5.3	4.1E-04	0.115	0.121	4.2E-02	2.0E-02	4.9E-03
BLCA	TDP2_AD_2.2_2.1_3	TDP2	AD	2.2	2.1	3	1.5E-05	0.164	-0.068	3.3E-02	1.4E-01	4.3E-01
BLCA	TEAD2_ES_6_5_7.1	TEAD2	ES	6	5	7.1	5.9E-05	0.164	-0.176	2.3E-01	9.5E-01	5.5E-01
BLCA	TECR_ES_2:4_1_5.3	TECR	ES	2:04	1	5.3	7.3E-05	0.172	-0.275	7.6E-01	7.4E-01	8.2E-01
BLCA	TEFM_RI_3.3_3.2_3.4	TEFM	RI	3.3	3.2	3.4	3.7E-07	0.212	0.225	1.4E-01	5.2E-02	1.7E-01
BLCA	TEX30_RI_3.3_3.2_3.4	TEX30	RI	3.3	3.2	3.4	1.5E-04	0.135	-0.250	4.6E-03	2.1E-03	2.9E-02
BLCA	TGFBR3_ES_5_4.2_6	TGFBR3	ES	5	4.2	6	1.4E-04	0.131	-0.520	3.6E-01	6.7E-01	1.8E-01
BLCA	THBS3_AA_10.1_9_10.2	THBS3	AA	10.1	9	10.2	2.0E-04	0.130	-0.122	1.7E-03	9.4E-05	9.8E-03
BLCA	THUMPD2_ES_4_3_5	THUMPD2	ES	4	3	5	8.1E-04	0.105	0.067	8.2E-01	8.4E-01	8.9E-02
BLCA	THUMPD2_ES_8_7_9	THUMPD2	ES	8	7	9	1.6E-07	0.223	0.240	2.6E-01	3.3E-02	6.3E-01
BLCA	TIA1_ES_8_7_9.1	TIA1	ES	8	7	9.1	8.4E-04	0.104	0.312	1.7E-02	5.4E-03	2.1E-03
BLCA	TIAL1_ES_6_5.1_7	TIAL1	ES	6	5.1	7	1.5E-08	0.253	0.303	3.3E-03	5.5E-03	1.3E-02
BLCA	TJP1_AD_30.2_30.1_31	TJP1	AD	30.2	30.1	31	1.2E-07	0.227	-0.146	1.9E-01	2.7E-02	1.7E-01
BLCA	TLE2_RI_10.2_10.1_10.3	TLE2	RI	10.2	10.1	10.3	4.4E-05	0.174	0.275	7.7E-03	1.1E-02	4.2E-02
BLCA	TLE2_RI_10.2:10.3_10.1_10.4	TLE2	RI	10.2:10.3	10.1	10.4	9.8E-04	0.123	0.211	1.7E-03	8.4E-03	3.4E-02
BLCA	TMCO6_RI_4.4_4.3_4.5	TMCO6	RI	4.4	4.3	4.5	3.9E-04	0.117	0.274	3.2E-02	1.3E-03	4.1E-02
BLCA	TMEM104_AA_11.1_10_11.2	TMEM104	AA	11.1	10	11.2	6.9E-04	0.107	0.153	5.1E-01	3.5E-01	6.3E-01
BLCA	TMEM104_ME_3|4_2.2_5	TMEM104	ME	3|4	2.2	5	2.5E-04	0.122	-0.437	9.0E-03	1.9E-04	3.0E-04
BLCA	TMEM150A_AD_4.2_4.1_5	TMEM150A	AD	4.2	4.1	5	3.3E-05	0.152	0.100	1.4E-01	5.1E-01	1.4E-01
BLCA	TMEM150A_ES_6:7_5_8	TMEM150A	ES	6:07	5	8	1.2E-04	0.139	0.121	9.0E-02	5.8E-01	4.0E-02
BLCA	TMEM175_ES_2:3:4.1:4.2_1_5.1	TMEM175	ES	2:3:4.1:4.2	1	5.1	5.6E-04	0.111	0.379	1.9E-01	2.6E-02	2.0E-01
BLCA	TMEM175_ES_4.1:4.2_3_5.1	TMEM175	ES	4.1:4.2	3	5.1	2.6E-04	0.123	0.372	1.2E-03	1.3E-03	9.5E-03
BLCA	TMEM180_ES_4_3_5	TMEM180	ES	4	3	5	2.4E-05	0.156	-0.265	3.5E-02	1.9E-02	2.8E-02
BLCA	TMEM205_AA_2.5_2.1_2.6	TMEM205	AA	2.5	2.1	2.6	3.7E-04	0.124	-0.243	8.4E-01	3.0E-01	3.5E-01

cancer type	id	Gene Symbol	splice_type	Exon	From.Exon	To.Exon	anova.p	adj.r2	r	p.50	p.25	p.10
BLCA	TMEM205_AD_2.2:2.3_2.1_2.6	TMEM205	AD	2.2:2.3	2.1	2.6	8.0E-04	0.105	-0.071	2.0E-01	2.6E-01	5.8E-01
BLCA	TMEM205_RI_2.3:2.4_2.2_2.5	TMEM205	RI	2.3:2.4	2.2	2.5	2.6E-05	0.155	0.259	1.3E-01	4.1E-02	1.4E-02
BLCA	TMEM205_RI_2.4:2.5_2.3_2.6	TMEM205	RI	2.4:2.5	2.3	2.6	5.7E-04	0.110	0.059	4.7E-02	3.5E-03	1.1E-02
BLCA	TMEM33_RI_8.2_8.1_8.3	TMEM33	RI	8.2	8.1	8.3	4.9E-04	0.112	0.491	4.8E-02	4.3E-02	2.3E-01
BLCA	TMEM45A_ES_2:3_1_4	TMEM45A	ES	2:03	1	4	3.4E-05	0.163	0.417	5.2E-01	3.6E-01	1.4E-01
BLCA	TMEM55B_RI_2.2_2.1_2.3	TMEM55B	RI	2.2	2.1	2.3	6.8E-04	0.107	-0.041	2.3E-02	8.9E-03	1.2E-02
BLCA	TMEM91_RI_7.2:7.3_7.1_7.4	TMEM91	RI	7.2:7.3	7.1	7.4	2.1E-04	0.125	-0.502	5.4E-01	8.9E-01	4.5E-01
BLCA	TMUB1_RI_4.2_4.1_4.3	TMUB1	RI	4.2	4.1	4.3	4.3E-05	0.148	0.152	4.4E-03	3.6E-04	4.1E-03
BLCA	TMUB2_AA_4.2_3_4.3	TMUB2	AA	4.2	3	4.3	7.5E-05	0.141	-0.035	7.9E-01	8.8E-01	3.7E-01
BLCA	TMUB2_AD_2.2:2.3:2.4:2.5_2.1_4.3	TMUB2	AD	2.2:2.3:2.4:2.5	2.1	4.3	8.8E-04	0.103	0.148	9.4E-01	9.9E-01	6.5E-01
BLCA	TMUB2_AD_2.3:2.4:2.5_2.2_4.3	TMUB2	AD	2.3:2.4:2.5	2.2	4.3	5.4E-05	0.144	0.211	3.6E-01	1.4E-02	3.1E-02
BLCA	TMUB2_ES_2.2:2.3:2.4:2.5:3_2.1_4.3	TMUB2	ES	2.2:2.3:2.4:2.5:3	2.1	4.3	2.7E-05	0.163	0.075	7.4E-01	7.8E-01	6.3E-01
BLCA	TMUB2_ES_2.3:2.4:2.5:3_2.2_4.3	TMUB2	ES	2.3:2.4:2.5:3	2.2	4.3	4.6E-05	0.150	0.114	5.4E-01	7.2E-02	1.5E-01
BLCA	TMUB2_ES_4.7:4.8_4.5_5	TMUB2	ES	4.7:4.8	4.5	5	2.1E-04	0.139	0.152	2.9E-02	6.6E-02	2.1E-01
BLCA	TMUB2_ES_4.8_4.5_5	TMUB2	ES	4.8	4.5	5	8.5E-04	0.114	0.104	2.4E-02	5.2E-02	8.9E-01
BLCA	TMX2_ES_3.1:3.2:3.3_2_4	TMX2	ES	3.1:3.2:3.3	2	4	1.8E-04	0.130	-0.321	3.7E-02	2.4E-01	1.6E-03
BLCA	TNC_ES_12:13:14:15:16:19_11_20	TNC	ES	12:13:14:15:16:19	11	20	1.5E-10	0.327	0.471	7.3E-01	3.0E-01	8.8E-01
BLCA	TNFRSF10B_RI_5.2_5.1_5.3	TNFRSF10B	RI	5.2	5.1	5.3	9.5E-06	0.169	0.026	9.0E-01	6.1E-01	9.5E-02
BLCA	TOP1MT_ES_4_3_5	TOP1MT	ES	4	3	5	2.2E-04	0.128	-0.184	9.7E-01	5.2E-01	3.7E-01
BLCA	TOP3B_ES_6_5_7	TOP3B	ES	6	5	7	6.6E-04	0.109	0.340	2.4E-01	4.1E-02	7.9E-01
BLCA	TOP3B_RI_16.2_16.1_16.3	TOP3B	RI	16.2	16.1	16.3	6.4E-06	0.174	0.529	1.6E-02	5.5E-03	5.0E-04
BLCA	TP53I3_RI_1.2_1.1_1.3	TP53I3	RI	1.2	1.1	1.3	1.7E-05	0.160	-0.052	1.3E-03	2.9E-03	6.9E-03
BLCA	TPD52L1_ES_8_6_10	TPD52L1	ES	8	6	10	8.4E-04	0.108	-0.198	2.5E-01	3.4E-01	3.1E-01
BLCA	TPD52L1_ES_8_6_9.1	TPD52L1	ES	8	6	9.1	1.3E-04	0.149	-0.166	1.5E-01	1.1E-01	4.9E-01
BLCA	TPD52L1_ES_8:9.1_6_10	TPD52L1	ES	08:09.1	6	10	7.6E-04	0.110	-0.047	2.5E-01	3.7E-01	3.5E-01
BLCA	TPM1_AA_2.1_1_2.2	TPM1	AA	2.1	1	2.2	5.1E-04	0.121	-0.083	3.9E-01	6.0E-01	6.9E-01
BLCA	TPM1_ES_8_7_9	TPM1	ES	8	7	9	1.3E-04	0.133	-0.159	1.1E-01	3.8E-01	7.4E-01
BLCA	TPM2_ES_6_5_7	TPM2	ES	6	5	7	1.6E-09	0.307	-0.488	2.9E-03	2.7E-04	5.6E-05
BLCA	TPM2_ES_7_6_8	TPM2	ES	7	6	8	2.2E-08	0.263	-0.552	1.1E-04	1.0E-03	1.8E-02
BLCA	TRA2A_ES_3.1:3.2_1_4	TRA2A	ES	3.1:3.2	1	4	1.0E-04	0.135	0.296	2.7E-02	9.4E-02	2.3E-01
BLCA	TRERF1_ES_8.1_7_9	TRERF1	ES	8.1	7	9	4.3E-04	0.121	0.171	4.8E-01	8.7E-01	4.5E-01
BLCA	TRERF1_ES_8.1:8.2_7_9	TRERF1	ES	8.1:8.2	7	9	3.2E-06	0.197	0.126	3.5E-01	8.4E-01	5.4E-01
BLCA	TREX1_RI_2.5_2.4_2.6	TREX1	RI	2.5	2.4	2.6	3.7E-04	0.116	-0.194	2.3E-01	4.4E-01	4.6E-01
BLCA	TRIM13_RI_1.2:1.3_1.1_1.4	TRIM13	RI	1.2:1.3	1.1	1.4	9.1E-05	0.139	0.058	2.0E-01	9.6E-03	2.8E-03
BLCA	TRIM13_RI_1.3_1.2_1.4	TRIM13	RI	1.3	1.2	1.4	5.1E-06	0.180	0.061	6.4E-02	2.1E-02	5.8E-03
BLCA	TRIM33_ES_20_19_21	TRIM33	ES	20	19	21	1.9E-04	0.126	-0.145	3.3E-01	4.5E-01	4.5E-01
BLCA	TRIM5_RI_12.2_12.1_12.3	TRIM5	RI	12.2	12.1	12.3	4.0E-04	0.116	-0.207	3.3E-02	1.3E-01	6.6E-01
BLCA	TRMT1_RI_2.2_2.1_2.3	TRMT1	RI	2.2	2.1	2.3	2.8E-12	0.378	0.090	6.1E-02	5.9E-02	5.6E-01
BLCA	TRMU_AA_6.1_5_6.2	TRMU	AA	6.1	5	6.2	1.6E-04	0.130	-0.108	4.4E-04	1.1E-02	1.5E-03
BLCA	TRPC4AP_ES_9.2_8_10	TRPC4AP	ES	9.2	8	10	9.2E-06	0.171	-0.126	4.1E-02	2.0E-02	8.7E-02

cancer type	id	Gene Symbol	splice_type	Exon	From.Exon	To.Exon	anova.p	adj.r2	r	p.50	p.25	p.10
BLCA	TSC2_ES_27:28.1_26_28.2	TSC2	ES	27:28.1	26	28.2	4.8E-04	0.113	0.083	8.6E-02	1.8E-02	6.6E-01
BLCA	TSPAN17_RI_7.2_7.1_7.3	TSPAN17	RI	7.2	7.1	7.3	1.2E-04	0.133	-0.172	1.2E-02	9.2E-02	2.1E-02
BLCA	TSTD1_ES_2.2:2.3_1_3.1	TSTD1	ES	2.2:2.3	1	3.1	4.7E-05	0.149	0.235	2.8E-01	6.7E-02	5.5E-01
BLCA	TTC31_RI_9.2_9.1_9.3	TTC31	RI	9.2	9.1	9.3	5.1E-04	0.113	0.214	3.7E-02	4.4E-02	2.3E-02
BLCA	TTC7B_ES_18_17_19	TTC7B	ES	18	17	19	9.1E-07	0.202	-0.134	7.8E-01	8.1E-01	9.3E-01
BLCA	TUBB3_ES_6.1:6.2_5.4_7.1	TUBB3	ES	6.1:6.2	5.4	7.1	4.5E-05	0.148	-0.637	2.3E-02	1.9E-03	1.2E-01
BLCA	TUBGCP6_AD_16.2_16.1_17	TUBGCP6	AD	16.2	16.1	17	2.8E-05	0.156	0.500	3.0E-02	2.9E-03	1.7E-01
BLCA	TXNRD1_AD_4.2_4.1_6.2	TXNRD1	AD	4.2	4.1	6.2	5.8E-04	0.126	0.062	5.6E-01	3.7E-01	1.5E-01
BLCA	TYMP_AA_2.1_1_2.2	TYMP	AA	2.1	1	2.2	8.4E-04	0.111	-0.279	2.5E-01	7.2E-01	4.8E-01
BLCA	UAP1_ES_9.2_8_10	UAP1	ES	9.2	8	10	2.0E-05	0.160	-0.043	1.3E-01	1.2E-01	3.0E-01
BLCA	UBE2C_RI_1.3_1.2_1.4	UBE2C	RI	1.3	1.2	1.4	7.5E-05	0.153	-0.137	3.4E-01	4.2E-01	7.2E-01
BLCA	UBE3A_ES_4.1:4.2:5.1:5.2_3_6.2	UBE3A	ES	4.1:4.2:5.1:5.2	3	6.2	1.4E-04	0.142	-0.219	5.9E-01	1.6E-01	6.0E-01
BLCA	UBP1_ES_13_12_14	UBP1	ES	13	12	14	2.1E-06	0.190	-0.116	2.7E-04	3.5E-03	4.3E-01
BLCA	UBXN1_AA_8.1:8.2_7_8.3	UBXN1	AA	8.1:8.2	7	8.3	1.2E-04	0.134	-0.120	5.9E-01	6.1E-01	5.1E-01
BLCA	UBXN4_RI_3.2_3.1_3.3	UBXN4	RI	3.2	3.1	3.3	5.4E-07	0.208	0.085	2.4E-03	3.8E-02	7.5E-04
BLCA	ULK3_RI_6.2_6.1_6.3	ULK3	RI	6.2	6.1	6.3	1.2E-05	0.166	0.589	9.6E-04	3.8E-04	4.8E-03
BLCA	UNC119_RI_4.2_4.1_4.3	UNC119	RI	4.2	4.1	4.3	9.2E-06	0.169	0.243	7.1E-02	7.7E-02	5.2E-05
BLCA	UPF3B_ES_8_7_9	UPF3B	ES	8	7	9	1.0E-06	0.200	0.021	3.6E-01	1.9E-01	5.7E-01
BLCA	UPP1_ES_6.2:7_6.1_9	UPP1	ES	6.2:7	6.1	9	1.2E-05	0.168	0.187	5.5E-01	2.0E-01	3.4E-02
BLCA	USO1_ES_14_13_15	USO1	ES	14	13	15	2.3E-09	0.280	0.148	9.7E-01	1.0E+00	6.7E-01
BLCA	VEGFA_ES_7.1:7.2:8.1:8.2_6_9.1	VEGFA	ES	7.1:7.2:8.1:8.2	6	9.1	3.5E-04	0.117	0.348	3.0E-03	6.8E-02	2.7E-01
BLCA	VLDLR_ES_16_15_17	VLDLR	ES	16	15	17	7.4E-04	0.118	-0.220	1.8E-01	1.6E-01	1.1E-01
BLCA	VPS16_RI_12.3_12.2_12.4	VPS16	RI	12.3	12.2	12.4	1.4E-04	0.131	0.200	3.8E-01	3.6E-01	5.8E-02
BLCA	VPS29_ES_3.1_1_5	VPS29	ES	3.1	1	5	2.9E-11	0.326	-0.100	5.7E-01	1.0E+00	9.0E-01
BLCA	VPS39_ES_3_2_4	VPS39	ES	3	2	4	2.9E-06	0.198	0.157	4.6E-01	3.1E-01	4.7E-01
BLCA	VPS8_RI_7.2_7.1_7.3	VPS8	RI	7.2	7.1	7.3	1.4E-04	0.136	0.203	8.1E-01	1.6E-01	4.7E-01
BLCA	VTI1B_ES_2:3_1_4	VTI1B	ES	2:03	1	4	5.0E-04	0.114	-0.182	6.5E-01	4.8E-01	1.0E+00
BLCA	WDR11_RI_27.2_27.1_27.3	WDR11	RI	27.2	27.1	27.3	1.9E-05	0.159	0.057	1.2E-03	1.6E-02	1.7E-02
BLCA	WDR41_ES_3_2_4	WDR41	ES	3	2	4	5.5E-09	0.265	-0.518	5.9E-01	5.0E-02	6.0E-01
BLCA	WDR55_RI_2.2_2.1_2.3	WDR55	RI	2.2	2.1	2.3	4.5E-06	0.179	-0.174	3.8E-01	1.3E-01	1.2E-01
BLCA	WDR55_RI_4.2_4.1_4.3	WDR55	RI	4.2	4.1	4.3	1.2E-04	0.133	-0.037	8.2E-03	1.1E-02	2.2E-04
BLCA	WDR6_RI_4.5_4.4_4.6	WDR6	RI	4.5	4.4	4.6	4.9E-06	0.178	0.443	1.2E-02	1.9E-03	6.7E-03
BLCA	WDR62_RI_25.6_25.5_25.7	WDR62	RI	25.6	25.5	25.7	2.0E-04	0.126	-0.546	5.1E-03	4.2E-02	6.7E-01
BLCA	WDR62_RI_25.6:25.7_25.5_25.8	WDR62	RI	25.6:25.7	25.5	25.8	2.8E-07	0.216	-0.426	1.4E-02	2.8E-03	2.6E-02
BLCA	WHSC1_AA_16.1_15.2_16.2	WHSC1	AA	16.1	15.2	16.2	8.6E-06	0.170	-0.075	6.7E-03	1.0E-02	2.1E-01
BLCA	WIBG_ES_4_2_6	WIBG	ES	4	2	6	1.3E-04	0.133	-0.427	1.8E-01	2.1E-01	2.9E-01
BLCA	WIPI2_ES_2_1_3	WIPI2	ES	2	1	3	2.9E-06	0.188	0.113	2.0E-01	8.8E-02	3.3E-01
BLCA	WNK1_ES_15_13.2_16	WNK1	ES	15	13.2	16	5.1E-06	0.180	-0.143	1.1E-01	2.1E-01	8.2E-01
BLCA	XAF1_AA_4.1:4.2_2.1_4.3	XAF1	AA	4.1:4.2	2.1	4.3	1.9E-04	0.130	-0.002	2.1E-02	2.4E-02	3.5E-01
BLCA	YBX3_ES_6_5_7	YBX3	ES	6	5	7	1.3E-05	0.165	-0.102	7.5E-01	5.3E-01	6.2E-01

cancer type	id	Gene Symbol	splice_type	Exon	From.Exon	To.Exon	anova.p	adj.r2	r	p.50	p.25	p.10
BLCA	ZBED5_AA_3.1_2_3.2	ZBED5	AA	3.1	2	3.2	5.5E-05	0.147	0.453	2.3E-01	5.7E-03	1.7E-02
BLCA	ZCCHC8_RI_6.2_6.1_6.3	ZCCHC8	RI	6.2	6.1	6.3	3.9E-04	0.115	0.214	5.8E-01	2.8E-01	1.2E-03
BLCA	ZDHHC7_ES_4_3_5	ZDHHC7	ES	4	3	5	4.6E-06	0.180	0.027	5.8E-01	3.8E-01	4.9E-01
BLCA	ZFAND5_ES_3_1_4.1	ZFAND5	ES	3	1	4.1	7.9E-06	0.171	-0.007	3.8E-01	2.7E-01	3.2E-02
BLCA	ZFC3H1_RI_32.2_32.1_32.3	ZFC3H1	RI	32.2	32.1	32.3	2.4E-06	0.188	0.390	1.2E-02	3.0E-03	2.4E-02
BLCA	ZFP36_AD_1.2_1.1_2	ZFP36	AD	1.2	1.1	2	9.9E-04	0.101	0.031	2.2E-01	2.6E-01	6.6E-01
BLCA	ZMAT5_ES_2_1_3	ZMAT5	ES	2	1	3	3.3E-04	0.121	-0.140	8.2E-01	9.2E-01	2.3E-03
BLCA	ZNF248_RI_7.2_7.1_7.3	ZNF248	RI	7.2	7.1	7.3	9.6E-04	0.102	-0.060	4.2E-01	5.4E-02	1.1E-02
BLCA	ZNF410_ES_14_13_16	ZNF410	ES	14	13	16	9.8E-04	0.102	-0.411	1.1E-02	7.4E-02	6.4E-02
BLCA	ZNF576_AD_1.2:1.3_1.1_1.6	ZNF576	AD	1.2:1.3	1.1	1.6	7.9E-05	0.162	0.044	2.6E-01	9.8E-01	7.3E-01
BLCA	ZNF655_AA_5.1:5.2_4_5.3	ZNF655	AA	5.1:5.2	4	5.3	8.4E-04	0.108	0.222	6.4E-02	7.4E-03	8.2E-04
BLCA	ZNF692_RI_3.5_3.4_3.6	ZNF692	RI	3.5	3.4	3.6	1.6E-05	0.163	0.628	3.8E-02	1.3E-02	2.4E-02
BLCA	ZNF7_AA_5.1_4.2_5.2	ZNF7	AA	5.1	4.2	5.2	2.4E-06	0.225	0.125	1.7E-01	5.8E-01	8.5E-01
BLCA	ZNF7_ES_5.1:5.2_4.2_6.1	ZNF7	ES	5.1:5.2	4.2	6.1	1.7E-05	0.164	0.078	4.4E-01	6.8E-01	4.5E-01
BLCA	ZNF76_AA_10.1_9_10.2	ZNF76	AA	10.1	9	10.2	2.0E-06	0.196	0.273	9.8E-03	1.6E-03	3.1E-02
BLCA	ZNF76_RI_12.4_12.3_12.5	ZNF76	RI	12.4	12.3	12.5	9.4E-05	0.137	0.202	3.2E-02	5.2E-03	3.1E-04
BLCA	ZNF83_ES_8:9.6_7_10.1	ZNF83	ES	08:09.6	7	10.1	7.7E-04	0.114	0.443	6.9E-02	1.1E-01	2.4E-01
BLCA	ZWINT_RI_2.2_2.1_2.3	ZWINT	RI	2.2	2.1	2.3	6.7E-05	0.141	-0.130	5.5E-02	1.0E-03	8.5E-02
BRCA	ABCB9_ES_10_9_11	ABCB9	ES	10	9	11	5.1E-46	0.181	0.056	4.5E-01	8.7E-02	2.0E-02
BRCA	ABHD14B_AA_3.1:3.2_2.2_3.3	ABHD14B	AA	3.1:3.2	2.2	3.3	2.7E-44	0.173	-0.293	2.8E-01	8.6E-01	6.2E-01
BRCA	ABI1_ES_11.2_9_12	ABI1	ES	11.2	9	12	7.6E-28	0.114	-0.263	3.5E-02	1.6E-01	4.8E-01
BRCA	ABLIM3_ES_15_13_16.2	ABLIM3	ES	15	13	16.2	6.2E-27	0.127	-0.474	7.6E-01	8.5E-01	9.8E-01
BRCA	ACADVL_RI_3.2_3.1_3.3	ACADVL	RI	3.2	3.1	3.3	4.0E-23	0.118	-0.058	4.0E-01	6.8E-01	9.9E-01
BRCA	ACIN1_ES_4_3_5	ACIN1	ES	4	3	5	4.0E-66	0.249	0.179	9.4E-01	7.1E-01	8.9E-01
BRCA	ACIN1_ES_9.3_9.1_10	ACIN1	ES	9.3	9.1	10	1.7E-25	0.107	-0.076	9.0E-01	6.1E-01	7.9E-01
BRCA	ACOT2_AD_2.3_2.2_3	ACOT2	AD	2.3	2.2	3	7.2E-27	0.108	0.325	6.3E-04	3.4E-02	9.4E-02
BRCA	ACY1_ES_8.1:8.2:9:10:11:12_7.2_15	ACY1	ES	3.1:8.2:9:10:11:12	7.2	15	2.1E-44	0.184	-0.199	NA	NA	NA
BRCA	ADAM15_ES_21.1:21.2_20_22.1	ADAM15	ES	21.1:21.2	20	22.1	4.1E-174	0.524	-0.232	6.3E-01	6.4E-01	7.7E-01
BRCA	ADAM15_ES_21.1:21.2:22.1_20_23	ADAM15	ES	21.1:21.2:22.1	20	23	6.0E-56	0.212	0.141	4.8E-01	1.1E-01	4.2E-01
BRCA	ADAM15_ES_21.1:21.2:22.1:22.2_20_23	ADAM15	ES	1.1:21.2:22.1:22.	20	23	2.0E-38	0.151	-0.106	5.5E-01	6.6E-01	2.6E-01
BRCA	ADAM15_ES_21.2_20_22.1	ADAM15	ES	21.2	20	22.1	5.4E-150	0.472	-0.232	8.3E-01	7.5E-01	5.3E-01
BRCA	ADAM15_ES_21.2:22.1_20_23	ADAM15	ES	21.2:22.1	20	23	3.3E-48	0.186	0.145	2.0E-01	2.3E-01	6.0E-01
BRCA	ADAM15_ES_21.2:22.1:22.2_20_23	ADAM15	ES	21.2:22.1:22.2	20	23	2.8E-34	0.136	-0.118	3.8E-01	1.0E+00	3.3E-01
BRCA	ADAM15_ES_22.1_20_23	ADAM15	ES	22.1	20	23	3.1E-32	0.129	0.432	8.2E-01	3.2E-01	3.3E-01
BRCA	ADAM15_ES_22.1_21.2_23	ADAM15	ES	22.1	21.2	23	4.4E-92	0.325	-0.004	2.9E-01	2.9E-01	1.1E-01
BRCA	ADAM15_ES_22.1:22.2_21.2_23	ADAM15	ES	22.1:22.2	21.2	23	8.6E-80	0.289	-0.257	7.1E-01	3.0E-01	3.2E-01
BRCA	ADHFE1_AA_11.1_10_11.2	ADHFE1	AA	11.1	10	11.2	2.3E-26	0.114	-0.152	3.9E-01	4.7E-01	9.0E-01
BRCA	AFMID_ES_5:6:7:8:12_2_13	AFMID	ES	5:6:7:8:12	2	13	2.1E-54	0.255	0.226	5.3E-02	5.9E-01	9.4E-01
BRCA	AFMID_ES_5:6:7:8:9:10:11.1:11.2:12_2_	AFMID	ES	7:8:9:10:11.1:11.	2	13	2.6E-57	0.232	0.131	5.2E-01	8.4E-01	8.9E-01
BRCA	AFMID_ES_5:6:7:8:9:10:11.1:12_2_13	AFMID	ES	:6:7:8:9:10:11.1:1	2	13	1.5E-76	0.286	0.152	8.6E-01	5.8E-01	6.8E-01

cancer type	id	Gene Symbol	splice_type	Exon	From.Exon	To.Exon	anova.p	adj.r2	r	p.50	p.25	p.10
BRCA	AFMID_ES_5:6:7:8:9:10:12_2_13	AFMID	ES	5:6:7:8:9:10:12	2	13	2.7E-40	0.192	0.126	9.0E-01	5.1E-01	8.5E-01
BRCA	AFMID_ES_5:6:8:9:10:11.1:11.2:12_2_13	AFMID	ES	:8:9:10:11.1:11.2	2	13	1.3E-40	0.188	0.279	9.2E-01	9.2E-01	2.4E-01
BRCA	AFMID_ES_5:6:8:9:10:11.1:12_2_13	AFMID	ES	5:6:8:9:10:11.1:12	2	13	4.4E-49	0.226	0.258	7.8E-01	5.9E-01	6.5E-01
BRCA	AFMID_ES_7_6_8	AFMID	ES	7	6	8	9.1E-47	0.188	0.025	6.3E-02	2.0E-01	5.2E-01
BRCA	AFMID_ES_7:8:9:10:11.1_6_12	AFMID	ES	7:8:9:10:11.1	6	12	3.5E-37	0.147	0.088	7.7E-01	6.3E-01	8.3E-01
BRCA	AFMID_ES_7:8:9:10:11.1:11.2_6_12	AFMID	ES	7:8:9:10:11.1:11.2	6	12	7.6E-30	0.120	0.062	4.2E-01	4.6E-01	4.9E-01
BRCA	AFMID_ES_7:8:9:10:11.1:11.2:12_6_13	AFMID	ES	8:9:10:11.1:11.2:	6	13	7.8E-50	0.204	0.120	8.8E-01	8.9E-01	7.7E-01
BRCA	AFMID_ES_7:8:9:10:11.1:12_6_13	AFMID	ES	7:8:9:10:11.1:12	6	13	1.1E-63	0.251	0.139	7.8E-01	9.9E-01	9.3E-01
BRCA	AFMID_ES_7:8:9:10:12_6_13	AFMID	ES	7:8:9:10:12	6	13	2.6E-29	0.130	0.087	1.9E-01	5.1E-01	9.3E-01
BRCA	AFMID_ES_8_6_12	AFMID	ES	8	6	12	2.6E-28	0.120	-0.008	4.9E-01	3.5E-01	3.7E-01
BRCA	AFMID_ES_8:9:10:11.1:12_6_13	AFMID	ES	8:9:10:11.1:12	6	13	8.3E-26	0.113	0.187	1.6E-01	2.4E-01	7.8E-01
BRCA	AFMID_ES_9:10:11.1_8_12	AFMID	ES	10:11.1	8	12	1.1E-56	0.221	0.120	1.1E-01	5.2E-01	6.2E-01
BRCA	AFMID_ES_9:10:11.1:11.2_8_12	AFMID	ES	9:10:11.1:11.2	8	12	5.1E-43	0.174	0.086	8.7E-02	3.5E-01	1.9E-02
BRCA	AFTPH_ES_7:8_6_9	AFTPH	ES	7:08	6	9	1.2E-51	0.250	0.567	2.1E-01	6.4E-01	7.4E-01
BRCA	AKAP11_ES_10_9_11	AKAP11	ES	10	9	11	5.7E-53	0.212	0.191	6.9E-01	5.8E-01	3.9E-01
BRCA	AKAP13_ES_12_11_14	AKAP13	ES	12	11	14	5.7E-37	0.169	0.238	6.8E-01	5.3E-01	6.6E-01
BRCA	AKAP8L_ES_10_9_11	AKAP8L	ES	10	9	11	1.4E-41	0.163	0.331	8.0E-01	4.5E-01	1.0E+00
BRCA	AKAP9_ES_20_19_21	AKAP9	ES	20	19	21	4.5E-30	0.142	-0.338	1.8E-01	4.7E-01	1.8E-01
BRCA	AKIP1_RI_1.2_1.1_1.3	AKIP1	RI	1.2	1.1	1.3	6.8E-32	0.132	-0.120	5.3E-01	7.2E-01	1.9E-01
BRCA	AKR1E2_ES_5:6:7_4_8	AKR1E2	ES	5:06:07	4	8	3.7E-27	0.140	-0.214	8.2E-01	8.2E-01	7.7E-01
BRCA	ALAS1_ES_2_1_3.1	ALAS1	ES	2	1	3.1	8.7E-58	0.222	0.101	9.3E-01	9.9E-01	8.7E-01
BRCA	ALDH18A1_AD_6.2_6.1_7	ALDH18A1	AD	6.2	6.1	7	1.4E-42	0.169	0.087	7.2E-01	8.9E-01	1.5E-01
BRCA	ALG2_ES_2_1_3	ALG2	ES	2	1	3	3.0E-80	0.291	-0.360	6.7E-01	7.2E-01	7.4E-01
BRCA	ALS2_ES_24_23_25	ALS2	ES	24	23	25	7.7E-30	0.131	-0.112	9.7E-01	5.3E-01	3.5E-01
BRCA	ANAPC10_ES_1.2:2_1.1_3	ANAPC10	ES	1.2:2	1.1	3	2.5E-37	0.175	0.002	9.4E-01	4.7E-01	9.7E-01
BRCA	ANAPC11_AD_3.3:3.4_3.2_6	ANAPC11	AD	3.3:3.4	3.2	6	5.7E-42	0.164	0.092	5.0E-01	8.0E-01	6.3E-01
BRCA	ANAPC11_ES_3.3:3.4:6_3.2_7.2	ANAPC11	ES	3.3:3.4:6	3.2	7.2	1.1E-31	0.127	-0.136	9.0E-01	4.0E-01	4.8E-01
BRCA	ANAPC11_ES_3.4:6_3.3_7.2	ANAPC11	ES	3.4:6	3.3	7.2	1.6E-39	0.156	-0.207	6.5E-01	8.8E-01	9.5E-01
BRCA	ANAPC15_ES_3.2_1.2_4.1	ANAPC15	ES	3.2	1.2	4.1	6.6E-32	0.128	-0.115	6.1E-01	7.1E-01	3.9E-01
BRCA	ANK3_ME_26|27_25_29.1	ANK3	ME	26|27	25	29.1	2.8E-47	0.221	-0.128	6.7E-01	4.8E-01	1.6E-02
BRCA	ANKMY1_ES_13:14_12_15	ANKMY1	ES	13:14	12	15	3.8E-41	0.188	0.142	7.7E-02	9.9E-01	7.6E-01
BRCA	ANKRD36_ES_73_72_74	ANKRD36	ES	73	72	74	5.4E-25	0.107	0.055	2.2E-01	2.4E-02	5.6E-01
BRCA	ANKS3_ES_7.1:7.2_6_8	ANKS3	ES	7.1:7.2	6	8	1.3E-24	0.100	0.210	6.1E-01	4.6E-01	2.4E-01
BRCA	AP1B1_ES_24_23_25	AP1B1	ES	24	23	25	3.1E-31	0.125	-0.209	9.0E-01	9.6E-01	3.8E-01
BRCA	AP2B1_ES_16_15_17	AP2B1	ES	16	15	17	1.0E-37	0.149	-0.404	4.6E-02	1.4E-01	5.6E-02
BRCA	AP3D1_AA_24.1_23_24.2	AP3D1	AA	24.1	23	24.2	7.1E-26	0.105	-0.038	7.9E-01	7.6E-01	1.5E-01
BRCA	APBB2_ES_8_7.2_9	APBB2	ES	8	7.2	9	1.1E-25	0.106	-0.310	3.3E-01	1.0E-01	1.9E-01
BRCA	APLP2_ES_9_8_10	APLP2	ES	9	8	10	6.0E-41	0.160	0.081	2.8E-01	9.2E-01	3.8E-01
BRCA	APTX_ES_6.1:6.2_5.2_7.1	APTX	ES	6.1:6.2	5.2	7.1	2.1E-25	0.107	-0.028	9.1E-01	7.2E-01	1.6E-01
BRCA	ARAP1_ES_32_31_33	ARAP1	ES	32	31	33	2.3E-32	0.129	0.193	5.0E-01	8.4E-01	7.8E-01

cancer type	id	Gene Symbol	splice_type	Exon	From.Exon	To.Exon	anova.p	adj.r2	r	p.50	p.25	p.10
BRCA	ARFIP1_ES_4_3_5	ARFIP1	ES	4	3	5	1.9E-37	0.150	0.266	5.4E-01	7.7E-01	4.3E-01
BRCA	ARFIP2_AA_2.1_1_2.2	ARFIP2	AA	2.1	1	2.2	2.7E-31	0.125	-0.229	5.6E-02	6.4E-01	3.7E-01
BRCA	ARHGEF1_ES_29_28_30	ARHGEF1	ES	29	28	30	1.5E-36	0.144	0.122	3.1E-01	3.6E-01	1.0E+00
BRCA	ARHGEF11_ES_39_38_40	ARHGEF11	ES	39	38	40	4.8E-32	0.129	-0.366	3.5E-01	5.7E-01	3.4E-01
BRCA	ARHGEF12_ES_5_4_6	ARHGEF12	ES	5	4	6	7.3E-58	0.242	-0.161	9.1E-01	9.2E-01	1.9E-01
BRCA	ARIH2_ES_5_4.2_6	ARIH2	ES	5	4.2	6	3.8E-37	0.149	0.050	5.8E-01	6.5E-01	9.3E-01
BRCA	ARL16_ES_3:4_2.3_5	ARL16	ES	3:04	2.3	5	4.1E-26	0.105	0.148	2.4E-01	3.0E-01	3.7E-01
BRCA	ARL6IP4_AD_1.2_1.1_2.1	ARL6IP4	AD	1.2	1.1	2.1	1.2E-24	0.110	0.103	4.6E-01	5.1E-01	3.4E-01
BRCA	ARMC6_AD_1.2_1.1_5	ARMC6	AD	1.2	1.1	5	1.4E-73	0.274	0.493	5.3E-01	9.8E-01	8.3E-01
BRCA	ARMCX2_AA_4.2_3.2_4.3	ARMCX2	AA	4.2	3.2	4.3	3.0E-31	0.132	0.205	2.9E-01	6.0E-02	2.0E-01
BRCA	ARMCX3_AA_3.1_1_3.2	ARMCX3	AA	3.1	1	3.2	1.9E-28	0.117	-0.141	3.9E-01	9.2E-02	8.1E-01
BRCA	ARNT_ES_7_6_8	ARNT	ES	7	6	8	5.6E-45	0.181	-0.023	6.3E-01	7.6E-01	9.3E-01
BRCA	ARSA_RI_1.3:1.4_1.2_1.5	ARSA	RI	1.3:1.4	1.2	1.5	1.4E-33	0.133	-0.021	5.8E-02	2.3E-01	2.7E-01
BRCA	ARVCF_ES_20_19_21	ARVCF	ES	20	19	21	1.6E-137	0.508	0.247	7.2E-01	4.4E-01	1.7E-01
BRCA	ASAP2_ES_23_22_24	ASAP2	ES	23	22	24	1.1E-23	0.100	-0.089	4.8E-01	7.3E-01	9.8E-01
BRCA	ASNS_AA_11.1_10_11.2	ASNS	AA	11.1	10	11.2	4.9E-48	0.186	-0.338	1.3E-01	1.6E-01	5.4E-01
BRCA	ASXL1_ES_11_10_12	ASXL1	ES	11	10	12	1.9E-35	0.181	0.003	7.4E-01	6.8E-01	5.2E-01
BRCA	ATF2_ES_7_6_8	ATF2	ES	7	6	8	5.4E-41	0.165	0.048	1.6E-01	6.1E-01	7.0E-01
BRCA	ATG16L1_ES_8_7_10	ATG16L1	ES	8	7	10	1.3E-75	0.281	-0.413	8.9E-02	3.8E-01	1.6E-01
BRCA	ATG16L2_AA_4.1:4.2_3_4.3	ATG16L2	AA	4.1:4.2	3	4.3	9.8E-30	0.120	-0.108	9.7E-01	8.3E-01	8.8E-01
BRCA	ATG4D_ES_4.1:5_3.2_6	ATG4D	ES	4.1:5	3.2	6	6.8E-20	0.103	0.050	5.2E-01	2.6E-01	2.3E-01
BRCA	ATHL1_AA_4.1_3_4.2	ATHL1	AA	4.1	3	4.2	4.5E-23	0.103	0.128	6.1E-01	5.2E-01	1.0E-01
BRCA	ATIC_RI_1.4_1.3_1.5	ATIC	RI	1.4	1.3	1.5	1.7E-27	0.115	-0.003	1.5E-01	6.2E-01	9.4E-01
BRCA	ATP2C2_ES_24_23_25	ATP2C2	ES	24	23	25	4.5E-32	0.140	-0.209	1.1E-01	4.7E-01	6.7E-01
BRCA	ATP5J_AD_1.3_1.2_3	ATP5J	AD	1.3	1.2	3	1.8E-28	0.114	-0.182	8.4E-01	5.8E-01	1.3E-01
BRCA	ATP6V0A1_AA_6.1_5_6.2	ATP6V0A1	AA	6.1	5	6.2	8.3E-31	0.134	-0.237	3.1E-01	6.0E-01	8.6E-01
BRCA	ATXN2_ES_25_23_26	ATXN2	ES	25	23	26	2.9E-50	0.193	0.105	8.8E-02	8.9E-01	5.2E-01
BRCA	AURKAIP1_RI_1.4_1.3_1.5	AURKAIP1	RI	1.4	1.3	1.5	1.8E-31	0.129	0.053	9.4E-01	9.3E-01	6.2E-01
BRCA	AXIN1_ES_9_8_10	AXIN1	ES	9	8	10	3.5E-25	0.102	0.281	1.6E-01	1.4E-01	5.5E-01
BRCA	BABAM1_AA_2.1_1.2_2.2	BABAM1	AA	2.1	1.2	2.2	3.4E-39	0.154	0.001	7.2E-01	4.0E-01	6.4E-01
BRCA	BCAT2_ES_4:5_1_6	BCAT2	ES	4:05	1	6	5.8E-136	0.439	0.120	7.8E-02	9.5E-01	9.6E-01
BRCA	BCS1L_AD_1.5:1.6:1.7:1.8_1.4_2	BCS1L	AD	1.5:1.6:1.7:1.8	1.4	2	3.3E-29	0.119	-0.113	1.2E-01	7.9E-02	7.7E-01
BRCA	BCS1L_ES_1.4_1.1_2	BCS1L	ES	1.4	1.1	2	4.7E-61	0.249	0.083	7.9E-01	4.7E-01	5.1E-01
BRCA	BCS1L_ES_1.4:1.5:1.6_1.1_2	BCS1L	ES	1.4:1.5:1.6	1.1	2	3.7E-23	0.108	0.105	NA	NA	NA
BRCA	BCS1L_RI_1.2:1.3_1.1_1.4	BCS1L	RI	1.2:1.3	1.1	1.4	5.3E-41	0.168	-0.082	3.1E-01	1.0E+00	6.4E-01
BRCA	BECN1_ES_10_9_12	BECN1	ES	10	9	12	1.5E-28	0.144	0.408	5.0E-01	5.7E-01	5.4E-01
BRCA	BICD2_RI_7.2_7.1_7.3	BICD2	RI	7.2	7.1	7.3	2.1E-73	0.270	-0.188	9.2E-01	7.0E-01	6.7E-01
BRCA	BOLA1_ES_2.2:3.1_2.1_3.2	BOLA1	ES	2.2:3.1	2.1	3.2	1.4E-24	0.103	-0.184	6.4E-01	9.0E-01	5.5E-01
BRCA	BSCL2_AD_4.2:4.3_4.1_5	BSCL2	AD	4.2:4.3	4.1	5	2.3E-29	0.118	-0.146	3.4E-01	3.4E-01	1.0E+00
BRCA	BTN2A1_ES_4_3_5	BTN2A1	ES	4	3	5	6.0E-34	0.137	-0.149	6.3E-01	1.3E-01	1.1E-02

cancer type	id	Gene Symbol	splice_type	Exon	From.Exon	To.Exon	anova.p	adj.r2	r	p.50	p.25	p.10
BRCA	BUD31_AD_1.2_1.1_2	BUD31	AD	1.2	1.1	2	3.4E-42	0.165	0.109	3.6E-01	8.7E-01	5.8E-01
BRCA	C12orf73_RI_2.3_2.2_2.4	C12orf73	RI	2.3	2.2	2.4	6.6E-32	0.127	-0.204	6.8E-01	4.2E-01	6.5E-01
BRCA	C14orf2_ES_3:5_2_7.1	C14orf2	ES	3:05	2	7.1	7.8E-42	0.171	-0.084	4.4E-01	9.4E-01	2.2E-02
BRCA	C14orf2_ME_3|4_2_5	C14orf2	ME	3|4	2	5	1.0E-54	0.210	-0.040	8.5E-01	6.5E-01	3.0E-01
BRCA	C14orf80_ES_9_8_10	C14orf80	ES	9	8	10	1.9E-39	0.155	-0.152	4.6E-01	3.1E-01	1.9E-01
BRCA	C16orf13_ES_2_1_3	C16orf13	ES	2	1	3	6.5E-235	0.631	0.088	2.1E-01	4.9E-01	7.3E-01
BRCA	C16orf13_ES_2_1_5	C16orf13	ES	2	1	5	1.8E-121	0.407	0.076	8.9E-01	8.7E-01	3.9E-01
BRCA	C16orf13_ES_2:3_1_5	C16orf13	ES	2:03	1	5	2.0E-115	0.388	0.090	2.4E-01	5.2E-01	6.1E-01
BRCA	C16orf13_ES_3_1_4	C16orf13	ES	3	1	4	7.1E-90	0.361	-0.043	7.5E-01	9.8E-01	7.8E-02
BRCA	C16orf13_ES_4_1_5	C16orf13	ES	4	1	5	1.7E-54	0.248	0.049	6.5E-01	3.8E-01	6.9E-01
BRCA	C16orf91_RI_4.2_4.1_4.3	C16orf91	RI	4.2	4.1	4.3	6.0E-25	0.102	0.107	4.8E-01	7.8E-01	8.4E-02
BRCA	C16orf93_ES_4.1:4.3:5_3.3_7	C16orf93	ES	4.1:4.3:5	3.3	7	2.3E-31	0.149	0.290	6.9E-01	7.2E-01	6.7E-01
BRCA	C19orf60_ES_3.2_2_4	C19orf60	ES	3.2	2	4	1.6E-75	0.295	0.008	5.0E-01	3.0E-01	1.9E-01
BRCA	C1orf159_AA_5.1:5.2:5.3:5.4_4.4_5.5	C1orf159	AA	5.1:5.2:5.3:5.4	4.4	5.5	5.8E-29	0.118	0.079	1.8E-01	4.2E-01	8.8E-01
BRCA	C1orf159_ES_11.3_11.1_12	C1orf159	ES	11.3	11.1	12	1.1E-32	0.136	-0.103	6.8E-02	1.2E-01	6.1E-01
BRCA	C1orf51_AA_2.2_1_2.3	C1orf51	AA	2.2	1	2.3	1.4E-21	0.113	-0.042	5.2E-01	9.3E-01	5.5E-01
BRCA	C1orf86_ES_6_5_7.1	C1orf86	ES	6	5	7.1	5.5E-66	0.245	-0.452	8.5E-01	1.9E-01	5.9E-01
BRCA	C20orf96_ES_3_2.2_4	C20orf96	ES	3	2.2	4	7.1E-43	0.167	0.274	9.3E-01	4.5E-01	1.2E-02
BRCA	C21orf59_AD_5.2:5.3_5.1_5.5	C21orf59	AD	5.2:5.3	5.1	5.5	7.5E-28	0.112	-0.083	8.6E-01	5.8E-01	5.7E-01
BRCA	C22orf29_RI_1.2:1.3_1.1_1.4	C22orf29	RI	1.2:1.3	1.1	1.4	8.6E-29	0.118	0.224	6.4E-01	1.8E-02	7.8E-03
BRCA	C22orf29_RI_1.3_1.2_1.4	C22orf29	RI	1.3	1.2	1.4	3.8E-47	0.191	0.146	1.5E-01	6.1E-01	7.2E-01
BRCA	C2ORF15_ES_3_2_4	C2ORF15	ES	3	2	4	1.9E-34	0.151	-0.236	1.2E-01	3.1E-01	6.2E-02
BRCA	C6orf89_ES_6_4_7	C6orf89	ES	6	4	7	8.7E-25	0.102	0.007	5.2E-01	7.5E-01	8.4E-01
BRCA	C8orf59_ES_2.1:2.2_1_3.2	C8orf59	ES	2.1:2.2	1	3.2	5.2E-32	0.128	0.088	1.1E-01	5.4E-02	4.9E-01
BRCA	C9orf117_RI_5.2_5.1_5.3	C9orf117	RI	5.2	5.1	5.3	1.5E-25	0.114	-0.516	5.3E-01	5.1E-01	2.3E-01
BRCA	C9orf117_RI_5.4_5.3_5.5	C9orf117	RI	5.4	5.3	5.5	3.3E-30	0.125	-0.503	6.8E-02	3.8E-01	4.0E-01
BRCA	C9orf9_RI_5.2_5.1_5.3	C9orf9	RI	5.2	5.1	5.3	6.4E-28	0.112	-0.056	8.8E-01	7.4E-01	9.2E-01
BRCA	CA12_ES_9_8_10	CA12	ES	9	8	10	1.2E-166	0.534	0.622	6.9E-02	1.9E-01	5.2E-01
BRCA	CA5B_ES_4_3_5	CA5B	ES	4	3	5	4.5E-37	0.149	0.688	1.8E-01	2.4E-02	2.0E-01
BRCA	CALD1_AD_8.3_8.2_9	CALD1	AD	8.3	8.2	9	3.5E-39	0.162	-0.212	6.4E-01	3.3E-01	8.6E-01
BRCA	CAMK2G_ES_19.1:19.2_18_21	CAMK2G	ES	19.1:19.2	18	21	4.9E-29	0.117	-0.061	8.7E-01	7.6E-01	6.9E-01
BRCA	CAMLG_ES_2_1_4	CAMLG	ES	2	1	4	8.4E-40	0.171	-0.275	7.2E-01	3.2E-01	9.8E-01
BRCA	CANT1_ES_2_1_3.1	CANT1	ES	2	1	3.1	8.9E-43	0.170	0.334	9.2E-01	4.6E-01	1.7E-01
BRCA	CARD8_ES_2.3_2.1_3	CARD8	ES	2.3	2.1	3	8.9E-25	0.100	-0.035	4.0E-02	5.1E-01	1.1E-01
BRCA	CASK_ES_20_19.1_21	CASK	ES	20	19.1	21	2.4E-41	0.172	0.082	9.6E-01	9.2E-01	3.9E-01
BRCA	CASK_ES_20_19.1_22	CASK	ES	20	19.1	22	2.7E-25	0.108	0.145	8.3E-01	5.1E-01	8.5E-01
BRCA	CASK_ES_20:21_19.1_22	CASK	ES	20:21	19.1	22	3.5E-28	0.122	0.059	2.1E-01	9.2E-01	5.1E-01
BRCA	CC2D2A_ES_5_4_6	CC2D2A	ES	5	4	6	5.9E-103	0.366	-0.216	2.1E-01	3.0E-02	1.5E-01
BRCA	CCDC106_RI_1.2_1.1_1.3	CCDC106	RI	1.2	1.1	1.3	6.9E-34	0.136	0.156	3.1E-01	3.3E-01	3.4E-01
BRCA	CCDC107_RI_3.4_3.3_3.5	CCDC107	RI	3.4	3.3	3.5	3.3E-41	0.161	-0.158	9.2E-01	3.7E-01	5.7E-01

cancer type	id	Gene Symbol	splice_type	Exon	From.Exon	To.Exon	anova.p	adj.r2	r	p.50	p.25	p.10
BRCA	CCDC50_ES_6_5_7	CCDC50	ES	6	5	7	5.5E-58	0.219	-0.158	3.2E-01	6.8E-01	2.4E-01
BRCA	CCDC64_ES_6_5_8	CCDC64	ES	6	5	8	1.6E-26	0.108	0.087	5.0E-01	4.3E-01	6.8E-01
BRCA	CCDC90B_AD_1.3_1.2_2	CCDC90B	AD	1.3	1.2	2	9.0E-57	0.215	0.087	3.6E-01	1.6E-01	3.8E-02
BRCA	CCDC90B_AD_1.3:1.4:1.5_1.2_2	CCDC90B	AD	1.3:1.4:1.5	1.2	2	6.7E-97	0.339	0.096	2.9E-01	3.7E-01	4.4E-01
BRCA	CCNT2_AD_7.2_7.1_8	CCNT2	AD	7.2	7.1	8	4.7E-24	0.102	-0.067	4.1E-01	2.9E-01	2.2E-01
BRCA	CCT3_ME_2|3_1_4	CCT3	ME	2|3	1	4	2.1E-32	0.129	0.306	7.4E-01	5.3E-01	1.8E-01
BRCA	CD44_ES_3.1:3.2:4:5:6:7:8:9.1:9.2:10:11	CD44	ES	.2:10:11:12.1:13	2.1	17.2	4.8E-32	0.132	0.023	5.3E-01	5.7E-01	3.2E-02
BRCA	CD44_ES_3.1:3.2:4:5:6:7:8:9.2:10:11:12.	CD44	ES	:10:11:12.1:13:1	2.1	17.2	3.0E-32	0.134	0.014	3.9E-01	5.4E-01	1.8E-02
BRCA	CD44_ES_6_5_7	CD44	ES	6	5	7	9.4E-36	0.146	-0.064	2.9E-01	4.3E-02	2.7E-01
BRCA	CD44_ES_6:7:8:9.1:9.2_5_10	CD44	ES	6:7:8:9.1:9.2	5	10	2.5E-35	0.146	-0.074	4.4E-01	4.4E-01	5.3E-01
BRCA	CD44_ES_6:7:8:9.1:9.2:10:11_5_12.1	CD44	ES	:7:8:9.1:9.2:10:1	5	12.1	6.0E-39	0.155	-0.095	8.9E-01	9.1E-01	3.3E-01
BRCA	CD44_ES_6:7:8:9.1:9.2:10:11:12.1:13_5_	CD44	ES	:9.1:9.2:10:11:12	5	14	2.3E-34	0.140	0.172	5.5E-01	7.4E-01	6.4E-01
BRCA	CD44_ES_6:7:8:9.1:9.2:10:11:12.1:13:14	CD44	ES	.1:9.2:10:11:12.1	5	15	5.2E-26	0.105	0.405	4.2E-01	7.9E-01	4.7E-01
BRCA	CD44_ES_6:7:8:9.2_5_10	CD44	ES	6:7:8:9.2	5	10	1.4E-32	0.135	-0.077	6.6E-01	7.1E-01	7.3E-01
BRCA	CD44_ES_6:7:8:9.2:10:11_5_12.1	CD44	ES	6:7:8:9.2:10:11	5	12.1	5.4E-35	0.141	-0.090	7.3E-01	3.2E-01	3.6E-01
BRCA	CD44_ES_6:7:8:9.2:10:11:12.1:13_5_14	CD44	ES	:8:9.2:10:11:12.1	5	14	7.8E-38	0.154	0.149	9.5E-01	4.8E-01	3.6E-01
BRCA	CD46_ES_13_12_14	CD46	ES	13	12	14	6.3E-109	0.371	-0.516	3.9E-01	3.1E-01	4.4E-01
BRCA	CD46_ES_7:8:9_6_10	CD46	ES	7:08:09	6	10	1.1E-34	0.141	0.147	6.4E-01	1.8E-01	5.6E-01
BRCA	CD46_ES_8_6_10	CD46	ES	8	6	10	9.6E-55	0.268	0.273	9.8E-01	5.2E-01	6.5E-01
BRCA	CD47_ES_8:9:10_7_11	CD47	ES	8:09:10	7	11	4.3E-121	0.453	-0.190	9.6E-03	4.7E-02	9.2E-01
BRCA	CD47_ES_9:10_8_11	CD47	ES	9:10	8	11	9.8E-167	0.508	-0.138	2.3E-01	6.3E-01	9.4E-01
BRCA	CDC42BPB_ES_23_22_24	CDC42BPB	ES	23	22	24	3.1E-28	0.116	0.262	7.6E-01	6.2E-02	7.8E-01
BRCA	CDC42SE1_ES_5_4_6	CDC42SE1	ES	5	4	6	2.5E-86	0.308	-0.078	7.1E-01	3.7E-01	7.2E-01
BRCA	CDH24_ES_9_8_10	CDH24	ES	9	8	10	7.4E-35	0.148	-0.048	2.0E-01	8.0E-01	8.9E-01
BRCA	CDKL1_AA_9.1_8_9.2	CDKL1	AA	9.1	8	9.2	5.6E-24	0.107	-0.539	9.5E-02	1.0E-01	1.9E-01
BRCA	CELF1_ES_3:4_1_6.2	CELF1	ES	3:04	1	6.2	4.1E-30	0.128	0.098	9.2E-01	1.5E-01	8.0E-01
BRCA	CEP164_ES_14_13_15	CEP164	ES	14	13	15	9.5E-28	0.137	0.096	8.7E-01	6.9E-01	1.6E-01
BRCA	CEP63_ES_12_11_13	CEP63	ES	12	11	13	7.0E-25	0.101	-0.168	7.0E-01	6.5E-01	8.5E-01
BRCA	CHEK2_ME_6|7.1:7.2_5_9	CHEK2	ME	6|7.1:7.2	5	9	1.6E-46	0.180	0.551	7.0E-01	3.0E-01	2.6E-01
BRCA	CHTF8_ES_4.1_3_4.3	CHTF8	ES	4.1	3	4.3	1.1E-34	0.138	0.176	6.2E-01	3.0E-01	6.6E-01
BRCA	CIRBP_RI_9.9:9.10_9.8_9.11	CIRBP	RI	9.9:9.10	9.8	9.11	4.6E-41	0.161	-0.370	7.8E-01	6.4E-01	4.1E-01
BRCA	CLCC1_ES_2_1_3.1	CLCC1	ES	2	1	3.1	5.5E-26	0.108	-0.060	9.9E-01	1.8E-01	2.7E-01
BRCA	CLEC2D_ES_7.3_7.1_8	CLEC2D	ES	7.3	7.1	8	3.8E-21	0.108	0.149	5.3E-01	5.3E-02	2.8E-01
BRCA	CLEC2D_RI_7.2_7.1_7.3	CLEC2D	RI	7.2	7.1	7.3	4.7E-24	0.115	-0.211	4.7E-01	2.4E-01	2.2E-01
BRCA	CLN3_RI_3.3_3.2_3.4	CLN3	RI	3.3	3.2	3.4	4.2E-25	0.101	0.066	4.3E-02	2.4E-01	3.1E-01
BRCA	CLSTN1_ES_11_10_12	CLSTN1	ES	11	10	12	9.5E-33	0.131	-0.135	7.1E-02	2.5E-01	7.7E-01
BRCA	CLSTN1_ES_3_2_4	CLSTN1	ES	3	2	4	1.5E-28	0.118	-0.232	5.2E-01	8.9E-01	4.9E-01
BRCA	CNDP2_AA_2.2_1_2.3	CNDP2	AA	2.2	1	2.3	1.7E-41	0.163	0.030	4.3E-01	7.9E-01	8.4E-01
BRCA	COASY_AA_1.3:1.4_1.1_1.5	COASY	AA	1.3:1.4	1.1	1.5	9.4E-38	0.161	-0.136	1.6E-01	5.5E-01	6.3E-01
BRCA	COASY_AD_1.2:1.3_1.1_1.5	COASY	AD	1.2:1.3	1.1	1.5	3.9E-58	0.231	-0.221	8.3E-01	4.2E-01	2.9E-01

cancer type	id	Gene Symbol	splice_type	Exon	From.Exon	To.Exon	anova.p	adj.r2	r	p.50	p.25	p.10
BRCA	COASY_RI_1.2:1.3:1.4_1.1_1.5	COASY	RI	1.2:1.3:1.4	1.1	1.5	6.1E-38	0.149	-0.369	7.7E-02	1.6E-01	8.8E-01
BRCA	COL16A1_ES_44_43_45.1	COL16A1	ES	44	43	45.1	4.0E-44	0.173	-0.034	4.0E-01	9.9E-02	2.6E-01
BRCA	COL4A3BP_ES_12_11_13	COL4A3BP	ES	12	11	13	3.1E-108	0.391	-0.460	3.5E-01	3.3E-01	6.0E-01
BRCA	COL4A5_ES_42:43_41_44	COL4A5	ES	42:43:00	41	44	1.9E-53	0.248	0.156	7.8E-01	2.9E-01	4.8E-01
BRCA	COMMD5_AD_2.2_2.1_3	COMMD5	AD	2.2	2.1	3	3.5E-38	0.150	0.179	9.3E-01	4.2E-01	1.3E-02
BRCA	COPB1_AD_1.2_1.1_2	COPB1	AD	1.2	1.1	2	1.1E-29	0.123	-0.295	2.6E-01	3.7E-01	9.6E-01
BRCA	COPE_ES_4:5_3_6	COPE	ES	4:05	3	6	1.1E-39	0.161	-0.062	8.6E-03	3.7E-01	8.9E-01
BRCA	COPS7B_ES_4.1:4.2:4.3:4.4:5_3_6	COPS7B	ES	4.1:4.2:4.3:4.4:5	3	6	6.6E-32	0.135	-0.126	5.4E-01	3.6E-01	6.2E-01
BRCA	COX11_RI_3.2_3.1_3.3	COX11	RI	3.2	3.1	3.3	2.4E-32	0.129	0.133	4.8E-01	5.9E-01	9.9E-01
BRCA	CPSF7_AD_7.2_7.1_8	CPSF7	AD	7.2	7.1	8	1.3E-42	0.169	0.118	2.2E-01	1.5E-01	6.0E-01
BRCA	CPSF7_ES_4:6.1:6.2:6.3_3_7.1	CPSF7	ES	4:6.1:6.2:6.3	3	7.1	2.9E-45	0.208	-0.160	5.4E-01	1.0E+00	9.4E-01
BRCA	CPSF7_ES_5.1:5.2:6.1:6.2:6.3_3_7.1	CPSF7	ES	5.1:5.2:6.1:6.2:6.3	3	7.1	9.1E-43	0.207	-0.142	4.7E-01	8.3E-01	4.9E-01
BRCA	CRB3_RI_5.2_5.1_5.3	CRB3	RI	5.2	5.1	5.3	2.3E-69	0.257	0.018	7.2E-01	6.3E-01	3.0E-01
BRCA	CREM_ES_9.2:10.1_4_15	CREM	ES	9.2:10.1	4	15	8.9E-27	0.108	0.036	4.7E-01	3.8E-01	7.0E-01
BRCA	CSPP1_ES_8_6_9	CSPP1	ES	8	6	9	2.0E-27	0.135	0.110	6.3E-01	6.9E-01	3.3E-01
BRCA	CTNND1_ES_21_20_22.1	CTNND1	ES	21	20	22.1	1.0E-105	0.363	0.175	6.8E-01	4.2E-01	9.2E-01
BRCA	CTNND1_ES_21_20_22.2	CTNND1	ES	21	20	22.2	6.7E-29	0.143	0.030	9.6E-01	6.9E-01	7.0E-01
BRCA	CTNND1_ES_21:22.1_20_22.2	CTNND1	ES	21:22.1	20	22.2	1.6E-162	0.503	0.081	9.7E-01	5.5E-01	9.4E-01
BRCA	CUL7_AD_3.2_3.1_4	CUL7	AD	3.2	3.1	4	1.1E-24	0.103	0.121	1.5E-01	1.1E-01	1.3E-02
BRCA	CXorf40A_AD_1.7_1.6_3.1	CXorf40A	AD	1.7	1.6	3.1	1.3E-30	0.123	0.196	8.0E-01	7.4E-01	7.6E-01
BRCA	CXorf40B_AD_2.3_2.2_3	CXorf40B	AD	2.3	2.2	3	2.1E-26	0.106	0.062	8.5E-01	4.6E-01	3.9E-01
BRCA	CYB561A3_AA_6.6_6.4_6.7	CYB561A3	AA	6.6	6.4	6.7	8.9E-26	0.104	0.029	3.0E-02	9.7E-02	4.4E-01
BRCA	DBNDD2_AD_1.2:1.3:1.4_1.1_3.3	DBNDD2	AD	1.2:1.3:1.4	1.1	3.3	4.7E-83	0.298	-0.366	4.2E-01	2.6E-01	2.0E-02
BRCA	DBNDD2_AD_1.3:1.4_1.2_3.3	DBNDD2	AD	1.3:1.4	1.2	3.3	7.8E-33	0.131	-0.249	3.6E-01	9.8E-01	6.4E-01
BRCA	DCAF10_ES_5_4_6	DCAF10	ES	5	4	6	4.3E-61	0.229	0.186	9.8E-01	7.3E-01	8.1E-01
BRCA	DCN_ES_2_1_3	DCN	ES	2	1	3	2.3E-29	0.118	0.045	6.3E-01	4.0E-01	5.6E-01
BRCA	DCTN2_ES_8_2_10	DCTN2	ES	8	2	10	1.6E-81	0.294	-0.033	9.2E-01	8.5E-01	9.7E-01
BRCA	DEDD2_ES_3_2_4.1	DEDD2	ES	3	2	4.1	1.6E-33	0.141	0.165	5.9E-01	2.5E-01	1.1E-01
BRCA	DEF8_ES_2.1_1_4	DEF8	ES	2.1	1	4	1.0E-86	0.318	-0.175	1.1E-01	9.7E-01	3.5E-01
BRCA	DENND1B_ES_9_8_10	DENND1B	ES	9	8	10	3.7E-106	0.432	0.245	8.7E-01	7.5E-01	2.4E-01
BRCA	DGUOK_ES_3_1_4	DGUOK	ES	3	1	4	2.0E-55	0.211	-0.241	3.5E-01	1.7E-01	4.8E-01
BRCA	DGUOK_ES_3:4:5_1_7	DGUOK	ES	3:04:05	1	7	6.0E-28	0.113	-0.254	4.4E-01	6.4E-01	3.0E-01
BRCA	DGUOK_ES_3:4:5:6_1_7	DGUOK	ES	3:4:5:6	1	7	9.0E-35	0.139	-0.285	1.8E-01	1.3E-02	5.9E-02
BRCA	DHRS12_ES_3_2_4	DHRS12	ES	3	2	4	3.2E-40	0.158	0.231	4.3E-01	9.8E-01	8.4E-01
BRCA	DIAPH1_ES_2_1_3	DIAPH1	ES	2	1	3	6.5E-58	0.252	-0.025	8.9E-01	8.1E-01	5.8E-01
BRCA	DMXL1_ES_34_33_35	DMXL1	ES	34	33	35	6.9E-31	0.134	0.251	3.3E-01	6.0E-01	9.8E-01
BRCA	DNAJC12_ES_2_1_3	DNAJC12	ES	2	1	3	3.6E-118	0.398	-0.766	3.0E-01	7.4E-01	6.9E-01
BRCA	DNAJC4_RI_7.2_7.1_7.3	DNAJC4	RI	7.2	7.1	7.3	9.5E-27	0.108	-0.154	4.5E-02	1.5E-01	1.6E-01
BRCA	DNASE1_AA_4.1:4.2_3.2_4.3	DNASE1	AA	4.1:4.2	3.2	4.3	2.8E-31	0.125	-0.140	5.6E-01	1.4E-01	1.6E-01
BRCA	DOCK7_ES_24_23_25	DOCK7	ES	24	23	25	3.3E-41	0.182	-0.228	7.5E-01	9.5E-01	8.2E-01

cancer type	id	Gene Symbol	splice_type	Exon	From.Exon	To.Exon	anova.p	adj.r2	r	p.50	p.25	p.10
BRCA	DOCK9_ES_48_47_49	DOCK9	ES	48	47	49	7.9E-29	0.120	-0.016	1.8E-01	4.7E-01	5.9E-01
BRCA	DONSON_ES_5:6_4.2_7	DONSON	ES	5:06	4.2	7	1.0E-39	0.175	0.270	4.8E-01	2.9E-01	6.7E-01
BRCA	DPY19L4_ES_3_2_4	DPY19L4	ES	3	2	4	5.1E-28	0.117	-0.005	4.2E-01	5.9E-01	6.3E-01
BRCA	DTD2_AD_1.2_1.1_2	DTD2	AD	1.2	1.1	2	1.6E-27	0.111	0.116	9.2E-01	6.3E-02	5.8E-03
BRCA	DUSP22_RI_7.4_7.3_7.5	DUSP22	RI	7.4	7.3	7.5	1.0E-46	0.181	-0.253	7.7E-01	3.6E-01	7.5E-01
BRCA	DVL1_AD_11.2_11.1_12	DVL1	AD	11.2	11.1	12	1.7E-69	0.257	-0.003	5.3E-01	2.5E-01	6.6E-01
BRCA	DYNC1I2_ES_7.3:8_4_9	DYNC1I2	ES	7.3:8	4	9	7.3E-85	0.330	0.131	6.9E-01	3.8E-01	4.9E-01
BRCA	DYNC1I2_ES_8_7.3_9	DYNC1I2	ES	8	7.3	9	1.2E-49	0.191	0.252	3.7E-02	9.5E-01	5.2E-01
BRCA	DYNC2LI1_AA_8.1_7_8.2	DYNC2LI1	AA	8.1	7	8.2	1.4E-28	0.115	-0.463	2.9E-02	8.1E-01	7.0E-01
BRCA	DYNLL1_ES_2:3_1_4.4	DYNLL1	ES	2:03	1	4.4	1.1E-43	0.173	-0.160	6.8E-01	3.2E-01	9.3E-01
BRCA	DYRK1B_AA_10.1:10.2_9_10.3	DYRK1B	AA	10.1:10.2	9	10.3	4.0E-72	0.268	-0.159	1.6E-01	6.4E-01	6.2E-01
BRCA	DYSF_ES_19_18_20	DYSF	ES	19	18	20	2.6E-34	0.176	0.286	7.9E-01	4.7E-01	6.1E-01
BRCA	ECHDC1_ES_7_6.3_10.2	ECHDC1	ES	7	6.3	10.2	1.5E-50	0.194	-0.134	9.0E-01	6.3E-01	5.7E-01
BRCA	ECHDC2_ES_3_2.1_5.1	ECHDC2	ES	3	2.1	5.1	3.4E-20	0.101	-0.012	3.7E-01	6.6E-01	4.5E-01
BRCA	ECHDC2_ES_4_2.1_5.1	ECHDC2	ES	4	2.1	5.1	2.2E-32	0.140	0.064	2.3E-01	1.2E-01	1.6E-01
BRCA	ECHDC2_ES_5.1_2.1_6.2	ECHDC2	ES	5.1	2.1	6.2	4.2E-36	0.148	-0.209	3.9E-01	3.7E-01	5.2E-01
BRCA	ECHDC2_ES_5.1:6.1_2.1_6.2	ECHDC2	ES	5.1:6.1	2.1	6.2	2.1E-35	0.141	-0.262	3.8E-01	2.4E-01	8.0E-02
BRCA	ECHDC2_ES_9.1_8.1_10	ECHDC2	ES	9.1	8.1	10	2.2E-78	0.286	0.079	7.3E-01	3.7E-01	2.9E-01
BRCA	EFCAB14_ES_7_6_8	EFCAB14	ES	7	6	8	3.9E-67	0.250	-0.214	5.2E-01	7.6E-01	7.3E-01
BRCA	EFCAB4A_ES_8_7_9	EFCAB4A	ES	8	7	9	4.0E-53	0.203	0.052	3.6E-01	3.0E-01	9.9E-01
BRCA	EHBP1_ES_18_17_19	EHBP1	ES	18	17	19	4.4E-28	0.114	0.003	2.1E-01	1.4E-02	1.4E-01
BRCA	EIF3E_ES_3:4.1_1_4.2	EIF3E	ES	03:04.1	1	4.2	5.3E-39	0.196	-0.181	9.0E-01	8.5E-01	4.7E-01
BRCA	EIF3K_AD_1.2_1.1_2	EIF3K	AD	1.2	1.1	2	2.1E-101	0.369	-0.126	5.0E-01	4.2E-01	2.3E-01
BRCA	EIF4G1_ES_2.3:2.4:3.2_2.2_5	EIF4G1	ES	2.3:2.4:3.2	2.2	5	2.1E-21	0.111	-0.365	3.2E-01	2.6E-01	5.0E-02
BRCA	ELF3_RI_1.3_1.2_1.4	ELF3	RI	1.3	1.2	1.4	3.8E-32	0.140	0.081	5.8E-02	1.2E-01	4.8E-01
BRCA	ELK1_ES_2_1_3	ELK1	ES	2	1	3	1.0E-69	0.261	0.182	7.7E-01	9.5E-01	8.4E-01
BRCA	ELP2_ES_5:6_4.2_7	ELP2	ES	5:06	4.2	7	4.0E-35	0.144	0.166	8.7E-01	2.0E-01	9.3E-01
BRCA	EMC10_ES_7_6_8.1	EMC10	ES	7	6	8.1	2.5E-57	0.217	0.194	7.0E-01	3.4E-01	9.5E-01
BRCA	ENDOV_ES_5_2.4_6.2	ENDOV	ES	5	2.4	6.2	6.4E-33	0.134	0.034	9.7E-01	9.2E-01	9.3E-01
BRCA	ENDOV_RI_12.2_12.1_12.3	ENDOV	RI	12.2	12.1	12.3	2.9E-25	0.103	-0.081	2.1E-01	3.4E-01	7.0E-02
BRCA	ENPP5_ES_2_1_3	ENPP5	ES	2	1	3	7.1E-25	0.116	-0.154	8.1E-01	2.4E-01	7.9E-01
BRCA	EPB41_ES_16_15_19.1	EPB41	ES	16	15	19.1	8.7E-49	0.225	0.044	4.0E-01	1.8E-01	4.5E-01
BRCA	EPB41_ES_18_16_19.1	EPB41	ES	18	16	19.1	2.1E-77	0.341	0.027	4.2E-01	1.7E-01	6.6E-02
BRCA	EPB41L1_ES_20_19_21	EPB41L1	ES	20	19	21	1.9E-149	0.470	-0.342	7.5E-01	8.2E-01	9.9E-01
BRCA	EPB41L3_ES_25_24_26	EPB41L3	ES	25	24	26	1.4E-35	0.141	-0.467	6.2E-01	7.7E-01	7.2E-01
BRCA	EPS8L1_RI_15.2_15.1_15.3	EPS8L1	RI	15.2	15.1	15.3	2.7E-27	0.112	-0.299	5.7E-01	3.9E-01	1.0E+00
BRCA	ERBB2IP_ES_22_21_24.1	ERBB2IP	ES	22	21	24.1	2.5E-74	0.281	0.236	1.0E+00	6.6E-01	2.0E-01
BRCA	ERBB2IP_ES_22:24.1:24.2:24.3_21_25	ERBB2IP	ES	22:24.1:24.2:24.3	21	25	1.1E-100	0.364	0.339	2.4E-01	3.2E-01	7.4E-01
BRCA	ERBB2IP_ME_22|23_21_24.1	ERBB2IP	ME	22|23	21	24.1	6.8E-125	0.438	0.342	9.8E-01	3.3E-01	5.8E-01
BRCA	ERC1_ES_7_6_8	ERC1	ES	7	6	8	2.2E-27	0.122	-0.049	4.7E-01	1.0E-01	7.8E-02

cancer type	id	Gene Symbol	splice_type	Exon	From.Exon	To.Exon	anova.p	adj.r2	r	p.50	p.25	p.10
BRCA	ERGIC1_RI_6.2_6.1_6.3	ERGIC1	RI	6.2	6.1	6.3	4.1E-25	0.107	-0.265	2.3E-02	9.9E-02	3.4E-01
BRCA	ESRP1_AD_12.2_12.1_13	ESRP1	AD	12.2	12.1	13	3.7E-36	0.144	-0.291	2.2E-01	2.0E-01	1.2E-01
BRCA	ETV7_ES_3_2_4	ETV7	ES	3	2	4	2.5E-31	0.135	-0.149	2.7E-01	1.1E-01	3.2E-02
BRCA	EXOC1_ES_11_10_12	EXOC1	ES	11	10	12	1.0E-61	0.234	0.267	1.3E-01	5.6E-02	4.0E-01
BRCA	EXOC7_ES_7:8.1:8.2_6_9	EXOC7	ES	7:8.1:8.2	6	9	2.0E-27	0.111	0.333	3.8E-01	3.5E-01	1.2E-01
BRCA	EXOC7_ES_7:8.2_6_9	EXOC7	ES	07:08.2	6	9	3.8E-36	0.144	0.266	1.3E-01	1.4E-01	5.2E-01
BRCA	EXOC7_ES_8.2_6_9	EXOC7	ES	8.2	6	9	3.0E-84	0.302	0.281	4.6E-01	8.9E-01	5.4E-01
BRCA	EZH2_ES_6:7_5.2_8	EZH2	ES	6:07	5.2	8	1.0E-20	0.104	-0.305	8.1E-01	6.2E-02	9.4E-02
BRCA	FAM114A1_ES_2_1_3	FAM114A1	ES	2	1	3	3.4E-30	0.125	-0.109	2.0E-01	5.9E-01	7.5E-02
BRCA	FAM126A_ES_11_10_12	FAM126A	ES	11	10	12	3.5E-67	0.258	-0.194	8.4E-01	6.5E-01	5.2E-01
BRCA	FAM131A_ES_4_2_6.2	FAM131A	ES	4	2	6.2	8.2E-35	0.143	-0.110	5.7E-01	3.7E-01	8.5E-01
BRCA	FAM136A_ES_1.3_1.1_2	FAM136A	ES	1.3	1.1	2	7.8E-42	0.164	-0.326	2.7E-01	9.5E-01	2.2E-01
BRCA	FAM13B_ES_19_18_20	FAM13B	ES	19	18	20	9.1E-26	0.107	0.148	3.0E-01	8.2E-02	6.1E-01
BRCA	FAM160A2_AD_8.2_8.1_9.1	FAM160A2	AD	8.2	8.1	9.1	7.0E-58	0.220	-0.203	5.9E-01	8.7E-01	4.3E-01
BRCA	FAM189B_ES_2:3:4_1_5	FAM189B	ES	2:03:04	1	5	8.1E-75	0.273	-0.136	6.8E-01	9.9E-01	9.2E-01
BRCA	FAM195A_ES_3_2_4	FAM195A	ES	3	2	4	1.0E-53	0.205	0.256	6.7E-01	8.5E-01	6.8E-01
BRCA	FAM195B_RI_4.4_4.3_4.5	FAM195B	RI	4.4	4.3	4.5	5.6E-41	0.160	0.040	8.3E-01	7.6E-01	9.0E-01
BRCA	FAM211B_ES_2_1_3	FAM211B	ES	2	1	3	1.6E-34	0.139	0.090	4.0E-01	3.5E-01	1.9E-01
BRCA	FAM211B_ES_4_3_5	FAM211B	ES	4	3	5	3.2E-67	0.249	0.380	1.2E-01	5.8E-02	1.7E-01
BRCA	FAM3A_ES_3.2_2_4	FAM3A	ES	3.2	2	4	2.5E-26	0.106	-0.301	7.4E-01	5.5E-01	9.9E-01
BRCA	FAM3A_ES_7.1:7.2_6_8	FAM3A	ES	7.1:7.2	6	8	2.2E-40	0.160	-0.066	5.1E-02	8.3E-01	2.6E-01
BRCA	FAM3A_RI_1.3_1.2_1.4	FAM3A	RI	1.3	1.2	1.4	4.3E-28	0.113	-0.073	5.2E-01	3.9E-01	6.3E-01
BRCA	FAM60A_ES_3_1_4	FAM60A	ES	3	1	4	1.0E-25	0.109	0.135	3.0E-02	6.9E-02	8.0E-02
BRCA	FBLN2_ES_11_10_12	FBLN2	ES	11	10	12	1.1E-46	0.183	0.077	1.7E-01	7.2E-01	9.5E-01
BRCA	FBXO3_RI_12.2:12.3_12.1_12.4	FBXO3	RI	12.2:12.3	12.1	12.4	3.9E-36	0.143	-0.508	7.8E-01	4.9E-01	6.2E-02
BRCA	FBXO44_ES_5.2:6_5.1_7	FBXO44	ES	5.2:6	5.1	7	3.1E-25	0.102	0.006	4.1E-01	6.4E-01	9.6E-01
BRCA	FDFT1_ES_3.2:5.2:6.3_2.2_6.4	FDFT1	ES	3.2:5.2:6.3	2.2	6.4	4.0E-24	0.116	-0.261	1.3E-02	1.6E-04	2.1E-02
BRCA	FDPS_AA_3.1_1.1_3.2	FDPS	AA	3.1	1.1	3.2	3.4E-66	0.246	0.263	5.7E-01	6.6E-01	1.6E-01
BRCA	FDPS_AA_3.1_1.2_3.2	FDPS	AA	3.1	1.2	3.2	8.7E-40	0.171	0.177	3.7E-01	6.8E-01	4.2E-01
BRCA	FDPS_ES_1.2:3.1_1.1_3.2	FDPS	ES	1.2:3.1	1.1	3.2	8.5E-67	0.269	0.246	1.6E-01	4.5E-01	7.9E-01
BRCA	FIP1L1_ES_11_10_12	FIP1L1	ES	11	10	12	6.9E-92	0.324	-0.217	2.9E-02	8.1E-01	9.5E-01
BRCA	FIP1L1_ES_14_13.1_15	FIP1L1	ES	14	13.1	15	2.1E-71	0.263	0.006	4.1E-01	5.3E-01	4.3E-01
BRCA	FLNB_ES_27_26_28	FLNB	ES	27	26	28	4.1E-32	0.130	0.174	7.4E-01	3.6E-01	3.2E-01
BRCA	FLNB_ES_32.1_31_33	FLNB	ES	32.1	31	33	3.1E-25	0.105	0.364	3.4E-01	9.7E-01	8.7E-01
BRCA	FLOT2_ES_3_2_6	FLOT2	ES	3	2	6	2.8E-20	0.105	0.024	8.5E-01	4.8E-01	2.4E-01
BRCA	FNIP1_ES_7_6_8	FNIP1	ES	7	6	8	1.2E-24	0.116	0.297	6.0E-01	5.5E-01	9.5E-01
BRCA	FXR1_ES_19_18_20	FXR1	ES	19	18	20	5.4E-46	0.178	0.100	4.2E-01	2.2E-01	8.9E-01
BRCA	FXYD5_RI_1.3:1.4:1.5_1.2_1.6	FXYD5	RI	1.3:1.4:1.5	1.2	1.6	2.5E-59	0.226	0.267	2.6E-01	6.8E-01	6.0E-01
BRCA	GAA_ES_2.2_1_3	GAA	ES	2.2	1	3	7.8E-38	0.164	0.222	9.9E-01	9.3E-01	6.6E-01
BRCA	GABRP_ES_10_9_12	GABRP	ES	10	9	12	3.3E-26	0.134	-0.270	4.5E-01	4.7E-01	6.3E-01

cancer type	id	Gene Symbol	splice_type	Exon	From.Exon	To.Exon	anova.p	adj.r2	r	p.50	p.25	p.10
BRCA	GIGYF2_ES_2_1_3.2	GIGYF2	ES	2	1	3.2	1.4E-81	0.307	-0.299	6.4E-01	7.1E-01	9.5E-01
BRCA	GIGYF2_ES_3.2:4_1_5	GIGYF2	ES	3.2:4	1	5	6.3E-24	0.116	0.120	4.1E-01	1.1E-02	1.0E-01
BRCA	GIT2_ES_19_18.2_20	GIT2	ES	19	18.2	20	9.4E-54	0.208	0.044	4.7E-01	3.6E-01	2.6E-01
BRCA	GK_ES_23_22_24	GK	ES	23	22	24	1.9E-55	0.211	-0.147	9.3E-01	5.9E-01	9.9E-01
BRCA	GLIPR1_RI_5.2_5.1_5.3	GLIPR1	RI	5.2	5.1	5.3	4.5E-42	0.164	-0.419	7.7E-01	7.6E-01	8.9E-01
BRCA	GMPR2_RI_2.2_2.1_2.3	GMPR2	RI	2.2	2.1	2.3	2.2E-47	0.184	-0.306	6.4E-01	8.1E-01	9.9E-01
BRCA	GOLGA2_ES_7_6_8	GOLGA2	ES	7	6	8	2.5E-27	0.112	0.094	7.5E-01	5.4E-01	1.9E-01
BRCA	GOLGA4_ES_24_23_25	GOLGA4	ES	24	23	25	1.5E-69	0.257	0.251	1.8E-01	4.6E-01	3.4E-01
BRCA	GOLIM4_ES_7_6_8	GOLIM4	ES	7	6	8	2.6E-54	0.212	0.074	4.2E-01	4.8E-01	9.8E-01
BRCA	GPATCH8_ES_6_5_7	GPATCH8	ES	6	5	7	1.7E-45	0.219	0.310	8.0E-01	9.4E-01	8.1E-01
BRCA	GPM6B_AA_11.1_10_11.2	GPM6B	AA	11.1	10	11.2	7.2E-34	0.134	-0.421	5.7E-02	4.2E-02	3.8E-01
BRCA	GPR137_ES_6_5_8	GPR137	ES	6	5	8	8.2E-60	0.251	0.195	6.4E-01	2.7E-01	1.3E-01
BRCA	GPR157_ES_3_2_4	GPR157	ES	3	2	4	5.8E-35	0.147	-0.077	9.4E-01	2.7E-01	4.9E-01
BRCA	GRHL2_AD_1.2_1.1_2	GRHL2	AD	1.2	1.1	2	2.4E-37	0.161	0.122	2.2E-01	2.2E-01	9.5E-01
BRCA	GSE1_ES_3_2_4	GSE1	ES	3	2	4	1.6E-27	0.121	0.145	1.5E-01	1.1E-01	3.7E-01
BRCA	GSN_ES_10:15.1:15.2_9_16	GSN	ES	10:15.1:15.2	9	16	6.1E-33	0.133	-0.062	4.6E-01	5.4E-01	4.7E-01
BRCA	GTF3C1_AD_34.2_34.1_35	GTF3C1	AD	34.2	34.1	35	9.2E-58	0.219	0.084	6.1E-01	9.5E-01	8.0E-01
BRCA	GUCY1A3_ES_4_2.2_5.1	GUCY1A3	ES	4	2.2	5.1	8.8E-22	0.118	-0.026	7.5E-01	1.2E-01	3.7E-01
BRCA	GUK1_ES_3_2_5.3	GUK1	ES	3	2	5.3	4.2E-28	0.113	-0.395	6.8E-01	9.1E-01	8.6E-01
BRCA	GUSB_ES_6_5.1_7	GUSB	ES	6	5.1	7	5.3E-46	0.178	0.128	1.3E-01	7.4E-01	2.3E-01
BRCA	H2AFY_ME_7|8_6.3_9	H2AFY	ME	7|8	6.3	9	2.1E-32	0.129	0.260	2.8E-01	5.2E-01	7.4E-01
BRCA	HAX1_ES_2.1:2.2:2.3:3.1_1_3.2	HAX1	ES	2.1:2.2:2.3:3.1	1	3.2	1.8E-30	0.133	-0.136	8.1E-01	9.6E-01	3.3E-01
BRCA	HCFC1_AA_19.1_18_19.2	HCFC1	AA	19.1	18	19.2	7.6E-30	0.121	-0.028	6.9E-01	8.9E-01	8.6E-01
BRCA	HDAC7_ES_11_10.2_12	HDAC7	ES	11	10.2	12	1.8E-53	0.205	0.349	3.9E-01	2.3E-01	5.2E-02
BRCA	HDHD2_ES_3_1_4.1	HDHD2	ES	3	1	4.1	1.2E-30	0.128	-0.241	4.9E-01	7.1E-01	5.6E-01
BRCA	HM13_ES_11:12.1:12.2_10_13	HM13	ES	11:12.1:12.2	10	13	8.0E-35	0.139	-0.195	7.3E-01	5.8E-01	9.8E-01
BRCA	HMBS_AD_12.2:12.3_12.1_13	HMBS	AD	12.2:12.3	12.1	13	1.3E-80	0.299	-0.170	7.7E-01	8.2E-01	2.9E-01
BRCA	HMGA1_AD_1.2:1.3_1.1_3.1	HMGA1	AD	1.2:1.3	1.1	3.1	7.9E-41	0.170	-0.294	8.3E-01	7.8E-01	8.3E-01
BRCA	HMGA1_ES_1.3_1.1_3.1	HMGA1	ES	1.3	1.1	3.1	3.2E-56	0.219	-0.365	4.3E-01	2.5E-01	7.9E-01
BRCA	HMGN1_ES_7_6.2_9	HMGN1	ES	7	6.2	9	5.3E-41	0.164	-0.154	2.3E-02	4.2E-02	2.3E-02
BRCA	HMHA1_RI_18.2_18.1_18.3	HMHA1	RI	18.2	18.1	18.3	2.1E-23	0.107	-0.156	3.8E-01	9.3E-02	6.3E-01
BRCA	HNRNPA2B1_AA_12.2:12.3_11_12.4	HNRNPA2B1	AA	12.2:12.3	11	12.4	3.0E-37	0.147	-0.145	2.3E-01	5.5E-01	9.0E-01
BRCA	HNRNPA2B1_ES_12.2_11_12.4	HNRNPA2B1	ES	12.2	11	12.4	8.3E-29	0.116	-0.141	3.5E-01	6.1E-01	7.2E-01
BRCA	HNRNPH3_AD_4.2_4.1_5	HNRNPH3	AD	4.2	4.1	5	5.1E-45	0.175	-0.043	4.0E-01	8.3E-01	5.4E-02
BRCA	HPS1_ES_9_8_10.1	HPS1	ES	9	8	10.1	1.1E-37	0.150	0.109	6.7E-01	1.3E-01	6.9E-02
BRCA	HPS4_ES_10.3_10.1_10.5	HPS4	ES	10.3	10.1	10.5	8.0E-33	0.142	-0.162	2.1E-02	1.4E-01	7.6E-02
BRCA	HPS4_RI_10.2_10.1_10.3	HPS4	RI	10.2	10.1	10.3	7.4E-26	0.119	0.096	3.1E-02	6.4E-02	2.2E-01
BRCA	HR_ES_17_16_18	HR	ES	17	16	18	5.4E-41	0.192	-0.236	1.7E-02	7.4E-03	1.1E-01
BRCA	HTATIP2_AD_1.2_1.1_1.5	HTATIP2	AD	1.2	1.1	1.5	2.6E-25	0.106	0.153	2.4E-01	1.9E-01	5.4E-01
BRCA	IFI44_ES_8_6_9	IFI44	ES	8	6	9	2.9E-39	0.188	-0.303	4.1E-01	5.5E-02	7.5E-01

cancer type	id	Gene Symbol	splice_type	Exon	From.Exon	To.Exon	anova.p	adj.r2	r	p.50	p.25	p.10
BRCA	IFT122_ES_5:7_3_8.2	IFT122	ES	5:07	3	8.2	4.1E-24	0.100	0.089	4.4E-02	7.0E-01	7.6E-01
BRCA	IFT172_ES_46_45_47	IFT172	ES	46	45	47	2.8E-109	0.415	0.208	9.6E-01	7.1E-01	9.8E-02
BRCA	IL17RC_ES_18_17_19	IL17RC	ES	18	17	19	8.7E-30	0.119	0.010	4.5E-01	7.4E-01	7.5E-01
BRCA	IL17RE_ES_4:5_2.2_6	IL17RE	ES	4:05	2.2	6	3.6E-19	0.105	-0.048	2.0E-01	4.0E-02	2.1E-01
BRCA	INCENP_ES_11_10_12	INCENP	ES	11	10	12	2.9E-85	0.317	0.286	6.0E-01	7.0E-01	1.1E-01
BRCA	INO80E_ES_6.1:6.2:6.3_5_11	INO80E	ES	6.1:6.2:6.3	5	11	2.0E-73	0.269	0.057	8.8E-01	3.6E-01	1.8E-01
BRCA	INO80E_ES_6.1:6.2:6.3:10_5_11	INO80E	ES	6.1:6.2:6.3:10	5	11	3.3E-34	0.140	0.131	4.6E-01	9.9E-01	5.0E-01
BRCA	INO80E_ES_6.1:6.2:6.3:7:8:9:10_5_11	INO80E	ES	.1:6.2:6.3:7:8:9:1	5	11	6.2E-28	0.122	0.083	2.6E-01	2.0E-01	1.7E-01
BRCA	INO80E_ES_6.2:6.3_5_11	INO80E	ES	6.2:6.3	5	11	1.7E-56	0.214	0.120	5.4E-01	7.9E-01	6.1E-01
BRCA	INO80E_ES_6.2:6.3:10_5_11	INO80E	ES	6.2:6.3:10	5	11	1.1E-42	0.167	0.164	3.0E-01	8.7E-01	1.7E-01
BRCA	INO80E_ES_6.2:6.3:7:8:9:10_5_11	INO80E	ES	6.2:6.3:7:8:9:10	5	11	5.8E-28	0.120	0.114	5.5E-01	8.9E-01	1.4E-01
BRCA	INPP5K_ES_2_1_4	INPP5K	ES	2	1	4	1.6E-27	0.116	-0.117	9.5E-02	4.2E-01	4.5E-01
BRCA	INSR_ES_11_10_12	INSR	ES	11	10	12	1.4E-28	0.121	-0.177	8.9E-01	7.8E-01	7.2E-01
BRCA	IRAK1_ES_10.1:10.3_9_11.1	IRAK1	ES	10.1:10.3	9	11.1	3.0E-28	0.132	-0.309	3.7E-01	4.5E-01	2.9E-01
BRCA	IRF3_AD_1.2:1.3:1.4_1.1_2	IRF3	AD	1.2:1.3:1.4	1.1	2	3.2E-70	0.259	0.074	1.1E-01	3.6E-01	4.9E-01
BRCA	IRF3_AD_5.2_5.1_6.2	IRF3	AD	5.2	5.1	6.2	2.7E-68	0.253	-0.247	5.0E-01	4.2E-01	9.0E-01
BRCA	IRF3_ES_1.2:1.3:1.4:1.5:2_1.1_3	IRF3	ES	1.2:1.3:1.4:1.5:2	1.1	3	1.1E-84	0.374	-0.118	2.5E-01	2.0E-01	3.3E-01
BRCA	IRF3_ES_1.2:1.3:1.4:2_1.1_3	IRF3	ES	1.2:1.3:1.4:2	1.1	3	9.3E-104	0.357	0.005	2.4E-01	2.4E-01	3.5E-01
BRCA	IRF3_ES_1.4:1.5:2_1.1_3	IRF3	ES	1.4:1.5:2	1.1	3	6.9E-104	0.402	-0.122	3.5E-01	7.2E-01	7.1E-01
BRCA	IRF3_ES_1.5:2_1.1_3	IRF3	ES	1.5:2	1.1	3	7.3E-55	0.253	-0.084	8.6E-01	4.2E-01	2.8E-01
BRCA	ISOC2_ES_3_2_4.2	ISOC2	ES	3	2	4.2	3.1E-116	0.390	-0.022	5.2E-01	6.0E-01	1.9E-01
BRCA	ISOC2_ES_3:4.1_2_4.2	ISOC2	ES	03:04.1	2	4.2	2.1E-72	0.266	-0.041	4.1E-01	4.9E-01	8.4E-02
BRCA	ITGA6_ES_27_26_28	ITGA6	ES	27	26	28	9.5E-109	0.374	0.193	7.1E-02	4.9E-02	5.8E-01
BRCA	ITGB1BP1_AA_8.1:8.2_7_8.3	ITGB1BP1	AA	8.1:8.2	7	8.3	1.3E-30	0.122	-0.177	1.0E+00	8.7E-01	3.0E-01
BRCA	ITGB3BP_ES_9_8_10	ITGB3BP	ES	9	8	10	1.8E-73	0.269	-0.096	4.4E-01	2.7E-01	4.8E-01
BRCA	ITGB4_ES_35_34_36	ITGB4	ES	35	34	36	1.2E-25	0.104	-0.496	8.8E-01	4.2E-01	9.5E-02
BRCA	JAG2_ES_10_9_11	JAG2	ES	10	9	11	7.1E-28	0.114	-0.345	7.5E-01	6.9E-01	1.5E-01
BRCA	JMJD6_ES_5.1:5.2_4_6	JMJD6	ES	5.1:5.2	4	6	4.9E-42	0.164	-0.215	4.3E-01	7.0E-01	5.8E-01
BRCA	JUP_RI_15.2_15.1_15.3	JUP	RI	15.2	15.1	15.3	7.7E-27	0.108	-0.071	9.0E-01	8.0E-01	4.7E-01
BRCA	KANSL3_AA_5.1_3.2_5.2	KANSL3	AA	5.1	3.2	5.2	1.8E-28	0.122	-0.197	5.1E-01	2.6E-01	3.6E-01
BRCA	KARS_ES_2_1_3	KARS	ES	2	1	3	1.4E-33	0.134	0.220	7.3E-01	7.9E-01	7.1E-01
BRCA	KAT5_ES_4_3_5	KAT5	ES	4	3	5	6.8E-47	0.181	0.107	9.4E-01	7.9E-01	4.5E-01
BRCA	KAT6A_ES_2.1:2.2_1_3	KAT6A	ES	2.1:2.2	1	3	2.6E-34	0.168	-0.015	7.7E-01	5.1E-01	8.6E-02
BRCA	KDM1A_ES_3_2_4	KDM1A	ES	3	2	4	2.3E-33	0.134	-0.250	8.4E-01	4.3E-01	4.9E-01
BRCA	KDM6A_ES_16_15_17	KDM6A	ES	16	15	17	1.7E-24	0.106	-0.129	8.1E-01	8.7E-01	3.4E-01
BRCA	KIAA0895L_ES_2.1:2.2_1_3.1	KIAA0895L	ES	2.1:2.2	1	3.1	5.2E-36	0.153	-0.060	9.9E-01	3.9E-02	5.6E-01
BRCA	KIAA1217_ES_12_11_13	KIAA1217	ES	12	11	13	2.9E-101	0.359	0.064	1.9E-02	9.5E-03	3.6E-01
BRCA	KIAA1217_RI_25.2_25.1_25.3	KIAA1217	RI	25.2	25.1	25.3	1.3E-28	0.115	-0.074	8.2E-01	9.2E-01	9.4E-01
BRCA	KIAA1715_ES_4_3_5	KIAA1715	ES	4	3	5	1.3E-34	0.169	-0.106	8.7E-01	7.1E-01	6.9E-01
BRCA	KIF12_ES_5_4.1_6	KIF12	ES	5	4.1	6	3.6E-62	0.277	0.670	5.9E-01	4.3E-01	5.8E-01

cancer type	id	Gene Symbol	splice_type	Exon	From.Exon	To.Exon	anova.p	adj.r2	r	p.50	p.25	p.10
BRCA	KIF21A_ES_24_23_25.2	KIF21A	ES	24	23	25.2	7.2E-27	0.130	-0.296	2.2E-01	5.7E-02	7.9E-01
BRCA	KIF23_ES_21_20_22.1	KIF23	ES	21	20	22.1	2.1E-30	0.123	0.082	5.8E-03	1.6E-02	7.5E-01
BRCA	KIF3A_ES_11_9_12	KIF3A	ES	11	9	12	1.0E-20	0.106	-0.223	5.1E-01	7.2E-01	1.8E-01
BRCA	KLHL24_ES_2_1_3	KLHL24	ES	2	1	3	5.5E-25	0.114	-0.200	5.8E-01	2.9E-01	8.1E-02
BRCA	KRAS_ES_6_5_7	KRAS	ES	6	5	7	2.2E-63	0.241	-0.069	6.6E-01	8.8E-01	6.3E-01
BRCA	KRBOX4_ES_5.2:6_5.1_8.1	KRBOX4	ES	5.2:6	5.1	8.1	4.9E-58	0.249	0.141	3.4E-01	3.7E-02	5.8E-01
BRCA	KRBOX4_ES_6_5.2_8.1	KRBOX4	ES	6	5.2	8.1	4.9E-49	0.236	0.137	1.2E-01	1.9E-01	4.9E-01
BRCA	KREMEN1_AD_7.2_7.1_8	KREMEN1	AD	7.2	7.1	8	3.6E-24	0.117	0.050	2.8E-01	8.2E-01	8.1E-01
BRCA	KTN1_ES_35_34.2_36	KTN1	ES	35	34.2	36	6.6E-27	0.108	0.108	3.9E-01	9.5E-01	9.3E-01
BRCA	LGALS8_ES_11_10.1_12	LGALS8	ES	11	10.1	12	7.6E-28	0.112	-0.090	1.4E-01	3.4E-01	5.0E-01
BRCA	LGALS8_RI_3.2_3.1_3.3	LGALS8	RI	3.2	3.1	3.3	9.8E-32	0.133	-0.423	2.0E-02	1.5E-01	6.6E-02
BRCA	LIMCH1_ES_28_27_29	LIMCH1	ES	28	27	29	6.0E-38	0.156	0.401	2.4E-01	2.6E-01	6.4E-01
BRCA	LMBR1L_ES_4.1:4.2_3_5	LMBR1L	ES	4.1:4.2	3	5	4.1E-45	0.177	0.237	9.8E-02	5.2E-02	4.3E-01
BRCA	LPAR2_AA_5.1_2.2_5.2	LPAR2	AA	5.1	2.2	5.2	3.1E-24	0.105	0.059	7.1E-01	5.6E-01	7.1E-02
BRCA	LPHN2_ES_15_14_16	LPHN2	ES	15	14	16	1.4E-46	0.207	-0.119	8.1E-01	9.4E-01	3.1E-01
BRCA	LPHN2_ES_29_27_31	LPHN2	ES	29	27	31	9.9E-38	0.167	-0.384	7.6E-01	9.2E-02	2.4E-01
BRCA	LRCH3_ES_15_14_16	LRCH3	ES	15	14	16	4.9E-26	0.107	-0.023	1.8E-01	3.9E-01	9.8E-02
BRCA	LRRC14_ES_2_1_3	LRRC14	ES	2	1	3	4.4E-32	0.157	0.007	6.6E-01	9.9E-01	1.9E-01
BRCA	LRRC23_AD_3.2_3.1_4	LRRC23	AD	3.2	3.1	4	3.8E-37	0.152	0.288	6.5E-01	7.3E-01	2.0E-01
BRCA	LRRC23_ES_5_4_6	LRRC23	ES	5	4	6	2.8E-27	0.110	0.156	1.6E-01	7.3E-01	4.9E-01
BRCA	LRRC46_RI_4.2:4.3:4.4_4.1_4.5	LRRC46	RI	4.2:4.3:4.4	4.1	4.5	1.3E-26	0.130	-0.378	2.0E-01	1.9E-02	1.4E-01
BRCA	LRRFIP2_ES_20_19_21	LRRFIP2	ES	20	19	21	3.5E-123	0.409	-0.266	3.7E-01	6.9E-01	1.5E-01
BRCA	LSR_ES_4:5_3_6	LSR	ES	4:05	3	6	1.0E-96	0.338	-0.371	6.3E-02	2.6E-01	8.9E-01
BRCA	LSR_ES_5_3_6	LSR	ES	5	3	6	1.6E-172	0.520	-0.447	1.3E-01	3.0E-01	2.6E-01
BRCA	LTBP3_ES_25_24_26	LTBP3	ES	25	24	26	1.0E-68	0.257	0.395	5.8E-02	1.7E-01	1.5E-01
BRCA	LTBP4_ES_25:26:27_24_28	LTBP4	ES	25:26:27	24	28	5.9E-36	0.146	0.186	7.9E-01	3.5E-01	2.2E-01
BRCA	LTBP4_ES_26:27_24_28	LTBP4	ES	26:27:00	24	28	1.3E-22	0.101	0.196	4.8E-01	1.9E-01	5.9E-01
BRCA	LTBP4_ES_29:30_28_31	LTBP4	ES	29:30:00	28	31	2.0E-42	0.210	0.384	6.5E-01	8.8E-01	6.8E-01
BRCA	LUC7L_ES_1.3_1.1_2.2	LUC7L	ES	1.3	1.1	2.2	1.2E-31	0.129	-0.008	5.1E-01	5.4E-01	2.3E-01
BRCA	LY6K_RI_2.2_2.1_2.3	LY6K	RI	2.2	2.1	2.3	3.1E-24	0.104	-0.546	5.0E-01	8.9E-01	3.7E-01
BRCA	LYRM1_ES_5_3_7	LYRM1	ES	5	3	7	6.8E-36	0.145	-0.089	8.7E-01	6.7E-01	5.0E-01
BRCA	MACF1_ES_103_102_104	MACF1	ES	103	102	104	3.0E-32	0.129	0.273	3.0E-01	5.4E-01	7.1E-01
BRCA	MACF1_ES_107_106_108	MACF1	ES	107	106	108	2.1E-33	0.133	0.245	7.1E-01	7.1E-01	7.4E-01
BRCA	MAGI3_ES_22.1_21_23	MAGI3	ES	22.1	21	23	1.1E-78	0.318	-0.268	1.1E-01	6.7E-02	8.5E-01
BRCA	MANBAL_ES_3_1_4.2	MANBAL	ES	3	1	4.2	1.4E-32	0.130	-0.023	8.0E-01	7.2E-01	6.8E-01
BRCA	MAP2K7_ES_2_1_3	MAP2K7	ES	2	1	3	1.1E-23	0.109	-0.178	6.6E-02	2.6E-02	1.6E-02
BRCA	MAP3K6_ES_3_2_4	MAP3K6	ES	3	2	4	1.2E-47	0.205	0.085	8.1E-01	3.3E-01	5.5E-01
BRCA	MAP3K7_ES_11_10_12	MAP3K7	ES	11	10	12	4.2E-85	0.307	-0.045	1.8E-02	3.0E-02	2.1E-01
BRCA	MAP4K4_ES_18_17_19	MAP4K4	ES	18	17	19	1.1E-22	0.105	0.160	8.8E-01	9.1E-01	8.1E-02
BRCA	MAP7_ES_9_8_10	MAP7	ES	9	8	10	7.3E-83	0.301	-0.002	3.5E-01	4.7E-01	2.3E-01

cancer type	id	Gene Symbol	splice_type	Exon	From.Exon	To.Exon	anova.p	adj.r2	r	p.50	p.25	p.10
BRCA	MARK2_ES_16.1:16.2_15.2_17	MARK2	ES	16.1:16.2	15.2	17	1.6E-146	0.464	0.028	8.3E-01	8.0E-01	9.9E-01
BRCA	MARK3_ES_17:18_16_19	MARK3	ES	17:18	16	19	6.3E-41	0.160	-0.229	6.1E-01	9.2E-01	9.7E-01
BRCA	MAX_ES_2_1.2_3	MAX	ES	2	1.2	3	3.4E-59	0.223	-0.113	6.6E-02	5.9E-01	9.1E-01
BRCA	MBD1_ES_13.1:13.2_12_14	MBD1	ES	13.1:13.2	12	14	7.7E-91	0.321	-0.024	4.5E-01	7.7E-01	4.1E-01
BRCA	MBNL1_ES_8_7_9	MBNL1	ES	8	7	9	5.4E-30	0.125	-0.434	5.9E-01	4.2E-01	2.1E-01
BRCA	MBNL2_ES_10_8_11	MBNL2	ES	10	8	11	1.1E-52	0.221	-0.010	3.7E-03	4.0E-03	1.8E-01
BRCA	MBNL2_ES_9_8_10	MBNL2	ES	9	8	10	7.4E-31	0.148	0.090	2.4E-01	2.7E-01	1.8E-01
BRCA	MDM4_ES_8_5_9	MDM4	ES	8	5	9	9.4E-34	0.145	0.127	3.6E-01	6.9E-01	6.7E-01
BRCA	MED11_AA_3.1_2.1_3.2	MED11	AA	3.1	2.1	3.2	1.2E-96	0.338	-0.104	6.1E-01	3.9E-02	4.8E-01
BRCA	MELK_ES_3_2_4	MELK	ES	3	2	4	8.9E-27	0.120	-0.249	4.8E-01	8.3E-01	8.8E-01
BRCA	METTL15_AA_10.1_9_10.2	METTL15	AA	10.1	9	10.2	1.5E-44	0.181	-0.169	8.9E-01	5.5E-01	8.8E-01
BRCA	MFF_ES_3:4_1_5	MFF	ES	3:04	1	5	1.3E-81	0.297	0.056	3.8E-01	8.0E-01	6.3E-01
BRCA	MFF_ES_3:4:5_1_6	MFF	ES	3:04:05	1	6	1.4E-69	0.257	0.049	6.2E-01	3.1E-01	4.8E-01
BRCA	MFF_ES_8:9_7_10	MFF	ES	8:09	7	10	1.9E-42	0.166	-0.160	4.2E-01	2.2E-01	7.3E-01
BRCA	MFF_ES_9_7_11	MFF	ES	9	7	11	8.7E-38	0.151	-0.026	1.4E-02	6.0E-02	2.2E-01
BRCA	MFSD10_RI_12.2_12.1_12.3	MFSD10	RI	12.2	12.1	12.3	1.4E-45	0.177	0.116	2.8E-01	3.0E-01	3.0E-01
BRCA	MFSD11_AD_2.2:2.3:2.4_2.1_2.6	MFSD11	AD	2.2:2.3:2.4	2.1	2.6	6.0E-24	0.101	-0.023	6.0E-01	6.5E-01	6.4E-01
BRCA	MKNK2_AA_14.1_13.1_14.2	MKNK2	AA	14.1	13.1	14.2	8.4E-26	0.104	0.130	3.9E-01	5.5E-01	7.5E-01
BRCA	MLF1_AD_1.2_1.1_5	MLF1	AD	1.2	1.1	5	1.2E-45	0.178	0.123	4.0E-01	5.7E-01	7.8E-01
BRCA	MLLT4_ES_16_15_17	MLLT4	ES	16	15	17	1.9E-31	0.138	-0.097	8.7E-01	7.2E-01	6.8E-01
BRCA	MMP23B_RI_3.4_3.3_3.5	MMP23B	RI	3.4	3.3	3.5	8.5E-32	0.158	-0.376	2.3E-01	3.8E-01	8.4E-01
BRCA	MND1_ES_5:6_4_7	MND1	ES	5:06	4	7	6.3E-26	0.106	-0.504	7.4E-01	3.4E-01	3.1E-01
BRCA	MRPL52_ES_3:4.1_2_5	MRPL52	ES	03:04.1	2	5	3.6E-29	0.118	-0.134	1.1E-01	7.2E-01	9.2E-01
BRCA	MRPS12_AD_1.2_1.1_1.4	MRPS12	AD	1.2	1.1	1.4	3.1E-129	0.423	0.211	9.3E-01	9.8E-01	9.4E-01
BRCA	MTA1_ES_4_3_5	MTA1	ES	4	3	5	2.2E-105	0.363	-0.008	5.9E-01	8.8E-01	8.3E-01
BRCA	MTMR14_ES_19_18_21	MTMR14	ES	19	18	21	2.0E-41	0.162	-0.039	6.7E-02	1.3E-02	9.4E-02
BRCA	MTMR2_ME_4|5_3_6	MTMR2	ME	4|5	3	6	1.8E-23	0.101	0.366	6.3E-01	8.6E-01	2.5E-01
BRCA	MTSS1L_ES_7_6_8	MTSS1L	ES	7	6	8	9.4E-30	0.126	0.222	2.5E-01	1.0E-01	1.5E-01
BRCA	MUC1_ES_3.2:3.3:3.4:4.1:4.2:4.3:6.1:6.2	MUC1	ES	:3.4:4.1:4.2:4.3:6.	3.1	9	1.2E-28	0.142	-0.143	3.9E-01	4.5E-01	5.5E-01
BRCA	MUC1_ES_6.2_5_7	MUC1	ES	6.2	5	7	1.5E-44	0.197	0.251	1.9E-01	4.4E-01	2.8E-01
BRCA	MUM1_ES_5.1:5.2_4_6	MUM1	ES	5.1:5.2	4	6	4.0E-36	0.144	-0.180	2.1E-01	1.4E-01	1.4E-01
BRCA	MVK_ES_4:5:6_3_7	MVK	ES	4:05:06	3	7	4.1E-25	0.104	-0.110	7.6E-02	7.5E-01	9.2E-01
BRCA	MYB_ES_17_16_19.1	MYB	ES	17	16	19.1	9.7E-44	0.170	-0.670	3.6E-01	1.3E-01	3.7E-01
BRCA	MYB_ES_17:18_16_19.1	MYB	ES	17:18	16	19.1	1.1E-51	0.200	-0.628	NA	NA	NA
BRCA	MYH10_ES_6.1_5_7	MYH10	ES	6.1	5	7	4.2E-46	0.207	0.206	4.6E-01	4.8E-01	9.0E-02
BRCA	MYL12A_ES_1.2:2_1.1_4	MYL12A	ES	1.2:2	1.1	4	1.5E-55	0.212	0.250	1.1E-01	3.9E-01	8.9E-01
BRCA	MYL6_ES_2.1_1.4_3.1	MYL6	ES	2.1	1.4	3.1	5.3E-83	0.298	0.137	9.5E-01	9.1E-01	7.9E-01
BRCA	MYO18A_ES_41_40_42	MYO18A	ES	41	40	42	7.2E-58	0.219	-0.185	7.3E-01	7.9E-01	7.5E-01
BRCA	MYO6_ES_29:30:31_28.1_32	MYO6	ES	29:30:31	28.1	32	1.1E-100	0.400	0.197	6.4E-01	9.7E-01	5.6E-01
BRCA	MYO6_ES_34_33_35	MYO6	ES	34	33	35	1.1E-49	0.191	-0.115	9.0E-01	3.2E-01	7.5E-01

cancer type	id	Gene Symbol	splice_type	Exon	From.Exon	To.Exon	anova.p	adj.r2	r	p.50	p.25	p.10
BRCA	MYOF_ES_17_16_18	MYOF	ES	17	16	18	2.2E-46	0.188	-0.406	8.5E-01	6.2E-01	5.7E-01
BRCA	NAA60_RI_10.4:10.5_10.3_10.6	NAA60	RI	10.4:10.5	10.3	10.6	1.0E-26	0.108	-0.027	2.9E-01	6.1E-01	2.6E-01
BRCA	NAA60_RI_10.5_10.4_10.6	NAA60	RI	10.5	10.4	10.6	3.3E-28	0.113	0.015	6.9E-01	1.9E-01	9.1E-01
BRCA	NABP2_RI_1.2:1.3_1.1_1.4	NABP2	RI	1.2:1.3	1.1	1.4	1.5E-35	0.141	-0.037	4.2E-01	6.3E-01	7.5E-01
BRCA	NAP1L4_ES_16_15_17	NAP1L4	ES	16	15	17	1.6E-36	0.144	-0.191	7.1E-01	9.6E-01	6.4E-01
BRCA	NAPA_ES_2:3:4_1_5	NAPA	ES	2:03:04	1	5	8.3E-31	0.129	0.037	1.0E-01	5.6E-02	1.3E-01
BRCA	NAT1_ES_5.2_3_7	NAT1	ES	5.2	3	7	4.6E-47	0.196	-0.288	7.2E-01	9.0E-01	6.6E-01
BRCA	NAT14_RI_2.3_2.2_2.4	NAT14	RI	2.3	2.2	2.4	1.5E-43	0.170	-0.199	3.1E-01	4.5E-01	6.6E-01
BRCA	NBEAL2_ES_10_9_11	NBEAL2	ES	10	9	11	1.5E-65	0.295	0.244	9.1E-01	7.2E-01	7.0E-01
BRCA	NBN_ES_6_5_7	NBN	ES	6	5	7	3.8E-27	0.116	-0.082	4.2E-01	3.8E-01	5.9E-01
BRCA	NBPF15_ES_2_1_3	NBPF15	ES	2	1	3	7.8E-24	0.103	0.300	4.6E-01	3.1E-01	2.1E-01
BRCA	NCOR2_AD_46.2_46.1_47	NCOR2	AD	46.2	46.1	47	1.0E-49	0.191	0.090	1.1E-01	1.6E-01	1.2E-01
BRCA	NCOR2_ES_46.1:46.2_45_47	NCOR2	ES	46.1:46.2	45	47	2.3E-28	0.131	-0.017	6.8E-01	6.7E-01	8.8E-01
BRCA	NDEL1_ES_11_10_12.1	NDEL1	ES	11	10	12.1	7.4E-45	0.175	0.155	4.3E-01	5.0E-01	7.7E-01
BRCA	NDRG2_ES_4.5_4.1_5.2	NDRG2	ES	4.5	4.1	5.2	1.0E-28	0.117	-0.117	3.0E-01	4.4E-01	5.0E-01
BRCA	NDUFAF6_ES_12_11_14	NDUFAF6	ES	12	11	14	3.0E-78	0.284	0.031	7.3E-01	9.4E-01	1.9E-01
BRCA	NDUFB10_RI_3.2_3.1_3.3	NDUFB10	RI	3.2	3.1	3.3	2.0E-49	0.190	-0.205	1.6E-01	2.2E-01	8.6E-01
BRCA	NEDD4L_ES_18_17_19	NEDD4L	ES	18	17	19	8.9E-157	0.500	0.411	9.4E-01	6.2E-01	2.0E-01
BRCA	NEIL2_AD_1.2:1.3_1.1_2.1	NEIL2	AD	1.2:1.3	1.1	2.1	1.2E-24	0.103	-0.202	8.3E-01	6.8E-01	3.8E-01
BRCA	NF2_ES_16.1_15_17	NF2	ES	16.1	15	17	4.1E-44	0.172	0.223	3.4E-02	1.8E-01	7.2E-01
BRCA	NFE2L1_ES_6_5.2_7	NFE2L1	ES	6	5.2	7	8.3E-51	0.195	0.220	1.4E-01	4.8E-01	1.6E-01
BRCA	NFIB_ES_12.1:12.2:13:14_11_15	NFIB	ES	12.1:12.2:13:14	11	15	1.4E-36	0.160	0.229	2.9E-01	5.2E-02	2.3E-01
BRCA	NFIB_ES_12.1:13_11_15	NFIB	ES	12.1:13	11	15	5.5E-25	0.132	0.037	3.8E-01	8.9E-01	1.0E+00
BRCA	NFIB_ES_12.1:13:14_11_15	NFIB	ES	12.1:13:14	11	15	6.3E-46	0.206	0.311	6.3E-01	5.8E-01	8.0E-01
BRCA	NFIC_ES_10:11_9_12	NFIC	ES	10:11	9	12	6.0E-25	0.101	0.087	8.8E-01	8.4E-01	7.7E-01
BRCA	NFYA_ES_3_2_4	NFYA	ES	3	2	4	7.4E-30	0.123	-0.178	8.1E-02	5.0E-01	3.0E-01
BRCA	NPIPB4_AD_2.2:2.3_2.1_2.5	NPIPB4	AD	2.2:2.3	2.1	2.5	1.3E-23	0.110	-0.066	3.0E-01	3.8E-01	1.0E+00
BRCA	NPIPB4_ES_3.1_2.5_4	NPIPB4	ES	3.1	2.5	4	4.1E-27	0.117	0.043	1.6E-01	1.3E-01	4.6E-01
BRCA	NPIPB4_RI_2.3:2.4_2.2_2.5	NPIPB4	RI	2.3:2.4	2.2	2.5	1.0E-53	0.206	0.273	2.2E-02	1.4E-02	1.7E-04
BRCA	NPIPB4_RI_2.4_2.3_2.5	NPIPB4	RI	2.4	2.3	2.5	1.1E-86	0.312	0.337	2.7E-01	9.8E-02	1.8E-02
BRCA	NPIPB5_AD_2.2:2.3_2.1_2.5	NPIPB5	AD	2.2:2.3	2.1	2.5	7.2E-37	0.159	-0.016	5.3E-01	6.2E-01	4.5E-01
BRCA	NPIPB5_ES_3.1_2.5_5	NPIPB5	ES	3.1	2.5	5	5.4E-27	0.114	0.071	2.0E-01	4.0E-01	8.7E-01
BRCA	NPIPB5_RI_2.3:2.4_2.2_2.5	NPIPB5	RI	2.3:2.4	2.2	2.5	5.7E-55	0.210	0.342	3.1E-02	1.1E-02	8.1E-05
BRCA	NPIPB5_RI_2.4_2.3_2.5	NPIPB5	RI	2.4	2.3	2.5	2.9E-89	0.319	0.217	3.0E-01	4.7E-02	2.6E-02
BRCA	NPRL3_ES_2:3_1.4_4	NPRL3	ES	2:03	1.4	4	2.2E-34	0.137	0.044	1.1E-01	6.4E-03	6.0E-02
BRCA	NPRL3_ES_5_4_6	NPRL3	ES	5	4	6	4.9E-28	0.113	-0.076	7.1E-02	2.0E-01	5.0E-01
BRCA	NSUN5_RI_9.2_9.1_9.3	NSUN5	RI	9.2	9.1	9.3	2.2E-36	0.144	0.128	1.8E-02	2.2E-01	9.7E-01
BRCA	NT5C3A_ES_3_1_5	NT5C3A	ES	3	1	5	1.8E-24	0.101	-0.071	4.3E-01	8.2E-01	2.7E-01
BRCA	NT5C3B_ES_3_2_4	NT5C3B	ES	3	2	4	3.1E-39	0.155	0.015	1.5E-01	9.0E-01	4.4E-01
BRCA	NUDT8_RI_3.2_3.1_3.3	NUDT8	RI	3.2	3.1	3.3	1.6E-54	0.212	-0.045	8.1E-02	3.3E-02	9.7E-02

cancer type	id	Gene Symbol	splice_type	Exon	From.Exon	To.Exon	anova.p	adj.r2	r	p.50	p.25	p.10
BRCA	NUMA1_ES_18_17_19	NUMA1	ES	18	17	19	5.0E-53	0.202	-0.294	4.4E-01	7.9E-01	3.2E-01
BRCA	NUMB_ES_13_12_14	NUMB	ES	13	12	14	2.1E-64	0.241	0.200	5.5E-01	2.0E-01	9.1E-01
BRCA	NUMB_ES_7_6_8.2	NUMB	ES	7	6	8.2	1.4E-32	0.134	-0.022	3.6E-01	4.3E-01	3.2E-01
BRCA	NXF1_RI_11.2_11.1_11.3	NXF1	RI	11.2	11.1	11.3	2.7E-33	0.132	0.643	8.0E-01	2.9E-01	1.7E-01
BRCA	ODF2L_AA_20.1_19_20.2	ODF2L	AA	20.1	19	20.2	1.0E-36	0.145	-0.411	1.5E-02	4.7E-02	1.3E-01
BRCA	ODF3B_ES_3.1:3.2_2.2_4.2	ODF3B	ES	3.1:3.2	2.2	4.2	2.1E-32	0.138	0.090	8.3E-01	4.6E-01	4.3E-01
BRCA	OGG1_RI_6.2:6.3_6.1_6.4	OGG1	RI	6.2:6.3	6.1	6.4	4.8E-27	0.109	-0.034	1.3E-01	6.5E-01	6.8E-01
BRCA	OPA1_ES_7_6_8	OPA1	ES	7	6	8	8.0E-27	0.111	-0.118	7.8E-01	8.5E-01	4.2E-01
BRCA	OS9_ES_13_12_14	OS9	ES	13	12	14	2.2E-88	0.314	0.133	6.1E-01	9.9E-01	6.9E-01
BRCA	OSBPL3_ES_9_8_10	OSBPL3	ES	9	8	10	1.9E-81	0.333	-0.475	4.4E-01	2.6E-01	7.3E-01
BRCA	OSBPL9_ES_17_15_18	OSBPL9	ES	17	15	18	5.2E-64	0.242	-0.143	1.5E-02	5.4E-02	3.0E-02
BRCA	P2RX4_ES_2:3_1_4	P2RX4	ES	2:03	1	4	1.8E-22	0.100	-0.120	5.4E-01	9.3E-01	7.4E-01
BRCA	P4HA1_ME_10|11_9_12.1	P4HA1	ME	10|11	9	12.1	1.2E-37	0.149	-0.219	4.6E-01	8.4E-01	9.7E-01
BRCA	PABPN1_RI_5.2_5.1_5.3	PABPN1	RI	5.2	5.1	5.3	1.7E-27	0.111	0.000	4.6E-01	8.0E-01	4.5E-01
BRCA	PAIP1_AD_2.2_2.1_3	PAIP1	AD	2.2	2.1	3	1.2E-30	0.123	0.041	3.6E-01	2.3E-01	1.2E-01
BRCA	PAN3_ES_5_4_6	PAN3	ES	5	4	6	3.9E-41	0.171	0.110	3.8E-01	1.7E-01	2.8E-01
BRCA	PAPOLA_ES_20_18_21	PAPOLA	ES	20	18	21	7.2E-30	0.125	-0.025	6.8E-01	7.1E-01	1.5E-01
BRCA	PARD3_ES_13_12_14	PARD3	ES	13	12	14	2.7E-31	0.127	-0.251	7.7E-01	9.1E-01	8.9E-01
BRCA	PARK7_AD_1.2_1.1_3	PARK7	AD	1.2	1.1	3	1.8E-28	0.114	0.176	9.2E-01	4.3E-01	1.0E-01
BRCA	PARPBP_ES_3_2_4.1	PARPBP	ES	3	2	4.1	2.3E-24	0.120	-0.482	9.0E-01	5.8E-01	2.1E-01
BRCA	PBX1_ES_11_10_12	PBX1	ES	11	10	12	3.6E-121	0.403	0.540	5.1E-01	4.0E-01	5.2E-01
BRCA	PCBP2_ES_15_14.1_16.1	PCBP2	ES	15	14.1	16.1	8.2E-39	0.153	-0.143	2.8E-01	5.4E-01	1.4E-01
BRCA	PCM1_ES_25_24_27	PCM1	ES	25	24	27	6.8E-38	0.150	0.259	7.9E-01	7.9E-01	6.0E-01
BRCA	PCYT2_ES_7_6_8	PCYT2	ES	7	6	8	1.6E-32	0.130	0.132	9.3E-01	5.4E-01	7.4E-01
BRCA	PFDN5_ES_4.1:4.2:5_1_6.2	PFDN5	ES	4.1:4.2:5	1	6.2	2.8E-37	0.157	-0.039	4.0E-01	6.2E-01	2.5E-01
BRCA	PFDN5_ES_4.2:5_1_6.2	PFDN5	ES	4.2:5	1	6.2	2.8E-30	0.137	-0.031	8.0E-01	4.8E-01	6.1E-01
BRCA	PGBD2_ES_3_2_4.1	PGBD2	ES	3	2	4.1	6.4E-21	0.102	-0.048	9.3E-01	6.9E-01	4.0E-01
BRCA	PHF8_ES_15_14_16	PHF8	ES	15	14	16	6.7E-30	0.121	0.199	5.4E-01	7.9E-01	8.1E-01
BRCA	PI4KB_ES_5_4_6	PI4KB	ES	5	4	6	1.1E-117	0.395	-0.094	7.1E-01	5.5E-01	2.8E-01
BRCA	PIGO_RI_9.2_9.1_9.3	PIGO	RI	9.2	9.1	9.3	2.1E-40	0.158	0.039	1.5E-01	7.0E-01	3.4E-01
BRCA	PIGQ_ES_12:13_11_14	PIGQ	ES	12:13	11	14	3.1E-47	0.184	0.398	5.0E-01	1.4E-01	5.6E-02
BRCA	PIGQ_ES_13_11_14	PIGQ	ES	13	11	14	4.5E-33	0.132	0.377	6.2E-01	1.3E-01	3.7E-01
BRCA	PIGS_RI_1.2_1.1_1.3	PIGS	RI	1.2	1.1	1.3	2.9E-25	0.108	-0.295	7.8E-01	7.0E-01	3.4E-01
BRCA	PIGT_ES_2.1_1_4	PIGT	ES	2.1	1	4	3.1E-26	0.113	0.201	8.7E-01	5.7E-01	4.4E-01
BRCA	PIGT_ES_2.1:2.2_1_4	PIGT	ES	2.1:2.2	1	4	1.6E-41	0.168	0.121	8.4E-01	6.9E-01	5.5E-01
BRCA	PILRB_RI_8.4_8.3_8.5	PILRB	RI	8.4	8.3	8.5	7.7E-38	0.152	0.177	4.8E-01	9.9E-01	6.2E-01
BRCA	PKMYT1_AA_9.1:9.2_8_9.3	PKMYT1	AA	9.1:9.2	8	9.3	2.9E-25	0.102	-0.227	8.5E-03	8.8E-02	1.5E-01
BRCA	PLA2G10_ES_4_3_5	PLA2G10	ES	4	3	5	5.0E-24	0.117	-0.709	7.8E-02	2.4E-01	2.1E-01
BRCA	PLCD4_ES_13_12_14	PLCD4	ES	13	12	14	6.1E-72	0.275	-0.758	7.4E-01	8.4E-01	6.6E-01
BRCA	PLD1_ES_16_15_17	PLD1	ES	16	15	17	2.3E-31	0.158	-0.303	8.9E-01	9.4E-01	9.5E-01

cancer type	id	Gene Symbol	splice_type	Exon	From.Exon	To.Exon	anova.p	adj.r2	r	p.50	p.25	p.10
BRCA	PLD3_ES_3_1.2_5.2	PLD3	ES	3	1.2	5.2	1.2E-26	0.111	-0.275	3.3E-01	5.3E-01	5.4E-01
BRCA	PLEKHA4_ES_18_17_19	PLEKHA4	ES	18	17	19	1.7E-37	0.152	0.122	5.5E-01	8.6E-01	8.9E-01
BRCA	PLOD2_ES_15_14_16	PLOD2	ES	15	14	16	7.1E-31	0.126	-0.350	4.6E-02	3.7E-01	6.9E-01
BRCA	PLXNB2_ES_2.1_1_3	PLXNB2	ES	2.1	1	3	1.9E-52	0.258	0.068	8.0E-01	5.8E-01	3.8E-01
BRCA	POLB_ES_2_1_3	POLB	ES	2	1	3	1.7E-49	0.190	0.013	1.1E-01	6.8E-02	1.3E-01
BRCA	POLR2G_AD_2.5_2.4_3	POLR2G	AD	2.5	2.4	3	7.1E-31	0.126	-0.255	5.5E-01	7.7E-01	8.8E-01
BRCA	POLR2H_ES_5:6.1_4_6.2	POLR2H	ES	05:06.1	4	6.2	2.4E-25	0.102	0.174	2.0E-01	2.2E-01	8.1E-01
BRCA	POLR2J3_ES_6:7_4.3_8	POLR2J3	ES	6:07	4.3	8	6.1E-27	0.108	0.405	6.7E-01	1.2E-01	3.4E-01
BRCA	POSTN_ES_17:18_16_19	POSTN	ES	17:18	16	19	2.9E-36	0.144	0.157	2.1E-02	3.2E-03	2.9E-02
BRCA	POSTN_ES_17:18:19_16_20	POSTN	ES	17:18:19	16	20	3.9E-78	0.284	0.222	3.3E-02	1.6E-02	5.5E-01
BRCA	POSTN_ES_17:19_16_20	POSTN	ES	17:19	16	20	1.0E-32	0.144	-0.033	7.2E-01	3.6E-01	7.4E-01
BRCA	PPIP5K1_ES_28_27_29	PPIP5K1	ES	28	27	29	2.3E-94	0.342	0.325	8.0E-01	8.3E-01	5.6E-01
BRCA	PPM1A_ES_3_2_5.1	PPM1A	ES	3	2	5.1	8.2E-25	0.109	0.063	8.4E-01	2.3E-01	4.4E-01
BRCA	PPP1R7_AD_1.2:1.3_1.1_3	PPP1R7	AD	1.2:1.3	1.1	3	2.1E-27	0.119	-0.081	9.5E-01	8.8E-01	7.1E-01
BRCA	PPP3CC_ES_14_13_15	PPP3CC	ES	14	13	15	2.9E-29	0.118	0.104	2.3E-02	7.7E-02	8.5E-01
BRCA	PPP6R2_ES_17_16_18	PPP6R2	ES	17	16	18	1.3E-35	0.141	-0.001	5.7E-01	4.5E-01	3.3E-02
BRCA	PQBP1_AD_1.2:1.3_1.1_2.3	PQBP1	AD	1.2:1.3	1.1	2.3	1.2E-61	0.232	-0.124	7.9E-01	3.9E-01	8.2E-01
BRCA	PQLC3_ES_5:6_4_7	PQLC3	ES	5:06	4	7	3.3E-26	0.115	-0.214	2.6E-01	5.9E-02	2.6E-01
BRCA	PQLC3_ES_6:7_5_8	PQLC3	ES	6:07	5	8	1.7E-40	0.166	-0.254	8.8E-01	5.7E-01	5.9E-01
BRCA	PRDX5_ES_3_1_4	PRDX5	ES	3	1	4	7.1E-80	0.289	-0.003	3.2E-02	9.9E-01	5.8E-01
BRCA	PREPL_ES_1.3:2.2:2.3_1.2_3	PREPL	ES	1.3:2.2:2.3	1.2	3	5.6E-27	0.135	-0.357	4.1E-01	6.4E-01	5.1E-01
BRCA	PREPL_ES_2.2:2.3_1.3_3	PREPL	ES	2.2:2.3	1.3	3	2.2E-40	0.174	-0.315	3.6E-01	6.9E-01	7.2E-01
BRCA	PRICKLE4_AA_8.1_7_8.2	PRICKLE4	AA	8.1	7	8.2	6.8E-43	0.173	0.130	8.6E-01	5.6E-01	6.5E-01
BRCA	PRMT1_ES_4.2:5_4.1_6	PRMT1	ES	4.2:5	4.1	6	1.8E-27	0.111	-0.225	9.1E-01	9.7E-01	9.1E-01
BRCA	PRMT2_ES_8_6.1_12	PRMT2	ES	8	6.1	12	1.3E-26	0.118	-0.180	1.6E-02	9.9E-02	2.0E-01
BRCA	PROM1_ES_27:28_25_30	PROM1	ES	27:28:00	25	30	2.0E-35	0.182	-0.276	8.9E-01	4.7E-01	9.5E-01
BRCA	PRPF3_ES_4_3_5	PRPF3	ES	4	3	5	9.4E-67	0.249	-0.132	8.9E-01	6.8E-01	7.4E-01
BRCA	PRPF40A_ES_8_7_9	PRPF40A	ES	8	7	9	3.9E-69	0.265	-0.015	7.2E-01	9.2E-01	5.9E-01
BRCA	PRRC2B_ES_16_15_17	PRRC2B	ES	16	15	17	2.4E-44	0.172	-0.001	8.6E-01	6.6E-01	4.7E-01
BRCA	PRRC2C_ES_34_33_35	PRRC2C	ES	34	33	35	1.8E-39	0.156	-0.124	6.0E-01	7.0E-01	6.2E-01
BRCA	PRRT3_RI_4.2_4.1_4.3	PRRT3	RI	4.2	4.1	4.3	1.5E-47	0.184	0.435	7.0E-01	3.7E-01	9.8E-01
BRCA	PSMC3IP_AD_4.2:4.3_4.1_5.1	PSMC3IP	AD	4.2:4.3	4.1	5.1	1.3E-41	0.196	-0.314	4.4E-01	5.3E-01	5.0E-01
BRCA	PSMC3IP_RI_1.2_1.1_1.3	PSMC3IP	RI	1.2	1.1	1.3	1.4E-23	0.114	0.045	9.4E-01	5.8E-01	3.5E-01
BRCA	PSMC5_RI_2.2_2.1_2.3	PSMC5	RI	2.2	2.1	2.3	2.1E-27	0.110	-0.033	7.8E-01	2.3E-01	3.8E-01
BRCA	PTBP2_ES_12_11.2_13	PTBP2	ES	12	11.2	13	3.1E-33	0.152	0.296	6.6E-01	4.6E-01	9.8E-01
BRCA	PTK2_ES_39.2_37_39.5	PTK2	ES	39.2	37	39.5	3.4E-74	0.272	0.016	3.3E-01	7.3E-01	6.8E-01
BRCA	PUM2_ES_16_15_17	PUM2	ES	16	15	17	1.3E-35	0.143	-0.094	2.4E-02	2.1E-02	6.4E-01
BRCA	PVRL4_ES_8_7_9	PVRL4	ES	8	7	9	1.3E-33	0.135	0.087	3.1E-01	2.2E-01	8.6E-01
BRCA	PXN_ES_13:14.1_9_14.2	PXN	ES	13:14.1	9	14.2	2.3E-24	0.100	-0.053	3.8E-01	4.4E-01	4.7E-01
BRCA	R3HDM1_ES_16_15_17	R3HDM1	ES	16	15	17	1.4E-54	0.216	0.227	5.2E-01	9.7E-01	8.1E-01

cancer type	id	Gene Symbol	splice_type	Exon	From.Exon	To.Exon	anova.p	adj.r2	r	p.50	p.25	p.10
BRCA	RAI14_ES_18_17_19	RAI14	ES	18	17	19	5.7E-27	0.113	0.044	7.8E-01	8.3E-01	6.1E-01
BRCA	RALGAPA1_ES_42_41_43	RALGAPA1	ES	42	41	43	1.4E-156	0.489	-0.505	8.5E-01	7.1E-01	8.8E-01
BRCA	RALGPS2_ES_15_14_16	RALGPS2	ES	15	14	16	8.7E-108	0.386	0.460	7.0E-01	8.1E-01	9.7E-01
BRCA	RANBP17_ES_30_29_31	RANBP17	ES	30	29	31	1.2E-27	0.136	-0.634	6.8E-01	4.9E-01	7.9E-01
BRCA	RAPH1_ES_5:6_4_7	RAPH1	ES	5:06	4	7	2.2E-21	0.111	-0.070	9.3E-01	5.1E-01	7.6E-01
BRCA	RASAL2_AA_14.1_13_14.2	RASAL2	AA	14.1	13	14.2	9.5E-31	0.146	0.085	7.9E-01	4.2E-01	7.4E-01
BRCA	RASSF7_RI_6.2_6.1_6.3	RASSF7	RI	6.2	6.1	6.3	1.0E-37	0.149	-0.141	4.0E-01	9.9E-01	1.0E+00
BRCA	RASSF7_RI_6.2:6.3:6.4_6.1_6.5	RASSF7	RI	6.2:6.3:6.4	6.1	6.5	5.4E-41	0.160	-0.051	7.4E-01	9.6E-01	4.6E-01
BRCA	RASSF7_RI_6.4_6.3_6.5	RASSF7	RI	6.4	6.3	6.5	5.3E-43	0.168	-0.154	9.4E-01	6.4E-01	3.1E-01
BRCA	RBM26_ES_14_13.2_15	RBM26	ES	14	13.2	15	1.6E-40	0.161	-0.169	1.0E+00	7.8E-01	5.8E-01
BRCA	RBM42_ES_3.2:4:6.1:6.2:6.3:7:8:9.1_3.1	RBM42	ES	:4:6.1:6.2:6.3:7:8	3.1	9.2	3.3E-25	0.112	-0.015	8.0E-01	2.9E-01	5.7E-01
BRCA	RCC1_ES_7_6_8	RCC1	ES	7	6	8	4.4E-44	0.173	-0.214	7.6E-01	6.5E-01	9.9E-02
BRCA	RCOR3_ES_14.1_13_15	RCOR3	ES	14.1	13	15	5.5E-41	0.161	-0.132	5.5E-01	3.7E-01	1.7E-01
BRCA	RDH13_ES_8_7_9.1	RDH13	ES	8	7	9.1	3.8E-33	0.133	-0.041	8.6E-01	7.9E-01	9.4E-01
BRCA	REPIN1_ES_4.1:4.2_3.2_5.2	REPIN1	ES	4.1:4.2	3.2	5.2	1.0E-24	0.101	-0.168	3.8E-01	5.9E-04	9.6E-03
BRCA	RGS12_ES_19_18_20.1	RGS12	ES	19	18	20.1	4.2E-28	0.113	0.120	1.8E-02	2.7E-01	1.8E-01
BRCA	RHOT1_ES_21_20_22	RHOT1	ES	21	20	22	3.3E-50	0.198	-0.047	1.5E-01	2.8E-01	3.7E-01
BRCA	RIBC1_ES_5.1_4_6	RIBC1	ES	5.1	4	6	2.5E-38	0.176	-0.300	6.2E-01	3.5E-01	5.0E-01
BRCA	RIPK2_ES_3_1_4	RIPK2	ES	3	1	4	6.6E-38	0.151	-0.328	6.9E-02	8.8E-02	6.1E-02
BRCA	RNF167_AD_1.2_1.1_2.2	RNF167	AD	1.2	1.1	2.2	1.7E-46	0.180	-0.140	2.4E-01	6.9E-01	1.0E+00
BRCA	RNF181_ES_3.2_2_4.1	RNF181	ES	3.2	2	4.1	1.5E-27	0.111	0.021	2.1E-01	8.8E-02	3.8E-01
BRCA	RNF34_ES_2_1_3	RNF34	ES	2	1	3	2.7E-54	0.213	0.002	6.0E-01	6.8E-01	4.9E-01
BRCA	RNH1_AA_4.2_1_4.3	RNH1	AA	4.2	1	4.3	9.8E-33	0.130	-0.199	5.8E-01	3.8E-01	1.6E-01
BRCA	RNPS1_ES_3_1.1_4	RNPS1	ES	3	1.1	4	2.0E-62	0.234	-0.086	9.7E-01	1.2E-01	3.0E-02
BRCA	RORC_ES_2_1_3	RORC	ES	2	1	3	3.0E-26	0.123	-0.233	6.4E-01	3.1E-01	2.5E-01
BRCA	RPL10_RI_2.2_2.1_2.3	RPL10	RI	2.2	2.1	2.3	3.3E-38	0.150	-0.074	9.2E-01	7.1E-01	6.1E-01
BRCA	RPL13_AD_1.3:1.4_1.2_2	RPL13	AD	1.3:1.4	1.2	2	1.2E-26	0.111	-0.066	1.8E-01	1.5E-02	3.3E-01
BRCA	RPL15_AD_1.3_1.2_3.2	RPL15	AD	1.3	1.2	3.2	1.1E-93	0.329	-0.243	5.2E-03	2.7E-02	6.8E-01
BRCA	RPL18A_ES_2.2:2.4_1_3	RPL18A	ES	2.2:2.4	1	3	2.5E-28	0.134	0.059	9.8E-01	7.8E-01	4.6E-01
BRCA	RPL29_RI_3.2_3.1_3.3	RPL29	RI	3.2	3.1	3.3	4.2E-27	0.109	-0.191	9.5E-01	4.1E-01	6.0E-01
BRCA	RPL30_ES_2.1:2.2_1.3_3.1	RPL30	ES	2.1:2.2	1.3	3.1	1.1E-49	0.191	-0.347	3.2E-01	9.6E-02	4.7E-01
BRCA	RPL32_RI_1.2_1.1_1.3	RPL32	RI	1.2	1.1	1.3	5.5E-28	0.112	-0.171	1.9E-01	7.2E-02	6.9E-01
BRCA	RPS15_ES_2:3.2_1.5_3.3	RPS15	ES	02:03.2	1.5	3.3	1.2E-30	0.123	-0.076	2.9E-01	5.5E-01	4.5E-01
BRCA	RPS20_RI_1.3_1.2_1.4	RPS20	RI	1.3	1.2	1.4	4.5E-27	0.111	-0.259	7.2E-01	8.3E-01	4.1E-01
BRCA	RPS24_AA_5.1_4_5.2	RPS24	AA	5.1	4	5.2	7.0E-156	0.485	-0.058	2.8E-01	7.7E-01	4.7E-01
BRCA	RPS24_ES_5.1:5.2_4_6	RPS24	ES	5.1:5.2	4	6	9.6E-206	0.583	-0.119	2.4E-01	8.2E-01	1.6E-01
BRCA	RPS24_ES_5.2_4_6	RPS24	ES	5.2	4	6	1.6E-128	0.421	-0.153	9.9E-01	9.3E-01	6.5E-01
BRCA	RPS25_ES_2.1:2.2_1_3.1	RPS25	ES	2.1:2.2	1	3.1	2.7E-32	0.166	-0.072	1.2E-01	4.3E-01	7.8E-01
BRCA	RPS6_RI_1.3_1.2_1.4	RPS6	RI	1.3	1.2	1.4	5.1E-29	0.123	-0.103	6.5E-01	3.0E-01	7.1E-01
BRCA	RPS9_ES_4.1:4.2:4.3_3_4.5	RPS9	ES	4.1:4.2:4.3	3	4.5	2.5E-33	0.133	-0.359	8.5E-03	3.4E-02	3.0E-01

cancer type	id	Gene Symbol	splice_type	Exon	From.Exon	To.Exon	anova.p	adj.r2	r	p.50	p.25	p.10
BRCA	RPS9_ES_4.1:4.3_3_4.5	RPS9	ES	4.1:4.3	3	4.5	7.0E-54	0.205	-0.106	3.2E-02	3.6E-01	1.5E-01
BRCA	RPS9_ES_4.1:4.3:4.4_3_4.5	RPS9	ES	4.1:4.3:4.4	3	4.5	1.6E-40	0.159	-0.291	2.8E-01	4.3E-01	1.1E-01
BRCA	RTN4_ES_6.1:6.2_5_8	RTN4	ES	6.1:6.2	5	8	2.1E-46	0.187	-0.088	1.4E-01	9.1E-02	1.4E-01
BRCA	SAR1B_ES_6_5.2_8	SAR1B	ES	6	5.2	8	1.1E-32	0.130	-0.341	NA	NA	NA
BRCA	SCMH1_ES_17_16_18	SCMH1	ES	17	16	18	3.2E-46	0.179	-0.203	2.9E-01	6.7E-01	9.7E-01
BRCA	SCP2_ES_12_11_13	SCP2	ES	12	11	13	1.3E-67	0.250	-0.652	4.8E-01	6.2E-01	2.8E-01
BRCA	SCRIB_ES_17_16_18	SCRIB	ES	17	16	18	3.4E-121	0.416	-0.335	1.8E-01	5.1E-01	9.7E-01
BRCA	SCRIB_ES_36_35_37	SCRIB	ES	36	35	37	9.1E-28	0.112	-0.129	6.3E-01	5.6E-01	5.3E-01
BRCA	SDCCAG3_ES_2_1_4	SDCCAG3	ES	2	1	4	5.1E-29	0.119	0.150	6.6E-01	1.2E-01	1.6E-01
BRCA	SEC16A_ES_24:25_23.12_26	SEC16A	ES	24:25:00	23.12	26	2.2E-59	0.224	-0.349	9.7E-01	7.2E-01	2.8E-01
BRCA	SEC16A_ES_25_23.12_26	SEC16A	ES	25	23.12	26	3.9E-112	0.397	-0.470	5.9E-01	7.7E-01	3.9E-01
BRCA	SEC16A_ES_25_24_26	SEC16A	ES	25	24	26	2.5E-162	0.499	-0.538	8.4E-01	7.9E-01	4.4E-01
BRCA	SEC31A_ES_16:17_15_18	SEC31A	ES	16:17	15	18	4.3E-28	0.114	-0.055	4.9E-01	4.4E-01	2.5E-01
BRCA	SEC31A_ES_26.1_25.1_28	SEC31A	ES	26.1	25.1	28	2.7E-41	0.162	-0.086	2.4E-01	6.2E-01	9.4E-01
BRCA	SEC31A_ES_26.1:26.2_25.1_28	SEC31A	ES	26.1:26.2	25.1	28	3.3E-44	0.172	-0.123	7.6E-01	9.3E-01	7.7E-01
BRCA	SEC31A_ES_26.1:26.2:27_25.1_28	SEC31A	ES	26.1:26.2:27	25.1	28	1.4E-47	0.184	-0.012	4.5E-01	5.4E-01	1.8E-01
BRCA	SEC31A_ES_26.2:27_26.1_28	SEC31A	ES	26.2:27	26.1	28	6.2E-141	0.452	0.044	7.1E-01	4.6E-01	2.1E-01
BRCA	SEC31A_ES_27_26.1_28	SEC31A	ES	27	26.1	28	1.1E-44	0.196	0.004	8.8E-01	5.6E-01	3.8E-01
BRCA	SEC31A_ES_27_26.2_28	SEC31A	ES	27	26.2	28	3.6E-88	0.314	0.077	8.0E-01	6.5E-01	2.6E-01
BRCA	SEC61A2_ES_9_6_10	SEC61A2	ES	9	6	10	7.0E-25	0.105	0.347	9.5E-01	5.0E-01	3.2E-01
BRCA	SEMA3F_ES_7_6_8	SEMA3F	ES	7	6	8	2.1E-52	0.202	0.522	6.6E-02	4.9E-01	2.7E-02
BRCA	SENP6_ES_8_6_9	SENP6	ES	8	6	9	5.2E-27	0.114	-0.055	4.3E-01	4.4E-01	6.5E-01
BRCA	SEPT6_AA_13.1_11.1_13.2	SEPT6	AA	13.1	11.1	13.2	3.2E-31	0.132	0.145	1.4E-01	5.7E-01	1.4E-01
BRCA	SEPT6_ES_12_11.1_13.1	SEPT6	ES	12	11.1	13.1	3.3E-55	0.211	0.087	5.5E-01	4.1E-01	6.5E-01
BRCA	SERHL2_AD_10.2_10.1_11	SERHL2	AD	10.2	10.1	11	1.1E-76	0.279	-0.832	9.5E-01	4.2E-01	3.1E-01
BRCA	SEZ6L2_ES_15_14_16	SEZ6L2	ES	15	14	16	5.5E-49	0.192	0.015	3.7E-01	9.2E-01	7.8E-01
BRCA	SH2B1_AD_9.2_9.1_10	SH2B1	AD	9.2	9.1	10	1.1E-48	0.187	0.035	3.8E-01	6.5E-01	5.0E-01
BRCA	SH3BP1_ES_16_15_17.2	SH3BP1	ES	16	15	17.2	7.0E-35	0.138	-0.570	5.2E-01	9.1E-01	9.7E-01
BRCA	SH3GL3_ES_2:3:4_1_5	SH3GL3	ES	2:03:04	1	5	7.0E-43	0.169	-0.710	2.7E-01	2.7E-01	2.4E-01
BRCA	SLC25A14_ES_2.2:2.3_1_3	SLC25A14	ES	2.2:2.3	1	3	3.9E-28	0.133	-0.076	9.0E-01	2.4E-01	3.3E-01
BRCA	SLC2A8_ES_2:3_1_4	SLC2A8	ES	2:03	1	4	4.1E-34	0.137	0.268	1.2E-01	3.4E-01	7.1E-01
BRCA	SLC43A3_AA_5.1_4.2_5.2	SLC43A3	AA	5.1	4.2	5.2	3.1E-34	0.148	0.328	8.0E-01	6.1E-01	3.3E-01
BRCA	SLC50A1_ES_4_3_6	SLC50A1	ES	4	3	6	6.8E-31	0.147	0.100	6.6E-01	8.3E-01	7.6E-01
BRCA	SLC52A2_AD_1.2:1.3:1.4_1.1_2.2	SLC52A2	AD	1.2:1.3:1.4	1.1	2.2	7.2E-27	0.110	-0.187	6.4E-01	6.9E-01	6.9E-01
BRCA	SLMAP_ES_25_24_26	SLMAP	ES	25	24	26	1.7E-118	0.398	-0.330	9.8E-01	6.1E-01	3.5E-01
BRCA	SMARCB1_AD_2.2_2.1_3	SMARCB1	AD	2.2	2.1	3	1.7E-33	0.133	-0.286	8.8E-01	4.9E-01	5.9E-02
BRCA	SMARCC2_AD_28.2:28.3_28.1_29	SMARCC2	AD	28.2:28.3	28.1	29	4.7E-32	0.128	0.207	2.0E-01	5.8E-01	2.7E-01
BRCA	SMARCC2_ES_18_17_19	SMARCC2	ES	18	17	19	1.4E-50	0.196	0.228	2.5E-01	9.2E-01	3.1E-01
BRCA	SMC5_ES_19_18_20	SMC5	ES	19	18	20	1.2E-33	0.143	-0.142	2.2E-01	9.4E-01	9.7E-01
BRCA	SMC6_ES_6_5_7	SMC6	ES	6	5	7	6.6E-38	0.180	0.254	6.5E-01	9.3E-01	5.1E-01

cancer type	id	Gene Symbol	splice_type	Exon	From.Exon	To.Exon	anova.p	adj.r2	r	p.50	p.25	p.10
BRCA	SMEK2_ES_10_9_11	SMEK2	ES	10	9	11	2.9E-30	0.123	0.095	5.8E-01	2.1E-01	5.7E-01
BRCA	SMG7_AD_16.2_16.1_17	SMG7	AD	16.2	16.1	17	5.6E-56	0.217	-0.128	3.8E-01	3.4E-01	4.5E-01
BRCA	SMG7_ES_20_19_21	SMG7	ES	20	19	21	5.3E-33	0.133	-0.054	7.3E-01	9.0E-01	4.7E-01
BRCA	SMPD4_ES_11_10_14	SMPD4	ES	11	10	14	2.0E-47	0.184	-0.008	9.4E-01	8.8E-01	6.6E-01
BRCA	SMPD4_ES_11:12_10_14	SMPD4	ES	11:12	10	14	2.2E-39	0.157	0.120	2.3E-01	8.6E-02	3.1E-01
BRCA	SMPD4_ES_11:13_10_14	SMPD4	ES	11:13	10	14	3.3E-40	0.160	0.170	4.4E-02	2.6E-01	5.1E-01
BRCA	SNAPC5_ES_1.2:2.1_1.1_3.1	SNAPC5	ES	1.2:2.1	1.1	3.1	1.2E-60	0.233	-0.124	7.5E-01	1.3E-01	9.5E-01
BRCA	SNRNP70_ES_8.1:8.2:8.3_7_9	SNRNP70	ES	8.1:8.2:8.3	7	9	2.9E-31	0.125	0.449	5.4E-01	6.5E-01	6.1E-01
BRCA	SORBS2_ES_20:22_19_23	SORBS2	ES	20:22	19	23	3.5E-47	0.203	-0.019	1.4E-01	2.6E-01	6.9E-01
BRCA	SORBS2_ES_8_7_9.1	SORBS2	ES	8	7	9.1	9.0E-69	0.327	-0.136	8.3E-01	5.6E-01	8.0E-01
BRCA	SORBS2_ES_8:9.1:9.2_7_10	SORBS2	ES	8:9.1:9.2	7	10	2.6E-50	0.226	0.087	1.2E-01	2.2E-01	6.3E-01
BRCA	SORBS2_ES_9.1:9.2_8_10	SORBS2	ES	9.1:9.2	8	10	9.6E-27	0.128	0.119	9.9E-01	4.4E-01	4.3E-01
BRCA	SPAG9_ES_30_29_31	SPAG9	ES	30	29	31	1.5E-39	0.156	-0.217	4.6E-01	5.2E-01	1.9E-01
BRCA	SPATA20_ES_4.2_3_6	SPATA20	ES	4.2	3	6	3.9E-26	0.123	0.296	2.9E-01	6.1E-01	2.5E-01
BRCA	SPATS2L_ES_10_9_11	SPATS2L	ES	10	9	11	2.7E-28	0.114	0.023	5.3E-01	3.8E-01	6.8E-01
BRCA	SPIDR_AD_23.2_23.1_24	SPIDR	AD	23.2	23.1	24	6.7E-28	0.116	0.024	5.4E-01	5.8E-01	4.0E-01
BRCA	SPINT1_AD_5.2_5.1_6	SPINT1	AD	5.2	5.1	6	3.8E-43	0.168	0.036	5.5E-01	9.5E-02	4.4E-02
BRCA	SPOPL_ES_6_5_7	SPOPL	ES	6	5	7	6.4E-31	0.133	-0.021	1.0E+00	9.1E-01	7.2E-01
BRCA	SPTAN1_ES_38_36.1_39	SPTAN1	ES	38	36.1	39	1.0E-25	0.104	0.096	2.3E-01	6.2E-01	2.5E-01
BRCA	SREBF1_ES_2_1_3.2	SREBF1	ES	2	1	3.2	1.1E-122	0.410	0.662	5.4E-01	4.3E-01	7.9E-01
BRCA	SRRM1_ES_16_15_17	SRRM1	ES	16	15	17	9.4E-36	0.143	-0.121	6.9E-01	4.7E-01	6.1E-01
BRCA	SRSF11_RI_6.4_6.3_6.5	SRSF11	RI	6.4	6.3	6.5	1.1E-24	0.101	-0.072	4.3E-01	9.4E-01	4.4E-01
BRCA	SRSF4_ES_2:3:4_1_5	SRSF4	ES	2:03:04	1	5	1.6E-34	0.137	0.014	6.7E-01	2.0E-01	4.6E-01
BRCA	SRSF5_AD_2.2:2.3_2.1_4	SRSF5	AD	2.2:2.3	2.1	4	1.6E-52	0.201	-0.224	7.6E-01	7.6E-01	5.6E-01
BRCA	SSBP3_ES_7_6_8	SSBP3	ES	7	6	8	1.6E-37	0.148	0.086	7.5E-01	5.9E-01	3.9E-01
BRCA	ST7_ES_10_9_11.1	ST7	ES	10	9	11.1	7.3E-87	0.311	-0.048	2.5E-01	2.5E-01	1.5E-01
BRCA	STAU1_ES_4_3_5	STAU1	ES	4	3	5	3.3E-119	0.403	-0.035	7.9E-01	3.3E-01	3.4E-01
BRCA	STK40_ES_2_1_3	STK40	ES	2	1	3	9.8E-27	0.110	-0.064	4.1E-01	2.6E-01	8.7E-02
BRCA	STOML1_ES_5_4_6	STOML1	ES	5	4	6	6.9E-56	0.213	-0.023	7.3E-01	2.4E-01	7.1E-01
BRCA	STRA13_ES_3.1_2_4.1	STRA13	ES	3.1	2	4.1	2.1E-77	0.281	-0.119	7.8E-01	7.9E-01	8.2E-01
BRCA	STX16_ES_7_6_8	STX16	ES	7	6	8	7.5E-27	0.109	-0.114	8.2E-01	7.8E-01	5.6E-01
BRCA	SUCO_ES_13_12_14	SUCO	ES	13	12	14	1.1E-28	0.123	-0.084	1.6E-01	6.9E-01	5.9E-01
BRCA	SUCO_ES_4_3_5	SUCO	ES	4	3	5	2.6E-34	0.147	-0.104	8.7E-01	3.4E-01	5.2E-01
BRCA	SUGP2_RI_12.4_12.3_12.5	SUGP2	RI	12.4	12.3	12.5	4.2E-27	0.109	0.502	4.0E-01	9.4E-01	5.3E-01
BRCA	SUGT1_ES_7_6_8	SUGT1	ES	7	6	8	1.4E-46	0.180	-0.091	4.7E-01	9.0E-01	7.8E-02
BRCA	SUPT20H_AA_21.1_20_21.2	SUPT20H	AA	21.1	20	21.2	3.8E-73	0.268	0.008	3.0E-01	9.9E-01	3.1E-01
BRCA	SUPT20H_AA_21.1:21.2_20_21.3	SUPT20H	AA	21.1:21.2	20	21.3	2.7E-49	0.190	0.068	9.2E-01	1.9E-01	6.0E-01
BRCA	SUPT20H_ES_4_3_5	SUPT20H	ES	4	3	5	4.6E-42	0.171	-0.037	6.9E-01	8.3E-02	3.4E-01
BRCA	SUV420H2_ES_3_2_4	SUV420H2	ES	3	2	4	9.2E-48	0.185	0.101	1.2E-01	2.6E-01	9.2E-01
BRCA	SVIL_ES_10:11:12_9_13	SVIL	ES	10:11:12	9	13	4.3E-33	0.137	-0.197	6.3E-01	5.8E-01	2.0E-01

cancer type	id	Gene Symbol	splice_type	Exon	From.Exon	To.Exon	anova.p	adj.r2	r	p.50	p.25	p.10
BRCA	SYNGR2_AA_4.1:4.2_3.2_4.3	SYNGR2	AA	4.1:4.2	3.2	4.3	5.1E-28	0.113	-0.099	6.8E-02	4.3E-01	2.4E-01
BRCA	SYTL2_AA_11.1_10.3_11.2	SYTL2	AA	11.1	10.3	11.2	2.6E-38	0.161	0.431	2.6E-01	8.2E-02	3.8E-01
BRCA	SYTL2_AD_8.3_8.2_9	SYTL2	AD	8.3	8.2	9	3.7E-96	0.338	0.563	5.9E-01	6.2E-01	6.1E-01
BRCA	SYTL2_ES_11.2_10.3_12.2	SYTL2	ES	11.2	10.3	12.2	1.2E-92	0.339	-0.655	5.6E-01	3.7E-01	7.0E-01
BRCA	SYTL2_ES_13_12.2_14	SYTL2	ES	13	12.2	14	1.3E-56	0.217	0.590	3.7E-01	4.8E-01	4.3E-01
BRCA	SZRD1_ES_3_1_4.1	SZRD1	ES	3	1	4.1	2.8E-30	0.121	-0.352	6.2E-01	4.4E-01	6.2E-01
BRCA	SZRD1_ES_3:4.1_1_4.2	SZRD1	ES	03:04.1	1	4.2	6.6E-43	0.168	-0.316	9.2E-01	7.7E-01	2.9E-01
BRCA	TACC1_ES_2:3_1_7	TACC1	ES	2:03	1	7	2.8E-42	0.213	-0.192	2.4E-02	3.9E-01	2.2E-01
BRCA	TAF1D_AA_8.1_7_8.2	TAF1D	AA	8.1	7	8.2	6.6E-49	0.188	0.035	8.2E-01	6.7E-01	7.2E-01
BRCA	TANGO2_ES_7.2_6_8	TANGO2	ES	7.2	6	8	2.0E-47	0.185	0.085	7.1E-01	8.2E-01	4.4E-01
BRCA	TANGO2_ES_7.2:8_6_9	TANGO2	ES	7.2:8	6	9	7.4E-43	0.168	0.136	6.1E-01	7.9E-01	5.9E-01
BRCA	TANK_ES_2.2:3_2.1_4	TANK	ES	2.2:3	2.1	4	3.4E-79	0.290	0.064	6.6E-01	1.0E-01	2.9E-01
BRCA	TANK_ES_3_2.2_4	TANK	ES	3	2.2	4	3.9E-81	0.294	-0.025	2.8E-01	8.4E-01	9.4E-01
BRCA	TATDN1_ES_1.2:2:3:4.1:5:6_1.1_7	TATDN1	ES	1.2:2:3:4.1:5:6	1.1	7	4.2E-33	0.153	0.257	3.4E-01	2.0E-01	1.8E-01
BRCA	TAZ_AD_3.2:3.3_3.1_4	TAZ	AD	3.2:3.3	3.1	4	3.8E-26	0.106	0.269	9.8E-02	6.2E-01	9.1E-01
BRCA	TBC1D13_ES_3_2_4	TBC1D13	ES	3	2	4	4.1E-73	0.310	0.173	3.6E-01	5.2E-01	4.6E-01
BRCA	TBC1D15_ES_10_9_11	TBC1D15	ES	10	9	11	1.2E-61	0.236	0.259	9.9E-01	9.6E-01	6.1E-01
BRCA	TBC1D23_ES_15_14_16	TBC1D23	ES	15	14	16	3.1E-70	0.263	-0.024	6.7E-01	9.3E-01	7.8E-01
BRCA	TBL1X_ES_6_5_7	TBL1X	ES	6	5	7	6.9E-29	0.126	-0.042	4.7E-01	4.0E-01	6.5E-02
BRCA	TCERG1_ES_22_21_23	TCERG1	ES	22	21	23	7.4E-69	0.256	-0.149	3.2E-02	9.4E-01	7.4E-01
BRCA	TCF12_ES_18_17_19	TCF12	ES	18	17	19	7.3E-28	0.117	0.034	7.1E-01	9.3E-01	8.9E-01
BRCA	TCF7L2_ES_4_3_5	TCF7L2	ES	4	3	5	1.5E-23	0.104	0.289	8.1E-01	4.1E-01	4.6E-01
BRCA	TEAD2_ES_6_5_7.1	TEAD2	ES	6	5	7.1	2.0E-28	0.116	0.049	3.5E-01	2.9E-01	9.3E-01
BRCA	TERF1_ES_7_6_8	TERF1	ES	7	6	8	1.2E-45	0.177	0.130	9.4E-01	2.3E-01	7.1E-01
BRCA	THNSL2_ES_9_8_11	THNSL2	ES	9	8	11	4.3E-71	0.267	0.128	6.7E-01	6.3E-01	6.5E-01
BRCA	THNSL2_ME_9|10_8_11	THNSL2	ME	9|10	8	11	1.9E-72	0.270	0.316	2.1E-01	2.2E-01	4.1E-01
BRCA	TIA1_ES_5:6_4_7	TIA1	ES	5:06	4	7	3.9E-32	0.130	0.084	9.2E-01	8.3E-01	3.6E-01
BRCA	TIA1_ES_6_5_7	TIA1	ES	6	5	7	1.0E-42	0.168	0.084	2.0E-01	4.5E-01	9.1E-01
BRCA	TIA1_ES_8_7_9.1	TIA1	ES	8	7	9.1	9.0E-29	0.116	0.121	6.9E-01	4.1E-01	1.8E-01
BRCA	TIAL1_AA_3.1_2_3.2	TIAL1	AA	3.1	2	3.2	1.7E-35	0.141	0.079	9.5E-01	8.7E-01	1.9E-01
BRCA	TIGD6_AD_1.2_1.1_2	TIGD6	AD	1.2	1.1	2	1.0E-43	0.170	-0.337	9.0E-01	6.1E-01	1.9E-01
BRCA	TJP1_AD_30.2_30.1_31	TJP1	AD	30.2	30.1	31	1.2E-30	0.123	-0.081	1.7E-01	1.5E-01	5.5E-01
BRCA	TJP1_ES_23_22_24	TJP1	ES	23	22	24	6.2E-69	0.261	0.085	8.7E-01	2.8E-01	1.7E-01
BRCA	TKT_RI_16.2_16.1_16.3	TKT	RI	16.2	16.1	16.3	3.4E-29	0.117	-0.226	1.7E-01	5.1E-02	4.5E-02
BRCA	TMEM101_AD_2.2_2.1_3	TMEM101	AD	2.2	2.1	3	1.1E-73	0.270	-0.479	1.0E-01	2.9E-02	1.0E-01
BRCA	TMEM159_AA_2.1:2.2_1_2.3	TMEM159	AA	2.1:2.2	1	2.3	1.1E-24	0.100	-0.120	9.0E-01	4.5E-01	9.2E-01
BRCA	TMEM18_ES_3_1_4	TMEM18	ES	3	1	4	4.0E-36	0.143	-0.147	6.2E-01	3.3E-01	5.9E-01
BRCA	TMEM205_RI_2.4:2.5_2.3_2.6	TMEM205	RI	2.4:2.5	2.3	2.6	6.0E-25	0.101	0.139	7.8E-01	9.1E-01	7.2E-01
BRCA	TMEM230_AD_1.2:1.3_1.1_4	TMEM230	AD	1.2:1.3	1.1	4	2.7E-35	0.140	-0.162	2.5E-01	5.5E-01	9.6E-01
BRCA	TMEM25_ES_5_4_6	TMEM25	ES	5	4	6	4.2E-152	0.477	0.518	2.8E-01	9.1E-01	7.6E-01

cancer type	id	Gene Symbol	splice_type	Exon	From.Exon	To.Exon	anova.p	adj.r2	r	p.50	p.25	p.10
BRCA	TMEM45A_ES_2_1_3	TMEM45A	ES	2	1	3	4.0E-30	0.142	0.556	7.9E-01	9.4E-01	4.1E-01
BRCA	TMEM5_ES_3_2_4	TMEM5	ES	3	2	4	1.5E-30	0.123	-0.291	9.1E-01	7.1E-01	6.3E-01
BRCA	TMEM67_ES_4_3.2_6	TMEM67	ES	4	3.2	6	1.3E-20	0.104	-0.408	3.2E-01	2.1E-01	6.8E-01
BRCA	TMPO_ES_6:7:8_5.1_9	TMPO	ES	6:07:08	5.1	9	2.9E-84	0.302	0.200	5.0E-01	1.7E-01	7.6E-01
BRCA	TMPO_ES_7:8_5.1_9	TMPO	ES	7:08	5.1	9	1.6E-60	0.243	0.183	8.9E-01	6.3E-01	4.6E-01
BRCA	TMUB2_ES_2.3:2.4:2.5:3_2.2_4.3	TMUB2	ES	2.3:2.4:2.5:3	2.2	4.3	3.6E-35	0.146	-0.060	2.5E-01	3.0E-01	7.2E-01
BRCA	TMUB2_ES_2.5:3_1_4.3	TMUB2	ES	2.5:3	1	4.3	4.2E-20	0.107	-0.013	3.7E-01	9.4E-01	6.1E-01
BRCA	TMUB2_ES_3_2.5_4.3	TMUB2	ES	3	2.5	4.3	3.0E-29	0.123	-0.055	4.5E-01	6.4E-01	3.5E-01
BRCA	TMUB2_ES_4.7:4.8_4.5_5	TMUB2	ES	4.7:4.8	4.5	5	1.1E-50	0.210	-0.017	2.7E-01	4.5E-01	8.5E-01
BRCA	TMUB2_ES_4.8_4.5_5	TMUB2	ES	4.8	4.5	5	3.4E-53	0.217	0.003	5.6E-01	9.5E-01	7.9E-01
BRCA	TNC_ES_12:13:14:15:16:18:19_11_20	TNC	ES	:13:14:15:16:18:	11	20	1.2E-33	0.138	-0.026	4.8E-01	2.6E-02	3.1E-03
BRCA	TNC_ES_12:13:14:15:16:19_11_20	TNC	ES	2:13:14:15:16:1	11	20	9.3E-34	0.135	0.055	3.3E-01	2.2E-01	2.3E-02
BRCA	TNC_ES_12:13:15:16:18:19_11_20	TNC	ES	2:13:15:16:18:1	11	20	5.6E-27	0.116	-0.051	6.4E-01	7.3E-02	3.1E-01
BRCA	TNC_ES_12:13:15:16:19_11_20	TNC	ES	12:13:15:16:19	11	20	2.5E-40	0.167	0.077	8.3E-01	2.6E-01	2.8E-01
BRCA	TNFRSF12A_ES_3.1:3.2_2.1_4	TNFRSF12A	ES	3.1:3.2	2.1	4	3.9E-43	0.199	-0.118	1.8E-01	6.9E-02	2.0E-01
BRCA	TOP1MT_ES_4_3_5	TOP1MT	ES	4	3	5	1.6E-26	0.113	-0.353	5.9E-01	1.8E-01	2.7E-01
BRCA	TPD52L1_ES_8_6_10	TPD52L1	ES	8	6	10	1.5E-41	0.164	0.068	1.8E-01	9.1E-01	6.1E-01
BRCA	TPM1_ES_12.1_11.1_13.1	TPM1	ES	12.1	11.1	13.1	7.9E-32	0.142	-0.017	1.7E-01	9.2E-01	8.2E-01
BRCA	TRAPPC6A_AD_1.2_1.1_2	TRAPPC6A	AD	1.2	1.1	2	2.0E-28	0.116	0.060	7.9E-01	6.6E-01	9.0E-01
BRCA	TRIM33_ES_12_11_13	TRIM33	ES	12	11	13	2.6E-24	0.110	-0.057	4.0E-01	3.1E-01	3.7E-01
BRCA	TRPM2_ES_29_28_30	TRPM2	ES	29	28	30	9.8E-55	0.214	-0.410	6.1E-02	1.7E-01	2.8E-01
BRCA	TSC2_ES_27_26_28.1	TSC2	ES	27	26	28.1	3.4E-66	0.248	-0.089	8.0E-01	4.6E-01	4.7E-01
BRCA	TSC2_ES_27_26_28.2	TSC2	ES	27	26	28.2	7.5E-32	0.160	-0.176	4.7E-01	1.5E-01	5.4E-01
BRCA	TSC2_ES_27:28.1_26_28.2	TSC2	ES	27:28.1	26	28.2	2.4E-52	0.202	-0.082	4.1E-01	6.5E-01	1.2E-01
BRCA	TTC12_ES_7_6_8	TTC12	ES	7	6	8	9.7E-34	0.138	-0.395	2.2E-01	5.8E-01	3.1E-01
BRCA	TTC23_AA_14.1_13.1_14.2	TTC23	AA	14.1	13.1	14.2	1.6E-28	0.129	0.162	1.4E-02	2.3E-02	1.8E-02
BRCA	TTC39A_ES_16.1_15.4_17	TTC39A	ES	16.1	15.4	17	4.3E-90	0.359	0.536	1.1E-01	1.1E-01	5.4E-01
BRCA	TTLL3_RI_12.2_12.1_12.3	TTLL3	RI	12.2	12.1	12.3	5.2E-31	0.124	-0.214	4.8E-01	6.5E-01	3.5E-01
BRCA	TUFT1_ES_2:3_1_4	TUFT1	ES	2:03	1	4	1.2E-31	0.139	-0.069	1.6E-01	8.3E-01	4.2E-01
BRCA	TUFT1_ES_3_1_4	TUFT1	ES	3	1	4	2.4E-92	0.340	-0.070	1.3E-01	7.4E-01	3.5E-01
BRCA	TXNL4A_ES_4:7.2_3_9	TXNL4A	ES	04:07.2	3	9	6.7E-40	0.157	-0.296	1.9E-01	2.3E-01	4.9E-01
BRCA	TXNL4A_ES_6:7.2_3_9	TXNL4A	ES	06:07.2	3	9	1.6E-30	0.125	-0.459	2.4E-02	5.4E-01	9.9E-01
BRCA	UBE2D3_AD_2.4_2.3_3.3	UBE2D3	AD	2.4	2.3	3.3	1.5E-33	0.133	-0.034	3.1E-03	1.3E-02	4.4E-01
BRCA	UBOX5_ES_4_3_5	UBOX5	ES	4	3	5	7.5E-68	0.251	0.098	4.6E-01	9.9E-01	3.1E-01
BRCA	UBXN11_ES_4:5:6_2_7	UBXN11	ES	4:05:06	2	7	1.8E-21	0.109	0.134	7.9E-01	5.0E-01	1.1E-01
BRCA	UBXN11_ES_4:5:6:7:8:9_2_10	UBXN11	ES	4:5:6:7:8:9	2	10	3.5E-37	0.148	0.039	7.5E-01	5.7E-01	2.2E-01
BRCA	UIMC1_ES_6.2:7.1:7.2_6.1_8	UIMC1	ES	6.2:7.1:7.2	6.1	8	2.5E-28	0.147	0.054	8.9E-01	6.4E-01	8.9E-01
BRCA	UPF3B_ES_8_7_9	UPF3B	ES	8	7	9	6.5E-43	0.168	-0.177	7.7E-01	6.3E-01	7.9E-01
BRCA	UQCRB_RI_6.2:6.3_6.1_6.4	UQCRB	RI	6.2:6.3	6.1	6.4	5.5E-29	0.118	-0.194	9.9E-01	4.2E-01	4.9E-01
BRCA	URI1_ES_3_2_5	URI1	ES	3	2	5	2.6E-34	0.138	-0.073	4.5E-01	3.8E-01	1.6E-01

cancer type	id	Gene Symbol	splice_type	Exon	From.Exon	To.Exon	anova.p	adj.r2	r	p.50	p.25	p.10
BRCA	USF2_ES_3_2.3_4	USF2	ES	3	2.3	4	2.6E-94	0.331	0.065	7.8E-01	8.0E-01	1.9E-01
BRCA	USO1_ES_14_13_15	USO1	ES	14	13	15	3.0E-46	0.183	0.209	3.4E-01	9.1E-01	7.7E-01
BRCA	USP4_ES_9_6_10	USP4	ES	9	6	10	1.1E-26	0.109	-0.170	4.6E-01	5.5E-01	6.9E-01
BRCA	USP46_AD_11.2_11.1_12	USP46	AD	11.2	11.1	12	3.7E-27	0.126	0.002	6.8E-01	7.5E-01	8.8E-01
BRCA	VEGFA_ES_7.1:7.2_6_8.1	VEGFA	ES	7.1:7.2	6	8.1	2.7E-50	0.194	0.309	7.9E-01	5.5E-01	5.3E-01
BRCA	VEGFA_ES_8.1:8.2_6_9.1	VEGFA	ES	8.1:8.2	6	9.1	2.6E-42	0.165	-0.150	5.9E-01	4.9E-01	9.4E-01
BRCA	VPS28_RI_3.6_3.5_3.7	VPS28	RI	3.6	3.5	3.7	7.4E-22	0.101	0.025	1.4E-02	6.2E-01	7.0E-01
BRCA	VPS29_ES_3.1_1_5	VPS29	ES	3.1	1	5	2.1E-26	0.106	0.089	2.2E-01	1.7E-01	5.1E-02
BRCA	VPS29_ES_3.1:3.2_1_5	VPS29	ES	3.1:3.2	1	5	6.9E-31	0.123	0.028	1.7E-01	8.7E-02	7.6E-01
BRCA	VWA5A_ES_1.3_1.1_2.2	VWA5A	ES	1.3	1.1	2.2	2.1E-32	0.156	-0.198	9.0E-01	6.9E-01	2.5E-01
BRCA	WASF3_ME_7|8_6_9	WASF3	ME	7|8	6	9	9.6E-35	0.159	0.564	3.1E-01	3.4E-01	1.0E+00
BRCA	WDR62_RI_25.6_25.5_25.7	WDR62	RI	25.6	25.5	25.7	3.2E-31	0.125	-0.444	3.4E-01	3.6E-01	2.3E-01
BRCA	WDYHV1_AD_1.2_1.1_2	WDYHV1	AD	1.2	1.1	2	1.3E-72	0.267	-0.148	1.7E-01	3.2E-02	4.6E-01
BRCA	WIBG_ES_4_2_6	WIBG	ES	4	2	6	5.3E-51	0.196	-0.393	8.2E-01	2.8E-01	7.0E-01
BRCA	WIZ_ES_9_8_10	WIZ	ES	9	8	10	6.1E-39	0.154	-0.054	8.6E-01	2.2E-01	4.8E-01
BRCA	WNK1_ES_11.2:12_9_13.2	WNK1	ES	11.2:12	9	13.2	6.9E-31	0.163	0.144	9.2E-01	3.0E-01	8.7E-01
BRCA	WNK1_ES_11.2:12:13.1_9_13.2	WNK1	ES	11.2:12:13.1	9	13.2	1.6E-28	0.150	0.173	9.4E-01	2.9E-01	5.1E-01
BRCA	WNK2_ES_26:27_25.3_28	WNK2	ES	26:27:00	25.3	28	5.9E-33	0.163	0.100	1.2E-01	8.5E-01	8.1E-01
BRCA	WRAP73_RI_11.2_11.1_11.3	WRAP73	RI	11.2	11.1	11.3	5.5E-47	0.182	0.079	8.7E-01	8.5E-01	5.4E-01
BRCA	YIPF1_ES_12_11_13	YIPF1	ES	12	11	13	2.2E-29	0.118	-0.227	8.8E-01	5.7E-01	3.4E-01
BRCA	YIPF1_ES_3_2_4	YIPF1	ES	3	2	4	4.2E-100	0.348	-0.378	9.0E-01	6.8E-01	4.0E-01
BRCA	YIPF3_ES_3.1_2.5_4.1	YIPF3	ES	3.1	2.5	4.1	1.1E-26	0.135	-0.059	2.1E-02	1.3E-02	3.0E-02
BRCA	ZBTB7B_ES_3:4:5_2.2_6.2	ZBTB7B	ES	3:04:05	2.2	6.2	1.3E-22	0.105	0.196	5.1E-01	6.5E-01	7.1E-01
BRCA	ZBTB7B_ES_4_3_6.2	ZBTB7B	ES	4	3	6.2	6.2E-24	0.112	0.227	4.2E-01	2.7E-01	3.2E-01
BRCA	ZBTB7B_ES_4:5_3_6.2	ZBTB7B	ES	4:05	3	6.2	1.7E-26	0.131	0.207	7.6E-02	6.7E-02	8.8E-02
BRCA	ZC3H11A_AD_2.2_2.1_3.2	ZC3H11A	AD	2.2	2.1	3.2	7.4E-27	0.117	0.272	3.7E-01	2.3E-01	2.8E-01
BRCA	ZDHHC4_ES_1.2:1.3:2.1_1.1_2.2	ZDHHC4	ES	1.2:1.3:2.1	1.1	2.2	1.1E-38	0.156	-0.040	1.7E-01	6.3E-01	2.5E-01
BRCA	ZFP62_ES_2_1_3	ZFP62	ES	2	1	3	4.7E-42	0.195	-0.097	5.2E-01	1.0E+00	7.3E-01
BRCA	ZFP90_AA_4.1_3_4.2	ZFP90	AA	4.1	3	4.2	1.3E-30	0.134	0.161	6.0E-01	3.8E-01	2.3E-01
BRCA	ZFYVE19_ES_8_7_9	ZFYVE19	ES	8	7	9	4.7E-34	0.135	0.241	3.0E-01	5.4E-01	3.6E-02
BRCA	ZMIZ2_AA_5.1:5.2_4_5.3	ZMIZ2	AA	5.1:5.2	4	5.3	6.4E-38	0.151	0.117	4.8E-01	6.0E-01	6.2E-01
BRCA	ZNF131_ES_5.2:7.1:7.2:7.3:7.4:8:9.1:9.2_	ZNF131	ES	.1:7.2:7.3:7.4:8:9.	5.1	9.3	1.7E-21	0.102	-0.232	3.4E-01	3.4E-01	7.8E-01
BRCA	ZNF182_ES_4_3_5	ZNF182	ES	4	3	5	2.8E-20	0.105	0.089	9.1E-02	1.5E-01	5.4E-01
BRCA	ZNF185_ES_11_10_12	ZNF185	ES	11	10	12	4.8E-24	0.116	0.020	9.3E-01	5.8E-01	9.9E-01
BRCA	ZNF185_ES_11:12_10_13	ZNF185	ES	11:12	10	13	3.7E-66	0.272	0.021	6.7E-01	9.3E-01	8.8E-01
BRCA	ZNF207_ES_10_9_11	ZNF207	ES	10	9	11	5.5E-34	0.135	-0.025	5.6E-02	3.6E-01	3.6E-01
BRCA	ZNF276_RI_12.2_12.1_12.3	ZNF276	RI	12.2	12.1	12.3	6.4E-25	0.101	0.021	7.1E-01	3.1E-01	7.5E-01
BRCA	ZNF283_ES_2_1_3.2	ZNF283	ES	2	1	3.2	3.2E-26	0.117	-0.009	3.3E-01	3.2E-02	7.8E-01
BRCA	ZNF384_ES_5_4_6.1	ZNF384	ES	5	4	6.1	1.3E-31	0.128	-0.207	8.1E-01	4.6E-01	5.1E-01
BRCA	ZNF384_ES_8_7_9	ZNF384	ES	8	7	9	1.1E-63	0.239	-0.119	4.4E-01	1.9E-01	6.3E-01

cancer type	id	Gene Symbol	splice_type	Exon	From.Exon	To.Exon	anova.p	adj.r2	r	p.50	p.25	p.10
BRCA	ZNF410_ES_12_11_13	ZNF410	ES	12	11	13	2.7E-33	0.132	0.122	8.4E-01	4.9E-01	5.4E-01
BRCA	ZNF552_AD_1.2_1.1_2	ZNF552	AD	1.2	1.1	2	1.5E-48	0.190	-0.452	3.1E-01	2.0E-01	9.4E-02
BRCA	ZNF605_ES_2_1_3	ZNF605	ES	2	1	3	7.6E-43	0.183	-0.101	5.0E-01	2.8E-01	5.6E-01
BRCA	ZNF664_ES_2.2:4_1.4_5	ZNF664	ES	2.2:4	1.4	5	4.7E-20	0.101	0.045	3.7E-01	1.7E-01	1.7E-01
BRCA	ZNF670_ES_7_6_8	ZNF670	ES	7	6	8	5.2E-53	0.215	-0.172	8.1E-01	5.5E-01	2.8E-01
BRCA	ZNF791_ES_2.1:2.2_1_3	ZNF791	ES	2.1:2.2	1	3	3.4E-26	0.115	-0.499	7.7E-02	5.7E-01	1.7E-01
BRCA	ZNF821_ES_4_3.3_6	ZNF821	ES	4	3.3	6	6.2E-54	0.215	-0.049	7.0E-02	9.3E-02	3.4E-01
BRCA	ZSWIM7_RI_7.2_7.1_7.3	ZSWIM7	RI	7.2	7.1	7.3	6.8E-59	0.222	0.153	8.6E-01	7.2E-01	3.1E-01
COAD	ABLIM1_AA_18.1_17_18.2	ABLIM1	AA	18.1	17	18.2	9.4E-14	0.176	0.070	3.6E-01	5.8E-01	5.0E-01
COAD	AFMID_ES_7_6_8	AFMID	ES	7	6	8	5.7E-12	0.159	-0.095	6.1E-01	4.6E-01	9.9E-01
COAD	AFMID_ES_7:8:9:10:11.1_6_12	AFMID	ES	7:8:9:10:11.1	6	12	9.9E-13	0.155	-0.056	1.9E-01	3.1E-02	1.9E-01
COAD	AFMID_ES_9:10:11.1_8_12	AFMID	ES	10:11.1	8	12	4.2E-09	0.119	-0.101	9.2E-01	6.9E-01	5.7E-02
COAD	ALDOC_ES_2_1_3	ALDOC	ES	2	1	3	4.4E-11	0.141	-0.146	9.4E-01	3.0E-01	6.5E-01
COAD	ALG8_ES_14_13_15	ALG8	ES	14	13	15	8.7E-16	0.194	0.144	9.8E-01	3.2E-01	2.9E-01
COAD	ANKRD49_AD_2.3:2.4:2.5:2.6_2.2_2.8	ANKRD49	AD	2.3:2.4:2.5:2.6	2.2	2.8	1.2E-09	0.122	0.460	8.3E-02	3.4E-01	6.3E-02
COAD	ASCC2_ES_3_1_5	ASCC2	ES	3	1	5	1.0E-09	0.128	0.163	7.4E-01	5.3E-01	6.9E-01
COAD	C16orf13_ES_2_1_3	C16orf13	ES	2	1	3	9.1E-13	0.154	-0.046	7.9E-01	6.6E-01	6.6E-01
COAD	C7orf43_AA_9.1_8_9.2	C7orf43	AA	9.1	8	9.2	3.0E-08	0.101	0.075	1.1E-01	5.2E-01	1.7E-01
COAD	CAMKK2_ES_15_14_16	CAMKK2	ES	15	14	16	3.8E-09	0.110	0.073	7.4E-01	5.0E-01	5.2E-01
COAD	CAPN15_ES_3_2_4	CAPN15	ES	3	2	4	1.0E-11	0.152	0.027	5.1E-01	6.6E-01	2.8E-01
COAD	CCDC112_ES_10_9_11	CCDC112	ES	10	9	11	4.2E-27	0.325	-0.030	4.7E-01	7.9E-01	3.2E-01
COAD	CCNDBP1_ES_10.1_9_10.3	CCNDBP1	ES	10.1	9	10.3	7.6E-82	0.675	0.188	3.5E-01	8.3E-01	3.1E-01
COAD	CCNDBP1_RI_10.2_10.1_10.3	CCNDBP1	RI	10.2	10.1	10.3	4.9E-15	0.213	-0.330	3.8E-01	4.9E-01	8.1E-01
COAD	CDCA8_RI_1.2_1.1_1.3	CDCA8	RI	1.2	1.1	1.3	1.1E-08	0.105	-0.102	4.4E-01	1.3E-01	8.3E-01
COAD	CELF2_RI_16.2_16.1_16.3	CELF2	RI	16.2	16.1	16.3	7.8E-12	0.147	-0.559	7.1E-01	5.9E-02	6.5E-02
COAD	CEP57_AD_10.2:10.3_10.1_11	CEP57	AD	10.2:10.3	10.1	11	1.1E-11	0.143	-0.048	5.4E-01	7.3E-01	7.4E-01
COAD	CEP78_ES_15_14_16.1	CEP78	ES	15	14	16.1	1.8E-20	0.258	-0.255	4.9E-01	8.3E-02	2.1E-01
COAD	CIRBP_AD_9.4:9.5:9.6_9.3_9.8	CIRBP	AD	9.4:9.5:9.6	9.3	9.8	1.2E-08	0.106	0.245	5.6E-02	2.5E-01	8.2E-01
COAD	CPNE1_AA_2.1_1.2_2.2	CPNE1	AA	2.1	1.2	2.2	2.7E-08	0.102	-0.533	2.4E-02	7.8E-01	5.4E-01
COAD	CPNE1_ES_2.1:2.2:3_1.2_5	CPNE1	ES	2.1:2.2:3	1.2	5	1.1E-13	0.165	-0.821	5.9E-01	7.2E-01	1.8E-01
COAD	CTDSPL_ES_4_3_5	CTDSPL	ES	4	3	5	8.5E-11	0.137	-0.017	8.8E-01	9.1E-01	1.7E-01
COAD	D2HGDH_ES_10:11.1_9_11.2	D2HGDH	ES	10:11.1	9	11.2	4.5E-09	0.109	0.457	3.4E-01	6.5E-01	8.4E-01
COAD	DAPK2_ES_13.3:13.4:14:15.1:15.2_13.1_	DAPK2	ES	.3:13.4:14:15.1:1	13.1	15.3	9.7E-11	0.140	0.243	9.1E-01	5.6E-01	9.3E-01
COAD	DCUN1D4_AD_9.2:9.3_9.1_10	DCUN1D4	AD	9.2:9.3	9.1	10	1.3E-14	0.188	0.079	4.2E-01	2.7E-01	9.5E-01
COAD	DHRS12_ES_3_2_4	DHRS12	ES	3	2	4	3.3E-09	0.116	-0.193	2.6E-01	2.8E-01	9.8E-01
COAD	DMTF1_AA_13.2_12_13.3	DMTF1	AA	13.2	12	13.3	3.4E-07	0.102	0.497	3.9E-01	3.0E-01	3.9E-01
COAD	DNM2_ES_15_14_17	DNM2	ES	15	14	17	2.1E-10	0.125	-0.085	3.1E-01	6.1E-01	3.8E-01
COAD	DPM1_ES_7:8_6_9	DPM1	ES	7:08	6	9	9.1E-11	0.152	0.206	5.3E-01	8.9E-01	9.1E-01
COAD	ECHDC2_ES_5.1:6.1_2.1_6.2	ECHDC2	ES	5.1:6.1	2.1	6.2	1.6E-10	0.132	0.063	1.1E-01	6.1E-01	3.5E-01
COAD	ECT2_ES_5_4.2_6	ECT2	ES	5	4.2	6	2.8E-15	0.199	0.129	5.4E-01	4.0E-01	3.5E-01

cancer type	id	Gene Symbol	splice_type	Exon	From.Exon	To.Exon	anova.p	adj.r2	r	p.50	p.25	p.10
COAD	EXOSC9_ES_10.3_10.1_10.5	EXOSC9	ES	10.3	10.1	10.5	7.0E-12	0.151	0.249	7.9E-01	6.8E-01	9.8E-01
COAD	EZH2_ES_13_12_14.1	EZH2	ES	13	12	14.1	2.6E-11	0.141	-0.135	2.0E-01	1.3E-02	9.1E-02
COAD	FAM189B_ES_2:3:4_1_5	FAM189B	ES	2:03:04	1	5	1.3E-12	0.152	0.180	7.5E-01	8.0E-01	3.0E-01
COAD	FASTK_RI_5.6_5.5_5.7	FASTK	RI	5.6	5.5	5.7	2.6E-11	0.136	0.228	2.1E-02	3.8E-02	5.5E-02
COAD	GMPR2_AD_1.2_1.1_2.3	GMPR2	AD	1.2	1.1	2.3	5.1E-14	0.171	-0.075	4.4E-02	3.5E-01	1.5E-01
COAD	HNRNPA2B1_ES_2_1_3	HNRNPA2B1	ES	2	1	3	1.8E-20	0.239	0.121	3.7E-01	7.3E-01	5.1E-01
COAD	IL18BP_RI_1.7_1.6_1.8	IL18BP	RI	1.7	1.6	1.8	1.6E-07	0.105	-0.457	4.8E-02	2.7E-01	9.5E-01
COAD	IL32_ES_1.5:1.8_1.4_1.9	IL32	ES	1.5:1.8	1.4	1.9	7.6E-10	0.122	0.450	5.5E-01	5.8E-01	4.7E-01
COAD	IL32_RI_2.2:2.3_2.1_2.4	IL32	RI	2.2:2.3	2.1	2.4	1.9E-09	0.113	0.243	5.7E-02	1.4E-01	5.3E-01
COAD	INO80C_ES_3:4.1:4.2:5.1:5.2:6:7_1_8	INO80C	ES	:4.1:4.2:5.1:5.2:6	1	8	4.0E-16	0.210	-0.495	6.1E-01	8.0E-01	1.2E-01
COAD	INO80C_ES_3:4.2:5.1:5.2:6:7_1_8	INO80C	ES	3:4.2:5.1:5.2:6:7	1	8	2.5E-10	0.146	-0.034	4.0E-01	1.4E-01	4.4E-01
COAD	INO80E_ES_6.2:6.3:10_5_11	INO80E	ES	6.2:6.3:10	5	11	7.8E-10	0.127	-0.040	9.3E-01	5.3E-01	9.8E-01
COAD	IRAK1_ES_10.1:10.3_9_11.1	IRAK1	ES	10.1:10.3	9	11.1	4.7E-08	0.108	-0.054	1.8E-01	4.5E-02	5.5E-01
COAD	IST1_ES_12:13_11_14.1	IST1	ES	12:13	11	14.1	4.7E-08	0.110	0.019	7.2E-02	6.2E-01	7.3E-01
COAD	ITGAE_ES_28_27_29	ITGAE	ES	28	27	29	4.4E-09	0.111	0.345	5.5E-01	9.3E-01	7.5E-01
COAD	IVNS1ABP_ES_9_8_10	IVNS1ABP	ES	9	8	10	5.1E-12	0.145	-0.278	2.2E-01	1.6E-01	4.0E-01
COAD	KLC1_ES_13.3:15:16_13.2_18	KLC1	ES	13.3:15:16	13.2	18	2.7E-09	0.127	0.045	2.7E-01	8.8E-01	7.8E-01
COAD	LPAR2_AA_5.1_2.2_5.2	LPAR2	AA	5.1	2.2	5.2	6.4E-08	0.110	-0.085	3.2E-01	8.8E-01	5.1E-01
COAD	LRR1_ES_4_3_5	LRR1	ES	4	3	5	1.9E-18	0.251	0.000	1.2E-01	9.0E-01	6.1E-01
COAD	LRR1_ES_4:5_3_7	LRR1	ES	4:05	3	7	1.7E-09	0.121	-0.079	3.2E-01	3.0E-01	2.8E-02
COAD	LUC7L_ES_1.3:1.4_1.1_2.2	LUC7L	ES	1.3:1.4	1.1	2.2	2.5E-10	0.131	0.343	9.6E-01	5.5E-01	3.2E-01
COAD	LUC7L_ES_4_3_5	LUC7L	ES	4	3	5	4.7E-11	0.140	0.333	1.5E-01	2.4E-02	9.8E-02
COAD	MACROD1_ES_10_9_11	MACROD1	ES	10	9	11	6.3E-09	0.112	-0.104	1.9E-01	2.8E-01	8.1E-01
COAD	MFF_ES_8:9_7_10	MFF	ES	8:09	7	10	5.1E-09	0.109	-0.278	6.3E-01	1.8E-01	3.0E-01
COAD	MFF_ES_8:9:10_7_11	MFF	ES	8:09:10	7	11	5.8E-18	0.212	-0.329	4.7E-01	6.5E-01	4.4E-01
COAD	MFF_ES_9:10_7_11	MFF	ES	9:10	7	11	1.9E-14	0.173	-0.244	2.0E-01	8.7E-01	7.2E-01
COAD	MFSD9_AD_1.2_1.1_2.1	MFSD9	AD	1.2	1.1	2.1	2.2E-08	0.119	0.014	7.6E-01	4.3E-01	3.5E-01
COAD	MOGAT3_RI_6.2_6.1_6.3	MOGAT3	RI	6.2	6.1	6.3	1.2E-08	0.115	0.005	9.9E-01	9.2E-01	7.9E-01
COAD	MORF4L2_ES_4_3.2_5.1	MORF4L2	ES	4	3.2	5.1	2.2E-09	0.113	-0.087	7.8E-01	9.6E-01	7.4E-01
COAD	MRPL55_ES_1.2:2.2:2.5:2.6_1.1_2.9	MRPL55	ES	1.2:2.2:2.5:2.6	1.1	2.9	2.0E-07	0.103	0.086	7.2E-01	6.6E-01	4.3E-01
COAD	MRPL55_ES_2.2:2.5:2.6_1.2_2.9	MRPL55	ES	2.2:2.5:2.6	1.2	2.9	4.8E-08	0.106	0.075	5.1E-01	3.6E-01	3.5E-01
COAD	MRRF_ES_5_4_6	MRRF	ES	5	4	6	4.6E-11	0.145	-0.037	3.4E-01	9.1E-02	4.9E-01
COAD	MSI2_AA_18.1_17_18.2	MSI2	AA	18.1	17	18.2	1.1E-08	0.107	0.035	6.9E-01	7.4E-01	7.7E-01
COAD	MTCH1_AD_8.2_8.1_9	MTCH1	AD	8.2	8.1	9	2.6E-14	0.171	-0.022	8.8E-01	2.4E-01	5.3E-01
COAD	NAA60_RI_10.4:10.5_10.3_10.6	NAA60	RI	10.4:10.5	10.3	10.6	2.0E-10	0.125	-0.072	6.3E-01	9.0E-01	4.4E-01
COAD	NABP2_RI_1.2:1.3_1.1_1.4	NABP2	RI	1.2:1.3	1.1	1.4	1.1E-08	0.104	0.015	1.7E-01	3.3E-01	7.2E-01
COAD	NADK2_ES_10_9_11	NADK2	ES	10	9	11	9.5E-17	0.210	0.046	2.0E-01	2.9E-01	4.4E-01
COAD	NDUFAF6_ES_10_9.2_11	NDUFAF6	ES	10	9.2	11	6.3E-08	0.108	-0.174	3.3E-01	8.1E-01	6.6E-01
COAD	NDUFAF6_ES_9.2:10_9.1_11	NDUFAF6	ES	9.2:10	9.1	11	5.8E-10	0.128	-0.101	1.8E-02	4.6E-02	7.7E-01
COAD	NPRL3_ES_2:3_1.4_4	NPRL3	ES	2:03	1.4	4	2.1E-10	0.149	-0.109	9.8E-01	6.3E-01	2.0E-01

cancer type	id	Gene Symbol	splice_type	Exon	From.Exon	To.Exon	anova.p	adj.r2	r	p.50	p.25	p.10
COAD	NPRL3_ES_3_2_4	NPRL3	ES	3	2	4	1.2E-09	0.120	-0.148	7.9E-01	9.6E-01	5.6E-01
COAD	NSUN5_ES_2:3.1:3.2_1_5	NSUN5	ES	2:3.1:3.2	1	5	3.3E-09	0.131	-0.294	2.8E-01	1.3E-01	2.5E-01
COAD	NT5C_AD_3.3_3.2_4.1	NT5C	AD	3.3	3.2	4.1	8.0E-09	0.106	-0.092	7.8E-01	7.0E-01	4.4E-01
COAD	NVL_ES_12_10_13	NVL	ES	12	10	13	5.1E-09	0.127	-0.117	8.3E-01	2.9E-01	5.4E-01
COAD	OPA1_ES_7_6_8	OPA1	ES	7	6	8	5.1E-15	0.189	-0.172	9.7E-01	9.9E-02	1.7E-01
COAD	ORC3_ES_3_2_4	ORC3	ES	3	2	4	3.7E-07	0.101	-0.311	9.1E-01	8.7E-01	4.4E-01
COAD	PAPOLA_ES_20_18_21	PAPOLA	ES	20	18	21	1.7E-18	0.252	-0.049	2.8E-01	9.4E-01	9.9E-01
COAD	PARP9_AD_4.2_4.1_5	PARP9	AD	4.2	4.1	5	1.3E-07	0.102	0.181	6.5E-01	7.6E-01	8.1E-01
COAD	PFDN5_ES_2:5_1_6.2	PFDN5	ES	2:05	1	6.2	5.0E-08	0.102	0.055	7.4E-01	8.8E-01	6.2E-01
COAD	PIGO_RI_9.2_9.1_9.3	PIGO	RI	9.2	9.1	9.3	1.0E-09	0.116	0.260	2.7E-02	2.2E-01	7.8E-01
COAD	PQBP1_AD_1.2_1.1_2.3	PQBP1	AD	1.2	1.1	2.3	1.4E-16	0.203	-0.226	4.5E-01	8.1E-01	1.8E-01
COAD	PQBP1_AD_1.2:1.3_1.1_2.3	PQBP1	AD	1.2:1.3	1.1	2.3	1.7E-10	0.127	-0.198	5.5E-01	9.4E-01	4.9E-01
COAD	PRAP1_AA_4.1_3_4.2	PRAP1	AA	4.1	3	4.2	2.7E-13	0.187	-0.814	9.9E-01	3.6E-01	2.2E-01
COAD	PRAP1_AD_4.3_4.2_5	PRAP1	AD	4.3	4.2	5	1.8E-28	0.356	0.776	3.9E-01	4.5E-01	5.0E-01
COAD	PRKDC_ES_81_80_82	PRKDC	ES	81	80	82	1.2E-15	0.186	-0.473	8.4E-01	8.3E-01	8.9E-01
COAD	PTPRO_RI_26.2_26.1_26.3	PTPRO	RI	26.2	26.1	26.3	5.6E-08	0.101	0.198	1.0E+00	7.1E-01	2.0E-01
COAD	QTRT1_RI_6.2_6.1_6.3	QTRT1	RI	6.2	6.1	6.3	8.2E-10	0.118	0.335	1.1E-01	5.2E-02	2.6E-01
COAD	RAB11FIP3_ES_7_6_8	RAB11FIP3	ES	7	6	8	7.4E-11	0.133	-0.115	7.9E-01	8.7E-01	1.6E-01
COAD	RAC1_ES_4_3_5	RAC1	ES	4	3	5	2.0E-12	0.149	0.160	4.8E-01	7.5E-01	3.7E-01
COAD	RBM39_ES_4:5_3_6	RBM39	ES	4:05	3	6	3.9E-08	0.109	0.695	2.0E-01	5.3E-01	4.0E-01
COAD	RBM42_ES_5:6.1_4_6.2	RBM42	ES	05:06.1	4	6.2	7.2E-15	0.178	-0.382	8.6E-01	9.8E-01	2.4E-01
COAD	RBM6_RI_14.2_14.1_14.3	RBM6	RI	14.2	14.1	14.3	4.1E-10	0.123	0.661	5.9E-03	6.8E-03	6.7E-02
COAD	RFC5_ES_2.1:2.2_1_3	RFC5	ES	2.1:2.2	1	3	9.8E-15	0.184	-0.335	2.0E-01	3.7E-01	4.5E-01
COAD	RFWD2_ES_9_8_10	RFWD2	ES	9	8	10	5.9E-14	0.171	-0.374	9.0E-01	7.9E-01	6.7E-01
COAD	RPL22L1_AA_3.1_2.2_3.2	RPL22L1	AA	3.1	2.2	3.2	8.9E-25	0.290	-0.831	4.6E-01	8.6E-01	6.5E-01
COAD	RPL37A_ES_6:7_4_8	RPL37A	ES	6:07	4	8	6.2E-12	0.164	-0.202	7.0E-01	2.0E-01	6.2E-01
COAD	RPLP0_ES_5.3_4.2_6.2	RPLP0	ES	5.3	4.2	6.2	3.9E-21	0.247	0.070	5.7E-03	5.3E-02	7.0E-01
COAD	SCRIB_ES_17_16_18	SCRIB	ES	17	16	18	3.4E-11	0.136	-0.427	7.8E-01	1.6E-01	1.3E-01
COAD	SEPN1_ES_3_2_4	SEPN1	ES	3	2	4	3.1E-12	0.151	-0.221	1.8E-01	1.3E-01	1.0E-01
COAD	SEPT2_ES_3:4_2_7	SEPT2	ES	3:04	2	7	3.3E-24	0.279	-0.277	3.0E-01	9.3E-01	8.0E-01
COAD	SH2B1_AD_9.2_9.1_10	SH2B1	AD	9.2	9.1	10	1.2E-09	0.117	0.187	8.8E-01	6.1E-01	6.9E-01
COAD	SLC22A18AS_ES_3_2_5	SLC22A18AS	ES	3	2	5	4.6E-17	0.221	-0.101	6.5E-01	9.7E-01	3.1E-01
COAD	SLC7A6_ES_3_2.2_4	SLC7A6	ES	3	2.2	4	7.3E-08	0.105	-0.434	4.5E-01	4.9E-01	6.3E-01
COAD	SMAD5_ES_2_1_3	SMAD5	ES	2	1	3	1.8E-13	0.194	0.057	7.2E-01	8.7E-01	5.1E-01
COAD	SMPD4_ES_11_10_14	SMPD4	ES	11	10	14	5.8E-17	0.202	0.090	5.5E-01	2.5E-01	9.9E-01
COAD	SNUPN_AD_1.2_1.1_4	SNUPN	AD	1.2	1.1	4	6.2E-13	0.166	0.045	2.2E-01	4.1E-01	8.2E-01
COAD	SNX27_AA_12.1_11_12.2	SNX27	AA	12.1	11	12.2	1.8E-11	0.144	0.068	1.6E-01	1.6E-01	6.9E-02
COAD	SPHK2_RI_3.2_3.1_3.3	SPHK2	RI	3.2	3.1	3.3	2.4E-09	0.127	-0.374	7.8E-01	9.4E-01	6.1E-01
COAD	SRP9_ES_3_2_5	SRP9	ES	3	2	5	1.2E-23	0.271	-0.072	2.3E-01	1.6E-01	6.1E-01
COAD	SRP9_ME_3|4_2_5	SRP9	ME	3|4	2	5	1.2E-09	0.116	0.105	3.4E-01	9.7E-01	9.4E-01

cancer type	id	Gene Symbol	splice_type	Exon	From.Exon	To.Exon	anova.p	adj.r2	r	p.50	p.25	p.10
COAD	SRSF6_ES_3_2_4	SRSF6	ES	3	2	4	1.4E-18	0.219	0.467	7.3E-02	1.5E-01	1.6E-01
COAD	SUPT4H1_AD_2.4_2.3_3.2	SUPT4H1	AD	2.4	2.3	3.2	2.2E-08	0.104	-0.279	6.4E-01	9.3E-01	8.0E-01
COAD	TAF1C_RI_8.2_8.1_8.3	TAF1C	RI	8.2	8.1	8.3	1.6E-12	0.157	0.472	5.3E-01	2.3E-01	8.1E-01
COAD	TATDN1_AD_4.2_4.1_5	TATDN1	AD	4.2	4.1	5	1.6E-14	0.176	-0.224	7.8E-01	9.8E-01	5.3E-01
COAD	TCF3_RI_19.2_19.1_19.3	TCF3	RI	19.2	19.1	19.3	2.9E-24	0.278	-0.011	6.4E-01	4.8E-01	6.5E-01
COAD	TIAL1_ES_6_5.1_7	TIAL1	ES	6	5.1	7	1.0E-08	0.108	0.164	2.5E-01	4.5E-01	7.2E-01
COAD	TMUB2_AA_4.2_3_4.3	TMUB2	AA	4.2	3	4.3	1.5E-29	0.361	0.270	6.1E-01	3.5E-01	3.6E-02
COAD	TMUB2_AD_2.2:2.3:2.4:2.5_2.1_4.3	TMUB2	AD	2.2:2.3:2.4:2.5	2.1	4.3	1.7E-12	0.161	-0.120	8.3E-01	8.2E-01	7.1E-01
COAD	TMUB2_AD_2.3:2.4:2.5_2.2_4.3	TMUB2	AD	2.3:2.4:2.5	2.2	4.3	1.2E-29	0.343	-0.189	8.6E-01	7.8E-01	7.4E-01
COAD	TMUB2_ES_2.2:2.3:2.4:2.5:3_2.1_4.3	TMUB2	ES	2.2:2.3:2.4:2.5:3	2.1	4.3	2.5E-08	0.121	-0.177	1.4E-01	2.5E-01	5.1E-01
COAD	TMUB2_ES_2.3:2.4:2.5:3_2.2_4.3	TMUB2	ES	2.3:2.4:2.5:3	2.2	4.3	9.3E-28	0.339	-0.267	4.3E-01	2.1E-01	9.1E-02
COAD	TMUB2_ES_3:4.2_2.5_4.3	TMUB2	ES	03:04.2	2.5	4.3	4.6E-23	0.277	0.180	2.6E-01	3.4E-01	3.2E-01
COAD	TNFRSF10B_RI_5.2_5.1_5.3	TNFRSF10B	RI	5.2	5.1	5.3	3.0E-11	0.135	-0.117	7.4E-01	1.5E-01	1.3E-01
COAD	TPD52L1_ES_8:9.1_6_10	TPD52L1	ES	08:09.1	6	10	2.5E-09	0.115	-0.188	4.0E-01	4.0E-01	8.6E-01
COAD	TRPM2_ES_29_28_30	TRPM2	ES	29	28	30	2.3E-10	0.135	-0.524	7.9E-01	1.4E-01	1.0E+00
COAD	TSPAN17_RI_7.5_7.4_7.6	TSPAN17	RI	7.5	7.4	7.6	5.6E-09	0.108	0.038	8.1E-01	4.1E-01	2.9E-01
COAD	TTC8_ES_10.1_9_11	TTC8	ES	10.1	9	11	3.9E-15	0.192	0.094	1.4E-01	9.2E-02	4.6E-01
COAD	UAP1_ES_9.2_8_10	UAP1	ES	9.2	8	10	3.6E-10	0.125	-0.011	2.1E-01	4.6E-01	7.2E-01
COAD	WDR6_AA_4.1_1_4.2	WDR6	AA	4.1	1	4.2	2.8E-09	0.112	-0.104	2.8E-01	6.4E-01	9.4E-01
COAD	WDR62_RI_25.6:25.7_25.5_25.8	WDR62	RI	25.6:25.7	25.5	25.8	7.5E-11	0.149	-0.166	7.5E-01	4.2E-01	2.2E-01
COAD	WWP2_ES_2.2:3_1.1_4.3	WWP2	ES	2.2:3	1.1	4.3	1.0E-07	0.109	0.125	6.4E-01	9.7E-01	5.2E-01
COAD	ZDHHC20_ES_14_13.2_15	ZDHHC20	ES	14	13.2	15	3.3E-12	0.154	0.042	3.9E-01	1.0E-01	8.5E-02
COAD	ZNF384_ES_8_7_9	ZNF384	ES	8	7	9	5.8E-16	0.191	-0.020	7.2E-01	2.7E-01	3.0E-01
COAD	ZNF692_RI_6.3_6.2_6.4	ZNF692	RI	6.3	6.2	6.4	1.6E-09	0.116	0.359	8.0E-01	1.2E-01	5.4E-01
ESCA	AASS_ES_20_19_21	AASS	ES	20	19	21	3.4E-08	0.208	-0.124	4.7E-01	5.6E-01	9.8E-01
ESCA	ABCC3_RI_16.2_16.1_16.3	ABCC3	RI	16.2	16.1	16.3	6.4E-07	0.164	-0.702	1.5E-01	7.9E-01	4.4E-01
ESCA	ABCD4_ES_4.1:4.2_3_5	ABCD4	ES	4.1:4.2	3	5	7.3E-07	0.164	0.192	6.0E-01	9.3E-01	7.2E-01
ESCA	ABI1_ES_11.1:11.2:12_9_13	ABI1	ES	11.1:11.2:12	9	13	2.8E-12	0.284	-0.063	3.3E-03	7.8E-03	4.4E-03
ESCA	ABI1_ES_11.2:12_9_13	ABI1	ES	11.2:12	9	13	6.1E-15	0.337	-0.065	3.9E-04	1.8E-02	9.5E-02
ESCA	ABI1_ES_12_11.2_13	ABI1	ES	12	11.2	13	4.3E-16	0.358	0.095	2.8E-02	3.1E-02	8.5E-02
ESCA	ABI1_ES_12_9_13	ABI1	ES	12	9	13	3.3E-14	0.324	0.216	1.1E-02	5.8E-02	5.9E-01
ESCA	ABI2_ES_15_14_16	ABI2	ES	15	14	16	1.0E-05	0.132	0.103	7.9E-01	7.0E-01	2.7E-01
ESCA	ABLIM2_ES_12_11_13	ABLIM2	ES	12	11	13	5.5E-10	0.258	0.173	9.1E-01	9.9E-01	8.0E-01
ESCA	ACAD10_ES_6_5_7	ACAD10	ES	6	5	7	1.8E-04	0.100	0.045	1.2E-01	5.3E-01	6.1E-01
ESCA	ACAP1_AD_21.3_21.2_22.2	ACAP1	AD	21.3	21.2	22.2	7.7E-05	0.117	0.108	7.7E-01	9.9E-01	5.7E-01
ESCA	ACHE_AA_6.1_5_6.2	ACHE	AA	6.1	5	6.2	7.1E-05	0.113	0.192	1.3E-01	3.3E-01	3.6E-01
ESCA	ACOT9_ES_5.1_4_7.1	ACOT9	ES	5.1	4	7.1	1.4E-05	0.129	0.054	8.5E-01	6.9E-01	8.4E-01
ESCA	ACOT9_ES_5.1:6_4_7.1	ACOT9	ES	5.1:6	4	7.1	1.0E-14	0.332	-0.187	4.0E-01	4.5E-01	1.0E+00
ESCA	ACOT9_ES_6_5.1_7.1	ACOT9	ES	6	5.1	7.1	8.1E-37	0.647	-0.119	3.7E-01	3.3E-01	8.0E-01
ESCA	ACOX1_ES_4_3_5	ACOX1	ES	4	3	5	7.3E-05	0.117	-0.192	4.2E-01	3.0E-01	7.4E-01

cancer type	id	Gene Symbol	splice_type	Exon	From.Exon	To.Exon	anova.p	adj.r2	r	p.50	p.25	p.10
ESCA	ACP1_ES_4.3:4.4:5_4.2_6	ACP1	ES	4.3:4.4:5	4.2	6	5.7E-06	0.150	-0.177	4.8E-01	8.7E-01	1.2E-01
ESCA	ACSF2_ES_5.1:5.2_4_6.1	ACSF2	ES	5.1:5.2	4	6.1	4.9E-19	0.415	0.272	1.8E-01	3.4E-01	1.1E-01
ESCA	ACSL1_ME_16|17_15_18.1	ACSL1	ME	16|17	15	18.1	4.9E-13	0.299	-0.486	5.7E-01	8.2E-01	9.8E-01
ESCA	ACSL4_AA_4.1_2_4.2	ACSL4	AA	4.1	2	4.2	3.0E-06	0.153	-0.036	4.5E-01	6.9E-01	6.0E-01
ESCA	ACSL4_ES_3:4.1_2_4.2	ACSL4	ES	03:04.1	2	4.2	1.9E-04	0.118	-0.376	NA	NA	NA
ESCA	ADAM15_ES_21.1:21.2_20_22.1	ADAM15	ES	21.1:21.2	20	22.1	2.5E-19	0.416	0.085	2.3E-01	7.3E-01	7.5E-01
ESCA	ADAM15_ES_21.1:21.2:22.1_20_23	ADAM15	ES	21.1:21.2:22.1	20	23	2.4E-10	0.242	0.370	6.2E-01	9.6E-01	8.7E-01
ESCA	ADAM15_ES_21.1:21.2:22.1:22.2_20_23	ADAM15	ES	1.1:21.2:22.1:22.	20	23	2.4E-08	0.197	0.124	6.6E-01	4.4E-01	7.1E-01
ESCA	ADAM15_ES_21.2_20_22.1	ADAM15	ES	21.2	20	22.1	8.0E-18	0.390	0.044	5.5E-01	9.2E-02	2.0E-01
ESCA	ADAM15_ES_21.2:22.1_20_23	ADAM15	ES	21.2:22.1	20	23	1.2E-09	0.226	0.354	9.7E-01	8.8E-01	9.4E-01
ESCA	ADAM15_ES_21.2:22.1:22.2_20_23	ADAM15	ES	21.2:22.1:22.2	20	23	4.3E-08	0.191	0.105	3.0E-01	6.8E-01	5.9E-01
ESCA	ADAM15_ES_22.1_21.2_23	ADAM15	ES	22.1	21.2	23	2.7E-10	0.241	0.225	6.3E-02	3.5E-01	1.1E-01
ESCA	ADAM15_ES_22.1:22.2_21.2_23	ADAM15	ES	22.1:22.2	21.2	23	1.4E-14	0.330	-0.088	3.7E-01	7.7E-01	9.2E-01
ESCA	ADD3_ES_15_14_16	ADD3	ES	15	14	16	9.3E-20	0.423	0.569	2.8E-01	4.7E-01	7.3E-02
ESCA	ADSSL1_AA_6.1_5_6.2	ADSSL1	AA	6.1	5	6.2	8.7E-06	0.147	-0.027	1.1E-01	1.4E-01	1.3E-01
ESCA	AFMID_ES_5:6:7:8:9:10:11.1:11.2:12_2_	AFMID	ES	7:8:9:10:11.1:11.	2	13	4.5E-06	0.144	0.032	5.3E-03	2.1E-02	5.9E-01
ESCA	AFMID_ES_5:6:7:8:9:10:11.1:12_2_13	AFMID	ES	:6:7:8:9:10:11.1:1	2	13	7.6E-05	0.110	0.027	9.1E-03	3.8E-03	4.4E-01
ESCA	AFMID_ES_5:6:8:9:10:11.1:11.2:12_2_1	AFMID	ES	:8:9:10:11.1:11.2	2	13	5.3E-06	0.149	0.125	5.6E-02	3.9E-02	5.5E-01
ESCA	AFMID_ES_5:6:8:9:10:11.1:12_2_13	AFMID	ES	5:6:8:9:10:11.1:1	2	13	1.5E-05	0.134	0.087	2.9E-02	1.7E-01	9.7E-01
ESCA	AFTPH_ES_7:8_6_9	AFTPH	ES	7:08	6	9	3.8E-06	0.145	0.411	7.5E-01	9.6E-01	5.4E-01
ESCA	AHI1_ES_31:32_30.1_33	AHI1	ES	31:32:00	30.1	33	8.5E-06	0.137	0.284	3.2E-01	9.6E-01	5.6E-01
ESCA	AIMP2_ES_1.2:2_1.1_4	AIMP2	ES	1.2:2	1.1	4	2.7E-05	0.122	-0.364	3.3E-01	9.9E-01	6.6E-01
ESCA	AKAP13_ES_12_11_14	AKAP13	ES	12	11	14	1.6E-28	0.554	-0.281	2.2E-01	8.7E-01	5.7E-02
ESCA	AKAP13_ES_12:13_11_14	AKAP13	ES	12:13	11	14	4.5E-24	0.522	-0.340	6.9E-01	7.6E-01	2.0E-01
ESCA	AKAP13_ME_12|13_11_14	AKAP13	ME	12|13	11	14	2.9E-05	0.122	-0.108	6.2E-01	8.8E-01	7.9E-01
ESCA	AKAP9_ES_5_4_6	AKAP9	ES	5	4	6	3.0E-18	0.434	-0.066	1.8E-01	2.2E-01	5.9E-01
ESCA	ALDH18A1_AD_6.2_6.1_7	ALDH18A1	AD	6.2	6.1	7	2.8E-25	0.508	-0.485	4.6E-01	9.2E-01	1.0E-01
ESCA	ALDH3A1_RI_2.2_2.1_2.3	ALDH3A1	RI	2.2	2.1	2.3	2.7E-04	0.113	0.019	6.3E-01	5.1E-01	4.5E-01
ESCA	ALG3_ES_6_5_8	ALG3	ES	6	5	8	2.0E-06	0.176	0.074	5.8E-02	2.6E-01	7.0E-01
ESCA	AMACR_ES_3_2_4.1	AMACR	ES	3	2	4.1	1.2E-09	0.227	0.287	5.1E-01	7.9E-01	9.7E-01
ESCA	ANK3_ES_27_26_29.1	ANK3	ES	27	26	29.1	6.7E-18	0.426	-0.128	3.7E-01	9.2E-01	5.9E-01
ESCA	ANK3_ME_26|27_25_29.1	ANK3	ME	26|27	25	29.1	2.1E-20	0.463	0.040	4.1E-01	9.5E-01	6.7E-01
ESCA	ANKMY1_ES_18_17_19	ANKMY1	ES	18	17	19	4.8E-04	0.110	0.377	9.3E-02	2.1E-02	3.6E-03
ESCA	ANKRD11_ES_5.3:6_3_7	ANKRD11	ES	5.3:6	3	7	4.5E-04	0.106	0.097	6.4E-01	1.8E-01	5.2E-01
ESCA	ANKRD46_AD_1.2_1.1_2.2	ANKRD46	AD	1.2	1.1	2.2	1.6E-04	0.101	0.013	5.3E-01	3.6E-01	6.4E-01
ESCA	ANKRD49_RI_2.3:2.4:2.5:2.6:2.7_2.2_2.	ANKRD49	RI	2.3:2.4:2.5:2.6:2.	2.2	2.8	1.1E-07	0.181	0.467	3.0E-02	1.3E-01	1.3E-02
ESCA	ANKRD49_RI_2.5:2.6:2.7_2.4_2.8	ANKRD49	RI	2.5:2.6:2.7	2.4	2.8	1.8E-05	0.126	0.408	6.6E-01	7.0E-01	7.0E-01
ESCA	ANKRD65_ES_1.4:2_1.3_3	ANKRD65	ES	1.4:2	1.3	3	2.8E-05	0.143	-0.567	5.0E-02	8.5E-02	1.5E-01
ESCA	ANKRD65_ES_2_1.4_3	ANKRD65	ES	2	1.4	3	5.5E-10	0.264	-0.565	5.1E-01	3.1E-01	5.5E-02
ESCA	AP1G1_ES_12_10.1_13.2	AP1G1	ES	12	10.1	13.2	2.9E-15	0.343	-0.146	2.0E-01	3.5E-01	2.2E-01

cancer type	id	Gene Symbol	splice_type	Exon	From.Exon	To.Exon	anova.p	adj.r2	r	p.50	p.25	p.10
ESCA	AP1S2_AA_5.1_4_5.2	AP1S2	AA	5.1	4	5.2	6.6E-05	0.111	-0.034	6.1E-01	6.9E-01	6.5E-01
ESCA	AP4B1_RI_1.2_1.1_1.3	AP4B1	RI	1.2	1.1	1.3	2.0E-06	0.161	0.230	5.9E-01	6.6E-01	1.0E+00
ESCA	APBB2_ES_8_7.2_9	APBB2	ES	8	7.2	9	4.0E-07	0.169	-0.085	4.7E-01	9.7E-01	7.9E-01
ESCA	APH1A_ES_2.2_1_3	APH1A	ES	2.2	1	3	2.6E-05	0.123	0.052	5.0E-01	6.0E-01	2.7E-01
ESCA	APH1A_ES_2.2:3_1_4	APH1A	ES	2.2:3	1	4	6.8E-12	0.294	0.177	9.9E-01	7.8E-01	8.4E-01
ESCA	APOL3_ES_4.1:4.2:4.3_3.3_5	APOL3	ES	4.1:4.2:4.3	3.3	5	1.9E-05	0.139	-0.179	3.3E-01	3.0E-01	3.9E-01
ESCA	APOO_AA_9.1_8_9.2	APOO	AA	9.1	8	9.2	5.5E-06	0.139	-0.037	8.3E-02	4.3E-01	4.4E-01
ESCA	ARAP1_ES_32_31_33	ARAP1	ES	32	31	33	7.6E-16	0.354	0.180	3.5E-01	6.5E-01	5.4E-01
ESCA	ARFGAP1_ES_13_12_14.1	ARFGAP1	ES	13	12	14.1	4.6E-06	0.141	0.321	2.6E-01	9.5E-01	3.5E-01
ESCA	ARFRP1_RI_8.2_8.1_8.3	ARFRP1	RI	8.2	8.1	8.3	1.2E-09	0.226	0.051	3.4E-01	2.0E-01	2.3E-01
ESCA	ARHGAP17_ES_18_17_19	ARHGAP17	ES	18	17	19	4.2E-55	0.791	0.300	3.2E-01	5.7E-01	2.9E-01
ESCA	ARHGAP21_ES_24:25_23_26	ARHGAP21	ES	24:25:00	23	26	2.4E-11	0.288	0.201	5.2E-01	8.7E-01	5.5E-01
ESCA	ARHGAP44_ES_21_20_22	ARHGAP44	ES	21	20	22	8.7E-06	0.173	-0.413	4.5E-01	9.5E-01	9.0E-01
ESCA	ARHGEF10L_ES_18_17_19	ARHGEF10L	ES	18	17	19	6.6E-05	0.111	0.525	1.0E+00	6.6E-01	5.3E-01
ESCA	ARHGEF12_ES_5_4_6	ARHGEF12	ES	5	4	6	4.1E-25	0.505	-0.222	5.5E-01	8.5E-01	2.8E-01
ESCA	ARHGEF4_RI_14.2_14.1_14.3	ARHGEF4	RI	14.2	14.1	14.3	2.0E-04	0.103	-0.309	7.6E-01	8.0E-01	3.4E-01
ESCA	ARID1B_ES_12_11_13	ARID1B	ES	12	11	13	6.8E-30	0.569	0.102	4.9E-03	3.6E-01	1.0E+00
ESCA	ARL16_ES_4_3_5	ARL16	ES	4	3	5	1.9E-05	0.125	0.044	3.0E-01	6.1E-01	8.3E-01
ESCA	ARNT_ES_7_6_8	ARNT	ES	7	6	8	9.5E-14	0.313	-0.110	5.9E-01	3.9E-01	4.7E-01
ESCA	ARPP19_ES_2.3:2.4:2.5:2.6:4_2.2_5.2	ARPP19	ES	2.3:2.4:2.5:2.6:4	2.2	5.2	1.3E-05	0.130	0.157	1.2E-01	2.6E-01	6.4E-01
ESCA	ARRB1_ES_13_12_15.2	ARRB1	ES	13	12	15.2	6.0E-05	0.113	-0.226	7.6E-01	9.8E-01	4.5E-01
ESCA	ARSA_RI_1.3:1.4_1.2_1.5	ARSA	RI	1.3:1.4	1.2	1.5	2.3E-05	0.123	-0.117	6.3E-01	9.1E-01	6.0E-01
ESCA	ARVCF_ES_20_19_21	ARVCF	ES	20	19	21	1.3E-36	0.656	-0.019	7.5E-01	7.9E-01	7.4E-01
ESCA	ASS1_ME_2|3_1_4	ASS1	ME	2|3	1	4	3.8E-10	0.289	-0.158	5.5E-02	5.1E-01	9.3E-01
ESCA	ATG16L1_ES_8_7_10	ATG16L1	ES	8	7	10	3.9E-08	0.192	-0.441	1.3E-01	3.0E-01	5.8E-01
ESCA	ATG4B_ES_5_3_6	ATG4B	ES	5	3	6	4.8E-05	0.115	0.106	8.2E-01	3.5E-01	5.7E-02
ESCA	ATL2_ES_15:16:17.1_14_17.2	ATL2	ES	16:17.1	14	17.2	4.5E-07	0.185	-0.229	7.9E-01	9.1E-01	5.0E-01
ESCA	ATL2_ES_16:17.1_15_17.2	ATL2	ES	16:17.1	15	17.2	6.7E-06	0.137	-0.233	5.6E-01	2.6E-01	9.7E-02
ESCA	ATL2_ES_3:5_1_6	ATL2	ES	3:05	1	6	1.0E-04	0.109	0.154	9.9E-01	4.4E-01	6.1E-01
ESCA	ATP5A1_ES_4.2:5_3.2_6.1	ATP5A1	ES	4.2:5	3.2	6.1	3.0E-05	0.138	-0.136	8.8E-01	6.7E-01	9.2E-01
ESCA	ATP5H_ES_5_3_6	ATP5H	ES	5	3	6	2.8E-04	0.100	-0.310	1.7E-01	3.2E-01	7.9E-01
ESCA	ATP9B_ES_30_29_31.1	ATP9B	ES	30	29	31.1	1.4E-11	0.269	0.067	7.9E-01	4.3E-01	5.4E-01
ESCA	ATXN2_ES_24:25_23_26	ATXN2	ES	24:25:00	23	26	1.2E-05	0.131	0.157	1.8E-01	2.7E-01	4.2E-01
ESCA	ATXN2_ES_25_23_26	ATXN2	ES	25	23	26	3.7E-11	0.260	0.113	6.3E-01	8.9E-01	6.0E-01
ESCA	ATXN2L_AA_22.6_22.1_22.7	ATXN2L	AA	22.6	22.1	22.7	1.6E-04	0.101	0.110	4.7E-01	2.4E-01	1.0E-01
ESCA	ATXN2L_AD_22.2:22.3:22.4_22.1_22.6	ATXN2L	AD	22.2:22.3:22.4	22.1	22.6	4.5E-05	0.115	-0.145	3.5E-01	2.6E-01	9.8E-01
ESCA	ATXN2L_ES_22.2:22.4:22.5_22.1_22.6	ATXN2L	ES	22.2:22.4:22.5	22.1	22.6	8.5E-06	0.134	-0.241	2.4E-01	1.5E-01	4.2E-02
ESCA	AURKA_ES_1.2:1.3:2:3_1.1_4	AURKA	ES	1.2:1.3:2:3	1.1	4	7.1E-05	0.111	0.116	3.4E-01	3.4E-02	9.9E-01
ESCA	AURKA_ES_1.2:2:3_1.1_4	AURKA	ES	1.2:2:3	1.1	4	4.3E-05	0.121	0.109	4.4E-02	4.7E-01	7.1E-01
ESCA	AURKA_ES_1.3:2:3_1.2_4	AURKA	ES	1.3:2:3	1.2	4	1.2E-05	0.134	0.140	1.4E-01	4.8E-02	2.5E-01

cancer type	id	Gene Symbol	splice_type	Exon	From.Exon	To.Exon	anova.p	adj.r2	r	p.50	p.25	p.10
ESCA	AURKA_ES_2:3_1.1_4	AURKA	ES	2:03	1.1	4	4.2E-07	0.170	0.137	9.4E-02	7.4E-01	8.5E-01
ESCA	AURKA_ES_2:3_1.2_4	AURKA	ES	2:03	1.2	4	4.5E-06	0.150	0.052	8.3E-01	9.3E-01	6.3E-01
ESCA	AXIN1_ES_9_8_10	AXIN1	ES	9	8	10	1.4E-04	0.103	0.108	1.1E-01	5.0E-01	5.0E-01
ESCA	B3GNT4_RI_2.2_2.1_2.3	B3GNT4	RI	2.2	2.1	2.3	5.5E-06	0.161	-0.413	8.5E-01	4.4E-01	6.2E-01
ESCA	B4GALT3_AA_3.1_2.2_3.2	B4GALT3	AA	3.1	2.2	3.2	2.9E-05	0.120	-0.059	1.8E-01	7.2E-01	4.9E-01
ESCA	BABAM1_AA_2.1_1.2_2.2	BABAM1	AA	2.1	1.2	2.2	1.1E-08	0.204	0.050	6.1E-01	7.2E-03	7.2E-02
ESCA	BAIAP2_RI_16.2_16.1_16.3	BAIAP2	RI	16.2	16.1	16.3	6.2E-06	0.138	-0.421	3.0E-02	1.0E-01	6.7E-01
ESCA	BCAR1_ES_9:10.1:10.2_8.2_11	BCAR1	ES	9:10.1:10.2	8.2	11	3.2E-15	0.355	0.284	9.3E-01	4.9E-01	6.5E-01
ESCA	BCAT2_ES_3:4:5_1_6	BCAT2	ES	3:04:05	1	6	6.5E-07	0.162	-0.018	2.6E-01	1.8E-01	6.8E-01
ESCA	BCAT2_ES_4:5_1_6	BCAT2	ES	4:05	1	6	5.6E-14	0.318	0.026	2.7E-01	8.0E-01	1.5E-01
ESCA	BCL2L12_ES_3.2_2_4	BCL2L12	ES	3.2	2	4	1.3E-05	0.131	0.047	9.7E-01	7.2E-01	1.4E-02
ESCA	BICD2_RI_7.2_7.1_7.3	BICD2	RI	7.2	7.1	7.3	4.2E-28	0.546	-0.736	8.8E-01	7.8E-01	1.1E-01
ESCA	BIN1_ES_7_6_8	BIN1	ES	7	6	8	1.1E-18	0.404	0.447	7.6E-01	6.6E-01	8.7E-01
ESCA	BMP1_ES_17.1:17.2_16_18.1	BMP1	ES	17.1:17.2	16	18.1	1.6E-09	0.224	-0.402	7.0E-01	4.1E-01	2.4E-01
ESCA	BOD1L1_ES_2_1_3	BOD1L1	ES	2	1	3	1.3E-06	0.154	-0.349	8.7E-01	5.6E-01	5.2E-01
ESCA	BPTF_ES_5:6_4_7	BPTF	ES	5:06	4	7	3.5E-07	0.169	-0.036	1.8E-01	2.4E-01	1.3E-01
ESCA	BPTF_ES_6_4_7	BPTF	ES	6	4	7	8.1E-05	0.110	0.166	1.4E-01	7.2E-01	9.7E-01
ESCA	BRD9_AD_2.2:2.3_2.1_3	BRD9	AD	2.2:2.3	2.1	3	1.1E-08	0.240	-0.250	5.0E-01	9.3E-01	3.2E-01
ESCA	BTN2A1_ES_4_3_5	BTN2A1	ES	4	3	5	3.5E-16	0.364	-0.273	1.1E-01	1.3E-01	1.6E-01
ESCA	C12orf76_AA_10.1_8_10.2	C12orf76	AA	10.1	8	10.2	1.4E-05	0.132	-0.243	6.9E-01	9.3E-01	4.7E-01
ESCA	C14orf79_ES_2.4_2.2_3.2	C14orf79	ES	2.4	2.2	3.2	8.9E-05	0.111	0.016	5.8E-01	8.6E-01	9.1E-01
ESCA	C14orf80_ES_8:9_7_10	C14orf80	ES	8:09	7	10	1.2E-04	0.104	0.017	2.4E-01	4.9E-01	6.3E-01
ESCA	C19orf57_ES_8_7_9	C19orf57	ES	8	7	9	1.6E-05	0.145	0.115	4.4E-01	7.2E-01	2.9E-01
ESCA	C1D_ES_2.2_1.1_3.1	C1D	ES	2.2	1.1	3.1	9.1E-06	0.133	0.102	1.1E-01	1.3E-02	3.8E-03
ESCA	C1orf63_AA_6.1_4.2_6.2	C1orf63	AA	6.1	4.2	6.2	2.0E-06	0.153	0.104	4.4E-01	9.2E-01	6.4E-02
ESCA	C1orf86_ES_6:7.1:8_5_10	C1orf86	ES	07:01.1	5	10	4.7E-06	0.158	-0.140	8.8E-01	7.1E-01	3.5E-01
ESCA	C1orf86_ES_6:7.1:8:9_5_10	C1orf86	ES	6:7.1:8:9	5	10	5.4E-07	0.175	-0.239	6.9E-01	2.1E-01	9.6E-01
ESCA	C22orf29_RI_1.2:1.3_1.1_1.4	C22orf29	RI	1.2:1.3	1.1	1.4	3.1E-08	0.195	-0.088	8.3E-01	8.7E-01	5.1E-01
ESCA	C2ORF15_ES_3_2_4	C2ORF15	ES	3	2	4	2.8E-04	0.108	-0.338	7.4E-01	5.8E-01	4.4E-01
ESCA	C6orf203_ES_1.2:2_1.1_3	C6orf203	ES	1.2:2	1.1	3	3.0E-05	0.120	0.087	4.5E-01	5.5E-01	5.2E-01
ESCA	C9orf3_ES_19.1_18_20	C9orf3	ES	19.1	18	20	9.9E-10	0.228	-0.394	1.5E-01	1.4E-01	2.0E-01
ESCA	C9orf89_RI_3.2_3.1_3.3	C9orf89	RI	3.2	3.1	3.3	1.1E-04	0.105	-0.251	6.5E-01	1.0E+00	7.8E-01
ESCA	CACNB3_ES_4_3_5	CACNB3	ES	4	3	5	2.0E-04	0.100	-0.069	1.6E-01	2.4E-01	3.0E-01
ESCA	CADPS2_ES_25_24.1_27	CADPS2	ES	25	24.1	27	3.3E-07	0.182	-0.261	2.5E-01	6.8E-01	9.9E-01
ESCA	CALCOCO1_RI_14.3_14.2_14.4	CALCOCO1	RI	14.3	14.2	14.4	1.1E-05	0.132	0.086	9.8E-01	5.9E-01	1.2E-01
ESCA	CALML4_ES_2_1_3	CALML4	ES	2	1	3	9.5E-08	0.215	-0.415	4.3E-01	8.3E-01	4.4E-01
ESCA	CALML4_ES_3_2_4	CALML4	ES	3	2	4	1.0E-05	0.132	0.247	5.2E-01	2.3E-01	9.2E-01
ESCA	CAMKK2_ES_17_16_19.1	CAMKK2	ES	17	16	19.1	7.9E-06	0.135	0.084	4.1E-01	1.1E-01	1.7E-01
ESCA	CANT1_ES_2_1_3.1	CANT1	ES	2	1	3.1	1.9E-06	0.151	0.441	8.5E-01	4.6E-01	1.9E-01
ESCA	CANT1_RI_3.2_3.1_3.3	CANT1	RI	3.2	3.1	3.3	6.5E-07	0.162	-0.418	6.0E-01	6.0E-01	2.4E-01

cancer type	id	Gene Symbol	splice_type	Exon	From.Exon	To.Exon	anova.p	adj.r2	r	p.50	p.25	p.10
ESCA	CASK_ES_20_19.1_22	CASK	ES	20	19.1	22	9.8E-08	0.183	0.069	5.4E-01	8.9E-01	1.2E-01
ESCA	CASK_ES_21_19.1_22	CASK	ES	21	19.1	22	2.6E-11	0.263	-0.324	5.7E-02	9.9E-01	8.9E-01
ESCA	CASK_ES_21_20_22	CASK	ES	21	20	22	7.3E-13	0.297	-0.332	8.1E-01	7.7E-01	5.5E-02
ESCA	CASP10_ES_7:8_5_9	CASP10	ES	7:08	5	9	7.3E-06	0.137	0.369	3.0E-01	6.7E-02	3.5E-01
ESCA	CBLB_ES_18.4_18.2_19	CBLB	ES	18.4	18.2	19	1.2E-05	0.130	0.216	5.5E-01	1.9E-01	6.1E-01
ESCA	CC2D2A_ES_5_4_6	CC2D2A	ES	5	4	6	3.3E-14	0.343	0.247	8.3E-01	5.3E-01	7.1E-01
ESCA	CCDC106_RI_1.2_1.1_1.3	CCDC106	RI	1.2	1.1	1.3	1.8E-04	0.101	-0.227	4.9E-01	2.7E-01	9.3E-02
ESCA	CCDC112_ES_10_9_11	CCDC112	ES	10	9	11	1.0E-06	0.157	0.082	5.0E-02	1.9E-01	1.5E-02
ESCA	CCDC138_ES_14_13_15	CCDC138	ES	14	13	15	3.8E-05	0.118	0.103	7.2E-01	2.6E-01	3.3E-01
ESCA	CCDC142_AA_4.1_3_4.2	CCDC142	AA	4.1	3	4.2	8.5E-05	0.108	-0.183	6.2E-01	4.7E-01	5.1E-01
ESCA	CCDC18_AA_12.1_11_12.2	CCDC18	AA	12.1	11	12.2	1.2E-04	0.124	-0.040	6.8E-01	5.5E-01	9.4E-01
ESCA	CCDC50_ES_6_5_7	CCDC50	ES	6	5	7	1.6E-12	0.289	0.052	4.9E-01	7.9E-01	4.6E-01
ESCA	CCDC92_AA_2.2:2.3_1_2.4	CCDC92	AA	2.2:2.3	1	2.4	1.7E-08	0.200	0.055	9.9E-01	5.4E-01	1.4E-01
ESCA	CCT4_ES_6:7:8:9:10:11_5_14	CCT4	ES	6:7:8:9:10:11	5	14	1.1E-05	0.157	-0.329	8.4E-01	9.8E-01	3.4E-01
ESCA	CCT7_ES_3:5.2:6:7_1_8	CCT7	ES	3:5.2:6:7	1	8	3.9E-05	0.117	0.167	8.0E-01	2.9E-01	9.5E-01
ESCA	CD200_ES_3_1_4	CD200	ES	3	1	4	2.2E-04	0.122	-0.034	9.8E-01	4.4E-01	6.4E-01
ESCA	CD44_ES_10:11_5_12.1	CD44	ES	10:11	5	12.1	8.9E-26	0.515	0.517	7.7E-01	2.7E-01	8.8E-01
ESCA	CD44_ES_12.1:13:14_5_15	CD44	ES	12.1:13:14	5	15	3.9E-15	0.340	-0.202	9.9E-01	5.5E-01	1.9E-01
ESCA	CD44_ES_3.1:3.2:4:5:6:7:8:9.1:9.2:10:11	CD44	ES	).2:10:11:12.1:13	2.1	17.2	1.1E-10	0.251	0.371	9.1E-01	4.2E-01	5.4E-01
ESCA	CD44_ES_3.1:3.2:4:5:6:7:8:9.2:10:11:12.	CD44	ES	2:10:11:12.1:13:1	2.1	17.2	7.6E-13	0.295	0.449	7.4E-01	7.7E-01	8.9E-01
ESCA	CD44_ES_3.1:3.2:4:5:7:8:9.2:10:11:12.1:	CD44	ES	:10:11:12.1:13:14	2.1	17.2	1.8E-05	0.126	0.302	2.7E-01	3.4E-01	1.6E-01
ESCA	CD44_ES_6:7:8:9.1:9.2_5_10	CD44	ES	6:7:8:9.1:9.2	5	10	5.8E-23	0.480	0.521	5.0E-01	8.1E-01	3.7E-01
ESCA	CD44_ES_6:7:8:9.1:9.2:10:11_5_12.1	CD44	ES	;:7:8:9.1:9.2:10:1	5	12.1	1.3E-36	0.645	0.615	8.1E-01	4.7E-01	3.2E-01
ESCA	CD44_ES_6:7:8:9.1:9.2:10:11:12.1:13_5_	CD44	ES	:9.1:9.2:10:11:12	5	14	8.3E-06	0.135	0.384	8.0E-01	4.1E-01	8.5E-01
ESCA	CD44_ES_6:7:8:9.1:9.2:10:11:12.1:13:14	CD44	ES	).1:9.2:10:11:12.1	5	15	7.6E-22	0.456	0.675	7.9E-01	9.8E-01	6.1E-01
ESCA	CD44_ES_6:7:8:9.2_5_10	CD44	ES	6:7:8:9.2	5	10	9.3E-24	0.490	0.537	7.6E-01	7.3E-01	6.6E-01
ESCA	CD44_ES_6:7:8:9.2:10:11_5_12.1	CD44	ES	6:7:8:9.2:10:11	5	12.1	2.7E-35	0.631	0.568	5.0E-01	9.6E-01	1.2E-01
ESCA	CD44_ES_6:7:8:9.2:10:11:12.1:13_5_14	CD44	ES	:8:9.2:10:11:12.1	5	14	5.9E-07	0.163	0.427	6.0E-01	5.5E-01	9.7E-01
ESCA	CD44_ES_6:7:8:9.2:10:11:12.1:13:14_5_	CD44	ES	3:9.2:10:11:12.1:1	5	15	2.0E-22	0.465	0.683	8.4E-01	6.0E-01	7.4E-01
ESCA	CD44_ES_7:12.1:13:14_5_15	CD44	ES	7:12.1:13:14	5	15	2.1E-12	0.302	-0.197	8.8E-01	4.2E-01	6.0E-01
ESCA	CD44_ES_7:8:9.1:9.2_5_10	CD44	ES	7:8:9.1:9.2	5	10	6.2E-34	0.618	0.604	7.1E-01	9.3E-01	1.3E-01
ESCA	CD44_ES_7:8:9.1:9.2:10:11_5_12.1	CD44	ES	7:8:9.1:9.2:10:11	5	12.1	6.2E-56	0.796	0.651	7.3E-01	5.3E-01	6.8E-01
ESCA	CD44_ES_7:8:9.1:9.2:10:11:12.1:13:14_5	CD44	ES	1:9.2:10:11:12.1:	5	15	4.1E-25	0.505	0.718	3.7E-01	4.5E-01	2.3E-01
ESCA	CD44_ES_7:8:9.2_5_10	CD44	ES	08:09.2	5	10	4.3E-36	0.641	0.613	9.6E-01	9.0E-01	8.6E-01
ESCA	CD44_ES_7:8:9.2:10:11_5_12.1	CD44	ES	7:8:9.2:10:11	5	12.1	6.9E-57	0.801	0.615	8.3E-01	6.3E-01	9.5E-01
ESCA	CD44_ES_7:8:9.2:10:11:12.1:13:14_5_15	CD44	ES	9.2:10:11:12.1:13	5	15	2.4E-26	0.523	0.727	5.3E-01	6.2E-01	4.6E-01
ESCA	CD44_ES_8:9.1:9.2_5_10	CD44	ES	8:9.1:9.2	5	10	9.4E-31	0.615	0.584	3.2E-01	4.3E-01	9.1E-01
ESCA	CD44_ES_8:9.1:9.2:10:11_5_12.1	CD44	ES	8:9.1:9.2:10:11	5	12.1	4.9E-31	0.621	0.605	2.6E-01	7.8E-01	6.4E-01
ESCA	CD44_ES_8:9.1:9.2:10:11:12.1:13:14_5_	CD44	ES	.:9.2:10:11:12.1:1	5	15	5.0E-28	0.580	0.704	3.5E-01	8.8E-01	9.7E-01
ESCA	CD44_ES_8:9.2_5_10	CD44	ES	08:09.2	5	10	4.9E-28	0.581	0.585	3.5E-01	4.1E-01	5.6E-01

cancer type	id	Gene Symbol	splice_type	Exon	From.Exon	To.Exon	anova.p	adj.r2	r	p.50	p.25	p.10
ESCA	CD44_ES_8:9.2:10:11_5_12.1	CD44	ES	8:9.2:10:11	5	12.1	4.9E-28	0.583	0.598	2.1E-01	5.3E-01	4.5E-01
ESCA	CD44_ES_8:9.2:10:11:12.1:13:14_5_15	CD44	ES	).2:10:11:12.1:13	5	15	7.4E-26	0.550	0.699	5.9E-01	9.6E-01	9.1E-01
ESCA	CD46_ES_13_12_14	CD46	ES	13	12	14	7.5E-15	0.335	-0.268	3.1E-01	1.6E-01	6.3E-01
ESCA	CD47_ES_8:9:10_7_11	CD47	ES	8:09:10	7	11	1.3E-15	0.360	-0.099	9.8E-01	5.6E-01	7.4E-01
ESCA	CD47_ES_9:10_8_11	CD47	ES	9:10	8	11	5.0E-39	0.668	0.026	3.7E-01	4.9E-01	3.5E-01
ESCA	CDC25C_ES_4_3.2_5	CDC25C	ES	4	3.2	5	1.2E-06	0.156	0.008	6.7E-01	5.9E-01	5.2E-01
ESCA	CDCA7_ES_3.1:3.2:3.3_2_4	CDCA7	ES	3.1:3.2:3.3	2	4	6.0E-10	0.233	0.269	5.8E-01	2.1E-01	5.4E-01
ESCA	CDK10_ES_2.2:4_2.1_5	CDK10	ES	2.2:4	2.1	5	1.3E-04	0.103	0.158	5.9E-01	9.9E-01	3.4E-01
ESCA	CDK10_ES_4_2.2_5	CDK10	ES	4	2.2	5	1.0E-04	0.107	0.170	7.6E-01	4.6E-01	3.9E-01
ESCA	CDK10_ES_4:5_2.2_6	CDK10	ES	4:05	2.2	6	2.6E-04	0.104	0.218	4.5E-01	3.8E-01	9.3E-01
ESCA	CDK18_AD_1.2_1.1_2.3	CDK18	AD	1.2	1.1	2.3	3.0E-11	0.270	0.070	2.8E-01	7.0E-01	7.2E-01
ESCA	CDKN2D_RI_1.2_1.1_1.3	CDKN2D	RI	1.2	1.1	1.3	2.4E-05	0.122	0.131	2.5E-01	1.3E-01	2.2E-01
ESCA	CEACAM1_ES_9_8_10	CEACAM1	ES	9	8	10	6.2E-10	0.238	0.034	3.9E-01	5.1E-01	6.2E-02
ESCA	CELF1_AA_12.1_11_12.2	CELF1	AA	12.1	11	12.2	8.8E-08	0.183	0.135	9.0E-01	4.7E-01	7.1E-01
ESCA	CELF2_RI_16.2_16.1_16.3	CELF2	RI	16.2	16.1	16.3	1.1E-04	0.106	-0.355	2.6E-01	5.3E-01	9.7E-02
ESCA	CENPA_ES_3.1:3.2_2_4	CENPA	ES	3.1:3.2	2	4	5.2E-05	0.125	-0.337	3.8E-01	5.9E-01	5.7E-01
ESCA	CENPE_ES_17_16_18	CENPE	ES	17	16	18	1.0E-07	0.186	0.103	9.3E-01	8.5E-01	6.2E-01
ESCA	CENPL_ES_5_4_6	CENPL	ES	5	4	6	5.0E-05	0.116	0.099	5.0E-02	2.7E-02	7.8E-01
ESCA	CEP170B_AA_13.1_12_13.2	CEP170B	AA	13.1	12	13.2	4.5E-24	0.490	-0.142	5.5E-01	4.0E-01	7.1E-01
ESCA	CES2_AD_1.2:1.3_1.1_2	CES2	AD	1.2:1.3	1.1	2	4.2E-07	0.167	-0.672	4.8E-01	7.8E-01	3.6E-01
ESCA	CGREF1_ES_2.2_1.1_3.2	CGREF1	ES	2.2	1.1	3.2	1.5E-05	0.138	-0.118	7.2E-01	4.7E-01	5.9E-01
ESCA	CHEK2_ME_6|7.1:7.2_5_9	CHEK2	ME	6|7.1:7.2	5	9	3.0E-05	0.120	0.556	4.7E-01	6.2E-01	8.6E-01
ESCA	CHST5_RI_3.2_3.1_3.3	CHST5	RI	3.2	3.1	3.3	1.8E-12	0.319	-0.648	8.8E-01	7.6E-01	6.2E-01
ESCA	CHTOP_ES_4.1:4.2:4.3:4.4_3.2_5	CHTOP	ES	4.1:4.2:4.3:4.4	3.2	5	2.3E-05	0.154	0.246	8.9E-01	3.2E-01	5.2E-01
ESCA	CHTOP_ES_4.2:4.3:4.4_3.2_5	CHTOP	ES	4.2:4.3:4.4	3.2	5	1.1E-05	0.157	0.314	9.6E-01	9.0E-01	4.3E-01
ESCA	CIDEB_ES_2_1_3	CIDEB	ES	2	1	3	1.1E-11	0.283	-0.391	8.1E-01	5.5E-01	4.4E-01
ESCA	CLCC1_ES_2_1_3.1	CLCC1	ES	2	1	3.1	8.9E-05	0.108	-0.134	2.9E-01	6.9E-01	3.9E-01
ESCA	CLCN5_AD_2.3_2.2_3	CLCN5	AD	2.3	2.2	3	4.4E-04	0.107	-0.223	7.9E-01	5.0E-01	2.1E-01
ESCA	CLSTN1_ES_11_10_12	CLSTN1	ES	11	10	12	1.3E-12	0.291	0.340	3.7E-01	8.9E-03	6.4E-01
ESCA	CMC2_ES_7:9_5_12	CMC2	ES	7:09	5	12	1.2E-04	0.108	-0.226	9.1E-01	3.3E-01	6.9E-01
ESCA	CMC2_ES_7:9:10_5_12	CMC2	ES	7:09:10	5	12	1.1E-05	0.138	-0.212	9.2E-01	4.2E-01	2.2E-01
ESCA	CNIH1_ES_4:5_3_6.1	CNIH1	ES	4:05	3	6.1	2.4E-10	0.268	0.061	3.8E-01	8.3E-02	9.4E-03
ESCA	CNIH2_RI_6.2_6.1_6.3	CNIH2	RI	6.2	6.1	6.3	4.2E-12	0.305	-0.501	8.1E-01	5.3E-01	8.5E-01
ESCA	COBL_ES_6_5_7	COBL	ES	6	5	7	1.7E-14	0.393	0.297	8.4E-01	5.0E-01	1.2E-01
ESCA	COCH_RI_1.2_1.1_1.3	COCH	RI	1.2	1.1	1.3	8.5E-05	0.134	0.192	6.7E-01	8.6E-01	6.7E-01
ESCA	COL1A1_ES_8:9:10:11:12:13:14:40:41:4	COL1A1	ES	?:13:14:40:41:42:	7	47	1.2E-04	0.107	0.074	9.6E-01	7.1E-01	2.4E-01
ESCA	COL4A5_ES_42:43_41_44	COL4A5	ES	42:43:00	41	44	2.4E-10	0.255	-0.396	6.2E-01	4.5E-01	9.0E-02
ESCA	COPS7A_ES_5:6_2.4_7	COPS7A	ES	5:06	2.4	7	6.4E-05	0.116	-0.272	7.0E-01	7.9E-01	4.7E-01
ESCA	CRAMP1L_ES_16_15_17	CRAMP1L	ES	16	15	17	5.9E-06	0.140	0.188	2.9E-02	5.3E-02	1.1E-01
ESCA	CRB3_RI_5.2_5.1_5.3	CRB3	RI	5.2	5.1	5.3	7.6E-10	0.232	0.108	4.2E-01	1.2E-01	3.9E-02

cancer type	id	Gene Symbol	splice_type	Exon	From.Exon	To.Exon	anova.p	adj.r2	r	p.50	p.25	p.10
ESCA	CSPP1_ES_7:8_6_9	CSPP1	ES	7:08	6	9	9.4E-21	0.467	0.083	4.1E-01	8.5E-01	2.6E-01
ESCA	CSPP1_ES_8_6_9	CSPP1	ES	8	6	9	1.2E-07	0.184	0.043	1.4E-01	3.4E-01	5.0E-01
ESCA	CTNND1_ES_21_20_22.1	CTNND1	ES	21	20	22.1	3.7E-46	0.730	-0.125	5.6E-01	8.0E-01	8.3E-01
ESCA	CTNND1_ES_21_20_22.2	CTNND1	ES	21	20	22.2	8.3E-31	0.650	-0.303	3.3E-01	1.9E-01	7.9E-01
ESCA	CTNND1_ES_21:22.1_20_22.2	CTNND1	ES	21:22.1	20	22.2	2.4E-40	0.681	-0.116	6.9E-01	8.2E-01	3.9E-01
ESCA	CTTN_ES_10:12_9_13	CTTN	ES	10:12	9	13	1.3E-05	0.135	-0.243	1.0E+00	5.0E-01	9.6E-01
ESCA	CXorf40A_AD_1.2:1.3_1.1_1.6	CXorf40A	AD	1.2:1.3	1.1	1.6	1.2E-07	0.196	0.040	4.6E-01	5.4E-01	5.6E-01
ESCA	CXorf40A_AD_1.2:1.3:1.4_1.1_1.6	CXorf40A	AD	1.2:1.3:1.4	1.1	1.6	2.4E-06	0.148	0.160	3.6E-01	3.5E-01	6.4E-01
ESCA	CXorf40A_RI_1.2:1.3:1.4:1.5_1.1_1.6	CXorf40A	RI	1.2:1.3:1.4:1.5	1.1	1.6	8.9E-05	0.111	-0.035	9.9E-01	8.8E-01	6.9E-01
ESCA	DCAF6_ME_11|12_10_13.1	DCAF6	ME	11|12	10	13.1	1.6E-06	0.154	0.284	6.1E-01	2.3E-01	6.9E-01
ESCA	DCAF8_AD_7.2_7.1_9	DCAF8	AD	7.2	7.1	9	3.9E-05	0.144	-0.060	8.2E-01	3.1E-01	3.2E-02
ESCA	DCAF8_AD_7.2:7.3_7.1_9	DCAF8	AD	7.2:7.3	7.1	9	1.6E-06	0.156	0.054	6.5E-01	6.6E-01	9.0E-01
ESCA	DCAF8_ES_7.2:7.3:8.1:8.2_7.1_9	DCAF8	ES	7.2:7.3:8.1:8.2	7.1	9	6.6E-06	0.137	0.155	3.0E-01	5.1E-01	4.0E-01
ESCA	DCN_ES_2_1_3	DCN	ES	2	1	3	7.7E-06	0.135	-0.304	6.7E-01	9.1E-01	4.2E-02
ESCA	DCTD_ES_1.2:2.1:2.2_1.1_5	DCTD	ES	1.2:2.1:2.2	1.1	5	4.0E-06	0.156	0.139	4.0E-01	9.5E-01	9.5E-01
ESCA	DCTN2_ES_8_2_10	DCTN2	ES	8	2	10	8.4E-08	0.184	-0.261	3.5E-01	9.8E-01	8.0E-01
ESCA	DCTN6_ES_3_2_4	DCTN6	ES	3	2	4	1.2E-04	0.104	-0.257	4.0E-01	2.0E-01	1.3E-02
ESCA	DDB2_ES_4:5:7_3_8	DDB2	ES	4:05:07	3	8	5.0E-05	0.118	-0.259	3.9E-01	1.9E-01	2.3E-01
ESCA	DDHD1_ES_13_12_14	DDHD1	ES	13	12	14	8.4E-06	0.138	-0.176	5.8E-01	8.3E-01	3.7E-01
ESCA	DDIT3_AA_2.3:2.4_2.1_2.5	DDIT3	AA	2.3:2.4	2.1	2.5	3.8E-05	0.131	0.128	9.9E-01	6.3E-01	3.6E-01
ESCA	DECR2_AA_7.1_6_7.2	DECR2	AA	7.1	6	7.2	3.0E-05	0.141	-0.385	5.8E-01	6.6E-01	8.9E-02
ESCA	DENND1B_ES_3_2_4	DENND1B	ES	3	2	4	9.5E-05	0.107	-0.314	9.8E-01	5.1E-01	7.0E-01
ESCA	DENND1B_ES_9_8_10	DENND1B	ES	9	8	10	1.7E-22	0.477	0.091	7.4E-01	4.3E-01	8.9E-01
ESCA	DEPDC1_ES_8_7_9	DEPDC1	ES	8	7	9	4.1E-12	0.280	-0.006	2.1E-01	8.6E-01	6.6E-01
ESCA	DEPDC5_AA_28.1_27.1_28.2	DEPDC5	AA	28.1	27.1	28.2	5.5E-09	0.211	-0.014	6.8E-01	1.3E-01	2.0E-02
ESCA	DGKH_ES_31_30_33	DGKH	ES	31	30	33	3.1E-08	0.208	-0.158	3.1E-01	5.5E-02	5.6E-03
ESCA	DHRS13_RI_1.2_1.1_1.3	DHRS13	RI	1.2	1.1	1.3	2.2E-13	0.319	0.125	3.7E-01	8.4E-01	3.8E-01
ESCA	DIAPH1_ES_2_1_3	DIAPH1	ES	2	1	3	2.0E-27	0.537	-0.163	5.7E-01	6.0E-01	1.1E-01
ESCA	DIAPH2_ES_2_1_3	DIAPH2	ES	2	1	3	5.7E-46	0.742	-0.171	1.1E-01	7.9E-01	6.8E-01
ESCA	DMKN_AD_20.2_20.1_21	DMKN	AD	20.2	20.1	21	3.5E-05	0.134	-0.426	8.5E-01	7.7E-01	8.4E-01
ESCA	DMKN_AD_20.2_20.1_22	DMKN	AD	20.2	20.1	22	1.0E-04	0.111	-0.360	1.5E-01	4.4E-01	7.6E-01
ESCA	DMKN_AD_6.2_6.1_6.4	DMKN	AD	6.2	6.1	6.4	1.5E-09	0.243	0.464	9.0E-01	7.8E-01	7.4E-01
ESCA	DMKN_ES_7_6.4_12	DMKN	ES	7	6.4	12	5.3E-06	0.144	0.379	6.1E-01	3.8E-01	2.7E-01
ESCA	DMKN_RI_6.2:6.3_6.1_6.4	DMKN	RI	6.2:6.3	6.1	6.4	4.9E-05	0.121	0.240	8.9E-01	9.7E-01	7.2E-01
ESCA	DMWD_ES_4_3_5	DMWD	ES	4	3	5	7.4E-08	0.185	0.221	8.5E-01	5.3E-01	4.1E-01
ESCA	DMXL1_ES_34_33_35	DMXL1	ES	34	33	35	9.7E-05	0.108	0.253	8.9E-01	4.0E-01	1.7E-01
ESCA	DNAAF2_ES_2_1_3	DNAAF2	ES	2	1	3	2.7E-05	0.121	0.014	2.7E-02	7.6E-02	8.0E-02
ESCA	DNAJC25_AA_2.1_1_2.2	DNAJC25	AA	2.1	1	2.2	2.2E-04	0.111	0.083	5.1E-02	5.6E-01	9.0E-01
ESCA	DNAJC4_RI_7.2_7.1_7.3	DNAJC4	RI	7.2	7.1	7.3	1.3E-04	0.103	0.142	2.8E-01	7.5E-01	9.8E-01
ESCA	DNASE1_AD_6.2_6.1_7	DNASE1	AD	6.2	6.1	7	4.4E-08	0.247	-0.509	7.9E-01	6.8E-01	4.8E-01

cancer type	id	Gene Symbol	splice_type	Exon	From.Exon	To.Exon	anova.p	adj.r2	r	p.50	p.25	p.10
ESCA	DNASE1_ES_6.1_5_7	DNASE1	ES	6.1	5	7	2.3E-11	0.268	0.617	2.1E-01	8.9E-01	9.0E-01
ESCA	DNASE1_RI_9.2_9.1_9.3	DNASE1	RI	9.2	9.1	9.3	4.2E-17	0.379	-0.684	7.1E-01	2.2E-01	9.3E-01
ESCA	DNM2_ES_15_14_17	DNM2	ES	15	14	17	3.1E-09	0.217	-0.275	8.9E-01	8.6E-01	3.9E-01
ESCA	DNMT3B_ES_22:23_21_24	DNMT3B	ES	22:23	21	24	2.2E-04	0.100	0.061	2.6E-01	4.9E-01	3.4E-01
ESCA	DOK1_ES_5_4_6	DOK1	ES	5	4	6	1.2E-05	0.132	-0.210	4.0E-02	2.4E-01	9.0E-02
ESCA	DPH2_ES_3.1:3.2_2.1_4	DPH2	ES	3.1:3.2	2.1	4	2.9E-05	0.122	-0.032	3.8E-01	8.5E-01	5.9E-01
ESCA	DPP8_ES_18_17_19	DPP8	ES	18	17	19	5.2E-05	0.114	-0.150	2.2E-01	1.0E-01	8.1E-01
ESCA	DPP8_ES_18:19_17_20	DPP8	ES	18:19	17	20	1.7E-04	0.101	-0.087	8.2E-01	5.3E-01	4.8E-01
ESCA	DPY19L4_ES_3_2_4	DPY19L4	ES	3	2	4	2.0E-05	0.125	-0.035	9.2E-01	2.5E-01	5.4E-01
ESCA	DRAM2_ES_6_4_7	DRAM2	ES	6	4	7	5.1E-06	0.159	0.293	9.4E-01	6.1E-01	8.9E-01
ESCA	DST_ES_104_103_105	DST	ES	104	103	105	4.4E-18	0.394	0.488	1.1E-01	6.4E-02	6.6E-01
ESCA	DST_ES_106_105_107	DST	ES	106	105	107	2.7E-19	0.415	0.348	5.9E-01	2.5E-01	5.6E-01
ESCA	DUOXA2_AA_2.1_1_2.2	DUOXA2	AA	2.1	1	2.2	6.0E-29	0.578	-0.660	5.3E-01	6.5E-01	5.2E-01
ESCA	DUSP22_RI_7.4_7.3_7.5	DUSP22	RI	7.4	7.3	7.5	4.1E-10	0.237	-0.233	4.7E-01	1.3E-01	6.3E-01
ESCA	DVL1_AD_11.2_11.1_12	DVL1	AD	11.2	11.1	12	7.6E-23	0.472	0.003	2.6E-01	9.1E-01	6.5E-01
ESCA	DYNC1I2_ES_7.3:8_4_9	DYNC1I2	ES	7.3:8	4	9	1.8E-12	0.310	-0.036	9.4E-02	8.2E-03	3.1E-02
ESCA	DYNC1I2_ES_8_7.3_9	DYNC1I2	ES	8	7.3	9	4.4E-18	0.394	0.067	5.4E-01	6.7E-01	8.7E-02
ESCA	DYSF_ES_19_18_20	DYSF	ES	19	18	20	3.6E-12	0.318	-0.050	3.7E-01	2.6E-01	7.6E-01
ESCA	DYSF_ES_7_6_8	DYSF	ES	7	6	8	8.3E-10	0.248	0.027	6.2E-01	8.3E-01	8.5E-01
ESCA	EDEM2_ES_10_9_14	EDEM2	ES	10	9	14	5.6E-04	0.104	0.183	4.2E-01	4.7E-01	3.2E-01
ESCA	EDEM2_ES_10:11:12:13_9_14	EDEM2	ES	10:11:12:13	9	14	1.7E-04	0.114	0.294	1.6E-01	5.0E-01	6.7E-01
ESCA	EED_ES_8:9:10_7_11.2	EED	ES	8:09:10	7	11.2	7.2E-05	0.111	0.323	6.5E-01	8.1E-01	8.0E-01
ESCA	EFCAB4A_ES_8_7_9	EFCAB4A	ES	8	7	9	2.6E-09	0.221	0.457	9.8E-01	6.6E-01	8.2E-01
ESCA	EHBP1_ES_18_17_19	EHBP1	ES	18	17	19	6.0E-15	0.337	-0.076	5.7E-02	1.0E+00	8.3E-02
ESCA	EIF1AD_AA_2.1:2.2_1.2_2.3	EIF1AD	AA	2.1:2.2	1.2	2.3	9.2E-05	0.111	-0.005	3.1E-01	1.6E-01	4.7E-01
ESCA	EIF1AD_AA_2.1:2.2:2.3_1.2_2.4	EIF1AD	AA	2.1:2.2:2.3	1.2	2.4	1.2E-06	0.164	0.069	7.4E-01	2.3E-01	8.4E-01
ESCA	EIF1AD_AD_1.2_1.1_2.1	EIF1AD	AD	1.2	1.1	2.1	4.3E-04	0.109	0.181	5.0E-01	2.3E-01	3.0E-01
ESCA	EIF4G1_AA_2.2:2.3_1_2.4	EIF4G1	AA	2.2:2.3	1	2.4	1.4E-04	0.104	-0.353	4.3E-01	4.1E-01	5.0E-01
ESCA	EIF4G1_ES_2.3:2.4:3.2_2.2_5	EIF4G1	ES	2.3:2.4:3.2	2.2	5	4.9E-06	0.142	-0.542	6.2E-01	7.6E-01	4.1E-01
ESCA	EIF4G1_ES_2.4_2.2_5	EIF4G1	ES	2.4	2.2	5	7.4E-05	0.119	-0.420	2.1E-01	3.8E-01	4.1E-01
ESCA	EIF4G1_ES_2.4:3.1:3.2_2.2_5	EIF4G1	ES	2.4:3.1:3.2	2.2	5	2.4E-05	0.122	-0.269	5.7E-01	8.0E-01	3.3E-01
ESCA	ELOVL6_RI_2.2_2.1_2.3	ELOVL6	RI	2.2	2.1	2.3	6.5E-07	0.165	-0.041	9.6E-01	5.3E-01	9.6E-01
ESCA	ELP5_RI_7.2_7.1_7.3	ELP5	RI	7.2	7.1	7.3	1.7E-04	0.100	-0.174	2.5E-01	3.7E-01	6.6E-01
ESCA	ELP5_RI_7.4_7.3_7.5	ELP5	RI	7.4	7.3	7.5	2.8E-08	0.195	-0.208	3.4E-01	6.6E-01	7.4E-01
ESCA	ENAH_ES_13_12_14	ENAH	ES	13	12	14	5.0E-09	0.213	-0.115	7.5E-01	7.8E-01	7.6E-01
ESCA	ENAH_ES_7.1:7.2_6_8	ENAH	ES	7.1:7.2	6	8	5.3E-07	0.174	-0.217	3.7E-01	3.6E-01	7.7E-01
ESCA	ENDOV_RI_2.2:2.3_2.1_2.4	ENDOV	RI	2.2:2.3	2.1	2.4	5.3E-05	0.119	-0.314	3.4E-01	5.4E-01	9.3E-01
ESCA	ENTPD6_ES_2_1_3.1	ENTPD6	ES	2	1	3.1	1.3E-14	0.330	0.336	6.0E-01	4.5E-01	8.2E-01
ESCA	ENTPD6_ES_3.1:3.2_1_4	ENTPD6	ES	3.1:3.2	1	4	8.3E-05	0.109	-0.078	4.3E-01	6.4E-01	7.5E-01
ESCA	EPB41_ES_16_15_19.1	EPB41	ES	16	15	19.1	1.9E-06	0.166	0.296	7.4E-01	7.6E-01	5.3E-01

cancer type	id	Gene Symbol	splice_type	Exon	From.Exon	To.Exon	anova.p	adj.r2	r	p.50	p.25	p.10
ESCA	EPB41_ES_17:18_16_19.1	EPB41	ES	17:18	16	19.1	5.3E-05	0.132	-0.284	1.7E-02	8.7E-01	9.2E-01
ESCA	EPB41_ES_18_16_19.1	EPB41	ES	18	16	19.1	3.0E-06	0.158	-0.320	1.3E-01	3.0E-01	5.7E-02
ESCA	EPB41L1_ES_20_19_21	EPB41L1	ES	20	19	21	1.8E-31	0.588	-0.418	1.1E-01	1.3E-01	6.9E-01
ESCA	EPB41L2_ES_17:18_14_20.1	EPB41L2	ES	17:18	14	20.1	1.5E-34	0.623	-0.439	3.1E-02	5.2E-02	7.8E-01
ESCA	EPN3_ES_4_3.2_5	EPN3	ES	4	3.2	5	1.5E-17	0.387	0.326	2.3E-01	7.6E-02	3.3E-01
ESCA	EPOR_RI_9.2_9.1_9.3	EPOR	RI	9.2	9.1	9.3	3.1E-10	0.249	-0.378	7.8E-01	2.7E-01	3.8E-01
ESCA	EPS8L1_ES_16_15.3_17	EPS8L1	ES	16	15.3	17	3.3E-07	0.170	0.123	1.5E-01	4.8E-01	6.8E-01
ESCA	ERBB2IP_ES_22_21_24.1	ERBB2IP	ES	22	21	24.1	2.4E-17	0.381	0.066	1.8E-01	5.7E-01	7.8E-01
ESCA	ERBB2IP_ES_22:24.1:24.2:24.3_21_25	ERBB2IP	ES	22:24.1:24.2:24.3	21	25	5.2E-17	0.377	0.133	3.5E-01	3.9E-01	5.6E-01
ESCA	ERBB2IP_ME_22|23_21_24.1	ERBB2IP	ME	22|23	21	24.1	8.3E-14	0.320	0.094	2.0E-01	3.2E-01	8.1E-01
ESCA	ERC1_ES_7_6_8	ERC1	ES	7	6	8	1.1E-06	0.157	0.153	1.2E-01	3.9E-01	9.1E-01
ESCA	ERGIC3_ME_10|11_8_12	ERGIC3	ME	10|11	8	12	2.4E-04	0.108	0.249	2.1E-01	7.7E-01	9.2E-01
ESCA	ESPN_RI_9.2_9.1_9.3	ESPN	RI	9.2	9.1	9.3	1.3E-24	0.518	-0.439	8.0E-01	2.1E-01	4.2E-01
ESCA	ETHE1_ES_1.2:2:3_1.1_4	ETHE1	ES	1.2:2:3	1.1	4	1.0E-04	0.127	-0.071	2.4E-01	8.9E-01	4.1E-01
ESCA	EVA1C_AD_6.2:6.3_6.1_7	EVA1C	AD	6.2:6.3	6.1	7	2.3E-06	0.168	-0.256	8.4E-01	3.7E-01	6.6E-01
ESCA	EVA1C_AD_6.3_6.2_7	EVA1C	AD	6.3	6.2	7	4.7E-05	0.117	-0.173	8.1E-01	1.0E+00	1.2E-01
ESCA	EVI5_ES_12_11_13	EVI5	ES	12	11	13	1.3E-04	0.105	-0.133	8.6E-02	3.1E-02	6.7E-02
ESCA	EXO5_AD_1.2:1.3_1.1_1.5	EXO5	AD	1.2:1.3	1.1	1.5	1.6E-04	0.108	0.134	6.1E-01	6.1E-01	6.7E-01
ESCA	EXOC1_ES_11_10_12	EXOC1	ES	11	10	12	8.7E-21	0.440	0.218	4.2E-01	9.7E-01	6.3E-01
ESCA	EXOC7_ES_7:8.1:8.2_6_9	EXOC7	ES	7:8.1:8.2	6	9	6.7E-13	0.296	-0.009	5.9E-01	4.0E-01	4.8E-01
ESCA	EXOC7_ES_7:8.2_6_9	EXOC7	ES	07:08.2	6	9	1.1E-06	0.156	-0.066	3.0E-01	6.2E-01	8.4E-01
ESCA	EXOC7_ES_8.1:8.2_7_9	EXOC7	ES	8.1:8.2	7	9	1.5E-10	0.247	-0.096	8.8E-01	4.4E-01	3.3E-01
ESCA	EXOC7_ES_8.2_6_9	EXOC7	ES	8.2	6	9	9.1E-13	0.294	-0.047	4.7E-01	6.7E-01	6.6E-01
ESCA	EXOC7_ES_8.2_7_9	EXOC7	ES	8.2	7	9	7.5E-09	0.221	-0.162	5.5E-01	1.2E-01	9.6E-01
ESCA	FAM122B_ES_3_2_4	FAM122B	ES	3	2	4	1.6E-06	0.152	-0.117	2.9E-01	4.2E-01	1.6E-01
ESCA	FAM126A_ES_11_10_12	FAM126A	ES	11	10	12	9.8E-24	0.485	-0.481	4.3E-01	3.7E-01	9.6E-01
ESCA	FAM126B_ES_13_12_14	FAM126B	ES	13	12	14	1.2E-10	0.250	-0.171	6.4E-01	5.8E-01	2.2E-02
ESCA	FAM135A_ES_11_10_12	FAM135A	ES	11	10	12	1.3E-07	0.179	0.095	6.3E-01	2.9E-01	5.2E-01
ESCA	FAM221A_ES_2:3.1:3.2:3.3_1_4	FAM221A	ES	2:3.1:3.2:3.3	1	4	7.4E-09	0.209	-0.654	7.7E-01	7.6E-01	1.8E-01
ESCA	FAM221A_ES_3.1:3.2:3.3_2_4	FAM221A	ES	3.1:3.2:3.3	2	4	2.4E-11	0.264	-0.746	9.5E-01	7.2E-01	7.3E-01
ESCA	FAM222B_ES_5_2.1_7.2	FAM222B	ES	5	2.1	7.2	1.3E-04	0.112	0.140	6.5E-02	5.9E-01	7.4E-01
ESCA	FAM228B_ES_4:5:6:7:8_3_9.1	FAM228B	ES	4:5:6:7:8	3	9.1	9.8E-05	0.116	-0.163	6.9E-02	8.4E-01	3.9E-01
ESCA	FAM32A_ES_2:3_1.3_4	FAM32A	ES	2:03	1.3	4	2.4E-04	0.103	0.046	7.8E-01	5.5E-01	6.1E-01
ESCA	FAM49B_ES_5_3_6	FAM49B	ES	5	3	6	2.1E-06	0.149	0.085	4.7E-01	8.9E-02	6.7E-01
ESCA	FAM64A_ES_7_5_9	FAM64A	ES	7	5	9	6.1E-05	0.112	0.066	2.3E-03	1.7E-02	6.4E-01
ESCA	FAM86C1_ES_3.2_2_5.1	FAM86C1	ES	3.2	2	5.1	2.3E-05	0.123	-0.587	7.3E-01	3.7E-01	9.0E-01
ESCA	FAXDC2_ES_6_5.2_7	FAXDC2	ES	6	5.2	7	2.2E-06	0.163	-0.047	5.8E-01	3.9E-01	5.4E-01
ESCA	FBXL6_ES_3.1_2_4	FBXL6	ES	3.1	2	4	3.4E-05	0.127	0.318	3.5E-01	9.2E-01	2.0E-01
ESCA	FBXO44_ES_5.2:6_5.1_7	FBXO44	ES	5.2:6	5.1	7	6.2E-05	0.112	-0.057	2.9E-02	7.4E-01	7.7E-01
ESCA	FGFR1_ME_12.1:12.2|13_11_14.1	FGFR1	ME	12.1:12.2|13	11	14.1	3.9E-13	0.303	0.201	7.2E-01	5.0E-01	6.0E-01

cancer type	id	Gene Symbol	splice_type	Exon	From.Exon	To.Exon	anova.p	adj.r2	r	p.50	p.25	p.10
ESCA	FGFR3_ES_9:10_7_11.2	FGFR3	ES	9:10	7	11.2	1.8E-04	0.112	-0.682	1.2E-02	6.5E-02	2.4E-01
ESCA	FHL2_ES_2.3:3.2_2.2_5.1	FHL2	ES	2.3:3.2	2.2	5.1	3.1E-05	0.120	0.077	2.4E-01	1.8E-01	2.6E-01
ESCA	FHL2_ES_3.2_2.2_5.1	FHL2	ES	3.2	2.2	5.1	3.0E-05	0.120	0.152	8.0E-01	7.0E-02	3.5E-01
ESCA	FHL2_ES_3.2_2.3_5.1	FHL2	ES	3.2	2.3	5.1	3.3E-05	0.119	0.216	5.7E-01	6.1E-01	8.8E-01
ESCA	FHL2_ES_6:7_5.1_8	FHL2	ES	6:07	5.1	8	2.2E-05	0.141	0.143	4.8E-01	5.0E-01	9.0E-01
ESCA	FIGNL1_RI_2.3:2.4:2.5_2.2_2.6	FIGNL1	RI	2.3:2.4:2.5	2.2	2.6	1.1E-05	0.131	0.139	6.4E-01	9.3E-01	4.2E-01
ESCA	FLNB_ES_27_26_28	FLNB	ES	27	26	28	3.8E-16	0.359	0.073	7.4E-01	7.2E-01	9.2E-02
ESCA	FLNB_ES_32.1_31_33	FLNB	ES	32.1	31	33	1.9E-06	0.151	0.181	9.6E-01	8.3E-01	6.2E-01
ESCA	FLNB_ES_32.1:32.2_31_33	FLNB	ES	32.1:32.2	31	33	1.7E-06	0.152	0.113	4.2E-01	8.9E-01	5.2E-01
ESCA	FMNL2_ES_27_26_28	FMNL2	ES	27	26	28	8.8E-05	0.125	-0.125	8.4E-01	7.4E-01	3.2E-01
ESCA	FNBP1_ES_10.1:10.2:10.3_9_14.2	FNBP1	ES	10.1:10.2:10.3	9	14.2	1.1E-09	0.243	-0.037	7.7E-01	8.2E-01	3.7E-01
ESCA	FNBP1_ES_10.1:10.2:10.3:12_9_14.2	FNBP1	ES	10.1:10.2:10.3:12	9	14.2	2.6E-23	0.501	0.200	2.7E-01	2.0E-01	1.6E-01
ESCA	FNBP1_ES_10.2:10.3_9_14.2	FNBP1	ES	10.2:10.3	9	14.2	1.0E-08	0.211	0.133	1.5E-01	2.1E-01	5.3E-01
ESCA	FNBP1_ES_10.2:10.3:12_9_14.2	FNBP1	ES	10.2:10.3:12	9	14.2	1.0E-15	0.382	0.214	1.9E-02	8.8E-02	4.7E-01
ESCA	FNBP1_ES_12_10.3_14.2	FNBP1	ES	12	10.3	14.2	8.1E-17	0.382	0.274	6.3E-01	2.5E-01	3.4E-01
ESCA	FNIP1_ES_7_6_8	FNIP1	ES	7	6	8	1.7E-06	0.155	-0.048	4.1E-01	3.7E-01	5.1E-01
ESCA	FNTA_AD_7.2_7.1_8	FNTA	AD	7.2	7.1	8	9.1E-05	0.107	0.013	7.1E-01	1.2E-01	5.6E-03
ESCA	FOSL1_ES_3.2_2_4	FOSL1	ES	3.2	2	4	1.7E-06	0.164	-0.127	6.5E-01	9.6E-01	1.5E-01
ESCA	FOXM1_ES_6_5_7	FOXM1	ES	6	5	7	3.7E-10	0.238	0.205	1.6E-01	1.5E-01	2.9E-01
ESCA	FYN_ME_11|12_10_13	FYN	ME	11|12	10	13	1.8E-07	0.176	-0.016	2.4E-01	2.1E-01	5.3E-01
ESCA	G6PC3_ES_5_4_6	G6PC3	ES	5	4	6	2.3E-25	0.509	0.393	2.8E-01	5.3E-01	2.3E-01
ESCA	GAA_ES_2.2_1_3	GAA	ES	2.2	1	3	8.6E-06	0.142	0.447	9.9E-01	4.5E-01	5.4E-01
ESCA	GAB1_ES_8_7_9	GAB1	ES	8	7	9	1.7E-27	0.538	0.070	8.1E-01	4.6E-01	7.9E-01
ESCA	GALNT7_ES_5:6.1_4_6.2	GALNT7	ES	05:06.1	4	6.2	8.7E-07	0.209	0.382	2.8E-01	4.3E-01	4.6E-01
ESCA	GCNT3_AA_2.1_1_2.2	GCNT3	AA	2.1	1	2.2	2.5E-13	0.386	-0.501	5.9E-01	6.6E-01	8.0E-01
ESCA	GDPD5_ES_4_3_5.2	GDPD5	ES	4	3	5.2	4.6E-06	0.148	0.270	7.6E-01	5.1E-01	9.8E-01
ESCA	GGT1_AA_15.3_14_15.4	GGT1	AA	15.3	14	15.4	6.3E-07	0.167	-0.536	7.2E-01	7.9E-01	6.8E-01
ESCA	GINS3_ES_2_1_3	GINS3	ES	2	1	3	7.2E-05	0.114	0.306	9.1E-01	9.7E-01	1.8E-01
ESCA	GIPC1_ES_2:4_1_5	GIPC1	ES	2:04	1	5	7.6E-05	0.132	-0.129	3.8E-01	8.2E-01	5.3E-01
ESCA	GIPC1_ES_3_1_5	GIPC1	ES	3	1	5	5.5E-05	0.118	-0.076	9.3E-02	8.7E-01	5.9E-01
ESCA	GIPC1_ES_4_1_5	GIPC1	ES	4	1	5	4.4E-05	0.120	-0.082	5.1E-01	1.7E-01	5.8E-01
ESCA	GIT2_ES_19_18.2_20	GIT2	ES	19	18.2	20	2.0E-32	0.599	0.192	3.1E-01	4.6E-01	9.0E-01
ESCA	GLS2_ES_4.1:4.2_3_5	GLS2	ES	4.1:4.2	3	5	9.9E-05	0.124	-0.148	9.4E-01	4.1E-01	5.1E-01
ESCA	GLYCTK_ES_3_2_4	GLYCTK	ES	3	2	4	8.2E-15	0.338	0.612	7.9E-01	6.5E-01	1.5E-01
ESCA	GMPR2_RI_10.2_10.1_10.3	GMPR2	RI	10.2	10.1	10.3	8.0E-05	0.109	-0.245	8.3E-01	9.4E-01	3.0E-01
ESCA	GNB1L_RI_1.2_1.1_1.3	GNB1L	RI	1.2	1.1	1.3	2.9E-07	0.171	-0.367	2.3E-01	9.6E-01	9.6E-01
ESCA	GNLY_ES_2.1:2.2_1_3	GNLY	ES	2.1:2.2	1	3	1.4E-06	0.181	-0.234	8.7E-01	9.9E-01	6.0E-01
ESCA	GOLGA4_ES_24_23_25	GOLGA4	ES	24	23	25	3.1E-32	0.597	0.465	9.3E-01	7.8E-01	9.4E-01
ESCA	GOLGA8B_ES_2_1_3	GOLGA8B	ES	2	1	3	2.9E-07	0.172	0.204	3.5E-02	3.5E-01	3.3E-01
ESCA	GPBP1_ES_9_8_10	GPBP1	ES	9	8	10	9.5E-06	0.133	-0.065	9.1E-01	5.5E-01	8.3E-03

cancer type	id	Gene Symbol	splice_type	Exon	From.Exon	To.Exon	anova.p	adj.r2	r	p.50	p.25	p.10
ESCA	GPR137_ES_6_5_8	GPR137	ES	6	5	8	2.5E-23	0.481	0.119	6.7E-01	6.5E-01	5.3E-01
ESCA	GPX2_ME_2|3_1_4	GPX2	ME	2|3	1	4	8.4E-08	0.188	0.150	2.6E-01	2.6E-01	7.0E-01
ESCA	GRHL2_AD_1.2_1.1_2	GRHL2	AD	1.2	1.1	2	2.7E-12	0.285	0.377	3.3E-01	3.2E-01	3.0E-02
ESCA	GRK6_AA_17.1_15_17.2	GRK6	AA	17.1	15	17.2	1.3E-11	0.270	0.035	7.2E-01	3.2E-01	8.8E-01
ESCA	GSDMB_AA_6.1_5_6.2	GSDMB	AA	6.1	5	6.2	1.1E-04	0.133	0.273	4.4E-01	8.7E-01	7.7E-01
ESCA	GSDMB_ES_5_4_7	GSDMB	ES	5	4	7	3.0E-08	0.248	0.550	1.0E-02	4.4E-01	9.3E-01
ESCA	GTF2A2_AD_1.2_1.1_3	GTF2A2	AD	1.2	1.1	3	1.2E-11	0.270	-0.224	8.5E-01	1.7E-01	1.9E-01
ESCA	GUSB_ES_6_5.1_7	GUSB	ES	6	5.1	7	3.3E-08	0.193	0.186	1.6E-01	2.0E-01	3.9E-02
ESCA	HDDC2_ES_3.1_2_5	HDDC2	ES	3.1	2	5	2.4E-06	0.148	-0.097	9.5E-01	2.1E-01	8.7E-01
ESCA	HKR1_ES_11_8.2_12	HKR1	ES	11	8.2	12	1.1E-04	0.130	-0.051	4.9E-01	1.2E-01	6.8E-01
ESCA	HMBOX1_RI_12.3_12.2_12.4	HMBOX1	RI	12.3	12.2	12.4	3.4E-05	0.134	-0.013	2.1E-01	2.0E-01	3.2E-01
ESCA	HMBS_AD_12.2:12.3_12.1_13	HMBS	AD	12.2:12.3	12.1	13	1.6E-08	0.201	-0.026	2.6E-01	1.9E-01	5.1E-01
ESCA	HMOX2_ES_7:8:9:10_6_11	HMOX2	ES	7:8:9:10	6	11	3.5E-05	0.119	0.390	2.5E-02	9.5E-02	1.4E-01
ESCA	HNRNPA1_ES_3:4:5:6.1:6.2:9.1:9.2:10:1	HNRNPA1	ES	l:6.2:9.1:9.2:10:1	2	11.3	5.2E-07	0.185	0.012	9.2E-01	4.4E-01	8.5E-01
ESCA	HNRNPA1_ES_6.2:9.1_6.1_9.2	HNRNPA1	ES	6.2:9.1	6.1	9.2	4.1E-10	0.267	-0.026	4.3E-01	4.5E-01	4.0E-01
ESCA	HNRNPA1_ES_8_6.2_9.1	HNRNPA1	ES	8	6.2	9.1	1.4E-12	0.329	0.081	5.9E-01	5.9E-01	8.3E-02
ESCA	HNRNPA1_ES_9.1:9.2_6.2_10	HNRNPA1	ES	9.1:9.2	6.2	10	1.7E-11	0.301	-0.043	7.4E-01	3.4E-01	8.4E-02
ESCA	HOPX_RI_4.2:4.3:4.4:4.5_4.1_4.6	HOPX	RI	4.2:4.3:4.4:4.5	4.1	4.6	6.6E-04	0.105	-0.140	5.7E-01	6.4E-01	9.7E-01
ESCA	HOXA1_RI_1.2_1.1_1.3	HOXA1	RI	1.2	1.1	1.3	2.0E-04	0.104	-0.031	6.5E-01	5.0E-02	3.2E-02
ESCA	HOXA9_RI_1.2_1.1_1.3	HOXA9	RI	1.2	1.1	1.3	6.1E-06	0.171	-0.559	8.8E-01	9.8E-01	8.4E-01
ESCA	HPS4_RI_10.4_10.3_10.5	HPS4	RI	10.4	10.3	10.5	2.4E-05	0.123	-0.162	7.3E-01	3.1E-01	4.5E-01
ESCA	HTATIP2_AD_1.2:1.3_1.1_1.5	HTATIP2	AD	1.2:1.3	1.1	1.5	1.5E-04	0.102	0.061	8.1E-01	5.2E-01	6.3E-01
ESCA	HTATIP2_RI_1.4_1.3_1.5	HTATIP2	RI	1.4	1.3	1.5	3.9E-05	0.118	0.225	1.8E-01	5.1E-01	5.8E-01
ESCA	HYAL1_RI_4.2_4.1_4.3	HYAL1	RI	4.2	4.1	4.3	2.4E-05	0.126	-0.356	8.3E-01	3.9E-01	3.9E-02
ESCA	HYOU1_AD_1.2_1.1_3	HYOU1	AD	1.2	1.1	3	4.2E-06	0.142	0.157	9.0E-01	9.3E-01	2.7E-01
ESCA	ICAM3_RI_3.2_3.1_3.3	ICAM3	RI	3.2	3.1	3.3	6.1E-06	0.139	-0.714	1.9E-02	3.7E-01	7.4E-01
ESCA	IFI27_ES_3.1:3.2_1_4.1	IFI27	ES	3.1:3.2	1	4.1	1.2E-04	0.104	0.052	5.8E-01	3.8E-01	4.6E-01
ESCA	IFI27_ES_3.2_1_4.1	IFI27	ES	3.2	1	4.1	1.5E-06	0.153	0.057	5.1E-01	8.8E-01	8.1E-01
ESCA	IFI44_ES_7.1:8_6_9	IFI44	ES	7.1:8	6	9	1.8E-04	0.108	-0.102	3.2E-01	9.5E-01	9.8E-02
ESCA	IFT122_ES_5:7_3_8.2	IFT122	ES	5:07	3	8.2	9.1E-05	0.109	-0.101	7.3E-01	9.6E-01	8.9E-01
ESCA	IFT172_ES_46_45_47	IFT172	ES	46	45	47	6.8E-20	0.444	-0.025	3.2E-01	2.9E-01	3.2E-01
ESCA	IGSF3_ES_6_5_7	IGSF3	ES	6	5	7	1.9E-08	0.199	-0.332	2.8E-01	2.8E-01	1.7E-01
ESCA	IL32_AD_1.2:1.3:1.4:1.5_1.1_1.9	IL32	AD	1.2:1.3:1.4:1.5	1.1	1.9	1.5E-05	0.127	0.343	8.6E-01	9.2E-01	2.8E-01
ESCA	IL7R_ES_6_5_7.1	IL7R	ES	6	5	7.1	6.7E-05	0.122	-0.106	6.9E-01	9.8E-01	6.2E-01
ESCA	INCENP_ES_11_10_12	INCENP	ES	11	10	12	6.5E-29	0.557	0.171	3.0E-01	1.4E-01	4.9E-01
ESCA	INO80E_ES_6.1:6.2:6.3:10_5_11	INO80E	ES	6.1:6.2:6.3:10	5	11	1.5E-04	0.102	-0.164	8.5E-02	2.1E-02	3.6E-03
ESCA	INPP4A_ES_14_13_16.2	INPP4A	ES	14	13	16.2	3.5E-05	0.119	0.085	7.8E-01	7.3E-01	5.4E-01
ESCA	INPP5B_AA_16.1_15_16.2	INPP5B	AA	16.1	15	16.2	1.9E-09	0.223	-0.142	4.7E-01	6.1E-01	9.2E-01
ESCA	INPP5B_AD_7.2_7.1_8	INPP5B	AD	7.2	7.1	8	1.4E-12	0.300	0.041	1.0E+00	3.5E-01	9.5E-01
ESCA	INSR_ES_11_10_12	INSR	ES	11	10	12	1.2E-07	0.180	-0.318	8.9E-01	3.1E-01	5.3E-01

cancer type	id	Gene Symbol	splice_type	Exon	From.Exon	To.Exon	anova.p	adj.r2	r	p.50	p.25	p.10
ESCA	INVS_RI_16.2_16.1_16.3	INVS	RI	16.2	16.1	16.3	3.5E-05	0.118	0.151	7.0E-01	6.9E-01	8.8E-01
ESCA	IP6K2_AA_11.4:11.5:11.6_11.2_11.7	IP6K2	AA	11.4:11.5:11.6	11.2	11.7	9.0E-07	0.159	0.019	5.1E-01	4.7E-01	8.3E-01
ESCA	IP6K2_AA_11.7:11.8_11.2_11.9	IP6K2	AA	11.7:11.8	11.2	11.9	8.9E-05	0.108	-0.048	9.6E-01	9.1E-01	6.5E-01
ESCA	IP6K2_AD_11.3:11.4:11.5_11.2_11.9	IP6K2	AD	11.3:11.4:11.5	11.2	11.9	5.3E-10	0.234	0.063	3.8E-01	4.2E-01	1.7E-02
ESCA	IP6K2_AD_11.3:11.4:11.5:11.6:11.7_11.2	IP6K2	AD	3:11.4:11.5:11.6:1	11.2	11.9	1.7E-04	0.100	0.007	4.6E-01	5.6E-01	8.6E-01
ESCA	IP6K2_AD_11.6:11.7_11.5_11.9	IP6K2	AD	11.6:11.7	11.5	11.9	3.3E-09	0.219	-0.003	6.6E-01	2.8E-01	2.0E-01
ESCA	IP6K2_ES_11.4:11.5:11.6:11.7_11.2_11.9	IP6K2	ES	1.4:11.5:11.6:11.	11.2	11.9	1.2E-04	0.104	-0.113	3.2E-01	6.9E-01	4.4E-01
ESCA	IP6K2_ES_11.7_11.2_11.9	IP6K2	ES	11.7	11.2	11.9	2.4E-07	0.173	-0.082	7.8E-01	7.2E-01	8.7E-01
ESCA	IP6K2_RI_11.3:11.4:11.5:11.6_11.2_11.7	IP6K2	RI	1.3:11.4:11.5:11.	11.2	11.7	5.7E-07	0.164	0.098	9.3E-01	2.0E-01	9.3E-02
ESCA	IP6K2_RI_11.8_11.7_11.9	IP6K2	RI	11.8	11.7	11.9	5.1E-09	0.212	0.090	6.6E-01	5.8E-01	2.8E-01
ESCA	IRAK1_ES_10.1:10.3_9_11.1	IRAK1	ES	10.1:10.3	9	11.1	1.1E-05	0.131	0.114	3.8E-01	2.8E-01	9.0E-01
ESCA	ISCU_AA_5.3:5.4:5.5_5.1_5.6	ISCU	AA	5.3:5.4:5.5	5.1	5.6	5.1E-05	0.120	0.056	8.7E-01	1.4E-01	5.5E-02
ESCA	ITGA6_ES_27_26_28	ITGA6	ES	27	26	28	1.0E-35	0.635	0.352	3.0E-01	4.1E-01	8.9E-01
ESCA	ITGA7_ES_29_28_30	ITGA7	ES	29	28	30	1.8E-18	0.427	-0.108	9.0E-01	7.8E-01	8.8E-01
ESCA	JAG2_ES_10_9_11	JAG2	ES	10	9	11	1.8E-18	0.401	-0.597	1.8E-01	6.8E-01	5.6E-01
ESCA	KDM6A_ES_13_12_14	KDM6A	ES	13	12	14	1.1E-09	0.260	0.029	9.3E-01	1.6E-01	7.9E-02
ESCA	KDM6A_ES_13_12_15	KDM6A	ES	13	12	15	1.7E-08	0.204	0.121	7.7E-01	8.5E-01	8.0E-01
ESCA	KDM6A_ES_13:14_12_15	KDM6A	ES	13:14	12	15	1.4E-05	0.166	0.086	9.2E-01	3.7E-01	4.9E-01
ESCA	KDM6A_ME_13|14_12_15	KDM6A	ME	13|14	12	15	8.9E-11	0.253	0.132	3.0E-01	3.5E-01	2.0E-01
ESCA	KIAA0226_ES_14_13_15	KIAA0226	ES	14	13	15	1.8E-05	0.126	-0.098	8.6E-01	2.2E-01	2.0E-01
ESCA	KIAA1217_ES_12_11_13	KIAA1217	ES	12	11	13	3.8E-42	0.697	-0.076	4.7E-01	2.5E-01	7.0E-01
ESCA	KIAA1217_RI_25.2_25.1_25.3	KIAA1217	RI	25.2	25.1	25.3	9.4E-22	0.455	0.152	7.9E-01	3.7E-01	5.6E-01
ESCA	KIF13A_ES_28_27_29	KIF13A	ES	28	27	29	4.6E-10	0.236	-0.090	1.8E-01	2.5E-02	2.5E-01
ESCA	KIF23_ES_9_8.2_10	KIF23	ES	9	8.2	10	2.1E-17	0.382	0.131	4.1E-02	2.8E-02	1.8E-01
ESCA	KIF3A_ES_11_9_12	KIF3A	ES	11	9	12	8.1E-10	0.230	-0.030	6.2E-01	2.3E-01	9.4E-01
ESCA	KIF9_ES_3_2.3_4	KIF9	ES	3	2.3	4	2.4E-05	0.143	-0.537	6.0E-01	2.7E-01	9.3E-01
ESCA	KIRREL_AA_9.1_8_9.2	KIRREL	AA	9.1	8	9.2	1.2E-04	0.108	0.025	7.9E-01	9.5E-01	7.5E-01
ESCA	KLC1_AD_13.3_13.2_15	KLC1	AD	13.3	13.2	15	2.8E-06	0.146	0.282	3.6E-01	1.8E-01	6.0E-01
ESCA	KLC1_ES_13.3:15_13.2_18	KLC1	ES	13.3:15	13.2	18	4.5E-06	0.141	0.197	9.7E-01	3.4E-02	5.4E-01
ESCA	KLC1_ES_15:16_13.2_18	KLC1	ES	15:16	13.2	18	1.6E-07	0.177	-0.377	5.9E-01	2.4E-01	7.8E-01
ESCA	KLC1_ES_15:16_13.3_18	KLC1	ES	15:16	13.3	18	8.0E-05	0.110	-0.195	4.6E-01	6.6E-01	7.6E-01
ESCA	KLC1_ES_16_15_18	KLC1	ES	16	15	18	1.5E-09	0.225	-0.324	6.1E-02	2.9E-01	9.8E-01
ESCA	KLHDC2_RI_7.2_7.1_7.3	KLHDC2	RI	7.2	7.1	7.3	1.1E-04	0.105	0.157	3.8E-01	4.1E-01	6.9E-01
ESCA	KRAS_ES_6_5_7	KRAS	ES	6	5	7	1.4E-19	0.420	0.197	8.2E-01	9.2E-01	5.5E-01
ESCA	KREMEN2_RI_7.2:7.3:7.4_7.1_7.5	KREMEN2	RI	7.2:7.3:7.4	7.1	7.5	9.3E-14	0.336	-0.742	9.3E-01	4.4E-01	8.8E-01
ESCA	KREMEN2_RI_7.4_7.3_7.5	KREMEN2	RI	7.4	7.3	7.5	4.8E-16	0.380	-0.711	5.7E-01	1.6E-01	1.3E-01
ESCA	KRT15_AA_1.3:1.4:1.5:1.6_1.1_1.7	KRT15	AA	1.3:1.4:1.5:1.6	1.1	1.7	1.0E-10	0.294	0.613	7.7E-01	4.9E-01	4.3E-01
ESCA	KRT15_RI_1.2:1.3:1.4:1.5:1.6_1.1_1.7	KRT15	RI	1.2:1.3:1.4:1.5:1.6	1.1	1.7	3.5E-16	0.375	0.615	6.0E-01	7.6E-01	9.7E-01
ESCA	KRT15_RI_7.2_7.1_7.3	KRT15	RI	7.2	7.1	7.3	2.4E-14	0.325	-0.796	3.6E-01	1.9E-01	4.2E-01
ESCA	KRT15_RI_7.4_7.3_7.5	KRT15	RI	7.4	7.3	7.5	5.1E-09	0.213	-0.548	5.7E-01	4.8E-01	9.9E-02

cancer type	id	Gene Symbol	splice_type	Exon	From.Exon	To.Exon	anova.p	adj.r2	r	p.50	p.25	p.10
ESCA	KSR1_AA_24.1_22_24.2	KSR1	AA	24.1	22	24.2	1.9E-14	0.327	0.170	1.2E-01	3.4E-01	3.8E-01
ESCA	KSR1_ES_13_12_15	KSR1	ES	13	12	15	1.9E-05	0.135	0.268	9.8E-01	7.1E-01	8.5E-01
ESCA	KTN1_ES_42_41_43	KTN1	ES	42	41	43	1.3E-12	0.291	0.594	4.7E-01	7.8E-01	1.3E-01
ESCA	LANCL1_RI_1.2:1.3:1.4:1.5_1.1_1.6	LANCL1	RI	1.2:1.3:1.4:1.5	1.1	1.6	1.1E-04	0.107	0.005	9.4E-01	7.2E-01	7.6E-01
ESCA	LCMT1_ES_2_1_3.1	LCMT1	ES	2	1	3.1	2.2E-05	0.124	-0.133	9.8E-01	3.1E-01	2.1E-01
ESCA	LDHA_ES_3:4_2.4_6.1	LDHA	ES	3:04	2.4	6.1	7.9E-13	0.295	-0.423	1.7E-01	1.8E-01	7.2E-02
ESCA	LDLRAD3_ES_5_4_6	LDLRAD3	ES	5	4	6	1.2E-06	0.155	-0.730	7.7E-01	1.3E-01	9.1E-01
ESCA	LEF1_ES_7_6.1_8	LEF1	ES	7	6.1	8	1.1E-04	0.108	0.449	1.4E-01	4.7E-01	3.7E-01
ESCA	LENG8_RI_15.2:15.3:15.4_15.1_15.5	LENG8	RI	15.2:15.3:15.4	15.1	15.5	2.1E-12	0.286	0.801	6.0E-01	3.2E-01	8.8E-01
ESCA	LETMD1_ES_3.2:3.3:4_2_5	LETMD1	ES	3.2:3.3:4	2	5	1.2E-04	0.105	0.177	7.5E-01	6.9E-01	1.8E-01
ESCA	LGALS3BP_ES_5.1:5.2:5.3:5.4_4.2_6	LGALS3BP	ES	5.1:5.2:5.3:5.4	4.2	6	3.2E-06	0.146	0.089	3.0E-01	9.9E-01	6.0E-01
ESCA	LGALS9_ES_5_4_6	LGALS9	ES	5	4	6	9.7E-20	0.423	0.557	3.3E-01	6.9E-02	1.5E-01
ESCA	LIMCH1_ES_28_27_29	LIMCH1	ES	28	27	29	7.4E-05	0.132	0.284	5.0E-01	5.9E-01	9.4E-01
ESCA	LMO7_AA_19.1_18_19.2	LMO7	AA	19.1	18	19.2	2.5E-26	0.522	-0.220	5.7E-01	4.4E-01	5.8E-01
ESCA	LMO7_ES_10:11_9_12	LMO7	ES	10:11	9	12	3.1E-13	0.303	0.414	2.8E-02	1.7E-02	7.2E-02
ESCA	LMO7_ES_10:11:12_9_13	LMO7	ES	10:11:12	9	13	4.0E-07	0.170	-0.330	3.3E-01	3.3E-01	2.8E-01
ESCA	LMO7_ES_12_9_13	LMO7	ES	12	9	13	8.7E-35	0.625	-0.619	4.7E-01	5.5E-01	1.9E-01
ESCA	LMO7_RI_5.2:5.3_5.1_5.4	LMO7	RI	5.2:5.3	5.1	5.4	3.8E-30	0.613	0.328	7.5E-01	9.8E-01	6.0E-01
ESCA	LPIN1_ES_10_9_12	LPIN1	ES	10	9	12	1.9E-10	0.248	0.362	6.3E-01	8.3E-01	6.8E-01
ESCA	LRR1_ES_4_3_5	LRR1	ES	4	3	5	4.3E-05	0.116	0.093	9.9E-02	8.2E-02	1.0E-01
ESCA	LRRFIP2_ES_20_19_21	LRRFIP2	ES	20	19	21	8.3E-36	0.636	-0.089	6.1E-01	9.6E-02	9.8E-01
ESCA	LRRFIP2_ES_7:8_5_18	LRRFIP2	ES	7:08	5	18	2.9E-09	0.229	-0.005	6.5E-01	5.7E-01	8.2E-01
ESCA	LSR_ES_4:5_3_6	LSR	ES	4:05	3	6	9.8E-14	0.313	0.120	9.0E-01	9.6E-01	6.4E-01
ESCA	LSR_ES_5_3_6	LSR	ES	5	3	6	1.6E-22	0.467	0.100	9.5E-01	4.8E-01	7.7E-01
ESCA	LSR_ES_5_4_6	LSR	ES	5	4	6	3.3E-15	0.347	0.156	3.6E-01	8.9E-01	4.8E-01
ESCA	LTB4R2_RI_1.2:1.3_1.1_1.4	LTB4R2	RI	1.2:1.3	1.1	1.4	1.2E-26	0.527	-0.766	4.7E-01	6.0E-01	5.7E-01
ESCA	LY6K_RI_2.2:2.3_2.1_2.4	LY6K	RI	2.2:2.3	2.1	2.4	4.2E-08	0.203	-0.752	5.5E-01	6.7E-02	6.8E-01
ESCA	LYSMD4_ES_4.2:4.3:5_4.1_6	LYSMD4	ES	4.2:4.3:5	4.1	6	2.8E-05	0.124	-0.151	2.0E-01	4.9E-01	6.8E-01
ESCA	MACF1_ES_43.2:44:45:46:47_42_48	MACF1	ES	43.2:44:45:46:47	42	48	3.4E-06	0.147	-0.101	5.7E-02	4.0E-01	9.0E-01
ESCA	MADD_ES_18_17_19	MADD	ES	18	17	19	1.7E-04	0.100	-0.205	8.1E-01	7.1E-02	3.7E-02
ESCA	MAF_RI_1.2_1.1_1.3	MAF	RI	1.2	1.1	1.3	7.6E-06	0.135	-0.251	2.2E-02	2.0E-01	2.7E-02
ESCA	MAGI3_ES_22.1_21_23	MAGI3	ES	22.1	21	23	2.2E-08	0.201	-0.246	5.9E-01	8.6E-01	7.0E-01
ESCA	MALT1_ES_7_6_8	MALT1	ES	7	6	8	3.7E-10	0.238	0.212	7.7E-01	1.3E-01	4.6E-01
ESCA	MAP2K7_ES_2_1_3	MAP2K7	ES	2	1	3	1.3E-18	0.406	0.128	5.8E-01	2.8E-01	5.1E-01
ESCA	MAP3K4_ES_18_17_19	MAP3K4	ES	18	17	19	1.2E-12	0.291	-0.216	2.1E-01	2.2E-01	9.2E-01
ESCA	MAP3K6_ES_3_2_4	MAP3K6	ES	3	2	4	1.5E-09	0.226	0.298	1.8E-01	3.2E-01	3.0E-01
ESCA	MAP3K7_ES_11_10_12	MAP3K7	ES	11	10	12	2.3E-21	0.449	-0.087	3.9E-01	8.3E-01	9.0E-01
ESCA	MAP4K4_ES_15:16.1:16.2:17:18_14_19	MAP4K4	ES	.5:16.1:16.2:17:1[illegible]	14	19	3.7E-04	0.105	0.054	8.9E-01	1.9E-01	7.2E-01
ESCA	MAP4K4_ES_16.1:16.2:17_14_19	MAP4K4	ES	16.1:16.2:17	14	19	1.1E-06	0.176	0.036	5.7E-01	1.2E-01	5.7E-01
ESCA	MAP4K4_ES_17_16.2_19	MAP4K4	ES	17	16.2	19	1.6E-21	0.451	-0.280	3.7E-01	8.0E-01	1.8E-01

cancer type	id	Gene Symbol	splice_type	Exon	From.Exon	To.Exon	anova.p	adj.r2	r	p.50	p.25	p.10
ESCA	MAP4K4_ES_17:18_16.2_19	MAP4K4	ES	17:18	16.2	19	7.8E-07	0.160	-0.077	8.0E-01	2.1E-01	7.5E-01
ESCA	MAP7_ES_9_8_10	MAP7	ES	9	8	10	1.0E-15	0.352	-0.126	6.9E-01	4.1E-01	7.2E-01
ESCA	MAPRE3_AD_5.2_5.1_6	MAPRE3	AD	5.2	5.1	6	2.6E-06	0.147	0.270	3.1E-01	3.0E-01	2.4E-01
ESCA	MARCH2_ES_2.2_1_3.1	MARCH2	ES	2.2	1	3.1	2.6E-05	0.122	-0.267	5.3E-01	6.9E-01	1.1E-01
ESCA	MARK2_ES_16.1:16.2_15.2_17	MARK2	ES	16.1:16.2	15.2	17	7.1E-26	0.516	-0.067	6.3E-01	8.4E-01	4.1E-01
ESCA	MARK2_ES_18_17_19	MARK2	ES	18	17	19	3.3E-11	0.261	0.258	4.2E-01	5.7E-01	4.7E-01
ESCA	MARK2_ES_18_17_20	MARK2	ES	18	17	20	1.5E-13	0.339	0.201	4.3E-01	1.3E-01	4.1E-01
ESCA	MARK2_ES_18:19_17_20	MARK2	ES	18:19	17	20	5.1E-07	0.165	0.176	5.4E-01	9.8E-01	6.2E-01
ESCA	MARK2_ES_19_18_20	MARK2	ES	19	18	20	3.1E-05	0.122	0.083	7.9E-01	5.8E-01	8.8E-01
ESCA	MARK3_ES_16:17_15_19	MARK3	ES	16:17	15	19	1.3E-06	0.202	0.323	5.3E-01	9.7E-01	9.1E-01
ESCA	MARK3_ES_16:17:18_15_19	MARK3	ES	16:17:18	15	19	9.1E-10	0.293	0.282	5.7E-01	9.8E-01	7.9E-01
ESCA	MARK3_ES_17_16_18	MARK3	ES	17	16	18	6.5E-12	0.276	0.170	7.8E-01	4.6E-02	1.2E-01
ESCA	MARK3_ES_17_16_19	MARK3	ES	17	16	19	9.8E-08	0.182	0.142	7.6E-01	3.3E-01	6.9E-01
ESCA	MARK3_ES_17:18_16_19	MARK3	ES	17:18	16	19	6.6E-11	0.254	0.086	6.4E-01	3.0E-01	3.8E-01
ESCA	MBNL2_ES_7_6.3_8	MBNL2	ES	7	6.3	8	6.2E-15	0.337	0.146	1.3E-01	9.1E-01	6.1E-01
ESCA	MBNL2_ES_9_8_10	MBNL2	ES	9	8	10	1.2E-16	0.369	0.133	2.5E-01	3.7E-01	1.9E-01
ESCA	MBNL2_ES_9:10_8_11	MBNL2	ES	9:10	8	11	5.2E-16	0.357	0.147	1.6E-01	2.0E-01	2.2E-01
ESCA	ME3_ES_10_8_11	ME3	ES	10	8	11	3.2E-04	0.103	0.246	2.9E-01	3.1E-01	2.1E-02
ESCA	MED11_AA_3.1_2.1_3.2	MED11	AA	3.1	2.1	3.2	1.5E-09	0.224	-0.110	2.6E-02	6.5E-01	2.8E-01
ESCA	MED24_ES_7_6_8	MED24	ES	7	6	8	4.4E-06	0.141	-0.109	2.8E-01	7.0E-01	5.2E-01
ESCA	METTL8_ES_2_1_3	METTL8	ES	2	1	3	9.6E-05	0.100	-0.074	5.4E-01	8.0E-01	6.5E-01
ESCA	METTL8_ES_3_2_4	METTL8	ES	3	2	4	8.9E-08	0.184	0.007	7.1E-01	4.3E-01	3.0E-01
ESCA	MEX3B_RI_1.2_1.1_1.3	MEX3B	RI	1.2	1.1	1.3	4.2E-07	0.186	-0.149	2.5E-01	5.9E-01	9.6E-01
ESCA	MFF_ES_8:9_7_10	MFF	ES	8:09	7	10	4.7E-13	0.299	0.046	5.8E-01	4.7E-01	6.3E-01
ESCA	MFF_ES_8:9:10_7_11	MFF	ES	8:09:10	7	11	1.7E-06	0.152	0.019	1.4E-01	5.4E-01	7.6E-01
ESCA	MFF_ES_9:10_7_11	MFF	ES	9:10	7	11	6.8E-05	0.111	0.144	3.5E-01	4.0E-01	3.9E-01
ESCA	MFSD10_ES_6.2_5.2_7	MFSD10	ES	6.2	5.2	7	1.7E-04	0.106	0.160	1.2E-03	1.6E-01	1.8E-01
ESCA	MGRN1_ES_12_11_13	MGRN1	ES	12	11	13	7.1E-11	0.254	-0.353	1.5E-01	3.6E-01	9.5E-01
ESCA	MKI67_ES_7_6_8	MKI67	ES	7	6	8	1.3E-04	0.103	-0.016	9.8E-02	4.4E-01	2.4E-01
ESCA	MLLT4_ES_16_15_17	MLLT4	ES	16	15	17	1.3E-49	0.755	-0.329	3.8E-01	5.2E-01	4.7E-01
ESCA	MLST8_ES_9:10.1_8.2_11.1	MLST8	ES	09:10.1	8.2	11.1	6.4E-06	0.148	0.041	8.8E-01	5.7E-01	9.4E-01
ESCA	MMRN2_AA_2.1_1_2.2	MMRN2	AA	2.1	1	2.2	6.9E-10	0.268	-0.111	8.8E-01	5.9E-01	4.0E-01
ESCA	MOCS1_ES_11.1:11.2_10_12.1	MOCS1	ES	11.1:11.2	10	12.1	2.2E-04	0.101	-0.054	6.6E-01	9.7E-01	8.8E-01
ESCA	MORF4L2_AA_5.1:5.2_3.2_5.3	MORF4L2	AA	5.1:5.2	3.2	5.3	6.3E-05	0.112	0.098	3.1E-02	8.3E-01	1.3E-01
ESCA	MORF4L2_AA_5.1:5.2_4_5.3	MORF4L2	AA	5.1:5.2	4	5.3	4.0E-05	0.117	0.137	2.1E-01	7.2E-01	8.7E-01
ESCA	MORF4L2_ES_4:5.1:5.2_3.2_5.3	MORF4L2	ES	4:5.1:5.2	3.2	5.3	3.6E-10	0.238	0.095	3.0E-01	5.6E-01	6.8E-01
ESCA	MPDU1_AD_5.3_5.2_7	MPDU1	AD	5.3	5.2	7	6.1E-06	0.142	-0.136	2.8E-01	4.5E-01	3.5E-01
ESCA	MPST_ES_1.2:2.2_1.1_3	MPST	ES	1.2:2.2	1.1	3	6.6E-07	0.163	0.125	8.2E-01	6.2E-01	4.7E-01
ESCA	MPV17_AA_6.1:6.2_3.2_6.3	MPV17	AA	6.1:6.2	3.2	6.3	9.7E-07	0.160	-0.192	9.0E-01	4.1E-01	4.3E-01
ESCA	MPV17_ES_6.1:6.2:6.3_3.2_7	MPV17	ES	6.1:6.2:6.3	3.2	7	7.7E-05	0.120	-0.180	3.5E-01	3.5E-01	5.8E-01

cancer type	id	Gene Symbol	splice_type	Exon	From.Exon	To.Exon	anova.p	adj.r2	r	p.50	p.25	p.10
ESCA	MROH1_ES_26_25.2_27	MROH1	ES	26	25.2	27	9.3E-07	0.158	-0.114	1.1E-02	1.8E-01	9.5E-01
ESCA	MRPL22_ES_2.1:2.2_1.3_3	MRPL22	ES	2.1:2.2	1.3	3	1.8E-06	0.160	0.032	9.3E-01	7.9E-01	6.1E-01
ESCA	MRPL33_ES_3_2_4	MRPL33	ES	3	2	4	9.2E-07	0.158	0.071	2.2E-01	7.2E-01	5.4E-01
ESCA	MRPL43_ES_4_3.1_5	MRPL43	ES	4	3.1	5	1.6E-12	0.289	-0.043	5.6E-02	1.2E-01	2.7E-02
ESCA	MRPL55_RI_2.7:2.8_2.6_2.9	MRPL55	RI	2.7:2.8	2.6	2.9	1.3E-05	0.129	-0.167	2.5E-01	8.5E-01	8.5E-01
ESCA	MRPS28_ES_4.2_1_5	MRPS28	ES	4.2	1	5	3.1E-05	0.137	-0.212	1.9E-01	1.5E-01	3.8E-02
ESCA	MSRB3_ES_3_1_4	MSRB3	ES	3	1	4	1.2E-04	0.116	0.225	4.7E-01	4.7E-01	9.1E-01
ESCA	MST1_RI_8.2_8.1_8.3	MST1	RI	8.2	8.1	8.3	4.8E-13	0.323	-0.391	1.2E-01	2.7E-01	5.8E-01
ESCA	MSTO1_AD_13.2_13.1_14	MSTO1	AD	13.2	13.1	14	5.7E-07	0.164	-0.112	1.4E-01	1.4E-01	3.0E-01
ESCA	MTCH1_AD_8.2_8.1_9	MTCH1	AD	8.2	8.1	9	2.5E-05	0.122	-0.096	6.3E-01	3.2E-01	3.2E-01
ESCA	MTFR1L_AA_10.2_9_10.3	MTFR1L	AA	10.2	9	10.3	2.9E-06	0.149	-0.042	9.5E-01	9.8E-01	8.6E-01
ESCA	MTFR1L_AA_10.2:10.3_9_10.4	MTFR1L	AA	10.2:10.3	9	10.4	2.2E-05	0.142	-0.062	8.1E-01	9.6E-01	7.3E-01
ESCA	MTSS1_ES_13_11_14.1	MTSS1	ES	13	11	14.1	1.3E-12	0.304	0.746	5.6E-01	3.4E-01	1.3E-01
ESCA	MTSS1L_ES_7_6_8	MTSS1L	ES	7	6	8	1.9E-05	0.125	0.191	2.1E-01	7.0E-01	4.1E-01
ESCA	MUC1_ES_4.1:4.2:4.3:5_3.4_7	MUC1	ES	4.1:4.2:4.3:5	3.4	7	6.2E-06	0.160	-0.196	8.6E-01	8.3E-01	8.0E-01
ESCA	MUTYH_AA_6.2:6.3:6.4_5_6.5	MUTYH	AA	6.2:6.3:6.4	5	6.5	3.9E-04	0.107	0.004	2.8E-01	4.4E-01	2.6E-01
ESCA	MUTYH_ES_6.2:6.3:6.4:6.5_5_7	MUTYH	ES	6.2:6.3:6.4:6.5	5	7	7.8E-08	0.223	-0.005	5.0E-01	3.6E-01	7.7E-01
ESCA	MYB_ES_17_16_19.1	MYB	ES	17	16	19.1	5.4E-08	0.198	-0.772	4.0E-01	3.4E-01	7.9E-01
ESCA	MYB_ES_17:18_16_19.1	MYB	ES	17:18	16	19.1	7.2E-08	0.195	-0.770	7.1E-01	9.9E-01	8.6E-01
ESCA	MYL6_ES_4.3_4.1_5	MYL6	ES	4.3	4.1	5	8.8E-31	0.580	-0.090	8.1E-02	3.0E-01	9.1E-01
ESCA	MYO18A_ES_12_11_13.2	MYO18A	ES	12	11	13.2	6.7E-09	0.229	0.465	2.8E-01	2.1E-01	3.6E-01
ESCA	MYO18A_ES_41_40_42	MYO18A	ES	41	40	42	3.5E-20	0.430	-0.302	4.6E-02	4.2E-02	9.7E-01
ESCA	MYO1B_ES_23:24_22_25	MYO1B	ES	23:24	22	25	7.1E-09	0.209	-0.282	9.8E-01	9.0E-01	7.3E-01
ESCA	MYO1B_ES_24_22_25	MYO1B	ES	24	22	25	2.7E-08	0.196	-0.417	1.4E-01	5.4E-01	7.4E-01
ESCA	MYO1B_ES_24_23_25	MYO1B	ES	24	23	25	1.6E-05	0.132	-0.181	5.0E-01	9.6E-01	7.4E-01
ESCA	MYO5A_ES_35_34_36	MYO5A	ES	35	34	36	2.7E-09	0.225	-0.416	8.4E-01	3.4E-01	8.7E-01
ESCA	MYO5B_ES_30_29_31	MYO5B	ES	30	29	31	9.6E-21	0.445	-0.382	6.9E-01	9.5E-01	4.2E-01
ESCA	MYO6_ES_29:30:31_28.1_32	MYO6	ES	29:30:31	28.1	32	9.4E-29	0.659	0.571	6.1E-01	4.1E-01	4.0E-01
ESCA	MYO6_ES_34_33_35	MYO6	ES	34	33	35	4.2E-17	0.377	-0.243	6.1E-01	9.5E-01	9.2E-01
ESCA	MYO9B_ES_37_36_38.1	MYO9B	ES	37	36	38.1	2.1E-11	0.266	-0.131	5.3E-01	2.0E-01	2.5E-02
ESCA	MYOF_ES_17_16_18	MYOF	ES	17	16	18	1.5E-11	0.268	-0.174	1.8E-01	6.2E-01	9.8E-01
ESCA	MZT2B_ES_2:3_1_4	MZT2B	ES	2:03	1	4	3.5E-05	0.118	-0.314	4.4E-01	4.9E-01	1.1E-01
ESCA	NABP1_AA_5.1_4_5.2	NABP1	AA	5.1	4	5.2	3.1E-10	0.240	-0.074	7.1E-01	7.8E-01	1.5E-01
ESCA	NABP1_ES_4:5.1_3_5.2	NABP1	ES	04:05.1	3	5.2	3.5E-06	0.144	0.155	3.7E-01	9.2E-01	3.8E-01
ESCA	NADK2_ES_10_9_11	NADK2	ES	10	9	11	1.3E-15	0.353	0.124	6.3E-01	9.0E-01	2.9E-01
ESCA	NADSYN1_ES_9.2:10_9.1_11	NADSYN1	ES	9.2:10	9.1	11	1.9E-05	0.125	-0.176	9.7E-01	8.7E-01	1.1E-01
ESCA	NAPA_ES_2:3:4_1_5	NAPA	ES	2:03:04	1	5	8.1E-05	0.110	-0.132	7.7E-02	2.1E-02	8.1E-01
ESCA	NCOA4_ES_2:5_1_6	NCOA4	ES	2:05	1	6	4.1E-07	0.187	-0.059	1.9E-02	1.7E-02	2.4E-01
ESCA	NCOR2_AD_46.2_46.1_47	NCOR2	AD	46.2	46.1	47	5.6E-27	0.531	-0.086	8.5E-01	6.9E-01	3.9E-01
ESCA	NCOR2_ES_46.1:46.2_45_47	NCOR2	ES	46.1:46.2	45	47	9.5E-23	0.483	-0.082	3.5E-01	4.4E-01	4.3E-01

cancer type	id	Gene Symbol	splice_type	Exon	From.Exon	To.Exon	anova.p	adj.r2	r	p.50	p.25	p.10
ESCA	NDEL1_ES_11_10_12.1	NDEL1	ES	11	10	12.1	1.7E-26	0.525	0.363	7.2E-01	4.0E-01	9.5E-01
ESCA	NDRG1_ES_1.2:2.1:2.2:2.3:3_1.1_4	NDRG1	ES	1.2:2.1:2.2:2.3:3	1.1	4	3.3E-04	0.102	-0.314	2.6E-01	2.0E-01	7.4E-01
ESCA	NDUFAF1_ES_4_3_5	NDUFAF1	ES	4	3	5	2.3E-06	0.149	-0.399	4.7E-01	7.4E-01	9.2E-01
ESCA	NDUFAF6_ES_10_9.2_11	NDUFAF6	ES	10	9.2	11	5.1E-05	0.114	-0.049	6.6E-01	3.0E-01	9.4E-01
ESCA	NDUFS5_AD_1.2_1.1_2	NDUFS5	AD	1.2	1.1	2	1.2E-04	0.104	-0.084	7.2E-01	2.0E-01	4.9E-01
ESCA	NEDD4L_ES_18_17_19	NEDD4L	ES	18	17	19	2.5E-23	0.479	0.281	9.0E-01	1.4E-01	9.7E-01
ESCA	NF1_ES_31_30_32	NF1	ES	31	30	32	9.1E-11	0.253	-0.311	2.2E-01	5.1E-01	8.8E-01
ESCA	NF2_ES_16.1_15_17	NF2	ES	16.1	15	17	1.3E-10	0.248	0.251	3.5E-01	7.7E-01	9.0E-01
ESCA	NFATC4_RI_12.2:12.3_12.1_12.4	NFATC4	RI	12.2:12.3	12.1	12.4	1.3E-04	0.103	0.011	3.8E-01	3.9E-01	4.7E-01
ESCA	NFYA_ES_3_2_4	NFYA	ES	3	2	4	2.3E-08	0.197	-0.348	1.7E-01	9.6E-02	2.4E-01
ESCA	NHSL1_ES_6_5_7	NHSL1	ES	6	5	7	6.2E-36	0.638	0.049	9.6E-01	6.8E-01	2.7E-01
ESCA	NKIRAS2_ES_5.5:5.6_5.3_7	NKIRAS2	ES	5.5:5.6	5.3	7	3.1E-11	0.264	-0.135	6.5E-01	9.9E-01	1.0E-01
ESCA	NKIRAS2_ES_5.6_5.3_7	NKIRAS2	ES	5.6	5.3	7	2.4E-06	0.167	-0.026	8.7E-01	6.5E-01	5.4E-01
ESCA	NMNAT3_ES_6:7_5_8	NMNAT3	ES	6:07	5	8	1.0E-04	0.122	-0.127	3.3E-01	4.8E-01	8.8E-02
ESCA	NOP2_AD_1.2_1.1_2.2	NOP2	AD	1.2	1.1	2.2	1.8E-07	0.176	0.066	4.3E-01	3.0E-01	7.8E-01
ESCA	NOSTRIN_ES_14_13_15	NOSTRIN	ES	14	13	15	3.2E-06	0.191	-0.562	2.0E-01	2.5E-01	7.0E-01
ESCA	NPHP3_ES_17_16_18	NPHP3	ES	17	16	18	1.4E-11	0.272	0.025	3.7E-02	1.9E-01	9.5E-01
ESCA	NPIPB4_AD_2.2:2.3_2.1_2.5	NPIPB4	AD	2.2:2.3	2.1	2.5	4.8E-06	0.161	-0.209	9.4E-01	3.9E-01	1.4E-01
ESCA	NPIPB4_ES_3.1_2.5_4	NPIPB4	ES	3.1	2.5	4	5.7E-08	0.193	0.219	7.2E-01	9.3E-01	9.2E-01
ESCA	NPIPB4_RI_2.4_2.3_2.5	NPIPB4	RI	2.4	2.3	2.5	4.5E-06	0.142	0.248	1.3E-01	4.3E-01	4.7E-01
ESCA	NPIPB5_AD_2.2:2.3_2.1_2.5	NPIPB5	AD	2.2:2.3	2.1	2.5	1.3E-05	0.130	-0.086	5.9E-01	2.9E-01	7.9E-02
ESCA	NPIPB5_ES_3.1_2.5_5	NPIPB5	ES	3.1	2.5	5	1.3E-07	0.179	0.195	8.7E-01	6.5E-01	7.3E-01
ESCA	NPIPB5_RI_2.4_2.3_2.5	NPIPB5	RI	2.4	2.3	2.5	3.0E-06	0.146	0.242	4.5E-01	3.4E-01	9.3E-01
ESCA	NPRL3_ES_2:3:4:5_1.4_6	NPRL3	ES	2:3:4:5	1.4	6	2.8E-05	0.145	-0.232	2.6E-01	6.5E-01	1.2E-01
ESCA	NPRL3_ES_3:4:5_2_6	NPRL3	ES	3:04:05	2	6	2.2E-06	0.149	-0.151	9.3E-01	9.6E-01	7.7E-01
ESCA	NRP1_ES_14.1_12_15.1	NRP1	ES	14.1	12	15.1	1.6E-04	0.103	-0.250	8.8E-01	6.0E-01	3.5E-01
ESCA	NSG1_AD_5.2:5.3_5.1_6	NSG1	AD	5.2:5.3	5.1	6	5.7E-07	0.166	-0.179	4.1E-01	8.3E-01	8.8E-02
ESCA	NTMT1_ES_4.1_2_5.1	NTMT1	ES	4.1	2	5.1	1.2E-06	0.159	-0.202	5.3E-01	7.3E-01	2.2E-01
ESCA	NUDT16L1_AD_2.2_2.1_2.4	NUDT16L1	AD	2.2	2.1	2.4	1.9E-05	0.125	0.041	4.6E-01	8.1E-01	1.8E-01
ESCA	NUMA1_ES_18_17_19	NUMA1	ES	18	17	19	1.2E-23	0.484	0.214	1.0E-01	3.1E-01	2.7E-01
ESCA	NUMA1_ES_4_3_5	NUMA1	ES	4	3	5	4.9E-06	0.140	-0.464	2.3E-01	1.1E-01	2.4E-01
ESCA	NUMB_ES_13_12_14	NUMB	ES	13	12	14	1.1E-13	0.312	0.345	2.5E-01	9.4E-01	5.2E-01
ESCA	NUMB_ES_7_6_8.2	NUMB	ES	7	6	8.2	2.7E-22	0.464	-0.279	5.1E-01	8.9E-01	8.9E-01
ESCA	OPA1_ES_7_6_8	OPA1	ES	7	6	8	1.2E-08	0.204	-0.224	4.3E-01	7.5E-01	5.5E-01
ESCA	OS9_ES_5.1:5.2:5.3:7.1:7.3:7.4:8:9.1_4_!	OS9	ES	.2:5.3:7.1:7.3:7.4	4	9.2	1.1E-05	0.149	-0.085	9.0E-01	2.9E-01	5.1E-01
ESCA	OS9_ES_5.1:5.2:5.3:7.1:7.4:8:9.1_4_9.2	OS9	ES	:5.2:5.3:7.1:7.4:8	4	9.2	1.1E-05	0.146	-0.073	7.2E-01	4.9E-01	5.0E-01
ESCA	OS9_ES_5.1:6:7.1:7.3:7.4:8:9.1_4_9.2	OS9	ES	l:6:7.1:7.3:7.4:8:!	4	9.2	6.6E-05	0.119	-0.094	7.9E-01	3.9E-01	6.3E-01
ESCA	OSBPL3_ES_9_8_10	OSBPL3	ES	9	8	10	1.8E-18	0.401	0.025	8.9E-01	4.6E-01	1.0E+00
ESCA	OSBPL8_ES_6_5_7.1	OSBPL8	ES	6	5	7.1	4.7E-06	0.141	-0.196	6.3E-01	3.6E-01	2.6E-01
ESCA	OSBPL8_ES_6:7.1_5_7.2	OSBPL8	ES	06:07.1	5	7.2	2.5E-06	0.150	-0.164	3.7E-01	1.5E-01	2.6E-01

cancer type	id	Gene Symbol	splice_type	Exon	From.Exon	To.Exon	anova.p	adj.r2	r	p.50	p.25	p.10
ESCA	OSBPL9_ES_17_15_18	OSBPL9	ES	17	15	18	7.4E-12	0.275	-0.184	7.1E-01	6.0E-01	9.1E-01
ESCA	PAK4_ES_2.2_1_4	PAK4	ES	2.2	1	4	3.6E-05	0.118	0.184	8.5E-01	2.1E-01	4.0E-01
ESCA	PAK4_ES_2.2:2.3_1_4	PAK4	ES	2.2:2.3	1	4	1.6E-04	0.101	0.187	9.8E-01	2.3E-01	9.6E-01
ESCA	PAPOLA_ES_20_18_21	PAPOLA	ES	20	18	21	2.9E-06	0.146	0.073	6.2E-01	6.4E-01	3.9E-01
ESCA	PAPSS2_ES_9_8_10	PAPSS2	ES	9	8	10	8.1E-07	0.164	0.346	3.6E-01	6.9E-01	1.7E-01
ESCA	PARD3_AD_19.2_19.1_20	PARD3	AD	19.2	19.1	20	5.6E-08	0.193	0.194	6.4E-01	5.7E-01	3.4E-02
ESCA	PARP11_ES_2_1_3	PARP11	ES	2	1	3	1.1E-06	0.164	-0.094	1.6E-01	4.3E-01	5.6E-01
ESCA	PARP8_ES_9_8_10	PARP8	ES	9	8	10	1.6E-04	0.105	0.209	8.9E-01	5.2E-01	3.5E-01
ESCA	PBX1_ES_11_10_12	PBX1	ES	11	10	12	2.8E-17	0.387	0.164	7.2E-01	6.6E-01	8.5E-01
ESCA	PCDHA4_RI_4.2_4.1_4.3	PCDHA4	RI	4.2	4.1	4.3	1.2E-04	0.133	-0.111	1.8E-01	6.4E-01	5.2E-01
ESCA	PDCD6_ES_6.1_4_7	PDCD6	ES	6.1	4	7	7.1E-04	0.101	-0.199	5.8E-01	6.9E-01	9.8E-02
ESCA	PDGFA_ES_6_5_7	PDGFA	ES	6	5	7	4.4E-24	0.490	0.222	7.6E-01	9.7E-01	6.1E-01
ESCA	PDP1_ES_2.2_1.1_3	PDP1	ES	2.2	1.1	3	3.4E-06	0.144	-0.121	3.9E-01	9.6E-01	8.6E-01
ESCA	PEX26_RI_1.2_1.1_1.3	PEX26	RI	1.2	1.1	1.3	1.8E-05	0.126	-0.083	7.6E-01	7.9E-01	9.8E-01
ESCA	PEX5_ES_9_8_10	PEX5	ES	9	8	10	5.8E-05	0.112	0.084	5.3E-02	5.3E-01	9.2E-01
ESCA	PFDN5_ES_2:4.1_1_4.2	PFDN5	ES	02:04.1	1	4.2	2.5E-04	0.121	0.030	6.6E-01	5.8E-01	9.3E-01
ESCA	PFDN5_ES_4.1:4.2:5_1_6.2	PFDN5	ES	4.1:4.2:5	1	6.2	2.9E-08	0.202	-0.170	4.2E-01	1.0E-01	6.2E-01
ESCA	PFDN5_ES_4.2:5_1_6.2	PFDN5	ES	4.2:5	1	6.2	1.8E-07	0.184	-0.184	1.8E-01	1.3E-01	4.7E-01
ESCA	PFKFB3_ES_17:18.1_15_20	PFKFB3	ES	17:18.1	15	20	6.2E-07	0.188	0.008	1.2E-01	7.4E-01	2.3E-01
ESCA	PFN2_AA_6.1_5.4_6.2	PFN2	AA	6.1	5.4	6.2	3.5E-11	0.262	-0.071	5.4E-01	4.0E-01	6.8E-01
ESCA	PGBD2_ES_3:4.1_2_4.2	PGBD2	ES	03:04.1	2	4.2	9.8E-05	0.118	0.140	3.3E-01	5.6E-01	1.7E-01
ESCA	PHF3_ES_3.2_1_5	PHF3	ES	3.2	1	5	6.5E-05	0.111	0.057	8.6E-02	1.4E-01	3.7E-01
ESCA	PHF3_ES_4_3.2_5	PHF3	ES	4	3.2	5	1.1E-06	0.161	0.237	3.3E-01	1.8E-01	6.4E-01
ESCA	PHLDB2_ES_14:15_13_16	PHLDB2	ES	14:15	13	16	9.8E-05	0.106	0.398	6.0E-01	8.5E-01	3.8E-01
ESCA	PHPT1_ES_4_3_5	PHPT1	ES	4	3	5	4.5E-05	0.115	-0.095	2.6E-01	1.4E-01	1.6E-01
ESCA	PICALM_ES_14.1:14.2_13_15	PICALM	ES	14.1:14.2	13	15	2.0E-05	0.124	0.025	8.3E-01	7.5E-01	7.9E-01
ESCA	PIGQ_ES_13_11_14	PIGQ	ES	13	11	14	3.2E-06	0.145	0.144	6.9E-01	8.6E-01	8.7E-01
ESCA	PILRB_RI_8.4_8.3_8.5	PILRB	RI	8.4	8.3	8.5	3.2E-11	0.261	0.148	5.7E-01	6.4E-01	6.7E-01
ESCA	PKP2_ES_6_5_7	PKP2	ES	6	5	7	1.3E-12	0.291	-0.828	6.4E-01	9.6E-01	4.0E-01
ESCA	PLA2G6_ES_11:12_10_13	PLA2G6	ES	11:12	10	13	7.4E-15	0.351	-0.044	2.5E-01	2.1E-01	6.9E-01
ESCA	PLCH2_ES_22.2:23.1_22.1_23.2	PLCH2	ES	22.2:23.1	22.1	23.2	1.3E-04	0.120	-0.352	6.0E-01	3.4E-01	1.9E-01
ESCA	PLD3_ES_4:5.1_1.2_5.2	PLD3	ES	04:05.1	1.2	5.2	4.7E-07	0.167	0.260	3.7E-01	3.6E-01	5.2E-01
ESCA	PLEKHM2_ES_7_6_8	PLEKHM2	ES	7	6	8	6.6E-11	0.254	0.435	1.3E-01	4.1E-01	6.1E-01
ESCA	PMPCB_RI_12.2_12.1_12.3	PMPCB	RI	12.2	12.1	12.3	7.4E-07	0.161	-0.066	6.7E-01	2.7E-01	6.5E-02
ESCA	POFUT2_RI_8.2:8.3:8.4_8.1_8.5	POFUT2	RI	8.2:8.3:8.4	8.1	8.5	1.9E-06	0.151	0.185	7.5E-01	6.0E-01	5.3E-01
ESCA	POGK_RI_6.2_6.1_6.3	POGK	RI	6.2	6.1	6.3	6.1E-07	0.163	-0.110	7.9E-01	5.7E-01	2.5E-01
ESCA	POLR2J3_AA_4.1:4.2_2_4.3	POLR2J3	AA	4.1:4.2	2	4.3	1.5E-08	0.201	-0.254	2.3E-03	6.0E-02	2.0E-01
ESCA	POSTN_ES_17:18:19_16_20	POSTN	ES	17:18:19	16	20	1.3E-10	0.259	0.145	3.3E-01	5.0E-01	6.3E-01
ESCA	POSTN_ES_21_20_22	POSTN	ES	21	20	22	1.2E-10	0.249	0.243	1.1E-01	6.6E-01	6.5E-01
ESCA	PPAP2A_ES_2_1_4	PPAP2A	ES	2	1	4	2.2E-04	0.104	0.119	6.3E-01	8.8E-01	2.5E-01

cancer type	id	Gene Symbol	splice_type	Exon	From.Exon	To.Exon	anova.p	adj.r2	r	p.50	p.25	p.10
ESCA	PPFIBP1_ES_20_19_21	PPFIBP1	ES	20	19	21	4.4E-06	0.141	0.004	2.2E-01	2.5E-01	3.1E-01
ESCA	PPIL2_AA_22.6_22.2_22.7	PPIL2	AA	22.6	22.2	22.7	6.1E-05	0.112	0.035	6.6E-02	2.2E-02	1.6E-01
ESCA	PPIP5K1_ES_27:28_26_29	PPIP5K1	ES	27:28:00	26	29	5.0E-07	0.165	0.222	8.8E-01	2.0E-01	7.7E-01
ESCA	PPIP5K1_ES_28_27_29	PPIP5K1	ES	28	27	29	1.1E-06	0.158	0.311	4.0E-02	2.0E-02	2.6E-01
ESCA	PPIP5K2_ES_26_25_27	PPIP5K2	ES	26	25	27	4.6E-27	0.532	-0.079	4.9E-01	1.3E-01	2.5E-01
ESCA	PPP3CB_ES_16_15.1_17	PPP3CB	ES	16	15.1	17	1.2E-04	0.104	-0.071	2.0E-03	4.2E-02	6.4E-01
ESCA	PQLC1_ES_6_5_9	PQLC1	ES	6	5	9	7.7E-07	0.160	0.057	1.3E-01	3.6E-01	5.2E-02
ESCA	PQLC1_ES_6:7:9_5_10	PQLC1	ES	6:07:09	5	10	2.1E-06	0.150	0.066	2.9E-01	6.2E-02	4.4E-01
ESCA	PRKRIP1_ES_6_4_7	PRKRIP1	ES	6	4	7	1.9E-06	0.154	0.129	7.4E-01	3.1E-01	9.8E-01
ESCA	PRKRIR_ES_3_2_4	PRKRIR	ES	3	2	4	7.1E-07	0.161	0.018	3.8E-01	9.5E-01	6.6E-01
ESCA	PRMT7_AA_17.1_16_17.2	PRMT7	AA	17.1	16	17.2	1.7E-06	0.152	-0.167	9.2E-01	2.4E-01	3.0E-01
ESCA	PRPF40A_ES_8_7_9	PRPF40A	ES	8	7	9	1.0E-26	0.528	-0.012	2.9E-01	2.5E-01	6.1E-01
ESCA	PRR13_AD_1.2_1.1_3	PRR13	AD	1.2	1.1	3	3.4E-10	0.239	-0.506	8.8E-01	8.2E-01	3.6E-01
ESCA	PRRC2C_ES_34_33_35	PRRC2C	ES	34	33	35	7.3E-09	0.209	0.137	1.9E-01	3.9E-01	8.1E-01
ESCA	PRRT2_RI_2.2_2.1_2.3	PRRT2	RI	2.2	2.1	2.3	2.1E-04	0.104	0.063	6.0E-02	4.5E-02	3.9E-01
ESCA	PSAP_AA_8.1_7_8.2	PSAP	AA	8.1	7	8.2	7.8E-21	0.440	-0.172	1.9E-01	2.1E-01	4.4E-01
ESCA	PSMB7_ES_6_4_7.1	PSMB7	ES	6	4	7.1	2.5E-08	0.234	0.545	9.2E-01	8.1E-01	3.7E-01
ESCA	PSMC3IP_AD_4.2:4.3_4.1_5.1	PSMC3IP	AD	4.2:4.3	4.1	5.1	6.0E-07	0.164	-0.388	9.9E-01	7.4E-01	2.1E-01
ESCA	PSMC3IP_AD_4.3_4.2_5.1	PSMC3IP	AD	4.3	4.2	5.1	3.8E-06	0.143	-0.380	7.9E-01	4.7E-01	8.5E-01
ESCA	PSMC5_RI_2.2_2.1_2.3	PSMC5	RI	2.2	2.1	2.3	8.9E-10	0.229	-0.222	3.3E-01	9.8E-01	9.9E-01
ESCA	PTGR2_AD_1.2:1.3_1.1_2	PTGR2	AD	1.2:1.3	1.1	2	1.4E-05	0.133	-0.253	1.8E-01	5.3E-01	7.9E-01
ESCA	PTK2_ES_39.2_37_39.5	PTK2	ES	39.2	37	39.5	8.2E-22	0.456	-0.029	3.9E-01	4.6E-01	3.7E-01
ESCA	PTK2_ME_5|6_1_8	PTK2	ME	5|6	1	8	1.3E-06	0.154	0.132	3.7E-01	5.0E-01	8.6E-01
ESCA	PTK2_RI_39.3:39.4_39.2_39.5	PTK2	RI	39.3:39.4	39.2	39.5	8.0E-05	0.109	-0.034	5.6E-01	3.4E-01	6.4E-01
ESCA	PTOV1_RI_13.2_13.1_13.3	PTOV1	RI	13.2	13.1	13.3	8.4E-07	0.159	-0.336	6.8E-01	5.2E-01	8.4E-01
ESCA	QKI_AA_8.5_8.2_8.6	QKI	AA	8.5	8.2	8.6	2.5E-12	0.286	-0.258	2.7E-02	3.7E-02	5.3E-02
ESCA	R3HDM2_ES_13_11_15	R3HDM2	ES	13	11	15	3.0E-05	0.120	-0.138	5.0E-01	7.1E-01	7.2E-01
ESCA	RAB11FIP3_ES_7_6_8	RAB11FIP3	ES	7	6	8	2.5E-08	0.196	0.111	6.8E-02	5.7E-01	6.7E-01
ESCA	RAB6A_ME_5|6_4_7	RAB6A	ME	5|6	4	7	7.5E-06	0.136	-0.103	9.3E-01	6.0E-01	6.9E-01
ESCA	RAD23A_ES_4.2_3_5	RAD23A	ES	4.2	3	5	1.5E-04	0.110	-0.160	5.7E-01	9.9E-01	7.7E-01
ESCA	RALGAPA1_ES_19_18_20.1	RALGAPA1	ES	19	18	20.1	7.2E-18	0.398	-0.220	8.3E-01	8.4E-01	8.8E-01
ESCA	RALGAPA1_ES_42_41_43	RALGAPA1	ES	42	41	43	3.5E-39	0.670	-0.163	4.1E-01	6.6E-01	8.8E-01
ESCA	RALGPS1_ES_16:17_15_18	RALGPS1	ES	16:17	15	18	1.3E-04	0.127	0.208	1.4E-02	6.3E-03	8.0E-02
ESCA	RANGAP1_ES_3_1_4.2	RANGAP1	ES	3	1	4.2	1.4E-04	0.122	-0.341	6.0E-01	3.6E-01	3.7E-01
ESCA	RAP1GAP2_ES_6_5_7	RAP1GAP2	ES	6	5	7	8.2E-06	0.157	0.105	2.3E-01	8.9E-01	4.2E-01
ESCA	RASEF_ES_5:6_2_7	RASEF	ES	5:06	2	7	7.9E-12	0.277	0.751	4.6E-01	2.1E-01	7.3E-01
ESCA	RBFOX2_ES_12_11.2_14	RBFOX2	ES	12	11.2	14	5.6E-05	0.113	-0.454	7.3E-01	4.3E-01	6.7E-01
ESCA	RBM26_ES_14_13.2_15	RBM26	ES	14	13.2	15	1.4E-14	0.330	-0.143	2.6E-02	1.0E-02	1.7E-01
ESCA	RBM42_ES_5:6.1_4_6.2	RBM42	ES	05:06.1	4	6.2	3.0E-05	0.120	-0.024	3.0E-01	2.1E-01	5.9E-01
ESCA	RBP7_ES_2_1_3	RBP7	ES	2	1	3	5.6E-05	0.120	-0.576	4.3E-01	4.1E-01	5.8E-01

cancer type	id	Gene Symbol	splice_type	Exon	From.Exon	To.Exon	anova.p	adj.r2	r	p.50	p.25	p.10
ESCA	RECQL5_ES_1.2:2.1_1.1_2.2	RECQL5	ES	1.2:2.1	1.1	2.2	8.1E-06	0.135	0.044	7.2E-01	7.8E-01	9.7E-01
ESCA	RELL2_ES_3_2_4	RELL2	ES	3	2	4	1.0E-07	0.206	0.389	2.9E-02	2.9E-01	3.2E-01
ESCA	REV1_RI_22.2_22.1_22.3	REV1	RI	22.2	22.1	22.3	3.6E-06	0.144	-0.127	5.6E-01	5.8E-01	2.6E-01
ESCA	RGPD5_ES_7_6_8	RGPD5	ES	7	6	8	9.9E-06	0.138	0.304	7.7E-01	4.3E-01	1.6E-01
ESCA	RGPD8_ES_7_6_8	RGPD8	ES	7	6	8	1.5E-06	0.159	0.134	6.7E-01	9.4E-01	2.0E-01
ESCA	RHNO1_ES_2.1_1_4	RHNO1	ES	2.1	1	4	2.7E-06	0.162	-0.223	8.8E-01	6.6E-01	4.0E-01
ESCA	RHOD_ES_2:3:4.1_1_4.2	RHOD	ES	03:04.1	1	4.2	9.9E-08	0.228	-0.376	6.9E-01	5.0E-01	7.3E-01
ESCA	RHOT1_ES_19.3_19.1_20	RHOT1	ES	19.3	19.1	20	1.5E-06	0.157	-0.027	3.5E-01	8.5E-01	1.5E-01
ESCA	RHOT1_ES_19.3_19.1_22	RHOT1	ES	19.3	19.1	22	2.3E-09	0.220	-0.105	9.4E-01	5.7E-01	9.9E-01
ESCA	RHOT1_ES_19.3:20_19.1_22	RHOT1	ES	19.3:20	19.1	22	1.6E-05	0.128	-0.061	5.0E-02	2.3E-03	3.0E-01
ESCA	RHOT1_ES_3_2.2_4	RHOT1	ES	3	2.2	4	2.9E-06	0.146	-0.192	9.3E-01	9.8E-01	3.3E-02
ESCA	RIMKLB_ES_3_2_4	RIMKLB	ES	3	2	4	2.7E-04	0.111	0.301	3.5E-01	9.6E-01	4.3E-01
ESCA	RNF181_ES_2_1_4.1	RNF181	ES	2	1	4.1	1.5E-04	0.102	-0.140	4.1E-01	5.2E-01	8.6E-01
ESCA	RNMT_ES_2.2_1_3.2	RNMT	ES	2.2	1	3.2	9.4E-06	0.133	0.285	7.9E-01	1.0E+00	9.2E-01
ESCA	RNPC3_AA_15.1_14_15.2	RNPC3	AA	15.1	14	15.2	9.9E-06	0.132	0.547	9.5E-01	9.3E-01	7.5E-01
ESCA	ROGDI_AD_4.2_4.1_5	ROGDI	AD	4.2	4.1	5	4.4E-17	0.377	-0.311	7.7E-02	1.1E-01	1.7E-01
ESCA	ROGDI_ES_4.1:4.2_3_5	ROGDI	ES	4.1:4.2	3	5	5.0E-17	0.381	-0.286	6.4E-01	9.3E-01	3.5E-01
ESCA	RPAIN_ES_4:5_3_7	RPAIN	ES	4:05	3	7	3.5E-07	0.169	-0.140	6.0E-01	6.6E-01	3.3E-01
ESCA	RPAIN_ES_4:5:6.1_3_7	RPAIN	ES	05:06.1	3	7	3.7E-08	0.192	-0.105	6.6E-02	8.2E-01	1.7E-01
ESCA	RPAIN_ES_5:6.1_3_7	RPAIN	ES	05:06.1	3	7	2.9E-06	0.147	-0.137	6.9E-01	8.1E-01	6.8E-01
ESCA	RPL18A_ES_2.2:2.4_1_3	RPL18A	ES	2.2:2.4	1	3	9.3E-07	0.159	-0.182	9.3E-01	9.7E-02	1.0E-01
ESCA	RPL37A_ES_6:7_4_8	RPL37A	ES	6:07	4	8	2.9E-08	0.222	-0.050	5.9E-01	6.3E-02	1.0E-01
ESCA	RPL5_ES_6_5_8	RPL5	ES	6	5	8	1.1E-04	0.107	0.071	3.8E-01	8.8E-01	3.3E-01
ESCA	RPRD2_ES_4_3.1_5	RPRD2	ES	4	3.1	5	4.0E-05	0.117	-0.149	8.1E-01	7.9E-01	7.8E-01
ESCA	RPS24_AA_5.1_4_5.2	RPS24	AA	5.1	4	5.2	5.7E-35	0.627	0.045	5.7E-01	2.7E-01	6.9E-01
ESCA	RPS24_ES_5.1:5.2_4_6	RPS24	ES	5.1:5.2	4	6	2.0E-24	0.495	-0.056	9.1E-01	8.8E-01	1.7E-01
ESCA	RPS6_RI_1.3_1.2_1.4	RPS6	RI	1.3	1.2	1.4	2.5E-07	0.173	0.057	5.5E-01	8.6E-01	9.7E-01
ESCA	RPS9_ES_4.1:4.2:4.3_3_4.5	RPS9	ES	4.1:4.2:4.3	3	4.5	1.3E-05	0.130	-0.227	9.7E-01	9.4E-01	7.4E-01
ESCA	RPS9_ES_4.1:4.3_3_4.5	RPS9	ES	4.1:4.3	3	4.5	6.6E-06	0.137	0.043	7.5E-01	8.0E-01	9.9E-01
ESCA	RPS9_ES_4.1:4.3:4.4_3_4.5	RPS9	ES	4.1:4.3:4.4	3	4.5	3.4E-06	0.144	-0.234	8.7E-01	6.0E-01	9.6E-01
ESCA	RRBP1_ES_3.3:3.4:4:5:7:8:9:10:11:12:13	RRBP1	ES	:9:10:11:12:13:1	3.2	19	2.7E-07	0.201	-0.278	8.4E-01	8.0E-01	3.7E-01
ESCA	RRBP1_ES_3.3:4:5:7:8:9:10:11:12:13:14:	RRBP1	ES	:10:11:12:13:14:	3.2	19	9.9E-09	0.237	-0.273	8.7E-01	7.8E-01	3.9E-01
ESCA	RTN2_ES_5_4.1_6	RTN2	ES	5	4.1	6	3.5E-05	0.120	-0.052	3.9E-01	2.0E-01	5.2E-01
ESCA	RTN4_ES_10_9_12	RTN4	ES	10	9	12	5.9E-08	0.208	-0.350	1.9E-01	1.8E-01	2.6E-01
ESCA	RWDD3_ES_2_1_3.1	RWDD3	ES	2	1	3.1	3.6E-07	0.168	0.001	5.5E-01	1.1E-01	1.0E-01
ESCA	SBF1_ES_29_28_30	SBF1	ES	29	28	30	3.5E-19	0.413	-0.006	2.2E-01	1.0E-01	1.6E-01
ESCA	SCOC_ES_4_3.2_6	SCOC	ES	4	3.2	6	6.5E-17	0.381	-0.118	5.8E-01	7.2E-01	3.2E-01
ESCA	SCP2_ES_12_11_13	SCP2	ES	12	11	13	3.9E-13	0.301	-0.514	3.4E-01	8.4E-01	3.8E-01
ESCA	SCRIB_ES_17_16_18	SCRIB	ES	17	16	18	2.4E-20	0.433	-0.391	9.0E-01	6.6E-01	3.8E-01
ESCA	SCRIB_ES_36_35_37	SCRIB	ES	36	35	37	5.0E-05	0.114	0.101	4.2E-02	3.7E-02	4.2E-02

cancer type	id	Gene Symbol	splice_type	Exon	From.Exon	To.Exon	anova.p	adj.r2	r	p.50	p.25	p.10
ESCA	SDC1_ES_2.2:3:4_1_5.1	SDC1	ES	2.2:3:4	1	5.1	2.0E-04	0.116	-0.442	8.3E-02	7.0E-01	9.8E-01
ESCA	SDC1_ES_4_1_5.1	SDC1	ES	4	1	5.1	1.4E-05	0.143	-0.589	8.7E-01	7.6E-01	9.7E-01
ESCA	SDCCAG8_ES_6_5_7	SDCCAG8	ES	6	5	7	1.7E-05	0.127	-0.056	4.8E-01	6.9E-01	7.4E-01
ESCA	SEC16A_ES_24:25_23.12_26	SEC16A	ES	24:25:00	23.12	26	2.0E-30	0.575	-0.450	3.8E-01	1.9E-01	8.9E-02
ESCA	SEC16A_ES_25_23.12_26	SEC16A	ES	25	23.12	26	5.3E-30	0.570	-0.347	7.2E-01	3.6E-01	2.0E-01
ESCA	SEC16A_ES_25_24_26	SEC16A	ES	25	24	26	7.0E-41	0.685	-0.454	9.7E-01	7.7E-01	9.0E-01
ESCA	SEC31A_ES_26.1:26.2:27_25.1_28	SEC31A	ES	26.1:26.2:27	25.1	28	9.9E-38	0.656	-0.036	6.1E-01	6.0E-01	1.5E-01
ESCA	SEC31A_ES_26.1:27_25.1_28	SEC31A	ES	26.1:27	25.1	28	4.8E-23	0.506	-0.041	7.0E-01	7.5E-01	3.2E-02
ESCA	SEC31A_ES_26.2:27_26.1_28	SEC31A	ES	26.2:27	26.1	28	6.5E-51	0.764	-0.003	5.7E-01	3.6E-01	7.4E-01
ESCA	SEC31A_ES_27_26.1_28	SEC31A	ES	27	26.1	28	6.9E-26	0.548	0.003	4.9E-01	9.3E-01	1.4E-01
ESCA	SEC31A_ES_27_26.2_28	SEC31A	ES	27	26.2	28	1.2E-36	0.645	0.044	8.4E-01	4.1E-01	6.0E-01
ESCA	SEMA3F_ES_7_6_8	SEMA3F	ES	7	6	8	9.8E-05	0.106	0.511	6.0E-01	6.0E-01	4.8E-01
ESCA	SEMA4G_RI_15.2_15.1_15.3	SEMA4G	RI	15.2	15.1	15.3	5.4E-06	0.157	-0.514	4.6E-01	3.7E-01	6.3E-01
ESCA	SENP6_ES_3_2_4	SENP6	ES	3	2	4	1.0E-04	0.106	-0.122	8.8E-02	2.1E-01	2.2E-01
ESCA	SEPT6_ES_12_11.1_13.1	SEPT6	ES	12	11.1	13.1	4.8E-18	0.395	0.058	6.5E-01	4.6E-01	2.7E-01
ESCA	SEPT6_ES_12_11.1_13.2	SEPT6	ES	12	11.1	13.2	5.7E-16	0.395	0.100	2.6E-01	4.4E-01	4.4E-01
ESCA	SEPT6_ES_12:13.1_11.1_13.2	SEPT6	ES	12:13.1	11.1	13.2	4.5E-14	0.320	0.113	8.8E-01	8.4E-02	1.4E-01
ESCA	SERHL2_AD_10.2_10.1_11	SERHL2	AD	10.2	10.1	11	1.1E-06	0.156	-0.476	1.4E-01	7.7E-01	5.5E-01
ESCA	SERPINA1_ES_1.2:2.1:2.2:2.3:2.4:2.5_1.1	SERPINA1	ES	2:2.1:2.2:2.3:2.4:2	1.1	3.2	1.5E-04	0.105	0.301	2.2E-02	2.3E-01	9.7E-01
ESCA	SERPINA1_ES_2.1:2.2:2.3:2.4:2.5_1.1_3.	SERPINA1	ES	2.1:2.2:2.3:2.4:2.5	1.1	3.2	1.4E-27	0.553	0.827	2.0E-02	5.9E-02	7.8E-01
ESCA	SERPINA1_ES_2.1:2.2:2.4:2.5_1.1_3.2	SERPINA1	ES	2.1:2.2:2.4:2.5	1.1	3.2	3.2E-07	0.176	0.459	2.9E-01	1.5E-01	2.0E-01
ESCA	SERPINA1_ES_2.1:2.4:2.5_1.1_3.2	SERPINA1	ES	2.1:2.4:2.5	1.1	3.2	1.6E-07	0.183	0.468	9.8E-01	9.0E-01	1.7E-01
ESCA	SERPINA1_ES_2.4:2.5_1.1_3.2	SERPINA1	ES	2.4:2.5	1.1	3.2	3.1E-21	0.455	0.698	5.2E-01	7.2E-01	7.3E-01
ESCA	SERPINA1_ES_2.5_1.1_3.2	SERPINA1	ES	2.5	1.1	3.2	8.1E-18	0.403	0.720	8.9E-01	5.6E-01	5.4E-01
ESCA	SERPINB8_ES_1.2:2_1.1_3	SERPINB8	ES	1.2:2	1.1	3	9.1E-05	0.111	0.103	5.2E-01	4.0E-01	5.8E-01
ESCA	SETD4_RI_13.2_13.1_13.3	SETD4	RI	13.2	13.1	13.3	1.1E-04	0.107	0.147	1.0E-01	2.2E-01	6.5E-01
ESCA	SETD5_ES_4_3_6	SETD5	ES	4	3	6	1.9E-05	0.125	-0.051	9.0E-01	4.4E-01	7.2E-01
ESCA	SEZ6L2_ES_15_14_16	SEZ6L2	ES	15	14	16	1.2E-06	0.158	0.202	2.3E-02	5.8E-01	4.0E-01
ESCA	SF1_ES_14.1_13_14.3	SF1	ES	14.1	13	14.3	6.9E-05	0.110	0.007	2.4E-01	3.9E-01	8.9E-01
ESCA	SFI1_ES_4:5_3_6	SFI1	ES	4:05	3	6	4.8E-06	0.145	0.032	3.4E-03	9.0E-02	4.1E-01
ESCA	SFI1_ES_5_3_6	SFI1	ES	5	3	6	1.2E-05	0.132	0.072	8.3E-01	2.0E-01	2.7E-01
ESCA	SFXN2_ES_8_7_9	SFXN2	ES	8	7	9	9.9E-05	0.109	0.382	7.7E-02	3.4E-01	6.3E-02
ESCA	SHROOM1_AA_3.1:3.2_2_3.3	SHROOM1	AA	3.1:3.2	2	3.3	2.8E-10	0.249	0.400	2.7E-01	2.1E-01	3.2E-01
ESCA	SHROOM1_ES_3.1_2_3.3	SHROOM1	ES	3.1	2	3.3	1.3E-05	0.133	0.308	7.4E-01	7.7E-01	5.6E-02
ESCA	SKA3_ES_1.2:2_1.1_3	SKA3	ES	1.2:2	1.1	3	2.4E-07	0.196	-0.133	9.8E-01	9.3E-01	5.5E-01
ESCA	SKA3_ES_2_1.1_3	SKA3	ES	2	1.1	3	3.2E-09	0.217	-0.096	9.9E-01	6.2E-01	9.5E-01
ESCA	SLAIN2_ES_8_6_9	SLAIN2	ES	8	6	9	6.0E-15	0.337	0.378	2.6E-02	8.7E-02	3.9E-01
ESCA	SLC12A8_ES_7_6_8	SLC12A8	ES	7	6	8	1.2E-06	0.178	0.184	8.4E-01	6.1E-01	2.8E-02
ESCA	SLC15A4_AA_3.2:3.3_2_3.4	SLC15A4	AA	3.2:3.3	2	3.4	1.6E-11	0.275	-0.156	6.9E-01	9.4E-01	4.4E-01
ESCA	SLC22A5_ES_8_7_9	SLC22A5	ES	8	7	9	9.5E-07	0.182	0.356	8.4E-01	9.8E-01	9.7E-01

cancer type	id	Gene Symbol	splice_type	Exon	From.Exon	To.Exon	anova.p	adj.r2	r	p.50	p.25	p.10
ESCA	SLC23A3_AD_5.2_5.1_6.1	SLC23A3	AD	5.2	5.1	6.1	1.4E-06	0.173	-0.450	9.2E-01	7.4E-01	2.1E-02
ESCA	SLC25A19_ES_3_1_4.2	SLC25A19	ES	3	1	4.2	8.1E-05	0.113	0.086	4.7E-01	7.8E-01	1.5E-01
ESCA	SLC25A29_AA_3.2:3.3:3.4:3.5:3.6_2_3.7	SLC25A29	AA	3.2:3.3:3.4:3.5:3.(	2	3.7	9.5E-05	0.109	0.335	6.2E-01	8.4E-01	3.0E-01
ESCA	SLC25A29_AA_3.4:3.5:3.6_2_3.7	SLC25A29	AA	3.4:3.5:3.6	2	3.7	5.3E-07	0.166	0.128	2.5E-02	3.9E-01	6.9E-01
ESCA	SLC25A29_ES_2:3.2:3.3:3.4:3.5:3.6_1_3.	SLC25A29	ES	:3.2:3.3:3.4:3.5:3	1	3.7	1.0E-05	0.164	0.006	3.9E-01	1.1E-01	3.5E-02
ESCA	SLC25A29_ES_2:3.4:3.5:3.6_1_3.7	SLC25A29	ES	2:3.4:3.5:3.6	1	3.7	3.8E-04	0.103	-0.028	8.7E-01	4.3E-01	1.1E-01
ESCA	SLC25A45_ES_8_7_10	SLC25A45	ES	8	7	10	1.5E-05	0.132	-0.145	9.9E-01	3.3E-01	6.2E-01
ESCA	SLC2A8_ES_2:3_1_4	SLC2A8	ES	2:03	1	4	1.7E-11	0.267	0.274	4.4E-02	6.0E-01	1.0E+00
ESCA	SLC2A9_ES_6_5_7	SLC2A9	ES	6	5	7	1.2E-04	0.125	0.548	3.5E-01	3.0E-01	9.6E-01
ESCA	SLC35C1_RI_1.3_1.2_1.4	SLC35C1	RI	1.3	1.2	1.4	9.5E-05	0.107	-0.392	7.8E-01	3.4E-01	1.0E-01
ESCA	SLC37A2_ES_12_11_13	SLC37A2	ES	12	11	13	6.1E-15	0.353	-0.244	5.8E-01	7.2E-01	1.3E-01
ESCA	SLC37A2_ES_18_17_19	SLC37A2	ES	18	17	19	6.0E-10	0.257	0.561	6.7E-01	2.1E-01	5.5E-01
ESCA	SLC39A14_ME_5|6_4_7	SLC39A14	ME	5|6	4	7	1.5E-12	0.289	0.331	6.6E-01	7.4E-01	6.2E-01
ESCA	SLC52A2_ES_3.1_2.4_5	SLC52A2	ES	3.1	2.4	5	4.2E-10	0.270	0.295	7.0E-01	2.6E-01	6.2E-01
ESCA	SLC52A2_ES_3.1:4.1_2.4_5	SLC52A2	ES	3.1:4.1	2.4	5	4.8E-11	0.295	0.285	4.5E-01	9.7E-01	5.3E-01
ESCA	SLC9A8_ES_7.1:7.2_6_8	SLC9A8	ES	7.1:7.2	6	8	3.6E-06	0.153	0.345	9.3E-01	7.0E-01	2.3E-01
ESCA	SLC9A8_ES_7.2_6_8	SLC9A8	ES	7.2	6	8	2.2E-07	0.174	0.366	3.4E-01	8.5E-01	3.8E-01
ESCA	SLK_ES_13_12_14	SLK	ES	13	12	14	5.0E-07	0.165	0.403	7.9E-01	5.7E-01	4.6E-01
ESCA	SLMAP_ES_25_24_26	SLMAP	ES	25	24	26	1.4E-14	0.330	-0.194	1.6E-01	8.2E-01	2.0E-01
ESCA	SMARCD1_ES_5_4_7.1	SMARCD1	ES	5	4	7.1	5.7E-11	0.256	-0.181	7.2E-01	4.7E-01	9.8E-01
ESCA	SMARCD2_ES_1.2:3_1.1_4	SMARCD2	ES	1.2:3	1.1	4	1.7E-09	0.254	-0.045	2.0E-01	9.4E-01	2.8E-01
ESCA	SMARCD3_RI_11.2_11.1_11.3	SMARCD3	RI	11.2	11.1	11.3	8.0E-05	0.109	-0.580	5.0E-01	5.7E-01	5.1E-01
ESCA	SMC5_ES_19_18_20	SMC5	ES	19	18	20	1.2E-11	0.276	-0.298	3.5E-02	3.7E-01	8.3E-01
ESCA	SMG7_AD_16.2_16.1_17	SMG7	AD	16.2	16.1	17	3.7E-06	0.143	0.115	9.3E-01	6.2E-01	3.8E-01
ESCA	SMPDL3A_ES_2_1_3	SMPDL3A	ES	2	1	3	6.5E-06	0.137	0.351	8.3E-02	1.6E-01	5.5E-01
ESCA	SNRPB2_AD_1.2_1.1_2	SNRPB2	AD	1.2	1.1	2	3.9E-07	0.168	0.008	4.9E-01	5.8E-01	5.5E-01
ESCA	SNUPN_AD_1.2_1.1_4	SNUPN	AD	1.2	1.1	4	4.0E-06	0.142	0.026	9.2E-01	4.2E-01	7.9E-01
ESCA	SNX11_ES_2_1.2_3.1	SNX11	ES	2	1.2	3.1	7.5E-06	0.136	0.131	9.5E-02	5.1E-02	2.8E-03
ESCA	SNX14_ES_16_15_17	SNX14	ES	16	15	17	2.0E-06	0.150	-0.315	6.5E-01	6.5E-01	8.2E-01
ESCA	SNX27_AA_12.1_11_12.2	SNX27	AA	12.1	11	12.2	1.4E-04	0.102	-0.027	6.5E-01	2.3E-01	4.7E-01
ESCA	SOCS4_ES_3_1_4	SOCS4	ES	3	1	4	2.8E-05	0.121	-0.040	4.1E-01	1.6E-01	2.0E-02
ESCA	SOCS7_ES_3_2_5	SOCS7	ES	3	2	5	3.4E-06	0.145	0.064	3.7E-01	5.8E-01	4.7E-01
ESCA	SORBS1_ES_20_19_23	SORBS1	ES	20	19	23	1.3E-08	0.226	0.332	2.9E-01	8.6E-01	1.0E+00
ESCA	SORBS1_ES_27_26_28	SORBS1	ES	27	26	28	1.7E-09	0.226	0.092	3.1E-01	2.1E-01	5.7E-01
ESCA	SORBS1_ES_9.1:9.2_8_10	SORBS1	ES	9.1:9.2	8	10	7.1E-21	0.445	0.058	3.5E-01	4.0E-01	8.4E-01
ESCA	SP110_ES_20_19_21	SP110	ES	20	19	21	5.5E-06	0.179	0.003	1.3E-01	2.4E-01	1.9E-01
ESCA	SP110_ES_20:21_19_22	SP110	ES	20:21	19	22	3.2E-04	0.113	0.136	3.8E-01	6.2E-01	4.7E-01
ESCA	SPATA20_AD_4.3_4.2_6	SPATA20	AD	4.3	4.2	6	1.6E-04	0.101	-0.485	1.2E-02	1.1E-01	9.5E-01
ESCA	SPHK2_RI_3.2_3.1_3.3	SPHK2	RI	3.2	3.1	3.3	1.6E-07	0.177	-0.489	4.7E-01	8.6E-01	3.9E-02
ESCA	SPINT1_AD_5.2_5.1_6	SPINT1	AD	5.2	5.1	6	8.2E-05	0.108	-0.311	1.0E+00	9.3E-01	8.8E-01

cancer type	id	Gene Symbol	splice_type	Exon	From.Exon	To.Exon	anova.p	adj.r2	r	p.50	p.25	p.10
ESCA	SREBF1_ES_2_1_3.2	SREBF1	ES	2	1	3.2	1.7E-11	0.267	0.672	1.4E-01	7.4E-01	1.0E+00
ESCA	SRPK2_ES_19_18_20	SRPK2	ES	19	18	20	4.5E-08	0.199	0.349	8.2E-01	7.5E-01	4.4E-01
ESCA	SRPX_ES_3_2_4	SRPX	ES	3	2	4	3.1E-04	0.103	-0.295	8.7E-01	3.9E-01	8.7E-01
ESCA	SRSF11_AA_3.2_1_3.3	SRSF11	AA	3.2	1	3.3	6.9E-06	0.136	-0.014	5.9E-01	8.6E-01	3.8E-01
ESCA	SRSF4_ES_2:3:4_1_5	SRSF4	ES	2:03:04	1	5	1.6E-05	0.127	-0.048	3.2E-01	5.8E-01	8.9E-01
ESCA	SRSF7_RI_4.2_4.1_4.3	SRSF7	RI	4.2	4.1	4.3	2.7E-08	0.195	-0.048	5.0E-01	7.5E-01	8.0E-01
ESCA	SRSF7_RI_4.5_4.4_4.6	SRSF7	RI	4.5	4.4	4.6	2.8E-07	0.171	0.002	8.2E-01	5.2E-01	7.1E-01
ESCA	SSBP4_ES_7_6_8	SSBP4	ES	7	6	8	2.7E-05	0.121	0.121	3.3E-01	5.0E-01	8.6E-01
ESCA	SSPN_AD_2.2_2.1_3	SSPN	AD	2.2	2.1	3	2.6E-04	0.112	0.059	5.2E-01	7.0E-01	8.9E-01
ESCA	ST3GAL5_ES_3_1_5	ST3GAL5	ES	3	1	5	7.7E-05	0.121	-0.176	4.7E-01	9.3E-01	4.2E-01
ESCA	ST7_ES_10_9_11.1	ST7	ES	10	9	11.1	1.9E-13	0.308	-0.028	8.1E-01	1.2E-01	2.2E-01
ESCA	STK40_ES_2_1_3	STK40	ES	2	1	3	1.3E-06	0.155	-0.117	9.8E-01	5.1E-01	8.0E-01
ESCA	STOML1_ES_8.2_7_9	STOML1	ES	8.2	7	9	1.9E-04	0.100	0.175	3.0E-01	9.4E-02	6.4E-02
ESCA	STRADA_RI_12.4_12.3_12.5	STRADA	RI	12.4	12.3	12.5	2.3E-06	0.148	-0.112	5.0E-01	4.3E-01	2.4E-01
ESCA	STRADA_RI_12.6_12.5_12.7	STRADA	RI	12.6	12.5	12.7	7.9E-05	0.109	-0.032	5.7E-01	4.1E-01	3.6E-01
ESCA	STT3A_ES_2:3_1_4	STT3A	ES	2:03	1	4	1.3E-04	0.115	0.118	1.2E-02	6.7E-02	4.7E-01
ESCA	STX1A_AD_8.2_8.1_9.1	STX1A	AD	8.2	8.1	9.1	3.8E-08	0.198	0.288	9.0E-01	1.5E-01	3.0E-01
ESCA	STX2_ES_10_9_11	STX2	ES	10	9	11	1.0E-08	0.209	0.071	4.4E-01	2.0E-02	2.9E-01
ESCA	STYK1_ES_2_1_3	STYK1	ES	2	1	3	2.8E-05	0.141	0.381	9.9E-01	2.9E-01	7.8E-01
ESCA	SUCO_ES_13_12_14	SUCO	ES	13	12	14	1.8E-05	0.127	-0.114	2.7E-01	1.3E-01	2.1E-01
ESCA	SULT1A2_AA_5.1_4_5.2	SULT1A2	AA	5.1	4	5.2	2.8E-04	0.108	-0.229	5.2E-01	2.5E-01	8.8E-01
ESCA	SULT1A2_RI_1.2:1.3_1.1_1.4	SULT1A2	RI	1.2:1.3	1.1	1.4	4.7E-09	0.214	-0.637	3.8E-01	8.8E-01	8.2E-01
ESCA	SUN1_ES_13_11_14	SUN1	ES	13	11	14	1.2E-05	0.146	0.107	4.9E-01	6.5E-01	8.3E-01
ESCA	SUPT20H_AA_21.1_20_21.2	SUPT20H	AA	21.1	20	21.2	8.9E-07	0.159	0.112	9.2E-01	7.7E-01	5.2E-01
ESCA	SUPT20H_AA_21.1:21.2_20_21.3	SUPT20H	AA	21.1:21.2	20	21.3	1.5E-05	0.128	0.102	7.5E-01	4.6E-01	9.6E-01
ESCA	SYNE2_ES_118_117.2_119.2	SYNE2	ES	118	117.2	119.2	7.1E-56	0.795	0.183	3.4E-01	2.3E-01	4.7E-01
ESCA	SYT8_RI_7.2_7.1_7.3	SYT8	RI	7.2	7.1	7.3	1.1E-04	0.113	-0.368	2.4E-01	3.5E-01	7.7E-02
ESCA	SYTL2_AA_11.1_10.3_11.2	SYTL2	AA	11.1	10.3	11.2	5.1E-13	0.345	0.609	1.2E-01	1.2E-01	6.4E-01
ESCA	SYTL2_AD_8.3_8.2_9	SYTL2	AD	8.3	8.2	9	6.4E-19	0.415	0.556	4.6E-01	5.5E-02	9.1E-02
ESCA	SYTL2_ES_11.2_10.3_12.2	SYTL2	ES	11.2	10.3	12.2	2.4E-29	0.605	-0.722	1.7E-01	5.4E-01	1.1E-01
ESCA	SYTL2_ES_13_12.2_14	SYTL2	ES	13	12.2	14	3.5E-20	0.446	0.599	4.1E-01	7.0E-02	1.8E-01
ESCA	SYTL2_RI_10.2_10.1_10.3	SYTL2	RI	10.2	10.1	10.3	9.4E-06	0.157	0.298	7.0E-01	3.3E-01	7.9E-01
ESCA	TACC2_ES_5.2:8_5.1_11	TACC2	ES	5.2:8	5.1	11	5.7E-04	0.103	-0.215	7.7E-01	8.8E-01	4.1E-01
ESCA	TAF1C_ES_2.2:3_1_4	TAF1C	ES	2.2:3	1	4	2.6E-04	0.103	-0.088	2.1E-01	6.9E-01	9.9E-01
ESCA	TAMM41_ES_5_4_7	TAMM41	ES	5	4	7	1.5E-04	0.101	0.115	3.5E-01	5.2E-01	7.2E-01
ESCA	TAMM41_ES_9.2:10_9.1_11.1	TAMM41	ES	9.2:10	9.1	11.1	6.7E-05	0.111	0.154	3.9E-01	5.3E-01	4.3E-01
ESCA	TANGO2_ES_7.2_6_8	TANGO2	ES	7.2	6	8	1.0E-05	0.132	0.177	1.3E-01	9.8E-01	9.1E-01
ESCA	TBC1D13_ES_3_2_4	TBC1D13	ES	3	2	4	2.1E-10	0.249	0.049	3.8E-01	7.7E-01	1.7E-01
ESCA	TBC1D15_ES_10_9_11	TBC1D15	ES	10	9	11	1.4E-19	0.420	0.081	7.1E-01	8.5E-01	3.3E-01
ESCA	TBC1D23_ES_15_14_16	TBC1D23	ES	15	14	16	2.8E-27	0.535	0.122	2.4E-01	6.3E-01	8.5E-01

cancer type	id	Gene Symbol	splice_type	Exon	From.Exon	To.Exon	anova.p	adj.r2	r	p.50	p.25	p.10
ESCA	TBRG1_ES_3:5_2_6	TBRG1	ES	3:05	2	6	1.4E-04	0.104	-0.219	5.5E-01	2.8E-01	8.0E-01
ESCA	TBRG4_ES_4_3_6	TBRG4	ES	4	3	6	2.2E-07	0.184	-0.010	1.8E-01	5.7E-01	1.9E-01
ESCA	TC2N_AD_2.2_2.1_3	TC2N	AD	2.2	2.1	3	3.2E-06	0.147	0.323	5.4E-01	6.6E-01	7.5E-01
ESCA	TCF12_ES_18_17_19	TCF12	ES	18	17	19	1.5E-23	0.483	0.347	5.3E-01	4.6E-01	4.3E-01
ESCA	TCF3_ES_17.3_17.1_18.1	TCF3	ES	17.3	17.1	18.1	1.7E-04	0.102	-0.171	2.0E-01	8.1E-01	8.9E-01
ESCA	TCF3_ES_17.3_17.1_18.2	TCF3	ES	17.3	17.1	18.2	2.7E-04	0.115	-0.303	9.0E-01	4.3E-01	4.8E-01
ESCA	TCF3_ES_17.3:18.1_17.1_18.2	TCF3	ES	17.3:18.1	17.1	18.2	4.7E-07	0.170	-0.191	1.2E-01	1.6E-01	6.3E-01
ESCA	TCF7L2_ES_17_14_19	TCF7L2	ES	17	14	19	6.1E-05	0.112	0.372	9.5E-01	8.7E-01	1.2E-01
ESCA	TCF7L2_ES_4_3_5	TCF7L2	ES	4	3	5	5.7E-06	0.139	0.380	7.1E-01	9.2E-01	7.3E-01
ESCA	TCIRG1_ES_18_17_19	TCIRG1	ES	18	17	19	1.1E-11	0.289	0.001	8.0E-01	8.6E-01	7.9E-01
ESCA	TEAD1_ES_6_5_7.2	TEAD1	ES	6	5	7.2	3.0E-16	0.396	0.105	4.8E-01	9.1E-01	1.6E-01
ESCA	TECR_ES_2:4_1_5.3	TECR	ES	2:04	1	5.3	4.7E-07	0.193	0.160	6.2E-01	3.7E-01	6.0E-01
ESCA	TEX30_RI_3.3_3.2_3.4	TEX30	RI	3.3	3.2	3.4	2.2E-05	0.127	-0.038	6.7E-01	3.3E-01	2.1E-01
ESCA	TEX9_AD_1.4_1.3_2	TEX9	AD	1.4	1.3	2	4.0E-04	0.104	-0.089	1.6E-01	3.6E-01	6.7E-01
ESCA	TFDP2_ES_4_3.1_6	TFDP2	ES	4	3.1	6	9.4E-06	0.134	-0.006	8.3E-01	1.7E-01	2.3E-01
ESCA	TGIF1_ES_4.3_4.1_7.2	TGIF1	ES	4.3	4.1	7.2	1.2E-10	0.250	0.008	4.8E-01	2.7E-01	8.4E-01
ESCA	THNSL2_ES_9_8_11	THNSL2	ES	9	8	11	6.4E-05	0.138	-0.243	6.1E-01	6.3E-01	1.8E-02
ESCA	THYN1_AD_1.2_1.1_1.4	THYN1	AD	1.2	1.1	1.4	3.5E-05	0.118	-0.041	2.0E-01	5.4E-01	9.6E-01
ESCA	TIA1_ES_6_5_7	TIA1	ES	6	5	7	1.2E-05	0.131	-0.014	8.2E-01	7.5E-01	1.9E-01
ESCA	TIA1_ES_8_7_9.1	TIA1	ES	8	7	9.1	1.0E-11	0.272	-0.012	3.5E-01	5.5E-01	8.0E-01
ESCA	TINAGL1_RI_5.2_5.1_5.3	TINAGL1	RI	5.2	5.1	5.3	1.4E-08	0.202	0.003	5.1E-01	1.4E-01	8.1E-01
ESCA	TJP1_AD_30.2_30.1_31	TJP1	AD	30.2	30.1	31	7.7E-17	0.372	-0.360	9.3E-01	4.8E-01	2.8E-01
ESCA	TJP1_ES_23_22_24	TJP1	ES	23	22	24	2.4E-27	0.536	0.139	7.9E-01	5.5E-01	4.0E-01
ESCA	TLK2_AA_4.1_2_4.2	TLK2	AA	4.1	2	4.2	1.3E-04	0.103	0.070	6.0E-01	5.6E-01	6.2E-01
ESCA	TMCO6_AD_1.3_1.2_2.1	TMCO6	AD	1.3	1.2	2.1	4.0E-06	0.148	0.062	7.8E-01	7.3E-01	2.6E-01
ESCA	TMCO6_RI_4.2_4.1_4.3	TMCO6	RI	4.2	4.1	4.3	6.3E-08	0.187	-0.082	9.2E-01	6.1E-01	7.1E-01
ESCA	TMCO6_RI_4.4_4.3_4.5	TMCO6	RI	4.4	4.3	4.5	1.5E-07	0.178	-0.099	3.0E-01	6.8E-01	5.1E-01
ESCA	TMEM120A_ES_10_9_11	TMEM120A	ES	10	9	11	1.2E-04	0.104	0.369	1.6E-01	6.3E-01	4.5E-01
ESCA	TMEM143_ES_4_2_5	TMEM143	ES	4	2	5	1.6E-04	0.102	0.053	7.2E-01	8.1E-01	1.6E-01
ESCA	TMEM171_AA_4.1_3_4.2	TMEM171	AA	4.1	3	4.2	7.1E-05	0.119	0.015	3.9E-01	5.8E-01	8.7E-01
ESCA	TMEM175_ES_2:3_1_5.1	TMEM175	ES	2:03	1	5.1	2.4E-07	0.173	-0.048	9.7E-01	4.7E-01	1.7E-01
ESCA	TMEM176A_AD_1.2_1.1_2.2	TMEM176A	AD	1.2	1.1	2.2	9.0E-05	0.108	0.187	8.3E-01	8.8E-01	8.8E-01
ESCA	TMEM180_ES_6_5_7	TMEM180	ES	6	5	7	3.7E-14	0.322	0.129	6.1E-01	3.1E-01	9.8E-01
ESCA	TMEM205_AD_2.2:2.3_2.1_2.6	TMEM205	AD	2.2:2.3	2.1	2.6	2.3E-05	0.127	-0.246	6.3E-01	5.6E-02	1.8E-01
ESCA	TMEM205_RI_2.4:2.5_2.3_2.6	TMEM205	RI	2.4:2.5	2.3	2.6	1.2E-04	0.104	-0.010	7.8E-02	6.0E-01	7.0E-01
ESCA	TMEM91_RI_7.2:7.3_7.1_7.4	TMEM91	RI	7.2:7.3	7.1	7.4	1.2E-06	0.156	-0.390	4.0E-01	2.6E-01	1.0E+00
ESCA	TMPO_ES_7_5.1_8	TMPO	ES	7	5.1	8	4.4E-08	0.209	0.251	7.4E-01	7.6E-01	6.7E-01
ESCA	TMPO_ES_7:8_5.1_9	TMPO	ES	7:08	5.1	9	2.3E-10	0.249	0.043	9.1E-01	1.7E-01	5.9E-01
ESCA	TMPO_ES_8_5.1_9	TMPO	ES	8	5.1	9	1.6E-23	0.526	-0.195	2.5E-01	2.1E-01	7.6E-01
ESCA	TMUB2_AA_4.2_3_4.3	TMUB2	AA	4.2	3	4.3	2.1E-08	0.201	-0.099	7.3E-01	9.2E-01	7.5E-01

cancer type	id	Gene Symbol	splice_type	Exon	From.Exon	To.Exon	anova.p	adj.r2	r	p.50	p.25	p.10
ESCA	TMUB2_ES_2.2:2.3:2.4:2.5:3:4.2_2.1_4.3	TMUB2	ES	.2:2.3:2.4:2.5:3:4	2.1	4.3	5.7E-05	0.113	-0.019	4.8E-01	4.1E-01	3.9E-01
ESCA	TMUB2_ES_3:4.2_2.5_4.3	TMUB2	ES	03:04.2	2.5	4.3	5.7E-08	0.188	-0.128	6.9E-01	9.6E-01	6.9E-01
ESCA	TMX2_AA_3.1_2_3.2	TMX2	AA	3.1	2	3.2	5.1E-05	0.117	-0.168	1.0E-01	1.1E-01	7.6E-01
ESCA	TNIK_ES_22_21.1_23	TNIK	ES	22	21.1	23	1.1E-05	0.174	0.134	9.6E-02	1.4E-01	1.2E-01
ESCA	TOM1L1_ES_3.3:4_2_6.1	TOM1L1	ES	3.3:4	2	6.1	3.8E-05	0.122	0.037	6.1E-01	1.4E-01	1.8E-01
ESCA	TOP3B_RI_15.2_15.1_15.3	TOP3B	RI	15.2	15.1	15.3	5.1E-05	0.114	0.119	1.5E-01	1.8E-01	9.6E-01
ESCA	TPM1_AD_12.2_12.1_13.1	TPM1	AD	12.2	12.1	13.1	1.0E-15	0.360	0.112	9.9E-01	8.4E-01	5.6E-01
ESCA	TPM1_ES_12.1_11.1_13.1	TPM1	ES	12.1	11.1	13.1	1.4E-12	0.351	-0.188	6.4E-01	9.5E-01	2.9E-01
ESCA	TPM1_ES_12.1:12.2_11.1_13.1	TPM1	ES	12.1:12.2	11.1	13.1	4.4E-04	0.101	0.020	8.4E-01	8.7E-01	5.8E-01
ESCA	TPM3_ES_12_11_13	TPM3	ES	12	11	13	2.1E-04	0.103	-0.175	8.4E-01	8.4E-01	9.9E-01
ESCA	TRA2B_ES_2_1_3	TRA2B	ES	2	1	3	5.2E-06	0.140	0.489	3.9E-01	7.9E-01	6.6E-01
ESCA	TREX1_RI_2.2:2.3_2.1_2.4	TREX1	RI	2.2:2.3	2.1	2.4	5.7E-05	0.113	0.017	3.5E-01	5.5E-01	5.0E-01
ESCA	TRO_AD_12.2:12.3_12.1_13	TRO	AD	12.2:12.3	12.1	13	1.5E-05	0.128	-0.403	2.5E-01	8.5E-01	3.8E-01
ESCA	TROAP_RI_12.4_12.3_12.5	TROAP	RI	12.4	12.3	12.5	1.3E-07	0.179	0.052	3.2E-01	5.5E-01	1.9E-01
ESCA	TSC2_AA_28.1_27_28.2	TSC2	AA	28.1	27	28.2	4.1E-06	0.154	0.036	9.6E-02	4.9E-02	4.7E-01
ESCA	TSC2_ES_27_26_28.1	TSC2	ES	27	26	28.1	4.9E-06	0.140	0.053	7.1E-01	2.7E-01	4.4E-01
ESCA	TSC2_ES_27_26_28.2	TSC2	ES	27	26	28.2	1.5E-06	0.166	0.003	3.7E-01	4.9E-01	5.4E-01
ESCA	TTC28_ES_20_19_21	TTC28	ES	20	19	21	7.3E-06	0.152	0.053	6.1E-01	9.3E-01	8.3E-02
ESCA	TTLL3_RI_1.2_1.1_1.3	TTLL3	RI	1.2	1.1	1.3	5.4E-05	0.125	0.109	5.2E-01	7.3E-01	4.5E-01
ESCA	TTLL5_ES_17_16_18	TTLL5	ES	17	16	18	1.4E-04	0.102	-0.140	6.0E-01	2.2E-01	4.8E-01
ESCA	TUBB4A_ES_5_3.1_6.1	TUBB4A	ES	5	3.1	6.1	2.6E-06	0.147	-0.829	7.1E-01	6.9E-01	9.8E-01
ESCA	TUBGCP4_ES_14_13.2_15	TUBGCP4	ES	14	13.2	15	2.8E-06	0.147	-0.261	7.4E-01	3.9E-01	3.4E-01
ESCA	TUFT1_ES_2_1_3	TUFT1	ES	2	1	3	1.4E-09	0.227	-0.194	4.5E-01	1.1E-02	2.3E-01
ESCA	TUFT1_ES_3_1_4	TUFT1	ES	3	1	4	2.7E-20	0.432	0.275	9.9E-01	7.0E-01	2.7E-01
ESCA	TUSC3_ES_11_10_12	TUSC3	ES	11	10	12	2.7E-05	0.122	-0.467	6.5E-01	5.2E-01	4.3E-01
ESCA	TXNRD1_ES_6.2_4.2_10	TXNRD1	ES	6.2	4.2	10	1.3E-07	0.188	0.029	9.3E-02	6.8E-01	8.8E-01
ESCA	U2AF1L4_ES_8_7_9.1	U2AF1L4	ES	8	7	9.1	1.2E-05	0.130	-0.105	5.7E-01	8.2E-01	5.1E-01
ESCA	UAP1_ES_9.2_8_10	UAP1	ES	9.2	8	10	1.7E-08	0.200	-0.177	7.1E-02	9.1E-02	4.9E-01
ESCA	UBALD1_RI_2.4_2.3_2.5	UBALD1	RI	2.4	2.3	2.5	1.5E-06	0.153	0.198	6.4E-01	4.3E-02	1.4E-01
ESCA	UBALD1_RI_2.4:2.5_2.3_2.6	UBALD1	RI	2.4:2.5	2.3	2.6	1.1E-05	0.131	0.149	1.7E-01	7.5E-02	3.3E-01
ESCA	UBE2I_AD_4.3_4.2_5.2	UBE2I	AD	4.3	4.2	5.2	5.9E-05	0.114	0.038	3.8E-01	4.7E-01	4.0E-02
ESCA	UBE2J2_ES_2.1:2.2_1.1_5	UBE2J2	ES	2.1:2.2	1.1	5	4.3E-05	0.118	-0.123	4.1E-01	9.6E-01	1.6E-01
ESCA	UBE2J2_ME_3|4_2.1_5	UBE2J2	ME	3|4	2.1	5	2.0E-04	0.120	-0.028	1.8E-01	4.8E-01	1.6E-01
ESCA	UBL5_ES_3_2.1_4	UBL5	ES	3	2.1	4	5.8E-06	0.147	-0.061	9.1E-01	4.0E-01	6.6E-01
ESCA	UBOX5_ES_4_3_5	UBOX5	ES	4	3	5	2.3E-06	0.149	-0.004	7.1E-01	2.5E-01	1.2E-01
ESCA	UBXN11_ES_6_5_7	UBXN11	ES	6	5	7	5.9E-06	0.139	-0.007	3.8E-02	3.7E-01	5.2E-01
ESCA	UIMC1_AD_6.2_6.1_7.1	UIMC1	AD	6.2	6.1	7.1	6.9E-06	0.138	0.118	1.3E-01	4.0E-02	1.4E-01
ESCA	UIMC1_ES_6.2:7.1:7.2_6.1_8	UIMC1	ES	6.2:7.1:7.2	6.1	8	4.4E-07	0.200	-0.026	3.1E-01	3.8E-01	5.6E-01
ESCA	UNC5B_ES_8_7_9	UNC5B	ES	8	7	9	8.3E-15	0.339	0.528	2.5E-01	8.3E-01	5.6E-01
ESCA	UPF3B_ES_8_7_9	UPF3B	ES	8	7	9	1.9E-27	0.540	-0.043	4.2E-01	5.0E-01	2.2E-01

cancer type	id	Gene Symbol	splice_type	Exon	From.Exon	To.Exon	anova.p	adj.r2	r	p.50	p.25	p.10
ESCA	USO1_ES_14_13_15	USO1	ES	14	13	15	5.0E-11	0.257	0.054	4.0E-01	8.2E-01	5.6E-01
ESCA	USP21_ES_2_1_3	USP21	ES	2	1	3	5.9E-07	0.163	-0.188	5.4E-01	9.8E-01	9.3E-02
ESCA	VAPB_ES_5_2_6	VAPB	ES	5	2	6	1.3E-04	0.125	-0.225	4.0E-01	5.4E-01	6.4E-01
ESCA	VAV3_ES_19_18.2_21	VAV3	ES	19	18.2	21	5.7E-05	0.127	-0.325	2.0E-01	4.0E-01	5.4E-01
ESCA	VEGFA_AD_7.2:7.3_7.1_8.1	VEGFA	AD	7.2:7.3	7.1	8.1	2.9E-08	0.204	0.186	4.6E-01	6.5E-01	3.9E-01
ESCA	VEZT_ES_6.1:6.2_5_9	VEZT	ES	6.1:6.2	5	9	1.1E-05	0.132	-0.155	5.0E-01	4.1E-01	4.1E-01
ESCA	VPS29_ES_3.1_1_5	VPS29	ES	3.1	1	5	1.4E-12	0.290	-0.210	3.4E-01	8.8E-01	9.1E-01
ESCA	VPS29_ES_3.1:3.2_1_5	VPS29	ES	3.1:3.2	1	5	5.9E-10	0.233	-0.243	9.8E-01	8.7E-01	7.7E-01
ESCA	VPS37A_ES_3:4_1_5	VPS37A	ES	3:04	1	5	6.9E-04	0.101	-0.032	1.7E-01	4.6E-01	2.9E-01
ESCA	VPS39_ES_3_2_4	VPS39	ES	3	2	4	1.7E-42	0.700	0.003	5.7E-01	6.2E-01	4.8E-01
ESCA	VTI1B_ES_2:3_1_4	VTI1B	ES	2:03	1	4	3.3E-06	0.145	-0.244	3.4E-01	7.4E-01	7.7E-01
ESCA	VWA9_AD_1.2_1.1_2.1	VWA9	AD	1.2	1.1	2.1	7.7E-06	0.136	-0.065	6.9E-01	6.3E-01	7.9E-01
ESCA	WDFY3_ES_15_14_16	WDFY3	ES	15	14	16	1.3E-12	0.306	-0.097	7.8E-01	5.8E-01	4.2E-01
ESCA	WDR27_ES_20_19_21	WDR27	ES	20	19	21	4.7E-04	0.102	0.008	9.5E-01	2.1E-01	1.7E-01
ESCA	WDR41_ES_3_2_4	WDR41	ES	3	2	4	8.0E-06	0.135	-0.597	2.5E-01	6.9E-02	5.5E-01
ESCA	WDR45B_ES_3:4_2_5	WDR45B	ES	3:04	2	5	2.6E-04	0.103	-0.069	5.9E-01	6.4E-01	4.2E-01
ESCA	WDR6_RI_4.5_4.4_4.6	WDR6	RI	4.5	4.4	4.6	2.3E-05	0.123	0.243	9.6E-01	7.2E-01	6.0E-01
ESCA	WDR74_RI_3.3:3.4_3.2_3.5	WDR74	RI	3.3:3.4	3.2	3.5	1.3E-04	0.118	-0.001	6.4E-01	4.0E-01	9.2E-01
ESCA	WIBG_ES_4_2_5	WIBG	ES	4	2	5	1.6E-04	0.109	-0.096	2.4E-01	8.5E-01	3.2E-01
ESCA	WIPI2_ES_2_1_3	WIPI2	ES	2	1	3	1.6E-08	0.201	0.052	9.5E-01	6.8E-01	8.3E-01
ESCA	WNK1_ES_12:13.1_9_13.2	WNK1	ES	12:13.1	9	13.2	8.0E-06	0.135	-0.207	7.5E-01	8.4E-01	4.6E-01
ESCA	WNK1_ES_15_13.2_16	WNK1	ES	15	13.2	16	3.8E-16	0.359	-0.262	8.3E-01	5.7E-01	4.4E-02
ESCA	WNK2_ES_25.1:25.2:25.3:26:27_24_28	WNK2	ES	5.1:25.2:25.3:26:2	24	28	2.9E-10	0.276	0.136	6.1E-02	1.9E-01	3.9E-01
ESCA	WNK2_ES_26:27_25.3_28	WNK2	ES	26:27:00	25.3	28	2.2E-15	0.383	-0.020	1.0E-02	3.3E-01	7.9E-01
ESCA	WSB1_RI_5.2_5.1_5.3	WSB1	RI	5.2	5.1	5.3	8.9E-05	0.108	-0.027	1.4E-01	2.4E-01	5.8E-01
ESCA	XRCC3_ES_2.2_1.1_3.2	XRCC3	ES	2.2	1.1	3.2	5.0E-04	0.101	-0.004	4.0E-01	7.8E-01	8.2E-01
ESCA	XRCC4_AA_8.1_7_8.2	XRCC4	AA	8.1	7	8.2	1.4E-07	0.180	-0.071	8.9E-01	9.3E-01	6.2E-01
ESCA	YIPF1_ES_3_2_4	YIPF1	ES	3	2	4	1.2E-13	0.311	-0.060	2.6E-01	3.6E-01	9.7E-01
ESCA	YPEL3_RI_2.2_2.1_2.3	YPEL3	RI	2.2	2.1	2.3	2.3E-06	0.148	0.082	9.5E-01	6.9E-01	8.7E-01
ESCA	YWHAZ_AD_5.2_5.1_7.1	YWHAZ	AD	5.2	5.1	7.1	2.0E-04	0.102	-0.074	1.6E-01	3.5E-01	2.8E-01
ESCA	ZBTB43_ES_2_1_3.2	ZBTB43	ES	2	1	3.2	7.1E-05	0.111	-0.222	5.8E-01	8.5E-01	4.5E-01
ESCA	ZCCHC10_ES_5.1:5.2_4_6.1	ZCCHC10	ES	5.1:5.2	4	6.1	2.2E-04	0.102	-0.039	9.4E-01	2.4E-01	1.3E-01
ESCA	ZCCHC11_AA_26.1_25_26.2	ZCCHC11	AA	26.1	25	26.2	4.0E-05	0.124	0.028	1.3E-01	3.4E-03	5.5E-02
ESCA	ZFAND5_ES_3_1_4.1	ZFAND5	ES	3	1	4.1	1.1E-13	0.313	-0.292	9.2E-01	4.6E-01	4.3E-01
ESCA	ZFAND5_ES_3_1_4.2	ZFAND5	ES	3	1	4.2	1.2E-04	0.111	-0.220	6.5E-01	5.8E-01	3.6E-01
ESCA	ZMYM2_ES_3_2_4.1	ZMYM2	ES	3	2	4.1	6.0E-07	0.163	0.129	5.3E-01	4.9E-01	3.8E-01
ESCA	ZMYM2_RI_4.2_4.1_4.3	ZMYM2	RI	4.2	4.1	4.3	2.0E-05	0.124	-0.002	8.2E-02	2.8E-01	2.5E-02
ESCA	ZMYND8_AD_19.2_19.1_20	ZMYND8	AD	19.2	19.1	20	5.3E-08	0.188	-0.091	4.2E-01	4.0E-01	2.8E-01
ESCA	ZNF131_RI_7.3_7.2_7.4	ZNF131	RI	7.3	7.2	7.4	7.9E-06	0.136	-0.057	3.9E-01	6.6E-01	2.4E-01
ESCA	ZNF195_ES_2:3_1_4	ZNF195	ES	2:03	1	4	1.1E-06	0.158	0.226	9.8E-01	2.2E-01	3.4E-01

cancer type	id	Gene Symbol	splice_type	Exon	From.Exon	To.Exon	anova.p	adj.r2	r	p.50	p.25	p.10
ESCA	ZNF195_ES_3_1_4	ZNF195	ES	3	1	4	5.1E-05	0.115	0.291	3.7E-01	9.1E-01	7.1E-01
ESCA	ZNF195_ES_9_5.1_11	ZNF195	ES	9	5.1	11	5.4E-09	0.220	0.368	9.7E-01	8.1E-01	8.5E-01
ESCA	ZNF248_RI_7.2_7.1_7.3	ZNF248	RI	7.2	7.1	7.3	1.0E-05	0.132	-0.040	5.2E-01	6.8E-01	4.0E-01
ESCA	ZNF263_ES_5.1:6_2_7	ZNF263	ES	5.1:6	2	7	1.1E-04	0.113	-0.012	4.9E-01	7.2E-01	9.5E-01
ESCA	ZNF263_ES_6_2_7	ZNF263	ES	6	2	7	5.8E-06	0.141	0.076	1.9E-01	1.4E-01	1.4E-01
ESCA	ZNF276_RI_12.2_12.1_12.3	ZNF276	RI	12.2	12.1	12.3	2.4E-10	0.242	-0.101	4.0E-01	5.1E-01	3.4E-03
ESCA	ZNF418_AD_5.2_5.1_6	ZNF418	AD	5.2	5.1	6	1.3E-05	0.153	-0.185	9.4E-01	9.4E-01	5.8E-01
ESCA	ZNF426_RI_1.2_1.1_1.3	ZNF426	RI	1.2	1.1	1.3	1.0E-04	0.110	0.043	7.3E-01	5.8E-01	7.6E-01
ESCA	ZNF438_ES_6_4_7	ZNF438	ES	6	4	7	8.3E-05	0.117	-0.012	3.7E-01	3.4E-01	5.3E-01
ESCA	ZNF550_ES_5.1:5.2_4_6	ZNF550	ES	5.1:5.2	4	6	6.6E-06	0.137	0.000	4.7E-01	6.7E-01	9.8E-01
ESCA	ZNF576_AD_1.2:1.3_1.1_1.6	ZNF576	AD	1.2:1.3	1.1	1.6	8.1E-05	0.127	-0.021	3.5E-01	1.6E-01	3.0E-01
ESCA	ZNF586_ES_3_1_4	ZNF586	ES	3	1	4	1.1E-05	0.134	0.044	2.0E-01	4.7E-01	5.5E-01
ESCA	ZNF655_ES_4:5.3_3.2_9	ZNF655	ES	04:05.3	3.2	9	4.2E-04	0.112	0.264	8.7E-01	9.8E-01	9.4E-01
ESCA	ZNF655_ES_4:9_3.2_10	ZNF655	ES	4:09	3.2	10	2.4E-04	0.124	-0.199	8.6E-01	2.8E-01	8.4E-01
ESCA	ZNF655_ES_8:9_3.2_10	ZNF655	ES	8:09	3.2	10	7.1E-07	0.193	-0.556	7.0E-01	9.8E-03	5.6E-01
ESCA	ZNF655_ES_9_3.2_10	ZNF655	ES	9	3.2	10	6.1E-05	0.129	-0.327	2.3E-01	2.8E-01	7.2E-01
ESCA	ZNF669_AD_1.2_1.1_2.1	ZNF669	AD	1.2	1.1	2.1	2.1E-06	0.156	-0.017	5.9E-01	7.8E-01	7.7E-01
ESCA	ZNF678_ES_4_3_5.1	ZNF678	ES	4	3	5.1	6.7E-09	0.232	0.068	2.5E-01	8.1E-01	6.5E-01
ESCA	ZNF707_ES_5.2_1_6	ZNF707	ES	5.2	1	6	6.3E-05	0.119	0.022	9.0E-01	6.0E-01	4.5E-01
ESCA	ZNF714_AA_4.1_3_4.2	ZNF714	AA	4.1	3	4.2	1.4E-04	0.110	-0.256	8.3E-01	6.1E-01	6.9E-01
ESCA	ZNF83_ES_8:9.6_7_10.1	ZNF83	ES	08:09.6	7	10.1	6.7E-04	0.103	0.155	4.6E-01	7.9E-01	7.7E-01
GBM	ADAM15_ES_21.1:21.2:22.1:22.2_20_23	ADAM15	ES	1.1:21.2:22.1:22.	20	23	1.9E-04	0.121	-0.161	1.0E-01	2.0E-01	2.8E-01
GBM	ADAM15_ES_21.2:22.1:22.2_20_23	ADAM15	ES	21.2:22.1:22.2	20	23	1.5E-04	0.124	-0.227	1.0E+00	5.6E-01	9.4E-01
GBM	ADORA1_ES_4_3.2_5	ADORA1	ES	4	3.2	5	3.2E-04	0.116	-0.086	2.0E-01	1.0E-01	5.1E-02
GBM	AGTRAP_AA_4.1_3_4.2	AGTRAP	AA	4.1	3	4.2	9.3E-04	0.104	-0.312	4.7E-02	7.9E-02	2.1E-01
GBM	ALG13_ES_25_24_26	ALG13	ES	25	24	26	1.4E-04	0.126	0.111	7.0E-01	4.9E-01	4.8E-01
GBM	ANXA2_ES_6:7_5_8.1	ANXA2	ES	6:07	5	8.1	4.8E-04	0.130	-0.195	8.0E-01	5.4E-01	6.0E-01
GBM	ARHGAP23_ES_24_23_25	ARHGAP23	ES	24	23	25	9.8E-04	0.102	0.285	6.6E-01	6.4E-01	7.9E-01
GBM	ARL6IP4_AD_1.3_1.2_2.1	ARL6IP4	AD	1.3	1.2	2.1	7.0E-05	0.134	-0.012	2.0E-02	2.4E-01	6.1E-01
GBM	ATP5D_RI_4.2:4.3_4.1_4.4	ATP5D	RI	4.2:4.3	4.1	4.4	2.5E-04	0.117	-0.132	2.1E-01	3.2E-01	1.4E-01
GBM	ATXN7_ES_15_14_16	ATXN7	ES	15	14	16	9.0E-04	0.128	-0.128	1.3E-01	2.0E-01	7.0E-01
GBM	B4GALT4_ES_4_3.2_5	B4GALT4	ES	4	3.2	5	5.4E-04	0.108	-0.249	8.7E-01	2.3E-01	1.1E-01
GBM	BMP1_AA_18.1_16_18.2	BMP1	AA	18.1	16	18.2	3.1E-04	0.114	0.241	9.2E-01	2.2E-01	6.5E-01
GBM	C18orf32_AD_1.2_1.1_3	C18orf32	AD	1.2	1.1	3	4.9E-08	0.220	0.016	3.3E-02	1.2E-01	3.6E-01
GBM	C1D_ES_2.2_1.1_3.1	C1D	ES	2.2	1.1	3.1	1.6E-10	0.284	-0.053	5.1E-01	9.0E-01	9.3E-01
GBM	C6orf1_AD_1.2_1.1_2.1	C6orf1	AD	1.2	1.1	2.1	4.7E-12	0.315	-0.040	9.6E-01	7.6E-01	4.5E-01
GBM	CA5B_ES_4_3_5	CA5B	ES	4	3	5	3.2E-04	0.114	0.682	3.7E-01	4.3E-01	7.3E-01
GBM	CADPS2_ES_19_18_20	CADPS2	ES	19	18	20	5.5E-05	0.170	-0.091	NA	NA	NA
GBM	CC2D2A_ES_5_4_6	CC2D2A	ES	5	4	6	2.4E-04	0.118	-0.533	1.0E-01	4.2E-01	7.9E-01
GBM	CD46_ES_13_12_14	CD46	ES	13	12	14	5.7E-04	0.106	0.087	6.0E-01	3.7E-01	8.5E-01

cancer type	id	Gene Symbol	splice_type	Exon	From.Exon	To.Exon	anova.p	adj.r2	r	p.50	p.25	p.10
GBM	CDCA3_ES_4_3.2_5	CDCA3	ES	4	3.2	5	3.6E-04	0.114	-0.177	4.1E-01	3.4E-01	2.4E-01
GBM	CERS5_ES_17.1:17.2_16_18	CERS5	ES	17.1:17.2	16	18	8.6E-08	0.214	-0.138	6.1E-01	4.6E-01	9.0E-01
GBM	CHEK1_RI_13.2_13.1_13.3	CHEK1	RI	13.2	13.1	13.3	6.4E-05	0.135	0.004	8.9E-01	7.3E-01	4.8E-01
GBM	CLDND1_ES_3.3:3.4_1_4.1	CLDND1	ES	3.3:3.4	1	4.1	6.9E-04	0.103	0.470	5.8E-01	7.9E-01	8.7E-01
GBM	CNTROB_AD_17.2_17.1_18.2	CNTROB	AD	17.2	17.1	18.2	6.5E-04	0.106	0.155	3.9E-01	5.2E-01	9.2E-01
GBM	COASY_RI_1.2:1.3:1.4_1.1_1.5	COASY	RI	1.2:1.3:1.4	1.1	1.5	3.8E-04	0.111	-0.332	6.6E-01	4.6E-01	2.6E-01
GBM	COX11_RI_3.2:3.3_3.1_3.4	COX11	RI	3.2:3.3	3.1	3.4	2.1E-05	0.149	0.115	2.7E-02	4.0E-02	2.1E-03
GBM	CXorf40A_AD_1.2:1.3_1.1_1.6	CXorf40A	AD	1.2:1.3	1.1	1.6	9.8E-06	0.165	-0.044	8.5E-01	5.6E-01	1.3E-01
GBM	CXorf40A_AD_1.2:1.3:1.4_1.1_1.6	CXorf40A	AD	1.2:1.3:1.4	1.1	1.6	4.4E-06	0.169	-0.002	8.2E-01	4.4E-01	3.7E-01
GBM	DNASE1L1_ES_2.2_1_3.2	DNASE1L1	ES	2.2	1	3.2	4.0E-04	0.126	-0.004	8.5E-01	4.5E-01	4.0E-01
GBM	DRAM2_AD_1.2:1.3_1.1_2	DRAM2	AD	1.2:1.3	1.1	2	5.4E-05	0.137	-0.194	8.3E-01	3.7E-01	4.2E-01
GBM	DRAM2_AD_1.3_1.2_2	DRAM2	AD	1.3	1.2	2	6.2E-07	0.191	-0.171	2.6E-01	4.8E-01	1.6E-01
GBM	DTNB_ES_17_16_19	DTNB	ES	17	16	19	6.3E-04	0.105	0.343	1.1E-01	1.7E-01	8.2E-01
GBM	EED_ES_8:9:10_7_11.2	EED	ES	8:09:10	7	11.2	4.3E-04	0.113	-0.097	6.1E-01	4.2E-01	1.0E-01
GBM	FAHD2B_ES_3:4:5:6_1_7	FAHD2B	ES	3:4:5:6	1	7	1.9E-07	0.226	0.463	3.4E-01	1.2E-02	1.3E-01
GBM	FAM126A_ES_11_10_12	FAM126A	ES	11	10	12	8.8E-05	0.133	-0.112	7.6E-01	3.6E-01	9.4E-01
GBM	FAM221A_ES_3.1:3.2:3.3_2_4	FAM221A	ES	3.1:3.2:3.3	2	4	2.5E-04	0.117	-0.631	4.9E-01	7.6E-01	8.5E-01
GBM	FAM86B1_AD_7.2:7.3_7.1_8.1	FAM86B1	AD	7.2:7.3	7.1	8.1	1.2E-04	0.128	-0.464	3.7E-01	2.4E-01	1.5E-01
GBM	FAM86B1_ES_7.3_7.1_8.1	FAM86B1	ES	7.3	7.1	8.1	3.9E-04	0.121	-0.101	5.0E-02	5.2E-01	8.5E-01
GBM	FNBP1L_RI_16.2_16.1_16.3	FNBP1L	RI	16.2	16.1	16.3	3.5E-04	0.112	-0.133	5.7E-01	3.0E-01	9.7E-01
GBM	FYN_ES_11_10_12	FYN	ES	11	10	12	7.3E-04	0.122	0.207	1.7E-02	5.6E-03	6.3E-03
GBM	GMPR2_RI_2.2_2.1_2.3	GMPR2	RI	2.2	2.1	2.3	6.3E-04	0.105	0.186	9.9E-01	5.1E-01	2.6E-01
GBM	HYAL2_RI_1.4_1.3_1.5	HYAL2	RI	1.4	1.3	1.5	2.2E-04	0.119	-0.270	3.1E-01	3.8E-01	2.7E-01
GBM	IDUA_RI_7.2_7.1_7.3	IDUA	RI	7.2	7.1	7.3	6.0E-04	0.109	0.142	7.0E-01	7.1E-01	7.0E-01
GBM	KCTD17_ES_7_6_8	KCTD17	ES	7	6	8	6.7E-04	0.106	-0.031	8.2E-01	5.7E-01	4.6E-01
GBM	KCTD7_ES_7_5_10	KCTD7	ES	7	5	10	3.2E-04	0.120	-0.024	3.5E-01	1.6E-01	1.4E-01
GBM	LGALS8_ES_11_10.1_12	LGALS8	ES	11	10.1	12	3.1E-05	0.146	-0.053	7.8E-01	2.9E-01	8.5E-01
GBM	LPPR4_ES_6_5_7	LPPR4	ES	6	5	7	9.5E-05	0.136	-0.005	8.8E-01	4.6E-01	3.2E-01
GBM	MAGI1_ES_24_23.1_25	MAGI1	ES	24	23.1	25	2.5E-05	0.168	0.038	2.1E-01	5.9E-01	7.1E-01
GBM	MANEAL_AA_4.1_3_4.2	MANEAL	AA	4.1	3	4.2	1.8E-04	0.122	-0.293	7.4E-01	2.1E-01	9.8E-01
GBM	MAP4_AA_24.1_23_24.2	MAP4	AA	24.1	23	24.2	3.1E-04	0.115	-0.171	1.0E+00	8.1E-01	6.1E-01
GBM	MARK3_ES_18_17_19	MARK3	ES	18	17	19	8.3E-04	0.101	0.043	6.9E-01	3.1E-01	6.1E-01
GBM	MBP_ES_5.3:5.4:6:8.1:8.2_5.2_8.3	MBP	ES	5.3:5.4:6:8.1:8.2	5.2	8.3	3.0E-04	0.130	-0.365	2.2E-01	5.6E-02	9.4E-01
GBM	MFF_ES_3:4:5_1_6	MFF	ES	3:04:05	1	6	1.5E-04	0.123	-0.052	1.5E-01	7.4E-01	8.2E-01
GBM	MORN1_ES_2_1_3	MORN1	ES	2	1	3	5.9E-04	0.113	0.191	7.4E-03	1.5E-01	2.9E-01
GBM	MRPL52_RI_1.3_1.2_1.4	MRPL52	RI	1.3	1.2	1.4	2.3E-06	0.176	-0.076	7.1E-01	3.3E-01	8.8E-01
GBM	MRPL55_ES_1.2:2.2_1.1_2.9	MRPL55	ES	1.2:2.2	1.1	2.9	2.3E-04	0.119	0.006	1.6E-01	2.6E-01	3.3E-01
GBM	MRPL55_ES_2.2_1.2_2.8	MRPL55	ES	2.2	1.2	2.8	5.5E-04	0.136	-0.016	1.8E-01	3.2E-01	7.0E-01
GBM	MT1A_ES_2_1_3	MT1A	ES	2	1	3	1.7E-04	0.151	0.604	5.9E-02	1.3E-01	5.2E-01
GBM	MYEF2_ES_15_14_16	MYEF2	ES	15	14	16	1.0E-04	0.131	0.231	9.2E-02	1.9E-01	4.4E-01

cancer type	id	Gene Symbol	splice_type	Exon	From.Exon	To.Exon	anova.p	adj.r2	r	p.50	p.25	p.10
GBM	NEO1_ES_27_26_28	NEO1	ES	27	26	28	8.5E-04	0.100	0.043	9.0E-01	8.9E-01	3.7E-01
GBM	NR4A1_ES_5.1:5.2_4.1_6.1	NR4A1	ES	5.1:5.2	4.1	6.1	2.5E-04	0.141	0.034	2.8E-01	2.9E-01	2.3E-01
GBM	NUMA1_ES_18_17_19	NUMA1	ES	18	17	19	2.4E-04	0.118	-0.257	8.1E-01	7.7E-02	5.6E-02
GBM	NUP62_AD_1.3:1.4:1.5_1.2_2.1	NUP62	AD	1.3:1.4:1.5	1.2	2.1	7.9E-04	0.101	-0.257	8.9E-01	1.9E-01	4.7E-01
GBM	OXLD1_AD_1.2_1.1_2	OXLD1	AD	1.2	1.1	2	3.0E-06	0.175	-0.099	7.1E-01	1.0E+00	4.8E-01
GBM	P4HA2_AD_2.3_2.2_3	P4HA2	AD	2.3	2.2	3	8.4E-04	0.131	-0.158	2.5E-01	8.3E-01	1.7E-01
GBM	PCBP4_ES_3:4.1_2.2_5	PCBP4	ES	03:04.1	2.2	5	1.7E-04	0.134	-0.150	3.6E-02	1.8E-02	1.6E-01
GBM	PLOD2_ES_15_14_16	PLOD2	ES	15	14	16	5.8E-04	0.106	-0.118	3.8E-01	1.4E-01	3.7E-01
GBM	POSTN_ES_18_16_19	POSTN	ES	18	16	19	5.9E-04	0.128	0.154	1.3E-01	8.5E-01	9.4E-02
GBM	PSMC3IP_RI_1.2_1.1_1.3	PSMC3IP	RI	1.2	1.1	1.3	1.6E-07	0.218	-0.082	6.8E-01	7.0E-01	4.9E-01
GBM	PTHLH_AD_3.3_3.2_4.2	PTHLH	AD	3.3	3.2	4.2	4.7E-04	0.133	0.295	7.9E-02	1.4E-01	1.0E-01
GBM	PTMS_ES_3.2_2_5	PTMS	ES	3.2	2	5	4.7E-05	0.163	0.199	1.5E-01	8.0E-03	9.1E-03
GBM	PTPRS_ES_26_24.1_27.2	PTPRS	ES	26	24.1	27.2	5.2E-04	0.120	-0.090	6.6E-01	2.1E-01	3.8E-01
GBM	PTRH2_RI_2.3_2.2_2.4	PTRH2	RI	2.3	2.2	2.4	8.8E-06	0.167	-0.334	7.2E-01	4.8E-01	5.4E-01
GBM	RBPJ_ES_4.2_2.3_5	RBPJ	ES	4.2	2.3	5	3.8E-04	0.113	0.006	9.8E-02	5.3E-02	3.3E-01
GBM	RHOC_ES_1.2:2.2_1.1_2.3	RHOC	ES	1.2:2.2	1.1	2.3	1.1E-06	0.195	-0.104	3.7E-01	8.5E-01	5.5E-01
GBM	RIMKLB_ES_3_2_4	RIMKLB	ES	3	2	4	5.7E-04	0.109	0.100	2.1E-01	1.7E-01	8.1E-03
GBM	RNF7_ES_1.2:2_1.1_3	RNF7	ES	1.2:2	1.1	3	3.1E-04	0.114	-0.045	3.6E-02	3.0E-03	1.3E-02
GBM	RPL32_AA_1.3_1.1_1.4	RPL32	AA	1.3	1.1	1.4	7.1E-05	0.133	0.095	1.5E-01	3.9E-02	8.3E-01
GBM	RPS6KB2_ES_6.1:6.2_5.1_7	RPS6KB2	ES	6.1:6.2	5.1	7	2.9E-04	0.115	-0.351	5.2E-01	2.4E-01	4.4E-02
GBM	SCAI_ES_3_2_4	SCAI	ES	3	2	4	1.2E-04	0.166	0.101	7.9E-01	8.0E-01	1.9E-01
GBM	SETD9_ES_2_1_3	SETD9	ES	2	1	3	5.6E-05	0.137	-0.248	1.3E-01	1.1E-02	1.6E-02
GBM	SLC44A2_AA_23.1_22_23.2	SLC44A2	AA	23.1	22	23.2	5.1E-04	0.107	-0.116	9.1E-01	1.8E-01	2.3E-01
GBM	SMPD4_ES_11_10_14	SMPD4	ES	11	10	14	2.4E-05	0.148	0.075	3.0E-01	9.0E-01	7.6E-01
GBM	SORBS1_ES_20_19_23	SORBS1	ES	20	19	23	4.0E-04	0.112	0.076	4.5E-01	4.5E-01	2.6E-01
GBM	SRPK2_ES_19_18_20	SRPK2	ES	19	18	20	7.2E-07	0.199	0.134	1.6E-01	4.6E-02	1.3E-01
GBM	SYNGR2_AA_4.1:4.2_3.2_4.3	SYNGR2	AA	4.1:4.2	3.2	4.3	8.6E-04	0.103	-0.049	7.7E-01	5.7E-01	8.7E-01
GBM	TM2D1_ES_2.3_1.3_3	TM2D1	ES	2.3	1.3	3	8.1E-04	0.126	0.066	4.9E-01	8.2E-01	1.6E-02
GBM	TMEM25_ES_5_4_6	TMEM25	ES	5	4	6	4.8E-07	0.194	-0.085	9.9E-02	6.1E-01	1.5E-01
GBM	TMEM62_ES_6_4_7	TMEM62	ES	6	4	7	5.3E-04	0.138	0.078	8.3E-02	1.8E-02	3.6E-01
GBM	TRAPPC5_AD_1.2:1.3_1.1_2.1	TRAPPC5	AD	1.2:1.3	1.1	2.1	2.4E-07	0.230	-0.168	7.6E-01	2.9E-01	6.8E-01
GBM	TRMT10B_ES_3.2:4.1_2_5	TRMT10B	ES	3.2:4.1	2	5	7.3E-04	0.109	0.010	7.1E-01	5.2E-01	4.9E-01
GBM	UBE2C_ES_3.1_2_4	UBE2C	ES	3.1	2	4	3.5E-04	0.142	-0.156	2.1E-02	1.8E-01	4.9E-01
GBM	UQCRQ_RI_1.3_1.2_1.4	UQCRQ	RI	1.3	1.2	1.4	8.3E-05	0.132	-0.499	4.7E-01	4.9E-01	7.6E-01
GBM	VCAN_ES_8_6.1_9	VCAN	ES	8	6.1	9	2.5E-05	0.158	-0.150	3.6E-01	2.9E-01	9.1E-01
GBM	VEZT_AA_12.1_11_12.2	VEZT	AA	12.1	11	12.2	1.7E-04	0.123	-0.072	2.1E-02	1.1E-01	8.6E-01
GBM	WHSC1_RI_13.2_13.1_13.3	WHSC1	RI	13.2	13.1	13.3	4.1E-04	0.110	0.089	9.4E-01	9.2E-01	1.9E-01
GBM	YIPF1_ES_12_11_13	YIPF1	ES	12	11	13	1.4E-06	0.181	-0.153	3.0E-01	6.5E-01	7.7E-01
GBM	ZNF254_ES_4:5_3_6	ZNF254	ES	4:05	3	6	5.0E-04	0.123	-0.108	5.4E-01	9.6E-01	5.8E-01
GBM	ZNF286A_AA_1.3_1.1_1.4	ZNF286A	AA	1.3	1.1	1.4	1.7E-04	0.150	0.162	6.7E-01	2.6E-01	9.3E-01

cancer type	id	Gene Symbol	splice_type	Exon	From.Exon	To.Exon	anova.p	adj.r2	r	p.50	p.25	p.10
GBM	ZNF302_AA_6.1_5.2_6.2	ZNF302	AA	6.1	5.2	6.2	1.5E-05	0.153	-0.101	4.7E-01	5.2E-01	2.2E-01
GBM	ZNF397_AD_1.2_1.1_2.2	ZNF397	AD	1.2	1.1	2.2	7.4E-05	0.142	0.189	4.7E-01	7.4E-01	1.2E-01
GBM	ZNF530_ES_2:3_1_5	ZNF530	ES	2:03	1	5	5.3E-05	0.162	0.150	3.4E-01	4.3E-01	5.6E-01
GBM	ZNF83_ES_9.6_7_10.1	ZNF83	ES	9.6	7	10.1	2.5E-04	0.145	0.007	7.2E-01	6.4E-01	5.1E-01
HNSC	ABCC3_RI_16.2_16.1_16.3	ABCC3	RI	16.2	16.1	16.3	2.4E-10	0.158	-0.469	9.1E-02	5.7E-01	3.4E-01
HNSC	ABI1_ES_10:11.1:11.2_9_12	ABI1	ES	10:11.1:11.2	9	12	1.8E-06	0.101	-0.310	7.9E-01	4.8E-01	3.9E-01
HNSC	ABI1_ES_5_4_7	ABI1	ES	5	4	7	1.6E-22	0.310	-0.403	9.8E-01	9.7E-01	1.8E-01
HNSC	ABLIM2_ES_14:15:16_13_17	ABLIM2	ES	14:15:16	13	17	1.1E-10	0.171	0.375	6.9E-02	2.0E-01	2.3E-03
HNSC	ACIN1_ES_4_3_5	ACIN1	ES	4	3	5	1.5E-07	0.109	-0.066	2.8E-01	3.6E-01	1.5E-01
HNSC	ACOT9_ES_6_5.1_7.1	ACOT9	ES	6	5.1	7.1	7.1E-09	0.129	0.120	7.0E-01	8.8E-01	3.1E-01
HNSC	ACP5_ES_2.1:2.2_1_3.2	ACP5	ES	2.1:2.2	1	3.2	2.9E-10	0.153	-0.175	1.9E-01	4.6E-01	7.4E-01
HNSC	ACTN1_ES_20_19_21	ACTN1	ES	20	19	21	3.0E-07	0.110	0.228	1.5E-01	7.5E-03	1.2E-03
HNSC	ADAM23_ES_26_24_27	ADAM23	ES	26	24	27	4.4E-09	0.145	-0.092	4.1E-01	8.2E-02	4.3E-01
HNSC	ADD3_ES_15_14_16	ADD3	ES	15	14	16	4.6E-20	0.280	0.239	2.8E-01	1.9E-01	1.2E-01
HNSC	AFTPH_ES_7:8_6_9	AFTPH	ES	7:08	6	9	5.6E-07	0.111	0.432	7.6E-01	1.4E-01	6.1E-01
HNSC	AKAP8L_ES_10_9_11	AKAP8L	ES	10	9	11	7.3E-08	0.113	0.204	2.8E-02	1.2E-01	2.0E-01
HNSC	AKIP1_RI_1.2_1.1_1.3	AKIP1	RI	1.2	1.1	1.3	6.4E-10	0.144	0.106	9.5E-01	7.6E-01	1.3E-01
HNSC	ANKRD65_ES_1.4:2_1.3_3	ANKRD65	ES	1.4:2	1.3	3	1.2E-06	0.110	-0.318	1.7E-02	1.9E-01	2.2E-01
HNSC	ANXA1_ES_6:7:8:9:11_5_12	ANXA1	ES	6:7:8:9:11	5	12	1.2E-06	0.110	-0.262	9.1E-01	8.9E-01	6.2E-01
HNSC	ANXA2_ES_2:4.2_1.1_7	ANXA2	ES	02:04.2	1.1	7	3.2E-07	0.115	-0.109	5.9E-01	4.7E-01	4.7E-01
HNSC	ANXA2_ES_5:6_4.2_7	ANXA2	ES	5:06	4.2	7	2.2E-07	0.109	0.100	7.3E-01	9.3E-01	9.8E-02
HNSC	ANXA7_ES_2:3.1:3.2:4:5:6_1_7	ANXA7	ES	2:3.1:3.2:4:5:6	1	7	6.4E-09	0.152	-0.139	5.9E-01	9.9E-01	5.9E-01
HNSC	AP2S1_ES_3.2_2_4.2	AP2S1	ES	3.2	2	4.2	8.3E-08	0.118	0.275	2.0E-01	4.0E-02	6.7E-01
HNSC	AP2S1_ES_3.2:4.1_1.1_4.2	AP2S1	ES	3.2:4.1	1.1	4.2	4.1E-07	0.102	-0.338	8.4E-01	4.1E-01	6.5E-02
HNSC	APOD_ES_3_2_4	APOD	ES	3	2	4	1.7E-09	0.140	-0.637	2.9E-01	6.2E-02	1.7E-01
HNSC	APP_ES_10_9_11	APP	ES	10	9	11	2.3E-18	0.259	0.230	1.2E-01	4.1E-03	3.1E-01
HNSC	ARAP1_ES_32_31_33	ARAP1	ES	32	31	33	1.2E-09	0.140	0.041	7.6E-03	3.0E-03	2.2E-02
HNSC	ARHGAP17_ES_18_17_19	ARHGAP17	ES	18	17	19	5.7E-16	0.228	0.013	4.4E-02	2.9E-02	5.0E-02
HNSC	ARHGEF1_ES_15_14_16	ARHGEF1	ES	15	14	16	9.3E-18	0.251	0.167	3.2E-02	8.7E-03	3.6E-02
HNSC	ARHGEF10L_ES_18_17_19	ARHGEF10L	ES	18	17	19	7.0E-12	0.176	0.196	2.8E-01	5.4E-01	5.7E-01
HNSC	ARHGEF11_ES_39_38_40	ARHGEF11	ES	39	38	40	2.5E-24	0.332	-0.058	1.9E-02	2.8E-02	5.9E-01
HNSC	ARVCF_ES_20_19_21	ARVCF	ES	20	19	21	4.5E-09	0.146	0.193	2.0E-01	1.2E-01	9.7E-01
HNSC	ATP11C_ES_32_29_33	ATP11C	ES	32	29	33	1.4E-11	0.168	-0.039	6.1E-01	7.2E-01	2.2E-01
HNSC	ATP2A2_RI_21.2:21.3_21.1_21.4	ATP2A2	RI	21.2:21.3	21.1	21.4	8.4E-09	0.128	-0.250	4.0E-02	1.6E-02	1.2E-01
HNSC	ATP5G2_ES_3:4.1_1.3_4.2	ATP5G2	ES	03:04.1	1.3	4.2	3.1E-06	0.102	-0.184	3.3E-01	4.7E-01	8.5E-01
HNSC	ATP5J_ES_1.3:1.4:1.5:2_1.2_3	ATP5J	ES	1.3:1.4:1.5:2	1.2	3	8.8E-08	0.112	0.076	3.9E-01	3.2E-01	2.5E-01
HNSC	AXIN1_ES_9_8_10	AXIN1	ES	9	8	10	2.0E-08	0.122	-0.102	2.5E-01	2.6E-01	4.7E-01
HNSC	B4GALT2_AD_2.2_2.1_3	B4GALT2	AD	2.2	2.1	3	8.1E-13	0.186	-0.208	1.9E-01	6.6E-02	7.6E-01
HNSC	BAZ1A_ES_13_12_14	BAZ1A	ES	13	12	14	2.6E-08	0.120	-0.056	9.6E-01	6.8E-01	3.7E-01
HNSC	BBX_ES_16_15_17	BBX	ES	16	15	17	2.6E-07	0.106	-0.003	6.0E-01	3.7E-01	7.0E-01

cancer type	id	Gene Symbol	splice_type	Exon	From.Exon	To.Exon	anova.p	adj.r2	r	p.50	p.25	p.10
HNSC	BCL2L12_ES_3.1:3.2_2_4	BCL2L12	ES	3.1:3.2	2	4	1.2E-20	0.287	0.052	9.1E-01	8.9E-01	4.4E-01
HNSC	BCL2L12_ES_3.2_2_4	BCL2L12	ES	3.2	2	4	2.4E-11	0.165	0.035	5.2E-01	7.5E-01	7.3E-01
HNSC	BICD2_RI_7.2_7.1_7.3	BICD2	RI	7.2	7.1	7.3	5.0E-09	0.131	0.014	3.1E-01	1.7E-01	2.0E-01
HNSC	BIN1_ES_11_10_12	BIN1	ES	11	10	12	4.0E-08	0.118	0.638	2.0E-01	1.3E-01	1.1E-01
HNSC	BMP1_AA_18.1_16_18.2	BMP1	AA	18.1	16	18.2	4.7E-07	0.101	0.208	2.3E-01	8.4E-01	4.8E-01
HNSC	BTN2A1_ES_4_3_5	BTN2A1	ES	4	3	5	3.7E-07	0.105	0.035	8.5E-01	9.8E-01	8.2E-01
HNSC	C11orf57_AD_1.2:1.3_1.1_2.1	C11orf57	AD	1.2:1.3	1.1	2.1	2.0E-07	0.107	0.114	9.6E-01	7.0E-01	7.2E-01
HNSC	C11orf88_RI_6.2_6.1_6.3	C11orf88	RI	6.2	6.1	6.3	2.7E-16	0.233	-0.726	5.2E-01	5.2E-01	3.8E-01
HNSC	C12orf29_ES_3_1_4	C12orf29	ES	3	1	4	1.5E-11	0.168	0.328	6.4E-01	4.6E-01	9.4E-02
HNSC	C12orf43_ES_2_1_3.1	C12orf43	ES	2	1	3.1	2.5E-07	0.105	-0.357	6.0E-03	1.2E-01	2.5E-01
HNSC	C1orf50_RI_1.2_1.1_1.3	C1orf50	RI	1.2	1.1	1.3	5.5E-07	0.105	-0.081	7.6E-03	6.9E-02	1.4E-01
HNSC	C2CD5_ES_26:27.1:27.2_25_28	C2CD5	ES	26:27.1:27.2	25	28	2.9E-15	0.220	-0.189	3.0E-01	2.3E-01	6.3E-01
HNSC	C7orf43_AA_9.1_8_9.2	C7orf43	AA	9.1	8	9.2	5.4E-10	0.145	-0.032	2.7E-01	6.3E-01	3.6E-01
HNSC	CA12_ES_10_8_11	CA12	ES	10	8	11	1.3E-10	0.171	-0.105	5.3E-01	4.0E-01	7.0E-01
HNSC	CALCOCO2_ES_3_2_6	CALCOCO2	ES	3	2	6	4.3E-07	0.102	-0.515	3.6E-01	2.7E-01	1.5E-02
HNSC	CAMK2D_ES_17_14.2_18	CAMK2D	ES	17	14.2	18	2.6E-09	0.136	0.135	9.7E-02	3.7E-01	7.6E-01
HNSC	CAMK2D_ES_22_21.1_23	CAMK2D	ES	22	21.1	23	4.5E-12	0.176	0.115	6.6E-01	8.0E-01	3.3E-01
HNSC	CAMKK2_ES_17_16_19.1	CAMKK2	ES	17	16	19.1	1.2E-07	0.110	0.004	3.4E-01	9.8E-03	1.6E-01
HNSC	CASK_ES_20:21_19.1_22	CASK	ES	20:21	19.1	22	8.6E-09	0.134	-0.115	6.2E-01	9.2E-01	3.3E-01
HNSC	CASK_ES_21_19.1_22	CASK	ES	21	19.1	22	4.5E-09	0.138	-0.109	8.4E-01	7.1E-01	9.7E-01
HNSC	CASP1_RI_10.2_10.1_10.3	CASP1	RI	10.2	10.1	10.3	3.4E-07	0.103	-0.279	2.2E-02	1.6E-03	6.2E-04
HNSC	CAST_ES_7.1:8.2_5.2_9	CAST	ES	7.1:8.2	5.2	9	4.6E-19	0.273	-0.128	7.4E-01	5.2E-01	2.2E-01
HNSC	CAST_ES_7.1:8.2:9_5.2_10	CAST	ES	7.1:8.2:9	5.2	10	2.4E-16	0.256	-0.100	7.7E-02	4.7E-02	4.1E-01
HNSC	CAST_ES_8.2:9_7.1_10	CAST	ES	8.2:9	7.1	10	2.4E-12	0.187	-0.123	7.0E-01	2.3E-01	1.6E-01
HNSC	CC2D2A_ES_5_4_6	CC2D2A	ES	5	4	6	4.2E-08	0.138	0.120	2.1E-01	8.2E-01	7.0E-01
HNSC	CCDC107_RI_3.4_3.3_3.5	CCDC107	RI	3.4	3.3	3.5	8.0E-12	0.172	-0.235	8.5E-01	9.3E-01	7.6E-01
HNSC	CCDC24_AA_4.1_3.2_4.2	CCDC24	AA	4.1	3.2	4.2	9.5E-11	0.174	0.165	7.4E-03	3.6E-02	5.8E-01
HNSC	CCDC50_ES_6_5_7	CCDC50	ES	6	5	7	4.8E-24	0.327	0.079	3.0E-01	7.9E-01	2.6E-01
HNSC	CCDC51_AA_3.1_2.2_3.2	CCDC51	AA	3.1	2.2	3.2	3.3E-07	0.103	0.511	3.9E-01	8.6E-01	4.7E-02
HNSC	CCNT2_AD_7.2_7.1_8	CCNT2	AD	7.2	7.1	8	1.0E-07	0.112	0.152	2.2E-01	8.1E-01	3.6E-01
HNSC	CCSER2_ES_11_10_12	CCSER2	ES	11	10	12	9.0E-16	0.226	0.140	9.0E-01	3.6E-01	8.8E-01
HNSC	CD44_ES_10:11_5_12.1	CD44	ES	10:11	5	12.1	1.3E-09	0.141	0.394	8.9E-01	2.1E-01	4.7E-02
HNSC	CD44_ES_10:11:12.1:13:14_5_15	CD44	ES	10:11:12.1:13:14	5	15	5.9E-11	0.160	0.394	7.8E-01	1.9E-02	3.4E-02
HNSC	CD44_ES_12.1:13_5_14	CD44	ES	12.1:13	5	14	2.2E-07	0.107	0.060	7.7E-02	3.0E-01	3.6E-01
HNSC	CD44_ES_12.1:13:14_5_15	CD44	ES	12.1:13:14	5	15	4.8E-09	0.131	-0.010	3.4E-01	1.0E+00	9.8E-01
HNSC	CD44_ES_6:7:8:9.1:9.2:10:11:12.1:13:14	CD44	ES	.1:9.2:10:11:12.1	5	15	5.8E-14	0.201	0.455	3.7E-02	7.7E-02	2.6E-02
HNSC	CD44_ES_6:7:8:9.2:10:11:12.1:13:14_5_	CD44	ES	:9.2:10:11:12.1:1	5	15	1.9E-14	0.208	0.469	8.1E-02	2.2E-01	5.8E-03
HNSC	CD44_ES_7:8:9.1:9.2:10:11_5_12.1	CD44	ES	7:8:9.1:9.2:10:11	5	12.1	3.3E-07	0.103	0.493	2.9E-01	3.0E-01	5.7E-02
HNSC	CD44_ES_7:8:9.1:9.2:10:11:12.1:13:14_5	CD44	ES	1:9.2:10:11:12.1:	5	15	1.5E-12	0.182	0.577	2.7E-01	1.4E-01	5.1E-02
HNSC	CD44_ES_7:8:9.2:10:11_5_12.1	CD44	ES	7:8:9.2:10:11	5	12.1	8.2E-08	0.113	0.478	3.6E-01	1.4E-01	3.0E-02

cancer type	id	Gene Symbol	splice_type	Exon	From.Exon	To.Exon	anova.p	adj.r2	r	p.50	p.25	p.10
HNSC	CD44_ES_7:8:9.2:10:11:12.1:13:14_5_15	CD44	ES	9.2:10:11:12.1:13	5	15	1.0E-13	0.198	0.592	2.0E-01	2.2E-02	5.7E-02
HNSC	CD44_ES_8:9.1:9.2:10:11:12.1:13:14_5_	CD44	ES	:9.2:10:11:12.1:1	5	15	1.1E-14	0.212	0.457	1.1E-01	3.6E-01	7.7E-02
HNSC	CD44_ES_8:9.2:10:11:12.1:13:14_5_15	CD44	ES	.2:10:11:12.1:13	5	15	4.8E-15	0.217	0.465	7.3E-02	8.8E-02	1.0E-01
HNSC	CD46_ES_13_12_14	CD46	ES	13	12	14	8.7E-12	0.171	-0.147	3.2E-01	1.1E-02	2.4E-02
HNSC	CD47_ES_9:10_8_11	CD47	ES	9:10	8	11	1.0E-08	0.126	-0.177	1.5E-01	3.6E-01	5.7E-01
HNSC	CEACAM1_ES_9_8_10	CEACAM1	ES	9	8	10	6.3E-07	0.111	-0.397	5.7E-01	7.0E-01	6.4E-01
HNSC	CERCAM_RI_3.2_3.1_3.3	CERCAM	RI	3.2	3.1	3.3	1.0E-07	0.111	-0.379	7.4E-03	7.3E-04	2.2E-02
HNSC	CERS5_ES_6.1:6.2_1_8	CERS5	ES	6.1:6.2	1	8	1.8E-09	0.138	0.114	7.1E-02	2.4E-01	1.3E-01
HNSC	CLSTN1_ES_11_10_12	CLSTN1	ES	11	10	12	6.2E-13	0.187	0.180	7.8E-01	5.0E-02	4.5E-02
HNSC	COL16A1_ES_44_43_45.1	COL16A1	ES	44	43	45.1	1.9E-15	0.223	-0.144	1.2E-01	1.2E-01	7.6E-01
HNSC	COL16A1_ES_53_52_54	COL16A1	ES	53	52	54	7.2E-10	0.149	0.186	5.2E-01	9.6E-01	2.1E-01
HNSC	COL4A5_ES_42:43_41_44	COL4A5	ES	42:43:00	41	44	8.0E-13	0.191	-0.184	6.7E-01	1.0E+00	2.8E-01
HNSC	COL6A3_ES_6_5_7	COL6A3	ES	6	5	7	2.2E-11	0.168	0.130	5.3E-01	5.7E-01	5.5E-01
HNSC	COX4I1_AA_5.1:5.2:5.3_4.1_5.4	COX4I1	AA	5.1:5.2:5.3	4.1	5.4	1.1E-08	0.126	-0.078	4.3E-02	3.7E-03	1.4E-02
HNSC	CSDE1_ES_2:3_1_4	CSDE1	ES	2:03	1	4	2.1E-08	0.128	0.078	1.4E-02	3.6E-02	3.6E-03
HNSC	CSDE1_ES_3_2_4	CSDE1	ES	3	2	4	2.1E-09	0.137	0.249	4.3E-02	8.6E-02	5.9E-02
HNSC	CSDE1_ES_3:4_2_5	CSDE1	ES	3:04	2	5	3.0E-09	0.140	0.062	1.8E-02	6.6E-02	2.6E-01
HNSC	CSF2RA_ES_13_12_14	CSF2RA	ES	13	12	14	2.2E-13	0.193	-0.686	2.5E-01	9.1E-01	2.9E-01
HNSC	CSNK1G3_ES_13_12_14	CSNK1G3	ES	13	12	14	8.0E-18	0.253	-0.103	6.8E-01	9.5E-01	7.9E-01
HNSC	CTNNB1_AA_18.3_18.1_18.4	CTNNB1	AA	18.3	18.1	18.4	2.0E-07	0.107	0.027	8.1E-01	3.3E-01	9.6E-01
HNSC	CTNND1_ES_21_20_22.1	CTNND1	ES	21	20	22.1	6.1E-08	0.115	0.088	6.2E-01	7.0E-01	6.3E-01
HNSC	CTNND1_ES_21:22.1_20_22.2	CTNND1	ES	21:22.1	20	22.2	5.0E-11	0.161	0.069	5.1E-01	9.1E-01	1.4E-01
HNSC	CTNND1_ES_3:4.1:4.2:4.3_2.1_5	CTNND1	ES	3:4.1:4.2:4.3	2.1	5	2.5E-22	0.309	-0.273	1.3E-01	2.4E-01	8.6E-01
HNSC	CTNND1_ES_3:4.1:4.2:4.3:5_2.1_6	CTNND1	ES	3:4.1:4.2:4.3:5	2.1	6	4.7E-16	0.235	-0.202	8.7E-01	2.2E-01	8.6E-01
HNSC	CTNND1_ES_3:4.2:4.3:5_2.1_6	CTNND1	ES	3:4.2:4.3:5	2.1	6	1.2E-09	0.149	-0.207	9.1E-01	7.9E-01	5.9E-01
HNSC	CTTN_ES_11_10_13	CTTN	ES	11	10	13	4.6E-16	0.229	-0.119	8.9E-02	1.3E-01	9.6E-01
HNSC	CUTC_ES_7_6_8	CUTC	ES	7	6	8	2.3E-08	0.121	0.335	8.9E-01	5.1E-01	4.5E-02
HNSC	CYB561A3_AA_6.6_6.4_6.7	CYB561A3	AA	6.6	6.4	6.7	5.0E-14	0.202	0.048	9.1E-02	5.9E-01	3.8E-01
HNSC	CYTH1_ES_11.2:13.1_11.1_13.2	CYTH1	ES	11.2:13.1	11.1	13.2	5.6E-09	0.132	0.047	4.5E-01	3.5E-01	9.2E-01
HNSC	CYTH1_ES_12_11.1_13.2	CYTH1	ES	12	11.1	13.2	7.5E-09	0.129	0.025	6.5E-01	8.5E-01	3.6E-01
HNSC	DAP3_AD_2.3_2.2_3	DAP3	AD	2.3	2.2	3	7.1E-09	0.129	0.136	6.0E-02	1.8E-02	2.8E-01
HNSC	DCAF10_ES_5_4_6	DCAF10	ES	5	4	6	1.1E-07	0.111	0.114	1.4E-01	8.7E-02	1.9E-02
HNSC	DCAF6_ES_11_10_13.1	DCAF6	ES	11	10	13.1	9.4E-10	0.143	0.183	5.0E-01	7.9E-01	6.9E-01
HNSC	DCAF6_ES_11:13.1_10_14	DCAF6	ES	11:13.1	10	14	4.2E-08	0.118	0.278	8.8E-01	2.8E-01	1.7E-01
HNSC	DCAF8_ES_18_17_19	DCAF8	ES	18	17	19	6.1E-10	0.145	0.136	1.0E-02	1.5E-01	1.0E-01
HNSC	DCTD_ES_1.2:2.1:2.2_1.1_5	DCTD	ES	1.2:2.1:2.2	1.1	5	7.0E-07	0.104	-0.014	9.0E-01	7.3E-01	9.6E-01
HNSC	DCTN2_ES_2:8_1_10	DCTN2	ES	2:08	1	10	1.1E-10	0.178	-0.044	5.3E-01	6.3E-01	9.7E-01
HNSC	DDX52_ES_3_2_5	DDX52	ES	3	2	5	3.4E-08	0.119	-0.488	6.9E-01	1.5E-01	4.0E-02
HNSC	DEF8_ES_2.1_1_4	DEF8	ES	2.1	1	4	2.1E-09	0.137	-0.265	1.8E-04	1.5E-03	4.4E-02
HNSC	DENND4C_ES_20_19_21	DENND4C	ES	20	19	21	1.5E-07	0.111	-0.030	8.6E-01	2.5E-01	5.8E-01

cancer type	id	Gene Symbol	splice_type	Exon	From.Exon	To.Exon	anova.p	adj.r2	r	p.50	p.25	p.10
HNSC	DEPDC1_ES_8_7_9	DEPDC1	ES	8	7	9	6.5E-13	0.187	-0.045	9.4E-01	3.4E-01	1.6E-01
HNSC	DHRS1_RI_6.3_6.2_6.4	DHRS1	RI	6.3	6.2	6.4	9.7E-10	0.142	-0.531	7.2E-01	1.7E-01	9.4E-01
HNSC	DIAPH1_ES_2_1_3	DIAPH1	ES	2	1	3	1.3E-12	0.185	0.026	3.1E-01	1.6E-02	3.9E-01
HNSC	DLG1_ES_9_7_10	DLG1	ES	9	7	10	7.5E-09	0.129	-0.169	8.8E-01	6.2E-01	4.2E-01
HNSC	DMKN_AD_20.2_20.1_21	DMKN	AD	20.2	20.1	21	8.4E-09	0.138	-0.504	7.5E-02	9.0E-02	7.5E-01
HNSC	DMKN_ES_7_6.4_12	DMKN	ES	7	6.4	12	1.7E-07	0.111	0.363	2.3E-01	5.9E-01	7.6E-01
HNSC	DMWD_ES_4_3_5	DMWD	ES	4	3	5	9.6E-15	0.212	0.024	4.0E-01	2.1E-01	7.2E-01
HNSC	DOCK6_ES_23_22_24	DOCK6	ES	23	22	24	1.0E-08	0.129	-0.205	6.7E-01	7.4E-01	3.4E-01
HNSC	DOCK7_ES_24_23_25	DOCK7	ES	24	23	25	2.2E-11	0.176	0.010	8.2E-02	4.0E-01	7.4E-01
HNSC	DOCK8_ES_2_1_3	DOCK8	ES	2	1	3	1.4E-07	0.110	-0.472	9.5E-02	1.8E-01	6.0E-01
HNSC	DSC2_ES_16_15_17	DSC2	ES	16	15	17	2.7E-11	0.164	0.119	4.2E-01	8.4E-01	1.6E-01
HNSC	DST_ES_43:44:45:46.1:48_42_49	DST	ES	43:44:45:46.1:48	42	49	3.8E-11	0.169	-0.166	7.8E-01	5.1E-01	8.5E-01
HNSC	DTX3_ES_1.3_1.1_1.5	DTX3	ES	1.3	1.1	1.5	2.2E-07	0.109	-0.268	5.1E-01	7.2E-01	6.4E-01
HNSC	DUOXA2_AA_2.1_1_2.2	DUOXA2	AA	2.1	1	2.2	7.7E-12	0.197	-0.684	3.0E-01	8.3E-01	5.3E-01
HNSC	DUSP22_RI_7.4_7.3_7.5	DUSP22	RI	7.4	7.3	7.5	5.5E-10	0.145	-0.381	1.1E-01	3.9E-03	2.7E-02
HNSC	DVL1_AD_11.2_11.1_12	DVL1	AD	11.2	11.1	12	2.9E-13	0.192	-0.024	3.2E-01	4.4E-01	6.7E-02
HNSC	ECHDC1_ES_7_6.3_10.2	ECHDC1	ES	7	6.3	10.2	9.0E-12	0.171	0.107	5.1E-01	5.3E-02	1.5E-01
HNSC	ECT2_ES_5_4.2_6	ECT2	ES	5	4.2	6	2.2E-11	0.167	-0.330	2.4E-03	1.2E-02	6.9E-02
HNSC	ELP4_ES_10:11_9_12	ELP4	ES	10:11	9	12	1.4E-07	0.109	-0.634	2.0E-04	3.4E-02	6.7E-02
HNSC	ENAH_ES_13_12_14	ENAH	ES	13	12	14	6.9E-09	0.129	-0.184	9.1E-01	5.2E-01	4.4E-01
HNSC	ENAH_ES_7.1:7.2_6_8	ENAH	ES	7.1:7.2	6	8	7.4E-13	0.193	-0.266	3.1E-01	1.4E-01	2.0E-01
HNSC	EPB41_ES_18_15_19.1	EPB41	ES	18	15	19.1	4.3E-07	0.107	-0.088	2.2E-02	2.5E-03	3.2E-03
HNSC	ERBB2IP_ES_22:24.1:24.2:24.3_21_25	ERBB2IP	ES	22:24.1:24.2:24.3	21	25	2.0E-23	0.326	0.208	2.2E-02	1.4E-02	1.1E-01
HNSC	ERBB2IP_ES_23:24.1:24.2:24.3_21_25	ERBB2IP	ES	23:24.1:24.2:24.3	21	25	1.8E-12	0.183	0.058	4.6E-01	4.9E-01	2.2E-01
HNSC	ERBB2IP_ES_24.1:24.2:24.3_21_25	ERBB2IP	ES	24.1:24.2:24.3	21	25	1.9E-20	0.286	0.123	2.9E-01	3.1E-01	6.2E-02
HNSC	ESRP1_AD_12.2_12.1_13	ESRP1	AD	12.2	12.1	13	3.6E-10	0.149	-0.337	1.4E-01	2.2E-01	7.5E-01
HNSC	ESRP1_ES_15_13_16	ESRP1	ES	15	13	16	1.9E-07	0.108	0.128	9.8E-01	9.1E-01	6.7E-01
HNSC	EVI5L_ES_12_11_13	EVI5L	ES	12	11	13	1.5E-17	0.251	-0.213	7.3E-01	5.1E-01	4.1E-01
HNSC	EXO1_ES_3_2_4	EXO1	ES	3	2	4	2.0E-07	0.111	-0.283	9.1E-01	5.0E-01	5.0E-01
HNSC	EXOC1_ES_11_10_12	EXOC1	ES	11	10	12	2.4E-10	0.151	0.157	2.1E-01	2.2E-01	8.2E-01
HNSC	EXOC7_ES_7:8.2_6_9	EXOC7	ES	07:08.2	6	9	3.4E-07	0.104	-0.015	3.4E-01	4.0E-01	9.5E-01
HNSC	EXOC7_ES_8.2_6_9	EXOC7	ES	8.2	6	9	8.3E-12	0.172	0.052	4.9E-01	8.6E-01	1.5E-01
HNSC	FAM115C_ES_7_6_8	FAM115C	ES	7	6	8	8.0E-09	0.131	-0.277	3.5E-01	2.6E-01	1.9E-01
HNSC	FAM195A_ES_3_2_4	FAM195A	ES	3	2	4	4.8E-08	0.116	0.014	1.5E-02	1.2E-02	4.4E-02
HNSC	FAM213A_AA_3.2_2_3.3	FAM213A	AA	3.2	2	3.3	3.0E-11	0.164	0.120	5.3E-01	6.8E-01	7.9E-01
HNSC	FAM221A_ES_3.1:3.2:3.3_2_4	FAM221A	ES	3.1:3.2:3.3	2	4	5.3E-11	0.160	-0.598	9.6E-01	9.6E-01	7.8E-01
HNSC	FAM76A_ES_3_2_4	FAM76A	ES	3	2	4	3.5E-07	0.104	-0.120	2.4E-01	6.3E-01	1.7E-01
HNSC	FASTK_AA_5.9:5.10_5.7_5.11	FASTK	AA	5.9:5.10	5.7	5.11	4.4E-06	0.100	0.250	2.1E-01	2.9E-01	4.7E-01
HNSC	FBLN2_ES_11_10_12	FBLN2	ES	11	10	12	6.6E-14	0.203	0.016	6.7E-01	9.1E-01	8.8E-01
HNSC	FBLN5_ES_7_5_8	FBLN5	ES	7	5	8	7.4E-11	0.158	-0.645	9.0E-01	5.5E-01	7.5E-02

cancer type	id	Gene Symbol	splice_type	Exon	From.Exon	To.Exon	anova.p	adj.r2	r	p.50	p.25	p.10
HNSC	FBXO7_AA_3.1_2_3.2	FBXO7	AA	3.1	2	3.2	5.4E-07	0.102	0.135	4.4E-01	3.5E-01	2.5E-01
HNSC	FCGR2B_ES_9_8_10	FCGR2B	ES	9	8	10	1.9E-08	0.134	0.080	4.9E-03	7.2E-02	3.4E-01
HNSC	FGFR1OP2_ES_5.1_4_6	FGFR1OP2	ES	5.1	4	6	9.1E-09	0.128	-0.003	5.6E-01	5.0E-01	8.9E-01
HNSC	FHL2_ES_3.2_2.3_5.1	FHL2	ES	3.2	2.3	5.1	2.6E-09	0.136	0.229	8.8E-01	4.0E-01	5.8E-01
HNSC	FHL2_ES_4_3.2_5.1	FHL2	ES	4	3.2	5.1	1.1E-11	0.170	-0.595	6.7E-03	2.6E-02	1.5E-01
HNSC	FKBP6_AD_1.2_1.1_2	FKBP6	AD	1.2	1.1	2	3.3E-13	0.240	-0.685	4.5E-01	1.6E-01	8.8E-02
HNSC	FMNL3_ES_26_25_27	FMNL3	ES	26	25	27	1.6E-09	0.139	-0.102	7.7E-02	1.7E-02	1.5E-02
HNSC	FNBP1_ES_12_10.3_14.2	FNBP1	ES	12	10.3	14.2	3.3E-07	0.108	-0.194	7.1E-01	3.4E-01	7.1E-01
HNSC	FYN_ME_11|12_10_13	FYN	ME	11|12	10	13	7.3E-17	0.240	-0.065	2.7E-02	7.1E-03	5.4E-04
HNSC	GK_ES_12_11_13	GK	ES	12	11	13	3.2E-12	0.191	-0.296	1.2E-01	2.7E-01	8.9E-01
HNSC	GLIPR1_RI_5.2_5.1_5.3	GLIPR1	RI	5.2	5.1	5.3	1.5E-16	0.236	-0.530	5.8E-01	7.0E-01	2.3E-02
HNSC	GNB2L1_AD_2.2:2.3:2.4_2.1_3	GNB2L1	AD	2.2:2.3:2.4	2.1	3	1.1E-07	0.116	-0.150	7.0E-01	8.3E-01	5.6E-01
HNSC	GOLGA2_ES_7_6_8	GOLGA2	ES	7	6	8	3.6E-07	0.103	0.097	2.6E-02	1.5E-01	9.1E-01
HNSC	GPATCH8_ES_6_5_7	GPATCH8	ES	6	5	7	1.2E-07	0.121	0.094	1.7E-01	8.8E-02	3.2E-02
HNSC	GPR89A_AA_7.1_6_7.2	GPR89A	AA	7.1	6	7.2	1.8E-08	0.124	0.175	7.5E-01	3.7E-01	6.3E-01
HNSC	GTF3C1_AD_34.2_34.1_35	GTF3C1	AD	34.2	34.1	35	5.0E-07	0.101	-0.001	9.7E-01	9.8E-01	1.0E-01
HNSC	H2AFY_ME_7|8_6.3_9	H2AFY	ME	7|8	6.3	9	9.2E-16	0.225	0.297	1.7E-01	3.3E-01	3.4E-01
HNSC	HKR1_ES_11_8.2_12	HKR1	ES	11	8.2	12	4.7E-07	0.106	-0.144	7.5E-01	2.7E-01	5.6E-01
HNSC	HOPX_AD_4.3:4.4_4.2_4.6	HOPX	AD	4.3:4.4	4.2	4.6	5.1E-09	0.140	-0.068	2.3E-01	8.6E-01	1.7E-01
HNSC	HPS4_ES_10.3_10.1_10.5	HPS4	ES	10.3	10.1	10.5	1.1E-07	0.113	0.107	2.4E-02	5.9E-02	3.9E-01
HNSC	HTATIP2_RI_1.4_1.3_1.5	HTATIP2	RI	1.4	1.3	1.5	2.2E-08	0.121	0.247	8.6E-02	1.0E-01	5.7E-02
HNSC	ICAM3_RI_3.2_3.1_3.3	ICAM3	RI	3.2	3.1	3.3	2.2E-09	0.136	-0.621	4.7E-02	5.1E-02	4.6E-01
HNSC	IL15_ES_6_5_7	IL15	ES	6	5	7	8.8E-07	0.116	0.074	3.2E-01	5.3E-02	3.4E-01
HNSC	IL1RN_ES_5_4_6.2	IL1RN	ES	5	4	6.2	1.3E-06	0.112	-0.219	1.8E-01	1.1E-01	3.2E-02
HNSC	ING3_ES_5_4.1_6	ING3	ES	5	4.1	6	1.4E-07	0.111	-0.038	7.8E-01	6.3E-01	5.8E-01
HNSC	INO80C_ES_3:4.2:5.1:5.2:6:7_1_8	INO80C	ES	3:4.2:5.1:5.2:6:7	1	8	3.5E-07	0.104	-0.028	6.9E-03	7.4E-02	3.8E-01
HNSC	INPP5B_AD_7.2_7.1_8	INPP5B	AD	7.2	7.1	8	2.0E-12	0.195	0.081	8.4E-01	1.1E-01	2.5E-01
HNSC	IPO11_ES_30_29_33	IPO11	ES	30	29	33	1.2E-13	0.200	-0.098	8.6E-01	5.5E-01	5.2E-01
HNSC	IRF3_AD_1.6_1.5_2	IRF3	AD	1.6	1.5	2	1.2E-15	0.231	0.087	3.9E-01	1.2E-01	2.4E-01
HNSC	IRF3_RI_1.2:1.3_1.1_1.4	IRF3	RI	1.2:1.3	1.1	1.4	2.5E-09	0.135	0.182	4.4E-01	2.7E-01	1.8E-01
HNSC	ISOC2_ES_3:4.1_2_4.2	ISOC2	ES	03:04.1	2	4.2	1.8E-11	0.167	-0.036	7.8E-01	4.3E-01	5.4E-01
HNSC	ITGB1_ME_17|18_16_19	ITGB1	ME	17|18	16	19	3.6E-10	0.158	0.149	2.2E-02	1.1E-02	4.0E-02
HNSC	ITGB4_ES_35:36_34_37	ITGB4	ES	35:36:00	34	37	8.9E-12	0.174	-0.160	3.6E-02	1.6E-02	1.7E-03
HNSC	KCTD7_ES_13_12_14	KCTD7	ES	13	12	14	5.7E-08	0.116	-0.142	2.5E-01	5.8E-01	8.1E-01
HNSC	KIAA0226_ES_14_13_15	KIAA0226	ES	14	13	15	7.6E-07	0.101	-0.170	4.6E-01	8.3E-01	6.8E-01
HNSC	KIAA1217_ES_12_11_13	KIAA1217	ES	12	11	13	3.3E-10	0.150	0.018	2.4E-01	7.6E-01	4.6E-01
HNSC	KIAA1217_ES_23:24_22_25.1	KIAA1217	ES	23:24	22	25.1	4.4E-22	0.304	0.125	8.0E-02	2.7E-01	2.9E-01
HNSC	KIAA1217_RI_25.2_25.1_25.3	KIAA1217	RI	25.2	25.1	25.3	1.5E-07	0.109	-0.065	2.1E-01	4.3E-01	1.6E-01
HNSC	KIAA1468_ME_24|25_23_26	KIAA1468	ME	24|25	23	26	2.2E-12	0.180	-0.408	7.9E-01	5.6E-01	6.1E-02
HNSC	KIF13A_ES_40_39_41.1	KIF13A	ES	40	39	41.1	6.5E-16	0.227	-0.033	6.6E-02	1.1E-01	2.7E-01

cancer type	id	Gene Symbol	splice_type	Exon	From.Exon	To.Exon	anova.p	adj.r2	r	p.50	p.25	p.10
HNSC	KIF21A_ES_24_23_25.2	KIF21A	ES	24	23	25.2	2.0E-07	0.110	-0.259	6.7E-01	9.3E-01	8.2E-01
HNSC	KIF23_ES_21_20_22.1	KIF23	ES	21	20	22.1	2.6E-15	0.220	0.028	4.7E-01	9.6E-01	8.3E-01
HNSC	KLC1_AD_13.3_13.2_18	KLC1	AD	13.3	13.2	18	5.9E-09	0.130	0.051	1.9E-01	2.9E-02	3.0E-02
HNSC	KLK8_ES_4.2:5_3_6	KLK8	ES	4.2:5	3	6	5.6E-07	0.108	0.088	3.2E-02	1.1E-01	5.9E-01
HNSC	KRAS_ES_6_5_7	KRAS	ES	6	5	7	4.2E-11	0.162	-0.079	2.0E-01	2.9E-01	9.3E-01
HNSC	KTN1_ES_42_41_43	KTN1	ES	42	41	43	2.0E-15	0.221	0.387	4.9E-01	6.4E-02	1.9E-01
HNSC	LEF1_ES_12_11_13	LEF1	ES	12	11	13	2.7E-08	0.126	-0.336	2.0E-02	2.1E-02	2.1E-01
HNSC	LEF1_ES_7_6.1_8	LEF1	ES	7	6.1	8	4.5E-21	0.318	0.214	4.8E-01	2.1E-01	2.5E-01
HNSC	LETMD1_ES_3.2:4:5:6_2_7	LETMD1	ES	3.2:4:5:6	2	7	6.0E-08	0.115	-0.140	1.1E-03	8.4E-02	7.2E-01
HNSC	LGALS3BP_ES_2.3:2.4:2.5:3.1:3.2_2.2_4.	LGALS3BP	ES	2.3:2.4:2.5:3.1:3.2	2.2	4.1	2.1E-08	0.124	-0.285	1.9E-01	3.5E-01	8.3E-01
HNSC	LGALS9_ES_5_4_6	LGALS9	ES	5	4	6	6.6E-08	0.114	-0.150	6.4E-01	7.4E-01	3.3E-01
HNSC	LGMN_ES_2_1_3	LGMN	ES	2	1	3	2.0E-07	0.107	-0.453	2.4E-01	9.5E-03	1.7E-01
HNSC	LIMCH1_ES_36_35_37	LIMCH1	ES	36	35	37	3.1E-06	0.101	0.265	1.5E-01	2.2E-02	9.3E-02
HNSC	LMBR1L_ES_4.1:4.2_3_5	LMBR1L	ES	4.1:4.2	3	5	4.8E-08	0.117	0.035	4.3E-01	2.0E-01	1.2E-01
HNSC	LMO7_ES_10:11_9_12	LMO7	ES	10:11	9	12	1.1E-07	0.112	0.053	5.9E-01	1.9E-02	1.2E-01
HNSC	LMO7_ES_10:11:12_9_13	LMO7	ES	10:11:12	9	13	9.2E-13	0.194	-0.183	2.4E-02	1.3E-01	8.8E-01
HNSC	LMO7_ES_12_9_13	LMO7	ES	12	9	13	4.5E-07	0.106	-0.331	4.0E-01	3.4E-01	7.5E-01
HNSC	LPHN2_ES_15_14_16	LPHN2	ES	15	14	16	1.3E-09	0.149	-0.198	4.6E-01	9.0E-01	6.2E-01
HNSC	LPXN_ES_6_5_7	LPXN	ES	6	5	7	2.9E-07	0.104	-0.519	3.0E-01	1.7E-01	4.1E-01
HNSC	LRRFIP2_ES_20_19_21	LRRFIP2	ES	20	19	21	2.4E-16	0.234	-0.092	7.6E-03	1.6E-01	1.3E-01
HNSC	LTBP3_ES_25_24_26	LTBP3	ES	25	24	26	1.2E-07	0.111	0.491	2.7E-01	2.6E-01	8.9E-01
HNSC	MACF1_ES_103_102_104	MACF1	ES	103	102	104	6.0E-08	0.115	0.232	3.1E-01	1.6E-01	5.5E-01
HNSC	MACF1_ES_107_106_108	MACF1	ES	107	106	108	2.8E-16	0.232	0.272	7.0E-01	5.5E-01	4.1E-01
HNSC	MACF1_ES_43.2:44:45:46:47_42_48	MACF1	ES	43.2:44:45:46:47	42	48	3.1E-08	0.121	-0.249	7.5E-01	4.1E-01	1.1E-01
HNSC	MADD_ES_18_17_19	MADD	ES	18	17	19	2.0E-11	0.170	0.002	2.0E-01	1.0E-01	2.6E-02
HNSC	MAP2K7_ES_2_1_3	MAP2K7	ES	2	1	3	1.4E-08	0.126	-0.190	1.2E-02	3.4E-02	1.5E-02
HNSC	MAP3K3_ES_3_2.2_4	MAP3K3	ES	3	2.2	4	1.1E-08	0.129	-0.023	8.7E-01	5.5E-01	7.3E-01
HNSC	MAP3K7_ES_11_10_12	MAP3K7	ES	11	10	12	1.8E-18	0.261	-0.205	1.9E-01	6.5E-02	3.8E-01
HNSC	MARK2_ES_16.1:16.2_15.2_17	MARK2	ES	16.1:16.2	15.2	17	5.0E-10	0.146	0.023	2.0E-01	4.3E-01	9.4E-01
HNSC	MARK3_ES_17_16_19	MARK3	ES	17	16	19	3.6E-12	0.176	0.003	3.4E-01	9.6E-01	4.3E-01
HNSC	MARK3_ES_17:18_16_19	MARK3	ES	17:18	16	19	4.9E-11	0.160	-0.170	1.5E-01	9.0E-01	6.7E-01
HNSC	MBD1_AA_18.1:18.2:18.3_17_18.4	MBD1	AA	18.1:18.2:18.3	17	18.4	2.3E-08	0.121	-0.081	1.0E+00	5.9E-01	2.1E-01
HNSC	MBNL1_ES_8_7_9	MBNL1	ES	8	7	9	1.5E-11	0.169	-0.309	9.6E-02	5.1E-02	6.5E-01
HNSC	MBNL2_ES_10_8_11	MBNL2	ES	10	8	11	2.4E-07	0.106	0.027	8.1E-01	9.2E-01	7.6E-01
HNSC	MDM1_RI_3.3_3.2_3.4	MDM1	RI	3.3	3.2	3.4	3.4E-07	0.103	-0.277	1.0E-03	2.9E-02	7.2E-01
HNSC	MEFV_ES_6_5_7	MEFV	ES	6	5	7	3.0E-10	0.151	-0.731	8.5E-01	4.6E-01	7.1E-01
HNSC	MEGF8_ES_30_29_31	MEGF8	ES	30	29	31	9.9E-08	0.115	-0.005	2.0E-01	6.9E-01	7.3E-01
HNSC	MEIS1_ES_12.3_12.1_12.5	MEIS1	ES	12.3	12.1	12.5	5.7E-07	0.103	-0.239	9.5E-01	9.2E-01	9.9E-01
HNSC	METTL17_AA_4.1_3_4.2	METTL17	AA	4.1	3	4.2	1.3E-08	0.125	0.325	8.2E-01	4.4E-02	1.3E-01
HNSC	METTL3_RI_8.2_8.1_8.3	METTL3	RI	8.2	8.1	8.3	2.1E-08	0.122	0.190	6.7E-02	1.0E-02	6.9E-03

cancer type	id	Gene Symbol	splice_type	Exon	From.Exon	To.Exon	anova.p	adj.r2	r	p.50	p.25	p.10
HNSC	METTL3_RI_8.4_8.3_8.5	METTL3	RI	8.4	8.3	8.5	5.0E-09	0.131	0.161	2.2E-02	1.2E-01	1.2E-02
HNSC	MFF_ES_8_7_9	MFF	ES	8	7	9	5.9E-09	0.130	-0.159	1.2E-01	1.0E-01	8.3E-01
HNSC	MFF_ES_8:9:10_7_11	MFF	ES	8:09:10	7	11	4.0E-09	0.132	-0.088	2.0E-01	7.1E-01	8.8E-01
HNSC	MFSD12_ES_14_12_15	MFSD12	ES	14	12	15	4.9E-09	0.131	-0.481	9.9E-01	1.7E-01	1.8E-01
HNSC	MPRIP_ES_24_23_25	MPRIP	ES	24	23	25	2.0E-16	0.234	0.083	2.8E-01	2.5E-01	3.8E-01
HNSC	MRPL52_ES_3:4.1_2_5	MRPL52	ES	03:04.1	2	5	7.4E-12	0.172	-0.089	3.2E-01	4.8E-01	1.2E-02
HNSC	MRPL52_RI_1.2:1.3:1.4_1.1_1.5	MRPL52	RI	1.2:1.3:1.4	1.1	1.5	3.5E-10	0.148	-0.001	8.8E-01	8.1E-01	6.4E-01
HNSC	MRPS18C_ES_3_2_4	MRPS18C	ES	3	2	4	3.4E-07	0.103	0.129	7.9E-01	6.8E-01	5.3E-01
HNSC	MTMR3_ES_20_19_21	MTMR3	ES	20	19	21	1.4E-11	0.168	-0.013	2.0E-01	2.0E-01	9.6E-02
HNSC	MYB_ES_17_16_19.1	MYB	ES	17	16	19.1	3.4E-17	0.246	-0.669	9.4E-01	5.7E-01	4.9E-01
HNSC	MYB_ES_17:18_16_19.1	MYB	ES	17:18	16	19.1	3.6E-20	0.322	-0.770	4.4E-01	2.3E-01	3.3E-01
HNSC	MYL12A_ES_1.2:2_1.1_4	MYL12A	ES	1.2:2	1.1	4	2.4E-15	0.220	0.169	7.7E-01	6.0E-01	8.7E-01
HNSC	MYO18A_ES_41_40_42	MYO18A	ES	41	40	42	1.6E-12	0.182	0.034	1.0E-01	6.7E-01	3.1E-01
HNSC	MYO5A_ES_35_34_36	MYO5A	ES	35	34	36	3.7E-16	0.233	-0.547	2.0E-01	1.4E-02	1.0E-02
HNSC	MYO9B_ES_37_36_38.1	MYO9B	ES	37	36	38.1	1.4E-11	0.172	-0.176	2.9E-02	2.0E-01	6.1E-01
HNSC	MYOF_ES_17_16_18	MYOF	ES	17	16	18	3.0E-07	0.105	-0.046	4.4E-01	8.5E-01	8.3E-01
HNSC	NAE1_ES_2.1:3.1_1_4	NAE1	ES	2.1:3.1	1	4	3.0E-07	0.111	-0.068	4.8E-01	6.1E-01	9.8E-02
HNSC	NAGK_ES_8_6.2_9.1	NAGK	ES	8	6.2	9.1	6.9E-06	0.104	-0.038	6.7E-01	3.4E-01	6.8E-01
HNSC	NAP1L1_ES_7.2:7.4_6_7.5	NAP1L1	ES	7.2:7.4	6	7.5	3.1E-08	0.134	-0.091	6.2E-01	6.9E-01	5.8E-01
HNSC	NASP_ES_2_1_3	NASP	ES	2	1	3	6.6E-09	0.129	0.136	7.6E-01	3.5E-01	8.2E-02
HNSC	NAV2_ES_34_33_35	NAV2	ES	34	33	35	1.8E-08	0.131	-0.200	1.5E-01	2.0E-01	8.5E-02
HNSC	NCOR2_AD_46.2_46.1_47	NCOR2	AD	46.2	46.1	47	2.7E-10	0.150	-0.304	3.5E-01	1.4E-01	9.3E-01
HNSC	NCOR2_ES_46.1:46.2_45_47	NCOR2	ES	46.1:46.2	45	47	2.0E-08	0.122	-0.251	9.2E-02	1.8E-01	4.8E-01
HNSC	NDRG2_ES_4.2:4.5_4.1_5.2	NDRG2	ES	4.2:4.5	4.1	5.2	6.3E-07	0.114	0.073	3.2E-01	2.0E-01	2.8E-01
HNSC	NDRG2_ES_6_5.2_7.2	NDRG2	ES	6	5.2	7.2	2.0E-07	0.107	0.110	5.0E-02	4.0E-02	5.7E-01
HNSC	NDUFA3_ES_4.3_4.1_5.1	NDUFA3	ES	4.3	4.1	5.1	1.4E-08	0.124	0.057	3.0E-01	1.1E-01	1.8E-01
HNSC	NDUFA3_RI_4.2_4.1_4.3	NDUFA3	RI	4.2	4.1	4.3	1.9E-07	0.107	-0.197	3.6E-03	2.8E-02	6.3E-02
HNSC	NDUFB5_ES_3.1:3.2:4.1:4.2_1_5	NDUFB5	ES	3.1:3.2:4.1:4.2	1	5	5.9E-08	0.115	0.115	3.1E-01	5.6E-01	9.3E-02
HNSC	NEDD4L_ES_18_17_19	NEDD4L	ES	18	17	19	2.5E-23	0.323	0.255	9.4E-01	3.7E-01	4.9E-01
HNSC	NF1_ES_31_30_32	NF1	ES	31	30	32	2.0E-16	0.241	-0.221	6.2E-01	4.3E-01	5.2E-01
HNSC	NFE2L1_ES_6_5.2_7	NFE2L1	ES	6	5.2	7	5.7E-13	0.188	0.345	2.5E-01	4.6E-01	4.8E-01
HNSC	NFYA_ES_3_2_4	NFYA	ES	3	2	4	2.3E-15	0.222	-0.008	2.8E-01	5.1E-02	4.4E-02
HNSC	NHSL1_ES_6_5_7	NHSL1	ES	6	5	7	1.0E-06	0.103	-0.081	5.8E-01	1.9E-01	4.2E-01
HNSC	NIN_ES_18_17_19	NIN	ES	18	17	19	4.8E-10	0.146	0.022	3.5E-01	1.6E-01	4.9E-01
HNSC	NISCH_RI_14.2_14.1_14.3	NISCH	RI	14.2	14.1	14.3	7.1E-08	0.114	0.334	2.0E-03	6.5E-03	2.7E-03
HNSC	NPHP3_ES_17_16_18	NPHP3	ES	17	16	18	4.2E-07	0.114	-0.335	2.3E-01	4.2E-01	3.2E-01
HNSC	NSFL1C_ES_5.2:7.2_4_7.3	NSFL1C	ES	5.2:7.2	4	7.3	2.4E-16	0.234	-0.102	7.0E-01	4.2E-01	3.7E-01
HNSC	NSUN5_RI_9.2_9.1_9.3	NSUN5	RI	9.2	9.1	9.3	1.7E-07	0.108	-0.082	5.2E-01	2.0E-01	5.5E-02
HNSC	NUMB_ES_13_12_14	NUMB	ES	13	12	14	1.8E-09	0.138	0.149	4.5E-01	9.4E-01	8.1E-01
HNSC	NVL_ES_2_1_3.1	NVL	ES	2	1	3.1	3.6E-08	0.119	-0.055	3.3E-02	3.1E-01	5.5E-01

cancer type	id	Gene Symbol	splice_type	Exon	From.Exon	To.Exon	anova.p	adj.r2	r	p.50	p.25	p.10
HNSC	OCIAD1_AD_2.3:2.4_2.2_3	OCIAD1	AD	2.3:2.4	2.2	3	2.4E-07	0.119	-0.205	2.8E-01	8.9E-01	3.5E-01
HNSC	ODF2L_AA_20.1_19_20.2	ODF2L	AA	20.1	19	20.2	1.1E-12	0.184	-0.462	1.1E-01	6.6E-02	6.8E-01
HNSC	OSBPL3_ES_9_8_10	OSBPL3	ES	9	8	10	1.6E-09	0.140	-0.266	4.8E-01	1.0E-01	8.2E-01
HNSC	OSBPL8_ES_6_5_7.1	OSBPL8	ES	6	5	7.1	1.4E-11	0.175	-0.038	2.6E-01	4.5E-02	3.2E-01
HNSC	OSBPL8_ES_6:7.1_5_7.2	OSBPL8	ES	06:07.1	5	7.2	1.1E-13	0.208	-0.012	4.9E-02	3.0E-03	7.7E-02
HNSC	OSBPL9_ES_17_15_18	OSBPL9	ES	17	15	18	1.1E-07	0.111	-0.246	5.7E-02	4.4E-02	9.9E-01
HNSC	PAM_ES_14_13_15	PAM	ES	14	13	15	3.6E-14	0.205	-0.015	8.5E-01	3.3E-01	3.5E-01
HNSC	PBRM1_ES_28:29_27_30	PBRM1	ES	28:29:00	27	30	5.6E-20	0.282	0.134	6.8E-02	1.3E-01	5.4E-01
HNSC	PBRM1_ES_29_27_30	PBRM1	ES	29	27	30	3.5E-15	0.219	0.082	1.2E-01	2.8E-01	3.8E-01
HNSC	PBRM1_ES_29_28_30	PBRM1	ES	29	28	30	5.3E-11	0.169	-0.007	1.9E-01	1.0E+00	8.4E-01
HNSC	PCBP2_ES_15_14.1_16.1	PCBP2	ES	15	14.1	16.1	3.7E-09	0.133	-0.202	7.5E-01	2.2E-01	9.8E-01
HNSC	PDE4DIP_AA_52.1_51_52.2	PDE4DIP	AA	52.1	51	52.2	9.4E-07	0.108	-0.533	9.3E-02	3.8E-03	2.1E-02
HNSC	PDGFA_ES_6_5_7	PDGFA	ES	6	5	7	8.6E-09	0.128	0.006	2.0E-01	8.4E-01	7.8E-01
HNSC	PHKA1_ES_28_27_30	PHKA1	ES	28	27	30	5.8E-07	0.109	0.013	2.2E-01	9.9E-01	7.2E-01
HNSC	PI4KB_ES_5_4_6	PI4KB	ES	5	4	6	1.6E-08	0.125	0.074	7.0E-02	1.5E-01	4.3E-01
HNSC	PICALM_ES_14.1:14.2_13_15	PICALM	ES	14.1:14.2	13	15	2.8E-08	0.120	-0.045	3.5E-01	8.8E-01	6.9E-01
HNSC	PIK3CD_RI_17.2_17.1_17.3	PIK3CD	RI	17.2	17.1	17.3	5.8E-09	0.134	-0.444	1.1E-02	2.0E-02	3.8E-02
HNSC	PLCD4_ES_13_12_14	PLCD4	ES	13	12	14	3.6E-11	0.200	-0.633	4.0E-01	7.9E-01	7.6E-01
HNSC	PLD3_ES_4:5.1_1.2_5.2	PLD3	ES	04:05.1	1.2	5.2	3.7E-09	0.137	0.275	9.0E-01	5.8E-01	4.8E-01
HNSC	PLEKHA1_ES_15.1_14_16	PLEKHA1	ES	15.1	14	16	1.1E-19	0.276	-0.353	8.6E-01	7.1E-01	5.2E-01
HNSC	PLEKHM2_ES_7_6_8	PLEKHM2	ES	7	6	8	1.3E-08	0.125	0.248	6.5E-01	2.9E-01	1.2E-01
HNSC	PLOD2_ES_15_14_16	PLOD2	ES	15	14	16	2.2E-23	0.323	-0.041	7.6E-01	8.0E-01	7.2E-01
HNSC	PLSCR1_ES_4_1_5	PLSCR1	ES	4	1	5	6.5E-08	0.115	0.079	9.3E-03	1.4E-01	5.3E-01
HNSC	PNPLA2_ES_2_1_3	PNPLA2	ES	2	1	3	7.0E-08	0.148	0.187	8.2E-01	6.9E-01	6.5E-01
HNSC	PPIA_AD_2.2_2.1_3	PPIA	AD	2.2	2.1	3	3.1E-09	0.134	-0.122	5.6E-01	7.0E-01	2.4E-01
HNSC	PPP1CB_RI_1.2:1.3_1.1_1.4	PPP1CB	RI	1.2:1.3	1.1	1.4	3.0E-10	0.149	0.207	4.3E-01	6.0E-01	9.8E-01
HNSC	PPP3CB_ES_16_15.1_17	PPP3CB	ES	16	15.1	17	4.4E-09	0.133	-0.034	3.6E-01	8.6E-01	9.9E-01
HNSC	PPP4R1_ES_15_14_16	PPP4R1	ES	15	14	16	1.4E-07	0.109	-0.222	6.5E-01	7.7E-01	8.5E-01
HNSC	PQBP1_AD_1.2:1.3_1.1_2.3	PQBP1	AD	1.2:1.3	1.1	2.3	1.3E-07	0.110	-0.082	8.4E-01	3.9E-01	6.3E-01
HNSC	PRMT1_ES_4.2:5_4.1_6	PRMT1	ES	4.2:5	4.1	6	5.1E-09	0.131	-0.012	5.2E-01	3.9E-01	7.8E-01
HNSC	PRMT2_ES_1.3_1.1_2	PRMT2	ES	1.3	1.1	2	1.8E-08	0.123	-0.118	7.9E-01	8.5E-01	4.6E-01
HNSC	PRMT7_AA_17.1_16_17.2	PRMT7	AA	17.1	16	17.2	1.6E-08	0.124	-0.183	7.9E-02	2.8E-02	2.4E-01
HNSC	PRRC2B_ES_31_30_32	PRRC2B	ES	31	30	32	1.8E-08	0.123	0.020	1.0E-02	6.6E-03	3.7E-01
HNSC	PSAP_AA_8.1_7_8.2	PSAP	AA	8.1	7	8.2	5.1E-07	0.100	-0.042	3.7E-01	1.8E-02	4.2E-01
HNSC	PSMB5_AD_1.2:1.3_1.1_2	PSMB5	AD	1.2:1.3	1.1	2	7.1E-08	0.116	-0.109	1.3E-01	3.3E-01	7.3E-02
HNSC	PSTPIP1_ES_3_2.2_5	PSTPIP1	ES	3	2.2	5	1.1E-08	0.127	-0.581	1.1E-01	4.7E-02	6.1E-01
HNSC	PTK2B_ES_28_27_29	PTK2B	ES	28	27	29	3.6E-17	0.244	0.133	1.4E-01	4.1E-03	8.1E-02
HNSC	R3HDM1_ES_16_15_17	R3HDM1	ES	16	15	17	2.5E-11	0.167	0.199	1.7E-01	1.7E-02	2.2E-01
HNSC	RAB6A_ME_5|6_4_7	RAB6A	ME	5|6	4	7	3.0E-08	0.119	0.027	7.1E-01	4.6E-01	3.4E-01
HNSC	RAI14_ES_18_17_19	RAI14	ES	18	17	19	4.3E-16	0.233	0.110	3.4E-02	3.4E-02	3.8E-02

cancer type	id	Gene Symbol	splice_type	Exon	From.Exon	To.Exon	anova.p	adj.r2	r	p.50	p.25	p.10
HNSC	RALGAPB_AA_16.1_15_16.2	RALGAPB	AA	16.1	15	16.2	7.2E-07	0.102	0.017	6.2E-01	8.2E-01	3.8E-01
HNSC	RALGPS2_ES_15_14_16	RALGPS2	ES	15	14	16	2.0E-10	0.153	0.158	3.9E-01	4.7E-01	7.2E-01
HNSC	RBFOX2_ES_12_11.2_14	RBFOX2	ES	12	11.2	14	1.9E-07	0.107	-0.334	2.2E-02	1.2E-02	2.5E-01
HNSC	RBM41_ES_5_4_6.1	RBM41	ES	5	4	6.1	2.4E-08	0.123	-0.189	7.5E-01	9.6E-01	9.2E-01
HNSC	RBP7_ES_2_1_3	RBP7	ES	2	1	3	1.3E-07	0.118	-0.740	4.9E-01	3.9E-01	5.4E-01
HNSC	REEP5_ES_4:5_3_6	REEP5	ES	4:05	3	6	4.5E-07	0.101	-0.227	3.1E-01	1.6E-02	1.3E-01
HNSC	RGS12_ES_19_18_20.1	RGS12	ES	19	18	20.1	3.8E-07	0.102	-0.192	6.8E-01	6.4E-01	6.9E-01
HNSC	RHEB_ES_1.2:3_1.1_4	RHEB	ES	1.2:3	1.1	4	4.6E-07	0.113	-0.228	4.1E-02	2.3E-01	3.6E-02
HNSC	RNF32_AA_12.1_11_12.2	RNF32	AA	12.1	11	12.2	5.4E-14	0.212	-0.635	3.6E-01	9.3E-01	7.5E-01
HNSC	RPRD2_ES_4_3.1_5	RPRD2	ES	4	3.1	5	9.9E-17	0.240	-0.311	7.4E-01	9.3E-01	3.1E-01
HNSC	RPS15_RI_1.2:1.3:1.4_1.1_1.5	RPS15	RI	1.2:1.3:1.4	1.1	1.5	3.4E-09	0.134	-0.186	6.3E-01	3.7E-02	2.4E-04
HNSC	RPS24_AA_5.1_4_5.2	RPS24	AA	5.1	4	5.2	8.3E-39	0.476	0.023	9.9E-01	1.0E-01	5.4E-01
HNSC	RPS24_ES_5.1:5.2_4_6	RPS24	ES	5.1:5.2	4	6	7.0E-30	0.390	-0.033	8.3E-01	4.2E-01	8.8E-02
HNSC	RPS24_ES_5.2_4_6	RPS24	ES	5.2	4	6	7.2E-16	0.227	-0.122	5.3E-01	7.9E-01	6.7E-01
HNSC	RPS3_ES_2.1_1_3.1	RPS3	ES	2.1	1	3.1	1.3E-11	0.170	0.017	8.9E-01	1.6E-01	2.6E-01
HNSC	RREB1_ES_12_11_13	RREB1	ES	12	11	13	3.2E-12	0.179	-0.224	2.3E-01	1.1E-01	7.9E-01
HNSC	RYR1_ES_94_93_95	RYR1	ES	94	93	95	9.3E-08	0.120	0.525	1.8E-01	6.5E-01	8.8E-01
HNSC	S100A6_AA_3.1_2_3.2	S100A6	AA	3.1	2	3.2	1.7E-07	0.114	-0.090	1.6E-02	2.2E-01	4.6E-02
HNSC	SCRIB_ES_17_16_18	SCRIB	ES	17	16	18	1.3E-07	0.110	-0.397	5.8E-01	6.6E-01	1.1E-01
HNSC	SDAD1_AA_3.1:3.2_2_3.3	SDAD1	AA	3.1:3.2	2	3.3	4.1E-10	0.148	-0.214	1.0E+00	5.1E-01	7.2E-01
HNSC	SDSL_ES_2_1_3	SDSL	ES	2	1	3	2.9E-10	0.150	-0.390	1.4E-03	2.8E-02	2.9E-02
HNSC	SEC16A_ES_25_24_26	SEC16A	ES	25	24	26	1.1E-09	0.141	0.005	1.3E-02	2.6E-04	1.4E-01
HNSC	SEC31A_ES_16:17_15_18	SEC31A	ES	16:17	15	18	4.5E-17	0.243	0.158	5.3E-01	4.9E-01	1.9E-01
HNSC	SEC31A_ES_26.1_25.1_28	SEC31A	ES	26.1	25.1	28	3.3E-28	0.374	-0.146	3.9E-02	3.7E-02	6.0E-01
HNSC	SEC31A_ES_26.1:26.2_25.1_28	SEC31A	ES	26.1:26.2	25.1	28	8.4E-30	0.389	-0.140	1.9E-01	8.6E-02	7.3E-02
HNSC	SEC31A_ES_26.1:26.2:27_25.1_28	SEC31A	ES	26.1:26.2:27	25.1	28	4.1E-28	0.373	-0.181	7.7E-01	6.4E-02	4.6E-02
HNSC	SENP6_ES_8_6_9	SENP6	ES	8	6	9	2.7E-11	0.166	0.083	2.1E-01	2.1E-02	1.1E-01
HNSC	SEPT6_ES_12_11.1_13.1	SEPT6	ES	12	11.1	13.1	2.3E-09	0.141	0.108	1.6E-02	1.1E-03	7.4E-04
HNSC	SEPT9_ES_4:5_1_10.1	SEPT9	ES	4:05	1	10.1	1.9E-06	0.101	-0.120	6.3E-01	3.8E-02	8.9E-01
HNSC	SH3D19_ES_13_12_14	SH3D19	ES	13	12	14	1.5E-10	0.154	0.086	1.9E-01	1.5E-01	3.4E-01
HNSC	SLAIN2_ES_8_6_9	SLAIN2	ES	8	6	9	3.8E-10	0.148	0.150	3.0E-01	5.3E-01	4.3E-01
HNSC	SLC15A3_ES_5_4_6	SLC15A3	ES	5	4	6	2.0E-09	0.137	-0.053	6.2E-01	6.4E-01	2.9E-01
HNSC	SLC25A10_ES_7_6_8.1	SLC25A10	ES	7	6	8.1	9.9E-10	0.143	0.082	2.7E-01	6.9E-01	7.4E-02
HNSC	SLC25A16_ES_2.1:2.2_1_3	SLC25A16	ES	2.1:2.2	1	3	2.3E-08	0.137	0.088	7.6E-01	5.8E-01	3.9E-02
HNSC	SLC37A2_ES_12_11_13	SLC37A2	ES	12	11	13	1.4E-09	0.139	0.159	1.9E-01	8.3E-01	8.9E-01
HNSC	SLC37A2_ES_18_17_19	SLC37A2	ES	18	17	19	2.0E-08	0.123	0.464	4.5E-01	2.8E-02	6.7E-01
HNSC	SLK_ES_13_12_14	SLK	ES	13	12	14	2.5E-13	0.193	0.250	9.1E-01	1.1E-01	1.8E-01
HNSC	SLMAP_ES_25_24_26	SLMAP	ES	25	24	26	4.4E-10	0.147	-0.080	4.0E-01	2.4E-01	4.9E-01
HNSC	SMARCC2_ES_28.3_28.1_29	SMARCC2	ES	28.3	28.1	29	3.4E-13	0.201	0.279	8.4E-01	5.4E-01	4.6E-01
HNSC	SMC5_ES_19_18_20	SMC5	ES	19	18	20	1.4E-08	0.133	-0.294	4.9E-02	1.8E-02	2.3E-02

cancer type	id	Gene Symbol	splice_type	Exon	From.Exon	To.Exon	anova.p	adj.r2	r	p.50	p.25	p.10
HNSC	SNX14_ES_16_15_17	SNX14	ES	16	15	17	2.0E-09	0.137	-0.050	9.3E-01	7.3E-01	5.9E-01
HNSC	SNX5_RI_1.2_1.1_1.3	SNX5	RI	1.2	1.1	1.3	1.1E-10	0.155	-0.182	1.4E-01	5.6E-03	9.0E-02
HNSC	SPAG9_ES_30_29_31	SPAG9	ES	30	29	31	1.6E-15	0.222	0.038	4.6E-01	5.9E-01	3.2E-01
HNSC	SREBF1_ES_2_1_3.2	SREBF1	ES	2	1	3.2	1.7E-18	0.263	0.688	7.7E-01	6.7E-01	5.1E-02
HNSC	SSBP3_ES_7_6_8	SSBP3	ES	7	6	8	1.5E-07	0.109	0.046	4.4E-01	6.4E-01	5.4E-01
HNSC	SSH1_ES_12_11_13	SSH1	ES	12	11	13	1.4E-07	0.112	0.039	5.3E-01	3.3E-01	1.0E-01
HNSC	ST7_ES_10_9_11.1	ST7	ES	10	9	11.1	7.0E-16	0.228	0.066	6.3E-02	1.1E-03	1.9E-03
HNSC	STK40_ES_2_1_3	STK40	ES	2	1	3	1.9E-07	0.107	-0.055	2.3E-01	1.0E+00	3.1E-01
HNSC	STRADA_RI_12.4_12.3_12.5	STRADA	RI	12.4	12.3	12.5	2.6E-07	0.105	0.186	4.5E-01	1.4E-01	6.7E-02
HNSC	STX2_ES_10_9_11	STX2	ES	10	9	11	1.0E-10	0.159	-0.242	3.7E-01	6.6E-01	4.1E-01
HNSC	SULF2_ES_20_19.2_21	SULF2	ES	20	19.2	21	2.6E-14	0.206	-0.423	5.2E-01	2.2E-01	4.7E-01
HNSC	SUPT4H1_AD_2.4_2.3_3.2	SUPT4H1	AD	2.4	2.3	3.2	1.5E-08	0.124	-0.111	4.1E-03	5.5E-02	8.6E-02
HNSC	SVIL_ES_10:11:12_9_13	SVIL	ES	10:11:12	9	13	4.1E-17	0.245	0.248	7.7E-01	6.1E-01	4.8E-01
HNSC	SVIL_ES_16_15_17	SVIL	ES	16	15	17	8.3E-09	0.129	0.360	5.1E-01	4.4E-01	7.4E-01
HNSC	SYK_ES_9_8_10	SYK	ES	9	8	10	6.0E-11	0.160	-0.422	2.5E-02	8.6E-02	6.1E-01
HNSC	SYNE2_ES_118_117.2_119.2	SYNE2	ES	118	117.2	119.2	1.6E-10	0.154	0.013	2.3E-03	8.3E-03	7.2E-02
HNSC	SYNM_RI_6.2_6.1_6.3	SYNM	RI	6.2	6.1	6.3	5.0E-08	0.116	-0.489	3.5E-01	9.2E-01	4.4E-01
HNSC	SYTL2_ES_13_12.2_14	SYTL2	ES	13	12.2	14	8.3E-14	0.231	-0.061	8.8E-01	9.8E-01	3.1E-01
HNSC	TACC2_AA_15.1_13_15.2	TACC2	AA	15.1	13	15.2	3.3E-07	0.104	-0.293	1.4E-01	2.5E-01	7.4E-01
HNSC	TACC2_ES_20:21_19_22	TACC2	ES	20:21	19	22	7.1E-10	0.144	0.175	1.7E-02	1.6E-01	1.2E-02
HNSC	TACC2_ES_4:5.1:5.2_3_8	TACC2	ES	4:5.1:5.2	3	8	7.8E-17	0.266	-0.295	6.8E-01	9.3E-01	9.0E-01
HNSC	TBC1D13_ES_3_2_4	TBC1D13	ES	3	2	4	6.8E-09	0.138	-0.044	5.4E-01	5.8E-01	6.2E-01
HNSC	TBC1D23_ES_15_14_16	TBC1D23	ES	15	14	16	7.4E-19	0.265	0.075	3.4E-02	5.8E-02	1.4E-01
HNSC	TBC1D4_ES_11_10_13	TBC1D4	ES	11	10	13	2.9E-09	0.143	-0.115	2.3E-01	7.0E-02	8.0E-01
HNSC	TCAIM_ES_8_7.2_9	TCAIM	ES	8	7.2	9	9.8E-08	0.112	-0.199	2.0E-01	9.8E-01	8.7E-01
HNSC	TCF20_ES_4.1:4.2_3_5	TCF20	ES	4.1:4.2	3	5	8.9E-13	0.185	0.213	9.5E-01	5.0E-01	9.8E-01
HNSC	TCF20_ES_4.2_3_5	TCF20	ES	4.2	3	5	2.8E-11	0.164	0.186	4.9E-01	7.5E-01	4.4E-01
HNSC	TIA1_ES_5_4_7	TIA1	ES	5	4	7	2.6E-11	0.165	-0.164	7.4E-01	5.7E-01	8.0E-01
HNSC	TIA1_ES_5:6_4_7	TIA1	ES	5:06	4	7	8.2E-11	0.158	-0.138	4.2E-01	4.9E-01	9.6E-01
HNSC	TJP1_ES_23_22_24	TJP1	ES	23	22	24	3.5E-09	0.134	-0.115	9.6E-01	1.4E-01	6.0E-01
HNSC	TM2D2_AA_2.1:2.2_1.2_2.3	TM2D2	AA	2.1:2.2	1.2	2.3	1.4E-07	0.109	-0.089	4.4E-01	8.0E-01	2.4E-01
HNSC	TMEM107_RI_3.3_3.2_3.4	TMEM107	RI	3.3	3.2	3.4	9.0E-11	0.157	-0.299	1.0E-01	1.8E-02	1.3E-01
HNSC	TMEM180_ES_6_5_7	TMEM180	ES	6	5	7	1.6E-08	0.124	-0.175	2.0E-01	9.2E-01	3.7E-01
HNSC	TMEM91_RI_7.2_7.1_7.3	TMEM91	RI	7.2	7.1	7.3	8.3E-08	0.113	-0.497	1.8E-01	2.3E-01	6.1E-02
HNSC	TMEM91_RI_7.2:7.3_7.1_7.4	TMEM91	RI	7.2:7.3	7.1	7.4	1.5E-08	0.124	-0.509	1.8E-02	1.7E-03	9.4E-02
HNSC	TMEM91_RI_7.2:7.3:7.4_7.1_7.5	TMEM91	RI	7.2:7.3:7.4	7.1	7.5	8.3E-08	0.113	-0.541	1.6E-01	2.2E-02	2.0E-01
HNSC	TMX2_ES_3.2:3.3_2_4	TMX2	ES	3.2:3.3	2	4	1.1E-09	0.141	-0.197	6.3E-01	8.7E-01	6.0E-01
HNSC	TNC_ES_12:13:14:15:16:17_11_19	TNC	ES	12:13:14:15:16:17	11	19	3.2E-09	0.158	0.043	7.5E-01	4.0E-01	9.2E-01
HNSC	TNC_ES_12:13:14:15:16:17:19_11_20	TNC	ES	:13:14:15:16:17:	11	20	8.0E-13	0.197	0.182	4.7E-01	9.0E-01	9.8E-01
HNSC	TNC_ES_12:13:14:15:16:18_11_19	TNC	ES	12:13:14:15:16:18	11	19	2.0E-10	0.153	0.189	1.0E+00	8.8E-01	5.5E-01

cancer type	id	Gene Symbol	splice_type	Exon	From.Exon	To.Exon	anova.p	adj.r2	r	p.50	p.25	p.10
HNSC	TNC_ES_12:13:14:15:16:18:19_11_20	TNC	ES	2:13:14:15:16:18:	11	20	1.0E-21	0.301	0.322	3.0E-01	9.4E-01	5.5E-01
HNSC	TNC_ES_12:13:14:15:16:19_11_20	TNC	ES	12:13:14:15:16:19	11	20	1.7E-19	0.273	0.273	2.0E-01	9.9E-01	2.3E-01
HNSC	TNC_ES_12:13:15:16_11_19	TNC	ES	12:13:15:16	11	19	3.1E-18	0.267	0.138	6.7E-01	3.6E-01	7.9E-01
HNSC	TNC_ES_12:13:15:16:18_11_19	TNC	ES	12:13:15:16:18	11	19	6.0E-20	0.290	0.179	8.1E-01	4.7E-01	1.6E-01
HNSC	TNC_ES_12:13:15:16:18:19_11_20	TNC	ES	12:13:15:16:18:19	11	20	4.4E-24	0.335	0.304	2.0E-01	8.1E-01	6.5E-01
HNSC	TNC_ES_12:13:15:16:19_11_20	TNC	ES	12:13:15:16:19	11	20	4.2E-30	0.400	0.316	3.7E-01	4.2E-01	8.5E-01
HNSC	TNC_ES_16:19_11_20	TNC	ES	16:19	11	20	1.5E-10	0.163	-0.020	9.2E-01	5.1E-01	1.5E-01
HNSC	TNFRSF25_RI_6.3:6.4_6.2_6.5	TNFRSF25	RI	6.3:6.4	6.2	6.5	2.1E-07	0.108	0.075	5.7E-01	4.4E-01	8.9E-01
HNSC	TPM1_AD_12.2_12.1_13.1	TPM1	AD	12.2	12.1	13.1	2.1E-07	0.129	-0.317	2.0E-01	3.5E-01	5.6E-01
HNSC	TPM2_ES_6_5_7	TPM2	ES	6	5	7	1.1E-05	0.114	-0.580	3.6E-01	1.9E-01	9.8E-02
HNSC	TRA2A_ES_3.1:3.2_1_4	TRA2A	ES	3.1:3.2	1	4	1.1E-09	0.141	0.183	2.5E-01	2.0E-01	9.5E-01
HNSC	TRA2B_ES_2_1_3	TRA2B	ES	2	1	3	3.4E-19	0.269	0.437	6.3E-01	6.5E-01	8.4E-01
HNSC	TRERF1_ES_8.1:8.2_7_9	TRERF1	ES	8.1:8.2	7	9	5.2E-08	0.120	0.045	7.3E-01	5.3E-01	3.1E-01
HNSC	TROAP_RI_2.5:2.6:2.7_2.4_2.8	TROAP	RI	2.5:2.6:2.7	2.4	2.8	1.2E-08	0.128	0.045	2.6E-01	3.9E-02	1.2E-02
HNSC	TRPM2_ES_29_28_30	TRPM2	ES	29	28	30	3.2E-07	0.116	-0.373	9.3E-01	1.8E-01	8.3E-02
HNSC	TSC2_ES_27_26_28.1	TSC2	ES	27	26	28.1	1.5E-12	0.182	-0.127	9.3E-01	3.2E-01	2.9E-01
HNSC	TSC2_ES_27_26_28.2	TSC2	ES	27	26	28.2	5.5E-08	0.133	-0.149	1.5E-01	3.7E-01	5.5E-01
HNSC	TSC2_ES_27:28.1_26_28.2	TSC2	ES	27:28.1	26	28.2	1.1E-13	0.198	-0.088	1.1E-01	8.9E-02	4.4E-01
HNSC	UAP1_ES_9.2_8_10	UAP1	ES	9.2	8	10	1.4E-08	0.125	0.265	7.0E-02	1.7E-01	7.3E-03
HNSC	UPP1_ES_5:6.1:6.2:7_4_9	UPP1	ES	5:6.1:6.2:7	4	9	4.5E-09	0.132	0.161	8.5E-01	3.3E-01	5.7E-01
HNSC	UQCRB_RI_6.2:6.3_6.1_6.4	UQCRB	RI	6.2:6.3	6.1	6.4	2.3E-07	0.110	-0.133	8.2E-03	6.9E-03	5.8E-02
HNSC	USO1_ES_14_13_15	USO1	ES	14	13	15	2.2E-09	0.137	0.184	3.0E-01	1.9E-01	1.9E-01
HNSC	VLDLR_ES_16_15_17	VLDLR	ES	16	15	17	5.9E-24	0.330	-0.136	4.9E-02	1.1E-02	3.4E-02
HNSC	VPS29_ES_3.1_1_5	VPS29	ES	3.1	1	5	1.0E-07	0.111	-0.128	8.4E-01	4.1E-01	1.9E-01
HNSC	VPS39_ES_3_2_4	VPS39	ES	3	2	4	6.1E-07	0.101	-0.059	2.0E-01	1.9E-01	3.8E-01
HNSC	WDFY3_ES_45_44_46	WDFY3	ES	45	44	46	8.9E-08	0.115	-0.084	3.4E-01	1.2E-01	4.2E-01
HNSC	WDR13_RI_1.2_1.1_1.3	WDR13	RI	1.2	1.1	1.3	3.5E-07	0.103	-0.185	6.4E-01	1.9E-01	8.7E-01
HNSC	WIPI2_ES_2_1_3	WIPI2	ES	2	1	3	1.8E-08	0.124	-0.015	8.1E-02	3.3E-02	2.0E-01
HNSC	XG_ES_8_7_9	XG	ES	8	7	9	2.8E-08	0.125	-0.395	3.3E-01	9.4E-02	6.0E-01
HNSC	ZMYND8_AD_19.2_19.1_20	ZMYND8	AD	19.2	19.1	20	1.2E-08	0.126	0.049	3.5E-01	9.6E-01	3.9E-01
HNSC	ZMYND8_ES_26_25_27	ZMYND8	ES	26	25	27	2.7E-08	0.120	0.009	9.9E-01	3.4E-01	9.2E-01
HNSC	ZNF197_ES_3_2.3_4	ZNF197	ES	3	2.3	4	7.5E-07	0.104	-0.087	8.2E-01	8.9E-01	6.9E-01
HNSC	ZNF226_RI_7.2_7.1_7.3	ZNF226	RI	7.2	7.1	7.3	1.2E-07	0.110	0.109	2.1E-03	6.1E-04	1.2E-02
HNSC	ZNF227_ES_3_2.2_4.1	ZNF227	ES	3	2.2	4.1	1.4E-07	0.109	-0.254	7.0E-01	8.2E-01	3.3E-01
HNSC	ZNF384_ES_8_7_9	ZNF384	ES	8	7	9	1.2E-11	0.169	0.057	1.5E-01	1.2E-01	5.1E-02
HNSC	ZNF586_ES_3_1_4	ZNF586	ES	3	1	4	7.9E-07	0.104	-0.113	2.8E-01	5.3E-01	7.0E-01
HNSC	ZNF821_ES_4_3.3_6	ZNF821	ES	4	3.3	6	7.2E-09	0.138	-0.204	9.8E-02	1.2E-01	1.9E-01
HNSC	ZRANB2_ES_10_9_11	ZRANB2	ES	10	9	11	5.9E-13	0.188	0.199	3.4E-01	5.1E-01	3.5E-01
KICH	ALKBH6_RI_1.2_1.1_1.3	ALKBH6	RI	1.2	1.1	1.3	3.7E-04	0.168	-0.187	7.1E-01	8.8E-01	3.5E-01
KICH	ANK3_ES_26_25_27	ANK3	ES	26	25	27	8.6E-05	0.294	0.174	6.5E-01	7.4E-01	4.3E-01

cancer type	id	Gene Symbol	splice_type	Exon	From.Exon	To.Exon	anova.p	adj.r2	r	p.50	p.25	p.10
KICH	ANK3_ME_26|27_25_29.1	ANK3	ME	26|27	25	29.1	2.7E-04	0.220	0.295	9.4E-01	5.5E-01	6.1E-01
KICH	ATG16L1_ES_8_7_10	ATG16L1	ES	8	7	10	1.9E-05	0.260	0.362	3.3E-01	4.5E-01	5.9E-01
KICH	ATXN2L_AA_22.4:22.5:22.6_22.1_22.7	ATXN2L	AA	22.4:22.5:22.6	22.1	22.7	7.1E-04	0.152	0.120	7.5E-01	7.5E-01	7.5E-01
KICH	AXIN1_ES_9_8_10	AXIN1	ES	9	8	10	2.8E-05	0.230	-0.128	5.0E-01	2.5E-01	3.4E-01
KICH	BTBD3_RI_2.2_2.1_2.3	BTBD3	RI	2.2	2.1	2.3	6.0E-06	0.268	0.238	6.9E-01	6.0E-01	9.6E-01
KICH	BUB3_AA_8.1_7_8.2	BUB3	AA	8.1	7	8.2	9.8E-04	0.144	0.245	8.4E-01	9.5E-01	8.0E-01
KICH	C14orf2_ES_5:6_2_7.1	C14orf2	ES	5:06	2	7.1	4.1E-05	0.252	0.465	4.1E-01	6.1E-01	5.6E-01
KICH	CAMK2D_AA_14.1_13_14.2	CAMK2D	AA	14.1	13	14.2	1.2E-05	0.262	0.298	3.2E-01	3.3E-01	2.9E-01
KICH	CCDC106_RI_1.2:1.3:1.4_1.1_1.5	CCDC106	RI	1.2:1.3:1.4	1.1	1.5	3.1E-04	0.172	0.166	9.0E-02	1.2E-01	1.4E-01
KICH	CD44_ES_12.1:13:14_5_15	CD44	ES	12.1:13:14	5	15	2.1E-04	0.184	0.488	6.8E-01	1.0E+00	1.8E-01
KICH	CD47_ES_9:10_8_11	CD47	ES	9:10	8	11	1.6E-04	0.189	0.137	5.6E-01	7.0E-01	4.8E-01
KICH	CTNND1_ES_21_20_22.1	CTNND1	ES	21	20	22.1	5.2E-04	0.160	0.450	8.4E-01	9.2E-01	9.7E-01
KICH	CTNND1_ES_21:22.1_20_22.2	CTNND1	ES	21:22.1	20	22.2	5.7E-04	0.160	0.360	7.7E-01	4.6E-01	1.0E+00
KICH	DEPDC5_AA_28.1_27.1_28.2	DEPDC5	AA	28.1	27.1	28.2	2.9E-05	0.239	0.318	6.4E-01	2.8E-01	1.0E+00
KICH	DERA_ES_4.2_3_5	DERA	ES	4.2	3	5	2.9E-05	0.258	0.101	2.5E-01	4.5E-02	5.6E-02
KICH	DYRK1B_AA_10.1:10.2_9_10.3	DYRK1B	AA	10.1:10.2	9	10.3	9.2E-04	0.146	-0.473	3.4E-01	2.8E-01	3.3E-01
KICH	EEF1D_ES_8.3:9:10.1:11:12.1:12.2:13.1_	EEF1D	ES	10.1:11:12.1:12.:	8.2	13.2	3.7E-04	0.168	0.225	5.3E-01	3.6E-01	3.7E-01
KICH	ELMO1_AD_1.2_1.1_3	ELMO1	AD	1.2	1.1	3	2.9E-04	0.226	0.038	5.1E-01	7.9E-01	8.5E-01
KICH	EPOR_ES_4.1:4.2_3_5	EPOR	ES	4.1:4.2	3	5	3.4E-06	0.277	0.088	5.6E-01	8.3E-01	1.3E-01
KICH	EXOC7_ES_7_6_8.2	EXOC7	ES	7	6	8.2	8.1E-04	0.151	0.338	2.3E-01	3.4E-01	5.0E-01
KICH	FAM13A_AA_16.1_15_16.2	FAM13A	AA	16.1	15	16.2	5.5E-05	0.214	0.581	2.0E-01	1.7E-02	5.0E-02
KICH	FAM21C_ES_11_10_12	FAM21C	ES	11	10	12	8.2E-04	0.158	0.492	7.9E-01	3.9E-01	1.0E+00
KICH	FBXO44_ES_3.1:3.2_2_4	FBXO44	ES	3.1:3.2	2	4	8.1E-04	0.151	0.244	7.6E-01	6.8E-01	1.0E+00
KICH	FBXW11_ES_3_1_4	FBXW11	ES	3	1	4	4.0E-04	0.228	-0.276	2.2E-01	8.7E-01	4.2E-01
KICH	FIP1L1_ES_9_8_10	FIP1L1	ES	9	8	10	1.0E-05	0.252	0.034	3.3E-01	5.1E-01	7.4E-01
KICH	GK_ES_12_11_13	GK	ES	12	11	13	6.4E-04	0.157	-0.159	8.1E-01	2.1E-01	4.8E-01
KICH	GOLGA4_ES_24_23_25	GOLGA4	ES	24	23	25	2.2E-04	0.181	0.161	6.8E-01	8.1E-01	5.4E-01
KICH	GPR89A_ES_3_1_4	GPR89A	ES	3	1	4	8.1E-04	0.156	0.399	8.9E-01	7.9E-01	4.0E-01
KICH	GTF3C1_AD_34.2_34.1_35	GTF3C1	AD	34.2	34.1	35	3.0E-04	0.173	-0.369	2.8E-01	6.3E-01	5.3E-01
KICH	GUSB_ES_3.1:3.2:4.1:4.2_2_5.1	GUSB	ES	3.1:3.2:4.1:4.2	2	5.1	3.1E-05	0.291	-0.091	2.8E-01	1.4E-02	1.9E-02
KICH	HHLA3_ES_4_3_5	HHLA3	ES	4	3	5	9.3E-05	0.220	-0.140	8.1E-01	1.1E-01	1.0E+00
KICH	LIMCH1_ES_20_12.1_21	LIMCH1	ES	20	12.1	21	1.9E-07	0.351	-0.450	8.6E-01	7.5E-01	7.4E-01
KICH	MARCH8_ES_7_6_8	MARCH8	ES	7	6	8	9.4E-06	0.261	0.029	8.4E-01	1.0E+00	6.8E-01
KICH	MFSD9_AD_1.2_1.1_2.1	MFSD9	AD	1.2	1.1	2.1	7.0E-04	0.185	0.056	4.0E-02	1.5E-01	2.6E-01
KICH	MTMR3_ES_18_17_19	MTMR3	ES	18	17	19	6.8E-05	0.209	0.054	8.8E-01	8.9E-01	4.6E-01
KICH	MYOF_ES_17_16_18	MYOF	ES	17	16	18	3.8E-07	0.346	0.090	7.8E-01	9.7E-01	3.8E-01
KICH	NAE1_ES_2.1_1_3.1	NAE1	ES	2.1	1	3.1	3.8E-04	0.173	0.053	6.2E-01	8.6E-01	3.8E-01
KICH	NDUFAF1_ES_4_3_5	NDUFAF1	ES	4	3	5	2.7E-05	0.230	-0.075	1.9E-01	1.2E-01	6.7E-01
KICH	NEK1_ES_19_18_20	NEK1	ES	19	18	20	7.3E-04	0.201	0.256	6.9E-01	7.5E-01	2.5E-02
KICH	NME6_ES_5.1:5.2_4_6	NME6	ES	5.1:5.2	4	6	1.1E-04	0.203	-0.488	6.9E-01	5.3E-01	5.4E-01

cancer type	id	Gene Symbol	splice_type	Exon	From.Exon	To.Exon	anova.p	adj.r2	r	p.50	p.25	p.10
KICH	NRCAM_ES_12_11_13	NRCAM	ES	12	11	13	2.0E-04	0.251	-0.162	6.2E-01	9.1E-01	7.6E-01
KICH	NTMT1_ES_4.1_2_5.1	NTMT1	ES	4.1	2	5.1	9.8E-04	0.144	-0.181	1.5E-01	2.9E-01	3.4E-01
KICH	NUDT2_ES_2.1:2.2_1_3	NUDT2	ES	2.1:2.2	1	3	1.0E-04	0.199	-0.304	5.4E-01	8.2E-01	9.2E-01
KICH	NUDT22_RI_1.3_1.2_1.4	NUDT22	RI	1.3	1.2	1.4	6.4E-04	0.155	-0.294	4.9E-02	3.3E-01	1.3E-01
KICH	OSBPL3_ES_9_8_10	OSBPL3	ES	9	8	10	7.8E-04	0.150	-0.138	9.8E-01	9.8E-01	5.5E-01
KICH	PAX8_ES_9.1:9.2_8_10	PAX8	ES	9.1:9.2	8	10	1.1E-04	0.201	0.182	3.9E-01	9.3E-01	1.0E+00
KICH	PAX8_ES_9.2_8_10	PAX8	ES	9.2	8	10	8.4E-04	0.150	0.304	3.6E-01	6.1E-01	2.2E-01
KICH	PCBP2_ES_15_14.1_16.1	PCBP2	ES	15	14.1	16.1	1.7E-04	0.187	-0.278	5.3E-01	1.5E-01	1.6E-02
KICH	PCBP2_ES_15_14.1_16.2	PCBP2	ES	15	14.1	16.2	7.2E-04	0.154	-0.181	5.1E-01	2.2E-01	3.8E-02
KICH	PFDN5_ES_4.1:4.2_2_5	PFDN5	ES	4.1:4.2	2	5	3.0E-04	0.187	-0.035	5.6E-01	8.6E-01	4.3E-01
KICH	PFN2_AA_6.1_5.4_6.2	PFN2	AA	6.1	5.4	6.2	1.6E-05	0.242	0.029	8.8E-01	4.2E-01	5.0E-01
KICH	PICALM_ES_14.1:14.2_13_15	PICALM	ES	14.1:14.2	13	15	7.8E-04	0.150	0.531	2.4E-01	5.3E-01	5.1E-01
KICH	PLEKHA6_AA_7.1_6_7.2	PLEKHA6	AA	7.1	6	7.2	3.9E-04	0.195	-0.199	2.7E-01	9.3E-01	5.8E-01
KICH	PPFIBP1_AD_13.2_13.1_14	PPFIBP1	AD	13.2	13.1	14	7.1E-04	0.184	0.170	3.1E-01	8.6E-01	4.1E-01
KICH	PPFIBP1_ES_20_19_21	PPFIBP1	ES	20	19	21	8.3E-04	0.160	0.109	7.5E-01	9.9E-01	5.5E-01
KICH	PPIL6_ES_7_6_9	PPIL6	ES	7	6	9	7.9E-04	0.150	-0.562	6.5E-01	8.5E-02	4.5E-01
KICH	PQLC1_ES_6:7:9_5_10	PQLC1	ES	6:07:09	5	10	8.9E-05	0.202	-0.063	1.8E-01	7.7E-01	7.6E-01
KICH	PQLC3_ES_6:7_5_8	PQLC3	ES	6:07	5	8	9.0E-04	0.158	0.184	8.0E-01	2.7E-01	6.3E-01
KICH	PRDM2_ES_10_9_12	PRDM2	ES	10	9	12	1.7E-04	0.193	0.041	4.1E-01	2.6E-01	5.6E-01
KICH	RNF111_AA_10.1_9_10.2	RNF111	AA	10.1	9	10.2	9.5E-05	0.210	0.129	9.1E-01	8.5E-01	5.6E-01
KICH	RPL31_RI_2.3_2.2_2.4	RPL31	RI	2.3	2.2	2.4	3.8E-04	0.167	-0.073	5.6E-01	5.6E-01	4.2E-01
KICH	RWDD4_ES_3.2_2_4	RWDD4	ES	3.2	2	4	5.4E-04	0.166	0.409	3.1E-01	5.1E-01	6.0E-01
KICH	S100A1_AA_4.1_3_4.2	S100A1	AA	4.1	3	4.2	3.4E-04	0.170	-0.617	1.8E-01	1.0E+00	1.0E+00
KICH	SCRIB_ES_36_35_37	SCRIB	ES	36	35	37	2.6E-04	0.177	-0.355	9.6E-01	9.3E-01	6.5E-01
KICH	SEC16A_ES_25_24_26	SEC16A	ES	25	24	26	1.1E-04	0.198	-0.442	6.4E-01	7.5E-01	7.4E-01
KICH	SEPT6_AA_13.1_11.1_13.2	SEPT6	AA	13.1	11.1	13.2	2.4E-05	0.237	0.129	3.5E-01	2.4E-01	2.8E-01
KICH	SEPT6_ES_12:13.1_11.1_13.2	SEPT6	ES	12:13.1	11.1	13.2	1.2E-05	0.248	0.336	5.2E-02	3.3E-01	4.1E-01
KICH	SLMAP_ES_25_24_26	SLMAP	ES	25	24	26	6.1E-04	0.156	-0.186	7.3E-01	1.2E-01	1.7E-01
KICH	THYN1_AD_1.2_1.1_1.4	THYN1	AD	1.2	1.1	1.4	1.9E-06	0.290	0.066	4.7E-01	5.3E-01	9.6E-01
KICH	TIA1_ES_5:6_4_7	TIA1	ES	5:06	4	7	1.2E-05	0.253	-0.118	6.4E-01	1.9E-01	5.6E-01
KICH	TMEM106C_AD_1.2_1.1_2.1	TMEM106C	AD	1.2	1.1	2.1	9.8E-04	0.144	0.764	1.0E-01	9.5E-02	1.8E-01
KICH	TMEM139_RI_2.2:2.3_2.1_2.4	TMEM139	RI	2.2:2.3	2.1	2.4	5.8E-04	0.162	-0.047	3.2E-01	1.5E-01	1.5E-02
KICH	URGCP_ES_4.3_3_4.6	URGCP	ES	4.3	3	4.6	6.9E-04	0.167	0.024	2.2E-01	5.7E-01	1.0E+00
KICH	VEZT_ES_5_4_6.1	VEZT	ES	5	4	6.1	7.2E-04	0.161	0.224	2.9E-01	1.5E-01	3.0E-01
KICH	ZBTB25_ES_7.1:7.2_6_8	ZBTB25	ES	7.1:7.2	6	8	1.6E-04	0.188	-0.386	7.1E-02	2.3E-02	3.7E-02
KICH	ZSWIM7_RI_7.6_7.5_7.7	ZSWIM7	RI	7.6	7.5	7.7	1.9E-04	0.184	-0.141	6.7E-01	9.1E-01	4.8E-01
KIRC	AAMDC_ES_5_4_6	AAMDC	ES	5	4	6	7.2E-17	0.163	-0.348	4.6E-05	3.9E-03	2.2E-02
KIRC	AASS_ES_20_19_21	AASS	ES	20	19	21	1.4E-17	0.185	0.338	1.8E-02	1.9E-02	4.5E-02
KIRC	ABAT_AA_19.1_18_19.2	ABAT	AA	19.1	18	19.2	1.7E-10	0.126	-0.285	1.6E-04	9.6E-06	1.5E-06
KIRC	ABCA2_AA_4.1:4.2:4.3:4.4:4.5_3_4.6	ABCA2	AA	4.1:4.2:4.3:4.4:4.[illegible]	3	4.6	2.2E-10	0.113	-0.012	4.1E-04	6.8E-04	3.0E-02

cancer type	id	Gene Symbol	splice_type	Exon	From.Exon	To.Exon	anova.p	adj.r2	r	p.50	p.25	p.10
KIRC	ABCA2_RI_4.2:4.3:4.4:4.5_4.1_4.6	ABCA2	RI	4.2:4.3:4.4:4.5	4.1	4.6	4.2E-14	0.145	0.129	5.7E-05	2.0E-04	4.0E-03
KIRC	ABCC3_ES_5_2.2_6	ABCC3	ES	5	2.2	6	1.6E-13	0.134	0.063	1.2E-03	3.3E-07	7.7E-05
KIRC	ABCC3_RI_16.2_16.1_16.3	ABCC3	RI	16.2	16.1	16.3	5.1E-20	0.191	-0.304	3.6E-02	4.3E-02	5.0E-01
KIRC	ABCC3_RI_27.2_27.1_27.3	ABCC3	RI	27.2	27.1	27.3	3.1E-35	0.314	0.092	1.1E-06	4.6E-05	2.1E-03
KIRC	ABCC5_RI_7.2:7.3_7.1_7.4	ABCC5	RI	7.2:7.3	7.1	7.4	1.6E-11	0.113	0.339	3.3E-02	1.8E-01	4.9E-01
KIRC	ABCC5_RI_7.5_7.4_7.6	ABCC5	RI	7.5	7.4	7.6	2.1E-11	0.113	0.398	1.8E-04	3.3E-02	1.6E-02
KIRC	ABCC6_ES_3_2.1_4	ABCC6	ES	3	2.1	4	5.2E-12	0.127	-0.032	2.8E-03	6.9E-03	1.9E-01
KIRC	ABCD4_AA_8.1_7_8.2	ABCD4	AA	8.1	7	8.2	5.3E-36	0.319	0.233	2.4E-06	1.0E-04	2.9E-03
KIRC	ABCD4_ES_8.1:8.2_7_9	ABCD4	ES	8.1:8.2	7	9	7.0E-10	0.106	0.099	4.5E-06	5.9E-05	4.2E-03
KIRC	ABCE1_ES_14_13_15	ABCE1	ES	14	13	15	1.5E-34	0.308	-0.522	1.5E-06	1.7E-05	1.8E-03
KIRC	ABHD3_AD_5.2_5.1_6	ABHD3	AD	5.2	5.1	6	2.1E-32	0.293	0.056	4.6E-04	4.8E-06	1.5E-04
KIRC	ABLIM1_ES_16_15_17	ABLIM1	ES	16	15	17	1.6E-11	0.118	0.125	6.5E-02	1.0E-01	5.9E-01
KIRC	ACAA1_AD_9.2:9.3_9.1_10	ACAA1	AD	9.2:9.3	9.1	10	2.0E-19	0.186	-0.059	2.1E-04	3.6E-06	1.1E-03
KIRC	ACAA1_RI_9.2_9.1_9.3	ACAA1	RI	9.2	9.1	9.3	4.8E-35	0.312	0.020	6.5E-06	3.8E-05	6.0E-03
KIRC	ACAD10_ES_6_5_7	ACAD10	ES	6	5	7	3.2E-11	0.118	-0.105	6.3E-01	4.7E-01	9.3E-01
KIRC	ACAD9_AD_1.2_1.1_2.1	ACAD9	AD	1.2	1.1	2.1	4.1E-11	0.110	-0.066	2.3E-01	2.1E-01	5.0E-02
KIRC	ACLY_ES_14_13_15	ACLY	ES	14	13	15	5.5E-14	0.137	-0.134	8.9E-04	2.0E-04	1.6E-03
KIRC	ACOT8_AA_6.1_5_6.2	ACOT8	AA	6.1	5	6.2	1.7E-11	0.113	-0.044	5.2E-03	3.3E-02	7.3E-02
KIRC	ACSM2A_ES_2.1:2.2_1_3.2	ACSM2A	ES	2.1:2.2	1	3.2	2.1E-11	0.115	-0.449	5.8E-02	5.4E-02	3.9E-05
KIRC	ACSM2B_ES_2.1:2.2_1_3	ACSM2B	ES	2.1:2.2	1	3	9.0E-12	0.117	-0.785	4.0E-02	5.4E-03	3.3E-02
KIRC	ACTG1_RI_1.3:1.4_1.2_1.5	ACTG1	RI	1.3:1.4	1.2	1.5	8.4E-19	0.184	0.042	6.6E-02	5.8E-03	8.3E-03
KIRC	ACTR10_ES_3_2_4	ACTR10	ES	3	2	4	5.4E-40	0.347	-0.471	3.5E-06	2.2E-05	2.7E-04
KIRC	ADAM15_AD_22.2_22.1_23	ADAM15	AD	22.2	22.1	23	4.2E-13	0.129	-0.054	2.9E-03	1.8E-02	4.1E-03
KIRC	ADAMTS10_AA_7.1_6_7.2	ADAMTS10	AA	7.1	6	7.2	7.0E-12	0.131	0.409	6.9E-05	7.1E-04	1.1E-01
KIRC	ADAMTS4_RI_2.2_2.1_2.3	ADAMTS4	RI	2.2	2.1	2.3	1.1E-15	0.158	0.055	4.0E-04	2.4E-05	5.6E-03
KIRC	ADCK1_ES_4_3_5	ADCK1	ES	4	3	5	2.2E-14	0.141	-0.037	5.9E-05	7.3E-04	1.1E-01
KIRC	ADCK5_ES_5_4_6.1	ADCK5	ES	5	4	6.1	7.6E-10	0.126	0.288	2.3E-02	3.9E-05	1.6E-04
KIRC	ADCK5_RI_6.2_6.1_6.3	ADCK5	RI	6.2	6.1	6.3	3.2E-16	0.187	0.419	1.5E-06	1.5E-04	5.0E-03
KIRC	ADCK5_RI_9.2_9.1_9.3	ADCK5	RI	9.2	9.1	9.3	3.5E-15	0.166	0.526	9.2E-04	8.4E-03	3.1E-01
KIRC	ADCY4_AA_8.1_7_8.2	ADCY4	AA	8.1	7	8.2	7.6E-14	0.161	0.182	1.7E-03	7.2E-04	5.9E-04
KIRC	ADCY4_RI_18.6_18.5_18.7	ADCY4	RI	18.6	18.5	18.7	2.0E-17	0.177	0.085	9.1E-06	2.0E-06	7.9E-06
KIRC	ADHFE1_AA_11.1_10_11.2	ADHFE1	AA	11.1	10	11.2	3.6E-16	0.160	0.152	1.9E-05	6.5E-06	8.3E-03
KIRC	ADM_RI_1.4_1.3_1.5	ADM	RI	1.4	1.3	1.5	1.6E-27	0.255	0.228	2.9E-05	3.9E-04	2.7E-03
KIRC	ADM_RI_1.6:1.7_1.5_1.8	ADM	RI	1.6:1.7	1.5	1.8	2.4E-28	0.261	0.228	6.5E-04	2.1E-03	1.4E-03
KIRC	ADPGK_AD_1.2:1.3_1.1_2	ADPGK	AD	1.2:1.3	1.1	2	1.1E-10	0.110	0.000	9.3E-01	2.2E-01	6.8E-03
KIRC	ADPRH_AD_1.2:1.3_1.1_2.2	ADPRH	AD	1.2:1.3	1.1	2.2	6.0E-14	0.153	-0.308	3.1E-05	3.1E-04	1.0E-02
KIRC	ADPRH_RI_1.2_1.1_1.3	ADPRH	RI	1.2	1.1	1.3	1.9E-14	0.166	-0.240	4.5E-03	5.1E-04	4.7E-02
KIRC	AFMID_ES_3:5_2_6	AFMID	ES	3:05	2	6	3.7E-09	0.108	-0.004	1.5E-01	1.7E-02	2.5E-01
KIRC	AFMID_ES_7:8:9:10:11.1_6_12	AFMID	ES	7:8:9:10:11.1	6	12	2.7E-10	0.102	0.199	6.9E-02	4.5E-01	8.2E-01
KIRC	AFMID_ES_9:10:11.1_8_12	AFMID	ES	10:11.1	8	12	2.5E-11	0.117	0.243	1.3E-01	1.5E-01	3.6E-01

cancer type	id	Gene Symbol	splice_type	Exon	From.Exon	To.Exon	anova.p	adj.r2	r	p.50	p.25	p.10
KIRC	AFTPH_ES_7:8_6_9	AFTPH	ES	7:08	6	9	1.8E-13	0.152	0.486	2.7E-04	8.3E-04	1.3E-03
KIRC	AGBL3_AA_2.1_1_2.2	AGBL3	AA	2.1	1	2.2	1.6E-16	0.184	0.125	9.4E-03	1.5E-04	2.4E-03
KIRC	AGPAT4_RI_5.2_5.1_5.3	AGPAT4	RI	5.2	5.1	5.3	1.5E-30	0.281	0.197	1.5E-03	1.1E-03	8.1E-03
KIRC	AGTRAP_AA_4.1_3_4.2	AGTRAP	AA	4.1	3	4.2	3.7E-19	0.188	-0.097	1.4E-03	8.8E-04	1.1E-03
KIRC	AHI1_ES_31:32_30.1_33	AHI1	ES	31:32:00	30.1	33	3.4E-22	0.212	0.421	1.3E-02	6.2E-04	3.1E-02
KIRC	AIMP2_ES_2:4_1.2_5	AIMP2	ES	2:04	1.2	5	1.4E-13	0.139	-0.372	4.2E-01	3.5E-01	9.3E-01
KIRC	AIMP2_ES_3_1.1_4	AIMP2	ES	3	1.1	4	5.8E-13	0.166	-0.224	7.6E-01	2.1E-01	4.5E-02
KIRC	AKAP10_ES_3_2_4.1	AKAP10	ES	3	2	4.1	3.3E-26	0.243	-0.553	7.6E-07	9.5E-05	3.3E-03
KIRC	AKAP17A_RI_4.2_4.1_4.3	AKAP17A	RI	4.2	4.1	4.3	1.9E-17	0.169	0.495	3.3E-05	3.8E-06	1.8E-02
KIRC	AKAP8L_ES_10_9_11	AKAP8L	ES	10	9	11	1.5E-22	0.213	0.632	3.4E-04	6.9E-05	9.3E-03
KIRC	AKT1_RI_2.4_2.3_2.5	AKT1	RI	2.4	2.3	2.5	5.0E-22	0.216	0.100	1.8E-03	5.8E-04	5.9E-04
KIRC	ALDH3A2_ES_4_3_5	ALDH3A2	ES	4	3	5	6.9E-30	0.274	-0.167	2.2E-04	1.5E-02	5.7E-02
KIRC	ALG2_ES_2_1_3	ALG2	ES	2	1	3	2.9E-14	0.140	-0.371	3.4E-04	1.2E-05	6.9E-04
KIRC	ALKBH6_AD_1.2:1.3_1.1_2	ALKBH6	AD	1.2:1.3	1.1	2	2.8E-10	0.102	0.080	3.8E-01	2.0E-01	6.6E-01
KIRC	ALPK1_AA_7.1_5_7.2	ALPK1	AA	7.1	5	7.2	2.2E-14	0.175	0.384	8.9E-03	1.1E-02	2.0E-02
KIRC	ALS2CL_RI_18.2:18.3_18.1_18.4	ALS2CL	RI	18.2:18.3	18.1	18.4	5.5E-11	0.128	0.186	1.7E-05	1.8E-06	1.1E-01
KIRC	ALS2CL_RI_18.5_18.4_18.6	ALS2CL	RI	18.5	18.4	18.6	2.6E-11	0.115	0.306	6.8E-05	4.0E-07	1.5E-03
KIRC	ANAPC1_ES_20_19_21	ANAPC1	ES	20	19	21	8.8E-10	0.112	0.530	1.3E-02	4.6E-03	7.2E-02
KIRC	ANKLE2_RI_1.2_1.1_1.3	ANKLE2	RI	1.2	1.1	1.3	2.5E-30	0.276	0.010	1.2E-05	1.2E-03	1.1E-01
KIRC	ANKRD10_ES_4_3_6.1	ANKRD10	ES	4	3	6.1	6.8E-24	0.224	0.529	7.3E-06	3.6E-06	9.4E-05
KIRC	ANKRD10_ES_5_3_6.1	ANKRD10	ES	5	3	6.1	4.7E-29	0.266	0.595	4.1E-08	4.7E-09	1.1E-06
KIRC	ANKRD49_AD_2.3:2.4:2.5:2.6_2.2_2.8	ANKRD49	AD	2.3:2.4:2.5:2.6	2.2	2.8	3.5E-20	0.195	0.474	6.7E-04	4.4E-04	3.1E-03
KIRC	ANKRD49_RI_2.3:2.4:2.5:2.6:2.7_2.2_2.	ANKRD49	RI	.3:2.4:2.5:2.6:2.	2.2	2.8	6.2E-30	0.278	0.604	4.0E-04	1.9E-03	4.2E-04
KIRC	ANKRD49_RI_2.5:2.6:2.7_2.4_2.8	ANKRD49	RI	2.5:2.6:2.7	2.4	2.8	2.1E-12	0.134	0.394	1.6E-03	1.3E-02	6.2E-02
KIRC	ANKS3_ES_7.1:7.2_6_8	ANKS3	ES	7.1:7.2	6	8	5.5E-17	0.166	0.289	1.2E-01	1.7E-01	9.4E-01
KIRC	ANO8_AA_13.1_12_13.2	ANO8	AA	13.1	12	13.2	2.9E-18	0.192	0.354	3.9E-03	2.9E-03	2.9E-01
KIRC	AP1G1_ES_2:3:4_1_5	AP1G1	ES	2:03:04	1	5	8.2E-16	0.165	-0.215	1.1E-02	2.6E-02	7.6E-02
KIRC	AP1G2_RI_1.2:1.3_1.1_1.4	AP1G2	RI	1.2:1.3	1.1	1.4	2.1E-16	0.161	0.446	1.1E-05	5.7E-03	8.6E-03
KIRC	APBB3_AA_6.5:6.6:6.7:6.8_6.2_6.9	APBB3	AA	6.5:6.6:6.7:6.8	6.2	6.9	2.5E-19	0.226	0.482	1.4E-01	1.8E-03	5.3E-04
KIRC	APBB3_AD_6.3:6.4:6.5:6.6_6.2_6.9	APBB3	AD	6.3:6.4:6.5:6.6	6.2	6.9	2.3E-19	0.195	0.538	1.1E-05	3.1E-06	3.4E-04
KIRC	APBB3_RI_6.3:6.4:6.5:6.6:6.7:6.8_6.2_6.	APBB3	RI	3:6.4:6.5:6.6:6.7:	6.2	6.9	6.8E-19	0.182	0.688	3.3E-07	8.1E-08	3.7E-05
KIRC	APLP2_ES_9_8_10	APLP2	ES	9	8	10	9.6E-11	0.106	0.000	7.9E-04	1.8E-02	1.8E-02
KIRC	APOBEC3B_RI_7.2_7.1_7.3	APOBEC3B	RI	7.2	7.1	7.3	1.3E-13	0.141	-0.279	8.0E-04	4.2E-02	1.2E-01
KIRC	APOD_ES_3_2_4	APOD	ES	3	2	4	8.6E-15	0.147	-0.369	1.3E-01	1.6E-01	7.2E-01
KIRC	APOL2_ES_3_2.2_4	APOL2	ES	3	2.2	4	7.0E-17	0.164	0.257	8.6E-04	2.5E-04	4.0E-03
KIRC	APOL3_AA_4.1:4.2_3.3_4.3	APOL3	AA	4.1:4.2	3.3	4.3	2.3E-12	0.124	-0.139	1.1E-02	1.3E-02	4.1E-02
KIRC	AQP7_ES_5_3.2_6	AQP7	ES	5	3.2	6	1.1E-10	0.119	0.012	1.2E-02	1.1E-02	1.4E-02
KIRC	ARFGAP1_ES_13_12_14.1	ARFGAP1	ES	13	12	14.1	2.0E-37	0.329	0.591	1.5E-05	9.9E-06	3.7E-04
KIRC	ARGLU1_ES_4_3_5	ARGLU1	ES	4	3	5	1.1E-32	0.294	0.803	1.5E-03	4.9E-05	9.7E-03
KIRC	ARHGAP26_AD_21.2_21.1_22	ARHGAP26	AD	21.2	21.1	22	3.4E-15	0.151	-0.107	8.4E-04	2.8E-05	2.7E-03

cancer type	id	Gene Symbol	splice_type	Exon	From.Exon	To.Exon	anova.p	adj.r2	r	p.50	p.25	p.10
KIRC	ARHGAP32_RI_21.4_21.3_21.5	ARHGAP32	RI	21.4	21.3	21.5	4.5E-30	0.276	-0.004	4.4E-06	5.3E-07	3.1E-03
KIRC	ARHGAP44_ES_21_20_22	ARHGAP44	ES	21	20	22	5.3E-11	0.116	-0.381	2.2E-04	9.7E-04	4.7E-03
KIRC	ARHGEF1_ES_15_14_16	ARHGEF1	ES	15	14	16	1.4E-30	0.279	0.731	5.2E-04	5.7E-07	1.9E-03
KIRC	ARHGEF10L_ES_18_17_19	ARHGEF10L	ES	18	17	19	2.1E-11	0.115	-0.030	3.5E-04	6.5E-03	6.9E-01
KIRC	ARHGEF6_ES_8_7_9	ARHGEF6	ES	8	7	9	5.9E-20	0.191	-0.498	1.9E-01	1.0E-02	2.1E-01
KIRC	ARHGEF9_ES_3_2.3_4	ARHGEF9	ES	3	2.3	4	5.0E-22	0.245	-0.080	6.3E-03	9.6E-03	1.5E-01
KIRC	ARID1B_ES_12_11_13	ARID1B	ES	12	11	13	2.1E-12	0.122	-0.083	1.0E-02	4.6E-02	1.6E-01
KIRC	ARID4B_ES_23_22_24	ARID4B	ES	23	22	24	3.7E-15	0.149	-0.054	5.9E-03	2.5E-03	1.8E-01
KIRC	ARIH2_ES_3_2_4.2	ARIH2	ES	3	2	4.2	1.4E-12	0.123	0.111	2.4E-07	7.1E-07	1.9E-04
KIRC	ARIH2_ES_5_4.2_6	ARIH2	ES	5	4.2	6	1.3E-14	0.145	0.345	2.7E-01	1.4E-01	1.1E-01
KIRC	ARL16_ES_3:4_2.3_5	ARL16	ES	3:04	2.3	5	1.1E-11	0.115	0.138	2.1E-05	1.0E-05	7.1E-04
KIRC	ARL16_ES_4_3_5	ARL16	ES	4	3	5	2.9E-24	0.227	0.278	1.2E-04	9.0E-05	4.8E-05
KIRC	ARMC5_RI_5.2_5.1_5.3	ARMC5	RI	5.2	5.1	5.3	8.7E-14	0.135	-0.156	4.6E-01	1.0E-01	2.4E-01
KIRC	ARMC5_RI_5.4_5.3_5.5	ARMC5	RI	5.4	5.3	5.5	5.3E-16	0.166	0.290	1.1E-06	1.3E-03	3.6E-02
KIRC	ARMC6_ES_4_2.2_5	ARMC6	ES	4	2.2	5	2.0E-24	0.252	0.068	3.5E-04	1.3E-02	1.2E-02
KIRC	ARMCX5_AA_2.6:2.7_2.4_2.8	ARMCX5	AA	2.6:2.7	2.4	2.8	1.7E-11	0.127	-0.047	4.4E-03	6.3E-02	3.7E-02
KIRC	ARMCX5_AD_2.5:2.6_2.4_2.8	ARMCX5	AD	2.5:2.6	2.4	2.8	4.7E-11	0.113	-0.096	9.4E-05	3.7E-04	1.7E-01
KIRC	ARMCX5_ES_2.6_2.4_2.8	ARMCX5	ES	2.6	2.4	2.8	4.4E-13	0.138	0.133	7.8E-01	8.6E-01	5.7E-01
KIRC	ARMCX5_RI_2.5_2.4_2.6	ARMCX5	RI	2.5	2.4	2.6	4.3E-27	0.268	-0.216	4.9E-03	3.6E-03	8.7E-02
KIRC	ARMCX5_RI_2.7_2.6_2.8	ARMCX5	RI	2.7	2.6	2.8	3.6E-28	0.297	-0.272	1.1E-02	1.2E-02	1.8E-01
KIRC	ARMCX5_RI_2.9_2.8_2.1	ARMCX5	RI	2.9	2.8	2.1	2.6E-28	0.271	-0.291	5.2E-03	4.0E-03	1.3E-03
KIRC	ARNT_ES_4_3_5	ARNT	ES	4	3	5	5.1E-31	0.284	-0.290	6.1E-09	3.3E-06	2.1E-05
KIRC	ARRB2_ES_2:3_1_4.2	ARRB2	ES	2:03	1	4.2	2.3E-22	0.214	0.076	1.8E-04	5.9E-03	4.3E-04
KIRC	ARRB2_ES_3_2_4.2	ARRB2	ES	3	2	4.2	2.0E-16	0.160	0.114	8.8E-03	5.1E-03	9.6E-04
KIRC	ARRDC1_RI_2.2_2.1_2.3	ARRDC1	RI	2.2	2.1	2.3	4.4E-33	0.297	0.439	3.0E-05	1.7E-05	5.0E-05
KIRC	ARSA_RI_1.3:1.4_1.2_1.5	ARSA	RI	1.3:1.4	1.2	1.5	2.0E-13	0.132	-0.067	1.7E-01	3.5E-02	1.9E-01
KIRC	ASAP1_ES_4_3.2_5	ASAP1	ES	4	3.2	5	1.2E-14	0.153	-0.256	3.7E-05	1.5E-04	2.3E-03
KIRC	ASCC1_ES_14:15_13_16	ASCC1	ES	14:15	13	16	4.4E-15	0.147	-0.389	2.6E-03	3.0E-04	5.6E-03
KIRC	ASCC2_ES_4_3_5	ASCC2	ES	4	3	5	5.5E-12	0.127	0.186	1.2E-04	1.2E-03	3.5E-03
KIRC	ASH1L_ES_4_3_5	ASH1L	ES	4	3	5	6.0E-36	0.318	-0.696	1.3E-05	2.6E-04	5.2E-03
KIRC	ASNS_AA_11.1_10_11.2	ASNS	AA	11.1	10	11.2	1.2E-18	0.180	0.080	7.0E-02	1.1E-01	1.1E-01
KIRC	ASNSD1_ES_2:3.3:4.1:4.2:5_1_6	ASNSD1	ES	2:3.3:4.1:4.2:5	1	6	6.4E-12	0.150	0.292	2.2E-02	6.6E-04	1.6E-01
KIRC	ASPHD1_RI_1.2_1.1_1.3	ASPHD1	RI	1.2	1.1	1.3	3.1E-10	0.101	-0.222	9.0E-02	5.3E-03	5.1E-02
KIRC	ATAD3A_AA_4.1_3_4.2	ATAD3A	AA	4.1	3	4.2	2.3E-25	0.240	0.094	3.3E-02	1.5E-02	7.4E-02
KIRC	ATF3_AA_5.1:5.2_4_5.3	ATF3	AA	5.1:5.2	4	5.3	2.4E-21	0.203	-0.013	2.1E-05	1.8E-06	1.5E-04
KIRC	ATG10_ES_2_1.1_3.2	ATG10	ES	2	1.1	3.2	1.1E-17	0.170	-0.018	7.3E-03	2.5E-02	1.1E-01
KIRC	ATG12_ES_2_1.1_3	ATG12	ES	2	1.1	3	5.3E-20	0.191	-0.187	1.0E-01	7.1E-02	3.6E-02
KIRC	ATG16L2_AD_6.4_6.3_7	ATG16L2	AD	6.4	6.3	7	2.7E-12	0.121	0.718	1.4E-02	1.5E-04	6.0E-03
KIRC	ATG4B_AD_14.2:14.3_14.1_15	ATG4B	AD	14.2:14.3	14.1	15	2.4E-26	0.244	0.495	3.3E-04	1.7E-03	4.8E-03
KIRC	ATL2_ES_3_1_5	ATL2	ES	3	1	5	2.4E-39	0.342	-0.361	1.0E-07	9.1E-05	1.8E-01

cancer type	id	Gene Symbol	splice_type	Exon	From.Exon	To.Exon	anova.p	adj.r2	r	p.50	p.25	p.10
KIRC	ATP11A_ES_29_28_30.3	ATP11A	ES	29	28	30.3	1.5E-09	0.101	0.183	1.3E-01	3.7E-01	9.3E-01
KIRC	ATP2B4_ES_21_20_22	ATP2B4	ES	21	20	22	1.6E-11	0.114	-0.138	8.2E-02	1.4E-01	1.5E-01
KIRC	ATP5A1_ES_3.3:4.2_3.2_6.1	ATP5A1	ES	3.3:4.2	3.2	6.1	5.2E-13	0.156	-0.276	2.5E-02	4.5E-02	1.3E-01
KIRC	ATP5A1_ES_4.2:4.3_3.2_6.1	ATP5A1	ES	4.2:4.3	3.2	6.1	3.0E-17	0.207	-0.379	6.8E-05	1.9E-04	1.0E-02
KIRC	ATP5A1_ES_4.2:5_3.2_6.1	ATP5A1	ES	4.2:5	3.2	6.1	1.4E-09	0.122	-0.199	1.4E-01	1.3E-01	8.0E-02
KIRC	ATP5D_RI_4.2:4.3_4.1_4.4	ATP5D	RI	4.2:4.3	4.1	4.4	1.3E-10	0.105	-0.284	6.6E-02	2.2E-01	3.2E-01
KIRC	ATP5H_ES_5_3_6	ATP5H	ES	5	3	6	8.4E-15	0.166	-0.247	9.3E-03	2.4E-03	9.2E-03
KIRC	ATP6V0B_ES_2.1:2.2_1.1_3.2	ATP6V0B	ES	2.1:2.2	1.1	3.2	8.4E-19	0.182	0.040	5.5E-03	1.8E-03	2.5E-02
KIRC	B3GALNT2_ES_2_1_3	B3GALNT2	ES	2	1	3	4.1E-13	0.135	-0.146	2.2E-01	1.1E-02	3.7E-01
KIRC	B3GAT3_AA_6.1:6.2_5_6.3	B3GAT3	AA	6.1:6.2	5	6.3	2.2E-10	0.102	-0.069	4.8E-03	2.0E-02	3.2E-01
KIRC	B9D1_RI_8.2_8.1_8.3	B9D1	RI	8.2	8.1	8.3	9.7E-15	0.155	-0.227	1.3E-03	6.0E-04	1.9E-02
KIRC	B9D1_RI_8.2:8.3:8.4_8.1_8.5	B9D1	RI	8.2:8.3:8.4	8.1	8.5	2.1E-19	0.186	-0.139	8.7E-04	1.4E-02	7.3E-02
KIRC	BCAT2_ES_3_1_4	BCAT2	ES	3	1	4	1.6E-15	0.151	0.081	9.3E-01	2.9E-01	6.7E-02
KIRC	BCAT2_ES_3:4:5_1_6	BCAT2	ES	3:04:05	1	6	3.0E-10	0.101	0.006	5.6E-01	4.1E-01	7.3E-01
KIRC	BCL7B_ES_2_1_3	BCL7B	ES	2	1	3	1.0E-27	0.255	-0.031	3.5E-05	1.9E-06	1.4E-06
KIRC	BCLAF1_ES_11_10_12	BCLAF1	ES	11	10	12	1.1E-28	0.264	-0.071	2.0E-02	6.8E-03	1.0E-03
KIRC	BCS1L_AD_1.2:1.3:1.4:1.5:1.6:1.7:1.8_1.	BCS1L	AD	1.3:1.4:1.5:1.6:1.7	1.1	2	4.6E-27	0.252	0.279	1.2E-03	1.4E-03	1.4E-03
KIRC	BCS1L_ES_1.2:1.3:1.4:1.5:1.6:1.7:1.8:2_1	BCS1L	ES	.3:1.4:1.5:1.6:1.7:	1.1	3	1.9E-16	0.186	0.199	5.4E-03	6.6E-05	9.4E-04
KIRC	BCS1L_ES_1.4:1.5:1.6:1.7:1.8_1.1_2	BCS1L	ES	1.4:1.5:1.6:1.7:1.8	1.1	2	1.6E-15	0.156	0.132	2.7E-04	1.0E-04	7.2E-04
KIRC	BCS1L_RI_1.2:1.3_1.1_1.4	BCS1L	RI	1.2:1.3	1.1	1.4	7.4E-13	0.132	0.258	3.1E-02	1.8E-01	2.5E-01
KIRC	BICD2_RI_7.2_7.1_7.3	BICD2	RI	7.2	7.1	7.3	1.2E-22	0.214	0.009	1.2E-02	3.1E-02	3.4E-02
KIRC	BIN1_ES_13:16:17_12_18	BIN1	ES	13:16:17	12	18	7.2E-13	0.134	0.335	5.9E-03	2.0E-03	2.9E-02
KIRC	BIN1_ES_16:17_12_18	BIN1	ES	16:17	12	18	1.8E-10	0.114	0.264	2.1E-02	1.1E-01	2.9E-01
KIRC	BIN1_ES_17_12_18	BIN1	ES	17	12	18	1.1E-12	0.125	0.300	2.7E-02	1.4E-02	6.6E-02
KIRC	BIRC5_ES_3_2.1_4	BIRC5	ES	3	2.1	4	9.1E-13	0.138	-0.177	7.3E-01	8.1E-01	9.3E-01
KIRC	BLOC1S6_ES_3:5_1_7.1	BLOC1S6	ES	3:05	1	7.1	9.5E-09	0.101	-0.102	3.0E-02	1.3E-03	7.1E-05
KIRC	BLOC1S6_ES_5_3_7.1	BLOC1S6	ES	5	3	7.1	7.8E-20	0.191	-0.549	6.9E-04	3.7E-05	8.1E-03
KIRC	BLOC1S6_ES_5:7.1_3_7.2	BLOC1S6	ES	05:07.1	3	7.2	2.3E-14	0.146	-0.444	1.9E-02	5.2E-03	4.3E-03
KIRC	BMP1_AA_15.1_14_15.2	BMP1	AA	15.1	14	15.2	6.5E-28	0.258	0.113	2.5E-03	3.1E-02	1.8E-01
KIRC	BMP1_AA_18.1_16_18.2	BMP1	AA	18.1	16	18.2	4.9E-18	0.174	0.331	3.4E-06	5.7E-06	5.6E-05
KIRC	BMP1_AA_5.1_4_5.2	BMP1	AA	5.1	4	5.2	6.9E-29	0.269	0.143	1.2E-02	2.9E-02	1.2E-01
KIRC	BMP1_ES_17.1:17.2_16_18.1	BMP1	ES	17.1:17.2	16	18.1	4.4E-11	0.110	-0.047	4.2E-01	9.8E-01	9.0E-02
KIRC	BMP1_ES_17.1:17.2:18.1_16_18.2	BMP1	ES	17.1:17.2:18.1	16	18.2	4.1E-26	0.244	0.206	3.2E-03	3.1E-04	5.9E-03
KIRC	BOD1L1_ES_2_1_3	BOD1L1	ES	2	1	3	2.0E-17	0.168	-0.578	2.1E-10	6.1E-08	2.0E-02
KIRC	BOLA1_ES_2.2:3.1_2.1_3.2	BOLA1	ES	2.2:3.1	2.1	3.2	9.7E-16	0.160	-0.188	4.1E-03	1.8E-02	2.2E-03
KIRC	BRD9_AA_5.1_4_5.2	BRD9	AA	5.1	4	5.2	2.1E-19	0.187	0.126	4.0E-01	7.4E-01	1.1E-01
KIRC	BSDC1_RI_11.2_11.1_11.3	BSDC1	RI	11.2	11.1	11.3	8.0E-27	0.248	-0.011	1.8E-05	5.1E-05	1.0E-02
KIRC	BTAF1_ES_25:26.1_24_26.2	BTAF1	ES	25:26.1	24	26.2	4.5E-10	0.103	0.237	1.2E-01	2.4E-03	2.8E-02
KIRC	BTBD3_ES_2.1_1_2.3	BTBD3	ES	2.1	1	2.3	1.9E-12	0.129	-0.217	6.2E-03	1.1E-03	1.2E-02
KIRC	BTN2A1_AA_9.1_8_9.2	BTN2A1	AA	9.1	8	9.2	1.8E-20	0.195	0.107	2.3E-09	1.2E-08	6.0E-08

cancer type	id	Gene Symbol	splice_type	Exon	From.Exon	To.Exon	anova.p	adj.r2	r	p.50	p.25	p.10
KIRC	BTN2A1_ES_4_3_5	BTN2A1	ES	4	3	5	1.1E-13	0.135	0.201	2.4E-03	1.7E-05	4.0E-02
KIRC	BTN2A2_AA_8.1_7_8.2	BTN2A2	AA	8.1	7	8.2	2.6E-24	0.229	0.203	1.1E-06	1.2E-07	2.7E-07
KIRC	BTN2A2_AD_1.2:1.3_1.1_2	BTN2A2	AD	1.2:1.3	1.1	2	7.4E-10	0.112	-0.016	2.7E-01	7.1E-02	6.0E-02
KIRC	BTN3A1_AA_10.1_9_10.2	BTN3A1	AA	10.1	9	10.2	3.7E-20	0.195	0.034	2.8E-06	2.2E-04	4.5E-04
KIRC	BTN3A1_AA_10.1:10.2_9_10.3	BTN3A1	AA	10.1:10.2	9	10.3	2.0E-24	0.229	0.104	4.7E-07	4.2E-03	1.1E-03
KIRC	BTN3A2_AA_4.1_3.2_4.2	BTN3A2	AA	4.1	3.2	4.2	1.5E-37	0.331	0.022	2.5E-06	5.8E-06	2.0E-05
KIRC	BTN3A2_ES_3.1:3.2:4.1_2.2_4.2	BTN3A2	ES	3.1:3.2:4.1	2.2	4.2	9.4E-35	0.312	0.126	4.2E-05	1.5E-05	7.5E-05
KIRC	BTNL9_RI_6.2:6.3:6.4_6.1_6.5	BTNL9	RI	6.2:6.3:6.4	6.1	6.5	5.4E-17	0.173	0.129	1.1E-03	1.0E-03	1.1E-01
KIRC	C11orf49_RI_14.2:14.3_14.1_14.4	C11orf49	RI	14.2:14.3	14.1	14.4	5.4E-14	0.137	-0.102	2.2E-02	2.3E-02	1.1E-01
KIRC	C11orf54_ES_2:3.1_1_3.2	C11orf54	ES	02:03.1	1	3.2	5.1E-19	0.200	-0.207	1.7E-03	1.6E-04	1.3E-03
KIRC	C11orf57_RI_4.2_4.1_4.3	C11orf57	RI	4.2	4.1	4.3	2.6E-30	0.276	-0.317	2.3E-03	3.1E-06	3.7E-04
KIRC	C11orf73_RI_5.2_5.1_5.3	C11orf73	RI	5.2	5.1	5.3	3.0E-25	0.235	-0.047	1.1E-02	2.4E-02	6.4E-02
KIRC	C12orf43_ES_2_1_3.1	C12orf43	ES	2	1	3.1	2.7E-23	0.219	-0.344	1.2E-06	4.5E-06	5.6E-04
KIRC	C12orf76_AA_10.1_8_10.2	C12orf76	AA	10.1	8	10.2	8.3E-24	0.234	0.275	8.7E-03	1.7E-03	1.7E-03
KIRC	C14orf105_AA_3.1_2.1_3.2	C14orf105	AA	3.1	2.1	3.2	7.4E-13	0.150	0.115	4.4E-03	1.7E-03	2.4E-02
KIRC	C14orf105_AA_3.1_2.2_3.2	C14orf105	AA	3.1	2.2	3.2	2.1E-15	0.154	-0.083	3.1E-05	1.1E-03	3.3E-03
KIRC	C14orf105_ES_2.2:3.1_2.1_3.2	C14orf105	ES	2.2:3.1	2.1	3.2	1.1E-18	0.182	0.060	6.4E-02	6.4E-02	5.8E-02
KIRC	C14orf159_ES_4:5.2:6_2_7	C14orf159	ES	05:02.2	2	7	1.1E-16	0.217	-0.184	2.7E-04	4.1E-03	6.3E-02
KIRC	C14orf159_ES_5.2:6_4_7	C14orf159	ES	5.2:6	4	7	2.7E-23	0.246	-0.186	1.1E-05	1.9E-03	2.8E-02
KIRC	C14orf159_ES_6_5.2_7	C14orf159	ES	6	5.2	7	3.0E-24	0.230	-0.131	2.2E-06	9.7E-06	1.4E-04
KIRC	C14orf79_AD_2.3:2.4_2.2_3.2	C14orf79	AD	2.3:2.4	2.2	3.2	1.6E-14	0.142	0.078	2.3E-04	4.0E-03	8.2E-03
KIRC	C16orf58_RI_8.3_8.2_8.4	C16orf58	RI	8.3	8.2	8.4	1.0E-38	0.338	0.222	5.2E-04	1.0E-04	2.6E-05
KIRC	C16orf93_AA_2.1_1_2.2	C16orf93	AA	2.1	1	2.2	2.9E-09	0.111	0.216	5.6E-03	1.4E-02	1.7E-03
KIRC	C19orf25_AA_2.1_1.5_2.2	C19orf25	AA	2.1	1.5	2.2	6.9E-11	0.107	-0.063	1.8E-06	4.7E-04	4.0E-03
KIRC	C19orf48_RI_5.2_5.1_5.3	C19orf48	RI	5.2	5.1	5.3	3.6E-21	0.202	0.010	6.1E-05	1.1E-05	7.8E-05
KIRC	C1orf131_RI_6.2_6.1_6.3	C1orf131	RI	6.2	6.1	6.3	3.9E-32	0.290	0.351	4.1E-07	1.3E-06	3.3E-02
KIRC	C1orf159_AA_5.1:5.2:5.3:5.4_4.4_5.5	C1orf159	AA	5.1:5.2:5.3:5.4	4.4	5.5	7.7E-21	0.208	0.466	2.8E-05	4.5E-03	8.7E-03
KIRC	C1orf159_AD_11.2:11.3_11.1_12	C1orf159	AD	11.2:11.3	11.1	12	5.2E-14	0.141	0.344	9.2E-03	2.7E-03	1.0E-01
KIRC	C1orf85_ES_5.1:5.2_4_6.1	C1orf85	ES	5.1:5.2	4	6.1	2.7E-12	0.125	0.040	6.1E-02	1.2E-01	2.2E-01
KIRC	C1orf86_ES_6_5_7.1	C1orf86	ES	6	5	7.1	3.9E-20	0.192	-0.438	1.7E-06	3.0E-06	3.6E-06
KIRC	C1RL_AA_5.1_4.2_5.2	C1RL	AA	5.1	4.2	5.2	4.8E-15	0.147	0.115	7.8E-05	2.6E-07	8.9E-03
KIRC	C5orf22_ES_8_7.1_9	C5orf22	ES	8	7.1	9	2.6E-20	0.196	-0.473	8.4E-07	2.3E-06	3.1E-03
KIRC	C5orf45_RI_7.2_7.1_7.3	C5orf45	RI	7.2	7.1	7.3	6.3E-26	0.241	0.111	3.4E-04	5.0E-05	2.0E-03
KIRC	C6orf1_AD_1.2_1.1_2.1	C6orf1	AD	1.2	1.1	2.1	1.3E-10	0.106	0.085	4.1E-03	5.3E-02	1.6E-01
KIRC	C7orf43_AA_9.1_8_9.2	C7orf43	AA	9.1	8	9.2	3.9E-16	0.158	0.445	4.3E-03	1.2E-03	5.7E-04
KIRC	C7orf49_AD_1.2_1.1_2.1	C7orf49	AD	1.2	1.1	2.1	6.9E-15	0.145	0.210	3.3E-03	3.9E-04	1.0E-01
KIRC	C7orf49_AD_2.3:2.4_2.2_3	C7orf49	AD	2.3:2.4	2.2	3	6.2E-11	0.110	-0.005	9.8E-01	7.9E-01	5.2E-01
KIRC	C8orf58_ES_6.2_5_7	C8orf58	ES	6.2	5	7	6.4E-11	0.109	-0.105	1.5E-03	2.0E-05	2.8E-03
KIRC	C9orf89_AA_3.3:3.4_3.1_3.5	C9orf89	AA	3.3:3.4	3.1	3.5	2.0E-16	0.172	-0.049	3.9E-01	3.1E-01	1.6E-01
KIRC	C9orf89_AD_3.2:3.3_3.1_3.5	C9orf89	AD	3.2:3.3	3.1	3.5	1.3E-16	0.176	-0.185	9.8E-02	5.6E-01	5.6E-01

cancer type	id	Gene Symbol	splice_type	Exon	From.Exon	To.Exon	anova.p	adj.r2	r	p.50	p.25	p.10
KIRC	C9orf89_RI_3.2_3.1_3.3	C9orf89	RI	3.2	3.1	3.3	1.6E-22	0.213	-0.172	2.4E-04	3.1E-03	2.2E-01
KIRC	C9orf89_RI_3.2:3.3:3.4_3.1_3.5	C9orf89	RI	3.2:3.3:3.4	3.1	3.5	1.8E-28	0.291	-0.131	3.3E-02	1.9E-02	4.5E-02
KIRC	C9orf89_RI_3.4_3.3_3.5	C9orf89	RI	3.4	3.3	3.5	6.0E-24	0.225	-0.041	2.4E-05	1.4E-03	3.5E-02
KIRC	CALCOCO1_RI_14.3_14.2_14.4	CALCOCO1	RI	14.3	14.2	14.4	2.2E-30	0.277	0.320	3.0E-05	1.7E-08	1.3E-03
KIRC	CALCOCO2_ES_3_2_6	CALCOCO2	ES	3	2	6	3.7E-37	0.327	-0.420	3.7E-08	2.1E-06	1.3E-02
KIRC	CALD1_AD_8.3_8.2_9	CALD1	AD	8.3	8.2	9	4.7E-13	0.128	0.024	1.9E-01	3.1E-02	2.1E-02
KIRC	CALD1_ES_8.3:9_8.2_10	CALD1	ES	8.3:9	8.2	10	2.5E-13	0.130	0.045	3.9E-01	4.4E-01	9.9E-01
KIRC	CAMTA2_AA_7.1:7.2_6_7.3	CAMTA2	AA	7.1:7.2	6	7.3	4.6E-11	0.113	0.276	1.4E-02	1.0E-02	5.1E-02
KIRC	CAPN10_ES_8_7_9	CAPN10	ES	8	7	9	1.5E-21	0.207	0.439	2.8E-05	6.6E-05	1.9E-03
KIRC	CARD8_AA_7.1_5_7.2	CARD8	AA	7.1	5	7.2	7.0E-34	0.305	0.304	3.9E-06	4.3E-05	1.3E-03
KIRC	CARD8_ES_6_5_7.2	CARD8	ES	6	5	7.2	2.6E-22	0.216	0.329	8.1E-06	8.6E-06	1.8E-04
KIRC	CARD8_ES_6:7.2_5_8	CARD8	ES	06:07.2	5	8	2.2E-15	0.164	0.327	6.8E-06	1.1E-05	5.9E-06
KIRC	CARD8_ES_7.1:7.2_5_8	CARD8	ES	7.1:7.2	5	8	1.6E-19	0.189	0.300	1.4E-06	1.1E-08	1.2E-03
KIRC	CARKD_AA_9.1_8_9.2	CARKD	AA	9.1	8	9.2	5.5E-25	0.234	0.288	1.7E-07	6.3E-07	3.0E-05
KIRC	CARKD_RI_7.2_7.1_7.3	CARKD	RI	7.2	7.1	7.3	9.8E-23	0.216	0.103	8.2E-05	3.9E-05	2.8E-03
KIRC	CARKD_RI_7.4_7.3_7.5	CARKD	RI	7.4	7.3	7.5	2.3E-32	0.292	0.254	1.1E-07	4.8E-07	4.9E-03
KIRC	CARM1_RI_16.2_16.1_16.3	CARM1	RI	16.2	16.1	16.3	1.8E-21	0.209	0.069	3.4E-03	7.1E-03	3.4E-01
KIRC	CASP1_AA_3.1:3.2:3.3_2_3.4	CASP1	AA	3.1:3.2:3.3	2	3.4	6.1E-10	0.100	-0.035	5.9E-01	2.3E-01	9.6E-01
KIRC	CATSPERG_ES_5_4_6.1	CATSPERG	ES	5	4	6.1	2.6E-16	0.188	-0.268	7.6E-05	5.5E-04	4.3E-04
KIRC	CBR4_ES_2.1:2.2_1_3	CBR4	ES	2.1:2.2	1	3	1.2E-31	0.290	-0.027	4.9E-04	1.8E-02	6.8E-02
KIRC	CC2D1B_RI_25.2_25.1_25.3	CC2D1B	RI	25.2	25.1	25.3	2.1E-15	0.150	0.178	7.6E-04	2.4E-06	2.4E-03
KIRC	CCDC107_RI_3.4_3.3_3.5	CCDC107	RI	3.4	3.3	3.5	2.6E-11	0.111	-0.239	8.5E-01	9.6E-01	4.2E-01
KIRC	CCDC14_RI_2.3_2.2_2.4	CCDC14	RI	2.3	2.2	2.4	3.7E-22	0.216	0.679	3.9E-06	1.0E-04	9.1E-03
KIRC	CCDC14_RI_2.5_2.4_2.6	CCDC14	RI	2.5	2.4	2.6	1.2E-28	0.268	0.703	9.8E-05	1.9E-05	6.6E-03
KIRC	CCDC14_RI_2.7_2.6_2.8	CCDC14	RI	2.7	2.6	2.8	2.0E-21	0.220	0.691	9.2E-05	9.6E-05	8.5E-03
KIRC	CCDC24_AA_4.1_3.2_4.2	CCDC24	AA	4.1	3.2	4.2	5.1E-10	0.112	0.075	2.2E-02	5.1E-03	2.1E-01
KIRC	CCDC24_RI_6.3_6.2_6.4	CCDC24	RI	6.3	6.2	6.4	1.7E-19	0.186	-0.038	1.8E-02	8.7E-03	3.1E-01
KIRC	CCDC28B_RI_6.2_6.1_6.3	CCDC28B	RI	6.2	6.1	6.3	1.5E-18	0.180	-0.219	3.2E-01	8.8E-01	9.5E-01
KIRC	CCDC53_ME_4|5_2_6	CCDC53	ME	4|5	2	6	1.0E-24	0.231	-0.006	4.2E-03	1.7E-02	4.2E-01
KIRC	CCDC74B_RI_3.2_3.1_3.3	CCDC74B	RI	3.2	3.1	3.3	1.3E-17	0.197	0.073	7.5E-04	1.2E-04	1.2E-03
KIRC	CCDC74B_RI_3.2:3.3:3.4_3.1_3.5	CCDC74B	RI	3.2:3.3:3.4	3.1	3.5	7.8E-12	0.126	-0.085	1.3E-04	1.7E-04	3.0E-02
KIRC	CCDC84_AD_6.2_6.1_7	CCDC84	AD	6.2	6.1	7	4.3E-13	0.131	0.475	1.9E-01	7.5E-01	4.7E-01
KIRC	CCDC84_ES_4_3_5	CCDC84	ES	4	3	5	2.7E-15	0.151	0.439	2.6E-01	9.1E-01	8.6E-01
KIRC	CCDC92_AA_2.2:2.3_1_2.4	CCDC92	AA	2.2:2.3	1	2.4	2.1E-10	0.105	0.233	1.1E-02	1.1E-02	1.1E-01
KIRC	CCNB1IP1_ES_6_5_8	CCNB1IP1	ES	6	5	8	7.5E-26	0.240	-0.228	2.5E-05	2.3E-05	5.3E-04
KIRC	CCND3_ES_3.2:4_3.1_5	CCND3	ES	3.2:4	3.1	5	1.4E-08	0.100	-0.012	3.9E-02	9.5E-02	1.9E-01
KIRC	CCNDBP1_AA_10.1:10.2_9_10.3	CCNDBP1	AA	10.1:10.2	9	10.3	3.4E-29	0.267	-0.267	2.7E-04	3.1E-04	4.0E-04
KIRC	CCNDBP1_RI_10.2_10.1_10.3	CCNDBP1	RI	10.2	10.1	10.3	1.1E-29	0.272	-0.454	3.7E-06	3.5E-05	9.2E-03
KIRC	CCNJL_ES_6_5_8	CCNJL	ES	6	5	8	1.8E-15	0.171	-0.448	1.4E-02	8.6E-03	2.9E-02
KIRC	CCNL1_ES_7:8_6_9	CCNL1	ES	7:08	6	9	7.2E-16	0.154	0.433	3.3E-06	7.1E-06	1.9E-05

cancer type	id	Gene Symbol	splice_type	Exon	From.Exon	To.Exon	anova.p	adj.r2	r	p.50	p.25	p.10
KIRC	CCNL2_ES_6.1:7.1_5_7.2	CCNL2	ES	6.1:7.1	5	7.2	5.8E-13	0.129	0.458	3.9E-02	5.7E-03	1.9E-02
KIRC	CCNT2_AD_7.2_7.1_8	CCNT2	AD	7.2	7.1	8	1.4E-12	0.125	0.318	9.8E-05	5.1E-02	5.6E-02
KIRC	CCNT2_ES_6.1:6.2_5_7.1	CCNT2	ES	6.1:6.2	5	7.1	9.0E-15	0.155	0.333	3.0E-03	7.0E-03	3.7E-02
KIRC	CCNT2_ES_6.1:6.2:6.3_5_7.1	CCNT2	ES	6.1:6.2:6.3	5	7.1	2.5E-16	0.172	0.381	3.0E-02	1.3E-01	1.2E-01
KIRC	CCPG1_RI_9.4_9.3_9.5	CCPG1	RI	9.4	9.3	9.5	1.6E-18	0.178	-0.333	3.3E-06	6.0E-04	9.0E-04
KIRC	CCT3_ES_3_1_4	CCT3	ES	3	1	4	1.0E-29	0.271	-0.013	7.5E-07	1.5E-04	2.1E-02
KIRC	CCT4_ES_2_1_3	CCT4	ES	2	1	3	4.1E-17	0.165	-0.353	1.3E-06	1.6E-05	2.1E-03
KIRC	CD72_RI_5.2_5.1_5.3	CD72	RI	5.2	5.1	5.3	5.6E-17	0.171	0.014	6.1E-05	7.8E-06	9.2E-05
KIRC	CD74_AA_10.1_4_10.2	CD74	AA	10.1	4	10.2	1.5E-19	0.187	0.111	5.4E-03	3.1E-01	2.8E-01
KIRC	CD74_ES_6:7.1:8:9_4_10.2	CD74	ES	6:7.1:8:9	4	10.2	2.9E-26	0.244	-0.095	2.2E-01	5.8E-01	2.7E-01
KIRC	CDC37_RI_4.2_4.1_4.3	CDC37	RI	4.2	4.1	4.3	3.1E-35	0.313	0.435	6.1E-06	4.5E-08	8.4E-06
KIRC	CDC42SE2_RI_5.2_5.1_5.3	CDC42SE2	RI	5.2	5.1	5.3	2.5E-25	0.236	-0.309	3.5E-04	1.7E-03	2.2E-02
KIRC	CDH16_ES_3:4:5.1:5.2:6.1:6.2:7.1:7.2:7.3	CDH16	ES	1:6.2:7.1:7.2:7.3:	2	12.1	1.4E-16	0.170	-0.060	7.6E-01	7.0E-01	1.8E-01
KIRC	CDH16_ES_3:4:5.1:5.2:6.2:7.2:7.3:7.4:8:	CDH16	ES	2:6.2:7.2:7.3:7.4:	2	12.1	4.8E-15	0.159	-0.120	5.1E-03	9.3E-02	2.1E-01
KIRC	CDH16_ES_5.2:6.1:6.2:7.1:7.2_5.1_7.3	CDH16	ES	5.2:6.1:6.2:7.1:7.2	5.1	7.3	5.8E-16	0.166	-0.158	4.8E-03	2.8E-01	2.1E-02
KIRC	CDH16_ES_5.2:6.2:7.2_5.1_7.3	CDH16	ES	5.2:6.2:7.2	5.1	7.3	1.1E-21	0.242	-0.313	4.6E-04	8.1E-05	1.2E-03
KIRC	CDH16_RI_14.2_14.1_14.3	CDH16	RI	14.2	14.1	14.3	4.7E-29	0.267	-0.134	7.0E-04	2.6E-03	2.1E-03
KIRC	CDH16_RI_14.2:14.3_14.1_14.4	CDH16	RI	14.2:14.3	14.1	14.4	3.7E-11	0.113	-0.276	2.4E-05	2.7E-04	9.8E-02
KIRC	CDH16_RI_14.2:14.3:14.4_14.1_14.5	CDH16	RI	14.2:14.3:14.4	14.1	14.5	5.5E-19	0.186	-0.228	9.1E-08	1.0E-06	1.9E-03
KIRC	CDK10_ES_2.2:4_2.1_5	CDK10	ES	2.2:4	2.1	5	1.6E-15	0.155	0.415	1.5E-03	1.5E-02	3.6E-03
KIRC	CDK10_ES_3_2.1_5	CDK10	ES	3	2.1	5	9.4E-23	0.221	0.468	7.9E-04	2.1E-04	5.5E-05
KIRC	CDK10_ES_4_2.1_5	CDK10	ES	4	2.1	5	1.6E-10	0.105	0.323	2.8E-02	3.8E-02	1.0E-01
KIRC	CDK10_RI_10.2_10.1_10.3	CDK10	RI	10.2	10.1	10.3	7.4E-27	0.249	0.496	4.4E-07	1.1E-07	3.5E-03
KIRC	CDK13_AA_13.1_12_13.2	CDK13	AA	13.1	12	13.2	4.7E-12	0.119	0.111	1.2E-01	1.2E-01	6.2E-01
KIRC	CDK18_RI_2.4_2.3_2.5	CDK18	RI	2.4	2.3	2.5	4.9E-36	0.320	0.172	2.8E-05	4.4E-06	6.2E-03
KIRC	CDK2_RI_1.2_1.1_1.3	CDK2	RI	1.2	1.1	1.3	3.3E-39	0.343	0.069	3.4E-06	1.2E-06	8.5E-04
KIRC	CDK5RAP3_RI_2.2:2.3:2.4_2.1_2.5	CDK5RAP3	RI	2.2:2.3:2.4	2.1	2.5	1.0E-23	0.223	0.683	3.2E-04	1.1E-03	6.0E-03
KIRC	CDKL1_AA_9.1_8_9.2	CDKL1	AA	9.1	8	9.2	8.3E-15	0.149	-0.465	9.7E-05	1.3E-04	8.7E-03
KIRC	CELF1_AA_18.1:18.2_17_18.3	CELF1	AA	18.1:18.2	17	18.3	5.2E-25	0.233	0.045	4.0E-06	8.6E-05	1.0E-02
KIRC	CELF2_RI_16.2_16.1_16.3	CELF2	RI	16.2	16.1	16.3	5.2E-15	0.146	-0.215	1.7E-02	2.4E-03	7.7E-02
KIRC	CENPV_AA_3.1_2_3.2	CENPV	AA	3.1	2	3.2	4.5E-11	0.109	-0.559	1.6E-03	8.5E-05	6.8E-03
KIRC	CEP57_RI_10.2_10.1_10.3	CEP57	RI	10.2	10.1	10.3	7.8E-26	0.240	-0.376	9.2E-07	1.4E-07	7.2E-03
KIRC	CEP57L1_ES_11_10.1_12	CEP57L1	ES	11	10.1	12	7.3E-15	0.157	0.083	2.8E-02	3.1E-01	4.0E-01
KIRC	CEP76_ES_7_6_8	CEP76	ES	7	6	8	1.3E-22	0.215	-0.373	1.7E-02	2.1E-02	1.3E-02
KIRC	CEP95_AD_13.2_13.1_14	CEP95	AD	13.2	13.1	14	1.9E-16	0.162	0.632	2.7E-02	1.1E-02	2.4E-02
KIRC	CERCAM_RI_3.2_3.1_3.3	CERCAM	RI	3.2	3.1	3.3	2.4E-37	0.328	-0.244	8.0E-03	1.1E-02	1.3E-01
KIRC	CERS5_ES_4_1_8	CERS5	ES	4	1	8	7.5E-31	0.280	0.243	9.6E-03	2.2E-04	3.9E-03
KIRC	CERS5_ES_6.1:6.2_1_8	CERS5	ES	6.1:6.2	1	8	2.2E-31	0.284	0.249	1.2E-03	9.4E-05	4.8E-04
KIRC	CES2_AD_1.2:1.3_1.1_2	CES2	AD	1.2:1.3	1.1	2	9.8E-16	0.153	-0.433	1.3E-02	3.7E-02	2.4E-01
KIRC	CFLAR_ES_10_8.1_11	CFLAR	ES	10	8.1	11	8.1E-30	0.272	-0.277	7.7E-06	7.7E-08	1.4E-03

cancer type	id	Gene Symbol	splice_type	Exon	From.Exon	To.Exon	anova.p	adj.r2	r	p.50	p.25	p.10
KIRC	CGGBP1_RI_5.3_5.2_5.4	CGGBP1	RI	5.3	5.2	5.4	3.6E-22	0.211	-0.100	3.2E-04	4.7E-04	1.9E-03
KIRC	CHCHD5_RI_2.4_2.3_2.5	CHCHD5	RI	2.4	2.3	2.5	3.2E-25	0.235	-0.302	4.1E-03	5.5E-03	4.4E-03
KIRC	CHCHD7_ES_2.1:2.2_1_4	CHCHD7	ES	2.1:2.2	1	4	1.0E-12	0.125	0.160	3.9E-02	1.8E-02	1.3E-01
KIRC	CHD6_RI_8.2_8.1_8.3	CHD6	RI	8.2	8.1	8.3	6.3E-21	0.207	-0.420	4.1E-08	5.7E-07	5.2E-05
KIRC	CHEK2_ES_2_1_3	CHEK2	ES	2	1	3	5.9E-12	0.146	-0.049	5.7E-01	2.8E-01	3.0E-01
KIRC	CHEK2_ME_6|7.1:7.2_5_9	CHEK2	ME	6|7.1:7.2	5	9	2.2E-21	0.203	0.157	3.7E-01	3.4E-02	6.2E-01
KIRC	CHMP7_ES_4_3_5	CHMP7	ES	4	3	5	1.4E-37	0.330	-0.234	1.4E-07	1.4E-04	1.9E-03
KIRC	CHORDC1_AA_9.2:9.3_8.3_9.4	CHORDC1	AA	9.2:9.3	8.3	9.4	5.1E-21	0.202	0.136	1.3E-04	9.1E-04	1.5E-02
KIRC	CHRD_RI_9.2_9.1_9.3	CHRD	RI	9.2	9.1	9.3	3.8E-22	0.230	0.241	1.3E-03	2.9E-04	1.2E-03
KIRC	CHRD_RI_9.5_9.4_9.6	CHRD	RI	9.5	9.4	9.6	3.6E-23	0.229	0.212	4.6E-04	1.5E-03	9.5E-03
KIRC	CHTF8_AA_4.1:4.2_3_4.3	CHTF8	AA	4.1:4.2	3	4.3	5.0E-14	0.140	-0.320	3.2E-01	3.1E-01	2.3E-02
KIRC	CHTOP_ES_4.1:4.2:4.3:4.4_3.2_5	CHTOP	ES	4.1:4.2:4.3:4.4	3.2	5	1.3E-08	0.102	0.386	2.6E-01	3.0E-01	4.3E-01
KIRC	CHTOP_RI_4.3_4.2_4.4	CHTOP	RI	4.3	4.2	4.4	1.3E-14	0.143	0.225	5.3E-04	3.0E-04	4.7E-04
KIRC	CIDEB_ES_2_1_3	CIDEB	ES	2	1	3	3.4E-10	0.103	-0.148	5.0E-07	7.3E-11	1.0E-07
KIRC	CIRBP_ES_9.5:9.6_9.3_9.8	CIRBP	ES	9.5:9.6	9.3	9.8	8.1E-25	0.232	-0.104	9.2E-07	1.1E-05	3.6E-03
KIRC	CIRBP_RI_9.4_9.3_9.5	CIRBP	RI	9.4	9.3	9.5	1.7E-28	0.261	0.174	3.8E-05	4.7E-08	1.3E-04
KIRC	CIRBP_RI_9.9:9.10_9.8_9.11	CIRBP	RI	9.9:9.10	9.8	9.11	6.5E-11	0.107	-0.023	3.1E-05	3.5E-07	6.5E-04
KIRC	CISD2_ES_3_1_4	CISD2	ES	3	1	4	1.3E-26	0.246	-0.297	1.4E-07	2.5E-06	7.8E-05
KIRC	CISH_ES_2_1_3	CISH	ES	2	1	3	5.8E-16	0.163	0.035	2.9E-02	1.2E-01	7.9E-01
KIRC	CLASRP_AD_15.2_15.1_16	CLASRP	AD	15.2	15.1	16	6.0E-13	0.128	0.641	8.4E-02	3.2E-01	5.7E-02
KIRC	CLCN6_ES_13_12_14	CLCN6	ES	13	12	14	4.4E-11	0.112	-0.018	4.9E-02	7.0E-02	6.5E-01
KIRC	CLEC10A_RI_7.2_7.1_7.3	CLEC10A	RI	7.2	7.1	7.3	9.9E-18	0.203	-0.174	1.8E-02	5.4E-04	4.3E-02
KIRC	CLEC10A_RI_7.4_7.3_7.5	CLEC10A	RI	7.4	7.3	7.5	6.1E-16	0.181	-0.117	1.8E-02	1.7E-02	2.5E-02
KIRC	CLEC1A_ES_2_1_3	CLEC1A	ES	2	1	3	4.9E-18	0.182	-0.453	2.1E-06	7.5E-11	6.4E-04
KIRC	CLEC2D_AD_7.2:7.3:7.4:7.5_7.1_8	CLEC2D	AD	7.2:7.3:7.4:7.5	7.1	8	7.9E-11	0.109	0.219	2.5E-04	5.5E-02	1.9E-01
KIRC	CLINT1_AA_11.1_10_11.2	CLINT1	AA	11.1	10	11.2	9.8E-16	0.153	-0.244	1.3E-02	1.3E-03	3.1E-03
KIRC	CLU_RI_3.2_3.1_3.3	CLU	RI	3.2	3.1	3.3	1.4E-17	0.172	0.026	2.3E-03	5.8E-05	1.2E-05
KIRC	CNOT10_ME_19|20_18_21	CNOT10	ME	19|20	18	21	4.4E-20	0.192	-0.216	1.3E-03	1.1E-01	8.6E-01
KIRC	CNOT6_ES_2_1.3_3	CNOT6	ES	2	1.3	3	5.1E-09	0.102	-0.171	2.9E-04	1.4E-05	1.4E-03
KIRC	COG4_ES_9_8_10	COG4	ES	9	8	10	3.5E-30	0.276	0.202	1.6E-04	9.6E-06	2.8E-03
KIRC	COL18A1_AD_3.2_3.1_4	COL18A1	AD	3.2	3.1	4	2.4E-14	0.148	-0.055	2.6E-04	2.5E-05	9.4E-04
KIRC	COL1A1_ES_20:21:22:24:25:26:27:28:29	COL1A1	ES	[illegible]8:29:30:31:32:3[illegible]	19	41	9.5E-12	0.129	-0.227	4.7E-01	3.2E-01	8.1E-01
KIRC	COL1A1_ES_29:30:31:32:33:43:44:45:46	COL1A1	ES	[illegible]2:33:43:44:45:4[illegible]	28	50	4.8E-12	0.139	-0.065	1.3E-01	3.2E-01	6.4E-01
KIRC	COL6A2_ES_14:15_12_16	COL6A2	ES	14:15	12	16	4.9E-11	0.113	0.007	2.9E-02	2.1E-02	4.4E-01
KIRC	COMMD4_ES_4:5.1_3_5.2	COMMD4	ES	04:05.1	3	5.2	2.4E-15	0.179	0.030	1.7E-03	1.3E-03	1.2E-02
KIRC	COPB1_AD_1.2_1.1_2	COPB1	AD	1.2	1.1	2	4.0E-16	0.159	-0.316	9.3E-06	6.4E-06	3.6E-02
KIRC	COPS7A_ES_3:4:6_2.4_7	COPS7A	ES	3:04:06	2.4	7	8.0E-11	0.110	-0.069	2.3E-01	1.0E-01	2.8E-02
KIRC	COPS7A_ES_5:6_2.4_7	COPS7A	ES	5:06	2.4	7	3.2E-10	0.104	-0.007	3.3E-02	6.8E-03	6.8E-02
KIRC	COPS7B_ES_4.1:4.2:4.3:4.4_3_5	COPS7B	ES	4.1:4.2:4.3:4.4	3	5	3.4E-21	0.202	0.486	1.9E-02	2.9E-03	1.0E-03
KIRC	CORO7_AD_18.3:18.4_18.2_19	CORO7	AD	18.3:18.4	18.2	19	2.1E-17	0.174	0.107	4.6E-03	2.8E-02	1.3E-02

cancer type	id	Gene Symbol	splice_type	Exon	From.Exon	To.Exon	anova.p	adj.r2	r	p.50	p.25	p.10
KIRC	CORO7_ES_8_7.1_9	CORO7	ES	8	7.1	9	2.5E-12	0.122	-0.035	3.0E-03	8.8E-04	8.5E-04
KIRC	COX11_RI_3.2_3.1_3.3	COX11	RI	3.2	3.1	3.3	8.4E-19	0.180	-0.014	7.0E-01	6.2E-01	1.9E-01
KIRC	COX4I1_AA_5.1:5.2:5.3_4.1_5.4	COX4I1	AA	5.1:5.2:5.3	4.1	5.4	8.7E-12	0.116	-0.380	2.5E-02	8.4E-02	6.5E-02
KIRC	COX4I1_ES_2.2:3_1.1_4.1	COX4I1	ES	2.2:3	1.1	4.1	3.9E-16	0.164	-0.173	2.1E-01	1.4E-01	6.1E-01
KIRC	COX4I1_ES_4.2:5.1_4.1_5.4	COX4I1	ES	4.2:5.1	4.1	5.4	1.1E-11	0.115	-0.386	4.2E-01	5.8E-01	7.5E-01
KIRC	COX4I1_RI_5.2:5.3:5.4_5.1_5.5	COX4I1	RI	5.2:5.3:5.4	5.1	5.5	3.1E-11	0.110	-0.429	1.8E-01	6.0E-01	3.4E-01
KIRC	COX7C_RI_2.2:2.3_2.1_2.4	COX7C	RI	2.2:2.3	2.1	2.4	1.3E-13	0.133	-0.261	2.9E-02	6.3E-03	1.1E-01
KIRC	CPEB2_ES_6_5_7	CPEB2	ES	6	5	7	1.2E-12	0.129	0.254	4.4E-05	7.9E-04	2.5E-01
KIRC	CPNE1_ES_1.2:2.1:2.2:3_1.1_5	CPNE1	ES	1.2:2.1:2.2:3	1.1	5	3.7E-13	0.129	-0.504	2.9E-02	3.1E-02	6.1E-02
KIRC	CPNE1_ES_2.1:2.2:3_1.2_5	CPNE1	ES	2.1:2.2:3	1.2	5	4.3E-13	0.128	-0.592	2.7E-01	1.0E-01	2.7E-01
KIRC	CPNE1_ES_8.1:8.2_7_9	CPNE1	ES	8.1:8.2	7	9	3.1E-16	0.164	-0.019	6.5E-02	2.6E-02	3.6E-03
KIRC	CPSF4_ES_4_3_5	CPSF4	ES	4	3	5	4.1E-26	0.243	0.114	6.5E-04	5.8E-03	8.3E-03
KIRC	CRB3_RI_5.2_5.1_5.3	CRB3	RI	5.2	5.1	5.3	1.5E-17	0.170	0.034	8.9E-05	4.3E-04	2.4E-02
KIRC	CRCP_ES_4_3_5	CRCP	ES	4	3	5	2.6E-32	0.291	-0.343	4.3E-06	2.5E-06	1.3E-03
KIRC	CRCP_ES_4:5_3_6	CRCP	ES	4:05	3	6	1.3E-10	0.104	-0.143	3.4E-04	1.7E-04	1.5E-04
KIRC	CREB3L4_RI_6.2_6.1_6.3	CREB3L4	RI	6.2	6.1	6.3	3.2E-33	0.299	0.094	3.1E-05	1.4E-04	5.9E-04
KIRC	CREBZF_RI_2.4_2.3_2.5	CREBZF	RI	2.4	2.3	2.5	5.4E-17	0.165	-0.428	7.4E-04	1.7E-05	4.8E-04
KIRC	CRELD1_ES_10_9_11	CRELD1	ES	10	9	11	1.3E-18	0.179	0.014	2.7E-07	1.6E-09	5.8E-06
KIRC	CRELD1_RI_1.2:1.3_1.1_1.4	CRELD1	RI	1.2:1.3	1.1	1.4	1.3E-10	0.105	-0.238	4.3E-05	4.9E-04	5.0E-03
KIRC	CRELD1_RI_1.3_1.2_1.4	CRELD1	RI	1.3	1.2	1.4	4.5E-13	0.129	-0.169	6.5E-03	5.2E-04	1.1E-02
KIRC	CS_ES_6_5.2_7	CS	ES	6	5.2	7	6.0E-28	0.257	-0.372	2.5E-06	4.1E-05	8.6E-03
KIRC	CSF2RA_ES_13_12_14	CSF2RA	ES	13	12	14	2.4E-15	0.149	-0.574	3.7E-01	6.0E-02	5.8E-01
KIRC	CSGALNACT1_ES_10_9_11	CSGALNACT1	ES	10	9	11	1.8E-25	0.244	-0.365	3.9E-03	1.7E-04	1.9E-04
KIRC	CSNK1D_ES_2:3.2_1_4	CSNK1D	ES	02:03.2	1	4	1.2E-11	0.114	0.052	2.1E-03	1.5E-02	7.4E-03
KIRC	CSTF3_RI_4.2_4.1_4.3	CSTF3	RI	4.2	4.1	4.3	1.6E-26	0.246	0.245	3.9E-06	2.1E-05	1.6E-04
KIRC	CTNND1_ES_21:22.1_20_22.2	CTNND1	ES	21:22.1	20	22.2	3.0E-12	0.121	0.022	1.2E-01	1.0E-02	1.5E-02
KIRC	CTTN_ES_12_10_13	CTTN	ES	12	10	13	1.6E-27	0.255	0.272	1.8E-04	2.3E-04	1.1E-03
KIRC	CWC25_AD_6.2_6.1_7	CWC25	AD	6.2	6.1	7	3.0E-27	0.252	0.118	6.3E-06	2.2E-06	4.2E-04
KIRC	CXorf23_ES_11_10.2_12	CXorf23	ES	11	10.2	12	2.3E-14	0.156	0.062	3.0E-02	3.7E-03	3.4E-03
KIRC	CXorf38_AA_2.1:2.2_1.3_2.3	CXorf38	AA	2.1:2.2	1.3	2.3	1.2E-21	0.206	-0.190	6.5E-02	1.2E-02	3.2E-02
KIRC	CXorf38_RI_1.2_1.1_1.3	CXorf38	RI	1.2	1.1	1.3	1.4E-13	0.167	-0.188	4.6E-01	2.3E-01	1.1E-01
KIRC	CXorf40A_RI_1.2:1.3:1.4:1.5_1.1_1.6	CXorf40A	RI	1.2:1.3:1.4:1.5	1.1	1.6	9.3E-24	0.226	-0.053	1.1E-03	2.0E-04	8.5E-04
KIRC	CXorf40A_RI_1.4:1.5_1.3_1.6	CXorf40A	RI	1.4:1.5	1.3	1.6	2.7E-16	0.169	-0.133	1.1E-04	3.3E-05	7.1E-04
KIRC	CXorf40A_RI_1.5_1.4_1.6	CXorf40A	RI	1.5	1.4	1.6	3.7E-28	0.260	-0.154	1.9E-05	5.8E-04	4.5E-02
KIRC	CYB561A3_RI_6.2_6.1_6.3	CYB561A3	RI	6.2	6.1	6.3	7.6E-38	0.331	-0.136	8.9E-07	2.6E-05	2.1E-02
KIRC	CYB561D2_RI_1.2:1.3_1.1_1.4	CYB561D2	RI	1.2:1.3	1.1	1.4	3.6E-12	0.122	-0.041	2.5E-03	2.3E-03	1.6E-03
KIRC	CYB561D2_RI_1.2:1.3:1.4_1.1_1.5	CYB561D2	RI	1.2:1.3:1.4	1.1	1.5	5.7E-14	0.138	0.009	1.9E-05	2.1E-05	7.3E-03
KIRC	CYB5RL_ES_8_7_9	CYB5RL	ES	8	7	9	4.3E-17	0.166	-0.434	8.9E-03	7.6E-02	5.7E-01
KIRC	CYHR1_RI_1.2:1.3_1.1_1.4	CYHR1	RI	1.2:1.3	1.1	1.4	1.4E-10	0.106	0.074	2.5E-02	6.8E-02	3.2E-01
KIRC	CYHR1_RI_1.3_1.2_1.4	CYHR1	RI	1.3	1.2	1.4	1.7E-25	0.239	0.260	2.4E-07	5.1E-07	2.1E-03

cancer type	id	Gene Symbol	splice_type	Exon	From.Exon	To.Exon	anova.p	adj.r2	r	p.50	p.25	p.10
KIRC	CYP3A5_ES_4:5.1_3_5.2	CYP3A5	ES	04:05.1	3	5.2	1.2E-16	0.181	0.019	5.8E-05	7.1E-04	7.4E-04
KIRC	CYP3A5_RI_10.2_10.1_10.3	CYP3A5	RI	10.2	10.1	10.3	1.6E-17	0.169	-0.200	8.9E-07	6.4E-09	1.4E-04
KIRC	D2HGDH_AA_5.1_4_5.2	D2HGDH	AA	5.1	4	5.2	5.1E-23	0.219	-0.585	1.4E-04	4.2E-04	4.2E-04
KIRC	D2HGDH_ES_10:11.1_9_11.2	D2HGDH	ES	10:11.1	9	11.2	9.5E-19	0.180	0.604	2.3E-04	6.5E-03	2.2E-01
KIRC	D2HGDH_ES_7.1:7.2:7.3_6_8	D2HGDH	ES	7.1:7.2:7.3	6	8	1.5E-18	0.179	0.450	4.1E-04	1.1E-04	1.3E-03
KIRC	D2HGDH_ES_7.1:7.3_6_8	D2HGDH	ES	7.1:7.3	6	8	2.3E-20	0.200	0.551	8.6E-02	2.1E-01	5.7E-02
KIRC	D2HGDH_RI_7.2_7.1_7.3	D2HGDH	RI	7.2	7.1	7.3	1.7E-10	0.109	-0.317	3.0E-02	2.3E-01	4.2E-01
KIRC	DAB2_ES_10_9_11	DAB2	ES	10	9	11	4.1E-21	0.200	-0.485	1.7E-05	5.6E-04	1.5E-02
KIRC	DAGLB_AD_12.3_12.2_13	DAGLB	AD	12.3	12.2	13	9.3E-26	0.239	0.199	2.6E-04	6.7E-05	8.2E-03
KIRC	DALRD3_RI_11.3_11.2_11.4	DALRD3	RI	11.3	11.2	11.4	9.2E-18	0.171	-0.157	5.8E-05	3.3E-06	6.3E-04
KIRC	DAPK2_ES_13.2:13.3:13.4:14:15.1:15.2_	DAPK2	ES	13.3:13.4:14:15.1	13.1	15.3	3.5E-10	0.102	-0.116	1.1E-01	1.6E-01	8.2E-01
KIRC	DCAF8_ES_18_17_19	DCAF8	ES	18	17	19	1.8E-30	0.277	0.259	1.7E-04	2.9E-04	2.5E-02
KIRC	DCLK2_ES_17_16_18	DCLK2	ES	17	16	18	5.5E-13	0.132	-0.134	4.7E-03	6.9E-04	1.2E-02
KIRC	DCTN3_RI_3.6_3.5_3.7	DCTN3	RI	3.6	3.5	3.7	1.0E-12	0.145	-0.302	1.7E-02	1.5E-01	6.1E-01
KIRC	DCTN5_ES_4_2_5	DCTN5	ES	4	2	5	2.0E-33	0.299	-0.485	3.6E-08	2.0E-06	5.5E-04
KIRC	DCTN6_ES_3_2_4	DCTN6	ES	3	2	4	2.0E-25	0.237	-0.636	2.7E-06	6.8E-07	1.3E-04
KIRC	DCUN1D4_AD_9.2:9.3_9.1_10	DCUN1D4	AD	9.2:9.3	9.1	10	3.4E-33	0.299	0.404	1.1E-04	7.7E-06	2.5E-04
KIRC	DCUN1D4_ES_12_11_13	DCUN1D4	ES	12	11	13	6.2E-27	0.253	0.322	2.0E-01	2.2E-02	6.8E-02
KIRC	DCUN1D4_ES_12:13_11_14	DCUN1D4	ES	12:13	11	14	2.3E-15	0.158	0.223	9.3E-03	1.6E-02	2.7E-01
KIRC	DCUN1D4_ES_4_1_5	DCUN1D4	ES	4	1	5	5.9E-15	0.152	0.279	6.1E-02	2.5E-02	2.4E-02
KIRC	DDIT3_RI_2.2:2.3_2.1_2.4	DDIT3	RI	2.2:2.3	2.1	2.4	6.6E-16	0.155	0.176	3.8E-03	1.5E-02	2.1E-01
KIRC	DDIT3_RI_2.2:2.3:2.4_2.1_2.5	DDIT3	RI	2.2:2.3:2.4	2.1	2.5	2.8E-10	0.101	0.217	3.5E-03	1.0E-03	6.6E-02
KIRC	DDX11_AA_12.1_11.1_12.2	DDX11	AA	12.1	11.1	12.2	3.9E-24	0.237	0.256	6.5E-03	2.0E-03	1.4E-04
KIRC	DDX11_RI_29.2_29.1_29.3	DDX11	RI	29.2	29.1	29.3	3.1E-20	0.195	0.440	2.3E-06	1.0E-06	2.3E-03
KIRC	DDX19A_ES_2_1_3	DDX19A	ES	2	1	3	9.7E-35	0.309	-0.388	4.6E-07	1.1E-05	1.4E-05
KIRC	DDX20_RI_5.10_5.9_5.11	DDX20	RI	5.1	5.9	5.11	4.0E-32	0.293	-0.006	4.7E-05	4.9E-06	2.4E-05
KIRC	DDX20_RI_5.2_5.1_5.3	DDX20	RI	5.2	5.1	5.3	2.4E-28	0.272	0.019	1.2E-02	4.7E-02	5.8E-03
KIRC	DDX20_RI_5.4_5.3_5.5	DDX20	RI	5.4	5.3	5.5	5.3E-21	0.212	0.074	8.2E-03	2.5E-05	8.3E-06
KIRC	DDX20_RI_5.6_5.5_5.7	DDX20	RI	5.6	5.5	5.7	2.9E-24	0.229	-0.057	3.0E-05	2.8E-05	1.8E-02
KIRC	DDX20_RI_5.8_5.7_5.9	DDX20	RI	5.8	5.7	5.9	2.0E-31	0.290	-0.052	3.7E-05	1.9E-05	5.0E-03
KIRC	DDX39A_AA_7.1_6_7.2	DDX39A	AA	7.1	6	7.2	2.1E-31	0.284	0.258	7.4E-03	1.4E-03	7.6E-03
KIRC	DDX3X_ES_6.2:7:8:10:11:12:13:14:15:16	DDX3X	ES	10:11:12:13:14:1	6.1	18	3.2E-10	0.124	-0.166	7.0E-03	1.2E-03	2.0E-03
KIRC	DDX41_AA_7.1_6_7.2	DDX41	AA	7.1	6	7.2	7.2E-33	0.296	0.236	3.8E-04	3.2E-04	9.1E-04
KIRC	DDX46_ES_13_12_14	DDX46	ES	13	12	14	1.4E-29	0.273	-0.327	4.7E-05	4.7E-06	3.1E-04
KIRC	DDX5_ES_6.2:7:8.1_4.1_8.2	DDX5	ES	6.2:7:8.1	4.1	8.2	1.4E-20	0.198	-0.067	7.7E-01	3.8E-01	2.1E-01
KIRC	DDX52_ES_3_2_5	DDX52	ES	3	2	5	1.1E-11	0.116	-0.465	1.4E-01	4.6E-01	9.0E-01
KIRC	DDX55_AA_2.1_1_2.2	DDX55	AA	2.1	1	2.2	1.5E-10	0.124	0.378	4.4E-01	7.7E-02	4.3E-02
KIRC	DECR2_AA_7.1_6_7.2	DECR2	AA	7.1	6	7.2	7.7E-16	0.160	-0.151	2.4E-06	9.9E-06	1.6E-03
KIRC	DECR2_ES_3_2_4.1	DECR2	ES	3	2	4.1	1.6E-16	0.176	-0.340	1.1E-04	3.1E-03	1.7E-02
KIRC	DECR2_ES_5.1:5.2:5.3_4.2_6	DECR2	ES	5.1:5.2:5.3	4.2	6	1.8E-21	0.206	0.122	6.1E-02	1.7E-01	1.7E-01

cancer type	id	Gene Symbol	splice_type	Exon	From.Exon	To.Exon	anova.p	adj.r2	r	p.50	p.25	p.10
KIRC	DERL2_RI_4.2_4.1_4.3	DERL2	RI	4.2	4.1	4.3	1.1E-10	0.108	-0.114	3.1E-01	5.2E-03	3.0E-01
KIRC	DHRS1_RI_6.3_6.2_6.4	DHRS1	RI	6.3	6.2	6.4	7.0E-28	0.257	-0.116	1.8E-07	1.0E-07	5.0E-04
KIRC	DHX30_ES_4_3_5	DHX30	ES	4	3	5	1.3E-23	0.222	-0.033	2.7E-02	1.7E-02	5.2E-03
KIRC	DIABLO_AD_2.4_2.3_3	DIABLO	AD	2.4	2.3	3	2.3E-24	0.233	-0.009	7.5E-04	4.9E-05	3.1E-05
KIRC	DIABLO_RI_2.2_2.1_2.3	DIABLO	RI	2.2	2.1	2.3	1.6E-24	0.229	0.064	1.8E-05	2.0E-05	5.5E-04
KIRC	DIAPH1_ES_2_1_3	DIAPH1	ES	2	1	3	3.4E-13	0.141	-0.416	1.4E-03	3.0E-05	2.9E-05
KIRC	DIS3L2_RI_24.2:24.3_24.1_24.4	DIS3L2	RI	24.2:24.3	24.1	24.4	3.8E-15	0.148	0.030	7.5E-05	5.2E-02	1.6E-01
KIRC	DMKN_RI_18.2_18.1_18.3	DMKN	RI	18.2	18.1	18.3	1.8E-15	0.192	-0.070	1.8E-04	1.3E-03	5.2E-02
KIRC	DMKN_RI_18.4_18.3_18.5	DMKN	RI	18.4	18.3	18.5	4.7E-11	0.138	-0.139	3.0E-04	5.4E-07	6.6E-06
KIRC	DMTF1_AA_13.1:13.2_12_13.3	DMTF1	AA	13.1:13.2	12	13.3	1.7E-13	0.135	0.529	1.3E-03	3.4E-03	1.7E-01
KIRC	DMTF1_AA_13.2_12_13.3	DMTF1	AA	13.2	12	13.3	2.8E-15	0.151	0.618	3.5E-04	1.8E-03	4.9E-04
KIRC	DMTF1_AA_17.1_16_17.2	DMTF1	AA	17.1	16	17.2	1.7E-11	0.116	0.487	9.5E-03	6.2E-04	6.3E-02
KIRC	DMTF1_ES_1.2:2:3_1.1_4.2	DMTF1	ES	1.2:2:3	1.1	4.2	1.1E-22	0.218	0.352	1.5E-02	1.6E-03	7.2E-03
KIRC	DNAJA3_ES_2_1_3	DNAJA3	ES	2	1	3	6.9E-30	0.272	-0.277	2.8E-06	1.3E-04	2.3E-02
KIRC	DNAJB2_RI_9.2_9.1_9.3	DNAJB2	RI	9.2	9.1	9.3	2.6E-10	0.101	-0.189	1.1E-03	2.2E-03	3.0E-01
KIRC	DNAJC12_ES_2_1_3	DNAJC12	ES	2	1	3	5.4E-21	0.202	-0.469	6.5E-01	6.6E-01	8.5E-01
KIRC	DNAJC25_ES_2.1:2.2_1_3.2	DNAJC25	ES	2.1:2.2	1	3.2	2.8E-17	0.176	-0.026	3.1E-04	8.2E-04	2.7E-02
KIRC	DNASE1L1_AD_3.3_3.2_4	DNASE1L1	AD	3.3	3.2	4	2.4E-16	0.159	-0.238	1.1E-01	1.1E-01	2.8E-02
KIRC	DNASE1L3_RI_9.2_9.1_9.3	DNASE1L3	RI	9.2	9.1	9.3	1.2E-11	0.115	-0.686	2.9E-05	2.3E-06	6.0E-02
KIRC	DOCK8_AD_20.2_20.1_22	DOCK8	AD	20.2	20.1	22	6.2E-23	0.233	-0.108	3.0E-02	1.0E-01	2.3E-01
KIRC	DOCK8_ES_2_1_3	DOCK8	ES	2	1	3	4.0E-29	0.266	-0.507	5.7E-06	1.2E-08	8.4E-04
KIRC	DOCK8_ES_21_20.1_22	DOCK8	ES	21	20.1	22	1.7E-25	0.255	-0.020	1.9E-02	2.2E-01	3.8E-01
KIRC	DPEP2_AA_11.1_10_11.2	DPEP2	AA	11.1	10	11.2	6.3E-10	0.102	-0.097	1.9E-01	8.9E-03	1.2E-01
KIRC	DPH2_AA_3.1_2.1_3.2	DPH2	AA	3.1	2.1	3.2	7.1E-39	0.352	0.000	7.0E-04	3.5E-04	2.1E-04
KIRC	DPH2_AD_2.2_2.1_3.2	DPH2	AD	2.2	2.1	3.2	1.8E-31	0.299	0.026	1.7E-03	2.4E-04	6.0E-03
KIRC	DPP8_ES_18_17_19	DPP8	ES	18	17	19	3.8E-15	0.157	-0.350	1.6E-02	8.1E-03	6.3E-02
KIRC	DPP8_ES_18:19_17_20	DPP8	ES	18:19	17	20	1.0E-10	0.114	-0.338	2.4E-02	4.9E-03	1.0E-03
KIRC	DPP9_ES_4_3_6	DPP9	ES	4	3	6	1.2E-24	0.232	0.224	4.5E-04	1.4E-04	4.4E-03
KIRC	DPP9_ES_5_3_6	DPP9	ES	5	3	6	1.5E-21	0.206	0.335	2.4E-04	2.7E-03	5.7E-04
KIRC	DRG2_RI_6.3_6.2_6.4	DRG2	RI	6.3	6.2	6.4	9.0E-37	0.324	0.227	5.1E-06	9.6E-06	6.3E-03
KIRC	DTD2_AD_1.2_1.1_2	DTD2	AD	1.2	1.1	2	1.4E-10	0.104	0.371	9.9E-01	2.4E-01	7.6E-02
KIRC	DTX3_AA_1.3:1.4_1.1_1.5	DTX3	AA	1.3:1.4	1.1	1.5	5.7E-11	0.123	0.174	1.9E-07	1.4E-04	2.5E-04
KIRC	DTX3_AD_1.2:1.3_1.1_1.5	DTX3	AD	1.2:1.3	1.1	1.5	1.3E-14	0.143	0.149	8.0E-06	1.1E-04	1.8E-03
KIRC	DTX3_RI_1.2_1.1_1.3	DTX3	RI	1.2	1.1	1.3	1.3E-16	0.162	0.129	2.2E-04	4.2E-04	3.9E-03
KIRC	DTX3_RI_1.2:1.3:1.4_1.1_1.5	DTX3	RI	1.2:1.3:1.4	1.1	1.5	1.7E-19	0.211	0.151	5.3E-07	2.5E-07	1.1E-04
KIRC	DTX3_RI_1.4_1.3_1.5	DTX3	RI	1.4	1.3	1.5	7.1E-19	0.189	0.169	1.6E-04	1.0E-04	1.9E-03
KIRC	DUS4L_AD_5.2_5.1_6	DUS4L	AD	5.2	5.1	6	9.8E-13	0.132	-0.044	2.1E-02	3.4E-04	1.8E-02
KIRC	DUSP28_RI_2.2_2.1_2.3	DUSP28	RI	2.2	2.1	2.3	1.1E-19	0.188	0.225	9.8E-02	5.1E-02	9.0E-01
KIRC	DYM_ES_3:5_2_6	DYM	ES	3:05	2	6	5.8E-15	0.146	-0.478	2.3E-02	2.8E-03	4.4E-05
KIRC	DYNC2LI1_AA_8.1_7_8.2	DYNC2LI1	AA	8.1	7	8.2	3.0E-28	0.259	-0.390	1.4E-06	2.8E-08	1.5E-04

cancer type	id	Gene Symbol	splice_type	Exon	From.Exon	To.Exon	anova.p	adj.r2	r	p.50	p.25	p.10
KIRC	DYNC2LI1_ES_2_1_3	DYNC2LI1	ES	2	1	3	6.2E-22	0.208	-0.310	1.1E-03	7.9E-06	1.1E-02
KIRC	DYNLL1_ES_2:3_1_4.4	DYNLL1	ES	2:03	1	4.4	1.5E-15	0.153	-0.263	5.7E-01	8.9E-01	4.1E-01
KIRC	DZIP3_RI_17.2_17.1_17.3	DZIP3	RI	17.2	17.1	17.3	1.3E-31	0.289	-0.113	4.9E-06	1.8E-04	1.1E-02
KIRC	ECHDC2_AA_6.1_5.1_6.2	ECHDC2	AA	6.1	5.1	6.2	3.4E-15	0.148	0.201	1.6E-05	1.2E-05	1.8E-05
KIRC	ECHDC2_ES_3:5.1:6.1_2.1_6.2	ECHDC2	ES	3:5.1:6.1	2.1	6.2	1.7E-12	0.123	0.168	4.0E-03	2.0E-03	8.1E-02
KIRC	ECHDC2_ES_4_2.1_5.1	ECHDC2	ES	4	2.1	5.1	1.6E-10	0.107	0.242	1.5E-01	6.2E-02	4.1E-01
KIRC	ECHDC2_ES_4:5.1:6.1_2.1_6.2	ECHDC2	ES	4:5.1:6.1	2.1	6.2	1.2E-16	0.162	0.175	4.5E-04	1.3E-03	1.2E-01
KIRC	EDF1_RI_4.2_4.1_4.3	EDF1	RI	4.2	4.1	4.3	4.1E-23	0.218	-0.035	4.4E-06	4.3E-04	2.9E-03
KIRC	EED_RI_12.2_12.1_12.3	EED	RI	12.2	12.1	12.3	2.6E-21	0.203	0.355	1.6E-04	5.9E-04	2.1E-02
KIRC	EEF1A1_ES_1.4:2:3:4:5_1.3_6	EEF1A1	ES	1.4:2:3:4:5	1.3	6	2.5E-19	0.187	-0.355	1.6E-05	3.4E-10	4.1E-08
KIRC	EEF1A1_RI_1.2:1.3_1.1_1.4	EEF1A1	RI	1.2:1.3	1.1	1.4	4.4E-31	0.283	-0.325	2.8E-08	1.5E-08	2.0E-05
KIRC	EEF1D_ES_8.3:9:10.1:10.2:11:12.2:13.1_	EEF1D	ES	10.1:10.2:11:12.2	8.2	13.2	5.0E-15	0.147	0.283	6.3E-01	4.5E-01	9.0E-01
KIRC	EFHC1_RI_5.2_5.1_5.3	EFHC1	RI	5.2	5.1	5.3	9.8E-34	0.302	0.245	1.2E-06	9.7E-06	4.2E-03
KIRC	EIF2A_ES_3_2_4	EIF2A	ES	3	2	4	6.2E-33	0.296	-0.429	5.1E-08	4.8E-07	2.8E-06
KIRC	EIF2A_ES_3:4_2_5.1	EIF2A	ES	3:04	2	5.1	2.2E-10	0.103	-0.269	5.0E-04	1.0E-04	1.4E-02
KIRC	EIF2AK2_AA_2.1_1_2.2	EIF2AK2	AA	2.1	1	2.2	2.5E-11	0.115	-0.340	1.0E-03	5.6E-03	3.7E-02
KIRC	EIF3M_ES_2.1:2.2:3_1_5	EIF3M	ES	2.1:2.2:3	1	5	7.8E-18	0.178	-0.105	7.3E-01	4.6E-01	6.0E-01
KIRC	EIF4A2_ES_11_10_12	EIF4A2	ES	11	10	12	1.1E-10	0.105	-0.258	1.0E-01	5.3E-02	1.2E-01
KIRC	EIF4A2_ES_9.1:9.2_8_10	EIF4A2	ES	9.1:9.2	8	10	5.1E-11	0.114	-0.097	1.1E-02	2.3E-03	1.3E-01
KIRC	EIF4G1_RI_2.3_2.2_2.4	EIF4G1	RI	2.3	2.2	2.4	2.5E-28	0.267	-0.259	7.9E-03	2.3E-02	1.5E-02
KIRC	ELMOD3_AA_2.1:2.2_1_2.3	ELMOD3	AA	2.1:2.2	1	2.3	4.3E-16	0.185	0.460	4.6E-03	2.1E-03	5.1E-02
KIRC	ELMOD3_ES_9_8_10	ELMOD3	ES	9	8	10	2.2E-16	0.160	0.386	8.3E-02	6.1E-02	1.6E-02
KIRC	ELMOD3_RI_11.2_11.1_11.3	ELMOD3	RI	11.2	11.1	11.3	2.7E-12	0.121	0.297	1.3E-01	1.3E-01	2.9E-01
KIRC	ELMOD3_RI_2.2_2.1_2.3	ELMOD3	RI	2.2	2.1	2.3	1.3E-24	0.253	0.474	8.9E-03	2.2E-02	4.8E-01
KIRC	ELMOD3_RI_2.2:2.3:2.4:2.5:2.6:2.7:2.8_	ELMOD3	RI	2.3:2.4:2.5:2.6:2.7	2.1	2.9	8.4E-17	0.207	0.344	6.7E-03	1.4E-02	2.7E-01
KIRC	ELMOD3_RI_2.4:2.5:2.6:2.7:2.8_2.3_2.9	ELMOD3	RI	2.4:2.5:2.6:2.7:2.8	2.3	2.9	2.4E-17	0.167	0.394	8.4E-03	2.1E-02	1.5E-01
KIRC	ELMOD3_RI_7.2_7.1_7.3	ELMOD3	RI	7.2	7.1	7.3	4.5E-17	0.177	0.368	1.9E-03	5.1E-03	1.9E-01
KIRC	ELP4_ES_10:11_9_12	ELP4	ES	10:11	9	12	2.6E-24	0.228	-0.562	1.5E-03	4.8E-05	6.7E-04
KIRC	ELP5_RI_7.2_7.1_7.3	ELP5	RI	7.2	7.1	7.3	1.0E-16	0.162	-0.203	5.4E-06	1.2E-03	1.3E-02
KIRC	ELP5_RI_7.4_7.3_7.5	ELP5	RI	7.4	7.3	7.5	1.4E-12	0.123	-0.100	4.8E-05	3.1E-05	1.8E-02
KIRC	EMC10_ES_7_6_8.1	EMC10	ES	7	6	8.1	3.3E-12	0.120	0.226	2.2E-04	5.4E-05	8.8E-03
KIRC	EMC9_AA_3.1:3.2_2_3.3	EMC9	AA	3.1:3.2	2	3.3	2.3E-19	0.185	-0.143	6.1E-02	3.6E-01	5.7E-01
KIRC	EMC9_AA_3.2_2_3.3	EMC9	AA	3.2	2	3.3	9.8E-18	0.171	-0.090	4.5E-03	2.0E-02	2.0E-02
KIRC	ENAH_AA_7.1_6_7.2	ENAH	AA	7.1	6	7.2	7.0E-11	0.108	-0.102	4.3E-04	3.7E-05	1.8E-02
KIRC	ENOSF1_ES_15:16_13_17	ENOSF1	ES	15:16	13	17	3.0E-14	0.151	0.117	1.9E-07	2.7E-06	2.2E-05
KIRC	ENOSF1_ES_6_5_7	ENOSF1	ES	6	5	7	7.8E-24	0.225	0.375	1.1E-04	7.0E-03	2.7E-02
KIRC	ENTHD2_AD_1.2_1.1_2	ENTHD2	AD	1.2	1.1	2	9.4E-17	0.167	0.625	1.3E-02	2.7E-04	5.7E-02
KIRC	EPB41L1_ES_20_19_21	EPB41L1	ES	20	19	21	5.9E-11	0.108	-0.084	7.7E-01	5.6E-02	3.1E-02
KIRC	EPB41L3_ES_25_24_26	EPB41L3	ES	25	24	26	2.7E-26	0.245	-0.303	9.1E-06	4.1E-03	9.5E-02
KIRC	EPHB2_RI_17.2_17.1_17.3	EPHB2	RI	17.2	17.1	17.3	2.8E-17	0.168	-0.618	9.8E-01	8.9E-01	6.6E-01

cancer type	id	Gene Symbol	splice_type	Exon	From.Exon	To.Exon	anova.p	adj.r2	r	p.50	p.25	p.10
KIRC	EPOR_ES_4.1:4.2_3_5	EPOR	ES	4.1:4.2	3	5	1.7E-11	0.143	0.388	9.8E-01	6.0E-01	1.5E-01
KIRC	EPS8L1_ES_16_15.3_17	EPS8L1	ES	16	15.3	17	2.3E-13	0.167	-0.305	2.8E-02	8.5E-05	2.7E-03
KIRC	EPS8L2_RI_4.2_4.1_4.3	EPS8L2	RI	4.2	4.1	4.3	3.0E-35	0.313	0.412	4.4E-05	2.4E-05	2.2E-03
KIRC	ERCC5_RI_11.3_11.2_11.4	ERCC5	RI	11.3	11.2	11.4	2.4E-32	0.292	0.250	5.1E-07	8.7E-08	4.4E-04
KIRC	ERCC8_ES_3_2_4	ERCC8	ES	3	2	4	4.4E-23	0.234	-0.150	2.4E-03	2.7E-02	1.4E-02
KIRC	ERGIC1_ES_6.2:6.3:8_6.1_9	ERGIC1	ES	6.2:6.3:8	6.1	9	1.7E-11	0.130	-0.018	1.7E-04	3.5E-04	7.1E-02
KIRC	ERGIC1_RI_6.2_6.1_6.3	ERGIC1	RI	6.2	6.1	6.3	2.7E-21	0.205	-0.226	9.4E-06	6.0E-10	1.8E-05
KIRC	ERLIN2_RI_7.2_7.1_7.3	ERLIN2	RI	7.2	7.1	7.3	8.8E-11	0.107	-0.127	1.9E-02	3.1E-01	6.7E-01
KIRC	ERMAP_RI_3.3_3.2_3.4	ERMAP	RI	3.3	3.2	3.4	5.4E-23	0.223	-0.348	1.4E-05	3.3E-05	1.8E-04
KIRC	ERRFI1_AA_3.1_2_3.2	ERRFI1	AA	3.1	2	3.2	1.3E-24	0.234	0.065	7.0E-02	6.7E-02	5.9E-02
KIRC	ERRFI1_AA_3.1:3.2:3.3_2_3.4	ERRFI1	AA	3.1:3.2:3.3	2	3.4	2.5E-17	0.173	-0.063	7.1E-06	7.2E-06	7.2E-04
KIRC	ERRFI1_AA_3.2:3.3_2_3.4	ERRFI1	AA	3.2:3.3	2	3.4	4.1E-11	0.110	-0.020	1.1E-06	2.3E-06	3.8E-03
KIRC	ERRFI1_RI_3.3_3.2_3.4	ERRFI1	RI	3.3	3.2	3.4	4.8E-31	0.282	0.085	1.2E-04	4.1E-06	5.9E-03
KIRC	EXD3_ES_5:6:7_4_8	EXD3	ES	5:06:07	4	8	9.0E-09	0.102	0.352	2.0E-02	2.5E-01	7.3E-01
KIRC	EXOC1_ES_11_10_12	EXOC1	ES	11	10	12	1.4E-12	0.123	0.063	9.8E-07	1.2E-05	2.1E-06
KIRC	EXOC3_RI_10.2_10.1_10.3	EXOC3	RI	10.2	10.1	10.3	6.8E-32	0.288	0.019	3.4E-06	9.8E-05	4.5E-03
KIRC	EXOC7_AA_8.1_7_8.2	EXOC7	AA	8.1	7	8.2	1.7E-28	0.277	0.093	1.0E-06	1.3E-07	1.0E-05
KIRC	EXOC7_ES_7:8.1_6_8.2	EXOC7	ES	07:08.1	6	8.2	2.4E-14	0.159	0.207	4.1E-04	2.6E-06	2.6E-03
KIRC	EXOC7_ES_8.1:8.2_7_9	EXOC7	ES	8.1:8.2	7	9	2.3E-26	0.253	0.035	4.8E-06	7.8E-07	3.6E-04
KIRC	EXOSC10_ES_4_3_5	EXOSC10	ES	4	3	5	1.9E-39	0.343	0.173	7.4E-06	9.3E-05	3.1E-03
KIRC	EXOSC10_RI_16.2_16.1_16.3	EXOSC10	RI	16.2	16.1	16.3	1.6E-31	0.285	0.364	8.2E-04	3.7E-04	6.1E-03
KIRC	EXOSC10_RI_16.4_16.3_16.5	EXOSC10	RI	16.4	16.3	16.5	2.6E-33	0.298	0.173	1.1E-04	1.8E-05	2.3E-03
KIRC	EXOSC9_RI_10.2:10.3:10.4_10.1_10.5	EXOSC9	RI	10.2:10.3:10.4	10.1	10.5	4.1E-15	0.147	0.042	1.5E-04	2.7E-04	7.6E-03
KIRC	EXOSC9_RI_10.4_10.3_10.5	EXOSC9	RI	10.4	10.3	10.5	3.2E-10	0.103	0.019	2.1E-02	2.7E-03	2.9E-03
KIRC	EZH2_RI_14.2_14.1_14.3	EZH2	RI	14.2	14.1	14.3	2.5E-15	0.162	0.131	5.8E-04	5.1E-04	8.8E-04
KIRC	EZH2_RI_14.4_14.3_14.5	EZH2	RI	14.4	14.3	14.5	5.9E-19	0.188	0.220	7.1E-05	6.6E-04	1.0E-02
KIRC	FADS3_RI_9.2_9.1_9.3	FADS3	RI	9.2	9.1	9.3	5.7E-26	0.241	0.303	1.8E-04	5.2E-04	1.9E-03
KIRC	FAIM_ES_3_1_6	FAIM	ES	3	1	6	3.6E-19	0.184	-0.258	8.1E-04	1.6E-03	1.1E-03
KIRC	FAM110A_ES_3_1_4.2	FAM110A	ES	3	1	4.2	2.0E-13	0.134	0.012	8.2E-03	3.4E-03	9.7E-04
KIRC	FAM111A_AA_3.1:3.2_2_3.3	FAM111A	AA	3.1:3.2	2	3.3	7.6E-18	0.173	0.578	9.7E-03	5.6E-04	1.1E-02
KIRC	FAM111A_RI_3.2_3.1_3.3	FAM111A	RI	3.2	3.1	3.3	1.6E-20	0.197	0.567	3.6E-03	2.4E-05	1.3E-01
KIRC	FAM115C_ES_7_6_8	FAM115C	ES	7	6	8	2.5E-19	0.190	-0.126	3.0E-04	3.2E-04	1.2E-02
KIRC	FAM131A_RI_6.3_6.2_6.4	FAM131A	RI	6.3	6.2	6.4	4.3E-23	0.222	0.398	5.9E-07	4.5E-06	1.8E-04
KIRC	FAM136A_ES_1.3_1.1_2	FAM136A	ES	1.3	1.1	2	2.8E-27	0.253	0.160	5.2E-01	2.4E-01	9.7E-01
KIRC	FAM13A_AA_16.1_15_16.2	FAM13A	AA	16.1	15	16.2	3.9E-11	0.110	-0.060	5.6E-05	7.3E-04	1.0E-03
KIRC	FAM156B_RI_2.2:2.3:2.4_2.1_2.5	FAM156B	RI	2.2:2.3:2.4	2.1	2.5	1.1E-13	0.134	0.669	6.7E-05	1.2E-05	1.2E-03
KIRC	FAM160B2_ES_2_1_3	FAM160B2	ES	2	1	3	5.8E-10	0.110	0.330	1.8E-02	1.5E-02	1.4E-01
KIRC	FAM173A_RI_4.2_4.1_4.3	FAM173A	RI	4.2	4.1	4.3	2.9E-19	0.186	-0.057	3.8E-04	5.6E-06	9.8E-03
KIRC	FAM179B_ES_8_7_9	FAM179B	ES	8	7	9	1.6E-13	0.147	-0.317	4.8E-03	3.2E-02	2.0E-03
KIRC	FAM193A_ES_22_21_23	FAM193A	ES	22	21	23	1.7E-12	0.123	0.158	6.7E-04	5.4E-03	1.3E-03

cancer type	id	Gene Symbol	splice_type	Exon	From.Exon	To.Exon	anova.p	adj.r2	r	p.50	p.25	p.10
KIRC	FAM193A_ES_6_5_7	FAM193A	ES	6	5	7	6.2E-21	0.207	0.323	3.7E-03	2.8E-05	1.3E-01
KIRC	FAM195A_ES_3_2_4	FAM195A	ES	3	2	4	1.2E-18	0.179	-0.002	4.4E-04	2.1E-04	2.8E-04
KIRC	FAM210A_ES_2_1_3	FAM210A	ES	2	1	3	1.4E-26	0.262	-0.403	3.9E-06	2.1E-06	4.8E-04
KIRC	FAM214A_AD_12.2_12.1_13	FAM214A	AD	12.2	12.1	13	8.7E-12	0.116	0.046	1.1E-01	1.9E-01	1.0E-01
KIRC	FAM3A_AA_3.1_2_3.2	FAM3A	AA	3.1	2	3.2	4.2E-12	0.119	0.177	5.1E-03	5.7E-02	8.3E-03
KIRC	FAM73B_AA_15.1_14_15.2	FAM73B	AA	15.1	14	15.2	4.8E-21	0.223	0.355	4.0E-06	3.3E-05	6.5E-03
KIRC	FAM73B_AD_15.3_15.2_16	FAM73B	AD	15.3	15.2	16	1.6E-21	0.206	0.499	1.4E-05	3.4E-06	1.0E-02
KIRC	FAM76A_ES_3_2_4	FAM76A	ES	3	2	4	2.3E-26	0.246	-0.009	3.3E-07	6.1E-07	3.8E-03
KIRC	FAM76B_ES_2_1_3	FAM76B	ES	2	1	3	5.3E-18	0.204	0.278	2.7E-03	9.6E-04	1.8E-02
KIRC	FAM76B_ES_9_8_10.1	FAM76B	ES	9	8	10.1	4.5E-21	0.204	0.325	1.9E-03	4.4E-03	2.4E-02
KIRC	FAM76B_ES_9:10.1_8_10.2	FAM76B	ES	09:10.1	8	10.2	3.0E-09	0.100	0.241	3.4E-01	1.8E-02	1.9E-02
KIRC	FAM86A_ES_3.2:5.1_3.1_5.2	FAM86A	ES	3.2:5.1	3.1	5.2	6.9E-14	0.163	0.042	5.5E-06	1.3E-07	1.7E-03
KIRC	FAM86B1_AA_3.1_1.2_3.2	FAM86B1	AA	3.1	1.2	3.2	3.7E-17	0.172	0.007	6.1E-03	9.2E-04	8.3E-05
KIRC	FAM86B1_AD_7.2:7.3_7.1_8.1	FAM86B1	AD	7.2:7.3	7.1	8.1	1.1E-13	0.135	-0.221	2.0E-04	9.0E-05	3.9E-04
KIRC	FAM86B1_ES_4:5:6:7.1:7.2:7.3_3.2_8.1	FAM86B1	ES	4:5:6:7.1:7.2:7.3	3.2	8.1	5.1E-18	0.205	0.073	1.1E-04	5.9E-06	1.5E-03
KIRC	FAM86B1_ES_4:7.1:7.2:7.3_3.2_8.1	FAM86B1	ES	4:7.1:7.2:7.3	3.2	8.1	1.2E-10	0.132	0.104	7.0E-02	3.1E-02	3.0E-02
KIRC	FAM86B1_ES_5:6:7.1:7.2:7.3_4_8.1	FAM86B1	ES	5:6:7.1:7.2:7.3	4	8.1	1.1E-13	0.152	0.088	7.4E-04	1.8E-05	2.8E-02
KIRC	FAM86B1_RI_7.2_7.1_7.3	FAM86B1	RI	7.2	7.1	7.3	1.9E-09	0.103	-0.270	1.7E-06	8.4E-05	5.4E-04
KIRC	FANCI_ES_1.2:2:3_1.1_4	FANCI	ES	1.2:2:3	1.1	4	3.0E-12	0.141	-0.380	4.1E-03	7.3E-02	1.3E-01
KIRC	FASTK_AD_1.2_1.1_2	FASTK	AD	1.2	1.1	2	4.3E-13	0.129	0.197	7.8E-02	1.1E-01	9.1E-02
KIRC	FASTK_RI_5.4_5.3_5.5	FASTK	RI	5.4	5.3	5.5	9.8E-31	0.279	0.302	3.8E-04	3.2E-03	1.7E-01
KIRC	FBLN5_ES_7_5_8	FBLN5	ES	7	5	8	8.0E-23	0.216	-0.625	2.9E-03	1.1E-02	5.3E-02
KIRC	FBXL12_ES_3.1:3.2_2.4_5	FBXL12	ES	3.1:3.2	2.4	5	4.3E-16	0.160	0.285	5.0E-02	5.6E-03	2.2E-03
KIRC	FBXL12_ES_3.2_2.4_5	FBXL12	ES	3.2	2.4	5	4.0E-13	0.132	0.400	2.9E-01	4.2E-02	2.6E-03
KIRC	FBXO17_AA_5.1_4_5.2	FBXO17	AA	5.1	4	5.2	2.6E-33	0.299	0.099	4.8E-05	2.2E-07	2.0E-03
KIRC	FBXO3_RI_12.2:12.3_12.1_12.4	FBXO3	RI	12.2:12.3	12.1	12.4	8.7E-27	0.248	-0.673	1.9E-11	4.6E-11	2.8E-03
KIRC	FBXO4_ES_3_2_4	FBXO4	ES	3	2	4	3.1E-27	0.251	-0.237	1.2E-04	8.4E-06	1.0E-05
KIRC	FBXO44_ES_6_5.1_7	FBXO44	ES	6	5.1	7	5.2E-10	0.101	0.192	4.1E-01	1.2E-01	6.3E-02
KIRC	FDXR_AA_6.1:6.2_3.2_6.3	FDXR	AA	6.1:6.2	3.2	6.3	5.7E-13	0.144	0.020	9.6E-05	2.4E-04	3.8E-02
KIRC	FES_RI_2.2_2.1_2.3	FES	RI	2.2	2.1	2.3	2.5E-20	0.203	0.095	9.5E-04	7.2E-05	1.8E-03
KIRC	FGFR1_ES_7_6_8.2	FGFR1	ES	7	6	8.2	8.7E-12	0.124	0.032	3.4E-03	2.2E-03	8.0E-03
KIRC	FGFR1OP_ES_7_6_8	FGFR1OP	ES	7	6	8	7.2E-13	0.127	-0.309	1.4E-03	1.9E-02	3.6E-02
KIRC	FGFR4_ES_3_2_4	FGFR4	ES	3	2	4	3.1E-17	0.180	0.093	3.1E-03	7.5E-03	3.4E-02
KIRC	FGFR4_RI_10.3_10.2_10.4	FGFR4	RI	10.3	10.2	10.4	7.3E-20	0.194	0.024	1.8E-03	1.2E-02	3.6E-03
KIRC	FHL2_ES_2.3:3.2:4_2.2_5.1	FHL2	ES	2.3:3.2:4	2.2	5.1	7.0E-19	0.190	-0.331	2.4E-02	4.2E-02	5.6E-02
KIRC	FHL2_ES_3.2:4_2.2_5.1	FHL2	ES	3.2:4	2.2	5.1	4.7E-19	0.192	-0.385	1.4E-02	1.2E-01	1.8E-01
KIRC	FHL2_ES_3.2:4_2.3_5.1	FHL2	ES	3.2:4	2.3	5.1	7.8E-18	0.177	-0.292	2.7E-02	8.1E-03	1.7E-02
KIRC	FHL2_ES_4_3.2_5.1	FHL2	ES	4	3.2	5.1	1.0E-20	0.198	-0.508	9.3E-03	1.6E-02	9.9E-03
KIRC	FIGNL1_AA_2.2:2.3:2.4:2.5_1_2.6	FIGNL1	AA	2.2:2.3:2.4:2.5	1	2.6	2.9E-13	0.148	0.068	1.1E-04	1.5E-06	4.2E-04
KIRC	FIGNL1_RI_2.3:2.4:2.5_2.2_2.6	FIGNL1	RI	2.3:2.4:2.5	2.2	2.6	9.0E-16	0.165	0.047	1.2E-04	2.9E-04	2.5E-02

cancer type	id	Gene Symbol	splice_type	Exon	From.Exon	To.Exon	anova.p	adj.r2	r	p.50	p.25	p.10
KIRC	FIGNL1_RI_2.5_2.4_2.6	FIGNL1	RI	2.5	2.4	2.6	2.8E-10	0.115	-0.113	1.6E-03	1.7E-03	1.2E-01
KIRC	FKBP3_ES_3_2_4	FKBP3	ES	3	2	4	9.7E-36	0.316	-0.022	6.3E-04	9.1E-04	2.3E-02
KIRC	FKTN_ES_7_6_8	FKTN	ES	7	6	8	1.6E-19	0.221	-0.202	1.5E-03	5.5E-03	2.1E-02
KIRC	FLAD1_RI_1.2_1.1_1.3	FLAD1	RI	1.2	1.1	1.3	1.2E-13	0.134	-0.118	1.6E-04	8.3E-05	8.6E-04
KIRC	FLAD1_RI_4.2_4.1_4.3	FLAD1	RI	4.2	4.1	4.3	8.3E-31	0.280	0.230	2.2E-04	6.3E-04	6.4E-02
KIRC	FLNA_ES_37:38:39:40:41:43:44:45_36_4	FLNA	ES	38:39:40:41:43:4	36	46	7.4E-28	0.264	0.087	6.5E-02	8.0E-02	9.3E-01
KIRC	FLOT2_ES_3:4:5_2_6	FLOT2	ES	3:04:05	2	6	4.6E-12	0.139	-0.186	9.1E-02	7.3E-02	6.7E-01
KIRC	FLOT2_ES_4:5_3_6	FLOT2	ES	4:05	3	6	1.8E-13	0.143	-0.130	9.2E-02	1.1E-03	1.5E-01
KIRC	FLYWCH1_ES_7:8_6_9	FLYWCH1	ES	7:08	6	9	3.1E-25	0.239	0.370	4.9E-03	3.3E-03	1.3E-05
KIRC	FLYWCH1_ES_7:8:9_6_10	FLYWCH1	ES	7:08:09	6	10	1.7E-12	0.157	0.396	4.2E-02	1.2E-02	4.1E-02
KIRC	FNIP1_ES_7_6_8	FNIP1	ES	7	6	8	5.8E-19	0.202	0.199	2.2E-05	2.8E-03	6.8E-03
KIRC	FOS_RI_2.3_2.2_2.4	FOS	RI	2.3	2.2	2.4	2.1E-16	0.159	-0.131	5.6E-04	1.2E-03	1.2E-02
KIRC	FOXM1_ES_9_8_10	FOXM1	ES	9	8	10	1.0E-14	0.161	-0.237	4.9E-02	6.3E-02	2.7E-01
KIRC	FOXP1_ES_7:8_6_10	FOXP1	ES	7:08	6	10	1.6E-10	0.103	0.084	1.1E-03	8.8E-05	7.7E-04
KIRC	FOXRED1_AD_1.2_1.1_2	FOXRED1	AD	1.2	1.1	2	1.7E-15	0.164	-0.009	2.1E-05	2.0E-04	1.4E-03
KIRC	FOXRED1_ES_1.2:2_1.1_3	FOXRED1	ES	1.2:2	1.1	3	4.2E-08	0.102	0.048	8.1E-03	1.9E-03	1.3E-01
KIRC	FPGT_ES_3_2.1_4	FPGT	ES	3	2.1	4	3.8E-30	0.276	-0.293	1.7E-06	4.1E-05	7.9E-04
KIRC	FYN_ME_11|12_10_13	FYN	ME	11|12	10	13	7.3E-11	0.107	0.329	2.3E-05	7.5E-06	1.8E-02
KIRC	GABARAPL1_AD_2.13:2.14_2.12_3	GABARAPL1	AD	2.13:2.14	2.12	3	1.9E-11	0.116	-0.234	1.1E-01	1.3E-02	3.2E-01
KIRC	GALK2_ES_15_14_16	GALK2	ES	15	14	16	2.2E-25	0.236	-0.416	1.2E-04	3.3E-05	4.2E-02
KIRC	GALK2_ES_5_1_6	GALK2	ES	5	1	6	4.9E-12	0.130	-0.321	1.2E-04	2.6E-02	1.7E-02
KIRC	GALK2_ES_5_3.2_6	GALK2	ES	5	3.2	6	1.0E-17	0.188	-0.378	8.9E-04	8.6E-05	8.9E-07
KIRC	GAPVD1_ES_2:3_1_4	GAPVD1	ES	2:03	1	4	9.8E-10	0.111	-0.301	4.4E-06	7.3E-05	1.7E-04
KIRC	GAS2L1_RI_4.2_4.1_4.3	GAS2L1	RI	4.2	4.1	4.3	7.3E-13	0.127	-0.032	2.0E-05	4.3E-05	3.0E-02
KIRC	GBA2_AA_5.1_4_5.2	GBA2	AA	5.1	4	5.2	1.8E-13	0.134	0.305	3.3E-01	2.4E-02	6.1E-02
KIRC	GBA2_ES_5.1:5.2_4_6	GBA2	ES	5.1:5.2	4	6	1.9E-09	0.122	0.217	4.5E-01	2.4E-02	1.5E-01
KIRC	GBA2_RI_14.3_14.2_14.4	GBA2	RI	14.3	14.2	14.4	1.2E-20	0.197	-0.094	8.3E-07	4.2E-05	1.2E-02
KIRC	GBA2_RI_14.5_14.4_14.6	GBA2	RI	14.5	14.4	14.6	8.2E-11	0.106	-0.219	2.5E-07	6.4E-04	4.6E-02
KIRC	GCDH_ME_4|5_3_6	GCDH	ME	4|5	3	6	9.9E-24	0.223	0.107	3.6E-07	8.2E-05	1.7E-02
KIRC	GDPD5_ES_16_15_17	GDPD5	ES	16	15	17	1.5E-12	0.146	0.151	2.3E-02	4.9E-02	2.6E-01
KIRC	GDPD5_ES_9:10_8_13	GDPD5	ES	9:10	8	13	1.3E-11	0.132	-0.032	5.3E-01	4.6E-02	2.7E-01
KIRC	GEMIN2_ES_5_4_6	GEMIN2	ES	5	4	6	1.2E-37	0.331	-0.299	1.2E-02	5.5E-03	2.8E-02
KIRC	GFM2_ES_2_1_3	GFM2	ES	2	1	3	2.4E-19	0.200	-0.357	1.0E-02	2.2E-04	4.4E-05
KIRC	GGA2_AA_4.1_3.1_4.2	GGA2	AA	4.1	3.1	4.2	7.4E-35	0.312	-0.084	2.9E-06	2.2E-07	1.0E-04
KIRC	GGA3_RI_14.2_14.1_14.3	GGA3	RI	14.2	14.1	14.3	2.3E-34	0.309	0.518	2.6E-07	7.4E-07	6.7E-02
KIRC	GGT1_AA_15.3_14_15.4	GGT1	AA	15.3	14	15.4	6.9E-11	0.107	-0.292	6.2E-06	4.2E-06	1.9E-05
KIRC	GGT1_AA_7.1:7.2_6.2_7.3	GGT1	AA	7.1:7.2	6.2	7.3	2.8E-10	0.101	-0.264	3.9E-03	2.4E-03	9.8E-03
KIRC	GIT2_AA_18.1_15_18.2	GIT2	AA	18.1	15	18.2	1.9E-13	0.139	-0.186	1.3E-02	3.4E-04	3.3E-03
KIRC	GIT2_AA_18.1_17.2_18.2	GIT2	AA	18.1	17.2	18.2	3.1E-35	0.313	-0.265	1.2E-08	2.2E-09	1.3E-05
KIRC	GIT2_ES_17.1:17.2:18.1_15_18.2	GIT2	ES	17.1:17.2:18.1	15	18.2	1.3E-32	0.293	-0.227	1.5E-05	7.5E-08	4.3E-06

cancer type	id	Gene Symbol	splice_type	Exon	From.Exon	To.Exon	anova.p	adj.r2	r	p.50	p.25	p.10
KIRC	GK_ES_23_22_24	GK	ES	23	22	24	3.2E-18	0.176	-0.476	5.2E-08	2.2E-07	4.2E-02
KIRC	GKAP1_ES_7_6_8	GKAP1	ES	7	6	8	2.1E-12	0.134	-0.026	4.7E-02	6.3E-03	1.3E-02
KIRC	GLIPR1_RI_5.2_5.1_5.3	GLIPR1	RI	5.2	5.1	5.3	7.5E-21	0.198	-0.648	6.6E-05	4.2E-06	3.2E-06
KIRC	GLOD4_ES_3_1_4.2	GLOD4	ES	3	1	4.2	3.4E-15	0.148	-0.382	6.8E-07	1.4E-06	1.6E-05
KIRC	GLRX_AA_3.1_2.1_3.2	GLRX	AA	3.1	2.1	3.2	1.2E-17	0.171	-0.080	9.4E-06	1.7E-07	8.7E-04
KIRC	GLUL_AD_1.3:1.4_1.2_2	GLUL	AD	1.3:1.4	1.2	2	1.3E-24	0.231	0.243	4.5E-10	2.4E-08	2.3E-06
KIRC	GMPPA_RI_9.2:9.3_9.1_9.4	GMPPA	RI	9.2:9.3	9.1	9.4	2.0E-31	0.285	-0.053	2.8E-05	3.3E-06	3.6E-04
KIRC	GNB2L1_ES_7.2:8.1:8.2_7.1_9	GNB2L1	ES	7.2:8.1:8.2	7.1	9	1.9E-17	0.169	-0.315	1.6E-02	2.6E-02	5.4E-02
KIRC	GNLY_ES_2.1:2.2_1_3	GNLY	ES	2.1:2.2	1	3	5.8E-12	0.119	-0.167	6.4E-01	3.8E-01	8.9E-01
KIRC	GNPDA1_RI_2.5_2.4_2.6	GNPDA1	RI	2.5	2.4	2.6	2.5E-21	0.213	-0.261	1.1E-01	3.5E-02	2.9E-01
KIRC	GOLGA4_ES_24_23_25	GOLGA4	ES	24	23	25	4.7E-17	0.165	0.174	1.2E-05	3.9E-04	9.9E-05
KIRC	GOLT1B_ES_4_2_5	GOLT1B	ES	4	2	5	9.4E-21	0.199	-0.223	2.5E-01	1.3E-02	5.1E-02
KIRC	GORASP1_AA_4.1_3_4.2	GORASP1	AA	4.1	3	4.2	9.5E-21	0.210	0.155	1.2E-06	8.0E-05	4.1E-03
KIRC	GPAM_RI_19.4_19.3_19.5	GPAM	RI	19.4	19.3	19.5	7.0E-15	0.170	-0.249	1.1E-03	3.4E-03	1.0E+00
KIRC	GPHN_ES_2_1_3	GPHN	ES	2	1	3	4.8E-24	0.226	-0.448	2.3E-06	2.3E-06	2.4E-03
KIRC	GPR89A_AA_7.1_6_7.2	GPR89A	AA	7.1	6	7.2	3.6E-16	0.158	-0.008	2.4E-01	5.0E-01	8.9E-01
KIRC	GPS1_RI_1.2_1.1_1.3	GPS1	RI	1.2	1.1	1.3	1.4E-12	0.151	0.138	1.9E-02	6.9E-03	4.2E-03
KIRC	GPS1_RI_1.2:1.3:1.4_1.1_1.5	GPS1	RI	1.2:1.3:1.4	1.1	1.5	4.7E-22	0.214	0.106	2.9E-02	2.8E-03	4.3E-03
KIRC	GRN_ES_4:5:6.1:6.2:7:8:9_3_11	GRN	ES	4:5:6.1:6.2:7:8:9	3	11	1.8E-10	0.108	-0.144	8.9E-01	3.8E-01	2.8E-01
KIRC	GSKIP_AD_1.2_1.1_2.2	GSKIP	AD	1.2	1.1	2.2	2.0E-14	0.141	-0.224	7.5E-03	1.2E-04	2.9E-03
KIRC	GSS_RI_5.2_5.1_5.3	GSS	RI	5.2	5.1	5.3	1.8E-37	0.329	0.158	6.4E-04	2.0E-04	5.2E-04
KIRC	GSTK1_AD_4.2:4.3_4.1_5	GSTK1	AD	4.2:4.3	4.1	5	6.7E-19	0.182	0.053	1.1E-04	4.6E-08	1.4E-02
KIRC	GSTT1_ES_2:3_1_4.1	GSTT1	ES	2:03	1	4.1	1.2E-08	0.103	-0.126	4.0E-01	6.8E-01	6.6E-01
KIRC	GTF2IRD1_RI_27.2_27.1_27.3	GTF2IRD1	RI	27.2	27.1	27.3	2.9E-15	0.149	0.004	1.6E-01	1.1E-01	6.8E-01
KIRC	GTPBP3_RI_6.2_6.1_6.3	GTPBP3	RI	6.2	6.1	6.3	1.2E-23	0.229	0.546	4.9E-05	1.5E-04	1.6E-02
KIRC	GUK1_ES_11.1:11.2:11.3_9.2_12	GUK1	ES	11.1:11.2:11.3	9.2	12	1.4E-20	0.197	-0.168	6.5E-02	6.7E-03	1.6E-01
KIRC	GUK1_ES_3_2_5.3	GUK1	ES	3	2	5.3	5.4E-11	0.108	-0.288	2.5E-04	2.1E-04	2.4E-04
KIRC	HACE1_ES_7_6_8	HACE1	ES	7	6	8	5.9E-25	0.253	-0.309	4.6E-01	5.7E-01	2.3E-01
KIRC	HAGHL_RI_6.4_6.3_6.5	HAGHL	RI	6.4	6.3	6.5	7.2E-10	0.130	-0.135	6.7E-04	2.1E-03	6.3E-01
KIRC	HAPLN3_ES_5_4_6	HAPLN3	ES	5	4	6	1.4E-19	0.190	0.043	2.5E-05	1.6E-03	7.4E-02
KIRC	HARS2_AA_2.1_1.3_2.2	HARS2	AA	2.1	1.3	2.2	5.3E-18	0.175	0.096	1.7E-03	4.8E-02	2.6E-02
KIRC	HARS2_RI_6.2_6.1_6.3	HARS2	RI	6.2	6.1	6.3	1.0E-33	0.302	0.190	1.1E-06	5.4E-04	3.0E-02
KIRC	HAUS1_ES_3_2.1_4	HAUS1	ES	3	2.1	4	2.2E-29	0.268	-0.275	3.3E-04	3.6E-03	2.4E-03
KIRC	HAUS5_RI_15.2_15.1_15.3	HAUS5	RI	15.2	15.1	15.3	3.2E-26	0.248	0.607	1.2E-05	1.2E-06	3.4E-03
KIRC	HCK_ES_2_1_4	HCK	ES	2	1	4	1.5E-10	0.110	-0.172	1.0E-04	4.6E-07	1.6E-04
KIRC	HDAC10_RI_18.2_18.1_18.3	HDAC10	RI	18.2	18.1	18.3	2.8E-19	0.186	0.419	3.3E-05	6.4E-04	2.2E-02
KIRC	HDAC6_RI_13.2_13.1_13.3	HDAC6	RI	13.2	13.1	13.3	3.5E-38	0.339	0.239	1.6E-05	5.3E-07	2.2E-04
KIRC	HDHD2_ES_3_1_4.1	HDHD2	ES	3	1	4.1	7.0E-19	0.187	-0.335	1.0E-07	4.6E-09	1.4E-06
KIRC	HEATR6_ES_3_2_4	HEATR6	ES	3	2	4	5.5E-17	0.194	-0.059	3.3E-03	1.3E-04	2.0E-04
KIRC	HERC2_ES_52_51_53	HERC2	ES	52	51	53	3.7E-21	0.202	0.331	1.5E-08	2.9E-08	1.6E-03

cancer type	id	Gene Symbol	splice_type	Exon	From.Exon	To.Exon	anova.p	adj.r2	r	p.50	p.25	p.10
KIRC	HINT1_RI_2.2_2.1_2.3	HINT1	RI	2.2	2.1	2.3	4.4E-11	0.115	-0.141	4.1E-06	9.8E-05	1.3E-03
KIRC	HKR1_ES_11_8.2_12	HKR1	ES	11	8.2	12	1.1E-25	0.240	0.380	3.8E-05	5.2E-05	1.5E-02
KIRC	HKR1_ES_14:15_13_17.2	HKR1	ES	14:15	13	17.2	1.7E-12	0.125	0.274	4.3E-04	2.1E-05	2.9E-04
KIRC	HKR1_ES_8.1:8.2:11_7_12	HKR1	ES	8.1:8.2:11	7	12	2.3E-13	0.152	0.353	9.1E-07	2.1E-05	5.4E-03
KIRC	HKR1_ES_9_8.2_12	HKR1	ES	9	8.2	12	3.4E-21	0.226	0.433	8.8E-04	1.2E-02	1.2E-01
KIRC	HLCS_ES_4_3_6	HLCS	ES	4	3	6	1.6E-08	0.102	-0.207	1.9E-02	1.0E-03	1.7E-01
KIRC	HLCS_ME_4|5_3_6	HLCS	ME	4|5	3	6	7.6E-14	0.136	-0.400	2.0E-01	2.6E-03	1.3E-01
KIRC	HMBS_RI_12.2_12.1_12.3	HMBS	RI	12.2	12.1	12.3	1.9E-30	0.279	-0.069	1.5E-03	1.6E-03	8.8E-02
KIRC	HMGB1_RI_4.2_4.1_4.3	HMGB1	RI	4.2	4.1	4.3	5.7E-23	0.217	0.019	2.0E-06	1.3E-08	4.7E-07
KIRC	HNRNPA1_ES_3:4:5:6.1:6.2:10:11.1:11.2	HNRNPA1	ES	;:6.1:6.2:10:11.1:	2	11.3	6.6E-19	0.183	-0.107	4.7E-06	1.7E-06	1.5E-03
KIRC	HNRNPA1_ES_3:4:5:6.1:6.2:7.1:7.2:8:9.1	HNRNPA1	ES	:7.1:7.2:8:9.1:9.2	2	11.3	3.9E-17	0.173	-0.112	1.3E-04	3.9E-06	5.1E-04
KIRC	HNRNPA1_ES_3:4:5:6.1:6.2:7.1:7.2:9.1:9	HNRNPA1	ES	6.2:7.1:7.2:9.1:9.	2	11.3	4.9E-27	0.251	-0.044	1.5E-04	6.9E-06	5.2E-05
KIRC	HNRNPA1_ES_3:4:5:6.1:9.2:10:11.1:11.2	HNRNPA1	ES	;:6.1:9.2:10:11.1:	2	11.3	5.9E-17	0.169	-0.119	1.6E-06	7.2E-05	6.7E-04
KIRC	HNRNPA1_RI_11.3_11.2_11.4	HNRNPA1	RI	11.3	11.2	11.4	1.0E-23	0.222	-0.128	5.7E-08	1.9E-07	4.2E-05
KIRC	HNRNPA2B1_AA_12.2:12.3_11_12.4	HNRNPA2B1	AA	12.2:12.3	11	12.4	2.0E-24	0.229	0.458	4.2E-03	5.6E-03	8.7E-02
KIRC	HNRNPA2B1_ES_12.2_11_12.4	HNRNPA2B1	ES	12.2	11	12.4	2.8E-12	0.121	0.298	4.0E-02	1.3E-01	4.0E-01
KIRC	HNRNPC_ES_2.2:2.3:2.4_1_3.2	HNRNPC	ES	2.2:2.3:2.4	1	3.2	2.1E-13	0.131	-0.233	7.4E-02	2.4E-01	1.2E-02
KIRC	HNRNPC_ES_2.4:2.5:2.6_1_3.2	HNRNPC	ES	2.4:2.5:2.6	1	3.2	3.6E-18	0.175	-0.151	1.5E-01	1.7E-01	1.5E-01
KIRC	HNRNPDL_ES_8_7_9	HNRNPDL	ES	8	7	9	1.0E-23	0.222	-0.034	2.4E-03	5.1E-05	1.7E-02
KIRC	HOOK2_AD_4.2_4.1_5	HOOK2	AD	4.2	4.1	5	3.4E-31	0.287	0.409	6.7E-08	2.7E-05	6.5E-03
KIRC	HPN_RI_2.2_2.1_2.3	HPN	RI	2.2	2.1	2.3	5.2E-30	0.276	-0.041	8.8E-05	6.0E-05	2.3E-04
KIRC	HPS4_RI_10.2_10.1_10.3	HPS4	RI	10.2	10.1	10.3	1.1E-10	0.112	0.290	2.6E-03	2.2E-03	2.5E-01
KIRC	HPS4_RI_10.2:10.3:10.4_10.1_10.5	HPS4	RI	10.2:10.3:10.4	10.1	10.5	7.9E-34	0.303	0.356	4.9E-06	4.2E-06	7.9E-02
KIRC	HPS4_RI_10.4_10.3_10.5	HPS4	RI	10.4	10.3	10.5	7.5E-11	0.108	0.251	4.9E-05	1.6E-02	2.4E-01
KIRC	HRAS_ES_6_5_7.1	HRAS	ES	6	5	7.1	1.6E-13	0.132	0.088	8.0E-01	1.0E+00	9.9E-01
KIRC	HRSP12_ES_2.2_1_3	HRSP12	ES	2.2	1	3	5.7E-08	0.101	0.047	9.4E-02	2.3E-01	2.0E-01
KIRC	HSD11B1L_ES_2:3_1_4.2	HSD11B1L	ES	2:03	1	4.2	4.7E-24	0.233	-0.190	2.4E-04	1.7E-04	3.0E-02
KIRC	HSD17B7_AA_5.1_4_5.2	HSD17B7	AA	5.1	4	5.2	1.5E-23	0.223	-0.179	4.0E-04	3.8E-05	1.0E-02
KIRC	HSF4_RI_14.2_14.1_14.3	HSF4	RI	14.2	14.1	14.3	4.4E-12	0.119	0.310	4.3E-03	1.8E-05	2.9E-03
KIRC	HYAL2_RI_1.2_1.1_1.3	HYAL2	RI	1.2	1.1	1.3	3.4E-10	0.109	-0.154	5.1E-04	5.4E-03	1.8E-01
KIRC	HYAL2_RI_1.4_1.3_1.5	HYAL2	RI	1.4	1.3	1.5	4.6E-17	0.166	0.170	9.8E-02	3.9E-02	7.1E-01
KIRC	HYI_ES_3_2.1_4	HYI	ES	3	2.1	4	7.4E-11	0.109	-0.191	5.4E-02	7.9E-04	3.0E-02
KIRC	HYKK_ES_4_3_5.1	HYKK	ES	4	3	5.1	9.7E-12	0.117	-0.281	9.2E-03	5.3E-03	5.7E-01
KIRC	HYOU1_ES_18.1:18.2_17_19	HYOU1	ES	18.1:18.2	17	19	1.1E-13	0.142	-0.218	9.7E-03	1.1E-03	4.1E-03
KIRC	ICA1_ES_8_7_10	ICA1	ES	8	7	10	3.6E-16	0.162	-0.223	3.3E-06	1.2E-06	2.8E-03
KIRC	ICOSLG_RI_6.2_6.1_6.3	ICOSLG	RI	6.2	6.1	6.3	8.3E-12	0.117	-0.153	3.0E-03	6.4E-05	1.4E-05
KIRC	IDE_ES_2:3:4:5_1_6	IDE	ES	2:3:4:5	1	6	7.2E-21	0.218	-0.675	5.3E-04	6.4E-03	1.7E-02
KIRC	IDE_ES_22_21_23	IDE	ES	22	21	23	3.7E-17	0.176	-0.506	9.9E-03	1.4E-03	4.7E-03
KIRC	IFFO1_ES_3_1_4	IFFO1	ES	3	1	4	3.8E-17	0.169	0.582	1.4E-03	4.8E-05	7.8E-04
KIRC	IFNAR2_AA_10.1_9_10.2	IFNAR2	AA	10.1	9	10.2	1.1E-21	0.205	-0.127	1.5E-03	1.0E-02	1.4E-02

cancer type	id	Gene Symbol	splice_type	Exon	From.Exon	To.Exon	anova.p	adj.r2	r	p.50	p.25	p.10
KIRC	IFT122_ES_4:5:7_3_8.2	IFT122	ES	4:05:07	3	8.2	5.2E-17	0.194	-0.098	1.2E-01	2.7E-02	9.4E-04
KIRC	IFT122_ES_5:6:7_3_8.2	IFT122	ES	5:06:07	3	8.2	3.7E-17	0.188	-0.162	1.2E-02	5.8E-02	4.7E-01
KIRC	IFT122_ES_6_5_7	IFT122	ES	6	5	7	2.6E-26	0.260	-0.002	7.3E-05	2.4E-04	5.3E-03
KIRC	IFT88_ES_2.1:2.2:2.3_1_3	IFT88	ES	2.1:2.2:2.3	1	3	1.6E-15	0.155	-0.177	8.3E-06	5.1E-04	2.5E-03
KIRC	IK_ES_3_2_4	IK	ES	3	2	4	2.3E-27	0.252	-0.139	8.6E-07	1.1E-07	1.3E-02
KIRC	IL15_ES_6_5_7	IL15	ES	6	5	7	2.3E-13	0.132	0.104	2.3E-04	1.9E-03	4.3E-02
KIRC	IL17RC_AD_8.2_8.1_9.1	IL17RC	AD	8.2	8.1	9.1	4.3E-28	0.259	0.096	4.7E-05	4.7E-05	2.3E-04
KIRC	IL18BP_AD_1.2:1.3:1.4:1.5:1.6_1.1_1.8	IL18BP	AD	1.2:1.3:1.4:1.5:1.[illegible]	1.1	1.8	9.3E-12	0.117	0.134	1.4E-06	1.0E-06	6.3E-05
KIRC	IL18BP_RI_1.2:1.3:1.4:1.5:1.6:1.7_1.1_1.	IL18BP	RI	2:1.3:1.4:1.5:1.6:[illegible]	1.1	1.8	4.7E-16	0.159	0.007	5.4E-06	1.1E-07	4.1E-06
KIRC	IL18BP_RI_1.7_1.6_1.8	IL18BP	RI	1.7	1.6	1.8	5.0E-16	0.156	-0.477	4.9E-01	2.6E-01	9.6E-02
KIRC	IL32_RI_1.2_1.1_1.3	IL32	RI	1.2	1.1	1.3	1.6E-10	0.104	-0.005	1.6E-01	6.4E-02	6.5E-03
KIRC	IL4R_ES_4_3.2_5	IL4R	ES	4	3.2	5	4.8E-14	0.139	0.283	1.6E-02	6.2E-03	4.8E-04
KIRC	ILK_ES_2:3_1.4_4	ILK	ES	2:03	1.4	4	2.9E-14	0.146	0.013	5.1E-02	1.3E-02	8.7E-03
KIRC	IMPA1_ES_7_6.2_8	IMPA1	ES	7	6.2	8	1.2E-38	0.341	-0.134	7.3E-03	4.0E-02	6.9E-01
KIRC	ING3_RI_4.2_4.1_4.3	ING3	RI	4.2	4.1	4.3	1.2E-19	0.200	-0.012	2.0E-02	8.3E-03	2.3E-02
KIRC	INO80C_ES_3:4.1:4.2_1_5.1	INO80C	ES	3:4.1:4.2	1	5.1	8.6E-26	0.240	0.078	5.8E-09	1.3E-07	2.7E-06
KIRC	INO80E_AA_6.1_5_6.2	INO80E	AA	6.1	5	6.2	1.3E-28	0.263	0.024	5.2E-01	8.1E-01	6.4E-01
KIRC	INO80E_ES_6.1:6.2:6.3:7:8:9:10_5_11	INO80E	ES	.1:6.2:6.3:7:8:9:1	5	11	7.8E-22	0.211	0.024	1.4E-01	1.0E-01	1.2E-01
KIRC	INO80E_ES_6.2:6.3_5_11	INO80E	ES	6.2:6.3	5	11	2.9E-11	0.111	0.020	9.2E-01	2.7E-01	4.0E-01
KIRC	INO80E_ES_6.2:6.3:7:8:9:10_5_11	INO80E	ES	6.2:6.3:7:8:9:10	5	11	1.1E-11	0.117	0.074	1.0E-01	2.4E-02	3.1E-02
KIRC	INO80E_ES_7:8:9_6.3_10	INO80E	ES	7:08:09	6.3	10	1.5E-12	0.130	-0.033	4.7E-01	9.0E-01	5.0E-01
KIRC	INO80E_ES_7:8:9:10_6.3_11	INO80E	ES	7:8:9:10	6.3	11	4.6E-21	0.201	0.032	8.1E-01	1.4E-01	8.1E-02
KIRC	INPP5B_AA_16.1_15_16.2	INPP5B	AA	16.1	15	16.2	8.1E-14	0.145	0.383	1.4E-01	1.3E-02	7.7E-03
KIRC	INPP5F_RI_5.2_5.1_5.3	INPP5F	RI	5.2	5.1	5.3	3.0E-25	0.247	-0.397	5.4E-04	1.9E-03	5.6E-04
KIRC	INTS3_RI_27.2_27.1_27.3	INTS3	RI	27.2	27.1	27.3	2.1E-20	0.194	0.569	9.3E-03	2.7E-02	1.1E-01
KIRC	INTS8_AA_8.1_7_8.2	INTS8	AA	8.1	7	8.2	4.8E-14	0.141	-0.158	4.3E-03	8.1E-02	3.4E-04
KIRC	INTU_AA_10.1:10.2_9_10.3	INTU	AA	10.1:10.2	9	10.3	1.3E-11	0.114	0.246	1.7E-04	1.1E-02	6.5E-03
KIRC	IP6K2_AD_11.3:11.4:11.5_11.2_11.9	IP6K2	AD	11.3:11.4:11.5	11.2	11.9	3.7E-10	0.100	0.204	1.6E-03	2.0E-04	7.8E-03
KIRC	IP6K2_ES_11.4:11.5_11.2_11.9	IP6K2	ES	11.4:11.5	11.2	11.9	6.1E-10	0.101	-0.198	4.1E-01	9.2E-01	3.1E-02
KIRC	IP6K2_RI_11.3_11.2_11.4	IP6K2	RI	11.3	11.2	11.4	9.4E-12	0.116	0.353	1.9E-03	1.0E-01	5.6E-01
KIRC	IP6K2_RI_11.3:11.4:11.5:11.6:11.7:11.8_	IP6K2	RI	1.4:11.5:11.6:11.	11.2	11.9	8.7E-20	0.190	0.440	3.2E-04	1.0E-04	9.5E-02
KIRC	IP6K2_RI_11.6:11.7:11.8_11.5_11.9	IP6K2	RI	11.6:11.7:11.8	11.5	11.9	7.1E-12	0.118	0.380	1.2E-02	5.3E-03	3.1E-01
KIRC	IQCC_AD_1.2_1.1_2	IQCC	AD	1.2	1.1	2	6.0E-17	0.191	0.324	1.5E-03	4.1E-05	1.1E-03
KIRC	IQCK_RI_6.2_6.1_6.3	IQCK	RI	6.2	6.1	6.3	9.8E-23	0.215	0.035	7.6E-06	2.1E-06	7.7E-04
KIRC	IRAK4_ES_4_3_5	IRAK4	ES	4	3	5	9.6E-15	0.181	0.155	1.4E-02	2.8E-02	6.3E-02
KIRC	IRAK4_ES_4:5_3_6	IRAK4	ES	4:05	3	6	1.9E-16	0.170	0.076	2.0E-05	1.4E-04	1.6E-04
KIRC	IREB2_RI_8.2_8.1_8.3	IREB2	RI	8.2	8.1	8.3	3.7E-37	0.327	-0.690	4.6E-05	1.6E-06	6.6E-03
KIRC	IRF3_AD_5.2_5.1_6.2	IRF3	AD	5.2	5.1	6.2	4.5E-24	0.225	0.400	1.6E-02	2.6E-02	1.3E-01
KIRC	IRF3_RI_1.2:1.3_1.1_1.4	IRF3	RI	1.2:1.3	1.1	1.4	1.0E-24	0.231	0.564	5.2E-04	4.2E-04	2.4E-03
KIRC	IRF3_RI_1.2:1.3:1.4_1.1_1.5	IRF3	RI	1.2:1.3:1.4	1.1	1.5	1.5E-20	0.239	0.479	3.3E-02	2.8E-02	6.6E-01

cancer type	id	Gene Symbol	splice_type	Exon	From.Exon	To.Exon	anova.p	adj.r2	r	p.50	p.25	p.10
KIRC	IRF5_ES_5_4_6.1	IRF5	ES	5	4	6.1	3.8E-19	0.186	0.021	7.4E-04	1.4E-03	1.0E-02
KIRC	IRF7_RI_5.2_5.1_5.3	IRF7	RI	5.2	5.1	5.3	5.5E-15	0.147	0.205	2.9E-02	4.2E-02	1.4E-01
KIRC	ISCU_RI_5.2_5.1_5.3	ISCU	RI	5.2	5.1	5.3	1.0E-12	0.127	-0.143	4.5E-04	7.9E-05	5.7E-04
KIRC	ISOC2_ES_3:4.1_2_4.2	ISOC2	ES	03:04.1	2	4.2	3.4E-10	0.101	0.119	2.4E-04	7.4E-04	1.1E-03
KIRC	IST1_ES_12:13_11_14.1	IST1	ES	12:13	11	14.1	8.0E-20	0.190	-0.159	3.1E-06	3.0E-04	8.7E-03
KIRC	IST1_ES_13_12_14.1	IST1	ES	13	12	14.1	9.8E-34	0.302	-0.140	1.1E-06	4.2E-06	6.2E-03
KIRC	IST1_ES_13:14.1_12_14.2	IST1	ES	13:14.1	12	14.2	1.0E-11	0.116	-0.203	5.9E-05	4.9E-04	1.9E-01
KIRC	ISY1_ES_9_8_10.1	ISY1	ES	9	8	10.1	1.5E-39	0.343	-0.379	3.6E-04	1.0E-04	7.6E-03
KIRC	ITCH_ES_7_6_8	ITCH	ES	7	6	8	4.4E-24	0.227	-0.586	1.7E-08	9.0E-08	1.9E-03
KIRC	ITGA6_ES_27_26_28	ITGA6	ES	27	26	28	1.5E-10	0.104	-0.165	1.0E-01	4.2E-03	3.0E-03
KIRC	ITGA7_ES_9_8_10	ITGA7	ES	9	8	10	9.4E-12	0.125	0.078	1.2E-02	1.1E-03	3.0E-02
KIRC	ITGAE_ES_28_27_29	ITGAE	ES	28	27	29	6.0E-12	0.118	0.301	5.2E-03	3.1E-05	9.4E-02
KIRC	ITGB1BP1_AA_8.1:8.2_7_8.3	ITGB1BP1	AA	8.1:8.2	7	8.3	4.6E-12	0.118	-0.067	4.5E-01	7.5E-02	6.7E-02
KIRC	IVNS1ABP_ES_9_8_10	IVNS1ABP	ES	9	8	10	5.7E-30	0.273	-0.024	1.2E-04	2.2E-05	2.3E-04
KIRC	JMJD6_ES_5.1:5.2_4_6	JMJD6	ES	5.1:5.2	4	6	2.9E-13	0.130	-0.036	5.1E-01	1.5E-01	8.9E-02
KIRC	KCNAB2_RI_22.2_22.1_22.3	KCNAB2	RI	22.2	22.1	22.3	9.6E-27	0.250	0.033	1.6E-03	2.4E-04	1.9E-03
KIRC	KCTD6_ES_2_1_3.2	KCTD6	ES	2	1	3.2	8.0E-21	0.208	0.292	3.4E-02	6.4E-03	4.8E-03
KIRC	KCTD7_ES_13_12_14	KCTD7	ES	13	12	14	8.9E-32	0.288	-0.121	1.3E-02	1.1E-03	2.8E-03
KIRC	KDM4C_ES_12_11_13	KDM4C	ES	12	11	13	1.5E-25	0.246	0.055	4.9E-03	1.7E-03	3.9E-03
KIRC	KDM5B_ES_6_5_7	KDM5B	ES	6	5	7	4.7E-34	0.304	-0.498	3.6E-04	4.9E-05	3.2E-02
KIRC	KDM5C_ES_4_3_5	KDM5C	ES	4	3	5	1.0E-17	0.171	-0.127	3.6E-04	8.8E-04	5.0E-02
KIRC	KHK_ES_4_3_5	KHK	ES	4	3	5	7.8E-11	0.121	0.268	3.6E-04	7.7E-02	4.5E-01
KIRC	KHK_ME_3|4_2_5	KHK	ME	3|4	2	5	4.4E-25	0.234	-0.587	7.7E-06	1.0E-04	1.9E-02
KIRC	KIAA0907_RI_13.2_13.1_13.3	KIAA0907	RI	13.2	13.1	13.3	9.0E-20	0.189	0.727	1.6E-05	5.3E-06	1.3E-03
KIRC	KIAA1217_ES_12_11_13	KIAA1217	ES	12	11	13	6.9E-24	0.226	0.130	1.1E-06	5.5E-10	2.1E-07
KIRC	KIAA1407_AD_13.2_13.1_14	KIAA1407	AD	13.2	13.1	14	1.5E-12	0.127	0.282	1.2E-01	1.0E-02	9.7E-03
KIRC	KIAA1468_ME_24|25_23_26	KIAA1468	ME	24|25	23	26	1.4E-13	0.133	0.074	3.5E-03	2.8E-05	9.6E-03
KIRC	KIDINS220_ES_3_2_4	KIDINS220	ES	3	2	4	8.2E-22	0.219	-0.608	7.1E-04	8.4E-04	5.4E-02
KIRC	KIF12_ES_15_14_16	KIF12	ES	15	14	16	2.2E-14	0.141	0.127	8.6E-06	7.1E-08	2.0E-05
KIRC	KIF13A_ES_40_39_41.1	KIF13A	ES	40	39	41.1	1.9E-11	0.113	-0.070	1.3E-02	1.8E-04	1.2E-01
KIRC	KIF3C_ES_6_5_8	KIF3C	ES	6	5	8	1.5E-17	0.175	-0.600	8.5E-02	1.0E-01	1.3E-02
KIRC	KLC1_RI_14.2_14.1_14.3	KLC1	RI	14.2	14.1	14.3	6.4E-13	0.127	0.079	4.1E-01	9.9E-01	4.7E-01
KIRC	KLC2_RI_16.2_16.1_16.3	KLC2	RI	16.2	16.1	16.3	6.4E-24	0.226	0.137	2.9E-03	2.7E-04	3.9E-02
KIRC	KLF8_ES_5_4_6	KLF8	ES	5	4	6	1.1E-13	0.145	0.401	7.9E-02	1.3E-01	1.6E-01
KIRC	KLHDC1_ES_2_1_3	KLHDC1	ES	2	1	3	6.0E-22	0.222	0.014	9.0E-09	2.4E-10	1.7E-05
KIRC	KLHDC2_RI_7.2_7.1_7.3	KLHDC2	RI	7.2	7.1	7.3	4.5E-39	0.341	-0.147	3.1E-06	2.1E-06	1.8E-05
KIRC	KLHDC4_ES_5_4.2_6.1	KLHDC4	ES	5	4.2	6.1	2.9E-17	0.167	0.203	2.3E-04	7.9E-06	2.1E-04
KIRC	KLHDC4_ES_5:6.1:6.2_4.2_7.1	KLHDC4	ES	5:6.1:6.2	4.2	7.1	7.9E-09	0.112	0.204	4.2E-03	1.2E-01	1.6E-01
KIRC	KLHL21_RI_4.2:4.3_4.1_4.4	KLHL21	RI	4.2:4.3	4.1	4.4	9.4E-28	0.255	-0.163	1.0E-06	4.6E-07	1.6E-02
KIRC	KLHL42_ES_3_2_4	KLHL42	ES	3	2	4	2.9E-19	0.189	-0.075	1.2E-05	9.2E-06	7.1E-05

cancer type	id	Gene Symbol	splice_type	Exon	From.Exon	To.Exon	anova.p	adj.r2	r	p.50	p.25	p.10
KIRC	KPNA1_ES_6_5_7	KPNA1	ES	6	5	7	1.1E-29	0.276	-0.095	1.5E-03	2.1E-03	2.0E-01
KIRC	KRT15_RI_7.2_7.1_7.3	KRT15	RI	7.2	7.1	7.3	2.6E-18	0.176	-0.743	5.6E-08	1.7E-07	8.0E-05
KIRC	KSR1_AA_24.1_22_24.2	KSR1	AA	24.1	22	24.2	1.1E-21	0.206	0.414	1.9E-07	6.2E-09	1.0E-03
KIRC	KSR1_ES_13_12_15	KSR1	ES	13	12	15	1.3E-09	0.103	0.118	3.8E-03	1.2E-02	2.7E-02
KIRC	L3MBTL2_ES_2_1_3.1	L3MBTL2	ES	2	1	3.1	6.6E-21	0.199	0.014	4.9E-06	1.6E-04	4.8E-03
KIRC	LAIR1_AD_7.2_7.1_8	LAIR1	AD	7.2	7.1	8	1.4E-33	0.302	-0.024	1.5E-05	1.2E-06	6.7E-04
KIRC	LAMTOR5_RI_1.2_1.1_1.3	LAMTOR5	RI	1.2	1.1	1.3	6.5E-16	0.155	-0.248	6.8E-01	7.4E-01	8.4E-01
KIRC	LARP1B_RI_9.2_9.1_9.3	LARP1B	RI	9.2	9.1	9.3	5.5E-15	0.147	-0.062	2.1E-02	1.4E-03	5.1E-04
KIRC	LBH_ES_4_3.1_5	LBH	ES	4	3.1	5	3.1E-22	0.210	-0.219	4.6E-08	4.3E-07	1.3E-03
KIRC	LCK_AA_10.1_9_10.2	LCK	AA	10.1	9	10.2	1.9E-22	0.224	0.093	1.0E-03	5.4E-04	1.2E-03
KIRC	LCK_RI_4.2_4.1_4.3	LCK	RI	4.2	4.1	4.3	1.5E-22	0.227	0.013	3.5E-05	9.4E-05	1.6E-03
KIRC	LCLAT1_ES_3_2_4	LCLAT1	ES	3	2	4	2.4E-23	0.278	-0.376	2.6E-02	3.1E-03	6.3E-03
KIRC	LDLRAD3_ES_5_4_6	LDLRAD3	ES	5	4	6	5.9E-28	0.257	-0.540	3.1E-03	2.0E-03	8.3E-03
KIRC	LENG8_RI_15.2:15.3:15.4_15.1_15.5	LENG8	RI	15.2:15.3:15.4	15.1	15.5	4.9E-15	0.147	0.804	3.0E-03	3.5E-04	1.2E-01
KIRC	LEPRE1_RI_14.2:14.3_14.1_14.4	LEPRE1	RI	14.2:14.3	14.1	14.4	8.3E-27	0.248	-0.086	7.6E-03	2.5E-03	5.6E-02
KIRC	LEPRE1_RI_14.3_14.2_14.4	LEPRE1	RI	14.3	14.2	14.4	2.7E-20	0.195	-0.116	7.1E-03	2.0E-03	8.9E-02
KIRC	LETMD1_AD_1.2:1.3_1.1_2	LETMD1	AD	1.2:1.3	1.1	2	1.3E-17	0.193	-0.089	2.6E-03	4.6E-04	7.9E-06
KIRC	LETMD1_AD_3.3_3.2_4	LETMD1	AD	3.3	3.2	4	2.0E-27	0.256	-0.138	2.5E-04	2.4E-04	9.4E-04
KIRC	LETMD1_ES_1.3:2_1.2_7	LETMD1	ES	1.3:2	1.2	7	3.5E-19	0.223	-0.142	5.4E-11	2.5E-08	5.0E-05
KIRC	LETMD1_ES_1.3:2:3.2:4:5:6_1.2_7	LETMD1	ES	1.3:2:3.2:4:5:6	1.2	7	1.6E-13	0.145	-0.206	5.7E-07	3.1E-09	4.7E-07
KIRC	LETMD1_ES_1.3:2:4:5:6_1.2_7	LETMD1	ES	1.3:2:4:5:6	1.2	7	2.7E-14	0.176	-0.201	4.0E-09	5.2E-07	6.4E-05
KIRC	LETMD1_ES_2:3.2:3.3:4:5:6_1.2_7	LETMD1	ES	2:3.2:3.3:4:5:6	1.2	7	7.5E-17	0.169	-0.192	1.4E-07	4.6E-07	4.0E-05
KIRC	LETMD1_ES_3.2:3.3_2_4	LETMD1	ES	3.2:3.3	2	4	3.4E-21	0.209	-0.182	2.7E-07	2.6E-06	6.9E-05
KIRC	LETMD1_ES_3.2:3.3:4_2_5	LETMD1	ES	3.2:3.3:4	2	5	1.3E-12	0.133	-0.250	2.7E-03	2.0E-05	2.5E-03
KIRC	LETMD1_ES_3.2:3.3:4:5:6_2_7	LETMD1	ES	3.2:3.3:4:5:6	2	7	4.5E-20	0.192	-0.214	2.3E-06	6.2E-06	1.9E-04
KIRC	LETMD1_ES_3.3:4:5:6_3.2_7	LETMD1	ES	3.3:4:5:6	3.2	7	1.0E-21	0.210	-0.166	1.3E-03	7.3E-05	6.4E-03
KIRC	LETMD1_ES_8.1:8.2_7_9	LETMD1	ES	8.1:8.2	7	9	4.5E-14	0.154	-0.036	2.4E-02	8.9E-03	8.0E-03
KIRC	LGALS3BP_ES_2.3:2.4:2.5:3.1:3.2_2.2_4.	LGALS3BP	ES	2.3:2.4:2.5:3.1:3.2	2.2	4.1	6.3E-13	0.147	-0.070	2.7E-04	5.9E-03	3.4E-02
KIRC	LGALS8_ES_11_10.1_12	LGALS8	ES	11	10.1	12	2.4E-13	0.131	0.015	1.1E-10	2.3E-07	4.0E-06
KIRC	LGMN_ES_2_1_3	LGMN	ES	2	1	3	1.6E-28	0.262	-0.712	1.2E-03	1.2E-05	5.1E-04
KIRC	LIG1_AD_24.2_24.1_25	LIG1	AD	24.2	24.1	25	9.5E-22	0.206	0.226	4.1E-07	1.7E-05	4.3E-04
KIRC	LIMS2_ES_3:4.2_2_6	LIMS2	ES	03:04.2	2	6	2.3E-24	0.252	0.137	1.6E-03	2.7E-06	1.9E-04
KIRC	LIN37_RI_6.2_6.1_6.3	LIN37	RI	6.2	6.1	6.3	1.4E-22	0.214	0.079	8.6E-03	5.0E-04	2.4E-02
KIRC	LIPT1_ES_3:5_1_6	LIPT1	ES	3:05	1	6	4.9E-10	0.113	0.006	4.5E-02	4.2E-02	4.5E-01
KIRC	LIPT1_ES_5_3_6	LIPT1	ES	5	3	6	7.3E-17	0.189	0.046	6.6E-03	6.4E-03	3.5E-02
KIRC	LMBR1L_ES_4.1:4.2_3_5	LMBR1L	ES	4.1:4.2	3	5	1.7E-19	0.189	0.632	1.7E-02	3.8E-03	3.6E-02
KIRC	LMF2_AA_2.1_1_2.2	LMF2	AA	2.1	1	2.2	5.5E-31	0.283	0.290	4.6E-06	3.3E-07	1.3E-03
KIRC	LMO7_AA_19.1_18_19.2	LMO7	AA	19.1	18	19.2	3.3E-11	0.113	-0.108	4.0E-01	5.6E-02	4.2E-02
KIRC	LMO7_ES_10:11:12_9_13	LMO7	ES	10:11:12	9	13	3.4E-09	0.102	-0.195	6.5E-09	1.9E-08	1.3E-05
KIRC	LMO7_ES_12_9_13	LMO7	ES	12	9	13	2.9E-20	0.206	-0.322	8.7E-04	1.7E-06	1.7E-03

cancer type	id	Gene Symbol	splice_type	Exon	From.Exon	To.Exon	anova.p	adj.r2	r	p.50	p.25	p.10
KIRC	LPAR2_ES_3:4_2.2_5.2	LPAR2	ES	3:04	2.2	5.2	9.0E-17	0.169	0.047	2.2E-07	2.0E-07	8.3E-04
KIRC	LPXN_ES_6_5_7	LPXN	ES	6	5	7	7.5E-29	0.264	-0.132	5.7E-03	1.6E-04	1.4E-01
KIRC	LRCH3_ES_21_20_22.1	LRCH3	ES	21	20	22.1	3.2E-12	0.123	-0.023	2.7E-02	4.6E-02	2.8E-01
KIRC	LRRFIP2_ES_7:8_5_18	LRRFIP2	ES	7:08	5	18	3.1E-18	0.193	-0.147	1.2E-04	5.2E-05	1.6E-03
KIRC	LRTOMT_RI_6.2_6.1_6.3	LRTOMT	RI	6.2	6.1	6.3	4.7E-20	0.192	-0.165	1.5E-02	2.4E-03	1.4E-02
KIRC	LSM12_ES_6_5_7	LSM12	ES	6	5	7	3.2E-33	0.298	-0.008	4.3E-03	7.1E-03	5.2E-02
KIRC	LTBR_ES_8:9.1_7_10	LTBR	ES	08:09.1	7	10	1.6E-15	0.151	-0.017	1.1E-01	5.8E-01	2.0E-01
KIRC	LUC7L_AD_1.2:1.3:1.4_1.1_2.2	LUC7L	AD	1.2:1.3:1.4	1.1	2.2	1.8E-20	0.195	0.714	2.2E-04	4.4E-03	3.6E-01
KIRC	LUC7L_ES_1.3:1.4_1.1_2.2	LUC7L	ES	1.3:1.4	1.1	2.2	8.7E-22	0.210	0.649	1.1E-02	1.4E-01	3.0E-01
KIRC	LUC7L_ES_4_3_5	LUC7L	ES	4	3	5	1.2E-22	0.214	0.617	3.2E-02	1.6E-01	2.9E-01
KIRC	LUC7L3_RI_12.3:12.4:12.5_12.2_12.6	LUC7L3	RI	12.3:12.4:12.5	12.2	12.6	1.3E-17	0.170	0.757	1.4E-03	1.3E-02	3.0E-02
KIRC	LYPLA2_RI_8.2:8.3_8.1_8.4	LYPLA2	RI	8.2:8.3	8.1	8.4	4.0E-10	0.102	-0.055	3.2E-04	5.2E-04	6.2E-02
KIRC	MACF1_ES_103_102_104	MACF1	ES	103	102	104	1.1E-11	0.115	-0.099	1.7E-03	5.2E-03	4.0E-03
KIRC	MACF1_ES_107_106_108	MACF1	ES	107	106	108	1.1E-11	0.115	-0.067	5.6E-04	1.3E-02	5.3E-03
KIRC	MAGED2_RI_14.2_14.1_14.3	MAGED2	RI	14.2	14.1	14.3	2.1E-10	0.102	-0.174	1.2E-02	9.0E-02	7.1E-01
KIRC	MAGI3_ES_8_7_9	MAGI3	ES	8	7	9	8.0E-27	0.305	-0.508	3.9E-07	6.9E-08	4.5E-03
KIRC	MAN2A2_AA_17.1_16_17.2	MAN2A2	AA	17.1	16	17.2	4.2E-22	0.212	0.354	2.0E-02	6.8E-02	7.0E-02
KIRC	MAN2C1_AA_19.1_18.2_19.2	MAN2C1	AA	19.1	18.2	19.2	7.7E-28	0.257	0.680	3.2E-04	5.6E-04	1.2E-02
KIRC	MANBA_ES_2_1_3	MANBA	ES	2	1	3	2.2E-14	0.143	-0.439	3.9E-01	2.9E-01	4.6E-01
KIRC	MAP2K7_ES_2_1_3	MAP2K7	ES	2	1	3	1.4E-13	0.147	-0.180	9.6E-04	1.7E-04	2.9E-05
KIRC	MAP3K4_ES_24_23_25	MAP3K4	ES	24	23	25	5.1E-13	0.129	-0.017	1.7E-02	2.2E-03	1.4E-04
KIRC	MAP3K4_ES_4_3_5	MAP3K4	ES	4	3	5	2.4E-28	0.280	-0.317	3.6E-07	3.8E-08	5.5E-03
KIRC	MAP4K1_ES_31_30_32	MAP4K1	ES	31	30	32	1.3E-10	0.104	-0.424	6.1E-01	9.8E-01	1.5E-01
KIRC	MAPK10_ME_12|13_11_14	MAPK10	ME	12|13	11	14	1.8E-14	0.143	0.056	7.5E-02	3.8E-04	5.2E-03
KIRC	MAPK11_RI_7.2_7.1_7.3	MAPK11	RI	7.2	7.1	7.3	3.9E-14	0.140	0.210	9.1E-07	1.2E-05	6.1E-03
KIRC	MAPK3_ES_6.1:6.2_5_7.1	MAPK3	ES	6.1:6.2	5	7.1	1.6E-11	0.129	-0.208	6.6E-01	1.8E-01	3.3E-01
KIRC	MAPK9_ES_3_2_4	MAPK9	ES	3	2	4	1.3E-10	0.106	-0.202	4.4E-04	1.8E-04	6.3E-03
KIRC	MARCH8_ES_7_6_8	MARCH8	ES	7	6	8	2.7E-10	0.102	-0.366	2.9E-01	3.8E-02	4.8E-03
KIRC	MARS_RI_20.2_20.1_20.3	MARS	RI	20.2	20.1	20.3	2.0E-25	0.237	0.206	1.6E-03	1.9E-03	4.6E-02
KIRC	MATR3_AD_7.2_7.1_9	MATR3	AD	7.2	7.1	9	4.7E-11	0.118	0.128	3.6E-03	1.2E-01	7.4E-02
KIRC	MATR3_ES_7.2:8_7.1_9	MATR3	ES	7.2:8	7.1	9	9.6E-09	0.101	0.014	6.4E-01	1.4E-01	5.3E-01
KIRC	MAX_RI_5.2:5.3:5.4_5.1_5.5	MAX	RI	5.2:5.3:5.4	5.1	5.5	5.8E-22	0.209	0.062	6.1E-04	2.2E-03	6.0E-02
KIRC	MAX_RI_5.6:5.7_5.5_5.8	MAX	RI	5.6:5.7	5.5	5.8	3.4E-14	0.139	0.061	4.7E-05	6.2E-04	8.3E-03
KIRC	MAX_RI_5.7_5.6_5.8	MAX	RI	5.7	5.6	5.8	2.5E-13	0.131	0.050	7.9E-03	8.4E-04	5.5E-01
KIRC	MBD1_RI_18.2:18.3_18.1_18.4	MBD1	RI	18.2:18.3	18.1	18.4	2.4E-15	0.149	0.045	4.7E-04	2.9E-03	7.2E-01
KIRC	MBNL1_ES_3:4_1_5	MBNL1	ES	3:04	1	5	1.8E-14	0.153	-0.250	3.2E-04	8.7E-03	1.6E-03
KIRC	MBTD1_RI_16.2_16.1_16.3	MBTD1	RI	16.2	16.1	16.3	8.0E-24	0.234	0.101	3.6E-05	1.4E-04	1.2E-02
KIRC	MCCC2_ES_11.2:12.1_11.1_12.2	MCCC2	ES	11.2:12.1	11.1	12.2	9.8E-26	0.241	-0.244	3.4E-04	1.1E-05	1.1E-03
KIRC	MCFD2_ES_5_3_6	MCFD2	ES	5	3	6	5.0E-35	0.311	-0.700	4.2E-06	5.2E-07	1.2E-03
KIRC	MCM7_RI_5.2_5.1_5.3	MCM7	RI	5.2	5.1	5.3	3.7E-40	0.348	0.144	3.0E-06	2.2E-05	1.1E-05

cancer type	id	Gene Symbol	splice_type	Exon	From.Exon	To.Exon	anova.p	adj.r2	r	p.50	p.25	p.10
KIRC	MCM8_ME_11|12_10.2_13	MCM8	ME	11|12	10.2	13	2.4E-22	0.219	0.236	1.2E-02	3.2E-01	2.4E-01
KIRC	ME3_ES_10_8_11	ME3	ES	10	8	11	2.9E-25	0.236	0.434	4.3E-04	1.4E-04	1.6E-02
KIRC	MED1_ES_3_2_4	MED1	ES	3	2	4	5.2E-28	0.267	-0.689	5.5E-08	1.1E-08	1.2E-03
KIRC	MEFV_ES_6_5_7	MEFV	ES	6	5	7	3.7E-13	0.129	-0.493	1.2E-01	2.0E-01	6.7E-01
KIRC	MEGF8_ES_30_29_31	MEGF8	ES	30	29	31	5.4E-11	0.111	-0.223	2.0E-02	2.0E-02	3.3E-02
KIRC	MEIS1_AA_12.3:12.4_12.1_12.5	MEIS1	AA	12.3:12.4	12.1	12.5	1.6E-11	0.114	0.237	2.1E-05	9.6E-05	6.7E-04
KIRC	MEIS1_AD_12.2:12.3_12.1_12.5	MEIS1	AD	12.2:12.3	12.1	12.5	1.3E-10	0.111	0.239	8.2E-03	7.4E-03	6.1E-03
KIRC	MEIS1_RI_12.2_12.1_12.3	MEIS1	RI	12.2	12.1	12.3	9.6E-17	0.169	0.286	4.0E-04	1.3E-03	6.5E-02
KIRC	MEIS1_RI_12.2:12.3:12.4_12.1_12.5	MEIS1	RI	12.2:12.3:12.4	12.1	12.5	1.0E-17	0.176	0.162	1.9E-06	5.1E-05	2.8E-04
KIRC	MEIS1_RI_12.4_12.3_12.5	MEIS1	RI	12.4	12.3	12.5	2.4E-15	0.150	0.170	3.9E-08	3.5E-07	2.7E-03
KIRC	MEIS3_AA_7.1_6_7.2	MEIS3	AA	7.1	6	7.2	3.1E-18	0.180	0.668	1.3E-06	1.2E-06	9.6E-05
KIRC	METTL17_AA_4.1_3_4.2	METTL17	AA	4.1	3	4.2	4.2E-20	0.192	0.514	8.7E-04	2.2E-02	6.5E-02
KIRC	METTL17_RI_10.2_10.1_10.3	METTL17	RI	10.2	10.1	10.3	8.2E-22	0.207	0.495	2.1E-04	1.7E-04	1.3E-02
KIRC	METTL17_RI_6.2_6.1_6.3	METTL17	RI	6.2	6.1	6.3	2.4E-28	0.260	0.374	6.1E-04	8.1E-05	5.2E-03
KIRC	METTL17_RI_8.2_8.1_8.3	METTL17	RI	8.2	8.1	8.3	8.4E-31	0.280	0.541	2.2E-07	1.3E-05	4.9E-04
KIRC	METTL17_RI_9.2_9.1_9.3	METTL17	RI	9.2	9.1	9.3	3.7E-32	0.290	0.498	4.6E-05	6.0E-04	4.3E-02
KIRC	METTL21B_ES_5_4_6	METTL21B	ES	5	4	6	2.5E-10	0.108	-0.030	5.8E-04	1.4E-03	1.7E-02
KIRC	METTL22_ES_4_3.4_5	METTL22	ES	4	3.4	5	1.1E-10	0.108	-0.119	6.8E-01	7.3E-01	8.5E-01
KIRC	METTL3_RI_8.2_8.1_8.3	METTL3	RI	8.2	8.1	8.3	4.3E-22	0.209	0.606	8.8E-04	3.4E-03	2.6E-02
KIRC	METTL3_RI_8.4_8.3_8.5	METTL3	RI	8.4	8.3	8.5	3.5E-26	0.243	0.551	1.1E-05	1.2E-04	9.5E-04
KIRC	MFSD10_RI_12.2_12.1_12.3	MFSD10	RI	12.2	12.1	12.3	5.2E-13	0.128	0.150	7.7E-04	2.0E-04	1.0E-03
KIRC	MFSD11_RI_2.2:2.3:2.4:2.5_2.1_2.6	MFSD11	RI	2.2:2.3:2.4:2.5	2.1	2.6	3.7E-11	0.110	-0.153	5.9E-04	3.5E-02	4.3E-02
KIRC	MFSD11_RI_2.5_2.4_2.6	MFSD11	RI	2.5	2.4	2.6	5.3E-11	0.108	-0.183	2.9E-02	1.2E-03	2.1E-01
KIRC	MFSD12_ES_14_12_15	MFSD12	ES	14	12	15	5.0E-16	0.155	-0.392	1.5E-04	1.1E-02	7.9E-01
KIRC	MGRN1_AA_17.1:17.2_16_17.3	MGRN1	AA	17.1:17.2	16	17.3	2.1E-17	0.168	0.116	2.2E-03	1.4E-05	1.2E-02
KIRC	MGRN1_ES_17.1_16_17.3	MGRN1	ES	17.1	16	17.3	3.0E-11	0.111	0.065	9.9E-02	1.3E-01	2.0E-01
KIRC	MGRN1_RI_17.2_17.1_17.3	MGRN1	RI	17.2	17.1	17.3	3.1E-23	0.218	0.072	3.7E-04	2.4E-04	3.1E-02
KIRC	MIS18BP1_ES_8_7_9	MIS18BP1	ES	8	7	9	4.1E-21	0.208	-0.500	1.6E-03	5.0E-05	2.4E-04
KIRC	MKNK1_ES_4_3_5	MKNK1	ES	4	3	5	2.0E-17	0.170	0.518	4.6E-04	1.1E-03	5.4E-03
KIRC	MKNK2_AA_14.1_13.1_14.2	MKNK2	AA	14.1	13.1	14.2	6.2E-13	0.127	-0.022	4.9E-04	6.1E-05	8.3E-04
KIRC	MKS1_AA_6.1_5_6.2	MKS1	AA	6.1	5	6.2	3.0E-10	0.106	0.060	4.7E-02	6.9E-02	8.5E-02
KIRC	MLH3_ES_7_6_8	MLH3	ES	7	6	8	3.0E-21	0.202	-0.145	7.1E-09	1.3E-08	6.4E-07
KIRC	MLH3_ES_7:8_6_9	MLH3	ES	7:08	6	9	7.1E-12	0.117	-0.025	2.3E-03	2.7E-07	3.5E-05
KIRC	MLXIPL_RI_16.2_16.1_16.3	MLXIPL	RI	16.2	16.1	16.3	1.1E-11	0.122	0.052	8.5E-05	1.0E-04	1.5E-02
KIRC	MMAB_ES_5_4_6	MMAB	ES	5	4	6	1.5E-24	0.230	-0.083	2.1E-04	2.2E-06	3.0E-06
KIRC	MMP19_RI_6.2:6.3_6.1_6.4	MMP19	RI	6.2:6.3	6.1	6.4	5.9E-11	0.123	0.096	8.7E-01	2.7E-01	4.2E-01
KIRC	MND1_ES_5:6_4_7	MND1	ES	5:06	4	7	4.4E-15	0.154	-0.262	1.7E-01	2.1E-02	4.0E-01
KIRC	MOK_RI_15.2:15.3:15.4_15.1_15.5	MOK	RI	15.2:15.3:15.4	15.1	15.5	9.8E-18	0.173	0.568	5.7E-03	9.1E-02	3.6E-01
KIRC	MON2_ES_31_30_32	MON2	ES	31	30	32	3.8E-10	0.101	-0.117	7.0E-03	2.1E-02	1.7E-01
KIRC	MORF4L2_AA_6.1_3.2_6.2	MORF4L2	AA	6.1	3.2	6.2	5.3E-29	0.270	0.001	9.1E-08	5.8E-09	4.0E-06

cancer type	id	Gene Symbol	splice_type	Exon	From.Exon	To.Exon	anova.p	adj.r2	r	p.50	p.25	p.10
KIRC	MPPE1_ES_12_11_13	MPPE1	ES	12	11	13	2.8E-12	0.121	-0.165	3.3E-03	1.0E-03	1.0E-02
KIRC	MPV17_AA_6.1:6.2_3.2_6.3	MPV17	AA	6.1:6.2	3.2	6.3	1.7E-14	0.145	-0.154	5.2E-02	1.6E-02	1.6E-02
KIRC	MPV17_ES_6.1:6.2:6.3_3.2_7	MPV17	ES	6.1:6.2:6.3	3.2	7	1.4E-13	0.156	-0.086	1.5E-01	1.2E-01	3.2E-02
KIRC	MRPL35_RI_4.2:4.3_4.1_4.4	MRPL35	RI	4.2:4.3	4.1	4.4	7.5E-16	0.154	0.136	2.6E-02	4.6E-03	3.8E-02
KIRC	MRPL45_ES_4_3_5	MRPL45	ES	4	3	5	6.5E-36	0.318	-0.567	6.3E-07	2.3E-05	3.2E-03
KIRC	MRPL52_RI_1.3_1.2_1.4	MRPL52	RI	1.3	1.2	1.4	1.0E-12	0.127	-0.235	4.7E-01	9.3E-01	6.5E-01
KIRC	MRPL55_AA_2.2:2.3:2.4:2.5:2.6:2.7:2.8_	MRPL55	AA	2.3:2.4:2.5:2.6:2.7	1.1	2.9	7.9E-11	0.111	-0.152	3.9E-03	1.8E-02	1.4E-01
KIRC	MRPL55_AA_2.2:2.3:2.4:2.5:2.6:2.7:2.8_	MRPL55	AA	2.3:2.4:2.5:2.6:2.7	1.2	2.9	1.5E-14	0.144	-0.078	4.1E-03	2.0E-03	3.4E-02
KIRC	MRPL55_AD_2.3:2.4:2.5:2.6_2.2_2.9	MRPL55	AD	2.3:2.4:2.5:2.6	2.2	2.9	5.2E-12	0.118	-0.255	3.0E-02	8.8E-03	3.6E-01
KIRC	MRPL55_ES_1.2:2.2:2.3:2.4:2.5:2.6:2.7:2	MRPL55	ES	2:2.3:2.4:2.5:2.6:2	1.1	2.9	4.0E-12	0.123	-0.100	3.4E-03	1.2E-02	1.7E-02
KIRC	MRPL55_RI_2.3:2.4_2.2_2.5	MRPL55	RI	2.3:2.4	2.2	2.5	2.3E-14	0.140	-0.140	1.0E-01	1.3E-02	2.7E-01
KIRC	MRPL55_RI_2.3:2.4:2.5:2.6:2.7_2.2_2.8	MRPL55	RI	2.3:2.4:2.5:2.6:2.7	2.2	2.8	2.9E-19	0.186	-0.194	3.8E-04	3.1E-03	6.2E-02
KIRC	MRPL55_RI_2.3:2.4:2.5:2.6:2.7:2.8_2.2_	MRPL55	RI	3:2.4:2.5:2.6:2.7:2	2.2	2.9	1.5E-17	0.170	-0.189	1.0E-02	2.0E-02	2.4E-01
KIRC	MRPL55_RI_2.7_2.6_2.8	MRPL55	RI	2.7	2.6	2.8	7.2E-11	0.118	-0.112	8.3E-03	1.2E-01	7.9E-01
KIRC	MRPL55_RI_2.7:2.8_2.6_2.9	MRPL55	RI	2.7:2.8	2.6	2.9	2.6E-15	0.150	-0.106	1.8E-02	1.8E-01	3.5E-01
KIRC	MRRF_ES_5:6_4_7	MRRF	ES	5:06	4	7	1.3E-25	0.239	0.263	1.3E-05	3.0E-07	4.8E-04
KIRC	MST1_RI_8.2_8.1_8.3	MST1	RI	8.2	8.1	8.3	1.6E-14	0.145	-0.160	3.6E-02	3.2E-02	1.2E-02
KIRC	MSTO1_RI_18.2_18.1_18.3	MSTO1	RI	18.2	18.1	18.3	1.8E-25	0.238	0.390	3.4E-05	4.3E-04	2.0E-02
KIRC	MTA3_ES_7_6_8	MTA3	ES	7	6	8	3.6E-31	0.282	-0.193	6.4E-05	2.3E-04	2.2E-02
KIRC	MTERFD3_RI_1.2_1.1_1.3	MTERFD3	RI	1.2	1.1	1.3	2.1E-25	0.237	0.548	7.6E-07	5.4E-08	6.5E-04
KIRC	MTERFD3_RI_1.2:1.3_1.1_1.4	MTERFD3	RI	1.2:1.3	1.1	1.4	2.4E-15	0.150	0.411	5.7E-07	8.0E-05	2.2E-01
KIRC	MTFR1L_AA_7.1_4.2_7.2	MTFR1L	AA	7.1	4.2	7.2	2.5E-21	0.203	0.088	3.1E-06	3.1E-04	2.1E-03
KIRC	MTFR1L_ES_5_4.2_7.2	MTFR1L	ES	5	4.2	7.2	4.5E-26	0.242	0.077	7.1E-05	2.4E-06	7.6E-06
KIRC	MTFR1L_ES_6_4.2_7.2	MTFR1L	ES	6	4.2	7.2	2.4E-25	0.237	0.088	2.4E-05	2.0E-05	3.4E-06
KIRC	MTIF2_ES_4_3_5	MTIF2	ES	4	3	5	1.0E-30	0.279	-0.386	4.0E-05	2.7E-05	1.9E-02
KIRC	MTMR10_ES_8_7_9	MTMR10	ES	8	7	9	7.8E-17	0.169	-0.078	7.8E-03	1.8E-02	4.2E-02
KIRC	MTMR10_RI_18.2_18.1_18.3	MTMR10	RI	18.2	18.1	18.3	1.7E-28	0.261	-0.378	2.3E-07	1.5E-05	1.5E-02
KIRC	MTO1_ES_8_7_9	MTO1	ES	8	7	9	3.4E-25	0.236	-0.182	6.2E-04	3.5E-04	5.4E-03
KIRC	MUTYH_AA_6.4_5_6.5	MUTYH	AA	6.4	5	6.5	7.7E-13	0.132	-0.340	1.1E-02	5.4E-03	3.6E-02
KIRC	MVD_ES_3:4_1_5	MVD	ES	3:04	1	5	1.1E-17	0.171	0.251	1.0E-05	2.0E-05	1.7E-04
KIRC	MXD1_AA_5.1_4_5.2	MXD1	AA	5.1	4	5.2	1.5E-18	0.198	0.089	1.5E-04	3.4E-05	1.5E-02
KIRC	MXRA7_RI_3.2_3.1_3.3	MXRA7	RI	3.2	3.1	3.3	6.3E-10	0.115	-0.081	1.1E-01	1.0E-02	1.7E-02
KIRC	MYH10_ES_6.1_5_7	MYH10	ES	6.1	5	7	6.2E-09	0.106	-0.118	5.5E-02	4.4E-02	2.9E-01
KIRC	MYO19_AA_21.1_20_21.2	MYO19	AA	21.1	20	21.2	3.2E-16	0.157	0.044	1.2E-04	2.1E-02	3.3E-02
KIRC	MYO19_ES_11.2:12_11.1_13	MYO19	ES	11.2:12	11.1	13	8.1E-25	0.232	-0.081	4.0E-05	1.9E-04	3.9E-04
KIRC	MYO19_ES_23_22_24	MYO19	ES	23	22	24	3.4E-11	0.111	0.157	1.2E-04	2.5E-07	1.5E-03
KIRC	MYO6_ES_29:30:31_28.1_32	MYO6	ES	29:30:31	28.1	32	9.4E-10	0.101	0.236	2.4E-01	5.7E-01	9.4E-01
KIRC	NAA16_AD_13.2:13.3_13.1_14	NAA16	AD	13.2:13.3	13.1	14	1.1E-12	0.156	0.168	3.3E-03	1.6E-02	1.3E-01
KIRC	NAA25_ES_17_16_18	NAA25	ES	17	16	18	8.7E-26	0.259	0.132	7.4E-02	1.3E-02	4.3E-01
KIRC	NAA25_ES_2_1_3	NAA25	ES	2	1	3	1.0E-23	0.225	-0.079	3.6E-02	4.0E-04	1.4E-03

cancer type	id	Gene Symbol	splice_type	Exon	From.Exon	To.Exon	anova.p	adj.r2	r	p.50	p.25	p.10
KIRC	NAA40_AA_4.1_3_4.2	NAA40	AA	4.1	3	4.2	8.6E-31	0.286	0.500	1.1E-07	4.8E-06	2.6E-04
KIRC	NABP1_ES_4_3_5.2	NABP1	ES	4	3	5.2	8.0E-12	0.121	0.254	2.5E-02	6.4E-02	1.0E-01
KIRC	NABP1_ES_4:5.1_3_5.2	NABP1	ES	04:05.1	3	5.2	1.1E-21	0.208	0.250	4.3E-02	1.7E-02	2.1E-01
KIRC	NACA_AD_3.4:3.5_3.3_4.2	NACA	AD	3.4:3.5	3.3	4.2	3.8E-17	0.179	-0.193	1.5E-03	9.6E-03	7.7E-02
KIRC	NADK2_ES_10_9_11	NADK2	ES	10	9	11	4.1E-13	0.129	0.197	4.6E-03	7.8E-04	5.8E-02
KIRC	NADSYN1_ES_9.2:10_9.1_11	NADSYN1	ES	9.2:10	9.1	11	8.1E-26	0.241	0.478	4.7E-05	9.9E-04	2.6E-02
KIRC	NAGPA_ES_6_5_7	NAGPA	ES	6	5	7	1.2E-18	0.183	-0.032	1.7E-01	2.9E-02	4.6E-02
KIRC	NAPA_ES_2:3:4_1_5	NAPA	ES	2:03:04	1	5	7.5E-10	0.107	0.039	1.3E-01	3.1E-01	4.5E-01
KIRC	NASP_ES_2_1_3	NASP	ES	2	1	3	7.5E-29	0.264	0.463	1.8E-04	2.6E-04	7.2E-04
KIRC	NASP_ES_9_8_10	NASP	ES	9	8	10	1.3E-11	0.114	0.524	1.7E-03	1.3E-04	1.4E-01
KIRC	NAT14_RI_2.3_2.2_2.4	NAT14	RI	2.3	2.2	2.4	2.9E-19	0.186	-0.095	1.4E-03	2.6E-05	1.3E-02
KIRC	NAT6_AA_3.1_1_3.2	NAT6	AA	3.1	1	3.2	2.2E-10	0.103	0.210	7.0E-05	6.2E-07	1.5E-04
KIRC	NAT9_RI_6.3:6.4_6.2_6.5	NAT9	RI	6.3:6.4	6.2	6.5	1.8E-33	0.301	0.500	3.7E-05	1.4E-06	1.9E-03
KIRC	NBPF1_ES_4_3_8	NBPF1	ES	4	3	8	5.7E-09	0.105	0.058	4.1E-03	1.1E-02	4.2E-02
KIRC	NBPF15_ES_2_1_3	NBPF15	ES	2	1	3	3.1E-13	0.132	-0.124	1.8E-05	1.1E-04	5.3E-05
KIRC	NBPF15_ES_4:5_3_6	NBPF15	ES	4:05	3	6	9.1E-18	0.172	-0.006	1.7E-09	2.0E-06	1.6E-03
KIRC	NBPF3_AA_12.1_11_12.2	NBPF3	AA	12.1	11	12.2	1.9E-15	0.153	-0.382	4.5E-04	4.1E-04	2.2E-03
KIRC	NCF4_RI_8.2_8.1_8.3	NCF4	RI	8.2	8.1	8.3	8.0E-31	0.280	0.202	3.9E-05	3.8E-05	1.4E-05
KIRC	NCOR2_AD_46.2_46.1_47	NCOR2	AD	46.2	46.1	47	3.0E-10	0.101	-0.123	9.6E-05	1.3E-03	7.9E-03
KIRC	NDRG1_ES_1.2:2.1:2.2:2.3:3_1.1_4	NDRG1	ES	1.2:2.1:2.2:2.3:3	1.1	4	3.4E-17	0.177	-0.275	2.9E-03	1.3E-03	1.9E-02
KIRC	NDRG1_ES_2.2:14:15:16.1_2.1_16.2	NDRG1	ES	2.2:14:15:16.1	2.1	16.2	3.2E-14	0.140	0.052	3.0E-03	3.4E-03	1.2E-02
KIRC	NDRG1_ES_2.2:2.3:3:4:5.1:5.2:6:7.1:7.2:	NDRG1	ES	6:7.1:7.2:8:9:10:1	2.1	16.2	3.1E-19	0.185	-0.080	3.0E-03	9.7E-04	6.4E-03
KIRC	NDRG1_ES_2.3:3:4:5.1:5.2:6:7.1:7.2:8:9:	NDRG1	ES	5.2:6:7.1:7.2:8:9:	2.2	14	5.1E-33	0.297	-0.090	1.0E-03	1.1E-04	8.6E-04
KIRC	NDRG2_AD_4.2:4.3:4.4:4.5_4.1_5.2	NDRG2	AD	4.2:4.3:4.4:4.5	4.1	5.2	3.2E-22	0.213	-0.282	5.3E-05	2.7E-05	8.9E-03
KIRC	NDRG2_ES_4.5:4.6_4.1_5.2	NDRG2	ES	4.5:4.6	4.1	5.2	2.5E-15	0.164	-0.188	1.7E-04	1.8E-02	4.6E-02
KIRC	NDRG2_RI_4.2:4.3:4.4_4.1_4.5	NDRG2	RI	4.2:4.3:4.4	4.1	4.5	3.1E-25	0.236	-0.045	4.6E-04	2.5E-04	1.7E-02
KIRC	NDUFA3_RI_4.2_4.1_4.3	NDUFA3	RI	4.2	4.1	4.3	1.9E-17	0.169	-0.264	6.1E-03	1.6E-01	6.7E-01
KIRC	NDUFAF1_ES_4_3_5	NDUFAF1	ES	4	3	5	4.7E-19	0.183	-0.428	5.2E-04	2.4E-03	2.5E-02
KIRC	NDUFAF5_ES_9_8_10	NDUFAF5	ES	9	8	10	4.7E-16	0.156	-0.144	2.0E-01	2.3E-01	6.5E-02
KIRC	NDUFS2_RI_14.2_14.1_14.3	NDUFS2	RI	14.2	14.1	14.3	1.7E-31	0.285	-0.224	1.9E-06	1.7E-04	1.2E-02
KIRC	NECAB3_RI_6.2:6.3_6.1_6.4	NECAB3	RI	6.2:6.3	6.1	6.4	5.6E-29	0.268	0.116	1.1E-05	8.9E-06	4.9E-05
KIRC	NEDD4L_ES_18_17_19	NEDD4L	ES	18	17	19	2.6E-12	0.124	0.426	1.0E-02	5.4E-02	5.1E-01
KIRC	NEK8_RI_5.2_5.1_5.3	NEK8	RI	5.2	5.1	5.3	4.3E-18	0.224	0.145	2.4E-05	4.3E-05	6.4E-04
KIRC	NFS1_ES_7_5_8	NFS1	ES	7	5	8	6.4E-34	0.303	-0.113	4.1E-04	1.1E-05	1.9E-02
KIRC	NHEJ1_ES_6_5_7	NHEJ1	ES	6	5	7	3.1E-11	0.116	0.552	3.3E-01	5.1E-01	6.1E-01
KIRC	NIPA1_ES_7:8_5_9	NIPA1	ES	7:08	5	9	2.9E-08	0.104	-0.109	3.9E-01	2.5E-01	5.1E-01
KIRC	NISCH_RI_14.2_14.1_14.3	NISCH	RI	14.2	14.1	14.3	7.4E-18	0.173	0.584	8.0E-04	4.0E-03	1.5E-01
KIRC	NKIRAS2_ES_5.4:5.5:5.6:6_5.3_7	NKIRAS2	ES	5.4:5.5:5.6:6	5.3	7	2.8E-17	0.176	0.012	1.1E-03	2.8E-03	6.3E-03
KIRC	NKIRAS2_ES_5.5:5.6:6_5.3_7	NKIRAS2	ES	5.5:5.6:6	5.3	7	2.0E-19	0.202	-0.007	1.8E-04	5.9E-03	2.9E-02
KIRC	NKIRAS2_ES_5.6:6_5.3_7	NKIRAS2	ES	5.6:6	5.3	7	2.2E-18	0.193	-0.008	2.2E-04	6.7E-03	9.6E-03

cancer type	id	Gene Symbol	splice_type	Exon	From.Exon	To.Exon	anova.p	adj.r2	r	p.50	p.25	p.10
KIRC	NKIRAS2_ES_6_5.6_7	NKIRAS2	ES	6	5.6	7	4.8E-27	0.250	0.029	2.3E-04	6.4E-05	7.1E-04
KIRC	NLN_ES_2_1_3	NLN	ES	2	1	3	1.7E-24	0.240	-0.728	1.3E-03	3.6E-03	1.6E-03
KIRC	NLRC5_AD_7.2_7.1_8	NLRC5	AD	7.2	7.1	8	9.0E-14	0.150	0.368	8.9E-04	3.0E-02	4.4E-02
KIRC	NME6_AA_5.1_4_5.2	NME6	AA	5.1	4	5.2	2.8E-11	0.113	-0.202	8.1E-02	9.1E-02	5.6E-02
KIRC	NMNAT3_ES_6_5_7	NMNAT3	ES	6	5	7	3.7E-09	0.100	-0.181	8.9E-05	5.4E-03	8.3E-04
KIRC	NMRAL1_RI_2.3:2.4:2.5_2.2_2.6	NMRAL1	RI	2.3:2.4:2.5	2.2	2.6	8.3E-13	0.126	-0.044	2.5E-02	2.0E-02	2.4E-02
KIRC	NMRAL1_RI_2.4:2.5_2.3_2.6	NMRAL1	RI	2.4:2.5	2.3	2.6	4.3E-17	0.167	-0.059	2.0E-03	6.8E-05	1.5E-04
KIRC	NMRAL1_RI_2.5_2.4_2.6	NMRAL1	RI	2.5	2.4	2.6	3.3E-13	0.131	-0.061	2.3E-02	1.0E-01	6.1E-02
KIRC	NMRK1_ES_3_2_4	NMRK1	ES	3	2	4	4.4E-16	0.156	-0.103	2.0E-03	4.2E-03	1.0E-01
KIRC	NNMT_AD_5.2:5.3_5.1_6	NNMT	AD	5.2:5.3	5.1	6	5.5E-13	0.128	-0.255	2.0E-02	2.4E-03	3.8E-01
KIRC	NOD1_AA_8.1_7_8.2	NOD1	AA	8.1	7	8.2	8.2E-16	0.155	0.329	7.0E-03	3.7E-02	7.4E-02
KIRC	NOL12_RI_6.2_6.1_6.3	NOL12	RI	6.2	6.1	6.3	1.5E-17	0.169	0.185	8.7E-03	3.1E-03	2.0E-01
KIRC	NOL8_AA_16.1_15_16.2	NOL8	AA	16.1	15	16.2	2.0E-22	0.212	0.434	3.4E-04	5.9E-04	2.3E-02
KIRC	NOL8_ES_4_3_5	NOL8	ES	4	3	5	4.7E-10	0.114	0.193	2.0E-01	1.1E-01	2.8E-01
KIRC	NOL8_ES_6.1:6.2:6.3_5_7	NOL8	ES	6.1:6.2:6.3	5	7	5.9E-11	0.114	0.203	3.0E-02	3.1E-02	3.3E-02
KIRC	NOL8_ES_6.2:6.3_5_7	NOL8	ES	6.2:6.3	5	7	1.1E-17	0.178	0.176	5.6E-05	1.1E-05	2.9E-07
KIRC	NOP2_ES_5_4.5_6	NOP2	ES	5	4.5	6	4.1E-16	0.156	0.104	8.2E-03	9.5E-02	3.9E-01
KIRC	NOP2_RI_4.3_4.2_4.4	NOP2	RI	4.3	4.2	4.4	2.2E-21	0.207	0.333	2.2E-05	1.4E-04	9.4E-03
KIRC	NOP2_RI_4.3:4.4_4.2_4.5	NOP2	RI	4.3:4.4	4.2	4.5	3.5E-13	0.140	0.236	8.6E-04	4.2E-04	1.3E-02
KIRC	NPEPPS_AD_9.2_9.1_10	NPEPPS	AD	9.2	9.1	10	1.2E-29	0.272	-0.186	3.3E-04	3.2E-04	2.0E-03
KIRC	NPHP1_AD_8.2_8.1_9.1	NPHP1	AD	8.2	8.1	9.1	7.5E-14	0.161	0.042	4.6E-03	3.5E-04	1.8E-03
KIRC	NPHP1_ES_8.2:9.1_8.1_9.2	NPHP1	ES	8.2:9.1	8.1	9.2	1.5E-11	0.139	0.022	1.8E-01	2.9E-02	4.1E-02
KIRC	NPIPB4_RI_9.3_9.2_9.4	NPIPB4	RI	9.3	9.2	9.4	1.4E-16	0.161	-0.337	3.1E-03	1.6E-02	2.5E-02
KIRC	NPNT_ES_5_4_6	NPNT	ES	5	4	6	2.7E-09	0.105	-0.122	2.2E-02	1.1E-02	2.3E-01
KIRC	NPRL2_RI_3.3_3.2_3.4	NPRL2	RI	3.3	3.2	3.4	5.8E-42	0.362	0.250	3.1E-04	6.8E-05	3.0E-03
KIRC	NPRL3_ES_3_2_4	NPRL3	ES	3	2	4	8.3E-17	0.163	0.093	5.3E-02	2.6E-01	4.1E-02
KIRC	NR1H3_RI_3.3:3.4_3.2_3.5	NR1H3	RI	3.3:3.4	3.2	3.5	2.1E-37	0.329	0.000	4.5E-04	3.6E-06	5.1E-04
KIRC	NR1H3_RI_3.4_3.3_3.5	NR1H3	RI	3.4	3.3	3.5	3.5E-10	0.101	-0.022	3.2E-02	1.1E-03	7.4E-04
KIRC	NRBP2_RI_5.2_5.1_5.3	NRBP2	RI	5.2	5.1	5.3	8.0E-35	0.315	0.681	4.2E-05	4.6E-04	2.0E-04
KIRC	NRD1_ES_4:5_3_6	NRD1	ES	4:05	3	6	1.8E-24	0.231	0.010	9.8E-03	1.9E-03	3.7E-05
KIRC	NSFL1C_ES_5.1:5.2:7.2_4_7.3	NSFL1C	ES	5.1:5.2:7.2	4	7.3	3.8E-13	0.134	-0.213	1.3E-03	2.0E-02	1.8E-01
KIRC	NSFL1C_ES_6:7.1:7.2_4_7.3	NSFL1C	ES	6:7.1:7.2	4	7.3	3.8E-10	0.116	-0.082	3.4E-03	2.9E-03	3.1E-03
KIRC	NSUN5_ES_2:3.2:4_1_5	NSUN5	ES	03:02.2	1	5	5.1E-11	0.134	-0.065	3.1E-01	1.2E-01	5.9E-01
KIRC	NSUN5_RI_9.2_9.1_9.3	NSUN5	RI	9.2	9.1	9.3	6.3E-24	0.226	0.184	4.1E-03	3.0E-04	5.2E-04
KIRC	NT5C_AD_3.3_3.2_4.1	NT5C	AD	3.3	3.2	4.1	2.8E-20	0.193	0.158	1.4E-08	5.9E-11	4.0E-04
KIRC	NT5C3B_ES_3_2_4	NT5C3B	ES	3	2	4	1.6E-14	0.142	0.099	7.5E-01	6.5E-01	6.4E-01
KIRC	NUBP2_ES_2_1_3	NUBP2	ES	2	1	3	3.2E-25	0.235	-0.182	4.1E-04	4.1E-04	1.8E-02
KIRC	NUDT16_RI_3.2_3.1_3.3	NUDT16	RI	3.2	3.1	3.3	2.3E-13	0.131	0.110	6.1E-02	7.2E-02	4.1E-01
KIRC	NUDT16L1_RI_2.3_2.2_2.4	NUDT16L1	RI	2.3	2.2	2.4	1.8E-23	0.233	-0.036	4.0E-03	6.7E-03	1.9E-03
KIRC	NUDT6_AD_2.2:2.3_2.1_3	NUDT6	AD	2.2:2.3	2.1	3	2.6E-20	0.209	-0.231	4.8E-03	8.3E-02	2.3E-01

cancer type	id	Gene Symbol	splice_type	Exon	From.Exon	To.Exon	anova.p	adj.r2	r	p.50	p.25	p.10
KIRC	NUP160_RI_3.2_3.1_3.3	NUP160	RI	3.2	3.1	3.3	2.7E-14	0.144	-0.458	4.1E-02	1.7E-04	4.8E-03
KIRC	NUP50_ES_4.1:4.2_3.2_5	NUP50	ES	4.1:4.2	3.2	5	3.6E-11	0.116	-0.030	3.2E-01	3.5E-01	4.0E-01
KIRC	NUP62_AD_1.3:1.4:1.5_1.2_2.1	NUP62	AD	1.3:1.4:1.5	1.2	2.1	5.3E-23	0.217	0.093	4.3E-04	1.7E-06	5.3E-05
KIRC	NUP62_RI_1.3_1.2_1.4	NUP62	RI	1.3	1.2	1.4	9.2E-22	0.206	0.114	7.7E-05	1.8E-06	1.2E-03
KIRC	NUP62_RI_1.3:1.4_1.2_1.5	NUP62	RI	1.3:1.4	1.2	1.5	3.5E-30	0.275	0.226	2.5E-08	5.1E-07	1.1E-03
KIRC	NUP85_RI_18.2_18.1_18.3	NUP85	RI	18.2	18.1	18.3	1.6E-28	0.262	0.438	8.6E-05	4.1E-06	8.8E-04
KIRC	NUP88_ES_2_1_3	NUP88	ES	2	1	3	5.5E-17	0.170	-0.161	1.3E-02	2.0E-02	8.9E-03
KIRC	NVL_ES_5_4_6	NVL	ES	5	4	6	4.1E-18	0.200	0.224	2.5E-03	3.9E-02	9.9E-02
KIRC	NXF1_RI_11.2_11.1_11.3	NXF1	RI	11.2	11.1	11.3	6.0E-19	0.182	0.782	1.5E-02	2.7E-04	3.8E-02
KIRC	OCIAD1_AD_2.3:2.4_2.2_3	OCIAD1	AD	2.3:2.4	2.2	3	1.0E-09	0.105	-0.166	1.3E-03	1.8E-05	2.6E-04
KIRC	OCIAD1_AD_2.4_2.3_3	OCIAD1	AD	2.4	2.3	3	6.4E-17	0.167	-0.186	3.0E-03	6.7E-04	5.5E-05
KIRC	OCIAD1_ES_2.2:2.3:3:4:5_2.1_6	OCIAD1	ES	2.2:2.3:3:4:5	2.1	6	1.8E-16	0.193	-0.235	1.7E-04	1.4E-03	3.4E-04
KIRC	OCIAD1_ES_3:4:5_2.1_6	OCIAD1	ES	3:04:05	2.1	6	4.3E-11	0.128	-0.169	3.9E-01	1.1E-02	5.1E-02
KIRC	ODF3B_AA_4.1_3.2_4.2	ODF3B	AA	4.1	3.2	4.2	1.1E-22	0.215	0.205	2.3E-05	3.3E-06	1.3E-06
KIRC	ODF3B_ES_3.1:3.2:4.1_2.2_4.2	ODF3B	ES	3.1:3.2:4.1	2.2	4.2	2.2E-13	0.137	0.165	2.6E-03	3.2E-03	8.9E-03
KIRC	OGFOD2_ES_6.3:8.1_6.2_8.2	OGFOD2	ES	6.3:8.1	6.2	8.2	8.0E-10	0.102	0.286	2.7E-04	4.7E-03	4.8E-05
KIRC	OGFOD2_ES_6.3:8.1:8.2_6.2_8.3	OGFOD2	ES	6.3:8.1:8.2	6.2	8.3	3.3E-24	0.228	0.320	2.2E-05	2.6E-07	8.2E-04
KIRC	OGFOD2_ES_7_6.2_8.2	OGFOD2	ES	7	6.2	8.2	8.3E-11	0.126	0.319	1.8E-02	1.2E-02	1.1E-02
KIRC	OGFOD2_ES_7:8.2_6.2_8.3	OGFOD2	ES	07:08.2	6.2	8.3	9.5E-17	0.163	0.368	8.6E-04	1.0E-03	1.6E-01
KIRC	OGFR_RI_2.3_2.2_2.4	OGFR	RI	2.3	2.2	2.4	3.2E-35	0.313	0.484	3.5E-06	9.7E-06	1.1E-04
KIRC	OGG1_ES_7.1:7.2_6.1_8	OGG1	ES	7.1:7.2	6.1	8	7.1E-16	0.163	-0.098	8.3E-05	1.4E-04	2.1E-04
KIRC	OGG1_RI_6.2:6.3_6.1_6.4	OGG1	RI	6.2:6.3	6.1	6.4	1.2E-11	0.114	-0.151	2.8E-02	4.3E-03	1.5E-01
KIRC	OPA1_ES_7_6_8	OPA1	ES	7	6	8	2.4E-11	0.113	0.152	4.0E-04	3.0E-04	5.5E-02
KIRC	ORAOV1_ES_2_1_3	ORAOV1	ES	2	1	3	2.4E-10	0.108	0.448	2.1E-04	1.4E-03	1.0E-02
KIRC	ORAOV1_RI_5.2_5.1_5.3	ORAOV1	RI	5.2	5.1	5.3	1.3E-16	0.162	0.119	1.5E-04	7.6E-05	4.2E-04
KIRC	ORMDL1_ES_2.2_1_2.4	ORMDL1	ES	2.2	1	2.4	8.4E-11	0.107	-0.132	2.0E-01	9.4E-01	6.2E-01
KIRC	ORMDL1_RI_2.3_2.2_2.4	ORMDL1	RI	2.3	2.2	2.4	1.3E-26	0.247	0.584	1.8E-03	2.4E-04	3.4E-04
KIRC	OS9_ES_5.1:5.2:5.3:6:7.1:7.2:7.3:7.4:8:9	OS9	ES	5.3:6:7.1:7.2:7.3:7	4	9.2	8.7E-31	0.282	-0.313	6.2E-03	2.3E-04	1.1E-02
KIRC	OS9_ES_5.1:6:7.1:7.4:8:9.1_4_9.2	OS9	ES	5.1:6:7.1:7.4:8:9.1	4	9.2	4.1E-25	0.242	-0.236	2.9E-05	2.2E-04	1.5E-05
KIRC	OS9_RI_7.2_7.1_7.3	OS9	RI	7.2	7.1	7.3	3.7E-34	0.305	-0.314	1.7E-03	1.5E-04	9.5E-04
KIRC	OSBPL3_AD_15.2_15.1_16	OSBPL3	AD	15.2	15.1	16	3.4E-25	0.237	0.266	3.3E-04	2.5E-05	1.2E-05
KIRC	OSGEP_AD_4.5_4.4_5	OSGEP	AD	4.5	4.4	5	3.3E-23	0.219	0.152	2.3E-04	4.2E-04	1.6E-04
KIRC	OSGEP_RI_4.3_4.2_4.4	OSGEP	RI	4.3	4.2	4.4	5.5E-31	0.281	0.267	9.8E-06	9.7E-09	1.2E-04
KIRC	OSTC_ES_3:4_2_5	OSTC	ES	3:04	2	5	1.1E-17	0.180	-0.372	2.3E-01	1.9E-03	1.7E-01
KIRC	OTUD6B_ES_4_3_5	OTUD6B	ES	4	3	5	1.0E-12	0.133	-0.109	1.2E-04	5.0E-03	2.6E-03
KIRC	P4HTM_ES_6.2:7.1_6.1_7.2	P4HTM	ES	6.2:7.1	6.1	7.2	1.5E-36	0.323	0.139	1.0E-05	8.6E-08	1.4E-04
KIRC	PABPN1_RI_5.2_5.1_5.3	PABPN1	RI	5.2	5.1	5.3	1.1E-16	0.161	0.374	2.8E-03	1.4E-02	4.5E-01
KIRC	PAPLN_ES_21.1:21.2_20_22	PAPLN	ES	21.1:21.2	20	22	3.5E-17	0.173	0.136	3.0E-03	5.1E-04	5.1E-03
KIRC	PAPOLA_RI_9.2_9.1_9.3	PAPOLA	RI	9.2	9.1	9.3	2.4E-31	0.284	-0.401	5.7E-02	1.4E-02	3.8E-02
KIRC	PAPSS2_ES_9_8_10	PAPSS2	ES	9	8	10	9.0E-19	0.191	0.112	9.1E-08	3.6E-06	3.9E-04

cancer type	id	Gene Symbol	splice_type	Exon	From.Exon	To.Exon	anova.p	adj.r2	r	p.50	p.25	p.10
KIRC	PAQR3_ES_7:8_6_9.1	PAQR3	ES	7:08	6	9.1	1.0E-16	0.182	-0.122	6.7E-03	2.6E-03	9.7E-02
KIRC	PARP2_RI_15.2_15.1_15.3	PARP2	RI	15.2	15.1	15.3	9.4E-28	0.256	0.356	1.9E-05	1.3E-05	4.4E-04
KIRC	PARPBP_ES_3_2_4.1	PARPBP	ES	3	2	4.1	7.4E-23	0.273	-0.467	9.3E-01	8.4E-01	2.7E-01
KIRC	PAX8_ES_8:9.2:10_7_11	PAX8	ES	09:02.2	7	11	4.3E-15	0.150	-0.247	2.8E-04	2.1E-02	2.9E-02
KIRC	PAXBP1_AD_8.2_8.1_9	PAXBP1	AD	8.2	8.1	9	2.1E-13	0.136	0.622	1.8E-05	2.0E-05	3.2E-03
KIRC	PCDP1_ES_4_3_6	PCDP1	ES	4	3	6	4.6E-17	0.178	-0.260	6.1E-03	1.7E-03	4.7E-03
KIRC	PCGF3_AA_5.3_5.1_5.4	PCGF3	AA	5.3	5.1	5.4	4.4E-26	0.244	0.716	3.7E-03	1.5E-04	6.4E-02
KIRC	PCGF3_RI_5.2:5.3_5.1_5.4	PCGF3	RI	5.2:5.3	5.1	5.4	2.8E-23	0.219	0.482	7.8E-07	8.4E-07	3.9E-06
KIRC	PCK1_RI_7.2_7.1_7.3	PCK1	RI	7.2	7.1	7.3	2.9E-12	0.146	-0.216	1.2E-01	3.3E-01	1.9E-01
KIRC	PCNP_AA_2.1_1_2.2	PCNP	AA	2.1	1	2.2	2.1E-22	0.212	-0.112	2.8E-04	7.8E-04	4.4E-03
KIRC	PCNP_AA_2.1:2.2_1_2.3	PCNP	AA	2.1:2.2	1	2.3	5.1E-23	0.217	-0.169	3.9E-06	1.6E-06	1.8E-06
KIRC	PCNP_ES_2.1:2.2:2.3_1_3	PCNP	ES	2.1:2.2:2.3	1	3	3.9E-13	0.129	-0.134	1.9E-08	3.3E-05	3.6E-03
KIRC	PCSK7_RI_8.2_8.1_8.3	PCSK7	RI	8.2	8.1	8.3	6.9E-31	0.282	0.370	2.8E-05	1.4E-06	1.9E-02
KIRC	PCTP_AA_6.1_5_6.2	PCTP	AA	6.1	5	6.2	2.0E-25	0.238	-0.329	1.1E-05	1.2E-06	2.1E-05
KIRC	PCYT1A_AA_7.1_6_7.2	PCYT1A	AA	7.1	6	7.2	3.7E-31	0.284	-0.274	1.3E-03	5.9E-06	8.9E-04
KIRC	PDCD11_ES_3_2_4	PDCD11	ES	3	2	4	4.1E-10	0.112	-0.092	3.9E-02	5.1E-02	2.3E-02
KIRC	PDE4A_RI_18.2_18.1_18.3	PDE4A	RI	18.2	18.1	18.3	2.9E-23	0.223	0.022	2.4E-04	8.2E-04	1.6E-01
KIRC	PDE4DIP_ES_5.2:5.3_4_6.1	PDE4DIP	ES	5.2:5.3	4	6.1	5.7E-11	0.114	-0.213	3.6E-01	5.5E-01	1.1E-01
KIRC	PDGFA_ES_6_5_7	PDGFA	ES	6	5	7	1.2E-23	0.222	-0.019	3.5E-04	3.6E-04	1.8E-02
KIRC	PDLIM7_AA_10.1_8_10.2	PDLIM7	AA	10.1	8	10.2	9.8E-18	0.172	0.151	3.4E-05	7.0E-07	1.3E-05
KIRC	PDPR_AA_13.1_12_13.2	PDPR	AA	13.1	12	13.2	2.7E-19	0.186	-0.068	2.6E-01	6.2E-01	5.5E-02
KIRC	PDPR_ES_18_17_19	PDPR	ES	18	17	19	1.1E-16	0.163	0.132	1.4E-01	2.8E-01	9.0E-01
KIRC	PDPR_ES_8_7_9	PDPR	ES	8	7	9	3.9E-11	0.127	0.463	1.7E-02	2.8E-02	1.5E-03
KIRC	PDZD3_RI_2.2:2.3_2.1_2.4	PDZD3	RI	2.2:2.3	2.1	2.4	8.0E-16	0.166	0.385	2.1E-07	4.1E-04	1.2E-01
KIRC	PELP1_ES_10.2:11.1_10.1_11.2	PELP1	ES	10.2:11.1	10.1	11.2	2.0E-37	0.330	0.302	1.1E-06	1.3E-06	2.0E-03
KIRC	PEX10_ES_2_1_3	PEX10	ES	2	1	3	8.2E-19	0.209	-0.141	1.4E-04	2.3E-05	1.7E-02
KIRC	PEX5_AD_2.2:2.3:2.4_2.1_3.2	PEX5	AD	2.2:2.3:2.4	2.1	3.2	1.3E-10	0.106	-0.084	5.8E-03	3.2E-03	2.5E-04
KIRC	PFKM_AD_16.2:16.3_16.1_17	PFKM	AD	16.2:16.3	16.1	17	4.3E-22	0.211	-0.282	2.8E-03	4.2E-04	1.8E-02
KIRC	PFKM_ES_8_7.3_9	PFKM	ES	8	7.3	9	8.3E-34	0.302	-0.591	1.5E-05	2.0E-05	7.6E-03
KIRC	PFKM_RI_16.2_16.1_16.3	PFKM	RI	16.2	16.1	16.3	6.7E-33	0.296	-0.285	7.1E-07	1.9E-06	6.4E-05
KIRC	PGAP2_AD_11.2:11.3_11.1_12	PGAP2	AD	11.2:11.3	11.1	12	2.8E-22	0.212	0.125	4.0E-05	8.0E-06	5.1E-04
KIRC	PGAP2_AD_11.3_11.2_12	PGAP2	AD	11.3	11.2	12	2.3E-26	0.247	0.175	1.1E-06	8.5E-06	6.6E-03
KIRC	PGAP2_ES_8:9_6_10	PGAP2	ES	8:09	6	10	8.4E-11	0.114	0.108	1.7E-05	3.5E-05	1.2E-02
KIRC	PGS1_AA_9.1_8_9.2	PGS1	AA	9.1	8	9.2	2.0E-15	0.151	-0.594	3.4E-04	7.3E-05	6.2E-02
KIRC	PGS1_AD_10.2_10.1_11	PGS1	AD	10.2	10.1	11	2.5E-10	0.102	0.335	5.9E-01	2.4E-01	4.6E-01
KIRC	PGS1_ES_2_1_3.1	PGS1	ES	2	1	3.1	3.8E-21	0.201	0.194	2.2E-02	1.4E-02	8.5E-02
KIRC	PHB2_ES_5.2:5.3:6.1_5.1_6.2	PHB2	ES	5.2:5.3:6.1	5.1	6.2	3.5E-16	0.161	-0.193	5.6E-04	2.0E-03	3.3E-01
KIRC	PHF12_RI_12.2_12.1_12.3	PHF12	RI	12.2	12.1	12.3	1.1E-18	0.179	-0.025	1.9E-04	8.5E-04	7.1E-03
KIRC	PHF12_RI_12.4_12.3_12.5	PHF12	RI	12.4	12.3	12.5	4.5E-30	0.274	0.349	2.3E-06	7.0E-05	4.2E-04
KIRC	PHF12_RI_12.6_12.5_12.7	PHF12	RI	12.6	12.5	12.7	3.2E-27	0.252	0.367	5.1E-07	1.1E-06	2.4E-04

cancer type	id	Gene Symbol	splice_type	Exon	From.Exon	To.Exon	anova.p	adj.r2	r	p.50	p.25	p.10
KIRC	PHF20_ES_2_1_3	PHF20	ES	2	1	3	9.1E-31	0.279	-0.528	8.4E-04	2.1E-04	3.8E-02
KIRC	PHF20L1_ES_15_14_16	PHF20L1	ES	15	14	16	1.9E-13	0.134	0.044	3.0E-02	5.6E-03	3.8E-03
KIRC	PHF6_RI_10.2_10.1_10.3	PHF6	RI	10.2	10.1	10.3	9.8E-17	0.165	0.028	7.7E-04	7.3E-04	2.5E-03
KIRC	PHLDB1_RI_18.2_18.1_18.3	PHLDB1	RI	18.2	18.1	18.3	8.0E-11	0.128	0.024	2.6E-02	8.2E-03	2.0E-02
KIRC	PHPT1_ES_4_3_5	PHPT1	ES	4	3	5	8.8E-15	0.144	0.080	1.8E-01	2.6E-01	3.1E-01
KIRC	PHYKPL_ES_2_1_3	PHYKPL	ES	2	1	3	3.2E-30	0.276	0.425	1.1E-03	1.6E-03	2.0E-04
KIRC	PI4KA_ES_37:38:39:40:41:42:43_36_44	PI4KA	ES	':38:39:40:41:42:·	36	44	2.1E-21	0.226	0.312	2.1E-03	4.8E-03	1.7E-03
KIRC	PIAS3_RI_7.2_7.1_7.3	PIAS3	RI	7.2	7.1	7.3	1.4E-29	0.275	0.358	6.8E-05	6.6E-05	2.0E-03
KIRC	PIDD_RI_3.3_3.2_3.4	PIDD	RI	3.3	3.2	3.4	4.8E-11	0.115	0.510	1.3E-06	2.2E-03	1.3E-01
KIRC	PIGG_AD_9.2_9.1_10.1	PIGG	AD	9.2	9.1	10.1	7.7E-10	0.118	0.125	1.9E-03	4.0E-03	2.3E-02
KIRC	PIGO_RI_9.2_9.1_9.3	PIGO	RI	9.2	9.1	9.3	4.6E-17	0.165	0.059	1.4E-02	5.3E-03	8.2E-03
KIRC	PIK3CD_RI_17.2_17.1_17.3	PIK3CD	RI	17.2	17.1	17.3	5.7E-25	0.248	0.144	1.1E-04	1.1E-05	5.5E-05
KIRC	PIP5K1A_AD_1.2_1.1_2.1	PIP5K1A	AD	1.2	1.1	2.1	2.0E-12	0.143	0.175	4.6E-01	1.4E-01	1.7E-03
KIRC	PKP2_ES_6_5_7	PKP2	ES	6	5	7	1.0E-28	0.265	-0.471	4.2E-05	1.3E-05	1.8E-02
KIRC	PLA2G15_ES_2_1_3	PLA2G15	ES	2	1	3	4.4E-26	0.244	-0.119	2.9E-05	2.7E-04	1.6E-02
KIRC	PLA2G4C_ES_2.1:2.2_1_3	PLA2G4C	ES	2.1:2.2	1	3	2.2E-23	0.225	-0.054	7.5E-05	1.9E-05	5.4E-03
KIRC	PLAGL1_AA_5.1:5.2_4_5.3	PLAGL1	AA	5.1:5.2	4	5.3	1.2E-14	0.165	0.345	1.2E-02	6.1E-03	1.2E-02
KIRC	PLAGL1_AA_5.1:5.2:5.3:5.4:5.5:5.6_4_5.	PLAGL1	AA	1:5.2:5.3:5.4:5.5:!	4	5.7	4.1E-20	0.204	0.231	7.8E-04	9.7E-04	3.8E-01
KIRC	PLAGL1_AA_5.2:5.3:5.4:5.5:5.6_4_5.7	PLAGL1	AA	5.2:5.3:5.4:5.5:5.(	4	5.7	6.6E-12	0.134	0.131	5.3E-02	5.6E-01	7.6E-01
KIRC	PLAGL1_AA_5.3:5.4:5.5:5.6_4_5.7	PLAGL1	AA	5.3:5.4:5.5:5.6	4	5.7	6.4E-12	0.126	0.209	4.1E-02	4.2E-02	8.6E-01
KIRC	PLAGL1_RI_5.4:5.5:5.6_5.3_5.7	PLAGL1	RI	5.4:5.5:5.6	5.3	5.7	2.7E-28	0.261	0.359	2.0E-07	5.0E-07	1.4E-02
KIRC	PLCB2_AD_25.2:25.3_25.1_25.5	PLCB2	AD	25.2:25.3	25.1	25.5	2.2E-12	0.138	0.248	1.4E-02	2.7E-03	3.2E-01
KIRC	PLCB2_RI_25.2_25.1_25.3	PLCB2	RI	25.2	25.1	25.3	9.9E-13	0.140	0.313	5.3E-05	1.0E-04	9.4E-02
KIRC	PLCH2_ES_22.2:23.1_22.1_23.2	PLCH2	ES	22.2:23.1	22.1	23.2	5.4E-15	0.181	-0.211	9.7E-06	6.1E-05	3.3E-02
KIRC	PLD2_RI_18.2_18.1_18.3	PLD2	RI	18.2	18.1	18.3	2.2E-29	0.271	0.312	2.1E-06	5.6E-06	5.1E-04
KIRC	PLEKHA1_AD_15.2_15.1_16	PLEKHA1	AD	15.2	15.1	16	2.4E-18	0.178	0.229	8.4E-02	1.1E-01	4.6E-02
KIRC	PLEKHA1_ES_15.1:15.2_14_16	PLEKHA1	ES	15.1:15.2	14	16	7.4E-11	0.107	0.006	2.9E-06	2.4E-05	5.7E-05
KIRC	PLEKHA5_ES_27_26_28	PLEKHA5	ES	27	26	28	7.6E-20	0.190	0.116	6.5E-04	1.7E-06	1.2E-03
KIRC	PLEKHH2_ES_4_3_5	PLEKHH2	ES	4	3	5	3.3E-18	0.191	-0.468	2.7E-05	1.8E-03	1.6E-01
KIRC	PLEKHM1_ES_6_5_7	PLEKHM1	ES	6	5	7	9.5E-18	0.172	0.166	2.1E-02	5.5E-04	3.1E-03
KIRC	PLOD2_ES_15_14_16	PLOD2	ES	15	14	16	9.4E-16	0.153	0.065	4.9E-04	1.1E-06	3.0E-06
KIRC	PLS3_ES_5_3_6	PLS3	ES	5	3	6	2.6E-30	0.275	-0.603	6.0E-04	5.8E-04	1.5E-03
KIRC	PLXDC1_ES_13:14.1_12_14.2	PLXDC1	ES	13:14.1	12	14.2	2.8E-21	0.202	-0.067	1.1E-03	3.9E-05	4.2E-05
KIRC	PMF1_ES_6_5_7	PMF1	ES	6	5	7	4.2E-15	0.151	0.027	4.7E-02	1.3E-01	2.9E-01
KIRC	PML_RI_6.3_6.2_6.4	PML	RI	6.3	6.2	6.4	4.9E-10	0.100	0.067	8.2E-05	1.5E-04	4.7E-03
KIRC	PML_RI_6.5:6.6_6.4_6.7	PML	RI	6.5:6.6	6.4	6.7	6.3E-14	0.136	0.129	3.7E-04	1.0E-05	2.9E-04
KIRC	PMPCB_RI_12.2_12.1_12.3	PMPCB	RI	12.2	12.1	12.3	8.0E-27	0.248	-0.108	7.2E-06	6.8E-07	3.1E-04
KIRC	PODXL_ES_3_2.2_4.1	PODXL	ES	3	2.2	4.1	4.1E-17	0.165	-0.521	3.1E-06	2.1E-06	1.5E-04
KIRC	POFUT2_AA_8.3:8.4_8.1_8.5	POFUT2	AA	8.3:8.4	8.1	8.5	2.1E-25	0.238	0.435	1.1E-04	2.9E-04	2.5E-04
KIRC	POFUT2_AD_8.2:8.3_8.1_8.5	POFUT2	AD	8.2:8.3	8.1	8.5	1.1E-24	0.231	0.474	2.4E-03	7.7E-03	3.2E-03

cancer type	id	Gene Symbol	splice_type	Exon	From.Exon	To.Exon	anova.p	adj.r2	r	p.50	p.25	p.10
KIRC	POFUT2_ES_8.3_8.1_8.5	POFUT2	ES	8.3	8.1	8.5	2.6E-17	0.169	0.410	3.9E-02	9.3E-04	1.1E-03
KIRC	POFUT2_RI_8.2:8.3:8.4_8.1_8.5	POFUT2	RI	8.2:8.3:8.4	8.1	8.5	6.1E-31	0.281	0.479	1.7E-02	8.5E-04	9.9E-04
KIRC	POGLUT1_ES_5:6.1_4_6.2	POGLUT1	ES	05:06.1	4	6.2	6.3E-16	0.158	0.053	1.6E-01	1.6E-01	9.8E-03
KIRC	POLL_AD_5.2_5.1_6.1	POLL	AD	5.2	5.1	6.1	1.0E-16	0.162	0.058	7.3E-07	3.8E-08	2.4E-04
KIRC	POLL_RI_6.2_6.1_6.3	POLL	RI	6.2	6.1	6.3	1.2E-35	0.317	0.199	5.2E-05	6.1E-06	1.3E-02
KIRC	POLM_ES_6.2:6.3:7:8_6.1_9.1	POLM	ES	6.2:6.3:7:8	6.1	9.1	1.1E-11	0.128	0.439	3.9E-03	5.8E-04	1.9E-02
KIRC	POLM_RI_9.3_9.2_9.4	POLM	RI	9.3	9.2	9.4	2.3E-18	0.180	0.483	3.9E-04	8.6E-05	1.7E-03
KIRC	POLM_RI_9.6_9.5_9.7	POLM	RI	9.6	9.5	9.7	3.5E-16	0.159	0.531	6.6E-04	8.3E-05	5.9E-03
KIRC	POLR2G_AD_2.5_2.4_3	POLR2G	AD	2.5	2.4	3	1.7E-15	0.155	-0.175	2.4E-02	1.1E-01	1.2E-01
KIRC	POLR2G_RI_2.2:2.3_2.1_2.4	POLR2G	RI	2.2:2.3	2.1	2.4	1.9E-16	0.160	-0.173	3.4E-03	7.6E-02	1.2E-01
KIRC	POLR2J3_AA_4.1:4.2_2_4.3	POLR2J3	AA	4.1:4.2	2	4.3	3.5E-14	0.138	-0.064	2.4E-01	7.8E-03	2.0E-01
KIRC	POLR2J3_ES_6:7_4.3_8	POLR2J3	ES	6:07	4.3	8	8.9E-22	0.207	0.399	8.9E-06	2.5E-05	8.9E-04
KIRC	POLR2J3_RI_4.2_4.1_4.3	POLR2J3	RI	4.2	4.1	4.3	6.0E-20	0.190	0.058	5.3E-05	1.1E-03	5.2E-02
KIRC	POLR2J3_RI_4.4_4.3_4.5	POLR2J3	RI	4.4	4.3	4.5	1.5E-16	0.160	0.074	1.3E-05	2.4E-03	1.9E-02
KIRC	POM121_ES_4_3_6	POM121	ES	4	3	6	2.9E-14	0.143	-0.096	4.8E-02	1.5E-03	4.0E-02
KIRC	POMGNT1_RI_12.2_12.1_12.3	POMGNT1	RI	12.2	12.1	12.3	1.0E-37	0.331	0.044	2.5E-05	1.3E-05	7.5E-03
KIRC	POMGNT1_RI_12.4_12.3_12.5	POMGNT1	RI	12.4	12.3	12.5	2.6E-32	0.291	0.061	2.6E-04	1.2E-04	1.1E-02
KIRC	POMT1_AD_8.2_8.1_9	POMT1	AD	8.2	8.1	9	6.8E-29	0.269	0.131	6.6E-05	9.3E-07	5.4E-05
KIRC	PON2_AD_3.3_3.2_4	PON2	AD	3.3	3.2	4	6.7E-35	0.311	-0.132	2.9E-03	1.4E-04	1.4E-01
KIRC	PPIA_ES_5.1:6_4_7	PPIA	ES	5.1:6	4	7	3.5E-09	0.114	-0.048	1.2E-01	1.2E-02	6.6E-02
KIRC	PPIE_AD_7.2_7.1_8	PPIE	AD	7.2	7.1	8	7.5E-14	0.135	0.229	1.0E-02	2.2E-05	1.4E-02
KIRC	PPIL3_ES_1.2:2.2:3:4.1_1.1_4.2	PPIL3	ES	1.2:2.2:3:4.1	1.1	4.2	1.2E-18	0.184	0.114	2.6E-01	2.6E-02	4.8E-01
KIRC	PPIL3_ES_2.2:3:4.1_1.1_4.2	PPIL3	ES	2.2:3:4.1	1.1	4.2	1.3E-16	0.166	0.244	2.2E-03	1.1E-02	1.9E-02
KIRC	PPIL6_ES_7_6_9	PPIL6	ES	7	6	9	6.8E-16	0.156	-0.246	5.3E-02	5.7E-02	7.8E-02
KIRC	PPIP5K1_ES_31_30_32	PPIP5K1	ES	31	30	32	3.6E-19	0.184	-0.243	7.9E-06	1.0E-06	7.1E-03
KIRC	PPIP5K2_ES_28:29_27_30	PPIP5K2	ES	28:29:00	27	30	9.3E-25	0.234	0.233	8.2E-05	1.8E-04	6.4E-04
KIRC	PPIP5K2_ES_29_28_30	PPIP5K2	ES	29	28	30	1.2E-13	0.135	0.068	2.6E-02	4.0E-02	1.1E-01
KIRC	PPM1M_RI_10.2_10.1_10.3	PPM1M	RI	10.2	10.1	10.3	2.2E-27	0.253	0.298	2.9E-06	5.2E-07	1.2E-03
KIRC	PPP2R4_ES_4:5:6:7:8:9:10_3.1_15.1	PPP2R4	ES	4:5:6:7:8:9:10	3.1	15.1	9.9E-11	0.106	-0.448	1.3E-02	1.9E-01	9.1E-02
KIRC	PPP4C_ES_5.1:5.2_4_6	PPP4C	ES	5.1:5.2	4	6	1.1E-20	0.207	-0.069	2.1E-02	5.8E-02	7.7E-04
KIRC	PPP4R1_ES_15_14_16	PPP4R1	ES	15	14	16	2.0E-16	0.160	-0.048	3.4E-02	2.2E-03	4.3E-03
KIRC	PPP4R1_ES_5_4.2_6	PPP4R1	ES	5	4.2	6	1.2E-29	0.277	-0.193	1.3E-04	2.1E-06	5.5E-05
KIRC	PPWD1_ES_4_3_5.1	PPWD1	ES	4	3	5.1	4.6E-21	0.211	0.425	2.1E-04	1.2E-03	1.6E-03
KIRC	PQLC1_ES_4.1:4.2_2_5	PQLC1	ES	4.1:4.2	2	5	7.5E-30	0.272	0.036	1.4E-06	2.9E-06	3.2E-04
KIRC	PRICKLE4_AA_4.1_3_4.2	PRICKLE4	AA	4.1	3	4.2	4.8E-11	0.111	0.283	3.1E-08	5.6E-08	1.2E-04
KIRC	PRKAB1_ES_3_1.3_4	PRKAB1	ES	3	1.3	4	1.0E-21	0.206	-0.268	1.6E-01	7.0E-02	2.7E-01
KIRC	PRKAR1A_RI_3.3_3.2_3.4	PRKAR1A	RI	3.3	3.2	3.4	2.4E-15	0.169	-0.354	1.4E-01	1.1E-02	1.4E-01
KIRC	PRMT2_AD_1.2:1.3_1.1_2	PRMT2	AD	1.2:1.3	1.1	2	4.1E-21	0.201	0.152	1.4E-05	6.8E-04	6.0E-04
KIRC	PRMT2_RI_1.2_1.1_1.3	PRMT2	RI	1.2	1.1	1.3	4.0E-33	0.298	0.089	5.7E-07	2.1E-05	1.8E-03
KIRC	PRMT7_AA_17.1_16_17.2	PRMT7	AA	17.1	16	17.2	4.2E-17	0.166	0.058	2.1E-02	3.3E-02	6.8E-02

cancer type	id	Gene Symbol	splice_type	Exon	From.Exon	To.Exon	anova.p	adj.r2	r	p.50	p.25	p.10
KIRC	PRPF3_ES_4_3_5	PRPF3	ES	4	3	5	2.3E-20	0.196	0.718	7.0E-03	1.4E-01	2.7E-01
KIRC	PRPF38B_ES_2_1_3	PRPF38B	ES	2	1	3	2.7E-14	0.141	0.290	3.8E-03	1.4E-03	2.6E-03
KIRC	PRPF39_ES_10_9_11	PRPF39	ES	10	9	11	6.2E-14	0.137	0.676	3.9E-03	4.1E-03	2.8E-03
KIRC	PRPF39_ES_4.1:4.2:4.3_3_5	PRPF39	ES	4.1:4.2:4.3	3	5	4.9E-17	0.166	0.697	3.6E-05	2.8E-04	1.7E-02
KIRC	PRPF40A_ES_8_7_9	PRPF40A	ES	8	7	9	1.3E-10	0.112	0.016	8.3E-05	3.8E-05	2.1E-05
KIRC	PRRC2C_ES_34_33_35	PRRC2C	ES	34	33	35	1.1E-17	0.171	0.058	5.3E-04	6.3E-03	4.2E-02
KIRC	PSIP1_AA_11.1_10_11.2	PSIP1	AA	11.1	10	11.2	1.2E-11	0.115	-0.071	6.4E-03	7.7E-03	1.1E-03
KIRC	PSMC3IP_AD_4.3_4.2_5.1	PSMC3IP	AD	4.3	4.2	5.1	9.2E-14	0.141	0.058	9.5E-04	8.2E-05	7.9E-03
KIRC	PSMC5_RI_2.2_2.1_2.3	PSMC5	RI	2.2	2.1	2.3	3.5E-26	0.244	0.116	8.8E-03	2.2E-04	3.3E-04
KIRC	PSME1_RI_10.3_10.2_10.4	PSME1	RI	10.3	10.2	10.4	1.0E-18	0.180	-0.001	1.3E-05	5.5E-06	4.5E-04
KIRC	PSTK_AD_4.4:4.5_4.3_5	PSTK	AD	4.4:4.5	4.3	5	7.9E-11	0.141	-0.332	7.2E-01	4.3E-01	7.3E-01
KIRC	PTBP1_ES_3:4:5:6:7:8:9.2:10_2_11	PTBP1	ES	3:4:5:6:7:8:9.2:1(	2	11	4.2E-16	0.181	-0.217	1.1E-02	4.1E-03	3.2E-01
KIRC	PTBP2_ES_12_11.2_13	PTBP2	ES	12	11.2	13	3.7E-22	0.223	-0.080	8.3E-06	4.8E-03	3.8E-02
KIRC	PTCD2_AD_1.2_1.1_2	PTCD2	AD	1.2	1.1	2	2.2E-22	0.211	-0.582	1.8E-04	2.1E-07	4.0E-04
KIRC	PTGR1_AA_12.1_11_12.2	PTGR1	AA	12.1	11	12.2	6.3E-30	0.273	-0.588	7.5E-04	2.6E-05	2.4E-01
KIRC	PTK2_ES_39.2_37_39.5	PTK2	ES	39.2	37	39.5	1.4E-20	0.196	-0.134	1.8E-06	9.7E-05	1.3E-01
KIRC	PTK2_RI_39.3:39.4_39.2_39.5	PTK2	RI	39.3:39.4	39.2	39.5	4.5E-31	0.282	-0.158	5.5E-07	1.1E-05	1.6E-03
KIRC	PTK2B_RI_13.2_13.1_13.3	PTK2B	RI	13.2	13.1	13.3	6.9E-25	0.239	0.055	5.8E-06	1.6E-03	3.3E-03
KIRC	PTOV1_RI_13.2_13.1_13.3	PTOV1	RI	13.2	13.1	13.3	6.7E-21	0.200	-0.201	4.5E-04	2.3E-02	5.0E-02
KIRC	PTPRE_AD_17.2_17.1_18	PTPRE	AD	17.2	17.1	18	4.7E-27	0.252	0.024	1.6E-05	3.6E-06	1.7E-03
KIRC	PTPRO_RI_26.2_26.1_26.3	PTPRO	RI	26.2	26.1	26.3	8.1E-16	0.156	-0.337	2.2E-04	4.4E-04	1.9E-02
KIRC	PUS7_ES_15_14_16	PUS7	ES	15	14	16	2.4E-16	0.159	-0.351	5.0E-03	9.2E-04	5.1E-02
KIRC	PXN_AA_14.1_9_14.2	PXN	AA	14.1	9	14.2	2.5E-15	0.150	0.156	3.3E-07	2.2E-04	4.1E-03
KIRC	PYROXD1_ES_4_3_5	PYROXD1	ES	4	3	5	5.8E-33	0.308	-0.095	9.5E-04	1.4E-03	1.3E-04
KIRC	QKI_RI_8.3_8.2_8.4	QKI	RI	8.3	8.2	8.4	4.3E-13	0.131	-0.272	2.0E-03	6.5E-06	1.8E-04
KIRC	QKI_RI_8.3:8.4_8.2_8.5	QKI	RI	8.3:8.4	8.2	8.5	9.5E-25	0.233	-0.403	1.4E-04	1.5E-05	2.4E-04
KIRC	QTRT1_RI_6.2_6.1_6.3	QTRT1	RI	6.2	6.1	6.3	2.1E-25	0.237	0.417	1.1E-03	1.8E-04	3.3E-02
KIRC	R3HDM4_ES_2:3.1:3.2_1_3.3	R3HDM4	ES	2:3.1:3.2	1	3.3	1.2E-14	0.144	0.078	6.1E-02	3.6E-02	7.8E-02
KIRC	R3HDM4_RI_3.2_3.1_3.3	R3HDM4	RI	3.2	3.1	3.3	9.4E-13	0.138	0.311	5.6E-03	3.6E-04	9.9E-05
KIRC	RAB15_AD_6.2:6.3_6.1_7	RAB15	AD	6.2:6.3	6.1	7	9.0E-23	0.236	-0.245	9.5E-02	2.5E-02	6.1E-01
KIRC	RAB3GAP1_ES_4:5_3.1_6	RAB3GAP1	ES	4:05	3.1	6	7.0E-37	0.325	-0.691	4.9E-06	1.4E-04	2.1E-01
KIRC	RAB3IP_AD_8.2_8.1_9.1	RAB3IP	AD	8.2	8.1	9.1	6.9E-17	0.163	-0.326	1.0E-05	1.4E-05	6.5E-03
KIRC	RAB40B_ES_2_1_3	RAB40B	ES	2	1	3	4.9E-12	0.119	0.017	5.2E-01	9.4E-01	4.1E-01
KIRC	RAB40C_ES_6_5_7	RAB40C	ES	6	5	7	1.3E-23	0.223	0.199	4.1E-04	1.1E-04	1.0E-04
KIRC	RAB5C_ES_2_1_4	RAB5C	ES	2	1	4	2.4E-29	0.268	-0.649	2.3E-05	2.4E-05	1.3E-04
KIRC	RABEP2_ES_3_2.2_4	RABEP2	ES	3	2.2	4	1.3E-22	0.214	0.241	2.0E-05	1.2E-04	1.1E-02
KIRC	RABGGTB_ES_5:6_4.1_7	RABGGTB	ES	5:06	4.1	7	1.1E-24	0.231	-0.157	8.1E-05	4.1E-07	1.5E-06
KIRC	RABL2A_ES_2_1_3.1	RABL2A	ES	2	1	3.1	8.7E-15	0.159	0.390	2.7E-03	7.7E-04	1.6E-02
KIRC	RABL2A_ES_2:3.1_1_3.2	RABL2A	ES	02:03.1	1	3.2	3.8E-20	0.203	0.478	2.6E-03	1.6E-03	3.3E-03
KIRC	RABL2B_AD_2.3_2.2_3.1	RABL2B	AD	2.3	2.2	3.1	1.4E-12	0.127	0.275	4.0E-07	2.8E-07	2.2E-03

cancer type	id	Gene Symbol	splice_type	Exon	From.Exon	To.Exon	anova.p	adj.r2	r	p.50	p.25	p.10
KIRC	RABL2B_ES_2.1:2.2:2.3_1_3.1	RABL2B	ES	2.1:2.2:2.3	1	3.1	1.7E-14	0.145	0.367	2.9E-04	2.7E-04	1.2E-03
KIRC	RABL2B_ES_2.1:2.2:2.3:3.1_1_3.2	RABL2B	ES	2.1:2.2:2.3:3.1	1	3.2	1.3E-17	0.173	0.397	6.3E-04	6.6E-05	1.1E-03
KIRC	RAD51_ME_4|5_3_6	RAD51	ME	4|5	3	6	8.9E-25	0.245	-0.371	1.8E-01	2.9E-01	1.7E-01
KIRC	RAD52_AA_12.1_11_12.2	RAD52	AA	12.1	11	12.2	1.3E-09	0.116	0.475	1.5E-03	1.9E-04	2.9E-03
KIRC	RAD52_AD_7.2_7.1_8	RAD52	AD	7.2	7.1	8	3.9E-08	0.100	0.406	3.5E-02	3.8E-02	5.6E-02
KIRC	RAD52_ES_9_8_10	RAD52	ES	9	8	10	1.3E-11	0.134	0.336	1.1E-06	2.8E-06	6.9E-05
KIRC	RAI14_ES_18_17_19	RAI14	ES	18	17	19	3.7E-10	0.101	-0.106	9.3E-02	1.5E-01	9.1E-03
KIRC	RANBP1_RI_7.2:7.3_7.1_7.4	RANBP1	RI	7.2:7.3	7.1	7.4	3.7E-31	0.282	-0.165	1.4E-03	1.1E-04	3.6E-02
KIRC	RANBP3_AA_10.1_8_10.2	RANBP3	AA	10.1	8	10.2	1.2E-29	0.271	0.414	5.9E-07	3.0E-06	6.0E-03
KIRC	RANBP3_ES_2_1_4.1	RANBP3	ES	2	1	4.1	2.6E-32	0.291	0.450	1.4E-06	2.1E-05	2.1E-04
KIRC	RAP1B_ES_2_1.1_3.1	RAP1B	ES	2	1.1	3.1	6.3E-22	0.207	-0.007	5.8E-06	2.9E-05	1.0E-03
KIRC	RAP1GAP_RI_22.2:22.3_22.1_22.4	RAP1GAP	RI	22.2:22.3	22.1	22.4	2.0E-12	0.155	-0.298	1.3E-02	1.8E-04	8.1E-03
KIRC	RASA1_ES_14_13_15	RASA1	ES	14	13	15	4.5E-23	0.226	-0.259	1.6E-05	5.4E-03	2.2E-04
KIRC	RASSF7_AA_6.3:6.4_6.1_6.5	RASSF7	AA	6.3:6.4	6.1	6.5	3.3E-11	0.111	0.186	3.6E-04	2.8E-03	7.0E-03
KIRC	RBM14_ES_3_1_4.1	RBM14	ES	3	1	4.1	1.3E-26	0.252	0.331	6.5E-04	1.2E-03	9.3E-02
KIRC	RBM3_RI_3.2_3.1_3.3	RBM3	RI	3.2	3.1	3.3	1.9E-38	0.336	-0.243	2.9E-05	1.0E-05	1.4E-06
KIRC	RBM39_ES_4_3_6	RBM39	ES	4	3	6	4.8E-21	0.200	0.669	2.0E-04	6.1E-05	9.4E-05
KIRC	RBM39_ES_4:5_3_6	RBM39	ES	4:05	3	6	4.6E-31	0.305	0.706	1.8E-08	4.9E-06	2.9E-04
KIRC	RBM39_ES_5_4_6	RBM39	ES	5	4	6	1.1E-14	0.156	0.484	1.2E-03	2.0E-03	3.8E-02
KIRC	RBM47_ES_8.1:8.2_7.2_9	RBM47	ES	8.1:8.2	7.2	9	1.7E-22	0.271	-0.416	1.5E-04	7.9E-04	6.7E-03
KIRC	RBM48_RI_4.2_4.1_4.3	RBM48	RI	4.2	4.1	4.3	2.4E-35	0.313	0.185	2.8E-06	1.8E-05	1.9E-03
KIRC	RBM5_RI_6.2_6.1_6.3	RBM5	RI	6.2	6.1	6.3	1.1E-24	0.233	0.646	2.9E-05	1.2E-03	4.7E-03
KIRC	RBM6_RI_14.2_14.1_14.3	RBM6	RI	14.2	14.1	14.3	8.2E-23	0.215	0.795	2.4E-06	1.6E-03	6.6E-03
KIRC	RBM7_RI_4.3_4.2_4.4	RBM7	RI	4.3	4.2	4.4	1.0E-36	0.323	-0.746	7.3E-10	1.4E-07	2.0E-02
KIRC	RBMX_RI_8.2_8.1_8.3	RBMX	RI	8.2	8.1	8.3	1.6E-27	0.254	0.104	2.5E-03	2.4E-05	5.0E-02
KIRC	RBMX_RI_8.2:8.3_8.1_8.4	RBMX	RI	8.2:8.3	8.1	8.4	4.9E-14	0.137	-0.043	1.3E-03	5.0E-03	2.9E-02
KIRC	RBP7_ES_2_1_3	RBP7	ES	2	1	3	4.7E-18	0.174	-0.566	1.4E-08	2.1E-08	8.9E-07
KIRC	RCAN1_ES_6_5_7.2	RCAN1	ES	6	5	7.2	1.2E-17	0.171	-0.087	2.5E-06	3.6E-06	2.4E-03
KIRC	RCOR3_ES_5_4_6	RCOR3	ES	5	4	6	4.9E-23	0.220	0.121	2.0E-02	4.1E-04	8.6E-03
KIRC	RDH13_AD_9.2_9.1_10	RDH13	AD	9.2	9.1	10	1.7E-18	0.182	-0.103	4.6E-05	1.2E-04	1.2E-03
KIRC	RDH13_ES_8_7_9.1	RDH13	ES	8	7	9.1	5.8E-20	0.196	-0.056	2.8E-01	1.4E-01	1.2E-02
KIRC	REEP5_ES_4:5_3_6	REEP5	ES	4:05	3	6	1.1E-11	0.115	-0.333	5.9E-02	2.3E-01	9.2E-01
KIRC	RELA_RI_10.2_10.1_10.3	RELA	RI	10.2	10.1	10.3	4.0E-24	0.226	0.054	8.5E-06	1.8E-06	2.7E-04
KIRC	RFC5_AD_2.2_2.1_3	RFC5	AD	2.2	2.1	3	3.4E-13	0.146	-0.062	2.8E-04	4.1E-05	5.2E-04
KIRC	RFC5_ES_2.1:2.2_1_3	RFC5	ES	2.1:2.2	1	3	1.1E-21	0.210	-0.038	3.4E-05	1.2E-05	1.2E-05
KIRC	RFX5_ES_7_6.2_8	RFX5	ES	7	6.2	8	7.8E-34	0.302	-0.080	2.9E-04	8.6E-04	1.5E-02
KIRC	RHBDF1_RI_11.2_11.1_11.3	RHBDF1	RI	11.2	11.1	11.3	3.2E-20	0.198	0.290	2.1E-05	5.3E-04	1.8E-03
KIRC	RHOC_AA_2.1:2.2_1.1_2.3	RHOC	AA	2.1:2.2	1.1	2.3	2.1E-17	0.168	-0.383	2.0E-03	1.0E-03	1.3E-01
KIRC	RHOT1_ES_21_20_22	RHOT1	ES	21	20	22	2.5E-14	0.141	-0.275	1.6E-03	3.0E-04	3.5E-04
KIRC	RHOT2_RI_9.4_9.3_9.5	RHOT2	RI	9.4	9.3	9.5	8.3E-31	0.281	0.716	4.1E-07	6.4E-09	2.1E-06

cancer type	id	Gene Symbol	splice_type	Exon	From.Exon	To.Exon	anova.p	adj.r2	r	p.50	p.25	p.10
KIRC	RIC8B_ES_13:14:15:16_11_17	RIC8B	ES	13:14:15:16	11	17	4.8E-14	0.139	-0.009	5.0E-05	4.8E-04	2.1E-02
KIRC	RIC8B_ES_14:15:16_13_17	RIC8B	ES	14:15:16	13	17	3.0E-15	0.154	-0.061	2.3E-05	5.0E-04	4.0E-04
KIRC	RIPK3_AD_5.2_5.1_6	RIPK3	AD	5.2	5.1	6	1.6E-18	0.209	0.346	1.6E-04	1.7E-07	7.8E-04
KIRC	RMDN1_ES_6_4_7	RMDN1	ES	6	4	7	7.6E-27	0.248	-0.286	4.1E-04	1.7E-03	1.4E-04
KIRC	RMND5B_AD_4.2_4.1_5.1	RMND5B	AD	4.2	4.1	5.1	2.8E-11	0.114	-0.076	1.0E-01	1.1E-01	5.2E-02
KIRC	RNASE4_ES_3_1.2_4	RNASE4	ES	3	1.2	4	3.3E-19	0.184	-0.204	2.3E-01	7.6E-02	1.0E-01
KIRC	RNF121_ES_4_3_6.1	RNF121	ES	4	3	6.1	2.9E-17	0.167	-0.226	1.9E-03	1.9E-03	6.7E-02
KIRC	RNF123_AD_27.2_27.1_28	RNF123	AD	27.2	27.1	28	1.2E-17	0.173	0.103	1.6E-04	8.1E-04	1.3E-03
KIRC	RNF146_ES_3_2_5.1	RNF146	ES	3	2	5.1	1.8E-11	0.119	0.110	1.5E-01	1.1E-02	1.4E-01
KIRC	RNF146_ES_4_2_5.1	RNF146	ES	4	2	5.1	2.6E-13	0.132	0.102	1.4E-03	5.7E-04	4.2E-02
KIRC	RNF166_RI_7.2_7.1_7.3	RNF166	RI	7.2	7.1	7.3	3.4E-32	0.291	0.494	7.5E-05	1.1E-04	3.0E-02
KIRC	RNF216_AA_6.1_5.2_6.2	RNF216	AA	6.1	5.2	6.2	5.7E-33	0.300	0.023	2.2E-06	4.2E-06	2.1E-01
KIRC	RNPC3_AA_15.1_14_15.2	RNPC3	AA	15.1	14	15.2	3.6E-15	0.148	0.662	4.6E-02	1.2E-01	8.0E-01
KIRC	RNPS1_AD_2.2:2.3:2.4_2.1_3	RNPS1	AD	2.2:2.3:2.4	2.1	3	5.7E-17	0.182	-0.006	9.3E-03	1.4E-02	1.4E-01
KIRC	ROGDI_AD_4.2_4.1_5	ROGDI	AD	4.2	4.1	5	1.9E-28	0.262	0.216	4.6E-05	8.5E-05	3.9E-03
KIRC	ROGDI_ES_4.1:4.2_3_5	ROGDI	ES	4.1:4.2	3	5	6.0E-09	0.106	0.042	1.4E-03	2.3E-03	3.7E-05
KIRC	RPAP1_ES_22.2:23.1_22.1_23.2	RPAP1	ES	22.2:23.1	22.1	23.2	3.5E-23	0.218	-0.232	1.0E-02	9.9E-06	1.7E-04
KIRC	RPE_ES_8_7_10.1	RPE	ES	8	7	10.1	3.1E-32	0.290	-0.468	1.2E-04	1.1E-04	3.1E-02
KIRC	RPGR_ES_14.1:14.2:14.3_13_16	RPGR	ES	14.1:14.2:14.3	13	16	9.5E-10	0.112	0.038	3.3E-01	3.6E-01	4.4E-03
KIRC	RPGR_RI_14.2_14.1_14.3	RPGR	RI	14.2	14.1	14.3	1.0E-09	0.101	-0.030	4.3E-02	3.7E-02	8.7E-02
KIRC	RPL22L1_AA_3.1_2.2_3.2	RPL22L1	AA	3.1	2.2	3.2	4.1E-16	0.156	-0.487	1.2E-05	6.3E-07	7.2E-06
KIRC	RPL23_AA_5.1:5.2_4_5.3	RPL23	AA	5.1:5.2	4	5.3	4.4E-14	0.147	0.006	2.7E-03	9.7E-05	1.6E-03
KIRC	RPL28_RI_2.3_2.2_2.4	RPL28	RI	2.3	2.2	2.4	8.6E-12	0.127	-0.104	9.9E-04	4.2E-06	6.2E-04
KIRC	RPL30_ES_2.2:3.1_2.1_3.2	RPL30	ES	2.2:3.1	2.1	3.2	4.1E-11	0.109	-0.193	4.5E-01	4.8E-01	4.4E-01
KIRC	RPL32_RI_1.2_1.1_1.3	RPL32	RI	1.2	1.1	1.3	2.4E-14	0.140	-0.154	3.2E-06	4.1E-06	4.1E-05
KIRC	RPL32_RI_1.2:1.3_1.1_1.4	RPL32	RI	1.2:1.3	1.1	1.4	4.4E-17	0.165	-0.106	3.9E-06	1.2E-04	1.4E-03
KIRC	RPL35_AD_4.2_4.1_5	RPL35	AD	4.2	4.1	5	3.5E-11	0.114	-0.270	8.8E-03	6.5E-04	2.1E-01
KIRC	RPL6_ES_1.2:2:3:4_1.1_5	RPL6	ES	1.2:2:3:4	1.1	5	9.0E-11	0.108	-0.325	5.7E-01	6.4E-01	5.5E-01
KIRC	RPLP0_AA_5.1:5.2_4.2_5.3	RPLP0	AA	5.1:5.2	4.2	5.3	4.9E-11	0.111	-0.076	2.4E-01	3.0E-01	1.0E-01
KIRC	RPS21_AA_3.3:3.4_3.1_3.5	RPS21	AA	3.3:3.4	3.1	3.5	1.4E-14	0.143	-0.254	2.4E-04	4.3E-05	1.9E-05
KIRC	RPS21_AD_3.2:3.3_3.1_3.5	RPS21	AD	3.2:3.3	3.1	3.5	6.7E-17	0.164	-0.230	3.9E-07	8.6E-10	3.1E-06
KIRC	RPS24_AA_5.1_4_5.2	RPS24	AA	5.1	4	5.2	8.7E-25	0.231	-0.116	7.9E-05	4.5E-07	1.7E-05
KIRC	RPS24_ES_5.1:5.2_4_6	RPS24	ES	5.1:5.2	4	6	6.6E-19	0.181	-0.089	2.8E-03	9.9E-06	1.7E-03
KIRC	RPS25_ES_2.2:3.1_2.1_3.2	RPS25	ES	2.2:3.1	2.1	3.2	1.3E-12	0.124	-0.135	3.6E-01	7.1E-01	4.0E-01
KIRC	RPS6_AA_1.4:1.5_1.1_1.6	RPS6	AA	1.4:1.5	1.1	1.6	1.3E-19	0.188	-0.203	6.4E-06	2.8E-06	1.7E-03
KIRC	RPS6_ES_1.2:1.3:1.4:1.6:2:3_1.1_4	RPS6	ES	.2:1.3:1.4:1.6:2:	1.1	4	1.6E-14	0.147	-0.222	3.1E-03	9.6E-03	9.0E-04
KIRC	RPS6_ES_1.4:1.5:1.6:2:3_1.1_4	RPS6	ES	1.4:1.5:1.6:2:3	1.1	4	3.8E-16	0.163	-0.331	8.3E-03	8.4E-03	4.6E-03
KIRC	RPS6_ES_1.5:1.6:2:3_1.4_4	RPS6	ES	1.5:1.6:2:3	1.4	4	7.2E-13	0.139	-0.274	4.0E-03	3.7E-03	3.8E-04
KIRC	RPS6KA5_ES_3_1_4	RPS6KA5	ES	3	1	4	5.4E-23	0.231	-0.592	1.6E-04	1.7E-03	2.8E-01
KIRC	RPS6KB1_ES_9_7_10	RPS6KB1	ES	9	7	10	3.1E-17	0.172	0.012	5.3E-03	4.0E-03	1.2E-01

cancer type	id	Gene Symbol	splice_type	Exon	From.Exon	To.Exon	anova.p	adj.r2	r	p.50	p.25	p.10
KIRC	RPS6KB2_ES_6.1:6.2_5.1_7	RPS6KB2	ES	6.1:6.2	5.1	7	7.6E-23	0.216	0.031	3.9E-01	5.5E-01	2.3E-01
KIRC	RPS9_AA_4.1:4.2:4.3:4.4_3_4.5	RPS9	AA	4.1:4.2:4.3:4.4	3	4.5	3.4E-15	0.148	-0.316	7.8E-06	3.0E-06	5.8E-04
KIRC	RPS9_ES_4.1:4.2:4.3_3_4.5	RPS9	ES	4.1:4.2:4.3	3	4.5	2.6E-10	0.102	-0.245	1.3E-03	7.0E-03	1.5E-01
KIRC	RPS9_ES_4.1:4.3:4.4_3_4.5	RPS9	ES	4.1:4.3:4.4	3	4.5	1.5E-13	0.132	-0.270	6.2E-04	1.4E-04	4.9E-02
KIRC	RPS9_RI_4.2_4.1_4.3	RPS9	RI	4.2	4.1	4.3	3.2E-13	0.130	-0.310	4.6E-07	4.6E-06	1.3E-03
KIRC	RPS9_RI_4.4_4.3_4.5	RPS9	RI	4.4	4.3	4.5	8.4E-16	0.153	-0.314	6.0E-08	4.4E-09	4.8E-04
KIRC	RQCD1_ES_2_1_3	RQCD1	ES	2	1	3	1.0E-29	0.275	-0.545	9.9E-04	2.8E-03	1.0E-03
KIRC	RQCD1_ES_7_6_8	RQCD1	ES	7	6	8	9.7E-30	0.271	-0.637	2.4E-03	1.9E-04	5.3E-03
KIRC	RRM2B_ES_2_1_3.1	RRM2B	ES	2	1	3.1	2.5E-30	0.276	-0.490	9.0E-05	8.9E-07	5.8E-05
KIRC	RRN3_ES_12:13_11.1_14	RRN3	ES	12:13	11.1	14	6.7E-29	0.296	0.307	4.8E-03	2.1E-03	3.7E-04
KIRC	RRNAD1_RI_2.2_2.1_2.3	RRNAD1	RI	2.2	2.1	2.3	6.3E-17	0.164	0.050	4.9E-02	5.6E-02	5.9E-01
KIRC	RRP8_AA_2.1_1_2.2	RRP8	AA	2.1	1	2.2	5.6E-15	0.151	0.143	3.4E-04	3.9E-04	2.8E-04
KIRC	RRP8_RI_3.2_3.1_3.3	RRP8	RI	3.2	3.1	3.3	1.1E-11	0.116	-0.098	3.8E-01	7.1E-01	5.5E-01
KIRC	RSAD1_AD_8.2_8.1_9	RSAD1	AD	8.2	8.1	9	4.2E-35	0.314	0.271	2.3E-05	7.9E-07	5.2E-06
KIRC	RUSC1_RI_3.3_3.2_3.4	RUSC1	RI	3.3	3.2	3.4	1.6E-10	0.104	0.163	7.9E-02	2.1E-02	2.3E-02
KIRC	RUVBL2_AD_1.3:1.4:1.5:1.6_1.2_2	RUVBL2	AD	1.3:1.4:1.5:1.6	1.2	2	3.5E-13	0.129	0.074	1.7E-02	1.4E-01	1.9E-01
KIRC	RUVBL2_ES_3_2_4	RUVBL2	ES	3	2	4	4.5E-28	0.258	0.182	1.8E-03	3.7E-05	1.5E-04
KIRC	RWDD2A_RI_2.3_2.2_2.4	RWDD2A	RI	2.3	2.2	2.4	1.5E-30	0.278	-0.062	1.0E-06	1.2E-08	2.5E-06
KIRC	SAMD9L_RI_3.2_3.1_3.3	SAMD9L	RI	3.2	3.1	3.3	4.2E-12	0.121	0.130	9.8E-03	4.7E-06	8.2E-05
KIRC	SAR1B_ES_3_2_4	SAR1B	ES	3	2	4	2.7E-11	0.111	-0.364	4.7E-01	6.5E-02	2.1E-02
KIRC	SAR1B_ES_6_5.2_8	SAR1B	ES	6	5.2	8	2.0E-13	0.131	-0.363	5.9E-03	4.8E-02	9.4E-05
KIRC	SAT1_AD_2.2_2.1_3	SAT1	AD	2.2	2.1	3	1.6E-10	0.112	0.056	2.0E-03	1.2E-01	2.9E-01
KIRC	SCARB1_AD_13.3_13.2_15	SCARB1	AD	13.3	13.2	15	1.1E-23	0.227	-0.037	1.8E-01	1.2E-02	2.5E-03
KIRC	SCNN1A_ES_10_9_11	SCNN1A	ES	10	9	11	9.3E-16	0.179	-0.453	2.7E-04	1.1E-05	4.9E-03
KIRC	SCP2_ES_12_11_13	SCP2	ES	12	11	13	1.7E-26	0.245	-0.583	1.4E-09	2.4E-08	2.4E-03
KIRC	SCRN2_AA_4.1_3_4.2	SCRN2	AA	4.1	3	4.2	3.8E-29	0.268	-0.023	1.8E-05	4.5E-06	2.0E-04
KIRC	SCRN2_RI_7.2_7.1_7.3	SCRN2	RI	7.2	7.1	7.3	5.7E-15	0.146	-0.085	1.8E-01	5.3E-01	1.5E-01
KIRC	SCYL3_ES_12_11_13	SCYL3	ES	12	11	13	5.0E-16	0.157	0.055	7.7E-02	1.4E-02	1.1E-01
KIRC	SDC3_RI_2.2_2.1_2.3	SDC3	RI	2.2	2.1	2.3	8.5E-27	0.250	-0.030	3.2E-07	1.1E-06	3.7E-07
KIRC	SDSL_ES_2_1_3	SDSL	ES	2	1	3	7.4E-26	0.241	-0.238	1.6E-03	3.4E-02	3.0E-02
KIRC	SEC16A_RI_23.10_23.9_23.11	SEC16A	RI	23.1	23.9	23.11	3.8E-17	0.182	-0.098	7.0E-05	1.4E-02	1.7E-01
KIRC	SEC24C_AA_23.1:23.2_22_23.3	SEC24C	AA	23.1:23.2	22	23.3	2.3E-13	0.135	0.039	3.6E-01	8.8E-01	4.5E-01
KIRC	SEC24C_RI_23.2_23.1_23.3	SEC24C	RI	23.2	23.1	23.3	8.1E-17	0.163	0.041	9.9E-01	1.6E-01	9.2E-01
KIRC	SEC24D_ES_27_26_28	SEC24D	ES	27	26	28	9.2E-22	0.206	-0.580	1.2E-03	8.9E-04	4.2E-02
KIRC	SEC31A_AD_10.2_10.1_11	SEC31A	AD	10.2	10.1	11	1.6E-32	0.292	-0.432	6.0E-05	8.8E-05	2.6E-03
KIRC	SEC31A_ES_26.1:26.2:27_25.1_28	SEC31A	ES	26.1:26.2:27	25.1	28	4.2E-28	0.259	0.089	1.7E-05	8.3E-07	1.3E-05
KIRC	SEC31A_ES_26.1:27_25.1_28	SEC31A	ES	26.1:27	25.1	28	1.8E-36	0.328	0.155	7.7E-06	1.1E-06	5.3E-04
KIRC	SEC31A_ES_27_26.1_28	SEC31A	ES	27	26.1	28	4.5E-32	0.295	0.134	6.8E-06	3.9E-08	3.4E-05
KIRC	SEC31A_ES_27_26.2_28	SEC31A	ES	27	26.2	28	9.4E-33	0.295	0.131	1.8E-06	1.9E-07	3.9E-05
KIRC	SEMA5B_AD_23.2_23.1_24	SEMA5B	AD	23.2	23.1	24	2.4E-32	0.297	-0.166	8.2E-08	2.2E-08	9.5E-05

cancer type	id	Gene Symbol	splice_type	Exon	From.Exon	To.Exon	anova.p	adj.r2	r	p.50	p.25	p.10
KIRC	SEMA6C_AD_1.2_1.1_2	SEMA6C	AD	1.2	1.1	2	5.8E-24	0.229	0.157	1.7E-07	2.2E-08	1.2E-03
KIRC	SEPN1_ES_3_2_4	SEPN1	ES	3	2	4	1.4E-10	0.105	-0.160	1.2E-02	1.6E-04	3.2E-03
KIRC	SEPSECS_ES_2_1_3	SEPSECS	ES	2	1	3	1.7E-25	0.238	-0.195	1.3E-07	3.6E-06	5.9E-05
KIRC	SEPT10_ES_12.1:12.2_11.1_13	SEPT10	ES	12.1:12.2	11.1	13	2.2E-11	0.112	-0.059	2.1E-01	2.2E-01	9.0E-01
KIRC	SEPT6_AA_13.1_11.1_13.2	SEPT6	AA	13.1	11.1	13.2	8.2E-15	0.150	0.105	8.4E-03	5.2E-03	4.2E-04
KIRC	SEPT6_ES_12:13.1_11.1_13.2	SEPT6	ES	12:13.1	11.1	13.2	1.5E-15	0.151	0.239	4.2E-01	6.1E-02	1.6E-01
KIRC	SEPT8_AA_12.1_11_12.2	SEPT8	AA	12.1	11	12.2	6.0E-27	0.250	0.078	1.8E-03	9.9E-05	3.8E-06
KIRC	SERAC1_RI_16.2:16.3:16.4_16.1_16.5	SERAC1	RI	16.2:16.3:16.4	16.1	16.5	2.4E-10	0.124	-0.231	6.2E-05	8.8E-05	1.0E-02
KIRC	SETD6_AA_6.1_5_6.2	SETD6	AA	6.1	5	6.2	3.0E-20	0.197	0.351	5.4E-01	1.7E-02	4.3E-02
KIRC	SF3B1_AD_4.2:4.3_4.1_5	SF3B1	AD	4.2:4.3	4.1	5	3.6E-23	0.219	0.242	9.4E-05	6.9E-04	6.3E-03
KIRC	SFSWAP_AD_2.2_2.1_3	SFSWAP	AD	2.2	2.1	3	2.5E-20	0.195	-0.566	3.5E-02	5.0E-02	6.7E-02
KIRC	SFXN2_ES_2.1:2.2_1_4.1	SFXN2	ES	2.1:2.2	1	4.1	8.3E-09	0.113	-0.193	1.4E-02	2.3E-02	1.3E-04
KIRC	SGK2_ES_2_1_3.3	SGK2	ES	2	1	3.3	2.9E-25	0.249	-0.065	2.3E-03	7.0E-04	1.5E-03
KIRC	SGSM2_RI_5.2_5.1_5.3	SGSM2	RI	5.2	5.1	5.3	2.4E-29	0.277	0.569	9.7E-06	1.3E-06	5.7E-05
KIRC	SGSM3_RI_19.2_19.1_19.3	SGSM3	RI	19.2	19.1	19.3	3.0E-24	0.227	0.519	4.3E-06	6.7E-06	2.6E-03
KIRC	SH3BP1_ES_16_15_17.2	SH3BP1	ES	16	15	17.2	1.2E-13	0.134	-0.421	1.2E-01	5.6E-02	3.4E-01
KIRC	SH3BP5_ES_6:7_5.2_8	SH3BP5	ES	6:07	5.2	8	1.6E-18	0.178	-0.505	9.9E-07	2.2E-07	1.9E-04
KIRC	SH3YL1_ES_5.1:5.2_1_8	SH3YL1	ES	5.1:5.2	1	8	3.9E-09	0.111	-0.008	4.9E-01	2.3E-01	1.3E-01
KIRC	SHROOM1_AA_3.1:3.2_2_3.3	SHROOM1	AA	3.1:3.2	2	3.3	1.4E-14	0.160	0.391	7.8E-04	5.1E-05	3.7E-03
KIRC	SHROOM1_ES_3.1_2_3.3	SHROOM1	ES	3.1	2	3.3	1.2E-09	0.103	0.367	1.1E-03	1.9E-04	3.7E-03
KIRC	SIDT2_ES_14_13_15.1	SIDT2	ES	14	13	15.1	1.5E-13	0.134	-0.147	3.7E-04	3.1E-03	1.5E-01
KIRC	SIDT2_RI_25.2_25.1_25.3	SIDT2	RI	25.2	25.1	25.3	2.2E-20	0.194	-0.174	6.7E-03	5.8E-05	1.0E-03
KIRC	SIN3B_ES_10_9_11.2	SIN3B	ES	10	9	11.2	2.9E-22	0.212	0.484	7.3E-03	5.9E-04	2.1E-03
KIRC	SIRT2_AA_3.1_2_3.2	SIRT2	AA	3.1	2	3.2	2.2E-23	0.225	0.049	4.3E-02	2.6E-04	6.7E-06
KIRC	SIRT2_ES_2:3.1_1_3.2	SIRT2	ES	02:03.1	1	3.2	3.2E-27	0.252	0.113	4.3E-03	2.6E-03	7.8E-06
KIRC	SIRT2_ES_6_4_7	SIRT2	ES	6	4	7	1.5E-34	0.308	0.162	1.1E-07	2.1E-07	2.1E-05
KIRC	SIRT5_ES_5_4_6	SIRT5	ES	5	4	6	7.8E-19	0.181	-0.320	1.0E-06	3.7E-05	1.1E-03
KIRC	SIX5_RI_2.2_2.1_2.3	SIX5	RI	2.2	2.1	2.3	3.0E-16	0.160	0.095	2.5E-01	1.8E-02	1.1E-01
KIRC	SKA2_ES_1.2:2:4.1_1.1_5	SKA2	ES	1.2:2:4.1	1.1	5	1.4E-14	0.149	-0.002	2.9E-03	2.3E-03	1.4E-02
KIRC	SKA2_ES_3_2_4.1	SKA2	ES	3	2	4.1	4.9E-18	0.174	-0.220	2.6E-03	5.8E-03	2.2E-02
KIRC	SKA2_ES_3:4.1_2_5	SKA2	ES	03:04.1	2	5	5.5E-16	0.155	-0.264	3.3E-02	2.9E-02	1.6E-01
KIRC	SLC15A4_AA_3.2:3.3_2_3.4	SLC15A4	AA	3.2:3.3	2	3.4	3.0E-16	0.160	-0.144	7.4E-04	1.0E-01	9.0E-01
KIRC	SLC15A4_RI_3.3_3.2_3.4	SLC15A4	RI	3.3	3.2	3.4	3.0E-28	0.260	-0.006	1.4E-03	3.1E-04	6.7E-02
KIRC	SLC17A3_ES_5:6_4_7	SLC17A3	ES	5:06	4	7	1.2E-10	0.109	-0.098	1.5E-02	7.4E-01	7.3E-01
KIRC	SLC17A3_RI_8.2_8.1_8.3	SLC17A3	RI	8.2	8.1	8.3	5.9E-19	0.189	-0.099	4.4E-05	4.1E-06	2.2E-02
KIRC	SLC17A3_RI_8.4_8.3_8.5	SLC17A3	RI	8.4	8.3	8.5	2.2E-31	0.293	-0.031	2.6E-04	9.1E-07	9.0E-05
KIRC	SLC17A4_AD_10.4_10.3_11	SLC17A4	AD	10.4	10.3	11	2.3E-10	0.104	-0.511	1.1E-05	4.1E-04	3.9E-03
KIRC	SLC23A3_AD_5.2_5.1_6.1	SLC23A3	AD	5.2	5.1	6.1	8.9E-27	0.273	-0.515	6.9E-09	3.5E-08	5.3E-04
KIRC	SLC25A14_ES_8_7_9.1	SLC25A14	ES	8	7	9.1	1.2E-15	0.156	0.252	4.8E-04	7.9E-04	6.7E-04
KIRC	SLC25A14_RI_9.2_9.1_9.3	SLC25A14	RI	9.2	9.1	9.3	5.1E-29	0.266	0.490	1.5E-05	4.1E-06	2.4E-04

cancer type	id	Gene Symbol	splice_type	Exon	From.Exon	To.Exon	anova.p	adj.r2	r	p.50	p.25	p.10
KIRC	SLC25A16_ES_2.1:2.2_1_3	SLC25A16	ES	2.1:2.2	1	3	7.2E-31	0.285	0.266	1.7E-06	1.4E-06	1.2E-05
KIRC	SLC25A29_AA_3.2:3.3:3.4:3.5:3.6_2_3.7	SLC25A29	AA	3.2:3.3:3.4:3.5:3.[illegible]	2	3.7	4.2E-17	0.190	0.374	2.2E-05	3.3E-06	6.3E-04
KIRC	SLC25A29_ES_3.2:3.3:3.4:3.5_2_3.7	SLC25A29	ES	3.2:3.3:3.4:3.5	2	3.7	2.2E-14	0.179	0.480	5.3E-04	6.0E-05	2.0E-07
KIRC	SLC25A3_AD_2.2_2.1_4.1	SLC25A3	AD	2.2	2.1	4.1	6.2E-18	0.174	-0.309	1.7E-04	1.1E-04	4.0E-02
KIRC	SLC25A36_ES_6.1:6.2_4.1_7	SLC25A36	ES	6.1:6.2	4.1	7	1.9E-17	0.171	0.126	7.4E-06	1.4E-02	5.1E-02
KIRC	SLC25A37_AA_3.1_2.1_3.2	SLC25A37	AA	3.1	2.1	3.2	8.7E-19	0.182	0.554	9.9E-08	3.7E-12	1.2E-06
KIRC	SLC25A37_AD_2.2_2.1_3.2	SLC25A37	AD	2.2	2.1	3.2	1.3E-13	0.133	0.348	1.7E-06	1.1E-07	5.7E-06
KIRC	SLC26A1_ES_2_1_3	SLC26A1	ES	2	1	3	3.2E-15	0.164	-0.522	2.6E-05	6.1E-04	2.3E-02
KIRC	SLC27A1_ES_3_2.2_4.1	SLC27A1	ES	3	2.2	4.1	8.4E-10	0.121	0.098	4.2E-02	9.3E-02	1.3E-01
KIRC	SLC27A1_RI_4.2:4.3_4.1_4.4	SLC27A1	RI	4.2:4.3	4.1	4.4	1.1E-33	0.312	0.144	1.0E-05	3.2E-05	9.8E-04
KIRC	SLC2A11_AA_10.1_9.1_10.2	SLC2A11	AA	10.1	9.1	10.2	1.5E-13	0.148	0.170	2.2E-03	2.2E-04	7.4E-02
KIRC	SLC2A11_ES_10.1:10.2_9.1_11	SLC2A11	ES	10.1:10.2	9.1	11	5.0E-16	0.157	0.293	1.2E-01	2.4E-02	3.6E-02
KIRC	SLC2A11_ES_10.2_9.1_11	SLC2A11	ES	10.2	9.1	11	8.6E-15	0.147	0.034	1.5E-09	1.8E-06	7.1E-04
KIRC	SLC2A5_RI_6.2_6.1_6.3	SLC2A5	RI	6.2	6.1	6.3	1.1E-26	0.248	-0.359	8.3E-07	1.3E-08	4.1E-03
KIRC	SLC30A5_AA_4.1_3_4.2	SLC30A5	AA	4.1	3	4.2	9.7E-33	0.294	-0.633	3.6E-03	8.2E-04	1.7E-02
KIRC	SLC30A6_ES_5_4_6	SLC30A6	ES	5	4	6	1.7E-09	0.100	-0.342	4.6E-01	7.1E-01	4.3E-01
KIRC	SLC37A3_AA_10.1_9_10.2	SLC37A3	AA	10.1	9	10.2	4.8E-31	0.283	-0.121	1.4E-04	8.2E-06	7.1E-05
KIRC	SLC37A3_ES_4_3_5	SLC37A3	ES	4	3	5	8.0E-25	0.233	-0.308	5.1E-05	5.9E-05	2.7E-02
KIRC	SLC38A9_AD_16.5_16.4_17	SLC38A9	AD	16.5	16.4	17	1.1E-11	0.125	-0.023	2.6E-01	5.0E-02	5.0E-01
KIRC	SLC39A1_RI_4.2_4.1_4.3	SLC39A1	RI	4.2	4.1	4.3	4.6E-11	0.115	-0.145	8.5E-02	4.8E-02	5.9E-03
KIRC	SLC39A13_RI_6.2_6.1_6.3	SLC39A13	RI	6.2	6.1	6.3	1.7E-33	0.300	0.212	4.7E-05	4.8E-05	6.0E-03
KIRC	SLC39A13_RI_6.6:6.7:6.8_6.5_6.9	SLC39A13	RI	6.6:6.7:6.8	6.5	6.9	1.5E-11	0.131	0.076	3.7E-04	5.3E-04	7.6E-02
KIRC	SLC39A14_ME_5|6_4_7	SLC39A14	ME	5|6	4	7	1.2E-23	0.223	-0.023	2.0E-04	1.5E-05	8.4E-05
KIRC	SLC4A2_AD_5.2_5.1_6	SLC4A2	AD	5.2	5.1	6	7.0E-11	0.133	-0.096	2.0E-03	1.8E-04	2.8E-03
KIRC	SLC6A13_ES_5_4.2_6	SLC6A13	ES	5	4.2	6	2.1E-15	0.159	-0.196	6.1E-03	4.5E-03	1.5E-02
KIRC	SLC7A6_ES_3_2.2_4	SLC7A6	ES	3	2.2	4	6.9E-14	0.137	0.039	4.8E-02	8.5E-03	6.4E-02
KIRC	SLC7A9_RI_7.2_7.1_7.3	SLC7A9	RI	7.2	7.1	7.3	6.0E-21	0.199	-0.814	1.5E-06	4.7E-08	2.0E-03
KIRC	SLCO2B1_RI_13.2_13.1_13.3	SLCO2B1	RI	13.2	13.1	13.3	1.1E-30	0.279	-0.236	1.4E-04	1.9E-03	4.0E-02
KIRC	SLCO4A1_RI_8.2_8.1_8.3	SLCO4A1	RI	8.2	8.1	8.3	5.2E-20	0.201	0.138	5.8E-02	2.9E-03	7.2E-03
KIRC	SLCO4A1_RI_8.4_8.3_8.5	SLCO4A1	RI	8.4	8.3	8.5	2.6E-20	0.201	0.179	1.6E-01	3.0E-02	2.0E-01
KIRC	SLK_ES_13_12_14	SLK	ES	13	12	14	1.2E-17	0.171	0.158	8.0E-07	4.1E-06	1.5E-02
KIRC	SMARCD3_RI_11.2_11.1_11.3	SMARCD3	RI	11.2	11.1	11.3	1.3E-22	0.214	0.107	2.4E-04	3.7E-04	1.3E-01
KIRC	SMC5_ES_19_18_20	SMC5	ES	19	18	20	4.6E-09	0.100	0.106	6.1E-03	6.8E-03	2.0E-02
KIRC	SMC6_ES_9_8_10	SMC6	ES	9	8	10	2.6E-15	0.153	-0.248	8.0E-05	2.3E-04	5.1E-02
KIRC	SMPD4_ES_11:12_10_14	SMPD4	ES	11:12	10	14	3.3E-11	0.112	0.319	4.8E-04	2.7E-05	4.7E-02
KIRC	SMPD4_ES_12_11_14	SMPD4	ES	12	11	14	2.1E-32	0.294	0.403	3.8E-03	6.4E-04	7.1E-03
KIRC	SMPD4_ES_13_11_14	SMPD4	ES	13	11	14	3.9E-27	0.253	0.471	1.3E-02	7.1E-04	1.1E-01
KIRC	SMUG1_RI_4.4:4.5_4.3_4.6	SMUG1	RI	4.4:4.5	4.3	4.6	2.7E-15	0.149	-0.297	1.4E-03	1.8E-02	1.3E-02
KIRC	SMURF2_ES_4_3_5	SMURF2	ES	4	3	5	2.6E-12	0.121	-0.443	5.7E-02	1.2E-03	2.3E-02
KIRC	SNAPC3_RI_9.2_9.1_9.3	SNAPC3	RI	9.2	9.1	9.3	5.4E-11	0.108	-0.142	6.0E-02	2.9E-03	2.5E-02

cancer type	id	Gene Symbol	splice_type	Exon	From.Exon	To.Exon	anova.p	adj.r2	r	p.50	p.25	p.10
KIRC	SNAPC5_ES_2.1:2.2_1.1_3.1	SNAPC5	ES	2.1:2.2	1.1	3.1	2.0E-15	0.164	-0.157	1.4E-01	4.2E-02	4.5E-02
KIRC	SNRNP200_ES_45_44_46.1	SNRNP200	ES	45	44	46.1	5.8E-19	0.182	-0.129	1.6E-02	1.4E-02	5.8E-01
KIRC	SNRNP70_ES_8.1:8.2:8.3_7_9	SNRNP70	ES	8.1:8.2:8.3	7	9	8.6E-24	0.223	0.701	3.2E-02	4.8E-01	6.7E-01
KIRC	SNRPD2_ME_3|4_1.3_5.1	SNRPD2	ME	3|4	1.3	5.1	1.2E-19	0.189	-0.107	1.6E-02	2.2E-02	4.8E-01
KIRC	SNRPN_ES_8:9_7_10.3	SNRPN	ES	8:09	7	10.3	1.4E-10	0.137	0.132	5.5E-03	8.9E-04	2.3E-02
KIRC	SNUPN_ES_1.2:2_1.1_4	SNUPN	ES	1.2:2	1.1	4	5.0E-13	0.128	0.136	1.0E-05	1.8E-05	4.0E-04
KIRC	SNX11_AD_3.2_3.1_4	SNX11	AD	3.2	3.1	4	6.4E-10	0.100	0.000	3.2E-01	2.7E-01	4.3E-02
KIRC	SNX11_ES_5_4_6	SNX11	ES	5	4	6	1.3E-34	0.308	-0.077	1.8E-05	7.2E-05	4.3E-04
KIRC	SNX29_ES_5_4_6	SNX29	ES	5	4	6	7.2E-33	0.296	-0.513	6.9E-09	5.2E-05	5.6E-03
KIRC	SOCS2_RI_4.4_4.3_4.5	SOCS2	RI	4.4	4.3	4.5	1.4E-13	0.135	-0.058	1.9E-03	8.7E-06	5.4E-02
KIRC	SON_ES_11_10_12	SON	ES	11	10	12	4.1E-36	0.320	-0.115	3.4E-03	2.6E-05	2.5E-02
KIRC	SORBS1_ES_14:16_13_17	SORBS1	ES	14:16	13	17	7.5E-24	0.228	-0.534	1.7E-07	1.2E-06	9.8E-05
KIRC	SORBS2_ES_8:9.1:9.2_7_10	SORBS2	ES	8:9.1:9.2	7	10	1.5E-09	0.100	0.217	6.5E-03	1.9E-03	3.2E-02
KIRC	SP140L_RI_16.2_16.1_16.3	SP140L	RI	16.2	16.1	16.3	4.6E-25	0.235	0.273	1.2E-05	1.1E-04	8.2E-05
KIRC	SP140L_RI_16.4_16.3_16.5	SP140L	RI	16.4	16.3	16.5	7.9E-32	0.289	0.330	5.2E-05	1.9E-07	1.4E-05
KIRC	SPAG9_ES_30_29_31	SPAG9	ES	30	29	31	1.5E-15	0.152	-0.395	4.3E-04	4.3E-05	1.1E-05
KIRC	SPATA20_ES_4.1:4.2:5_3_6	SPATA20	ES	4.1:4.2:5	3	6	4.8E-10	0.121	-0.008	2.1E-03	1.2E-04	8.9E-02
KIRC	SPATA20_RI_11.2_11.1_11.3	SPATA20	RI	11.2	11.1	11.3	2.7E-36	0.322	0.203	2.3E-05	1.6E-07	1.2E-04
KIRC	SPATC1L_ES_2_1_3	SPATC1L	ES	2	1	3	4.7E-17	0.175	0.087	3.2E-02	6.8E-01	3.9E-01
KIRC	SPIN1_ES_2:3:4_1_5	SPIN1	ES	2:03:04	1	5	8.0E-17	0.163	-0.283	1.3E-04	9.0E-07	7.3E-03
KIRC	SPOP_ES_2_1_3	SPOP	ES	2	1	3	7.9E-12	0.123	-0.184	4.5E-04	1.2E-03	2.4E-02
KIRC	SPOP_ES_2_1_6	SPOP	ES	2	1	6	3.3E-10	0.102	-0.235	2.7E-02	1.3E-02	8.2E-02
KIRC	SPOP_ES_2:3_1_6	SPOP	ES	2:03	1	6	1.1E-15	0.154	-0.250	6.1E-06	1.1E-04	1.6E-05
KIRC	SPRTN_RI_4.2_4.1_4.3	SPRTN	RI	4.2	4.1	4.3	3.6E-17	0.167	-0.323	2.0E-05	1.2E-06	2.5E-05
KIRC	SRCAP_ES_23_22_24	SRCAP	ES	23	22	24	1.2E-10	0.111	0.081	1.3E-01	8.4E-02	2.7E-01
KIRC	SREBF1_RI_3.3_3.2_3.4	SREBF1	RI	3.3	3.2	3.4	2.3E-28	0.262	0.096	9.6E-05	1.3E-04	1.4E-03
KIRC	SREK1_ES_4_3.2_5	SREK1	ES	4	3.2	5	2.7E-23	0.221	0.604	1.9E-04	1.2E-03	4.9E-03
KIRC	SRP19_AA_5.1_4_5.2	SRP19	AA	5.1	4	5.2	2.7E-17	0.167	0.192	4.2E-03	1.2E-04	1.7E-02
KIRC	SRP9_ES_4_3_5	SRP9	ES	4	3	5	1.1E-14	0.157	-0.119	7.4E-01	5.2E-02	3.8E-01
KIRC	SRRM1_ES_4:5_3_6	SRRM1	ES	4:05	3	6	1.0E-32	0.297	0.376	1.8E-04	1.7E-05	2.7E-03
KIRC	SRSF1_RI_3.2_3.1_3.3	SRSF1	RI	3.2	3.1	3.3	4.3E-35	0.312	0.413	6.5E-07	2.2E-07	3.2E-03
KIRC	SRSF1_RI_3.6_3.5_3.7	SRSF1	RI	3.6	3.5	3.7	3.0E-34	0.305	-0.370	1.0E-05	1.8E-06	3.2E-06
KIRC	SRSF11_AA_6.1:6.2:6.3:6.4_4.2_6.5	SRSF11	AA	6.1:6.2:6.3:6.4	4.2	6.5	1.3E-13	0.134	0.422	3.1E-02	4.8E-02	4.7E-06
KIRC	SRSF11_ES_5:6.1:6.2:6.3_4.2_6.5	SRSF11	ES	5:6.1:6.2:6.3	4.2	6.5	7.3E-29	0.265	0.706	1.1E-04	1.3E-06	4.7E-05
KIRC	SRSF11_ES_5:6.1:6.2:6.3:6.4_4.2_6.5	SRSF11	ES	5:6.1:6.2:6.3:6.4	4.2	6.5	2.1E-26	0.245	0.710	5.3E-07	1.7E-05	3.4E-02
KIRC	SRSF2_AD_2.2:2.3_2.1_2.5	SRSF2	AD	2.2:2.3	2.1	2.5	1.2E-23	0.222	0.608	1.4E-02	1.4E-03	2.3E-02
KIRC	SRSF2_RI_2.2_2.1_2.3	SRSF2	RI	2.2	2.1	2.3	4.2E-13	0.129	0.577	4.2E-02	1.6E-01	7.7E-01
KIRC	SRSF2_RI_2.2:2.3:2.4_2.1_2.5	SRSF2	RI	2.2:2.3:2.4	2.1	2.5	2.6E-30	0.276	0.702	3.5E-03	8.1E-04	2.4E-02
KIRC	SRSF2_RI_2.4_2.3_2.5	SRSF2	RI	2.4	2.3	2.5	9.1E-17	0.163	0.649	1.4E-02	8.9E-04	1.0E-01
KIRC	SRSF3_ES_4.1_3_5	SRSF3	ES	4.1	3	5	2.6E-15	0.149	0.063	4.1E-03	4.9E-01	2.6E-01

cancer type	id	Gene Symbol	splice_type	Exon	From.Exon	To.Exon	anova.p	adj.r2	r	p.50	p.25	p.10
KIRC	SRSF4_ES_2:3:4_1_5	SRSF4	ES	2:03:04	1	5	4.8E-30	0.274	0.487	5.3E-03	3.9E-04	8.9E-03
KIRC	SRSF4_ES_6_5_7	SRSF4	ES	6	5	7	1.6E-31	0.285	0.482	9.2E-03	4.9E-03	6.2E-02
KIRC	SRSF5_AA_8.1_7.2_8.2	SRSF5	AA	8.1	7.2	8.2	1.5E-22	0.213	0.717	2.1E-02	1.1E-02	1.1E-01
KIRC	SRSF6_ES_3_2_4	SRSF6	ES	3	2	4	9.9E-19	0.180	0.469	7.4E-04	3.9E-02	2.0E-02
KIRC	SRSF7_AA_4.3:4.4:4.5_4.1_4.6	SRSF7	AA	4.3:4.4:4.5	4.1	4.6	2.8E-32	0.291	0.392	6.0E-04	9.6E-04	5.8E-02
KIRC	SRSF7_AD_4.2:4.3:4.4_4.1_4.6	SRSF7	AD	4.2:4.3:4.4	4.1	4.6	6.5E-30	0.273	0.384	6.2E-03	1.1E-02	1.4E-03
KIRC	SRSF7_RI_4.2:4.3:4.4:4.5_4.1_4.6	SRSF7	RI	4.2:4.3:4.4:4.5	4.1	4.6	6.3E-27	0.249	0.307	1.2E-02	3.2E-03	8.2E-03
KIRC	SS18_ES_6_3_9	SS18	ES	6	3	9	6.0E-19	0.184	0.027	2.1E-03	1.4E-04	4.6E-02
KIRC	SSB_ES_6_5_7	SSB	ES	6	5	7	1.8E-33	0.300	-0.345	1.3E-04	3.7E-03	9.1E-03
KIRC	SSH3_RI_4.2_4.1_4.3	SSH3	RI	4.2	4.1	4.3	1.9E-25	0.239	0.244	2.0E-03	7.2E-05	2.1E-03
KIRC	ST3GAL5_AA_9.1_6_9.2	ST3GAL5	AA	9.1	6	9.2	1.1E-29	0.279	0.040	1.7E-02	4.9E-04	5.9E-02
KIRC	ST7_ES_10_9_11.1	ST7	ES	10	9	11.1	1.5E-14	0.143	-0.513	2.3E-03	1.5E-02	2.0E-04
KIRC	ST7_ES_4_3_5	ST7	ES	4	3	5	7.0E-13	0.138	-0.107	3.6E-05	3.6E-06	3.6E-05
KIRC	STAG3_ES_5:6:7:8:9_4_10	STAG3	ES	5:6:7:8:9	4	10	1.3E-16	0.162	-0.194	4.5E-01	5.6E-01	5.3E-01
KIRC	STAMBP_RI_2.2_2.1_2.3	STAMBP	RI	2.2	2.1	2.3	1.1E-09	0.100	-0.283	2.6E-01	1.5E-01	2.3E-01
KIRC	STAP2_AA_14.1:14.2_13_14.3	STAP2	AA	14.1:14.2	13	14.3	4.4E-15	0.147	-0.343	4.4E-01	5.0E-01	3.7E-01
KIRC	STAP2_AA_14.2_13_14.3	STAP2	AA	14.2	13	14.3	8.4E-11	0.106	-0.318	1.6E-03	4.4E-04	1.1E-03
KIRC	STAU1_ES_2_1_3	STAU1	ES	2	1	3	8.6E-32	0.287	-0.612	2.4E-06	1.8E-05	1.2E-01
KIRC	STEAP3_ES_3_1.1_5.3	STEAP3	ES	3	1.1	5.3	8.8E-30	0.282	-0.762	2.4E-03	3.0E-04	3.4E-02
KIRC	STEAP3_ME_2|3_1.1_5.3	STEAP3	ME	2|3	1.1	5.3	1.4E-20	0.198	0.774	2.3E-06	1.4E-04	6.0E-03
KIRC	STK11IP_AD_3.2_3.1_4	STK11IP	AD	3.2	3.1	4	3.7E-09	0.104	0.192	9.7E-02	2.1E-01	1.8E-01
KIRC	STRA13_ES_3.1:3.2_2_4.1	STRA13	ES	3.1:3.2	2	4.1	3.3E-13	0.130	-0.099	1.2E-04	2.1E-05	7.9E-04
KIRC	STRA6_AD_18.2_18.1_19	STRA6	AD	18.2	18.1	19	1.7E-15	0.153	-0.683	1.4E-02	4.3E-03	1.4E-03
KIRC	STRADA_ES_5_3_6	STRADA	ES	5	3	6	2.4E-16	0.193	0.275	7.2E-02	2.7E-02	4.5E-02
KIRC	STRADA_RI_12.3:12.4_12.2_12.5	STRADA	RI	12.3:12.4	12.2	12.5	2.6E-11	0.112	0.358	2.4E-04	1.6E-05	3.0E-03
KIRC	STRADA_RI_12.4_12.3_12.5	STRADA	RI	12.4	12.3	12.5	8.3E-17	0.163	0.291	2.0E-01	1.2E-01	6.4E-02
KIRC	STRADA_RI_12.6_12.5_12.7	STRADA	RI	12.6	12.5	12.7	2.2E-17	0.168	0.361	7.6E-03	7.1E-03	7.4E-02
KIRC	STX16_ES_7_6_8	STX16	ES	7	6	8	4.5E-13	0.129	-0.437	2.8E-05	5.5E-04	5.2E-03
KIRC	STX17_ES_6_5_7	STX17	ES	6	5	7	1.6E-24	0.238	-0.297	1.8E-04	2.9E-04	2.0E-02
KIRC	STX2_ES_10_9_11	STX2	ES	10	9	11	1.9E-11	0.113	0.013	3.2E-02	3.5E-01	5.1E-02
KIRC	STX3_ME_11|12_10_14	STX3	ME	11|12	10	14	2.0E-24	0.242	-0.024	1.4E-02	6.7E-03	1.3E-03
KIRC	SUGP1_AA_5.1_4_5.2	SUGP1	AA	5.1	4	5.2	2.5E-31	0.285	0.456	4.5E-06	3.6E-06	5.5E-07
KIRC	SUGP2_AD_12.4:12.5_12.3_14	SUGP2	AD	12.4:12.5	12.3	14	3.6E-11	0.111	0.433	1.6E-05	6.2E-03	2.3E-01
KIRC	SUGP2_ES_13_12.3_14	SUGP2	ES	13	12.3	14	5.2E-11	0.108	0.381	3.8E-03	6.7E-04	1.2E-02
KIRC	SUGP2_RI_12.2_12.1_12.3	SUGP2	RI	12.2	12.1	12.3	1.2E-20	0.197	0.628	2.2E-04	5.6E-04	6.4E-02
KIRC	SUGP2_RI_12.4_12.3_12.5	SUGP2	RI	12.4	12.3	12.5	3.3E-13	0.129	0.708	1.8E-02	5.1E-03	1.5E-02
KIRC	SULT1A2_AA_5.1_4_5.2	SULT1A2	AA	5.1	4	5.2	1.5E-16	0.166	0.149	2.8E-03	2.7E-03	3.5E-01
KIRC	SULT1C2_AA_5.1_3_5.2	SULT1C2	AA	5.1	3	5.2	6.8E-30	0.292	0.212	3.7E-04	1.6E-03	2.3E-02
KIRC	SULT1C4_ES_3:4_2_5	SULT1C4	ES	3:04	2	5	6.5E-12	0.123	-0.323	1.9E-03	9.9E-03	1.4E-01
KIRC	SUMO1_ES_3:4_1_5.1	SUMO1	ES	3:04	1	5.1	4.1E-14	0.144	-0.186	2.3E-03	1.7E-02	6.1E-01

cancer type	id	Gene Symbol	splice_type	Exon	From.Exon	To.Exon	anova.p	adj.r2	r	p.50	p.25	p.10
KIRC	SUOX_ES_3_2_5.2	SUOX	ES	3	2	5.2	8.1E-18	0.172	-0.197	3.0E-04	1.6E-05	1.7E-03
KIRC	SUOX_ES_4_1_5.2	SUOX	ES	4	1	5.2	3.7E-18	0.176	-0.122	1.3E-05	2.2E-04	1.5E-01
KIRC	SUPT20H_AA_21.1:21.2_20_21.3	SUPT20H	AA	21.1:21.2	20	21.3	1.4E-15	0.152	0.327	3.6E-01	4.3E-01	3.4E-01
KIRC	SUPT4H1_AD_2.4_2.3_3.2	SUPT4H1	AD	2.4	2.3	3.2	4.4E-15	0.147	-0.150	2.1E-02	1.4E-02	1.3E-02
KIRC	SUPT6H_ES_2_1_3.1	SUPT6H	ES	2	1	3.1	1.3E-11	0.119	-0.220	3.0E-04	7.1E-05	1.8E-02
KIRC	SUPT7L_RI_2.2_2.1_2.3	SUPT7L	RI	2.2	2.1	2.3	2.3E-17	0.168	0.536	4.8E-05	1.9E-06	1.7E-03
KIRC	SUPT7L_RI_2.2:2.3_2.1_2.4	SUPT7L	RI	2.2:2.3	2.1	2.4	4.2E-35	0.312	0.650	7.7E-06	3.1E-07	9.4E-05
KIRC	SVIL_ES_10:11:12_9_13	SVIL	ES	10:11:12	9	13	4.2E-10	0.101	-0.038	3.9E-02	6.0E-03	7.8E-04
KIRC	SYDE1_RI_2.4_2.3_2.5	SYDE1	RI	2.4	2.3	2.5	1.1E-24	0.236	0.010	7.8E-04	1.3E-05	2.3E-02
KIRC	SYNGR2_AA_4.1:4.2_3.2_4.3	SYNGR2	AA	4.1:4.2	3.2	4.3	6.0E-20	0.191	-0.087	2.1E-05	6.0E-06	4.0E-05
KIRC	SYNJ2_AD_19.3_19.2_20	SYNJ2	AD	19.3	19.2	20	7.0E-25	0.232	-0.565	1.4E-04	2.2E-03	2.4E-02
KIRC	SYTL2_AA_11.1_10.3_11.2	SYTL2	AA	11.1	10.3	11.2	6.6E-17	0.165	0.100	4.7E-06	4.4E-06	3.6E-05
KIRC	SYTL2_ES_11.1:11.2_10.3_12.2	SYTL2	ES	11.1:11.2	10.3	12.2	7.7E-14	0.140	-0.194	3.4E-04	7.9E-07	6.7E-06
KIRC	SYTL2_ES_11.1:11.2:12.2_10.3_13	SYTL2	ES	11.1:11.2:12.2	10.3	13	1.2E-14	0.164	-0.123	1.1E-03	4.6E-03	3.3E-02
KIRC	TAB3_AD_9.2:9.3:9.4:9.5_9.1_11	TAB3	AD	9.2:9.3:9.4:9.5	9.1	11	1.3E-14	0.166	-0.232	1.1E-03	7.5E-05	5.9E-04
KIRC	TAB3_RI_9.2:9.3:9.4_9.1_9.5	TAB3	RI	9.2:9.3:9.4	9.1	9.5	4.4E-11	0.113	-0.215	6.2E-05	2.3E-05	2.5E-03
KIRC	TACC2_ES_12_11_13	TACC2	ES	12	11	13	3.3E-29	0.267	-0.562	2.3E-08	5.0E-06	4.3E-02
KIRC	TADA2A_AD_6.2_6.1_7	TADA2A	AD	6.2	6.1	7	1.3E-10	0.113	0.087	1.6E-02	2.7E-03	3.1E-03
KIRC	TAF1C_RI_8.2_8.1_8.3	TAF1C	RI	8.2	8.1	8.3	4.9E-19	0.185	0.659	9.8E-07	3.9E-06	1.7E-03
KIRC	TAF1D_AA_12.3:12.4_12.1_12.5	TAF1D	AA	12.3:12.4	12.1	12.5	6.0E-11	0.116	-0.099	8.6E-06	3.9E-07	4.6E-05
KIRC	TAF1D_AA_8.1_7_8.2	TAF1D	AA	8.1	7	8.2	5.0E-16	0.155	-0.334	1.8E-10	7.5E-12	7.1E-05
KIRC	TAF1D_RI_12.2_12.1_12.3	TAF1D	RI	12.2	12.1	12.3	2.0E-18	0.177	-0.139	1.2E-07	8.5E-08	5.7E-07
KIRC	TAF1D_RI_12.2:12.3:12.4_12.1_12.5	TAF1D	RI	12.2:12.3:12.4	12.1	12.5	4.0E-17	0.182	-0.121	2.4E-08	4.1E-08	2.6E-07
KIRC	TAF1D_RI_12.4_12.3_12.5	TAF1D	RI	12.4	12.3	12.5	1.3E-23	0.222	-0.092	4.5E-10	5.5E-10	2.4E-07
KIRC	TAF8_ES_9_8.2_10	TAF8	ES	9	8.2	10	1.5E-26	0.247	-0.048	4.3E-05	7.6E-07	1.0E-06
KIRC	TARBP2_AD_4.2_4.1_5.1	TARBP2	AD	4.2	4.1	5.1	6.9E-20	0.193	0.178	1.1E-03	3.6E-07	1.9E-06
KIRC	TARDBP_ES_3_2_4	TARDBP	ES	3	2	4	2.7E-32	0.291	0.088	1.1E-05	6.0E-05	1.0E-03
KIRC	TAZ_AD_3.2:3.3_3.1_4	TAZ	AD	3.2:3.3	3.1	4	1.6E-19	0.188	0.503	4.6E-02	9.4E-03	4.8E-02
KIRC	TBC1D10A_ES_11_10_12	TBC1D10A	ES	11	10	12	1.0E-20	0.198	-0.030	9.5E-04	5.9E-05	7.8E-04
KIRC	TBC1D10C_AD_7.2_7.1_8	TBC1D10C	AD	7.2	7.1	8	7.2E-12	0.132	0.164	2.7E-05	1.6E-03	7.2E-03
KIRC	TBC1D15_ES_10_9_11	TBC1D15	ES	10	9	11	6.7E-20	0.192	0.272	9.0E-03	4.0E-03	5.1E-04
KIRC	TBC1D17_ES_4_3_5	TBC1D17	ES	4	3	5	1.8E-35	0.315	0.411	1.4E-05	2.7E-05	1.7E-02
KIRC	TBC1D22A_ES_5_1_6	TBC1D22A	ES	5	1	6	1.6E-20	0.196	-0.100	1.2E-04	1.0E-06	7.0E-06
KIRC	TBC1D23_ES_15_14_16	TBC1D23	ES	15	14	16	2.8E-13	0.131	-0.241	2.2E-03	2.6E-04	5.4E-03
KIRC	TBC1D32_ES_24_23_25	TBC1D32	ES	24	23	25	1.5E-19	0.228	-0.324	4.5E-01	3.4E-01	3.8E-02
KIRC	TBCE_ES_8_7_9	TBCE	ES	8	7	9	6.1E-18	0.174	-0.015	4.1E-03	5.5E-03	1.1E-02
KIRC	TBCEL_ES_2:3_1.2_4	TBCEL	ES	2:03	1.2	4	4.2E-17	0.195	-0.328	1.5E-02	3.0E-05	3.9E-03
KIRC	TBL2_AA_4.1:4.2_1_4.3	TBL2	AA	4.1:4.2	1	4.3	6.3E-22	0.208	-0.242	1.5E-03	2.4E-04	6.7E-05
KIRC	TBRG1_ES_5_3_6	TBRG1	ES	5	3	6	1.1E-13	0.135	0.358	1.3E-01	8.8E-02	3.0E-03
KIRC	TBXAS1_ES_8_7.1_9	TBXAS1	ES	8	7.1	9	4.0E-23	0.218	-0.402	4.5E-05	7.1E-06	2.4E-03

cancer type	id	Gene Symbol	splice_type	Exon	From.Exon	To.Exon	anova.p	adj.r2	r	p.50	p.25	p.10
KIRC	TCAIM_ES_8_7.2_9	TCAIM	ES	8	7.2	9	9.2E-37	0.324	-0.479	1.4E-07	2.4E-06	4.1E-03
KIRC	TCERG1_ES_22_21_23	TCERG1	ES	22	21	23	2.2E-17	0.169	0.509	5.5E-01	7.6E-01	7.1E-01
KIRC	TCF20_ES_4.1:4.2_3_5	TCF20	ES	4.1:4.2	3	5	1.8E-12	0.123	0.092	2.7E-06	2.7E-03	7.3E-03
KIRC	TCF20_ES_4.2_3_5	TCF20	ES	4.2	3	5	8.5E-13	0.126	0.067	1.3E-03	3.4E-07	1.5E-04
KIRC	TCIRG1_RI_5.2_5.1_5.3	TCIRG1	RI	5.2	5.1	5.3	7.3E-20	0.193	0.588	9.8E-06	2.9E-07	3.3E-05
KIRC	TCP11L1_AD_1.2:1.3:1.4_1.1_2	TCP11L1	AD	1.2:1.3:1.4	1.1	2	2.8E-13	0.133	0.018	9.1E-03	4.3E-03	2.7E-01
KIRC	TCP11L1_AD_1.4_1.3_2	TCP11L1	AD	1.4	1.3	2	8.6E-19	0.187	-0.188	9.9E-04	2.6E-05	1.7E-03
KIRC	TCTEX1D2_ES_4_3_5	TCTEX1D2	ES	4	3	5	1.1E-18	0.180	-0.298	1.3E-03	1.4E-03	7.8E-03
KIRC	TCTN1_ES_2:4_1.1_5	TCTN1	ES	2:04	1.1	5	9.3E-12	0.136	-0.164	2.2E-01	2.5E-02	3.6E-02
KIRC	TDP2_AD_2.2_2.1_3	TDP2	AD	2.2	2.1	3	1.4E-17	0.169	-0.231	3.0E-03	4.4E-03	5.6E-03
KIRC	TEFM_RI_3.3_3.2_3.4	TEFM	RI	3.3	3.2	3.4	2.1E-34	0.307	0.180	8.0E-05	1.9E-06	1.7E-05
KIRC	TEP1_ES_49_48_50	TEP1	ES	49	48	50	2.6E-14	0.140	-0.249	4.8E-01	4.8E-01	6.7E-01
KIRC	TET2_RI_4.2_4.1_4.3	TET2	RI	4.2	4.1	4.3	4.8E-24	0.226	-0.256	7.9E-05	7.9E-05	1.1E-04
KIRC	TEX30_RI_3.3_3.2_3.4	TEX30	RI	3.3	3.2	3.4	6.0E-26	0.245	-0.089	2.0E-03	3.9E-02	3.4E-02
KIRC	TFDP1_ES_13.1:13.2:13.3_12_14	TFDP1	ES	13.1:13.2:13.3	12	14	2.8E-18	0.183	0.025	3.4E-02	1.1E-01	8.4E-01
KIRC	TFEC_RI_11.2_11.1_11.3	TFEC	RI	11.2	11.1	11.3	7.2E-14	0.136	0.211	1.3E-01	1.2E-01	5.9E-01
KIRC	TFIP11_ES_3_2.2_4	TFIP11	ES	3	2.2	4	1.4E-11	0.118	0.053	2.3E-01	8.1E-02	1.9E-01
KIRC	TGFBR3_ES_5_4.2_6	TGFBR3	ES	5	4.2	6	3.5E-28	0.259	-0.501	1.1E-08	1.2E-06	1.7E-02
KIRC	THAP9_ES_2:3_1_4.1	THAP9	ES	2:03	1	4.1	6.8E-13	0.126	-0.174	5.2E-07	4.2E-07	5.7E-03
KIRC	THBS3_AA_10.1_9_10.2	THBS3	AA	10.1	9	10.2	3.4E-26	0.246	0.343	3.1E-01	8.7E-02	5.2E-03
KIRC	THEM4_ES_3_2.1_4	THEM4	ES	3	2.1	4	3.9E-14	0.138	0.009	2.9E-01	1.6E-03	5.5E-03
KIRC	THUMPD1_ES_3_2_4	THUMPD1	ES	3	2	4	3.5E-30	0.283	-0.080	8.8E-04	1.7E-05	9.4E-04
KIRC	THUMPD2_ES_4_3_5	THUMPD2	ES	4	3	5	2.5E-15	0.154	0.141	8.7E-03	1.8E-02	3.3E-01
KIRC	THUMPD2_ES_8_7_9	THUMPD2	ES	8	7	9	5.2E-15	0.147	0.272	9.0E-02	4.5E-02	1.7E-01
KIRC	TIA1_ES_5:6_4_7	TIA1	ES	5:06	4	7	6.7E-11	0.109	0.490	6.1E-02	1.0E-02	5.3E-02
KIRC	TIA1_ES_6_5_7	TIA1	ES	6	5	7	5.4E-18	0.175	0.555	5.1E-02	3.7E-02	6.7E-02
KIRC	TIA1_ES_8_7_9.1	TIA1	ES	8	7	9.1	3.1E-19	0.186	0.448	8.8E-01	5.6E-01	1.4E-01
KIRC	TIAL1_ES_6_5.1_7	TIAL1	ES	6	5.1	7	5.8E-29	0.266	0.299	1.1E-06	2.0E-05	1.5E-04
KIRC	TIGD6_AD_1.2_1.1_2	TIGD6	AD	1.2	1.1	2	7.9E-12	0.116	-0.007	8.6E-07	8.8E-10	2.6E-04
KIRC	TIMM17B_AA_4.1_3_4.2	TIMM17B	AA	4.1	3	4.2	1.4E-24	0.230	-0.145	1.5E-04	4.0E-03	3.0E-01
KIRC	TIMM17B_AA_4.1:4.2_3_4.3	TIMM17B	AA	4.1:4.2	3	4.3	7.6E-11	0.107	-0.124	4.2E-05	2.2E-05	5.0E-04
KIRC	TLE2_RI_10.2_10.1_10.3	TLE2	RI	10.2	10.1	10.3	8.0E-18	0.224	-0.130	3.5E-04	2.3E-05	3.3E-02
KIRC	TLE2_RI_10.2:10.3_10.1_10.4	TLE2	RI	10.2:10.3	10.1	10.4	2.6E-18	0.230	-0.144	4.1E-04	3.5E-05	3.4E-03
KIRC	TM2D3_RI_1.2_1.1_1.3	TM2D3	RI	1.2	1.1	1.3	1.3E-20	0.200	-0.014	3.7E-03	2.0E-04	3.2E-03
KIRC	TMC6_AD_14.2:14.3_14.1_15	TMC6	AD	14.2:14.3	14.1	15	7.6E-13	0.138	0.106	1.5E-02	1.2E-01	6.4E-01
KIRC	TMCO6_ES_3_2.1_4.1	TMCO6	ES	3	2.1	4.1	1.9E-14	0.150	0.249	2.5E-05	2.6E-05	4.9E-03
KIRC	TMCO6_RI_4.2_4.1_4.3	TMCO6	RI	4.2	4.1	4.3	2.1E-21	0.215	0.415	2.0E-03	1.3E-02	1.9E-01
KIRC	TMCO6_RI_4.4_4.3_4.5	TMCO6	RI	4.4	4.3	4.5	5.0E-19	0.185	0.442	3.4E-05	7.2E-03	4.2E-03
KIRC	TMEM104_ME_3|4_2.2_5	TMEM104	ME	3|4	2.2	5	6.7E-27	0.250	-0.260	1.0E-03	2.7E-02	1.0E-01
KIRC	TMEM106B_ES_2_1_3	TMEM106B	ES	2	1	3	1.1E-11	0.116	-0.183	1.1E-01	8.7E-03	1.7E-01

cancer type	id	Gene Symbol	splice_type	Exon	From.Exon	To.Exon	anova.p	adj.r2	r	p.50	p.25	p.10
KIRC	TMEM107_ES_2:3.2:3.3:3.4_1_3.7	TMEM107	ES	2:3.2:3.3:3.4	1	3.7	9.2E-13	0.130	-0.083	9.2E-03	9.3E-02	1.2E-01
KIRC	TMEM107_ES_2:3.2:3.3:3.4:3.5_1_3.7	TMEM107	ES	2:3.2:3.3:3.4:3.5	1	3.7	1.1E-11	0.115	-0.147	9.1E-03	4.4E-02	1.6E-02
KIRC	TMEM107_ES_2:3.2:3.3:3.4:3.5:3.6_1_3.	TMEM107	ES	:3.2:3.3:3.4:3.5:3	1	3.7	5.0E-17	0.169	-0.154	8.4E-04	4.6E-03	1.2E-02
KIRC	TMEM107_RI_3.3_3.2_3.4	TMEM107	RI	3.3	3.2	3.4	7.1E-22	0.207	-0.243	1.2E-04	2.0E-05	4.8E-03
KIRC	TMEM107_RI_3.6_3.5_3.7	TMEM107	RI	3.6	3.5	3.7	2.4E-18	0.177	-0.075	1.6E-04	6.3E-04	5.5E-03
KIRC	TMEM136_RI_3.2_3.1_3.3	TMEM136	RI	3.2	3.1	3.3	4.3E-17	0.165	0.148	1.1E-03	3.1E-03	9.5E-03
KIRC	TMEM138_RI_3.3_3.2_3.4	TMEM138	RI	3.3	3.2	3.4	3.5E-25	0.235	0.225	1.2E-03	4.2E-02	1.8E-01
KIRC	TMEM143_ES_3.1:3.2_2_4	TMEM143	ES	3.1:3.2	2	4	2.2E-21	0.216	0.131	7.9E-03	6.2E-02	3.4E-02
KIRC	TMEM144_RI_1.2_1.1_1.3	TMEM144	RI	1.2	1.1	1.3	1.8E-18	0.189	-0.366	6.5E-06	1.4E-06	1.8E-04
KIRC	TMEM150A_AD_4.2_4.1_5	TMEM150A	AD	4.2	4.1	5	1.3E-31	0.286	0.234	4.1E-03	3.9E-04	7.3E-02
KIRC	TMEM150A_ES_6:7_5_8	TMEM150A	ES	6:07	5	8	1.3E-21	0.208	0.156	3.6E-05	4.2E-04	6.7E-03
KIRC	TMEM161B_AD_11.5:11.6_11.4_12.1	TMEM161B	AD	11.5:11.6	11.4	12.1	2.3E-15	0.151	-0.138	1.1E-06	1.2E-06	2.4E-03
KIRC	TMEM161B_RI_11.2_11.1_11.3	TMEM161B	RI	11.2	11.1	11.3	2.7E-16	0.158	-0.263	2.6E-04	4.4E-06	7.1E-04
KIRC	TMEM161B_RI_11.5_11.4_11.6	TMEM161B	RI	11.5	11.4	11.6	2.3E-26	0.246	-0.175	5.1E-07	4.3E-06	5.5E-03
KIRC	TMEM168_ES_2_1_3	TMEM168	ES	2	1	3	5.3E-25	0.257	-0.090	5.5E-04	4.4E-04	7.8E-04
KIRC	TMEM175_ES_2:3:4.1:4.2_1_5.1	TMEM175	ES	2:3:4.1:4.2	1	5.1	1.5E-11	0.115	0.417	3.9E-03	6.7E-04	5.6E-04
KIRC	TMEM175_ES_4.1:4.2_3_5.1	TMEM175	ES	4.1:4.2	3	5.1	3.2E-23	0.219	0.535	1.4E-03	1.6E-04	1.2E-02
KIRC	TMEM180_ES_4_3_5	TMEM180	ES	4	3	5	1.5E-14	0.143	0.024	1.1E-04	3.4E-03	1.1E-01
KIRC	TMEM205_AD_2.2:2.3_2.1_2.6	TMEM205	AD	2.2:2.3	2.1	2.6	1.7E-11	0.114	-0.076	1.4E-06	3.2E-05	1.6E-01
KIRC	TMEM205_RI_2.2:2.3:2.4_2.1_2.5	TMEM205	RI	2.2:2.3:2.4	2.1	2.5	7.8E-16	0.154	-0.191	4.1E-02	2.2E-03	1.4E-03
KIRC	TMEM205_RI_2.2:2.3:2.4:2.5_2.1_2.6	TMEM205	RI	2.2:2.3:2.4:2.5	2.1	2.6	1.8E-20	0.195	-0.205	4.6E-05	2.0E-05	2.6E-03
KIRC	TMEM205_RI_2.3:2.4_2.2_2.5	TMEM205	RI	2.3:2.4	2.2	2.5	1.5E-26	0.246	-0.129	2.9E-03	1.8E-03	2.9E-02
KIRC	TMEM205_RI_2.3:2.4:2.5_2.2_2.6	TMEM205	RI	2.3:2.4:2.5	2.2	2.6	1.6E-28	0.262	-0.141	2.1E-05	1.9E-03	4.6E-01
KIRC	TMEM205_RI_2.4_2.3_2.5	TMEM205	RI	2.4	2.3	2.5	4.2E-17	0.166	-0.132	1.4E-01	7.9E-02	1.6E-01
KIRC	TMEM205_RI_2.4:2.5_2.3_2.6	TMEM205	RI	2.4:2.5	2.3	2.6	1.1E-27	0.255	-0.123	7.7E-01	7.8E-01	9.0E-02
KIRC	TMEM234_ES_4_3_5.1	TMEM234	ES	4	3	5.1	2.1E-17	0.168	0.279	1.8E-03	4.0E-03	4.4E-05
KIRC	TMEM234_RI_5.2:5.3_5.1_5.4	TMEM234	RI	5.2:5.3	5.1	5.4	3.6E-24	0.227	0.354	6.3E-05	8.1E-07	2.5E-07
KIRC	TMEM234_RI_5.7_5.6_5.8	TMEM234	RI	5.7	5.6	5.8	1.8E-10	0.103	-0.108	5.5E-02	9.1E-02	2.1E-01
KIRC	TMEM26_ES_5:6.1_4_6.2	TMEM26	ES	05:06.1	4	6.2	1.3E-11	0.137	0.002	7.5E-03	6.6E-05	1.7E-02
KIRC	TMEM33_RI_8.2_8.1_8.3	TMEM33	RI	8.2	8.1	8.3	6.9E-29	0.264	0.455	1.5E-04	1.1E-03	8.0E-02
KIRC	TMEM44_ES_7_6.1_8	TMEM44	ES	7	6.1	8	2.3E-20	0.201	0.090	1.7E-03	3.8E-03	6.9E-03
KIRC	TMEM45A_ES_2_1_3	TMEM45A	ES	2	1	3	2.1E-10	0.119	0.555	7.4E-04	1.5E-04	3.1E-04
KIRC	TMEM55B_RI_2.2_2.1_2.3	TMEM55B	RI	2.2	2.1	2.3	8.7E-31	0.280	0.075	5.8E-04	9.8E-03	1.0E-02
KIRC	TMEM63A_ES_10:11.1_9.2_11.2	TMEM63A	ES	10:11.1	9.2	11.2	1.1E-32	0.296	0.143	1.3E-08	8.9E-05	7.6E-02
KIRC	TMEM67_ES_4_3.2_6	TMEM67	ES	4	3.2	6	8.8E-35	0.313	-0.204	1.6E-02	1.8E-02	1.5E-03
KIRC	TMEM9_RI_3.2_3.1_3.3	TMEM9	RI	3.2	3.1	3.3	1.9E-31	0.295	-0.196	3.6E-03	3.9E-04	1.5E-04
KIRC	TMOD2_ES_3_2_4	TMOD2	ES	3	2	4	1.2E-19	0.230	-0.477	5.7E-04	1.4E-07	7.0E-04
KIRC	TMUB2_ES_2.3:2.4:2.5:3_2.2_4.3	TMUB2	ES	2.3:2.4:2.5:3	2.2	4.3	2.7E-10	0.104	-0.079	1.2E-01	5.7E-02	8.2E-01
KIRC	TMX2_AA_3.1_2_3.2	TMX2	AA	3.1	2	3.2	1.3E-22	0.224	-0.288	6.8E-04	3.6E-03	1.5E-02
KIRC	TMX2_ES_3.1:3.2:3.3_2_4	TMX2	ES	3.1:3.2:3.3	2	4	7.6E-25	0.234	-0.330	6.2E-03	2.3E-04	1.1E-01

cancer type	id	Gene Symbol	splice_type	Exon	From.Exon	To.Exon	anova.p	adj.r2	r	p.50	p.25	p.10
KIRC	TNFRSF10B_RI_5.2_5.1_5.3	TNFRSF10B	RI	5.2	5.1	5.3	8.2E-24	0.224	0.279	1.8E-03	4.2E-03	2.6E-03
KIRC	TNFRSF11B_ES_3_2_4	TNFRSF11B	ES	3	2	4	4.7E-22	0.209	-0.703	5.3E-05	5.9E-03	1.3E-01
KIRC	TNFRSF14_AA_7.1_6_7.2	TNFRSF14	AA	7.1	6	7.2	8.4E-30	0.272	0.356	5.1E-04	1.1E-04	2.3E-03
KIRC	TNK2_AA_15.1_14_15.2	TNK2	AA	15.1	14	15.2	9.2E-17	0.164	0.418	1.8E-03	1.0E-03	7.0E-02
KIRC	TNK2_ES_7_6_8	TNK2	ES	7	6	8	7.9E-18	0.175	0.385	4.8E-04	2.2E-03	2.1E-03
KIRC	TOM1L2_ES_14_13_15	TOM1L2	ES	14	13	15	1.4E-12	0.127	0.016	1.4E-01	1.0E-01	1.1E-01
KIRC	TOP3B_ES_6_5_7	TOP3B	ES	6	5	7	1.9E-19	0.194	0.631	2.2E-04	2.5E-04	5.4E-03
KIRC	TOP3B_RI_15.2_15.1_15.3	TOP3B	RI	15.2	15.1	15.3	4.8E-21	0.205	0.648	1.9E-04	1.9E-03	2.7E-02
KIRC	TOP3B_RI_16.2_16.1_16.3	TOP3B	RI	16.2	16.1	16.3	1.2E-17	0.171	0.698	2.5E-05	5.1E-04	3.3E-02
KIRC	TOPORS_ES_2_1_3	TOPORS	ES	2	1	3	3.5E-15	0.155	0.312	9.5E-03	8.1E-03	1.3E-01
KIRC	TOR2A_RI_3.2_3.1_3.3	TOR2A	RI	3.2	3.1	3.3	1.9E-28	0.262	0.017	4.9E-04	1.8E-03	3.6E-01
KIRC	TP53BP2_ES_3_2_4	TP53BP2	ES	3	2	4	2.2E-31	0.284	-0.459	3.1E-06	1.4E-05	3.4E-03
KIRC	TP53I3_RI_1.2_1.1_1.3	TP53I3	RI	1.2	1.1	1.3	1.4E-29	0.271	-0.023	3.0E-07	1.8E-05	2.1E-04
KIRC	TPCN2_ES_17_16_18	TPCN2	ES	17	16	18	4.6E-09	0.104	0.291	9.2E-03	3.1E-03	4.5E-02
KIRC	TPI1_AD_2.2_2.1_3	TPI1	AD	2.2	2.1	3	5.3E-14	0.138	-0.241	1.7E-04	9.5E-06	1.1E-02
KIRC	TPM1_AA_2.1_1_2.2	TPM1	AA	2.1	1	2.2	2.6E-17	0.168	-0.087	2.2E-06	1.2E-07	1.2E-04
KIRC	TPM1_ES_12.1:12.2_11.1_13.1	TPM1	ES	12.1:12.2	11.1	13.1	2.9E-17	0.171	0.096	6.2E-01	4.3E-01	8.3E-01
KIRC	TPM1_ES_3.2:5.2:6:7:8:10:11.1:12.1:13.[illegible]	TPM1	ES	:6:7:8:10:11.1:12	3.1	13.2	7.0E-10	0.107	-0.111	7.8E-01	7.9E-01	6.0E-01
KIRC	TPM1_ES_8_7_9	TPM1	ES	8	7	9	3.5E-16	0.157	0.126	4.0E-02	1.5E-02	4.6E-02
KIRC	TRA2A_ES_3.1:3.2_1_4	TRA2A	ES	3.1:3.2	1	4	9.6E-27	0.248	0.663	7.7E-04	2.2E-04	3.7E-04
KIRC	TRA2B_ES_2_1_3	TRA2B	ES	2	1	3	8.6E-13	0.126	0.226	4.5E-03	5.8E-04	2.5E-03
KIRC	TRAFD1_ES_5_4_6.1	TRAFD1	ES	5	4	6.1	5.4E-32	0.288	-0.206	5.9E-03	1.6E-04	3.4E-03
KIRC	TRAPPC10_ES_8_6_9	TRAPPC10	ES	8	6	9	8.5E-23	0.240	0.079	2.8E-03	7.3E-04	2.7E-02
KIRC	TRAPPC2L_AD_2.2_2.1_3.2	TRAPPC2L	AD	2.2	2.1	3.2	4.1E-20	0.222	-0.158	4.4E-06	4.9E-06	8.1E-04
KIRC	TRIM13_ES_2_1.4_3	TRIM13	ES	2	1.4	3	2.0E-20	0.195	-0.185	1.4E-09	2.1E-09	9.1E-05
KIRC	TRIM13_RI_1.2:1.3_1.1_1.4	TRIM13	RI	1.2:1.3	1.1	1.4	8.7E-28	0.256	-0.156	3.7E-05	3.8E-07	2.4E-04
KIRC	TRIM13_RI_1.3_1.2_1.4	TRIM13	RI	1.3	1.2	1.4	3.7E-27	0.251	-0.094	2.6E-07	8.3E-07	1.5E-02
KIRC	TRIM5_RI_12.2_12.1_12.3	TRIM5	RI	12.2	12.1	12.3	1.7E-28	0.262	-0.142	2.9E-05	4.0E-05	4.1E-04
KIRC	TRIM6_AD_4.2_4.1_5	TRIM6	AD	4.2	4.1	5	1.6E-18	0.198	-0.212	4.2E-05	6.0E-05	9.7E-04
KIRC	TRIM69_RI_4.2:4.3_4.1_4.4	TRIM69	RI	4.2:4.3	4.1	4.4	2.3E-10	0.107	-0.011	1.1E-05	1.9E-06	3.1E-02
KIRC	TRIP10_AA_12.1_11.2_12.2	TRIP10	AA	12.1	11.2	12.2	9.6E-13	0.127	0.276	4.4E-06	4.3E-02	7.5E-01
KIRC	TRIP10_ES_11.1:11.2_10_12.2	TRIP10	ES	11.1:11.2	10	12.2	3.4E-15	0.149	0.142	1.0E-01	2.4E-02	4.4E-02
KIRC	TRMT11_ES_8_7_9.1	TRMT11	ES	8	7	9.1	2.5E-20	0.199	0.320	2.1E-01	2.6E-01	1.4E-01
KIRC	TRMT13_RI_6.3_6.2_6.4	TRMT13	RI	6.3	6.2	6.4	2.8E-20	0.228	0.529	1.4E-02	1.2E-01	2.7E-03
KIRC	TRMT2B_AD_2.2:2.3_2.1_3	TRMT2B	AD	2.2:2.3	2.1	3	2.6E-10	0.107	-0.192	3.2E-02	7.7E-03	8.9E-02
KIRC	TRMT2B_RI_2.2_2.1_2.3	TRMT2B	RI	2.2	2.1	2.3	1.7E-10	0.110	-0.223	2.2E-04	5.6E-04	2.1E-02
KIRC	TRMU_AA_6.1_5_6.2	TRMU	AA	6.1	5	6.2	1.5E-14	0.142	-0.290	9.4E-05	1.1E-03	3.5E-03
KIRC	TRNT1_ES_4_2_5	TRNT1	ES	4	2	5	6.9E-25	0.232	-0.331	6.9E-04	5.8E-02	1.1E-01
KIRC	TRO_RI_12.2_12.1_12.3	TRO	RI	12.2	12.1	12.3	4.0E-13	0.129	-0.464	5.1E-03	3.6E-02	4.8E-03
KIRC	TRPM4_AD_11.2_11.1_12	TRPM4	AD	11.2	11.1	12	1.6E-21	0.208	-0.189	1.4E-03	1.4E-04	4.2E-02

cancer type	id	Gene Symbol	splice_type	Exon	From.Exon	To.Exon	anova.p	adj.r2	r	p.50	p.25	p.10
KIRC	TSC2_ES_27_26_28.1	TSC2	ES	27	26	28.1	1.9E-16	0.161	0.314	1.9E-02	9.9E-03	7.1E-05
KIRC	TSC2_ES_27:28.1_26_28.2	TSC2	ES	27:28.1	26	28.2	8.1E-16	0.156	0.325	1.3E-01	1.9E-02	1.3E-04
KIRC	TSPAN17_RI_7.5_7.4_7.6	TSPAN17	RI	7.5	7.4	7.6	5.1E-19	0.182	0.119	3.1E-01	1.0E-03	3.9E-03
KIRC	TSSC4_ES_2_1_3.2	TSSC4	ES	2	1	3.2	8.4E-16	0.154	0.198	7.6E-05	2.3E-05	2.8E-03
KIRC	TTC12_ES_7_6_8	TTC12	ES	7	6	8	2.2E-17	0.173	-0.263	1.1E-04	7.7E-05	4.8E-03
KIRC	TTC13_ME_9|10_8_11	TTC13	ME	9|10	8	11	3.1E-10	0.109	-0.092	9.4E-01	1.3E-01	9.8E-02
KIRC	TTC14_AD_5.2_5.1_5.4	TTC14	AD	5.2	5.1	5.4	1.6E-19	0.209	0.377	3.0E-06	1.3E-05	4.3E-06
KIRC	TTC14_RI_3.9_3.8_3.1	TTC14	RI	3.9	3.8	3.1	9.6E-23	0.214	0.256	1.8E-04	1.0E-04	1.1E-01
KIRC	TTC14_RI_5.2:5.3_5.1_5.4	TTC14	RI	5.2:5.3	5.1	5.4	5.8E-18	0.174	0.667	6.2E-04	5.1E-04	1.9E-02
KIRC	TTC14_RI_5.5_5.4_5.6	TTC14	RI	5.5	5.4	5.6	3.3E-19	0.184	0.649	1.0E-03	7.7E-06	2.4E-03
KIRC	TTC23_AA_14.1_13.1_14.2	TTC23	AA	14.1	13.1	14.2	1.4E-15	0.156	-0.035	3.8E-04	1.0E-04	1.6E-03
KIRC	TTC31_RI_5.2_5.1_5.3	TTC31	RI	5.2	5.1	5.3	1.6E-31	0.287	0.527	2.5E-05	1.5E-05	4.8E-02
KIRC	TTC31_RI_9.2_9.1_9.3	TTC31	RI	9.2	9.1	9.3	8.1E-23	0.217	0.460	5.7E-04	2.0E-04	1.8E-04
KIRC	TTC31_RI_9.4_9.3_9.5	TTC31	RI	9.4	9.3	9.5	9.5E-24	0.226	0.407	1.8E-04	1.7E-04	1.4E-02
KIRC	TTC31_RI_9.6_9.5_9.7	TTC31	RI	9.6	9.5	9.7	2.4E-24	0.230	0.435	3.2E-04	8.2E-04	2.1E-02
KIRC	TTC8_ES_6_2_7	TTC8	ES	6	2	7	2.2E-30	0.276	-0.058	8.3E-06	3.7E-06	6.7E-03
KIRC	TTI2_RI_1.2_1.1_1.3	TTI2	RI	1.2	1.1	1.3	4.2E-19	0.183	-0.214	6.0E-06	2.0E-05	3.6E-04
KIRC	TUBB3_ES_6.1:6.2_5.4_7.1	TUBB3	ES	6.1:6.2	5.4	7.1	1.9E-21	0.225	-0.626	3.3E-02	2.5E-01	1.1E-01
KIRC	TUBB4A_ES_5_3.1_6.1	TUBB4A	ES	5	3.1	6.1	6.0E-21	0.240	-0.380	2.2E-04	2.7E-04	4.3E-04
KIRC	TUBD1_ES_4_3_5	TUBD1	ES	4	3	5	2.7E-14	0.158	-0.182	1.3E-02	1.5E-02	1.9E-04
KIRC	TUBE1_AA_9.1_8_9.2	TUBE1	AA	9.1	8	9.2	1.9E-25	0.245	0.426	6.1E-06	1.1E-05	6.3E-03
KIRC	TUBGCP2_ES_7_6_8	TUBGCP2	ES	7	6	8	1.5E-29	0.271	0.030	1.7E-05	3.0E-04	7.2E-03
KIRC	TUBGCP6_AD_16.2_16.1_17	TUBGCP6	AD	16.2	16.1	17	5.4E-20	0.194	0.736	7.2E-05	3.4E-05	1.1E-02
KIRC	TUFT1_ES_2:3_1_4	TUFT1	ES	2:03	1	4	1.0E-11	0.152	-0.152	4.7E-04	2.0E-05	2.9E-04
KIRC	TXN2_ES_4:5_3.2_6	TXN2	ES	4:05	3.2	6	4.0E-11	0.114	-0.223	5.4E-03	1.4E-03	3.1E-02
KIRC	TXNDC9_ES_4_3_5.1	TXNDC9	ES	4	3	5.1	9.8E-13	0.125	-0.095	4.0E-01	8.3E-01	4.8E-01
KIRC	TXNL4A_ES_6:7.2_3_9	TXNL4A	ES	06:07.2	3	9	7.6E-11	0.111	-0.407	4.9E-02	2.6E-01	4.2E-01
KIRC	TXNRD2_RI_19.2_19.1_19.3	TXNRD2	RI	19.2	19.1	19.3	1.2E-11	0.115	-0.195	9.1E-05	3.9E-03	3.8E-02
KIRC	TYMP_AA_2.1_1_2.2	TYMP	AA	2.1	1	2.2	4.2E-14	0.140	-0.006	1.8E-01	1.0E-01	1.1E-01
KIRC	TYMP_AA_2.1:2.2_1_2.3	TYMP	AA	2.1:2.2	1	2.3	2.0E-14	0.142	0.012	3.8E-02	1.4E-02	6.5E-02
KIRC	U2AF1L4_AA_3.1_2.2_3.2	U2AF1L4	AA	3.1	2.2	3.2	3.8E-11	0.110	0.449	7.2E-04	5.3E-04	2.4E-02
KIRC	U2SURP_ES_13_12_14	U2SURP	ES	13	12	14	1.0E-17	0.173	-0.414	6.2E-02	4.4E-02	1.8E-01
KIRC	UAP1L1_RI_2.2_2.1_2.3	UAP1L1	RI	2.2	2.1	2.3	1.1E-35	0.347	0.439	8.4E-05	4.8E-06	9.4E-05
KIRC	UBA5_AD_2.2_2.1_3	UBA5	AD	2.2	2.1	3	1.5E-17	0.172	0.188	8.9E-02	2.7E-02	7.7E-01
KIRC	UBALD1_RI_2.4_2.3_2.5	UBALD1	RI	2.4	2.3	2.5	9.7E-21	0.199	0.237	9.0E-05	5.6E-04	7.8E-04
KIRC	UBB_AD_2.2_2.1_3.1	UBB	AD	2.2	2.1	3.1	1.1E-11	0.116	-0.146	2.4E-05	1.4E-05	1.1E-04
KIRC	UBE2D4_ES_6_5.1_7.1	UBE2D4	ES	6	5.1	7.1	1.6E-24	0.230	-0.220	3.5E-05	1.6E-04	3.9E-03
KIRC	UBE2G2_ES_7_6_8	UBE2G2	ES	7	6	8	9.1E-37	0.324	0.376	9.3E-05	1.0E-04	1.6E-04
KIRC	UBE2I_AD_4.4_4.3_5.2	UBE2I	AD	4.4	4.3	5.2	3.1E-10	0.110	-0.218	1.3E-02	1.1E-01	9.9E-01
KIRC	UBE2N_RI_4.2:4.3_4.1_4.4	UBE2N	RI	4.2:4.3	4.1	4.4	7.0E-11	0.121	-0.139	6.9E-03	7.6E-02	2.3E-01

cancer type	id	Gene Symbol	splice_type	Exon	From.Exon	To.Exon	anova.p	adj.r2	r	p.50	p.25	p.10
KIRC	UBE3A_ES_2:3:4.1:4.2:5.1:5.2_1_6.2	UBE3A	ES	2:3:4.1:4.2:5.1:5.2	1	6.2	3.0E-16	0.183	-0.145	3.0E-04	6.0E-04	7.0E-02
KIRC	UBE3A_ES_4.1:4.2:5.1:5.2_3_6.2	UBE3A	ES	4.1:4.2:5.1:5.2	3	6.2	5.8E-30	0.280	-0.392	1.1E-04	2.6E-05	1.5E-03
KIRC	UBE3B_AA_8.3_8.1_8.4	UBE3B	AA	8.3	8.1	8.4	6.0E-15	0.146	-0.347	1.6E-02	5.7E-04	3.2E-04
KIRC	UBE3B_RI_8.2_8.1_8.3	UBE3B	RI	8.2	8.1	8.3	2.0E-16	0.166	-0.038	2.7E-04	2.3E-04	5.2E-03
KIRC	UBE3B_RI_8.2:8.3_8.1_8.4	UBE3B	RI	8.2:8.3	8.1	8.4	2.7E-15	0.149	-0.301	2.1E-06	4.1E-06	4.7E-06
KIRC	UBP1_ES_13_12_14	UBP1	ES	13	12	14	3.3E-26	0.247	0.001	3.8E-03	3.2E-03	1.5E-01
KIRC	UBR3_ES_31_30_32	UBR3	ES	31	30	32	9.9E-22	0.213	-0.254	4.0E-04	3.9E-03	5.4E-02
KIRC	UBXN4_RI_3.2_3.1_3.3	UBXN4	RI	3.2	3.1	3.3	3.5E-27	0.251	-0.210	1.2E-05	2.9E-06	5.3E-04
KIRC	UHRF2_ES_11_10_12	UHRF2	ES	11	10	12	4.1E-10	0.102	0.298	5.1E-02	8.1E-03	5.5E-02
KIRC	ULK3_RI_6.2_6.1_6.3	ULK3	RI	6.2	6.1	6.3	1.7E-26	0.246	0.674	1.3E-04	3.6E-04	8.5E-04
KIRC	UNC119_RI_4.2_4.1_4.3	UNC119	RI	4.2	4.1	4.3	7.3E-27	0.249	0.107	2.4E-04	4.9E-03	4.6E-02
KIRC	UNC50_RI_1.2:1.3_1.1_1.4	UNC50	RI	1.2:1.3	1.1	1.4	1.7E-13	0.164	-0.073	1.1E-03	9.2E-05	6.1E-05
KIRC	UNK_ES_6_2_7	UNK	ES	6	2	7	1.4E-09	0.102	0.277	1.8E-01	7.9E-03	7.2E-02
KIRC	UPF3A_ES_4_3_5	UPF3A	ES	4	3	5	2.1E-12	0.122	0.206	1.2E-04	1.0E-04	2.5E-02
KIRC	URGCP_AA_4.2:4.3:4.4:4.5_3_4.6	URGCP	AA	4.2:4.3:4.4:4.5	3	4.6	1.1E-17	0.209	0.293	1.6E-02	3.7E-02	6.6E-01
KIRC	URGCP_RI_4.4:4.5_4.3_4.6	URGCP	RI	4.4:4.5	4.3	4.6	4.4E-28	0.259	0.298	2.2E-06	2.0E-06	9.9E-04
KIRC	USHBP1_AA_7.1:7.2_6_7.3	USHBP1	AA	7.1:7.2	6	7.3	4.7E-11	0.109	-0.160	3.2E-05	3.5E-09	1.0E-03
KIRC	USHBP1_RI_3.2_3.1_3.3	USHBP1	RI	3.2	3.1	3.3	8.5E-16	0.153	-0.266	3.5E-08	3.5E-09	7.6E-05
KIRC	USP3_ES_2_1_3.2	USP3	ES	2	1	3.2	2.0E-18	0.178	-0.180	7.1E-03	3.6E-03	4.0E-04
KIRC	USP33_ES_2_1_3	USP33	ES	2	1	3	1.3E-23	0.222	-0.258	3.4E-08	1.6E-04	4.8E-03
KIRC	USP36_RI_21.2_21.1_21.3	USP36	RI	21.2	21.1	21.3	5.8E-20	0.192	0.421	9.5E-03	1.1E-01	9.9E-01
KIRC	USP42_ES_3_2_4	USP42	ES	3	2	4	6.4E-25	0.278	-0.143	9.1E-03	2.6E-03	1.9E-03
KIRC	USP42_RI_8.2_8.1_8.3	USP42	RI	8.2	8.1	8.3	4.5E-11	0.121	-0.095	6.5E-04	1.4E-04	4.9E-02
KIRC	VAMP2_RI_5.2_5.1_5.3	VAMP2	RI	5.2	5.1	5.3	4.1E-18	0.175	-0.066	3.0E-04	4.9E-04	3.7E-02
KIRC	VCL_ES_19_18.2_20	VCL	ES	19	18.2	20	1.9E-18	0.178	0.130	5.3E-02	6.7E-02	1.2E-01
KIRC	VIM_ES_4:5:6:7.1:7.2:7.3:7.5_3.2_8	VIM	ES	:5:6:7.1:7.2:7.3:7.	3.2	8	5.9E-13	0.132	-0.199	3.1E-04	6.3E-03	1.5E-01
KIRC	VIM_ES_4:5:6:7.1:7.3:7.4:7.5_3.2_8	VIM	ES	:5:6:7.1:7.3:7.4:7.	3.2	8	2.9E-13	0.135	-0.218	1.6E-03	1.7E-03	2.0E-01
KIRC	VPS16_RI_12.3_12.2_12.4	VPS16	RI	12.3	12.2	12.4	3.7E-32	0.291	0.414	4.4E-04	5.5E-07	1.1E-04
KIRC	VPS28_RI_3.5:3.6_3.4_3.7	VPS28	RI	3.5:3.6	3.4	3.7	1.2E-11	0.130	-0.030	7.9E-04	6.0E-04	1.2E-01
KIRC	VPS29_AD_3.2_3.1_5	VPS29	AD	3.2	3.1	5	3.5E-16	0.158	-0.206	7.7E-02	5.8E-02	1.1E-01
KIRC	VPS8_ES_3_2_4	VPS8	ES	3	2	4	2.0E-27	0.257	-0.123	1.4E-04	1.6E-07	1.4E-04
KIRC	VPS8_RI_7.2_7.1_7.3	VPS8	RI	7.2	7.1	7.3	2.0E-29	0.276	0.102	5.8E-04	1.8E-03	3.7E-03
KIRC	VRK3_AD_13.2_13.1_15	VRK3	AD	13.2	13.1	15	7.0E-31	0.297	-0.408	1.1E-06	2.4E-06	1.8E-04
KIRC	VSIG4_RI_7.2_7.1_7.3	VSIG4	RI	7.2	7.1	7.3	8.8E-30	0.273	-0.240	1.0E-01	7.9E-02	8.1E-04
KIRC	VWA5A_AD_1.2:1.3_1.1_2.2	VWA5A	AD	1.2:1.3	1.1	2.2	2.4E-10	0.112	-0.202	2.2E-08	1.6E-06	1.1E-05
KIRC	WDR11_RI_27.2_27.1_27.3	WDR11	RI	27.2	27.1	27.3	5.6E-37	0.326	-0.098	1.4E-04	3.4E-06	4.5E-03
KIRC	WDR13_RI_1.2_1.1_1.3	WDR13	RI	1.2	1.1	1.3	6.4E-12	0.118	0.062	4.2E-03	4.3E-02	2.8E-02
KIRC	WDR24_ES_1.6:2:3_1.5_4	WDR24	ES	1.6:2:3	1.5	4	1.6E-12	0.124	-0.076	3.1E-01	1.6E-02	7.1E-01
KIRC	WDR33_RI_6.2_6.1_6.3	WDR33	RI	6.2	6.1	6.3	3.1E-28	0.259	-0.043	1.8E-05	3.1E-04	1.8E-02
KIRC	WDR41_ES_3_2_4	WDR41	ES	3	2	4	1.4E-25	0.238	-0.395	6.6E-03	2.2E-05	1.2E-04

cancer type	id	Gene Symbol	splice_type	Exon	From.Exon	To.Exon	anova.p	adj.r2	r	p.50	p.25	p.10
KIRC	WDR55_RI_2.2_2.1_2.3	WDR55	RI	2.2	2.1	2.3	3.7E-23	0.220	-0.032	6.9E-04	1.5E-03	7.4E-04
KIRC	WDR55_RI_4.2_4.1_4.3	WDR55	RI	4.2	4.1	4.3	7.0E-35	0.312	0.116	6.8E-04	9.1E-06	9.8E-04
KIRC	WDR6_AA_4.1_1_4.2	WDR6	AA	4.1	1	4.2	1.3E-11	0.116	0.163	5.1E-11	6.1E-07	4.9E-04
KIRC	WDR6_RI_4.5_4.4_4.6	WDR6	RI	4.5	4.4	4.6	1.1E-32	0.295	0.472	1.5E-04	1.0E-04	2.2E-04
KIRC	WDR75_AA_4.1_2_4.2	WDR75	AA	4.1	2	4.2	1.3E-23	0.222	0.338	1.0E-03	3.7E-06	7.0E-05
KIRC	WHSC1_AA_16.1_15.2_16.2	WHSC1	AA	16.1	15.2	16.2	5.6E-34	0.310	0.209	4.9E-04	1.4E-02	1.2E-03
KIRC	WNK1_ES_14_13.2_15	WNK1	ES	14	13.2	15	1.7E-09	0.110	-0.250	1.5E-04	2.3E-03	2.8E-02
KIRC	WNK1_ES_14:15_13.2_16	WNK1	ES	14:15	13.2	16	5.9E-10	0.100	-0.130	3.1E-03	1.5E-01	8.3E-02
KIRC	WRAP73_RI_11.2_11.1_11.3	WRAP73	RI	11.2	11.1	11.3	2.6E-17	0.167	0.402	2.9E-07	8.9E-06	1.5E-03
KIRC	WWC2_AD_20.2_20.1_21.2	WWC2	AD	20.2	20.1	21.2	1.1E-25	0.244	-0.144	3.5E-01	4.6E-01	1.9E-01
KIRC	XAF1_AA_4.1:4.2_2.1_4.3	XAF1	AA	4.1:4.2	2.1	4.3	7.0E-21	0.201	0.440	1.9E-03	3.0E-04	9.7E-03
KIRC	XAF1_ES_4.3:6_2.1_8	XAF1	ES	4.3:6	2.1	8	9.6E-10	0.110	-0.369	5.2E-01	4.1E-01	4.1E-01
KIRC	XAF1_ES_7_6_8	XAF1	ES	7	6	8	7.2E-13	0.129	0.356	6.3E-03	3.3E-03	2.3E-02
KIRC	XPNPEP3_ES_4_3_5	XPNPEP3	ES	4	3	5	1.9E-13	0.142	-0.005	3.7E-01	8.5E-03	2.6E-02
KIRC	YAF2_ES_5.1:5.2:6_2_9.1	YAF2	ES	5.1:5.2:6	2	9.1	2.9E-10	0.104	0.029	7.4E-01	3.4E-01	5.8E-01
KIRC	YME1L1_RI_3.2_3.1_3.3	YME1L1	RI	3.2	3.1	3.3	6.0E-14	0.137	-0.497	1.7E-02	2.0E-03	1.0E-02
KIRC	YPEL3_RI_2.2_2.1_2.3	YPEL3	RI	2.2	2.1	2.3	2.6E-13	0.131	0.148	6.6E-03	4.2E-02	4.4E-01
KIRC	YPEL5_ES_4_3.2_5	YPEL5	ES	4	3.2	5	3.0E-12	0.131	-0.199	2.7E-03	1.4E-02	1.8E-03
KIRC	ZBED5_AA_3.1_2_3.2	ZBED5	AA	3.1	2	3.2	1.6E-27	0.258	0.498	2.7E-05	2.1E-06	6.3E-04
KIRC	ZBTB1_ES_3_2.2_4.1	ZBTB1	ES	3	2.2	4.1	5.0E-12	0.128	0.160	2.0E-01	1.5E-01	4.1E-02
KIRC	ZBTB11_RI_2.2_2.1_2.3	ZBTB11	RI	2.2	2.1	2.3	8.1E-15	0.154	-0.187	4.5E-04	7.4E-04	4.0E-03
KIRC	ZBTB17_RI_9.2_9.1_9.3	ZBTB17	RI	9.2	9.1	9.3	3.8E-27	0.253	0.590	2.6E-03	3.1E-05	2.3E-05
KIRC	ZBTB38_ES_4_2_5	ZBTB38	ES	4	2	5	1.5E-10	0.107	-0.326	3.6E-03	4.2E-04	3.1E-03
KIRC	ZC4H2_ES_3_2_4.2	ZC4H2	ES	3	2	4.2	1.7E-15	0.163	-0.233	3.7E-03	9.9E-04	2.0E-03
KIRC	ZCCHC8_RI_6.2_6.1_6.3	ZCCHC8	RI	6.2	6.1	6.3	2.3E-16	0.161	0.319	1.7E-03	8.2E-05	4.7E-04
KIRC	ZCCHC9_RI_1.2:1.3:1.4_1.1_1.5	ZCCHC9	RI	1.2:1.3:1.4	1.1	1.5	2.2E-22	0.211	-0.070	3.8E-04	8.4E-04	1.3E-02
KIRC	ZCCHC9_RI_1.3:1.4_1.2_1.5	ZCCHC9	RI	1.3:1.4	1.2	1.5	7.3E-28	0.256	-0.056	5.3E-04	2.5E-06	6.0E-04
KIRC	ZDHHC16_ME_2|3_1_4	ZDHHC16	ME	2|3	1	4	7.5E-13	0.134	-0.099	4.8E-01	4.4E-01	2.6E-01
KIRC	ZDHHC17_ES_8_7_9	ZDHHC17	ES	8	7	9	2.0E-15	0.159	0.268	2.9E-02	6.8E-02	3.2E-01
KIRC	ZEB1_ES_3:4_1_5	ZEB1	ES	3:04	1	5	1.3E-19	0.196	-0.206	1.3E-01	2.1E-02	1.1E-03
KIRC	ZFAND2B_RI_4.10_4.9_4.11	ZFAND2B	RI	4.1	4.9	4.11	8.4E-26	0.240	0.177	2.5E-05	7.9E-04	7.5E-03
KIRC	ZFAND2B_RI_4.8_4.7_4.9	ZFAND2B	RI	4.8	4.7	4.9	1.3E-30	0.278	0.177	3.5E-04	2.2E-04	1.3E-03
KIRC	ZFAT_ES_3_1_4	ZFAT	ES	3	1	4	4.3E-12	0.139	-0.159	2.3E-01	7.1E-02	4.2E-02
KIRC	ZFC3H1_RI_32.2_32.1_32.3	ZFC3H1	RI	32.2	32.1	32.3	3.0E-25	0.235	0.450	2.3E-05	1.4E-03	1.1E-02
KIRC	ZFP2_ME_6|7_3_8	ZFP2	ME	6|7	3	8	2.2E-15	0.150	0.135	5.3E-08	1.3E-06	3.5E-02
KIRC	ZFP36_AD_1.2_1.1_2	ZFP36	AD	1.2	1.1	2	5.5E-25	0.234	0.158	5.0E-06	7.0E-06	1.2E-02
KIRC	ZFP90_AA_5.1_4.2_5.2	ZFP90	AA	5.1	4.2	5.2	6.0E-17	0.174	0.001	5.0E-05	5.2E-04	6.3E-03
KIRC	ZGPAT_AA_6.1_5.2_6.2	ZGPAT	AA	6.1	5.2	6.2	4.9E-14	0.137	0.311	3.2E-02	4.2E-02	1.6E-04
KIRC	ZKSCAN1_ES_2_1_3.1	ZKSCAN1	ES	2	1	3.1	2.6E-17	0.167	-0.451	4.3E-05	5.0E-05	3.9E-01
KIRC	ZMAT1_ES_2_1_3	ZMAT1	ES	2	1	3	4.9E-14	0.138	0.045	2.7E-04	5.6E-07	5.5E-02

cancer type	id	Gene Symbol	splice_type	Exon	From.Exon	To.Exon	anova.p	adj.r2	r	p.50	p.25	p.10
KIRC	ZNF148_ES_4_3.2_5	ZNF148	ES	4	3.2	5	2.3E-22	0.213	-0.596	7.9E-05	1.7E-04	3.1E-02
KIRC	ZNF180_ES_4_2.2_6	ZNF180	ES	4	2.2	6	2.1E-19	0.201	-0.243	4.5E-04	3.1E-04	2.8E-03
KIRC	ZNF195_ES_11_5.1_13	ZNF195	ES	11	5.1	13	7.0E-15	0.146	0.162	1.9E-05	1.1E-05	1.2E-03
KIRC	ZNF195_ES_5.2:11_5.1_13	ZNF195	ES	5.2:11	5.1	13	1.8E-12	0.126	0.147	7.7E-01	3.7E-01	1.8E-01
KIRC	ZNF195_ES_6:9:11_5.1_13	ZNF195	ES	6:09:11	5.1	13	3.2E-19	0.191	0.265	3.1E-01	1.6E-01	2.7E-02
KIRC	ZNF211_ES_3.1:3.2_2.2_4	ZNF211	ES	3.1:3.2	2.2	4	1.5E-08	0.102	0.121	7.3E-04	3.1E-02	3.5E-01
KIRC	ZNF223_ES_8_7.1_9.1	ZNF223	ES	8	7.1	9.1	2.9E-17	0.192	-0.264	1.4E-04	4.0E-03	1.8E-04
KIRC	ZNF226_RI_7.2_7.1_7.3	ZNF226	RI	7.2	7.1	7.3	2.8E-32	0.291	0.516	8.5E-04	3.6E-04	2.6E-02
KIRC	ZNF226_RI_7.2:7.3_7.1_7.4	ZNF226	RI	7.2:7.3	7.1	7.4	7.7E-22	0.207	0.585	4.3E-04	1.8E-04	2.2E-03
KIRC	ZNF232_RI_4.3_4.2_4.4	ZNF232	RI	4.3	4.2	4.4	2.1E-10	0.103	0.007	1.4E-01	6.3E-02	1.5E-01
KIRC	ZNF24_AA_4.1_3_4.2	ZNF24	AA	4.1	3	4.2	1.0E-18	0.180	-0.369	1.8E-07	7.5E-07	2.8E-05
KIRC	ZNF254_ES_4:5_3_6	ZNF254	ES	4:05	3	6	4.6E-12	0.119	-0.186	1.8E-03	5.6E-04	3.8E-02
KIRC	ZNF260_ES_3_2.2_5	ZNF260	ES	3	2.2	5	4.9E-25	0.234	-0.404	1.5E-04	6.4E-06	6.7E-03
KIRC	ZNF266_RI_6.3_6.2_6.4	ZNF266	RI	6.3	6.2	6.4	6.1E-17	0.164	0.315	3.8E-05	1.6E-05	7.0E-02
KIRC	ZNF266_RI_6.5_6.4_6.6	ZNF266	RI	6.5	6.4	6.6	5.4E-10	0.102	0.342	9.7E-06	4.0E-04	3.0E-01
KIRC	ZNF300_RI_6.2:6.3_6.1_6.4	ZNF300	RI	6.2:6.3	6.1	6.4	1.7E-10	0.106	0.233	5.2E-05	7.3E-08	3.0E-03
KIRC	ZNF302_AA_6.1_5.2_6.2	ZNF302	AA	6.1	5.2	6.2	4.8E-24	0.225	-0.040	1.8E-03	1.4E-04	2.7E-04
KIRC	ZNF302_ES_3_2_4	ZNF302	ES	3	2	4	2.2E-15	0.153	0.171	6.8E-03	4.1E-02	2.5E-01
KIRC	ZNF304_ES_4_3_5	ZNF304	ES	4	3	5	1.1E-18	0.224	-0.160	1.0E-01	2.4E-02	1.1E-02
KIRC	ZNF317_AA_5.1_4_5.2	ZNF317	AA	5.1	4	5.2	8.9E-32	0.294	-0.138	1.6E-06	2.7E-06	4.0E-05
KIRC	ZNF329_ES_5_4_6.1	ZNF329	ES	5	4	6.1	4.7E-33	0.298	-0.449	3.9E-07	3.3E-06	2.0E-04
KIRC	ZNF333_AA_12.1_9_12.2	ZNF333	AA	12.1	9	12.2	6.1E-14	0.152	0.364	1.4E-01	9.4E-02	3.7E-02
KIRC	ZNF333_ES_7_6_8	ZNF333	ES	7	6	8	1.3E-16	0.179	0.457	3.3E-06	8.3E-04	3.5E-01
KIRC	ZNF354A_ES_4_3_5	ZNF354A	ES	4	3	5	1.3E-23	0.222	0.107	7.5E-07	1.5E-06	1.3E-03
KIRC	ZNF383_AD_8.2_8.1_9	ZNF383	AD	8.2	8.1	9	7.5E-12	0.134	0.074	1.5E-05	3.3E-04	5.1E-04
KIRC	ZNF384_ES_5_4_6.1	ZNF384	ES	5	4	6.1	2.4E-14	0.140	-0.039	1.8E-03	2.3E-06	3.4E-03
KIRC	ZNF410_ES_14_13_16	ZNF410	ES	14	13	16	1.4E-32	0.293	-0.198	1.1E-04	4.1E-05	7.9E-04
KIRC	ZNF528_ES_9_7.1_10	ZNF528	ES	9	7.1	10	1.1E-11	0.115	0.345	6.6E-07	4.5E-04	7.2E-03
KIRC	ZNF544_ES_9:10.1_7.2_10.2	ZNF544	ES	09:10.1	7.2	10.2	1.2E-25	0.239	-0.280	3.5E-08	4.1E-06	9.9E-05
KIRC	ZNF548_ES_2:3_1_4	ZNF548	ES	2:03	1	4	4.9E-27	0.267	0.347	8.8E-05	1.5E-05	2.1E-04
KIRC	ZNF554_ES_6_5_7	ZNF554	ES	6	5	7	7.9E-17	0.163	-0.082	1.5E-09	3.7E-07	1.1E-03
KIRC	ZNF559_AD_3.2:3.3_3.1_4	ZNF559	AD	3.2:3.3	3.1	4	7.7E-12	0.126	0.178	4.1E-03	1.7E-02	3.6E-01
KIRC	ZNF559_RI_6.2_6.1_6.3	ZNF559	RI	6.2	6.1	6.3	1.3E-15	0.155	0.015	8.3E-05	2.6E-05	1.5E-06
KIRC	ZNF559_RI_6.2:6.3_6.1_6.4	ZNF559	RI	6.2:6.3	6.1	6.4	3.4E-12	0.122	-0.068	2.2E-05	7.0E-06	1.9E-05
KIRC	ZNF561_AA_6.1_4_6.2	ZNF561	AA	6.1	4	6.2	6.9E-15	0.149	-0.119	5.2E-03	2.3E-02	2.9E-01
KIRC	ZNF564_ES_2_1_4	ZNF564	ES	2	1	4	2.3E-28	0.264	-0.142	4.9E-07	3.8E-08	9.2E-07
KIRC	ZNF611_ES_10_8_12	ZNF611	ES	10	8	12	1.4E-24	0.242	-0.290	1.3E-07	1.7E-08	4.2E-04
KIRC	ZNF611_ES_6_5_7	ZNF611	ES	6	5	7	8.0E-09	0.104	-0.054	3.5E-03	3.6E-02	2.5E-01
KIRC	ZNF611_ME_9|10_8_12	ZNF611	ME	9|10	8	12	1.5E-11	0.128	0.260	2.8E-06	6.1E-04	1.2E-03
KIRC	ZNF655_AA_5.1:5.2_4_5.3	ZNF655	AA	5.1:5.2	4	5.3	4.6E-16	0.165	0.524	2.1E-06	1.3E-02	1.9E-01

cancer type	id	Gene Symbol	splice_type	Exon	From.Exon	To.Exon	anova.p	adj.r2	r	p.50	p.25	p.10
KIRC	ZNF655_ES_4:5.1:5.2:5.3_3.2_7	ZNF655	ES	4:5.1:5.2:5.3	3.2	7	6.2E-09	0.104	0.378	4.0E-05	7.4E-04	6.3E-02
KIRC	ZNF655_ES_4:9_3.2_10	ZNF655	ES	4:09	3.2	10	3.1E-10	0.125	0.168	1.4E-02	1.1E-01	2.4E-01
KIRC	ZNF655_ES_6.1:6.2_5.3_7	ZNF655	ES	6.1:6.2	5.3	7	2.5E-14	0.143	0.517	6.7E-02	3.7E-01	7.9E-01
KIRC	ZNF671_AD_1.2_1.1_2	ZNF671	AD	1.2	1.1	2	3.6E-13	0.157	0.025	8.8E-04	6.0E-02	4.4E-02
KIRC	ZNF687_ES_2_1_3	ZNF687	ES	2	1	3	6.3E-22	0.208	-0.323	6.7E-07	3.3E-05	3.6E-04
KIRC	ZNF691_ES_2.1:2.2:2.3_1_4	ZNF691	ES	2.1:2.2:2.3	1	4	5.2E-21	0.204	0.115	5.2E-04	2.4E-04	8.5E-04
KIRC	ZNF691_ES_3_2.3_4	ZNF691	ES	3	2.3	4	5.7E-12	0.124	0.124	2.5E-06	3.2E-05	3.3E-04
KIRC	ZNF691_RI_2.2_2.1_2.3	ZNF691	RI	2.2	2.1	2.3	4.9E-16	0.178	0.125	2.3E-05	7.7E-07	2.2E-03
KIRC	ZNF692_RI_3.5_3.4_3.6	ZNF692	RI	3.5	3.4	3.6	5.4E-17	0.169	0.684	1.6E-05	5.1E-06	1.3E-01
KIRC	ZNF692_RI_6.3_6.2_6.4	ZNF692	RI	6.3	6.2	6.4	5.0E-11	0.110	0.516	5.8E-03	7.3E-04	2.3E-02
KIRC	ZNF701_ES_3.1:3.2_1_4	ZNF701	ES	3.1:3.2	1	4	9.6E-15	0.154	0.013	2.0E-02	4.4E-02	2.3E-01
KIRC	ZNF706_RI_3.3_3.2_3.4	ZNF706	RI	3.3	3.2	3.4	2.1E-10	0.102	0.024	3.3E-02	3.1E-02	9.4E-03
KIRC	ZNF707_ES_4.2:5.1:5.2_1_6	ZNF707	ES	4.2:5.1:5.2	1	6	1.2E-11	0.142	0.432	4.8E-06	1.3E-06	2.5E-03
KIRC	ZNF720_AD_1.2_1.1_2	ZNF720	AD	1.2	1.1	2	6.4E-11	0.116	0.049	1.6E-02	8.8E-04	1.3E-02
KIRC	ZNF74_ES_2:3_1.3_4	ZNF74	ES	2:03	1.3	4	6.6E-09	0.107	0.018	2.1E-01	1.9E-02	5.8E-04
KIRC	ZNF74_ES_3_2_4	ZNF74	ES	3	2	4	1.0E-15	0.159	0.071	3.2E-03	1.5E-03	1.9E-03
KIRC	ZNF76_AA_10.1_9_10.2	ZNF76	AA	10.1	9	10.2	1.1E-31	0.294	0.402	1.2E-07	2.0E-08	1.0E-04
KIRC	ZNF76_AA_12.1:12.2_11_12.3	ZNF76	AA	12.1:12.2	11	12.3	1.1E-25	0.241	0.275	1.6E-04	1.5E-06	1.5E-03
KIRC	ZNF76_RI_12.2_12.1_12.3	ZNF76	RI	12.2	12.1	12.3	2.1E-25	0.237	0.246	4.0E-05	1.6E-05	7.4E-03
KIRC	ZNF76_RI_12.4_12.3_12.5	ZNF76	RI	12.4	12.3	12.5	1.0E-26	0.248	0.285	3.2E-06	6.1E-08	8.3E-06
KIRC	ZNF766_ES_7_6_8.1	ZNF766	ES	7	6	8.1	9.1E-24	0.225	-0.313	5.9E-06	1.3E-03	1.4E-03
KIRC	ZNF773_RI_6.2_6.1_6.3	ZNF773	RI	6.2	6.1	6.3	6.6E-13	0.131	0.049	7.3E-04	4.5E-04	3.9E-02
KIRC	ZNF793_RI_10.2:10.3_10.1_10.4	ZNF793	RI	10.2:10.3	10.1	10.4	7.1E-14	0.136	-0.110	1.2E-03	8.2E-04	8.9E-02
KIRC	ZNF799_ES_3_1_4	ZNF799	ES	3	1	4	9.2E-21	0.198	0.134	1.9E-07	4.4E-05	1.8E-03
KIRC	ZNF83_ES_8_7_9.6	ZNF83	ES	8	7	9.6	1.1E-12	0.129	0.601	1.2E-06	2.2E-05	2.9E-03
KIRC	ZNF83_ES_8:9.6_7_10.1	ZNF83	ES	08:09.6	7	10.1	3.0E-24	0.229	0.768	4.5E-06	1.1E-03	1.6E-02
KIRC	ZRANB2_ES_10_9_11	ZRANB2	ES	10	9	11	4.1E-18	0.174	0.328	1.4E-02	9.0E-04	1.3E-02
KIRC	ZSWIM7_ES_3_2_4	ZSWIM7	ES	3	2	4	1.1E-20	0.197	0.043	4.4E-06	5.4E-07	7.2E-06
KIRC	ZSWIM7_RI_7.6_7.5_7.7	ZSWIM7	RI	7.6	7.5	7.7	3.4E-14	0.139	0.234	1.1E-05	7.5E-05	2.7E-02
KIRC	ZWINT_RI_2.2_2.1_2.3	ZWINT	RI	2.2	2.1	2.3	1.9E-33	0.307	0.069	2.4E-05	1.4E-07	6.7E-06
KIRP	ABCA2_RI_4.2:4.3:4.4:4.5_4.1_4.6	ABCA2	RI	4.2:4.3:4.4:4.5	4.1	4.6	5.0E-04	0.108	0.013	3.6E-01	7.5E-01	3.7E-01
KIRP	ABCC5_RI_7.2:7.3_7.1_7.4	ABCC5	RI	7.2:7.3	7.1	7.4	1.3E-07	0.185	0.278	2.0E-01	1.3E-01	5.6E-01
KIRP	ABCC6_ES_3_2.1_4	ABCC6	ES	3	2.1	4	2.8E-05	0.153	-0.211	1.8E-01	2.0E-01	3.2E-01
KIRP	ABCD4_RI_18.2_18.1_18.3	ABCD4	RI	18.2	18.1	18.3	1.6E-04	0.103	0.103	1.6E-01	1.8E-01	5.7E-02
KIRP	ABI1_ES_5_4_7	ABI1	ES	5	4	7	5.1E-05	0.126	-0.052	2.7E-01	3.4E-01	8.6E-01
KIRP	ABI3BP_ES_22:23:24:25:26:27:28:29:30:	ABI3BP	ES	:25:26:27:28:29:	21	51	1.2E-04	0.125	0.041	1.8E-01	7.7E-02	1.4E-01
KIRP	ABLIM1_ES_16_15_17	ABLIM1	ES	16	15	17	8.9E-14	0.354	0.122	5.2E-02	1.5E-01	3.0E-03
KIRP	ACAA1_AD_9.2:9.3_9.1_10	ACAA1	AD	9.2:9.3	9.1	10	1.7E-04	0.102	-0.138	6.8E-01	7.5E-01	9.3E-01
KIRP	ACBD4_AA_8.1_7_8.2	ACBD4	AA	8.1	7	8.2	1.9E-05	0.128	-0.124	3.5E-01	5.0E-01	7.6E-01
KIRP	ACLY_ES_14_13_15	ACLY	ES	14	13	15	2.4E-06	0.155	0.136	1.9E-02	3.9E-03	2.5E-02

cancer type	id	Gene Symbol	splice_type	Exon	From.Exon	To.Exon	anova.p	adj.r2	r	p.50	p.25	p.10
KIRP	ACMSD_ES_5_4_6	ACMSD	ES	5	4	6	3.2E-07	0.178	0.196	7.3E-04	1.3E-02	2.0E-02
KIRP	ACSM2B_ES_2.1:2.2_1_3	ACSM2B	ES	2.1:2.2	1	3	7.7E-05	0.112	-0.742	3.5E-01	7.5E-01	9.5E-01
KIRP	ADAM15_ES_21.1:21.2_20_22.1	ADAM15	ES	21.1:21.2	20	22.1	6.2E-11	0.272	-0.310	2.2E-01	1.0E-01	2.8E-01
KIRP	ADAM15_ES_21.1:21.2:22.1_20_23	ADAM15	ES	21.1:21.2:22.1	20	23	2.0E-04	0.101	-0.096	7.0E-01	9.2E-01	9.8E-01
KIRP	ADAM15_ES_21.2_20_22.1	ADAM15	ES	21.2	20	22.1	7.0E-12	0.292	-0.338	5.3E-01	3.3E-01	4.1E-01
KIRP	ADAM15_ES_21.2:22.1_20_23	ADAM15	ES	21.2:22.1	20	23	1.7E-05	0.130	-0.103	7.7E-01	2.4E-01	9.8E-01
KIRP	ADCK1_ES_5_3_6	ADCK1	ES	5	3	6	8.5E-04	0.106	0.147	2.1E-02	1.7E-02	4.4E-03
KIRP	ADHFE1_ES_4_3_5	ADHFE1	ES	4	3	5	9.3E-04	0.105	-0.127	2.1E-03	1.8E-01	1.1E-01
KIRP	ADNP_ES_2_1_4.2	ADNP	ES	2	1	4.2	2.7E-04	0.116	0.043	9.7E-02	2.9E-02	2.9E-01
KIRP	AFMID_ES_5:6:7:8:9:10:11.1:11.2:12_2_	AFMID	ES	7:8:9:10:11.1:11.	2	13	9.9E-08	0.195	0.156	7.3E-01	1.7E-01	2.6E-01
KIRP	AFMID_ES_5:6:7:8:9:10:11.1:12_2_13	AFMID	ES	:6:7:8:9:10:11.1:1	2	13	1.2E-09	0.234	0.154	7.0E-01	3.3E-01	6.8E-02
KIRP	AFMID_ES_5:6:8:9:10:11.1:11.2:12_2_1	AFMID	ES	:8:9:10:11.1:11.2	2	13	1.2E-04	0.143	0.325	2.0E-01	1.0E+00	5.3E-01
KIRP	AFMID_ES_7:8_6_12	AFMID	ES	7:08	6	12	1.8E-08	0.211	0.110	2.3E-02	6.5E-02	1.1E-03
KIRP	AFMID_ES_7:8:9:10:11.1_6_12	AFMID	ES	7:8:9:10:11.1	6	12	3.9E-05	0.120	0.167	2.7E-01	3.3E-01	8.8E-01
KIRP	AFMID_ES_7:8:9:10:11.1:11.2:12_6_13	AFMID	ES	8:9:10:11.1:11.2:	6	13	4.5E-04	0.101	0.152	5.5E-01	8.4E-01	7.8E-01
KIRP	AFMID_ES_7:8:9:10:11.1:12_6_13	AFMID	ES	7:8:9:10:11.1:12	6	13	3.4E-05	0.139	0.125	3.6E-01	1.3E-01	2.6E-01
KIRP	AFMID_ES_8_6_12	AFMID	ES	8	6	12	1.4E-10	0.265	0.219	3.0E-02	1.2E-02	1.5E-02
KIRP	AFMID_ES_8:9:10:11.1_6_12	AFMID	ES	8:9:10:11.1	6	12	2.8E-07	0.178	0.409	5.3E-01	5.3E-01	4.0E-01
KIRP	AFMID_ES_8:9:10:11.1:12_6_13	AFMID	ES	8:9:10:11.1:12	6	13	3.7E-04	0.104	0.386	2.9E-01	4.7E-01	2.9E-01
KIRP	AFMID_ES_9:10:11.1_8_12	AFMID	ES	10:11.1	8	12	4.8E-08	0.201	0.128	5.0E-02	5.2E-03	1.9E-01
KIRP	AFMID_ES_9:10:11.1:11.2_8_12	AFMID	ES	9:10:11.1:11.2	8	12	1.7E-05	0.134	0.107	1.5E-02	2.4E-03	2.1E-01
KIRP	AKAP17A_ES_4.3_4.1_5	AKAP17A	ES	4.3	4.1	5	2.9E-10	0.281	-0.223	7.1E-01	8.5E-01	8.5E-01
KIRP	AKAP17A_RI_4.2_4.1_4.3	AKAP17A	RI	4.2	4.1	4.3	2.8E-06	0.151	0.388	5.7E-02	4.7E-01	4.4E-01
KIRP	AKIP1_ES_2_1.3_3	AKIP1	ES	2	1.3	3	8.0E-05	0.111	-0.033	4.6E-02	1.5E-02	2.0E-02
KIRP	ALS2CL_AA_18.3_18.1_18.4	ALS2CL	AA	18.3	18.1	18.4	1.2E-04	0.131	0.207	4.4E-01	5.3E-02	3.8E-01
KIRP	AMDHD2_RI_8.2_8.1_8.3	AMDHD2	RI	8.2	8.1	8.3	1.8E-05	0.129	-0.246	5.9E-01	1.4E-01	8.4E-01
KIRP	AMPD2_ES_2.2_1_4	AMPD2	ES	2.2	1	4	8.0E-04	0.107	-0.002	3.0E-02	1.1E-01	7.8E-02
KIRP	ANAPC11_ES_3.3:3.4:6_3.2_7.2	ANAPC11	ES	3.3:3.4:6	3.2	7.2	1.6E-06	0.156	-0.263	2.0E-01	1.8E-02	7.1E-02
KIRP	ANAPC5_AA_9.1_8.3_9.2	ANAPC5	AA	9.1	8.3	9.2	5.1E-13	0.305	-0.046	2.2E-01	4.2E-02	1.5E-01
KIRP	ANKRD13D_ES_2_1_3.1	ANKRD13D	ES	2	1	3.1	3.9E-05	0.134	0.053	2.8E-01	9.1E-01	4.7E-01
KIRP	ANKRD49_RI_2.5:2.6:2.7_2.4_2.8	ANKRD49	RI	2.5:2.6:2.7	2.4	2.8	2.2E-06	0.191	0.298	5.1E-01	9.5E-01	3.8E-01
KIRP	ANKS3_ES_4_3_5.1	ANKS3	ES	4	3	5.1	1.4E-07	0.184	-0.019	2.9E-05	6.1E-04	7.8E-03
KIRP	ANKS3_ES_5.1:5.2_3_6	ANKS3	ES	5.1:5.2	3	6	6.0E-11	0.273	-0.061	5.0E-01	2.8E-01	3.6E-01
KIRP	ANO8_AA_13.1_12_13.2	ANO8	AA	13.1	12	13.2	5.0E-05	0.128	0.178	7.0E-01	9.7E-01	1.8E-01
KIRP	ANXA13_ES_2_1_3	ANXA13	ES	2	1	3	4.2E-07	0.193	-0.026	1.7E-01	1.7E-03	1.4E-02
KIRP	AP1B1_ES_24_23_25	AP1B1	ES	24	23	25	6.4E-12	0.282	-0.009	9.1E-01	5.7E-01	2.3E-01
KIRP	AP2A1_ES_16_15_17	AP2A1	ES	16	15	17	3.5E-06	0.147	0.215	9.8E-01	7.1E-01	6.0E-01
KIRP	AP2B1_ES_16_15_17	AP2B1	ES	16	15	17	2.3E-11	0.274	-0.060	7.5E-02	3.6E-01	6.6E-02
KIRP	AP2M1_ES_7_6_8	AP2M1	ES	7	6	8	1.6E-04	0.103	0.064	1.1E-01	3.1E-01	3.5E-01
KIRP	APLP2_ES_9_8_10	APLP2	ES	9	8	10	2.2E-06	0.153	-0.225	3.7E-01	1.0E-01	3.0E-02

cancer type	id	Gene Symbol	splice_type	Exon	From.Exon	To.Exon	anova.p	adj.r2	r	p.50	p.25	p.10
KIRP	APOL2_ES_3_2.2_4	APOL2	ES	3	2.2	4	1.8E-07	0.186	0.153	4.1E-02	9.0E-04	9.4E-03
KIRP	APP_ES_10_9_11	APP	ES	10	9	11	7.2E-13	0.302	-0.078	3.1E-01	1.6E-02	3.8E-02
KIRP	AQP7_ES_5_3.2_6	AQP7	ES	5	3.2	6	7.0E-05	0.133	0.132	7.9E-01	3.2E-01	4.1E-01
KIRP	AQP7_RI_8.2_8.1_8.3	AQP7	RI	8.2	8.1	8.3	2.0E-11	0.281	-0.424	2.0E-01	4.4E-01	8.4E-01
KIRP	AQP7_RI_8.2:8.3_8.1_8.4	AQP7	RI	8.2:8.3	8.1	8.4	8.9E-08	0.194	-0.285	6.0E-01	5.8E-01	8.0E-01
KIRP	ARFRP1_AA_3.2_1_3.3	ARFRP1	AA	3.2	1	3.3	5.9E-05	0.116	0.036	2.0E-01	3.5E-02	5.2E-03
KIRP	ARHGEF10_ES_10_9_11	ARHGEF10	ES	10	9	11	1.5E-06	0.178	0.055	6.4E-04	3.7E-04	3.3E-03
KIRP	ARHGEF10L_ES_18_17_19	ARHGEF10L	ES	18	17	19	6.6E-10	0.240	0.138	1.0E-01	2.1E-02	2.2E-01
KIRP	ARID1B_ES_12_11_13	ARID1B	ES	12	11	13	2.8E-06	0.167	-0.031	3.6E-02	1.1E-03	8.1E-02
KIRP	ARL6IP4_AD_1.2:1.3_1.1_2.1	ARL6IP4	AD	1.2:1.3	1.1	2.1	1.6E-04	0.117	0.000	4.4E-01	5.3E-01	6.7E-01
KIRP	ARL6IP4_AD_1.2:1.3:1.4_1.1_2.1	ARL6IP4	AD	1.2:1.3:1.4	1.1	2.1	5.0E-05	0.166	0.033	8.6E-02	8.1E-02	1.2E-01
KIRP	ARMC10_ES_2_1_5.1	ARMC10	ES	2	1	5.1	2.1E-04	0.100	0.008	9.3E-02	1.8E-01	3.5E-02
KIRP	ARMC5_RI_5.2_5.1_5.3	ARMC5	RI	5.2	5.1	5.3	1.8E-04	0.101	-0.281	6.2E-01	1.1E-01	1.1E-01
KIRP	ARMC6_AD_1.2_1.1_5	ARMC6	AD	1.2	1.1	5	9.7E-15	0.364	0.295	1.3E-01	9.7E-03	7.6E-04
KIRP	ARSA_RI_1.3:1.4_1.2_1.5	ARSA	RI	1.3:1.4	1.2	1.5	2.0E-04	0.101	-0.038	5.0E-01	2.3E-01	8.2E-01
KIRP	ARVCF_ES_20_19_21	ARVCF	ES	20	19	21	5.0E-10	0.259	0.326	2.0E-01	1.3E-02	1.3E-03
KIRP	ASAP2_ES_23_22_24	ASAP2	ES	23	22	24	4.4E-05	0.135	0.063	8.6E-02	2.5E-01	4.7E-01
KIRP	ASCC2_ES_6:7_5_8	ASCC2	ES	6:07	5	8	1.5E-04	0.112	0.306	5.2E-01	1.5E-01	6.4E-02
KIRP	ASPH_ES_12_11_13	ASPH	ES	12	11	13	1.8E-04	0.103	0.213	2.1E-01	9.6E-02	2.3E-02
KIRP	ASPHD1_RI_1.2_1.1_1.3	ASPHD1	RI	1.2	1.1	1.3	6.6E-05	0.114	-0.270	5.1E-02	5.0E-02	2.1E-01
KIRP	ATF4_RI_2.2_2.1_2.3	ATF4	RI	2.2	2.1	2.3	9.4E-06	0.136	0.154	4.4E-02	1.2E-02	4.4E-02
KIRP	ATG2A_AD_27.2_27.1_28	ATG2A	AD	27.2	27.1	28	2.7E-04	0.116	-0.089	1.2E-01	5.0E-02	5.3E-02
KIRP	ATP5H_ES_5_3_6	ATP5H	ES	5	3	6	2.1E-05	0.143	-0.169	9.9E-01	4.2E-01	4.6E-01
KIRP	ATP9B_ES_30_29_31.1	ATP9B	ES	30	29	31.1	1.1E-05	0.136	0.137	8.2E-02	3.4E-02	5.3E-01
KIRP	ATPIF1_RI_2.2:2.3_2.1_2.4	ATPIF1	RI	2.2:2.3	2.1	2.4	1.6E-11	0.273	-0.320	1.1E-01	7.8E-01	8.3E-01
KIRP	ATRAID_AD_1.2_1.1_2	ATRAID	AD	1.2	1.1	2	6.7E-05	0.113	-0.415	1.9E-01	3.4E-01	9.1E-01
KIRP	ATXN2L_RI_22.2:22.3:22.4:22.5_22.1_22	ATXN2L	RI	2.2:22.3:22.4:22.	22.1	22.6	1.3E-05	0.132	0.215	9.0E-01	6.8E-01	9.3E-01
KIRP	AXIN1_ES_9_8_10	AXIN1	ES	9	8	10	6.7E-12	0.281	0.120	3.5E-01	5.1E-01	5.7E-02
KIRP	AXL_ES_11_10_12	AXL	ES	11	10	12	1.5E-04	0.118	0.060	1.6E-01	3.2E-01	1.6E-02
KIRP	BAIAP2_ES_17.1_16.1_18.1	BAIAP2	ES	17.1	16.1	18.1	2.9E-05	0.124	0.061	3.6E-01	6.4E-01	5.7E-01
KIRP	BAIAP2_ES_17.1:17.2_16.1_18.1	BAIAP2	ES	17.1:17.2	16.1	18.1	9.6E-05	0.109	0.117	3.2E-01	2.7E-01	1.3E-01
KIRP	BANP_AA_14.1_13_14.2	BANP	AA	14.1	13	14.2	2.3E-04	0.102	-0.037	6.7E-01	9.9E-01	4.7E-02
KIRP	BAX_RI_6.2_6.1_6.3	BAX	RI	6.2	6.1	6.3	1.2E-10	0.255	-0.284	5.5E-01	2.7E-01	1.5E-02
KIRP	BCAT2_ES_3:4:5_1_6	BCAT2	ES	3:04:05	1	6	1.9E-05	0.128	-0.226	7.8E-01	5.7E-02	9.5E-02
KIRP	BCL2_RI_1.2_1.1_1.3	BCL2	RI	1.2	1.1	1.3	1.3E-05	0.134	0.364	5.9E-02	2.2E-01	6.8E-01
KIRP	BCL2L1_AA_2.2_1_2.3	BCL2L1	AA	2.2	1	2.3	5.9E-05	0.116	-0.304	9.8E-01	8.6E-01	5.0E-01
KIRP	BCS1L_AD_1.3:1.4:1.5:1.6:1.7:1.8_1.2_2	BCS1L	AD	3:1.4:1.5:1.6:1.7:1	1.2	2	2.2E-04	0.106	0.061	4.8E-01	1.5E-01	6.3E-01
KIRP	BCS1L_AD_1.5:1.6:1.7:1.8_1.4_2	BCS1L	AD	1.5:1.6:1.7:1.8	1.4	2	7.7E-05	0.113	0.119	4.3E-01	9.6E-01	9.4E-01
KIRP	BCS1L_RI_1.2:1.3_1.1_1.4	BCS1L	RI	1.2:1.3	1.1	1.4	7.5E-05	0.118	0.299	5.7E-01	3.4E-01	1.8E-01
KIRP	BIN1_ES_13_12_17	BIN1	ES	13	12	17	1.8E-13	0.314	-0.239	2.3E-02	3.4E-03	1.5E-02

cancer type	id	Gene Symbol	splice_type	Exon	From.Exon	To.Exon	anova.p	adj.r2	r	p.50	p.25	p.10
KIRP	BIN1_ES_13_12_18	BIN1	ES	13	12	18	2.1E-08	0.230	-0.142	4.0E-02	8.2E-03	1.2E-03
KIRP	BIN1_ES_13:16:17_12_18	BIN1	ES	13:16:17	12	18	7.9E-09	0.216	0.166	2.0E-01	3.8E-01	9.1E-01
KIRP	BIN1_ES_16:17_13_18	BIN1	ES	16:17	13	18	1.1E-10	0.288	0.225	5.7E-01	3.0E-01	8.8E-02
KIRP	BRD8_ES_22_21_23	BRD8	ES	22	21	23	3.0E-07	0.174	0.049	3.2E-01	4.4E-01	5.5E-01
KIRP	BRF1_AD_1.2_1.1_2	BRF1	AD	1.2	1.1	2	3.8E-07	0.172	0.107	1.9E-01	1.8E-01	6.5E-02
KIRP	BTBD3_RI_2.2_2.1_2.3	BTBD3	RI	2.2	2.1	2.3	1.1E-05	0.163	-0.048	2.5E-01	8.3E-01	9.7E-01
KIRP	BTN2A1_ES_4_3_5	BTN2A1	ES	4	3	5	4.4E-05	0.129	0.169	2.5E-01	1.4E-01	2.2E-01
KIRP	C11orf73_RI_5.2_5.1_5.3	C11orf73	RI	5.2	5.1	5.3	6.7E-06	0.140	0.081	6.7E-01	2.5E-01	7.3E-01
KIRP	C12orf76_ES_9.1_8_10.2	C12orf76	ES	9.1	8	10.2	2.4E-06	0.193	0.033	5.1E-01	2.2E-01	3.1E-02
KIRP	C14orf159_ES_4_1_7	C14orf159	ES	4	1	7	2.1E-04	0.109	0.070	3.3E-01	7.2E-01	4.5E-01
KIRP	C14orf159_ES_4:5.2_1_7	C14orf159	ES	04:05.2	1	7	9.7E-08	0.194	0.012	3.8E-01	1.1E-01	1.2E-01
KIRP	C14orf79_RI_2.3_2.2_2.4	C14orf79	RI	2.3	2.2	2.4	3.2E-06	0.148	0.189	4.3E-01	4.3E-01	8.5E-01
KIRP	C16orf13_ES_2_1_5	C16orf13	ES	2	1	5	1.8E-04	0.106	-0.058	1.5E-02	1.5E-02	2.8E-03
KIRP	C16orf13_ES_3_1_4	C16orf13	ES	3	1	4	4.2E-05	0.129	0.101	3.3E-04	3.8E-04	8.4E-03
KIRP	C16orf13_ES_3:4_1_5	C16orf13	ES	3:04	1	5	2.7E-05	0.130	-0.037	7.0E-02	1.0E-01	1.4E-01
KIRP	C16orf13_ES_4_1_5	C16orf13	ES	4	1	5	3.4E-11	0.314	0.021	4.3E-03	1.9E-03	4.1E-04
KIRP	C16orf59_RI_8.2_8.1_8.3	C16orf59	RI	8.2	8.1	8.3	2.1E-05	0.138	0.134	1.9E-03	1.9E-03	2.0E-01
KIRP	C16orf89_AA_9.1_8_9.2	C16orf89	AA	9.1	8	9.2	3.9E-12	0.319	-0.801	3.1E-03	4.6E-03	5.0E-03
KIRP	C16orf93_ES_4.1:4.3:5_3.3_7	C16orf93	ES	4.1:4.3:5	3.3	7	1.3E-05	0.148	0.259	7.6E-01	3.9E-01	1.7E-01
KIRP	C16orf93_ES_6_5_7	C16orf93	ES	6	5	7	2.3E-04	0.105	0.073	1.2E-01	3.4E-01	6.0E-01
KIRP	C18orf32_AD_1.2_1.1_3	C18orf32	AD	1.2	1.1	3	1.3E-05	0.133	-0.158	2.3E-01	8.4E-02	1.5E-01
KIRP	C1orf85_ES_5.1:5.2_4_6.1	C1orf85	ES	5.1:5.2	4	6.1	2.5E-09	0.249	-0.019	2.3E-02	1.5E-01	3.9E-02
KIRP	C8orf59_ES_2.1:2.2_1_3.2	C8orf59	ES	2.1:2.2	1	3.2	1.9E-06	0.154	0.176	1.7E-01	1.4E-03	4.9E-03
KIRP	C9orf117_RI_5.2_5.1_5.3	C9orf117	RI	5.2	5.1	5.3	1.7E-05	0.149	-0.470	7.1E-02	1.1E-01	1.3E-01
KIRP	C9orf117_RI_5.4_5.3_5.5	C9orf117	RI	5.4	5.3	5.5	4.8E-06	0.154	-0.434	5.0E-02	9.6E-02	1.2E-01
KIRP	C9orf89_AA_3.3:3.4_3.1_3.5	C9orf89	AA	3.3:3.4	3.1	3.5	5.0E-09	0.227	0.013	1.6E-01	5.2E-02	1.0E-01
KIRP	C9orf89_AD_3.2:3.3_3.1_3.5	C9orf89	AD	3.2:3.3	3.1	3.5	4.2E-09	0.229	-0.121	1.4E-01	5.1E-02	2.4E-01
KIRP	C9orf89_RI_3.2:3.3:3.4_3.1_3.5	C9orf89	RI	3.2:3.3:3.4	3.1	3.5	1.8E-04	0.108	-0.107	4.1E-01	4.8E-02	9.9E-01
KIRP	C9orf89_RI_3.4_3.3_3.5	C9orf89	RI	3.4	3.3	3.5	1.5E-04	0.104	-0.080	8.2E-01	8.2E-01	9.4E-01
KIRP	C9orf9_RI_5.2_5.1_5.3	C9orf9	RI	5.2	5.1	5.3	1.6E-05	0.130	0.041	2.0E-01	4.0E-01	5.8E-01
KIRP	CADM1_ES_10_9_12	CADM1	ES	10	9	12	1.1E-05	0.139	-0.251	8.6E-01	3.6E-02	6.8E-02
KIRP	CAMK2D_AA_14.1_13_14.2	CAMK2D	AA	14.1	13	14.2	8.1E-05	0.132	0.003	7.6E-01	6.4E-01	6.8E-01
KIRP	CAMK2G_ES_19.1:19.2_18_21	CAMK2G	ES	19.1:19.2	18	21	7.9E-06	0.143	-0.078	9.4E-01	8.0E-01	3.9E-01
KIRP	CANT1_RI_3.2_3.1_3.3	CANT1	RI	3.2	3.1	3.3	1.0E-04	0.111	-0.304	1.3E-02	4.9E-03	3.0E-01
KIRP	CARD8_RI_2.2_2.1_2.3	CARD8	RI	2.2	2.1	2.3	7.1E-04	0.107	-0.075	2.0E-01	2.8E-01	6.4E-01
KIRP	CARM1_ES_16.1_15_16.3	CARM1	ES	16.1	15	16.3	1.3E-06	0.158	-0.142	2.6E-01	4.5E-02	1.4E-01
KIRP	CARM1_RI_16.2_16.1_16.3	CARM1	RI	16.2	16.1	16.3	7.8E-05	0.114	0.021	4.7E-01	4.0E-01	3.2E-01
KIRP	CASP1_RI_10.2_10.1_10.3	CASP1	RI	10.2	10.1	10.3	3.6E-09	0.220	-0.063	1.0E-01	6.5E-02	8.1E-01
KIRP	CAV1_AD_1.2:1.3_1.1_2.3	CAV1	AD	1.2:1.3	1.1	2.3	2.4E-09	0.251	0.086	1.2E-01	9.0E-02	3.2E-02
KIRP	CBLB_ES_18.4_18.2_19	CBLB	ES	18.4	18.2	19	9.2E-05	0.114	-0.123	6.3E-01	6.5E-02	1.4E-01

cancer type	id	Gene Symbol	splice_type	Exon	From.Exon	To.Exon	anova.p	adj.r2	r	p.50	p.25	p.10
KIRP	CBY1_ES_2_1_3	CBY1	ES	2	1	3	1.1E-04	0.108	-0.332	1.2E-01	1.8E-01	4.5E-02
KIRP	CC2D2A_ES_5_4_6	CC2D2A	ES	5	4	6	1.2E-10	0.257	-0.434	2.7E-02	4.4E-02	7.8E-03
KIRP	CCDC106_AA_1.3:1.4_1.1_1.5	CCDC106	AA	1.3:1.4	1.1	1.5	1.2E-05	0.137	-0.006	2.8E-01	3.4E-01	2.3E-01
KIRP	CCDC148_ES_5_4_6	CCDC148	ES	5	4	6	1.1E-07	0.226	0.280	5.5E-01	3.2E-01	6.5E-02
KIRP	CCDC51_AA_3.1_2.2_3.2	CCDC51	AA	3.1	2.2	3.2	4.1E-06	0.146	0.337	3.8E-01	2.5E-01	3.3E-01
KIRP	CCDC74B_AD_1.2_1.1_3.1	CCDC74B	AD	1.2	1.1	3.1	2.9E-06	0.173	-0.339	6.7E-01	1.6E-01	4.6E-01
KIRP	CCDC84_AD_6.2_6.1_7	CCDC84	AD	6.2	6.1	7	9.4E-06	0.141	0.445	1.4E-01	1.9E-01	4.1E-01
KIRP	CCDC84_ES_4_3_5	CCDC84	ES	4	3	5	8.8E-05	0.119	0.441	1.6E-01	1.2E-01	4.7E-01
KIRP	CCDC88A_ES_26_25_27	CCDC88A	ES	26	25	27	7.8E-05	0.137	-0.054	1.4E-01	6.3E-01	2.6E-01
KIRP	CCDC90B_AD_1.3_1.2_2	CCDC90B	AD	1.3	1.2	2	3.9E-06	0.146	-0.011	1.6E-02	1.8E-02	2.4E-02
KIRP	CCDC91_ES_4:5:6_3_8.2	CCDC91	ES	4:05:06	3	8.2	1.1E-04	0.129	-0.017	7.9E-03	1.4E-03	2.1E-03
KIRP	CCNDBP1_ES_10.1_9_10.3	CCNDBP1	ES	10.1	9	10.3	2.2E-08	0.202	-0.066	1.0E-01	2.5E-01	3.4E-01
KIRP	CD40_ES_6_5_7	CD40	ES	6	5	7	3.7E-06	0.147	-0.220	2.1E-02	1.1E-01	4.0E-03
KIRP	CD44_ES_12.1:13_5_14	CD44	ES	12.1:13	5	14	3.7E-07	0.200	-0.050	1.4E-01	2.4E-01	3.3E-02
KIRP	CD44_ES_3.1:3.2:4:5:12.1:13:14:15:16.1	CD44	ES	12.1:13:14:15:16.	2.1	17.2	1.1E-04	0.120	-0.076	2.4E-01	7.5E-01	8.1E-01
KIRP	CD46_ES_7:8:9_6_10	CD46	ES	7:08:09	6	10	1.1E-04	0.138	-0.166	1.8E-02	1.7E-02	7.5E-02
KIRP	CD47_ES_8:9:10_7_11	CD47	ES	8:09:10	7	11	2.9E-07	0.192	-0.085	8.8E-02	6.7E-02	1.9E-02
KIRP	CD47_ES_9:10_8_11	CD47	ES	9:10	8	11	7.8E-05	0.112	0.136	5.9E-03	1.1E-02	2.5E-03
KIRP	CDC42SE1_ES_5_4_6	CDC42SE1	ES	5	4	6	6.1E-06	0.144	0.022	9.1E-02	1.1E-01	1.5E-01
KIRP	CDH16_RI_14.2:14.3_14.1_14.4	CDH16	RI	14.2:14.3	14.1	14.4	1.2E-06	0.159	-0.281	5.3E-01	3.4E-01	1.0E+00
KIRP	CDH16_RI_14.2:14.3:14.4_14.1_14.5	CDH16	RI	14.2:14.3:14.4	14.1	14.5	3.3E-05	0.122	-0.308	6.1E-01	8.7E-01	3.8E-01
KIRP	CDH24_ES_9_8_10	CDH24	ES	9	8	10	1.9E-11	0.301	0.054	1.9E-01	6.8E-02	2.9E-01
KIRP	CDK10_ES_5_2.2_6	CDK10	ES	5	2.2	6	1.1E-08	0.212	-0.143	7.3E-01	6.2E-01	9.1E-01
KIRP	CDK18_AD_1.2_1.1_2.3	CDK18	AD	1.2	1.1	2.3	5.2E-05	0.133	0.018	1.8E-01	2.8E-01	8.8E-02
KIRP	CDK20_ES_5_4_6	CDK20	ES	5	4	6	1.6E-04	0.113	0.039	5.3E-01	4.8E-01	8.8E-01
KIRP	CDKL1_AA_9.1_8_9.2	CDKL1	AA	9.1	8	9.2	1.3E-05	0.145	-0.608	6.5E-01	8.0E-01	2.2E-01
KIRP	CDKL1_ES_9.1:9.2_8_10	CDKL1	ES	9.1:9.2	8	10	4.6E-04	0.118	-0.277	5.4E-01	4.9E-01	6.9E-01
KIRP	CDKL2_ES_11:12_10_13	CDKL2	ES	11:12	10	13	2.3E-04	0.104	0.005	7.0E-02	2.1E-01	7.7E-02
KIRP	CEACAM1_ES_9_8_10	CEACAM1	ES	9	8	10	9.5E-10	0.270	-0.272	2.1E-01	2.8E-01	7.3E-02
KIRP	CEP57_AD_10.2:10.3_10.1_11	CEP57	AD	10.2:10.3	10.1	11	9.4E-07	0.164	0.189	8.6E-01	7.8E-01	3.7E-01
KIRP	CEP57_RI_10.2_10.1_10.3	CEP57	RI	10.2	10.1	10.3	1.9E-06	0.154	0.014	7.0E-02	1.9E-02	7.6E-01
KIRP	CES2_AD_1.2:1.3_1.1_2	CES2	AD	1.2:1.3	1.1	2	1.3E-06	0.159	-0.599	5.7E-01	1.3E-01	1.8E-01
KIRP	CIRBP_ES_9.5:9.6_9.3_9.8	CIRBP	ES	9.5:9.6	9.3	9.8	1.0E-04	0.109	-0.055	3.1E-01	1.8E-01	9.5E-02
KIRP	CIRBP_RI_9.4_9.3_9.5	CIRBP	RI	9.4	9.3	9.5	1.9E-05	0.128	0.302	7.2E-01	9.1E-02	3.3E-01
KIRP	CLASP1_ES_22_21_24	CLASP1	ES	22	21	24	3.8E-04	0.120	0.014	1.1E-01	3.6E-01	9.0E-01
KIRP	CLEC16A_AD_11.2_11.1_12	CLEC16A	AD	11.2	11.1	12	2.2E-04	0.120	0.075	2.3E-01	2.2E-01	6.7E-02
KIRP	CLEC16A_ES_24_23_25	CLEC16A	ES	24	23	25	2.9E-04	0.105	-0.216	2.9E-02	8.6E-03	1.1E-01
KIRP	CLEC1A_ES_2_1_3	CLEC1A	ES	2	1	3	1.5E-07	0.213	-0.552	1.9E-01	4.8E-03	7.5E-02
KIRP	CLIP1_ES_10_8_11.1	CLIP1	ES	10	8	11.1	1.1E-06	0.190	-0.012	8.0E-01	9.8E-01	5.0E-01
KIRP	CLN3_RI_3.3_3.2_3.4	CLN3	RI	3.3	3.2	3.4	5.3E-07	0.168	-0.068	2.0E-01	1.1E-01	3.6E-02

cancer type	id	Gene Symbol	splice_type	Exon	From.Exon	To.Exon	anova.p	adj.r2	r	p.50	p.25	p.10
KIRP	CLU_RI_3.2_3.1_3.3	CLU	RI	3.2	3.1	3.3	4.2E-05	0.131	0.069	8.6E-01	3.8E-01	2.6E-01
KIRP	CLYBL_RI_9.2_9.1_9.3	CLYBL	RI	9.2	9.1	9.3	4.4E-08	0.195	-0.038	8.0E-02	2.2E-02	1.3E-02
KIRP	COA6_RI_2.2_2.1_2.3	COA6	RI	2.2	2.1	2.3	1.9E-05	0.128	-0.318	9.6E-02	3.0E-01	8.9E-01
KIRP	COASY_AD_1.2:1.3_1.1_1.5	COASY	AD	1.2:1.3	1.1	1.5	9.9E-11	0.271	0.000	2.7E-02	8.7E-02	1.2E-01
KIRP	COBL_ES_6_5_7	COBL	ES	6	5	7	5.2E-04	0.115	-0.142	7.6E-01	6.4E-01	6.5E-01
KIRP	COL4A3BP_ES_12_11_13	COL4A3BP	ES	12	11	13	1.2E-04	0.130	0.086	5.4E-02	2.6E-01	1.1E-01
KIRP	COPE_ES_4:5_3_6	COPE	ES	4:05	3	6	3.7E-09	0.228	-0.073	2.9E-02	1.8E-02	9.4E-03
KIRP	COX20_ES_3_1_4	COX20	ES	3	1	4	4.9E-08	0.194	-0.308	1.9E-01	5.5E-03	1.9E-03
KIRP	COX4I1_AA_5.1:5.2:5.3_4.1_5.4	COX4I1	AA	5.1:5.2:5.3	4.1	5.4	1.9E-09	0.227	-0.212	1.9E-01	1.1E-02	9.0E-03
KIRP	COX4I1_ES_4.2:5.1_4.1_5.4	COX4I1	ES	4.2:5.1	4.1	5.4	4.6E-05	0.118	-0.383	4.7E-01	3.5E-01	2.2E-02
KIRP	COX4I1_RI_5.2:5.3:5.4_5.1_5.5	COX4I1	RI	5.2:5.3:5.4	5.1	5.5	1.2E-07	0.184	-0.277	5.0E-01	6.0E-01	7.9E-01
KIRP	COX7C_RI_2.2:2.3_2.1_2.4	COX7C	RI	2.2:2.3	2.1	2.4	8.9E-07	0.162	-0.294	5.4E-01	9.1E-01	9.5E-01
KIRP	CPEB2_ES_6_5_7	CPEB2	ES	6	5	7	2.0E-10	0.306	-0.225	5.1E-03	1.6E-02	9.2E-03
KIRP	CREBZF_RI_2.4_2.3_2.5	CREBZF	RI	2.4	2.3	2.5	1.2E-04	0.107	-0.414	6.5E-01	1.2E-01	1.1E-01
KIRP	CRIP1_RI_1.2:1.3_1.1_1.4	CRIP1	RI	1.2:1.3	1.1	1.4	9.5E-04	0.101	0.072	7.4E-01	6.2E-01	5.0E-01
KIRP	CSF1_AA_6.1:6.2_5_6.3	CSF1	AA	6.1:6.2	5	6.3	1.4E-04	0.104	0.161	4.8E-01	2.1E-01	2.2E-01
KIRP	CTNND1_ES_21:22.1_20_22.2	CTNND1	ES	21:22.1	20	22.2	4.1E-08	0.212	-0.079	5.7E-01	3.1E-01	3.3E-01
KIRP	CTNND1_ES_3_2.1_4.1	CTNND1	ES	3	2.1	4.1	1.1E-05	0.179	-0.236	7.7E-01	4.6E-01	6.8E-01
KIRP	CTNND1_ES_3:4.1:4.2:4.3_2.1_5	CTNND1	ES	3:4.1:4.2:4.3	2.1	5	8.6E-06	0.158	-0.316	4.5E-01	4.8E-01	6.9E-01
KIRP	CTNND1_ES_3:4.2:4.3_2.1_5	CTNND1	ES	3:4.2:4.3	2.1	5	1.2E-06	0.199	-0.223	4.2E-01	2.7E-01	4.1E-01
KIRP	D2HGDH_AD_7.2:7.3_7.1_8	D2HGDH	AD	7.2:7.3	7.1	8	2.8E-06	0.184	0.244	6.5E-01	8.3E-01	3.1E-01
KIRP	DAB2_ES_10_9_11	DAB2	ES	10	9	11	8.8E-08	0.187	-0.515	6.9E-02	2.8E-01	4.0E-01
KIRP	DBNDD2_AD_1.2:1.3:1.4_1.1_3.3	DBNDD2	AD	1.2:1.3:1.4	1.1	3.3	5.4E-07	0.168	-0.074	2.2E-01	3.6E-01	1.1E-01
KIRP	DCAF6_ES_13.1_10_14	DCAF6	ES	13.1	10	14	7.0E-08	0.200	0.050	4.2E-02	1.3E-01	4.2E-01
KIRP	DCTN2_ES_8_2_10	DCTN2	ES	8	2	10	3.6E-07	0.172	0.090	2.0E-01	7.9E-01	4.0E-01
KIRP	DCTN3_RI_3.6_3.5_3.7	DCTN3	RI	3.6	3.5	3.7	1.1E-04	0.129	-0.240	3.1E-01	6.0E-01	5.1E-01
KIRP	DDIT3_AA_2.3:2.4_2.1_2.5	DDIT3	AA	2.3:2.4	2.1	2.5	7.3E-04	0.100	0.019	1.5E-02	1.4E-02	1.9E-02
KIRP	DDX55_AA_7.1_6_7.2	DDX55	AA	7.1	6	7.2	5.2E-05	0.118	-0.337	2.2E-01	5.8E-01	6.0E-01
KIRP	DECR1_ES_2:3:4.1:4.2_1_5.2	DECR1	ES	2:3:4.1:4.2	1	5.2	6.1E-06	0.143	-0.275	1.3E-01	1.7E-01	1.8E-01
KIRP	DHRS12_RI_10.2_10.1_10.3	DHRS12	RI	10.2	10.1	10.3	2.8E-06	0.150	-0.421	7.3E-01	4.0E-01	5.1E-01
KIRP	DIAPH1_ES_2_1_3	DIAPH1	ES	2	1	3	1.5E-08	0.262	-0.033	4.7E-01	6.4E-01	9.8E-01
KIRP	DLG1_ES_9_7_10	DLG1	ES	9	7	10	2.1E-04	0.113	-0.073	4.1E-01	7.5E-02	3.5E-01
KIRP	DMAP1_RI_1.2_1.1_1.3	DMAP1	RI	1.2	1.1	1.3	2.5E-08	0.203	-0.036	1.8E-01	2.5E-01	3.8E-02
KIRP	DMKN_ES_11_9_12	DMKN	ES	11	9	12	5.5E-06	0.182	0.038	2.6E-02	2.0E-04	1.8E-03
KIRP	DMTN_ES_6_5.2_7	DMTN	ES	6	5.2	7	8.7E-10	0.236	-0.095	5.7E-02	3.4E-02	2.6E-02
KIRP	DMWD_ES_4_3_5	DMWD	ES	4	3	5	9.0E-07	0.162	-0.077	2.5E-01	1.7E-01	4.8E-01
KIRP	DNAJC25_ES_2.2_1_3.2	DNAJC25	ES	2.2	1	3.2	4.7E-05	0.133	-0.043	5.9E-02	8.6E-03	4.6E-02
KIRP	DNASE1_ES_6.1_5_7	DNASE1	ES	6.1	5	7	6.5E-04	0.117	-0.005	3.2E-01	3.5E-01	3.4E-01
KIRP	DNM1L_ES_18_16_19	DNM1L	ES	18	16	19	1.2E-05	0.136	-0.068	2.7E-01	1.3E-01	2.8E-01
KIRP	DNMT3B_ES_22:23_21_24	DNMT3B	ES	22:23	21	24	3.5E-04	0.115	-0.174	5.0E-03	2.7E-03	4.4E-02

cancer type	id	Gene Symbol	splice_type	Exon	From.Exon	To.Exon	anova.p	adj.r2	r	p.50	p.25	p.10
KIRP	DTD2_AD_1.2_1.1_2	DTD2	AD	1.2	1.1	2	1.6E-05	0.131	0.272	2.0E-01	7.1E-03	2.8E-01
KIRP	DTX3_AA_1.3:1.4_1.1_1.5	DTX3	AA	1.3:1.4	1.1	1.5	2.8E-04	0.108	0.489	7.7E-01	3.7E-01	7.9E-01
KIRP	DTX3_ES_1.3_1.1_1.5	DTX3	ES	1.3	1.1	1.5	1.2E-05	0.139	0.223	1.3E-02	1.0E-03	7.1E-02
KIRP	DUS4L_RI_1.3:1.4_1.2_1.5	DUS4L	RI	1.3:1.4	1.2	1.5	3.5E-04	0.124	-0.110	6.3E-01	9.1E-01	2.3E-02
KIRP	DUSP15_RI_2.2:2.3_2.1_2.4	DUSP15	RI	2.2:2.3	2.1	2.4	7.0E-06	0.158	-0.066	4.5E-01	9.1E-01	4.5E-01
KIRP	DUSP28_RI_2.2_2.1_2.3	DUSP28	RI	2.2	2.1	2.3	4.3E-05	0.119	0.346	6.4E-01	6.0E-01	8.9E-02
KIRP	DVL1_AD_11.2_11.1_12	DVL1	AD	11.2	11.1	12	3.1E-08	0.199	0.265	4.8E-01	2.0E-01	1.4E-02
KIRP	ECHDC2_ES_9.1_8.1_10	ECHDC2	ES	9.1	8.1	10	1.7E-04	0.103	0.097	9.9E-01	6.4E-01	1.8E-01
KIRP	ECHDC2_ES_9.1:10_8.1_11	ECHDC2	ES	9.1:10	8.1	11	3.7E-07	0.190	0.172	3.2E-01	8.5E-01	1.5E-01
KIRP	ECI2_AA_2.1_1.1_2.2	ECI2	AA	2.1	1.1	2.2	9.4E-06	0.139	0.139	2.6E-01	2.7E-02	2.0E-01
KIRP	EDEM2_ES_12_11_13	EDEM2	ES	12	11	13	1.8E-04	0.102	-0.176	2.5E-01	1.1E-01	5.6E-02
KIRP	EDF1_RI_4.2_4.1_4.3	EDF1	RI	4.2	4.1	4.3	2.8E-05	0.123	0.049	9.5E-01	9.1E-01	7.3E-01
KIRP	EEF1B2_AA_1.3_1.1_1.4	EEF1B2	AA	1.3	1.1	1.4	2.2E-10	0.250	0.457	2.6E-02	6.7E-03	2.7E-02
KIRP	EIF3K_AD_1.2_1.1_2	EIF3K	AD	1.2	1.1	2	6.2E-15	0.382	0.150	5.2E-01	2.8E-01	2.1E-01
KIRP	ELK1_ES_2_1_3	ELK1	ES	2	1	3	8.8E-08	0.205	0.249	4.2E-01	3.0E-01	4.5E-01
KIRP	ELMOD3_AA_2.1:2.2_1_2.3	ELMOD3	AA	2.1:2.2	1	2.3	6.7E-04	0.117	0.305	7.2E-01	5.0E-01	6.6E-01
KIRP	ELMOD3_ES_9_8_10	ELMOD3	ES	9	8	10	1.2E-04	0.109	0.321	5.6E-01	1.5E-02	4.7E-02
KIRP	ELMOD3_RI_2.2_2.1_2.3	ELMOD3	RI	2.2	2.1	2.3	1.0E-04	0.138	0.358	9.0E-01	3.5E-01	6.3E-01
KIRP	ELOVL6_RI_2.2_2.1_2.3	ELOVL6	RI	2.2	2.1	2.3	5.1E-06	0.168	0.194	1.1E-01	5.5E-01	4.6E-01
KIRP	ELP5_RI_7.4_7.3_7.5	ELP5	RI	7.4	7.3	7.5	1.6E-08	0.205	-0.022	2.7E-04	1.5E-04	4.5E-03
KIRP	EMC10_ES_7_6_8.1	EMC10	ES	7	6	8.1	1.9E-10	0.251	0.426	1.0E-01	2.6E-03	9.3E-04
KIRP	EML4_ES_7_6_8	EML4	ES	7	6	8	1.3E-04	0.125	-0.106	1.5E-03	3.4E-04	2.2E-02
KIRP	ENAH_ES_13_12_14	ENAH	ES	13	12	14	6.1E-10	0.254	-0.250	7.3E-03	3.9E-04	1.9E-02
KIRP	ENTPD4_AD_8.2_8.1_9	ENTPD4	AD	8.2	8.1	9	6.8E-06	0.149	-0.058	2.6E-01	1.1E-01	2.9E-01
KIRP	ENTPD6_ES_2:3.1:3.2_1_4	ENTPD6	ES	2:3.1:3.2	1	4	5.2E-04	0.110	0.099	9.8E-01	5.9E-01	2.3E-01
KIRP	ENTPD6_ES_3.1:3.2_1_4	ENTPD6	ES	3.1:3.2	1	4	1.9E-04	0.116	0.130	3.3E-01	1.1E-01	2.7E-04
KIRP	ENTPD8_ES_8_7_9	ENTPD8	ES	8	7	9	2.8E-05	0.156	-0.260	2.3E-01	7.7E-01	8.7E-01
KIRP	EPB41_ES_18_15_19.1	EPB41	ES	18	15	19.1	5.6E-04	0.113	-0.209	7.8E-02	5.6E-01	2.6E-01
KIRP	EPB41L1_ES_20_19_21	EPB41L1	ES	20	19	21	7.7E-08	0.190	-0.252	3.8E-01	7.0E-01	1.4E-01
KIRP	EPB41L1_ES_7_3_8	EPB41L1	ES	7	3	8	1.3E-04	0.117	-0.057	3.4E-01	2.2E-01	9.4E-01
KIRP	EPB41L2_ES_17:18_14_20.1	EPB41L2	ES	17:18	14	20.1	2.4E-07	0.188	-0.115	3.2E-02	2.6E-02	4.2E-03
KIRP	EPB41L3_ES_25_24_26	EPB41L3	ES	25	24	26	4.3E-05	0.119	-0.591	4.1E-02	8.0E-03	6.9E-02
KIRP	EPOR_AA_7.1_6_7.2	EPOR	AA	7.1	6	7.2	7.5E-06	0.187	-0.080	1.9E-03	1.0E-02	9.3E-03
KIRP	EPOR_ES_4.1:4.2_3_5	EPOR	ES	4.1:4.2	3	5	1.6E-09	0.280	0.174	1.2E-02	1.4E-02	2.9E-02
KIRP	EPS15L1_ES_22_21_23.1	EPS15L1	ES	22	21	23.1	2.0E-04	0.100	0.115	6.2E-01	4.7E-01	8.4E-01
KIRP	ERGIC3_ME_10|11_8_12	ERGIC3	ME	10|11	8	12	3.7E-05	0.139	0.102	5.1E-01	6.9E-01	3.4E-02
KIRP	ERLIN2_RI_7.2_7.1_7.3	ERLIN2	RI	7.2	7.1	7.3	5.2E-05	0.123	-0.204	2.3E-01	7.3E-01	8.8E-01
KIRP	EVA1C_ES_2:3_1_4	EVA1C	ES	2:03	1	4	3.4E-07	0.178	0.055	1.7E-01	5.8E-01	4.3E-01
KIRP	EVC_ES_23_22.1_24	EVC	ES	23	22.1	24	5.1E-05	0.118	-0.257	3.6E-02	1.1E-03	2.2E-02
KIRP	EVC_ES_23_22.1_25	EVC	ES	23	22.1	25	2.4E-09	0.228	-0.399	1.2E-02	3.0E-02	2.8E-02

cancer type	id	Gene Symbol	splice_type	Exon	From.Exon	To.Exon	anova.p	adj.r2	r	p.50	p.25	p.10
KIRP	EVI5L_ES_12_11_13	EVI5L	ES	12	11	13	8.8E-05	0.116	-0.097	2.8E-01	1.5E-01	2.4E-01
KIRP	EXOC7_ES_7_6_8.2	EXOC7	ES	7	6	8.2	5.8E-05	0.129	-0.007	1.5E-01	2.4E-01	4.3E-01
KIRP	EXOC7_ES_7:8.2_6_9	EXOC7	ES	07:08.2	6	9	3.9E-06	0.148	-0.246	8.4E-01	6.4E-01	8.8E-01
KIRP	EXOSC9_RI_10.2_10.1_10.3	EXOSC9	RI	10.2	10.1	10.3	2.5E-05	0.125	-0.071	1.9E-01	7.3E-01	7.6E-01
KIRP	EXOSC9_RI_10.4_10.3_10.5	EXOSC9	RI	10.4	10.3	10.5	1.4E-04	0.116	0.056	8.2E-01	4.0E-01	5.3E-01
KIRP	FADS3_RI_9.2_9.1_9.3	FADS3	RI	9.2	9.1	9.3	8.8E-06	0.137	0.225	6.3E-01	3.0E-01	2.3E-01
KIRP	FAH_RI_1.2:1.3_1.1_1.4	FAH	RI	1.2:1.3	1.1	1.4	4.1E-05	0.123	0.234	2.1E-01	2.4E-02	1.1E-01
KIRP	FAM122B_ES_3_2_4	FAM122B	ES	3	2	4	3.7E-05	0.132	0.111	2.4E-01	1.6E-02	5.9E-03
KIRP	FAM131A_ES_4_2_6.2	FAM131A	ES	4	2	6.2	1.5E-06	0.171	-0.205	7.1E-01	6.0E-01	3.2E-01
KIRP	FAM13A_AA_16.1_15_16.2	FAM13A	AA	16.1	15	16.2	7.9E-14	0.353	0.401	7.7E-03	4.6E-02	1.3E-02
KIRP	FAM156B_RI_2.3:2.4_2.2_2.5	FAM156B	RI	2.3:2.4	2.2	2.5	1.5E-05	0.131	0.327	9.7E-02	2.8E-03	7.8E-02
KIRP	FAM193B_ES_7_6_8	FAM193B	ES	7	6	8	3.5E-06	0.148	0.395	3.9E-01	4.4E-02	2.0E-01
KIRP	FAM193B_ES_8_6_9	FAM193B	ES	8	6	9	1.6E-08	0.215	-0.280	5.1E-02	2.0E-02	2.2E-02
KIRP	FAM195A_ES_3_2_4	FAM195A	ES	3	2	4	2.4E-06	0.152	-0.170	7.5E-02	6.2E-02	9.4E-03
KIRP	FAM195B_RI_2.3_2.2_2.4	FAM195B	RI	2.3	2.2	2.4	8.3E-09	0.212	-0.082	7.3E-01	1.2E-01	1.1E-01
KIRP	FAM3A_ES_7.1:7.2_6_8	FAM3A	ES	7.1:7.2	6	8	4.7E-10	0.241	-0.020	3.3E-01	3.7E-01	2.7E-01
KIRP	FAM63A_AA_4.1_2.2_4.2	FAM63A	AA	4.1	2.2	4.2	2.0E-07	0.202	-0.107	2.3E-01	1.3E-01	1.7E-02
KIRP	FAM71E1_AA_3.1_2_3.2	FAM71E1	AA	3.1	2	3.2	1.6E-04	0.113	-0.030	6.5E-01	7.6E-01	5.2E-01
KIRP	FAM73B_AA_15.1_14_15.2	FAM73B	AA	15.1	14	15.2	2.7E-06	0.157	0.153	5.0E-02	1.7E-01	1.2E-01
KIRP	FASTKD3_AA_5.1_4_5.2	FASTKD3	AA	5.1	4	5.2	1.0E-04	0.109	-0.160	4.3E-01	7.6E-01	7.4E-01
KIRP	FAU_AA_2.4:2.5_2.2_2.6	FAU	AA	2.4:2.5	2.2	2.6	1.4E-04	0.109	-0.015	2.5E-02	7.5E-01	6.5E-01
KIRP	FAU_AD_2.3:2.4_2.2_2.6	FAU	AD	2.3:2.4	2.2	2.6	8.1E-06	0.146	-0.184	7.7E-02	3.8E-01	7.7E-01
KIRP	FBXL8_RI_2.2:2.3_2.1_2.4	FBXL8	RI	2.2:2.3	2.1	2.4	2.9E-07	0.175	-0.066	7.4E-01	1.3E-01	2.2E-01
KIRP	FBXL8_RI_2.6_2.5_2.7	FBXL8	RI	2.6	2.5	2.7	2.0E-04	0.101	-0.030	7.9E-02	1.6E-01	1.1E-01
KIRP	FBXO44_ES_5.2:6_5.1_7	FBXO44	ES	5.2:6	5.1	7	3.3E-05	0.122	0.111	7.6E-01	9.0E-01	4.6E-01
KIRP	FCHSD1_ES_19_18_20	FCHSD1	ES	19	18	20	1.8E-07	0.184	0.155	7.7E-01	3.0E-01	4.9E-01
KIRP	FDPS_ES_2_1.1_3.1	FDPS	ES	2	1.1	3.1	7.5E-05	0.114	-0.047	6.4E-02	1.4E-02	2.5E-01
KIRP	FGFR1OP_ES_7_6_8	FGFR1OP	ES	7	6	8	3.9E-06	0.152	-0.135	1.2E-01	7.7E-02	2.1E-01
KIRP	FGFR1OP2_ES_5.1_4_6	FGFR1OP2	ES	5.1	4	6	1.3E-07	0.200	0.048	5.2E-01	1.9E-01	1.8E-01
KIRP	FGFR3_ME_8|9_7_10	FGFR3	ME	8|9	7	10	2.5E-08	0.209	0.144	8.8E-01	2.9E-01	1.6E-01
KIRP	FHL1_ES_8:9_7_10	FHL1	ES	8:09	7	10	4.6E-13	0.314	0.508	2.1E-01	2.6E-01	3.6E-01
KIRP	FIP1L1_ES_14_13.1_15	FIP1L1	ES	14	13.1	15	1.1E-04	0.108	0.097	6.6E-04	1.1E-02	1.9E-02
KIRP	FLAD1_RI_4.2_4.1_4.3	FLAD1	RI	4.2	4.1	4.3	2.0E-04	0.101	0.021	4.5E-01	5.0E-01	9.7E-01
KIRP	FLNB_ES_27_26_28	FLNB	ES	27	26	28	7.1E-07	0.176	-0.035	4.6E-01	4.1E-01	6.8E-02
KIRP	FLNB_ES_32.1_31_33	FLNB	ES	32.1	31	33	6.8E-04	0.101	0.257	7.5E-01	7.7E-01	8.5E-01
KIRP	FMNL3_ES_26_25_27	FMNL3	ES	26	25	27	7.8E-05	0.116	-0.065	8.6E-03	5.5E-03	3.7E-05
KIRP	FNTA_AD_7.2_7.1_8	FNTA	AD	7.2	7.1	8	1.5E-04	0.104	0.254	3.0E-01	5.2E-01	9.0E-01
KIRP	FUT6_ES_2:3_1.3_4.1	FUT6	ES	2:03	1.3	4.1	8.4E-07	0.180	-0.081	8.9E-01	1.7E-01	1.1E-02
KIRP	FUZ_AD_2.3:2.4_2.2_3	FUZ	AD	2.3:2.4	2.2	3	3.0E-04	0.101	-0.010	2.1E-01	6.3E-02	1.0E-01
KIRP	FUZ_AD_2.4_2.3_3	FUZ	AD	2.4	2.3	3	6.6E-07	0.167	0.029	3.2E-01	4.8E-01	3.0E-01

cancer type	id	Gene Symbol	splice_type	Exon	From.Exon	To.Exon	anova.p	adj.r2	r	p.50	p.25	p.10
KIRP	FXR1_ES_2:5:6:7:8:9:10:11:12:13:14:15_	FXR1	ES	7:8:9:10:11:12:13	1	16	1.3E-05	0.150	0.005	5.3E-01	1.7E-01	2.2E-02
KIRP	GABARAPL1_ES_2.7:2.8:2.9:2.10:2.11:2.	GABARAPL1	ES	.8:2.9:2.10:2.11:2	2.6	4.1	7.2E-06	0.151	-0.022	3.3E-01	7.4E-02	2.8E-02
KIRP	GCAT_AA_2.1_1_2.2	GCAT	AA	2.1	1	2.2	4.9E-08	0.204	-0.051	8.4E-01	4.6E-01	4.5E-01
KIRP	GK_ES_23_22_24	GK	ES	23	22	24	4.2E-08	0.195	-0.460	3.8E-01	5.7E-02	4.2E-02
KIRP	GLI4_RI_3.3_3.2_3.4	GLI4	RI	3.3	3.2	3.4	2.5E-06	0.154	0.060	6.3E-01	7.2E-01	6.1E-02
KIRP	GLUL_RI_1.2:1.3_1.1_1.4	GLUL	RI	1.2:1.3	1.1	1.4	4.7E-04	0.115	-0.233	2.7E-01	3.2E-01	6.9E-02
KIRP	GLYCTK_ES_3_2_4	GLYCTK	ES	3	2	4	3.2E-05	0.122	0.547	1.3E-01	6.3E-01	1.8E-01
KIRP	GMPR2_RI_2.2_2.1_2.3	GMPR2	RI	2.2	2.1	2.3	7.2E-06	0.140	-0.038	4.3E-01	7.9E-01	9.2E-01
KIRP	GNB2L1_ES_7.2:8.1:8.2_7.1_9	GNB2L1	ES	7.2:8.1:8.2	7.1	9	7.3E-05	0.112	-0.315	9.5E-01	5.6E-01	5.7E-01
KIRP	GNPDA1_RI_2.2_2.1_2.3	GNPDA1	RI	2.2	2.1	2.3	6.5E-05	0.137	-0.487	7.7E-01	2.8E-01	4.6E-01
KIRP	GOLGA2_ES_7_6_8	GOLGA2	ES	7	6	8	1.2E-11	0.300	0.046	1.6E-01	1.0E-02	1.2E-02
KIRP	GPBP1_ES_9_8_10	GPBP1	ES	9	8	10	1.7E-04	0.103	0.119	5.0E-02	1.1E-01	5.5E-02
KIRP	GPR126_ES_25_24_26	GPR126	ES	25	24	26	2.8E-07	0.198	0.245	4.3E-01	5.4E-01	1.0E+00
KIRP	GPS1_RI_1.2_1.1_1.3	GPS1	RI	1.2	1.1	1.3	2.4E-04	0.106	0.360	7.8E-01	1.5E-01	2.2E-01
KIRP	GPS1_RI_1.2:1.3:1.4_1.1_1.5	GPS1	RI	1.2:1.3:1.4	1.1	1.5	4.7E-05	0.119	0.314	7.1E-02	4.3E-03	8.8E-02
KIRP	GSTT1_ES_2:3_1_4.1	GSTT1	ES	2:03	1	4.1	6.9E-07	0.217	-0.553	5.2E-02	1.7E-01	2.6E-02
KIRP	GSTT1_ES_2:3:4.1_1_4.2	GSTT1	ES	03:04.1	1	4.2	5.3E-04	0.117	-0.408	2.5E-01	5.0E-03	2.2E-02
KIRP	GUSB_ES_4.1_2_5.1	GUSB	ES	4.1	2	5.1	5.4E-05	0.127	-0.059	4.6E-01	9.0E-01	9.9E-02
KIRP	HARS2_AD_6.2:6.3_6.1_7	HARS2	AD	6.2:6.3	6.1	7	4.5E-05	0.120	-0.165	3.1E-01	4.9E-01	8.2E-01
KIRP	HES4_RI_1.2_1.1_1.3	HES4	RI	1.2	1.1	1.3	1.4E-06	0.160	0.197	4.8E-02	2.4E-01	3.0E-01
KIRP	HM13_ES_11:12.1_10_13	HM13	ES	11:12.1	10	13	2.1E-07	0.187	0.297	7.6E-01	2.1E-01	2.3E-01
KIRP	HMGA1_AD_1.2:1.3_1.1_3.1	HMGA1	AD	1.2:1.3	1.1	3.1	8.9E-05	0.129	-0.140	5.0E-02	7.8E-01	8.7E-02
KIRP	HNRNPA2B1_AA_12.2:12.3_11_12.4	HNRNPA2B1	AA	12.2:12.3	11	12.4	5.9E-06	0.142	0.367	3.3E-01	2.5E-01	6.6E-01
KIRP	HOXA9_RI_1.2_1.1_1.3	HOXA9	RI	1.2	1.1	1.3	1.8E-05	0.141	0.255	2.4E-02	6.7E-03	6.1E-04
KIRP	HSBP1L1_ES_2.2:3.1_2.1_3.2	HSBP1L1	ES	2.2:3.1	2.1	3.2	4.0E-06	0.146	-0.026	5.9E-01	5.2E-01	8.0E-01
KIRP	HSF4_AA_10.1_9_10.2	HSF4	AA	10.1	9	10.2	2.5E-07	0.219	-0.061	5.0E-01	2.5E-01	2.0E-01
KIRP	HSF4_RI_14.2_14.1_14.3	HSF4	RI	14.2	14.1	14.3	1.9E-04	0.103	-0.016	2.9E-01	1.0E-01	5.1E-02
KIRP	HTATIP2_AD_1.2:1.3_1.1_1.5	HTATIP2	AD	1.2:1.3	1.1	1.5	4.4E-06	0.149	-0.061	2.3E-02	3.7E-02	1.5E-01
KIRP	HTATIP2_AD_1.3_1.2_1.5	HTATIP2	AD	1.3	1.2	1.5	1.2E-04	0.113	-0.131	1.5E-03	2.1E-02	7.8E-03
KIRP	HYOU1_AD_1.2_1.1_3	HYOU1	AD	1.2	1.1	3	2.7E-04	0.110	0.123	9.6E-01	6.1E-01	4.0E-01
KIRP	IFFO1_ES_3_1_4	IFFO1	ES	3	1	4	1.6E-06	0.161	0.435	8.0E-01	1.5E-01	2.8E-01
KIRP	IFT81_AD_1.2_1.1_2	IFT81	AD	1.2	1.1	2	2.7E-05	0.133	-0.028	3.1E-01	1.2E-02	1.1E-02
KIRP	IL18BP_RI_1.7_1.6_1.8	IL18BP	RI	1.7	1.6	1.8	1.6E-06	0.160	-0.312	4.6E-01	4.2E-02	2.9E-04
KIRP	IL32_AA_1.3:1.4:1.5:1.6:1.7_1.1_1.8	IL32	AA	1.3:1.4:1.5:1.6:1.	1.1	1.8	2.9E-06	0.150	0.325	6.3E-02	9.8E-02	8.3E-01
KIRP	IL32_AA_1.3:1.4:1.5:1.6:1.7:1.8_1.1_1.9	IL32	AA	3:1.4:1.5:1.6:1.7:	1.1	1.9	1.6E-07	0.181	0.260	5.9E-02	1.4E-01	9.2E-01
KIRP	IL32_ES_1.3:1.4:1.5_1.1_1.9	IL32	ES	1.3:1.4:1.5	1.1	1.9	1.0E-06	0.161	0.348	1.8E-01	5.3E-01	7.8E-01
KIRP	IL32_RI_1.2_1.1_1.3	IL32	RI	1.2	1.1	1.3	4.6E-05	0.119	0.102	1.6E-01	8.1E-01	7.4E-01
KIRP	IMMP1L_ES_3:4:5:6_1_7	IMMP1L	ES	3:4:5:6	1	7	1.2E-04	0.114	-0.149	8.0E-01	9.5E-01	3.2E-01
KIRP	IMPA1_ES_2_1_3	IMPA1	ES	2	1	3	9.2E-08	0.229	0.215	2.3E-01	3.9E-02	1.2E-01
KIRP	INF2_ES_22_21_23	INF2	ES	22	21	23	3.7E-08	0.198	0.239	8.0E-02	5.1E-02	4.0E-01

cancer type	id	Gene Symbol	splice_type	Exon	From.Exon	To.Exon	anova.p	adj.r2	r	p.50	p.25	p.10
KIRP	INO80E_AA_6.1_5_6.2	INO80E	AA	6.1	5	6.2	7.8E-09	0.213	0.196	9.9E-02	7.3E-02	3.4E-02
KIRP	INO80E_ES_10_6.3_11	INO80E	ES	10	6.3	11	6.4E-05	0.114	0.179	1.7E-01	2.4E-02	3.0E-02
KIRP	INO80E_ES_6.1:6.2:6.3:10_5_11	INO80E	ES	6.1:6.2:6.3:10	5	11	6.8E-06	0.140	0.062	5.1E-01	3.5E-01	6.2E-01
KIRP	INO80E_ES_6.2:6.3:10_5_11	INO80E	ES	6.2:6.3:10	5	11	4.4E-07	0.170	-0.056	2.3E-01	5.9E-01	3.0E-02
KIRP	INO80E_ES_7:8:9:10_6.3_11	INO80E	ES	7:8:9:10	6.3	11	9.7E-05	0.109	0.208	1.3E-01	7.9E-02	3.9E-01
KIRP	IP6K2_AD_11.3:11.4:11.5_11.2_11.9	IP6K2	AD	11.3:11.4:11.5	11.2	11.9	2.1E-05	0.129	0.241	1.1E-01	2.5E-01	2.3E-01
KIRP	IP6K2_ES_11.4:11.5_11.2_11.9	IP6K2	ES	11.4:11.5	11.2	11.9	1.0E-04	0.111	-0.306	1.7E-01	4.5E-01	2.4E-01
KIRP	IP6K2_RI_11.3_11.2_11.4	IP6K2	RI	11.3	11.2	11.4	8.8E-05	0.110	0.442	7.0E-01	9.4E-01	3.4E-01
KIRP	IP6K2_RI_11.3:11.4:11.5:11.6:11.7:11.8_	IP6K2	RI	1.4:11.5:11.6:11.	11.2	11.9	4.4E-05	0.118	0.472	8.2E-01	9.3E-01	3.3E-01
KIRP	IPO11_ES_30_29_33	IPO11	ES	30	29	33	5.1E-07	0.210	-0.092	1.9E-02	1.3E-03	4.8E-03
KIRP	IRAK1_ES_10.1:10.3_9_11.1	IRAK1	ES	10.1:10.3	9	11.1	9.3E-06	0.166	-0.147	3.2E-01	1.4E-01	5.7E-03
KIRP	IRF3_AD_1.2:1.3:1.4:1.5_1.1_2	IRF3	AD	1.2:1.3:1.4:1.5	1.1	2	5.3E-05	0.150	0.174	2.9E-02	1.7E-03	8.1E-06
KIRP	IRF3_AD_5.2_5.1_6.2	IRF3	AD	5.2	5.1	6.2	3.1E-05	0.123	0.296	2.6E-01	3.2E-01	5.5E-02
KIRP	IRF3_RI_1.2:1.3_1.1_1.4	IRF3	RI	1.2:1.3	1.1	1.4	1.3E-05	0.133	0.600	2.0E-01	8.5E-01	2.0E-01
KIRP	IRF3_RI_1.2:1.3:1.4_1.1_1.5	IRF3	RI	1.2:1.3:1.4	1.1	1.5	1.2E-05	0.172	0.312	4.3E-02	5.6E-02	5.1E-03
KIRP	ISCU_RI_5.2_5.1_5.3	ISCU	RI	5.2	5.1	5.3	3.0E-05	0.126	-0.429	2.0E-01	2.4E-01	4.8E-01
KIRP	ISOC2_ES_3:4.1_2_4.2	ISOC2	ES	03:04.1	2	4.2	4.6E-19	0.419	0.059	2.1E-03	3.9E-03	6.3E-05
KIRP	ITGA6_ES_27_26_28	ITGA6	ES	27	26	28	6.2E-08	0.197	-0.298	1.5E-01	8.1E-01	4.3E-01
KIRP	ITGB1BP1_AA_8.1:8.2_7_8.3	ITGB1BP1	AA	8.1:8.2	7	8.3	2.5E-10	0.247	-0.128	1.9E-03	5.3E-03	7.5E-02
KIRP	ITGB3BP_ES_9_8_10	ITGB3BP	ES	9	8	10	1.3E-05	0.133	-0.243	8.0E-01	6.0E-01	8.8E-01
KIRP	ITGB4_ES_35_34_36	ITGB4	ES	35	34	36	1.7E-04	0.103	-0.347	4.6E-01	3.0E-01	6.5E-01
KIRP	JUP_RI_15.2_15.1_15.3	JUP	RI	15.2	15.1	15.3	2.5E-10	0.247	0.102	3.7E-01	5.0E-02	1.9E-01
KIRP	KIAA1217_ES_12_11_13	KIAA1217	ES	12	11	13	1.1E-08	0.236	-0.246	3.6E-01	2.6E-01	9.0E-02
KIRP	KIF12_ES_15_14_16	KIF12	ES	15	14	16	1.6E-04	0.103	0.214	3.7E-02	1.0E-01	1.8E-03
KIRP	KIF13A_ES_40_39_41.1	KIF13A	ES	40	39	41.1	5.2E-05	0.119	-0.066	1.6E-01	1.9E-01	2.9E-01
KIRP	KIF13B_ES_39_38_40	KIF13B	ES	39	38	40	2.1E-05	0.133	0.034	3.7E-02	4.6E-02	2.0E-01
KIRP	KLC1_RI_14.2_14.1_14.3	KLC1	RI	14.2	14.1	14.3	9.9E-05	0.109	-0.014	2.8E-02	5.2E-01	1.8E-02
KIRP	KRT15_RI_7.2_7.1_7.3	KRT15	RI	7.2	7.1	7.3	1.8E-05	0.129	-0.713	8.2E-01	2.1E-01	6.4E-02
KIRP	LAMTOR5_RI_1.2_1.1_1.3	LAMTOR5	RI	1.2	1.1	1.3	5.1E-06	0.143	-0.151	1.6E-01	6.2E-02	1.9E-01
KIRP	LCLAT1_ES_3_2_4	LCLAT1	ES	3	2	4	1.4E-04	0.129	-0.418	6.4E-01	2.3E-01	2.8E-01
KIRP	LEKR1_AD_1.2_1.1_2	LEKR1	AD	1.2	1.1	2	2.1E-06	0.156	0.250	7.5E-01	9.1E-01	7.0E-01
KIRP	LENG8_RI_15.2:15.3:15.4_15.1_15.5	LENG8	RI	15.2:15.3:15.4	15.1	15.5	1.2E-04	0.106	0.860	7.6E-01	4.0E-01	3.7E-01
KIRP	LETMD1_ES_3.2:3.3:4_2_5	LETMD1	ES	3.2:3.3:4	2	5	3.4E-05	0.147	-0.165	4.6E-01	5.8E-01	3.1E-01
KIRP	LETMD1_ES_4:5:6_3.2_7	LETMD1	ES	4:05:06	3.2	7	4.0E-06	0.148	0.084	4.9E-01	7.4E-02	7.5E-02
KIRP	LIG1_AD_24.2_24.1_25	LIG1	AD	24.2	24.1	25	6.4E-08	0.191	0.582	4.3E-01	9.3E-01	8.2E-01
KIRP	LIMCH1_ES_28_27_29	LIMCH1	ES	28	27	29	3.6E-04	0.109	0.016	1.6E-01	6.7E-02	1.3E-02
KIRP	LIN37_RI_6.2_6.1_6.3	LIN37	RI	6.2	6.1	6.3	1.5E-05	0.131	-0.037	9.5E-02	7.8E-03	3.0E-02
KIRP	LMBR1L_ES_4.1:4.2_3_5	LMBR1L	ES	4.1:4.2	3	5	7.3E-05	0.116	0.404	6.1E-01	5.6E-01	5.8E-01
KIRP	LMO7_AA_19.1_18_19.2	LMO7	AA	19.1	18	19.2	6.3E-10	0.246	0.067	5.0E-01	2.0E-01	3.1E-01
KIRP	LMO7_ES_12_9_13	LMO7	ES	12	9	13	3.6E-04	0.109	-0.135	1.9E-01	3.4E-03	1.9E-01

cancer type	id	Gene Symbol	splice_type	Exon	From.Exon	To.Exon	anova.p	adj.r2	r	p.50	p.25	p.10
KIRP	LPAR2_ES_3:4_2.2_5.2	LPAR2	ES	3:04	2.2	5.2	3.9E-06	0.151	-0.015	2.0E-02	1.0E-02	5.0E-02
KIRP	LPHN2_ES_29_27_31	LPHN2	ES	29	27	31	5.7E-07	0.188	-0.114	3.8E-02	3.3E-02	7.4E-02
KIRP	LRCH3_ES_15_14_16	LRCH3	ES	15	14	16	4.1E-04	0.104	-0.159	4.8E-01	6.1E-01	7.2E-01
KIRP	LRRC23_ES_8_7.1_10	LRRC23	ES	8	7.1	10	1.1E-04	0.108	-0.010	8.7E-01	6.3E-01	6.9E-01
KIRP	LRRFIP2_AD_1.2_1.1_3	LRRFIP2	AD	1.2	1.1	3	8.4E-07	0.170	-0.190	5.6E-02	8.5E-03	3.8E-03
KIRP	LRRFIP2_ES_20_19_21	LRRFIP2	ES	20	19	21	6.9E-11	0.260	-0.003	2.1E-02	1.7E-02	4.7E-02
KIRP	LRRFIP2_ES_7:8_5_18	LRRFIP2	ES	7:08	5	18	7.9E-05	0.124	-0.181	5.4E-01	4.0E-01	4.0E-01
KIRP	LSR_ES_4:5_3_6	LSR	ES	4:05	3	6	9.5E-12	0.278	-0.372	4.9E-01	7.0E-01	2.5E-01
KIRP	LTBR_ES_8:9.1_7_10	LTBR	ES	08:09.1	7	10	3.5E-15	0.350	0.016	7.8E-03	4.1E-02	5.6E-03
KIRP	LUC7L_AD_1.2:1.3:1.4_1.1_2.2	LUC7L	AD	1.2:1.3:1.4	1.1	2.2	1.0E-09	0.234	0.625	6.8E-02	8.1E-02	1.7E-01
KIRP	LUC7L_AD_1.4_1.3_2.2	LUC7L	AD	1.4	1.3	2.2	6.2E-06	0.142	0.312	3.7E-01	1.1E-01	5.7E-01
KIRP	LUC7L_ES_1.3:1.4_1.1_2.2	LUC7L	ES	1.3:1.4	1.1	2.2	1.2E-08	0.210	0.647	2.0E-01	2.7E-01	9.8E-01
KIRP	LUC7L_ES_4_3_5	LUC7L	ES	4	3	5	3.5E-17	0.386	0.643	1.3E-02	1.8E-02	1.0E-01
KIRP	LUC7L3_RI_12.3:12.4:12.5_12.2_12.6	LUC7L3	RI	12.3:12.4:12.5	12.2	12.6	2.0E-06	0.153	0.764	2.1E-01	5.6E-01	9.7E-01
KIRP	LYRM9_RI_6.2_6.1_6.3	LYRM9	RI	6.2	6.1	6.3	4.1E-05	0.130	0.174	9.2E-01	9.7E-01	7.1E-01
KIRP	MACF1_ES_103_102_104	MACF1	ES	103	102	104	8.5E-06	0.138	0.163	2.4E-03	3.3E-04	2.6E-02
KIRP	MACF1_ES_107_106_108	MACF1	ES	107	106	108	4.4E-10	0.242	0.136	7.8E-03	8.1E-04	2.1E-04
KIRP	MANBAL_ES_2.2:3_1_4.2	MANBAL	ES	2.2:3	1	4.2	3.5E-05	0.121	-0.186	5.4E-01	1.8E-01	1.2E-01
KIRP	MAP3K4_ES_18_17_19	MAP3K4	ES	18	17	19	1.2E-06	0.163	-0.113	2.6E-01	5.0E-01	3.7E-01
KIRP	MAP3K4_ES_24_23_25	MAP3K4	ES	24	23	25	7.2E-05	0.116	0.152	2.1E-01	8.7E-02	1.7E-01
KIRP	MAPK10_ME_12|13_11_14	MAPK10	ME	12|13	11	14	1.1E-05	0.139	-0.199	3.2E-01	6.1E-01	6.3E-01
KIRP	MARK3_ES_17_16_18	MARK3	ES	17	16	18	3.3E-06	0.150	0.012	8.6E-01	6.4E-01	6.8E-01
KIRP	MARK3_ES_17_16_19	MARK3	ES	17	16	19	6.6E-11	0.260	0.137	9.0E-01	8.1E-01	3.1E-01
KIRP	MARK3_ES_17:18_16_19	MARK3	ES	17:18	16	19	4.4E-08	0.195	0.086	1.4E-01	4.6E-01	5.1E-01
KIRP	MARK3_ES_18_17_19	MARK3	ES	18	17	19	5.5E-05	0.119	-0.108	5.8E-01	8.0E-01	8.0E-01
KIRP	MAX_RI_5.2:5.3:5.4_5.1_5.5	MAX	RI	5.2:5.3:5.4	5.1	5.5	1.2E-06	0.167	-0.108	3.9E-01	3.0E-01	3.3E-01
KIRP	MAX_RI_5.6:5.7_5.5_5.8	MAX	RI	5.6:5.7	5.5	5.8	3.2E-07	0.175	-0.043	4.3E-02	2.8E-01	4.4E-01
KIRP	MAX_RI_5.7_5.6_5.8	MAX	RI	5.7	5.6	5.8	1.9E-08	0.205	-0.030	1.3E-01	7.4E-02	2.5E-02
KIRP	MEAF6_ES_7_5_8.1	MEAF6	ES	7	5	8.1	7.1E-12	0.283	-0.181	3.6E-01	8.8E-01	4.6E-01
KIRP	MEIS3_AA_7.1_6_7.2	MEIS3	AA	7.1	6	7.2	7.8E-08	0.193	0.769	1.5E-01	1.6E-01	3.1E-02
KIRP	METTL15_ES_11_10.2_12.1	METTL15	ES	11	10.2	12.1	3.7E-04	0.116	-0.058	9.8E-01	8.7E-01	7.0E-01
KIRP	METTL17_RI_10.2_10.1_10.3	METTL17	RI	10.2	10.1	10.3	4.9E-07	0.169	0.317	5.6E-01	1.4E-01	6.6E-01
KIRP	METTL23_ES_2_1.4_3	METTL23	ES	2	1.4	3	2.1E-04	0.101	0.046	1.3E-01	1.0E-02	5.9E-02
KIRP	METTL3_RI_8.2_8.1_8.3	METTL3	RI	8.2	8.1	8.3	1.9E-05	0.128	0.642	7.5E-01	3.9E-01	1.6E-01
KIRP	METTL3_RI_8.4_8.3_8.5	METTL3	RI	8.4	8.3	8.5	3.2E-05	0.122	0.605	5.9E-01	2.4E-01	3.4E-01
KIRP	MFF_ES_3:4_1_5	MFF	ES	3:04	1	5	2.5E-05	0.131	0.076	1.3E-01	2.6E-02	1.5E-02
KIRP	MFF_ES_3:4:5_1_6	MFF	ES	3:04:05	1	6	7.0E-08	0.191	0.221	2.0E-02	3.9E-02	1.7E-02
KIRP	MGRN1_ES_17.1_16_17.3	MGRN1	ES	17.1	16	17.3	1.7E-10	0.252	-0.070	6.4E-01	6.1E-01	9.4E-02
KIRP	MLLT4_ES_16_15_17	MLLT4	ES	16	15	17	1.4E-18	0.514	-0.208	1.2E-01	9.3E-04	5.8E-02
KIRP	MOCS1_ES_11.1:11.2_10_12.1	MOCS1	ES	11.1:11.2	10	12.1	2.9E-06	0.155	0.202	1.2E-01	3.2E-01	7.7E-01

cancer type	id	Gene Symbol	splice_type	Exon	From.Exon	To.Exon	anova.p	adj.r2	r	p.50	p.25	p.10
KIRP	MORF4L2_AA_5.1:5.2_3.2_5.3	MORF4L2	AA	5.1:5.2	3.2	5.3	4.2E-06	0.146	-0.198	2.8E-02	4.5E-01	8.4E-01
KIRP	MORF4L2_ES_5.2:5.3_3.2_6.2	MORF4L2	ES	5.2:5.3	3.2	6.2	5.6E-04	0.117	0.038	1.6E-01	1.8E-02	4.6E-02
KIRP	MPPE1_ES_12_11_13	MPPE1	ES	12	11	13	1.1E-07	0.185	-0.188	8.8E-01	9.4E-01	7.2E-01
KIRP	MPV17_ES_6.3_3.2_7	MPV17	ES	6.3	3.2	7	1.4E-04	0.125	0.131	4.7E-01	4.6E-02	1.6E-02
KIRP	MROH1_ES_26_25.2_27	MROH1	ES	26	25.2	27	1.5E-04	0.110	-0.049	4.8E-01	8.6E-01	1.2E-01
KIRP	MRPL2_ES_3:4:5_2.1_7	MRPL2	ES	3:04:05	2.1	7	4.7E-06	0.144	0.230	5.3E-02	7.9E-02	2.8E-01
KIRP	MRPL33_ES_3_2_4	MRPL33	ES	3	2	4	2.6E-09	0.224	0.047	5.1E-02	1.4E-02	2.2E-01
KIRP	MRPL35_AA_4.3_4.1_4.4	MRPL35	AA	4.3	4.1	4.4	8.1E-05	0.120	-0.057	2.2E-01	9.6E-01	1.2E-01
KIRP	MRPL52_RI_1.3_1.2_1.4	MRPL52	RI	1.3	1.2	1.4	2.0E-06	0.153	-0.141	6.7E-02	1.8E-01	9.7E-01
KIRP	MRPL55_AD_1.2_1.1_2.2	MRPL55	AD	1.2	1.1	2.2	8.1E-05	0.111	-0.124	5.7E-03	6.6E-03	1.5E-02
KIRP	MRPL55_AD_2.3:2.4:2.5:2.6_2.2_2.9	MRPL55	AD	2.3:2.4:2.5:2.6	2.2	2.9	2.7E-05	0.124	-0.239	9.7E-01	7.5E-01	6.1E-01
KIRP	MRPL55_RI_2.3:2.4:2.5:2.6:2.7_2.2_2.8	MRPL55	RI	2.3:2.4:2.5:2.6:2.7	2.2	2.8	4.0E-06	0.147	-0.120	6.7E-01	2.7E-01	2.1E-01
KIRP	MRPL55_RI_2.7_2.6_2.8	MRPL55	RI	2.7	2.6	2.8	9.4E-08	0.196	-0.106	2.2E-01	2.2E-01	1.4E-01
KIRP	MRPS33_ES_5.2_2_6	MRPS33	ES	5.2	2	6	2.1E-06	0.164	0.127	2.7E-02	1.9E-02	6.7E-03
KIRP	MSTO1_RI_18.2_18.1_18.3	MSTO1	RI	18.2	18.1	18.3	2.7E-05	0.129	0.157	1.1E-01	4.2E-01	1.5E-01
KIRP	MTA1_ES_4_3_5	MTA1	ES	4	3	5	7.1E-22	0.468	-0.083	2.0E-02	5.1E-02	2.9E-03
KIRP	MTERFD3_RI_1.2_1.1_1.3	MTERFD3	RI	1.2	1.1	1.3	1.1E-05	0.134	0.568	9.6E-01	8.9E-01	9.7E-01
KIRP	MTRR_ES_4.1:4.2_2_5	MTRR	ES	4.1:4.2	2	5	3.2E-06	0.161	0.151	8.7E-01	5.0E-01	4.2E-01
KIRP	MXRA7_ES_5_4_6	MXRA7	ES	5	4	6	2.9E-05	0.123	0.173	4.5E-01	5.1E-01	4.9E-01
KIRP	MYEF2_ES_3_2_4	MYEF2	ES	3	2	4	1.5E-04	0.111	-0.499	2.3E-02	3.7E-03	2.6E-02
KIRP	MYH10_ES_6.1_5_7	MYH10	ES	6.1	5	7	4.1E-14	0.399	-0.128	7.3E-02	1.7E-02	4.8E-03
KIRP	MYL5_AD_5.2_5.1_6	MYL5	AD	5.2	5.1	6	2.5E-05	0.125	0.073	9.3E-01	8.5E-01	4.1E-01
KIRP	MYL5_ES_3_2_4.1	MYL5	ES	3	2	4.1	1.3E-08	0.262	0.253	1.3E-01	2.4E-01	1.4E-01
KIRP	MYL6B_RI_7.2_7.1_7.3	MYL6B	RI	7.2	7.1	7.3	3.6E-06	0.147	-0.155	5.7E-01	1.0E+00	5.8E-01
KIRP	MYNN_ES_2_1_3	MYNN	ES	2	1	3	1.7E-05	0.171	-0.107	4.1E-04	1.1E-03	9.5E-03
KIRP	MYO18A_ES_41_40_42	MYO18A	ES	41	40	42	2.5E-05	0.125	0.045	6.8E-01	3.4E-01	9.5E-01
KIRP	MYO1B_ES_23_22_24	MYO1B	ES	23	22	24	1.3E-05	0.143	-0.008	1.8E-01	3.6E-01	7.2E-01
KIRP	MYO1B_ES_23:24_22_25	MYO1B	ES	23:24	22	25	6.0E-07	0.172	-0.040	4.8E-01	2.9E-01	9.3E-01
KIRP	N4BP2L1_ME_5|6_4_7.1	N4BP2L1	ME	5|6	4	7.1	4.6E-09	0.226	0.272	7.1E-02	5.1E-01	8.8E-02
KIRP	NAA25_ES_17_16_18	NAA25	ES	17	16	18	1.5E-04	0.135	0.165	9.3E-01	7.8E-02	1.8E-01
KIRP	NAA60_RI_10.4:10.5_10.3_10.6	NAA60	RI	10.4:10.5	10.3	10.6	4.6E-08	0.194	0.019	2.4E-02	2.6E-04	4.5E-04
KIRP	NAA60_RI_10.5_10.4_10.6	NAA60	RI	10.5	10.4	10.6	1.0E-07	0.186	0.023	1.4E-01	7.1E-03	6.2E-04
KIRP	NADK2_ES_10_9_11	NADK2	ES	10	9	11	2.4E-05	0.134	-0.081	1.4E-01	7.0E-02	5.7E-02
KIRP	NAGA_RI_1.2_1.1_1.3	NAGA	RI	1.2	1.1	1.3	9.4E-05	0.111	-0.099	8.4E-02	1.6E-02	1.9E-01
KIRP	NAGA_RI_1.2:1.3_1.1_1.4	NAGA	RI	1.2:1.3	1.1	1.4	2.3E-05	0.128	-0.090	2.7E-01	3.9E-01	2.0E-01
KIRP	NAPRT1_RI_11.5:11.6:11.7_11.4_11.8	NAPRT1	RI	11.5:11.6:11.7	11.4	11.8	5.3E-06	0.145	-0.281	7.3E-01	5.1E-01	5.1E-01
KIRP	NAT14_RI_2.3_2.2_2.4	NAT14	RI	2.3	2.2	2.4	4.7E-05	0.118	-0.055	1.6E-01	3.8E-01	6.1E-01
KIRP	NAT9_RI_6.3:6.4_6.2_6.5	NAT9	RI	6.3:6.4	6.2	6.5	2.2E-05	0.127	0.448	9.8E-01	7.4E-01	5.7E-01
KIRP	NBPF3_AD_12.3_12.2_13	NBPF3	AD	12.3	12.2	13	2.5E-04	0.108	0.224	1.8E-01	3.8E-01	7.6E-01
KIRP	NCOA1_ES_24_23_25.1	NCOA1	ES	24	23	25.1	2.9E-07	0.177	-0.127	2.2E-01	6.6E-03	1.7E-02

cancer type	id	Gene Symbol	splice_type	Exon	From.Exon	To.Exon	anova.p	adj.r2	r	p.50	p.25	p.10
KIRP	NDEL1_ES_11_10_12.1	NDEL1	ES	11	10	12.1	6.9E-07	0.166	0.250	2.2E-01	3.6E-01	8.9E-01
KIRP	NDRG2_ES_4.5_4.1_5.2	NDRG2	ES	4.5	4.1	5.2	1.9E-05	0.133	-0.065	2.4E-01	2.4E-01	3.5E-01
KIRP	NDUFA3_RI_4.2_4.1_4.3	NDUFA3	RI	4.2	4.1	4.3	1.3E-06	0.158	-0.346	1.2E-01	3.5E-01	4.2E-01
KIRP	NDUFC1_ES_2.2:3_2.1_5	NDUFC1	ES	2.2:3	2.1	5	2.3E-04	0.122	-0.263	1.9E-01	8.2E-02	1.9E-01
KIRP	NDUFS7_RI_9.2_9.1_9.3	NDUFS7	RI	9.2	9.1	9.3	2.7E-07	0.176	-0.142	3.3E-01	4.3E-01	3.8E-01
KIRP	NDUFV3_ES_3_2_4	NDUFV3	ES	3	2	4	1.1E-05	0.135	-0.529	4.1E-01	3.8E-01	1.5E-01
KIRP	NECAB3_RI_7.2_7.1_7.3	NECAB3	RI	7.2	7.1	7.3	1.7E-05	0.129	-0.190	3.2E-03	2.9E-02	3.4E-01
KIRP	NEDD4L_ES_18_17_19	NEDD4L	ES	18	17	19	3.7E-23	0.525	0.245	9.7E-02	7.1E-03	1.3E-03
KIRP	NF2_ES_16.1_15_17	NF2	ES	16.1	15	17	2.4E-07	0.179	-0.039	3.4E-01	5.0E-01	9.1E-01
KIRP	NFIB_ES_12.1:12.2:13:14_11_15	NFIB	ES	12.1:12.2:13:14	11	15	2.4E-04	0.106	-0.204	5.0E-01	1.5E-01	7.5E-03
KIRP	NHLRC3_ES_4_3_5	NHLRC3	ES	4	3	5	3.8E-07	0.172	-0.145	8.9E-03	4.1E-02	1.4E-01
KIRP	NISCH_RI_14.2_14.1_14.3	NISCH	RI	14.2	14.1	14.3	6.3E-08	0.192	0.420	8.7E-01	8.7E-01	2.4E-01
KIRP	NPHP1_ES_20_19_21	NPHP1	ES	20	19	21	2.3E-04	0.105	0.026	3.4E-02	4.9E-02	3.6E-02
KIRP	NPRL3_ES_5_4_6	NPRL3	ES	5	4	6	4.4E-06	0.146	-0.195	2.2E-01	2.6E-01	6.6E-01
KIRP	NSMF_AA_10.1:10.2_9.2_10.3	NSMF	AA	10.1:10.2	9.2	10.3	9.8E-05	0.130	0.232	1.4E-01	5.4E-01	1.1E-01
KIRP	NSUN5_RI_9.2_9.1_9.3	NSUN5	RI	9.2	9.1	9.3	1.6E-04	0.103	0.144	9.9E-01	3.8E-01	4.1E-01
KIRP	NT5C3B_ES_3_2_4	NT5C3B	ES	3	2	4	7.9E-07	0.164	0.159	4.8E-02	4.6E-02	4.2E-02
KIRP	NTAN1_ES_2_1_3	NTAN1	ES	2	1	3	7.7E-05	0.112	0.153	9.0E-02	1.3E-02	3.6E-02
KIRP	NUMA1_ES_18_17_19	NUMA1	ES	18	17	19	1.4E-04	0.105	0.242	6.0E-02	6.0E-01	8.7E-01
KIRP	NUMB_ES_7_6_8.2	NUMB	ES	7	6	8.2	1.9E-06	0.164	0.158	5.9E-01	8.1E-01	9.9E-01
KIRP	OCIAD1_ES_2.2:2.3:2.4:3:4_2.1_6	OCIAD1	ES	2.2:2.3:2.4:3:4	2.1	6	1.0E-04	0.117	-0.197	3.2E-01	3.5E-01	2.7E-01
KIRP	OCRL_ES_19_18_20	OCRL	ES	19	18	20	2.5E-04	0.124	0.197	4.3E-01	2.5E-01	4.7E-01
KIRP	OGFOD2_ES_6.3:8.1_6.2_8.2	OGFOD2	ES	6.3:8.1	6.2	8.2	8.1E-05	0.115	0.133	8.2E-01	7.4E-01	6.6E-01
KIRP	OGFOD2_ES_6.3:8.1:8.2_6.2_8.3	OGFOD2	ES	6.3:8.1:8.2	6.2	8.3	1.6E-09	0.229	0.192	7.1E-01	5.1E-01	6.7E-01
KIRP	OGFOD2_ES_7:8.2_6.2_8.3	OGFOD2	ES	07:08.2	6.2	8.3	2.5E-05	0.125	0.193	4.9E-01	4.7E-01	8.3E-01
KIRP	OGG1_ES_5:6.1:7.2_4_8	OGG1	ES	5:6.1:7.2	4	8	5.5E-09	0.241	-0.050	1.5E-01	2.3E-02	6.7E-03
KIRP	OGG1_RI_6.2:6.3_6.1_6.4	OGG1	RI	6.2:6.3	6.1	6.4	1.1E-05	0.134	-0.347	4.9E-02	1.9E-02	3.7E-02
KIRP	OPA1_ES_7_6_8	OPA1	ES	7	6	8	8.8E-07	0.177	0.139	8.0E-01	6.0E-01	2.2E-01
KIRP	ORMDL1_ES_2.2_1_2.4	ORMDL1	ES	2.2	1	2.4	8.4E-05	0.116	-0.188	2.2E-01	4.2E-01	4.2E-01
KIRP	ORMDL1_RI_2.3_2.2_2.4	ORMDL1	RI	2.3	2.2	2.4	5.6E-05	0.116	0.645	4.5E-01	1.7E-01	4.1E-01
KIRP	OSGEP_AD_4.5_4.4_5	OSGEP	AD	4.5	4.4	5	2.9E-08	0.200	-0.071	8.0E-01	9.1E-01	6.8E-01
KIRP	P4HA2_ES_2.1:2.2_1_3	P4HA2	ES	2.1:2.2	1	3	1.1E-08	0.223	0.258	6.9E-01	7.5E-02	4.6E-02
KIRP	PABPN1_RI_5.2_5.1_5.3	PABPN1	RI	5.2	5.1	5.3	8.3E-06	0.138	0.291	7.7E-01	2.9E-01	4.2E-01
KIRP	PAK4_ES_2.2:2.3_1_4	PAK4	ES	2.2:2.3	1	4	2.3E-08	0.205	-0.199	5.8E-01	4.4E-01	5.1E-01
KIRP	PALM_ES_8_7_9	PALM	ES	8	7	9	6.9E-06	0.141	-0.394	2.0E-01	1.7E-01	3.5E-01
KIRP	PAPSS2_ES_9_8_10	PAPSS2	ES	9	8	10	1.3E-09	0.272	0.069	6.4E-02	1.2E-01	3.3E-01
KIRP	PARD3_AD_19.2_19.1_20	PARD3	AD	19.2	19.1	20	1.4E-08	0.209	0.139	1.3E-01	9.4E-02	1.1E-01
KIRP	PARD3_AD_19.2:19.3_19.1_20	PARD3	AD	19.2:19.3	19.1	20	4.9E-12	0.284	0.106	7.5E-03	4.7E-03	2.0E-03
KIRP	PARD3_ES_13_12_14	PARD3	ES	13	12	14	2.9E-05	0.128	-0.062	3.7E-01	4.8E-01	3.1E-01
KIRP	PARP6_ES_18.1:18.2_17_19	PARP6	ES	18.1:18.2	17	19	1.5E-12	0.297	0.047	7.0E-01	3.3E-01	3.7E-01

cancer type	id	Gene Symbol	splice_type	Exon	From.Exon	To.Exon	anova.p	adj.r2	r	p.50	p.25	p.10
KIRP	PARP6_ES_18.2_17_19	PARP6	ES	18.2	17	19	2.8E-12	0.290	0.017	6.1E-01	4.8E-01	5.6E-01
KIRP	PAX8_ES_10_8_11	PAX8	ES	10	8	11	1.6E-14	0.344	0.248	1.7E-01	4.1E-02	2.9E-02
KIRP	PAX8_ES_9.2_8_10	PAX8	ES	9.2	8	10	5.9E-12	0.287	-0.175	1.9E-01	1.1E-01	8.4E-02
KIRP	PCBP4_ES_3_2.2_5	PCBP4	ES	3	2.2	5	7.2E-05	0.134	0.409	2.1E-01	3.7E-01	3.4E-01
KIRP	PCYT2_ES_7_6_8	PCYT2	ES	7	6	8	1.7E-08	0.206	0.006	3.2E-01	4.5E-01	9.8E-01
KIRP	PDGFA_ES_6_5_7	PDGFA	ES	6	5	7	9.4E-07	0.162	0.247	2.9E-02	2.4E-02	6.1E-03
KIRP	PDLIM7_AA_10.1_8_10.2	PDLIM7	AA	10.1	8	10.2	2.6E-05	0.124	0.049	3.0E-01	1.3E-01	5.5E-01
KIRP	PFDN5_ES_2:4.2:5_1_6.2	PFDN5	ES	04:02.2	1	6.2	4.1E-04	0.112	-0.236	1.2E-01	4.8E-03	4.1E-02
KIRP	PFDN5_ES_4.2:5_1_6.2	PFDN5	ES	4.2:5	1	6.2	1.4E-04	0.122	-0.210	2.1E-04	3.2E-05	1.1E-04
KIRP	PGAP2_AD_11.2:11.3_11.1_12	PGAP2	AD	11.2:11.3	11.1	12	1.5E-04	0.107	0.237	5.3E-02	2.5E-01	1.4E-01
KIRP	PHB2_ES_5.2:5.3:6.1_5.1_6.2	PHB2	ES	5.2:5.3:6.1	5.1	6.2	3.0E-08	0.205	-0.209	1.2E-01	7.2E-01	7.3E-01
KIRP	PHF6_RI_10.2_10.1_10.3	PHF6	RI	10.2	10.1	10.3	1.5E-09	0.260	0.071	2.5E-01	1.6E-01	4.4E-01
KIRP	PHPT1_ES_4_3_5	PHPT1	ES	4	3	5	2.2E-08	0.202	0.107	6.3E-02	3.0E-02	4.6E-02
KIRP	PI4KB_ES_5_4_6	PI4KB	ES	5	4	6	5.6E-07	0.172	-0.061	9.9E-01	7.4E-01	8.4E-01
KIRP	PIDD_AA_3.1_2.2_3.2	PIDD	AA	3.1	2.2	3.2	1.3E-04	0.118	0.361	8.4E-01	3.9E-01	5.3E-02
KIRP	PIGQ_ES_12_11_13	PIGQ	ES	12	11	13	4.1E-06	0.145	0.156	2.5E-01	7.2E-02	1.7E-01
KIRP	PIGQ_ES_12:13_11_14	PIGQ	ES	12:13	11	14	1.7E-13	0.315	0.255	2.8E-02	2.5E-02	1.0E-01
KIRP	PIGQ_ES_13_11_14	PIGQ	ES	13	11	14	2.5E-13	0.313	0.213	2.2E-02	3.7E-02	2.6E-01
KIRP	PILRB_RI_8.4_8.3_8.5	PILRB	RI	8.4	8.3	8.5	1.4E-04	0.106	-0.127	4.6E-01	5.5E-01	9.0E-01
KIRP	PLEKHA1_ES_4_1.1_5.2	PLEKHA1	ES	4	1.1	5.2	6.4E-05	0.145	0.099	5.5E-02	1.2E-01	7.0E-02
KIRP	PLEKHA4_ES_18_17_19	PLEKHA4	ES	18	17	19	2.0E-10	0.253	0.190	5.1E-02	1.4E-02	1.1E-02
KIRP	PLEKHA6_AA_7.1_6_7.2	PLEKHA6	AA	7.1	6	7.2	4.8E-04	0.102	-0.291	2.3E-01	3.6E-01	2.1E-01
KIRP	PLEKHJ1_RI_5.2_5.1_5.3	PLEKHJ1	RI	5.2	5.1	5.3	4.2E-06	0.145	-0.052	9.3E-01	6.3E-01	1.4E-01
KIRP	PLXNB2_ES_2.1_1_3	PLXNB2	ES	2.1	1	3	1.2E-05	0.170	0.011	1.8E-01	2.6E-01	1.0E-01
KIRP	POLR2J3_RI_4.2_4.1_4.3	POLR2J3	RI	4.2	4.1	4.3	1.8E-04	0.102	0.344	1.8E-02	1.9E-01	2.2E-01
KIRP	POLR3H_AD_5.2_5.1_6	POLR3H	AD	5.2	5.1	6	3.1E-07	0.175	-0.334	1.0E-02	8.3E-02	2.4E-02
KIRP	POLR3H_ES_4_3_5.1	POLR3H	ES	4	3	5.1	1.6E-04	0.103	-0.171	3.5E-03	1.3E-02	4.5E-02
KIRP	POPDC2_AD_3.2_3.1_5	POPDC2	AD	3.2	3.1	5	5.9E-04	0.102	-0.146	1.3E-01	1.2E-01	2.2E-01
KIRP	PPFIA3_RI_29.2_29.1_29.3	PPFIA3	RI	29.2	29.1	29.3	3.3E-07	0.176	-0.184	1.5E-03	1.1E-04	3.0E-03
KIRP	PPHLN1_ES_6_5_7	PPHLN1	ES	6	5	7	1.6E-04	0.104	-0.082	3.6E-01	1.2E-02	1.2E-02
KIRP	PPIP5K2_ES_26_25_27	PPIP5K2	ES	26	25	27	1.4E-04	0.119	0.202	1.0E+00	4.4E-01	5.3E-01
KIRP	PPIP5K2_ES_28:29_27_30	PPIP5K2	ES	28:29:00	27	30	2.5E-12	0.307	0.005	5.9E-01	1.8E-01	4.7E-01
KIRP	PPP1R1A_ES_4_3_6	PPP1R1A	ES	4	3	6	4.5E-07	0.191	0.286	4.6E-01	8.2E-01	7.1E-01
KIRP	PPP4C_ES_5.1:5.2_4_6	PPP4C	ES	5.1:5.2	4	6	6.6E-06	0.150	-0.073	7.6E-01	2.0E-01	6.0E-01
KIRP	PPP4R1_ES_15_14_16	PPP4R1	ES	15	14	16	1.8E-04	0.104	-0.045	5.9E-01	7.6E-01	4.9E-01
KIRP	PQBP1_AD_1.2:1.3_1.1_2.3	PQBP1	AD	1.2:1.3	1.1	2.3	7.3E-08	0.192	-0.109	3.9E-03	1.9E-03	3.7E-02
KIRP	PREPL_ES_2.2:2.3_1.3_3	PREPL	ES	2.2:2.3	1.3	3	2.1E-04	0.122	-0.294	6.9E-01	1.4E-01	3.8E-02
KIRP	PRMT2_AD_1.2:1.3_1.1_2	PRMT2	AD	1.2:1.3	1.1	2	2.4E-05	0.126	0.174	6.4E-02	4.5E-01	8.4E-01
KIRP	PRMT2_ES_1.3_1.1_2	PRMT2	ES	1.3	1.1	2	2.1E-06	0.155	0.151	2.9E-03	4.9E-02	5.4E-02
KIRP	PTBP2_ES_12_11.2_13	PTBP2	ES	12	11.2	13	2.9E-05	0.158	0.023	3.7E-02	9.7E-03	3.9E-03

cancer type	id	Gene Symbol	splice_type	Exon	From.Exon	To.Exon	anova.p	adj.r2	r	p.50	p.25	p.10
KIRP	PTK2_ES_39.2_37_39.5	PTK2	ES	39.2	37	39.5	9.8E-13	0.304	-0.105	8.1E-02	8.9E-02	3.5E-02
KIRP	PTOV1_RI_13.2_13.1_13.3	PTOV1	RI	13.2	13.1	13.3	4.1E-08	0.197	-0.082	3.8E-01	7.6E-01	4.2E-01
KIRP	PTPRS_ES_28_27.2_29	PTPRS	ES	28	27.2	29	2.4E-05	0.136	0.046	2.9E-01	1.3E-01	2.4E-01
KIRP	QKI_AA_8.1_7_8.2	QKI	AA	8.1	7	8.2	1.6E-04	0.111	0.068	9.6E-02	6.6E-02	7.7E-01
KIRP	QKI_RI_8.3_8.2_8.4	QKI	RI	8.3	8.2	8.4	2.0E-05	0.148	-0.207	2.0E-01	7.4E-02	1.9E-01
KIRP	QTRT1_RI_6.2_6.1_6.3	QTRT1	RI	6.2	6.1	6.3	1.7E-05	0.129	0.596	4.5E-01	7.2E-01	4.1E-01
KIRP	RAB9A_ES_2_1_3	RAB9A	ES	2	1	3	1.5E-04	0.107	0.089	2.0E-01	6.3E-01	2.1E-01
KIRP	RABGGTA_RI_1.2:1.3_1.1_1.4	RABGGTA	RI	1.2:1.3	1.1	1.4	3.0E-05	0.124	0.207	2.3E-02	1.8E-03	1.4E-02
KIRP	RABL2A_ES_2_1_3.1	RABL2A	ES	2	1	3.1	2.9E-04	0.110	0.364	2.0E-01	2.9E-01	5.9E-01
KIRP	RABL2A_ES_2:3.1_1_3.2	RABL2A	ES	02:03.1	1	3.2	3.6E-06	0.159	0.427	1.1E-01	1.7E-01	2.0E-01
KIRP	RABL2B_AD_2.3_2.2_3.1	RABL2B	AD	2.3	2.2	3.1	1.4E-04	0.108	0.320	2.3E-01	8.2E-01	2.1E-01
KIRP	RABL2B_ES_2.1:2.2:2.3:3.1_1_3.2	RABL2B	ES	2.1:2.2:2.3:3.1	1	3.2	7.5E-06	0.142	0.399	2.9E-01	1.6E-01	9.2E-01
KIRP	RAC1_ES_4_3_5	RAC1	ES	4	3	5	2.0E-10	0.249	0.151	7.2E-01	7.9E-01	3.5E-01
KIRP	RALGPS2_ES_15_14_16	RALGPS2	ES	15	14	16	1.1E-05	0.166	-0.138	1.5E-02	6.9E-03	3.3E-04
KIRP	RANBP1_RI_7.2:7.3_7.1_7.4	RANBP1	RI	7.2:7.3	7.1	7.4	1.0E-04	0.109	-0.158	3.1E-01	1.9E-01	5.0E-01
KIRP	RANGRF_AD_3.2:3.3_3.1_3.5	RANGRF	AD	3.2:3.3	3.1	3.5	4.5E-05	0.119	-0.169	1.8E-01	3.1E-01	1.6E-01
KIRP	RANGRF_RI_3.2:3.3:3.4_3.1_3.5	RANGRF	RI	3.2:3.3:3.4	3.1	3.5	6.9E-06	0.140	-0.066	7.5E-01	8.5E-01	5.3E-01
KIRP	RANGRF_RI_3.4_3.3_3.5	RANGRF	RI	3.4	3.3	3.5	4.9E-09	0.218	0.256	5.6E-01	5.7E-01	4.3E-01
KIRP	RAP1GAP_RI_22.2:22.3_22.1_22.4	RAP1GAP	RI	22.2:22.3	22.1	22.4	1.6E-04	0.127	-0.393	4.2E-01	9.5E-01	5.5E-02
KIRP	RARRES2_RI_1.3_1.2_1.4	RARRES2	RI	1.3	1.2	1.4	9.8E-09	0.212	-0.537	5.8E-01	5.8E-01	7.0E-01
KIRP	RBM42_ES_5:6.1_4_6.2	RBM42	ES	05:06.1	4	6.2	6.1E-07	0.167	-0.228	5.4E-01	5.8E-01	9.9E-01
KIRP	RBMX_RI_8.2_8.1_8.3	RBMX	RI	8.2	8.1	8.3	2.9E-05	0.123	0.220	5.3E-02	1.8E-01	2.2E-01
KIRP	RDH13_AD_9.2_9.1_10	RDH13	AD	9.2	9.1	10	3.3E-07	0.173	0.175	8.2E-01	8.8E-01	1.2E-01
KIRP	RDH13_ES_8_7_9.1	RDH13	ES	8	7	9.1	6.2E-07	0.167	0.171	1.5E-01	7.6E-02	5.0E-02
KIRP	REPIN1_ES_4.1:4.2_3.2_5.3	REPIN1	ES	4.1:4.2	3.2	5.3	4.8E-04	0.106	0.090	6.4E-01	1.7E-01	4.9E-01
KIRP	REPIN1_ES_4.2_3.2_5.2	REPIN1	ES	4.2	3.2	5.2	2.9E-06	0.155	0.358	1.0E-01	2.9E-01	7.9E-01
KIRP	REPIN1_ES_4.2:5.2_3.2_5.3	REPIN1	ES	4.2:5.2	3.2	5.3	4.7E-07	0.187	0.358	5.2E-01	1.4E-01	7.4E-01
KIRP	REV1_RI_22.2_22.1_22.3	REV1	RI	22.2	22.1	22.3	4.7E-05	0.119	0.350	4.2E-01	1.0E+00	7.1E-01
KIRP	RGS12_ES_19_18_20.1	RGS12	ES	19	18	20.1	3.2E-10	0.245	0.382	6.8E-02	9.2E-04	2.1E-03
KIRP	RHEB_ES_1.2:3_1.1_4	RHEB	ES	1.2:3	1.1	4	1.5E-05	0.148	-0.172	1.2E-01	5.4E-02	1.4E-01
KIRP	RHOC_AA_2.1:2.2_1.1_2.3	RHOC	AA	2.1:2.2	1.1	2.3	2.1E-05	0.127	-0.399	7.7E-01	8.1E-01	7.7E-01
KIRP	RHOC_ES_2.2:2.3_1.1_3	RHOC	ES	2.2:2.3	1.1	3	9.0E-13	0.300	0.108	7.1E-01	9.2E-01	8.3E-01
KIRP	RIF1_ES_31_30_32	RIF1	ES	31	30	32	9.2E-07	0.180	-0.263	3.7E-03	1.0E-01	2.5E-01
KIRP	RNF167_AD_1.2_1.1_2.2	RNF167	AD	1.2	1.1	2.2	1.6E-05	0.130	-0.040	6.8E-01	3.2E-01	1.4E-02
KIRP	RNF181_ES_3.2_2_4.1	RNF181	ES	3.2	2	4.1	1.9E-04	0.105	0.019	1.3E-01	2.2E-01	2.6E-01
KIRP	RNF41_AD_1.2_1.1_2	RNF41	AD	1.2	1.1	2	2.6E-04	0.101	0.050	4.7E-04	2.2E-03	3.2E-03
KIRP	RNF7_ES_1.2:2_1.1_3	RNF7	ES	1.2:2	1.1	3	6.9E-06	0.140	-0.087	4.2E-01	2.7E-01	2.2E-01
KIRP	RNH1_AA_4.2_1_4.3	RNH1	AA	4.2	1	4.3	1.0E-04	0.108	-0.005	8.8E-01	2.9E-01	4.0E-01
KIRP	RNH1_AA_4.2_3_4.3	RNH1	AA	4.2	3	4.3	1.5E-05	0.131	0.055	1.7E-01	3.0E-01	1.3E-01
KIRP	RPL15_AD_1.3_1.2_3.2	RPL15	AD	1.3	1.2	3.2	1.5E-13	0.316	-0.022	9.7E-02	4.5E-02	1.4E-02

cancer type	id	Gene Symbol	splice_type	Exon	From.Exon	To.Exon	anova.p	adj.r2	r	p.50	p.25	p.10
KIRP	RPL35_AD_4.2_4.1_5	RPL35	AD	4.2	4.1	5	2.0E-07	0.193	-0.138	5.9E-01	5.3E-01	7.0E-01
KIRP	RPRD2_ES_4_3.1_5	RPRD2	ES	4	3.1	5	2.5E-06	0.177	-0.139	1.5E-01	1.4E-01	9.0E-03
KIRP	RPS15_ES_1.2:1.4_1.1_1.5	RPS15	ES	1.2:1.4	1.1	1.5	6.1E-05	0.118	-0.100	1.6E-01	1.0E+00	5.1E-01
KIRP	RPS24_AA_5.1_4_5.2	RPS24	AA	5.1	4	5.2	8.2E-13	0.301	-0.178	1.4E-04	2.1E-03	6.8E-02
KIRP	RPS24_ES_5.1:5.2_4_6	RPS24	ES	5.1:5.2	4	6	4.9E-25	0.513	-0.147	2.6E-03	6.0E-04	9.5E-05
KIRP	RPS24_ES_5.2_4_6	RPS24	ES	5.2	4	6	2.7E-17	0.388	-0.130	3.2E-02	1.1E-02	1.5E-03
KIRP	RPS9_AA_4.1:4.2:4.3:4.4_3_4.5	RPS9	AA	4.1:4.2:4.3:4.4	3	4.5	1.2E-06	0.160	-0.352	3.8E-01	9.9E-01	8.7E-01
KIRP	RPS9_RI_4.2_4.1_4.3	RPS9	RI	4.2	4.1	4.3	3.4E-09	0.221	-0.426	6.1E-01	8.8E-01	4.4E-01
KIRP	RPS9_RI_4.4_4.3_4.5	RPS9	RI	4.4	4.3	4.5	1.0E-08	0.210	-0.308	4.9E-01	8.8E-01	3.6E-01
KIRP	RRBP1_ES_2_1_3.1	RRBP1	ES	2	1	3.1	7.1E-09	0.217	-0.002	5.0E-02	3.9E-02	2.2E-02
KIRP	RREB1_ES_12_11_13	RREB1	ES	12	11	13	6.4E-05	0.127	-0.265	1.2E-01	3.4E-03	2.9E-03
KIRP	RRNAD1_RI_2.2_2.1_2.3	RRNAD1	RI	2.2	2.1	2.3	2.9E-08	0.199	0.044	5.4E-02	5.1E-02	1.1E-01
KIRP	RUSC1_RI_3.3_3.2_3.4	RUSC1	RI	3.3	3.2	3.4	7.9E-05	0.113	0.085	5.2E-03	6.2E-02	3.5E-01
KIRP	RWDD1_ES_3_1_4	RWDD1	ES	3	1	4	2.2E-05	0.127	-0.030	8.3E-01	9.2E-01	2.6E-01
KIRP	S100A16_AD_2.2_2.1_4	S100A16	AD	2.2	2.1	4	9.8E-07	0.161	-0.156	6.2E-02	4.6E-02	7.5E-02
KIRP	SCAMP5_ES_7_6_8.2	SCAMP5	ES	7	6	8.2	8.2E-10	0.263	0.112	7.1E-01	5.7E-01	2.7E-02
KIRP	SCNN1A_ES_10_9_11	SCNN1A	ES	10	9	11	3.4E-06	0.149	-0.493	2.1E-01	3.1E-01	9.7E-01
KIRP	SCRIB_ES_36_35_37	SCRIB	ES	36	35	37	1.7E-06	0.155	-0.201	8.1E-01	7.8E-01	5.8E-01
KIRP	SDHD_ES_3.1:3.2:4_2_5	SDHD	ES	3.1:3.2:4	2	5	6.2E-04	0.114	-0.359	2.9E-01	8.8E-01	7.9E-01
KIRP	SEC24C_RI_23.2_23.1_23.3	SEC24C	RI	23.2	23.1	23.3	1.9E-04	0.101	-0.304	1.7E-01	5.7E-01	4.6E-01
KIRP	SEC31A_ES_26.1_25.1_28	SEC31A	ES	26.1	25.1	28	3.4E-07	0.176	-0.090	2.9E-04	4.6E-04	2.6E-02
KIRP	SEC31A_ES_26.1:26.2_25.1_28	SEC31A	ES	26.1:26.2	25.1	28	2.4E-08	0.202	-0.235	1.3E-03	6.3E-04	9.3E-01
KIRP	SEC31A_ES_26.1:26.2:27_25.1_28	SEC31A	ES	26.1:26.2:27	25.1	28	1.1E-38	0.674	-0.008	1.3E-03	1.9E-04	2.8E-05
KIRP	SEC31A_ES_26.1:27_25.1_28	SEC31A	ES	26.1:27	25.1	28	2.7E-18	0.435	0.007	4.2E-02	4.1E-03	2.2E-02
KIRP	SEC31A_ES_26.2:27_26.1_28	SEC31A	ES	26.2:27	26.1	28	5.2E-22	0.467	0.023	5.0E-01	4.5E-01	1.3E-02
KIRP	SEC31A_ES_27_26.1_28	SEC31A	ES	27	26.1	28	7.3E-09	0.226	0.079	8.8E-01	2.1E-01	8.0E-02
KIRP	SEC31A_ES_27_26.2_28	SEC31A	ES	27	26.2	28	4.8E-14	0.328	0.164	1.8E-01	2.8E-02	8.7E-02
KIRP	SEMA3F_ES_7_6_8	SEMA3F	ES	7	6	8	2.7E-04	0.105	0.422	5.3E-02	6.3E-01	5.5E-02
KIRP	SEMA4D_ES_5_3_6	SEMA4D	ES	5	3	6	1.7E-04	0.142	-0.123	9.0E-01	8.7E-01	8.0E-01
KIRP	SEPT8_AA_12.1_11_12.2	SEPT8	AA	12.1	11	12.2	9.9E-07	0.161	0.159	4.7E-01	3.5E-02	1.8E-01
KIRP	SERGEF_AD_1.2_1.1_3	SERGEF	AD	1.2	1.1	3	2.0E-04	0.111	0.011	1.1E-01	1.8E-03	1.3E-01
KIRP	SERPINA1_AA_2.1:2.2:2.3_1.1_2.4	SERPINA1	AA	2.1:2.2:2.3	1.1	2.4	3.2E-06	0.159	0.353	9.5E-02	7.4E-02	9.8E-01
KIRP	SERPINA1_AA_2.1:2.2:2.3:2.4_1.1_2.5	SERPINA1	AA	2.1:2.2:2.3:2.4	1.1	2.5	1.1E-06	0.172	0.354	7.9E-02	4.1E-01	4.4E-01
KIRP	SFSWAP_AD_2.2_2.1_3	SFSWAP	AD	2.2	2.1	3	4.6E-05	0.118	-0.380	3.8E-01	4.9E-01	2.4E-01
KIRP	SH3D19_ES_13_12_14	SH3D19	ES	13	12	14	4.5E-05	0.127	-0.074	8.0E-01	8.9E-01	6.7E-01
KIRP	SLAIN2_ES_8_6_9	SLAIN2	ES	8	6	9	3.7E-07	0.174	-0.031	5.0E-01	2.1E-01	7.9E-01
KIRP	SLC17A3_ES_5_4_6	SLC17A3	ES	5	4	6	5.2E-06	0.151	-0.199	5.4E-02	6.9E-02	2.2E-01
KIRP	SLC25A10_ES_7_6_8.1	SLC25A10	ES	7	6	8.1	1.1E-09	0.232	-0.185	6.5E-01	3.5E-02	6.3E-01
KIRP	SLC25A37_AA_3.1_2.1_3.2	SLC25A37	AA	3.1	2.1	3.2	6.2E-07	0.167	0.651	2.3E-01	8.5E-01	8.6E-01
KIRP	SLC25A37_AD_2.2_2.1_3.2	SLC25A37	AD	2.2	2.1	3.2	1.1E-04	0.108	0.481	3.6E-01	9.2E-01	6.3E-01

cancer type	id	Gene Symbol	splice_type	Exon	From.Exon	To.Exon	anova.p	adj.r2	r	p.50	p.25	p.10
KIRP	SLC25A40_ES_3_2_4	SLC25A40	ES	3	2	4	6.7E-04	0.112	-0.107	1.3E-02	1.3E-02	4.0E-02
KIRP	SLC26A1_ES_2_1_3	SLC26A1	ES	2	1	3	1.3E-04	0.121	-0.591	4.0E-01	3.0E-01	2.9E-01
KIRP	SLC29A2_AA_10.1_9_10.2	SLC29A2	AA	10.1	9	10.2	4.6E-04	0.101	-0.192	1.6E-01	1.6E-01	3.4E-01
KIRP	SLC35C2_AA_3.1_1_3.2	SLC35C2	AA	3.1	1	3.2	4.3E-05	0.120	0.138	6.2E-01	9.6E-01	8.5E-01
KIRP	SLC38A1_AD_1.2_1.1_4	SLC38A1	AD	1.2	1.1	4	4.6E-06	0.173	-0.123	5.8E-01	8.5E-01	5.0E-01
KIRP	SLC7A9_RI_7.2_7.1_7.3	SLC7A9	RI	7.2	7.1	7.3	3.4E-07	0.173	-0.846	1.7E-01	8.5E-01	4.4E-01
KIRP	SLK_ES_13_12_14	SLK	ES	13	12	14	4.4E-08	0.202	-0.048	2.0E-02	5.5E-03	2.3E-02
KIRP	SLMAP_ES_12:14_11_15.2	SLMAP	ES	12:14	11	15.2	8.2E-04	0.101	-0.086	6.4E-01	2.5E-01	2.7E-01
KIRP	SLMAP_ES_25_24_26	SLMAP	ES	25	24	26	2.1E-07	0.179	-0.067	3.3E-01	5.5E-01	7.1E-01
KIRP	SMARCC2_AD_28.2:28.3_28.1_29	SMARCC2	AD	28.2:28.3	28.1	29	3.4E-09	0.221	0.160	1.2E-01	1.4E-01	3.1E-02
KIRP	SMARCC2_ES_18_17_19	SMARCC2	ES	18	17	19	4.8E-07	0.170	-0.073	6.9E-03	5.7E-04	8.9E-04
KIRP	SMARCC2_ES_28.3_28.1_29	SMARCC2	ES	28.3	28.1	29	2.1E-06	0.155	0.157	3.2E-03	7.2E-04	1.5E-02
KIRP	SMG7_AD_16.2_16.1_17	SMG7	AD	16.2	16.1	17	1.1E-04	0.116	-0.223	7.5E-02	5.8E-02	3.8E-01
KIRP	SMIM19_AD_2.2:2.3_2.1_3.2	SMIM19	AD	2.2:2.3	2.1	3.2	8.8E-07	0.163	-0.435	5.1E-01	4.4E-02	4.4E-01
KIRP	SMUG1_ES_1.2:2.2:2.3_1.1_3	SMUG1	ES	1.2:2.2:2.3	1.1	3	9.3E-06	0.178	-0.149	6.9E-03	9.2E-02	7.3E-02
KIRP	SMUG1_ES_2.2:2.3_1.1_3	SMUG1	ES	2.2:2.3	1.1	3	2.0E-04	0.103	0.019	2.1E-01	9.9E-01	5.5E-01
KIRP	SNAPC5_ES_1.2:2.1_1.1_3.1	SNAPC5	ES	1.2:2.1	1.1	3.1	9.9E-09	0.225	-0.041	2.0E-01	1.7E-02	5.1E-03
KIRP	SNRNP70_AD_8.2:8.3_8.1_9	SNRNP70	AD	8.2:8.3	8.1	9	2.6E-05	0.124	0.002	1.1E-02	1.0E-01	3.7E-01
KIRP	SNX27_AA_12.1_11_12.2	SNX27	AA	12.1	11	12.2	3.3E-06	0.154	-0.233	6.9E-01	3.3E-01	3.2E-01
KIRP	SNX5_RI_1.2_1.1_1.3	SNX5	RI	1.2	1.1	1.3	5.8E-09	0.218	-0.203	7.7E-04	1.2E-02	3.6E-02
KIRP	SORBS2_ES_8:9.1:9.2_7_10	SORBS2	ES	8:9.1:9.2	7	10	1.5E-17	0.433	0.263	1.5E-02	8.9E-04	8.1E-03
KIRP	SORBS2_ES_9.1:9.2_8_10	SORBS2	ES	9.1:9.2	8	10	2.5E-05	0.143	0.321	5.6E-01	3.5E-01	2.3E-01
KIRP	SPAG6_ES_3:4:5_2.1_6	SPAG6	ES	3:04:05	2.1	6	3.5E-04	0.105	-0.798	7.8E-01	9.8E-01	4.6E-01
KIRP	SPAG9_ES_30_29_31	SPAG9	ES	30	29	31	5.7E-05	0.116	0.073	1.2E-01	7.0E-01	9.2E-01
KIRP	SPTAN1_ES_38_36.1_39	SPTAN1	ES	38	36.1	39	2.4E-10	0.250	0.116	2.2E-03	4.7E-02	5.4E-01
KIRP	SREBF1_ES_2_1_3.2	SREBF1	ES	2	1	3.2	2.1E-08	0.227	0.318	8.4E-01	5.8E-01	4.1E-01
KIRP	SRSF2_AA_2.3:2.4_2.1_2.5	SRSF2	AA	2.3:2.4	2.1	2.5	9.4E-07	0.162	0.568	4.2E-01	3.2E-01	9.3E-01
KIRP	SRSF2_AD_2.2:2.3_2.1_2.5	SRSF2	AD	2.2:2.3	2.1	2.5	5.9E-08	0.192	0.586	5.5E-01	5.2E-01	8.0E-01
KIRP	SRSF2_ES_2.3_2.1_2.5	SRSF2	ES	2.3	2.1	2.5	3.6E-06	0.147	0.099	9.4E-01	9.0E-01	9.9E-01
KIRP	SRSF2_RI_2.2:2.3:2.4_2.1_2.5	SRSF2	RI	2.2:2.3:2.4	2.1	2.5	3.3E-06	0.148	0.679	6.4E-01	6.8E-01	5.3E-01
KIRP	SRSF5_AA_8.1_7.2_8.2	SRSF5	AA	8.1	7.2	8.2	1.0E-07	0.186	0.615	1.6E-01	4.4E-01	8.2E-01
KIRP	SRSF5_AD_2.2:2.3_2.1_4	SRSF5	AD	2.2:2.3	2.1	4	2.2E-08	0.204	-0.255	2.0E-03	4.5E-03	1.3E-02
KIRP	SRSF7_RI_4.2_4.1_4.3	SRSF7	RI	4.2	4.1	4.3	1.3E-05	0.133	0.208	3.1E-03	1.1E-02	8.2E-01
KIRP	SRSF7_RI_4.2:4.3:4.4:4.5_4.1_4.6	SRSF7	RI	4.2:4.3:4.4:4.5	4.1	4.6	1.7E-04	0.102	0.387	2.5E-01	5.4E-02	1.6E-01
KIRP	SRSF7_RI_4.5_4.4_4.6	SRSF7	RI	4.5	4.4	4.6	1.5E-07	0.182	0.228	4.7E-03	3.0E-02	2.4E-01
KIRP	SS18_ES_15_14_16	SS18	ES	15	14	16	4.2E-05	0.121	-0.204	3.2E-01	1.1E-01	4.1E-02
KIRP	SSBP4_ES_7_6_8	SSBP4	ES	7	6	8	3.5E-08	0.197	-0.013	8.2E-03	1.0E-01	1.0E-01
KIRP	ST3GAL1_RI_5.4_5.3_5.5	ST3GAL1	RI	5.4	5.3	5.5	3.2E-05	0.146	-0.231	7.6E-02	1.6E-01	1.8E-01
KIRP	STAG3_ES_13_12_14	STAG3	ES	13	12	14	1.0E-04	0.134	0.265	9.3E-01	3.7E-01	4.2E-01
KIRP	STAG3_ES_5:6:7:8:9_4_10	STAG3	ES	5:6:7:8:9	4	10	6.5E-05	0.115	-0.141	6.0E-01	9.5E-01	9.8E-01

cancer type	id	Gene Symbol	splice_type	Exon	From.Exon	To.Exon	anova.p	adj.r2	r	p.50	p.25	p.10
KIRP	STAG3_ES_5:6:7:8:9:10_4_11	STAG3	ES	5:6:7:8:9:10	4	11	9.8E-05	0.112	-0.080	7.6E-01	6.2E-01	7.1E-01
KIRP	STAP2_AA_14.1:14.2_13_14.3	STAP2	AA	14.1:14.2	13	14.3	1.8E-06	0.155	-0.599	4.1E-02	7.8E-02	7.0E-03
KIRP	STAP2_AA_14.2_13_14.3	STAP2	AA	14.2	13	14.3	5.9E-05	0.115	-0.381	1.0E-02	6.3E-03	1.5E-03
KIRP	STAT6_ES_4_2.1_6	STAT6	ES	4	2.1	6	4.8E-04	0.110	-0.012	2.0E-03	3.7E-03	2.6E-03
KIRP	STK36_AA_23.1_22_23.2	STK36	AA	23.1	22	23.2	4.9E-10	0.244	-0.050	4.6E-01	8.3E-01	1.7E-01
KIRP	STRA6_AD_18.2_18.1_19	STRA6	AD	18.2	18.1	19	9.4E-11	0.258	-0.810	2.3E-03	6.6E-07	4.1E-07
KIRP	STRADA_ES_2.2:3_2.1_6	STRADA	ES	2.2:3	2.1	6	1.1E-04	0.117	0.121	7.7E-01	1.2E-01	4.7E-01
KIRP	SUGP2_RI_12.4_12.3_12.5	SUGP2	RI	12.4	12.3	12.5	5.6E-06	0.142	0.737	8.6E-01	5.5E-01	4.9E-01
KIRP	SUGT1_ES_7_6_8	SUGT1	ES	7	6	8	1.2E-09	0.232	0.266	4.7E-01	8.5E-01	8.3E-01
KIRP	SULT1A2_RI_1.2:1.3_1.1_1.4	SULT1A2	RI	1.2:1.3	1.1	1.4	5.1E-05	0.122	-0.467	6.5E-01	5.5E-01	8.5E-01
KIRP	SUPT20H_AA_21.1_20_21.2	SUPT20H	AA	21.1	20	21.2	1.6E-12	0.297	0.164	3.2E-02	2.2E-02	1.2E-01
KIRP	SUPT20H_AA_21.1:21.2_20_21.3	SUPT20H	AA	21.1:21.2	20	21.3	2.9E-06	0.150	0.387	7.5E-01	1.1E-01	3.8E-01
KIRP	SUPT20H_AA_21.2_20_21.3	SUPT20H	AA	21.2	20	21.3	1.6E-05	0.132	0.058	4.7E-01	5.5E-02	2.1E-01
KIRP	SYNE1_ES_151_150_152	SYNE1	ES	151	150	152	5.4E-15	0.350	0.011	1.7E-02	3.2E-03	6.5E-05
KIRP	SYNE2_ES_118_117.2_119.2	SYNE2	ES	118	117.2	119.2	1.7E-04	0.105	-0.214	1.3E-03	5.0E-04	6.7E-03
KIRP	SYTL2_AD_8.3_8.2_9	SYTL2	AD	8.3	8.2	9	1.3E-04	0.106	0.123	1.3E-01	4.6E-02	3.6E-01
KIRP	TAF1D_RI_12.2_12.1_12.3	TAF1D	RI	12.2	12.1	12.3	1.9E-04	0.101	0.092	6.7E-01	6.5E-01	9.8E-01
KIRP	TAGLN_RI_1.2_1.1_1.3	TAGLN	RI	1.2	1.1	1.3	7.2E-05	0.131	-0.559	1.1E-03	3.3E-04	4.4E-03
KIRP	TANGO2_ES_7.2:8_6_9	TANGO2	ES	7.2:8	6	9	1.9E-05	0.130	0.131	4.9E-01	1.0E-01	6.6E-03
KIRP	TATDN2_AD_7.2_7.1_8	TATDN2	AD	7.2	7.1	8	2.2E-05	0.127	0.039	3.3E-03	1.9E-02	2.8E-02
KIRP	TAZ_AD_3.2:3.3_3.1_4	TAZ	AD	3.2:3.3	3.1	4	1.6E-05	0.132	0.512	4.6E-01	3.7E-01	6.2E-02
KIRP	TAZ_ES_5_4_6	TAZ	ES	5	4	6	8.8E-05	0.110	0.113	1.6E-01	3.2E-02	9.4E-02
KIRP	TBC1D15_ES_10_9_11	TBC1D15	ES	10	9	11	6.7E-13	0.327	0.010	4.3E-01	6.1E-02	1.0E-03
KIRP	TBC1D7_AD_1.2_1.1_2.2	TBC1D7	AD	1.2	1.1	2.2	3.1E-04	0.101	-0.068	7.0E-01	6.9E-01	5.7E-01
KIRP	TBC1D7_AD_1.2:1.3_1.1_2.2	TBC1D7	AD	1.2:1.3	1.1	2.2	1.2E-04	0.112	-0.021	3.3E-01	3.5E-01	3.0E-01
KIRP	TBL1X_ES_6_5_7	TBL1X	ES	6	5	7	2.7E-04	0.119	-0.210	6.4E-01	8.9E-01	1.5E-01
KIRP	TCEA3_ES_2.2_1_3	TCEA3	ES	2.2	1	3	1.0E-10	0.285	0.063	1.2E-01	6.1E-01	9.1E-01
KIRP	TCEB1_AD_1.2_1.1_6	TCEB1	AD	1.2	1.1	6	1.8E-07	0.180	0.024	2.6E-02	4.0E-04	7.3E-03
KIRP	TCEB1_ES_1.2:5_1.1_6	TCEB1	ES	1.2:5	1.1	6	3.5E-06	0.151	-0.074	3.8E-01	6.1E-03	9.7E-02
KIRP	TCERG1_ES_22_21_23	TCERG1	ES	22	21	23	1.8E-04	0.103	0.279	5.7E-02	1.2E-01	1.2E-01
KIRP	TCF20_ES_4.1:4.2_3_5	TCF20	ES	4.1:4.2	3	5	1.9E-05	0.128	0.034	3.0E-02	1.3E-02	1.7E-01
KIRP	TCF20_ES_4.2_3_5	TCF20	ES	4.2	3	5	5.1E-05	0.117	-0.057	6.3E-02	5.8E-03	9.4E-02
KIRP	THBS3_AD_7.2_7.1_8	THBS3	AD	7.2	7.1	8	1.9E-05	0.134	-0.283	9.0E-03	5.6E-03	9.9E-02
KIRP	THUMPD2_ES_4_3_5	THUMPD2	ES	4	3	5	9.9E-06	0.168	0.227	1.2E-01	1.4E-01	6.4E-01
KIRP	TIA1_ES_5:6_4_7	TIA1	ES	5:06	4	7	5.3E-06	0.151	0.378	2.7E-02	9.0E-02	5.3E-02
KIRP	TIA1_ES_6_5_7	TIA1	ES	6	5	7	5.0E-05	0.121	0.520	4.3E-01	5.7E-01	1.6E-01
KIRP	TIMMDC1_ES_1.2:2_1.1_3	TIMMDC1	ES	1.2:2	1.1	3	1.3E-04	0.121	-0.055	6.6E-02	7.0E-03	4.9E-02
KIRP	TJAP1_AA_6.1_5_6.2	TJAP1	AA	6.1	5	6.2	2.9E-04	0.112	0.077	7.5E-01	4.6E-01	1.6E-01
KIRP	TJAP1_AA_6.1:6.2_5_6.3	TJAP1	AA	6.1:6.2	5	6.3	1.6E-04	0.126	0.100	9.6E-01	7.3E-01	4.7E-02
KIRP	TJP1_AD_30.2_30.1_31	TJP1	AD	30.2	30.1	31	1.0E-05	0.135	0.081	2.4E-02	3.3E-03	3.1E-02

cancer type	id	Gene Symbol	splice_type	Exon	From.Exon	To.Exon	anova.p	adj.r2	r	p.50	p.25	p.10
KIRP	TKT_RI_16.2_16.1_16.3	TKT	RI	16.2	16.1	16.3	3.8E-15	0.348	-0.341	2.3E-01	3.3E-02	3.8E-02
KIRP	TLE2_ES_10.2:10.3:10.4:10.5:11_10.1_1	TLE2	ES	.2:10.3:10.4:10.5	10.1	12	1.6E-11	0.318	0.015	9.3E-01	2.9E-01	4.0E-02
KIRP	TLE2_RI_10.2:10.3_10.1_10.4	TLE2	RI	10.2:10.3	10.1	10.4	9.3E-04	0.100	0.308	4.2E-01	5.4E-01	9.8E-01
KIRP	TMEM126B_ES_3_1_4	TMEM126B	ES	3	1	4	9.1E-06	0.137	-0.041	3.3E-02	7.6E-02	7.4E-01
KIRP	TMEM139_AD_2.2:2.3:2.4_2.1_5	TMEM139	AD	2.2:2.3:2.4	2.1	5	1.7E-04	0.123	0.435	3.3E-01	3.2E-01	4.9E-01
KIRP	TMEM139_RI_2.3_2.2_2.4	TMEM139	RI	2.3	2.2	2.4	1.7E-04	0.102	0.210	9.7E-01	8.4E-01	6.5E-01
KIRP	TMEM175_ES_4.1:4.2_3_5.1	TMEM175	ES	4.1:4.2	3	5.1	9.6E-05	0.110	0.494	9.3E-01	9.0E-01	3.7E-01
KIRP	TMEM176A_AD_1.2_1.1_2.2	TMEM176A	AD	1.2	1.1	2.2	1.4E-04	0.105	0.176	6.3E-02	1.5E-01	9.1E-02
KIRP	TMEM180_ES_6_5_7	TMEM180	ES	6	5	7	1.1E-09	0.234	-0.153	4.2E-01	4.3E-01	5.2E-01
KIRP	TMEM205_AD_2.2:2.3_2.1_2.6	TMEM205	AD	2.2:2.3	2.1	2.6	9.4E-09	0.213	-0.154	2.4E-01	9.4E-02	1.4E-02
KIRP	TMEM205_ES_2.2:2.3:2.5_2.1_2.6	TMEM205	ES	2.2:2.3:2.5	2.1	2.6	3.7E-06	0.149	0.006	2.2E-01	3.9E-02	5.4E-03
KIRP	TMEM205_ES_2.2:2.5_2.1_2.6	TMEM205	ES	2.2:2.5	2.1	2.6	1.3E-10	0.255	-0.033	8.5E-01	6.6E-02	1.0E-02
KIRP	TMEM205_RI_2.2:2.3:2.4_2.1_2.5	TMEM205	RI	2.2:2.3:2.4	2.1	2.5	1.4E-07	0.183	-0.065	6.6E-01	5.0E-01	5.5E-01
KIRP	TMEM205_RI_2.2:2.3:2.4:2.5_2.1_2.6	TMEM205	RI	2.2:2.3:2.4:2.5	2.1	2.6	2.0E-06	0.154	-0.175	6.2E-01	7.1E-01	3.4E-01
KIRP	TMEM205_RI_2.3:2.4_2.2_2.5	TMEM205	RI	2.3:2.4	2.2	2.5	9.9E-06	0.136	-0.187	3.0E-01	1.1E-01	8.9E-02
KIRP	TMEM205_RI_2.3:2.4:2.5_2.2_2.6	TMEM205	RI	2.3:2.4:2.5	2.2	2.6	6.0E-05	0.115	-0.233	1.0E+00	6.3E-01	6.6E-01
KIRP	TMEM205_RI_2.4_2.3_2.5	TMEM205	RI	2.4	2.3	2.5	1.4E-05	0.131	-0.094	1.3E-01	1.7E-01	5.2E-04
KIRP	TMEM205_RI_2.4:2.5_2.3_2.6	TMEM205	RI	2.4:2.5	2.3	2.6	6.6E-09	0.214	-0.050	9.2E-02	8.6E-02	7.3E-02
KIRP	TMEM251_AD_1.2_1.1_2	TMEM251	AD	1.2	1.1	2	1.1E-11	0.277	0.189	4.4E-03	2.9E-02	2.0E-03
KIRP	TMEM91_AA_7.4_7.1_7.5	TMEM91	AA	7.4	7.1	7.5	1.8E-04	0.155	0.159	4.4E-02	1.3E-01	1.7E-01
KIRP	TMEM91_RI_7.2:7.3:7.4_7.1_7.5	TMEM91	RI	7.2:7.3:7.4	7.1	7.5	2.4E-08	0.201	-0.282	4.9E-01	5.5E-01	7.6E-01
KIRP	TMPO_ES_6:7:8_5.1_9	TMPO	ES	6:07:08	5.1	9	7.0E-06	0.142	0.036	2.5E-02	1.5E-02	6.9E-02
KIRP	TMUB1_RI_4.2_4.1_4.3	TMUB1	RI	4.2	4.1	4.3	1.4E-13	0.318	0.109	4.0E-02	7.4E-03	1.4E-03
KIRP	TMX2_ES_3.2:3.3_2_4	TMX2	ES	3.2:3.3	2	4	2.2E-05	0.129	-0.037	2.1E-02	1.1E-01	1.5E-01
KIRP	TNFRSF14_AA_7.1_6_7.2	TNFRSF14	AA	7.1	6	7.2	7.0E-05	0.113	0.294	6.8E-02	2.7E-01	5.5E-01
KIRP	TNFRSF25_AA_6.1:6.2:6.3:6.4_5_6.5	TNFRSF25	AA	6.1:6.2:6.3:6.4	5	6.5	8.3E-04	0.121	-0.032	5.2E-01	4.3E-01	4.4E-01
KIRP	TOP3B_ES_6_5_7	TOP3B	ES	6	5	7	1.4E-04	0.113	0.589	8.7E-02	4.3E-02	1.9E-01
KIRP	TOR2A_AD_3.2:3.3_3.1_3.5	TOR2A	AD	3.2:3.3	3.1	3.5	1.7E-04	0.108	-0.074	2.6E-01	3.8E-02	4.5E-04
KIRP	TOR2A_RI_3.2_3.1_3.3	TOR2A	RI	3.2	3.1	3.3	1.0E-05	0.135	-0.046	5.4E-01	9.8E-01	5.8E-01
KIRP	TPM1_AA_2.1_1_2.2	TPM1	AA	2.1	1	2.2	6.4E-05	0.118	0.158	9.7E-01	3.1E-01	3.2E-02
KIRP	TPM1_ES_8_7_9	TPM1	ES	8	7	9	6.3E-09	0.221	0.329	8.5E-04	6.8E-04	2.1E-02
KIRP	TPM2_ES_7_6_8	TPM2	ES	7	6	8	3.8E-07	0.225	-0.167	6.1E-02	4.8E-02	1.9E-01
KIRP	TRA2A_ES_3.1:3.2_1_4	TRA2A	ES	3.1:3.2	1	4	7.7E-05	0.113	0.755	5.4E-01	8.8E-01	9.8E-01
KIRP	TREX1_RI_2.5_2.4_2.6	TREX1	RI	2.5	2.4	2.6	1.5E-04	0.104	0.004	7.1E-01	1.5E-01	1.1E-01
KIRP	TREX1_RI_2.5:2.6:2.7:2.8_2.4_2.9	TREX1	RI	2.5:2.6:2.7:2.8	2.4	2.9	1.2E-04	0.128	0.073	3.5E-01	6.1E-01	8.7E-01
KIRP	TREX1_RI_2.5:2.6:2.7:2.8:2.9_2.4_2.1	TREX1	RI	2.5:2.6:2.7:2.8:2.	2.4	2.1	1.2E-04	0.108	-0.210	2.8E-01	1.7E-01	9.9E-01
KIRP	TRIQK_ES_5_4_6.1	TRIQK	ES	5	4	6.1	8.0E-05	0.128	0.079	1.6E-01	4.2E-01	6.7E-01
KIRP	TSNAXIP1_AD_1.2_1.1_2	TSNAXIP1	AD	1.2	1.1	2	1.2E-04	0.146	-0.374	8.6E-01	8.3E-01	2.0E-01
KIRP	TSTD1_ES_2.2:2.3_1_3.1	TSTD1	ES	2.2:2.3	1	3.1	1.8E-04	0.103	0.040	4.0E-01	7.3E-01	6.9E-01
KIRP	TTC14_RI_5.2:5.3_5.1_5.4	TTC14	RI	5.2:5.3	5.1	5.4	9.4E-07	0.164	0.491	5.3E-01	1.9E-01	6.7E-01

cancer type	id	Gene Symbol	splice_type	Exon	From.Exon	To.Exon	anova.p	adj.r2	r	p.50	p.25	p.10
KIRP	TTC14_RI_5.5_5.4_5.6	TTC14	RI	5.5	5.4	5.6	2.4E-06	0.152	0.545	8.8E-01	5.1E-01	6.8E-02
KIRP	TUSC3_ES_11_10_12	TUSC3	ES	11	10	12	2.5E-07	0.176	0.017	5.6E-02	5.8E-02	3.2E-01
KIRP	TXNDC9_ES_4_3_5.1	TXNDC9	ES	4	3	5.1	4.8E-07	0.169	-0.132	2.0E-01	2.5E-01	6.7E-03
KIRP	TXNL4A_ES_6:7.2_3_9	TXNL4A	ES	06:07.2	3	9	4.5E-05	0.139	-0.355	7.3E-01	7.5E-01	5.3E-01
KIRP	UAP1_ES_9.1:9.2_8_10	UAP1	ES	9.1:9.2	8	10	5.0E-09	0.218	-0.181	4.0E-01	6.8E-02	2.3E-01
KIRP	UAP1_ES_9.2_8_10	UAP1	ES	9.2	8	10	1.3E-08	0.225	-0.098	2.6E-01	1.4E-01	4.8E-01
KIRP	UBALD1_RI_2.4_2.3_2.5	UBALD1	RI	2.4	2.3	2.5	1.1E-04	0.108	0.057	1.9E-01	4.2E-01	5.3E-01
KIRP	UBE2D3_AD_2.4_2.3_3.3	UBE2D3	AD	2.4	2.3	3.3	1.5E-05	0.131	-0.303	3.9E-01	4.4E-02	7.7E-02
KIRP	UBP1_ES_8_7_9	UBP1	ES	8	7	9	1.4E-04	0.106	-0.127	9.1E-01	2.7E-02	3.8E-02
KIRP	UHRF2_ES_11_10_12	UHRF2	ES	11	10	12	2.5E-04	0.113	0.216	9.2E-03	1.4E-01	8.7E-01
KIRP	ULK3_RI_6.2_6.1_6.3	ULK3	RI	6.2	6.1	6.3	8.2E-06	0.139	0.615	5.7E-01	8.7E-01	8.0E-01
KIRP	UNC119_RI_4.2_4.1_4.3	UNC119	RI	4.2	4.1	4.3	3.4E-06	0.148	0.244	9.1E-01	3.0E-01	6.4E-02
KIRP	UPF3B_ES_8_7_9	UPF3B	ES	8	7	9	3.2E-09	0.236	0.026	8.6E-03	1.2E-02	1.0E-02
KIRP	UQCRB_RI_6.2:6.3_6.1_6.4	UQCRB	RI	6.2:6.3	6.1	6.4	1.2E-06	0.162	-0.084	4.4E-01	1.1E-01	1.9E-01
KIRP	USHBP1_AA_7.1:7.2_6_7.3	USHBP1	AA	7.1:7.2	6	7.3	5.2E-06	0.145	-0.423	4.8E-01	7.5E-01	7.9E-01
KIRP	USP33_AA_15.1_14_15.2	USP33	AA	15.1	14	15.2	1.5E-09	0.249	0.069	1.0E-01	6.6E-03	2.9E-03
KIRP	VAMP2_RI_5.2_5.1_5.3	VAMP2	RI	5.2	5.1	5.3	7.0E-08	0.190	-0.050	5.3E-01	5.6E-01	7.8E-01
KIRP	VAMP4_RI_8.2_8.1_8.3	VAMP4	RI	8.2	8.1	8.3	4.8E-06	0.144	0.164	5.2E-01	6.1E-02	1.3E-02
KIRP	VEPH1_ES_6:7_5_8	VEPH1	ES	6:07	5	8	3.7E-04	0.115	-0.270	6.6E-01	4.2E-01	2.4E-03
KIRP	VWA9_AD_1.2:1.3_1.1_2.1	VWA9	AD	1.2:1.3	1.1	2.1	3.6E-05	0.142	-0.120	1.4E-02	1.8E-02	3.1E-03
KIRP	WDFY3_ES_45_44_46	WDFY3	ES	45	44	46	6.6E-05	0.135	0.092	6.7E-01	1.3E-01	9.9E-01
KIRP	WDR27_ES_26_25_27	WDR27	ES	26	25	27	1.2E-04	0.117	-0.194	6.8E-01	2.4E-01	3.0E-01
KIRP	WDR55_RI_2.2_2.1_2.3	WDR55	RI	2.2	2.1	2.3	7.0E-05	0.121	-0.436	4.0E-01	3.4E-01	3.1E-01
KIRP	WDR62_RI_25.6_25.5_25.7	WDR62	RI	25.6	25.5	25.7	1.9E-07	0.179	-0.368	1.5E-01	3.2E-03	1.2E-05
KIRP	WDR62_RI_25.6:25.7_25.5_25.8	WDR62	RI	25.6:25.7	25.5	25.8	6.1E-05	0.115	-0.275	4.4E-02	9.5E-02	3.5E-01
KIRP	WIPI2_ES_2_1_3	WIPI2	ES	2	1	3	3.7E-06	0.152	-0.034	8.7E-03	4.3E-03	2.6E-03
KIRP	XRRA1_ES_18.1_17_18.3	XRRA1	ES	18.1	17	18.3	7.0E-05	0.122	0.055	5.2E-01	9.5E-02	2.5E-02
KIRP	YAP1_AD_6.2_6.1_8	YAP1	AD	6.2	6.1	8	2.6E-04	0.117	-0.036	1.5E-01	7.7E-03	4.1E-02
KIRP	YAP1_ES_7_6.1_8	YAP1	ES	7	6.1	8	2.5E-07	0.185	0.029	9.7E-01	6.5E-01	5.2E-01
KIRP	YBEY_AD_1.2_1.1_2.1	YBEY	AD	1.2	1.1	2.1	2.7E-05	0.124	-0.096	7.9E-01	4.9E-01	3.4E-01
KIRP	YBEY_ES_3_2.2_4	YBEY	ES	3	2.2	4	1.7E-07	0.181	-0.260	8.6E-02	5.7E-02	1.2E-01
KIRP	YDJC_AD_2.2_2.1_3	YDJC	AD	2.2	2.1	3	6.9E-06	0.140	0.022	7.4E-02	2.0E-01	1.2E-01
KIRP	YIPF1_ES_12_11_13	YIPF1	ES	12	11	13	1.8E-04	0.102	0.003	7.1E-02	6.7E-02	6.2E-01
KIRP	YPEL5_ES_3.1:3.2_1_5	YPEL5	ES	3.1:3.2	1	5	5.2E-22	0.474	0.070	1.4E-02	3.0E-04	7.9E-03
KIRP	ZDHHC4_AD_1.2:1.3_1.1_2.2	ZDHHC4	AD	1.2:1.3	1.1	2.2	2.2E-05	0.128	-0.254	1.3E-02	1.8E-02	1.0E-02
KIRP	ZDHHC7_ES_4_3_5	ZDHHC7	ES	4	3	5	2.2E-05	0.132	-0.016	3.1E-02	2.1E-02	8.3E-02
KIRP	ZFAND2B_RI_4.10_4.9_4.11	ZFAND2B	RI	4.1	4.9	4.11	1.1E-04	0.107	0.238	2.9E-01	5.3E-01	8.7E-01
KIRP	ZFC3H1_RI_32.2_32.1_32.3	ZFC3H1	RI	32.2	32.1	32.3	4.1E-06	0.147	0.490	7.2E-01	2.4E-01	7.2E-01
KIRP	ZMYM2_RI_4.2_4.1_4.3	ZMYM2	RI	4.2	4.1	4.3	1.5E-07	0.196	0.197	2.0E-01	3.6E-02	1.8E-02
KIRP	ZNF174_RI_2.2_2.1_2.3	ZNF174	RI	2.2	2.1	2.3	2.7E-04	0.112	-0.163	4.2E-01	3.6E-01	3.2E-01

cancer type	id	Gene Symbol	splice_type	Exon	From.Exon	To.Exon	anova.p	adj.r2	r	p.50	p.25	p.10
KIRP	ZNF302_AD_1.2:1.3_1.1_2	ZNF302	AD	1.2:1.3	1.1	2	6.4E-04	0.116	0.187	7.4E-01	1.0E+00	4.3E-01
KIRP	ZNF384_ES_8_7_9	ZNF384	ES	8	7	9	1.3E-04	0.106	0.069	1.3E-02	7.8E-02	3.8E-02
KIRP	ZNF446_AD_1.2_1.1_2	ZNF446	AD	1.2	1.1	2	6.5E-11	0.266	-0.079	3.8E-01	4.0E-01	6.9E-02
KIRP	ZNF563_RI_4.2_4.1_4.3	ZNF563	RI	4.2	4.1	4.3	7.1E-06	0.143	-0.015	2.7E-01	8.6E-02	1.2E-01
KIRP	ZNF584_ES_4.2_3_5	ZNF584	ES	4.2	3	5	4.5E-04	0.123	-0.017	5.1E-01	8.8E-01	8.6E-01
KIRP	ZNF621_ES_3_2_4	ZNF621	ES	3	2	4	3.5E-04	0.105	-0.056	7.8E-01	8.5E-01	3.3E-01
KIRP	ZNF655_ES_4:5.1:5.2:5.3_3.2_7	ZNF655	ES	4:5.1:5.2:5.3	3.2	7	5.3E-05	0.147	0.450	9.6E-01	8.4E-01	5.8E-01
KIRP	ZNF667_ES_9_8_12	ZNF667	ES	9	8	12	2.6E-05	0.147	-0.671	1.6E-02	2.6E-01	8.7E-01
LAML	ABI1_ES_5_4_7	ABI1	ES	5	4	7	1.3E-04	0.116	0.091	2.7E-01	6.3E-02	3.1E-02
LAML	ACBD5_ES_6_5_7	ACBD5	ES	6	5	7	5.5E-05	0.143	0.036	9.1E-01	5.2E-01	9.5E-01
LAML	ACSM3_ES_5_4_6	ACSM3	ES	5	4	6	1.7E-06	0.173	-0.692	9.6E-01	6.5E-01	8.7E-01
LAML	ADD3_ES_15_14_16	ADD3	ES	15	14	16	1.7E-13	0.319	-0.163	6.6E-01	6.4E-01	1.4E-01
LAML	AGTRAP_ES_4.2_3_5	AGTRAP	ES	4.2	3	5	1.7E-04	0.132	0.060	8.2E-02	2.2E-01	4.7E-02
LAML	AHDC1_ES_3:4.1_2_4.2	AHDC1	ES	03:04.1	2	4.2	1.5E-04	0.135	-0.314	8.6E-01	8.8E-01	7.6E-01
LAML	AKAP13_ES_12_11_14	AKAP13	ES	12	11	14	1.8E-04	0.144	0.390	2.3E-01	2.4E-01	2.6E-01
LAML	ANAPC5_RI_8.2_8.1_8.3	ANAPC5	RI	8.2	8.1	8.3	2.7E-07	0.185	-0.305	1.1E-01	7.4E-02	2.2E-02
LAML	ANKRD10_ES_4_3_6.1	ANKRD10	ES	4	3	6.1	4.3E-04	0.102	-0.034	4.6E-03	6.2E-04	1.9E-03
LAML	ANKRD13D_ES_2_1_3.1	ANKRD13D	ES	2	1	3.1	2.0E-06	0.165	0.118	5.5E-02	4.8E-01	8.0E-01
LAML	AP2M1_ES_7_6_8	AP2M1	ES	7	6	8	1.0E-05	0.146	0.013	7.6E-01	2.6E-01	5.4E-01
LAML	ARFIP1_ES_4_3_5	ARFIP1	ES	4	3	5	1.4E-04	0.137	0.205	8.4E-01	8.1E-01	3.1E-01
LAML	ARHGEF6_ES_8_7_9	ARHGEF6	ES	8	7	9	1.4E-06	0.167	-0.565	1.5E-02	5.8E-01	3.9E-01
LAML	ARHGEF7_RI_2.2_2.1_2.3	ARHGEF7	RI	2.2	2.1	2.3	3.2E-04	0.117	0.256	8.4E-01	5.6E-01	3.2E-01
LAML	ARL8B_ES_2_1_3.1	ARL8B	ES	2	1	3.1	4.4E-04	0.102	0.223	1.0E-02	5.5E-03	5.2E-02
LAML	ARMC5_RI_5.2_5.1_5.3	ARMC5	RI	5.2	5.1	5.3	1.5E-05	0.142	-0.382	3.3E-01	2.2E-01	4.6E-01
LAML	ARPC1B_ES_8_7_9	ARPC1B	ES	8	7	9	3.2E-13	0.313	-0.752	6.8E-03	5.7E-03	2.7E-01
LAML	ARRB2_ES_2_1_4.2	ARRB2	ES	2	1	4.2	6.6E-06	0.151	-0.289	5.4E-01	7.4E-01	7.2E-01
LAML	ARRB2_ES_2:3_1_4.2	ARRB2	ES	2:03	1	4.2	5.0E-04	0.100	-0.252	8.9E-01	2.5E-01	2.0E-01
LAML	ATG16L2_AD_6.4_6.3_7	ATG16L2	AD	6.4	6.3	7	9.5E-05	0.120	-0.030	4.0E-01	8.4E-01	6.7E-01
LAML	ATP5J_AD_1.4:1.5_1.3_3	ATP5J	AD	1.4:1.5	1.3	3	2.2E-11	0.276	-0.013	6.2E-01	3.4E-01	9.4E-01
LAML	BABAM1_AA_2.1_1.2_2.2	BABAM1	AA	2.1	1.2	2.2	4.2E-04	0.102	-0.056	4.4E-02	1.8E-01	5.8E-01
LAML	BCAS4_ES_4_3_5	BCAS4	ES	4	3	5	2.0E-05	0.152	-0.078	2.2E-04	5.2E-02	2.3E-02
LAML	BCKDK_RI_13.2_13.1_13.3	BCKDK	RI	13.2	13.1	13.3	3.4E-04	0.105	-0.142	6.9E-01	4.2E-01	3.7E-01
LAML	BCL7B_ES_2_1_3	BCL7B	ES	2	1	3	2.7E-04	0.108	-0.539	1.6E-01	4.9E-02	8.9E-01
LAML	BEX4_ES_2_1_3	BEX4	ES	2	1	3	2.7E-04	0.115	-0.018	9.1E-01	9.1E-01	2.5E-01
LAML	BLOC1S1_RI_4.2_4.1_4.3	BLOC1S1	RI	4.2	4.1	4.3	8.4E-05	0.122	-0.422	8.1E-02	6.8E-01	4.0E-01
LAML	BRCC3_ES_8_7_9	BRCC3	ES	8	7	9	5.8E-04	0.104	-0.205	5.8E-01	7.3E-01	8.4E-01
LAML	BSDC1_RI_11.2_11.1_11.3	BSDC1	RI	11.2	11.1	11.3	7.7E-05	0.123	-0.003	8.5E-01	1.4E-01	7.9E-01
LAML	BUD31_AD_1.2_1.1_2	BUD31	AD	1.2	1.1	2	4.8E-05	0.128	-0.226	4.1E-01	9.2E-01	6.3E-01
LAML	C11orf30_ES_7.1:7.2_6_8	C11orf30	ES	7.1:7.2	6	8	3.7E-04	0.120	0.285	8.1E-01	6.8E-01	8.7E-01
LAML	C14orf159_ES_4:5.2:6_2_7	C14orf159	ES	05:02.2	2	7	4.9E-04	0.104	-0.388	9.6E-01	7.5E-01	8.3E-01

cancer type	id	Gene Symbol	splice_type	Exon	From.Exon	To.Exon	anova.p	adj.r2	r	p.50	p.25	p.10
LAML	C16orf58_RI_8.3_8.2_8.4	C16orf58	RI	8.3	8.2	8.4	2.2E-04	0.110	0.329	6.9E-01	8.7E-01	2.2E-01
LAML	C21orf58_AA_6.1_5_6.2	C21orf58	AA	6.1	5	6.2	1.0E-04	0.123	0.186	3.6E-01	2.4E-01	6.4E-01
LAML	C2orf68_RI_1.2_1.1_1.3	C2orf68	RI	1.2	1.1	1.3	1.2E-05	0.164	0.174	9.4E-01	7.4E-01	7.8E-01
LAML	C6orf203_AD_1.2_1.1_3	C6orf203	AD	1.2	1.1	3	2.9E-04	0.115	-0.366	7.5E-02	3.9E-02	1.9E-01
LAML	CA5B_ES_4:5:6:7:8.1_3_8.2	CA5B	ES	4:5:6:7:8.1	3	8.2	5.6E-04	0.106	0.650	3.1E-02	1.9E-01	1.4E-01
LAML	CAAP1_AD_1.2_1.1_2	CAAP1	AD	1.2	1.1	2	4.2E-04	0.109	-0.230	1.3E-01	1.2E-01	5.0E-01
LAML	CALCOCO2_ES_3_2_6	CALCOCO2	ES	3	2	6	2.4E-04	0.109	-0.505	4.6E-01	5.1E-01	8.0E-01
LAML	CARD16_ES_4_3_5	CARD16	ES	4	3	5	1.1E-04	0.119	0.228	4.4E-01	8.2E-02	6.1E-01
LAML	CARD8_ES_7.2_5_8	CARD8	ES	7.2	5	8	2.0E-05	0.141	0.015	1.4E-01	4.3E-02	2.8E-01
LAML	CCAR1_ES_5:6.1:6.2:7:8:9:10:11:12:13_3	CCAR1	ES	:6.2:7:8:9:10:11:	3	14	2.9E-04	0.127	0.013	1.0E-01	2.1E-01	2.3E-01
LAML	CCDC14_AD_2.9_2.8_3	CCDC14	AD	2.9	2.8	3	8.5E-05	0.122	0.189	4.4E-01	8.0E-01	1.6E-01
LAML	CCDC53_ES_5_2_6	CCDC53	ES	5	2	6	2.1E-04	0.111	-0.407	1.2E-02	7.3E-02	3.7E-01
LAML	CCDC53_ME_4|5_2_6	CCDC53	ME	4|5	2	6	2.2E-04	0.110	0.066	4.5E-01	7.4E-01	8.7E-01
LAML	CCDC84_AD_6.2_6.1_7	CCDC84	AD	6.2	6.1	7	3.8E-07	0.189	-0.027	1.0E-04	1.6E-02	3.0E-02
LAML	CCND3_AD_3.2_3.1_4	CCND3	AD	3.2	3.1	4	3.1E-04	0.106	-0.353	6.4E-02	5.8E-02	9.5E-01
LAML	CCNL1_AD_4.2:4.3_4.1_5	CCNL1	AD	4.2:4.3	4.1	5	1.9E-04	0.112	0.121	4.7E-02	1.7E-01	4.4E-01
LAML	CCT3_ES_3_1_4	CCT3	ES	3	1	4	2.6E-04	0.108	-0.558	2.3E-01	1.2E-01	2.4E-02
LAML	CD160_RI_5.2_5.1_5.3	CD160	RI	5.2	5.1	5.3	1.3E-04	0.116	-0.104	7.8E-01	8.3E-01	9.7E-01
LAML	CD320_AA_2.1_1_2.2	CD320	AA	2.1	1	2.2	2.2E-04	0.112	-0.562	1.1E-01	5.8E-01	5.9E-01
LAML	CD46_ES_13_12_14	CD46	ES	13	12	14	8.7E-06	0.148	-0.169	8.0E-02	7.4E-01	2.9E-01
LAML	CD46_ES_7_6_8	CD46	ES	7	6	8	3.0E-04	0.108	-0.101	5.6E-02	8.9E-02	9.9E-01
LAML	CD7_RI_2.2_2.1_2.3	CD7	RI	2.2	2.1	2.3	4.0E-04	0.116	-0.393	4.9E-01	2.5E-01	1.0E-01
LAML	CD74_ES_6:7.1:8:9_4_10.2	CD74	ES	6:7.1:8:9	4	10.2	6.6E-04	0.106	-0.036	4.5E-01	7.2E-02	6.6E-01
LAML	CDC37_RI_4.2_4.1_4.3	CDC37	RI	4.2	4.1	4.3	7.3E-05	0.123	0.363	1.4E-01	2.9E-01	3.5E-01
LAML	CDC42SE2_RI_5.2_5.1_5.3	CDC42SE2	RI	5.2	5.1	5.3	2.6E-04	0.108	0.060	6.6E-01	9.9E-01	2.4E-01
LAML	CDCA5_RI_5.2_5.1_5.3	CDCA5	RI	5.2	5.1	5.3	9.2E-06	0.158	-0.204	8.3E-01	1.5E-01	6.9E-01
LAML	CELF2_RI_16.2_16.1_16.3	CELF2	RI	16.2	16.1	16.3	1.2E-04	0.117	0.260	4.1E-01	5.5E-01	5.5E-01
LAML	CEP68_AD_3.3_3.2_4.1	CEP68	AD	3.3	3.2	4.1	2.1E-06	0.163	-0.363	9.6E-01	9.2E-01	5.4E-01
LAML	CFLAR_ES_10_8.1_11	CFLAR	ES	10	8.1	11	2.7E-06	0.161	-0.484	1.2E-01	3.8E-02	2.7E-01
LAML	CHID1_ES_6.1:6.2:6.3_5.2_7	CHID1	ES	6.1:6.2:6.3	5.2	7	3.5E-05	0.132	-0.615	4.4E-01	8.7E-01	6.9E-01
LAML	CHORDC1_AA_9.2:9.3_8.3_9.4	CHORDC1	AA	9.2:9.3	8.3	9.4	7.1E-05	0.126	-0.262	4.0E-04	9.6E-04	7.2E-02
LAML	CIITA_ES_7.1:7.2_6_8	CIITA	ES	7.1:7.2	6	8	2.1E-04	0.117	0.187	6.8E-02	1.0E-01	5.8E-01
LAML	CISD2_ES_3_1_4	CISD2	ES	3	1	4	4.0E-07	0.181	-0.053	1.1E-01	2.7E-02	6.3E-02
LAML	CKLF_ES_2_1_3.2	CKLF	ES	2	1	3.2	2.9E-05	0.134	0.015	1.3E-02	4.5E-02	2.6E-02
LAML	CLIP1_AD_24.3_24.2_25	CLIP1	AD	24.3	24.2	25	4.4E-08	0.204	-0.333	4.2E-02	1.1E-02	3.0E-01
LAML	CLN3_ES_17_15_18	CLN3	ES	17	15	18	6.1E-05	0.125	-0.530	8.1E-04	3.5E-02	4.1E-01
LAML	COL24A1_ES_25_24_26	COL24A1	ES	25	24	26	4.0E-06	0.161	-0.698	8.9E-02	1.9E-02	6.6E-02
LAML	COMMD1_ES_2_1_3	COMMD1	ES	2	1	3	1.1E-04	0.118	-0.243	3.5E-01	2.5E-02	7.9E-03
LAML	COPS7A_ES_3:4_2.4_6	COPS7A	ES	3:04	2.4	6	2.4E-04	0.109	-0.452	2.1E-01	1.4E-01	2.5E-01
LAML	CORO1B_AD_5.6:5.7_5.5_6	CORO1B	AD	5.6:5.7	5.5	6	4.7E-04	0.101	-0.453	2.7E-01	8.4E-01	6.8E-01

cancer type	id	Gene Symbol	splice_type	Exon	From.Exon	To.Exon	anova.p	adj.r2	r	p.50	p.25	p.10
LAML	CSF2RA_ES_13_12_14	CSF2RA	ES	13	12	14	6.7E-12	0.287	-0.779	1.2E-01	6.7E-02	4.8E-01
LAML	CTSH_AA_8.1_7.2_8.2	CTSH	AA	8.1	7.2	8.2	2.0E-06	0.180	-0.613	4.1E-01	6.1E-01	7.4E-01
LAML	CYB561A3_RI_6.2_6.1_6.3	CYB561A3	RI	6.2	6.1	6.3	6.9E-07	0.175	-0.350	1.2E-01	3.3E-01	1.4E-01
LAML	DDX17_ES_11.2:12.2:12.3_11.1_12.4	DDX17	ES	11.2:12.2:12.3	11.1	12.4	1.3E-04	0.116	0.634	6.0E-01	4.0E-01	2.4E-01
LAML	DDX3X_ES_6.2:7:8:10:11:12:13:14:15:16	DDX3X	ES	8:10:11:12:13:14	6.1	18	9.9E-04	0.118	0.349	2.3E-02	6.0E-03	4.6E-03
LAML	DDX41_AA_7.1_6_7.2	DDX41	AA	7.1	6	7.2	9.8E-06	0.146	0.078	8.5E-02	1.5E-01	7.8E-02
LAML	DDX49_AA_10.1_9_10.2	DDX49	AA	10.1	9	10.2	5.3E-04	0.102	-0.103	1.9E-01	3.1E-02	4.5E-02
LAML	DEF8_AA_6.1_5_6.2	DEF8	AA	6.1	5	6.2	4.2E-10	0.306	0.255	4.8E-01	7.5E-01	1.7E-02
LAML	DEPDC5_AA_28.1_27.1_28.2	DEPDC5	AA	28.1	27.1	28.2	1.5E-04	0.127	-0.020	1.1E-01	6.2E-02	2.4E-01
LAML	DIAPH2_ES_2_1_3	DIAPH2	ES	2	1	3	6.9E-06	0.174	-0.106	3.2E-01	5.9E-02	3.9E-01
LAML	DNAJA3_ES_2_1_3	DNAJA3	ES	2	1	3	3.8E-05	0.131	-0.381	3.8E-02	5.6E-02	2.3E-02
LAML	DNM1L_ES_4_2_5	DNM1L	ES	4	2	5	5.6E-05	0.128	0.101	2.4E-02	2.9E-01	1.3E-01
LAML	DOCK8_ES_2_1_3	DOCK8	ES	2	1	3	3.6E-11	0.272	-0.444	5.3E-01	2.1E-01	1.0E-02
LAML	DPP9_ES_4_3_6	DPP9	ES	4	3	6	2.2E-04	0.110	-0.571	8.6E-03	8.1E-02	5.2E-01
LAML	DPY19L2_ES_8_7_9	DPY19L2	ES	8	7	9	9.7E-04	0.107	-0.434	2.2E-02	4.1E-02	1.5E-01
LAML	DYNC2LI1_ES_4_3_5	DYNC2LI1	ES	4	3	5	2.3E-05	0.138	-0.479	1.9E-01	6.5E-01	2.8E-01
LAML	DYNLT1_RI_3.2_3.1_3.3	DYNLT1	RI	3.2	3.1	3.3	3.5E-06	0.158	-0.186	1.1E-02	6.8E-02	5.0E-01
LAML	EIF2B1_RI_5.2_5.1_5.3	EIF2B1	RI	5.2	5.1	5.3	7.9E-05	0.122	-0.187	1.4E-03	8.7E-03	3.5E-02
LAML	EIF4G1_RI_2.3_2.2_2.4	EIF4G1	RI	2.3	2.2	2.4	3.5E-04	0.105	-0.307	2.4E-01	3.6E-01	4.6E-01
LAML	ELP5_RI_7.2_7.1_7.3	ELP5	RI	7.2	7.1	7.3	5.0E-04	0.100	0.005	9.4E-01	7.1E-01	5.7E-02
LAML	ELP5_RI_7.4_7.3_7.5	ELP5	RI	7.4	7.3	7.5	2.2E-04	0.110	0.072	7.3E-01	5.3E-01	3.8E-01
LAML	EMC9_AA_3.1:3.2_2_3.3	EMC9	AA	3.1:3.2	2	3.3	6.0E-04	0.114	-0.407	6.8E-01	2.9E-01	1.4E-01
LAML	ENG_RI_14.2_14.1_14.3	ENG	RI	14.2	14.1	14.3	2.0E-05	0.141	-0.576	2.3E-01	1.7E-01	7.0E-02
LAML	EPB41_ES_18_15_19.1	EPB41	ES	18	15	19.1	1.5E-15	0.359	0.518	4.0E-01	1.4E-01	2.0E-01
LAML	EPHB2_RI_17.2_17.1_17.3	EPHB2	RI	17.2	17.1	17.3	6.6E-07	0.176	-0.676	4.7E-01	4.7E-01	5.2E-01
LAML	ERBB2IP_ES_22:24.1:24.2:24.3_21_25	ERBB2IP	ES	22:24.1:24.2:24.3	21	25	1.3E-04	0.144	-0.135	3.1E-01	2.2E-01	2.8E-01
LAML	ERBB2IP_ES_24.1:24.2:24.3_21_25	ERBB2IP	ES	24.1:24.2:24.3	21	25	5.5E-06	0.153	-0.242	7.4E-01	4.5E-01	3.6E-01
LAML	ERLIN1_RI_1.2_1.1_1.3	ERLIN1	RI	1.2	1.1	1.3	8.7E-05	0.121	0.321	1.3E-01	7.7E-01	6.4E-01
LAML	FAM129C_AA_14.1_13_14.2	FAM129C	AA	14.1	13	14.2	1.6E-05	0.141	-0.822	5.8E-01	8.0E-01	5.4E-02
LAML	FAM133B_ES_3_1_4	FAM133B	ES	3	1	4	1.7E-05	0.147	0.150	2.2E-02	4.4E-02	4.7E-02
LAML	FAM193A_ES_22_21_23	FAM193A	ES	22	21	23	1.4E-04	0.116	0.146	3.7E-01	1.1E-01	1.6E-01
LAML	FAM210A_ES_2_1_3	FAM210A	ES	2	1	3	3.2E-04	0.122	-0.033	4.9E-01	5.9E-01	7.2E-01
LAML	FAM89B_AA_2.1:2.2_1_2.3	FAM89B	AA	2.1:2.2	1	2.3	2.5E-04	0.112	-0.087	5.7E-01	9.9E-01	4.7E-01
LAML	FAM91A1_ES_14_13_15	FAM91A1	ES	14	13	15	2.2E-05	0.149	0.577	8.3E-02	1.7E-02	5.1E-02
LAML	FBXO38_ES_15.1:15.2_14_16	FBXO38	ES	15.1:15.2	14	16	4.1E-06	0.156	0.044	4.5E-01	2.8E-01	1.9E-01
LAML	FHL2_ES_4_3.2_5.1	FHL2	ES	4	3.2	5.1	9.1E-07	0.190	-0.672	5.5E-02	5.5E-02	3.5E-04
LAML	FLNA_ES_30_29_31	FLNA	ES	30	29	31	8.8E-11	0.264	-0.314	5.0E-01	9.7E-01	6.2E-01
LAML	FLNB_ES_32.1:32.2_31_33	FLNB	ES	32.1:32.2	31	33	4.2E-04	0.118	0.107	1.9E-01	2.6E-02	7.0E-02
LAML	FUBP1_AA_21.1_20_21.2	FUBP1	AA	21.1	20	21.2	5.5E-05	0.126	0.431	9.8E-02	2.9E-02	3.4E-02
LAML	FXR1_ES_2_1_5	FXR1	ES	2	1	5	2.2E-04	0.111	0.026	6.9E-03	3.6E-02	9.0E-03

cancer type	id	Gene Symbol	splice_type	Exon	From.Exon	To.Exon	anova.p	adj.r2	r	p.50	p.25	p.10
LAML	FYB_ES_13_12_14	FYB	ES	13	12	14	1.0E-14	0.342	0.308	2.0E-01	1.5E-01	4.6E-02
LAML	GIT2_AA_18.1_15_18.2	GIT2	AA	18.1	15	18.2	5.2E-04	0.126	-0.311	3.6E-01	7.9E-01	7.8E-01
LAML	GK_ES_23_22_24	GK	ES	23	22	24	5.5E-05	0.126	-0.263	6.8E-02	8.1E-02	2.9E-02
LAML	GMFB_RI_5.3_5.2_5.4	GMFB	RI	5.3	5.2	5.4	4.5E-05	0.130	-0.020	7.4E-01	2.4E-01	2.0E-01
LAML	GMFG_ES_3.1:3.2_2_4	GMFG	ES	3.1:3.2	2	4	4.3E-08	0.204	-0.175	2.1E-01	4.7E-01	1.9E-01
LAML	GPBAR1_AA_3.1:3.2_2_3.3	GPBAR1	AA	3.1:3.2	2	3.3	6.9E-08	0.255	-0.686	6.5E-01	6.0E-01	3.1E-01
LAML	GPR114_AD_1.2_1.1_2	GPR114	AD	1.2	1.1	2	6.8E-05	0.144	-0.114	6.3E-01	7.0E-01	4.4E-01
LAML	GPR132_ES_2_1_3	GPR132	ES	2	1	3	3.7E-05	0.145	-0.530	3.7E-01	6.3E-01	5.2E-01
LAML	GPR18_ES_3_2_4	GPR18	ES	3	2	4	1.5E-05	0.151	-0.413	5.5E-01	3.3E-01	5.4E-01
LAML	GPR56_ES_7.2_5.2_8.1	GPR56	ES	7.2	5.2	8.1	7.4E-04	0.112	0.837	1.4E-01	7.2E-02	2.2E-01
LAML	GUSB_ES_3.1:3.2_2_4.1	GUSB	ES	3.1:3.2	2	4.1	6.4E-05	0.125	0.673	6.1E-01	7.4E-01	9.8E-01
LAML	H2AFY_AA_6.1_5_6.2	H2AFY	AA	6.1	5	6.2	4.6E-06	0.155	0.555	1.7E-02	6.5E-03	4.7E-02
LAML	H2AFY_AA_6.1:6.2_5_6.3	H2AFY	AA	6.1:6.2	5	6.3	5.9E-06	0.152	0.536	1.0E-02	1.4E-02	3.3E-02
LAML	HAUS1_ES_3_2.1_4	HAUS1	ES	3	2.1	4	3.6E-04	0.104	-0.525	1.5E-01	4.9E-02	3.4E-02
LAML	HBP1_AD_1.2_1.1_4	HBP1	AD	1.2	1.1	4	2.8E-04	0.110	-0.035	7.0E-01	4.7E-01	9.7E-01
LAML	HERC2_ES_5:6:7:8:9:10:11_3_25	HERC2	ES	5:6:7:8:9:10:11	3	25	1.7E-05	0.159	0.172	5.0E-02	4.1E-02	5.3E-03
LAML	HNRNPC_ES_2.4:2.5:2.6_1_3.2	HNRNPC	ES	2.4:2.5:2.6	1	3.2	4.9E-04	0.100	-0.093	9.8E-02	1.1E-03	5.6E-02
LAML	HNRNPDL_ES_8_7_9	HNRNPDL	ES	8	7	9	2.0E-04	0.111	-0.288	8.8E-01	7.3E-01	8.1E-01
LAML	HNRNPH1_ES_3:5:6:7:8:9_2_10.1	HNRNPH1	ES	3:5:6:7:8:9	2	10.1	3.8E-04	0.104	0.429	9.6E-01	6.0E-01	6.0E-01
LAML	HPS4_RI_10.2_10.1_10.3	HPS4	RI	10.2	10.1	10.3	3.0E-04	0.107	0.075	1.4E-01	5.9E-02	5.6E-03
LAML	HSPB1_RI_1.2:1.3_1.1_1.4	HSPB1	RI	1.2:1.3	1.1	1.4	6.8E-05	0.125	-0.619	2.1E-01	4.3E-01	2.1E-01
LAML	IDE_ES_22_21_23	IDE	ES	22	21	23	3.1E-04	0.114	-0.238	1.2E-01	2.4E-02	3.7E-01
LAML	IDH3A_ES_3_2_4.1	IDH3A	ES	3	2	4.1	1.4E-04	0.116	-0.168	1.9E-01	2.6E-01	5.9E-01
LAML	IDH3G_RI_11.4_11.3_11.5	IDH3G	RI	11.4	11.3	11.5	1.6E-04	0.114	0.092	8.2E-01	1.4E-01	2.6E-01
LAML	ILK_RI_5.4_5.3_5.5	ILK	RI	5.4	5.3	5.5	5.7E-05	0.126	-0.369	4.5E-02	3.0E-02	4.7E-02
LAML	IMPA2_AD_6.2_6.1_7	IMPA2	AD	6.2	6.1	7	4.5E-08	0.205	-0.301	5.2E-04	7.0E-03	1.1E-03
LAML	ING3_RI_4.2_4.1_4.3	ING3	RI	4.2	4.1	4.3	5.0E-04	0.101	-0.073	8.2E-01	4.9E-01	2.2E-01
LAML	INO80C_ES_3:4.1:4.2_1_5.1	INO80C	ES	3:4.1:4.2	1	5.1	3.2E-04	0.106	-0.450	5.2E-01	6.4E-01	6.2E-01
LAML	IPO11_ES_30_29_33	IPO11	ES	30	29	33	2.3E-06	0.184	0.134	2.3E-01	2.0E-01	3.0E-02
LAML	IRAK4_ES_4:5_3_6	IRAK4	ES	4:05	3	6	3.9E-04	0.105	0.157	6.8E-01	8.1E-01	2.6E-01
LAML	IRF5_ES_5_4_6.1	IRF5	ES	5	4	6.1	6.8E-12	0.287	-0.771	8.3E-02	3.3E-01	5.2E-01
LAML	ISCU_RI_5.2_5.1_5.3	ISCU	RI	5.2	5.1	5.3	7.6E-05	0.126	-0.109	1.7E-01	1.2E-01	1.8E-01
LAML	KCTD7_ES_13_12_14	KCTD7	ES	13	12	14	5.7E-05	0.126	-0.298	1.4E-02	4.6E-02	2.4E-01
LAML	KDELR2_ES_2_1_3	KDELR2	ES	2	1	3	2.7E-05	0.135	-0.166	1.2E-01	1.5E-03	2.2E-03
LAML	KDM5B_ES_6_5_7	KDM5B	ES	6	5	7	2.2E-05	0.137	-0.548	1.8E-01	4.6E-01	1.1E-01
LAML	KLF7_ES_4_3.3_6.1	KLF7	ES	4	3.3	6.1	1.7E-04	0.120	-0.154	3.8E-03	3.1E-02	8.5E-02
LAML	KPNA1_ES_6_5_7	KPNA1	ES	6	5	7	2.8E-04	0.108	0.005	7.3E-02	4.0E-02	4.2E-02
LAML	KTN1_ES_35_34.2_36	KTN1	ES	35	34.2	36	2.0E-04	0.112	-0.193	7.3E-01	2.4E-01	5.6E-01
LAML	KTN1_ES_42_41_43	KTN1	ES	42	41	43	1.0E-05	0.146	-0.216	6.2E-01	6.8E-01	7.5E-02
LAML	LAMTOR5_AD_1.2:1.3_1.1_2.2	LAMTOR5	AD	1.2:1.3	1.1	2.2	4.5E-04	0.101	-0.085	7.6E-01	1.2E-01	6.3E-01

cancer type	id	Gene Symbol	splice_type	Exon	From.Exon	To.Exon	anova.p	adj.r2	r	p.50	p.25	p.10
LAML	LAS1L_AD_11.2_11.1_12	LAS1L	AD	11.2	11.1	12	7.2E-05	0.123	-0.174	2.3E-01	6.7E-01	4.7E-01
LAML	LAS1L_ES_9_8_10	LAS1L	ES	9	8	10	2.3E-05	0.140	0.072	9.4E-01	9.8E-01	1.7E-01
LAML	LBH_ES_4_3.1_5	LBH	ES	4	3.1	5	1.7E-04	0.113	-0.533	9.7E-01	5.3E-01	4.8E-01
LAML	LDHA_RI_2.2_2.1_2.3	LDHA	RI	2.2	2.1	2.3	1.9E-05	0.143	0.312	6.4E-01	9.7E-01	6.3E-01
LAML	LPCAT3_RI_10.2_10.1_10.3	LPCAT3	RI	10.2	10.1	10.3	3.0E-05	0.134	-0.698	8.8E-02	4.0E-02	5.8E-02
LAML	LPPR3_RI_6.2_6.1_6.3	LPPR3	RI	6.2	6.1	6.3	7.2E-04	0.112	-0.585	7.8E-01	9.1E-01	9.6E-01
LAML	LPXN_ES_6_5_7	LPXN	ES	6	5	7	2.0E-04	0.112	-0.502	1.2E-01	4.8E-02	6.9E-01
LAML	LRCH3_ES_15_14_16	LRCH3	ES	15	14	16	1.1E-05	0.146	0.127	6.8E-01	4.9E-01	4.8E-01
LAML	LRTOMT_RI_6.2_6.1_6.3	LRTOMT	RI	6.2	6.1	6.3	4.7E-05	0.130	0.322	3.3E-01	2.9E-01	3.8E-01
LAML	LTB4R_AD_2.2_2.1_3	LTB4R	AD	2.2	2.1	3	9.0E-04	0.101	-0.151	3.0E-01	7.1E-01	5.7E-02
LAML	LUC7L3_ES_4.1:4.2_3_5	LUC7L3	ES	4.1:4.2	3	5	4.8E-05	0.130	-0.440	8.7E-01	3.5E-01	5.8E-01
LAML	LYPLA2_RI_8.5_8.4_8.6	LYPLA2	RI	8.5	8.4	8.6	5.3E-05	0.128	-0.027	7.4E-02	1.9E-02	4.2E-03
LAML	MAP4K1_ES_31_30_32	MAP4K1	ES	31	30	32	2.9E-07	0.184	-0.614	1.6E-02	9.7E-04	7.0E-02
LAML	MARCH8_ES_2_1_4	MARCH8	ES	2	1	4	3.3E-05	0.133	-0.417	4.2E-01	9.4E-01	1.4E-01
LAML	MARCH8_ES_7_6_8	MARCH8	ES	7	6	8	5.4E-10	0.247	0.005	4.0E-01	7.0E-02	1.9E-01
LAML	MBNL1_ES_8_7_9	MBNL1	ES	8	7	9	1.4E-08	0.216	-0.199	4.5E-01	7.2E-01	6.7E-01
LAML	MCCC2_ES_11.2:12.1_11.1_12.2	MCCC2	ES	11.2:12.1	11.1	12.2	1.4E-04	0.122	-0.271	4.7E-01	2.3E-01	5.3E-01
LAML	MCM7_RI_5.2_5.1_5.3	MCM7	RI	5.2	5.1	5.3	3.5E-04	0.104	-0.089	1.8E-02	4.0E-01	6.8E-01
LAML	MEF2C_AA_14.1:14.2_13_14.3	MEF2C	AA	14.1:14.2	13	14.3	7.2E-05	0.128	-0.112	8.2E-02	8.1E-01	3.3E-01
LAML	MEFV_ES_6_5_7	MEFV	ES	6	5	7	1.9E-15	0.355	-0.801	2.1E-02	1.2E-01	2.1E-02
LAML	METTL3_RI_8.2_8.1_8.3	METTL3	RI	8.2	8.1	8.3	3.3E-05	0.132	0.366	2.6E-01	5.4E-01	1.9E-01
LAML	MFF_ES_9:10_7_11	MFF	ES	9:10	7	11	4.9E-06	0.154	0.097	6.2E-02	1.4E-01	9.7E-04
LAML	MGAM_ES_39:40:41:42:43:44:45:46:47:	MGAM	ES	:48:49:50:51:52:!	38	62	1.4E-05	0.162	-0.315	6.7E-01	6.3E-01	9.4E-01
LAML	MIB2_AD_5.3:5.4_5.2_6	MIB2	AD	5.3:5.4	5.2	6	2.7E-04	0.115	-0.265	3.9E-01	8.7E-01	7.9E-01
LAML	MIB2_RI_12.2_12.1_12.3	MIB2	RI	12.2	12.1	12.3	5.4E-05	0.155	-0.003	1.4E-01	1.1E-01	4.7E-01
LAML	MLC1_AD_1.2_1.1_2	MLC1	AD	1.2	1.1	2	1.8E-08	0.223	0.291	7.9E-03	2.5E-01	8.6E-01
LAML	MMAB_ES_5_4_6	MMAB	ES	5	4	6	3.4E-05	0.132	-0.471	2.0E-04	3.0E-02	2.2E-01
LAML	MND1_ES_5:6_4_7	MND1	ES	5:06	4	7	4.5E-05	0.129	-0.543	2.5E-01	7.2E-01	7.8E-01
LAML	MPST_ES_4_3_5	MPST	ES	4	3	5	6.0E-05	0.125	-0.634	4.2E-01	6.2E-02	3.4E-02
LAML	MRPL27_RI_5.2:5.3:5.4:5.5_5.1_5.6	MRPL27	RI	5.2:5.3:5.4:5.5	5.1	5.6	7.0E-05	0.124	-0.012	3.7E-01	2.4E-02	1.2E-01
LAML	MS4A7_ES_3_2_4	MS4A7	ES	3	2	4	3.6E-04	0.129	-0.169	6.3E-01	4.6E-01	7.4E-02
LAML	MSRB3_ES_2:3_1_4	MSRB3	ES	2:03	1	4	1.1E-07	0.212	-0.028	7.3E-02	1.2E-01	8.6E-01
LAML	MSRB3_ES_3_1_4	MSRB3	ES	3	1	4	8.4E-06	0.186	-0.035	4.5E-01	6.1E-01	2.2E-01
LAML	MYL5_AD_5.2_5.1_6	MYL5	AD	5.2	5.1	6	3.0E-04	0.132	0.101	2.3E-01	6.1E-01	8.6E-01
LAML	MZB1_AA_2.1:2.2_1_2.3	MZB1	AA	2.1:2.2	1	2.3	5.0E-05	0.143	0.044	4.8E-01	4.3E-01	2.6E-01
LAML	NARFL_AD_1.6_1.5_2.4	NARFL	AD	1.6	1.5	2.4	9.4E-04	0.109	0.028	1.6E-01	1.5E-01	6.8E-01
LAML	NBPF3_AA_12.1_11_12.2	NBPF3	AA	12.1	11	12.2	7.2E-05	0.130	-0.654	3.6E-01	2.6E-01	2.0E-01
LAML	NCOR2_AD_46.2_46.1_47	NCOR2	AD	46.2	46.1	47	6.9E-05	0.124	-0.019	3.8E-01	1.2E-01	3.3E-01
LAML	NDUFAF6_ES_12_11_14	NDUFAF6	ES	12	11	14	4.9E-04	0.103	0.110	3.9E-01	3.3E-01	9.6E-01
LAML	NDUFS7_ES_3_2.3_4	NDUFS7	ES	3	2.3	4	2.9E-06	0.160	-0.542	1.2E-01	2.2E-03	4.5E-02

cancer type	id	Gene Symbol	splice_type	Exon	From.Exon	To.Exon	anova.p	adj.r2	r	p.50	p.25	p.10
LAML	NEIL1_ES_5:6.1_4_6.2	NEIL1	ES	05:06.1	4	6.2	3.9E-04	0.135	-0.416	4.2E-01	5.0E-01	1.3E-01
LAML	NFE2_RI_3.2_3.1_3.3	NFE2	RI	3.2	3.1	3.3	1.7E-04	0.132	-0.044	6.8E-01	4.6E-01	2.1E-01
LAML	NFS1_ES_7_5_8	NFS1	ES	7	5	8	3.5E-04	0.105	-0.351	3.1E-01	2.2E-01	2.0E-01
LAML	NIN_ES_18_17_19	NIN	ES	18	17	19	2.6E-04	0.108	0.076	3.2E-01	3.1E-01	6.1E-01
LAML	NMT2_RI_8.2_8.1_8.3	NMT2	RI	8.2	8.1	8.3	5.0E-05	0.165	-0.670	6.3E-03	2.4E-03	1.9E-01
LAML	NOL8_AD_1.5_1.4_2.2	NOL8	AD	1.5	1.4	2.2	6.2E-04	0.103	0.247	4.9E-01	4.1E-01	4.2E-01
LAML	NOL8_AD_1.6_1.5_2.2	NOL8	AD	1.6	1.5	2.2	4.1E-04	0.108	-0.430	1.5E-01	7.9E-01	9.7E-01
LAML	NPEPPS_AD_9.2_9.1_10	NPEPPS	AD	9.2	9.1	10	5.0E-06	0.154	0.145	5.3E-03	2.9E-02	8.6E-02
LAML	NPRL3_ES_3_2_4	NPRL3	ES	3	2	4	1.9E-07	0.189	-0.406	9.7E-01	6.0E-01	6.0E-01
LAML	NR4A2_AA_9.1:9.2_8_9.3	NR4A2	AA	9.1:9.2	8	9.3	5.7E-04	0.109	-0.158	1.4E-02	5.4E-01	5.4E-02
LAML	NSUN5_RI_9.2_9.1_9.3	NSUN5	RI	9.2	9.1	9.3	4.0E-05	0.130	-0.234	5.2E-02	3.8E-02	4.9E-01
LAML	NUP54_ES_2:3_1_5	NUP54	ES	2:03	1	5	3.8E-05	0.169	-0.004	5.6E-03	2.6E-01	7.1E-01
LAML	OCIAD1_AD_2.2:2.3_2.1_3	OCIAD1	AD	2.2:2.3	2.1	3	4.9E-04	0.100	0.037	8.2E-01	8.0E-01	8.3E-01
LAML	OPN3_ES_3_1_4	OPN3	ES	3	1	4	8.7E-04	0.118	0.436	6.4E-02	1.8E-01	5.3E-01
LAML	OSBPL9_ES_17_15_18	OSBPL9	ES	17	15	18	1.1E-04	0.119	-0.307	6.0E-01	2.8E-01	9.5E-01
LAML	PABPN1_RI_5.2_5.1_5.3	PABPN1	RI	5.2	5.1	5.3	2.8E-04	0.107	0.068	1.1E-01	3.0E-01	6.1E-01
LAML	PAPOLA_RI_9.2_9.1_9.3	PAPOLA	RI	9.2	9.1	9.3	7.7E-06	0.149	-0.372	4.6E-04	1.6E-02	1.3E-02
LAML	PAQR3_ME_2|3_1_4	PAQR3	ME	2|3	1	4	6.4E-04	0.100	-0.008	2.9E-01	2.5E-01	1.2E-01
LAML	PARL_AD_4.2_4.1_5	PARL	AD	4.2	4.1	5	2.1E-04	0.111	0.142	5.8E-01	7.0E-01	5.6E-01
LAML	PARP6_ES_20_19_21	PARP6	ES	20	19	21	1.1E-05	0.145	-0.368	5.3E-01	9.7E-01	4.6E-01
LAML	PDCD5_RI_5.2_5.1_5.3	PDCD5	RI	5.2	5.1	5.3	3.4E-05	0.132	0.187	9.2E-03	7.3E-02	3.2E-02
LAML	PDCD5_RI_5.4_5.3_5.5	PDCD5	RI	5.4	5.3	5.5	3.9E-05	0.130	0.155	8.3E-03	5.0E-05	3.4E-02
LAML	PDHB_AD_9.2_9.1_10	PDHB	AD	9.2	9.1	10	5.8E-10	0.246	-0.171	4.8E-04	7.0E-03	9.3E-02
LAML	PICALM_ES_19_18_20	PICALM	ES	19	18	20	3.6E-31	0.583	0.600	3.0E-02	8.7E-01	4.0E-01
LAML	PICALM_ES_19:20_18_21	PICALM	ES	19:20	18	21	4.9E-10	0.265	0.235	1.4E-01	3.1E-01	6.0E-01
LAML	PIGG_AD_9.2:9.3_9.1_10.1	PIGG	AD	9.2:9.3	9.1	10.1	5.8E-04	0.102	-0.256	3.7E-01	4.4E-01	6.2E-01
LAML	PKN2_RI_12.2_12.1_12.3	PKN2	RI	12.2	12.1	12.3	3.3E-04	0.107	0.008	8.1E-01	6.3E-01	2.1E-01
LAML	PLA2G15_ES_2_1_3	PLA2G15	ES	2	1	3	2.8E-05	0.136	-0.432	5.6E-03	8.0E-03	6.1E-01
LAML	PLEKHJ1_RI_5.2_5.1_5.3	PLEKHJ1	RI	5.2	5.1	5.3	4.8E-05	0.129	-0.080	2.8E-01	4.1E-01	5.6E-02
LAML	PLEKHM2_ES_7_6_8	PLEKHM2	ES	7	6	8	1.2E-04	0.118	0.021	5.1E-01	3.1E-01	2.8E-01
LAML	PLK1_AD_1.2_1.1_2	PLK1	AD	1.2	1.1	2	8.9E-05	0.155	-0.533	9.5E-01	8.7E-01	8.6E-01
LAML	PNISR_RI_9.2_9.1_9.3	PNISR	RI	9.2	9.1	9.3	1.1E-06	0.171	-0.074	6.2E-01	9.8E-01	7.5E-02
LAML	PPA1_ES_7_6_8	PPA1	ES	7	6	8	4.2E-13	0.311	-0.884	5.8E-02	9.4E-02	3.5E-02
LAML	PPIA_AD_2.2_2.1_3	PPIA	AD	2.2	2.1	3	2.5E-08	0.209	0.252	7.2E-02	6.2E-01	5.3E-01
LAML	PPIL3_AD_1.2_1.1_2.2	PPIL3	AD	1.2	1.1	2.2	2.0E-04	0.112	-0.381	6.7E-02	1.0E-01	1.3E-01
LAML	PPIP5K2_ES_28_27_30	PPIP5K2	ES	28	27	30	1.7E-04	0.114	0.017	7.9E-01	9.7E-01	1.7E-01
LAML	PPM1B_AA_6.1_5_6.2	PPM1B	AA	6.1	5	6.2	7.8E-05	0.122	0.325	1.8E-02	1.5E-01	5.2E-01
LAML	PPM1M_RI_10.2_10.1_10.3	PPM1M	RI	10.2	10.1	10.3	6.6E-09	0.223	-0.471	2.2E-01	1.3E-01	4.8E-02
LAML	PPP1CC_RI_5.2_5.1_5.3	PPP1CC	RI	5.2	5.1	5.3	3.3E-09	0.229	-0.484	6.4E-02	1.6E-02	1.0E-01
LAML	PPP1R27_RI_2.2_2.1_2.3	PPP1R27	RI	2.2	2.1	2.3	4.7E-05	0.134	-0.647	6.1E-01	4.2E-01	8.1E-01

cancer type	id	Gene Symbol	splice_type	Exon	From.Exon	To.Exon	anova.p	adj.r2	r	p.50	p.25	p.10
LAML	PQLC1_ES_4.1:4.2_2_5	PQLC1	ES	4.1:4.2	2	5	7.1E-10	0.244	-0.728	5.1E-02	2.6E-02	1.4E-01
LAML	PQLC3_ES_6:7_5_8	PQLC3	ES	6:07	5	8	7.6E-04	0.109	-0.382	5.8E-01	4.9E-01	4.5E-01
LAML	PQLC3_ES_7_5_8	PQLC3	ES	7	5	8	2.0E-04	0.112	-0.231	2.7E-01	1.4E-01	7.9E-01
LAML	PRKAB1_ES_3_1.3_4	PRKAB1	ES	3	1.3	4	1.5E-04	0.114	-0.475	8.2E-03	3.0E-02	2.2E-01
LAML	PRKAR1A_RI_3.3_3.2_3.4	PRKAR1A	RI	3.3	3.2	3.4	6.6E-07	0.177	-0.028	7.4E-01	2.2E-01	9.3E-01
LAML	PRMT2_ES_1.3_1.1_2	PRMT2	ES	1.3	1.1	2	6.0E-04	0.104	-0.010	8.3E-02	8.6E-01	1.7E-01
LAML	PRR13_AD_1.2_1.1_3	PRR13	AD	1.2	1.1	3	7.4E-08	0.199	-0.396	2.9E-01	6.6E-01	2.3E-01
LAML	PRR5_ES_2_1_3.2	PRR5	ES	2	1	3.2	3.0E-09	0.230	-0.556	2.2E-01	2.6E-01	1.6E-01
LAML	PRRC1_RI_8.2_8.1_8.3	PRRC1	RI	8.2	8.1	8.3	9.1E-05	0.121	0.337	6.5E-02	1.1E-01	3.7E-02
LAML	PRRC2C_ES_34_33_35	PRRC2C	ES	34	33	35	4.7E-04	0.101	-0.012	4.8E-03	1.2E-01	2.0E-01
LAML	PSTPIP1_ES_3_2.2_5	PSTPIP1	ES	3	2.2	5	4.1E-09	0.227	-0.840	4.2E-01	2.9E-01	2.8E-01
LAML	PTBP1_ES_9.1:9.2_8_10	PTBP1	ES	9.1:9.2	8	10	4.7E-04	0.101	-0.193	6.6E-01	4.6E-01	5.6E-01
LAML	PTOV1_RI_13.2:13.3_13.1_13.4	PTOV1	RI	13.2:13.3	13.1	13.4	3.0E-04	0.106	-0.438	8.9E-02	2.5E-01	5.5E-01
LAML	PTPN6_AA_4.1_3_4.2	PTPN6	AA	4.1	3	4.2	1.3E-04	0.116	-0.066	3.1E-02	1.1E-02	1.8E-03
LAML	PTPRC_ES_5:6:7_3.1_8	PTPRC	ES	5:06:07	3.1	8	3.8E-08	0.205	-0.298	4.2E-01	5.9E-01	1.6E-01
LAML	PTPRC_ES_7_6_8	PTPRC	ES	7	6	8	1.3E-10	0.260	-0.369	9.4E-02	2.6E-01	7.9E-01
LAML	PUM1_ES_2.2:3_2.1_4	PUM1	ES	2.2:3	2.1	4	4.1E-04	0.117	-0.093	1.1E-02	1.8E-02	6.8E-01
LAML	QKI_AA_8.5_8.2_8.6	QKI	AA	8.5	8.2	8.6	4.5E-16	0.373	0.473	7.6E-01	6.0E-01	4.6E-01
LAML	QKI_RI_8.3:8.4_8.2_8.5	QKI	RI	8.3:8.4	8.2	8.5	1.5E-06	0.168	-0.198	3.8E-01	4.5E-01	8.7E-01
LAML	QKI_RI_8.3:8.4:8.5_8.2_8.6	QKI	RI	8.3:8.4:8.5	8.2	8.6	3.4E-04	0.105	0.408	4.4E-03	9.0E-04	1.5E-01
LAML	RAB37_RI_12.2_12.1_12.3	RAB37	RI	12.2	12.1	12.3	6.9E-08	0.200	-0.094	9.7E-01	1.9E-01	7.8E-02
LAML	RAB43_ES_6_5_7	RAB43	ES	6	5	7	3.9E-08	0.207	-0.636	1.5E-01	6.6E-01	4.9E-01
LAML	RANBP1_RI_7.2_7.1_7.3	RANBP1	RI	7.2	7.1	7.3	4.5E-04	0.101	-0.214	4.0E-01	3.4E-01	1.3E-01
LAML	RANBP1_RI_7.2:7.3_7.1_7.4	RANBP1	RI	7.2:7.3	7.1	7.4	2.5E-05	0.136	-0.212	5.1E-01	7.2E-01	5.4E-01
LAML	RBM14_ES_2_1_4.1	RBM14	ES	2	1	4.1	1.0E-05	0.146	0.296	9.0E-03	2.0E-04	4.3E-03
LAML	RBM14_ES_3_1_4.1	RBM14	ES	3	1	4.1	1.8E-05	0.140	0.245	6.4E-03	2.0E-03	1.6E-02
LAML	RBMX_RI_8.2_8.1_8.3	RBMX	RI	8.2	8.1	8.3	1.7E-04	0.113	0.095	2.7E-01	1.5E-01	2.1E-01
LAML	RBP7_ES_2_1_3	RBP7	ES	2	1	3	4.2E-07	0.180	-0.617	7.7E-01	8.8E-01	6.7E-01
LAML	REEP5_ES_4_3_5	REEP5	ES	4	3	5	1.7E-09	0.236	-0.531	6.5E-01	7.9E-01	5.4E-01
LAML	REL_ES_9_8_10	REL	ES	9	8	10	1.8E-05	0.139	-0.597	2.5E-02	3.2E-03	5.9E-02
LAML	REPS1_AD_9.2:9.3_9.1_10	REPS1	AD	9.2:9.3	9.1	10	3.5E-07	0.182	0.185	9.8E-02	7.7E-02	3.7E-02
LAML	REPS1_ES_9.3_9.1_10	REPS1	ES	9.3	9.1	10	2.4E-13	0.315	0.303	5.4E-01	9.0E-01	7.3E-01
LAML	RFX5_ES_7_6.2_8	RFX5	ES	7	6.2	8	9.7E-05	0.120	-0.431	2.5E-01	1.1E-01	6.9E-01
LAML	RHOC_AA_2.1_1.1_2.2	RHOC	AA	2.1	1.1	2.2	4.5E-06	0.184	-0.253	5.6E-01	7.5E-01	3.3E-02
LAML	RIPK2_ES_2:3_1_4	RIPK2	ES	2:03	1	4	1.3E-04	0.133	-0.254	2.0E-02	2.1E-02	1.3E-02
LAML	RMDN1_ES_6_4_7	RMDN1	ES	6	4	7	1.2E-04	0.118	-0.241	3.6E-02	5.7E-02	2.2E-01
LAML	RNF10_ES_2_1_3	RNF10	ES	2	1	3	1.8E-07	0.189	-0.122	5.0E-02	8.2E-03	8.1E-02
LAML	RPS24_ES_5.2_4_6	RPS24	ES	5.2	4	6	1.5E-09	0.237	0.088	1.1E-01	1.6E-01	5.0E-01
LAML	RQCD1_ES_7_6_8	RQCD1	ES	7	6	8	4.0E-04	0.103	-0.683	1.2E-01	2.6E-02	1.2E-01
LAML	RREB1_ES_12_11_13	RREB1	ES	12	11	13	6.4E-06	0.152	-0.027	9.1E-01	1.7E-01	3.0E-01

cancer type	id	Gene Symbol	splice_type	Exon	From.Exon	To.Exon	anova.p	adj.r2	r	p.50	p.25	p.10
LAML	RRM2B_ES_2_1_3.1	RRM2B	ES	2	1	3.1	3.1E-04	0.107	-0.171	6.4E-01	9.0E-01	8.9E-01
LAML	SCP2_ES_12_11_13	SCP2	ES	12	11	13	5.2E-05	0.127	-0.609	3.6E-01	8.8E-01	4.5E-01
LAML	SDSL_ES_2_1_3	SDSL	ES	2	1	3	4.9E-06	0.154	-0.609	3.5E-03	2.4E-02	2.0E-02
LAML	SEC24C_RI_23.2_23.1_23.3	SEC24C	RI	23.2	23.1	23.3	6.4E-05	0.125	0.025	6.7E-02	2.8E-02	2.3E-01
LAML	SEC31A_ES_16:17_15_18	SEC31A	ES	16:17	15	18	2.0E-12	0.297	0.033	8.5E-01	4.6E-01	4.4E-02
LAML	SEPT2_ES_8_7_9	SEPT2	ES	8	7	9	1.2E-04	0.118	-0.489	2.3E-01	9.0E-02	9.7E-02
LAML	SEPT7_AD_1.2_1.1_4	SEPT7	AD	1.2	1.1	4	4.0E-06	0.160	-0.085	7.5E-01	4.1E-01	3.6E-02
LAML	SETMAR_AA_2.1:2.2_1_2.3	SETMAR	AA	2.1:2.2	1	2.3	4.8E-05	0.128	0.216	9.3E-01	1.4E-01	2.9E-01
LAML	SH3BP1_ES_16_15_17.2	SH3BP1	ES	16	15	17.2	4.3E-10	0.249	-0.821	9.6E-05	8.7E-03	8.0E-02
LAML	SH3BP5_ES_6:7_5.2_8	SH3BP5	ES	6:07	5.2	8	1.5E-06	0.167	-0.724	7.6E-02	2.0E-02	6.2E-02
LAML	SIDT2_RI_23.2_23.1_23.3	SIDT2	RI	23.2	23.1	23.3	4.2E-05	0.134	-0.443	6.7E-01	4.8E-01	7.8E-02
LAML	SIDT2_RI_23.4_23.3_23.5	SIDT2	RI	23.4	23.3	23.5	9.0E-05	0.121	-0.549	1.3E-01	1.6E-01	1.9E-01
LAML	SIDT2_RI_25.2_25.1_25.3	SIDT2	RI	25.2	25.1	25.3	3.2E-08	0.209	-0.568	5.5E-02	9.4E-03	8.2E-01
LAML	SKA2_ES_2:3_1.1_4.1	SKA2	ES	2:03	1.1	4.1	8.9E-04	0.101	-0.389	5.4E-02	3.9E-03	1.5E-02
LAML	SKA2_ES_3_2_4.1	SKA2	ES	3	2	4.1	1.7E-04	0.113	-0.469	7.3E-02	1.1E-01	5.1E-02
LAML	SKA2_ES_3:4.1_2_5	SKA2	ES	03:04.1	2	5	1.4E-04	0.116	-0.291	8.5E-02	5.1E-02	4.6E-02
LAML	SLC37A3_ES_4_3_5	SLC37A3	ES	4	3	5	7.2E-06	0.152	-0.660	8.8E-01	9.1E-01	7.4E-02
LAML	SMARCA2_ES_33_32_34	SMARCA2	ES	33	32	34	2.0E-05	0.138	-0.113	3.7E-01	8.8E-01	7.9E-01
LAML	SMARCD3_RI_11.2_11.1_11.3	SMARCD3	RI	11.2	11.1	11.3	5.9E-06	0.152	-0.589	8.0E-01	8.1E-01	7.7E-01
LAML	SMPD4_ES_12_11_14	SMPD4	ES	12	11	14	1.4E-04	0.145	-0.483	6.5E-01	9.2E-01	9.7E-01
LAML	SMPD4_ES_13_11_14	SMPD4	ES	13	11	14	7.2E-04	0.121	-0.418	1.5E-01	2.7E-01	3.6E-01
LAML	SNRNP70_ES_8.1:8.2:8.3_7_9	SNRNP70	ES	8.1:8.2:8.3	7	9	2.0E-04	0.111	0.771	2.8E-02	3.5E-01	3.7E-01
LAML	SNRPD2_ME_3|4_1.3_5.1	SNRPD2	ME	3|4	1.3	5.1	3.1E-04	0.106	-0.297	4.8E-01	3.6E-01	6.2E-01
LAML	SNX29_ES_5_4_6	SNX29	ES	5	4	6	2.1E-05	0.137	-0.356	9.6E-01	5.2E-01	2.3E-01
LAML	SORBS1_ES_14:16_13_17	SORBS1	ES	14:16	13	17	2.4E-05	0.137	-0.624	3.9E-01	2.5E-01	2.4E-01
LAML	SPAG7_RI_7.2_7.1_7.3	SPAG7	RI	7.2	7.1	7.3	5.2E-04	0.101	-0.026	1.7E-01	5.8E-01	5.6E-02
LAML	SREBF1_RI_3.3_3.2_3.4	SREBF1	RI	3.3	3.2	3.4	1.7E-04	0.117	-0.374	7.1E-02	2.4E-01	9.2E-02
LAML	SRSF1_RI_3.2_3.1_3.3	SRSF1	RI	3.2	3.1	3.3	1.4E-05	0.142	-0.109	1.0E-02	5.6E-02	4.1E-01
LAML	SRSF1_RI_3.6_3.5_3.7	SRSF1	RI	3.6	3.5	3.7	6.5E-09	0.223	0.368	3.9E-01	5.7E-01	5.9E-02
LAML	SRSF2_RI_2.2:2.3:2.4_2.1_2.5	SRSF2	RI	2.2:2.3:2.4	2.1	2.5	7.5E-06	0.149	0.180	7.3E-02	6.1E-02	5.0E-01
LAML	SSB_ES_6_5_7	SSB	ES	6	5	7	7.2E-05	0.123	-0.721	2.7E-01	7.0E-02	1.4E-01
LAML	SSBP3_ES_7_6_8	SSBP3	ES	7	6	8	2.8E-10	0.253	0.146	5.5E-01	2.8E-01	9.1E-01
LAML	STK16_ES_4_3.2_5	STK16	ES	4	3.2	5	1.4E-04	0.131	0.011	7.5E-01	6.0E-01	4.1E-01
LAML	STRA13_ES_3.1:3.2_2_4.1	STRA13	ES	3.1:3.2	2	4.1	7.1E-04	0.105	-0.083	5.2E-01	7.3E-01	4.4E-01
LAML	STX16_ES_3_1.4_5.1	STX16	ES	3	1.4	5.1	2.0E-04	0.111	0.100	6.7E-03	3.4E-02	2.0E-01
LAML	SULT1A1_ES_4_3_6.2	SULT1A1	ES	4	3	6.2	2.0E-04	0.147	-0.240	9.7E-01	8.4E-01	9.3E-01
LAML	SULT1A2_AA_5.1_4_5.2	SULT1A2	AA	5.1	4	5.2	2.4E-04	0.124	-0.062	1.4E-01	2.0E-01	2.1E-01
LAML	SUPT4H1_AD_2.3:2.4_2.2_3.2	SUPT4H1	AD	2.3:2.4	2.2	3.2	9.6E-07	0.172	-0.101	5.7E-03	5.7E-02	4.8E-02
LAML	SUPT4H1_AD_2.4_2.3_3.2	SUPT4H1	AD	2.4	2.3	3.2	1.8E-04	0.115	-0.052	1.9E-01	1.3E-01	1.5E-01
LAML	SYNRG_ES_7_6_8	SYNRG	ES	7	6	8	1.8E-06	0.167	-0.272	8.3E-01	7.0E-01	3.3E-01

cancer type	id	Gene Symbol	splice_type	Exon	From.Exon	To.Exon	anova.p	adj.r2	r	p.50	p.25	p.10
LAML	SZRD1_ES_2:3_1_4.1	SZRD1	ES	2:03	1	4.1	1.2E-04	0.118	-0.318	5.4E-01	4.2E-01	5.7E-02
LAML	TATDN1_AD_4.2_4.1_5	TATDN1	AD	4.2	4.1	5	4.8E-04	0.101	-0.055	9.8E-01	6.4E-01	3.2E-01
LAML	TBCB_ES_3_2.3_4	TBCB	ES	3	2.3	4	2.3E-04	0.110	-0.517	3.3E-03	7.3E-03	5.9E-02
LAML	TBRG1_ES_5_3_6	TBRG1	ES	5	3	6	1.1E-04	0.121	0.241	9.7E-01	6.2E-01	9.3E-01
LAML	TCEB1_AD_1.2_1.1_6	TCEB1	AD	1.2	1.1	6	2.6E-06	0.161	-0.079	5.7E-03	4.8E-02	2.1E-01
LAML	TERF1_ES_7_6_8	TERF1	ES	7	6	8	2.7E-04	0.108	0.129	1.4E-01	9.6E-01	6.3E-01
LAML	TET2_RI_4.2_4.1_4.3	TET2	RI	4.2	4.1	4.3	8.4E-05	0.122	-0.408	8.0E-01	9.6E-01	3.1E-01
LAML	TFDP1_AA_13.1_12_13.2	TFDP1	AA	13.1	12	13.2	7.5E-09	0.223	-0.358	9.4E-01	2.4E-01	1.7E-02
LAML	TFR2_ES_10_9_11	TFR2	ES	10	9	11	3.6E-11	0.272	-0.805	7.0E-01	7.1E-01	6.6E-01
LAML	TGOLN2_AA_4.1:4.2_3.1_4.3	TGOLN2	AA	4.1:4.2	3.1	4.3	1.8E-05	0.139	-0.208	1.7E-01	9.2E-02	6.8E-02
LAML	THUMPD1_ES_3_2_4	THUMPD1	ES	3	2	4	1.1E-05	0.149	0.609	4.4E-02	8.5E-03	1.7E-01
LAML	TMEM104_ME_3|4_2.2_5	TMEM104	ME	3|4	2.2	5	1.3E-11	0.281	-0.778	1.6E-02	6.2E-02	1.4E-01
LAML	TMEM161B_AD_11.5:11.6_11.4_12.1	TMEM161B	AD	11.5:11.6	11.4	12.1	5.1E-05	0.127	0.269	7.1E-02	4.0E-01	7.0E-02
LAML	TMEM205_RI_2.2:2.3:2.4:2.5_2.1_2.6	TMEM205	RI	2.2:2.3:2.4:2.5	2.1	2.6	1.1E-04	0.118	0.125	8.5E-02	3.3E-02	5.5E-02
LAML	TMEM67_ES_4_3.2_6	TMEM67	ES	4	3.2	6	1.9E-06	0.173	-0.533	7.2E-01	2.9E-01	8.6E-01
LAML	TMX4_ES_2_1_3	TMX4	ES	2	1	3	2.0E-04	0.112	-0.065	1.8E-01	4.7E-02	5.9E-02
LAML	TNRC6B_ES_11_10_12	TNRC6B	ES	11	10	12	8.9E-05	0.122	-0.211	5.6E-01	8.6E-01	7.2E-01
LAML	TOMM40_RI_8.2_8.1_8.3	TOMM40	RI	8.2	8.1	8.3	1.7E-05	0.140	-0.571	2.0E-02	4.6E-02	3.8E-03
LAML	TPX2_ES_11_10_12	TPX2	ES	11	10	12	7.4E-05	0.124	-0.722	9.6E-01	7.7E-01	9.2E-02
LAML	TRAPPC1_RI_1.2_1.1_1.3	TRAPPC1	RI	1.2	1.1	1.3	3.9E-08	0.205	0.014	5.3E-01	9.3E-02	2.5E-02
LAML	TRERF1_ES_8.1_7_9	TRERF1	ES	8.1	7	9	3.3E-04	0.123	-0.170	2.2E-01	8.3E-01	7.8E-01
LAML	TRIM13_RI_1.2:1.3_1.1_1.4	TRIM13	RI	1.2:1.3	1.1	1.4	2.5E-04	0.109	-0.131	9.0E-01	4.4E-01	9.0E-01
LAML	TRIP12_ES_3_2_4.1	TRIP12	ES	3	2	4.1	4.8E-05	0.128	0.135	3.3E-01	3.5E-01	8.1E-01
LAML	TRMT13_AA_6.1_5_6.2	TRMT13	AA	6.1	5	6.2	4.9E-04	0.106	-0.032	6.9E-01	5.1E-01	4.4E-01
LAML	TUBGCP2_ES_7_6_8	TUBGCP2	ES	7	6	8	4.7E-05	0.128	-0.540	6.0E-01	2.3E-01	3.0E-01
LAML	UBE2D3_AD_2.2:2.3:2.4_2.1_3.3	UBE2D3	AD	2.2:2.3:2.4	2.1	3.3	3.7E-04	0.104	-0.140	3.7E-01	2.2E-01	6.3E-01
LAML	USP54_ES_23_22_24	USP54	ES	23	22	24	3.4E-04	0.105	-0.436	8.5E-01	5.2E-01	2.2E-01
LAML	VAMP2_RI_5.2_5.1_5.3	VAMP2	RI	5.2	5.1	5.3	3.8E-05	0.131	-0.029	5.3E-03	3.3E-03	1.2E-02
LAML	VCL_ES_19_18.2_20	VCL	ES	19	18.2	20	2.7E-09	0.234	-0.640	7.5E-02	6.5E-02	4.0E-04
LAML	VPS29_ES_3.1:3.2_1_5	VPS29	ES	3.1:3.2	1	5	7.6E-05	0.123	-0.209	9.4E-02	5.9E-01	7.0E-01
LAML	VSTM1_ME_3|4_2_5	VSTM1	ME	3|4	2	5	3.0E-09	0.230	0.952	4.0E-02	3.6E-02	3.6E-02
LAML	WBP2_ES_3_2.1_4	WBP2	ES	3	2.1	4	7.0E-05	0.124	-0.376	2.1E-01	4.6E-03	9.3E-01
LAML	WDR24_ES_1.6:2:3_1.5_4	WDR24	ES	1.6:2:3	1.5	4	2.5E-04	0.118	-0.297	1.3E-02	3.7E-02	2.1E-01
LAML	WDR55_RI_2.2_2.1_2.3	WDR55	RI	2.2	2.1	2.3	4.5E-05	0.131	-0.061	1.0E-01	9.7E-02	5.8E-01
LAML	WHSC1_AA_16.1_15.2_16.2	WHSC1	AA	16.1	15.2	16.2	7.8E-06	0.149	0.188	2.9E-03	4.8E-03	2.3E-02
LAML	YWHAZ_ES_3.2_2_7.1	YWHAZ	ES	3.2	2	7.1	3.1E-06	0.160	0.185	6.2E-01	7.0E-01	3.8E-01
LAML	YY1AP1_RI_13.2_13.1_13.3	YY1AP1	RI	13.2	13.1	13.3	4.7E-04	0.101	-0.172	4.3E-08	1.1E-03	5.8E-02
LAML	ZBTB7B_ES_3:4_2.2_6.2	ZBTB7B	ES	3:04	2.2	6.2	9.6E-07	0.205	0.294	8.5E-01	9.7E-01	5.5E-01
LAML	ZBTB7B_ES_5_2.2_6.2	ZBTB7B	ES	5	2.2	6.2	5.9E-04	0.125	-0.320	3.5E-01	6.5E-01	7.4E-01
LAML	ZC3HC1_ES_4:6:7_1_8	ZC3HC1	ES	4:06:07	1	8	1.2E-04	0.137	-0.037	3.7E-01	3.7E-01	5.3E-01

cancer type	id	Gene Symbol	splice_type	Exon	From.Exon	To.Exon	anova.p	adj.r2	r	p.50	p.25	p.10
LAML	ZCWPW1_ES_16:17_15_18	ZCWPW1	ES	16:17	15	18	3.6E-04	0.129	-0.101	9.0E-01	4.7E-01	2.1E-01
LAML	ZDHHC16_ES_2_1_4	ZDHHC16	ES	2	1	4	7.4E-04	0.123	-0.378	5.7E-01	8.7E-01	3.2E-01
LAML	ZFAND2B_RI_4.8_4.7_4.9	ZFAND2B	RI	4.8	4.7	4.9	1.5E-04	0.115	0.365	7.0E-02	7.4E-02	1.6E-01
LAML	ZFAND5_ES_3_1_4.1	ZFAND5	ES	3	1	4.1	3.8E-04	0.103	-0.181	6.5E-01	9.3E-01	2.7E-01
LAML	ZFAT_ES_3_1_4	ZFAT	ES	3	1	4	3.3E-04	0.106	-0.087	2.5E-02	2.8E-01	3.9E-01
LAML	ZKSCAN1_ES_2_1_3.1	ZKSCAN1	ES	2	1	3.1	8.1E-05	0.122	-0.210	5.0E-01	2.5E-01	1.9E-01
LAML	ZNF121_ES_5_4_6	ZNF121	ES	5	4	6	4.3E-04	0.106	-0.248	9.6E-01	5.5E-01	7.1E-01
LAML	ZNF197_ES_3_2.3_4	ZNF197	ES	3	2.3	4	2.3E-05	0.164	0.308	2.7E-01	1.5E-01	2.4E-01
LAML	ZNF207_ES_10_9_11	ZNF207	ES	10	9	11	7.4E-07	0.175	0.278	1.3E-01	4.2E-02	1.7E-01
LAML	ZNF410_ES_14_13_16	ZNF410	ES	14	13	16	8.7E-07	0.173	-0.254	2.2E-01	1.4E-01	7.5E-01
LAML	ZNF511_RI_5.2_5.1_5.3	ZNF511	RI	5.2	5.1	5.3	4.3E-05	0.129	0.027	7.3E-02	1.4E-01	4.2E-01
LAML	ZNF544_ES_9:10.1_7.2_10.2	ZNF544	ES	09:10.1	7.2	10.2	3.7E-04	0.104	-0.132	9.2E-03	5.5E-02	4.0E-01
LAML	ZNF655_AA_5.1:5.2_4_5.3	ZNF655	AA	5.1:5.2	4	5.3	6.3E-08	0.207	0.312	2.2E-03	1.0E-02	3.1E-02
LAML	ZNF667_ES_9_8_12	ZNF667	ES	9	8	12	4.7E-08	0.203	-0.572	2.4E-01	3.9E-01	1.3E-01
LAML	ZNF76_RI_12.2_12.1_12.3	ZNF76	RI	12.2	12.1	12.3	4.7E-05	0.128	-0.369	8.0E-01	7.2E-01	3.7E-01
LAML	ZNF766_ES_7_6_8.1	ZNF766	ES	7	6	8.1	1.2E-04	0.117	-0.464	4.3E-01	1.7E-01	6.7E-01
LAML	ZNF793_RI_10.2:10.3_10.1_10.4	ZNF793	RI	10.2:10.3	10.1	10.4	2.7E-07	0.185	-0.589	4.6E-01	5.5E-01	1.1E-01
LGG	ABCA2_AA_4.1:4.2:4.3:4.4:4.5_3_4.6	ABCA2	AA	4.1:4.2:4.3:4.4:4.5	3	4.6	2.6E-48	0.376	0.155	1.3E-07	1.8E-08	3.1E-07
LGG	ABCA2_ES_4.1_3_4.6	ABCA2	ES	4.1	3	4.6	1.2E-12	0.119	0.535	2.6E-05	4.5E-05	2.2E-03
LGG	ABCC5_AA_7.3:7.4:7.5_7.1_7.6	ABCC5	AA	7.3:7.4:7.5	7.1	7.6	4.3E-27	0.247	0.114	4.2E-01	4.0E-01	9.2E-01
LGG	ABCC5_AA_7.4:7.5_7.1_7.6	ABCC5	AA	7.4:7.5	7.1	7.6	1.9E-18	0.166	-0.007	9.8E-03	1.3E-02	1.5E-01
LGG	ABCC5_AD_7.2:7.3:7.4_7.1_7.6	ABCC5	AD	7.2:7.3:7.4	7.1	7.6	6.5E-29	0.240	0.207	1.6E-01	1.6E-02	2.6E-02
LGG	ABCC5_RI_7.2:7.3:7.4:7.5_7.1_7.6	ABCC5	RI	7.2:7.3:7.4:7.5	7.1	7.6	1.4E-11	0.105	0.408	4.0E-02	8.2E-03	1.6E-02
LGG	ABCD4_ES_8.2_7_9	ABCD4	ES	8.2	7	9	3.1E-12	0.111	-0.067	1.6E-01	1.7E-02	4.4E-03
LGG	ABCD4_RI_18.2_18.1_18.3	ABCD4	RI	18.2	18.1	18.3	2.0E-42	0.329	0.014	4.3E-01	4.9E-01	5.9E-01
LGG	ABI1_ES_10_9_11.1	ABI1	ES	10	9	11.1	5.4E-11	0.101	0.190	3.3E-01	1.6E-01	3.9E-01
LGG	ABI1_ES_10_9_13	ABI1	ES	10	9	13	5.4E-18	0.172	0.365	3.6E-03	5.5E-03	2.4E-03
LGG	ABI1_ES_10:11.1_9_11.2	ABI1	ES	10:11.1	9	11.2	2.6E-14	0.129	0.164	6.1E-01	1.8E-01	1.7E-01
LGG	ABI1_ES_10:11.1:11.2_9_13	ABI1	ES	10:11.1:11.2	9	13	1.5E-25	0.219	0.393	3.7E-05	1.4E-04	9.1E-05
LGG	ABI1_ES_10:11.1:11.2:12_9_13	ABI1	ES	10:11.1:11.2:12	9	13	8.3E-20	0.174	0.353	6.6E-04	1.8E-04	1.1E-02
LGG	ABI1_ES_10:11.2_9_13	ABI1	ES	10:11.2	9	13	3.6E-20	0.196	0.413	1.3E-02	9.0E-05	5.5E-03
LGG	ABI1_ES_10:11.2:12_9_13	ABI1	ES	11:02.2	9	13	1.2E-16	0.169	0.361	1.1E-02	1.3E-03	3.7E-02
LGG	ABI1_ES_11.2_9_13	ABI1	ES	11.2	9	13	4.8E-29	0.241	0.307	4.1E-04	2.8E-04	9.6E-03
LGG	ABI1_ES_11.2:12_9_13	ABI1	ES	11.2:12	9	13	6.0E-28	0.234	0.285	9.4E-03	2.7E-03	9.2E-02
LGG	ABI2_ES_11_10_12	ABI2	ES	11	10	12	2.4E-11	0.103	-0.352	2.0E-04	5.6E-04	1.6E-02
LGG	ABI2_ES_13.1:13.2_12_14	ABI2	ES	13.1:13.2	12	14	8.0E-19	0.164	0.318	2.4E-05	1.0E-07	2.0E-05
LGG	ABI2_ES_5.3:8:10:11:12:13.1:13.2_5.2_1	ABI2	ES	8:10:11:12:13.1:1	5.2	14	6.9E-27	0.230	0.209	2.1E-04	5.4E-06	1.5E-05
LGG	ABI2_ES_5.3:8:10:12:13.1:13.2_5.2_14	ABI2	ES	3:8:10:12:13.1:13	5.2	14	7.1E-19	0.175	0.256	5.4E-05	1.9E-05	1.5E-05
LGG	ABI3BP_ES_34_33_35	ABI3BP	ES	34	33	35	1.1E-38	0.367	0.061	2.0E-04	3.9E-08	5.8E-07
LGG	ABLIM1_AA_18.1_17_18.2	ABLIM1	AA	18.1	17	18.2	1.8E-10	0.115	0.254	1.9E-02	2.0E-04	2.8E-03

cancer type	id	Gene Symbol	splice_type	Exon	From.Exon	To.Exon	anova.p	adj.r2	r	p.50	p.25	p.10
LGG	ABLIM1_ES_16_15_17	ABLIM1	ES	16	15	17	1.6E-14	0.132	0.177	5.9E-02	1.5E-02	3.9E-01
LGG	ABLIM1_ES_18.2_17_19	ABLIM1	ES	18.2	17	19	6.3E-15	0.137	-0.358	2.6E-04	1.6E-05	1.0E-03
LGG	ABLIM3_RI_1.2_1.1_1.3	ABLIM3	RI	1.2	1.1	1.3	7.5E-11	0.102	-0.483	7.7E-04	4.6E-03	2.7E-02
LGG	ACADM_ES_2.2_1_3	ACADM	ES	2.2	1	3	2.5E-11	0.103	-0.055	2.8E-01	3.6E-01	2.7E-02
LGG	ACAP1_AD_21.2:21.3_21.1_22.2	ACAP1	AD	21.2:21.3	21.1	22.2	4.0E-16	0.144	-0.317	4.6E-02	4.6E-03	1.8E-07
LGG	ACBD4_RI_3.4_3.3_3.5	ACBD4	RI	3.4	3.3	3.5	3.0E-21	0.183	-0.340	5.3E-04	8.9E-06	1.0E-04
LGG	ACHE_AA_6.1_5_6.2	ACHE	AA	6.1	5	6.2	4.8E-34	0.276	-0.356	2.4E-02	7.1E-05	1.9E-04
LGG	ACIN1_ES_4_3_5	ACIN1	ES	4	3	5	1.2E-21	0.189	0.049	8.0E-05	4.6E-07	9.9E-06
LGG	ACOT8_ES_6.1:6.2_5_8	ACOT8	ES	6.1:6.2	5	8	2.9E-09	0.109	-0.073	9.3E-02	4.1E-04	1.6E-01
LGG	ACP1_AA_4.1:4.2:4.3_3_4.4	ACP1	AA	4.1:4.2:4.3	3	4.4	1.1E-11	0.107	-0.142	7.2E-01	5.9E-01	1.9E-01
LGG	ACP1_ES_4.1_3_4.4	ACP1	ES	4.1	3	4.4	3.5E-17	0.151	-0.059	2.0E-01	9.0E-01	2.5E-01
LGG	ACTR3B_ES_3_2_4	ACTR3B	ES	3	2	4	3.6E-13	0.119	0.136	3.1E-02	6.6E-02	2.3E-02
LGG	ADAM15_ES_21.1:21.2_20_23	ADAM15	ES	21.1:21.2	20	23	9.0E-13	0.115	-0.076	9.4E-04	1.5E-05	1.7E-04
LGG	ADAM15_ES_21.2_20_23	ADAM15	ES	21.2	20	23	1.7E-17	0.154	-0.101	9.0E-03	1.2E-04	1.1E-04
LGG	ADAM22_ES_30_29.1_31	ADAM22	ES	30	29.1	31	3.8E-33	0.271	0.097	3.0E-03	3.6E-04	4.3E-04
LGG	ADARB1_ES_5_4_6	ADARB1	ES	5	4	6	1.1E-18	0.164	0.004	4.9E-01	3.8E-01	1.2E-01
LGG	ADCY5_ES_21_20_22	ADCY5	ES	21	20	22	2.0E-39	0.319	0.089	1.1E-01	7.1E-03	3.8E-02
LGG	ADCYAP1R1_ES_5:6_4_7	ADCYAP1R1	ES	5:06	4	7	1.5E-26	0.225	0.040	3.8E-01	8.3E-01	7.1E-01
LGG	ADD1_AD_12.2_12.1_13	ADD1	AD	12.2	12.1	13	5.8E-24	0.204	-0.022	1.1E-04	2.2E-06	2.6E-04
LGG	ADD3_ES_15_14_16	ADD3	ES	15	14	16	3.7E-20	0.176	-0.223	2.6E-02	2.7E-02	1.4E-01
LGG	ADHFE1_AA_11.1_10_11.2	ADHFE1	AA	11.1	10	11.2	6.1E-25	0.211	-0.292	2.6E-04	1.8E-03	2.4E-03
LGG	ADORA1_ES_4_3.2_5	ADORA1	ES	4	3.2	5	4.9E-18	0.159	-0.400	1.3E-02	1.4E-03	1.6E-03
LGG	AFAP1_ES_13_12_14	AFAP1	ES	13	12	14	9.9E-11	0.103	-0.231	2.4E-03	2.2E-03	3.2E-02
LGG	AFTPH_ES_7:8_6_9	AFTPH	ES	7:08	6	9	1.8E-10	0.122	0.537	1.2E-02	6.0E-03	4.4E-03
LGG	AGAP3_ES_15:16_14_17	AGAP3	ES	15:16	14	17	8.0E-17	0.148	-0.300	1.9E-01	1.3E-01	2.8E-01
LGG	AGBL5_ES_14_13_15	AGBL5	ES	14	13	15	2.1E-18	0.162	-0.293	2.6E-01	6.6E-02	1.1E-03
LGG	AGFG1_ES_6_5_8	AGFG1	ES	6	5	8	3.9E-19	0.168	0.018	2.2E-03	2.7E-05	2.7E-05
LGG	AGTPBP1_AA_11.1_10_11.2	AGTPBP1	AA	11.1	10	11.2	3.9E-11	0.106	0.021	7.2E-02	4.1E-01	9.8E-02
LGG	AKIP1_ES_2_1.3_3	AKIP1	ES	2	1.3	3	1.0E-32	0.266	-0.139	3.8E-03	4.6E-06	6.7E-06
LGG	AKIP1_RI_1.2_1.1_1.3	AKIP1	RI	1.2	1.1	1.3	1.8E-22	0.193	0.035	1.2E-02	2.1E-04	2.3E-02
LGG	ALG13_ES_25_24_26	ALG13	ES	25	24	26	6.9E-20	0.174	-0.158	7.4E-01	5.8E-01	1.5E-01
LGG	ALG2_ES_2_1_3	ALG2	ES	2	1	3	1.8E-15	0.140	-0.022	9.6E-02	1.2E-01	3.3E-02
LGG	AMDHD2_RI_8.2_8.1_8.3	AMDHD2	RI	8.2	8.1	8.3	1.2E-18	0.163	-0.014	7.9E-05	7.1E-03	4.8E-02
LGG	AMN1_ES_2:3:5_1_6	AMN1	ES	2:03:05	1	6	6.2E-12	0.113	-0.209	6.2E-02	5.9E-03	2.1E-02
LGG	ANAPC11_ES_3.3:3.4:6_3.2_7.2	ANAPC11	ES	3.3:3.4:6	3.2	7.2	9.3E-21	0.179	-0.223	1.5E-01	2.7E-01	1.6E-01
LGG	ANK2_ES_9_8_10	ANK2	ES	9	8	10	4.4E-16	0.155	0.316	2.2E-01	8.0E-02	8.7E-02
LGG	ANK3_ES_42_41_43	ANK3	ES	42	41	43	1.5E-18	0.164	0.170	7.2E-04	6.3E-05	7.4E-03
LGG	ANK3_ES_46_45_47	ANK3	ES	46	45	47	2.7E-17	0.155	-0.357	1.2E-07	5.3E-07	4.7E-04
LGG	ANKRD11_ES_9_7_10	ANKRD11	ES	9	7	10	1.6E-23	0.202	-0.028	5.3E-02	4.9E-03	4.0E-02
LGG	ANKRD12_ES_6_4_7	ANKRD12	ES	6	4	7	2.4E-11	0.114	0.108	1.9E-04	2.0E-04	5.4E-03

cancer type	id	Gene Symbol	splice_type	Exon	From.Exon	To.Exon	anova.p	adj.r2	r	p.50	p.25	p.10
LGG	ANXA7_ES_6_5_7	ANXA7	ES	6	5	7	2.8E-21	0.183	0.171	1.9E-02	5.8E-04	2.0E-03
LGG	AP1AR_ES_6_5_7	AP1AR	ES	6	5	7	3.1E-25	0.217	-0.141	4.8E-05	4.3E-04	2.2E-03
LGG	AP1B1_ES_17_16_18	AP1B1	ES	17	16	18	5.3E-42	0.326	-0.295	1.4E-05	5.3E-10	8.2E-09
LGG	AP1G1_ES_12_10.1_13.2	AP1G1	ES	12	10.1	13.2	1.3E-12	0.121	0.053	1.5E-02	3.4E-02	4.1E-02
LGG	AP1S2_AA_5.1_4_5.2	AP1S2	AA	5.1	4	5.2	7.2E-13	0.116	-0.248	3.0E-02	2.5E-02	1.2E-01
LGG	AP2A1_ES_16_15_17	AP2A1	ES	16	15	17	2.1E-37	0.297	0.180	1.2E-04	1.1E-06	6.8E-06
LGG	AP3S1_ES_5:6:7_4_8	AP3S1	ES	5:06:07	4	8	1.3E-19	0.208	-0.120	9.1E-04	2.0E-04	1.5E-07
LGG	AP3S1_ES_6:7_5_8	AP3S1	ES	6:07	5	8	6.1E-17	0.149	0.192	6.6E-05	4.2E-08	7.4E-06
LGG	APBB1_ES_13_12_14.2	APBB1	ES	13	12	14.2	1.5E-33	0.272	0.582	4.0E-04	4.1E-04	6.0E-04
LGG	APBB2_ES_14_12_15.2	APBB2	ES	14	12	15.2	2.7E-22	0.212	0.088	1.9E-05	8.7E-06	3.7E-05
LGG	APBB2_ES_14_13_15.2	APBB2	ES	14	13	15.2	5.0E-45	0.392	0.088	3.2E-05	4.0E-08	1.8E-08
LGG	APBB3_AA_6.6:6.7:6.8_6.2_6.9	APBB3	AA	6.6:6.7:6.8	6.2	6.9	6.2E-26	0.236	0.238	3.8E-02	4.2E-02	2.5E-03
LGG	APBB3_RI_6.3:6.4:6.5_6.2_6.6	APBB3	RI	6.3:6.4:6.5	6.2	6.6	4.5E-12	0.109	0.040	5.5E-03	8.6E-04	1.1E-02
LGG	APEX1_AD_1.2_1.1_2.1	APEX1	AD	1.2	1.1	2.1	2.3E-20	0.177	0.167	3.0E-03	1.4E-03	9.9E-03
LGG	APLP2_ES_9_8_10	APLP2	ES	9	8	10	6.2E-16	0.141	0.149	1.5E-02	8.2E-02	3.8E-02
LGG	APOC1_ES_4:5.1_3.2_7	APOC1	ES	04:05.1	3.2	7	2.5E-14	0.131	0.080	1.8E-03	5.8E-04	9.2E-03
LGG	APP_ES_10_9_11	APP	ES	10	9	11	4.5E-20	0.174	-0.029	1.8E-02	5.7E-05	1.2E-02
LGG	APP_ES_9_8_11	APP	ES	9	8	11	2.0E-18	0.161	-0.142	3.6E-05	1.2E-05	2.9E-06
LGG	APP_ES_9:10_8_11	APP	ES	9:10	8	11	5.1E-29	0.240	-0.115	2.2E-05	6.1E-05	1.0E-04
LGG	ARAP1_ES_32_31_33	ARAP1	ES	32	31	33	2.1E-38	0.303	0.381	1.4E-03	1.5E-03	1.3E-04
LGG	ARFGAP1_AA_14.1_12_14.2	ARFGAP1	AA	14.1	12	14.2	8.1E-31	0.253	-0.054	3.0E-07	1.4E-05	3.7E-04
LGG	ARFGAP1_ES_13:14.1_12_14.2	ARFGAP1	ES	13:14.1	12	14.2	2.2E-27	0.229	0.306	1.2E-05	5.7E-06	7.5E-03
LGG	ARHGAP12_ES_9_8_10	ARHGAP12	ES	9	8	10	8.4E-14	0.129	0.095	1.8E-03	7.3E-05	4.8E-03
LGG	ARHGAP17_ES_18_17_19	ARHGAP17	ES	18	17	19	2.8E-12	0.111	0.119	9.0E-01	7.8E-01	4.7E-01
LGG	ARHGAP21_ES_8.2_7_9	ARHGAP21	ES	8.2	7	9	3.0E-17	0.161	0.226	1.1E-03	1.2E-03	1.0E-03
LGG	ARHGAP23_ES_24_23_25	ARHGAP23	ES	24	23	25	9.1E-32	0.260	0.369	3.4E-06	2.9E-07	7.1E-04
LGG	ARHGAP32_RI_21.2_21.1_21.3	ARHGAP32	RI	21.2	21.1	21.3	6.4E-12	0.112	-0.138	2.7E-01	6.4E-03	4.7E-02
LGG	ARHGAP33_RI_22.2_22.1_22.3	ARHGAP33	RI	22.2	22.1	22.3	3.2E-46	0.352	0.068	4.3E-03	8.8E-03	2.3E-05
LGG	ARHGEF1_ES_15_14_16	ARHGEF1	ES	15	14	16	3.4E-27	0.227	0.128	4.1E-04	9.2E-05	1.1E-02
LGG	ARHGEF10L_ES_8_7_9	ARHGEF10L	ES	8	7	9	1.1E-33	0.275	-0.298	6.8E-04	2.5E-04	2.2E-05
LGG	ARHGEF40_ES_21_20_22	ARHGEF40	ES	21	20	22	4.6E-21	0.182	-0.007	2.6E-03	2.7E-04	1.7E-05
LGG	ARHGEF7_ES_25.1_24_26	ARHGEF7	ES	25.1	24	26	6.7E-25	0.211	0.363	3.8E-05	8.0E-06	1.1E-07
LGG	ARIH2_ES_5_4.2_6	ARIH2	ES	5	4.2	6	3.3E-11	0.104	0.166	5.4E-01	3.8E-01	7.7E-03
LGG	ARL13B_ES_2:3.2:4_1_5.1	ARL13B	ES	03:02.2	1	5.1	3.6E-12	0.138	0.088	1.4E-02	1.1E-02	1.9E-01
LGG	ARMC8_ES_2.2_1_3	ARMC8	ES	2.2	1	3	3.1E-16	0.148	-0.133	5.8E-03	4.6E-02	2.0E-02
LGG	ARMCX3_AA_3.1_1_3.2	ARMCX3	AA	3.1	1	3.2	9.5E-50	0.377	0.112	1.8E-05	1.7E-06	1.6E-07
LGG	ARMCX5_ES_2.6_2.4_2.8	ARMCX5	ES	2.6	2.4	2.8	3.1E-27	0.242	-0.136	7.6E-04	4.5E-04	3.7E-04
LGG	ARNTL_ES_6.1:6.2_5_7	ARNTL	ES	6.1:6.2	5	7	2.6E-12	0.117	0.162	4.6E-01	4.6E-02	5.4E-03
LGG	ARPP21_ES_15_14_17	ARPP21	ES	15	14	17	5.3E-17	0.188	-0.015	5.0E-02	2.8E-02	4.9E-02
LGG	ARPP21_ES_16_15_17	ARPP21	ES	16	15	17	6.6E-10	0.105	0.047	1.8E-01	1.8E-01	9.0E-02

cancer type	id	Gene Symbol	splice_type	Exon	From.Exon	To.Exon	anova.p	adj.r2	r	p.50	p.25	p.10
LGG	ARRB2_ES_2_1_4.2	ARRB2	ES	2	1	4.2	1.4E-54	0.400	-0.379	9.1E-04	8.8E-05	2.2E-03
LGG	ARRDC1_RI_2.2_2.1_2.3	ARRDC1	RI	2.2	2.1	2.3	7.6E-12	0.107	0.036	1.1E-02	9.1E-03	9.9E-02
LGG	ASCC2_ES_6:7_5_8	ASCC2	ES	6:07	5	8	1.2E-17	0.158	0.034	5.0E-01	3.6E-02	7.3E-02
LGG	ASCC2_ES_7_5_8	ASCC2	ES	7	5	8	3.8E-31	0.256	0.099	7.3E-06	9.6E-03	7.3E-03
LGG	ASIC3_RI_9.2_9.1_9.3	ASIC3	RI	9.2	9.1	9.3	8.2E-21	0.180	-0.529	1.2E-04	1.4E-04	7.0E-02
LGG	ASPH_ES_12_11_13	ASPH	ES	12	11	13	8.7E-33	0.267	0.079	1.3E-02	5.0E-04	1.9E-06
LGG	ASTE1_ES_5_4.1_6	ASTE1	ES	5	4.1	6	1.2E-19	0.173	-0.152	3.6E-03	3.7E-03	1.2E-02
LGG	ATF2_ES_7_6_8	ATF2	ES	7	6	8	1.3E-14	0.133	0.124	2.7E-05	8.9E-07	3.8E-05
LGG	ATG13_ES_13:14_12_15	ATG13	ES	13:14	12	15	2.6E-39	0.309	0.422	8.6E-06	5.8E-06	5.8E-05
LGG	ATG13_ES_14_12_15	ATG13	ES	14	12	15	1.9E-37	0.297	0.451	4.0E-08	8.2E-08	6.1E-06
LGG	ATG16L1_ES_8_7_10	ATG16L1	ES	8	7	10	1.5E-19	0.176	0.017	4.6E-04	2.7E-05	4.9E-05
LGG	ATG16L2_RI_6.2_6.1_6.3	ATG16L2	RI	6.2	6.1	6.3	1.0E-13	0.123	0.427	2.8E-03	3.9E-02	9.4E-02
LGG	ATG4D_ES_4.1:4.2_3.2_5	ATG4D	ES	4.1:4.2	3.2	5	1.3E-18	0.163	0.164	6.4E-02	4.3E-02	5.6E-02
LGG	ATG4D_ES_5_3.2_6	ATG4D	ES	5	3.2	6	9.1E-15	0.138	-0.096	9.5E-01	3.7E-02	2.1E-01
LGG	ATG9A_ES_3.1:3.2_1_4	ATG9A	ES	3.1:3.2	1	4	4.8E-11	0.104	-0.012	2.3E-01	2.3E-01	1.5E-02
LGG	ATL1_ES_15_13_16	ATL1	ES	15	13	16	1.9E-23	0.200	0.618	1.1E-01	2.7E-03	3.4E-04
LGG	ATL2_ES_16:17.1_15_17.2	ATL2	ES	16:17.1	15	17.2	1.7E-30	0.251	0.216	1.5E-02	1.1E-03	2.3E-03
LGG	ATP2B2_ES_10:11:12_9_13	ATP2B2	ES	10:11:12	9	13	9.6E-27	0.238	-0.516	9.3E-04	8.3E-07	5.4E-04
LGG	ATP2B4_ES_21_20_22	ATP2B4	ES	21	20	22	1.7E-11	0.104	-0.202	4.9E-03	6.4E-04	4.3E-03
LGG	ATP5C1_ES_9_8.1_10	ATP5C1	ES	9	8.1	10	8.5E-43	0.331	-0.003	6.8E-01	6.7E-01	6.5E-01
LGG	ATP5D_RI_4.2:4.3_4.1_4.4	ATP5D	RI	4.2:4.3	4.1	4.4	1.7E-12	0.113	0.103	5.0E-01	3.5E-01	4.3E-01
LGG	ATP5H_ES_5_3_6	ATP5H	ES	5	3	6	1.5E-18	0.162	-0.224	6.6E-02	2.7E-02	1.3E-01
LGG	ATP9B_ES_30_29_31.1	ATP9B	ES	30	29	31.1	3.8E-23	0.198	0.172	4.2E-02	5.2E-03	2.8E-05
LGG	ATXN1_ES_3_1_4	ATXN1	ES	3	1	4	1.5E-18	0.206	-0.252	1.9E-02	1.6E-03	6.1E-03
LGG	ATXN2L_AA_22.4:22.5_22.1_22.6	ATXN2L	AA	22.4:22.5	22.1	22.6	1.2E-17	0.157	-0.021	7.5E-02	3.1E-02	2.9E-02
LGG	ATXN2L_AA_22.4:22.5:22.6_22.2_22.7	ATXN2L	AA	22.4:22.5:22.6	22.2	22.7	4.8E-12	0.113	0.136	3.7E-01	2.5E-01	2.3E-01
LGG	ATXN2L_AD_22.2_22.1_22.4	ATXN2L	AD	22.2	22.1	22.4	1.0E-10	0.105	0.061	7.2E-01	2.0E-01	3.0E-01
LGG	ATXN2L_ES_22.2:22.4:22.5_22.1_22.6	ATXN2L	ES	22.2:22.4:22.5	22.1	22.6	1.1E-24	0.209	0.059	8.9E-01	4.3E-01	5.2E-01
LGG	ATXN2L_ES_22.2:22.4:22.5:22.6_22.1_2	ATXN2L	ES	2.2:22.4:22.5:22.	22.1	22.7	1.1E-22	0.195	0.118	3.5E-01	1.4E-01	2.5E-02
LGG	ATXN2L_ES_22.2:22.6_22.1_22.7	ATXN2L	ES	22.2:22.6	22.1	22.7	4.0E-13	0.120	0.066	2.2E-02	9.6E-02	2.1E-01
LGG	ATXN2L_RI_22.2:22.3:22.4:22.5_22.1_2	ATXN2L	RI	2.2:22.3:22.4:22.	22.1	22.6	4.9E-17	0.150	0.221	2.5E-06	1.8E-04	1.6E-02
LGG	ATXN7_ES_15_14_16	ATXN7	ES	15	14	16	7.1E-37	0.317	-0.044	1.8E-03	8.2E-05	1.1E-04
LGG	AUTS2_ES_13_12_14	AUTS2	ES	13	12	14	6.4E-28	0.237	0.113	2.5E-03	9.7E-04	3.5E-02
LGG	AXIN2_ES_7_6_8	AXIN2	ES	7	6	8	4.1E-11	0.102	0.165	3.5E-01	1.1E-01	1.6E-01
LGG	B3GALNT1_ES_7:8_5_9.1	B3GALNT1	ES	7:08	5	9.1	1.2E-12	0.124	0.039	2.6E-02	3.4E-02	2.2E-02
LGG	B3GALNT1_ES_8_7_9.1	B3GALNT1	ES	8	7	9.1	6.3E-13	0.120	-0.025	2.6E-06	1.2E-04	5.8E-03
LGG	B3GAT1_RI_2.3:2.4_2.2_2.5	B3GAT1	RI	2.3:2.4	2.2	2.5	5.9E-17	0.151	0.022	7.1E-01	1.2E-01	2.7E-01
LGG	B4GALT3_AA_3.1_2.2_3.2	B4GALT3	AA	3.1	2.2	3.2	3.1E-33	0.269	0.000	7.9E-04	2.3E-05	2.7E-03
LGG	B4GALT4_ES_4_3.2_5	B4GALT4	ES	4	3.2	5	3.9E-18	0.160	-0.433	3.4E-05	1.9E-09	4.9E-06
LGG	BAALC_ES_4:5_1_6	BAALC	ES	4:05	1	6	1.2E-20	0.179	-0.014	3.4E-09	1.5E-05	4.5E-04

cancer type	id	Gene Symbol	splice_type	Exon	From.Exon	To.Exon	anova.p	adj.r2	r	p.50	p.25	p.10
LGG	BABAM1_AA_2.1_1.2_2.2	BABAM1	AA	2.1	1.2	2.2	1.1E-17	0.156	-0.044	4.7E-03	4.0E-05	1.2E-02
LGG	BAI2_ES_26_25_27	BAI2	ES	26	25	27	2.3E-35	0.284	0.289	7.1E-04	3.4E-03	2.2E-03
LGG	BAI2_ES_7_6.2_8	BAI2	ES	7	6.2	8	1.5E-18	0.164	-0.210	3.2E-03	8.3E-05	1.3E-05
LGG	BAI2_ES_8_7_9	BAI2	ES	8	7	9	2.4E-34	0.279	0.204	5.1E-05	4.2E-05	3.6E-03
LGG	BAI3_ES_2_1_3	BAI3	ES	2	1	3	4.9E-10	0.107	0.043	4.8E-01	3.5E-01	3.6E-01
LGG	BBIP1_ES_3:4_1_5.1	BBIP1	ES	3:04	1	5.1	3.0E-18	0.163	-0.149	8.4E-01	6.2E-01	4.0E-01
LGG	BCAN_ES_10_8.1_11	BCAN	ES	10	8.1	11	4.8E-24	0.229	0.008	2.2E-03	7.8E-03	1.3E-01
LGG	BCAN_ES_9_8.1_11	BCAN	ES	9	8.1	11	2.1E-13	0.152	-0.057	6.6E-01	8.3E-01	3.7E-01
LGG	BCAS3_ES_28_27_29	BCAS3	ES	28	27	29	1.9E-10	0.119	-0.099	3.6E-01	9.3E-04	1.7E-04
LGG	BCAS3_ES_28:29_27_30	BCAS3	ES	28:29:00	27	30	4.0E-50	0.375	-0.060	1.8E-03	4.2E-05	2.3E-05
LGG	BCOR_ES_6_5_7	BCOR	ES	6	5	7	3.3E-18	0.165	0.106	2.4E-06	1.1E-07	2.7E-04
LGG	BCS1L_ES_1.4:1.5:1.6:1.7:1.8_1.1_2	BCS1L	ES	1.4:1.5:1.6:1.7:1.8	1.1	2	4.7E-32	0.263	0.081	8.4E-03	2.8E-07	3.8E-05
LGG	BCS1L_ES_1.4:1.5:1.6:1.7:1.8:2_1.1_3	BCS1L	ES	.4:1.5:1.6:1.7:1.8	1.1	3	2.2E-14	0.132	0.117	1.5E-03	8.1E-05	2.2E-04
LGG	BCS1L_ES_1.4:1.5:1.6:1.7:1.8:2:3_1.1_4	BCS1L	ES	4:1.5:1.6:1.7:1.8:2	1.1	4	2.1E-16	0.182	0.120	9.5E-03	2.5E-03	3.0E-05
LGG	BCS1L_ES_1.4:2_1.1_3	BCS1L	ES	1.4:2	1.1	3	1.7E-10	0.103	0.075	2.8E-03	1.1E-03	1.2E-02
LGG	BCS1L_ES_3_1.1_4	BCS1L	ES	3	1.1	4	2.9E-11	0.122	0.033	5.2E-01	3.7E-01	9.5E-01
LGG	BCS1L_RI_1.2:1.3_1.1_1.4	BCS1L	RI	1.2:1.3	1.1	1.4	4.4E-16	0.144	0.002	3.1E-04	4.7E-02	2.2E-02
LGG	BEND6_ES_6_3_7	BEND6	ES	6	3	7	2.2E-19	0.173	0.090	3.0E-01	6.2E-02	1.6E-02
LGG	BEST3_AA_11.1_10_11.2	BEST3	AA	11.1	10	11.2	1.8E-15	0.159	-0.140	5.1E-02	2.1E-01	5.3E-01
LGG	BEST3_ES_9_8_10	BEST3	ES	9	8	10	7.4E-48	0.378	0.124	6.7E-03	4.5E-05	3.9E-08
LGG	BID_ES_4_2_5	BID	ES	4	2	5	1.7E-19	0.170	-0.426	4.7E-05	2.2E-06	1.0E-03
LGG	BID_ES_4:5_2_7	BID	ES	4:05	2	7	1.9E-23	0.200	-0.313	5.3E-05	7.0E-06	6.8E-07
LGG	BIN1_ES_13:16:17_12_18	BIN1	ES	13:16:17	12	18	3.7E-37	0.296	-0.125	1.2E-01	3.4E-01	5.6E-01
LGG	BIN1_ES_13:17_12_18	BIN1	ES	13:17	12	18	1.6E-12	0.113	-0.343	9.7E-02	2.3E-02	1.4E-01
LGG	BIN1_ES_16_13_17	BIN1	ES	16	13	17	5.4E-26	0.219	0.112	1.4E-01	8.7E-02	2.1E-03
LGG	BIN1_ES_16:17_12_18	BIN1	ES	16:17	12	18	7.7E-23	0.196	0.181	9.5E-01	8.6E-01	3.6E-01
LGG	BIN1_ES_16:17_13_18	BIN1	ES	16:17	13	18	9.8E-22	0.210	0.048	3.9E-01	3.8E-02	1.7E-01
LGG	BIN1_ES_7_6_8	BIN1	ES	7	6	8	3.0E-26	0.220	-0.241	1.3E-02	1.3E-06	4.5E-06
LGG	BLM_AA_6.1_5_6.2	BLM	AA	6.1	5	6.2	6.7E-18	0.179	-0.735	1.8E-05	1.1E-03	5.2E-03
LGG	BLOC1S6_ES_3_1_8	BLOC1S6	ES	3	1	8	1.7E-10	0.111	0.106	3.6E-01	4.1E-02	2.0E-01
LGG	BMP1_AA_5.1_4_5.2	BMP1	AA	5.1	4	5.2	2.4E-21	0.199	-0.151	3.8E-05	2.2E-04	3.5E-03
LGG	BMP1_ES_6_5.2_7	BMP1	ES	6	5.2	7	1.3E-36	0.303	-0.229	6.9E-05	8.7E-05	3.7E-03
LGG	BMP2K_ES_16_15.1_17	BMP2K	ES	16	15.1	17	1.3E-18	0.207	0.465	5.6E-01	5.0E-01	6.6E-01
LGG	BOLA3_ES_3_2_4	BOLA3	ES	3	2	4	2.7E-15	0.136	0.333	7.4E-03	1.6E-03	7.2E-03
LGG	BPHL_ES_2_1.2_3	BPHL	ES	2	1.2	3	1.7E-25	0.216	-0.057	3.1E-04	7.9E-05	7.2E-03
LGG	BRD1_AA_11.1:11.2_10_11.3	BRD1	AA	11.1:11.2	10	11.3	2.1E-20	0.178	0.057	8.6E-05	1.2E-04	1.3E-03
LGG	BRD9_AA_9.1_8_9.2	BRD9	AA	9.1	8	9.2	2.1E-28	0.236	0.310	1.5E-04	2.7E-07	8.8E-08
LGG	BRE_ME_13|14_12_15	BRE	ME	13|14	12	15	2.3E-25	0.214	0.023	4.9E-04	7.0E-03	1.6E-02
LGG	BRSK2_ES_15_14_16	BRSK2	ES	15	14	16	6.1E-21	0.185	-0.195	1.8E-01	1.4E-01	1.0E-02
LGG	BTN2A1_ES_4_3_5	BTN2A1	ES	4	3	5	3.0E-13	0.121	0.086	1.9E-04	9.1E-03	4.6E-03

cancer type	id	Gene Symbol	splice_type	Exon	From.Exon	To.Exon	anova.p	adj.r2	r	p.50	p.25	p.10
LGG	BTN3A1_AA_10.1_9_10.2	BTN3A1	AA	10.1	9	10.2	9.8E-11	0.106	-0.086	3.2E-03	6.5E-04	5.0E-02
LGG	BUB3_AA_8.1_7_8.2	BUB3	AA	8.1	7	8.2	3.8E-57	0.414	-0.453	2.3E-02	3.3E-02	1.0E-01
LGG	BUD31_AD_1.2_1.1_2	BUD31	AD	1.2	1.1	2	5.9E-28	0.233	-0.230	1.9E-07	1.4E-05	6.5E-04
LGG	C11orf73_ES_2:3_1_4	C11orf73	ES	2:03	1	4	4.3E-14	0.127	-0.155	2.6E-03	4.9E-03	1.2E-03
LGG	C11orf88_RI_6.2_6.1_6.3	C11orf88	RI	6.2	6.1	6.3	7.0E-45	0.344	-0.738	6.3E-06	6.3E-06	7.6E-08
LGG	C14orf159_ES_4:5.2_1_7	C14orf159	ES	04:05.2	1	7	1.9E-15	0.139	-0.242	7.3E-02	5.6E-04	2.6E-02
LGG	C14orf159_ES_6_5.2_7	C14orf159	ES	6	5.2	7	2.7E-11	0.103	-0.291	3.4E-01	2.9E-01	4.5E-02
LGG	C14orf79_AD_2.3:2.4_2.2_3.2	C14orf79	AD	2.3:2.4	2.2	3.2	3.6E-12	0.110	-0.088	1.3E-02	4.2E-04	3.2E-01
LGG	C16orf13_ES_2_1_5	C16orf13	ES	2	1	5	4.2E-14	0.126	0.038	3.3E-02	8.8E-02	1.1E-01
LGG	C16orf93_AA_2.1_1_2.2	C16orf93	AA	2.1	1	2.2	1.2E-11	0.106	-0.282	5.9E-02	3.1E-02	2.9E-01
LGG	C17orf58_AD_1.4_1.3_2	C17orf58	AD	1.4	1.3	2	2.2E-11	0.103	-0.174	6.2E-01	2.8E-01	7.2E-01
LGG	C17orf62_ES_5.1:5.2_4.3_6	C17orf62	ES	5.1:5.2	4.3	6	8.7E-16	0.173	0.052	2.1E-02	7.4E-03	1.0E-03
LGG	C19orf25_RI_1.4_1.3_1.5	C19orf25	RI	1.4	1.3	1.5	2.2E-18	0.201	-0.110	3.3E-01	2.6E-01	1.0E-01
LGG	C19orf57_ES_8_7_9	C19orf57	ES	8	7	9	9.7E-28	0.232	0.026	1.3E-02	8.1E-04	2.1E-05
LGG	C1D_AD_1.2_1.1_3.1	C1D	AD	1.2	1.1	3.1	1.5E-12	0.113	0.051	4.1E-05	8.6E-06	1.3E-02
LGG	C1D_ES_2.2_1.1_3.1	C1D	ES	2.2	1.1	3.1	1.0E-23	0.202	0.085	3.8E-06	2.1E-05	8.3E-02
LGG	C1orf194_ES_3:4_2_5	C1orf194	ES	3:04	2	5	1.6E-10	0.106	-0.192	4.4E-01	9.4E-01	7.1E-01
LGG	C1orf61_ES_2:3:4:5_1_7	C1orf61	ES	2:3:4:5	1	7	2.0E-10	0.100	0.069	6.9E-02	6.8E-03	1.4E-02
LGG	C1orf61_ES_4:5_3_7	C1orf61	ES	4:05	3	7	8.9E-14	0.124	0.159	4.2E-01	4.2E-03	3.8E-04
LGG	C1orf61_ES_4:5:6_2_7	C1orf61	ES	4:05:06	2	7	8.0E-12	0.107	-0.272	5.4E-02	1.9E-02	2.5E-02
LGG	C1orf61_ES_6_5_7	C1orf61	ES	6	5	7	9.0E-33	0.267	-0.309	9.0E-06	8.7E-06	1.6E-06
LGG	C1QTNF3_AD_1.2_1.1_2	C1QTNF3	AD	1.2	1.1	2	5.5E-20	0.175	-0.124	2.8E-03	2.0E-05	5.2E-04
LGG	C2CD5_ES_26:27.1:27.2_25_28	C2CD5	ES	26:27.1:27.2	25	28	4.0E-30	0.249	0.306	2.9E-06	1.8E-07	3.5E-05
LGG	C3orf17_ES_2_1_3.1	C3orf17	ES	2	1	3.1	1.2E-18	0.165	-0.071	3.5E-02	5.2E-02	3.2E-01
LGG	C3orf17_ES_3.2:4.1:4.2:5.1_3.1_6.1	C3orf17	ES	3.2:4.1:4.2:5.1	3.1	6.1	1.8E-10	0.118	-0.122	1.7E-01	2.2E-02	4.5E-02
LGG	C4orf36_ES_7.1:7.2_6_8	C4orf36	ES	7.1:7.2	6	8	1.1E-14	0.141	-0.250	1.6E-02	1.9E-03	4.8E-05
LGG	C5orf45_ES_2.2:2.3:4:5.1:5.2_2.1_6	C5orf45	ES	2.2:2.3:4:5.1:5.2	2.1	6	1.4E-13	0.125	-0.100	6.0E-04	1.1E-02	7.4E-02
LGG	C5orf45_ES_2.3:4_2.1_5.1	C5orf45	ES	2.3:4	2.1	5.1	4.5E-11	0.117	-0.052	8.9E-03	3.9E-02	1.6E-01
LGG	C5orf45_ES_2.3:4:5.1:5.2_2.1_6	C5orf45	ES	2.3:4:5.1:5.2	2.1	6	1.1E-23	0.208	-0.098	4.8E-03	3.3E-04	8.1E-04
LGG	C5orf45_ES_3.1:3.2:4:5.1:5.2_2.1_6	C5orf45	ES	3.1:3.2:4:5.1:5.2	2.1	6	1.6E-11	0.113	-0.104	3.2E-02	1.2E-01	2.5E-04
LGG	C5orf45_ES_4_2.1_6	C5orf45	ES	4	2.1	6	4.4E-18	0.165	-0.143	3.3E-04	1.2E-03	4.0E-04
LGG	C5orf45_ES_4:5.1:5.2_2.1_6	C5orf45	ES	4:5.1:5.2	2.1	6	5.5E-68	0.469	-0.216	3.2E-04	1.1E-05	9.7E-04
LGG	C5orf63_ES_4_3_5.1	C5orf63	ES	4	3	5.1	2.7E-38	0.303	-0.656	2.4E-05	4.6E-04	4.1E-06
LGG	C8orf58_ES_6.2_5_7	C8orf58	ES	6.2	5	7	2.4E-11	0.109	-0.060	2.3E-01	3.8E-01	3.2E-01
LGG	C9orf142_ES_3_2_4	C9orf142	ES	3	2	4	7.1E-16	0.141	-0.030	1.1E-03	1.0E-03	9.8E-04
LGG	CA5B_ES_4_3_5	CA5B	ES	4	3	5	8.7E-23	0.196	0.660	1.6E-01	1.1E-01	1.0E-01
LGG	CABIN1_AD_27.2_27.1_28	CABIN1	AD	27.2	27.1	28	9.3E-33	0.266	-0.131	1.5E-08	4.2E-08	1.0E-04
LGG	CADM1_ES_10_9_12	CADM1	ES	10	9	12	1.2E-17	0.156	0.083	9.8E-04	2.3E-04	2.4E-04
LGG	CADM1_ES_10:11_9_12	CADM1	ES	10:11	9	12	1.1E-16	0.147	0.082	1.2E-02	1.9E-03	8.0E-04
LGG	CADM1_ES_11_10_12	CADM1	ES	11	10	12	8.6E-22	0.187	0.002	6.0E-01	4.4E-01	7.6E-01

cancer type	id	Gene Symbol	splice_type	Exon	From.Exon	To.Exon	anova.p	adj.r2	r	p.50	p.25	p.10
LGG	CADM1_ES_9_8_10	CADM1	ES	9	8	10	1.4E-31	0.258	-0.074	2.5E-06	1.2E-06	1.5E-05
LGG	CADM1_ES_9_8_12	CADM1	ES	9	8	12	1.3E-12	0.126	-0.169	4.7E-05	3.6E-04	1.4E-04
LGG	CADM1_ES_9:10:11_8_12	CADM1	ES	9:10:11	8	12	1.9E-20	0.177	-0.161	2.1E-02	1.2E-01	6.4E-01
LGG	CADM2_ES_9_8_10	CADM2	ES	9	8	10	1.2E-19	0.172	-0.415	5.5E-05	6.7E-04	2.7E-02
LGG	CADPS_ES_18_17_20	CADPS	ES	18	17	20	3.1E-25	0.224	0.228	3.1E-02	1.3E-04	1.7E-03
LGG	CALD1_ES_17_16_18	CALD1	ES	17	16	18	7.2E-33	0.269	-0.657	2.5E-07	3.1E-08	4.5E-07
LGG	CALHM2_AA_4.1_3.2_4.2	CALHM2	AA	4.1	3.2	4.2	2.2E-13	0.120	-0.441	1.9E-03	2.0E-03	6.6E-02
LGG	CAMK2B_AA_14.1_12_14.2	CAMK2B	AA	14.1	12	14.2	1.1E-22	0.218	-0.369	2.6E-04	1.7E-09	1.6E-07
LGG	CAMK2B_ES_14.2:15:16:17_12_21.1	CAMK2B	ES	14.2:15:16:17	12	21.1	8.4E-10	0.109	0.160	5.4E-01	6.6E-01	3.5E-01
LGG	CAMK2B_ES_16_15_17	CAMK2B	ES	16	15	17	1.1E-21	0.192	0.468	1.4E-02	1.5E-04	6.3E-03
LGG	CAMK2D_AA_14.1_13_14.2	CAMK2D	AA	14.1	13	14.2	1.1E-19	0.175	0.012	8.8E-03	1.1E-03	1.3E-03
LGG	CAMK2D_ES_22_21.1_23	CAMK2D	ES	22	21.1	23	3.1E-19	0.170	0.177	4.8E-01	3.6E-02	4.2E-01
LGG	CAMK2G_ES_19.1:19.2_18_21	CAMK2G	ES	19.1:19.2	18	21	5.1E-16	0.142	0.333	1.5E-06	4.5E-07	2.2E-07
LGG	CAMTA2_ES_21_20_22	CAMTA2	ES	21	20	22	5.4E-22	0.189	0.718	7.5E-03	3.4E-03	1.5E-02
LGG	CANT1_RI_3.2_3.1_3.3	CANT1	RI	3.2	3.1	3.3	1.8E-14	0.130	-0.266	6.1E-03	3.4E-04	8.1E-01
LGG	CAPN3_ES_11_10_12	CAPN3	ES	11	10	12	3.4E-12	0.122	0.007	4.8E-03	2.4E-04	1.7E-04
LGG	CAPRIN2_ES_5_4_6	CAPRIN2	ES	5	4	6	1.3E-20	0.183	-0.018	9.9E-01	7.4E-01	8.9E-01
LGG	CARD8_ES_4.1_3_5	CARD8	ES	4.1	3	5	9.3E-18	0.161	0.177	4.6E-04	2.2E-05	1.0E-03
LGG	CARD8_ES_6:7.2_5_8	CARD8	ES	06:07.2	5	8	9.2E-27	0.249	0.339	7.1E-05	1.5E-08	2.1E-07
LGG	CARD8_ES_7.1:7.2_5_8	CARD8	ES	7.1:7.2	5	8	2.4E-33	0.279	0.327	6.1E-08	1.1E-08	4.4E-07
LGG	CARD8_ES_7.2_5_8	CARD8	ES	7.2	5	8	1.3E-59	0.450	0.456	1.4E-08	8.1E-09	5.4E-07
LGG	CARM1_AA_16.1:16.2_15_16.3	CARM1	AA	16.1:16.2	15	16.3	1.5E-18	0.163	-0.010	5.5E-03	4.1E-04	1.6E-03
LGG	CARM1_ES_16.1_15_16.3	CARM1	ES	16.1	15	16.3	8.3E-39	0.306	-0.185	6.8E-05	1.2E-08	5.3E-07
LGG	CARS_AA_5.1_4_5.2	CARS	AA	5.1	4	5.2	1.6E-32	0.267	0.077	3.2E-03	1.4E-05	6.4E-06
LGG	CASK_ES_20_19.1_21	CASK	ES	20	19.1	21	2.2E-21	0.186	0.165	1.7E-01	5.5E-01	7.2E-01
LGG	CASP3_ES_2_1_4	CASP3	ES	2	1	4	1.4E-12	0.116	0.126	1.9E-04	1.5E-05	3.5E-04
LGG	CC2D2A_ES_5_4_6	CC2D2A	ES	5	4	6	2.3E-18	0.161	-0.180	3.6E-01	6.6E-02	2.9E-01
LGG	CCDC120_RI_11.2_11.1_11.3	CCDC120	RI	11.2	11.1	11.3	1.1E-13	0.123	-0.126	4.9E-03	2.7E-04	2.8E-04
LGG	CCDC136_AD_5.2_5.1_6	CCDC136	AD	5.2	5.1	6	2.8E-13	0.120	0.051	3.9E-04	1.7E-05	9.0E-02
LGG	CCDC136_ES_12:13:14:15:16:17_11_18	CCDC136	ES	l2:13:14:15:16:1	11	18	9.8E-12	0.115	-0.202	1.5E-08	1.5E-07	6.0E-05
LGG	CCDC136_ES_9:10:11:12:13:14:15:16:17	CCDC136	ES	:11:12:13:14:15:1	8	18	1.0E-20	0.183	-0.033	3.2E-06	8.3E-07	5.5E-08
LGG	CCDC53_ES_2_1_4	CCDC53	ES	2	1	4	2.5E-11	0.121	0.008	6.6E-07	5.5E-07	5.4E-05
LGG	CCDC64_ES_6:7_5_8	CCDC64	ES	6:07	5	8	3.2E-09	0.106	-0.022	2.1E-01	7.5E-01	6.8E-01
LGG	CCDC74B_AA_3.3:3.4_3.1_3.5	CCDC74B	AA	3.3:3.4	3.1	3.5	2.6E-12	0.114	-0.216	5.7E-01	3.7E-03	6.0E-03
LGG	CCDC84_AD_6.2_6.1_7	CCDC84	AD	6.2	6.1	7	8.8E-12	0.107	0.380	3.3E-03	5.0E-04	2.6E-03
LGG	CCM2_ES_7_5_8	CCM2	ES	7	5	8	2.6E-22	0.201	-0.161	1.3E-02	2.6E-02	7.8E-02
LGG	CCNDBP1_ES_10.1_9_10.3	CCNDBP1	ES	10.1	9	10.3	3.8E-15	0.135	-0.108	9.0E-04	2.9E-02	6.9E-03
LGG	CCNL1_ES_4.1_3_5	CCNL1	ES	4.1	3	5	5.6E-42	0.331	0.285	2.3E-05	3.9E-04	3.8E-02
LGG	CCNL1_ES_4.1:4.2:4.3_3_5	CCNL1	ES	4.1:4.2:4.3	3	5	1.2E-21	0.189	0.291	2.1E-02	1.4E-04	4.0E-03
LGG	CCNL1_ES_7_6_9	CCNL1	ES	7	6	9	1.9E-20	0.178	0.275	9.7E-01	4.8E-01	5.5E-01

cancer type	id	Gene Symbol	splice_type	Exon	From.Exon	To.Exon	anova.p	adj.r2	r	p.50	p.25	p.10
LGG	CCNL1_ES_7:8_6_9	CCNL1	ES	7:08	6	9	2.0E-24	0.208	0.122	1.2E-01	1.5E-01	3.1E-01
LGG	CCNT2_RI_10.2_10.1_10.3	CCNT2	RI	10.2	10.1	10.3	1.0E-24	0.213	-0.151	1.5E-02	5.2E-07	4.9E-04
LGG	CCP110_ES_2_1_3	CCP110	ES	2	1	3	6.0E-18	0.174	-0.015	2.6E-03	1.2E-02	4.4E-02
LGG	CCPG1_RI_9.4_9.3_9.5	CCPG1	RI	9.4	9.3	9.5	1.5E-12	0.113	-0.066	1.7E-02	4.1E-02	2.3E-03
LGG	CCSER2_ES_11_10_12	CCSER2	ES	11	10	12	1.1E-15	0.139	0.318	3.3E-04	1.9E-04	2.8E-04
LGG	CCT7_ES_2:3:4:5.1:5.2:6:7_1_8	CCT7	ES	2:3:4:5.1:5.2:6:7	1	8	7.3E-11	0.109	-0.068	4.1E-02	1.3E-04	8.0E-02
LGG	CD200_ES_3_1_4	CD200	ES	3	1	4	3.0E-14	0.129	0.439	6.9E-03	4.8E-04	8.2E-03
LGG	CD320_ES_2.1:2.2:3.2_1_4	CD320	ES	2.1:2.2:3.2	1	4	1.1E-09	0.111	-0.216	6.3E-01	3.6E-01	9.5E-02
LGG	CD46_ES_13_12_14	CD46	ES	13	12	14	5.3E-21	0.182	0.001	3.4E-06	2.1E-05	1.2E-06
LGG	CD46_ES_7:8:9_6_10	CD46	ES	7:08:09	6	10	2.8E-09	0.103	0.237	8.7E-01	6.7E-02	6.2E-02
LGG	CDC42SE1_ES_5_4_6	CDC42SE1	ES	5	4	6	3.2E-33	0.269	-0.045	5.5E-03	1.1E-02	1.0E-03
LGG	CDH24_ES_9_8_10	CDH24	ES	9	8	10	3.6E-18	0.161	-0.154	8.9E-03	3.1E-03	1.1E-05
LGG	CDIPT_ES_2.2_1.5_3.2	CDIPT	ES	2.2	1.5	3.2	2.4E-10	0.102	0.070	2.3E-04	1.2E-03	6.5E-04
LGG	CDK20_ES_5_4_6	CDK20	ES	5	4	6	4.2E-18	0.160	-0.019	2.7E-02	5.7E-04	1.7E-02
LGG	CDKAL1_ES_13_12_14	CDKAL1	ES	13	12	14	1.0E-12	0.116	-0.072	7.9E-01	3.4E-01	4.7E-01
LGG	CELF1_AA_18.1:18.2_17_18.3	CELF1	AA	18.1:18.2	17	18.3	3.0E-19	0.168	0.047	2.7E-02	1.2E-02	9.1E-03
LGG	CELF2_AD_12.2_12.1_14.2	CELF2	AD	12.2	12.1	14.2	1.3E-13	0.128	-0.393	7.5E-03	6.2E-04	1.1E-02
LGG	CELF3_ES_8_7_9	CELF3	ES	8	7	9	2.6E-16	0.147	0.198	7.1E-01	5.4E-01	7.2E-02
LGG	CELSR3_ES_17_16_18	CELSR3	ES	17	16	18	1.0E-12	0.150	-0.216	8.3E-01	9.1E-01	2.2E-01
LGG	CEP170_AD_16.3_16.2_17	CEP170	AD	16.3	16.2	17	7.3E-34	0.278	0.047	4.7E-06	5.0E-05	2.4E-05
LGG	CEP70_AA_3.1_2_3.2	CEP70	AA	3.1	2	3.2	3.2E-21	0.189	-0.147	6.0E-03	2.0E-04	4.7E-03
LGG	CERS5_ES_15_14_16	CERS5	ES	15	14	16	1.4E-20	0.178	-0.035	4.4E-06	2.3E-08	1.9E-04
LGG	CERS5_ES_17.1:17.2_16_18	CERS5	ES	17.1:17.2	16	18	1.0E-23	0.202	0.042	1.3E-05	3.0E-07	1.6E-05
LGG	CERS5_ES_17.2_16_18	CERS5	ES	17.2	16	18	1.2E-19	0.171	-0.176	5.8E-05	1.2E-04	2.7E-06
LGG	CES2_AD_1.2:1.3_1.1_2	CES2	AD	1.2:1.3	1.1	2	1.3E-13	0.123	-0.052	3.7E-03	6.0E-04	1.8E-02
LGG	CHD3_ES_34_33_35	CHD3	ES	34	33	35	4.7E-66	0.460	0.365	1.0E-04	3.0E-05	3.9E-03
LGG	CHEK2_ME_6|7.1:7.2_5_9	CHEK2	ME	6|7.1:7.2	5	9	8.1E-17	0.148	0.668	1.5E-03	8.7E-06	1.3E-07
LGG	CHFR_ES_5:6.1:6.2_4_8	CHFR	ES	5:6.1:6.2	4	8	5.6E-24	0.211	0.074	2.2E-04	4.7E-04	5.0E-02
LGG	CHFR_ES_5:6.2_4_8	CHFR	ES	05:06.2	4	8	1.2E-26	0.226	0.091	2.5E-04	2.7E-07	2.8E-04
LGG	CHI3L2_ES_4.2_3.2_5	CHI3L2	ES	4.2	3.2	5	1.5E-11	0.120	0.204	2.0E-02	2.8E-03	5.3E-03
LGG	CIRBP_AD_9.4:9.5:9.6_9.3_9.8	CIRBP	AD	9.4:9.5:9.6	9.3	9.8	7.6E-16	0.141	0.158	3.4E-03	1.2E-02	3.2E-02
LGG	CIRBP_ES_9.5:9.6_9.3_9.8	CIRBP	ES	9.5:9.6	9.3	9.8	2.1E-12	0.112	0.020	3.6E-02	3.3E-04	2.1E-04
LGG	CLASP1_ES_22_21_24	CLASP1	ES	22	21	24	2.8E-26	0.232	-0.218	1.7E-03	2.4E-02	1.6E-02
LGG	CLASP1_ES_29_28.1_31.1	CLASP1	ES	29	28.1	31.1	9.2E-27	0.231	0.261	3.6E-03	1.6E-03	5.0E-04
LGG	CLASP2_ES_27_26_28	CLASP2	ES	27	26	28	5.1E-20	0.175	0.327	2.1E-02	7.0E-02	5.0E-01
LGG	CLASRP_AD_15.2_15.1_16	CLASRP	AD	15.2	15.1	16	2.7E-20	0.176	0.486	2.6E-02	1.4E-02	1.7E-01
LGG	CLEC16A_AD_11.2_11.1_12	CLEC16A	AD	11.2	11.1	12	6.9E-20	0.179	-0.173	9.8E-03	2.9E-05	8.2E-04
LGG	CLEC16A_ES_24_23_25	CLEC16A	ES	24	23	25	5.1E-11	0.102	0.016	1.1E-02	3.3E-03	5.6E-02
LGG	CLEC2D_ES_7.3:7.4:7.5_7.1_8	CLEC2D	ES	7.3:7.4:7.5	7.1	8	8.0E-11	0.117	-0.284	8.9E-02	6.0E-02	3.1E-01
LGG	CLIP1_ES_11.2:12_11.1_13	CLIP1	ES	11.2:12	11.1	13	1.7E-32	0.267	0.222	7.0E-03	1.7E-04	9.6E-04

cancer type	id	Gene Symbol	splice_type	Exon	From.Exon	To.Exon	anova.p	adj.r2	r	p.50	p.25	p.10
LGG	CLIP2_ES_9_8_10	CLIP2	ES	9	8	10	1.6E-39	0.311	-0.074	7.2E-05	8.1E-07	5.5E-07
LGG	CLK2_ES_5.2_4.2_6	CLK2	ES	5.2	4.2	6	6.8E-15	0.161	0.037	3.0E-01	3.5E-01	1.6E-02
LGG	CLSTN1_ES_3_2_4	CLSTN1	ES	3	2	4	1.1E-27	0.234	-0.499	4.4E-03	4.4E-02	1.7E-01
LGG	CLYBL_RI_9.2_9.1_9.3	CLYBL	RI	9.2	9.1	9.3	3.8E-40	0.316	-0.049	5.4E-03	1.2E-04	1.3E-02
LGG	CMC2_ES_3_2_5	CMC2	ES	3	2	5	4.7E-17	0.150	-0.267	4.4E-03	9.7E-03	1.6E-02
LGG	CMC2_ES_3:5:9:10_2_12	CMC2	ES	3:5:9:10	2	12	2.5E-10	0.123	-0.034	9.3E-02	3.1E-01	4.4E-01
LGG	CMTM3_ES_6:7.1_5_7.2	CMTM3	ES	06:07.1	5	7.2	2.6E-12	0.119	-0.014	5.1E-01	4.9E-01	8.8E-01
LGG	CMTM5_ES_2.1:2.2:3_1_4	CMTM5	ES	2.1:2.2:3	1	4	7.4E-13	0.136	0.027	3.5E-02	6.0E-03	2.1E-03
LGG	CNEP1R1_ES_2.2_1_3	CNEP1R1	ES	2.2	1	3	6.2E-10	0.100	0.016	5.3E-04	5.0E-04	1.8E-02
LGG	CNIH2_RI_6.2_6.1_6.3	CNIH2	RI	6.2	6.1	6.3	1.0E-11	0.106	-0.291	1.9E-03	4.3E-02	1.7E-01
LGG	CNOT4_ES_13_10.2_14	CNOT4	ES	13	10.2	14	1.1E-28	0.238	0.188	1.7E-03	8.3E-03	3.3E-01
LGG	COL16A1_ES_53_52_54	COL16A1	ES	53	52	54	7.4E-09	0.102	0.395	8.8E-04	1.1E-03	1.2E-01
LGG	COPS3_ES_5:6.1_4_6.2	COPS3	ES	05:06.1	4	6.2	4.9E-23	0.204	-0.016	3.4E-01	8.1E-02	3.5E-01
LGG	COX11_AA_3.3_3.1_3.4	COX11	AA	3.3	3.1	3.4	1.6E-16	0.146	0.339	1.9E-03	9.2E-05	5.5E-06
LGG	COX11_RI_3.2:3.3_3.1_3.4	COX11	RI	3.2:3.3	3.1	3.4	4.1E-28	0.234	0.344	1.3E-03	9.1E-05	6.1E-06
LGG	COX20_ES_2:3_1_4	COX20	ES	2:03	1	4	1.6E-15	0.138	0.173	3.3E-02	2.2E-04	3.3E-04
LGG	COX20_ES_3_1_4	COX20	ES	3	1	4	2.4E-29	0.243	0.127	2.3E-05	2.6E-03	3.3E-03
LGG	COX4I1_ES_4.2:5.1_4.1_5.4	COX4I1	ES	4.2:5.1	4.1	5.4	3.9E-12	0.110	-0.266	1.6E-01	2.4E-01	4.7E-02
LGG	COX4I1_ES_4.2:5.1:5.2:5.3_4.1_5.4	COX4I1	ES	4.2:5.1:5.2:5.3	4.1	5.4	1.1E-24	0.209	-0.162	2.4E-01	8.3E-02	1.2E-01
LGG	COX7A2_ES_4.1:4.2_3_5	COX7A2	ES	4.1:4.2	3	5	9.4E-11	0.115	-0.299	4.4E-01	7.4E-02	1.4E-02
LGG	CPEB2_ES_6_5_7	CPEB2	ES	6	5	7	1.1E-29	0.280	-0.002	3.2E-07	6.5E-07	1.5E-05
LGG	CPNE1_AD_1.2_1.1_5	CPNE1	AD	1.2	1.1	5	1.0E-20	0.179	0.028	3.4E-03	1.3E-04	2.5E-04
LGG	CPNE1_ES_2.1:2.2:3_1.2_5	CPNE1	ES	2.1:2.2:3	1.2	5	3.0E-14	0.128	-0.662	2.7E-01	6.1E-01	3.0E-01
LGG	CPNE1_ES_2.2:3_1.2_5	CPNE1	ES	2.2:3	1.2	5	7.1E-19	0.165	-0.476	1.5E-01	1.1E-01	3.8E-01
LGG	CPSF4_AA_9.1_8_9.2	CPSF4	AA	9.1	8	9.2	2.3E-31	0.257	-0.230	2.7E-06	1.5E-06	9.6E-06
LGG	CREBZF_AA_2.7_2.5_2.8	CREBZF	AA	2.7	2.5	2.8	5.7E-18	0.160	0.131	1.0E-03	1.4E-04	6.5E-04
LGG	CREBZF_RI_2.4_2.3_2.5	CREBZF	RI	2.4	2.3	2.5	1.1E-15	0.139	-0.115	4.2E-01	7.9E-01	8.5E-02
LGG	CREM_ES_4:9.2:10.1_1_15	CREM	ES	4:9.2:10.1	1	15	3.8E-13	0.119	0.136	2.1E-01	2.9E-03	3.6E-01
LGG	CRTC1_ES_14_13_15	CRTC1	ES	14	13	15	4.8E-14	0.126	0.090	6.3E-03	2.6E-03	9.0E-01
LGG	CSF2RA_ES_13_12_14	CSF2RA	ES	13	12	14	3.6E-24	0.205	-0.726	4.5E-01	2.6E-02	3.0E-01
LGG	CSMD3_ES_35:36_34_37	CSMD3	ES	35:36:00	34	37	2.5E-17	0.196	-0.261	3.2E-01	1.8E-01	2.3E-01
LGG	CSNK1D_ES_11_10_12	CSNK1D	ES	11	10	12	1.0E-46	0.355	0.085	2.2E-09	2.4E-09	8.3E-06
LGG	CSNK1G3_ES_11_10_12	CSNK1G3	ES	11	10	12	1.1E-13	0.125	0.087	3.6E-01	1.1E-01	1.1E-01
LGG	CSPG5_AD_4.2_4.1_5	CSPG5	AD	4.2	4.1	5	4.1E-31	0.255	-0.172	2.4E-05	4.8E-05	2.4E-05
LGG	CTNNA2_ES_26:27_25_28	CTNNA2	ES	26:27:00	25	28	4.3E-31	0.258	-0.284	6.6E-03	2.3E-01	6.0E-02
LGG	CTNNB1_AA_18.3_18.1_18.4	CTNNB1	AA	18.3	18.1	18.4	2.2E-38	0.303	-0.278	1.3E-04	9.0E-08	7.4E-04
LGG	CTNND1_ES_12_11_13	CTNND1	ES	12	11	13	1.6E-52	0.395	-0.284	1.0E-04	2.5E-06	2.9E-04
LGG	CTNS_ES_5_4_7	CTNS	ES	5	4	7	6.6E-26	0.222	0.168	3.5E-06	2.0E-09	2.2E-06
LGG	CTTN_ES_11_10_13	CTTN	ES	11	10	13	7.1E-22	0.188	0.229	2.0E-02	1.6E-02	8.6E-03
LGG	CTTNBP2_AD_4.2_4.1_5	CTTNBP2	AD	4.2	4.1	5	3.8E-28	0.243	-0.499	2.1E-03	3.4E-03	2.4E-03

cancer type	id	Gene Symbol	splice_type	Exon	From.Exon	To.Exon	anova.p	adj.r2	r	p.50	p.25	p.10
LGG	CUL7_AD_3.2_3.1_4	CUL7	AD	3.2	3.1	4	2.2E-11	0.119	-0.176	6.1E-05	1.8E-04	1.8E-02
LGG	CYB561A3_RI_6.2_6.1_6.3	CYB561A3	RI	6.2	6.1	6.3	9.4E-17	0.148	-0.326	4.1E-02	4.8E-02	7.2E-01
LGG	CYLD_ES_3_2.3_4.2	CYLD	ES	3	2.3	4.2	6.4E-16	0.164	0.135	1.3E-01	4.3E-02	6.8E-03
LGG	CYTH2_AA_9.1_8.1_9.2	CYTH2	AA	9.1	8.1	9.2	2.6E-67	0.466	-0.007	1.8E-05	7.6E-07	1.7E-05
LGG	D2HGDH_AA_5.1_4_5.2	D2HGDH	AA	5.1	4	5.2	3.0E-24	0.206	-0.481	2.7E-02	2.4E-03	3.1E-01
LGG	D2HGDH_ES_10:11.1_9_11.2	D2HGDH	ES	10:11.1	9	11.2	7.7E-14	0.124	0.593	1.2E-01	9.9E-02	4.8E-01
LGG	D2HGDH_ES_7.1:7.3_6_8	D2HGDH	ES	7.1:7.3	6	8	3.4E-23	0.199	0.540	1.3E-02	1.8E-02	4.1E-01
LGG	D2HGDH_ES_7.3_7.1_8	D2HGDH	ES	7.3	7.1	8	1.4E-23	0.256	0.581	4.3E-02	6.0E-02	1.3E-01
LGG	D2HGDH_RI_7.2_7.1_7.3	D2HGDH	RI	7.2	7.1	7.3	7.3E-18	0.161	-0.226	5.5E-03	1.6E-02	6.4E-01
LGG	DCAF10_ES_3_2_4	DCAF10	ES	3	2	4	1.8E-14	0.146	0.187	2.4E-02	7.6E-03	1.5E-02
LGG	DCAF16_ES_2_1_3.1	DCAF16	ES	2	1	3.1	1.4E-17	0.169	-0.059	9.6E-02	3.9E-01	4.7E-01
LGG	DCAF6_ES_11_10_13.1	DCAF6	ES	11	10	13.1	1.8E-12	0.115	-0.401	5.1E-01	8.5E-03	2.7E-04
LGG	DCAF6_ES_11:13.1_10_14	DCAF6	ES	11:13.1	10	14	4.3E-22	0.191	-0.350	8.1E-01	7.2E-01	9.6E-02
LGG	DCAF8_AD_7.3_7.2_8.1	DCAF8	AD	7.3	7.2	8.1	4.3E-12	0.110	-0.146	2.1E-01	2.2E-01	3.0E-01
LGG	DCAF8_ES_7.2:7.3:8.1:8.2_7.1_9	DCAF8	ES	7.2:7.3:8.1:8.2	7.1	9	2.1E-13	0.124	0.038	3.8E-02	2.4E-02	3.1E-02
LGG	DCTN2_ES_8_2_10	DCTN2	ES	8	2	10	1.9E-63	0.447	-0.052	1.2E-07	9.1E-11	7.0E-09
LGG	DCUN1D2_ES_5_4_6	DCUN1D2	ES	5	4	6	1.4E-12	0.115	-0.239	1.1E-01	4.6E-02	2.9E-01
LGG	DDHD1_ES_13_12_14	DDHD1	ES	13	12	14	1.5E-64	0.455	-0.311	6.6E-06	3.7E-04	1.7E-03
LGG	DDX55_ES_2.1:2.2:3:4_1_5.1	DDX55	ES	2.1:2.2:3:4	1	5.1	6.0E-12	0.116	-0.011	4.8E-01	9.2E-01	8.9E-01
LGG	DEPDC5_AA_28.1_27.1_28.2	DEPDC5	AA	28.1	27.1	28.2	4.6E-11	0.108	0.294	1.1E-02	1.5E-04	2.8E-04
LGG	DEPDC5_ES_36_35_37	DEPDC5	ES	36	35	37	1.7E-32	0.270	0.362	7.1E-04	5.5E-05	2.0E-05
LGG	DGKG_ES_12_11_13	DGKG	ES	12	11	13	4.6E-10	0.103	-0.088	3.2E-01	1.4E-02	9.2E-02
LGG	DGUOK_ES_2:4:5:6_1_7	DGUOK	ES	2:4:5:6	1	7	2.6E-14	0.128	0.075	3.3E-01	3.2E-01	5.9E-01
LGG	DGUOK_ES_3:4:5_1_7	DGUOK	ES	3:04:05	1	7	2.1E-24	0.207	-0.090	3.6E-02	1.2E-03	2.5E-03
LGG	DGUOK_ES_3:4:5:6_1_7	DGUOK	ES	3:4:5:6	1	7	9.3E-12	0.108	-0.063	8.5E-02	8.4E-01	4.3E-01
LGG	DGUOK_ES_4:5_1_7	DGUOK	ES	4:05	1	7	9.9E-23	0.194	-0.021	9.9E-04	8.2E-03	5.5E-03
LGG	DGUOK_ES_5_4_7	DGUOK	ES	5	4	7	4.9E-46	0.351	-0.038	2.0E-05	7.2E-07	9.3E-06
LGG	DGUOK_ES_5:6_4_7	DGUOK	ES	5:06	4	7	2.5E-36	0.290	-0.004	1.1E-01	4.8E-05	6.2E-03
LGG	DHX30_ES_6_5_10	DHX30	ES	6	5	10	9.6E-20	0.174	-0.115	3.9E-02	2.7E-02	2.2E-01
LGG	DLG3_ME_16|17_15_18	DLG3	ME	16|17	15	18	4.2E-34	0.302	-0.588	5.2E-06	4.9E-12	3.0E-08
LGG	DLL3_RI_8.2_8.1_8.3	DLL3	RI	8.2	8.1	8.3	2.6E-35	0.284	0.117	2.2E-02	6.0E-02	2.0E-01
LGG	DMD_ES_82_81_83	DMD	ES	82	81	83	1.8E-11	0.108	0.266	5.4E-01	5.7E-02	2.3E-01
LGG	DMTF1_AA_13.1:13.2_12_13.3	DMTF1	AA	13.1:13.2	12	13.3	3.8E-18	0.163	0.352	3.8E-06	1.5E-05	1.5E-04
LGG	DNAH1_ES_75_74_76	DNAH1	ES	75	74	76	1.4E-12	0.136	0.290	6.7E-02	1.3E-02	3.7E-02
LGG	DNAJB2_RI_9.2_9.1_9.3	DNAJB2	RI	9.2	9.1	9.3	5.3E-15	0.134	0.134	1.5E-01	4.2E-02	5.7E-01
LGG	DNAJB5_RI_2.4_2.3_2.5	DNAJB5	RI	2.4	2.3	2.5	4.4E-14	0.128	-0.285	2.1E-03	7.1E-04	7.5E-03
LGG	DNAJC4_RI_7.2_7.1_7.3	DNAJC4	RI	7.2	7.1	7.3	3.5E-37	0.296	0.200	1.8E-04	7.6E-05	5.8E-02
LGG	DNASE1_AA_4.1:4.2_3.2_4.3	DNASE1	AA	4.1:4.2	3.2	4.3	6.2E-12	0.108	0.172	7.2E-01	3.9E-01	9.3E-01
LGG	DNM1_ME_10|11_9_12	DNM1	ME	10|11	9	12	2.9E-13	0.121	-0.518	3.6E-03	3.0E-05	8.8E-05
LGG	DNM1L_ES_17_16_18	DNM1L	ES	17	16	18	5.7E-16	0.142	0.388	1.3E-03	2.9E-06	8.0E-05

cancer type	id	Gene Symbol	splice_type	Exon	From.Exon	To.Exon	anova.p	adj.r2	r	p.50	p.25	p.10
LGG	DNM1L_ES_17:18_16_19	DNM1L	ES	17:18	16	19	4.0E-12	0.110	0.240	1.6E-03	3.9E-05	3.2E-04
LGG	DNM1L_ES_18_16_19	DNM1L	ES	18	16	19	8.0E-11	0.101	-0.182	3.3E-02	4.9E-01	7.8E-01
LGG	DNM2_ES_15_14_17	DNM2	ES	15	14	17	1.9E-48	0.365	0.069	2.9E-03	4.6E-02	2.1E-02
LGG	DNM2_ME_10|11_9_12	DNM2	ME	10|11	9	12	2.9E-32	0.263	0.022	1.7E-04	2.8E-05	2.3E-03
LGG	DOCK10_ES_50_49_51	DOCK10	ES	50	49	51	1.2E-13	0.125	-0.211	4.4E-03	2.1E-04	5.9E-03
LGG	DOCK3_ES_34_33_35	DOCK3	ES	34	33	35	1.7E-11	0.125	0.617	3.0E-01	3.5E-01	6.2E-01
LGG	DOCK7_ES_24_23_25	DOCK7	ES	24	23	25	9.9E-18	0.168	-0.336	2.5E-01	1.7E-01	2.7E-04
LGG	DPH7_ES_2_1_3	DPH7	ES	2	1	3	5.7E-13	0.119	-0.102	1.0E-04	1.1E-02	1.2E-01
LGG	DRAM2_ES_6_4_7	DRAM2	ES	6	4	7	7.0E-12	0.122	0.216	1.9E-01	4.4E-01	9.7E-01
LGG	DST_ES_104_103_105	DST	ES	104	103	105	1.7E-14	0.129	-0.070	8.4E-02	7.9E-02	1.0E-02
LGG	DTNA_ES_19_17_22	DTNA	ES	19	17	22	2.2E-33	0.271	0.250	3.8E-03	3.4E-02	6.0E-04
LGG	DTNB_ES_12_11_13	DTNB	ES	12	11	13	6.5E-24	0.203	-0.571	2.3E-03	6.2E-04	1.1E-02
LGG	DTNBP1_ES_2_1_3	DTNBP1	ES	2	1	3	2.4E-15	0.138	-0.169	2.0E-07	2.9E-06	7.5E-05
LGG	DTX2_ES_7_6_8	DTX2	ES	7	6	8	2.9E-19	0.168	0.485	4.5E-04	7.2E-04	6.1E-04
LGG	DTX3_AD_1.2:1.3_1.1_1.5	DTX3	AD	1.2:1.3	1.1	1.5	1.5E-11	0.105	0.232	4.9E-06	1.3E-08	1.5E-03
LGG	DTX3_ES_1.3_1.1_1.5	DTX3	ES	1.3	1.1	1.5	6.0E-31	0.254	-0.021	8.6E-05	4.4E-08	7.9E-07
LGG	DTYMK_ES_3_2_4	DTYMK	ES	3	2	4	2.2E-39	0.310	0.220	2.0E-06	4.3E-07	9.5E-04
LGG	DZIP1_ES_6_5_7	DZIP1	ES	6	5	7	6.0E-23	0.202	-0.157	2.7E-03	2.1E-04	6.6E-02
LGG	E2F6_ES_4_3_5.1	E2F6	ES	4	3	5.1	1.0E-11	0.109	0.051	2.8E-02	2.1E-02	3.4E-01
LGG	EAF1_ES_3_2_4	EAF1	ES	3	2	4	5.4E-19	0.169	-0.196	5.7E-01	5.6E-02	5.6E-02
LGG	EAF2_ES_2_1_3	EAF2	ES	2	1	3	5.1E-28	0.237	-0.086	9.1E-04	1.7E-05	6.7E-04
LGG	EARS2_RI_11.2_11.1_11.3	EARS2	RI	11.2	11.1	11.3	9.4E-18	0.156	0.137	2.0E-02	1.3E-01	5.6E-02
LGG	EBPL_ES_2_1_6	EBPL	ES	2	1	6	1.3E-08	0.101	-0.189	2.9E-04	9.6E-05	1.4E-02
LGG	EBPL_ES_4.1:4.2_1_6	EBPL	ES	4.1:4.2	1	6	9.1E-11	0.101	-0.267	6.0E-02	1.5E-01	8.5E-01
LGG	ECHDC1_ES_1.2:4_1.1_6.1	ECHDC1	ES	1.2:4	1.1	6.1	1.6E-18	0.172	-0.111	1.5E-03	1.3E-05	1.5E-10
LGG	ECM2_AA_4.1_3.1_4.2	ECM2	AA	4.1	3.1	4.2	2.1E-28	0.286	-0.518	1.4E-02	1.5E-02	8.3E-02
LGG	EEF1A1_ES_1.4:2:3:4:5_1.3_6	EEF1A1	ES	1.4:2:3:4:5	1.3	6	1.6E-14	0.130	-0.284	2.0E-02	8.0E-04	7.6E-03
LGG	EEF1B2_AA_1.3_1.1_1.4	EEF1B2	AA	1.3	1.1	1.4	2.5E-36	0.290	0.665	4.1E-08	4.1E-06	3.8E-05
LGG	EEF1D_ES_5:7.2:8.1_1_8.2	EEF1D	ES	5:7.2:8.1	1	8.2	5.7E-43	0.332	-0.137	1.0E-03	1.4E-04	3.2E-04
LGG	EEF1D_ES_8.3:10.1:11:12.1:12.2:13.1_8.	EEF1D	ES	.0.1:11:12.1:12.2	8.2	13.2	1.7E-20	0.179	0.213	3.3E-01	1.1E-01	3.0E-02
LGG	EEF1D_ES_8.3:9:10.1:11:12.1:12.2:13.1_	EEF1D	ES	10.1:11:12.1:12.2	8.2	13.2	2.4E-11	0.103	0.204	2.1E-01	2.0E-01	1.2E-01
LGG	EEF1D_ME_5|6_1_7.2	EEF1D	ME	5|6	1	7.2	6.1E-65	0.455	0.018	2.1E-06	1.1E-05	3.3E-03
LGG	EFCAB2_ES_2_1.3_3	EFCAB2	ES	2	1.3	3	3.1E-17	0.153	0.032	6.3E-02	3.9E-03	5.4E-03
LGG	EFCAB2_ES_2_1.3_4	EFCAB2	ES	2	1.3	4	3.5E-19	0.216	-0.083	1.7E-01	3.6E-02	1.8E-01
LGG	EFCAB2_ES_2:3_1.3_4	EFCAB2	ES	2:03	1.3	4	1.1E-18	0.165	-0.045	7.1E-05	1.2E-05	1.6E-02
LGG	EFEMP1_ES_2.2_1_3	EFEMP1	ES	2.2	1	3	3.9E-15	0.137	-0.031	8.7E-03	2.7E-02	6.3E-02
LGG	EIF2B4_RI_1.3_1.2_1.4	EIF2B4	RI	1.3	1.2	1.4	6.5E-10	0.102	-0.006	7.3E-02	1.4E-01	1.9E-01
LGG	EIF2D_ES_9_8_11	EIF2D	ES	9	8	11	3.1E-10	0.100	0.100	8.8E-01	3.9E-01	5.7E-01
LGG	EIF4G1_ES_2.2_1_2.4	EIF4G1	ES	2.2	1	2.4	6.8E-17	0.157	0.087	1.3E-01	4.5E-02	2.9E-01
LGG	EIF4G1_ES_2.2:2.3:2.4:3.2:5_1_6	EIF4G1	ES	2.2:2.3:2.4:3.2:5	1	6	2.1E-11	0.120	0.103	2.4E-01	2.1E-01	9.0E-03

cancer type	id	Gene Symbol	splice_type	Exon	From.Exon	To.Exon	anova.p	adj.r2	r	p.50	p.25	p.10
LGG	EIF4G1_ES_2.2:2.4:3.1:3.2_1_5	EIF4G1	ES	2.2:2.4:3.1:3.2	1	5	9.7E-11	0.122	0.093	3.8E-02	2.9E-03	3.6E-01
LGG	EIF4G1_ES_2.2:2.4:3.1:3.2:5_1_6	EIF4G1	ES	2.2:2.4:3.1:3.2:5	1	6	9.5E-17	0.167	0.159	3.5E-02	2.0E-02	2.1E-01
LGG	EIF4G1_ES_2.2:2.4:3.2_1_5	EIF4G1	ES	2.2:2.4:3.2	1	5	2.3E-16	0.149	0.069	1.6E-02	1.3E-01	9.9E-02
LGG	ELMOD3_ES_2.1:2.3_1_2.9	ELMOD3	ES	2.1:2.3	1	2.9	1.4E-08	0.101	0.097	1.3E-05	4.6E-05	6.7E-03
LGG	ELN_ES_23_21_24.2	ELN	ES	23	21	24.2	1.3E-18	0.163	0.278	3.2E-02	2.5E-02	9.7E-02
LGG	ELN_ES_32_31_33	ELN	ES	32	31	33	2.1E-22	0.192	0.120	1.5E-04	2.2E-04	9.9E-04
LGG	EML4_ES_4_3_5	EML4	ES	4	3	5	3.5E-27	0.237	0.316	2.7E-05	1.2E-07	2.2E-05
LGG	ENTPD6_ES_2_1_3.1	ENTPD6	ES	2	1	3.1	6.1E-33	0.272	0.020	1.8E-02	1.3E-03	6.3E-04
LGG	EPB41L1_ES_18_17_19	EPB41L1	ES	18	17	19	1.7E-20	0.178	0.385	6.3E-03	4.3E-05	3.5E-03
LGG	EPB41L1_ES_20_19_21	EPB41L1	ES	20	19	21	7.6E-17	0.149	0.401	6.4E-02	2.7E-02	2.7E-02
LGG	EPB41L1_ES_22.1:22.2_21_23	EPB41L1	ES	22.1:22.2	21	23	7.5E-13	0.116	0.293	3.2E-01	4.6E-01	6.0E-01
LGG	EPB41L1_ES_22.2_21_23	EPB41L1	ES	22.2	21	23	5.6E-13	0.117	0.351	4.9E-01	9.5E-01	3.5E-01
LGG	EPB41L1_ES_7_6_8	EPB41L1	ES	7	6	8	1.7E-12	0.121	0.493	2.1E-02	4.4E-01	3.6E-01
LGG	EPB41L2_AD_20.2_20.1_21	EPB41L2	AD	20.2	20.1	21	1.2E-39	0.311	-0.010	2.9E-08	8.6E-10	3.4E-05
LGG	EPB41L2_ES_17:18_14_19	EPB41L2	ES	17:18	14	19	1.6E-11	0.111	-0.279	6.0E-03	2.3E-02	4.1E-03
LGG	EPB41L2_ES_17:18:19_14_20.1	EPB41L2	ES	17:18:19	14	20.1	3.6E-12	0.111	-0.355	9.0E-01	1.9E-01	3.7E-02
LGG	EPB41L2_ES_17:18:19:20.1:20.2_14_21	EPB41L2	ES	.7:18:19:20.1:20.[illegible]	14	21	1.1E-12	0.118	-0.287	8.0E-02	7.9E-04	3.3E-03
LGG	EPB41L2_ES_17:18:19:20.1:20.2:21:22_1	EPB41L2	ES	.8:19:20.1:20.2:2[illegible]	14	23	2.0E-11	0.115	-0.131	5.5E-03	6.9E-03	6.1E-02
LGG	EPB41L2_ES_17:18:20.1:20.2_14_21	EPB41L2	ES	17:18:20.1:20.2	14	21	6.5E-16	0.144	-0.277	1.7E-04	8.4E-04	1.1E-02
LGG	EPB41L2_ES_17:18:20.1:20.2:21:22_14_	EPB41L2	ES	:18:20.1:20.2:21:	14	23	1.8E-25	0.222	-0.189	2.3E-06	5.0E-05	4.3E-03
LGG	EPB41L2_ES_17:18:20.1:21:22_14_23	EPB41L2	ES	17:18:20.1:21:22	14	23	7.6E-13	0.121	-0.219	2.0E-03	2.9E-02	1.9E-01
LGG	EPB41L2_ES_19:21:22_14_23	EPB41L2	ES	19:21:22	14	23	1.2E-13	0.134	-0.053	1.2E-01	8.3E-02	2.6E-01
LGG	EPB41L2_ES_20.1:20.2_14_21	EPB41L2	ES	20.1:20.2	14	21	2.8E-16	0.144	0.127	3.0E-04	2.7E-03	1.7E-02
LGG	EPB41L2_ES_20.1:20.2:21_14_22	EPB41L2	ES	20.1:20.2:21	14	22	5.7E-15	0.134	0.009	3.0E-01	1.3E-01	9.5E-03
LGG	EPB41L2_ES_20.1:20.2:21:22_14_23	EPB41L2	ES	20.1:20.2:21:22	14	23	2.6E-50	0.376	0.146	6.0E-04	7.0E-05	4.9E-02
LGG	EPB41L2_ES_20.1:21:22_14_23	EPB41L2	ES	20.1:21:22	14	23	6.6E-31	0.253	0.132	7.3E-01	4.3E-01	1.3E-01
LGG	EPB41L2_ES_21:22_14_23	EPB41L2	ES	21:22	14	23	3.3E-25	0.214	0.028	5.1E-01	1.6E-01	1.1E-01
LGG	EPB41L2_ES_22_14_23	EPB41L2	ES	22	14	23	1.3E-21	0.196	0.121	4.8E-04	1.2E-02	1.5E-02
LGG	EPB41L3_ES_25_24_26	EPB41L3	ES	25	24	26	1.0E-44	0.343	0.040	1.5E-05	5.1E-06	4.1E-02
LGG	EPHA4_AD_17.2_17.1_18	EPHA4	AD	17.2	17.1	18	5.8E-19	0.207	0.141	1.8E-02	6.9E-03	1.3E-01
LGG	EPN2_ES_3_2.3_4	EPN2	ES	3	2.3	4	5.8E-14	0.126	0.138	3.5E-04	8.4E-05	3.2E-03
LGG	EPS8L2_RI_4.2_4.1_4.3	EPS8L2	RI	4.2	4.1	4.3	9.1E-15	0.151	-0.176	5.5E-03	9.3E-05	2.0E-03
LGG	ERC1_ES_7_6_8	ERC1	ES	7	6	8	1.8E-10	0.102	-0.041	1.6E-01	2.0E-01	7.3E-01
LGG	ERGIC3_ES_10_8_12	ERGIC3	ES	10	8	12	5.8E-27	0.226	-0.441	1.9E-04	1.2E-04	4.8E-03
LGG	ERGIC3_ME_10|11_8_12	ERGIC3	ME	10|11	8	12	1.4E-36	0.293	-0.355	1.5E-05	8.8E-05	3.4E-04
LGG	ETV1_ES_5_4_6	ETV1	ES	5	4	6	3.8E-16	0.147	-0.024	4.6E-01	3.5E-01	4.4E-01
LGG	EVI5_ES_12_11_13	EVI5	ES	12	11	13	4.1E-31	0.279	-0.341	2.3E-02	6.5E-04	9.8E-06
LGG	EVI5L_ES_12_11_13	EVI5L	ES	12	11	13	6.6E-16	0.141	0.483	9.9E-03	2.9E-03	1.2E-03
LGG	EXOC7_ES_7:8.2_6_9	EXOC7	ES	07:08.2	6	9	1.7E-15	0.138	0.099	5.8E-03	1.1E-02	7.5E-03
LGG	EXOSC3_ES_3_2_4.1	EXOSC3	ES	3	2	4.1	7.9E-62	0.438	0.201	1.5E-03	2.2E-03	5.6E-04

cancer type	id	Gene Symbol	splice_type	Exon	From.Exon	To.Exon	anova.p	adj.r2	r	p.50	p.25	p.10
LGG	FAM104A_ES_4_3.2_5	FAM104A	ES	4	3.2	5	1.6E-23	0.200	-0.182	2.5E-01	4.6E-02	7.1E-02
LGG	FAM122B_ES_3_2_4	FAM122B	ES	3	2	4	1.5E-16	0.149	0.116	4.1E-02	1.4E-02	8.0E-02
LGG	FAM13A_AA_16.1_15_16.2	FAM13A	AA	16.1	15	16.2	1.3E-33	0.279	0.306	2.7E-05	1.5E-06	2.3E-05
LGG	FAM13B_ES_14_13_15	FAM13B	ES	14	13	15	2.0E-21	0.199	-0.202	2.1E-04	9.6E-05	2.5E-03
LGG	FAM156B_AD_2.2_2.1_2.5	FAM156B	AD	2.2	2.1	2.5	3.4E-55	0.404	0.206	2.8E-04	4.5E-06	2.3E-05
LGG	FAM156B_RI_2.3:2.4_2.2_2.5	FAM156B	RI	2.3:2.4	2.2	2.5	9.9E-21	0.179	0.173	9.6E-08	4.6E-07	3.6E-05
LGG	FAM168A_ES_4_3_5	FAM168A	ES	4	3	5	3.7E-31	0.258	0.180	3.5E-06	2.8E-07	6.8E-04
LGG	FAM211B_ES_2_1_3	FAM211B	ES	2	1	3	1.0E-23	0.202	0.121	8.4E-02	1.1E-02	1.3E-01
LGG	FAM211B_ES_4_3_5	FAM211B	ES	4	3	5	1.0E-70	0.482	0.362	8.6E-01	4.4E-01	7.0E-01
LGG	FAM219A_AD_2.2_2.1_3	FAM219A	AD	2.2	2.1	3	1.8E-11	0.105	-0.329	1.4E-03	9.4E-05	9.7E-03
LGG	FAM21A_ES_28_27_29	FAM21A	ES	28	27	29	2.8E-39	0.309	-0.126	6.7E-05	3.5E-06	8.6E-06
LGG	FAM221A_ES_2:3.1:3.2:3.3_1_4	FAM221A	ES	2:3.1:3.2:3.3	1	4	2.7E-16	0.144	-0.622	7.9E-03	5.6E-02	3.1E-02
LGG	FAM221A_ES_3.1:3.2:3.3_2_4	FAM221A	ES	3.1:3.2:3.3	2	4	1.4E-21	0.186	-0.681	4.8E-04	7.5E-04	1.1E-03
LGG	FAM228B_ES_2.2_1_3	FAM228B	ES	2.2	1	3	3.0E-20	0.182	0.056	4.2E-03	2.3E-02	1.5E-01
LGG	FAM228B_ES_5:6:7:8_3_9.1	FAM228B	ES	5:6:7:8	3	9.1	4.1E-12	0.112	-0.019	2.5E-02	4.9E-03	1.7E-01
LGG	FAM3A_ES_7.1:7.2_6_8	FAM3A	ES	7.1:7.2	6	8	7.8E-15	0.132	0.138	3.7E-02	2.8E-03	3.3E-02
LGG	FAM60A_ES_3_1_4	FAM60A	ES	3	1	4	3.0E-14	0.143	-0.245	3.5E-01	2.7E-01	8.5E-02
LGG	FAM86B1_AA_3.1_1.2_3.2	FAM86B1	AA	3.1	1.2	3.2	2.6E-15	0.136	-0.132	5.0E-05	1.5E-02	5.0E-02
LGG	FAM86B1_AD_7.2:7.3_7.1_8.1	FAM86B1	AD	7.2:7.3	7.1	8.1	1.2E-58	0.422	-0.478	2.1E-06	1.0E-06	6.9E-06
LGG	FAM86B1_ES_1.2:3.1_1.1_3.2	FAM86B1	ES	1.2:3.1	1.1	3.2	2.1E-27	0.237	-0.538	7.1E-02	8.9E-04	8.8E-06
LGG	FAM86B1_ES_4:5:6_3.2_7.1	FAM86B1	ES	4:05:06	3.2	7.1	5.0E-13	0.137	-0.281	2.6E-03	3.6E-03	4.0E-04
LGG	FAM86B1_ES_4:5:6:7.1_3.2_8.1	FAM86B1	ES	4:5:6:7.1	3.2	8.1	4.6E-11	0.122	0.383	4.0E-03	3.2E-03	3.3E-01
LGG	FAM86B1_ES_4:5:6:7.1:7.2:7.3_3.2_8.1	FAM86B1	ES	4:5:6:7.1:7.2:7.3	3.2	8.1	2.2E-16	0.148	-0.108	1.3E-03	6.1E-04	4.1E-02
LGG	FAM86B1_ES_5_4_6	FAM86B1	ES	5	4	6	1.2E-12	0.121	-0.146	5.4E-03	1.8E-03	8.6E-02
LGG	FAM86B1_ES_5:6_4_7.1	FAM86B1	ES	5:06	4	7.1	4.4E-24	0.232	-0.439	6.4E-01	9.4E-03	8.5E-03
LGG	FAM86B1_ES_5:6:7.1:7.2:7.3_4_8.1	FAM86B1	ES	5:6:7.1:7.2:7.3	4	8.1	4.4E-19	0.169	-0.091	8.4E-03	9.7E-03	1.6E-03
LGG	FANCI_ES_1.2:2:3_1.1_4	FANCI	ES	1.2:2:3	1.1	4	5.0E-10	0.104	-0.537	5.4E-03	9.4E-05	2.1E-02
LGG	FAT3_ES_26:27_25_28	FAT3	ES	26:27:00	25	28	6.9E-10	0.107	-0.210	6.9E-01	1.5E-01	1.1E-01
LGG	FBLN5_ES_7_5_8	FBLN5	ES	7	5	8	1.9E-15	0.137	-0.733	4.6E-03	1.0E-03	2.5E-03
LGG	FBXO4_ES_7_6.1_8	FBXO4	ES	7	6.1	8	1.5E-26	0.223	-0.006	5.4E-03	5.6E-02	8.7E-02
LGG	FBXW11_ES_2_1_4	FBXW11	ES	2	1	4	2.5E-17	0.160	0.137	9.6E-04	1.4E-03	5.1E-03
LGG	FBXW11_ES_2:3_1_4	FBXW11	ES	2:03	1	4	4.4E-16	0.162	0.123	2.6E-04	2.8E-04	2.2E-05
LGG	FBXW11_ES_2:3:4_1_5	FBXW11	ES	2:03:04	1	5	3.8E-12	0.123	0.079	4.3E-02	1.8E-03	3.6E-02
LGG	FBXW11_ES_3_1_4	FBXW11	ES	3	1	4	2.4E-13	0.135	0.127	5.2E-02	7.0E-05	1.3E-04
LGG	FDPS_ES_1.2:2:3.1:3.2_1.1_4	FDPS	ES	1.2:2:3.1:3.2	1.1	4	1.6E-11	0.137	0.348	1.6E-01	1.2E-02	3.2E-03
LGG	FDPS_ES_1.2:3.1:3.2_1.1_4	FDPS	ES	1.2:3.1:3.2	1.1	4	8.5E-18	0.200	0.368	5.5E-02	3.5E-02	2.1E-01
LGG	FERMT2_ES_14_13_15	FERMT2	ES	14	13	15	5.3E-19	0.167	0.049	1.9E-02	5.1E-04	1.4E-03
LGG	FEZ2_ES_8_7_9	FEZ2	ES	8	7	9	5.3E-48	0.362	-0.136	3.6E-06	4.2E-09	1.6E-08
LGG	FGF11_RI_3.2:3.3_3.1_3.4	FGF11	RI	3.2:3.3	3.1	3.4	9.0E-24	0.207	-0.065	2.6E-04	6.7E-10	1.9E-05
LGG	FGFR1_ES_5:6_4_8.2	FGFR1	ES	5:06	4	8.2	1.5E-13	0.143	0.118	2.2E-04	1.2E-01	1.0E-01

cancer type	id	Gene Symbol	splice_type	Exon	From.Exon	To.Exon	anova.p	adj.r2	r	p.50	p.25	p.10
LGG	FGFR1_ES_6_4_8.2	FGFR1	ES	6	4	8.2	4.7E-23	0.198	0.103	6.2E-04	7.0E-04	4.9E-03
LGG	FGFR1OP_ES_7_6_8	FGFR1OP	ES	7	6	8	5.7E-22	0.195	0.075	2.4E-01	4.9E-01	8.5E-01
LGG	FHL1_ES_9_8_10	FHL1	ES	9	8	10	8.4E-13	0.115	-0.316	2.7E-02	2.2E-01	2.8E-01
LGG	FIP1L1_ES_14_13.1_15	FIP1L1	ES	14	13.1	15	8.6E-45	0.343	-0.099	2.9E-06	4.4E-09	4.8E-09
LGG	FIP1L1_ES_2_1_3	FIP1L1	ES	2	1	3	3.9E-25	0.214	-0.201	5.3E-02	5.6E-01	1.0E-01
LGG	FIP1L1_ES_9_8_10	FIP1L1	ES	9	8	10	2.3E-14	0.129	-0.064	5.9E-02	1.5E-04	4.1E-03
LGG	FIS1_AD_2.2_2.1_4	FIS1	AD	2.2	2.1	4	5.2E-26	0.219	-0.004	3.8E-03	1.3E-05	5.8E-07
LGG	FKBP4_ES_2:3_1_4	FKBP4	ES	2:03	1	4	4.1E-10	0.101	0.000	4.9E-02	2.1E-02	2.0E-03
LGG	FLAD1_ES_2.2:4.1:4.3_1.3_6.1	FLAD1	ES	2.2:4.1:4.3	1.3	6.1	1.3E-13	0.123	0.021	3.3E-01	1.4E-01	1.1E-01
LGG	FLNA_ES_30_29_31	FLNA	ES	30	29	31	6.8E-16	0.141	0.044	1.7E-02	3.4E-02	3.3E-01
LGG	FLNB_ES_32.1:32.2_31_33	FLNB	ES	32.1:32.2	31	33	2.1E-14	0.133	-0.283	5.0E-01	2.4E-01	4.1E-01
LGG	FLNC_ES_31_30_32	FLNC	ES	31	30	32	3.8E-25	0.262	0.442	1.1E-07	1.1E-07	7.0E-08
LGG	FLOT2_ES_3_2_6	FLOT2	ES	3	2	6	2.0E-13	0.122	-0.156	8.9E-02	6.6E-02	9.8E-03
LGG	FLOT2_ES_3:5_2_6	FLOT2	ES	3:05	2	6	9.0E-13	0.118	0.087	9.0E-04	5.2E-03	4.9E-03
LGG	FLOT2_ES_5_3_6	FLOT2	ES	5	3	6	1.7E-29	0.250	0.198	1.5E-04	2.3E-05	3.6E-03
LGG	FMNL1_ES_26_25_27	FMNL1	ES	26	25	27	3.5E-12	0.111	0.101	2.6E-03	4.8E-04	1.7E-01
LGG	FMNL3_ES_26_25_27	FMNL3	ES	26	25	27	6.6E-49	0.368	-0.283	3.9E-02	1.5E-01	6.2E-01
LGG	FMNL3_ES_6_5_7	FMNL3	ES	6	5	7	1.8E-27	0.233	-0.148	1.5E-01	3.7E-01	3.3E-01
LGG	FN1_ES_25_24_26	FN1	ES	25	24	26	1.6E-24	0.210	0.364	8.9E-04	6.1E-04	5.9E-02
LGG	FNBP1_ES_12_10.3_14.2	FNBP1	ES	12	10.3	14.2	5.8E-14	0.128	0.188	3.9E-03	9.1E-04	5.5E-03
LGG	FNBP1L_ES_10:11_9_12	FNBP1L	ES	10:11	9	12	1.5E-12	0.129	-0.376	1.8E-01	4.3E-01	7.4E-01
LGG	FNBP1L_RI_16.2_16.1_16.3	FNBP1L	RI	16.2	16.1	16.3	3.2E-14	0.128	-0.282	2.3E-01	7.2E-02	8.6E-02
LGG	FRMD5_RI_17.2:17.3_17.1_17.4	FRMD5	RI	17.2:17.3	17.1	17.4	7.0E-25	0.211	-0.346	2.4E-02	4.8E-01	3.0E-01
LGG	FRYL_ES_62_60_63	FRYL	ES	62	60	63	4.0E-19	0.170	-0.020	6.3E-04	9.4E-06	4.6E-05
LGG	FSD1L_ES_12_11_14.1	FSD1L	ES	12	11	14.1	1.1E-12	0.142	0.224	5.6E-01	4.7E-01	7.7E-01
LGG	FUBP1_ES_3_2_4	FUBP1	ES	3	2	4	4.5E-31	0.259	0.095	2.0E-02	6.2E-04	1.6E-03
LGG	FUZ_ES_2.1:2.2_1.2_3	FUZ	ES	2.1:2.2	1.2	3	1.1E-68	0.473	-0.132	3.4E-02	3.9E-01	4.9E-01
LGG	FUZ_ES_2.1:2.2:2.3_1.2_3	FUZ	ES	2.1:2.2:2.3	1.2	3	1.9E-56	0.410	-0.175	6.4E-02	1.2E-02	9.1E-02
LGG	FUZ_ES_2.1:2.2:2.3:2.4_1.2_3	FUZ	ES	2.1:2.2:2.3:2.4	1.2	3	9.6E-19	0.168	-0.104	2.0E-01	3.7E-01	8.2E-01
LGG	FUZ_ES_2.2:2.3_1.2_3	FUZ	ES	2.2:2.3	1.2	3	1.2E-40	0.347	-0.081	5.6E-02	3.2E-02	6.0E-01
LGG	FXYD1_RI_10.2_10.1_10.3	FXYD1	RI	10.2	10.1	10.3	1.9E-15	0.143	0.028	4.2E-03	7.9E-04	9.3E-05
LGG	GAB1_ES_8_7_9	GAB1	ES	8	7	9	2.8E-35	0.286	-0.107	5.1E-03	5.4E-07	1.8E-05
LGG	GALM_ES_3_2_4	GALM	ES	3	2	4	1.6E-11	0.112	-0.546	3.9E-03	8.4E-03	7.7E-02
LGG	GDPD2_ES_13_12_14	GDPD2	ES	13	12	14	2.8E-14	0.128	-0.779	1.6E-02	1.1E-03	6.0E-05
LGG	GEMIN8_ES_2_1_3	GEMIN8	ES	2	1	3	3.5E-23	0.198	0.000	2.7E-02	2.1E-01	2.9E-01
LGG	GGCT_ES_4_3_5	GGCT	ES	4	3	5	2.4E-17	0.153	0.117	3.0E-02	4.8E-02	3.1E-02
LGG	GGT1_AA_15.3_14_15.4	GGT1	AA	15.3	14	15.4	5.1E-10	0.102	0.057	2.5E-02	3.2E-02	1.3E-02
LGG	GIGYF2_ES_2_1_3.2	GIGYF2	ES	2	1	3.2	2.4E-20	0.182	-0.142	4.9E-01	8.4E-02	5.6E-04
LGG	GIGYF2_ES_2:3.2_1_5	GIGYF2	ES	02:03.2	1	5	6.2E-11	0.105	-0.175	8.2E-01	6.5E-01	1.7E-01
LGG	GIGYF2_ES_2:3.2:4_1_5	GIGYF2	ES	03:02.2	1	5	7.0E-11	0.125	-0.281	4.9E-01	7.8E-01	1.2E-01

cancer type	id	Gene Symbol	splice_type	Exon	From.Exon	To.Exon	anova.p	adj.r2	r	p.50	p.25	p.10
LGG	GIT1_ES_8_7_9	GIT1	ES	8	7	9	4.7E-11	0.101	0.536	3.1E-02	5.6E-02	1.9E-01
LGG	GLIPR1_RI_5.2_5.1_5.3	GLIPR1	RI	5.2	5.1	5.3	1.5E-13	0.122	-0.614	9.4E-01	2.1E-01	5.1E-02
LGG	GLIPR2_ES_4:5_1_7	GLIPR2	ES	4:05	1	7	3.7E-23	0.210	-0.081	2.3E-02	2.5E-05	2.2E-03
LGG	GLYCTK_ES_3_2_4	GLYCTK	ES	3	2	4	5.3E-27	0.226	0.168	1.8E-04	1.7E-05	6.4E-03
LGG	GMEB1_AA_3.1_2.2_3.2	GMEB1	AA	3.1	2.2	3.2	7.5E-18	0.185	-0.168	2.9E-03	2.3E-04	7.0E-04
LGG	GMPPA_AA_2.1_1.1_2.2	GMPPA	AA	2.1	1.1	2.2	9.8E-21	0.180	0.130	1.1E-05	4.7E-08	8.7E-05
LGG	GMPPA_RI_9.2:9.3_9.1_9.4	GMPPA	RI	9.2:9.3	9.1	9.4	1.2E-18	0.163	-0.385	1.0E-01	2.5E-02	2.6E-02
LGG	GMPR2_RI_2.2_2.1_2.3	GMPR2	RI	2.2	2.1	2.3	7.1E-14	0.125	0.022	1.7E-02	1.6E-02	3.1E-02
LGG	GNAS_ES_6:8.1_5_8.2	GNAS	ES	06:08.1	5	8.2	5.2E-13	0.117	0.183	8.4E-03	2.3E-03	2.7E-01
LGG	GNB1L_RI_1.2_1.1_1.3	GNB1L	RI	1.2	1.1	1.3	5.2E-11	0.100	0.003	2.1E-02	6.1E-03	4.3E-02
LGG	GNB2L1_ES_5:6_3_9	GNB2L1	ES	5:06	3	9	3.1E-12	0.113	0.006	4.1E-01	8.2E-02	8.2E-02
LGG	GOLGA8B_ES_2_1_3	GOLGA8B	ES	2	1	3	1.6E-13	0.148	0.156	3.4E-01	1.2E-01	7.4E-02
LGG	GOLIM4_ES_7_6_8	GOLIM4	ES	7	6	8	1.4E-25	0.217	-0.038	1.4E-01	4.2E-02	8.4E-02
LGG	GOPC_ES_3_2.1_5.1	GOPC	ES	3	2.1	5.1	2.4E-13	0.128	0.089	1.2E-04	3.4E-06	5.3E-04
LGG	GPATCH4_AD_5.2:5.3:5.4_5.1_6	GPATCH4	AD	5.2:5.3:5.4	5.1	6	1.3E-13	0.122	-0.498	3.5E-03	8.8E-06	1.6E-03
LGG	GPBP1_ES_3.1:4_2_6	GPBP1	ES	3.1:4	2	6	9.2E-12	0.108	-0.055	3.3E-02	3.4E-03	3.2E-02
LGG	GPHN_ES_11_10_12	GPHN	ES	11	10	12	1.7E-20	0.180	-0.309	5.4E-06	5.4E-05	7.1E-03
LGG	GPR155_ES_2_1_4	GPR155	ES	2	1	4	2.1E-15	0.173	-0.285	2.3E-04	1.9E-04	3.3E-03
LGG	GPS1_RI_1.2_1.1_1.3	GPS1	RI	1.2	1.1	1.3	2.8E-15	0.141	0.109	6.8E-03	3.9E-03	6.9E-03
LGG	GRAMD1A_ES_19_18_20	GRAMD1A	ES	19	18	20	3.1E-30	0.249	-0.414	5.8E-05	1.4E-04	8.8E-04
LGG	GRB10_ES_10_5.2_11	GRB10	ES	10	5.2	11	4.4E-10	0.107	-0.345	1.1E-01	5.7E-02	6.9E-02
LGG	GRIA1_ME_16|17_15_18	GRIA1	ME	16|17	15	18	2.4E-17	0.155	0.045	3.3E-01	1.4E-01	1.0E-01
LGG	GRIA2_ES_16_15_17	GRIA2	ES	16	15	17	1.0E-22	0.207	-0.059	9.2E-01	5.8E-01	2.6E-01
LGG	GRIA2_ES_17_15_18	GRIA2	ES	17	15	18	1.1E-30	0.255	-0.059	2.0E-01	2.2E-01	5.1E-01
LGG	GRIA2_ES_18_17_19	GRIA2	ES	18	17	19	1.6E-24	0.211	-0.115	6.0E-01	6.4E-01	5.8E-01
LGG	GRIA3_ES_19_18_20	GRIA3	ES	19	18	20	1.6E-15	0.148	-0.167	1.0E-01	8.7E-02	1.6E-01
LGG	GRIK2_ES_19_17_20	GRIK2	ES	19	17	20	3.5E-21	0.189	-0.436	1.7E-03	1.7E-05	4.5E-04
LGG	GRIK2_ME_18|19_17_20	GRIK2	ME	18|19	17	20	3.2E-28	0.268	0.536	6.0E-03	3.5E-04	5.8E-03
LGG	GRK6_AA_17.1_15_17.2	GRK6	AA	17.1	15	17.2	3.2E-13	0.119	-0.103	8.2E-04	4.2E-06	1.6E-04
LGG	GRSF1_AD_1.3_1.2_3	GRSF1	AD	1.3	1.2	3	2.1E-17	0.155	-0.288	2.1E-02	6.2E-03	3.9E-02
LGG	GSN_ES_10:15.1:15.2_9_16	GSN	ES	10:15.1:15.2	9	16	2.4E-11	0.103	-0.025	5.6E-01	5.0E-01	9.4E-01
LGG	GSN_ES_10:15.2_9_16	GSN	ES	10:15.2	9	16	8.1E-12	0.118	-0.010	2.3E-01	3.7E-01	1.2E-01
LGG	GSN_ES_15.1:15.2_10_16	GSN	ES	15.1:15.2	10	16	2.2E-19	0.169	0.084	1.9E-01	6.1E-01	3.8E-01
LGG	GSTK1_AD_4.2:4.3_4.1_5	GSTK1	AD	4.2:4.3	4.1	5	2.6E-25	0.214	0.446	5.1E-08	1.6E-07	1.9E-05
LGG	GTF2H2_ES_2_1_3	GTF2H2	ES	2	1	3	1.0E-23	0.222	0.039	2.1E-03	4.9E-05	2.7E-03
LGG	GTF2H2C_ES_2_1.1_3	GTF2H2C	ES	2	1.1	3	2.1E-11	0.109	-0.027	3.1E-03	4.7E-04	2.3E-03
LGG	GTF2H2C_ES_2_1.2_3	GTF2H2C	ES	2	1.2	3	8.9E-12	0.114	0.000	1.8E-05	2.8E-04	5.3E-03
LGG	GTF2I_ES_10_9_11.1	GTF2I	ES	10	9	11.1	2.2E-28	0.237	0.121	5.7E-04	5.3E-04	2.4E-02
LGG	GTF2I_ES_12_11.1_13	GTF2I	ES	12	11.1	13	1.2E-20	0.180	-0.004	8.1E-05	8.4E-08	1.7E-04
LGG	GTF2IRD1_AA_20.1_19_20.2	GTF2IRD1	AA	20.1	19	20.2	2.8E-18	0.160	0.031	1.6E-03	6.6E-06	9.6E-04

cancer type	id	Gene Symbol	splice_type	Exon	From.Exon	To.Exon	anova.p	adj.r2	r	p.50	p.25	p.10
LGG	GTF3C1_AD_34.2_34.1_35	GTF3C1	AD	34.2	34.1	35	2.2E-22	0.192	-0.162	1.6E-03	9.0E-04	1.6E-03
LGG	GUSB_ES_3.1:3.2_2_4.1	GUSB	ES	3.1:3.2	2	4.1	1.8E-15	0.138	0.363	1.6E-02	5.6E-04	1.2E-02
LGG	GYG1_ES_6:7_5_8	GYG1	ES	6:07	5	8	1.9E-19	0.177	-0.259	4.0E-02	2.8E-03	5.4E-03
LGG	GYG1_ES_7_6_8	GYG1	ES	7	6	8	7.7E-16	0.141	-0.414	4.1E-04	1.2E-03	4.6E-03
LGG	GYG2_ES_10_9_12	GYG2	ES	10	9	12	3.6E-30	0.282	0.008	3.9E-03	2.1E-03	1.7E-03
LGG	GYG2_ES_10:11_9_12	GYG2	ES	10:11	9	12	2.4E-46	0.362	0.008	3.1E-05	7.2E-08	1.3E-04
LGG	H2AFY_ME_7|8_6.3_9	H2AFY	ME	7|8	6.3	9	3.0E-12	0.111	0.503	4.6E-02	3.7E-02	1.7E-04
LGG	HAPLN3_ES_5_4_6	HAPLN3	ES	5	4	6	1.9E-13	0.122	-0.141	9.7E-03	2.2E-05	2.3E-03
LGG	HAUS4_AD_1.3_1.2_2	HAUS4	AD	1.3	1.2	2	2.6E-12	0.111	0.047	2.2E-03	2.0E-02	2.4E-01
LGG	HAUS7_ES_12_11.2_13	HAUS7	ES	12	11.2	13	2.0E-12	0.112	0.192	8.7E-03	1.2E-01	1.5E-01
LGG	HDAC6_AD_2.2:2.3_2.1_3	HDAC6	AD	2.2:2.3	2.1	3	4.4E-14	0.139	-0.204	1.1E-02	1.1E-03	3.3E-03
LGG	HDHD2_ES_3_1_4.1	HDHD2	ES	3	1	4.1	5.1E-12	0.110	-0.115	1.4E-02	8.0E-02	1.7E-01
LGG	HEMK1_ES_7:8_6_9.1	HEMK1	ES	7:08	6	9.1	1.6E-26	0.226	0.045	5.5E-03	7.2E-04	3.7E-03
LGG	HES6_AD_1.2:1.3_1.1_2.1	HES6	AD	1.2:1.3	1.1	2.1	1.0E-24	0.239	-0.190	7.1E-02	5.7E-02	7.5E-02
LGG	HES6_RI_2.3:2.4_2.2_2.5	HES6	RI	2.3:2.4	2.2	2.5	3.3E-27	0.228	-0.019	4.4E-07	1.6E-07	4.3E-06
LGG	HHLA3_AD_1.2:1.3_1.1_3	HHLA3	AD	1.2:1.3	1.1	3	3.7E-15	0.135	-0.110	2.1E-05	5.6E-05	5.9E-04
LGG	HHLA3_AD_1.3_1.2_3	HHLA3	AD	1.3	1.2	3	2.8E-11	0.102	-0.157	9.3E-04	1.8E-04	2.5E-04
LGG	HHLA3_ES_4_3_5	HHLA3	ES	4	3	5	1.8E-18	0.162	0.184	8.5E-03	3.7E-03	4.1E-02
LGG	HIPK2_AA_8.1_7_8.2	HIPK2	AA	8.1	7	8.2	6.0E-29	0.248	-0.062	2.1E-02	4.4E-01	4.3E-01
LGG	HLTF_RI_25.2_25.1_25.3	HLTF	RI	25.2	25.1	25.3	2.7E-14	0.128	0.186	7.7E-01	1.1E-01	1.1E-01
LGG	HM13_ES_11:12.1_10_13	HM13	ES	11:12.1	10	13	3.4E-19	0.177	-0.330	2.1E-02	1.5E-03	3.9E-04
LGG	HM13_ES_11:12.1:12.2_10_13	HM13	ES	11:12.1:12.2	10	13	3.2E-24	0.230	-0.296	1.3E-02	1.2E-02	1.6E-02
LGG	HM13_ES_12.1:12.2_10_13	HM13	ES	12.1:12.2	10	13	9.4E-14	0.159	-0.377	5.7E-02	8.0E-03	1.7E-02
LGG	HM13_ES_12.1:12.2_11_13	HM13	ES	12.1:12.2	11	13	5.0E-14	0.126	-0.459	2.0E-02	1.0E-03	6.3E-04
LGG	HMGCL_ES_7:8_6_9	HMGCL	ES	7:08	6	9	8.7E-39	0.306	0.129	2.6E-08	1.7E-11	2.7E-08
LGG	HNRNPA2B1_ES_12.2_11_12.4	HNRNPA2B1	ES	12.2	11	12.4	1.1E-26	0.224	-0.086	2.5E-01	1.8E-02	2.5E-01
LGG	HNRNPR_ES_2.2_1_3	HNRNPR	ES	2.2	1	3	1.0E-31	0.261	-0.267	3.4E-04	1.2E-03	5.8E-04
LGG	HOGA1_ES_2:3:4:5_1_6	HOGA1	ES	2:3:4:5	1	6	1.4E-15	0.142	-0.553	5.1E-03	2.0E-03	2.1E-02
LGG	HOOK2_ES_18_17_19	HOOK2	ES	18	17	19	6.3E-49	0.371	0.024	3.9E-05	1.7E-05	6.4E-02
LGG	HP1BP3_ES_3_1_4	HP1BP3	ES	3	1	4	3.2E-15	0.144	0.364	3.3E-03	1.5E-04	8.0E-05
LGG	HSBP1L1_ES_2.2:3.1_2.1_3.2	HSBP1L1	ES	2.2:3.1	2.1	3.2	2.2E-21	0.185	-0.562	7.2E-04	1.0E-04	8.7E-04
LGG	HSCB_ES_2.1:2.2:3.1:4_1_5	HSCB	ES	2.1:2.2:3.1:4	1	5	3.1E-11	0.103	0.051	1.3E-04	2.0E-04	5.7E-02
LGG	HSCB_ES_2.1:3.1:4_1_5	HSCB	ES	2.1:3.1:4	1	5	6.3E-16	0.147	0.101	1.2E-02	5.2E-02	1.4E-02
LGG	HSD11B1L_AA_4.1_1_4.2	HSD11B1L	AA	4.1	1	4.2	4.4E-14	0.126	0.062	1.2E-03	1.5E-04	3.8E-03
LGG	HSD11B1L_ES_5.2:5.3:7_4.2_8	HSD11B1L	ES	5.2:5.3:7	4.2	8	2.7E-22	0.191	-0.058	3.1E-02	1.6E-02	3.8E-02
LGG	HSD11B1L_ES_5.3:7_4.2_8	HSD11B1L	ES	5.3:7	4.2	8	2.3E-12	0.116	0.009	3.6E-01	5.4E-01	6.2E-01
LGG	HSD11B1L_ES_7_5.3_8	HSD11B1L	ES	7	5.3	8	5.7E-12	0.108	-0.214	7.0E-02	1.0E-02	2.7E-02
LGG	HSF4_ES_14.1_13_14.3	HSF4	ES	14.1	13	14.3	5.6E-19	0.172	0.130	4.3E-01	3.4E-01	3.8E-01
LGG	ICAM3_RI_3.2_3.1_3.3	ICAM3	RI	3.2	3.1	3.3	1.4E-19	0.170	-0.474	2.5E-02	9.1E-04	6.2E-04
LGG	ICMT_ES_3_2_4	ICMT	ES	3	2	4	5.4E-28	0.238	-0.339	5.1E-09	2.4E-09	4.9E-05

cancer type	id	Gene Symbol	splice_type	Exon	From.Exon	To.Exon	anova.p	adj.r2	r	p.50	p.25	p.10
LGG	IDI1_ES_2_1_3	IDI1	ES	2	1	3	1.4E-32	0.266	-0.228	2.7E-02	1.8E-04	4.7E-03
LGG	IDNK_ES_4_1.2_5	IDNK	ES	4	1.2	5	1.1E-13	0.124	-0.261	1.4E-04	2.6E-04	1.1E-03
LGG	IFFO1_ES_3_1_4	IFFO1	ES	3	1	4	1.2E-21	0.186	0.029	3.1E-01	6.9E-01	3.7E-02
LGG	IFI16_ES_9_7_10	IFI16	ES	9	7	10	1.1E-11	0.106	-0.169	2.3E-01	3.6E-01	4.6E-01
LGG	IFI44_ES_7.1_6_8	IFI44	ES	7.1	6	8	1.0E-10	0.102	0.104	6.9E-02	5.8E-02	2.5E-01
LGG	IFI44_ES_8_6_9	IFI44	ES	8	6	9	1.8E-10	0.123	0.295	8.4E-01	6.9E-01	3.7E-01
LGG	IFT46_ES_4_3_5	IFT46	ES	4	3	5	1.3E-11	0.105	0.028	3.8E-03	2.2E-03	1.5E-04
LGG	IFT88_ES_27_26_28	IFT88	ES	27	26	28	3.3E-70	0.506	0.128	1.1E-05	2.8E-05	6.0E-04
LGG	IFT88_ES_5_4_6	IFT88	ES	5	4	6	3.5E-19	0.171	0.309	1.4E-01	3.7E-03	6.5E-03
LGG	IL18BP_RI_1.7_1.6_1.8	IL18BP	RI	1.7	1.6	1.8	4.2E-13	0.118	-0.366	2.5E-03	5.3E-03	1.2E-02
LGG	IL18BP_RI_4.3_4.2_4.4	IL18BP	RI	4.3	4.2	4.4	4.3E-16	0.143	-0.204	4.3E-02	3.1E-03	7.4E-02
LGG	ILDR2_ES_4_3_5	ILDR2	ES	4	3	5	1.3E-14	0.154	0.083	3.0E-03	2.7E-03	8.8E-04
LGG	INCENP_ES_11_10_12	INCENP	ES	11	10	12	1.0E-11	0.109	-0.332	8.8E-04	1.6E-02	3.9E-01
LGG	ING4_ES_2:3_1_4	ING4	ES	2:03	1	4	2.7E-26	0.222	-0.257	6.6E-03	9.3E-03	5.2E-04
LGG	ING4_ES_3:4_2_5.1	ING4	ES	3:04	2	5.1	3.0E-24	0.206	-0.315	2.4E-01	2.8E-02	2.5E-02
LGG	INO80C_ES_3:4.2:5.1:5.2:6:7_1_8	INO80C	ES	3:4.2:5.1:5.2:6:7	1	8	1.6E-11	0.105	-0.043	1.0E-04	3.2E-04	1.1E-02
LGG	INO80E_ES_6.2:6.3_5_11	INO80E	ES	6.2:6.3	5	11	4.0E-16	0.143	0.117	1.3E-01	1.6E-01	2.5E-01
LGG	INO80E_ES_6.2:6.3:10_5_11	INO80E	ES	6.2:6.3:10	5	11	3.9E-23	0.197	0.173	8.5E-02	6.0E-01	9.7E-01
LGG	INTS12_ES_4_1_5	INTS12	ES	4	1	5	1.8E-16	0.146	0.108	1.3E-04	3.6E-04	2.3E-02
LGG	IP6K2_AA_11.4:11.5:11.6:11.7:11.8_11.2	IP6K2	AA	4:11.5:11.6:11.7:1	11.2	11.9	3.1E-34	0.276	0.289	1.7E-04	1.8E-07	8.8E-07
LGG	IP6K2_AD_11.3:11.4:11.5_11.2_11.9	IP6K2	AD	11.3:11.4:11.5	11.2	11.9	8.3E-33	0.267	0.201	4.7E-06	6.0E-07	1.5E-06
LGG	IP6K2_ES_11.4:11.5_11.2_11.9	IP6K2	ES	11.4:11.5	11.2	11.9	1.2E-42	0.331	0.092	2.0E-03	3.4E-04	2.2E-06
LGG	IRAK4_ES_4:5_3_6	IRAK4	ES	4:05	3	6	6.4E-14	0.139	0.286	2.2E-02	2.5E-02	1.6E-02
LGG	IRF3_AD_1.5:1.6_1.4_2	IRF3	AD	1.5:1.6	1.4	2	5.4E-14	0.125	-0.277	9.1E-01	1.5E-02	5.2E-02
LGG	IRF3_AD_1.6_1.5_2	IRF3	AD	1.6	1.5	2	3.5E-18	0.159	-0.264	3.4E-03	2.9E-03	3.7E-05
LGG	IRF3_ES_1.4:1.5:1.6_1.1_2	IRF3	ES	1.4:1.5:1.6	1.1	2	3.4E-09	0.107	-0.205	3.9E-02	2.7E-01	2.8E-01
LGG	IRF3_ES_1.5:1.6_1.1_2	IRF3	ES	1.5:1.6	1.1	2	8.4E-09	0.101	-0.213	2.5E-02	6.4E-02	5.1E-02
LGG	IRF5_ES_5_4_6.1	IRF5	ES	5	4	6.1	2.0E-21	0.189	-0.603	1.4E-01	1.1E-01	6.0E-01
LGG	IRF7_RI_2.4_2.3_2.5	IRF7	RI	2.4	2.3	2.5	1.5E-12	0.118	0.118	2.2E-01	1.3E-01	1.6E-01
LGG	ISG20L2_RI_1.2_1.1_1.3	ISG20L2	RI	1.2	1.1	1.3	4.6E-14	0.126	0.164	8.3E-01	8.2E-01	8.6E-01
LGG	ITGB3BP_ES_9_8_10	ITGB3BP	ES	9	8	10	1.9E-12	0.113	-0.070	6.7E-03	5.1E-03	3.6E-02
LGG	JKAMP_AD_1.2_1.1_2.1	JKAMP	AD	1.2	1.1	2.1	1.6E-13	0.123	-0.119	3.6E-02	4.7E-03	8.9E-04
LGG	JPH4_ES_2_1_3	JPH4	ES	2	1	3	1.5E-15	0.159	-0.064	5.3E-01	6.8E-01	1.3E-01
LGG	KALRN_ES_40.2_39_41	KALRN	ES	40.2	39	41	1.2E-32	0.273	0.259	4.8E-04	3.4E-07	4.3E-04
LGG	KANSL1_ES_11_10_12	KANSL1	ES	11	10	12	4.6E-25	0.213	-0.238	2.7E-05	6.1E-04	1.7E-03
LGG	KANSL3_ES_18_17_19.1	KANSL3	ES	18	17	19.1	1.6E-56	0.415	-0.447	1.7E-06	9.8E-08	5.1E-06
LGG	KBTBD4_AD_1.2_1.1_2.4	KBTBD4	AD	1.2	1.1	2.4	9.5E-16	0.142	-0.183	1.1E-02	2.1E-02	3.1E-03
LGG	KCTD15_ES_3_2.3_4	KCTD15	ES	3	2.3	4	3.3E-20	0.176	-0.151	9.8E-01	7.6E-01	7.1E-01
LGG	KCTD17_ES_6:7:8_5_9	KCTD17	ES	6:07:08	5	9	3.8E-21	0.182	0.279	6.3E-03	2.9E-04	3.3E-04
LGG	KCTD17_ES_6:8_5_9	KCTD17	ES	6:08	5	9	3.9E-31	0.255	-0.149	3.7E-05	3.0E-05	1.5E-05

cancer type	id	Gene Symbol	splice_type	Exon	From.Exon	To.Exon	anova.p	adj.r2	r	p.50	p.25	p.10
LGG	KCTD17_ES_8_5_9	KCTD17	ES	8	5	9	2.2E-12	0.119	0.381	3.1E-02	2.4E-01	1.3E-01
LGG	KCTD7_ES_6_5_7	KCTD7	ES	6	5	7	6.3E-19	0.178	0.182	2.5E-04	1.8E-04	4.3E-04
LGG	KCTD7_ES_7_5_10	KCTD7	ES	7	5	10	6.7E-22	0.199	0.029	2.7E-03	1.6E-03	1.3E-03
LGG	KCTD9_AA_5.1_4_5.2	KCTD9	AA	5.1	4	5.2	1.6E-10	0.110	-0.067	6.0E-01	5.8E-01	3.0E-01
LGG	KDM4B_ES_13_10_14	KDM4B	ES	13	10	14	9.1E-17	0.149	0.173	2.5E-01	8.0E-01	8.3E-02
LGG	KHK_ES_2:3_1_5	KHK	ES	2:03	1	5	1.3E-19	0.177	-0.061	2.1E-02	1.4E-03	9.4E-02
LGG	KIAA0226_ES_3_2_4	KIAA0226	ES	3	2	4	4.6E-10	0.120	0.072	5.5E-02	2.8E-01	9.3E-02
LGG	KIAA0226_ES_9_8.1_10	KIAA0226	ES	9	8.1	10	1.6E-42	0.341	0.146	5.6E-06	4.8E-06	9.0E-07
LGG	KIAA0895L_ES_2.1_1_3.1	KIAA0895L	ES	2.1	1	3.1	9.0E-17	0.151	0.274	4.7E-01	8.6E-01	9.8E-01
LGG	KIAA1191_ES_2_1_3	KIAA1191	ES	2	1	3	1.4E-19	0.172	-0.103	1.7E-01	2.2E-04	3.8E-04
LGG	KIAA1191_ES_2:4_1_5	KIAA1191	ES	2:04	1	5	3.5E-15	0.149	0.004	2.5E-01	2.5E-01	7.1E-01
LGG	KIAA1191_ES_2:4:5:6_1_7	KIAA1191	ES	2:4:5:6	1	7	1.8E-09	0.106	-0.024	6.3E-01	5.8E-01	7.3E-01
LGG	KIAA1407_ES_2:3_1_4.1	KIAA1407	ES	2:03	1	4.1	4.7E-09	0.103	0.105	7.9E-02	2.0E-02	4.4E-01
LGG	KIAA1598_ES_17:18_16_20.1	KIAA1598	ES	17:18	16	20.1	1.8E-18	0.163	-0.475	4.6E-04	3.7E-04	1.3E-03
LGG	KIDINS220_ES_27_26_29	KIDINS220	ES	27	26	29	1.7E-41	0.328	-0.351	1.3E-07	2.0E-07	9.4E-09
LGG	KIDINS220_ES_27:28_26_29	KIDINS220	ES	27:28:00	26	29	3.7E-36	0.292	-0.368	5.2E-05	8.9E-06	5.8E-04
LGG	KIF21A_ES_12_11_13	KIF21A	ES	12	11	13	1.7E-19	0.172	-0.184	5.2E-06	7.7E-07	3.3E-04
LGG	KIF21A_ES_24_23_25.2	KIF21A	ES	24	23	25.2	1.5E-36	0.297	0.057	8.8E-04	3.2E-02	2.6E-03
LGG	KIF21A_ES_30:32_29_33.2	KIF21A	ES	30:32:00	29	33.2	5.6E-26	0.270	0.043	2.6E-03	1.6E-04	3.8E-04
LGG	KIF2A_ES_19_18_20	KIF2A	ES	19	18	20	2.9E-49	0.373	0.284	1.0E-08	2.9E-09	1.2E-07
LGG	KIF3A_ES_10_9_11	KIF3A	ES	10	9	11	2.6E-11	0.105	0.304	8.6E-02	2.6E-02	1.3E-02
LGG	KIF3A_ES_10:11_9_12	KIF3A	ES	10:11	9	12	2.3E-23	0.207	0.377	1.2E-02	9.9E-06	6.4E-05
LGG	KIF3A_ES_11_9_12	KIF3A	ES	11	9	12	1.3E-18	0.167	0.305	1.0E-02	8.8E-04	7.7E-05
LGG	KIFC3_ES_28_27_29	KIFC3	ES	28	27	29	2.1E-14	0.129	0.065	7.8E-01	8.8E-01	8.4E-01
LGG	KLC1_AD_13.3_13.2_14.1	KLC1	AD	13.3	13.2	14.1	1.6E-33	0.271	-0.582	6.0E-05	4.6E-05	2.1E-05
LGG	KLC1_AD_13.3_13.2_15	KLC1	AD	13.3	13.2	15	3.9E-19	0.167	-0.005	2.3E-01	7.6E-01	2.8E-01
LGG	KLC1_AD_13.3_13.2_18	KLC1	AD	13.3	13.2	18	4.2E-25	0.212	-0.093	6.4E-02	5.0E-02	8.4E-02
LGG	KLC1_ES_13.3:14.1_13.2_14.3	KLC1	ES	13.3:14.1	13.2	14.3	1.3E-90	0.570	-0.082	1.2E-06	5.7E-08	2.2E-05
LGG	KLC1_ES_13.3:14.1:14.2_13.2_14.3	KLC1	ES	13.3:14.1:14.2	13.2	14.3	5.4E-44	0.340	-0.418	3.1E-06	1.4E-06	6.0E-05
LGG	KLC1_ES_13.3:15_13.2_18	KLC1	ES	13.3:15	13.2	18	3.5E-32	0.262	-0.183	2.2E-02	6.5E-03	5.5E-01
LGG	KLC1_ES_13.3:15:16_13.2_18	KLC1	ES	13.3:15:16	13.2	18	7.7E-57	0.412	-0.135	1.7E-07	2.5E-07	1.2E-05
LGG	KLC1_ES_14.1_13.2_14.3	KLC1	ES	14.1	13.2	14.3	1.2E-25	0.217	0.359	2.2E-03	3.2E-03	9.1E-02
LGG	KLC1_ES_15:16_13.3_18	KLC1	ES	15:16	13.3	18	3.3E-27	0.227	-0.066	1.4E-02	8.1E-06	2.6E-05
LGG	KLC1_ES_16_15_18	KLC1	ES	16	15	18	2.6E-29	0.242	0.028	8.1E-03	4.9E-05	1.3E-04
LGG	KLHDC10_ES_2_1_3	KLHDC10	ES	2	1	3	1.3E-16	0.158	-0.099	2.2E-04	8.7E-04	6.0E-03
LGG	KRIT1_ES_3_2.2_4	KRIT1	ES	3	2.2	4	7.8E-48	0.371	0.187	6.6E-03	1.4E-03	1.7E-02
LGG	KSR1_AA_24.1_22_24.2	KSR1	AA	24.1	22	24.2	1.6E-28	0.238	0.360	1.6E-05	7.9E-08	1.2E-05
LGG	KSR1_ES_13_12_15	KSR1	ES	13	12	15	1.0E-17	0.185	0.384	1.6E-02	2.0E-05	6.4E-04
LGG	KTN1_ES_26_25_27	KTN1	ES	26	25	27	6.8E-94	0.582	0.131	4.0E-08	3.1E-08	1.9E-04
LGG	KTN1_ES_42_41_43	KTN1	ES	42	41	43	4.9E-77	0.511	0.001	1.3E-11	6.5E-12	3.0E-08

cancer type	id	Gene Symbol	splice_type	Exon	From.Exon	To.Exon	anova.p	adj.r2	r	p.50	p.25	p.10
LGG	L1CAM_ES_29_28_30	L1CAM	ES	29	28	30	1.3E-20	0.202	-0.175	7.5E-03	1.3E-01	1.1E-01
LGG	L3MBTL2_ES_16_15_17	L3MBTL2	ES	16	15	17	4.7E-13	0.117	-0.157	8.0E-02	2.6E-02	8.0E-02
LGG	LANCL1_AD_1.2:1.3:1.4_1.1_1.6	LANCL1	AD	1.2:1.3:1.4	1.1	1.6	3.0E-11	0.104	0.234	2.6E-01	1.4E-01	6.4E-02
LGG	LARP4_ES_10_9_11	LARP4	ES	10	9	11	7.1E-17	0.153	0.063	1.2E-01	1.0E-01	8.7E-01
LGG	LAS1L_AD_11.2_11.1_12	LAS1L	AD	11.2	11.1	12	3.9E-38	0.302	-0.031	2.6E-04	1.0E-05	3.9E-05
LGG	LAS1L_ES_9_8_10	LAS1L	ES	9	8	10	7.2E-22	0.188	0.164	6.7E-03	6.8E-05	4.1E-03
LGG	LAT_RI_7.2_7.1_7.3	LAT	RI	7.2	7.1	7.3	2.2E-09	0.102	-0.196	2.1E-01	5.6E-01	4.7E-02
LGG	LCA5_RI_8.2_8.1_8.3	LCA5	RI	8.2	8.1	8.3	9.6E-31	0.256	-0.468	8.9E-06	1.9E-06	4.7E-04
LGG	LETMD1_ES_1.3:2_1.2_7	LETMD1	ES	1.3:2	1.2	7	1.1E-16	0.165	-0.348	2.3E-05	5.2E-06	3.1E-05
LGG	LETMD1_ES_1.3:2:3.2:3.3:4:5:6_1.2_7	LETMD1	ES	l.3:2:3.2:3.3:4:5:(	1.2	7	9.2E-12	0.134	-0.338	7.6E-03	3.3E-05	7.9E-04
LGG	LETMD1_ES_1.3:2:3.2:4_1.2_5	LETMD1	ES	1.3:2:3.2:4	1.2	5	2.1E-23	0.214	-0.358	1.3E-03	1.8E-05	5.3E-04
LGG	LETMD1_ES_1.3:2:3.2:4:5:6_1.2_7	LETMD1	ES	1.3:2:3.2:4:5:6	1.2	7	4.4E-16	0.146	-0.331	8.2E-02	3.5E-04	9.6E-03
LGG	LETMD1_ES_2_1.2_7	LETMD1	ES	2	1.2	7	2.4E-27	0.229	-0.381	2.2E-06	1.4E-06	1.5E-04
LGG	LETMD1_ES_2:3.2_1.2_7	LETMD1	ES	02:03.2	1.2	7	2.2E-28	0.266	-0.483	2.9E-07	1.2E-09	2.3E-05
LGG	LETMD1_ES_2:3.2:3.3:4_1.2_5	LETMD1	ES	2:3.2:3.3:4	1.2	5	1.6E-14	0.148	-0.338	2.3E-05	3.6E-05	6.2E-04
LGG	LETMD1_ES_2:3.2:3.3:4:5:6_1.2_7	LETMD1	ES	2:3.2:3.3:4:5:6	1.2	7	6.9E-15	0.139	-0.384	2.9E-04	1.4E-04	2.2E-04
LGG	LETMD1_ES_2:3.2:4:5:6_1.2_7	LETMD1	ES	2:3.2:4:5:6	1.2	7	6.1E-24	0.204	-0.342	1.2E-02	2.4E-03	9.3E-03
LGG	LETMD1_ES_2:4_1.2_5	LETMD1	ES	2:04	1.2	5	6.2E-12	0.110	-0.228	9.4E-04	1.6E-04	2.8E-03
LGG	LETMD1_ES_3.2:3.3_2_4	LETMD1	ES	3.2:3.3	2	4	1.2E-10	0.102	-0.234	1.5E-03	1.4E-03	1.6E-04
LGG	LETMD1_ES_3.2:4_2_5	LETMD1	ES	3.2:4	2	5	4.6E-25	0.212	-0.269	2.2E-02	2.8E-02	2.9E-02
LGG	LETMD1_ES_4:5:6_2_7	LETMD1	ES	4:05:06	2	7	8.6E-23	0.196	0.244	1.3E-05	1.0E-05	5.4E-05
LGG	LETMD1_ES_5:6_2_7	LETMD1	ES	5:06	2	7	1.1E-26	0.226	0.285	4.7E-05	8.3E-06	1.3E-05
LGG	LGALS8_ES_11_10.1_12	LGALS8	ES	11	10.1	12	8.6E-12	0.108	-0.488	1.8E-02	8.2E-05	1.5E-04
LGG	LGMN_ES_14_12_15	LGMN	ES	14	12	15	2.7E-15	0.144	0.379	3.1E-01	1.2E-01	9.6E-02
LGG	LMAN2L_ES_3_2_4	LMAN2L	ES	3	2	4	1.2E-11	0.107	-0.157	4.4E-03	6.5E-03	7.4E-02
LGG	LMBR1_ES_20_19_21	LMBR1	ES	20	19	21	4.7E-46	0.351	-0.209	1.2E-02	5.5E-04	6.0E-03
LGG	LMO3_ES_9.2:10:11.1:11.2_9.1_12	LMO3	ES	9.2:10:11.1:11.2	9.1	12	1.0E-43	0.375	-0.035	1.1E-08	8.3E-06	1.2E-03
LGG	LPCAT3_RI_10.2_10.1_10.3	LPCAT3	RI	10.2	10.1	10.3	7.6E-16	0.141	-0.530	7.1E-03	1.3E-03	1.7E-04
LGG	LPCAT3_RI_10.4_10.3_10.5	LPCAT3	RI	10.4	10.3	10.5	6.8E-13	0.116	-0.466	3.0E-04	2.0E-04	2.6E-04
LGG	LPHN3_ES_14_13_15	LPHN3	ES	14	13	15	1.4E-11	0.110	0.132	6.1E-04	2.5E-04	8.5E-03
LGG	LPHN3_ES_8_7_9	LPHN3	ES	8	7	9	5.4E-18	0.196	0.278	1.3E-01	3.4E-03	1.0E-02
LGG	LPPR4_ES_6_5_7	LPPR4	ES	6	5	7	3.4E-13	0.127	0.172	2.6E-03	8.4E-03	3.8E-02
LGG	LRCH3_ES_21_20_22.1	LRCH3	ES	21	20	22.1	3.6E-12	0.111	0.062	1.5E-03	4.7E-03	2.7E-02
LGG	LRP2BP_RI_2.3:2.4_2.2_2.5	LRP2BP	RI	2.3:2.4	2.2	2.5	9.1E-21	0.180	-0.400	3.6E-04	6.5E-05	1.2E-03
LGG	LRRC17_ES_4_3_5	LRRC17	ES	4	3	5	3.2E-16	0.166	0.070	2.2E-02	3.5E-03	6.9E-03
LGG	LRRC23_AD_3.2_3.1_4	LRRC23	AD	3.2	3.1	4	1.1E-44	0.343	0.391	2.6E-02	7.3E-03	3.9E-03
LGG	LRRC23_ES_5_4_6	LRRC23	ES	5	4	6	3.8E-11	0.101	0.220	5.1E-01	9.8E-01	3.6E-01
LGG	LRRC27_ES_11_10_12	LRRC27	ES	11	10	12	8.5E-17	0.148	-0.265	2.1E-03	9.0E-06	1.9E-04
LGG	LRRC27_ES_8_7_9	LRRC27	ES	8	7	9	1.3E-23	0.201	-0.210	2.4E-04	1.3E-04	1.3E-03
LGG	LRRFIP1_ES_15_14_16	LRRFIP1	ES	15	14	16	3.4E-18	0.161	-0.059	2.7E-02	6.3E-03	2.4E-01

cancer type	id	Gene Symbol	splice_type	Exon	From.Exon	To.Exon	anova.p	adj.r2	r	p.50	p.25	p.10
LGG	LRRFIP2_ES_20_19_21	LRRFIP2	ES	20	19	21	1.9E-24	0.207	0.138	1.2E-04	2.6E-07	2.3E-05
LGG	LRRFIP2_ES_23:24_22_25	LRRFIP2	ES	23:24	22	25	1.4E-12	0.113	-0.194	5.5E-01	1.4E-01	5.2E-01
LGG	LRRFIP2_ES_24_22_25	LRRFIP2	ES	24	22	25	1.2E-12	0.119	-0.161	7.0E-04	3.6E-04	5.5E-04
LGG	LRTOMT_ES_2.1:2.2:3_1_5	LRTOMT	ES	2.1:2.2:3	1	5	5.7E-21	0.181	-0.014	3.5E-06	4.7E-05	9.3E-04
LGG	LRTOMT_ES_2.2:3_1_5	LRTOMT	ES	2.2:3	1	5	1.2E-20	0.185	-0.043	4.8E-04	3.7E-05	3.5E-07
LGG	LSAMP_ES_8:9_7_10	LSAMP	ES	8:09	7	10	3.7E-12	0.110	-0.027	7.0E-01	6.6E-01	7.3E-01
LGG	LTB4R2_RI_1.2:1.3_1.1_1.4	LTB4R2	RI	1.2:1.3	1.1	1.4	1.3E-10	0.101	-0.163	6.8E-01	7.2E-01	4.9E-01
LGG	LTBP4_ES_25_24_28	LTBP4	ES	25	24	28	7.1E-13	0.127	-0.147	8.2E-01	8.0E-01	5.5E-01
LGG	LTBP4_ES_26:27_25_28	LTBP4	ES	26:27:00	25	28	1.6E-42	0.351	0.380	8.0E-02	1.9E-02	1.0E-01
LGG	LUC7L_ES_1.3_1.1_2.2	LUC7L	ES	1.3	1.1	2.2	9.6E-12	0.107	0.208	3.9E-01	1.5E-01	2.2E-01
LGG	LUC7L_ES_4_3_5	LUC7L	ES	4	3	5	5.1E-28	0.233	0.455	5.2E-01	3.7E-01	8.3E-03
LGG	LYPLAL1_AA_3.1_2_3.2	LYPLAL1	AA	3.1	2	3.2	2.8E-18	0.160	0.279	4.3E-02	4.4E-01	1.9E-01
LGG	LYRM1_ES_5_3_8.1	LYRM1	ES	5	3	8.1	1.5E-15	0.142	0.093	1.1E-03	6.3E-06	2.5E-04
LGG	LYRM1_ES_6:7_5_8.1	LYRM1	ES	6:07	5	8.1	8.2E-11	0.126	-0.059	3.1E-01	1.2E-01	1.7E-02
LGG	MACF1_ES_69_68_70	MACF1	ES	69	68	70	6.8E-25	0.214	0.338	2.7E-03	3.9E-04	4.0E-03
LGG	MAD2L2_ES_2:3_1_4	MAD2L2	ES	2:03	1	4	1.1E-39	0.312	-0.264	3.6E-03	3.5E-04	1.8E-04
LGG	MADD_AD_25.2_25.1_26	MADD	AD	25.2	25.1	26	8.1E-25	0.211	-0.545	2.6E-04	4.6E-05	6.5E-05
LGG	MADD_AD_25.2:25.3_25.1_26	MADD	AD	25.2:25.3	25.1	26	3.5E-30	0.250	-0.574	1.0E-04	7.1E-07	1.4E-04
LGG	MADD_ES_28_27_29	MADD	ES	28	27	29	2.9E-20	0.176	0.775	8.3E-04	6.0E-03	1.8E-02
LGG	MAGED2_RI_14.2_14.1_14.3	MAGED2	RI	14.2	14.1	14.3	9.8E-20	0.172	0.193	1.4E-01	1.7E-01	4.0E-02
LGG	MAGED4B_AA_2.1:2.2_1_2.3	MAGED4B	AA	2.1:2.2	1	2.3	3.4E-95	0.587	0.109	7.3E-08	6.0E-10	2.1E-08
LGG	MAGI1_ES_15_14_16	MAGI1	ES	15	14	16	8.9E-13	0.120	0.261	1.4E-02	1.8E-02	7.8E-02
LGG	MAGI1_ES_24_23.1_25	MAGI1	ES	24	23.1	25	7.0E-18	0.161	0.153	2.6E-06	3.8E-05	3.2E-04
LGG	MAGI2_ES_24_23_25	MAGI2	ES	24	23	25	3.6E-13	0.121	0.015	1.2E-04	5.9E-07	3.9E-05
LGG	MAGI2_ES_5_4_6	MAGI2	ES	5	4	6	2.6E-13	0.120	-0.416	5.2E-01	3.2E-01	9.2E-01
LGG	MAGI3_ES_22.1_21_23	MAGI3	ES	22.1	21	23	3.5E-22	0.206	0.147	7.6E-04	5.2E-03	1.6E-04
LGG	MAN2C1_ES_8_6_9	MAN2C1	ES	8	6	9	2.4E-12	0.124	0.232	4.2E-02	8.3E-03	1.1E-01
LGG	MAP2_ES_13_12_14	MAP2	ES	13	12	14	2.4E-21	0.185	-0.486	3.6E-04	7.6E-05	1.6E-02
LGG	MAP2_ES_16_15_17	MAP2	ES	16	15	17	2.0E-64	0.452	-0.636	2.5E-09	1.3E-10	1.3E-04
LGG	MAP2_ES_9.1:9.2:10:11_8_12	MAP2	ES	9.1:9.2:10:11	8	12	1.9E-13	0.121	0.507	2.4E-04	1.5E-03	5.2E-03
LGG	MAP3K12_RI_3.3_3.2_3.4	MAP3K12	RI	3.3	3.2	3.4	7.1E-14	0.126	-0.124	2.9E-04	1.1E-04	1.4E-02
LGG	MAP3K4_ES_18_17_19	MAP3K4	ES	18	17	19	7.0E-13	0.116	0.045	6.0E-03	1.8E-03	1.5E-03
LGG	MAP3K7_ES_11_10_12	MAP3K7	ES	11	10	12	1.6E-26	0.224	0.006	1.6E-01	8.9E-02	4.3E-02
LGG	MAP4_AA_24.1_23_24.2	MAP4	AA	24.1	23	24.2	1.2E-85	0.548	-0.085	3.7E-07	1.5E-09	3.4E-07
LGG	MAP4_ES_19:20_18_21	MAP4	ES	19:20	18	21	9.5E-66	0.458	-0.021	7.4E-08	3.3E-11	2.5E-09
LGG	MAP4_ES_23:24.1_22_24.2	MAP4	ES	23:24.1	22	24.2	4.2E-60	0.429	0.240	8.0E-06	6.6E-09	1.1E-06
LGG	MAP4K3_ES_16_15_18	MAP4K3	ES	16	15	18	4.9E-13	0.121	-0.048	8.4E-01	8.8E-01	6.2E-01
LGG	MAP4K4_ES_17_16.2_18	MAP4K4	ES	17	16.2	18	1.2E-11	0.108	-0.001	6.3E-02	4.3E-06	1.5E-03
LGG	MAP4K4_ES_17:18_16.2_19	MAP4K4	ES	17:18	16.2	19	2.5E-12	0.111	0.257	9.7E-03	1.8E-04	1.0E-01
LGG	MAP7D1_RI_7.2_7.1_7.3	MAP7D1	RI	7.2	7.1	7.3	3.2E-11	0.102	0.110	1.5E-01	2.2E-02	4.9E-01

cancer type	id	Gene Symbol	splice_type	Exon	From.Exon	To.Exon	anova.p	adj.r2	r	p.50	p.25	p.10
LGG	MAPK8_ME_7|8_6_11.1	MAPK8	ME	7|8	6	11.1	1.0E-27	0.233	0.491	1.7E-05	1.1E-07	9.4E-06
LGG	MAPT_ES_13_12_14	MAPT	ES	13	12	14	2.8E-21	0.184	-0.306	1.9E-04	4.0E-04	6.8E-06
LGG	MAPT_ES_9_8_10	MAPT	ES	9	8	10	2.8E-37	0.297	-0.567	1.6E-07	3.9E-07	9.1E-04
LGG	MARCH7_ES_2:3_1_4	MARCH7	ES	2:03	1	4	1.1E-11	0.130	-0.042	1.9E-03	4.7E-03	1.0E-05
LGG	MARCH8_ES_2_1_4	MARCH8	ES	2	1	4	5.6E-15	0.136	-0.466	2.0E-04	9.3E-06	3.4E-04
LGG	MARK1_ES_17_16_18	MARK1	ES	17	16	18	2.3E-11	0.103	-0.097	6.1E-01	6.7E-01	9.9E-01
LGG	MARK2_ES_16.1:16.2_15.2_17	MARK2	ES	16.1:16.2	15.2	17	5.4E-15	0.134	0.050	6.4E-04	1.1E-03	1.7E-03
LGG	MARK2_ES_18:19_17_20	MARK2	ES	18:19	17	20	6.2E-14	0.132	-0.083	9.4E-02	1.8E-01	1.1E-01
LGG	MARK2_ES_19_17_20	MARK2	ES	19	17	20	3.3E-15	0.136	-0.143	2.3E-01	4.8E-02	5.8E-02
LGG	MARK3_ES_17_16_19	MARK3	ES	17	16	19	8.1E-22	0.188	0.058	5.3E-01	1.7E-02	3.7E-03
LGG	MARK3_ES_18_16_19	MARK3	ES	18	16	19	3.6E-11	0.101	-0.094	6.1E-03	7.7E-05	1.3E-05
LGG	MARK4_ES_16_15_17	MARK4	ES	16	15	17	5.5E-15	0.134	0.199	3.2E-04	1.7E-05	1.0E-03
LGG	MBD1_ES_10_9_11	MBD1	ES	10	9	11	1.0E-17	0.156	0.220	2.3E-04	1.9E-05	1.4E-05
LGG	MBNL1_ES_8_7_9	MBNL1	ES	8	7	9	2.1E-15	0.142	-0.433	3.1E-01	1.6E-01	3.0E-01
LGG	MBP_ES_5.3:5.4:6_5.2_8.1	MBP	ES	5.3:5.4:6	5.2	8.1	4.6E-21	0.195	-0.237	4.0E-07	3.1E-08	8.9E-04
LGG	MBP_ES_5.3:5.4:6:8.1:8.2_5.2_8.3	MBP	ES	5.3:5.4:6:8.1:8.2	5.2	8.3	2.8E-21	0.190	-0.258	2.7E-04	1.1E-08	1.6E-03
LGG	MCFD2_ES_6_3_7	MCFD2	ES	6	3	7	7.5E-12	0.109	0.145	2.9E-03	5.6E-04	3.1E-04
LGG	MDK_RI_2.3_2.2_2.4	MDK	RI	2.3	2.2	2.4	2.7E-15	0.161	-0.071	1.1E-02	1.4E-02	2.2E-01
LGG	MDM1_RI_3.3_3.2_3.4	MDM1	RI	3.3	3.2	3.4	3.7E-22	0.190	-0.134	1.5E-04	9.0E-06	5.4E-04
LGG	MDM4_ES_8_5_9	MDM4	ES	8	5	9	2.3E-10	0.113	-0.116	6.8E-03	2.4E-03	1.9E-04
LGG	MEAF6_ES_6_5_7	MEAF6	ES	6	5	7	3.2E-21	0.185	-0.105	2.3E-04	2.3E-06	4.5E-04
LGG	MEAF6_ES_6:7:8.1:9.1_5_9.2	MEAF6	ES	6:7:8.1:9.1	5	9.2	1.9E-30	0.256	-0.109	4.4E-05	5.9E-04	5.5E-03
LGG	MEAF6_ES_6:8.1:9.1_5_9.2	MEAF6	ES	6:8.1:9.1	5	9.2	5.8E-31	0.254	-0.006	1.4E-02	6.3E-04	5.4E-04
LGG	MEAF6_ES_7_5_8.1	MEAF6	ES	7	5	8.1	1.6E-41	0.323	0.268	1.1E-03	1.3E-03	6.4E-04
LGG	MED15_ES_12_11_13	MED15	ES	12	11	13	3.8E-21	0.183	-0.209	5.0E-03	1.4E-05	7.4E-04
LGG	MED22_RI_5.2_5.1_5.3	MED22	RI	5.2	5.1	5.3	3.6E-23	0.198	0.146	4.9E-04	5.8E-07	4.6E-07
LGG	MED23_ES_12_10.1_13.2	MED23	ES	12	10.1	13.2	9.0E-16	0.166	0.152	4.9E-03	1.2E-04	3.3E-03
LGG	MED31_ES_4_3_5	MED31	ES	4	3	5	4.9E-15	0.134	0.112	2.9E-01	7.2E-02	2.1E-03
LGG	MEF2B_AA_14.2:14.3_13_14.4	MEF2B	AA	14.2:14.3	13	14.4	6.3E-16	0.157	0.111	1.1E-01	7.5E-01	6.5E-01
LGG	MEF2B_ES_14.2_13_14.4	MEF2B	ES	14.2	13	14.4	1.6E-19	0.181	-0.028	8.4E-01	6.2E-01	6.4E-01
LGG	MEF2B_ES_2:3_1_4	MEF2B	ES	2:03	1	4	7.0E-32	0.269	-0.251	2.4E-02	3.2E-02	2.0E-01
LGG	MEGF8_ES_30_29_31	MEGF8	ES	30	29	31	1.9E-33	0.276	-0.607	2.8E-03	2.6E-02	4.1E-01
LGG	MEIS1_AD_12.2:12.3_12.1_12.5	MEIS1	AD	12.2:12.3	12.1	12.5	6.8E-12	0.139	-0.289	9.5E-06	1.8E-04	2.0E-05
LGG	MEIS1_RI_12.2:12.3:12.4_12.1_12.5	MEIS1	RI	12.2:12.3:12.4	12.1	12.5	4.9E-12	0.132	-0.361	2.6E-03	3.1E-05	1.3E-05
LGG	MEIS3_AA_7.1_6_7.2	MEIS3	AA	7.1	6	7.2	1.0E-33	0.273	0.710	1.2E-01	1.0E-02	8.4E-03
LGG	MELK_ES_8_7_9	MELK	ES	8	7	9	4.8E-17	0.186	-0.662	3.4E-06	1.1E-04	1.1E-04
LGG	MFF_ES_10_7_11	MFF	ES	10	7	11	5.7E-14	0.125	0.200	6.5E-03	1.9E-03	2.6E-06
LGG	MFF_ES_3:4:5_1_6	MFF	ES	3:04:05	1	6	9.8E-13	0.115	0.064	9.3E-02	2.6E-02	2.5E-02
LGG	MFF_ES_8:9_7_10	MFF	ES	8:09	7	10	2.0E-28	0.236	-0.178	6.1E-01	2.8E-01	6.2E-01
LGG	MFF_ES_8:9:10_7_11	MFF	ES	8:09:10	7	11	1.8E-39	0.310	-0.050	5.3E-02	2.1E-02	1.7E-01

cancer type	id	Gene Symbol	splice_type	Exon	From.Exon	To.Exon	anova.p	adj.r2	r	p.50	p.25	p.10
LGG	MFF_ES_9:10_7_11	MFF	ES	9:10	7	11	6.2E-46	0.350	0.088	6.3E-02	9.2E-03	1.8E-02
LGG	MFSD12_ES_14_12_15	MFSD12	ES	14	12	15	1.3E-19	0.171	-0.413	4.1E-03	3.6E-04	1.2E-02
LGG	MGRN1_ES_17.1_16_17.3	MGRN1	ES	17.1	16	17.3	3.4E-16	0.144	0.182	3.9E-01	1.5E-01	2.1E-01
LGG	MIB2_ES_10.2_9_11	MIB2	ES	10.2	9	11	4.6E-27	0.231	-0.256	5.8E-02	9.7E-03	1.2E-02
LGG	MICAL3_ES_29_28_30	MICAL3	ES	29	28	30	7.2E-15	0.139	0.231	2.9E-02	1.3E-02	2.6E-02
LGG	MIER3_ES_6_5_7	MIER3	ES	6	5	7	1.3E-17	0.192	-0.026	1.7E-02	6.2E-02	4.8E-02
LGG	MINK1_ES_19_18_20	MINK1	ES	19	18	20	5.4E-23	0.196	-0.370	5.1E-05	4.5E-05	2.4E-04
LGG	MKNK1_ES_11_10_12	MKNK1	ES	11	10	12	1.1E-11	0.106	-0.459	5.5E-01	3.8E-02	9.7E-02
LGG	MKNK1_ES_16_15.2_17	MKNK1	ES	16	15.2	17	2.7E-12	0.111	0.230	7.2E-01	4.9E-01	1.5E-01
LGG	MKNK2_AA_14.1_13.1_14.2	MKNK2	AA	14.1	13.1	14.2	1.2E-11	0.105	-0.067	3.2E-01	8.0E-02	3.4E-02
LGG	MLIP_ES_15_13_16	MLIP	ES	15	13	16	1.2E-09	0.104	-0.108	1.1E-01	6.5E-02	1.5E-01
LGG	MLIP_ES_6_5_7	MLIP	ES	6	5	7	3.0E-11	0.118	-0.255	2.8E-01	1.0E-01	3.5E-03
LGG	MLIP_ES_6:7_5_8	MLIP	ES	6:07	5	8	9.0E-10	0.108	-0.266	4.9E-01	4.4E-02	3.6E-05
LGG	MLLT1_ES_5_4_6	MLLT1	ES	5	4	6	7.2E-31	0.255	-0.006	3.9E-07	6.3E-09	2.0E-06
LGG	MLST8_ES_9:10.1_8.2_11.1	MLST8	ES	09:10.1	8.2	11.1	4.7E-29	0.245	0.228	1.3E-03	6.2E-03	2.6E-01
LGG	MMAB_ES_5_4_6	MMAB	ES	5	4	6	8.4E-20	0.172	-0.618	1.7E-01	9.2E-01	4.8E-01
LGG	MMP23B_RI_3.4_3.3_3.5	MMP23B	RI	3.4	3.3	3.5	4.8E-12	0.122	-0.097	2.0E-01	2.0E-02	4.3E-02
LGG	MOCS1_ES_11.1_10_12.1	MOCS1	ES	11.1	10	12.1	2.7E-09	0.109	0.128	5.8E-02	3.3E-03	5.0E-02
LGG	MOK_ES_5:6.1_2_7	MOK	ES	05:06.1	2	7	2.9E-12	0.141	0.136	9.3E-02	1.1E-03	6.1E-02
LGG	MPP5_AA_3.1_2_3.2	MPP5	AA	3.1	2	3.2	1.5E-13	0.125	0.073	1.4E-02	5.2E-04	4.3E-03
LGG	MPPE1_ES_12_11_13	MPPE1	ES	12	11	13	3.1E-44	0.340	0.103	6.0E-04	2.6E-07	8.8E-07
LGG	MPRIP_ES_24_23_25	MPRIP	ES	24	23	25	4.3E-35	0.282	-0.064	1.6E-01	9.6E-04	2.3E-05
LGG	MPV17_ES_6.3_3.2_7	MPV17	ES	6.3	3.2	7	4.8E-17	0.157	0.207	7.7E-01	3.3E-01	5.4E-01
LGG	MPZL1_ES_5_4_6	MPZL1	ES	5	4	6	8.7E-54	0.395	0.043	1.4E-07	1.9E-07	2.8E-04
LGG	MR1_ES_4_3.2_5	MR1	ES	4	3.2	5	4.3E-14	0.126	0.092	2.4E-04	5.0E-06	2.3E-04
LGG	MRO_ES_8_7_9	MRO	ES	8	7	9	7.1E-23	0.196	-0.203	3.4E-08	1.8E-07	1.2E-05
LGG	MRPL55_ES_1.2:2.2_1.1_2.9	MRPL55	ES	1.2:2.2	1.1	2.9	1.2E-16	0.147	0.079	4.3E-01	6.6E-01	3.6E-01
LGG	MRPL55_ES_1.2:2.2:2.5:2.6_1.1_2.9	MRPL55	ES	1.2:2.2:2.5:2.6	1.1	2.9	2.6E-14	0.128	0.068	6.4E-01	6.7E-01	9.0E-01
LGG	MRPL55_ES_1.2:2.2:2.8_1.1_2.9	MRPL55	ES	1.2:2.2:2.8	1.1	2.9	3.1E-11	0.102	0.024	1.9E-01	6.8E-01	3.8E-01
LGG	MRPL55_ES_2.2_1.1_2.9	MRPL55	ES	2.2	1.1	2.9	1.9E-19	0.169	0.116	4.7E-01	4.3E-01	4.8E-02
LGG	MRPL55_ES_2.2_1.2_2.9	MRPL55	ES	2.2	1.2	2.9	1.8E-19	0.170	0.156	7.3E-02	4.3E-01	1.0E-01
LGG	MRPL55_ES_2.2:2.3:2.4:2.5:2.6_1.1_2.9	MRPL55	ES	2.2:2.3:2.4:2.5:2.[illegible]	1.1	2.9	8.9E-13	0.115	-0.077	8.9E-01	1.1E-01	5.2E-02
LGG	MRPL55_ES_2.2:2.3:2.4:2.5:2.6_1.2_2.9	MRPL55	ES	2.2:2.3:2.4:2.5:2.[illegible]	1.2	2.9	5.7E-12	0.108	-0.044	4.3E-01	2.7E-01	2.3E-02
LGG	MRPL55_ES_2.2:2.8_1.1_2.9	MRPL55	ES	2.2:2.8	1.1	2.9	1.6E-12	0.115	0.072	3.0E-01	1.8E-01	5.6E-03
LGG	MRPS28_ES_3_2_4.1	MRPS28	ES	3	2	4.1	5.0E-24	0.229	-0.316	8.1E-04	6.0E-07	6.4E-08
LGG	MRPS28_ES_4.2_1_5	MRPS28	ES	4.2	1	5	5.6E-18	0.161	0.042	2.2E-02	3.7E-01	2.1E-01
LGG	MSI1_ES_11_10_12	MSI1	ES	11	10	12	1.1E-21	0.191	-0.221	3.4E-01	6.0E-01	1.4E-01
LGG	MSI2_AA_18.1_17_18.2	MSI2	AA	18.1	17	18.2	2.2E-32	0.264	0.221	6.5E-02	5.6E-01	7.2E-01
LGG	MTA1_ES_18_17_20	MTA1	ES	18	17	20	1.2E-37	0.299	-0.512	1.1E-02	3.5E-02	4.4E-02
LGG	MTERFD2_ES_2_1_3.1	MTERFD2	ES	2	1	3.1	4.5E-15	0.134	0.271	4.7E-02	1.3E-02	1.4E-01

cancer type	id	Gene Symbol	splice_type	Exon	From.Exon	To.Exon	anova.p	adj.r2	r	p.50	p.25	p.10
LGG	MTHFD2L_ES_7_6_8	MTHFD2L	ES	7	6	8	1.3E-11	0.106	0.295	1.8E-01	4.6E-01	4.2E-02
LGG	MTIF3_ES_5_4.2_6	MTIF3	ES	5	4.2	6	3.9E-16	0.143	0.072	1.1E-03	4.2E-04	2.4E-03
LGG	MTMR1_ES_4_3_5	MTMR1	ES	4	3	5	1.0E-12	0.132	-0.432	7.6E-01	1.9E-01	4.4E-02
LGG	MTMR14_ES_19:20_18_21	MTMR14	ES	19:20	18	21	6.7E-14	0.125	-0.218	4.7E-02	3.3E-02	9.3E-02
LGG	MTMR3_ES_20_19_21	MTMR3	ES	20	19	21	8.0E-17	0.149	0.166	7.7E-03	3.6E-04	1.2E-02
LGG	MTSS1_ES_12_11_13	MTSS1	ES	12	11	13	3.1E-40	0.329	0.396	6.5E-07	9.3E-11	3.9E-06
LGG	MTSS1_ES_12_11_14.1	MTSS1	ES	12	11	14.1	6.1E-21	0.195	0.005	8.2E-03	2.9E-06	1.0E-04
LGG	MTSS1_ES_12:14.1_11_14.2	MTSS1	ES	12:14.1	11	14.2	1.6E-10	0.103	-0.148	1.7E-01	2.7E-02	1.5E-04
LGG	MTSS1_ES_13_11_14.1	MTSS1	ES	13	11	14.1	1.1E-37	0.311	-0.439	1.2E-05	2.5E-07	9.1E-07
LGG	MTSS1L_ES_7_6_8	MTSS1L	ES	7	6	8	1.2E-30	0.252	0.306	1.3E-03	8.7E-06	1.7E-05
LGG	MTX3_ES_8_7_9	MTX3	ES	8	7	9	1.2E-44	0.385	-0.011	6.3E-06	8.1E-07	1.7E-04
LGG	MUTYH_ES_6.2:6.3:6.4:6.5:7:8:9_5_10	MUTYH	ES	.2:6.3:6.4:6.5:7:8	5	10	1.3E-12	0.148	-0.176	9.9E-01	7.7E-01	7.9E-01
LGG	MUTYH_ES_6.3:6.4:6.5:7:8:9_5_10	MUTYH	ES	6.3:6.4:6.5:7:8:9	5	10	6.1E-13	0.139	-0.138	3.5E-01	5.8E-01	1.2E-01
LGG	MUTYH_ES_6.5:7:8:9_5_10	MUTYH	ES	6.5:7:8:9	5	10	1.6E-13	0.130	-0.081	7.3E-01	7.7E-01	4.4E-01
LGG	MYCBPAP_ES_20:21_19_22	MYCBPAP	ES	20:21	19	22	2.3E-15	0.137	-0.529	2.9E-02	3.2E-02	2.8E-03
LGG	MYEF2_ES_12_11.1_13	MYEF2	ES	12	11.1	13	1.8E-17	0.157	0.154	7.8E-03	1.2E-03	2.0E-03
LGG	MYH14_ES_7_6.1_9	MYH14	ES	7	6.1	9	3.8E-23	0.207	0.075	3.2E-05	2.4E-07	6.2E-07
LGG	MYL6_ES_4.3_4.1_5	MYL6	ES	4.3	4.1	5	3.9E-12	0.110	0.230	3.2E-02	8.0E-03	2.6E-03
LGG	MYL6_RI_1.2_1.1_1.3	MYL6	RI	1.2	1.1	1.3	3.8E-17	0.151	-0.336	1.8E-03	1.2E-03	3.5E-03
LGG	MYO18A_ES_41_40_42	MYO18A	ES	41	40	42	8.5E-22	0.187	0.020	5.2E-01	9.3E-01	3.0E-03
LGG	MYO19_ES_23_22_24	MYO19	ES	23	22	24	4.9E-37	0.299	-0.010	2.1E-07	1.1E-09	6.4E-06
LGG	MYO5A_ES_30_29_32	MYO5A	ES	30	29	32	2.4E-19	0.185	0.357	7.4E-05	6.8E-06	2.2E-04
LGG	MYO5A_ES_33_32_34	MYO5A	ES	33	32	34	3.3E-18	0.168	-0.650	5.4E-04	4.5E-04	3.8E-03
LGG	MYO9A_ES_27_26_28	MYO9A	ES	27	26	28	7.2E-19	0.176	-0.291	1.5E-04	9.8E-06	6.1E-03
LGG	MYO9B_ES_37_36_38.1	MYO9B	ES	37	36	38.1	3.2E-17	0.158	-0.078	4.0E-01	8.4E-01	8.1E-01
LGG	MYT1_AA_13.1_12_13.2	MYT1	AA	13.1	12	13.2	6.7E-34	0.300	-0.243	6.2E-01	9.8E-02	4.6E-01
LGG	MZT2B_ES_2:3_1_4	MZT2B	ES	2:03	1	4	1.2E-24	0.209	-0.134	5.0E-06	3.1E-07	3.6E-04
LGG	NABP2_AD_1.2_1.1_1.4	NABP2	AD	1.2	1.1	1.4	1.8E-20	0.178	0.101	7.5E-04	2.3E-05	4.4E-04
LGG	NABP2_RI_1.2:1.3_1.1_1.4	NABP2	RI	1.2:1.3	1.1	1.4	6.5E-24	0.203	0.143	2.2E-01	2.6E-01	1.0E-01
LGG	NAGPA_ES_6_5_7	NAGPA	ES	6	5	7	2.7E-17	0.154	0.025	2.7E-04	1.2E-03	2.0E-03
LGG	NAP1L1_RI_14.2_14.1_14.3	NAP1L1	RI	14.2	14.1	14.3	6.1E-19	0.165	-0.248	8.8E-02	6.7E-02	2.3E-02
LGG	NAP1L4_ES_16_15_17	NAP1L4	ES	16	15	17	9.3E-56	0.406	-0.175	7.4E-04	1.8E-05	5.3E-05
LGG	NARG2_AA_4.1_3_4.2	NARG2	AA	4.1	3	4.2	2.2E-18	0.162	-0.151	2.1E-01	4.1E-04	4.7E-02
LGG	NASP_ES_9_8_10	NASP	ES	9	8	10	8.7E-15	0.132	0.085	4.2E-01	4.3E-01	5.5E-01
LGG	NAV1_ES_15_14_16	NAV1	ES	15	14	16	1.3E-50	0.393	0.353	6.9E-07	1.1E-08	8.0E-05
LGG	NAV2_ES_34_33_35	NAV2	ES	34	33	35	1.2E-19	0.180	-0.074	1.4E-01	1.3E-02	1.7E-02
LGG	NBEA_ES_3:4:5:6_2_7	NBEA	ES	3:4:5:6	2	7	2.7E-11	0.109	0.509	4.5E-03	2.1E-03	5.7E-04
LGG	NBN_ES_6_5_7	NBN	ES	6	5	7	1.9E-19	0.180	0.009	1.8E-02	1.6E-03	9.3E-02
LGG	NCAM1_ES_9_8_10	NCAM1	ES	9	8	10	1.4E-33	0.274	-0.336	5.5E-03	2.8E-02	1.1E-01
LGG	NCKAP1_ES_2_1_3	NCKAP1	ES	2	1	3	9.0E-15	0.136	0.360	3.9E-05	1.7E-03	1.8E-01

cancer type	id	Gene Symbol	splice_type	Exon	From.Exon	To.Exon	anova.p	adj.r2	r	p.50	p.25	p.10
LGG	NCKAP5_ES_19_18_20	NCKAP5	ES	19	18	20	6.2E-13	0.142	0.310	2.7E-01	3.5E-01	8.4E-01
LGG	NCOA2_ES_14_13_15.1	NCOA2	ES	14	13	15.1	1.3E-19	0.180	-0.125	8.1E-03	1.6E-02	5.4E-03
LGG	NCOA7_AA_11.1_10_11.2	NCOA7	AA	11.1	10	11.2	1.7E-09	0.104	-0.477	1.2E-01	9.0E-03	1.3E-02
LGG	NCOR2_AD_46.2_46.1_47	NCOR2	AD	46.2	46.1	47	5.6E-17	0.150	0.399	5.1E-07	2.2E-06	5.1E-03
LGG	NCOR2_ES_20_19_21	NCOR2	ES	20	19	21	1.7E-11	0.106	-0.308	3.4E-02	9.8E-02	8.2E-01
LGG	NDRG2_ES_4.5_2_5.2	NDRG2	ES	4.5	2	5.2	6.8E-51	0.380	-0.551	5.2E-09	5.4E-13	1.9E-06
LGG	NDRG2_ES_4.5_4.1_5.2	NDRG2	ES	4.5	4.1	5.2	5.1E-35	0.281	-0.373	1.7E-06	4.2E-08	1.3E-07
LGG	NDRG2_ES_6_5.2_7.2	NDRG2	ES	6	5.2	7.2	2.9E-13	0.119	-0.219	1.7E-02	8.2E-05	1.4E-02
LGG	NDRG2_RI_4.3:4.4_4.2_4.5	NDRG2	RI	4.3:4.4	4.2	4.5	2.0E-11	0.104	-0.226	9.6E-01	8.3E-01	3.6E-01
LGG	NDUFA5_ES_3:4_2_5	NDUFA5	ES	3:04	2	5	6.8E-13	0.138	-0.142	8.3E-01	7.3E-01	7.4E-01
LGG	NDUFAF6_ES_9.2:10_9.1_11	NDUFAF6	ES	9.2:10	9.1	11	6.6E-18	0.164	-0.093	1.1E-04	2.6E-05	9.5E-07
LGG	NDUFS8_ES_3:4.1:4.2:5:6_1_7	NDUFS8	ES	3:4.1:4.2:5:6	1	7	1.6E-31	0.258	-0.157	1.9E-01	4.5E-01	3.4E-01
LGG	NDUFV3_ES_2:3_1_4	NDUFV3	ES	2:03	1	4	6.2E-15	0.139	-0.330	1.1E-03	1.3E-03	5.9E-03
LGG	NDUFV3_ES_3_2_4	NDUFV3	ES	3	2	4	8.6E-27	0.224	-0.368	1.1E-04	1.9E-05	3.0E-03
LGG	NECAB3_RI_7.2_7.1_7.3	NECAB3	RI	7.2	7.1	7.3	8.7E-15	0.132	-0.215	3.7E-02	1.4E-03	2.8E-02
LGG	NEIL2_ES_1.3_1.1_2.1	NEIL2	ES	1.3	1.1	2.1	3.9E-15	0.136	0.140	9.7E-08	4.3E-05	3.7E-02
LGG	NEIL2_ES_1.3:2.1_1.1_3	NEIL2	ES	1.3:2.1	1.1	3	8.9E-11	0.101	0.145	2.9E-02	6.2E-02	2.2E-02
LGG	NEK1_ES_17_16_18	NEK1	ES	17	16	18	3.0E-11	0.103	0.095	6.6E-02	1.3E-03	4.0E-05
LGG	NEK1_ES_19_18_20	NEK1	ES	19	18	20	7.7E-39	0.315	-0.304	5.6E-01	2.5E-01	3.8E-01
LGG	NEURL4_ES_3_2_4	NEURL4	ES	3	2	4	4.0E-27	0.231	-0.170	8.3E-02	7.3E-02	2.6E-02
LGG	NFIB_ES_12.1:12.2:13_11_14	NFIB	ES	12.1:12.2:13	11	14	2.3E-20	0.211	-0.232	6.2E-05	7.1E-05	2.4E-03
LGG	NFIB_ES_12.1:12.2:13_11_15	NFIB	ES	12.1:12.2:13	11	15	1.2E-14	0.134	-0.086	2.0E-01	4.1E-03	1.4E-02
LGG	NFIB_ES_12.1:13_11_14	NFIB	ES	12.1:13	11	14	2.6E-17	0.191	-0.264	2.8E-05	5.2E-03	2.7E-03
LGG	NFYA_ES_3_2_4	NFYA	ES	3	2	4	7.3E-14	0.127	0.226	3.9E-04	1.6E-03	2.0E-04
LGG	NKAIN4_ES_8_7_9	NKAIN4	ES	8	7	9	2.8E-33	0.270	-0.147	1.2E-02	3.6E-04	2.7E-03
LGG	NKIRAS2_ES_5.5:5.6_5.3_7	NKIRAS2	ES	5.5:5.6	5.3	7	1.2E-08	0.102	-0.145	2.8E-01	1.6E-01	9.9E-01
LGG	NLGN3_ES_3:4_2_5	NLGN3	ES	3:04	2	5	9.2E-16	0.143	0.267	8.0E-02	4.2E-02	1.7E-01
LGG	NLGN3_ES_4_2_5	NLGN3	ES	4	2	5	5.0E-17	0.152	0.346	7.7E-02	1.9E-01	3.9E-01
LGG	NME4_ES_4_2_5	NME4	ES	4	2	5	1.6E-22	0.193	-0.132	7.8E-06	6.9E-09	2.4E-05
LGG	NME6_ES_5.1:5.2_4_6	NME6	ES	5.1:5.2	4	6	1.1E-10	0.100	-0.232	3.0E-03	5.2E-02	2.5E-02
LGG	NOL8_AD_1.5_1.4_2.2	NOL8	AD	1.5	1.4	2.2	6.0E-13	0.125	-0.056	2.0E-01	2.3E-02	1.3E-02
LGG	NPEPL1_AD_2.2_2.1_4	NPEPL1	AD	2.2	2.1	4	7.0E-10	0.102	0.065	2.6E-03	8.5E-03	2.1E-02
LGG	NPHP1_ES_20_19_21	NPHP1	ES	20	19	21	1.1E-16	0.186	0.233	5.3E-05	2.6E-04	1.6E-02
LGG	NPL_AA_14.1_13_14.2	NPL	AA	14.1	13	14.2	6.6E-38	0.300	0.061	4.3E-03	1.4E-03	3.4E-02
LGG	NR1H3_RI_8.2_8.1_8.3	NR1H3	RI	8.2	8.1	8.3	4.7E-15	0.134	-0.147	7.3E-04	4.1E-05	1.3E-04
LGG	NRCAM_ES_21_20_22	NRCAM	ES	21	20	22	9.4E-15	0.134	0.209	1.8E-06	2.1E-07	2.9E-05
LGG	NRCAM_ES_5_4_8	NRCAM	ES	5	4	8	3.2E-11	0.107	0.218	2.2E-03	2.2E-03	3.8E-04
LGG	NRG2_ES_7_6_10	NRG2	ES	7	6	10	1.2E-25	0.242	0.165	5.4E-04	1.0E-03	5.7E-04
LGG	NRG3_ES_16_15_17	NRG3	ES	16	15	17	8.1E-25	0.230	-0.054	2.1E-01	8.0E-02	1.6E-01
LGG	NRXN1_ES_18_17_19	NRXN1	ES	18	17	19	1.5E-10	0.105	0.225	1.3E-01	3.2E-01	6.0E-02

cancer type	id	Gene Symbol	splice_type	Exon	From.Exon	To.Exon	anova.p	adj.r2	r	p.50	p.25	p.10
LGG	NSG1_AD_5.2:5.3_5.1_6	NSG1	AD	5.2:5.3	5.1	6	3.4E-17	0.167	-0.281	1.6E-03	3.9E-03	2.2E-03
LGG	NSG1_RI_5.2_5.1_5.3	NSG1	RI	5.2	5.1	5.3	3.3E-17	0.191	-0.325	2.2E-04	4.8E-03	4.3E-02
LGG	NSMAF_ES_5_3_6	NSMAF	ES	5	3	6	8.9E-13	0.119	-0.169	2.2E-01	1.4E-01	8.0E-01
LGG	NT5C2_ES_4:5_2_6	NT5C2	ES	4:05	2	6	1.1E-08	0.101	0.372	7.2E-02	4.7E-01	5.7E-01
LGG	NT5C3A_ES_3_1_5	NT5C3A	ES	3	1	5	7.9E-13	0.117	0.168	9.5E-02	3.4E-01	3.2E-01
LGG	NT5M_ES_2_1.1_3	NT5M	ES	2	1.1	3	4.5E-12	0.112	-0.013	8.5E-02	2.3E-02	4.8E-02
LGG	NTAN1_ES_2:3_1_4	NTAN1	ES	2:03	1	4	3.6E-18	0.185	0.273	4.4E-04	3.8E-04	6.0E-05
LGG	NTM_ES_9_8.1_10	NTM	ES	9	8.1	10	3.8E-17	0.153	-0.281	2.5E-04	7.1E-06	1.6E-04
LGG	NTRK2_ES_17_15_18	NTRK2	ES	17	15	18	3.5E-19	0.180	0.203	3.7E-01	5.9E-02	1.7E-01
LGG	NUDT22_RI_1.3_1.2_1.4	NUDT22	RI	1.3	1.2	1.4	3.5E-11	0.101	-0.263	2.3E-03	6.5E-02	2.0E-01
LGG	NUMA1_ES_18_17_19	NUMA1	ES	18	17	19	1.8E-14	0.129	-0.535	2.9E-02	5.4E-03	1.8E-01
LGG	OARD1_ES_5_4.1_7	OARD1	ES	5	4.1	7	2.4E-11	0.116	-0.039	3.2E-01	1.3E-01	1.0E-01
LGG	OAZ1_ES_3.2:3.3:3.4:3.5_1_4	OAZ1	ES	3.2:3.3:3.4:3.5	1	4	9.4E-13	0.115	0.229	1.6E-02	9.0E-03	1.3E-01
LGG	OBSL1_ES_13_12_14.1	OBSL1	ES	13	12	14.1	5.5E-39	0.307	0.464	2.9E-01	5.2E-01	8.3E-01
LGG	OCRL_ES_19_18_20	OCRL	ES	19	18	20	8.2E-24	0.211	0.098	1.8E-06	5.0E-07	9.3E-05
LGG	OPA1_ES_7_6_8	OPA1	ES	7	6	8	6.4E-14	0.129	-0.294	6.8E-02	6.0E-04	4.4E-03
LGG	OSBPL6_ES_16_15_17	OSBPL6	ES	16	15	17	3.5E-17	0.155	0.137	4.4E-02	1.7E-03	8.7E-05
LGG	OSBPL9_ES_17_15_18	OSBPL9	ES	17	15	18	1.4E-19	0.175	0.167	1.0E-01	6.1E-03	3.5E-03
LGG	OXLD1_AD_1.2:1.3:1.4_1.1_2	OXLD1	AD	1.2:1.3:1.4	1.1	2	1.1E-12	0.114	-0.059	7.7E-02	2.4E-01	4.0E-01
LGG	OXR1_ES_16_15.2_17	OXR1	ES	16	15.2	17	1.9E-30	0.255	0.129	3.3E-04	1.5E-03	1.7E-04
LGG	PAAF1_AA_4.1_3.2_4.2	PAAF1	AA	4.1	3.2	4.2	3.3E-16	0.145	-0.197	3.5E-02	1.3E-01	7.5E-02
LGG	PAAF1_ES_4.1:4.2_3.2_5	PAAF1	ES	4.1:4.2	3.2	5	2.8E-30	0.250	0.466	4.6E-06	3.7E-06	4.5E-04
LGG	PAAF1_ES_4.2_3.2_5	PAAF1	ES	4.2	3.2	5	3.1E-54	0.400	0.532	2.1E-08	1.6E-06	4.2E-09
LGG	PACRGL_ES_6:7:8_5_9	PACRGL	ES	6:07:08	5	9	1.4E-18	0.163	-0.064	3.5E-01	1.6E-01	3.1E-02
LGG	PACRGL_ES_6:8_5_9	PACRGL	ES	6:08	5	9	1.8E-17	0.158	-0.103	4.6E-02	5.1E-01	8.1E-02
LGG	PACRGL_ES_7:8_5_9	PACRGL	ES	7:08	5	9	7.9E-10	0.105	0.029	9.1E-01	9.6E-01	6.9E-01
LGG	PACRGL_ES_8_5_9	PACRGL	ES	8	5	9	1.8E-23	0.209	-0.121	1.2E-02	2.5E-02	5.3E-03
LGG	PACRGL_ES_9_5_13.1	PACRGL	ES	9	5	13.1	1.7E-20	0.223	-0.073	2.5E-01	3.3E-03	6.4E-03
LGG	PACSIN2_ES_12_11_13	PACSIN2	ES	12	11	13	8.8E-26	0.217	0.170	6.1E-06	8.3E-08	1.3E-07
LGG	PALLD_ES_25_24_26	PALLD	ES	25	24	26	1.6E-13	0.122	-0.211	6.4E-04	3.6E-03	6.5E-03
LGG	PALM_ES_8_7_9	PALM	ES	8	7	9	1.0E-29	0.245	-0.092	1.2E-04	3.5E-05	2.0E-05
LGG	PAM_ES_14_13_15	PAM	ES	14	13	15	1.1E-18	0.164	-0.104	2.9E-01	1.8E-01	1.0E-03
LGG	PAPOLA_ES_19_18_20	PAPOLA	ES	19	18	20	2.5E-25	0.215	0.008	1.0E-03	5.7E-07	4.7E-05
LGG	PAPOLA_ES_20_18_21	PAPOLA	ES	20	18	21	2.3E-15	0.139	0.007	6.8E-02	3.5E-03	3.3E-06
LGG	PAQR6_AD_4.2:4.3_4.1_6	PAQR6	AD	4.2:4.3	4.1	6	7.1E-12	0.129	0.223	2.2E-01	3.1E-01	5.8E-01
LGG	PAQR6_RI_4.2_4.1_4.3	PAQR6	RI	4.2	4.1	4.3	7.6E-18	0.157	-0.224	2.4E-01	3.0E-01	7.0E-01
LGG	PARD3_ES_13_12_14	PARD3	ES	13	12	14	4.4E-20	0.176	0.099	2.7E-01	7.2E-02	8.9E-01
LGG	PARD3_ES_20_19.3_21	PARD3	ES	20	19.3	21	8.9E-14	0.124	0.258	5.2E-01	6.2E-01	3.2E-01
LGG	PARP11_ES_2_1_3	PARP11	ES	2	1	3	7.7E-11	0.106	0.167	2.7E-01	4.7E-01	9.8E-01
LGG	PARP3_ES_2_1_3.1	PARP3	ES	2	1	3.1	2.3E-13	0.123	0.144	1.4E-01	1.7E-01	5.2E-01

cancer type	id	Gene Symbol	splice_type	Exon	From.Exon	To.Exon	anova.p	adj.r2	r	p.50	p.25	p.10
LGG	PARP3_ES_2:3.1_1_3.2	PARP3	ES	02:03.1	1	3.2	2.2E-16	0.146	0.166	5.0E-01	2.2E-01	2.4E-01
LGG	PARP6_ES_18.1:18.2_17_19	PARP6	ES	18.1:18.2	17	19	1.7E-12	0.113	0.238	2.2E-03	2.0E-03	4.4E-02
LGG	PARP6_ES_20_19_21	PARP6	ES	20	19	21	8.3E-38	0.300	0.393	3.3E-07	3.9E-05	8.2E-05
LGG	PATZ1_AA_5.1_4_5.2	PATZ1	AA	5.1	4	5.2	2.1E-14	0.129	-0.113	5.1E-02	2.2E-01	6.6E-01
LGG	PATZ1_ES_5.1:5.2_4_6	PATZ1	ES	5.1:5.2	4	6	8.6E-20	0.172	-0.179	2.2E-01	1.7E-01	1.1E-02
LGG	PAX6_ES_8_7_9	PAX6	ES	8	7	9	1.6E-19	0.174	0.159	7.3E-03	2.9E-04	7.0E-05
LGG	PBRM1_ES_28:29_27_30	PBRM1	ES	28:29:00	27	30	8.9E-16	0.144	-0.097	4.5E-01	5.4E-02	4.2E-01
LGG	PBRM1_ES_29_27_30	PBRM1	ES	29	27	30	2.1E-22	0.195	-0.191	2.5E-02	1.1E-01	4.9E-01
LGG	PBX1_ES_11_10_12	PBX1	ES	11	10	12	1.8E-12	0.113	0.043	1.1E-01	2.0E-02	1.1E-03
LGG	PC_ES_2_1_3	PC	ES	2	1	3	8.3E-12	0.108	0.286	1.4E-02	3.7E-03	2.6E-03
LGG	PCBP2_ES_15_14.1_16.1	PCBP2	ES	15	14.1	16.1	5.2E-109	0.635	-0.168	1.9E-06	1.5E-08	1.3E-06
LGG	PCBP2_ES_15_14.1_16.2	PCBP2	ES	15	14.1	16.2	1.5E-90	0.569	-0.196	6.4E-07	6.5E-06	1.5E-06
LGG	PCBP3_AD_11.2_11.1_12	PCBP3	AD	11.2	11.1	12	5.5E-37	0.322	0.363	8.6E-01	3.6E-02	4.9E-03
LGG	PCBP4_AD_9.2:9.3_9.1_10	PCBP4	AD	9.2:9.3	9.1	10	1.8E-19	0.170	0.062	2.8E-03	7.1E-02	2.9E-01
LGG	PCBP4_ES_3_2.2_5	PCBP4	ES	3	2.2	5	1.6E-15	0.138	0.164	2.6E-03	2.5E-04	2.5E-03
LGG	PCDH15_ES_39_37_40	PCDH15	ES	39	37	40	3.3E-28	0.297	0.113	7.9E-04	8.8E-04	3.8E-03
LGG	PCDH15_ES_41:44_40_45.2	PCDH15	ES	41:44:00	40	45.2	2.9E-14	0.143	0.026	7.5E-05	1.9E-04	9.5E-03
LGG	PCM1_ES_25:26_24_27	PCM1	ES	25:26:00	24	27	1.7E-17	0.155	-0.064	9.0E-02	9.0E-02	6.8E-01
LGG	PCM1_ES_26_25_27	PCM1	ES	26	25	27	1.1E-24	0.211	-0.071	1.9E-02	1.4E-02	9.5E-02
LGG	PCNP_AA_2.1_1_2.2	PCNP	AA	2.1	1	2.2	4.4E-20	0.175	-0.058	6.3E-04	5.8E-06	1.8E-05
LGG	PCNP_ES_2.1:2.2:2.3_1_3	PCNP	ES	2.1:2.2:2.3	1	3	6.9E-18	0.157	-0.021	6.4E-02	9.4E-03	4.6E-04
LGG	PCNXL2_RI_34.2_34.1_34.3	PCNXL2	RI	34.2	34.1	34.3	9.2E-20	0.172	-0.079	4.2E-02	1.1E-02	4.5E-01
LGG	PDCD10_ES_4.2_1.1_5	PDCD10	ES	4.2	1.1	5	3.3E-27	0.228	0.046	1.1E-04	1.4E-02	2.4E-04
LGG	PDCD2_RI_3.2_3.1_3.3	PDCD2	RI	3.2	3.1	3.3	1.5E-23	0.201	0.145	3.4E-01	1.0E-02	3.1E-02
LGG	PDCD6IP_AA_8.1_7_8.2	PDCD6IP	AA	8.1	7	8.2	1.0E-16	0.151	0.125	2.3E-02	1.1E-04	2.2E-03
LGG	PDE4DIP_AD_32.2_32.1_33	PDE4DIP	AD	32.2	32.1	33	4.2E-26	0.222	-0.008	1.6E-01	1.1E-04	1.5E-01
LGG	PDE9A_ES_6_5_7	PDE9A	ES	6	5	7	4.7E-20	0.174	0.000	8.9E-01	6.5E-01	3.5E-01
LGG	PDLIM7_ES_6_5_7	PDLIM7	ES	6	5	7	2.7E-21	0.187	-0.199	1.2E-06	4.8E-07	4.9E-05
LGG	PDPR_ES_2.1:2.2_1_3.2	PDPR	ES	2.1:2.2	1	3.2	1.9E-10	0.106	-0.051	3.3E-01	3.0E-01	3.9E-01
LGG	PDZD4_AD_3.2_3.1_5	PDZD4	AD	3.2	3.1	5	2.0E-13	0.121	-0.164	2.4E-01	7.1E-02	6.7E-01
LGG	PEA15_ES_2:3:4.1:4.2_1_5	PEA15	ES	2:3:4.1:4.2	1	5	2.5E-10	0.104	-0.047	4.8E-01	7.0E-01	3.8E-01
LGG	PEX1_ES_9_8_10	PEX1	ES	9	8	10	2.2E-11	0.108	-0.217	9.0E-01	5.4E-02	6.4E-02
LGG	PEX11A_ES_2_1_3.1	PEX11A	ES	2	1	3.1	1.8E-11	0.104	0.041	1.2E-04	7.0E-03	2.1E-01
LGG	PEX2_ES_4.2_2.2_5	PEX2	ES	4.2	2.2	5	1.3E-19	0.173	-0.149	8.8E-03	5.0E-02	2.6E-01
LGG	PEX5_ES_9_8_10	PEX5	ES	9	8	10	2.1E-24	0.207	-0.085	9.1E-01	7.9E-01	1.3E-01
LGG	PFDN5_ES_2:4.1:4.2_1_5	PFDN5	ES	2:4.1:4.2	1	5	2.8E-17	0.163	-0.045	3.4E-01	9.1E-01	2.5E-01
LGG	PFDN5_ES_2:4.2_1_5	PFDN5	ES	02:04.2	1	5	5.9E-14	0.143	-0.054	6.8E-02	1.6E-01	5.9E-01
LGG	PFKM_AD_16.2:16.3_16.1_17	PFKM	AD	16.2:16.3	16.1	17	2.6E-12	0.112	0.135	6.7E-02	1.4E-01	1.7E-02
LGG	PGAP2_ES_8:9_6_10	PGAP2	ES	8:09	6	10	1.7E-18	0.177	0.268	3.8E-01	5.7E-02	6.9E-01
LGG	PHF21A_ME_16|17_15_18	PHF21A	ME	16|17	15	18	2.7E-12	0.111	-0.028	4.8E-04	2.9E-04	3.1E-02

cancer type	id	Gene Symbol	splice_type	Exon	From.Exon	To.Exon	anova.p	adj.r2	r	p.50	p.25	p.10
LGG	PHF21B_ES_11_10_12	PHF21B	ES	11	10	12	4.5E-23	0.228	0.315	1.6E-02	3.4E-02	1.3E-01
LGG	PHF6_RI_10.2_10.1_10.3	PHF6	RI	10.2	10.1	10.3	2.6E-30	0.254	0.082	3.3E-01	1.9E-02	5.3E-02
LGG	PHKB_ES_2_1_3	PHKB	ES	2	1	3	9.3E-14	0.129	-0.317	3.7E-02	6.2E-02	3.6E-02
LGG	PHLDB1_ES_16_14_17	PHLDB1	ES	16	14	17	1.7E-21	0.185	0.221	1.4E-06	3.3E-04	1.8E-04
LGG	PHYHD1_RI_1.2_1.1_1.3	PHYHD1	RI	1.2	1.1	1.3	1.8E-19	0.169	-0.283	1.7E-03	2.9E-03	6.7E-03
LGG	PHYKPL_ES_2_1_3	PHYKPL	ES	2	1	3	2.8E-13	0.120	0.046	7.4E-01	7.1E-01	3.1E-01
LGG	PI4KB_ES_5_4_6	PI4KB	ES	5	4	6	5.1E-15	0.134	-0.159	2.5E-04	7.0E-04	1.6E-04
LGG	PICALM_ES_14.1:14.2_13_15	PICALM	ES	14.1:14.2	13	15	7.0E-13	0.116	-0.223	2.2E-03	3.4E-04	8.2E-05
LGG	PICALM_ES_19_18_20	PICALM	ES	19	18	20	1.4E-14	0.130	0.263	4.7E-03	8.0E-04	9.2E-01
LGG	PIDD_AD_12.2_12.1_13	PIDD	AD	12.2	12.1	13	4.1E-14	0.127	0.000	2.4E-02	3.7E-03	6.5E-03
LGG	PIGX_ES_5_4_6.1	PIGX	ES	5	4	6.1	4.4E-34	0.275	-0.195	5.4E-04	1.1E-05	1.5E-06
LGG	PINX1_ES_6_5_8	PINX1	ES	6	5	8	8.6E-20	0.172	0.101	4.9E-08	6.0E-09	3.6E-06
LGG	PITPNM2_ES_21_20_22	PITPNM2	ES	21	20	22	6.8E-12	0.117	-0.187	1.0E-03	1.6E-03	7.9E-03
LGG	PLA2G6_ES_11:12_10_13	PLA2G6	ES	11:12	10	13	1.0E-20	0.185	-0.192	1.4E-05	7.4E-03	2.5E-05
LGG	PLA2G6_ES_12_11_13	PLA2G6	ES	12	11	13	1.5E-40	0.321	-0.277	6.8E-04	5.7E-05	1.3E-05
LGG	PLA2G6_ES_14_13_15	PLA2G6	ES	14	13	15	6.2E-29	0.240	-0.169	1.1E-03	2.7E-04	3.0E-03
LGG	PLB1_ES_42:43:44_41_45	PLB1	ES	42:43:44	41	45	5.0E-11	0.116	-0.184	3.5E-01	5.9E-01	5.0E-01
LGG	PLCB2_ES_29_28_30	PLCB2	ES	29	28	30	4.2E-17	0.151	0.467	2.1E-01	4.1E-02	2.0E-01
LGG	PLD3_ES_1.2:5.1_1.1_5.2	PLD3	ES	1.2:5.1	1.1	5.2	8.0E-23	0.204	-0.098	7.5E-01	9.9E-01	6.1E-01
LGG	PLEKHA3_ES_3_2_4	PLEKHA3	ES	3	2	4	4.1E-23	0.201	0.045	4.7E-01	1.1E-01	2.6E-01
LGG	PLEKHA5_ES_27_26_28	PLEKHA5	ES	27	26	28	6.2E-22	0.193	-0.274	1.7E-01	3.5E-03	8.5E-03
LGG	PLK1_AD_1.2_1.1_2	PLK1	AD	1.2	1.1	2	1.1E-17	0.184	-0.582	1.1E-05	1.6E-05	3.1E-04
LGG	PLOD2_ES_15_14_16	PLOD2	ES	15	14	16	8.9E-39	0.313	-0.292	2.9E-03	2.3E-04	9.4E-05
LGG	PLSCR1_ES_6_5_7	PLSCR1	ES	6	5	7	1.9E-11	0.104	0.226	4.6E-03	1.4E-03	1.7E-02
LGG	PLXNB3_ES_3_2_4	PLXNB3	ES	3	2	4	8.0E-31	0.259	0.407	5.1E-06	1.1E-02	8.6E-02
LGG	PNISR_ES_2_1_3	PNISR	ES	2	1	3	5.3E-27	0.227	-0.271	4.5E-04	2.1E-04	1.1E-03
LGG	POFUT2_ES_8.3_8.1_8.5	POFUT2	ES	8.3	8.1	8.5	2.9E-17	0.154	0.410	8.7E-02	4.7E-01	2.6E-01
LGG	POLB_ES_2_1_3	POLB	ES	2	1	3	1.1E-11	0.106	-0.148	5.5E-01	5.3E-02	2.5E-01
LGG	POLDIP3_ES_3.1:3.2_2_4	POLDIP3	ES	3.1:3.2	2	4	5.0E-11	0.104	-0.277	1.7E-01	2.1E-01	5.6E-02
LGG	POLM_ES_6.2:8_6.1_9.1	POLM	ES	6.2:8	6.1	9.1	3.0E-12	0.112	0.096	5.1E-01	5.8E-01	6.1E-01
LGG	POLM_ES_9.4:9.5_9.2_9.7	POLM	ES	9.4:9.5	9.2	9.7	3.7E-10	0.104	-0.035	4.6E-01	8.6E-01	5.4E-01
LGG	POLR2F_ES_6:7:8.1:8.2_4.1_11	POLR2F	ES	6:7:8.1:8.2	4.1	11	2.6E-13	0.135	-0.081	1.7E-04	2.3E-02	1.0E-03
LGG	POLR2H_ES_5:6.1_4_6.2	POLR2H	ES	05:06.1	4	6.2	2.5E-13	0.120	0.144	2.3E-03	5.6E-05	2.0E-04
LGG	POLR2J3_ES_4.5_4.3_8	POLR2J3	ES	4.5	4.3	8	6.0E-18	0.157	0.216	6.0E-03	1.0E-03	3.9E-01
LGG	POLR2J3_RI_4.4_4.3_4.5	POLR2J3	RI	4.4	4.3	4.5	5.9E-19	0.165	-0.268	2.3E-01	3.5E-01	7.4E-01
LGG	POLR3H_ES_4:5.1_3_6	POLR3H	ES	04:05.1	3	6	1.1E-29	0.248	-0.169	1.4E-04	5.1E-05	1.7E-03
LGG	POLR3H_ES_4:5.1:5.2_3_6	POLR3H	ES	4:5.1:5.2	3	6	1.4E-23	0.216	-0.234	6.8E-03	8.4E-04	1.4E-03
LGG	POMGNT2_ES_2_1_3	POMGNT2	ES	2	1	3	3.7E-39	0.311	0.252	3.3E-05	8.9E-07	2.2E-05
LGG	POMT1_ES_1.2:2:3_1.1_4	POMT1	ES	1.2:2:3	1.1	4	1.5E-14	0.161	0.234	3.2E-03	2.5E-04	9.0E-03
LGG	POMT1_ES_3_1.1_4	POMT1	ES	3	1.1	4	1.1E-21	0.200	0.170	5.5E-03	8.9E-05	1.7E-02

cancer type	id	Gene Symbol	splice_type	Exon	From.Exon	To.Exon	anova.p	adj.r2	r	p.50	p.25	p.10
LGG	PORCN_ES_7:8_6_9	PORCN	ES	7:08	6	9	4.9E-72	0.488	0.433	6.4E-06	1.7E-08	7.7E-07
LGG	PORCN_ES_8_7_9	PORCN	ES	8	7	9	1.1E-55	0.423	0.381	8.1E-07	1.4E-07	3.3E-05
LGG	PPAP2A_ES_2_1_4	PPAP2A	ES	2	1	4	2.6E-15	0.137	0.370	7.3E-03	1.3E-03	5.2E-02
LGG	PPAPDC1A_ES_4_3_7	PPAPDC1A	ES	4	3	7	4.8E-09	0.102	-0.063	3.4E-01	8.1E-02	1.3E-01
LGG	PPFIA1_ES_17_16_18	PPFIA1	ES	17	16	18	1.2E-36	0.297	-0.413	3.5E-05	2.1E-08	2.3E-06
LGG	PPFIA3_RI_29.2_29.1_29.3	PPFIA3	RI	29.2	29.1	29.3	2.5E-35	0.283	-0.586	1.0E-05	7.5E-07	1.6E-05
LGG	PPFIBP1_ES_20_19_21	PPFIBP1	ES	20	19	21	1.0E-13	0.149	-0.064	3.4E-04	1.0E-03	6.5E-03
LGG	PPHLN1_ES_10_9_11	PPHLN1	ES	10	9	11	6.4E-39	0.307	-0.053	3.9E-05	1.3E-04	2.8E-03
LGG	PPHLN1_ES_6_5_7	PPHLN1	ES	6	5	7	4.7E-29	0.241	0.097	1.0E-02	8.1E-05	2.6E-04
LGG	PPIE_ES_11_9.1_12	PPIE	ES	11	9.1	12	5.5E-25	0.211	0.254	2.0E-02	1.2E-04	2.0E-02
LGG	PPIL2_AA_22.4:22.5_22.2_22.6	PPIL2	AA	22.4:22.5	22.2	22.6	1.8E-11	0.104	0.171	5.2E-02	4.1E-02	4.1E-02
LGG	PPIL3_AD_1.2_1.1_2.2	PPIL3	AD	1.2	1.1	2.2	1.6E-26	0.222	-0.508	1.6E-06	2.6E-07	1.6E-04
LGG	PPIL3_ES_2.2:3:4.1_1.1_4.2	PPIL3	ES	2.2:3:4.1	1.1	4.2	3.5E-12	0.113	0.409	3.4E-02	7.0E-03	4.2E-03
LGG	PPIP5K2_ES_26_25_27	PPIP5K2	ES	26	25	27	4.6E-14	0.135	0.181	1.4E-03	2.5E-02	2.1E-01
LGG	PPP1R12A_ES_15_14_16	PPP1R12A	ES	15	14	16	1.5E-12	0.115	-0.010	6.2E-02	1.6E-02	8.5E-01
LGG	PPP2R2B_ES_5:8.4_1.4_10	PPP2R2B	ES	05:08.4	1.4	10	1.1E-11	0.109	-0.114	1.5E-01	2.5E-01	3.5E-01
LGG	PPP2R2B_RI_1.3_1.2_1.4	PPP2R2B	RI	1.3	1.2	1.4	2.5E-19	0.173	0.000	1.6E-02	3.1E-02	1.0E-03
LGG	PPP2R3C_ES_3_1.3_4.2	PPP2R3C	ES	3	1.3	4.2	4.3E-21	0.182	-0.157	5.2E-02	3.2E-03	7.6E-05
LGG	PPP3CA_ES_13_12_14	PPP3CA	ES	13	12	14	5.2E-45	0.348	0.543	3.4E-06	2.1E-07	3.2E-06
LGG	PPP3CC_ES_14_13_15	PPP3CC	ES	14	13	15	2.8E-34	0.276	-0.081	3.2E-03	7.7E-03	3.3E-02
LGG	PPP5C_ES_5_4_6	PPP5C	ES	5	4	6	3.4E-21	0.183	-0.121	1.7E-02	1.4E-01	1.2E-01
LGG	PPP6R2_ES_17_16_18	PPP6R2	ES	17	16	18	5.7E-17	0.150	-0.164	8.6E-01	9.6E-01	8.3E-01
LGG	PQBP1_AD_1.2_1.1_2.3	PQBP1	AD	1.2	1.1	2.3	1.3E-23	0.205	-0.320	5.5E-04	4.2E-04	2.6E-04
LGG	PQBP1_AD_1.2:1.3_1.1_2.3	PQBP1	AD	1.2:1.3	1.1	2.3	2.9E-19	0.168	-0.327	5.7E-03	1.2E-04	8.8E-04
LGG	PQLC1_ES_6:7_5_9	PQLC1	ES	6:07	5	9	8.9E-13	0.115	0.222	3.7E-04	1.3E-03	2.8E-02
LGG	PQLC1_ES_6:7:9_5_10	PQLC1	ES	6:07:09	5	10	1.2E-24	0.209	0.033	2.7E-02	2.3E-07	1.6E-04
LGG	PQLC1_ES_7:9_6_10	PQLC1	ES	7:09	6	10	6.3E-11	0.120	0.001	9.2E-02	6.7E-03	3.4E-02
LGG	PQLC3_ES_6:7_5_8	PQLC3	ES	6:07	5	8	3.1E-14	0.161	-0.315	4.4E-02	6.2E-04	2.9E-04
LGG	PRKAB2_ES_3_2_4	PRKAB2	ES	3	2	4	2.2E-16	0.152	0.232	2.5E-02	7.7E-03	2.8E-02
LGG	PRKD1_ES_5_4_6	PRKD1	ES	5	4	6	9.2E-15	0.137	-0.058	6.1E-03	5.9E-03	4.4E-03
LGG	PRKRIP1_ES_6_4_7	PRKRIP1	ES	6	4	7	1.1E-11	0.107	0.343	1.8E-02	1.0E-04	1.1E-06
LGG	PRMT1_ES_4.2:5_4.1_6	PRMT1	ES	4.2:5	4.1	6	2.0E-29	0.243	-0.377	4.4E-04	4.6E-05	6.2E-04
LGG	PRPF3_ES_4_3_5	PRPF3	ES	4	3	5	7.2E-14	0.125	0.381	1.8E-01	2.5E-01	3.5E-01
LGG	PRRX1_ES_4_3.1_5	PRRX1	ES	4	3.1	5	6.5E-13	0.118	0.029	3.7E-01	2.0E-01	4.2E-01
LGG	PSIP1_AA_11.1_10_11.2	PSIP1	AA	11.1	10	11.2	6.3E-14	0.125	-0.029	7.9E-01	4.2E-01	6.8E-02
LGG	PSMB7_ES_6_4_7.1	PSMB7	ES	6	4	7.1	1.0E-15	0.147	0.337	3.0E-05	6.4E-04	3.7E-03
LGG	PSMC3IP_AD_4.2:4.3_4.1_5.1	PSMC3IP	AD	4.2:4.3	4.1	5.1	1.2E-11	0.113	-0.344	2.5E-02	1.1E-05	4.6E-08
LGG	PSTPIP1_ES_3_2.2_5	PSTPIP1	ES	3	2.2	5	2.8E-64	0.451	-0.776	3.2E-07	1.9E-07	8.5E-05
LGG	PTBP2_ES_12_11.2_13	PTBP2	ES	12	11.2	13	7.7E-39	0.310	0.338	8.0E-05	1.1E-03	7.0E-04
LGG	PTGR1_AA_12.1_11_12.2	PTGR1	AA	12.1	11	12.2	4.4E-12	0.109	-0.643	2.2E-02	1.8E-03	4.5E-04

cancer type	id	Gene Symbol	splice_type	Exon	From.Exon	To.Exon	anova.p	adj.r2	r	p.50	p.25	p.10
LGG	PTGR2_AD_1.2_1.1_2	PTGR2	AD	1.2	1.1	2	1.4E-11	0.108	0.294	3.3E-01	4.4E-02	1.5E-02
LGG	PTGR2_AD_1.2:1.3_1.1_2	PTGR2	AD	1.2:1.3	1.1	2	9.6E-27	0.228	0.406	3.7E-03	1.6E-04	4.3E-04
LGG	PTGR2_AD_1.3_1.2_2	PTGR2	AD	1.3	1.2	2	5.8E-25	0.214	0.181	2.2E-03	1.9E-03	2.7E-05
LGG	PTK2_AA_39.2:39.3:39.4_37_39.5	PTK2	AA	39.2:39.3:39.4	37	39.5	1.8E-11	0.104	0.135	1.6E-02	1.7E-03	4.8E-03
LGG	PTK2_ES_20_19_21.2	PTK2	ES	20	19	21.2	2.4E-41	0.328	0.019	1.3E-07	2.2E-07	7.0E-10
LGG	PTK2_ES_39.2_37_39.5	PTK2	ES	39.2	37	39.5	7.8E-18	0.157	0.006	2.4E-05	6.2E-06	1.9E-04
LGG	PTMS_ES_3.2_2_5	PTMS	ES	3.2	2	5	3.0E-22	0.201	0.088	1.4E-01	2.0E-02	4.7E-01
LGG	PTPN13_ES_21_20_22	PTPN13	ES	21	20	22	1.5E-11	0.115	0.083	2.8E-03	1.8E-02	9.8E-02
LGG	PTPRA_ES_12_11_13	PTPRA	ES	12	11	13	2.8E-21	0.184	0.365	1.4E-06	2.3E-07	2.5E-04
LGG	PTPRA_ES_8:9_7_10	PTPRA	ES	8:09	7	10	4.5E-22	0.194	-0.170	3.9E-05	2.0E-06	1.5E-02
LGG	PTPRF_ES_12_11_13	PTPRF	ES	12	11	13	3.5E-11	0.103	-0.030	2.4E-05	1.3E-04	4.4E-03
LGG	PTPRK_ES_24_23.1_25	PTPRK	ES	24	23.1	25	6.9E-21	0.191	0.320	9.4E-05	1.5E-07	3.2E-04
LGG	PTPRS_ES_2.2:3:4:6.1:6.2:9.2:11:12:13:1	PTPRS	ES	:11:12:13:14:20:2	1	29	6.6E-09	0.101	-0.093	3.4E-01	2.9E-01	6.0E-01
LGG	PTPRZ1_AD_12.2_12.1_13	PTPRZ1	AD	12.2	12.1	13	3.7E-52	0.387	0.146	1.2E-08	6.6E-06	8.5E-05
LGG	PTPRZ1_ES_16_15_17	PTPRZ1	ES	16	15	17	8.3E-23	0.195	-0.168	2.4E-04	2.3E-06	9.8E-06
LGG	PTS_ES_4_3_5	PTS	ES	4	3	5	3.1E-50	0.375	-0.074	7.1E-04	6.2E-07	3.7E-05
LGG	PUM1_AD_18.2_18.1_19	PUM1	AD	18.2	18.1	19	5.1E-17	0.153	-0.057	3.5E-03	1.4E-03	9.9E-03
LGG	PUM2_ES_16_15_17	PUM2	ES	16	15	17	1.5E-21	0.189	-0.304	1.2E-05	1.9E-04	1.5E-03
LGG	PVRL3_AA_6.1_5_6.2	PVRL3	AA	6.1	5	6.2	6.8E-57	0.434	-0.092	1.5E-03	3.3E-05	9.8E-04
LGG	PXN_ES_13_9_14.2	PXN	ES	13	9	14.2	5.3E-21	0.183	-0.205	4.7E-01	6.3E-03	8.0E-02
LGG	QKI_AA_8.4_8.2_8.5	QKI	AA	8.4	8.2	8.5	1.4E-12	0.113	-0.134	4.1E-05	5.3E-04	7.0E-02
LGG	R3HDM1_ES_16_15_17	R3HDM1	ES	16	15	17	3.0E-25	0.216	0.445	7.2E-04	4.0E-07	3.0E-03
LGG	R3HDM4_ES_2:3.1_1_3.3	R3HDM4	ES	02:03.1	1	3.3	4.3E-14	0.126	-0.282	4.4E-04	7.9E-04	2.6E-03
LGG	RAB1A_ES_7_5_8	RAB1A	ES	7	5	8	1.2E-12	0.116	-0.026	1.3E-01	2.8E-01	4.3E-01
LGG	RAB6A_ME_5|6_4_7	RAB6A	ME	5|6	4	7	3.0E-14	0.128	-0.540	1.1E-02	6.1E-04	8.1E-04
LGG	RAB7A_ES_4.1:5.1:5.2_3.2_6	RAB7A	ES	4.1:5.1:5.2	3.2	6	3.7E-09	0.100	-0.025	2.6E-02	2.8E-02	3.0E-01
LGG	RAD1_ES_6:7_5_8	RAD1	ES	6:07	5	8	4.7E-12	0.116	-0.029	1.2E-03	1.5E-02	9.3E-03
LGG	RAD1_ES_7_5_8	RAD1	ES	7	5	8	6.4E-13	0.117	0.025	7.2E-03	5.7E-03	1.7E-03
LGG	RAD51D_ES_5:6:7:8_4_9	RAD51D	ES	5:6:7:8	4	9	3.2E-11	0.118	0.011	4.4E-01	4.4E-01	5.5E-01
LGG	RAD51D_ES_5:7:8_4_9	RAD51D	ES	5:07:08	4	9	1.5E-15	0.139	0.082	7.3E-02	8.8E-02	7.5E-01
LGG	RAD51D_ES_6:7:8_4_9	RAD51D	ES	6:07:08	4	9	9.3E-10	0.108	0.137	6.0E-01	3.6E-01	5.6E-01
LGG	RAD52_ES_9_8_10	RAD52	ES	9	8	10	5.4E-20	0.179	0.298	2.3E-02	1.3E-03	3.3E-02
LGG	RALGAPA1_ES_17_16_18	RALGAPA1	ES	17	16	18	1.8E-23	0.214	0.309	3.2E-03	4.9E-04	1.6E-03
LGG	RALGAPB_AA_16.1_15_16.2	RALGAPB	AA	16.1	15	16.2	3.5E-09	0.101	0.041	2.6E-01	2.1E-01	1.4E-03
LGG	RALGDS_AA_13.1_12_13.2	RALGDS	AA	13.1	12	13.2	3.3E-25	0.214	-0.123	2.6E-05	3.1E-05	5.1E-04
LGG	RALGPS2_ES_15_14_16	RALGPS2	ES	15	14	16	6.4E-19	0.211	0.081	8.5E-01	4.5E-01	8.3E-01
LGG	RANBP17_ES_30_29_31	RANBP17	ES	30	29	31	2.3E-35	0.301	-0.739	1.1E-01	2.5E-02	1.0E-01
LGG	RANBP3_ES_5_4.1_7	RANBP3	ES	5	4.1	7	7.9E-19	0.165	-0.043	2.0E-02	1.4E-02	1.0E-03
LGG	RANGRF_AD_3.2:3.3_3.1_3.5	RANGRF	AD	3.2:3.3	3.1	3.5	9.4E-15	0.132	-0.023	9.6E-01	3.8E-01	7.5E-01
LGG	RANGRF_ES_3.3_3.1_3.5	RANGRF	ES	3.3	3.1	3.5	4.7E-14	0.126	-0.007	6.6E-01	3.2E-01	1.4E-01

cancer type	id	Gene Symbol	splice_type	Exon	From.Exon	To.Exon	anova.p	adj.r2	r	p.50	p.25	p.10
LGG	RAP1GDS1_ES_6_5_7	RAP1GDS1	ES	6	5	7	8.5E-13	0.116	-0.432	4.7E-02	3.5E-04	1.3E-04
LGG	RAPGEF6_ES_23_22_24	RAPGEF6	ES	23	22	24	1.0E-11	0.138	-0.071	2.9E-01	7.0E-02	2.6E-01
LGG	RASAL2_AA_14.1_13_14.2	RASAL2	AA	14.1	13	14.2	7.3E-15	0.172	-0.241	3.2E-04	6.3E-03	2.4E-02
LGG	RBBP5_AD_13.2_13.1_14	RBBP5	AD	13.2	13.1	14	2.0E-11	0.105	0.027	2.3E-03	1.0E-04	4.0E-03
LGG	RBFOX3_ES_10_9_11	RBFOX3	ES	10	9	11	3.5E-13	0.151	0.164	1.4E-01	5.5E-02	1.8E-01
LGG	RBM41_ES_5_4_6.1	RBM41	ES	5	4	6.1	2.2E-10	0.102	-0.026	1.5E-03	1.6E-06	1.1E-11
LGG	RBM42_ES_3.2:4:6.3:7:8:9.1_3.1_9.2	RBM42	ES	3.2:4:6.3:7:8:9.1	3.1	9.2	5.4E-10	0.117	-0.207	1.5E-02	1.5E-02	2.3E-02
LGG	RBM6_ES_3.1:3.2_2_4	RBM6	ES	3.1:3.2	2	4	8.3E-25	0.213	0.111	1.6E-01	2.4E-01	5.6E-01
LGG	RBM6_ES_3.1:3.2:4:5:6_2_7	RBM6	ES	3.1:3.2:4:5:6	2	7	8.3E-27	0.228	0.047	2.7E-01	1.8E-02	8.4E-02
LGG	RBM6_ES_4:5_2_7	RBM6	ES	4:05	2	7	2.3E-11	0.107	0.268	1.5E-02	3.8E-02	3.5E-02
LGG	RBM6_ES_6_5_7	RBM6	ES	6	5	7	1.1E-27	0.231	-0.399	7.6E-02	1.2E-02	1.9E-01
LGG	RBPJ_ES_4.2_2.3_5	RBPJ	ES	4.2	2.3	5	1.5E-14	0.131	-0.108	8.0E-01	1.2E-01	3.9E-03
LGG	RCSD1_ES_3_2_4	RCSD1	ES	3	2	4	5.4E-11	0.102	0.363	1.9E-01	5.4E-01	9.6E-01
LGG	REC8_RI_1.2_1.1_1.3	REC8	RI	1.2	1.1	1.3	2.4E-21	0.186	-0.216	6.3E-01	2.4E-01	1.3E-03
LGG	RECQL5_ES_1.2:2.1_1.1_2.2	RECQL5	ES	1.2:2.1	1.1	2.2	3.5E-14	0.131	0.054	1.5E-02	1.8E-01	3.3E-02
LGG	REPS1_ES_9.3_9.1_10	REPS1	ES	9.3	9.1	10	2.3E-14	0.128	0.050	1.2E-03	1.6E-05	1.4E-03
LGG	RERGL_ES_2_1_3	RERGL	ES	2	1	3	9.6E-14	0.142	0.018	1.4E-01	2.4E-01	2.2E-01
LGG	REV1_ES_4_3_5	REV1	ES	4	3	5	4.5E-23	0.216	-0.009	1.0E-02	6.6E-03	3.1E-05
LGG	RFC5_ES_2.1_1_3	RFC5	ES	2.1	1	3	2.6E-17	0.157	0.015	8.6E-03	8.7E-03	2.5E-01
LGG	RHBDL3_ES_2.2_1_3	RHBDL3	ES	2.2	1	3	4.7E-19	0.186	-0.255	1.2E-02	3.4E-03	4.8E-03
LGG	RHNO1_ES_2.1_1_4	RHNO1	ES	2.1	1	4	2.9E-16	0.149	0.019	3.4E-04	6.4E-06	4.2E-05
LGG	RHOC_ES_2.2:2.3_1.1_3	RHOC	ES	2.2:2.3	1.1	3	3.2E-16	0.144	-0.249	1.4E-01	4.8E-01	3.5E-01
LGG	RIF1_ES_31_30_32	RIF1	ES	31	30	32	2.4E-19	0.172	0.046	1.0E-03	1.4E-05	3.6E-05
LGG	RIMKLB_ES_3_2_4	RIMKLB	ES	3	2	4	2.2E-16	0.146	-0.150	7.8E-03	6.2E-04	1.1E-02
LGG	RIMS2_ME_7|8_6_9	RIMS2	ME	7|8	6	9	1.1E-10	0.123	0.064	2.2E-02	2.4E-01	3.9E-01
LGG	RIPK2_ES_2:3_1_4	RIPK2	ES	2:03	1	4	7.4E-11	0.113	-0.258	7.0E-02	1.0E-01	3.4E-01
LGG	RIPK2_ES_3_1_4	RIPK2	ES	3	1	4	2.1E-25	0.216	-0.382	4.2E-05	8.4E-07	7.2E-04
LGG	RMND5B_ES_2:3_1_4.1	RMND5B	ES	2:03	1	4.1	2.3E-21	0.189	0.363	1.8E-04	2.3E-05	1.9E-04
LGG	RNF14_ES_6_4_7	RNF14	ES	6	4	7	5.9E-11	0.100	-0.237	7.8E-01	9.4E-01	6.9E-01
LGG	RNF146_AD_1.2:1.3_1.1_2	RNF146	AD	1.2:1.3	1.1	2	2.5E-12	0.111	0.165	1.8E-01	7.5E-02	3.1E-01
LGG	RNF146_ES_4:5.1_2_6	RNF146	ES	04:05.1	2	6	1.2E-11	0.106	0.028	2.5E-01	3.6E-01	2.7E-01
LGG	RNF146_ES_5.1_2_6	RNF146	ES	5.1	2	6	2.2E-15	0.139	-0.183	9.0E-01	5.2E-01	7.8E-01
LGG	RNF32_AA_12.1_11_12.2	RNF32	AA	12.1	11	12.2	1.2E-33	0.281	-0.736	7.5E-03	2.1E-02	1.3E-02
LGG	RNFT1_ES_3:4_2_5	RNFT1	ES	3:04	2	5	6.4E-18	0.160	0.405	1.5E-02	2.7E-04	2.3E-02
LGG	RNH1_AA_4.2_1_4.3	RNH1	AA	4.2	1	4.3	1.9E-12	0.112	-0.002	1.7E-01	1.4E-02	1.6E-03
LGG	RNH1_AA_4.2_3_4.3	RNH1	AA	4.2	3	4.3	1.7E-20	0.177	0.007	8.7E-04	1.1E-04	1.5E-02
LGG	RNPS1_ES_3_1.1_4	RNPS1	ES	3	1.1	4	4.4E-20	0.174	0.226	6.9E-04	3.5E-05	3.5E-03
LGG	ROBO1_ES_14_13_15	ROBO1	ES	14	13	15	2.0E-27	0.260	-0.308	3.6E-02	1.7E-04	2.3E-02
LGG	ROBO1_ES_26_25_27	ROBO1	ES	26	25	27	1.7E-14	0.136	-0.035	5.5E-02	3.1E-02	3.7E-02
LGG	ROBO2_ES_27_26_28	ROBO2	ES	27	26	28	2.7E-51	0.440	0.185	6.0E-06	4.2E-06	2.2E-06

cancer type	id	Gene Symbol	splice_type	Exon	From.Exon	To.Exon	anova.p	adj.r2	r	p.50	p.25	p.10
LGG	RPAIN_ES_4:5:6.1_3_7	RPAIN	ES	05:06.1	3	7	3.4E-24	0.205	-0.175	1.9E-04	4.4E-06	1.6E-05
LGG	RPAIN_ES_5_3_6.1	RPAIN	ES	5	3	6.1	2.2E-12	0.113	-0.260	1.0E-02	3.6E-03	8.7E-02
LGG	RPAIN_ES_5_4_7	RPAIN	ES	5	4	7	1.3E-46	0.354	-0.290	1.3E-07	1.2E-10	1.6E-09
LGG	RPAIN_ES_5:6.1_3_7	RPAIN	ES	05:06.1	3	7	9.2E-35	0.280	-0.277	2.5E-04	1.8E-09	1.4E-06
LGG	RPAIN_ES_5:6.1_4_7	RPAIN	ES	05:06.1	4	7	1.1E-29	0.245	-0.136	1.7E-05	1.7E-08	6.9E-09
LGG	RPL10_RI_2.2_2.1_2.3	RPL10	RI	2.2	2.1	2.3	2.3E-20	0.176	0.042	1.4E-01	7.8E-01	8.3E-01
LGG	RPL13A_ES_2.1:2.2_1_3	RPL13A	ES	2.1:2.2	1	3	5.3E-09	0.100	0.035	2.2E-01	5.1E-02	2.7E-01
LGG	RPL22L1_AA_3.1_2.2_3.2	RPL22L1	AA	3.1	2.2	3.2	5.9E-75	0.502	-0.624	3.6E-01	1.7E-01	5.1E-02
LGG	RPL30_ES_2.2:3.1_2.1_3.2	RPL30	ES	2.2:3.1	2.1	3.2	3.6E-39	0.308	-0.319	4.1E-01	2.3E-02	1.5E-02
LGG	RPL32_RI_1.2_1.1_1.3	RPL32	RI	1.2	1.1	1.3	1.7E-27	0.230	-0.359	6.5E-07	1.0E-08	1.5E-10
LGG	RPLP0_AD_4.2_4.1_5.1	RPLP0	AD	4.2	4.1	5.1	1.5E-10	0.118	-0.057	2.1E-02	2.9E-01	8.8E-01
LGG	RPLP0_ES_4.2:5.1_4.1_5.2	RPLP0	ES	4.2:5.1	4.1	5.2	1.8E-42	0.381	-0.134	4.2E-03	5.1E-04	5.0E-02
LGG	RPN2_ES_17_16_18	RPN2	ES	17	16	18	1.2E-24	0.209	-0.577	1.6E-03	1.5E-04	1.6E-03
LGG	RPP14_AD_1.2_1.1_2.2	RPP14	AD	1.2	1.1	2.2	1.3E-12	0.118	-0.153	8.6E-03	5.7E-02	6.1E-03
LGG	RPRD2_ES_4_3.1_5	RPRD2	ES	4	3.1	5	3.2E-15	0.141	-0.203	9.0E-02	1.4E-03	4.7E-03
LGG	RPS15_ES_1.2:1.4_1.1_1.5	RPS15	ES	1.2:1.4	1.1	1.5	1.5E-30	0.251	-0.053	1.1E-01	5.3E-02	2.5E-02
LGG	RPS15_RI_1.3_1.2_1.4	RPS15	RI	1.3	1.2	1.4	1.7E-31	0.258	0.111	2.3E-02	2.3E-03	1.4E-02
LGG	RPS20_AA_2.1:2.2_1.4_2.3	RPS20	AA	2.1:2.2	1.4	2.3	3.9E-16	0.143	-0.341	2.3E-04	5.2E-04	8.4E-04
LGG	RPS20_RI_1.3_1.2_1.4	RPS20	RI	1.3	1.2	1.4	1.0E-12	0.115	-0.206	2.5E-02	2.1E-03	1.2E-02
LGG	RPS24_ES_5.1:5.2_4_6	RPS24	ES	5.1:5.2	4	6	4.0E-17	0.151	0.177	6.7E-03	1.6E-04	1.9E-05
LGG	RPS25_ES_2.1:2.2_1_3.1	RPS25	ES	2.1:2.2	1	3.1	2.1E-16	0.156	0.071	8.0E-01	7.2E-01	3.5E-01
LGG	RPS25_ES_2.2:3.1_2.1_3.2	RPS25	ES	2.2:3.1	2.1	3.2	2.0E-15	0.137	0.042	5.2E-04	1.1E-03	8.1E-04
LGG	RPS25_ES_3.1:3.2_1_4	RPS25	ES	3.1:3.2	1	4	2.4E-13	0.152	-0.058	1.3E-01	2.9E-01	6.2E-01
LGG	RPS6KB2_ES_6.1:6.2_5.1_7	RPS6KB2	ES	6.1:6.2	5.1	7	7.6E-14	0.124	0.045	1.0E-01	1.9E-02	1.6E-01
LGG	RPS6KB2_ES_6.2_5.1_7	RPS6KB2	ES	6.2	5.1	7	3.8E-21	0.182	0.056	5.3E-03	2.4E-03	1.2E-03
LGG	RPS6KL1_ES_5_4_6	RPS6KL1	ES	5	4	6	1.1E-12	0.116	0.123	9.6E-01	8.7E-01	9.3E-01
LGG	RPS7_ES_1.2:2.1_1.1_2.2	RPS7	ES	1.2:2.1	1.1	2.2	1.1E-15	0.140	-0.147	1.5E-02	2.2E-03	2.7E-04
LGG	RPS9_ES_4.1:4.3_3_4.5	RPS9	ES	4.1:4.3	3	4.5	5.9E-15	0.133	0.053	3.7E-02	2.3E-01	2.8E-01
LGG	RRAGB_ES_4_3_5	RRAGB	ES	4	3	5	2.2E-44	0.341	0.484	5.1E-05	3.2E-07	4.3E-06
LGG	RTN4_ES_5:6.1:6.2_2.5_8	RTN4	ES	5:6.1:6.2	2.5	8	8.9E-48	0.361	0.401	7.9E-05	4.0E-07	4.7E-04
LGG	RTN4_ES_6.1:6.2_5_8	RTN4	ES	6.1:6.2	5	8	5.5E-53	0.391	0.259	1.5E-05	2.2E-06	9.8E-07
LGG	RWDD1_ES_2_1_3	RWDD1	ES	2	1	3	1.1E-15	0.139	-0.052	6.7E-04	2.0E-05	7.6E-05
LGG	RWDD1_ES_3_1_4	RWDD1	ES	3	1	4	5.9E-14	0.125	-0.020	8.8E-01	8.2E-01	6.1E-01
LGG	S100A6_ES_1.2:3.1_1.1_3.2	S100A6	ES	1.2:3.1	1.1	3.2	1.3E-23	0.228	0.557	5.8E-03	5.9E-05	2.3E-05
LGG	S100A6_ES_2_1.1_3.2	S100A6	ES	2	1.1	3.2	9.2E-18	0.177	0.435	8.5E-03	6.5E-03	1.9E-03
LGG	SAAL1_ES_10.2_9.1_11	SAAL1	ES	10.2	9.1	11	1.2E-11	0.115	-0.001	6.8E-02	3.2E-01	7.1E-01
LGG	SAMD4A_ES_4_3_5	SAMD4A	ES	4	3	5	3.1E-12	0.112	-0.114	4.1E-01	1.4E-01	1.4E-01
LGG	SAMD4B_ES_4_1_5	SAMD4B	ES	4	1	5	1.3E-12	0.117	-0.136	4.1E-01	2.7E-01	4.7E-01
LGG	SCARB1_ES_14_13.2_15	SCARB1	ES	14	13.2	15	5.4E-33	0.268	-0.493	3.0E-04	8.6E-06	6.6E-05
LGG	SCN1A_AD_11.3_11.2_12	SCN1A	AD	11.3	11.2	12	1.8E-16	0.185	0.018	6.6E-01	3.4E-01	8.5E-02

cancer type	id	Gene Symbol	splice_type	Exon	From.Exon	To.Exon	anova.p	adj.r2	r	p.50	p.25	p.10
LGG	SCN3A_ME_7|8_6_9	SCN3A	ME	7|8	6	9	3.7E-12	0.123	-0.164	3.2E-03	3.7E-03	1.3E-02
LGG	SCN3B_RI_5.2_5.1_5.3	SCN3B	RI	5.2	5.1	5.3	6.6E-14	0.143	-0.399	1.8E-02	1.7E-02	5.5E-02
LGG	SCP2_ES_12_11_13	SCP2	ES	12	11	13	5.1E-13	0.117	-0.411	2.9E-01	2.8E-01	9.5E-01
LGG	SCRIB_ES_36_35_37	SCRIB	ES	36	35	37	1.4E-35	0.285	-0.423	2.5E-04	4.3E-09	3.1E-05
LGG	SEC24B_ES_5_4_6	SEC24B	ES	5	4	6	9.5E-18	0.163	-0.029	1.4E-03	2.0E-05	3.9E-04
LGG	SEC24C_ES_2_1_3	SEC24C	ES	2	1	3	3.7E-34	0.284	0.366	2.5E-06	2.4E-05	4.3E-02
LGG	SEC24C_ES_8_7_9	SEC24C	ES	8	7	9	6.6E-12	0.110	0.088	2.9E-03	1.4E-04	1.4E-04
LGG	SEC31A_AD_26.2_26.1_28	SEC31A	AD	26.2	26.1	28	2.1E-34	0.278	0.198	1.2E-09	7.6E-14	1.3E-08
LGG	SEC31A_ES_16:17_15_18	SEC31A	ES	16:17	15	18	1.4E-20	0.181	-0.081	1.0E-04	1.0E-06	2.7E-07
LGG	SEH1L_AA_9.1_8_9.2	SEH1L	AA	9.1	8	9.2	4.8E-25	0.212	-0.122	1.2E-09	2.2E-08	7.5E-06
LGG	SEMA6A_ES_19_18_20.2	SEMA6A	ES	19	18	20.2	4.5E-20	0.177	0.329	8.4E-01	1.7E-02	8.6E-02
LGG	SEMA6D_AA_21.1_20_21.2	SEMA6D	AA	21.1	20	21.2	7.7E-12	0.117	-0.201	1.9E-01	1.9E-01	5.5E-02
LGG	SENP6_ES_8_6_9	SENP6	ES	8	6	9	1.9E-14	0.133	-0.194	2.1E-01	6.3E-01	2.1E-01
LGG	SEPT2_ES_6:7_2_9	SEPT2	ES	6:07	2	9	2.4E-12	0.132	0.052	2.3E-06	1.4E-05	5.0E-03
LGG	SEPT2_ES_6:7:9_2_10	SEPT2	ES	6:07:09	2	10	3.6E-09	0.108	0.012	7.0E-04	4.7E-05	6.7E-02
LGG	SEPT4_ES_7.1_3_8	SEPT4	ES	7.1	3	8	3.8E-35	0.287	0.323	2.7E-04	1.1E-06	4.7E-04
LGG	SEPT6_ES_12:13.1_11.1_13.2	SEPT6	ES	12:13.1	11.1	13.2	7.5E-17	0.157	-0.076	5.5E-01	4.0E-01	6.0E-04
LGG	SEPT8_AA_12.1_11_12.2	SEPT8	AA	12.1	11	12.2	1.1E-63	0.448	0.512	6.3E-05	2.1E-05	2.7E-02
LGG	SERHL2_AD_10.2_10.1_11	SERHL2	AD	10.2	10.1	11	3.5E-13	0.118	-0.569	2.9E-01	1.8E-01	1.2E-01
LGG	SERP2_ES_4_3_6	SERP2	ES	4	3	6	3.0E-52	0.387	-0.642	7.9E-06	6.5E-07	3.1E-05
LGG	SETD4_ES_4_3.4_5	SETD4	ES	4	3.4	5	3.6E-18	0.160	0.021	3.0E-03	9.6E-03	6.9E-03
LGG	SETD4_RI_13.2_13.1_13.3	SETD4	RI	13.2	13.1	13.3	6.3E-13	0.116	0.097	7.6E-02	2.8E-01	9.6E-01
LGG	SETD5_AA_8.1_6_8.2	SETD5	AA	8.1	6	8.2	2.2E-30	0.259	-0.252	4.2E-04	4.2E-04	2.2E-03
LGG	SETD5_ES_11_10_12	SETD5	ES	11	10	12	6.1E-16	0.146	-0.283	9.0E-01	8.2E-01	9.5E-01
LGG	SETD5_ES_7_6_8.1	SETD5	ES	7	6	8.1	1.3E-68	0.504	0.322	7.1E-05	8.2E-08	3.5E-07
LGG	SETD5_ES_7:8.1_6_8.2	SETD5	ES	07:08.1	6	8.2	5.7E-37	0.303	0.284	1.3E-05	1.9E-06	2.0E-06
LGG	SFI1_ES_13_12_14	SFI1	ES	13	12	14	3.1E-17	0.155	0.112	7.0E-04	1.1E-03	3.7E-03
LGG	SGCE_ES_3_1_4	SGCE	ES	3	1	4	1.8E-18	0.164	0.124	1.3E-02	4.3E-02	3.3E-02
LGG	SGCE_ES_3:4_1_5	SGCE	ES	3:04	1	5	9.5E-12	0.111	0.083	9.1E-02	6.7E-03	2.5E-01
LGG	SGIP1_ES_22_21_24	SGIP1	ES	22	21	24	5.0E-11	0.113	-0.092	8.6E-02	2.1E-02	2.8E-03
LGG	SGOL1_ES_6.1:6.2_5_7	SGOL1	ES	6.1:6.2	5	7	8.3E-10	0.104	-0.173	2.1E-08	2.2E-05	2.6E-02
LGG	SH2B1_AD_9.2_9.1_10	SH2B1	AD	9.2	9.1	10	1.1E-34	0.279	0.206	4.2E-04	2.7E-06	9.2E-05
LGG	SH3BP1_ES_16_15_17.2	SH3BP1	ES	16	15	17.2	9.6E-14	0.123	-0.579	7.5E-02	3.5E-01	3.0E-01
LGG	SH3GLB1_ES_6:7_5_8	SH3GLB1	ES	6:07	5	8	6.9E-13	0.116	-0.448	2.9E-04	9.7E-08	1.2E-05
LGG	SHF_ES_7_6_8.1	SHF	ES	7	6	8.1	4.3E-12	0.109	0.268	1.6E-04	3.6E-04	1.3E-02
LGG	SHF_ES_7:8.1_6_9.1	SHF	ES	07:08.1	6	9.1	2.5E-40	0.373	0.552	2.2E-04	1.2E-05	3.4E-06
LGG	SHF_ES_7:8.1:8.2_6_9.1	SHF	ES	7:8.1:8.2	6	9.1	9.7E-37	0.349	0.526	1.3E-04	9.4E-04	7.0E-04
LGG	SHF_ES_7:8.1:8.2:9.1_6_10	SHF	ES	7:8.1:8.2:9.1	6	10	8.6E-24	0.203	0.313	3.1E-03	3.6E-04	1.3E-02
LGG	SHF_ES_7:8.1:9.1_6_10	SHF	ES	7:8.1:9.1	6	10	1.1E-22	0.195	0.312	2.8E-02	3.3E-04	4.3E-04
LGG	SHF_ES_8.1_6_9.1	SHF	ES	8.1	6	9.1	1.7E-13	0.147	0.363	1.6E-02	7.6E-04	1.1E-03

cancer type	id	Gene Symbol	splice_type	Exon	From.Exon	To.Exon	anova.p	adj.r2	r	p.50	p.25	p.10
LGG	SHF_ES_8.1:8.2_6_9.1	SHF	ES	8.1:8.2	6	9.1	4.2E-16	0.172	0.400	2.5E-02	3.0E-02	2.8E-02
LGG	SHF_ES_8.1:8.2:9.1_6_10	SHF	ES	8.1:8.2:9.1	6	10	5.3E-14	0.125	0.125	1.2E-02	1.0E-01	5.8E-01
LGG	SIDT2_ES_11_10_12	SIDT2	ES	11	10	12	1.5E-27	0.233	0.156	2.4E-01	2.4E-03	2.2E-03
LGG	SIK3_ES_16_15_17	SIK3	ES	16	15	17	2.2E-31	0.259	-0.290	6.7E-06	1.3E-05	2.1E-04
LGG	SIMC1_ES_2:3_1_4	SIMC1	ES	2:03	1	4	1.5E-14	0.131	-0.579	1.7E-02	7.8E-04	2.3E-05
LGG	SIRT2_ES_2:3.2:4:6_1_7	SIRT2	ES	2:3.2:4:6	1	7	4.2E-18	0.176	-0.558	3.6E-02	7.4E-03	5.7E-02
LGG	SKA2_ES_4.1_2_5	SKA2	ES	4.1	2	5	1.3E-22	0.194	0.065	2.5E-02	1.3E-02	1.4E-01
LGG	SLAIN1_ES_6_4_7	SLAIN1	ES	6	4	7	6.4E-26	0.223	0.184	8.5E-07	2.5E-06	4.5E-05
LGG	SLC10A7_ES_13_12_14	SLC10A7	ES	13	12	14	9.1E-16	0.157	0.134	5.1E-01	8.0E-01	6.8E-02
LGG	SLC12A6_ES_4.2_1.7_5	SLC12A6	ES	4.2	1.7	5	4.3E-13	0.135	-0.195	5.0E-03	3.1E-03	5.0E-02
LGG	SLC25A14_ES_2.2:2.3_1_3	SLC25A14	ES	2.2:2.3	1	3	1.8E-23	0.203	0.142	9.6E-04	1.7E-04	4.5E-02
LGG	SLC25A16_ES_2.1:2.2_1_3	SLC25A16	ES	2.1:2.2	1	3	9.9E-13	0.125	0.298	4.4E-01	6.7E-01	5.8E-02
LGG	SLC25A40_ES_3_2_4	SLC25A40	ES	3	2	4	6.6E-11	0.106	-0.074	8.5E-01	8.0E-01	8.5E-01
LGG	SLC2A6_ES_8_7_9	SLC2A6	ES	8	7	9	9.1E-14	0.123	0.173	5.6E-02	9.6E-02	2.3E-01
LGG	SLC35F5_AD_1.2_1.1_2	SLC35F5	AD	1.2	1.1	2	6.4E-16	0.149	-0.135	3.4E-01	6.2E-01	8.0E-01
LGG	SLC37A4_ES_11_10_12	SLC37A4	ES	11	10	12	4.5E-84	0.542	-0.038	4.4E-08	2.2E-10	2.2E-08
LGG	SLC38A6_ES_4_3_5	SLC38A6	ES	4	3	5	2.3E-17	0.167	-0.317	9.5E-05	4.4E-05	3.1E-04
LGG	SLC39A13_RI_6.2_6.1_6.3	SLC39A13	RI	6.2	6.1	6.3	1.5E-13	0.122	0.356	6.7E-03	5.0E-02	6.6E-01
LGG	SLC44A2_AA_23.1_22_23.2	SLC44A2	AA	23.1	22	23.2	2.4E-94	0.583	-0.185	3.0E-07	1.3E-08	8.9E-05
LGG	SLC4A10_ES_28_27_29	SLC4A10	ES	28	27	29	1.2E-09	0.115	0.354	5.9E-01	9.4E-01	7.0E-01
LGG	SLC6A9_ES_5_3_6	SLC6A9	ES	5	3	6	7.6E-11	0.119	-0.268	3.6E-06	3.7E-06	1.1E-03
LGG	SLC8A1_ES_11:12_10_13.2	SLC8A1	ES	11:12	10	13.2	1.2E-14	0.162	-0.007	1.2E-03	8.8E-05	7.2E-04
LGG	SLC8A1_ES_12_10_13.2	SLC8A1	ES	12	10	13.2	7.0E-44	0.364	0.067	1.1E-05	1.6E-05	2.3E-05
LGG	SLC9A7_ES_13_12_14	SLC9A7	ES	13	12	14	1.3E-10	0.126	-0.090	1.1E-02	5.6E-05	9.7E-03
LGG	SLC9B2_ES_16_15_17.1	SLC9B2	ES	16	15	17.1	3.5E-36	0.289	-0.064	6.2E-05	8.3E-05	9.3E-04
LGG	SMAP1_ES_6_5.2_7	SMAP1	ES	6	5.2	7	3.4E-52	0.388	0.175	9.3E-05	3.1E-06	2.0E-05
LGG	SMARCA4_ES_32_31_33	SMARCA4	ES	32	31	33	3.0E-27	0.228	-0.311	2.3E-05	1.5E-05	9.6E-08
LGG	SMARCC2_AD_28.2:28.3_28.1_29	SMARCC2	AD	28.2:28.3	28.1	29	7.0E-15	0.133	-0.465	8.2E-05	6.6E-04	7.6E-02
LGG	SMARCC2_ES_18_17_19	SMARCC2	ES	18	17	19	9.6E-38	0.299	0.298	2.8E-04	3.8E-07	2.0E-03
LGG	SMARCE1_ES_4.1:5_3_6	SMARCE1	ES	4.1:5	3	6	1.3E-20	0.203	0.155	1.7E-04	2.2E-03	1.3E-03
LGG	SMEK2_ES_10_9_11	SMEK2	ES	10	9	11	4.8E-55	0.407	-0.082	5.0E-03	5.2E-04	2.6E-02
LGG	SMG7_ES_20_19_21	SMG7	ES	20	19	21	3.0E-41	0.324	0.109	3.2E-06	2.3E-07	3.4E-06
LGG	SMN2_ES_9.1_7_10	SMN2	ES	9.1	7	10	3.1E-19	0.181	-0.366	1.4E-01	3.9E-02	2.6E-02
LGG	SMPD4_ES_11_10_14	SMPD4	ES	11	10	14	5.6E-12	0.109	-0.096	2.1E-02	7.5E-03	4.0E-03
LGG	SMPDL3A_ES_4_3_5	SMPDL3A	ES	4	3	5	5.9E-10	0.117	0.365	8.6E-04	4.1E-03	1.1E-03
LGG	SNAP91_ES_17_16_19	SNAP91	ES	17	16	19	6.9E-13	0.130	0.391	2.1E-02	1.7E-02	9.3E-02
LGG	SNAP91_ES_22_21_23	SNAP91	ES	22	21	23	3.3E-17	0.172	0.679	7.4E-01	3.1E-01	2.6E-01
LGG	SNAP91_ES_36_35_37	SNAP91	ES	36	35	37	6.1E-11	0.115	0.625	4.0E-02	2.1E-01	5.1E-01
LGG	SNCAIP_ES_17_16.1_18	SNCAIP	ES	17	16.1	18	1.2E-15	0.140	0.306	7.5E-02	2.1E-03	2.2E-04
LGG	SNX11_ES_2_1.2_3.1	SNX11	ES	2	1.2	3.1	2.1E-13	0.121	0.025	3.4E-01	3.5E-01	4.1E-01

cancer type	id	Gene Symbol	splice_type	Exon	From.Exon	To.Exon	anova.p	adj.r2	r	p.50	p.25	p.10
LGG	SNX21_ES_3_2_4.1	SNX21	ES	3	2	4.1	8.1E-50	0.374	0.290	9.4E-04	7.9E-05	1.7E-02
LGG	SNX22_ES_2_1_3	SNX22	ES	2	1	3	1.0E-34	0.307	0.410	6.8E-04	2.0E-04	2.7E-03
LGG	SNX5_RI_1.2_1.1_1.3	SNX5	RI	1.2	1.1	1.3	2.3E-18	0.161	0.272	3.6E-01	9.4E-01	9.8E-01
LGG	SOCS4_ES_3_1_4	SOCS4	ES	3	1	4	1.3E-09	0.103	-0.039	7.0E-02	3.7E-03	7.4E-01
LGG	SON_ES_11_10_12	SON	ES	11	10	12	2.4E-24	0.206	0.095	7.6E-01	7.0E-02	1.6E-01
LGG	SORBS1_ES_14_13_17	SORBS1	ES	14	13	17	5.1E-14	0.129	0.373	1.3E-02	1.6E-03	1.7E-02
LGG	SORBS1_ES_15_14_17	SORBS1	ES	15	14	17	1.9E-15	0.143	-0.198	2.1E-01	1.5E-01	7.7E-01
LGG	SORBS1_ES_20_19_23	SORBS1	ES	20	19	23	1.8E-20	0.181	0.266	4.4E-04	1.6E-02	1.9E-03
LGG	SORBS1_ES_20:21_19_23	SORBS1	ES	20:21	19	23	1.5E-11	0.109	0.097	3.7E-03	4.4E-04	5.5E-02
LGG	SORBS1_ES_7_6_8	SORBS1	ES	7	6	8	3.7E-16	0.147	0.065	7.1E-01	5.3E-01	1.1E-01
LGG	SORBS1_ES_9.1:9.2_8_10	SORBS1	ES	9.1:9.2	8	10	9.2E-23	0.197	-0.279	3.7E-03	5.6E-05	2.1E-05
LGG	SP100_ES_3_1_5	SP100	ES	3	1	5	1.1E-18	0.171	-0.199	3.7E-02	2.7E-03	1.8E-03
LGG	SPAG8_RI_4.7_4.6_4.8	SPAG8	RI	4.7	4.6	4.8	1.6E-11	0.106	-0.119	5.7E-02	3.7E-02	3.8E-01
LGG	SPAST_ES_4_3_5	SPAST	ES	4	3	5	7.5E-13	0.121	-0.388	9.9E-02	3.7E-02	4.0E-01
LGG	SPATA7_ES_5_3_6.1	SPATA7	ES	5	3	6.1	4.1E-22	0.195	-0.236	5.9E-01	6.1E-02	1.4E-01
LGG	SPATS2_ES_2_1_5	SPATS2	ES	2	1	5	6.0E-13	0.121	0.125	8.0E-05	1.4E-03	1.4E-02
LGG	SPHK2_RI_3.2_3.1_3.3	SPHK2	RI	3.2	3.1	3.3	5.3E-16	0.143	-0.165	9.4E-03	5.6E-03	1.2E-01
LGG	SRCAP_ES_23_22_24	SRCAP	ES	23	22	24	1.0E-21	0.193	0.064	1.6E-06	2.5E-08	1.0E-06
LGG	SRGAP1_ME_12|13_11_14	SRGAP1	ME	12|13	11	14	7.8E-23	0.226	0.066	6.7E-05	1.6E-06	4.6E-05
LGG	SRRM1_ES_16_15_17	SRRM1	ES	16	15	17	4.2E-20	0.176	-0.129	1.9E-04	4.3E-08	3.1E-06
LGG	SRSF1_AD_3.2:3.3_3.1_3.5	SRSF1	AD	3.2:3.3	3.1	3.5	1.3E-15	0.155	0.183	4.6E-03	2.4E-02	1.7E-01
LGG	SRSF1_ES_3.3_3.1_3.5	SRSF1	ES	3.3	3.1	3.5	3.3E-14	0.139	-0.047	1.1E-01	2.8E-02	1.8E-02
LGG	SRSF11_ES_5:6.1:6.2:6.3_4.2_6.5	SRSF11	ES	5:6.1:6.2:6.3	4.2	6.5	7.5E-22	0.191	0.677	4.2E-01	1.8E-01	4.8E-01
LGG	SRSF11_RI_6.4_6.3_6.5	SRSF11	RI	6.4	6.3	6.5	2.1E-15	0.138	-0.196	2.8E-01	7.3E-02	7.8E-01
LGG	SRSF5_AD_2.2:2.3_2.1_4	SRSF5	AD	2.2:2.3	2.1	4	7.9E-21	0.180	-0.069	1.4E-02	5.7E-03	1.3E-01
LGG	SSBP3_ES_7_6_8	SSBP3	ES	7	6	8	2.2E-35	0.284	-0.102	1.2E-01	7.7E-03	7.0E-02
LGG	ST7_ES_4_3_5	ST7	ES	4	3	5	3.4E-61	0.440	-0.139	4.8E-05	3.1E-05	4.3E-04
LGG	ST8SIA1_ES_2_1_3	ST8SIA1	ES	2	1	3	8.6E-14	0.146	0.105	4.2E-01	6.8E-02	1.5E-01
LGG	STAG2_ES_35_34_36	STAG2	ES	35	34	36	3.6E-39	0.310	0.080	6.4E-04	3.4E-03	1.2E-03
LGG	STAG2_ES_4_1_5	STAG2	ES	4	1	5	1.1E-27	0.232	-0.050	2.4E-03	6.0E-05	1.5E-05
LGG	STAG2_ES_4_3.2_5	STAG2	ES	4	3.2	5	2.1E-13	0.154	-0.188	5.3E-02	9.2E-02	3.2E-05
LGG	STAG3_ES_10_4_11	STAG3	ES	10	4	11	9.1E-12	0.110	0.099	9.3E-04	1.4E-04	1.5E-02
LGG	STAU1_ES_2:3:4_1_5	STAU1	ES	2:03:04	1	5	3.6E-13	0.119	-0.376	5.9E-03	2.2E-02	3.0E-03
LGG	STAU1_ES_3:4_1_5	STAU1	ES	3:04	1	5	3.4E-13	0.122	-0.046	2.8E-03	2.7E-03	3.6E-03
LGG	STAU1_ES_4_3_5	STAU1	ES	4	3	5	6.1E-15	0.134	-0.228	3.5E-05	6.2E-03	3.8E-02
LGG	STAU2_ES_3:4:5_2_7	STAU2	ES	3:04:05	2	7	6.4E-13	0.120	-0.280	1.0E-01	4.4E-02	1.1E-01
LGG	STEAP3_ME_2|3_1.1_5.3	STEAP3	ME	2|3	1.1	5.3	1.6E-19	0.211	0.604	2.6E-07	7.5E-10	3.6E-08
LGG	STK25_ES_5:6_3.2_7	STK25	ES	5:06	3.2	7	4.5E-12	0.135	-0.279	3.2E-01	1.6E-01	2.3E-02
LGG	STRA13_ES_3.1_2_4.1	STRA13	ES	3.1	2	4.1	1.5E-11	0.105	-0.151	2.2E-01	8.8E-02	1.5E-02
LGG	STRA6_AD_18.2_18.1_19	STRA6	AD	18.2	18.1	19	1.2E-14	0.131	-0.720	5.6E-06	4.3E-05	6.1E-05

cancer type	id	Gene Symbol	splice_type	Exon	From.Exon	To.Exon	anova.p	adj.r2	r	p.50	p.25	p.10
LGG	STRADA_ES_2.2:3:4_2.1_6	STRADA	ES	2.2:3:4	2.1	6	1.5E-22	0.197	-0.052	4.2E-06	8.5E-05	1.4E-05
LGG	STRADA_ES_4_3_6	STRADA	ES	4	3	6	1.2E-11	0.107	-0.175	1.2E-02	6.7E-04	2.7E-04
LGG	STRN4_AA_10.1_9.2_10.2	STRN4	AA	10.1	9.2	10.2	1.2E-38	0.306	-0.260	8.8E-05	2.5E-06	4.4E-06
LGG	STX1B_RI_9.2_9.1_9.3	STX1B	RI	9.2	9.1	9.3	4.2E-32	0.268	-0.544	5.6E-04	9.6E-06	1.9E-04
LGG	SUCO_ES_13_12_14	SUCO	ES	13	12	14	1.3E-57	0.442	-0.253	2.3E-04	6.3E-05	1.1E-13
LGG	SUCO_ES_4_3_5	SUCO	ES	4	3	5	1.2E-10	0.103	-0.105	7.2E-01	1.8E-01	9.8E-01
LGG	SUGP2_ES_13_12.3_14	SUGP2	ES	13	12.3	14	4.5E-31	0.255	0.204	4.1E-06	7.5E-08	8.8E-07
LGG	SUGP2_RI_12.4_12.3_12.5	SUGP2	RI	12.4	12.3	12.5	1.2E-20	0.179	0.457	2.6E-03	8.1E-04	5.8E-02
LGG	SULF2_AD_1.2_1.1_2	SULF2	AD	1.2	1.1	2	3.1E-17	0.157	-0.394	1.5E-02	9.4E-02	8.4E-01
LGG	SUMF2_ES_3:4_2_6	SUMF2	ES	3:04	2	6	6.7E-19	0.165	0.258	9.5E-03	2.9E-03	8.8E-05
LGG	SUMF2_ES_4_3_6	SUMF2	ES	4	3	6	1.8E-33	0.272	0.321	1.0E-04	4.6E-04	7.7E-03
LGG	SUMF2_ES_4:5.1_3_6	SUMF2	ES	04:05.1	3	6	5.7E-09	0.102	0.239	2.8E-02	5.8E-03	1.4E-04
LGG	SUN1_ES_10:11_9.1_12	SUN1	ES	10:11	9.1	12	1.4E-23	0.205	0.249	1.1E-07	2.7E-09	1.3E-05
LGG	SUN1_ES_10:11:12_9.1_13	SUN1	ES	10:11:12	9.1	13	9.3E-12	0.108	0.232	8.1E-05	6.7E-05	4.1E-02
LGG	SUN1_ES_12_9.1_13	SUN1	ES	12	9.1	13	1.9E-22	0.202	-0.154	2.5E-05	1.3E-04	3.4E-04
LGG	SUPT20H_ES_26_25_27	SUPT20H	ES	26	25	27	2.6E-30	0.249	0.109	1.4E-02	5.1E-05	4.2E-07
LGG	SUPT20H_ES_4_3_5	SUPT20H	ES	4	3	5	7.7E-11	0.102	0.107	9.6E-02	3.8E-01	2.7E-01
LGG	SYN1_AA_13.1_12_13.2	SYN1	AA	13.1	12	13.2	7.4E-18	0.159	0.038	9.5E-06	2.4E-08	2.0E-08
LGG	SYNE1_ES_143_142_144	SYNE1	ES	143	142	144	3.1E-21	0.184	-0.335	1.3E-02	3.4E-04	6.4E-03
LGG	SYTL4_ES_3.1:4_2_5	SYTL4	ES	3.1:4	2	5	2.4E-23	0.209	-0.237	1.2E-03	5.3E-04	1.7E-03
LGG	SZRD1_ES_3_1_4.1	SZRD1	ES	3	1	4.1	3.9E-13	0.119	-0.406	6.7E-01	7.1E-01	8.2E-01
LGG	TACC2_ES_20_19_22	TACC2	ES	20	19	22	1.5E-14	0.130	0.143	5.0E-04	8.5E-05	1.0E-04
LGG	TACC2_ES_21_20_22	TACC2	ES	21	20	22	1.8E-10	0.102	-0.143	4.4E-02	2.0E-02	1.6E-02
LGG	TAF1D_AA_10.1_9_10.2	TAF1D	AA	10.1	9	10.2	1.6E-13	0.121	-0.236	9.3E-02	7.9E-03	7.5E-03
LGG	TAF1D_AA_8.1_7_8.2	TAF1D	AA	8.1	7	8.2	4.4E-18	0.159	-0.412	4.4E-02	1.4E-02	7.4E-04
LGG	TAF6_AD_2.2:2.3_2.1_3.2	TAF6	AD	2.2:2.3	2.1	3.2	2.9E-17	0.152	0.016	2.3E-01	2.6E-02	8.1E-02
LGG	TAF6_AD_2.3_2.2_3.2	TAF6	AD	2.3	2.2	3.2	1.5E-31	0.258	-0.071	1.9E-02	4.0E-02	1.7E-02
LGG	TAMM41_ES_5_4_7	TAMM41	ES	5	4	7	1.3E-15	0.140	0.152	2.1E-03	1.9E-03	3.3E-04
LGG	TANK_ES_2.2:3_2.1_4	TANK	ES	2.2:3	2.1	4	1.3E-25	0.218	-0.022	6.6E-02	1.0E-02	4.7E-03
LGG	TANK_ES_3_2.2_4	TANK	ES	3	2.2	4	4.1E-21	0.183	-0.045	3.6E-03	8.5E-05	3.7E-03
LGG	TAZ_ES_5_4_6	TAZ	ES	5	4	6	3.1E-15	0.136	0.028	2.8E-03	2.5E-02	1.8E-02
LGG	TAZ_ES_7_6_8.1	TAZ	ES	7	6	8.1	6.8E-29	0.239	0.017	2.3E-01	2.3E-01	5.4E-01
LGG	TBC1D5_ES_22_21_23	TBC1D5	ES	22	21	23	4.7E-14	0.133	-0.030	8.5E-02	5.4E-04	6.9E-07
LGG	TBC1D9B_ES_20_19_21	TBC1D9B	ES	20	19	21	7.5E-60	0.429	0.010	3.9E-04	1.8E-06	5.7E-05
LGG	TBL2_AA_4.1:4.2_1_4.3	TBL2	AA	4.1:4.2	1	4.3	2.1E-14	0.130	-0.358	4.8E-01	6.6E-01	1.1E-01
LGG	TBRG4_ES_4:5_3_6	TBRG4	ES	4:05	3	6	2.7E-18	0.160	-0.160	6.8E-02	4.8E-03	1.5E-02
LGG	TBXAS1_ES_8_7.1_9	TBXAS1	ES	8	7.1	9	1.7E-16	0.146	-0.576	1.2E-02	1.8E-02	3.9E-01
LGG	TCAIM_AA_7.1_6_7.2	TCAIM	AA	7.1	6	7.2	3.0E-13	0.123	-0.047	9.3E-04	3.4E-04	2.1E-02
LGG	TCEB1_AD_1.2_1.1_6	TCEB1	AD	1.2	1.1	6	1.1E-20	0.179	0.148	3.3E-05	3.7E-08	2.3E-05
LGG	TCEB1_AD_1.2:1.3_1.1_6	TCEB1	AD	1.2:1.3	1.1	6	1.1E-20	0.180	0.118	1.1E-01	4.6E-04	2.9E-02

cancer type	id	Gene Symbol	splice_type	Exon	From.Exon	To.Exon	anova.p	adj.r2	r	p.50	p.25	p.10
LGG	TCEB1_ES_1.2:5_1.1_6	TCEB1	ES	1.2:5	1.1	6	1.7E-35	0.285	0.064	6.5E-06	5.5E-07	2.1E-07
LGG	TCERG1_ES_6_5_7	TCERG1	ES	6	5	7	7.1E-15	0.138	0.163	1.2E-01	2.4E-03	2.0E-03
LGG	TCF12_ES_18_17_19	TCF12	ES	18	17	19	5.2E-67	0.468	0.431	5.9E-07	1.0E-10	8.2E-09
LGG	TCF20_AA_4.1_3_4.2	TCF20	AA	4.1	3	4.2	4.0E-40	0.315	0.317	1.3E-03	5.2E-04	5.3E-06
LGG	TCF20_ES_4.1:4.2_3_5	TCF20	ES	4.1:4.2	3	5	3.6E-47	0.357	0.366	1.1E-08	3.1E-09	4.1E-06
LGG	TCTN1_ES_1.2:4_1.1_5	TCTN1	ES	1.2:4	1.1	5	1.0E-09	0.101	-0.144	5.7E-02	2.3E-01	2.5E-01
LGG	TCTN1_ES_13:14:16.2_12_17.2	TCTN1	ES	14:16.2	12	17.2	1.7E-15	0.170	0.243	2.8E-03	3.2E-05	3.7E-04
LGG	TCTN1_ES_9_8_11	TCTN1	ES	9	8	11	2.8E-10	0.117	0.308	2.4E-02	1.8E-03	4.3E-04
LGG	TECPR1_AD_2.2_2.1_3	TECPR1	AD	2.2	2.1	3	1.9E-13	0.127	-0.017	1.1E-02	1.3E-02	2.5E-03
LGG	TEX9_AD_1.4_1.3_2	TEX9	AD	1.4	1.3	2	1.8E-18	0.168	-0.008	5.1E-04	3.4E-03	1.5E-01
LGG	TEX9_RI_1.2_1.1_1.3	TEX9	RI	1.2	1.1	1.3	4.3E-10	0.116	0.035	1.8E-02	1.2E-02	1.9E-02
LGG	TFDP1_ES_13.1:13.2_12_14	TFDP1	ES	13.1:13.2	12	14	1.5E-18	0.184	0.103	9.0E-04	4.8E-04	8.2E-04
LGG	TFDP1_ES_13.1:13.2:13.3_12_14	TFDP1	ES	13.1:13.2:13.3	12	14	2.3E-11	0.112	0.095	1.7E-04	1.1E-04	1.5E-01
LGG	TFDP1_ES_13.2_12_14	TFDP1	ES	13.2	12	14	2.3E-38	0.323	0.193	5.3E-05	1.3E-07	8.2E-07
LGG	THBS3_AA_17.1_16_17.2	THBS3	AA	17.1	16	17.2	5.8E-17	0.150	-0.414	1.7E-04	2.0E-04	5.3E-04
LGG	THBS3_ES_4:5_3_6	THBS3	ES	4:05	3	6	1.2E-13	0.128	0.297	7.2E-04	3.3E-05	2.7E-04
LGG	THUMPD1_RI_5.2_5.1_5.3	THUMPD1	RI	5.2	5.1	5.3	6.7E-19	0.166	0.162	5.5E-04	3.5E-03	1.8E-01
LGG	THUMPD3_AD_1.2_1.1_2	THUMPD3	AD	1.2	1.1	2	1.8E-11	0.105	-0.003	4.0E-01	1.2E-01	4.0E-01
LGG	THYN1_AD_1.2_1.1_1.4	THYN1	AD	1.2	1.1	1.4	1.6E-24	0.208	0.124	2.2E-03	8.9E-03	3.4E-02
LGG	THYN1_RI_1.3_1.2_1.4	THYN1	RI	1.3	1.2	1.4	3.9E-11	0.101	-0.171	5.6E-01	3.6E-02	3.6E-03
LGG	TIA1_ES_5:6_4_7	TIA1	ES	5:06	4	7	5.6E-24	0.206	0.326	3.8E-07	4.6E-06	9.1E-05
LGG	TIA1_ES_6_5_7	TIA1	ES	6	5	7	3.6E-32	0.265	0.359	8.8E-06	1.4E-05	9.7E-04
LGG	TIA1_ES_8_7_9.1	TIA1	ES	8	7	9.1	1.2E-22	0.195	0.328	4.3E-06	3.3E-09	2.9E-04
LGG	TIAM2_ES_2_1_3	TIAM2	ES	2	1	3	3.3E-15	0.177	-0.132	3.8E-01	1.8E-01	7.0E-02
LGG	TIMMDC1_ES_1.2:2_1.1_3	TIMMDC1	ES	1.2:2	1.1	3	5.0E-11	0.101	-0.076	3.1E-04	1.9E-03	2.2E-03
LGG	TIMMDC1_ES_1.2:2:3:4_1.1_5	TIMMDC1	ES	1.2:2:3:4	1.1	5	3.0E-15	0.136	-0.118	2.7E-03	7.4E-04	4.6E-05
LGG	TIMMDC1_ES_2:3:4_1.1_6	TIMMDC1	ES	2:03:04	1.1	6	3.0E-09	0.103	-0.099	1.4E-03	6.7E-04	3.3E-05
LGG	TJAP1_ES_10_9_11	TJAP1	ES	10	9	11	5.6E-19	0.166	0.244	1.5E-05	3.6E-06	5.4E-05
LGG	TJP2_ES_23_22.1_24	TJP2	ES	23	22.1	24	6.1E-47	0.357	-0.494	5.3E-08	6.1E-08	2.7E-08
LGG	TJP2_ES_23:24_22.1_25	TJP2	ES	23:24	22.1	25	2.9E-19	0.168	-0.198	1.1E-03	6.9E-06	4.2E-06
LGG	TLR4_ES_2_1_3	TLR4	ES	2	1	3	8.5E-10	0.104	0.075	3.9E-01	2.3E-01	8.2E-01
LGG	TM2D1_ES_2.3_1.3_3	TM2D1	ES	2.3	1.3	3	3.7E-19	0.205	0.197	9.8E-05	4.9E-06	2.8E-03
LGG	TM2D2_AA_2.1:2.2_1.2_2.3	TM2D2	AA	2.1:2.2	1.2	2.3	3.2E-13	0.119	-0.209	2.3E-01	1.4E-01	9.1E-02
LGG	TMEM107_ES_2:3.2:3.3:3.4:3.5_1_3.7	TMEM107	ES	2:3.2:3.3:3.4:3.5	1	3.7	1.7E-12	0.113	-0.334	4.0E-01	9.1E-01	3.3E-01
LGG	TMEM107_ES_2:3.2:3.4:3.5_1_3.7	TMEM107	ES	2:3.2:3.4:3.5	1	3.7	1.5E-12	0.113	-0.030	1.5E-01	7.3E-02	5.0E-02
LGG	TMEM134_ES_6.1:6.2_4.1_7	TMEM134	ES	6.1:6.2	4.1	7	5.1E-24	0.204	0.121	1.2E-05	6.1E-08	2.0E-08
LGG	TMEM138_RI_3.3_3.2_3.4	TMEM138	RI	3.3	3.2	3.4	1.1E-14	0.131	-0.095	1.1E-02	4.3E-05	2.0E-03
LGG	TMEM175_ES_2:3:4.1:4.2_1_5.1	TMEM175	ES	2:3:4.1:4.2	1	5.1	7.2E-26	0.218	0.568	8.0E-01	8.4E-02	6.6E-02
LGG	TMEM175_ES_4.1:4.2_3_5.1	TMEM175	ES	4.1:4.2	3	5.1	1.5E-38	0.304	0.675	9.6E-03	2.6E-04	3.5E-02
LGG	TMEM178A_ES_3_2_4	TMEM178A	ES	3	2	4	1.0E-35	0.297	0.108	5.2E-03	5.6E-08	5.5E-08

cancer type	id	Gene Symbol	splice_type	Exon	From.Exon	To.Exon	anova.p	adj.r2	r	p.50	p.25	p.10
LGG	TMEM205_AA_2.5_2.2_2.6	TMEM205	AA	2.5	2.2	2.6	3.6E-14	0.127	-0.126	3.7E-03	8.5E-03	9.4E-02
LGG	TMEM205_RI_2.3:2.4:2.5_2.2_2.6	TMEM205	RI	2.3:2.4:2.5	2.2	2.6	3.8E-14	0.127	-0.320	4.3E-03	4.2E-03	2.3E-03
LGG	TMEM25_ES_5_4_6	TMEM25	ES	5	4	6	1.5E-38	0.304	-0.039	5.7E-05	4.3E-06	2.8E-09
LGG	TMEM255A_ES_7_5_8	TMEM255A	ES	7	5	8	2.2E-29	0.258	-0.346	3.7E-04	2.7E-05	6.0E-04
LGG	TMEM255A_ES_7:8:9_5_10	TMEM255A	ES	7:08:09	5	10	1.7E-16	0.153	-0.312	9.4E-03	1.0E-03	6.7E-02
LGG	TMEM5_ES_3_2_4	TMEM5	ES	3	2	4	5.0E-30	0.248	-0.088	5.5E-06	1.1E-04	5.7E-04
LGG	TMEM62_ES_5:6_4_7	TMEM62	ES	5:06	4	7	9.2E-10	0.106	0.263	3.8E-02	4.4E-02	1.3E-01
LGG	TMEM63B_ES_5_4_6	TMEM63B	ES	5	4	6	1.3E-12	0.122	-0.324	1.0E-01	2.0E-03	1.8E-03
LGG	TMEM66_ES_1.2:2.2_1.1_2.3	TMEM66	ES	1.2:2.2	1.1	2.3	2.6E-13	0.121	0.028	2.2E-06	1.1E-05	3.8E-03
LGG	TMEM66_ES_2.1:2.2:2.4_1.2_3	TMEM66	ES	2.1:2.2:2.4	1.2	3	3.1E-20	0.176	0.145	8.7E-02	4.5E-03	1.8E-02
LGG	TMEM66_ES_2.2:2.3:2.4_1.2_3	TMEM66	ES	2.2:2.3:2.4	1.2	3	1.6E-16	0.147	0.069	4.3E-01	5.0E-01	2.9E-01
LGG	TMEM91_RI_7.2:7.3_7.1_7.4	TMEM91	RI	7.2:7.3	7.1	7.4	5.0E-13	0.117	-0.588	4.8E-01	8.2E-01	5.2E-01
LGG	TMEM91_RI_7.2:7.3:7.4_7.1_7.5	TMEM91	RI	7.2:7.3:7.4	7.1	7.5	1.0E-14	0.131	-0.509	7.4E-01	3.3E-01	9.6E-01
LGG	TMPO_ES_6:7:8_5.1_9	TMPO	ES	6:07:08	5.1	9	3.8E-16	0.144	0.165	9.3E-03	2.3E-03	3.4E-04
LGG	TMPRSS5_ES_2.1:2.2:3_1_4.1	TMPRSS5	ES	2.1:2.2:3	1	4.1	5.0E-11	0.106	-0.007	5.3E-02	3.1E-03	1.0E-01
LGG	TMUB2_ES_2.2:2.3:2.4:2.5:4.2_2.1_4.3	TMUB2	ES	2.2:2.3:2.4:2.5:4.2	2.1	4.3	3.4E-13	0.120	0.006	5.0E-02	8.3E-02	2.6E-02
LGG	TMUB2_ES_2.3:2.4:2.5:4.2_2.2_4.3	TMUB2	ES	2.3:2.4:2.5:4.2	2.2	4.3	8.6E-18	0.158	0.069	8.3E-04	5.6E-03	5.1E-04
LGG	TMUB2_ES_2.5:3_1_4.3	TMUB2	ES	2.5:3	1	4.3	1.4E-16	0.178	-0.043	3.7E-01	2.5E-01	4.3E-01
LGG	TMUB2_ES_4.7:4.8_4.5_5	TMUB2	ES	4.7:4.8	4.5	5	1.5E-11	0.115	-0.141	6.6E-01	6.4E-01	5.2E-01
LGG	TMUB2_ES_4.8_4.5_5	TMUB2	ES	4.8	4.5	5	2.5E-15	0.152	-0.167	7.6E-01	1.1E-01	5.9E-02
LGG	TMX2_ES_3.1:3.2:3.3_2_4	TMX2	ES	3.1:3.2:3.3	2	4	2.7E-18	0.161	-0.047	8.0E-03	1.6E-03	9.4E-03
LGG	TMX2_ES_3.2:3.3_2_4	TMX2	ES	3.2:3.3	2	4	5.0E-58	0.419	0.071	2.0E-06	3.4E-08	1.4E-06
LGG	TMX2_ES_4:5.1:5.3_2_6	TMX2	ES	4:5.1:5.3	2	6	2.1E-16	0.145	-0.195	1.6E-02	1.4E-02	2.4E-01
LGG	TNC_ES_12:13:14:15:16:18:19_11_20	TNC	ES	:13:14:15:16:18:	11	20	2.1E-12	0.113	0.000	3.6E-01	7.6E-01	8.6E-01
LGG	TNFRSF11B_ES_3_2_4	TNFRSF11B	ES	3	2	4	3.8E-35	0.286	-0.791	1.9E-08	9.1E-11	9.8E-12
LGG	TNK2_ES_13:14:15.2:16:17_12_18	TNK2	ES	13:14:15.2:16:17	12	18	7.0E-34	0.275	0.439	9.0E-06	5.1E-08	4.0E-07
LGG	TNK2_ES_18_17_19	TNK2	ES	18	17	19	2.0E-24	0.207	0.359	4.3E-04	3.8E-06	9.0E-05
LGG	TPCN2_ES_17_16_18	TPCN2	ES	17	16	18	1.4E-18	0.173	-0.048	2.5E-03	8.7E-05	1.3E-02
LGG	TPD52L1_ES_8_6_10	TPD52L1	ES	8	6	10	4.1E-12	0.112	0.118	3.5E-01	1.2E-01	3.8E-01
LGG	TPD52L1_ES_8:9.1_6_10	TPD52L1	ES	08:09.1	6	10	8.2E-22	0.190	0.132	7.9E-01	2.8E-01	6.4E-01
LGG	TPD52L2_ES_4_3_5	TPD52L2	ES	4	3	5	3.5E-59	0.425	0.344	1.6E-05	7.4E-08	1.2E-06
LGG	TPM1_ES_12.1_11.1_13.1	TPM1	ES	12.1	11.1	13.1	1.4E-19	0.176	0.144	1.2E-01	6.3E-05	4.5E-04
LGG	TPM1_ES_9_8_10	TPM1	ES	9	8	10	2.6E-19	0.168	0.079	9.2E-06	1.5E-06	4.3E-03
LGG	TPM2_AA_11.1_9_11.2	TPM2	AA	11.1	9	11.2	7.7E-13	0.116	0.017	3.2E-02	1.4E-04	2.7E-02
LGG	TRA2A_ES_2_1_3.1	TRA2A	ES	2	1	3.1	2.1E-20	0.181	-0.031	2.4E-01	2.8E-02	7.1E-01
LGG	TRA2A_ES_2:3.1:3.2_1_4	TRA2A	ES	2:3.1:3.2	1	4	1.2E-12	0.114	0.401	6.6E-01	8.0E-01	1.6E-01
LGG	TRAF3IP2_ES_2_1_3	TRAF3IP2	ES	2	1	3	4.3E-51	0.381	-0.275	8.4E-08	2.2E-11	1.9E-08
LGG	TRAPPC6A_AD_1.2_1.1_2	TRAPPC6A	AD	1.2	1.1	2	8.3E-24	0.203	0.193	2.2E-03	4.1E-03	3.1E-01
LGG	TRAPPC6A_AD_1.2_1.1_3	TRAPPC6A	AD	1.2	1.1	3	3.0E-21	0.184	0.122	1.3E-02	2.0E-02	1.3E-02
LGG	TRAPPC6A_ES_1.2:2_1.1_3	TRAPPC6A	ES	1.2:2	1.1	3	4.0E-19	0.167	0.145	8.0E-02	7.0E-02	3.0E-01

cancer type	id	Gene Symbol	splice_type	Exon	From.Exon	To.Exon	anova.p	adj.r2	r	p.50	p.25	p.10
LGG	TRAPPC9_ES_6_5_7	TRAPPC9	ES	6	5	7	1.6E-16	0.149	0.237	2.3E-03	2.4E-03	1.9E-03
LGG	TRERF1_AD_8.2_8.1_9	TRERF1	AD	8.2	8.1	9	6.0E-10	0.111	0.266	7.0E-01	9.3E-02	5.4E-01
LGG	TRIM16_ES_2_1.3_3	TRIM16	ES	2	1.3	3	2.1E-27	0.233	-0.246	3.0E-03	5.7E-05	3.5E-02
LGG	TRIM16_ES_4_3_6.2	TRIM16	ES	4	3	6.2	5.4E-11	0.114	0.001	7.0E-02	6.6E-03	7.5E-02
LGG	TRIM16_ES_5_4_6.2	TRIM16	ES	5	4	6.2	3.9E-18	0.183	0.137	1.6E-05	3.5E-05	1.2E-02
LGG	TRIM16L_ES_2_1.3_3	TRIM16L	ES	2	1.3	3	3.5E-42	0.329	-0.297	1.2E-03	1.1E-05	6.2E-04
LGG	TRIM33_ES_12_11_13	TRIM33	ES	12	11	13	7.7E-10	0.108	-0.262	5.4E-01	3.1E-01	7.4E-01
LGG	TRIM33_ES_20_19_21	TRIM33	ES	20	19	21	6.4E-16	0.141	0.226	2.2E-01	5.5E-01	3.4E-01
LGG	TRIM9_ES_6.2:7.1_5_11	TRIM9	ES	6.2:7.1	5	11	1.8E-11	0.124	-0.031	3.1E-02	1.4E-02	1.8E-02
LGG	TRIP10_ES_11.1:11.2_10_12.2	TRIP10	ES	11.1:11.2	10	12.2	6.1E-21	0.182	-0.327	2.1E-04	3.0E-03	7.5E-04
LGG	TRMT10B_ES_3.2_2_4.1	TRMT10B	ES	3.2	2	4.1	1.2E-23	0.203	-0.161	1.0E-02	6.5E-04	1.6E-02
LGG	TRMU_AA_6.1_5_6.2	TRMU	AA	6.1	5	6.2	6.5E-23	0.196	-0.159	1.4E-01	2.6E-01	3.4E-01
LGG	TRMU_ES_4_3_5	TRMU	ES	4	3	5	2.7E-14	0.128	-0.129	1.6E-04	2.2E-04	2.2E-03
LGG	TRO_ES_12.3_12.1_13	TRO	ES	12.3	12.1	13	2.8E-13	0.120	0.104	7.2E-01	3.1E-01	3.8E-01
LGG	TRO_RI_12.2_12.1_12.3	TRO	RI	12.2	12.1	12.3	7.5E-14	0.124	0.018	3.0E-02	1.1E-01	1.8E-01
LGG	TRPT1_ES_2.2_1_3	TRPT1	ES	2.2	1	3	6.2E-15	0.133	-0.083	4.8E-01	7.7E-01	2.6E-01
LGG	TSC2_ES_33_32_34	TSC2	ES	33	32	34	1.1E-35	0.286	0.101	7.2E-03	2.5E-04	6.9E-02
LGG	TSEN15_ES_5.1:5.2_3_6	TSEN15	ES	5.1:5.2	3	6	5.0E-39	0.308	0.088	3.1E-02	1.9E-03	1.2E-02
LGG	TSFM_ES_10_8_11	TSFM	ES	10	8	11	1.4E-21	0.193	-0.015	1.0E-01	8.2E-03	1.4E-03
LGG	TSPAN5_ES_4:5_3_6	TSPAN5	ES	4:05	3	6	1.4E-12	0.131	0.026	3.9E-02	7.4E-02	5.3E-01
LGG	TTC14_RI_5.2:5.3_5.1_5.4	TTC14	RI	5.2:5.3	5.1	5.4	5.8E-15	0.133	0.549	3.0E-01	3.3E-01	8.2E-01
LGG	TTC3_ES_3.2:4:5:6:7:8:9:10:11_1_13	TTC3	ES	2:4:5:6:7:8:9:10::	1	13	7.8E-16	0.155	-0.343	6.1E-05	3.0E-04	1.0E-03
LGG	TTLL11_ES_3_2_4.1	TTLL11	ES	3	2	4.1	2.6E-12	0.116	0.093	7.4E-05	8.2E-05	3.2E-02
LGG	TXNL4A_ES_7.2:8_3_9	TXNL4A	ES	7.2:8	3	9	9.0E-17	0.159	-0.103	3.3E-01	4.9E-03	5.3E-05
LGG	UAP1_ES_9.1:9.2_8_10	UAP1	ES	9.1:9.2	8	10	6.5E-16	0.141	0.111	1.4E-01	6.3E-04	2.9E-02
LGG	UAP1_ES_9.2_8_10	UAP1	ES	9.2	8	10	8.3E-14	0.124	0.166	4.5E-02	1.9E-02	4.6E-03
LGG	UBE2D4_ES_4:5.1:5.2_3_7.1	UBE2D4	ES	4:5.1:5.2	3	7.1	5.7E-13	0.137	-0.199	2.8E-02	1.8E-01	2.0E-01
LGG	UBL7_AD_1.2_1.1_3	UBL7	AD	1.2	1.1	3	1.1E-15	0.139	-0.221	7.9E-03	1.7E-02	1.2E-01
LGG	UBXN11_ES_3:4_2_5	UBXN11	ES	3:04	2	5	4.5E-27	0.243	0.096	1.3E-05	2.6E-06	1.2E-04
LGG	UBXN11_ES_3:4:5_2_7	UBXN11	ES	3:04:05	2	7	7.8E-18	0.189	-0.015	3.5E-02	5.5E-03	1.5E-05
LGG	UBXN11_ES_3:4:5:6:7:8:9_2_10	UBXN11	ES	3:4:5:6:7:8:9	2	10	1.7E-32	0.324	0.001	1.8E-04	1.2E-06	3.0E-07
LGG	UBXN11_ES_3:4:5:7:8:9_2_10	UBXN11	ES	3:4:5:7:8:9	2	10	2.5E-28	0.258	-0.018	1.9E-04	6.7E-06	3.7E-04
LGG	UBXN11_ES_4_2_5	UBXN11	ES	4	2	5	2.4E-25	0.217	0.126	2.1E-06	4.4E-07	1.3E-05
LGG	UBXN11_ES_4:5_2_7	UBXN11	ES	4:05	2	7	5.0E-20	0.186	0.092	5.5E-05	9.0E-07	5.7E-05
LGG	UBXN11_ES_4:5:6_2_7	UBXN11	ES	4:05:06	2	7	2.2E-37	0.326	0.070	2.0E-06	7.0E-07	4.0E-05
LGG	UBXN11_ES_4:5:6:7:8:9_2_10	UBXN11	ES	4:5:6:7:8:9	2	10	7.5E-45	0.359	0.028	4.4E-05	2.5E-06	1.4E-04
LGG	UBXN11_ES_4:5:6:7:8:9:10_2_11	UBXN11	ES	4:5:6:7:8:9:10	2	11	5.0E-38	0.379	0.190	3.0E-04	5.8E-05	2.9E-05
LGG	UBXN11_ES_4:5:7:8:9_2_10	UBXN11	ES	4:5:7:8:9	2	10	9.4E-30	0.249	0.011	1.4E-04	3.1E-05	2.8E-04
LGG	UBXN11_ES_4:5:7:8:9:10_2_11	UBXN11	ES	4:5:7:8:9:10	2	11	2.1E-17	0.197	0.165	9.6E-04	1.2E-03	3.2E-03
LGG	UBXN11_ES_5_2_7	UBXN11	ES	5	2	7	1.5E-10	0.103	-0.079	4.9E-02	1.1E-02	1.6E-02

cancer type	id	Gene Symbol	splice_type	Exon	From.Exon	To.Exon	anova.p	adj.r2	r	p.50	p.25	p.10
LGG	UBXN11_ES_5:6_2_7	UBXN11	ES	5:06	2	7	9.1E-13	0.125	-0.072	4.6E-01	5.8E-01	8.0E-01
LGG	UBXN11_ES_5:6:7:8:9_2_10	UBXN11	ES	5:6:7:8:9	2	10	1.1E-24	0.213	-0.112	4.8E-01	2.8E-01	5.2E-01
LGG	UBXN11_ES_5:6:7:8:9:10_2_11	UBXN11	ES	5:6:7:8:9:10	2	11	1.6E-15	0.176	0.058	4.2E-03	9.1E-02	5.0E-01
LGG	UBXN11_ES_5:7:8:9_2_10	UBXN11	ES	5:7:8:9	2	10	1.4E-14	0.131	-0.119	2.9E-01	3.2E-01	1.8E-01
LGG	UBXN11_ES_6_5_7	UBXN11	ES	6	5	7	1.1E-15	0.141	-0.014	1.1E-03	4.7E-04	2.1E-03
LGG	UIMC1_ES_6.1_5_8	UIMC1	ES	6.1	5	8	7.4E-14	0.131	-0.011	6.4E-01	1.3E-01	4.4E-02
LGG	UIMC1_ES_6.1:7.1:7.2_5_8	UIMC1	ES	6.1:7.1:7.2	5	8	1.9E-15	0.137	0.159	3.0E-01	6.3E-01	6.4E-01
LGG	UNK_ES_6_2_7	UNK	ES	6	2	7	2.2E-21	0.193	0.244	1.5E-02	1.2E-01	2.2E-01
LGG	UPF3A_ES_4_3_5	UPF3A	ES	4	3	5	6.4E-16	0.141	-0.180	1.1E-03	2.3E-06	6.3E-04
LGG	UPF3B_ES_8_7_9	UPF3B	ES	8	7	9	1.4E-31	0.259	0.027	1.3E-02	4.2E-03	1.4E-03
LGG	UPP1_ES_5:6.1:6.2:7_4_9	UPP1	ES	5:6.1:6.2:7	4	9	2.1E-28	0.236	0.359	1.3E-04	2.7E-07	2.3E-08
LGG	UQCRQ_AD_1.2:1.3:1.4_1.1_2	UQCRQ	AD	1.2:1.3:1.4	1.1	2	3.2E-41	0.328	-0.295	5.3E-07	3.8E-10	3.0E-05
LGG	UQCRQ_ES_1.2:1.4_1.1_2	UQCRQ	ES	1.2:1.4	1.1	2	4.9E-22	0.194	-0.188	1.5E-04	7.5E-08	9.0E-07
LGG	UQCRQ_RI_1.3_1.2_1.4	UQCRQ	RI	1.3	1.2	1.4	7.8E-13	0.116	-0.254	1.0E-01	6.5E-02	3.2E-02
LGG	URGCP_ES_4.2:4.3_3_4.6	URGCP	ES	4.2:4.3	3	4.6	2.1E-13	0.122	-0.127	4.5E-02	1.7E-01	3.9E-01
LGG	URGCP_ES_4.3_3_4.6	URGCP	ES	4.3	3	4.6	6.2E-10	0.104	-0.183	3.8E-01	7.3E-03	1.4E-01
LGG	USP16_ES_2_1_3.1	USP16	ES	2	1	3.1	3.2E-25	0.221	0.001	8.9E-06	2.7E-06	1.7E-04
LGG	USP21_ES_2_1_3	USP21	ES	2	1	3	3.3E-27	0.228	0.019	7.3E-05	1.6E-06	1.0E-06
LGG	USP28_ES_21_20_22	USP28	ES	21	20	22	4.4E-28	0.244	-0.366	1.7E-07	7.3E-09	2.0E-07
LGG	USP5_AD_15.2_15.1_16	USP5	AD	15.2	15.1	16	6.2E-24	0.203	0.201	3.0E-03	1.5E-05	1.8E-05
LGG	VAV2_ES_28_27_29	VAV2	ES	28	27	29	2.4E-54	0.399	-0.207	1.6E-08	4.2E-07	2.2E-07
LGG	VDAC3_ES_5.2_4.1_6	VDAC3	ES	5.2	4.1	6	1.2E-14	0.131	0.524	7.7E-03	6.8E-02	1.3E-01
LGG	VEZT_ES_5_4_6.1	VEZT	ES	5	4	6.1	2.1E-24	0.214	-0.220	7.9E-02	1.1E-01	7.5E-02
LGG	VKORC1_ES_4.1:4.2_2_6	VKORC1	ES	4.1:4.2	2	6	5.5E-15	0.134	-0.001	4.6E-03	4.8E-04	1.9E-02
LGG	VLDLR_ES_16_15_17	VLDLR	ES	16	15	17	1.5E-11	0.107	-0.118	7.0E-02	1.5E-01	3.2E-01
LGG	VPS29_ES_3.1_1_5	VPS29	ES	3.1	1	5	5.9E-19	0.165	0.070	4.3E-03	5.8E-03	9.5E-03
LGG	VRK3_AD_1.2_1.1_2.2	VRK3	AD	1.2	1.1	2.2	1.8E-11	0.105	-0.216	1.0E-01	4.1E-02	1.8E-01
LGG	VSTM1_ME_3|4_2_5	VSTM1	ME	3|4	2	5	1.2E-55	0.407	0.867	1.7E-07	2.1E-10	4.4E-12
LGG	VTI1B_ES_2:3_1_4	VTI1B	ES	2:03	1	4	1.5E-39	0.311	-0.210	4.3E-03	3.4E-06	3.6E-06
LGG	VWA5A_ES_1.3_1.1_2.2	VWA5A	ES	1.3	1.1	2.2	8.9E-13	0.118	0.033	1.5E-01	1.5E-01	6.8E-03
LGG	VWA9_ES_1.2:1.3:2.1_1.1_2.2	VWA9	ES	1.2:1.3:2.1	1.1	2.2	1.9E-12	0.123	-0.084	9.3E-01	2.1E-01	3.6E-02
LGG	VWA9_ES_1.2:2.1:2.2:2.3_1.1_3.1	VWA9	ES	1.2:2.1:2.2:2.3	1.1	3.1	5.4E-20	0.179	0.024	6.4E-02	1.7E-03	5.3E-02
LGG	VWA9_ES_1.2:2.2:2.3_1.1_3.1	VWA9	ES	1.2:2.2:2.3	1.1	3.1	2.1E-10	0.119	-0.074	2.4E-02	9.1E-03	2.7E-03
LGG	VWA9_ES_2.2:2.3_1.1_3.1	VWA9	ES	2.2:2.3	1.1	3.1	2.8E-21	0.215	0.012	4.0E-03	3.5E-03	2.4E-01
LGG	WASF1_ES_2:3_1.2_4	WASF1	ES	2:03	1.2	4	5.7E-11	0.105	-0.444	2.4E-01	1.4E-01	1.8E-01
LGG	WDR20_ES_3.1:3.2:4:7.1:7.2_1.2_9	WDR20	ES	3.1:3.2:4:7.1:7.2	1.2	9	2.9E-09	0.106	0.059	4.3E-01	7.7E-01	4.2E-01
LGG	WDR26_AD_3.2_3.1_4.2	WDR26	AD	3.2	3.1	4.2	3.0E-30	0.255	0.107	3.3E-01	9.8E-03	6.6E-02
LGG	WDR62_RI_25.6_25.5_25.7	WDR62	RI	25.6	25.5	25.7	6.1E-12	0.108	-0.555	3.9E-04	6.2E-04	1.4E-03
LGG	WHSC1_RI_13.2_13.1_13.3	WHSC1	RI	13.2	13.1	13.3	4.6E-20	0.174	-0.085	1.0E-01	3.0E-01	1.1E-01
LGG	WHSC1L1_AD_21.2_21.1_22	WHSC1L1	AD	21.2	21.1	22	7.6E-13	0.123	-0.225	7.5E-05	8.9E-05	1.2E-02

cancer type	id	Gene Symbol	splice_type	Exon	From.Exon	To.Exon	anova.p	adj.r2	r	p.50	p.25	p.10
LGG	WIBG_ES_4_2_6	WIBG	ES	4	2	6	1.1E-23	0.202	0.037	2.1E-04	3.1E-04	1.9E-01
LGG	WNK1_ES_12_9_13.1	WNK1	ES	12	9	13.1	1.4E-11	0.110	0.084	8.9E-02	3.1E-03	1.7E-03
LGG	WNK1_ES_12:13.1_9_13.2	WNK1	ES	12:13.1	9	13.2	2.9E-11	0.109	0.101	7.7E-03	2.5E-03	2.7E-02
LGG	WSB1_RI_5.2_5.1_5.3	WSB1	RI	5.2	5.1	5.3	6.7E-19	0.166	-0.422	3.1E-04	9.9E-05	1.4E-03
LGG	XRCC4_AD_1.2_1.1_2	XRCC4	AD	1.2	1.1	2	1.5E-16	0.159	-0.129	1.1E-02	5.1E-03	9.0E-06
LGG	YAF2_ES_6:7_5.2_9.1	YAF2	ES	6:07	5.2	9.1	8.7E-15	0.139	0.162	5.3E-01	9.5E-02	3.2E-02
LGG	YIPF1_ES_12_11_13	YIPF1	ES	12	11	13	5.0E-14	0.126	-0.174	4.4E-01	9.1E-01	6.2E-01
LGG	ZC3HAV1_AD_4.2_4.1_5	ZC3HAV1	AD	4.2	4.1	5	2.4E-10	0.101	0.030	2.6E-01	2.1E-01	1.1E-01
LGG	ZCWPW1_ES_16:17_15_18	ZCWPW1	ES	16:17	15	18	2.1E-21	0.185	0.163	2.6E-01	1.8E-01	2.6E-01
LGG	ZDHHC20_ES_14_13.2_15	ZDHHC20	ES	14	13.2	15	1.3E-14	0.139	-0.201	6.2E-01	4.8E-01	2.5E-01
LGG	ZFYVE20_ES_2_1_3	ZFYVE20	ES	2	1	3	1.5E-15	0.143	-0.319	3.8E-01	2.0E-02	3.7E-02
LGG	ZFYVE21_ES_6.1_5_7	ZFYVE21	ES	6.1	5	7	4.6E-12	0.109	0.172	2.5E-03	9.7E-04	7.6E-05
LGG	ZMAT3_ES_2_1_3	ZMAT3	ES	2	1	3	2.2E-09	0.106	-0.105	6.9E-02	1.0E-02	1.8E-03
LGG	ZMIZ2_ES_9_8_10	ZMIZ2	ES	9	8	10	2.3E-20	0.177	-0.191	9.7E-01	3.9E-01	8.2E-01
LGG	ZMYM3_AD_15.2_15.1_16	ZMYM3	AD	15.2	15.1	16	4.6E-15	0.137	0.068	4.9E-01	6.1E-05	7.6E-05
LGG	ZMYND8_ES_26_25_27	ZMYND8	ES	26	25	27	4.4E-31	0.255	-0.041	3.1E-02	4.0E-03	2.8E-02
LGG	ZNF131_RI_7.2:7.3_7.1_7.4	ZNF131	RI	7.2:7.3	7.1	7.4	2.8E-33	0.272	-0.163	3.2E-02	1.0E-02	9.4E-03
LGG	ZNF195_ES_11_5.1_13	ZNF195	ES	11	5.1	13	4.9E-14	0.127	-0.050	3.6E-01	4.2E-01	1.8E-02
LGG	ZNF226_AA_7.3_7.1_7.4	ZNF226	AA	7.3	7.1	7.4	5.6E-15	0.133	0.299	2.3E-01	2.5E-01	5.3E-02
LGG	ZNF226_RI_7.2_7.1_7.3	ZNF226	RI	7.2	7.1	7.3	7.2E-12	0.107	0.064	8.3E-01	7.8E-01	7.3E-02
LGG	ZNF227_ES_3_2.2_4.1	ZNF227	ES	3	2.2	4.1	5.9E-16	0.142	-0.032	9.9E-01	7.1E-01	9.0E-01
LGG	ZNF23_AD_1.2_1.1_2.2	ZNF23	AD	1.2	1.1	2.2	7.8E-15	0.133	0.033	8.4E-03	7.6E-02	7.8E-01
LGG	ZNF248_ES_3_2_4	ZNF248	ES	3	2	4	2.4E-32	0.280	-0.140	2.4E-02	1.6E-03	3.5E-02
LGG	ZNF248_RI_7.2_7.1_7.3	ZNF248	RI	7.2	7.1	7.3	1.6E-28	0.237	0.457	4.1E-03	1.6E-02	3.2E-03
LGG	ZNF267_ES_4_3_5	ZNF267	ES	4	3	5	3.3E-11	0.113	-0.007	8.2E-02	1.4E-01	9.6E-02
LGG	ZNF276_AD_7.2_7.1_8	ZNF276	AD	7.2	7.1	8	1.6E-16	0.146	-0.376	8.6E-04	7.8E-03	9.7E-02
LGG	ZNF415_ES_3_1.1_4	ZNF415	ES	3	1.1	4	3.9E-12	0.112	-0.145	1.4E-04	6.1E-04	4.2E-03
LGG	ZNF544_AA_7.1_6.2_7.2	ZNF544	AA	7.1	6.2	7.2	2.2E-11	0.103	-0.117	4.0E-02	5.7E-02	9.6E-02
LGG	ZNF559_ES_3.1:3.2:3.3_2.2_4	ZNF559	ES	3.1:3.2:3.3	2.2	4	4.5E-17	0.155	-0.183	1.4E-02	6.2E-03	1.0E-02
LGG	ZNF655_ES_4:5.3_3.2_7	ZNF655	ES	04:05.3	3.2	7	3.6E-52	0.392	0.215	1.0E-05	2.2E-07	4.5E-05
LGG	ZNF655_ES_4:5.3:6.1:6.2_3.2_7	ZNF655	ES	4:5.3:6.1:6.2	3.2	7	1.4E-19	0.174	0.187	2.3E-01	8.4E-01	9.6E-02
LGG	ZNF655_ES_6.1:6.2_5.3_7	ZNF655	ES	6.1:6.2	5.3	7	1.0E-15	0.154	-0.031	4.2E-04	6.8E-04	1.5E-04
LGG	ZNF670_ES_7_6_8	ZNF670	ES	7	6	8	2.5E-11	0.108	-0.226	3.6E-01	7.9E-02	7.1E-01
LGG	ZNF691_ES_2.1_1_4	ZNF691	ES	2.1	1	4	2.1E-12	0.126	0.219	1.0E-03	5.2E-04	1.7E-05
LGG	ZNF691_ES_2.3_2.1_4	ZNF691	ES	2.3	2.1	4	1.1E-11	0.139	-0.214	2.5E-04	2.2E-04	1.6E-03
LGG	ZNF692_RI_6.3_6.2_6.4	ZNF692	RI	6.3	6.2	6.4	5.6E-34	0.274	0.218	2.2E-06	3.3E-06	3.1E-08
LGG	ZNF706_ES_3.4_3.2_4	ZNF706	ES	3.4	3.2	4	3.4E-11	0.103	0.007	2.0E-01	1.3E-02	2.3E-02
LGG	ZNF711_ES_8_7_9	ZNF711	ES	8	7	9	1.4E-43	0.342	0.227	8.1E-04	5.5E-06	6.3E-03
LGG	ZNF720_RI_5.2:5.3_5.1_5.4	ZNF720	RI	5.2:5.3	5.1	5.4	1.4E-13	0.122	0.372	7.4E-02	5.0E-02	1.5E-01
LGG	ZNF821_RI_3.2_3.1_3.3	ZNF821	RI	3.2	3.1	3.3	5.0E-11	0.114	-0.463	9.5E-03	6.2E-03	3.9E-02

cancer type	id	Gene Symbol	splice_type	Exon	From.Exon	To.Exon	anova.p	adj.r2	r	p.50	p.25	p.10
LGG	ZWINT_RI_2.2_2.1_2.3	ZWINT	RI	2.2	2.1	2.3	6.9E-10	0.102	0.106	3.1E-01	2.6E-01	3.1E-02
LIHC	ABCG5_AA_4.1_3_4.2	ABCG5	AA	4.1	3	4.2	9.6E-07	0.154	-0.698	2.8E-02	3.4E-01	6.7E-01
LIHC	ACSM2B_AA_2.1_1_2.2	ACSM2B	AA	2.1	1	2.2	7.0E-07	0.143	-0.490	6.5E-01	9.6E-01	6.3E-01
LIHC	ACSM2B_ES_2.1:2.2_1_3	ACSM2B	ES	2.1:2.2	1	3	1.6E-05	0.112	-0.699	4.9E-03	1.0E-02	1.6E-01
LIHC	AKAP8L_RI_4.2:4.3_4.1_4.4	AKAP8L	RI	4.2:4.3	4.1	4.4	1.7E-06	0.135	-0.032	1.2E-01	1.4E-01	1.2E-02
LIHC	ANO1_ES_17_16_18	ANO1	ES	17	16	18	5.1E-07	0.186	-0.703	3.2E-01	1.9E-01	1.4E-01
LIHC	AP1G2_RI_1.2:1.3_1.1_1.4	AP1G2	RI	1.2:1.3	1.1	1.4	7.2E-06	0.121	-0.458	6.4E-01	7.3E-01	8.4E-01
LIHC	AP1G2_RI_1.3_1.2_1.4	AP1G2	RI	1.3	1.2	1.4	8.8E-06	0.120	-0.424	2.4E-01	1.3E-01	2.4E-01
LIHC	APBB3_AD_6.3:6.4:6.5:6.6_6.2_6.9	APBB3	AD	6.3:6.4:6.5:6.6	6.2	6.9	2.6E-05	0.115	-0.298	4.4E-03	2.7E-01	2.0E-01
LIHC	BABAM1_AA_2.1_1.2_2.2	BABAM1	AA	2.1	1.2	2.2	2.5E-06	0.131	0.048	4.1E-02	4.1E-03	7.3E-02
LIHC	BAHD1_AA_7.1_6_7.2	BAHD1	AA	7.1	6	7.2	1.1E-07	0.176	-0.062	7.7E-01	6.9E-01	5.2E-01
LIHC	BCAT2_ES_3_1_4	BCAT2	ES	3	1	4	7.6E-07	0.141	-0.584	7.6E-02	2.4E-01	2.3E-02
LIHC	BCAT2_ES_3:4:5_1_6	BCAT2	ES	3:04:05	1	6	1.4E-09	0.199	-0.359	5.1E-03	1.5E-01	1.6E-01
LIHC	BRD8_ES_22_21_23	BRD8	ES	22	21	23	2.2E-07	0.153	0.073	7.3E-01	4.3E-01	6.1E-02
LIHC	BTN3A1_AA_10.2_9_10.3	BTN3A1	AA	10.2	9	10.3	3.2E-05	0.125	-0.049	9.1E-01	2.1E-01	7.6E-02
LIHC	C16orf13_ES_4_3_5	C16orf13	ES	4	3	5	8.7E-07	0.140	-0.155	2.7E-01	4.1E-02	4.1E-01
LIHC	CCL16_ES_2:3_1_4	CCL16	ES	2:03	1	4	1.9E-10	0.216	-0.907	2.0E-03	5.0E-02	1.3E-01
LIHC	CD46_ES_7:8_6_9	CD46	ES	7:08	6	9	8.5E-05	0.102	-0.162	1.7E-01	7.4E-02	3.5E-01
LIHC	CHERP_AA_6.1_5_6.2	CHERP	AA	6.1	5	6.2	4.4E-05	0.116	-0.061	7.3E-01	5.4E-01	9.4E-01
LIHC	DHRS1_RI_6.3_6.2_6.4	DHRS1	RI	6.3	6.2	6.4	6.9E-06	0.120	-0.612	1.3E-02	2.7E-02	8.9E-01
LIHC	ELMO1_AD_1.2_1.1_3	ELMO1	AD	1.2	1.1	3	3.7E-05	0.126	-0.199	8.7E-01	5.9E-01	3.9E-01
LIHC	ERLIN1_RI_1.2_1.1_1.3	ERLIN1	RI	1.2	1.1	1.3	3.4E-05	0.132	0.498	3.8E-02	1.4E-01	8.6E-01
LIHC	FAM73B_AD_15.3_15.2_16	FAM73B	AD	15.3	15.2	16	1.8E-05	0.115	-0.067	9.9E-01	7.3E-01	5.0E-01
LIHC	FBXO44_ES_3.1:3.2_2_4	FBXO44	ES	3.1:3.2	2	4	5.0E-05	0.106	-0.194	2.3E-01	3.1E-01	7.6E-01
LIHC	FDPS_AD_1.2_1.1_3.2	FDPS	AD	1.2	1.1	3.2	5.0E-07	0.147	0.047	5.8E-01	8.6E-01	4.5E-01
LIHC	FIP1L1_ES_2_1_3	FIP1L1	ES	2	1	3	8.5E-05	0.103	-0.261	6.5E-03	1.5E-02	8.6E-03
LIHC	FN1_ES_25_24_26	FN1	ES	25	24	26	8.9E-08	0.162	-0.044	2.2E-01	2.7E-01	1.2E-01
LIHC	FN1_ES_33_32_34	FN1	ES	33	32	34	1.8E-07	0.155	-0.171	1.4E-02	5.2E-03	2.8E-01
LIHC	GMPPA_AA_2.1_1.1_2.2	GMPPA	AA	2.1	1.1	2.2	1.8E-10	0.221	-0.196	1.9E-01	2.2E-01	5.3E-01
LIHC	GMPPA_AA_2.1_1.3_2.2	GMPPA	AA	2.1	1.3	2.2	5.3E-05	0.116	-0.165	1.2E-01	3.8E-02	4.2E-01
LIHC	GUSB_ES_6_5.1_7	GUSB	ES	6	5.1	7	9.4E-06	0.118	0.116	9.8E-02	5.6E-02	4.2E-01
LIHC	HHLA3_ES_4_3_5	HHLA3	ES	4	3	5	3.2E-05	0.106	0.113	1.1E-01	1.9E-01	1.1E-01
LIHC	HM13_ES_11:12.1:12.2_10_13	HM13	ES	11:12.1:12.2	10	13	1.3E-05	0.117	-0.185	5.6E-01	6.4E-01	5.4E-05
LIHC	HNRNPA1_ES_3:4:5:6.1:6.2:7.1:7.2:8:9.1	HNRNPA1	ES	:7.1:7.2:8:9.1:9.2	2	11.3	1.3E-05	0.116	0.179	4.7E-01	4.7E-01	9.4E-01
LIHC	HNRNPA1_ES_6.2:7.1:7.2:8:9.1_6.1_9.2	HNRNPA1	ES	6.2:7.1:7.2:8:9.1	6.1	9.2	5.0E-05	0.100	0.108	1.7E-01	9.5E-02	6.1E-01
LIHC	IL17RC_ES_4_3.3_5.1	IL17RC	ES	4	3.3	5.1	2.1E-06	0.133	-0.243	5.0E-01	8.7E-01	1.1E-01
LIHC	IL17RE_AA_15.1:15.2_14_15.3	IL17RE	AA	15.1:15.2	14	15.3	1.9E-04	0.105	-0.527	8.9E-01	8.0E-01	7.6E-01
LIHC	INO80C_ES_3:4.1:4.2_1_5.1	INO80C	ES	3:4.1:4.2	1	5.1	2.9E-05	0.106	-0.007	7.7E-02	1.7E-01	1.4E-01
LIHC	INVS_RI_16.2_16.1_16.3	INVS	RI	16.2	16.1	16.3	4.9E-05	0.101	-0.033	1.6E-01	6.3E-01	3.4E-01
LIHC	ITGB4_ES_35_34_36	ITGB4	ES	35	34	36	7.3E-06	0.131	-0.200	1.2E-01	1.4E-03	3.1E-03

cancer type	id	Gene Symbol	splice_type	Exon	From.Exon	To.Exon	anova.p	adj.r2	r	p.50	p.25	p.10
LIHC	KHK_ME_3|4_2_5	KHK	ME	3|4	2	5	1.7E-10	0.217	-0.663	1.4E-02	1.3E-02	7.3E-02
LIHC	KIAA1217_ES_12_11_13	KIAA1217	ES	12	11	13	1.6E-04	0.107	-0.046	3.3E-01	4.9E-01	6.8E-01
LIHC	KIF22_AD_1.2:1.3_1.1_2.2	KIF22	AD	1.2:1.3	1.1	2.2	3.2E-05	0.122	0.234	5.6E-01	1.3E-01	1.7E-01
LIHC	LARP1B_RI_9.2_9.1_9.3	LARP1B	RI	9.2	9.1	9.3	5.1E-08	0.173	-0.016	8.4E-01	8.3E-01	4.0E-01
LIHC	MACF1_ES_103_102_104	MACF1	ES	103	102	104	6.1E-07	0.148	0.007	3.6E-01	3.8E-01	7.9E-01
LIHC	MED11_AA_3.1_2.1_3.2	MED11	AA	3.1	2.1	3.2	5.0E-05	0.100	0.249	3.1E-01	4.9E-01	8.6E-01
LIHC	MTSS1L_ES_7_6_8	MTSS1L	ES	7	6	8	4.2E-05	0.115	0.008	1.7E-02	1.1E-01	1.3E-01
LIHC	NFE2L1_ES_6_5.2_7	NFE2L1	ES	6	5.2	7	3.5E-09	0.196	0.381	1.5E-01	1.2E-01	9.3E-01
LIHC	NIPA2_ES_1.2:3_1.1_4	NIPA2	ES	1.2:3	1.1	4	6.6E-05	0.122	-0.295	5.1E-01	8.6E-01	7.9E-01
LIHC	NIPA2_ES_2_1.1_4	NIPA2	ES	2	1.1	4	6.3E-05	0.116	-0.189	9.8E-01	2.6E-01	2.0E-01
LIHC	OGG1_RI_6.2_6.1_6.3	OGG1	RI	6.2	6.1	6.3	1.3E-05	0.113	-0.045	8.7E-01	4.9E-01	9.2E-01
LIHC	PALM_ES_8_7_9	PALM	ES	8	7	9	4.4E-12	0.276	-0.506	7.5E-02	1.8E-01	7.1E-01
LIHC	PAPSS2_ES_9_8_10	PAPSS2	ES	9	8	10	7.0E-05	0.115	0.338	3.2E-03	1.4E-03	3.0E-03
LIHC	PCSK6_ES_17_16_18	PCSK6	ES	17	16	18	3.3E-06	0.132	0.399	8.5E-02	1.7E-02	3.7E-02
LIHC	PI4KB_ES_5_4_6	PI4KB	ES	5	4	6	2.0E-06	0.141	-0.183	4.1E-01	3.3E-02	3.6E-02
LIHC	PLA2G10_ES_4_3_5	PLA2G10	ES	4	3	5	1.9E-05	0.127	-0.777	4.5E-01	4.5E-01	1.2E-01
LIHC	PLEKHM2_ES_7_6_8	PLEKHM2	ES	7	6	8	2.2E-06	0.141	-0.078	1.0E-01	5.9E-02	1.2E-01
LIHC	POFUT2_RI_8.4_8.3_8.5	POFUT2	RI	8.4	8.3	8.5	3.7E-05	0.124	0.189	3.1E-01	2.6E-01	8.0E-01
LIHC	POLB_ES_2_1_3	POLB	ES	2	1	3	6.7E-05	0.101	0.052	8.3E-02	7.9E-02	1.3E-01
LIHC	PRKCDBP_ES_2_1_3	PRKCDBP	ES	2	1	3	1.1E-08	0.197	0.229	5.1E-01	4.0E-01	1.7E-02
LIHC	PTGR1_AA_12.1_11_12.2	PTGR1	AA	12.1	11	12.2	1.2E-05	0.115	-0.790	5.3E-01	2.9E-01	2.2E-01
LIHC	PTK2B_ES_28_27_29	PTK2B	ES	28	27	29	7.8E-05	0.101	-0.093	8.7E-01	5.8E-01	7.9E-01
LIHC	RABGGTB_ES_5:6_4.1_7	RABGGTB	ES	5:06	4.1	7	2.1E-05	0.109	-0.205	3.1E-02	3.6E-01	8.2E-01
LIHC	RAI14_ES_18_17_19	RAI14	ES	18	17	19	4.7E-06	0.145	0.151	5.7E-01	1.8E-01	1.3E-01
LIHC	RNF41_ES_4.1:4.2_3_5	RNF41	ES	4.1:4.2	3	5	2.2E-04	0.100	-0.040	8.8E-01	4.2E-01	6.8E-01
LIHC	RPS7_ES_1.2:2.1_1.1_2.2	RPS7	ES	1.2:2.1	1.1	2.2	6.8E-07	0.146	-0.069	5.0E-01	2.0E-01	6.7E-01
LIHC	S100A16_AD_2.2_2.1_4	S100A16	AD	2.2	2.1	4	3.3E-05	0.104	-0.344	3.7E-01	8.6E-01	8.8E-01
LIHC	SAMD4A_ES_13_12_14	SAMD4A	ES	13	12	14	1.0E-06	0.143	0.342	2.7E-01	3.2E-01	7.9E-01
LIHC	SCP2_ES_12_11_13	SCP2	ES	12	11	13	3.8E-06	0.126	-0.858	5.4E-03	9.7E-03	1.3E-01
LIHC	SEC24D_ES_27_26_28	SEC24D	ES	27	26	28	4.8E-05	0.100	-0.625	2.9E-01	2.0E-01	6.5E-01
LIHC	SEC31A_ES_26.1:27_25.1_28	SEC31A	ES	26.1:27	25.1	28	2.3E-08	0.208	0.055	1.1E-01	3.2E-01	2.3E-01
LIHC	SEC31A_ES_27_26.2_28	SEC31A	ES	27	26.2	28	8.4E-06	0.120	0.038	7.4E-01	2.5E-01	2.4E-02
LIHC	SERHL2_AD_10.2_10.1_11	SERHL2	AD	10.2	10.1	11	2.2E-05	0.108	-0.795	5.6E-01	5.6E-01	2.2E-01
LIHC	SIDT2_ES_14_13_15.1	SIDT2	ES	14	13	15.1	3.9E-05	0.114	0.291	2.5E-01	1.6E-01	3.2E-02
LIHC	SLC7A2_ME_9|10_8_11	SLC7A2	ME	9|10	8	11	1.6E-06	0.166	-0.394	6.5E-01	2.6E-01	1.5E-02
LIHC	SMARCD3_RI_11.2_11.1_11.3	SMARCD3	RI	11.2	11.1	11.3	6.9E-10	0.207	-0.529	5.4E-01	1.8E-01	1.5E-01
LIHC	SPTAN1_ES_23_22_24	SPTAN1	ES	23	22	24	1.3E-04	0.106	0.068	1.8E-01	1.6E-01	1.3E-01
LIHC	SREBF1_ES_2_1_3.2	SREBF1	ES	2	1	3.2	4.7E-06	0.133	0.368	5.0E-02	6.0E-03	3.6E-01
LIHC	STRA6_AD_18.2_18.1_19	STRA6	AD	18.2	18.1	19	6.4E-05	0.102	-0.750	9.1E-02	5.1E-01	6.6E-01
LIHC	SYBU_ES_7.3:7.4_6.2_8	SYBU	ES	7.3:7.4	6.2	8	1.9E-04	0.109	-0.169	9.1E-01	4.5E-01	6.4E-01

cancer type	id	Gene Symbol	splice_type	Exon	From.Exon	To.Exon	anova.p	adj.r2	r	p.50	p.25	p.10
LIHC	TMEM159_ES_5:6_4_7	TMEM159	ES	5:06	4	7	4.4E-05	0.120	-0.427	5.5E-01	3.0E-01	7.6E-01
LIHC	TNFRSF25_RI_6.3:6.4_6.2_6.5	TNFRSF25	RI	6.3:6.4	6.2	6.5	8.9E-05	0.104	-0.419	4.7E-01	9.0E-01	3.9E-01
LIHC	TPD52L2_ES_4_3_5	TPD52L2	ES	4	3	5	3.9E-05	0.103	-0.265	1.3E-01	7.5E-01	8.6E-01
LIHC	TSC2_AA_28.1_26_28.2	TSC2	AA	28.1	26	28.2	1.7E-05	0.118	0.098	3.4E-04	1.7E-02	2.4E-01
LIHC	WIBG_ES_4_2_6	WIBG	ES	4	2	6	2.7E-05	0.108	-0.133	1.2E-01	1.2E-01	8.1E-01
LUAD	ABHD17A_ES_3:4.1_2.2_4.2	ABHD17A	ES	03:04.1	2.2	4.2	4.3E-08	0.179	0.103	1.6E-01	1.4E-01	5.9E-01
LUAD	ABI1_ES_11.1:11.2:12_9_13	ABI1	ES	11.1:11.2:12	9	13	1.0E-05	0.109	-0.235	1.8E-01	7.0E-01	6.9E-01
LUAD	ABI1_ES_11.2:12_9_13	ABI1	ES	11.2:12	9	13	7.8E-09	0.169	-0.157	4.0E-01	3.1E-01	6.3E-01
LUAD	ABI1_ES_12_11.2_13	ABI1	ES	12	11.2	13	5.2E-07	0.135	-0.030	2.8E-02	2.1E-02	1.6E-02
LUAD	ABI1_ES_5_4_7	ABI1	ES	5	4	7	7.9E-09	0.169	-0.084	1.3E-02	2.5E-03	9.6E-02
LUAD	ACADVL_RI_3.2_3.1_3.3	ACADVL	RI	3.2	3.1	3.3	1.8E-05	0.121	-0.046	4.0E-01	8.8E-01	4.1E-01
LUAD	ACHE_AA_6.1_5_6.2	ACHE	AA	6.1	5	6.2	2.7E-05	0.106	0.001	4.3E-01	8.0E-01	5.9E-01
LUAD	AKAP13_ES_12_11_14	AKAP13	ES	12	11	14	5.0E-06	0.142	0.087	6.6E-01	2.1E-01	3.9E-01
LUAD	ALDOC_ES_2_1_3	ALDOC	ES	2	1	3	1.2E-04	0.101	-0.210	8.3E-01	8.3E-01	3.7E-02
LUAD	ALG2_ES_2_1_3	ALG2	ES	2	1	3	7.4E-06	0.112	-0.393	1.1E-02	1.7E-01	8.3E-01
LUAD	ALG8_ES_14_13_15	ALG8	ES	14	13	15	8.7E-06	0.110	-0.130	5.9E-01	5.1E-01	7.4E-01
LUAD	AMPD2_ES_2.2_1_4	AMPD2	ES	2.2	1	4	1.4E-04	0.109	0.071	5.9E-01	1.4E-01	6.4E-01
LUAD	ANKRD11_ES_9_7_10	ANKRD11	ES	9	7	10	1.8E-10	0.201	0.078	4.7E-02	3.9E-01	5.2E-01
LUAD	ANKRD65_RI_1.2_1.1_1.3	ANKRD65	RI	1.2	1.1	1.3	3.2E-09	0.202	0.004	2.9E-01	7.1E-02	8.9E-01
LUAD	AP1B1_ES_24_23_25	AP1B1	ES	24	23	25	3.4E-11	0.212	0.093	2.5E-01	5.9E-01	7.7E-01
LUAD	AP2B1_AD_1.2_1.1_2	AP2B1	AD	1.2	1.1	2	1.1E-07	0.151	0.237	3.7E-03	3.6E-03	9.6E-05
LUAD	AP2M1_ES_7_6_8	AP2M1	ES	7	6	8	3.3E-06	0.119	-0.248	9.1E-01	8.7E-01	6.2E-01
LUAD	AP4B1_RI_1.2_1.1_1.3	AP4B1	RI	1.2	1.1	1.3	2.8E-04	0.107	0.182	1.5E-01	1.0E-01	9.2E-02
LUAD	APH1A_ES_3_1_4	APH1A	ES	3	1	4	9.4E-07	0.161	0.138	1.5E-01	5.1E-03	1.6E-01
LUAD	ARHGEF10L_ES_18_17_19	ARHGEF10L	ES	18	17	19	6.1E-07	0.145	0.161	9.1E-01	1.2E-01	4.0E-01
LUAD	ARHGEF11_ES_39_38_40	ARHGEF11	ES	39	38	40	3.6E-10	0.194	-0.256	2.7E-01	6.5E-01	2.2E-01
LUAD	ARHGEF12_ES_5_4_6	ARHGEF12	ES	5	4	6	4.0E-05	0.106	-0.029	5.2E-01	5.7E-01	8.2E-01
LUAD	ARMC6_AD_1.2_1.1_5	ARMC6	AD	1.2	1.1	5	6.6E-06	0.121	0.243	1.1E-02	4.1E-02	9.4E-01
LUAD	ARPP19_RI_2.3:2.4_2.2_2.5	ARPP19	RI	2.3:2.4	2.2	2.5	1.4E-07	0.145	0.142	3.7E-01	1.9E-01	9.3E-01
LUAD	ARSA_RI_1.3:1.4_1.2_1.5	ARSA	RI	1.3:1.4	1.2	1.5	1.9E-06	0.127	-0.073	3.3E-02	2.2E-01	7.0E-01
LUAD	ASAP2_ES_23_22_24	ASAP2	ES	23	22	24	6.2E-08	0.190	-0.044	8.6E-02	8.7E-03	6.6E-03
LUAD	ATL2_AA_17.1_15_17.2	ATL2	AA	17.1	15	17.2	1.4E-05	0.106	0.107	1.8E-01	1.1E-01	4.6E-01
LUAD	ATP11A_ES_29_28_30.3	ATP11A	ES	29	28	30.3	2.2E-05	0.114	0.166	5.3E-01	4.1E-01	1.2E-01
LUAD	ATP9B_ES_30_29_31.1	ATP9B	ES	30	29	31.1	1.9E-14	0.285	0.150	5.4E-01	9.1E-01	1.9E-01
LUAD	ATXN7_ES_15_14_16	ATXN7	ES	15	14	16	5.6E-05	0.102	0.027	4.3E-01	2.4E-01	3.1E-01
LUAD	AURKAIP1_RI_1.4_1.3_1.5	AURKAIP1	RI	1.4	1.3	1.5	1.8E-05	0.109	-0.106	2.2E-01	7.9E-01	7.5E-02
LUAD	BABAM1_AA_2.1_1.2_2.2	BABAM1	AA	2.1	1.2	2.2	1.1E-05	0.110	-0.077	3.7E-01	5.7E-01	1.8E-01
LUAD	BAIAP2_ES_17.1_16.1_18.1	BAIAP2	ES	17.1	16.1	18.1	7.8E-06	0.117	0.391	8.3E-01	3.8E-01	9.1E-01
LUAD	BAX_RI_6.2_6.1_6.3	BAX	RI	6.2	6.1	6.3	1.2E-05	0.107	-0.128	3.6E-01	8.9E-01	6.8E-01
LUAD	BIN1_ES_13_12_17	BIN1	ES	13	12	17	6.8E-06	0.115	0.009	5.8E-02	9.7E-02	3.7E-01

cancer type	id	Gene Symbol	splice_type	Exon	From.Exon	To.Exon	anova.p	adj.r2	r	p.50	p.25	p.10
LUAD	BPTF_AD_23.2_23.1_24	BPTF	AD	23.2	23.1	24	2.2E-07	0.141	-0.078	6.5E-01	5.0E-01	9.5E-01
LUAD	BRD8_ES_12_11_13	BRD8	ES	12	11	13	2.5E-06	0.121	0.215	6.2E-01	4.2E-01	6.9E-02
LUAD	C12orf73_RI_2.3_2.2_2.4	C12orf73	RI	2.3	2.2	2.4	2.0E-06	0.131	-0.016	9.0E-01	7.1E-01	6.7E-01
LUAD	C14orf2_ME_3|4_2_5	C14orf2	ME	3|4	2	5	1.4E-05	0.111	-0.090	6.6E-01	5.8E-01	5.6E-01
LUAD	C14orf80_ES_9_8_10	C14orf80	ES	9	8	10	1.0E-06	0.135	-0.187	9.3E-02	4.7E-02	2.8E-01
LUAD	C16orf13_ES_2_1_3	C16orf13	ES	2	1	3	6.6E-09	0.171	0.008	1.7E-02	3.9E-02	3.2E-01
LUAD	C16orf13_ES_2_1_5	C16orf13	ES	2	1	5	3.9E-05	0.101	0.000	2.2E-01	4.8E-01	6.7E-02
LUAD	C16orf13_ES_2:3_1_5	C16orf13	ES	2:03	1	5	5.5E-06	0.116	-0.020	6.3E-01	4.0E-01	3.6E-02
LUAD	C16orf13_ES_3_1_4	C16orf13	ES	3	1	4	1.5E-05	0.124	0.058	2.1E-01	2.0E-01	1.4E-01
LUAD	C16orf59_RI_8.2_8.1_8.3	C16orf59	RI	8.2	8.1	8.3	6.3E-05	0.109	0.170	3.8E-02	2.6E-03	2.7E-03
LUAD	C16orf91_RI_4.2_4.1_4.3	C16orf91	RI	4.2	4.1	4.3	1.3E-06	0.133	-0.017	1.9E-02	6.5E-03	1.1E-01
LUAD	C19orf60_ES_3.2_2_4	C19orf60	ES	3.2	2	4	9.4E-09	0.178	0.127	3.0E-02	1.4E-01	8.9E-02
LUAD	CADM1_ES_10_9_12	CADM1	ES	10	9	12	1.4E-06	0.128	0.120	5.6E-01	2.3E-01	4.0E-01
LUAD	CASK_ES_20_19.1_22	CASK	ES	20	19.1	22	1.6E-05	0.116	0.086	4.7E-01	4.8E-02	1.8E-02
LUAD	CASK_ES_20:21_19.1_22	CASK	ES	20:21	19.1	22	2.8E-06	0.139	-0.035	5.5E-01	6.6E-01	4.2E-01
LUAD	CASK_ES_21_19.1_22	CASK	ES	21	19.1	22	3.5E-05	0.109	-0.057	1.9E-01	5.9E-01	1.0E+00
LUAD	CC2D2A_ES_5_4_6	CC2D2A	ES	5	4	6	7.7E-08	0.158	-0.273	1.0E-01	8.6E-03	4.9E-02
LUAD	CCDC106_AA_1.3:1.4_1.1_1.5	CCDC106	AA	1.3:1.4	1.1	1.5	1.1E-04	0.103	-0.115	4.5E-01	1.7E-02	1.1E-01
LUAD	CCDC107_RI_3.4_3.3_3.5	CCDC107	RI	3.4	3.3	3.5	1.4E-08	0.166	-0.304	4.3E-01	7.1E-01	4.1E-01
LUAD	CCDC50_ES_6_5_7	CCDC50	ES	6	5	7	9.3E-06	0.110	0.059	8.5E-01	9.0E-01	3.1E-01
LUAD	CD164L2_RI_4.2_4.1_4.3	CD164L2	RI	4.2	4.1	4.3	3.5E-06	0.132	-0.275	7.0E-01	7.6E-01	8.9E-01
LUAD	CD44_ES_10:11:12.1:13:14_5_15	CD44	ES	10:11:12.1:13:14	5	15	7.3E-08	0.155	0.240	1.3E-01	9.3E-02	4.0E-02
LUAD	CD44_ES_12.1:13:14_5_15	CD44	ES	12.1:13:14	5	15	2.2E-08	0.160	0.232	9.8E-01	7.7E-01	5.3E-01
LUAD	CD44_ES_7:12.1:13:14_5_15	CD44	ES	7:12.1:13:14	5	15	1.1E-06	0.167	-0.003	9.1E-01	1.2E-01	6.3E-01
LUAD	CD47_ES_9:10_8_11	CD47	ES	9:10	8	11	2.4E-09	0.177	-0.128	9.0E-01	6.3E-01	3.6E-01
LUAD	CELF1_AA_18.1:18.2_17_18.3	CELF1	AA	18.1:18.2	17	18.3	1.0E-06	0.128	0.032	1.3E-01	5.1E-02	2.5E-01
LUAD	CHCHD4_ES_2_1_4	CHCHD4	ES	2	1	4	1.9E-05	0.106	0.130	9.4E-02	2.9E-01	9.9E-01
LUAD	CHEK2_ME_6|7.1:7.2_5_9	CHEK2	ME	6|7.1:7.2	5	9	8.6E-09	0.167	0.589	1.0E-01	7.4E-02	6.4E-01
LUAD	CIRBP_ES_9.5:9.6_9.3_9.8	CIRBP	ES	9.5:9.6	9.3	9.8	1.7E-05	0.105	0.161	6.1E-01	3.9E-01	6.0E-01
LUAD	CIRBP_RI_9.9:9.10_9.8_9.11	CIRBP	RI	9.9:9.10	9.8	9.11	1.5E-05	0.108	-0.068	7.1E-01	3.7E-01	8.2E-01
LUAD	COASY_AA_1.3:1.4_1.1_1.5	COASY	AA	1.3:1.4	1.1	1.5	1.9E-05	0.129	0.178	4.6E-02	1.4E-01	9.3E-01
LUAD	COASY_AD_1.2:1.3_1.1_1.5	COASY	AD	1.2:1.3	1.1	1.5	3.0E-05	0.117	0.132	1.2E-01	2.4E-02	5.9E-02
LUAD	COL16A1_ES_44_43_45.1	COL16A1	ES	44	43	45.1	3.1E-08	0.174	0.039	6.1E-04	5.2E-03	5.4E-01
LUAD	COL6A3_ES_3_2_4	COL6A3	ES	3	2	4	7.3E-05	0.105	-0.033	4.5E-01	1.3E-01	1.3E-01
LUAD	COL6A3_ES_6_5_7	COL6A3	ES	6	5	7	2.3E-08	0.164	0.265	2.0E-03	2.0E-05	1.2E-02
LUAD	COPE_ES_4:5_3_6	COPE	ES	4:05	3	6	8.4E-06	0.123	-0.116	4.6E-02	3.5E-02	4.8E-02
LUAD	COQ4_RI_2.2_2.1_2.3	COQ4	RI	2.2	2.1	2.3	2.4E-08	0.160	-0.134	8.7E-02	1.2E-01	7.2E-01
LUAD	CSF2RA_ES_13_12_14	CSF2RA	ES	13	12	14	2.0E-08	0.161	-0.775	9.3E-01	5.2E-01	8.7E-01
LUAD	CUTC_ES_7_6_8	CUTC	ES	7	6	8	5.0E-06	0.117	0.304	8.9E-01	4.7E-01	9.6E-01
LUAD	CYB561A3_AA_6.6_6.4_6.7	CYB561A3	AA	6.6	6.4	6.7	5.8E-06	0.114	0.266	4.0E-02	2.3E-02	8.1E-01

cancer type	id	Gene Symbol	splice_type	Exon	From.Exon	To.Exon	anova.p	adj.r2	r	p.50	p.25	p.10
LUAD	CYTH1_ES_11.2:13.1_11.1_13.2	CYTH1	ES	11.2:13.1	11.1	13.2	3.4E-06	0.122	0.021	3.6E-02	2.7E-03	1.0E-02
LUAD	CYTH1_ES_12_11.1_13.2	CYTH1	ES	12	11.1	13.2	2.8E-07	0.144	0.012	5.2E-03	1.0E-02	5.5E-03
LUAD	D2HGDH_RI_7.2_7.1_7.3	D2HGDH	RI	7.2	7.1	7.3	5.0E-05	0.103	-0.179	4.7E-01	4.4E-01	8.3E-01
LUAD	DDX11_AA_22.1_21_22.2	DDX11	AA	22.1	21	22.2	2.5E-05	0.108	-0.143	5.6E-01	4.5E-01	3.7E-01
LUAD	DEDD2_ES_3_2_4.1	DEDD2	ES	3	2	4.1	1.2E-05	0.120	0.190	2.5E-01	5.0E-01	6.6E-01
LUAD	DEF8_ES_2.1_1_4	DEF8	ES	2.1	1	4	3.7E-07	0.143	0.077	1.9E-02	2.4E-03	7.2E-01
LUAD	DNAJC4_RI_7.2_7.1_7.3	DNAJC4	RI	7.2	7.1	7.3	2.8E-06	0.120	-0.063	2.0E-01	1.9E-01	1.1E-01
LUAD	DVL1_AD_11.2_11.1_12	DVL1	AD	11.2	11.1	12	4.5E-06	0.119	0.000	4.8E-01	7.3E-01	6.5E-01
LUAD	ECHDC2_ES_9.1_8.1_10	ECHDC2	ES	9.1	8.1	10	6.5E-06	0.117	0.230	4.9E-01	7.9E-01	4.4E-01
LUAD	ENAH_ES_13_12_14	ENAH	ES	13	12	14	1.2E-06	0.127	0.197	9.2E-01	5.5E-01	1.2E-01
LUAD	EPB41_ES_16_15_19.1	EPB41	ES	16	15	19.1	4.5E-07	0.171	0.122	3.6E-01	6.9E-01	7.9E-01
LUAD	EPB41_ES_16:18_15_19.1	EPB41	ES	16:18	15	19.1	2.2E-07	0.167	0.103	6.2E-01	1.4E-01	2.0E-01
LUAD	EPB41_ES_18_15_19.1	EPB41	ES	18	15	19.1	6.3E-09	0.186	0.126	8.8E-01	2.3E-01	2.3E-01
LUAD	EPB41L3_ES_25_24_26	EPB41L3	ES	25	24	26	7.3E-07	0.133	-0.288	3.3E-01	1.0E+00	3.8E-01
LUAD	EPS15L1_ES_22:23.1_21_24	EPS15L1	ES	22:23.1	21	24	3.7E-05	0.106	0.166	8.1E-01	4.5E-01	9.5E-01
LUAD	ERBB2IP_ES_24.1:24.2:24.3_21_25	ERBB2IP	ES	24.1:24.2:24.3	21	25	1.9E-05	0.103	0.132	5.7E-01	1.5E-01	7.0E-01
LUAD	EXOC7_ES_8.2_6_9	EXOC7	ES	8.2	6	9	2.5E-13	0.246	0.005	2.5E-01	2.7E-01	3.9E-01
LUAD	FAM114A1_ES_2_1_3	FAM114A1	ES	2	1	3	2.0E-05	0.105	0.066	7.8E-01	7.2E-01	5.5E-01
LUAD	FAM160A2_AD_8.2_8.1_9.1	FAM160A2	AD	8.2	8.1	9.1	5.8E-09	0.178	-0.191	5.6E-01	2.8E-01	1.3E-01
LUAD	FAM184A_ES_17_16_18	FAM184A	ES	17	16	18	8.2E-05	0.101	-0.127	3.2E-01	3.3E-01	4.5E-01
LUAD	FAM195B_RI_4.4_4.3_4.5	FAM195B	RI	4.4	4.3	4.5	1.6E-05	0.105	0.103	8.1E-01	5.4E-01	2.9E-01
LUAD	FBLN2_ES_11_10_12	FBLN2	ES	11	10	12	8.1E-12	0.224	-0.180	2.3E-02	2.9E-04	9.4E-02
LUAD	FDPS_AD_1.2_1.1_3.1	FDPS	AD	1.2	1.1	3.1	6.3E-06	0.118	-0.014	2.3E-01	7.8E-01	9.9E-01
LUAD	FDPS_AD_1.2_1.1_3.2	FDPS	AD	1.2	1.1	3.2	1.7E-09	0.186	0.107	1.2E-01	5.9E-01	7.1E-01
LUAD	FDPS_ES_1.2:3.1_1.1_3.2	FDPS	ES	1.2:3.1	1.1	3.2	1.5E-10	0.207	0.049	9.6E-01	6.8E-01	7.3E-02
LUAD	FES_ES_10.1:10.2_9_11.2	FES	ES	10.1:10.2	9	11.2	1.1E-06	0.129	-0.033	1.3E-01	5.2E-01	8.3E-01
LUAD	FGFR1_ES_6_4_8.2	FGFR1	ES	6	4	8.2	2.5E-06	0.129	0.234	6.4E-01	1.2E-01	9.5E-01
LUAD	FGFR1OP_ES_7_6_8	FGFR1OP	ES	7	6	8	1.6E-05	0.108	-0.032	2.4E-01	8.6E-01	3.3E-01
LUAD	FN1_ES_25_24_26	FN1	ES	25	24	26	4.4E-08	0.154	0.011	6.2E-04	9.3E-03	1.6E-01
LUAD	FN1_ES_40.2_39_40.4	FN1	ES	40.2	39	40.4	2.9E-11	0.211	0.279	4.1E-02	2.4E-03	8.1E-02
LUAD	FNBP1_ES_10.1:10.2:10.3:12_9_14.2	FNBP1	ES	10.1:10.2:10.3:12	9	14.2	1.8E-05	0.122	-0.116	1.6E-03	3.9E-03	1.5E-03
LUAD	FNBP1_ES_10.2:10.3_9_14.2	FNBP1	ES	10.2:10.3	9	14.2	2.0E-05	0.129	-0.151	9.6E-02	5.8E-02	6.0E-02
LUAD	FNBP1_ES_10.2:10.3:12_9_14.2	FNBP1	ES	10.2:10.3:12	9	14.2	2.8E-05	0.127	-0.135	6.8E-02	7.5E-02	8.3E-02
LUAD	FNBP1L_RI_16.2_16.1_16.3	FNBP1L	RI	16.2	16.1	16.3	2.5E-05	0.101	0.229	1.1E-01	3.3E-01	3.4E-02
LUAD	FOXM1_ES_6_5_7	FOXM1	ES	6	5	7	1.3E-06	0.137	-0.163	4.8E-01	2.6E-01	6.3E-01
LUAD	FRMD4A_ES_30_29_31	FRMD4A	ES	30	29	31	2.2E-04	0.106	-0.087	9.6E-01	3.9E-01	4.7E-01
LUAD	FYN_ME_11|12_10_13	FYN	ME	11|12	10	13	6.6E-09	0.170	0.074	7.9E-01	8.2E-01	5.8E-01
LUAD	GIT2_ES_19_18.2_20	GIT2	ES	19	18.2	20	4.8E-07	0.136	0.165	3.9E-01	1.1E-01	7.8E-01
LUAD	GLUL_AD_1.3:1.4_1.2_2	GLUL	AD	1.3:1.4	1.2	2	1.7E-05	0.105	0.022	6.2E-01	8.7E-01	6.6E-01
LUAD	GNB2L1_ES_4.1:4.2:5:6:7.2:8.1:8.2_3_9	GNB2L1	ES	l:4.2:5:6:7.2:8.1:8	3	9	1.2E-06	0.129	-0.475	7.4E-01	3.1E-01	4.5E-01

cancer type	id	Gene Symbol	splice_type	Exon	From.Exon	To.Exon	anova.p	adj.r2	r	p.50	p.25	p.10
LUAD	GOLGA2_ES_7_6_8	GOLGA2	ES	7	6	8	4.2E-07	0.137	0.046	2.4E-01	4.2E-02	1.1E-01
LUAD	GOLGA4_ES_24_23_25	GOLGA4	ES	24	23	25	4.5E-08	0.154	0.142	9.6E-01	1.1E-02	8.3E-01
LUAD	GPR116_ES_22_21_23	GPR116	ES	22	21	23	2.7E-13	0.246	-0.719	2.3E-03	1.7E-03	9.0E-02
LUAD	GRK6_AA_17.1_15_17.2	GRK6	AA	17.1	15	17.2	1.8E-05	0.106	0.158	4.6E-01	8.4E-01	2.2E-01
LUAD	GTF3C1_AD_34.2_34.1_35	GTF3C1	AD	34.2	34.1	35	3.2E-05	0.100	-0.018	8.2E-01	9.7E-01	3.4E-01
LUAD	GUCD1_ES_6:7.1_5_7.2	GUCD1	ES	06:07.1	5	7.2	7.5E-07	0.144	0.012	4.3E-01	4.3E-02	5.7E-02
LUAD	H2AFY_ME_7|8_6.3_9	H2AFY	ME	7|8	6.3	9	4.8E-08	0.154	0.188	6.3E-01	7.0E-01	1.4E-01
LUAD	HEXDC_RI_11.3:11.4_11.2_11.5	HEXDC	RI	11.3:11.4	11.2	11.5	1.2E-07	0.149	0.139	2.1E-01	3.1E-01	9.5E-01
LUAD	HM13_ES_11:12.1_10_13	HM13	ES	11:12.1	10	13	3.1E-09	0.185	-0.109	5.2E-01	1.1E-01	6.8E-02
LUAD	HMGA1_ES_1.3_1.1_3.1	HMGA1	ES	1.3	1.1	3.1	4.6E-09	0.182	-0.377	2.4E-01	1.4E-01	6.5E-01
LUAD	HOPX_AD_4.3:4.4_4.2_4.6	HOPX	AD	4.3:4.4	4.2	4.6	1.6E-08	0.178	0.492	4.2E-01	2.2E-01	1.9E-02
LUAD	HOPX_RI_4.3:4.4:4.5_4.2_4.6	HOPX	RI	4.3:4.4:4.5	4.2	4.6	6.1E-07	0.141	0.443	8.1E-01	6.4E-02	8.3E-03
LUAD	HSD17B1_ES_4_3_5.2	HSD17B1	ES	4	3	5.2	1.4E-06	0.141	-0.546	9.1E-01	8.4E-01	8.8E-02
LUAD	HYAL3_AA_3.1_1_3.2	HYAL3	AA	3.1	1	3.2	6.7E-06	0.120	-0.008	9.6E-01	4.5E-01	6.0E-03
LUAD	IGFLR1_AA_4.1_3_4.2	IGFLR1	AA	4.1	3	4.2	1.7E-04	0.109	0.171	1.6E-01	3.0E-01	7.5E-01
LUAD	IGFLR1_ES_3:4.2_2.2_4.3	IGFLR1	ES	03:04.2	2.2	4.3	8.7E-06	0.143	-0.375	1.6E-01	3.6E-01	1.3E-01
LUAD	IGFLR1_ES_3:4.2:4.3_2.2_5	IGFLR1	ES	3:4.2:4.3	2.2	5	4.3E-05	0.116	-0.300	4.5E-01	7.8E-01	1.8E-01
LUAD	IL32_AA_1.3:1.4:1.5:1.6:1.7_1.1_1.8	IL32	AA	1.3:1.4:1.5:1.6:1.[illegible]	1.1	1.8	1.5E-06	0.131	0.283	3.7E-02	1.4E-01	7.6E-03
LUAD	IMMP1L_ES_3:4:5:6_1_7	IMMP1L	ES	3:4:5:6	1	7	4.1E-05	0.113	-0.124	1.7E-02	5.0E-02	2.5E-01
LUAD	IMMP1L_ES_4:5:6_1_7	IMMP1L	ES	4:05:06	1	7	8.1E-07	0.134	-0.165	3.7E-01	1.3E-02	1.3E-01
LUAD	INO80E_ES_6.1:6.2:6.3:10_5_11	INO80E	ES	6.1:6.2:6.3:10	5	11	3.6E-05	0.110	-0.039	7.3E-01	4.4E-01	3.5E-02
LUAD	IRAK1_ES_10.1:10.3_9_11.1	IRAK1	ES	10.1:10.3	9	11.1	5.9E-06	0.127	-0.136	7.5E-01	5.1E-01	3.2E-01
LUAD	IRF3_ES_1.2:1.3:1.4:1.5:2_1.1_3	IRF3	ES	1.2:1.3:1.4:1.5:2	1.1	3	5.0E-05	0.143	0.162	3.5E-02	3.9E-02	4.1E-02
LUAD	IRF3_ES_1.4:1.5:2_1.1_3	IRF3	ES	1.4:1.5:2	1.1	3	3.0E-06	0.148	0.045	1.5E-01	8.0E-02	5.2E-01
LUAD	ISOC2_ES_3_2_4.2	ISOC2	ES	3	2	4.2	1.3E-05	0.108	-0.103	2.8E-01	8.3E-02	7.8E-02
LUAD	ITGAE_ES_28_27_29	ITGAE	ES	28	27	29	1.8E-08	0.164	0.301	5.7E-01	3.5E-01	1.3E-01
LUAD	ITGB3BP_ES_9_8_10	ITGB3BP	ES	9	8	10	8.8E-06	0.110	-0.130	6.4E-01	4.8E-01	8.6E-01
LUAD	KANSL3_AA_5.1_3.2_5.2	KANSL3	AA	5.1	3.2	5.2	1.5E-05	0.116	-0.031	6.0E-03	1.5E-02	5.6E-02
LUAD	KCNRG_ES_2_1_3	KCNRG	ES	2	1	3	8.8E-05	0.114	-0.596	2.9E-01	3.1E-01	8.7E-01
LUAD	KCTD18_AD_1.2_1.1_2	KCTD18	AD	1.2	1.1	2	1.1E-05	0.113	0.001	2.7E-01	5.7E-01	7.9E-01
LUAD	KDM1A_ES_3_2_4	KDM1A	ES	3	2	4	2.7E-08	0.160	-0.266	3.3E-01	3.6E-02	5.5E-02
LUAD	KIAA0895L_ES_2.1:2.2_1_3.1	KIAA0895L	ES	2.1:2.2	1	3.1	3.3E-06	0.137	0.079	8.4E-01	6.6E-01	7.7E-01
LUAD	KIAA1715_ES_4_3_5	KIAA1715	ES	4	3	5	1.4E-05	0.127	0.022	2.3E-01	3.7E-01	4.6E-01
LUAD	KIF3C_ES_6_5_8	KIF3C	ES	6	5	8	3.6E-08	0.174	-0.734	2.8E-01	4.2E-01	8.3E-01
LUAD	KLHDC10_ES_2_1_3	KLHDC10	ES	2	1	3	1.1E-04	0.105	0.166	5.3E-01	5.3E-01	8.9E-01
LUAD	KREMEN1_AD_7.2_7.1_8	KREMEN1	AD	7.2	7.1	8	9.6E-05	0.106	0.104	1.9E-01	6.2E-01	1.1E-01
LUAD	KSR1_AA_24.1_22_24.2	KSR1	AA	24.1	22	24.2	1.3E-05	0.110	0.324	2.3E-01	4.4E-01	9.8E-01
LUAD	KTN1_ES_35_34.2_36	KTN1	ES	35	34.2	36	7.2E-06	0.112	0.209	6.6E-02	3.8E-01	3.2E-01
LUAD	LEF1_ES_7_6.1_8	LEF1	ES	7	6.1	8	7.0E-06	0.122	0.030	5.0E-02	1.5E-01	1.9E-01
LUAD	LGMN_ES_13_12_15	LGMN	ES	13	12	15	7.3E-05	0.131	-0.141	3.1E-01	1.9E-02	1.7E-01

cancer type	id	Gene Symbol	splice_type	Exon	From.Exon	To.Exon	anova.p	adj.r2	r	p.50	p.25	p.10
LUAD	LGMN_ES_14_12_15	LGMN	ES	14	12	15	2.5E-06	0.126	0.047	4.7E-01	3.4E-01	2.5E-01
LUAD	LRRFIP2_ES_20_19_21	LRRFIP2	ES	20	19	21	3.6E-08	0.156	-0.029	9.5E-01	8.1E-01	9.6E-01
LUAD	LTBP3_ES_25_24_26	LTBP3	ES	25	24	26	2.1E-05	0.107	0.407	7.9E-02	9.2E-01	6.7E-01
LUAD	MACF1_ES_103_102_104	MACF1	ES	103	102	104	6.6E-08	0.151	0.105	6.7E-01	1.0E-01	5.8E-03
LUAD	MACF1_ES_107_106_108	MACF1	ES	107	106	108	2.4E-05	0.101	-0.016	6.0E-01	8.2E-01	2.8E-01
LUAD	MAGIX_AA_4.1_3_4.2	MAGIX	AA	4.1	3	4.2	3.6E-05	0.107	0.022	5.9E-01	6.7E-01	2.6E-01
LUAD	MAP3K4_ES_18_17_19	MAP3K4	ES	18	17	19	5.0E-07	0.140	0.028	8.5E-01	4.9E-01	3.5E-01
LUAD	MAP3K7_ES_11_10_12	MAP3K7	ES	11	10	12	1.9E-05	0.103	-0.102	4.4E-02	4.0E-01	4.6E-01
LUAD	MAPKAPK5_AD_15.2_15.1_16	MAPKAPK5	AD	15.2	15.1	16	2.2E-06	0.122	0.165	1.8E-01	1.1E-02	5.1E-01
LUAD	MARK3_ES_17_16_18	MARK3	ES	17	16	18	8.5E-05	0.101	-0.197	4.5E-01	7.3E-02	3.1E-01
LUAD	MBD1_ES_13.1:13.2_12_14	MBD1	ES	13.1:13.2	12	14	7.0E-08	0.155	-0.057	2.7E-01	8.9E-02	3.4E-01
LUAD	MBNL1_ES_8_7_9	MBNL1	ES	8	7	9	2.0E-09	0.179	-0.367	2.1E-02	1.7E-04	2.0E-01
LUAD	MBNL2_ES_7_6.3_8	MBNL2	ES	7	6.3	8	1.6E-06	0.131	-0.252	4.6E-01	5.1E-01	3.1E-01
LUAD	MEFV_ES_6_5_7	MEFV	ES	6	5	7	1.8E-05	0.109	-0.534	4.9E-01	3.4E-01	4.2E-01
LUAD	MEIS3_AA_7.1_6_7.2	MEIS3	AA	7.1	6	7.2	3.2E-08	0.164	0.676	1.0E-01	1.8E-01	4.0E-01
LUAD	MELK_ES_8_7_9	MELK	ES	8	7	9	1.6E-05	0.115	-0.599	3.0E-02	4.1E-04	2.7E-02
LUAD	MFSD10_RI_12.2_12.1_12.3	MFSD10	RI	12.2	12.1	12.3	8.7E-11	0.209	-0.076	3.8E-01	8.2E-01	2.3E-01
LUAD	MLST8_AA_3.1_1_3.2	MLST8	AA	3.1	1	3.2	6.0E-06	0.121	0.067	9.8E-01	7.0E-01	9.7E-01
LUAD	MND1_ES_5:6_4_7	MND1	ES	5:06	4	7	9.8E-06	0.111	-0.368	5.4E-03	1.9E-02	2.7E-01
LUAD	MRPL52_RI_1.2:1.3:1.4_1.1_1.5	MRPL52	RI	1.2:1.3:1.4	1.1	1.5	2.9E-08	0.163	-0.006	4.3E-01	5.7E-01	7.9E-01
LUAD	MRPS12_AD_1.2_1.1_1.4	MRPS12	AD	1.2	1.1	1.4	1.2E-07	0.150	0.181	8.2E-01	6.3E-01	7.0E-01
LUAD	MTA1_ES_4_3_5	MTA1	ES	4	3	5	3.0E-06	0.121	-0.179	4.0E-01	8.7E-02	1.5E-01
LUAD	MTMR1_ES_4_3_5	MTMR1	ES	4	3	5	1.1E-07	0.164	-0.190	3.5E-01	2.4E-01	1.1E-01
LUAD	MUM1_ES_5.1:5.2_4_6	MUM1	ES	5.1:5.2	4	6	1.6E-10	0.205	-0.107	4.5E-01	3.3E-01	4.9E-01
LUAD	MYH10_ES_6.1_5_7	MYH10	ES	6.1	5	7	4.2E-07	0.170	-0.007	7.4E-01	5.3E-01	4.6E-01
LUAD	MYL6_ES_2.1_1.4_3.1	MYL6	ES	2.1	1.4	3.1	7.4E-10	0.190	-0.095	2.3E-01	1.3E-01	6.7E-01
LUAD	MYL6_ES_4.3_4.1_5	MYL6	ES	4.3	4.1	5	3.1E-06	0.119	0.016	6.8E-01	2.4E-01	9.2E-01
LUAD	MYO1B_ES_23:24_22_25	MYO1B	ES	23:24	22	25	1.2E-06	0.127	-0.015	8.6E-01	5.2E-01	4.0E-01
LUAD	MYO9B_ES_37_36_38.1	MYO9B	ES	37	36	38.1	2.9E-05	0.104	-0.263	2.2E-01	2.6E-01	1.0E-02
LUAD	MYOF_ES_17_16_18	MYOF	ES	17	16	18	1.1E-08	0.173	-0.209	2.1E-01	2.9E-01	2.1E-01
LUAD	NAPSA_ES_4_2_5	NAPSA	ES	4	2	5	1.3E-05	0.129	-0.188	6.8E-01	8.3E-01	2.2E-01
LUAD	NAT14_RI_2.3_2.2_2.4	NAT14	RI	2.3	2.2	2.4	1.7E-06	0.126	-0.041	7.2E-01	8.9E-01	7.3E-01
LUAD	NBEAL2_ES_10_9_11	NBEAL2	ES	10	9	11	1.0E-05	0.142	0.411	9.1E-01	9.0E-01	7.4E-01
LUAD	NDEL1_ES_11_10_12.1	NDEL1	ES	11	10	12.1	2.7E-05	0.102	0.027	1.7E-01	4.0E-02	6.3E-01
LUAD	NDUFB10_RI_3.2_3.1_3.3	NDUFB10	RI	3.2	3.1	3.3	1.2E-10	0.201	-0.148	4.2E-01	6.4E-01	9.8E-01
LUAD	NEDD4L_ES_18_17_19	NEDD4L	ES	18	17	19	2.8E-05	0.101	0.283	1.7E-03	1.9E-03	3.1E-02
LUAD	NF2_ES_16.1_15_17	NF2	ES	16.1	15	17	4.5E-10	0.191	0.203	6.3E-01	7.7E-01	1.5E-01
LUAD	NFIB_ES_12.1:12.2:13:14_11_15	NFIB	ES	12.1:12.2:13:14	11	15	3.5E-05	0.103	0.063	3.3E-01	8.2E-02	5.4E-03
LUAD	NFIB_ES_12.1:13:14_11_15	NFIB	ES	12.1:13:14	11	15	3.3E-08	0.168	0.063	2.4E-01	4.0E-01	5.0E-01
LUAD	NOP2_AD_1.2_1.1_2.2	NOP2	AD	1.2	1.1	2.2	7.5E-06	0.117	0.129	7.2E-01	5.4E-01	3.6E-01

cancer type	id	Gene Symbol	splice_type	Exon	From.Exon	To.Exon	anova.p	adj.r2	r	p.50	p.25	p.10
LUAD	NPRL3_ES_2:3_1.4_4	NPRL3	ES	2:03	1.4	4	7.0E-07	0.137	-0.090	2.4E-02	1.0E-01	6.7E-01
LUAD	NRIP1_ES_3_2_4	NRIP1	ES	3	2	4	4.9E-06	0.130	-0.061	8.1E-01	4.7E-01	3.9E-01
LUAD	NUMA1_ES_18_17_19	NUMA1	ES	18	17	19	1.8E-08	0.162	-0.047	6.5E-02	4.7E-02	3.0E-01
LUAD	NWD1_AD_7.2_7.1_8	NWD1	AD	7.2	7.1	8	3.7E-05	0.108	-0.697	1.6E-01	3.3E-02	1.8E-02
LUAD	OGFOD2_AA_8.2_6.2_8.3	OGFOD2	AA	8.2	6.2	8.3	2.4E-05	0.109	0.000	9.0E-01	9.3E-01	6.2E-01
LUAD	OGG1_RI_6.2:6.3_6.1_6.4	OGG1	RI	6.2:6.3	6.1	6.4	4.4E-08	0.158	-0.071	6.8E-01	2.3E-01	7.0E-01
LUAD	PAIP1_AD_2.2_2.1_3	PAIP1	AD	2.2	2.1	3	1.5E-05	0.107	0.135	3.0E-02	6.0E-02	8.8E-02
LUAD	PAK4_ES_2.2:2.3_1_4	PAK4	ES	2.2:2.3	1	4	8.7E-06	0.113	-0.112	1.2E-01	1.9E-01	3.7E-01
LUAD	PARP3_ES_2:3.1_1_3.2	PARP3	ES	02:03.1	1	3.2	5.6E-05	0.104	0.264	9.2E-01	4.7E-01	7.3E-01
LUAD	PDGFA_ES_6_5_7	PDGFA	ES	6	5	7	1.1E-07	0.148	0.421	5.8E-01	3.7E-01	1.1E-01
LUAD	PEX5_ES_9_8_10	PEX5	ES	9	8	10	8.4E-07	0.131	-0.077	6.5E-02	6.6E-01	6.4E-01
LUAD	PHACTR2_ES_8_6_9	PHACTR2	ES	8	6	9	1.5E-06	0.164	-0.066	1.2E-01	6.2E-01	3.8E-01
LUAD	PICALM_ES_14.1:14.2_13_15	PICALM	ES	14.1:14.2	13	15	7.6E-13	0.237	-0.082	1.8E-01	2.9E-01	7.4E-01
LUAD	PKP4_ES_25_24_26	PKP4	ES	25	24	26	1.5E-08	0.163	-0.039	7.1E-02	7.2E-03	1.2E-03
LUAD	PLA2G10_ES_4_3_5	PLA2G10	ES	4	3	5	1.9E-06	0.125	-0.791	9.7E-01	6.9E-01	4.2E-01
LUAD	PLEKHM2_ES_7_6_8	PLEKHM2	ES	7	6	8	2.8E-05	0.100	0.072	7.8E-02	3.4E-01	9.4E-01
LUAD	POSTN_ES_17:18:19_16_20	POSTN	ES	17:18:19	16	20	2.5E-05	0.110	-0.246	3.7E-02	1.3E-02	1.8E-02
LUAD	PPCS_AD_2.2_2.1_3.1	PPCS	AD	2.2	2.1	3.1	2.2E-05	0.102	-0.249	1.0E-01	1.1E-01	7.7E-02
LUAD	PPHLN1_ES_6_5_7	PPHLN1	ES	6	5	7	7.4E-07	0.131	0.005	2.7E-01	7.5E-01	8.5E-01
LUAD	PPIP5K1_ES_28_27_29	PPIP5K1	ES	28	27	29	6.8E-05	0.104	0.148	6.6E-01	5.1E-01	1.4E-01
LUAD	PPP1CA_ES_2.1:2.2_1_3	PPP1CA	ES	2.1:2.2	1	3	1.5E-05	0.108	-0.183	4.6E-01	1.6E-01	6.1E-01
LUAD	PPP1R9A_ES_15.1:15.2:16:17:18.1_14_1	PPP1R9A	ES	5.1:15.2:16:17:18	14	19	7.0E-07	0.149	-0.013	7.2E-01	3.6E-01	7.1E-01
LUAD	PRDX5_ES_3_1_4	PRDX5	ES	3	1	4	3.1E-06	0.121	0.061	1.9E-01	2.5E-02	1.6E-01
LUAD	PRKAG1_AD_1.2_1.1_2	PRKAG1	AD	1.2	1.1	2	8.9E-07	0.130	-0.375	5.5E-01	7.2E-01	9.8E-01
LUAD	PRPF40A_ES_8_7_9	PRPF40A	ES	8	7	9	2.4E-06	0.126	0.008	8.8E-01	8.1E-01	8.3E-01
LUAD	PSMA3_ES_3.2_2_4	PSMA3	ES	3.2	2	4	4.8E-05	0.113	-0.082	8.8E-01	7.3E-01	4.5E-01
LUAD	PSTPIP1_ES_3_2.2_5	PSTPIP1	ES	3	2.2	5	7.5E-08	0.150	-0.703	8.5E-03	7.3E-04	3.0E-04
LUAD	PTK2_ES_39.2_37_39.5	PTK2	ES	39.2	37	39.5	2.6E-06	0.122	0.020	8.4E-01	7.3E-01	9.1E-01
LUAD	PTK2B_ES_28_27_29	PTK2B	ES	28	27	29	3.5E-07	0.139	-0.027	2.3E-02	7.0E-03	2.3E-02
LUAD	PTPN18_ES_3:4:5:6_1_7	PTPN18	ES	3:4:5:6	1	7	1.0E-06	0.130	0.061	5.4E-01	4.2E-01	3.6E-01
LUAD	PXN_ES_4_1_5	PXN	ES	4	1	5	8.2E-06	0.117	0.025	1.6E-01	9.2E-01	1.0E+00
LUAD	QSOX1_RI_12.2_12.1_12.3	QSOX1	RI	12.2	12.1	12.3	6.7E-10	0.187	-0.020	7.9E-01	5.5E-01	9.7E-01
LUAD	R3HDM1_ES_16_15_17	R3HDM1	ES	16	15	17	1.4E-05	0.111	0.201	1.0E+00	8.4E-01	6.3E-01
LUAD	RAI14_ES_18_17_19	RAI14	ES	18	17	19	9.8E-06	0.109	0.322	3.0E-01	7.4E-01	8.6E-01
LUAD	RALGAPA1_ES_42_41_43	RALGAPA1	ES	42	41	43	7.0E-08	0.153	-0.037	2.8E-01	1.0E+00	1.5E-01
LUAD	RANGRF_RI_3.4_3.3_3.5	RANGRF	RI	3.4	3.3	3.5	2.3E-05	0.102	-0.201	5.2E-02	1.5E-02	7.6E-02
LUAD	RASSF7_RI_6.2_6.1_6.3	RASSF7	RI	6.2	6.1	6.3	1.7E-08	0.165	-0.304	6.9E-01	4.1E-01	1.3E-01
LUAD	RASSF7_RI_6.2:6.3:6.4_6.1_6.5	RASSF7	RI	6.2:6.3:6.4	6.1	6.5	2.7E-09	0.179	-0.088	2.7E-01	1.6E-02	5.6E-01
LUAD	RASSF7_RI_6.4_6.3_6.5	RASSF7	RI	6.4	6.3	6.5	2.2E-08	0.163	-0.285	2.4E-01	5.5E-01	6.5E-01
LUAD	RBM42_ES_3.2:4:6.1:6.2:6.3:7:8:9.1_3.1	RBM42	ES	:4:6.1:6.2:6.3:7:8	3.1	9.2	2.3E-05	0.115	-0.159	8.3E-01	8.6E-01	7.8E-01

cancer type	id	Gene Symbol	splice_type	Exon	From.Exon	To.Exon	anova.p	adj.r2	r	p.50	p.25	p.10
LUAD	RCC1_ES_7_6_8	RCC1	ES	7	6	8	1.7E-08	0.165	-0.157	3.7E-01	9.1E-02	1.4E-01
LUAD	RECQL5_ES_1.2:2.1_1.1_2.2	RECQL5	ES	1.2:2.1	1.1	2.2	1.2E-06	0.134	0.031	3.9E-01	5.9E-01	7.8E-01
LUAD	RNASEH2C_RI_1.2_1.1_1.3	RNASEH2C	RI	1.2	1.1	1.3	2.9E-07	0.144	0.075	8.4E-01	9.1E-01	5.9E-01
LUAD	RORC_ES_2_1_3	RORC	ES	2	1	3	1.4E-05	0.121	-0.179	2.2E-01	2.4E-02	9.5E-03
LUAD	RPL15_AD_1.3_1.2_3.2	RPL15	AD	1.3	1.2	3.2	3.7E-07	0.138	-0.103	3.5E-01	3.0E-03	5.2E-02
LUAD	RPL37A_RI_2.2:2.3_2.1_2.4	RPL37A	RI	2.2:2.3	2.1	2.4	3.6E-04	0.102	-0.207	5.4E-01	6.5E-01	3.7E-01
LUAD	RPS24_AA_5.1_4_5.2	RPS24	AA	5.1	4	5.2	2.3E-15	0.277	0.240	2.6E-02	5.4E-02	9.6E-02
LUAD	RPS24_ES_5.1:5.2_4_6	RPS24	ES	5.1:5.2	4	6	1.3E-10	0.200	0.079	3.8E-02	3.5E-02	2.4E-02
LUAD	S100A16_AD_2.2_2.1_4	S100A16	AD	2.2	2.1	4	1.7E-07	0.144	0.122	8.7E-01	9.6E-01	3.7E-01
LUAD	SCP2_ES_12_11_13	SCP2	ES	12	11	13	1.0E-10	0.201	-0.623	1.1E-01	5.1E-01	5.9E-01
LUAD	SEC31A_ES_26.1_25.1_28	SEC31A	ES	26.1	25.1	28	2.1E-06	0.122	0.041	6.3E-01	5.6E-01	7.2E-02
LUAD	SEC31A_ES_26.1:26.2:27_25.1_28	SEC31A	ES	26.1:26.2:27	25.1	28	2.2E-05	0.102	0.122	8.9E-01	5.5E-01	5.1E-01
LUAD	SEC31A_ES_26.1:27_25.1_28	SEC31A	ES	26.1:27	25.1	28	1.9E-06	0.138	0.108	5.8E-01	7.2E-01	2.1E-01
LUAD	SERPINA1_ES_2.1:2.2:2.3:2.4:2.5_1.1_3.	SERPINA1	ES	.1:2.2:2.3:2.4:2.	1.1	3.2	2.1E-05	0.103	0.648	6.6E-01	4.8E-01	5.1E-01
LUAD	SETD8_ES_2_1_3	SETD8	ES	2	1	3	1.3E-05	0.111	-0.314	8.6E-01	3.5E-01	9.8E-03
LUAD	SFTA3_ES_2:4.1:4.2_1.3_6	SFTA3	ES	2:4.1:4.2	1.3	6	1.5E-04	0.101	0.051	6.9E-01	6.2E-01	2.6E-01
LUAD	SH3BP1_ES_16_15_17.2	SH3BP1	ES	16	15	17.2	5.5E-06	0.114	-0.505	9.0E-03	1.9E-02	4.0E-01
LUAD	SLAIN2_ES_8_6_9	SLAIN2	ES	8	6	9	2.2E-09	0.178	0.070	4.7E-02	3.8E-01	6.3E-01
LUAD	SLC25A39_AA_5.1_4_5.2	SLC25A39	AA	5.1	4	5.2	2.3E-05	0.102	-0.226	3.0E-01	9.3E-01	7.5E-01
LUAD	SLC50A1_ES_4_3_6	SLC50A1	ES	4	3	6	2.2E-06	0.139	0.209	2.1E-01	2.8E-01	3.1E-01
LUAD	SLK_ES_13_12_14	SLK	ES	13	12	14	1.4E-06	0.126	0.205	4.7E-03	6.4E-02	7.7E-01
LUAD	SMAP1_ES_6_5.2_7	SMAP1	ES	6	5.2	7	2.6E-10	0.197	0.143	3.6E-03	2.0E-04	7.4E-02
LUAD	SMARCC2_ES_28.3_28.1_29	SMARCC2	ES	28.3	28.1	29	1.3E-09	0.219	0.276	7.7E-01	8.9E-01	3.6E-01
LUAD	SMEK2_ES_10_9_11	SMEK2	ES	10	9	11	8.0E-06	0.111	-0.144	3.6E-01	3.3E-01	5.8E-01
LUAD	SNX21_ES_3_2_4.1	SNX21	ES	3	2	4.1	1.6E-05	0.113	-0.181	2.1E-01	3.7E-01	5.1E-02
LUAD	SNX5_RI_1.2_1.1_1.3	SNX5	RI	1.2	1.1	1.3	4.5E-05	0.100	-0.095	3.8E-02	1.5E-01	2.8E-01
LUAD	SORBS2_ES_20:22_19_23	SORBS2	ES	20:22	19	23	9.1E-08	0.180	0.027	1.4E-01	9.2E-01	7.5E-02
LUAD	SPATS2_ES_2_1_5	SPATS2	ES	2	1	5	5.3E-05	0.102	0.011	3.0E-01	5.8E-01	4.4E-01
LUAD	SRRM1_ES_16_15_17	SRRM1	ES	16	15	17	1.2E-05	0.108	-0.042	8.9E-01	4.8E-01	8.7E-01
LUAD	SSBP3_ES_7_6_8	SSBP3	ES	7	6	8	1.1E-11	0.220	0.114	4.8E-01	1.1E-01	9.9E-01
LUAD	SSH2_RI_1.2_1.1_1.3	SSH2	RI	1.2	1.1	1.3	4.5E-07	0.141	0.191	2.0E-01	1.1E-01	1.5E-01
LUAD	STOML1_ES_5_4_6	STOML1	ES	5	4	6	2.8E-05	0.101	0.061	1.4E-01	6.6E-01	2.1E-01
LUAD	STRA13_ES_3.1_2_4.1	STRA13	ES	3.1	2	4.1	8.3E-07	0.134	-0.252	1.9E-02	1.1E-02	7.5E-01
LUAD	STRA6_AD_18.2_18.1_19	STRA6	AD	18.2	18.1	19	4.4E-06	0.116	-0.795	2.8E-01	4.1E-01	8.3E-01
LUAD	STX16_ES_7_6_8	STX16	ES	7	6	8	9.8E-07	0.129	-0.302	8.9E-01	4.7E-01	4.2E-01
LUAD	SUN2_ES_6_3_7	SUN2	ES	6	3	7	2.3E-05	0.119	-0.036	8.4E-01	7.4E-01	1.0E+00
LUAD	SWI5_AA_3.1_2_3.2	SWI5	AA	3.1	2	3.2	2.2E-07	0.143	-0.109	1.2E-01	2.1E-01	1.6E-01
LUAD	SYNE1_ES_151_150_152	SYNE1	ES	151	150	152	3.0E-06	0.136	-0.026	1.5E-01	7.1E-01	1.3E-01
LUAD	SYTL2_ES_11.2_10.3_12.2	SYTL2	ES	11.2	10.3	12.2	3.0E-07	0.148	-0.231	4.0E-01	5.0E-01	6.4E-01
LUAD	TACC2_ES_14_13_15.1	TACC2	ES	14	13	15.1	5.7E-07	0.136	0.049	9.6E-01	8.1E-01	4.6E-01

cancer type	id	Gene Symbol	splice_type	Exon	From.Exon	To.Exon	anova.p	adj.r2	r	p.50	p.25	p.10
LUAD	TANGO2_ES_7.2_6_8	TANGO2	ES	7.2	6	8	1.1E-10	0.207	0.067	4.5E-01	7.6E-01	4.9E-01
LUAD	TBC1D23_ES_15_14_16	TBC1D23	ES	15	14	16	1.4E-08	0.164	0.089	7.1E-01	9.6E-01	3.4E-01
LUAD	TCF12_ES_18_17_19	TCF12	ES	18	17	19	9.6E-12	0.226	0.225	2.0E-01	2.2E-01	6.1E-01
LUAD	TCOF1_ES_22_21_23	TCOF1	ES	22	21	23	2.6E-08	0.160	-0.215	7.3E-01	9.4E-01	5.8E-01
LUAD	TEAD2_ES_6_5_7.1	TEAD2	ES	6	5	7.1	2.9E-09	0.178	-0.047	8.2E-01	5.3E-01	5.1E-01
LUAD	TGFBR3_ES_5_4.2_6	TGFBR3	ES	5	4.2	6	7.4E-06	0.112	-0.616	5.3E-01	3.3E-01	7.0E-01
LUAD	THNSL2_ES_9_8_11	THNSL2	ES	9	8	11	3.6E-06	0.126	-0.077	1.3E-01	4.4E-01	5.1E-01
LUAD	TIGD6_AD_1.2_1.1_2	TIGD6	AD	1.2	1.1	2	2.0E-05	0.103	-0.238	8.0E-01	1.0E-01	2.2E-02
LUAD	TKT_RI_16.2_16.1_16.3	TKT	RI	16.2	16.1	16.3	7.3E-06	0.112	-0.032	1.0E+00	5.8E-01	6.4E-01
LUAD	TMEM159_AA_2.1:2.2_1_2.3	TMEM159	AA	2.1:2.2	1	2.3	1.4E-05	0.107	-0.197	8.7E-01	9.4E-01	6.0E-01
LUAD	TMEM180_ES_6_5_7	TMEM180	ES	6	5	7	2.4E-05	0.103	-0.196	8.6E-01	9.5E-01	7.4E-01
LUAD	TMUB2_ES_2.2:2.3:2.4:2.5:4.2_2.1_4.3	TMUB2	ES	2.2:2.3:2.4:2.5:4.[illegible]	2.1	4.3	2.6E-05	0.105	0.054	5.6E-01	3.2E-01	8.6E-01
LUAD	TMUB2_ES_4.7:4.8_4.5_5	TMUB2	ES	4.7:4.8	4.5	5	4.2E-05	0.115	-0.095	3.7E-02	1.5E-01	4.3E-01
LUAD	TMUB2_ES_4.8_4.5_5	TMUB2	ES	4.8	4.5	5	9.0E-05	0.104	-0.084	1.1E-02	3.4E-02	2.6E-01
LUAD	TNC_ES_12:13:14:15_11_16	TNC	ES	12:13:14:15	11	16	2.2E-05	0.105	-0.062	4.1E-01	1.5E-01	2.8E-02
LUAD	TNC_ES_12:13:14:15:16_11_19	TNC	ES	12:13:14:15:16	11	19	3.8E-07	0.140	-0.063	5.1E-03	7.9E-02	8.8E-02
LUAD	TNC_ES_12:13:14:15:16:18_11_19	TNC	ES	12:13:14:15:16:1[illegible]	11	19	8.3E-07	0.142	-0.045	2.6E-02	4.7E-02	9.1E-02
LUAD	TNC_ES_12:13:14:15:16:18:19_11_20	TNC	ES	[illegible]:13:14:15:16:18:[illegible]	11	20	2.5E-07	0.149	0.254	1.3E-03	5.5E-03	1.7E-01
LUAD	TNC_ES_12:13:14:15:16:19_11_20	TNC	ES	12:13:14:15:16:1[illegible]	11	20	3.3E-07	0.141	0.137	4.3E-02	1.4E-01	1.7E-01
LUAD	TNC_ES_12:13:15:16_11_19	TNC	ES	12:13:15:16	11	19	3.1E-05	0.123	-0.228	7.5E-01	2.4E-01	1.1E-01
LUAD	TNC_ES_12:13:15:16:18_11_19	TNC	ES	12:13:15:16:18	11	19	1.1E-04	0.118	-0.124	6.0E-01	1.8E-01	4.1E-01
LUAD	TNC_ES_12:13:15:16:18:19_11_20	TNC	ES	12:13:15:16:18:1[illegible]	11	20	5.0E-06	0.135	0.184	1.6E-02	3.5E-01	7.1E-01
LUAD	TPD52L1_ES_8_6_10	TPD52L1	ES	8	6	10	5.0E-06	0.122	0.101	8.0E-01	8.5E-01	7.9E-01
LUAD	TPD52L1_ES_8:9.1_6_10	TPD52L1	ES	08:09.1	6	10	1.2E-06	0.132	0.179	2.2E-01	2.0E-01	5.9E-01
LUAD	TPM2_ES_6_5_7	TPM2	ES	6	5	7	9.2E-05	0.116	-0.480	2.4E-02	2.1E-02	2.4E-01
LUAD	TRAPPC6A_AD_1.2_1.1_2	TRAPPC6A	AD	1.2	1.1	2	1.4E-05	0.109	-0.101	8.4E-01	5.0E-01	4.6E-01
LUAD	TRIM13_AD_1.2_1.1_1.4	TRIM13	AD	1.2	1.1	1.4	2.3E-05	0.106	-0.087	8.9E-01	3.0E-01	7.1E-01
LUAD	TRPM2_ES_29_28_30	TRPM2	ES	29	28	30	9.7E-10	0.208	-0.465	3.7E-02	1.1E-01	5.9E-01
LUAD	TSC2_ES_27_26_28.1	TSC2	ES	27	26	28.1	8.2E-07	0.131	0.007	6.2E-01	6.8E-01	8.7E-01
LUAD	TTC8_ES_10.1_9_11	TTC8	ES	10.1	9	11	1.9E-09	0.184	-0.083	2.7E-01	8.5E-01	1.3E-01
LUAD	TXNL4A_ES_4:7.2:8_3_9	TXNL4A	ES	07:02.2	3	9	1.0E-05	0.131	-0.210	6.5E-01	5.0E-01	2.2E-01
LUAD	TXNL4A_ES_7.2:8_3_9	TXNL4A	ES	7.2:8	3	9	4.1E-07	0.138	-0.205	6.3E-01	9.5E-01	7.3E-01
LUAD	UAP1_ES_9.2_8_10	UAP1	ES	9.2	8	10	5.1E-06	0.116	0.286	7.4E-01	9.6E-01	8.9E-01
LUAD	UBXN11_ES_3:4_2_5	UBXN11	ES	3:04	2	5	3.4E-05	0.124	0.016	6.9E-01	3.4E-01	2.6E-01
LUAD	WDR62_RI_25.6_25.5_25.7	WDR62	RI	25.6	25.5	25.7	2.9E-06	0.120	-0.471	1.2E-02	1.4E-03	2.0E-03
LUAD	WNK1_ES_15_13.2_16	WNK1	ES	15	13.2	16	7.4E-10	0.193	0.075	4.4E-01	4.4E-01	3.4E-01
LUAD	YDJC_ES_4_3_5	YDJC	ES	4	3	5	2.3E-05	0.106	-0.153	6.4E-01	5.5E-01	5.8E-01
LUAD	YY1AP1_AA_12.1_11_12.2	YY1AP1	AA	12.1	11	12.2	1.0E-07	0.147	0.122	1.9E-01	1.8E-01	8.0E-01
LUAD	ZC3H11A_AD_1.2_1.1_2.1	ZC3H11A	AD	1.2	1.1	2.1	3.8E-07	0.169	0.027	8.7E-01	6.0E-01	3.0E-01
LUAD	ZDHHC7_ES_4_3_5	ZDHHC7	ES	4	3	5	5.6E-06	0.115	0.040	4.0E-01	5.2E-02	2.1E-01

cancer type	id	Gene Symbol	splice_type	Exon	From.Exon	To.Exon	anova.p	adj.r2	r	p.50	p.25	p.10
LUAD	ZNF283_ES_2_1_3.2	ZNF283	ES	2	1	3.2	1.6E-04	0.109	0.008	1.9E-01	3.2E-01	1.2E-01
LUAD	ZNF384_ES_8_7_9	ZNF384	ES	8	7	9	1.1E-06	0.128	-0.183	6.7E-02	4.8E-01	2.1E-01
LUAD	ZNF670_ES_7_6_8	ZNF670	ES	7	6	8	4.8E-05	0.120	-0.227	3.0E-01	3.3E-01	4.3E-01
LUAD	ZRANB2_ES_10_9_11	ZRANB2	ES	10	9	11	2.0E-06	0.123	0.403	2.1E-01	2.8E-01	2.8E-01
LUSC	ABCC3_RI_16.2_16.1_16.3	ABCC3	RI	16.2	16.1	16.3	3.8E-08	0.191	-0.541	4.1E-01	1.1E-01	1.6E-01
LUSC	ABCC5_ES_7.4_7.1_7.6	ABCC5	ES	7.4	7.1	7.6	4.1E-05	0.111	-0.112	7.4E-01	3.5E-01	4.0E-01
LUSC	ABHD14B_AA_3.1:3.2_2.2_3.3	ABHD14B	AA	3.1:3.2	2.2	3.3	2.6E-05	0.114	-0.145	9.1E-02	6.6E-02	7.3E-01
LUSC	ABHD14B_ES_3.1:3.2:3.3_2.2_6.1	ABHD14B	ES	3.1:3.2:3.3	2.2	6.1	2.8E-06	0.146	0.106	8.2E-01	4.7E-01	9.2E-01
LUSC	ABI1_ES_11.1:11.2_9_12	ABI1	ES	11.1:11.2	9	12	1.5E-05	0.119	-0.294	4.0E-01	8.1E-01	4.0E-01
LUSC	ABI1_ES_11.1:11.2_9_13	ABI1	ES	11.1:11.2	9	13	1.4E-08	0.189	-0.289	4.5E-01	1.7E-01	5.9E-02
LUSC	ABI1_ES_11.2_9_12	ABI1	ES	11.2	9	12	6.1E-05	0.104	-0.277	5.3E-01	4.2E-01	1.4E-01
LUSC	ABI1_ES_11.2_9_13	ABI1	ES	11.2	9	13	2.7E-07	0.160	-0.274	7.0E-01	6.5E-01	1.0E-01
LUSC	ABI1_ES_5_4_7	ABI1	ES	5	4	7	9.0E-08	0.171	-0.223	3.0E-01	4.3E-01	5.6E-01
LUSC	ABI2_ES_15_14_16	ABI2	ES	15	14	16	2.0E-06	0.140	-0.048	1.0E+00	9.5E-01	9.3E-01
LUSC	ACHE_ES_4.1:4.2:4.3:4.4_3.5_5	ACHE	ES	4.1:4.2:4.3:4.4	3.5	5	3.6E-05	0.119	-0.056	3.1E-01	2.0E-01	9.7E-01
LUSC	ACSL1_ME_16|17_15_18.1	ACSL1	ME	16|17	15	18.1	2.8E-09	0.204	-0.196	1.1E-01	2.5E-02	1.4E-01
LUSC	ADAM15_ES_21.1:21.2_20_22.1	ADAM15	ES	21.1:21.2	20	22.1	4.4E-06	0.132	-0.191	3.2E-01	6.7E-02	2.5E-01
LUSC	ADAM15_ES_21.2_20_22.1	ADAM15	ES	21.2	20	22.1	6.6E-06	0.129	-0.097	4.2E-02	3.7E-02	4.1E-01
LUSC	AFMID_ES_11.1:11.2_10_12	AFMID	ES	11.1:11.2	10	12	8.7E-05	0.105	-0.094	6.6E-01	4.6E-01	7.6E-01
LUSC	AFMID_ES_5:6:7:8:9:10:11.1:11.2:12_2_	AFMID	ES	7:8:9:10:11.1:11.[illegible]	2	13	5.5E-06	0.131	-0.318	9.1E-01	6.1E-01	4.7E-01
LUSC	AP1B1_ES_24_23_25	AP1B1	ES	24	23	25	4.7E-15	0.320	-0.189	1.8E-01	1.8E-01	1.5E-01
LUSC	AP1G1_ES_12_10.1_13.2	AP1G1	ES	12	10.1	13.2	5.7E-06	0.133	-0.031	6.6E-01	8.2E-01	5.9E-01
LUSC	AP2M1_ES_7_6_8	AP2M1	ES	7	6	8	6.1E-06	0.128	-0.113	2.6E-01	3.8E-01	4.1E-01
LUSC	AP2S1_ES_3.2:4.1_1.1_4.2	AP2S1	ES	3.2:4.1	1.1	4.2	3.8E-07	0.156	-0.103	8.9E-01	6.4E-01	7.3E-01
LUSC	APH1A_ES_2.1:2.2_1_4	APH1A	ES	2.1:2.2	1	4	1.1E-04	0.101	-0.087	1.9E-01	4.4E-01	7.4E-01
LUSC	APH1A_ES_2.2_1_3	APH1A	ES	2.2	1	3	1.2E-06	0.145	-0.045	2.7E-01	8.0E-01	9.8E-02
LUSC	APH1A_ES_3_1_4	APH1A	ES	3	1	4	2.7E-05	0.113	0.002	3.9E-01	5.7E-01	4.8E-02
LUSC	APP_ES_10_9_11	APP	ES	10	9	11	1.1E-05	0.122	0.072	3.7E-01	4.3E-01	2.3E-01
LUSC	ARAP1_ES_32_31_33	ARAP1	ES	32	31	33	3.8E-13	0.282	0.040	6.2E-01	7.3E-01	7.3E-01
LUSC	ARFIP1_ES_4_3_5	ARFIP1	ES	4	3	5	4.8E-05	0.107	0.095	2.2E-01	1.7E-01	4.2E-01
LUSC	ARHGAP17_ES_18_17_19	ARHGAP17	ES	18	17	19	2.3E-06	0.138	0.175	9.2E-02	6.6E-02	5.2E-01
LUSC	ARHGEF11_ES_39_38_40	ARHGEF11	ES	39	38	40	2.5E-08	0.183	-0.176	1.5E-01	1.3E-01	7.3E-01
LUSC	ARMC8_ES_2.1:2.2:3_1_4	ARMC8	ES	2.1:2.2:3	1	4	4.5E-05	0.110	0.051	7.1E-01	9.4E-01	8.2E-01
LUSC	ARMC8_ES_2.2:3_1_4	ARMC8	ES	2.2:3	1	4	6.3E-05	0.104	-0.067	8.2E-01	4.7E-01	9.5E-01
LUSC	ATP5H_ES_5_3_6	ATP5H	ES	5	3	6	2.8E-07	0.169	-0.374	3.1E-01	3.2E-02	1.2E-01
LUSC	ATP5J_ES_1.4:1.5:2_1.3_3	ATP5J	ES	1.4:1.5:2	1.3	3	1.4E-06	0.143	0.060	5.0E-01	4.1E-01	6.9E-01
LUSC	ATXN2L_AA_22.4:22.5_22.1_22.6	ATXN2L	AA	22.4:22.5	22.1	22.6	1.3E-05	0.131	-0.109	6.2E-02	3.1E-02	4.8E-01
LUSC	AZI1_AD_12.2_12.1_13	AZI1	AD	12.2	12.1	13	1.6E-04	0.102	0.049	7.2E-01	8.4E-01	1.6E-01
LUSC	B4GALT2_AD_2.2_2.1_3	B4GALT2	AD	2.2	2.1	3	6.0E-07	0.153	-0.168	2.3E-01	3.2E-01	9.8E-02
LUSC	B4GALT3_AA_3.1_2.2_3.2	B4GALT3	AA	3.1	2.2	3.2	3.1E-07	0.159	-0.060	5.4E-01	2.7E-01	1.2E-01

cancer type	id	Gene Symbol	splice_type	Exon	From.Exon	To.Exon	anova.p	adj.r2	r	p.50	p.25	p.10
LUSC	B4GALT4_ES_6.2:7:8.1:9.1:9.2:9.3_6.1_9	B4GALT4	ES	.2:7:8.1:9.1:9.2:9	6.1	9.4	7.5E-06	0.127	-0.415	6.3E-01	6.7E-01	5.2E-01
LUSC	BABAM1_AA_2.1_1.2_2.2	BABAM1	AA	2.1	1.2	2.2	5.7E-08	0.175	0.000	1.7E-01	2.1E-01	4.7E-01
LUSC	BCAT2_ES_4:5_1_6	BCAT2	ES	4:05	1	6	1.1E-05	0.122	-0.005	7.0E-01	4.8E-01	2.2E-01
LUSC	BCL2L12_ES_3.1:3.2_2_4	BCL2L12	ES	3.1:3.2	2	4	1.4E-17	0.363	0.210	9.6E-01	1.9E-01	1.5E-01
LUSC	BCL2L12_ES_3.2_2_4	BCL2L12	ES	3.2	2	4	3.5E-08	0.180	0.163	4.3E-01	1.9E-01	4.7E-01
LUSC	BCS1L_ES_1.2:1.8_1.1_2	BCS1L	ES	1.2:1.8	1.1	2	1.0E-04	0.117	-0.034	7.2E-01	8.5E-01	5.9E-01
LUSC	BCS1L_ES_1.4_1.1_2	BCS1L	ES	1.4	1.1	2	3.0E-06	0.142	-0.055	5.3E-01	3.4E-01	1.0E+00
LUSC	BMP1_ES_17.1:17.2:18.1_16_18.2	BMP1	ES	17.1:17.2:18.1	16	18.2	8.8E-05	0.100	-0.071	1.3E-01	1.3E-01	2.8E-01
LUSC	C11orf49_RI_14.2:14.3_14.1_14.4	C11orf49	RI	14.2:14.3	14.1	14.4	5.6E-09	0.197	-0.064	1.3E-01	6.9E-01	6.0E-01
LUSC	C11orf57_AD_1.2:1.3_1.1_2.1	C11orf57	AD	1.2:1.3	1.1	2.1	2.3E-06	0.138	0.007	5.8E-01	8.0E-01	7.9E-01
LUSC	C14orf159_ES_4:5.2_1_7	C14orf159	ES	04:05.2	1	7	2.3E-05	0.117	-0.065	6.0E-01	2.4E-01	2.9E-01
LUSC	C16orf13_ES_2_1_3	C16orf13	ES	2	1	3	1.3E-05	0.120	-0.037	7.0E-02	2.1E-01	7.2E-01
LUSC	C16orf91_RI_4.2_4.1_4.3	C16orf91	RI	4.2	4.1	4.3	6.4E-06	0.128	0.053	6.3E-01	9.3E-01	5.6E-01
LUSC	C7orf49_AD_2.3:2.4_2.2_3	C7orf49	AD	2.3:2.4	2.2	3	1.2E-05	0.129	-0.290	7.7E-01	6.9E-01	3.8E-02
LUSC	CA12_ES_9_8_10	CA12	ES	9	8	10	9.0E-07	0.155	0.507	1.3E-01	3.3E-01	9.4E-01
LUSC	CA5B_ES_4_3_5	CA5B	ES	4	3	5	3.5E-05	0.110	0.556	9.4E-01	6.9E-01	2.9E-01
LUSC	CALU_ES_2:3:5_1_6	CALU	ES	2:03:05	1	6	4.2E-05	0.110	-0.087	6.9E-01	6.0E-01	5.7E-01
LUSC	CAMK2D_AD_21.2_21.1_23	CAMK2D	AD	21.2	21.1	23	4.9E-05	0.109	-0.118	6.0E-01	2.5E-01	8.4E-01
LUSC	CASC4_ES_10_9_11	CASC4	ES	10	9	11	2.4E-06	0.138	-0.147	2.4E-02	4.1E-01	6.5E-01
LUSC	CAST_ES_10_9_11.2	CAST	ES	10	9	11.2	5.6E-09	0.197	0.232	8.8E-01	7.8E-01	1.3E-01
LUSC	CAST_ES_7.1:8.2_5.2_9	CAST	ES	7.1:8.2	5.2	9	7.5E-08	0.185	-0.117	9.2E-03	2.6E-01	7.4E-01
LUSC	CAST_ES_8.2:9_7.1_10	CAST	ES	8.2:9	7.1	10	1.3E-04	0.104	-0.134	1.0E+00	4.4E-01	1.3E-01
LUSC	CC2D2A_ES_5_4_6	CC2D2A	ES	5	4	6	5.1E-08	0.180	-0.129	2.6E-01	1.2E-01	7.8E-01
LUSC	CCBL1_ES_2:3_1_4	CCBL1	ES	2:03	1	4	1.6E-04	0.114	0.145	1.7E-01	1.4E-01	7.3E-02
LUSC	CCDC107_RI_3.4_3.3_3.5	CCDC107	RI	3.4	3.3	3.5	9.5E-08	0.170	-0.259	8.3E-01	8.2E-01	7.5E-01
LUSC	CCDC88A_ES_26_25_27	CCDC88A	ES	26	25	27	1.9E-05	0.118	-0.111	6.5E-01	6.7E-01	4.2E-02
LUSC	CCNL2_ES_6.1_5_7.2	CCNL2	ES	6.1	5	7.2	1.6E-04	0.107	0.344	4.9E-01	7.9E-01	5.5E-01
LUSC	CD3EAP_AD_1.2_1.1_2	CD3EAP	AD	1.2	1.1	2	5.5E-05	0.112	0.051	5.7E-01	5.4E-01	8.7E-01
LUSC	CD44_ES_10:11_5_12.1	CD44	ES	10:11	5	12.1	3.6E-06	0.136	0.125	4.2E-01	6.6E-01	6.5E-01
LUSC	CD44_ES_10:11:12.1:13:14_5_15	CD44	ES	10:11:12.1:13:14	5	15	1.2E-06	0.148	0.406	7.5E-01	2.3E-01	8.5E-01
LUSC	CD44_ES_12.1:13_5_14	CD44	ES	12.1:13	5	14	1.4E-05	0.119	0.272	3.9E-02	3.5E-01	7.2E-01
LUSC	CD44_ES_12.1:13:14_5_15	CD44	ES	12.1:13:14	5	15	2.5E-08	0.183	0.277	9.2E-02	4.3E-01	3.7E-01
LUSC	CD44_ES_3.1:3.2:4:5:12.1:13:14:15:16.1	CD44	ES	12.1:13:14:15:16.	2.1	17.2	6.0E-07	0.152	-0.057	4.7E-01	4.5E-01	9.6E-01
LUSC	CD44_ES_3.1:3.2:4:5:6:7:8:9.1:9.2:10:11	CD44	ES	).2:10:11:12.1:13	2.1	17.2	4.4E-10	0.224	0.286	7.7E-01	3.3E-01	2.2E-01
LUSC	CD44_ES_3.1:3.2:4:5:6:7:8:9.2:10:11:12.	CD44	ES	2:10:11:12.1:13:1	2.1	17.2	1.5E-11	0.255	0.284	3.5E-01	7.7E-02	3.7E-01
LUSC	CD44_ES_3.1:3.2:4:5:7:8:9.2:10:11:12.1:	CD44	ES	:10:11:12.1:13:14	2.1	17.2	1.2E-05	0.122	0.311	1.5E-02	3.3E-02	4.4E-01
LUSC	CD44_ES_6:7:8:9.1:9.2_5_10	CD44	ES	6:7:8:9.1:9.2	5	10	2.9E-08	0.187	0.241	9.4E-01	7.5E-01	6.1E-01
LUSC	CD44_ES_6:7:8:9.1:9.2:10:11_5_12.1	CD44	ES	5:7:8:9.1:9.2:10:1	5	12.1	1.3E-09	0.214	0.263	5.5E-01	7.2E-01	6.4E-01
LUSC	CD44_ES_6:7:8:9.1:9.2:10:11:12.1:13_5_	CD44	ES	:9.1:9.2:10:11:12	5	14	6.8E-09	0.198	0.354	7.0E-01	5.7E-01	4.5E-01
LUSC	CD44_ES_6:7:8:9.1:9.2:10:11:12.1:13:14	CD44	ES	).1:9.2:10:11:12.1	5	15	1.4E-12	0.271	0.437	5.5E-01	2.4E-01	8.3E-01

cancer type	id	Gene Symbol	splice_type	Exon	From.Exon	To.Exon	anova.p	adj.r2	r	p.50	p.25	p.10
LUSC	CD44_ES_6:7:8:9.2_5_10	CD44	ES	6:7:8:9.2	5	10	2.0E-09	0.211	0.240	8.0E-01	5.5E-01	5.6E-01
LUSC	CD44_ES_6:7:8:9.2:10:11_5_12.1	CD44	ES	6:7:8:9.2:10:11	5	12.1	1.0E-10	0.238	0.253	6.3E-01	7.5E-01	5.2E-01
LUSC	CD44_ES_6:7:8:9.2:10:11:12.1:13_5_14	CD44	ES	:8:9.2:10:11:12.1	5	14	2.5E-10	0.230	0.372	5.8E-01	9.7E-01	4.0E-01
LUSC	CD44_ES_6:7:8:9.2:10:11:12.1:13:14_5_	CD44	ES	:9.2:10:11:12.1:1	5	15	1.3E-12	0.272	0.429	2.1E-01	2.7E-01	9.4E-01
LUSC	CD44_ES_7:8:9.1:9.2_5_10	CD44	ES	7:8:9.1:9.2	5	10	1.2E-05	0.125	0.341	8.5E-01	4.2E-01	6.0E-01
LUSC	CD44_ES_7:8:9.1:9.2:10:11_5_12.1	CD44	ES	7:8:9.1:9.2:10:11	5	12.1	5.2E-07	0.155	0.335	8.9E-01	7.4E-01	3.6E-01
LUSC	CD44_ES_7:8:9.1:9.2:10:11:12.1:13:14_5	CD44	ES	1:9.2:10:11:12.1:	5	15	9.4E-14	0.294	0.549	2.5E-01	6.0E-02	4.4E-01
LUSC	CD44_ES_7:8:9.2_5_10	CD44	ES	08:09.2	5	10	1.6E-06	0.146	0.330	9.9E-01	3.3E-01	4.5E-01
LUSC	CD44_ES_7:8:9.2:10:11_5_12.1	CD44	ES	7:8:9.2:10:11	5	12.1	4.2E-08	0.180	0.322	9.3E-01	5.6E-01	3.9E-01
LUSC	CD44_ES_7:8:9.2:10:11:12.1:13_5_14	CD44	ES	8:9.2:10:11:12.1:	5	14	7.8E-06	0.127	0.422	5.3E-01	1.1E-01	7.2E-01
LUSC	CD44_ES_7:8:9.2:10:11:12.1:13:14_5_15	CD44	ES	9.2:10:11:12.1:13	5	15	1.2E-13	0.292	0.542	2.1E-01	6.1E-02	5.9E-01
LUSC	CD44_ES_8:9.1:9.2_5_10	CD44	ES	8:9.1:9.2	5	10	1.8E-05	0.122	0.147	7.5E-01	5.5E-01	7.4E-01
LUSC	CD44_ES_8:9.1:9.2:10:11_5_12.1	CD44	ES	8:9.1:9.2:10:11	5	12.1	7.3E-10	0.225	0.136	6.5E-01	6.6E-01	3.4E-01
LUSC	CD44_ES_8:9.1:9.2:10:11:12.1:13:14_5_	CD44	ES	:9.2:10:11:12.1:1	5	15	3.9E-12	0.272	0.410	1.3E-01	1.3E-01	4.3E-02
LUSC	CD44_ES_8:9.2_5_10	CD44	ES	08:09.2	5	10	4.8E-06	0.137	0.155	7.9E-01	4.0E-01	8.0E-02
LUSC	CD44_ES_8:9.2:10:11_5_12.1	CD44	ES	8:9.2:10:11	5	12.1	2.3E-10	0.235	0.144	3.8E-01	8.9E-01	1.1E-01
LUSC	CD44_ES_8:9.2:10:11:12.1:13:14_5_15	CD44	ES	.2:10:11:12.1:13	5	15	1.1E-11	0.262	0.415	5.8E-02	1.6E-01	4.1E-01
LUSC	CD46_ES_7:8_6_9	CD46	ES	7:08	6	9	1.1E-06	0.146	-0.135	8.2E-01	3.2E-01	8.6E-01
LUSC	CD46_ES_7:8:9_6_10	CD46	ES	7:08:09	6	10	1.3E-09	0.212	-0.168	7.4E-01	7.0E-01	8.9E-01
LUSC	CD46_ES_8_6_9	CD46	ES	8	6	9	2.0E-08	0.185	-0.125	9.4E-01	7.2E-01	5.1E-03
LUSC	CDC16_RI_18.2_18.1_18.3	CDC16	RI	18.2	18.1	18.3	2.6E-05	0.113	0.168	4.6E-02	5.5E-02	8.3E-01
LUSC	CEP70_AA_3.1_2_3.2	CEP70	AA	3.1	2	3.2	6.1E-05	0.104	0.106	2.6E-01	2.8E-01	9.2E-01
LUSC	CFLAR_ES_10_8.1_11	CFLAR	ES	10	8.1	11	4.9E-06	0.131	-0.569	5.7E-02	1.4E-01	1.7E-01
LUSC	CGREF1_ES_2.2_1.1_3.2	CGREF1	ES	2.2	1.1	3.2	1.8E-05	0.135	-0.026	2.3E-01	1.8E-01	7.3E-01
LUSC	CLASP1_ES_22_21_24	CLASP1	ES	22	21	24	1.7E-10	0.239	0.139	2.8E-01	9.9E-01	1.4E-01
LUSC	CLEC1A_ES_2_1_3	CLEC1A	ES	2	1	3	2.6E-08	0.246	-0.700	3.7E-01	8.7E-01	2.9E-01
LUSC	CLINT1_AA_11.1_10_11.2	CLINT1	AA	11.1	10	11.2	9.5E-06	0.124	0.026	5.0E-01	8.5E-01	7.1E-01
LUSC	COA1_ME_2|3.1:3.2_1_4.2	COA1	ME	2|3.1:3.2	1	4.2	2.1E-05	0.115	0.003	9.6E-01	6.9E-01	8.3E-01
LUSC	COASY_AA_1.3:1.4:1.5_1.1_1.6	COASY	AA	1.3:1.4:1.5	1.1	1.6	1.1E-04	0.107	-0.074	9.9E-01	7.8E-01	2.5E-01
LUSC	COX4I1_AA_5.3:5.4_5.1_5.5	COX4I1	AA	5.3:5.4	5.1	5.5	1.1E-06	0.158	-0.043	2.1E-01	4.3E-01	7.2E-01
LUSC	COX4I1_ES_5.1:5.3_4.1_5.4	COX4I1	ES	5.1:5.3	4.1	5.4	1.6E-05	0.118	0.059	3.3E-01	4.3E-01	5.4E-01
LUSC	COX4I1_RI_5.2_5.1_5.3	COX4I1	RI	5.2	5.1	5.3	5.9E-06	0.129	-0.278	4.1E-01	2.9E-01	2.0E-01
LUSC	CPNE1_ES_1.2:2.1:2.2:3_1.1_5	CPNE1	ES	1.2:2.1:2.2:3	1.1	5	8.7E-05	0.100	-0.724	8.3E-02	8.7E-02	5.3E-02
LUSC	CPNE1_ES_1.2:2.2:3_1.1_5	CPNE1	ES	1.2:2.2:3	1.1	5	4.9E-05	0.106	-0.510	3.0E-01	2.4E-01	3.0E-02
LUSC	CPNE1_ES_2.2:3_1.2_5	CPNE1	ES	2.2:3	1.2	5	5.5E-05	0.105	-0.632	7.1E-01	3.1E-01	1.8E-01
LUSC	CSF2RA_ES_13_12_14	CSF2RA	ES	13	12	14	2.2E-06	0.138	-0.776	4.7E-02	3.1E-01	3.4E-01
LUSC	CTNNB1_AA_18.3_18.1_18.4	CTNNB1	AA	18.3	18.1	18.4	2.1E-05	0.115	-0.056	3.4E-01	6.7E-01	5.0E-01
LUSC	CTNNB1_RI_18.2_18.1_18.3	CTNNB1	RI	18.2	18.1	18.3	1.5E-07	0.166	-0.083	2.8E-01	5.5E-01	5.0E-01
LUSC	CTNND1_ES_3:4.1:4.2:4.3_2.1_5	CTNND1	ES	3:4.1:4.2:4.3	2.1	5	5.9E-07	0.153	-0.279	2.8E-01	5.8E-02	6.5E-01
LUSC	CTNND1_ES_3:4.1:4.2:4.3:5_2.1_6	CTNND1	ES	3:4.1:4.2:4.3:5	2.1	6	6.8E-05	0.106	-0.178	9.8E-01	2.5E-02	6.6E-01

cancer type	id	Gene Symbol	splice_type	Exon	From.Exon	To.Exon	anova.p	adj.r2	r	p.50	p.25	p.10
LUSC	CTTN_ES_11_9_13	CTTN	ES	11	9	13	4.0E-04	0.121	-0.030	7.8E-01	6.7E-01	3.5E-01
LUSC	CUTC_ES_7_6_8	CUTC	ES	7	6	8	1.2E-05	0.121	0.120	6.8E-01	2.1E-01	8.0E-01
LUSC	CYB561A3_AA_6.6_6.4_6.7	CYB561A3	AA	6.6	6.4	6.7	2.9E-07	0.159	0.139	8.6E-01	3.7E-01	9.2E-01
LUSC	CYTH1_ES_11.2:13.1_11.1_13.2	CYTH1	ES	11.2:13.1	11.1	13.2	1.8E-06	0.142	-0.004	3.8E-01	2.5E-03	4.2E-02
LUSC	CYTH1_ES_12_11.1_13.2	CYTH1	ES	12	11.1	13.2	1.3E-07	0.168	0.015	2.5E-01	1.2E-02	1.7E-01
LUSC	DAPK2_RI_13.2_13.1_13.3	DAPK2	RI	13.2	13.1	13.3	8.6E-04	0.106	-0.082	6.5E-01	9.8E-01	5.4E-01
LUSC	DCTD_ES_1.2:2.1:2.2_1.1_5	DCTD	ES	1.2:2.1:2.2	1.1	5	5.6E-06	0.131	0.059	4.8E-01	8.7E-01	9.5E-01
LUSC	DGUOK_ES_4:5:6_1_7	DGUOK	ES	4:05:06	1	7	6.2E-05	0.104	0.105	7.6E-01	8.4E-01	4.6E-01
LUSC	DGUOK_ES_5:6_4_7	DGUOK	ES	5:06	4	7	4.7E-08	0.177	0.082	7.8E-02	6.5E-01	5.4E-01
LUSC	DIAPH1_ES_2_1_3	DIAPH1	ES	2	1	3	6.4E-06	0.136	-0.104	3.1E-01	7.1E-01	4.5E-01
LUSC	DMKN_AD_20.2_20.1_21	DMKN	AD	20.2	20.1	21	8.8E-05	0.110	-0.303	8.5E-01	7.4E-01	6.1E-01
LUSC	DMKN_AD_20.2_20.1_22	DMKN	AD	20.2	20.1	22	2.1E-07	0.166	-0.076	3.9E-01	7.1E-01	8.3E-01
LUSC	DMKN_ES_7:11:12_6.4_13	DMKN	ES	7:11:12	6.4	13	9.8E-06	0.138	0.076	6.3E-01	4.1E-01	8.8E-01
LUSC	DMKN_ES_7:8:11:12_6.4_13	DMKN	ES	7:8:11:12	6.4	13	2.7E-04	0.107	0.020	6.3E-02	4.5E-01	8.8E-01
LUSC	DMKN_ES_8:11:12_7_13	DMKN	ES	8:11:12	7	13	2.3E-04	0.115	-0.035	5.3E-01	7.7E-01	1.2E-01
LUSC	DMTN_ES_18.2_16_19	DMTN	ES	18.2	16	19	4.1E-06	0.134	-0.080	8.9E-01	3.2E-02	1.4E-01
LUSC	DNAJB12_RI_9.2:9.3_9.1_9.4	DNAJB12	RI	9.2:9.3	9.1	9.4	1.8E-05	0.117	-0.029	5.2E-01	8.4E-01	4.2E-01
LUSC	DNAJB12_RI_9.3_9.2_9.4	DNAJB12	RI	9.3	9.2	9.4	2.1E-05	0.115	-0.073	6.7E-01	8.5E-01	7.1E-01
LUSC	DNM1L_ES_17_16_18	DNM1L	ES	17	16	18	8.0E-06	0.125	-0.170	1.6E-01	2.5E-01	3.1E-01
LUSC	DNM1L_ES_17:18_16_19	DNM1L	ES	17:18	16	19	5.0E-05	0.106	-0.149	3.5E-01	1.2E-01	2.5E-01
LUSC	DST_ES_104_103_105	DST	ES	104	103	105	1.3E-06	0.144	0.245	1.4E-01	7.7E-02	9.5E-01
LUSC	DTD2_AD_1.2_1.1_2	DTD2	AD	1.2	1.1	2	7.1E-05	0.102	0.365	6.3E-01	8.7E-01	6.8E-01
LUSC	DTNBP1_ES_2_1_3	DTNBP1	ES	2	1	3	4.6E-05	0.107	0.051	7.8E-01	3.6E-01	1.0E+00
LUSC	DUOXA2_AA_2.1_1_2.2	DUOXA2	AA	2.1	1	2.2	3.1E-07	0.212	-0.632	8.6E-01	8.5E-01	5.3E-01
LUSC	DUSP22_RI_7.4_7.3_7.5	DUSP22	RI	7.4	7.3	7.5	1.8E-07	0.164	-0.225	8.6E-01	7.4E-01	9.5E-01
LUSC	ECHDC2_ES_5.1_2.1_6.2	ECHDC2	ES	5.1	2.1	6.2	1.4E-06	0.151	-0.143	1.5E-03	3.2E-03	1.1E-01
LUSC	ECHDC2_ES_5.1:6.1_2.1_6.2	ECHDC2	ES	5.1:6.1	2.1	6.2	4.1E-10	0.224	-0.043	1.0E-03	6.0E-02	8.8E-01
LUSC	ECT2_ES_2_1_3.2	ECT2	ES	2	1	3.2	3.0E-06	0.135	-0.530	8.6E-01	6.3E-01	5.1E-01
LUSC	ENTHD2_ES_8_7_9	ENTHD2	ES	8	7	9	7.8E-07	0.151	-0.020	9.1E-01	8.3E-01	1.0E+00
LUSC	EPB41_ES_16:18_15_19.1	EPB41	ES	16:18	15	19.1	1.1E-08	0.212	-0.022	9.1E-01	7.6E-01	4.1E-01
LUSC	EPB41_ES_18_15_19.1	EPB41	ES	18	15	19.1	4.2E-09	0.210	0.053	9.4E-01	5.2E-01	4.4E-01
LUSC	EPHB2_RI_17.2_17.1_17.3	EPHB2	RI	17.2	17.1	17.3	5.6E-05	0.105	-0.749	8.4E-02	1.8E-01	8.7E-01
LUSC	ERLIN2_RI_7.2_7.1_7.3	ERLIN2	RI	7.2	7.1	7.3	3.2E-05	0.131	0.031	1.5E-01	2.7E-01	6.4E-01
LUSC	ESRP1_AD_12.2_12.1_13	ESRP1	AD	12.2	12.1	13	2.9E-05	0.112	-0.342	4.5E-01	4.7E-01	9.1E-01
LUSC	EVI5L_ES_12_11_13	EVI5L	ES	12	11	13	3.7E-06	0.136	-0.310	1.7E-02	7.7E-03	2.3E-02
LUSC	EXOC7_ES_7_6_8.2	EXOC7	ES	7	6	8.2	9.9E-07	0.148	0.073	2.4E-02	1.2E-01	8.3E-02
LUSC	EXOC7_ES_7_6_9	EXOC7	ES	7	6	9	2.1E-06	0.139	0.184	5.8E-01	9.3E-01	4.7E-01
LUSC	EXOC7_ES_7:8.2_6_9	EXOC7	ES	07:08.2	6	9	1.3E-09	0.211	0.100	1.7E-01	4.7E-01	9.0E-01
LUSC	EXOSC8_ES_2_1_3	EXOSC8	ES	2	1	3	2.5E-07	0.161	-0.002	8.4E-01	6.6E-01	3.3E-01
LUSC	FAM122B_AA_10.1_9_10.2	FAM122B	AA	10.1	9	10.2	7.8E-07	0.149	-0.130	2.0E-01	3.0E-02	1.9E-01

cancer type	id	Gene Symbol	splice_type	Exon	From.Exon	To.Exon	anova.p	adj.r2	r	p.50	p.25	p.10
LUSC	FAM173A_RI_4.2_4.1_4.3	FAM173A	RI	4.2	4.1	4.3	6.8E-05	0.103	-0.063	7.0E-01	3.8E-01	5.4E-01
LUSC	FBLN5_ES_7_5_8	FBLN5	ES	7	5	8	4.4E-05	0.108	-0.657	2.1E-01	4.9E-01	1.9E-01
LUSC	FBXO44_ES_5.2:6_5.1_7	FBXO44	ES	5.2:6	5.1	7	2.7E-06	0.137	0.092	5.8E-01	4.5E-01	9.2E-01
LUSC	FDPS_ES_1.2:3.1_1.1_3.2	FDPS	ES	1.2:3.1	1.1	3.2	7.4E-08	0.173	0.236	6.5E-01	7.3E-01	7.5E-01
LUSC	FHL2_ES_4_3.2_5.1	FHL2	ES	4	3.2	5.1	5.3E-06	0.130	-0.753	5.1E-01	1.5E-01	1.6E-01
LUSC	FLOT2_ES_3_2_6	FLOT2	ES	3	2	6	9.3E-05	0.108	0.087	6.7E-02	4.7E-01	7.3E-01
LUSC	FNIP1_ES_7_6_8	FNIP1	ES	7	6	8	5.9E-08	0.192	-0.044	6.3E-01	9.9E-01	9.2E-01
LUSC	G6PC3_ES_5_4_6	G6PC3	ES	5	4	6	1.7E-08	0.189	0.056	6.5E-02	1.4E-01	8.2E-02
LUSC	GINS3_ES_2_1_3	GINS3	ES	2	1	3	6.9E-05	0.108	0.116	6.8E-01	2.8E-01	4.8E-01
LUSC	GIT2_ES_19_18.2_20	GIT2	ES	19	18.2	20	2.4E-05	0.114	-0.061	9.7E-01	7.7E-01	4.9E-01
LUSC	GK_ES_23_22_24	GK	ES	23	22	24	1.5E-05	0.119	-0.212	2.3E-01	2.9E-01	6.2E-01
LUSC	GPATCH8_ES_6_5_7	GPATCH8	ES	6	5	7	9.9E-05	0.128	0.267	7.9E-01	5.7E-01	5.6E-02
LUSC	GPR137_ES_6_5_8	GPR137	ES	6	5	8	1.4E-05	0.123	0.153	1.8E-01	6.8E-01	4.2E-01
LUSC	GPR63_ES_2_1_3	GPR63	ES	2	1	3	8.1E-07	0.160	-0.529	2.4E-01	3.6E-01	2.7E-01
LUSC	GRB7_ES_16_15_17	GRB7	ES	16	15	17	3.3E-05	0.110	0.001	5.3E-02	2.1E-01	4.8E-01
LUSC	GSTM5_RI_5.2_5.1_5.3	GSTM5	RI	5.2	5.1	5.3	1.1E-04	0.112	-0.398	9.9E-02	2.5E-02	5.3E-01
LUSC	H2AFY_ME_7|8_6.3_9	H2AFY	ME	7|8	6.3	9	1.4E-06	0.143	0.177	4.5E-03	3.7E-02	7.6E-01
LUSC	HAUS7_ES_12_11.2_13	HAUS7	ES	12	11.2	13	1.3E-07	0.167	-0.018	7.3E-01	9.3E-01	9.4E-01
LUSC	HCFC1R1_AA_3.1_1.5_3.2	HCFC1R1	AA	3.1	1.5	3.2	1.2E-05	0.122	0.185	3.9E-01	9.0E-01	7.4E-01
LUSC	HMBS_AD_12.2:12.3_12.1_13	HMBS	AD	12.2:12.3	12.1	13	2.1E-05	0.116	-0.359	6.8E-01	6.1E-01	8.4E-01
LUSC	HNRNPC_ES_2.4:2.5:2.6_1_3.2	HNRNPC	ES	2.4:2.5:2.6	1	3.2	7.4E-05	0.102	-0.409	9.4E-01	8.5E-01	9.3E-01
LUSC	HSBP1L1_ES_2.2:3.1_2.1_3.2	HSBP1L1	ES	2.2:3.1	2.1	3.2	3.4E-05	0.111	-0.310	6.1E-01	3.2E-01	9.7E-01
LUSC	HTATIP2_RI_1.2:1.3:1.4_1.1_1.5	HTATIP2	RI	1.2:1.3:1.4	1.1	1.5	5.3E-08	0.176	0.375	9.4E-01	4.3E-01	9.7E-01
LUSC	HTATIP2_RI_1.4_1.3_1.5	HTATIP2	RI	1.4	1.3	1.5	4.7E-07	0.154	0.278	5.0E-01	6.3E-01	5.9E-01
LUSC	ICAM3_RI_3.2_3.1_3.3	ICAM3	RI	3.2	3.1	3.3	2.3E-09	0.207	-0.737	2.0E-01	2.3E-01	1.6E-01
LUSC	IFI44_ES_8_6_9	IFI44	ES	8	6	9	1.5E-04	0.122	-0.220	8.4E-01	5.1E-01	2.9E-01
LUSC	IL18BP_RI_1.2_1.1_1.3	IL18BP	RI	1.2	1.1	1.3	2.8E-04	0.110	-0.369	3.1E-01	3.9E-01	3.5E-01
LUSC	ING4_ES_3:4_2_5.1	ING4	ES	3:04	2	5.1	9.2E-06	0.125	-0.019	2.4E-01	4.7E-01	2.4E-01
LUSC	INO80C_ES_3:4.1:4.2_1_5.1	INO80C	ES	3:4.1:4.2	1	5.1	7.8E-05	0.101	-0.539	5.8E-01	2.8E-01	2.5E-01
LUSC	IPO11_ES_30_29_33	IPO11	ES	30	29	33	4.4E-07	0.156	-0.042	2.3E-01	1.2E-01	5.0E-01
LUSC	ITGA6_ES_27_26_28	ITGA6	ES	27	26	28	2.5E-05	0.114	0.548	7.5E-01	9.2E-01	2.6E-01
LUSC	ITGAE_ES_28_27_29	ITGAE	ES	28	27	29	5.5E-07	0.153	0.255	8.1E-01	6.3E-01	8.1E-01
LUSC	IVNS1ABP_ES_9_8_10	IVNS1ABP	ES	9	8	10	6.9E-05	0.103	-0.111	1.4E-01	3.8E-01	8.2E-01
LUSC	KAT5_ES_4_3_5	KAT5	ES	4	3	5	3.6E-06	0.134	0.048	4.7E-01	2.0E-01	4.3E-01
LUSC	KCTD17_ES_7_6_8	KCTD17	ES	7	6	8	8.3E-06	0.127	-0.091	2.1E-01	7.9E-01	7.9E-01
LUSC	KIF23_ES_21_20_22.1	KIF23	ES	21	20	22.1	5.3E-06	0.130	0.051	4.1E-01	2.2E-01	7.1E-02
LUSC	KRT15_RI_7.2_7.1_7.3	KRT15	RI	7.2	7.1	7.3	2.3E-07	0.161	-0.722	1.0E+00	8.5E-02	2.4E-01
LUSC	LAS1L_ES_2_1_3	LAS1L	ES	2	1	3	2.4E-06	0.138	0.045	7.2E-01	8.7E-01	8.3E-01
LUSC	LCLAT1_ES_3_2_4	LCLAT1	ES	3	2	4	8.2E-05	0.116	-0.383	8.8E-01	4.7E-01	9.8E-01
LUSC	LEF1_ES_12_11_13	LEF1	ES	12	11	13	2.2E-07	0.165	-0.286	5.1E-01	6.6E-02	5.8E-02

cancer type	id	Gene Symbol	splice_type	Exon	From.Exon	To.Exon	anova.p	adj.r2	r	p.50	p.25	p.10
LUSC	LLGL2_ES_26_25_27	LLGL2	ES	26	25	27	7.2E-07	0.152	-0.029	9.7E-01	9.0E-01	5.5E-01
LUSC	LPIN1_ES_10_9_12	LPIN1	ES	10	9	12	4.1E-09	0.236	-0.097	8.3E-02	4.5E-01	1.9E-01
LUSC	LRCH3_ES_15_14_16	LRCH3	ES	15	14	16	6.3E-06	0.128	0.010	3.6E-02	1.3E-01	5.1E-01
LUSC	LTB4R2_RI_1.2:1.3_1.1_1.4	LTB4R2	RI	1.2:1.3	1.1	1.4	1.0E-08	0.198	-0.581	2.7E-01	6.4E-01	4.1E-01
LUSC	LTBP3_ES_25_24_26	LTBP3	ES	25	24	26	1.6E-09	0.210	0.429	1.4E-01	5.1E-02	8.7E-02
LUSC	LTBP4_ES_29_28_31	LTBP4	ES	29	28	31	1.3E-05	0.135	0.096	9.1E-03	2.1E-01	1.3E-01
LUSC	LY6K_RI_2.2_2.1_2.3	LY6K	RI	2.2	2.1	2.3	1.4E-06	0.143	-0.487	5.2E-01	5.0E-01	5.2E-01
LUSC	MACF1_ES_107_106_108	MACF1	ES	107	106	108	1.1E-05	0.122	-0.068	6.4E-01	8.9E-01	8.5E-02
LUSC	MAD2L2_ES_2:3_1_4	MAD2L2	ES	2:03	1	4	3.9E-05	0.109	-0.348	4.5E-01	4.0E-01	6.2E-01
LUSC	MAP2_ES_13_12_14	MAP2	ES	13	12	14	1.7E-04	0.115	-0.226	5.8E-01	4.4E-01	8.9E-01
LUSC	MAP2_ES_16_15_17	MAP2	ES	16	15	17	8.6E-07	0.174	-0.250	9.8E-01	5.8E-01	2.8E-01
LUSC	MAP4K1_RI_24.2_24.1_24.3	MAP4K1	RI	24.2	24.1	24.3	5.8E-06	0.143	-0.183	4.2E-01	1.6E-01	8.4E-02
LUSC	MBNL1_ES_8_7_9	MBNL1	ES	8	7	9	7.7E-06	0.127	-0.374	5.3E-01	9.2E-01	8.0E-01
LUSC	ME3_ES_10_8_11	ME3	ES	10	8	11	2.5E-04	0.103	0.043	1.3E-01	5.9E-01	7.2E-01
LUSC	MEFV_ES_6_5_7	MEFV	ES	6	5	7	1.3E-05	0.122	-0.602	3.5E-01	1.7E-01	1.8E-01
LUSC	MFF_ES_3:4_1_5	MFF	ES	3:04	1	5	1.2E-05	0.122	0.040	4.4E-01	6.8E-01	6.2E-01
LUSC	MFF_ES_3:4:5_1_6	MFF	ES	3:04:05	1	6	1.4E-06	0.143	0.090	4.6E-01	5.6E-01	5.5E-01
LUSC	MFF_ES_8:9_7_10	MFF	ES	8:09	7	10	6.0E-05	0.104	-0.053	1.1E-01	8.3E-02	3.3E-01
LUSC	MFSD10_RI_12.2_12.1_12.3	MFSD10	RI	12.2	12.1	12.3	1.7E-05	0.118	-0.174	2.4E-02	3.7E-01	5.9E-01
LUSC	MLLT4_ES_16_15_17	MLLT4	ES	16	15	17	3.7E-05	0.119	-0.042	9.2E-02	3.5E-01	6.5E-01
LUSC	MPRIP_ES_24_23_25	MPRIP	ES	24	23	25	1.8E-06	0.141	0.065	3.4E-02	6.1E-01	2.0E-01
LUSC	MRPL2_ES_3:4:5_2.1_7	MRPL2	ES	3:04:05	2.1	7	5.6E-05	0.105	0.236	8.3E-01	8.9E-01	7.3E-01
LUSC	MRPL52_ES_3_2_5	MRPL52	ES	3	2	5	4.2E-05	0.114	-0.097	7.9E-01	9.2E-01	3.7E-01
LUSC	MRPL52_ES_3:4.1_2_5	MRPL52	ES	03:04.1	2	5	3.5E-06	0.134	-0.130	5.3E-01	3.5E-01	3.0E-01
LUSC	MRPL52_RI_1.2:1.3:1.4_1.1_1.5	MRPL52	RI	1.2:1.3:1.4	1.1	1.5	2.0E-06	0.140	-0.049	8.0E-01	9.2E-01	4.7E-01
LUSC	MRPL55_ES_1.2:2.2_1.1_2.9	MRPL55	ES	1.2:2.2	1.1	2.9	3.4E-06	0.135	-0.008	8.0E-01	7.2E-01	9.9E-01
LUSC	MRPL55_ES_1.2:2.2:2.5:2.6_1.1_2.9	MRPL55	ES	1.2:2.2:2.5:2.6	1.1	2.9	6.7E-09	0.199	-0.050	2.4E-01	9.3E-01	5.0E-01
LUSC	MRPL55_ES_1.2:2.2:2.5:2.6:2.7:2.8_1.1_	MRPL55	ES	2:2.2:2.5:2.6:2.7:2	1.1	2.9	3.9E-06	0.144	0.015	1.2E-01	1.6E-01	7.1E-01
LUSC	MRPL55_ES_1.2:2.2:2.5:2.6:2.8_1.1_2.9	MRPL55	ES	1.2:2.2:2.5:2.6:2.8	1.1	2.9	1.9E-05	0.128	0.034	2.5E-01	2.3E-01	6.8E-01
LUSC	MRPL55_ES_1.2:2.2:2.8_1.1_2.9	MRPL55	ES	1.2:2.2:2.8	1.1	2.9	7.1E-06	0.129	0.003	5.9E-01	7.0E-01	5.9E-01
LUSC	MRPL55_ES_2.2_1.1_2.9	MRPL55	ES	2.2	1.1	2.9	9.4E-07	0.150	-0.012	4.5E-01	4.4E-01	8.6E-01
LUSC	MRPL55_ES_2.2_1.2_2.9	MRPL55	ES	2.2	1.2	2.9	1.1E-05	0.123	-0.008	5.7E-01	5.2E-01	9.6E-01
LUSC	MRPL55_ES_2.2:2.5:2.6_1.1_2.9	MRPL55	ES	2.2:2.5:2.6	1.1	2.9	1.1E-08	0.205	0.026	4.1E-02	3.8E-01	6.6E-01
LUSC	MRPL55_ES_2.2:2.5:2.6_1.2_2.9	MRPL55	ES	2.2:2.5:2.6	1.2	2.9	3.1E-08	0.184	-0.046	6.3E-02	4.1E-01	4.8E-01
LUSC	MRPL55_ES_2.2:2.8_1.1_2.9	MRPL55	ES	2.2:2.8	1.1	2.9	5.1E-06	0.137	0.051	3.8E-01	5.0E-01	3.6E-01
LUSC	MRPL55_ES_2.2:2.8_1.2_2.9	MRPL55	ES	2.2:2.8	1.2	2.9	7.4E-05	0.104	-0.006	5.0E-01	8.8E-01	8.6E-01
LUSC	MRPL55_RI_2.3:2.4_2.2_2.5	MRPL55	RI	2.3:2.4	2.2	2.5	3.6E-06	0.134	-0.019	1.3E-02	1.6E-01	2.3E-01
LUSC	MRRF_ES_5:6_4_7	MRRF	ES	5:06	4	7	1.7E-05	0.118	-0.110	6.0E-01	9.6E-01	7.0E-01
LUSC	MTMR1_ES_4_3_5	MTMR1	ES	4	3	5	1.5E-07	0.170	-0.321	4.0E-02	4.7E-03	5.8E-01
LUSC	MTSS1_ES_7_6_8.3	MTSS1	ES	7	6	8.3	5.7E-06	0.135	0.159	8.9E-02	7.1E-01	4.2E-01

cancer type	id	Gene Symbol	splice_type	Exon	From.Exon	To.Exon	anova.p	adj.r2	r	p.50	p.25	p.10
LUSC	MYH10_ES_6.1_5_7	MYH10	ES	6.1	5	7	2.0E-05	0.152	-0.076	8.5E-01	6.4E-01	8.5E-01
LUSC	MYH11_ES_42_41_43	MYH11	ES	42	41	43	3.1E-05	0.115	-0.132	9.1E-01	6.0E-02	9.4E-01
LUSC	MYL12A_ES_1.2:2_1.1_4	MYL12A	ES	1.2:2	1.1	4	4.3E-05	0.108	0.344	4.0E-01	3.1E-01	6.4E-02
LUSC	MYL6_ES_2.1_1.4_3.1	MYL6	ES	2.1	1.4	3.1	4.3E-07	0.155	0.116	1.3E-01	2.4E-01	4.6E-01
LUSC	MYL6_ES_4.3_4.1_5	MYL6	ES	4.3	4.1	5	5.4E-07	0.153	-0.173	1.4E-01	2.0E-01	9.7E-03
LUSC	MYO1B_ES_23:24_22_25	MYO1B	ES	23:24	22	25	1.2E-07	0.168	-0.147	7.1E-01	7.0E-01	9.0E-01
LUSC	MYO1B_ES_24_22_25	MYO1B	ES	24	22	25	1.1E-06	0.145	-0.290	4.2E-01	8.7E-01	9.4E-01
LUSC	MYO5A_ES_35_34_36	MYO5A	ES	35	34	36	2.8E-09	0.208	-0.397	6.2E-01	6.3E-01	6.9E-01
LUSC	MYO6_ES_34_33_35	MYO6	ES	34	33	35	6.7E-05	0.103	-0.007	6.8E-02	5.9E-01	2.3E-01
LUSC	MYO9B_ES_37_36_38.1	MYO9B	ES	37	36	38.1	6.2E-06	0.130	-0.152	8.0E-01	9.6E-01	6.6E-02
LUSC	MYOF_ES_17_16_18	MYOF	ES	17	16	18	2.5E-06	0.141	-0.211	8.8E-01	5.9E-01	3.8E-01
LUSC	NETO2_AD_5.2_5.1_6	NETO2	AD	5.2	5.1	6	3.4E-09	0.204	-0.134	2.2E-02	3.5E-01	8.7E-01
LUSC	NF2_ES_16.1_15_17	NF2	ES	16.1	15	17	4.3E-07	0.156	0.286	8.6E-01	4.8E-01	4.4E-01
LUSC	NFE2L1_ES_6_5.2_7	NFE2L1	ES	6	5.2	7	1.2E-08	0.190	0.059	3.5E-01	1.5E-01	8.1E-01
LUSC	NPHP3_ES_17_16_18	NPHP3	ES	17	16	18	1.0E-04	0.108	-0.249	8.6E-01	4.5E-01	8.5E-01
LUSC	NPNT_ES_11_10_12	NPNT	ES	11	10	12	3.4E-06	0.137	-0.092	4.5E-01	2.3E-01	4.7E-03
LUSC	NPTN_ES_2:3:4_1_5	NPTN	ES	2:03:04	1	5	1.5E-06	0.143	-0.037	1.7E-02	9.0E-02	3.4E-01
LUSC	NSFL1C_ES_5.2:7.2_4_7.3	NSFL1C	ES	5.2:7.2	4	7.3	4.5E-06	0.132	-0.230	4.2E-01	9.7E-01	3.4E-01
LUSC	NUDT8_RI_3.2_3.1_3.3	NUDT8	RI	3.2	3.1	3.3	1.6E-11	0.252	0.078	4.0E-02	1.3E-01	7.7E-01
LUSC	NUMB_ES_7_6_8.2	NUMB	ES	7	6	8.2	1.1E-05	0.122	-0.040	2.5E-01	9.3E-01	3.1E-01
LUSC	NVL_ES_2_1_3.1	NVL	ES	2	1	3.1	8.5E-06	0.126	-0.087	2.7E-01	1.8E-01	4.3E-01
LUSC	OAZ1_ES_3.2:3.3:3.4:3.5_1_4	OAZ1	ES	3.2:3.3:3.4:3.5	1	4	2.7E-05	0.113	0.047	2.5E-01	1.7E-01	5.9E-01
LUSC	OSBPL3_ES_9_8_10	OSBPL3	ES	9	8	10	2.6E-05	0.114	-0.128	9.5E-01	9.4E-01	2.1E-01
LUSC	OSBPL8_ES_6_5_7.1	OSBPL8	ES	6	5	7.1	2.3E-05	0.121	-0.140	1.3E-01	9.0E-01	3.4E-01
LUSC	OSBPL9_ES_17_15_18	OSBPL9	ES	17	15	18	5.1E-09	0.198	0.036	7.2E-01	9.3E-01	5.2E-01
LUSC	PEX13_AA_2.1_1.3_2.2	PEX13	AA	2.1	1.3	2.2	4.2E-05	0.118	0.022	5.6E-01	9.9E-01	5.7E-01
LUSC	PFDN5_ES_2_1_5	PFDN5	ES	2	1	5	7.8E-06	0.126	0.013	7.7E-02	1.7E-01	5.6E-01
LUSC	PFDN5_ES_2:4.1:4.2_1_5	PFDN5	ES	2:4.1:4.2	1	5	7.1E-05	0.105	-0.149	3.8E-01	9.9E-01	1.4E-01
LUSC	PIGO_RI_9.2_9.1_9.3	PIGO	RI	9.2	9.1	9.3	2.5E-08	0.183	0.217	7.3E-01	5.9E-01	9.1E-01
LUSC	PIK3CD_RI_17.2_17.1_17.3	PIK3CD	RI	17.2	17.1	17.3	1.5E-04	0.106	-0.190	2.6E-01	1.5E-01	7.4E-01
LUSC	PKP4_ES_25_24_26	PKP4	ES	25	24	26	2.5E-05	0.113	-0.003	3.7E-01	3.6E-01	6.9E-01
LUSC	PLOD2_ES_15_14_16	PLOD2	ES	15	14	16	1.6E-06	0.142	-0.269	9.9E-02	1.5E-01	3.8E-01
LUSC	PLSCR1_ES_3:4_1_5	PLSCR1	ES	3:04	1	5	3.9E-06	0.134	-0.025	2.1E-02	6.2E-04	2.2E-01
LUSC	PLSCR1_ES_4_1_5	PLSCR1	ES	4	1	5	1.6E-11	0.250	-0.103	1.7E-01	1.0E-01	5.7E-01
LUSC	PODXL_ES_3_2.2_4.1	PODXL	ES	3	2.2	4.1	5.3E-05	0.106	-0.646	2.8E-01	1.9E-01	3.2E-01
LUSC	PPIL2_RI_22.3:22.4:22.5:22.6_22.2_22.7	PPIL2	RI	2.3:22.4:22.5:22.	22.2	22.7	1.4E-05	0.119	-0.023	5.1E-01	1.6E-01	3.2E-01
LUSC	PPP3CB_ES_16_15.1_17	PPP3CB	ES	16	15.1	17	2.2E-05	0.115	0.031	8.9E-01	1.5E-01	5.9E-01
LUSC	PQLC1_ES_6_5_9	PQLC1	ES	6	5	9	5.0E-05	0.107	-0.053	8.0E-01	9.8E-01	6.0E-01
LUSC	PRDX5_ES_3_1_4	PRDX5	ES	3	1	4	4.4E-05	0.107	0.026	8.1E-01	7.3E-01	3.4E-01
LUSC	PRKDC_ES_81_80_82	PRKDC	ES	81	80	82	2.3E-06	0.138	-0.415	8.3E-01	6.0E-01	5.1E-01

cancer type	id	Gene Symbol	splice_type	Exon	From.Exon	To.Exon	anova.p	adj.r2	r	p.50	p.25	p.10
LUSC	PRMT2_ES_1.3_1.1_2	PRMT2	ES	1.3	1.1	2	1.0E-08	0.192	-0.210	3.4E-01	5.4E-01	2.1E-01
LUSC	PRMT2_RI_1.2_1.1_1.3	PRMT2	RI	1.2	1.1	1.3	5.9E-05	0.105	0.009	2.0E-01	2.2E-01	8.4E-01
LUSC	PRR13_AD_1.2_1.1_3	PRR13	AD	1.2	1.1	3	1.1E-06	0.146	-0.278	3.8E-01	2.5E-01	6.4E-01
LUSC	PRR5_ES_2_1_3.2	PRR5	ES	2	1	3.2	4.7E-10	0.222	-0.249	1.7E-01	9.7E-02	5.7E-01
LUSC	PSAP_AA_8.1_7_8.2	PSAP	AA	8.1	7	8.2	3.8E-06	0.133	-0.291	4.0E-01	4.2E-01	3.5E-01
LUSC	PSMA4_ES_5.2_4_6	PSMA4	ES	5.2	4	6	2.5E-05	0.121	-0.059	5.9E-01	3.0E-01	5.0E-01
LUSC	PSTPIP1_ES_3_2.2_5	PSTPIP1	ES	3	2.2	5	2.3E-10	0.229	-0.619	8.1E-01	7.7E-01	1.6E-01
LUSC	PTBP2_ES_12_11.2_13	PTBP2	ES	12	11.2	13	2.5E-04	0.103	0.373	8.9E-01	7.1E-01	1.4E-01
LUSC	PTGR1_AA_12.1_11_12.2	PTGR1	AA	12.1	11	12.2	2.1E-06	0.139	-0.752	3.0E-01	6.6E-01	1.5E-01
LUSC	PTK2B_ES_28_27_29	PTK2B	ES	28	27	29	8.6E-06	0.125	0.146	7.9E-01	9.3E-01	8.5E-01
LUSC	PTPN3_ES_13_12_14	PTPN3	ES	13	12	14	2.4E-05	0.117	-0.115	9.2E-01	4.2E-01	4.8E-01
LUSC	R3HDM1_ES_16_15_17	R3HDM1	ES	16	15	17	5.3E-08	0.177	0.143	4.7E-02	1.4E-01	1.8E-02
LUSC	RAB6A_ME_5|6_4_7	RAB6A	ME	5|6	4	7	2.6E-12	0.266	0.047	4.8E-01	5.7E-01	7.0E-01
LUSC	RANBP3_ES_6_5_7	RANBP3	ES	6	5	7	2.8E-05	0.116	-0.004	4.7E-01	7.8E-01	6.0E-01
LUSC	RANGRF_AD_3.2:3.3_3.1_3.5	RANGRF	AD	3.2:3.3	3.1	3.5	2.4E-07	0.163	-0.185	5.0E-01	5.7E-01	2.3E-01
LUSC	RASA4_ES_5:6_4_7	RASA4	ES	5:06	4	7	9.4E-05	0.120	0.479	1.1E-01	5.9E-03	1.7E-01
LUSC	RASSF7_ES_6.3_6.1_6.5	RASSF7	ES	6.3	6.1	6.5	1.1E-06	0.170	0.088	8.9E-01	1.9E-01	5.0E-01
LUSC	RASSF7_RI_6.4_6.3_6.5	RASSF7	RI	6.4	6.3	6.5	3.3E-09	0.203	-0.098	9.1E-01	3.3E-01	9.5E-01
LUSC	RBFOX2_ES_12_11.2_14	RBFOX2	ES	12	11.2	14	7.1E-05	0.102	-0.453	7.2E-01	6.8E-01	7.1E-01
LUSC	RGS12_ES_19_18_20.1	RGS12	ES	19	18	20.1	3.0E-06	0.136	-0.112	8.1E-01	9.4E-01	5.3E-01
LUSC	RIF1_ES_31_30_32	RIF1	ES	31	30	32	3.9E-05	0.109	-0.180	9.3E-01	6.0E-01	1.0E+00
LUSC	RNF41_AD_1.2_1.1_2	RNF41	AD	1.2	1.1	2	1.1E-05	0.122	-0.043	2.2E-01	4.3E-01	7.2E-01
LUSC	RPAIN_ES_4:5:6.1_3_7	RPAIN	ES	05:06.1	3	7	1.0E-05	0.123	-0.152	4.2E-01	4.1E-01	8.1E-01
LUSC	RPL10_RI_2.2_2.1_2.3	RPL10	RI	2.2	2.1	2.3	2.1E-10	0.227	0.111	7.8E-01	3.6E-01	3.1E-02
LUSC	RPRD2_ES_4_3.1_5	RPRD2	ES	4	3.1	5	1.3E-06	0.147	-0.155	1.3E-01	2.2E-01	3.4E-02
LUSC	RPS24_AA_5.1_4_5.2	RPS24	AA	5.1	4	5.2	6.7E-07	0.151	0.033	7.1E-01	5.6E-01	6.0E-01
LUSC	RPS24_ES_5.1:5.2_4_6	RPS24	ES	5.1:5.2	4	6	1.2E-08	0.190	-0.110	9.0E-01	8.0E-01	2.6E-01
LUSC	RPS3_ES_2.1_1_3.1	RPS3	ES	2.1	1	3.1	2.0E-06	0.140	0.154	6.0E-01	1.6E-01	1.3E-01
LUSC	RTBDN_AA_9.1_8_9.2	RTBDN	AA	9.1	8	9.2	3.3E-05	0.110	-0.764	1.5E-01	5.4E-01	9.9E-01
LUSC	RTN4_ES_6.1:6.2_5_8	RTN4	ES	6.1:6.2	5	8	4.9E-07	0.156	-0.212	5.0E-01	7.6E-01	3.3E-01
LUSC	S100A6_AA_3.1_2_3.2	S100A6	AA	3.1	2	3.2	8.9E-08	0.184	-0.058	1.3E-01	7.9E-03	1.7E-01
LUSC	SCRIB_ES_17_16_18	SCRIB	ES	17	16	18	5.0E-06	0.132	-0.392	2.4E-02	7.9E-01	4.1E-01
LUSC	SDHA_ES_5_4_6	SDHA	ES	5	4	6	7.4E-06	0.126	0.467	8.9E-02	3.5E-01	3.3E-01
LUSC	SEC16A_ES_24_23.12_26	SEC16A	ES	24	23.12	26	8.9E-05	0.100	-0.065	1.7E-02	2.0E-02	3.7E-01
LUSC	SEC31A_ES_26.1_25.1_28	SEC31A	ES	26.1	25.1	28	8.6E-08	0.171	-0.171	4.0E-01	8.1E-01	5.1E-01
LUSC	SEC31A_ES_26.1:26.2_25.1_28	SEC31A	ES	26.1:26.2	25.1	28	4.5E-07	0.155	-0.179	6.9E-03	4.2E-01	4.7E-01
LUSC	SEC31A_ES_26.2:27_26.1_28	SEC31A	ES	26.2:27	26.1	28	6.8E-05	0.103	0.068	9.3E-01	2.6E-01	5.3E-01
LUSC	SEC31A_ES_27_26.2_28	SEC31A	ES	27	26.2	28	7.2E-05	0.102	0.031	4.2E-01	6.2E-01	7.8E-01
LUSC	SEPT6_ES_12_11.1_13.1	SEPT6	ES	12	11.1	13.1	2.2E-05	0.115	0.190	3.6E-01	4.2E-01	4.3E-01
LUSC	SETDB1_AD_1.2_1.1_2	SETDB1	AD	1.2	1.1	2	7.7E-06	0.126	-0.077	2.1E-01	6.1E-01	1.1E-01

cancer type	id	Gene Symbol	splice_type	Exon	From.Exon	To.Exon	anova.p	adj.r2	r	p.50	p.25	p.10
LUSC	SH2B1_AD_9.2_9.1_10	SH2B1	AD	9.2	9.1	10	3.9E-05	0.109	0.164	9.8E-01	4.1E-01	5.5E-01
LUSC	SH3BP1_ES_16_15_17.2	SH3BP1	ES	16	15	17.2	4.4E-06	0.132	-0.654	7.1E-01	6.0E-01	2.5E-01
LUSC	SIGIRR_ES_9_8_10.1	SIGIRR	ES	9	8	10.1	3.4E-05	0.120	0.246	1.0E-01	9.3E-03	2.1E-01
LUSC	SLAIN2_ES_8_6_9	SLAIN2	ES	8	6	9	1.8E-06	0.141	0.171	3.7E-01	5.9E-01	6.4E-01
LUSC	SLC37A2_ES_12_11_13	SLC37A2	ES	12	11	13	1.7E-10	0.231	-0.130	1.4E-01	1.1E-01	3.7E-01
LUSC	SLC37A2_ES_18_17_19	SLC37A2	ES	18	17	19	4.8E-11	0.246	0.377	4.5E-01	5.8E-01	6.7E-01
LUSC	SLC39A13_RI_6.2_6.1_6.3	SLC39A13	RI	6.2	6.1	6.3	8.5E-05	0.100	-0.002	5.5E-01	2.0E-01	1.0E+00
LUSC	SLMAP_ES_13:14_12_15.2	SLMAP	ES	13:14	12	15.2	1.5E-04	0.118	-0.222	2.3E-02	5.7E-03	3.4E-01
LUSC	SMARCA2_ES_33_32_34	SMARCA2	ES	33	32	34	7.3E-07	0.150	0.090	9.9E-01	8.7E-01	8.4E-01
LUSC	SMARCC2_ES_28.3_28.1_29	SMARCC2	ES	28.3	28.1	29	5.9E-07	0.167	0.267	7.3E-02	8.7E-01	1.4E-01
LUSC	SRSF5_AD_2.2:2.3_2.1_4	SRSF5	AD	2.2:2.3	2.1	4	1.8E-05	0.117	-0.095	2.0E-01	2.4E-01	6.4E-01
LUSC	SS18_ES_15_14_16	SS18	ES	15	14	16	1.6E-06	0.142	0.068	4.0E-01	5.8E-01	8.7E-01
LUSC	SSBP3_ES_7_6_8	SSBP3	ES	7	6	8	1.8E-06	0.142	0.375	3.5E-01	8.4E-02	3.3E-01
LUSC	ST3GAL1_RI_5.4_5.3_5.5	ST3GAL1	RI	5.4	5.3	5.5	3.6E-06	0.136	-0.295	9.1E-01	2.8E-01	2.7E-01
LUSC	ST3GAL3_ES_10_9_13	ST3GAL3	ES	10	9	13	5.4E-08	0.176	0.394	1.9E-01	4.7E-01	4.6E-01
LUSC	ST3GAL3_ES_12_9_13	ST3GAL3	ES	12	9	13	1.0E-05	0.123	0.447	7.2E-01	8.7E-01	9.8E-01
LUSC	STARD3NL_ES_2_1_3	STARD3NL	ES	2	1	3	6.9E-05	0.103	0.115	7.0E-01	4.8E-01	7.7E-01
LUSC	STRADA_RI_12.4_12.3_12.5	STRADA	RI	12.4	12.3	12.5	1.4E-05	0.120	0.117	3.1E-01	3.0E-01	6.7E-01
LUSC	SVIL_ES_10:11:12_9_13	SVIL	ES	10:11:12	9	13	3.8E-06	0.137	-0.381	8.2E-01	8.2E-01	9.8E-01
LUSC	SYK_ES_9_8_10	SYK	ES	9	8	10	1.3E-05	0.120	-0.190	4.7E-01	2.3E-01	2.8E-01
LUSC	SYNE2_ES_118_117.2_119.2	SYNE2	ES	118	117.2	119.2	1.8E-07	0.164	0.134	7.2E-01	3.4E-01	5.5E-01
LUSC	TACC2_AA_15.1_13_15.2	TACC2	AA	15.1	13	15.2	7.6E-05	0.102	-0.252	5.8E-01	5.2E-01	8.0E-01
LUSC	TACC2_ES_14_13_15.1	TACC2	ES	14	13	15.1	7.5E-07	0.150	0.241	5.6E-01	3.2E-01	9.9E-01
LUSC	TAF1D_AA_12.3:12.4_12.1_12.5	TAF1D	AA	12.3:12.4	12.1	12.5	9.7E-05	0.106	0.066	7.9E-01	9.6E-01	4.6E-01
LUSC	TAF1D_RI_12.2:12.3:12.4_12.1_12.5	TAF1D	RI	12.2:12.3:12.4	12.1	12.5	7.7E-05	0.109	0.047	9.3E-01	1.7E-01	1.0E-01
LUSC	TANK_ES_2.2:3_2.1_4	TANK	ES	2.2:3	2.1	4	2.8E-07	0.159	-0.351	2.8E-02	1.8E-02	2.1E-01
LUSC	TANK_ES_3_2.2_4	TANK	ES	3	2.2	4	1.6E-09	0.209	-0.414	3.5E-01	1.1E-02	4.8E-01
LUSC	TBRG1_ES_4_3_6	TBRG1	ES	4	3	6	8.1E-05	0.101	0.101	7.7E-01	3.4E-01	5.8E-01
LUSC	TBRG1_ES_5_3_6	TBRG1	ES	5	3	6	1.8E-07	0.164	0.171	2.8E-01	8.3E-01	3.7E-01
LUSC	TBX3_ES_3_2_4	TBX3	ES	3	2	4	5.7E-06	0.139	0.138	7.3E-02	3.6E-02	1.1E-02
LUSC	TCF12_ES_18_17_19	TCF12	ES	18	17	19	3.5E-05	0.111	0.090	2.9E-01	3.8E-01	6.6E-02
LUSC	TEAD2_ES_6:7.1_5_8	TEAD2	ES	06:07.1	5	8	7.1E-06	0.144	0.067	3.0E-01	8.3E-02	2.1E-02
LUSC	TEAD2_ES_6:7.1:7.2_5_8	TEAD2	ES	6:7.1:7.2	5	8	9.1E-06	0.141	0.117	3.5E-01	6.1E-03	3.6E-02
LUSC	TKT_RI_16.2_16.1_16.3	TKT	RI	16.2	16.1	16.3	1.2E-05	0.121	-0.261	9.5E-01	5.7E-01	2.4E-01
LUSC	TMEM106C_AD_5.2_5.1_6	TMEM106C	AD	5.2	5.1	6	1.8E-09	0.208	0.136	4.1E-01	4.6E-01	5.1E-01
LUSC	TMEM107_ES_2:3.2:3.4:3.5_1_3.7	TMEM107	ES	2:3.2:3.4:3.5	1	3.7	3.2E-06	0.135	0.207	1.0E+00	6.3E-01	6.9E-01
LUSC	TMEM120A_ES_10_9_11	TMEM120A	ES	10	9	11	2.7E-09	0.204	0.059	5.9E-02	8.1E-02	4.9E-02
LUSC	TMEM180_ES_6_5_7	TMEM180	ES	6	5	7	9.9E-10	0.216	-0.105	2.1E-01	1.3E-01	7.5E-02
LUSC	TMEM205_AA_2.5_2.2_2.6	TMEM205	AA	2.5	2.2	2.6	3.2E-05	0.111	0.130	9.6E-01	8.9E-01	9.0E-01
LUSC	TMEM241_ES_7_6_8	TMEM241	ES	7	6	8	1.0E-04	0.102	0.081	4.2E-01	8.3E-01	7.2E-01

cancer type	id	Gene Symbol	splice_type	Exon	From.Exon	To.Exon	anova.p	adj.r2	r	p.50	p.25	p.10
LUSC	TMEM251_AD_1.2_1.1_2	TMEM251	AD	1.2	1.1	2	2.1E-05	0.115	-0.261	1.4E-01	4.1E-01	5.9E-01
LUSC	TMEM91_AA_7.4:7.5:7.6_7.1_7.7	TMEM91	AA	7.4:7.5:7.6	7.1	7.7	5.3E-04	0.102	0.095	4.4E-01	8.8E-01	1.7E-01
LUSC	TMEM91_RI_7.2:7.3:7.4:7.5:7.6_7.1_7.7	TMEM91	RI	7.2:7.3:7.4:7.5:7.[illegible]	7.1	7.7	2.4E-05	0.114	-0.406	6.3E-01	9.4E-01	9.2E-01
LUSC	TOP1MT_ES_4_3_5	TOP1MT	ES	4	3	5	4.0E-05	0.110	-0.264	3.9E-03	1.7E-01	7.7E-02
LUSC	TPRA1_ES_8:10:11_7_12	TPRA1	ES	8:10:11	7	12	9.0E-06	0.138	-0.228	1.3E-01	6.4E-01	5.5E-01
LUSC	TRA2B_ES_2_1_3	TRA2B	ES	2	1	3	1.5E-13	0.290	0.671	2.7E-01	1.3E-02	3.1E-01
LUSC	TREX2_ES_9_8.2_10	TREX2	ES	9	8.2	10	1.5E-06	0.151	0.086	6.9E-01	8.1E-01	7.0E-01
LUSC	TRIM16L_ES_2_1.3_3	TRIM16L	ES	2	1.3	3	7.0E-06	0.132	-0.239	4.9E-01	6.9E-01	7.4E-01
LUSC	TRPM2_ES_29_28_30	TRPM2	ES	29	28	30	3.3E-05	0.113	-0.337	3.9E-01	4.5E-01	1.3E-01
LUSC	TSC2_ES_27_26_28.1	TSC2	ES	27	26	28.1	2.3E-10	0.227	-0.013	1.7E-02	3.4E-02	1.9E-01
LUSC	TSC2_ES_27_26_28.2	TSC2	ES	27	26	28.2	2.9E-04	0.106	-0.171	3.7E-01	7.2E-02	1.0E-01
LUSC	TSC2_ES_27:28.1_26_28.2	TSC2	ES	27:28.1	26	28.2	7.5E-12	0.258	-0.048	2.3E-01	8.4E-02	1.5E-01
LUSC	TSTD1_ES_2.2:2.3_1_3.1	TSTD1	ES	2.2:2.3	1	3.1	2.0E-07	0.164	0.047	7.1E-01	3.8E-01	7.3E-01
LUSC	TUSC3_ES_11_10_12	TUSC3	ES	11	10	12	6.1E-05	0.104	-0.153	7.1E-01	5.0E-01	4.2E-01
LUSC	TYMP_AA_2.1_1_2.2	TYMP	AA	2.1	1	2.2	4.9E-05	0.107	-0.190	2.4E-01	2.3E-01	5.4E-01
LUSC	UAP1_ES_9.2_8_10	UAP1	ES	9.2	8	10	1.9E-06	0.140	0.117	1.1E-01	3.3E-01	4.2E-01
LUSC	UBL5_ES_3_2.1_4	UBL5	ES	3	2.1	4	9.6E-05	0.125	0.003	1.6E-01	4.1E-01	3.8E-01
LUSC	UCHL5_ES_13.2_12_15	UCHL5	ES	13.2	12	15	1.3E-05	0.120	0.102	2.3E-01	5.8E-01	3.4E-01
LUSC	UGT1A6_ES_6.1_5_7	UGT1A6	ES	6.1	5	7	4.7E-08	0.193	0.218	8.7E-01	8.9E-01	4.6E-01
LUSC	URI1_AA_13.1_12_13.2	URI1	AA	13.1	12	13.2	1.6E-10	0.230	-0.297	4.1E-01	8.1E-01	3.1E-01
LUSC	VKORC1_ES_4.2:5_2_6	VKORC1	ES	4.2:5	2	6	3.9E-05	0.109	0.161	3.9E-01	8.5E-01	3.3E-01
LUSC	VLDLR_ES_16_15_17	VLDLR	ES	16	15	17	1.9E-11	0.249	-0.378	3.6E-02	1.7E-01	1.4E-01
LUSC	VPS39_ES_3_2_4	VPS39	ES	3	2	4	4.6E-13	0.288	0.119	4.4E-01	8.2E-02	9.2E-01
LUSC	WASF3_ME_7|8_6_9	WASF3	ME	7|8	6	9	5.4E-06	0.145	0.469	7.4E-01	5.8E-01	5.6E-01
LUSC	WNK1_ES_14:15_13.2_16	WNK1	ES	14:15	13.2	16	1.2E-05	0.122	0.040	5.8E-01	7.6E-01	9.9E-01
LUSC	YPEL5_ES_3.1:3.2_1_5	YPEL5	ES	3.1:3.2	1	5	3.3E-06	0.135	-0.108	1.7E-01	4.6E-02	1.6E-01
LUSC	ZMIZ1_ES_24_23_25	ZMIZ1	ES	24	23	25	7.1E-08	0.173	0.130	7.4E-01	4.7E-01	7.5E-01
LUSC	ZMYND8_AD_19.2_19.1_20	ZMYND8	AD	19.2	19.1	20	6.6E-05	0.103	-0.064	9.3E-01	6.1E-01	5.3E-01
LUSC	ZMYND8_ES_26_25_27	ZMYND8	ES	26	25	27	1.6E-05	0.118	0.090	3.7E-01	5.5E-01	4.3E-01
LUSC	ZSCAN32_ES_3:4:5.1:5.2_2.2_6.1	ZSCAN32	ES	3:4:5.1:5.2	2.2	6.1	3.1E-05	0.135	-0.161	4.8E-01	7.6E-01	6.9E-01
LUSC	ZSWIM7_RI_7.2_7.1_7.3	ZSWIM7	RI	7.2	7.1	7.3	4.8E-05	0.106	0.066	7.0E-01	9.4E-01	4.8E-01
OV	ABI1_ES_11.1:11.2:12_9_13	ABI1	ES	11.1:11.2:12	9	13	1.1E-13	0.140	-0.129	9.5E-01	6.1E-01	7.3E-01
OV	ABI1_ES_11.2:12_9_13	ABI1	ES	11.2:12	9	13	1.8E-16	0.169	-0.139	7.5E-01	7.1E-01	8.3E-01
OV	ABI1_ES_12_11.2_13	ABI1	ES	12	11.2	13	1.4E-12	0.129	-0.034	7.0E-01	6.3E-01	1.0E+00
OV	ACP5_AA_2.1_1_2.2	ACP5	AA	2.1	1	2.2	4.2E-10	0.108	-0.370	1.5E-01	6.9E-01	5.0E-01
OV	ADD3_ES_15_14_16	ADD3	ES	15	14	16	2.8E-11	0.116	0.116	5.5E-01	4.9E-01	9.6E-02
OV	ADORA1_ES_4_3.2_5	ADORA1	ES	4	3.2	5	3.8E-09	0.104	-0.030	2.6E-01	1.8E-01	1.9E-01
OV	AFTPH_ES_8_7_9	AFTPH	ES	8	7	9	1.2E-11	0.120	0.155	1.9E-01	9.7E-01	2.3E-01
OV	ALAS1_ES_2_1_3.1	ALAS1	ES	2	1	3.1	3.7E-10	0.104	0.027	8.0E-01	5.9E-01	7.0E-01
OV	ANO1_ES_17_16_18	ANO1	ES	17	16	18	8.1E-19	0.208	-0.610	4.0E-01	2.0E-01	8.7E-01

cancer type	id	Gene Symbol	splice_type	Exon	From.Exon	To.Exon	anova.p	adj.r2	r	p.50	p.25	p.10
OV	ANXA6_ES_22_21_23	ANXA6	ES	22	21	23	2.8E-13	0.136	-0.025	2.7E-01	4.2E-01	9.3E-01
OV	AP1B1_ES_24_23_25	AP1B1	ES	24	23	25	3.0E-15	0.156	0.017	9.9E-01	2.6E-01	2.7E-01
OV	AP1G1_ES_12_10.1_13.2	AP1G1	ES	12	10.1	13.2	5.7E-10	0.115	-0.035	3.2E-01	8.3E-01	6.5E-01
OV	APP_ES_10_9_11	APP	ES	10	9	11	5.6E-13	0.133	-0.172	9.8E-01	8.2E-01	4.8E-01
OV	APP_ES_9:10_8_11	APP	ES	9:10	8	11	9.7E-13	0.131	-0.096	2.0E-01	7.2E-01	3.2E-01
OV	ARFIP1_ES_4_3_5	ARFIP1	ES	4	3	5	9.9E-16	0.163	0.174	1.6E-01	9.2E-02	3.2E-01
OV	ARHGEF1_ES_15_14_16	ARHGEF1	ES	15	14	16	5.0E-11	0.113	0.243	4.3E-02	2.2E-02	4.7E-02
OV	ARHGEF10L_ES_18_17_19	ARHGEF10L	ES	18	17	19	2.3E-09	0.101	0.139	1.4E-01	3.6E-01	6.9E-01
OV	ATP5J_AD_1.4:1.5_1.3_3	ATP5J	AD	1.4:1.5	1.3	3	8.0E-12	0.121	0.210	3.7E-01	8.6E-01	1.5E-01
OV	ATP6V1C2_ES_11_10_12	ATP6V1C2	ES	11	10	12	5.1E-11	0.136	0.045	5.8E-01	7.0E-01	6.2E-02
OV	BMP1_AA_5.1_4_5.2	BMP1	AA	5.1	4	5.2	5.3E-13	0.135	-0.039	4.5E-02	5.9E-01	3.2E-01
OV	C1orf54_ES_7_6_8	C1orf54	ES	7	6	8	4.8E-14	0.145	0.119	5.6E-02	1.8E-01	9.5E-01
OV	CA12_ES_9_8_10	CA12	ES	9	8	10	3.4E-22	0.270	0.233	3.5E-01	3.0E-01	2.2E-01
OV	CAMK2D_AA_14.1_13_14.2	CAMK2D	AA	14.1	13	14.2	3.3E-09	0.110	0.257	3.3E-01	2.3E-01	4.2E-01
OV	CARD9_AA_8.1_7_8.2	CARD9	AA	8.1	7	8.2	4.4E-10	0.109	-0.529	4.4E-01	2.7E-01	1.9E-01
OV	CARM1_ES_16.1_15_16.3	CARM1	ES	16.1	15	16.3	6.0E-25	0.246	0.117	8.7E-01	5.2E-01	7.0E-01
OV	CCDC107_RI_3.4_3.3_3.5	CCDC107	RI	3.4	3.3	3.5	3.0E-11	0.115	-0.412	3.5E-01	8.0E-01	2.9E-01
OV	CCDC50_ES_6_5_7	CCDC50	ES	6	5	7	2.1E-10	0.107	-0.080	5.9E-01	4.9E-01	6.4E-02
OV	CCSER2_ES_11_10_12	CCSER2	ES	11	10	12	6.0E-17	0.173	-0.037	8.9E-01	5.8E-01	3.4E-01
OV	CD44_ES_12.1:13_5_14	CD44	ES	12.1:13	5	14	1.7E-09	0.102	-0.159	5.3E-01	3.8E-01	3.1E-01
OV	CD44_ES_12.1:13:14_5_15	CD44	ES	12.1:13:14	5	15	4.5E-14	0.145	0.125	8.6E-01	9.2E-01	8.7E-01
OV	CHTF8_ES_4.1_3_4.3	CHTF8	ES	4.1	3	4.3	4.7E-13	0.134	0.262	6.1E-01	8.4E-01	6.9E-02
OV	CLASP1_ES_29_28.1_31.1	CLASP1	ES	29	28.1	31.1	4.3E-11	0.133	0.173	3.4E-01	6.8E-01	9.7E-01
OV	CLEC16A_ES_24_23_25	CLEC16A	ES	24	23	25	2.3E-12	0.130	0.056	4.3E-01	8.2E-01	9.1E-01
OV	CLSTN1_ES_11_10_12	CLSTN1	ES	11	10	12	1.3E-16	0.169	-0.028	2.4E-01	3.9E-01	1.9E-01
OV	CMTM7_ES_3:4.1_2_4.2	CMTM7	ES	03:04.1	2	4.2	1.0E-12	0.131	-0.149	5.9E-01	8.3E-01	2.6E-01
OV	CTNND1_ES_21_20_22.1	CTNND1	ES	21	20	22.1	5.9E-21	0.210	0.101	6.1E-01	9.2E-01	9.5E-01
OV	CTNND1_ES_21:22.1_20_22.2	CTNND1	ES	21:22.1	20	22.2	9.7E-17	0.172	0.120	7.3E-01	9.0E-01	7.6E-01
OV	DBNDD2_AD_1.2:1.3:1.4_1.1_3.3	DBNDD2	AD	1.2:1.3:1.4	1.1	3.3	2.4E-10	0.106	-0.221	3.5E-01	7.3E-01	5.8E-01
OV	DCAF6_ES_12:13.1:14_10_15	DCAF6	ES	13:01.1	10	15	2.1E-08	0.101	0.222	9.3E-01	5.7E-01	8.5E-01
OV	DCAF6_ES_13.1:14_10_15	DCAF6	ES	13.1:14	10	15	4.3E-11	0.133	0.334	2.8E-01	7.1E-01	9.5E-01
OV	DEPDC5_ES_36_35_37	DEPDC5	ES	36	35	37	1.3E-09	0.112	0.015	3.5E-01	4.6E-02	2.2E-01
OV	DGUOK_ES_5:6_4_7	DGUOK	ES	5:06	4	7	1.2E-10	0.109	0.013	6.9E-01	8.2E-01	8.8E-01
OV	DMKN_RI_6.2:6.3_6.1_6.4	DMKN	RI	6.2:6.3	6.1	6.4	9.4E-10	0.100	0.245	6.3E-01	7.9E-01	9.5E-01
OV	DYRK1B_AA_10.1:10.2_9_10.3	DYRK1B	AA	10.1:10.2	9	10.3	1.3E-10	0.109	0.172	8.2E-01	6.3E-01	4.7E-01
OV	EAF2_ES_4_3_5	EAF2	ES	4	3	5	6.1E-10	0.104	0.173	6.9E-02	7.0E-01	2.7E-01
OV	EHBP1_ES_18_17_19	EHBP1	ES	18	17	19	3.1E-10	0.106	0.150	1.8E-01	2.2E-01	5.0E-01
OV	ELN_AA_24.1_23_24.2	ELN	AA	24.1	23	24.2	1.2E-09	0.120	-0.353	1.4E-01	8.9E-02	5.5E-01
OV	ELN_ES_23:24.1_21_24.2	ELN	ES	23:24.1	21	24.2	5.1E-08	0.103	-0.325	8.6E-02	5.6E-02	9.6E-02
OV	EPB41_ES_18_15_19.1	EPB41	ES	18	15	19.1	1.6E-09	0.104	0.132	4.7E-01	4.0E-02	5.5E-01

cancer type	id	Gene Symbol	splice_type	Exon	From.Exon	To.Exon	anova.p	adj.r2	r	p.50	p.25	p.10
OV	EPB41L2_ES_17:18_14_20.1	EPB41L2	ES	17:18	14	20.1	9.3E-11	0.113	-0.039	3.2E-01	2.9E-01	6.6E-01
OV	EPB41L2_ES_17:18:20.1_14_21	EPB41L2	ES	18:20.1	14	21	4.6E-08	0.104	0.065	1.8E-01	2.5E-01	9.2E-01
OV	EPOR_ES_4.1:4.2_3_5	EPOR	ES	4.1:4.2	3	5	1.5E-09	0.112	0.157	9.1E-01	6.0E-01	5.3E-01
OV	ESRP1_AD_12.2_12.1_13	ESRP1	AD	12.2	12.1	13	2.4E-15	0.158	-0.474	6.3E-01	9.6E-01	7.9E-01
OV	EXOC1_ES_11_10_12	EXOC1	ES	11	10	12	1.7E-10	0.109	0.233	9.2E-01	9.6E-01	1.7E-01
OV	EXOC7_ES_8.2_7_9	EXOC7	ES	8.2	7	9	2.8E-12	0.131	-0.118	3.4E-01	2.1E-01	5.3E-01
OV	FAM168A_ES_4_3_5	FAM168A	ES	4	3	5	5.1E-12	0.125	0.088	9.6E-01	9.9E-01	7.9E-01
OV	FGFR1_ES_6_4_8.2	FGFR1	ES	6	4	8.2	1.4E-11	0.119	0.421	8.9E-01	1.2E-01	3.4E-02
OV	FKBP7_ES_2:3_1_4.1	FKBP7	ES	2:03	1	4.1	2.9E-14	0.174	-0.140	4.1E-01	6.6E-01	5.8E-01
OV	FKBP7_ES_3_2_4.1	FKBP7	ES	3	2	4.1	8.9E-12	0.126	-0.110	6.5E-01	9.2E-01	5.8E-02
OV	FKBP7_ES_3:4.1_2_4.2	FKBP7	ES	03:04.1	2	4.2	3.2E-13	0.162	-0.242	9.5E-01	5.3E-01	8.8E-01
OV	FLNA_ES_30_29_31	FLNA	ES	30	29	31	1.4E-15	0.159	-0.063	8.1E-01	7.1E-01	7.6E-01
OV	FLNB_ES_32.1_31_33	FLNB	ES	32.1	31	33	1.4E-15	0.170	0.215	2.2E-01	7.4E-01	6.7E-01
OV	FLNB_ES_32.1:32.2_31_33	FLNB	ES	32.1:32.2	31	33	6.0E-13	0.133	0.125	1.1E-01	3.8E-01	2.9E-01
OV	FMNL3_ES_26_25_27	FMNL3	ES	26	25	27	4.5E-16	0.164	0.179	7.7E-01	8.7E-01	6.1E-01
OV	FMNL3_ES_6_5_7	FMNL3	ES	6	5	7	1.6E-14	0.150	0.052	7.3E-01	5.4E-01	8.3E-01
OV	FN1_AA_40.1:40.2:40.3_39_40.4	FN1	AA	40.1:40.2:40.3	39	40.4	1.3E-15	0.159	0.226	3.9E-01	8.8E-01	5.5E-01
OV	FN1_ES_18:19:20:21:22:23:24:25:26:27:	FN1	ES	:25:26:27:28:29:	17	37	2.9E-11	0.131	-0.049	4.2E-01	9.9E-01	8.4E-01
OV	FN1_ES_18:19:20:21:22:23:24:25:26:27:	FN1	ES	24:25:26:27:28:29	17	37	1.7E-10	0.118	0.012	8.2E-01	6.5E-01	7.3E-01
OV	FN1_ES_25_24_26	FN1	ES	25	24	26	4.7E-33	0.314	-0.243	7.9E-01	7.4E-01	7.0E-01
OV	FNBP1_ES_10.1:10.2:10.3_9_14.2	FNBP1	ES	10.1:10.2:10.3	9	14.2	1.7E-08	0.104	0.226	1.2E-01	7.1E-01	5.2E-01
OV	FNBP1_ES_10.2:10.3_9_14.2	FNBP1	ES	10.2:10.3	9	14.2	1.4E-11	0.145	0.154	7.6E-01	4.5E-01	6.7E-01
OV	FNBP1_ES_10.2:10.3:12_9_14.2	FNBP1	ES	10.2:10.3:12	9	14.2	2.1E-10	0.130	0.186	8.5E-02	3.4E-01	7.0E-01
OV	FNIP1_ES_7_6_8	FNIP1	ES	7	6	8	4.3E-13	0.161	0.094	3.1E-01	7.8E-01	9.0E-02
OV	FRMD4A_ES_30_29_31	FRMD4A	ES	30	29	31	1.7E-11	0.123	0.256	1.6E-01	7.4E-01	7.2E-01
OV	FYN_ME_11|12_10_13	FYN	ME	11|12	10	13	1.8E-34	0.325	0.440	9.7E-01	4.7E-01	5.0E-02
OV	GK_ES_23_22_24	GK	ES	23	22	24	1.4E-13	0.143	-0.239	4.6E-01	5.0E-01	9.4E-01
OV	GNAS_ES_6_5_8.2	GNAS	ES	6	5	8.2	6.4E-14	0.143	0.154	2.8E-01	8.2E-02	1.1E-01
OV	GNAS_ES_6:8.1_5_8.2	GNAS	ES	06:08.1	5	8.2	2.3E-10	0.106	0.166	3.0E-01	1.1E-01	1.3E-01
OV	GOLGA2_ES_7_6_8	GOLGA2	ES	7	6	8	2.6E-12	0.126	0.125	4.0E-01	1.4E-01	9.0E-01
OV	GPR132_ES_4_3_6	GPR132	ES	4	3	6	1.1E-10	0.120	0.081	1.1E-01	2.3E-01	3.0E-01
OV	GPR132_ES_4:5_3_6	GPR132	ES	4:05	3	6	5.2E-15	0.184	-0.125	3.5E-01	7.2E-01	5.1E-01
OV	GUSB_ES_6_5.1_7	GUSB	ES	6	5.1	7	7.5E-10	0.101	-0.056	7.0E-01	9.1E-01	9.4E-01
OV	H2AFY_ME_7|8_6.3_9	H2AFY	ME	7|8	6.3	9	2.7E-12	0.126	0.358	9.2E-01	4.7E-01	7.2E-02
OV	HHLA3_ES_4_3_5	HHLA3	ES	4	3	5	5.5E-10	0.102	-0.124	6.9E-01	6.7E-01	7.2E-02
OV	ICAM3_RI_3.2_3.1_3.3	ICAM3	RI	3.2	3.1	3.3	1.2E-30	0.294	-0.203	9.3E-01	2.4E-01	7.6E-02
OV	IFT172_ES_46_45_47	IFT172	ES	46	45	47	6.1E-24	0.241	0.092	9.9E-01	6.0E-01	9.9E-01
OV	IGFLR1_ES_3_2.2_4.1	IGFLR1	ES	3	2.2	4.1	1.5E-09	0.118	0.034	6.6E-01	2.1E-01	3.4E-01
OV	IGFLR1_ES_3_2.2_5	IGFLR1	ES	3	2.2	5	2.5E-14	0.170	-0.098	4.2E-01	7.6E-01	3.8E-01
OV	IGFLR1_ES_3:4.2_2.2_4.3	IGFLR1	ES	03:04.2	2.2	4.3	1.0E-15	0.169	-0.180	1.1E-01	3.3E-01	3.1E-01

cancer type	id	Gene Symbol	splice_type	Exon	From.Exon	To.Exon	anova.p	adj.r2	r	p.50	p.25	p.10
OV	IGFLR1_ES_3:4.2:4.3_2.2_5	IGFLR1	ES	3:4.2:4.3	2.2	5	1.1E-22	0.237	-0.169	9.5E-01	4.6E-01	2.5E-01
OV	IL15_ES_3_2_4.2	IL15	ES	3	2	4.2	4.0E-15	0.160	-0.589	1.7E-02	1.8E-02	1.1E-01
OV	IL18BP_RI_1.2:1.3:1.4:1.5:1.6:1.7_1.1_1.	IL18BP	RI	2:1.3:1.4:1.5:1.6:1	1.1	1.8	3.8E-18	0.193	-0.283	6.4E-01	2.4E-01	5.6E-01
OV	IL18BP_RI_1.7_1.6_1.8	IL18BP	RI	1.7	1.6	1.8	1.4E-27	0.271	-0.525	9.5E-01	7.0E-01	6.1E-01
OV	IL32_AD_1.2:1.3:1.4:1.5_1.1_1.9	IL32	AD	1.2:1.3:1.4:1.5	1.1	1.9	4.4E-10	0.103	0.039	3.0E-01	1.7E-01	4.4E-01
OV	ITGB2_ES_2_1_4.2	ITGB2	ES	2	1	4.2	1.3E-09	0.104	0.005	2.9E-01	1.4E-01	8.4E-02
OV	ITGB3BP_ES_9_8_10	ITGB3BP	ES	9	8	10	1.6E-11	0.118	-0.211	2.6E-01	9.5E-01	3.1E-01
OV	KIAA1468_ME_24|25_23_26	KIAA1468	ME	24|25	23	26	7.3E-11	0.114	-0.113	1.6E-01	7.7E-01	5.7E-01
OV	KIF13A_ES_28_27_29	KIF13A	ES	28	27	29	5.2E-09	0.105	-0.098	7.1E-01	7.5E-01	9.1E-01
OV	KIF13A_ES_40_39_41.1	KIF13A	ES	40	39	41.1	5.8E-14	0.144	0.080	7.4E-01	7.4E-01	3.1E-01
OV	KLC1_AD_13.3_13.2_14.1	KLC1	AD	13.3	13.2	14.1	1.7E-11	0.118	-0.129	7.7E-01	5.1E-01	4.1E-02
OV	KLC1_ES_13.3:15_13.2_18	KLC1	ES	13.3:15	13.2	18	9.8E-11	0.110	-0.074	1.3E-01	9.0E-02	2.7E-01
OV	KRAS_ES_6_5_7	KRAS	ES	6	5	7	2.5E-14	0.152	-0.129	5.4E-01	3.1E-01	7.7E-01
OV	MAD2L2_ES_2:3_1_4	MAD2L2	ES	2:03	1	4	9.6E-25	0.244	-0.491	3.3E-02	3.2E-01	9.7E-01
OV	MAGI3_ES_22.1_21_23	MAGI3	ES	22.1	21	23	5.4E-17	0.187	0.132	2.8E-01	4.4E-01	6.8E-02
OV	MAP3K7_ES_11_10_12	MAP3K7	ES	11	10	12	3.8E-12	0.125	-0.171	4.9E-02	4.8E-02	1.9E-01
OV	MARCH8_ES_7_6_8	MARCH8	ES	7	6	8	2.3E-12	0.128	-0.013	7.3E-01	5.9E-02	2.4E-01
OV	MARK2_ES_18:19_17_20	MARK2	ES	18:19	17	20	4.5E-10	0.105	0.129	7.1E-01	6.0E-02	6.8E-01
OV	MARK2_ES_19_18_20	MARK2	ES	19	18	20	2.6E-11	0.120	0.129	6.2E-01	9.8E-01	2.2E-01
OV	MARK3_ES_17_16_18	MARK3	ES	17	16	18	2.6E-19	0.205	-0.266	6.4E-01	4.1E-01	3.5E-03
OV	MBNL1_ES_8_7_9	MBNL1	ES	8	7	9	1.0E-28	0.280	-0.442	5.3E-01	1.2E-01	5.1E-02
OV	MBNL2_ES_10_8_11	MBNL2	ES	10	8	11	5.6E-14	0.151	0.076	4.9E-01	5.5E-01	3.3E-01
OV	MBNL2_ES_7_6.3_8	MBNL2	ES	7	6.3	8	4.2E-17	0.177	-0.065	6.3E-01	5.7E-01	3.2E-01
OV	MBNL2_ES_9:10_8_11	MBNL2	ES	9:10	8	11	9.2E-11	0.121	0.024	3.9E-01	3.4E-01	4.2E-01
OV	MEIS1_ES_12.3_12.1_12.5	MEIS1	ES	12.3	12.1	12.5	7.0E-14	0.142	0.162	4.2E-01	7.4E-01	2.8E-01
OV	MEIS3_AA_7.1_6_7.2	MEIS3	AA	7.1	6	7.2	2.8E-23	0.250	0.752	9.3E-01	1.0E+00	4.0E-01
OV	MFAP5_ES_7_6_8	MFAP5	ES	7	6	8	3.2E-14	0.154	0.171	5.3E-01	5.7E-01	3.3E-01
OV	MOV10_ES_3:4.1_2.3_4.2	MOV10	ES	03:04.1	2.3	4.2	4.7E-10	0.103	0.000	3.8E-01	1.9E-01	6.4E-01
OV	MPRIP_ES_24_23_25	MPRIP	ES	24	23	25	5.8E-33	0.313	0.143	7.4E-01	6.5E-01	3.1E-01
OV	MRPL55_ES_2.2:2.8:2.9_1.2_4.1	MRPL55	ES	2.2:2.8:2.9	1.2	4.1	3.1E-12	0.146	0.266	9.9E-01	5.2E-01	9.1E-01
OV	MTCH1_AD_8.2_8.1_9	MTCH1	AD	8.2	8.1	9	1.5E-13	0.139	-0.006	8.1E-01	8.7E-01	3.6E-01
OV	MYO18A_ES_41_40_42	MYO18A	ES	41	40	42	1.6E-15	0.160	-0.178	1.6E-02	2.0E-03	1.2E-02
OV	MYO1B_ES_23_22_24	MYO1B	ES	23	22	24	8.3E-10	0.122	0.057	6.3E-01	1.7E-01	5.1E-01
OV	MYO1B_ES_23:24_22_25	MYO1B	ES	23:24	22	25	4.0E-11	0.123	0.123	6.1E-01	2.1E-01	4.4E-02
OV	MYO5A_ES_33_32_34	MYO5A	ES	33	32	34	5.4E-08	0.101	-0.066	3.9E-01	7.9E-01	7.8E-01
OV	MYO9B_ES_37_36_38.1	MYO9B	ES	37	36	38.1	7.9E-27	0.268	0.280	5.5E-02	7.9E-01	9.7E-01
OV	MYOF_ES_17_16_18	MYOF	ES	17	16	18	9.4E-10	0.105	-0.444	3.5E-01	8.6E-01	9.1E-01
OV	NAALADL1_ES_12:13_11_14	NAALADL1	ES	12:13	11	14	4.8E-14	0.173	0.417	6.3E-01	7.5E-01	6.5E-01
OV	NEDD4L_ES_18_17_19	NEDD4L	ES	18	17	19	6.7E-12	0.126	0.085	7.3E-01	2.3E-01	4.0E-01
OV	NFE2L1_ES_6_5.2_7	NFE2L1	ES	6	5.2	7	3.6E-16	0.165	0.118	2.0E-01	6.8E-01	5.1E-01

cancer type	id	Gene Symbol	splice_type	Exon	From.Exon	To.Exon	anova.p	adj.r2	r	p.50	p.25	p.10
OV	NUBP2_ES_4_3_5	NUBP2	ES	4	3	5	3.6E-13	0.135	0.024	3.8E-01	8.3E-01	6.9E-01
OV	NUMA1_ES_18_17_19	NUMA1	ES	18	17	19	3.1E-18	0.185	-0.002	1.6E-01	4.0E-01	1.5E-01
OV	OSBPL3_ES_9_8_10	OSBPL3	ES	9	8	10	3.1E-13	0.144	-0.142	3.4E-01	6.7E-01	9.4E-01
OV	OSBPL9_ES_17_15_18	OSBPL9	ES	17	15	18	4.6E-20	0.202	-0.164	4.9E-01	8.2E-02	9.1E-01
OV	PBRM1_ES_28:29_27_30	PBRM1	ES	28:29:00	27	30	5.4E-12	0.125	0.168	2.9E-01	2.9E-02	1.7E-01
OV	PCYT2_ES_7_6_8	PCYT2	ES	7	6	8	8.6E-13	0.131	0.173	3.0E-01	3.7E-01	2.2E-02
OV	PDGFA_ES_6_5_7	PDGFA	ES	6	5	7	2.6E-11	0.119	0.472	1.2E-01	7.1E-01	6.9E-01
OV	PHLDB1_RI_18.2_18.1_18.3	PHLDB1	RI	18.2	18.1	18.3	2.1E-09	0.122	0.084	7.3E-01	9.3E-01	7.1E-01
OV	PICALM_ES_19_18_20	PICALM	ES	19	18	20	1.0E-13	0.141	0.235	6.2E-01	6.0E-01	8.7E-01
OV	PLB1_ES_42:43:44_41_45	PLB1	ES	42:43:44	41	45	4.7E-09	0.108	-0.050	2.4E-01	6.4E-01	7.7E-01
OV	PLCB2_ES_29_28_30	PLCB2	ES	29	28	30	4.4E-17	0.178	0.377	1.9E-01	3.9E-01	3.3E-01
OV	PLOD2_ES_15_14_16	PLOD2	ES	15	14	16	2.5E-25	0.251	-0.148	8.1E-01	1.0E-01	1.5E-01
OV	PLSCR1_ES_3:4_1_5	PLSCR1	ES	3:04	1	5	1.2E-10	0.112	0.045	9.2E-01	7.5E-01	8.7E-01
OV	PLSCR4_ES_2.2:3_1_4	PLSCR4	ES	2.2:3	1	4	1.7E-08	0.102	-0.160	1.8E-02	1.6E-01	5.8E-01
OV	PODNL1_ES_5_4_8	PODNL1	ES	5	4	8	7.0E-20	0.246	0.349	8.3E-02	5.3E-01	2.3E-01
OV	PODNL1_ES_5:6:7_4_8	PODNL1	ES	5:06:07	4	8	4.7E-22	0.263	0.335	1.6E-01	7.8E-01	3.6E-01
OV	PPHLN1_ES_10_9_11	PPHLN1	ES	10	9	11	1.1E-17	0.180	-0.267	7.9E-01	5.9E-01	2.8E-01
OV	PPP1R9A_ES_15.1:15.2:16:17:18.1:18.2_	PPP1R9A	ES	:15.2:16:17:18.1:	14	19	2.1E-09	0.108	-0.237	3.8E-01	9.0E-01	7.3E-01
OV	PPP3CB_ES_16_15.1_17	PPP3CB	ES	16	15.1	17	5.3E-11	0.115	0.081	7.2E-01	9.7E-01	5.5E-01
OV	PQBP1_AD_1.2_1.1_2.3	PQBP1	AD	1.2	1.1	2.3	1.6E-14	0.149	-0.091	2.6E-01	8.7E-01	5.4E-01
OV	PRDX5_ES_3_1_4	PRDX5	ES	3	1	4	1.3E-13	0.140	0.090	6.6E-01	3.9E-01	9.9E-01
OV	PRMT2_ES_1.3_1.1_2	PRMT2	ES	1.3	1.1	2	1.5E-10	0.108	-0.214	8.4E-01	3.2E-01	7.9E-01
OV	PRR13_AD_1.2_1.1_3	PRR13	AD	1.2	1.1	3	4.7E-12	0.124	-0.275	1.1E-01	2.8E-02	2.5E-01
OV	PRSS36_ES_12:13.1:13.2:14_11_15	PRSS36	ES	12:13.1:13.2:14	11	15	7.6E-12	0.138	0.033	7.8E-01	7.0E-01	9.0E-01
OV	PTBP2_ES_12_11.2_13	PTBP2	ES	12	11.2	13	1.3E-11	0.125	0.623	2.0E-01	1.8E-01	3.5E-01
OV	PTK2B_ES_28_27_29	PTK2B	ES	28	27	29	2.3E-29	0.284	0.034	5.2E-01	8.3E-01	8.4E-01
OV	QKI_AA_8.5_8.2_8.6	QKI	AA	8.5	8.2	8.6	8.8E-13	0.132	0.125	1.1E-01	7.9E-02	6.6E-01
OV	QKI_RI_8.3:8.4_8.2_8.5	QKI	RI	8.3:8.4	8.2	8.5	5.4E-14	0.150	-0.211	1.1E-01	4.1E-02	8.2E-01
OV	RABGGTA_RI_1.2:1.3_1.1_1.4	RABGGTA	RI	1.2:1.3	1.1	1.4	4.9E-10	0.103	0.086	6.1E-01	1.8E-01	4.8E-01
OV	RAI14_ES_18_17_19	RAI14	ES	18	17	19	2.6E-18	0.188	0.160	7.4E-01	7.2E-01	3.9E-01
OV	RALGPS2_ES_15_14_16	RALGPS2	ES	15	14	16	1.6E-09	0.112	-0.017	5.5E-02	5.1E-04	1.4E-01
OV	RBPJ_ES_4.2_2.3_5	RBPJ	ES	4.2	2.3	5	7.0E-10	0.101	-0.125	3.8E-01	9.0E-01	8.0E-01
OV	REPS1_ES_9.3_9.1_10	REPS1	ES	9.3	9.1	10	3.5E-12	0.125	0.359	6.3E-01	2.3E-01	4.6E-01
OV	RPS24_ES_5.2_4_6	RPS24	ES	5.2	4	6	1.7E-10	0.108	0.098	1.4E-01	2.0E-01	2.8E-01
OV	RTN2_ES_5_4.1_6	RTN2	ES	5	4.1	6	1.1E-12	0.134	0.305	4.7E-01	4.8E-01	4.9E-01
OV	RTN4_ES_6.1:6.2_5_8	RTN4	ES	6.1:6.2	5	8	7.3E-16	0.162	0.040	3.4E-01	2.0E-01	2.8E-01
OV	SCP2_ES_12_11_13	SCP2	ES	12	11	13	3.2E-13	0.136	-0.461	2.0E-01	4.7E-01	1.8E-01
OV	SDK1_ES_34_33_35	SDK1	ES	34	33	35	1.5E-08	0.106	0.265	3.4E-01	9.4E-02	3.7E-01
OV	SEC31A_ES_16:17_15_18	SEC31A	ES	16:17	15	18	2.2E-13	0.137	0.062	9.0E-01	1.5E-01	2.8E-01
OV	SEC31A_ES_26.1_25.1_28	SEC31A	ES	26.1	25.1	28	2.6E-37	0.347	-0.047	3.5E-01	8.5E-01	7.2E-01

cancer type	id	Gene Symbol	splice_type	Exon	From.Exon	To.Exon	anova.p	adj.r2	r	p.50	p.25	p.10
OV	SEC31A_ES_26.1:26.2_25.1_28	SEC31A	ES	26.1:26.2	25.1	28	6.2E-51	0.442	-0.036	4.9E-01	9.2E-01	4.6E-01
OV	SEC31A_ES_26.1:26.2:27_25.1_28	SEC31A	ES	26.1:26.2:27	25.1	28	1.3E-15	0.159	0.025	4.8E-01	4.5E-01	7.5E-01
OV	SEC31A_ES_26.1:27_25.1_28	SEC31A	ES	26.1:27	25.1	28	6.3E-13	0.165	-0.040	3.2E-01	5.1E-01	9.7E-01
OV	SEMA3F_ES_7_6_8	SEMA3F	ES	7	6	8	1.6E-12	0.129	0.329	3.1E-01	4.7E-01	7.2E-01
OV	SEPT6_ES_12_11.1_13.1	SEPT6	ES	12	11.1	13.1	1.5E-12	0.129	-0.147	1.7E-01	1.9E-01	9.5E-01
OV	SEPT6_ES_12_11.1_13.2	SEPT6	ES	12	11.1	13.2	2.1E-11	0.142	0.088	2.7E-01	3.6E-02	3.2E-01
OV	SEPT6_ES_12:13.1_11.1_13.2	SEPT6	ES	12:13.1	11.1	13.2	3.7E-16	0.166	-0.008	2.0E-01	6.3E-01	8.0E-01
OV	SLAIN2_ES_8_6_9	SLAIN2	ES	8	6	9	9.0E-13	0.131	-0.009	1.1E-03	4.1E-02	6.9E-02
OV	SLC35C1_RI_1.3_1.2_1.4	SLC35C1	RI	1.3	1.2	1.4	1.3E-11	0.119	-0.141	9.7E-02	1.7E-01	4.7E-01
OV	SLK_ES_13_12_14	SLK	ES	13	12	14	2.1E-11	0.118	0.129	1.8E-01	1.9E-01	3.0E-01
OV	SMARCB1_AD_2.2_2.1_3	SMARCB1	AD	2.2	2.1	3	1.1E-10	0.110	0.146	7.6E-01	2.6E-01	5.2E-01
OV	SORBS1_ES_9.1:9.2_8_10	SORBS1	ES	9.1:9.2	8	10	3.1E-19	0.218	0.078	7.3E-01	9.0E-01	8.2E-01
OV	SORBS2_ES_8:9.1:9.2_7_10	SORBS2	ES	8:9.1:9.2	7	10	3.5E-09	0.121	0.285	6.3E-01	4.4E-01	9.6E-01
OV	SOS1_ES_24_23_25	SOS1	ES	24	23	25	2.3E-13	0.137	-0.282	2.3E-01	1.2E-01	6.8E-02
OV	SPAG9_ES_30_29_31	SPAG9	ES	30	29	31	7.7E-34	0.329	-0.015	2.5E-01	1.6E-01	8.4E-02
OV	SPTAN1_ES_38_36.1_39	SPTAN1	ES	38	36.1	39	3.2E-13	0.136	0.143	2.8E-01	4.8E-01	7.8E-01
OV	STRADA_ES_3:4_2.2_6	STRADA	ES	3:04	2.2	6	1.0E-09	0.120	0.044	9.6E-01	8.4E-01	3.2E-01
OV	STX2_ES_10_9_11	STX2	ES	10	9	11	1.1E-12	0.151	0.222	7.9E-01	8.9E-01	4.7E-01
OV	SUCO_ES_4_3_5	SUCO	ES	4	3	5	1.4E-10	0.117	0.216	8.8E-01	9.8E-01	4.7E-01
OV	SUPT20H_ES_26_25_27	SUPT20H	ES	26	25	27	5.9E-10	0.102	0.156	9.1E-02	2.5E-01	9.7E-01
OV	SYNC_ES_4_3_5	SYNC	ES	4	3	5	4.3E-10	0.132	-0.090	1.0E+00	7.8E-01	6.8E-01
OV	SYNE1_ES_143_142_144	SYNE1	ES	143	142	144	7.6E-12	0.149	-0.042	7.7E-01	5.3E-01	2.6E-01
OV	SYTL2_ES_13_12.2_14	SYTL2	ES	13	12.2	14	5.9E-19	0.229	-0.106	7.7E-01	6.2E-01	8.9E-01
OV	TBC1D23_ES_15_14_16	TBC1D23	ES	15	14	16	6.0E-11	0.113	0.033	5.5E-01	8.1E-01	2.4E-01
OV	TCF12_ES_18_17_19	TCF12	ES	18	17	19	2.9E-10	0.109	0.236	5.4E-02	3.2E-01	6.3E-01
OV	TCF20_ES_4.1:4.2_3_5	TCF20	ES	4.1:4.2	3	5	1.1E-12	0.130	0.109	7.4E-01	6.1E-01	3.2E-01
OV	TCF7L2_ES_17_14_19	TCF7L2	ES	17	14	19	3.6E-10	0.104	-0.030	2.0E-03	3.7E-02	7.7E-01
OV	TCF7L2_ES_4_3_5	TCF7L2	ES	4	3	5	1.7E-10	0.108	0.063	1.3E-01	9.5E-01	7.3E-01
OV	TJP1_AD_30.2_30.1_31	TJP1	AD	30.2	30.1	31	6.6E-12	0.122	0.040	7.6E-01	5.2E-01	8.8E-01
OV	TJP1_ES_23_22_24	TJP1	ES	23	22	24	2.4E-10	0.106	-0.109	2.7E-02	1.9E-02	8.5E-01
OV	TMUB2_ES_4.4:4.5:4.7:4.8_4.3_5	TMUB2	ES	4.4:4.5:4.7:4.8	4.3	5	9.5E-10	0.105	0.020	7.2E-01	7.0E-01	1.0E+00
OV	TMUB2_ES_4.4:4.5:4.8_4.3_5	TMUB2	ES	4.4:4.5:4.8	4.3	5	3.3E-11	0.120	-0.039	3.9E-01	8.5E-01	8.2E-01
OV	TPD52L2_ES_4_3_5	TPD52L2	ES	4	3	5	2.7E-10	0.106	-0.056	3.5E-01	7.7E-01	5.5E-01
OV	TPM2_ES_7_6_8	TPM2	ES	7	6	8	8.3E-12	0.122	-0.428	2.5E-01	4.3E-02	5.7E-01
OV	TPM2_ME_6|7_5_8	TPM2	ME	6|7	5	8	4.8E-17	0.173	0.043	3.3E-02	1.2E-01	1.8E-01
OV	TRPM2_ES_29_28_30	TRPM2	ES	29	28	30	4.0E-11	0.116	0.013	1.7E-01	1.4E-01	5.7E-01
OV	TTC28_ES_20_19_21	TTC28	ES	20	19	21	7.1E-09	0.104	0.124	7.8E-01	3.2E-01	3.7E-01
OV	UBXN11_ES_4:5:6_2_7	UBXN11	ES	4:05:06	2	7	3.0E-10	0.107	0.028	1.4E-01	3.2E-01	5.3E-01
OV	UPP1_ES_5:6.1:6.2:7_4_9	UPP1	ES	5:6.1:6.2:7	4	9	3.8E-11	0.116	0.061	1.6E-01	2.8E-01	7.4E-01
OV	VPS29_AD_3.2_3.1_5	VPS29	AD	3.2	3.1	5	8.6E-12	0.121	-0.227	1.1E-01	2.1E-01	6.4E-01

cancer type	id	Gene Symbol	splice_type	Exon	From.Exon	To.Exon	anova.p	adj.r2	r	p.50	p.25	p.10
OV	VPS29_ES_3.1:3.2_1_5	VPS29	ES	3.1:3.2	1	5	3.4E-12	0.125	-0.076	7.1E-01	9.3E-01	9.5E-01
OV	WIBG_ES_4_2_6	WIBG	ES	4	2	6	4.1E-20	0.202	-0.265	1.5E-01	2.5E-01	2.3E-01
OV	WISP3_ES_5_4.2_6	WISP3	ES	5	4.2	6	2.7E-13	0.172	-0.252	1.9E-01	6.9E-01	8.9E-01
OV	WNK1_ES_15_13.2_16	WNK1	ES	15	13.2	16	8.4E-11	0.111	0.247	8.9E-02	1.3E-01	8.5E-02
OV	YBX3_ES_6_5_7	YBX3	ES	6	5	7	4.1E-15	0.155	-0.033	1.1E-01	4.2E-01	4.2E-01
OV	ZFAND5_ES_3_1_4.1	ZFAND5	ES	3	1	4.1	7.9E-11	0.111	-0.339	8.1E-01	4.5E-01	6.9E-01
PCPG	ABCG1_AD_16.2_16.1_17	ABCG1	AD	16.2	16.1	17	1.8E-04	0.103	0.065	6.3E-01	5.0E-01	2.7E-01
PCPG	ABI1_ES_5_4_7	ABI1	ES	5	4	7	8.6E-05	0.120	0.146	7.8E-02	4.7E-02	1.0E+00
PCPG	ABLIM2_ES_21:22_18.2_23	ABLIM2	ES	21:22	18.2	23	7.4E-05	0.125	-0.359	5.4E-01	4.7E-01	3.5E-01
PCPG	ACTN1_ES_20_19_21	ACTN1	ES	20	19	21	5.3E-07	0.181	0.292	9.9E-02	4.6E-02	1.2E-02
PCPG	ACTR3B_ES_2_1_3	ACTR3B	ES	2	1	3	2.7E-04	0.114	-0.237	7.6E-01	9.7E-01	3.6E-01
PCPG	ADAM15_ES_22.1_21.2_23	ADAM15	ES	22.1	21.2	23	2.3E-05	0.122	0.038	9.1E-01	3.2E-01	3.4E-01
PCPG	ADAM23_ME_25|26_24_27	ADAM23	ME	25|26	24	27	3.8E-06	0.142	-0.167	9.0E-01	2.0E-01	3.9E-01
PCPG	ADARB1_RI_13.2:13.3_13.1_13.4	ADARB1	RI	13.2:13.3	13.1	13.4	5.7E-09	0.207	0.285	1.8E-02	1.4E-01	1.0E+00
PCPG	ADD3_ES_15_14_16	ADD3	ES	15	14	16	1.9E-08	0.202	0.346	1.1E-01	5.3E-01	9.8E-01
PCPG	AHDC1_AA_4.1_3_4.2	AHDC1	AA	4.1	3	4.2	9.4E-05	0.134	0.025	1.6E-01	3.9E-01	9.6E-01
PCPG	AHDC1_ES_3_2_4.2	AHDC1	ES	3	2	4.2	1.6E-04	0.121	-0.009	2.7E-01	3.7E-01	1.3E-01
PCPG	AKAP17A_AD_4.2:4.3_4.1_5	AKAP17A	AD	4.2:4.3	4.1	5	6.6E-05	0.113	0.187	1.9E-01	9.3E-01	2.4E-01
PCPG	AKIP1_RI_1.2_1.1_1.3	AKIP1	RI	1.2	1.1	1.3	9.8E-05	0.107	-0.136	2.4E-02	3.2E-01	1.0E+00
PCPG	ALAS1_ES_2_1_3.1	ALAS1	ES	2	1	3.1	1.3E-04	0.111	0.242	1.6E-01	2.4E-01	2.9E-01
PCPG	AMDHD2_AD_8.2:8.3_8.1_9	AMDHD2	AD	8.2:8.3	8.1	9	9.4E-04	0.103	0.332	1.0E+00	3.2E-01	1.0E+00
PCPG	AMDHD2_ES_8.3_8.1_9	AMDHD2	ES	8.3	8.1	9	6.2E-08	0.231	0.271	3.5E-01	1.0E+00	1.0E+00
PCPG	AMPD2_ES_2.2_1_4	AMPD2	ES	2.2	1	4	1.4E-05	0.146	0.015	6.0E-01	9.2E-01	2.8E-01
PCPG	ANK2_ES_48_47_49	ANK2	ES	48	47	49	2.4E-07	0.177	-0.114	3.5E-03	8.6E-03	1.7E-01
PCPG	ANK2_ES_48:49:50_47_51	ANK2	ES	48:49:50	47	51	5.8E-05	0.143	-0.023	2.6E-01	1.3E-01	1.2E-01
PCPG	ANKRD11_ES_9_7_10	ANKRD11	ES	9	7	10	4.7E-08	0.192	0.029	6.2E-01	6.4E-01	5.3E-01
PCPG	AP2A1_ES_16_15_17	AP2A1	ES	16	15	17	3.3E-10	0.233	-0.485	8.2E-01	1.9E-01	1.0E+00
PCPG	APBB1_ES_13_12_14.2	APBB1	ES	13	12	14.2	2.4E-08	0.193	0.489	8.1E-01	1.5E-01	1.0E+00
PCPG	APBB3_AA_6.6:6.7:6.8_6.2_6.9	APBB3	AA	6.6:6.7:6.8	6.2	6.9	9.4E-05	0.110	0.235	2.0E-02	9.3E-02	2.8E-01
PCPG	APBB3_ES_6.6:6.8_6.2_6.9	APBB3	ES	6.6:6.8	6.2	6.9	5.4E-19	0.484	0.202	9.5E-02	6.6E-02	2.3E-01
PCPG	APBB3_RI_6.3:6.4:6.5_6.2_6.6	APBB3	RI	6.3:6.4:6.5	6.2	6.6	3.4E-11	0.254	-0.011	3.2E-01	3.3E-01	1.0E+00
PCPG	APEX1_AD_1.2_1.1_2.1	APEX1	AD	1.2	1.1	2.1	2.3E-04	0.109	0.108	6.0E-02	2.9E-01	3.5E-01
PCPG	APLP2_ES_16_15_17	APLP2	ES	16	15	17	1.6E-07	0.174	0.002	9.5E-01	3.2E-01	2.7E-01
PCPG	APOPT1_ES_2_1_5	APOPT1	ES	2	1	5	1.4E-05	0.146	-0.098	6.4E-01	2.5E-01	4.3E-01
PCPG	APP_ES_10_9_11	APP	ES	10	9	11	1.4E-11	0.262	-0.259	7.2E-02	5.5E-02	6.9E-02
PCPG	APP_ES_9:10_8_11	APP	ES	9:10	8	11	1.6E-05	0.126	-0.232	4.1E-02	2.3E-02	9.6E-02
PCPG	ARFGAP1_AA_14.1_12_14.2	ARFGAP1	AA	14.1	12	14.2	3.4E-09	0.211	-0.175	7.8E-01	3.2E-01	1.0E+00
PCPG	ARFGAP1_ES_10_9_11	ARFGAP1	ES	10	9	11	9.3E-09	0.204	0.038	5.2E-01	3.2E-01	1.0E+00
PCPG	ARFIP1_ES_4_3_5	ARFIP1	ES	4	3	5	6.4E-08	0.201	-0.285	1.3E-01	3.2E-01	3.2E-01
PCPG	ARHGAP17_ES_18_17_19	ARHGAP17	ES	18	17	19	2.3E-06	0.147	0.086	6.0E-01	1.4E-01	1.0E+00

cancer type	id	Gene Symbol	splice_type	Exon	From.Exon	To.Exon	anova.p	adj.r2	r	p.50	p.25	p.10
PCPG	ARHGAP26_AD_21.2_21.1_22	ARHGAP26	AD	21.2	21.1	22	2.7E-08	0.226	-0.422	7.4E-01	1.5E-01	1.4E-01
PCPG	ARHGAP44_ES_17_16_18	ARHGAP44	ES	17	16	18	1.0E-04	0.135	0.176	1.3E-01	2.0E-01	1.0E+00
PCPG	ARIH2_ES_5_4.2_6	ARIH2	ES	5	4.2	6	3.7E-05	0.128	0.163	5.3E-01	5.6E-01	9.8E-01
PCPG	ARL6IP4_AD_1.4_1.3_2.1	ARL6IP4	AD	1.4	1.3	2.1	1.6E-04	0.103	-0.168	9.8E-01	1.5E-01	1.0E+00
PCPG	ARMC10_ES_2_1_5.1	ARMC10	ES	2	1	5.1	4.5E-05	0.125	-0.199	7.0E-01	9.7E-01	9.8E-01
PCPG	ARMC4_ES_24_23_25	ARMC4	ES	24	23	25	2.8E-05	0.120	-0.802	4.5E-01	3.2E-01	5.3E-01
PCPG	ARMC6_AD_1.2_1.1_5	ARMC6	AD	1.2	1.1	5	1.6E-04	0.110	0.020	2.0E-01	1.0E+00	1.0E+00
PCPG	ARPC1B_ES_8_7_9	ARPC1B	ES	8	7	9	1.1E-04	0.105	-0.492	5.5E-01	2.8E-01	1.0E+00
PCPG	ARRB2_ES_2_1_4.2	ARRB2	ES	2	1	4.2	2.3E-06	0.152	0.003	5.1E-01	4.4E-01	3.2E-01
PCPG	ASCC2_ES_3_1_5	ASCC2	ES	3	1	5	6.2E-06	0.154	0.044	6.9E-01	2.5E-01	1.0E+00
PCPG	ASPH_ES_4_3_5	ASPH	ES	4	3	5	3.2E-18	0.406	-0.395	1.6E-01	9.9E-01	1.0E+00
PCPG	ASS1_ES_2_1_4	ASS1	ES	2	1	4	1.4E-05	0.164	-0.025	5.6E-01	9.7E-01	3.8E-01
PCPG	ASTN2_AA_10.1_9_10.2	ASTN2	AA	10.1	9	10.2	2.8E-05	0.138	-0.148	1.1E-01	3.5E-01	3.7E-01
PCPG	ATG13_ES_2.1:2.2_1.1_3	ATG13	ES	2.1:2.2	1.1	3	8.6E-04	0.103	-0.169	8.4E-01	9.1E-01	9.8E-01
PCPG	ATG4A_ES_3_2_4	ATG4A	ES	3	2	4	3.3E-04	0.106	-0.147	5.3E-01	9.7E-01	1.0E+00
PCPG	ATG4D_ES_4.1_3.2_5	ATG4D	ES	4.1	3.2	5	3.9E-07	0.195	-0.024	7.3E-01	8.6E-01	3.8E-01
PCPG	ATL2_ES_16:17.1_15_17.2	ATL2	ES	16:17.1	15	17.2	6.6E-20	0.411	0.069	3.6E-01	9.9E-01	1.0E+00
PCPG	ATP5A1_ES_3.3:4.2_3.2_6.1	ATP5A1	ES	3.3:4.2	3.2	6.1	4.4E-04	0.104	-0.209	1.4E-01	3.2E-01	1.0E+00
PCPG	ATP5G2_AD_1.2_1.1_3	ATP5G2	AD	1.2	1.1	3	5.5E-05	0.135	-0.010	9.8E-01	9.7E-01	7.6E-01
PCPG	ATP5G2_ES_2_1.2_3	ATP5G2	ES	2	1.2	3	1.6E-07	0.191	0.074	5.3E-01	6.0E-01	3.2E-01
PCPG	ATP5H_ES_3_1_4	ATP5H	ES	3	1	4	2.0E-06	0.162	-0.301	3.5E-02	3.9E-01	2.0E-01
PCPG	ATP5J_AD_1.2:1.3_1.1_3	ATP5J	AD	1.2:1.3	1.1	3	1.4E-06	0.152	-0.116	2.5E-01	6.5E-01	3.3E-01
PCPG	ATP5J_AD_1.2:1.3:1.4_1.1_3	ATP5J	AD	1.2:1.3:1.4	1.1	3	2.6E-07	0.171	-0.063	6.8E-01	9.3E-01	3.3E-01
PCPG	ATP5J_ES_1.2:1.3:1.4:1.5:2_1.1_3	ATP5J	ES	1.2:1.3:1.4:1.5:2	1.1	3	1.4E-05	0.128	0.014	7.0E-01	3.3E-01	3.8E-01
PCPG	ATP5J_ES_1.3:1.4:1.5:2_1.2_3	ATP5J	ES	1.3:1.4:1.5:2	1.2	3	1.8E-04	0.100	-0.087	1.1E-01	2.4E-01	4.8E-01
PCPG	ATP5J_ES_1.5:2_1.4_3	ATP5J	ES	1.5:2	1.4	3	5.8E-09	0.209	0.010	6.7E-01	1.7E-01	3.8E-01
PCPG	ATP9B_ES_30_29_31.1	ATP9B	ES	30	29	31.1	1.1E-06	0.157	0.112	8.1E-01	9.7E-01	3.2E-01
PCPG	ATPIF1_RI_2.2_2.1_2.3	ATPIF1	RI	2.2	2.1	2.3	5.9E-06	0.137	-0.343	2.0E-01	3.8E-01	3.1E-01
PCPG	ATXN2_ES_10_9_11	ATXN2	ES	10	9	11	3.2E-04	0.119	0.219	1.0E+00	3.4E-01	1.0E+00
PCPG	BBIP1_ES_3:4_1_5.1	BBIP1	ES	3:04	1	5.1	4.9E-07	0.164	0.050	9.6E-01	6.8E-01	9.4E-01
PCPG	BCKDHB_RI_11.2_11.1_11.3	BCKDHB	RI	11.2	11.1	11.3	1.6E-04	0.101	0.224	2.4E-01	3.2E-01	1.0E+00
PCPG	BCL2L1_AA_2.2_1_2.3	BCL2L1	AA	2.2	1	2.3	1.7E-06	0.162	0.002	2.2E-02	6.4E-02	4.1E-01
PCPG	BCL2L11_ES_5_3.1_7	BCL2L11	ES	5	3.1	7	4.8E-04	0.107	-0.468	5.7E-01	6.9E-01	7.8E-01
PCPG	BCLAF1_AA_5.1_4.2_5.2	BCLAF1	AA	5.1	4.2	5.2	3.3E-10	0.233	-0.202	4.4E-01	6.1E-01	2.9E-01
PCPG	BCS1L_ES_2_1.1_3	BCS1L	ES	2	1.1	3	5.2E-06	0.159	-0.072	1.9E-01	3.5E-01	1.2E-01
PCPG	BICD2_RI_7.2_7.1_7.3	BICD2	RI	7.2	7.1	7.3	3.4E-07	0.170	-0.005	6.6E-02	1.4E-01	1.5E-01
PCPG	BIN1_ES_13_12_17	BIN1	ES	13	12	17	3.8E-14	0.312	-0.076	2.5E-02	1.4E-01	1.0E+00
PCPG	BIN1_ES_13:14:15:16_12_17	BIN1	ES	13:14:15:16	12	17	5.3E-12	0.270	0.171	5.8E-01	9.4E-01	3.5E-01
PCPG	BIN1_ES_13:14:15:16:17_12_18	BIN1	ES	13:14:15:16:17	12	18	2.1E-06	0.148	0.125	6.1E-01	9.3E-01	1.0E+00
PCPG	BIN1_ES_13:16_12_17	BIN1	ES	13:16	12	17	8.4E-09	0.203	0.133	5.1E-01	8.7E-01	1.0E+00

cancer type	id	Gene Symbol	splice_type	Exon	From.Exon	To.Exon	anova.p	adj.r2	r	p.50	p.25	p.10
PCPG	BIN1_ES_13:16:17_12_18	BIN1	ES	13:16:17	12	18	6.6E-05	0.111	0.158	7.9E-01	1.7E-01	1.0E+00
PCPG	BIN1_ES_7_6_8	BIN1	ES	7	6	8	1.1E-05	0.130	0.127	4.4E-02	2.6E-01	1.0E+00
PCPG	BRD8_ES_10_9_11	BRD8	ES	10	9	11	1.3E-05	0.134	-0.274	5.0E-01	9.9E-01	1.0E+00
PCPG	BRD9_AA_9.1_8_9.2	BRD9	AA	9.1	8	9.2	2.9E-06	0.156	0.250	3.7E-02	3.4E-01	1.0E+00
PCPG	BRSK2_AA_24.2_23_24.3	BRSK2	AA	24.2	23	24.3	7.6E-05	0.112	-0.125	2.6E-01	1.3E-01	1.8E-01
PCPG	BUD31_AD_1.2_1.1_2	BUD31	AD	1.2	1.1	2	2.5E-07	0.169	-0.316	8.4E-02	1.3E-01	2.5E-01
PCPG	C11orf74_AD_1.2_1.1_4	C11orf74	AD	1.2	1.1	4	1.8E-04	0.101	0.092	1.7E-01	6.8E-02	1.7E-01
PCPG	C11orf88_RI_6.2_6.1_6.3	C11orf88	RI	6.2	6.1	6.3	4.0E-07	0.165	-0.840	4.4E-01	4.4E-01	4.9E-01
PCPG	C12orf29_ES_2:3_1_4	C12orf29	ES	2:03	1	4	2.8E-05	0.147	-0.125	6.2E-01	2.8E-01	5.4E-01
PCPG	C12orf29_ES_3_1_4	C12orf29	ES	3	1	4	7.6E-10	0.230	-0.271	5.7E-02	8.9E-02	3.2E-01
PCPG	C12orf76_ES_9.1_8_10.2	C12orf76	ES	9.1	8	10.2	5.6E-04	0.102	-0.050	6.2E-01	3.2E-01	7.1E-01
PCPG	C14orf2_ES_3:5_2_7.1	C14orf2	ES	3:05	2	7.1	2.3E-05	0.124	-0.195	1.3E-01	1.6E-01	4.0E-01
PCPG	C14orf2_ES_4:5_2_7.1	C14orf2	ES	4:05	2	7.1	2.1E-06	0.148	0.061	1.3E-01	2.2E-01	6.0E-01
PCPG	C14orf2_ME_3|4_2_5	C14orf2	ME	3|4	2	5	3.7E-06	0.146	-0.274	3.2E-01	3.2E-01	6.4E-01
PCPG	C14orf79_ES_2.4_2.2_3.2	C14orf79	ES	2.4	2.2	3.2	2.5E-04	0.119	-0.043	3.6E-01	3.0E-01	1.0E+00
PCPG	C1QC_AD_1.3_1.2_2	C1QC	AD	1.3	1.2	2	1.2E-05	0.166	-0.285	8.5E-01	1.0E+00	1.0E+00
PCPG	C21orf2_AD_6.3_6.2_7	C21orf2	AD	6.3	6.2	7	1.3E-07	0.176	-0.148	1.5E-02	3.3E-02	8.7E-02
PCPG	C2CD5_ES_26:27.1:27.2_25_28	C2CD5	ES	26:27.1:27.2	25	28	2.1E-10	0.241	-0.043	8.8E-02	3.2E-02	1.2E-01
PCPG	C2CD5_ES_26:27.2_25_28	C2CD5	ES	26:27.2	25	28	8.8E-10	0.241	-0.030	6.3E-01	1.2E-01	3.2E-01
PCPG	C2orf68_RI_1.2_1.1_1.3	C2orf68	RI	1.2	1.1	1.3	1.9E-05	0.139	-0.056	6.0E-01	4.1E-01	2.8E-01
PCPG	C4orf48_AD_1.2_1.1_2	C4orf48	AD	1.2	1.1	2	7.2E-06	0.147	0.021	6.5E-01	4.4E-02	3.2E-01
PCPG	CADM1_ES_9:10_8_12	CADM1	ES	9:10	8	12	4.3E-06	0.144	-0.390	8.6E-01	4.6E-01	4.3E-01
PCPG	CADM1_ES_9:10:11_8_12	CADM1	ES	9:10:11	8	12	8.3E-07	0.174	-0.408	7.8E-01	7.4E-01	1.1E-01
PCPG	CADPS2_ES_19_18_20	CADPS2	ES	19	18	20	1.3E-08	0.238	0.323	5.4E-01	9.8E-01	7.9E-01
PCPG	CALD1_ES_8.3:9_8.2_10	CALD1	ES	8.3:9	8.2	10	1.6E-07	0.174	0.193	2.1E-01	7.6E-01	1.0E+00
PCPG	CAMK2B_ES_18:19:20_17_21.1	CAMK2B	ES	18:19:20	17	21.1	1.3E-04	0.104	-0.073	8.0E-02	3.3E-01	1.0E+00
PCPG	CAMTA2_ES_21_20_22	CAMTA2	ES	21	20	22	8.1E-09	0.203	0.354	1.1E-01	3.0E-01	2.4E-01
PCPG	CASK_AA_24.1_23_24.2	CASK	AA	24.1	23	24.2	2.6E-11	0.315	-0.105	1.5E-02	1.5E-01	2.2E-01
PCPG	CAST_ES_10_9_11.2	CAST	ES	10	9	11.2	5.9E-06	0.142	0.062	7.8E-02	7.3E-02	1.4E-01
PCPG	CAST_ES_8.2_7.1_9	CAST	ES	8.2	7.1	9	5.9E-11	0.266	-0.168	6.1E-01	3.2E-01	1.0E+00
PCPG	CCDC136_AD_5.2_5.1_6	CCDC136	AD	5.2	5.1	6	6.9E-05	0.132	-0.126	2.3E-02	5.7E-02	1.1E-01
PCPG	CCDC136_ES_9_8_10	CCDC136	ES	9	8	10	5.3E-05	0.116	0.154	8.7E-01	1.7E-01	1.3E-01
PCPG	CCDC24_ES_4.1:4.2:5:6.1_3.2_6.2	CCDC24	ES	4.1:4.2:5:6.1	3.2	6.2	2.5E-04	0.101	-0.195	2.8E-01	1.5E-01	1.5E-01
PCPG	CCDC24_ES_4.2:5:6.1_3.2_6.2	CCDC24	ES	4.2:5:6.1	3.2	6.2	1.4E-04	0.112	-0.286	4.0E-01	5.3E-01	3.5E-01
PCPG	CCDC74B_AA_3.4_3.1_3.5	CCDC74B	AA	3.4	3.1	3.5	8.4E-07	0.170	0.074	2.5E-01	9.9E-01	3.4E-01
PCPG	CCNDBP1_RI_10.2_10.1_10.3	CCNDBP1	RI	10.2	10.1	10.3	1.2E-05	0.131	-0.384	4.7E-01	6.9E-01	1.0E+00
PCPG	CCNL1_ES_4.1_3_5	CCNL1	ES	4.1	3	5	3.1E-06	0.156	0.270	2.8E-01	1.5E-01	1.8E-01
PCPG	CCSER2_ES_11_10_12	CCSER2	ES	11	10	12	1.8E-05	0.131	-0.181	5.4E-02	7.3E-02	1.1E-01
PCPG	CD46_ES_7:8:9_6_10	CD46	ES	7:08:09	6	10	7.0E-06	0.159	-0.092	6.8E-01	1.0E+00	2.4E-01
PCPG	CD47_ES_9:10_8_11	CD47	ES	9:10	8	11	3.2E-16	0.349	0.407	6.5E-01	1.4E-01	1.0E+00

cancer type	id	Gene Symbol	splice_type	Exon	From.Exon	To.Exon	anova.p	adj.r2	r	p.50	p.25	p.10
PCPG	CD59_ES_3_1_5.4	CD59	ES	3	1	5.4	7.6E-07	0.158	0.144	3.1E-01	8.3E-02	1.0E+00
PCPG	CDC42BPA_ES_23_21_24	CDC42BPA	ES	23	21	24	5.2E-06	0.177	-0.340	7.6E-01	2.9E-01	3.9E-01
PCPG	CDIPT_ES_2.2_1.5_3.2	CDIPT	ES	2.2	1.5	3.2	1.1E-04	0.117	0.210	6.6E-01	3.4E-01	1.1E-01
PCPG	CDKL1_AA_9.1_8_9.2	CDKL1	AA	9.1	8	9.2	3.7E-05	0.145	-0.445	6.6E-01	1.0E+00	1.0E+00
PCPG	CEACAM1_ES_9_8_10	CEACAM1	ES	9	8	10	4.7E-04	0.108	0.069	3.0E-01	8.2E-02	3.1E-02
PCPG	CEP170_ES_10_9_11	CEP170	ES	10	9	11	3.2E-06	0.168	-0.196	8.1E-01	8.1E-01	1.0E+00
PCPG	CEP63_ES_16:17_15_18	CEP63	ES	16:17	15	18	1.8E-12	0.279	-0.074	3.9E-01	5.2E-01	3.4E-01
PCPG	CERS6_ES_10_9_11	CERS6	ES	10	9	11	4.0E-05	0.128	0.181	2.5E-01	8.7E-01	1.0E+00
PCPG	CHCHD3_AD_6.2_6.1_8	CHCHD3	AD	6.2	6.1	8	3.3E-09	0.224	0.394	1.4E-01	5.2E-01	6.8E-02
PCPG	CHD5_ES_40_39_41	CHD5	ES	40	39	41	6.8E-08	0.206	-0.070	5.3E-01	1.5E-01	3.5E-01
PCPG	CHGA_ES_2_1_4	CHGA	ES	2	1	4	1.2E-12	0.285	0.037	4.9E-01	1.5E-01	1.0E+00
PCPG	CHPT1_ES_5_4_6	CHPT1	ES	5	4	6	2.8E-10	0.241	-0.203	6.1E-01	5.1E-01	3.6E-01
PCPG	CHPT1_ES_6_4_7	CHPT1	ES	6	4	7	9.4E-08	0.183	0.294	7.7E-01	4.8E-01	2.6E-01
PCPG	CIRBP_AD_9.4:9.5:9.6_9.3_9.8	CIRBP	AD	9.4:9.5:9.6	9.3	9.8	3.4E-05	0.118	0.310	5.3E-01	1.4E-01	3.5E-01
PCPG	CLIP2_ES_9_8_10	CLIP2	ES	9	8	10	9.0E-06	0.148	0.281	9.2E-01	3.6E-01	1.0E+00
PCPG	CLSTN1_ES_3_2_4	CLSTN1	ES	3	2	4	4.5E-04	0.104	-0.174	7.7E-01	9.4E-01	1.0E+00
PCPG	CLTA_ES_6.1_4_7.1	CLTA	ES	6.1	4	7.1	4.4E-09	0.209	0.093	2.3E-01	5.2E-01	9.8E-01
PCPG	CLYBL_RI_9.2_9.1_9.3	CLYBL	RI	9.2	9.1	9.3	1.2E-08	0.200	-0.068	6.5E-01	4.6E-01	3.2E-01
PCPG	CMC2_ES_3_2_5	CMC2	ES	3	2	5	5.1E-05	0.114	-0.047	1.1E-01	1.1E-01	3.3E-01
PCPG	COQ3_ES_5_4_6	COQ3	ES	5	4	6	1.5E-07	0.174	-0.054	9.0E-01	5.3E-01	9.5E-01
PCPG	COX4I1_AA_5.1:5.2:5.3_4.1_5.4	COX4I1	AA	5.1:5.2:5.3	4.1	5.4	8.3E-07	0.173	-0.266	5.4E-01	5.9E-01	1.0E+00
PCPG	COX4I1_ES_4.2:5.1_4.1_5.4	COX4I1	ES	4.2:5.1	4.1	5.4	1.7E-06	0.150	-0.537	5.6E-01	5.6E-01	1.0E+00
PCPG	COX4I1_ES_4.2:5.1:5.2:5.3_4.1_5.4	COX4I1	ES	4.2:5.1:5.2:5.3	4.1	5.4	5.4E-05	0.113	-0.370	5.8E-01	5.8E-01	1.0E+00
PCPG	CPNE1_ES_1.2:2.1:2.2:3_1.1_5	CPNE1	ES	1.2:2.1:2.2:3	1.1	5	1.3E-04	0.103	-0.438	4.2E-01	3.3E-01	1.0E+00
PCPG	CPNE1_ES_1.2:2.2:3_1.1_5	CPNE1	ES	1.2:2.2:3	1.1	5	1.5E-06	0.155	-0.348	5.9E-01	9.8E-01	2.9E-01
PCPG	CPNE1_ES_2.2:3_1.2_5	CPNE1	ES	2.2:3	1.2	5	7.8E-05	0.110	-0.459	6.1E-01	5.5E-01	3.8E-01
PCPG	CPSF4_AA_9.1_8_9.2	CPSF4	AA	9.1	8	9.2	9.3E-05	0.107	0.072	5.1E-01	1.8E-01	1.0E+00
PCPG	CRAT_ES_2_1_4.1	CRAT	ES	2	1	4.1	2.0E-04	0.102	-0.150	3.3E-01	3.5E-01	3.2E-01
PCPG	CREM_ES_4:9.2:10.1:11_1_15	CREM	ES	4:9.2:10.1:11	1	15	1.0E-05	0.154	0.069	1.8E-02	5.9E-02	1.6E-01
PCPG	CREM_ES_9.2:10.1:11_4_15	CREM	ES	9.2:10.1:11	4	15	3.1E-05	0.137	0.201	3.0E-01	3.5E-01	2.5E-01
PCPG	CUTC_ES_7_6_8	CUTC	ES	7	6	8	4.2E-05	0.118	0.258	3.7E-01	2.8E-01	3.2E-01
PCPG	CXorf40B_AD_2.3_2.2_3	CXorf40B	AD	2.3	2.2	3	6.4E-08	0.185	0.183	1.3E-01	4.7E-02	1.0E+00
PCPG	CYP2R1_AA_6.1_5.3_6.2	CYP2R1	AA	6.1	5.3	6.2	1.1E-11	0.265	-0.203	3.6E-01	5.5E-01	9.8E-01
PCPG	DCAF6_ES_11_10_13.1	DCAF6	ES	11	10	13.1	1.6E-05	0.148	-0.182	8.0E-01	5.6E-01	3.9E-01
PCPG	DCAF6_ES_11:13.1_10_14	DCAF6	ES	11:13.1	10	14	2.7E-05	0.132	-0.187	8.9E-01	5.4E-01	3.2E-01
PCPG	DCAF8_ES_7.2:7.3:8.1:8.2_7.1_9	DCAF8	ES	7.2:7.3:8.1:8.2	7.1	9	8.8E-06	0.149	0.098	1.8E-01	5.5E-01	1.5E-01
PCPG	DCAF8_ES_7.2:8.1:8.2_7.1_9	DCAF8	ES	7.2:8.1:8.2	7.1	9	2.5E-05	0.136	-0.023	7.0E-01	6.0E-01	2.8E-01
PCPG	DCAF8_ES_8.1:8.2_7.1_9	DCAF8	ES	8.1:8.2	7.1	9	1.0E-04	0.120	-0.009	1.8E-01	5.8E-01	1.0E+00
PCPG	DCLK2_ES_5_4_6	DCLK2	ES	5	4	6	8.7E-04	0.100	0.249	3.1E-01	5.8E-01	1.3E-01
PCPG	DCN_ES_2_1_3	DCN	ES	2	1	3	1.6E-06	0.170	-0.390	1.3E-01	3.4E-01	1.0E+00

cancer type	id	Gene Symbol	splice_type	Exon	From.Exon	To.Exon	anova.p	adj.r2	r	p.50	p.25	p.10
PCPG	DCTN1_ES_27_26_28	DCTN1	ES	27	26	28	2.2E-07	0.171	0.524	6.9E-01	9.8E-01	3.3E-01
PCPG	DCTN1_ES_6:7.2:8_5_9	DCTN1	ES	07:02.2	5	9	8.8E-05	0.117	0.480	2.5E-02	7.1E-02	1.0E+00
PCPG	DCTN2_ES_7:8_2_10	DCTN2	ES	7:08	2	10	4.7E-06	0.145	0.244	7.1E-01	8.9E-01	9.9E-01
PCPG	DCTN2_ES_8_2_10	DCTN2	ES	8	2	10	2.8E-12	0.276	0.129	6.6E-02	3.4E-01	3.8E-01
PCPG	DGUOK_ES_4:5_1_7	DGUOK	ES	4:05	1	7	1.1E-07	0.178	-0.111	9.0E-02	1.6E-01	3.2E-01
PCPG	DGUOK_ES_5_4_7	DGUOK	ES	5	4	7	6.2E-07	0.160	0.086	4.8E-01	2.1E-01	1.0E+00
PCPG	DHRS12_RI_10.2_10.1_10.3	DHRS12	RI	10.2	10.1	10.3	1.7E-05	0.126	-0.237	1.0E+00	6.6E-01	7.5E-01
PCPG	DHX30_ES_6_5_10	DHX30	ES	6	5	10	1.1E-05	0.155	0.122	2.1E-01	3.5E-01	1.0E+00
PCPG	DMD_ES_75:76:77:78_74_79	DMD	ES	75:76:77:78	74	79	9.8E-04	0.101	-0.343	4.6E-01	4.8E-01	6.5E-01
PCPG	DNAJB2_RI_9.2_9.1_9.3	DNAJB2	RI	9.2	9.1	9.3	5.5E-07	0.162	-0.351	9.7E-01	5.2E-01	3.5E-01
PCPG	DNAJC19_ES_5.1_4_6	DNAJC19	ES	5.1	4	6	1.9E-07	0.174	-0.068	9.4E-01	3.2E-01	1.0E+00
PCPG	DNAJC5_ES_5_4_6	DNAJC5	ES	5	4	6	1.7E-07	0.173	-0.356	1.1E-01	3.5E-01	1.9E-01
PCPG	DNM2_ES_15_14_17	DNM2	ES	15	14	17	3.5E-09	0.211	0.088	3.7E-01	9.7E-01	1.0E+00
PCPG	DNM2_ME_10|11_9_12	DNM2	ME	10|11	9	12	2.3E-05	0.122	-0.088	3.6E-01	6.4E-01	1.0E+00
PCPG	DNM3_ES_19_18.1_20	DNM3	ES	19	18.1	20	7.7E-04	0.101	0.319	6.8E-02	1.1E-01	9.8E-01
PCPG	DOCK9_ES_48_47_49	DOCK9	ES	48	47	49	3.0E-05	0.139	0.217	7.0E-01	9.1E-01	9.3E-01
PCPG	DRAM2_ES_5_4_7	DRAM2	ES	5	4	7	1.0E-05	0.149	0.261	6.3E-01	3.2E-01	1.0E+00
PCPG	DST_ES_106_105_107	DST	ES	106	105	107	1.7E-08	0.196	-0.203	8.3E-01	9.7E-01	1.0E+00
PCPG	DST_ES_43:44:45:46.1:48_42_49	DST	ES	43:44:45:46.1:48	42	49	3.0E-04	0.112	-0.358	7.2E-01	9.9E-01	1.2E-01
PCPG	DST_ES_70_69_71	DST	ES	70	69	71	2.4E-04	0.101	-0.114	8.7E-01	8.6E-01	9.3E-02
PCPG	DTNA_ES_16_13_17	DTNA	ES	16	13	17	1.1E-08	0.241	0.178	8.6E-01	5.1E-01	3.1E-01
PCPG	DUSP12_AA_3.1_1.2_3.2	DUSP12	AA	3.1	1.2	3.2	1.7E-04	0.109	0.168	7.0E-01	3.2E-01	1.0E+00
PCPG	DVL1_AD_11.2_11.1_12	DVL1	AD	11.2	11.1	12	9.2E-06	0.132	0.083	6.7E-01	1.9E-01	3.2E-01
PCPG	DYNC1I2_ES_8_7.3_9	DYNC1I2	ES	8	7.3	9	2.6E-10	0.237	0.269	1.8E-01	3.4E-01	1.0E+00
PCPG	DYRK1B_AA_10.1:10.2_9_10.3	DYRK1B	AA	10.1:10.2	9	10.3	1.0E-05	0.132	-0.013	4.4E-01	2.5E-01	7.9E-02
PCPG	EEF1D_ES_5:7.2_1_8.1	EEF1D	ES	05:07.2	1	8.1	2.9E-13	0.295	-0.568	6.6E-02	1.4E-01	2.9E-01
PCPG	EEF1D_ES_5:7.2_1_8.2	EEF1D	ES	05:07.2	1	8.2	5.9E-07	0.166	-0.514	4.9E-02	1.6E-01	3.2E-01
PCPG	EEF1D_ES_5:7.2:8.1_1_8.2	EEF1D	ES	5:7.2:8.1	1	8.2	1.8E-14	0.318	-0.586	6.2E-02	7.1E-02	3.4E-01
PCPG	EEF1D_ES_6:7.2:8.1_1_8.2	EEF1D	ES	6:7.2:8.1	1	8.2	2.3E-06	0.147	-0.322	3.3E-01	1.3E-01	1.0E+00
PCPG	EFCAB14_ES_7_6_8	EFCAB14	ES	7	6	8	6.6E-07	0.163	-0.225	1.3E-02	2.2E-01	1.0E+00
PCPG	EGFLAM_ES_20.2_19_21.2	EGFLAM	ES	20.2	19	21.2	1.8E-08	0.212	0.475	2.4E-01	1.6E-01	2.9E-01
PCPG	EHBP1_ES_18_17_19	EHBP1	ES	18	17	19	4.1E-09	0.213	0.333	9.1E-01	8.6E-01	2.9E-01
PCPG	EIF4G1_ES_4_3.2_5	EIF4G1	ES	4	3.2	5	4.2E-04	0.107	-0.034	1.6E-02	1.4E-02	3.2E-02
PCPG	ELK1_ES_2_1_3	ELK1	ES	2	1	3	4.6E-08	0.199	-0.043	5.6E-01	9.1E-01	1.0E+00
PCPG	ELP5_RI_7.4_7.3_7.5	ELP5	RI	7.4	7.3	7.5	3.7E-07	0.166	-0.050	7.3E-01	4.9E-01	1.5E-01
PCPG	EMC9_AA_3.2_2_3.3	EMC9	AA	3.2	2	3.3	1.8E-05	0.125	-0.225	3.5E-01	3.2E-01	3.2E-01
PCPG	EML4_ES_4_3_5	EML4	ES	4	3	5	9.6E-05	0.118	-0.421	5.4E-01	7.6E-01	9.1E-01
PCPG	EPB41L1_ES_18_17_19	EPB41L1	ES	18	17	19	6.8E-15	0.326	-0.100	9.8E-01	3.2E-01	1.0E+00
PCPG	EPB41L2_ES_17:18_14_20.1	EPB41L2	ES	17:18	14	20.1	1.1E-04	0.118	0.110	2.9E-01	5.8E-01	4.1E-01
PCPG	EPB41L3_ES_16_15_17	EPB41L3	ES	16	15	17	5.7E-08	0.186	0.254	6.0E-01	9.1E-01	5.6E-01

cancer type	id	Gene Symbol	splice_type	Exon	From.Exon	To.Exon	anova.p	adj.r2	r	p.50	p.25	p.10
PCPG	ERBB2IP_ES_24.1:24.2:24.3_21_25	ERBB2IP	ES	24.1:24.2:24.3	21	25	1.5E-04	0.108	-0.225	8.4E-01	7.6E-01	3.2E-01
PCPG	ERC1_ES_20_19_21	ERC1	ES	20	19	21	1.3E-08	0.248	0.225	7.5E-01	5.2E-01	1.0E+00
PCPG	ERGIC3_ES_10_8_12	ERGIC3	ES	10	8	12	4.2E-13	0.292	-0.148	7.5E-01	1.4E-01	3.0E-01
PCPG	EXOC1_ES_11_10_12	EXOC1	ES	11	10	12	9.3E-07	0.165	0.415	7.1E-02	1.9E-01	5.7E-01
PCPG	FAM110A_ES_3_1_4.2	FAM110A	ES	3	1	4.2	5.8E-05	0.113	-0.028	8.6E-01	4.7E-01	3.2E-01
PCPG	FAM122B_AA_10.1_9_10.2	FAM122B	AA	10.1	9	10.2	2.6E-06	0.146	-0.472	9.8E-01	3.2E-01	1.0E+00
PCPG	FAM195A_ES_3_2_4	FAM195A	ES	3	2	4	1.4E-08	0.198	-0.054	1.5E-01	2.3E-01	3.6E-01
PCPG	FAM228B_ES_4_3_5	FAM228B	ES	4	3	5	1.3E-06	0.160	0.073	2.7E-01	9.9E-01	1.0E+00
PCPG	FAM228B_ES_4:5:6:7:8_3_9.1	FAM228B	ES	4:5:6:7:8	3	9.1	1.6E-12	0.280	-0.106	5.8E-01	6.8E-01	5.5E-01
PCPG	FAM228B_ES_5:6:7:8_3_9.1	FAM228B	ES	5:6:7:8	3	9.1	9.6E-06	0.138	-0.094	7.0E-01	5.9E-01	3.2E-01
PCPG	FAM47E_ES_4_3_5	FAM47E	ES	4	3	5	2.1E-04	0.100	-0.412	4.1E-01	5.4E-02	3.2E-01
PCPG	FAM65A_ES_2_1_4	FAM65A	ES	2	1	4	5.4E-05	0.122	0.020	5.7E-01	1.0E+00	1.0E+00
PCPG	FAM73B_AD_15.3_15.2_16	FAM73B	AD	15.3	15.2	16	9.7E-05	0.106	0.023	1.5E-01	5.3E-01	2.1E-01
PCPG	FAM86B1_ES_5:6:7.1:7.2:7.3_4_8.1	FAM86B1	ES	5:6:7.1:7.2:7.3	4	8.1	2.4E-05	0.123	0.194	6.9E-01	4.8E-01	3.3E-01
PCPG	FAM86B1_ES_5:6:7.1:7.3_4_8.1	FAM86B1	ES	5:6:7.1:7.3	4	8.1	3.1E-04	0.120	0.065	9.6E-01	8.8E-01	1.0E+00
PCPG	FAM86B1_ES_7.1:7.2:7.3_4_8.1	FAM86B1	ES	7.1:7.2:7.3	4	8.1	1.9E-07	0.196	-0.057	7.3E-01	6.5E-01	1.0E+00
PCPG	FAM86C1_AA_3.1_2_3.2	FAM86C1	AA	3.1	2	3.2	1.3E-05	0.159	-0.138	1.4E-01	6.1E-02	1.0E+00
PCPG	FAM86C1_ES_3.2_2_5.1	FAM86C1	ES	3.2	2	5.1	2.6E-04	0.100	-0.114	4.2E-01	1.6E-01	1.0E+00
PCPG	FAM86C1_ES_4_3.2_5.1	FAM86C1	ES	4	3.2	5.1	1.8E-09	0.231	0.052	6.9E-01	1.8E-01	1.0E+00
PCPG	FBLN5_ES_7_5_8	FBLN5	ES	7	5	8	4.1E-05	0.116	-0.577	5.2E-01	5.3E-01	3.5E-01
PCPG	FBXO38_ES_15.1_14_16	FBXO38	ES	15.1	14	16	3.7E-04	0.103	-0.050	1.3E-01	3.5E-01	1.0E+00
PCPG	FDPS_ES_2:3.1:3.2_1.1_4	FDPS	ES	2:3.1:3.2	1.1	4	9.9E-07	0.196	0.294	7.0E-01	9.8E-01	1.0E+00
PCPG	FEZ2_ES_8_7_9	FEZ2	ES	8	7	9	1.3E-05	0.132	0.217	8.2E-01	1.5E-01	2.6E-01
PCPG	FGFR1_ES_6_4_8.2	FGFR1	ES	6	4	8.2	4.2E-08	0.192	0.316	9.2E-01	1.9E-01	1.0E+00
PCPG	FGFR1OP_ES_7_6_8	FGFR1OP	ES	7	6	8	1.0E-04	0.114	-0.103	6.1E-02	4.6E-02	2.3E-01
PCPG	FGFR1OP2_ES_5.1_4_6	FGFR1OP2	ES	5.1	4	6	7.0E-05	0.116	-0.219	9.3E-01	9.2E-01	5.2E-01
PCPG	FHL1_ES_8:9_7_10	FHL1	ES	8:09	7	10	2.0E-07	0.176	0.094	6.7E-01	1.0E+00	3.3E-01
PCPG	FHOD3_ES_17_16_18	FHOD3	ES	17	16	18	4.5E-07	0.206	-0.458	2.3E-01	2.9E-01	1.0E+00
PCPG	FLNA_ES_30_29_31	FLNA	ES	30	29	31	2.5E-08	0.198	-0.422	4.7E-01	2.9E-01	2.7E-01
PCPG	FLNB_ES_32.1:32.2_31_33	FLNB	ES	32.1:32.2	31	33	6.8E-08	0.215	-0.324	1.5E-01	1.3E-01	1.7E-01
PCPG	FMNL3_ES_26_25_27	FMNL3	ES	26	25	27	1.0E-08	0.203	0.309	2.8E-01	2.1E-01	7.5E-01
PCPG	FN1_ES_25_24_26	FN1	ES	25	24	26	5.7E-10	0.243	-0.013	4.0E-01	9.1E-01	9.4E-01
PCPG	FNBP1L_RI_16.2_16.1_16.3	FNBP1L	RI	16.2	16.1	16.3	2.3E-07	0.173	-0.236	1.9E-01	3.6E-01	1.0E+00
PCPG	FRMD4A_ES_30_29_31	FRMD4A	ES	30	29	31	1.4E-05	0.162	0.237	5.4E-01	5.8E-01	5.7E-01
PCPG	FTSJ2_ES_4_2_5	FTSJ2	ES	4	2	5	1.4E-04	0.103	-0.012	5.7E-01	1.9E-01	3.6E-01
PCPG	FUBP1_ES_3_2_4	FUBP1	ES	3	2	4	1.0E-04	0.115	-0.159	5.8E-01	5.0E-01	8.4E-01
PCPG	FUZ_ES_2.1:2.2:2.3:2.4_1.2_3	FUZ	ES	2.1:2.2:2.3:2.4	1.2	3	2.2E-05	0.154	0.088	4.4E-01	1.5E-01	3.2E-01
PCPG	FXYD1_RI_10.2_10.1_10.3	FXYD1	RI	10.2	10.1	10.3	2.3E-06	0.180	-0.127	6.1E-01	6.7E-01	1.0E+00
PCPG	GABARAP_AD_1.2:1.3:1.4:1.5_1.1_2.1	GABARAP	AD	1.2:1.3:1.4:1.5	1.1	2.1	7.3E-13	0.287	-0.044	6.1E-01	3.4E-01	1.0E+00
PCPG	GABARAP_ES_1.2:1.3:1.5_1.1_2.1	GABARAP	ES	1.2:1.3:1.5	1.1	2.1	1.1E-07	0.181	0.030	8.1E-01	1.0E+00	1.0E+00

cancer type	id	Gene Symbol	splice_type	Exon	From.Exon	To.Exon	anova.p	adj.r2	r	p.50	p.25	p.10
PCPG	GALT_ES_3.1:3.2:3.3:4_2_5	GALT	ES	3.1:3.2:3.3:4	2	5	1.3E-06	0.159	-0.086	1.4E-01	1.5E-01	2.7E-01
PCPG	GALT_ES_3.3:4_2_5	GALT	ES	3.3:4	2	5	1.3E-09	0.252	-0.151	4.6E-01	5.2E-01	5.2E-01
PCPG	GCH1_ES_6.1_5_6.5	GCH1	ES	6.1	5	6.5	6.4E-08	0.209	-0.022	7.6E-01	8.0E-01	8.0E-01
PCPG	GEMIN8_ES_2_1_3	GEMIN8	ES	2	1	3	8.4E-12	0.277	-0.106	9.4E-01	5.0E-01	1.0E+00
PCPG	GGCT_ES_4_3_5	GGCT	ES	4	3	5	2.1E-09	0.216	0.153	6.1E-01	7.7E-01	8.9E-01
PCPG	GINS3_ES_3_1_4	GINS3	ES	3	1	4	8.9E-05	0.119	-0.051	6.1E-01	1.5E-01	3.2E-01
PCPG	GIPC1_ES_4_3_5	GIPC1	ES	4	3	5	9.4E-08	0.179	0.042	7.5E-01	7.1E-01	3.6E-01
PCPG	GIT1_ES_8_7_9	GIT1	ES	8	7	9	2.5E-04	0.102	0.146	5.0E-01	5.2E-01	1.0E+00
PCPG	GIT2_ES_18.1:18.2:19_17.2_20	GIT2	ES	18.1:18.2:19	17.2	20	2.9E-07	0.173	-0.111	9.6E-01	8.0E-01	5.3E-01
PCPG	GIT2_ES_18.2:19_17.2_20	GIT2	ES	18.2:19	17.2	20	1.6E-05	0.152	0.007	8.1E-01	7.3E-01	4.9E-01
PCPG	GK_ES_23_22_24	GK	ES	23	22	24	1.3E-08	0.199	-0.353	4.1E-01	4.8E-01	4.8E-01
PCPG	GLIPR1_RI_5.2_5.1_5.3	GLIPR1	RI	5.2	5.1	5.3	1.5E-04	0.103	-0.585	8.4E-01	2.1E-01	1.0E+00
PCPG	GNAS_ES_6_5_7	GNAS	ES	6	5	7	1.7E-18	0.397	-0.439	6.0E-01	1.6E-01	3.8E-01
PCPG	GNB2L1_ES_4.1:4.2:5:6:7.2:8.1:8.2_3_9	GNB2L1	ES	l:4.2:5:6:7.2:8.1:8	3	9	1.9E-05	0.125	-0.416	4.0E-01	3.5E-01	9.0E-02
PCPG	GOLGA4_ES_24_23_25	GOLGA4	ES	24	23	25	4.6E-07	0.163	0.239	5.3E-01	9.6E-01	3.3E-01
PCPG	GPS1_RI_1.2_1.1_1.3	GPS1	RI	1.2	1.1	1.3	4.7E-06	0.157	0.258	5.5E-01	1.5E-01	2.7E-01
PCPG	GRAMD1A_ES_19_18_20	GRAMD1A	ES	19	18	20	2.2E-08	0.194	-0.253	7.7E-01	1.2E-01	1.0E+00
PCPG	GRIA2_ES_18_17_19	GRIA2	ES	18	17	19	3.2E-05	0.126	0.310	2.4E-01	8.8E-01	7.2E-01
PCPG	GRIA4_ME_16|17_15_18.1	GRIA4	ME	16|17	15	18.1	2.5E-04	0.111	-0.005	1.9E-01	9.3E-01	3.9E-01
PCPG	GSN_ES_15.1:15.2_10_16	GSN	ES	15.1:15.2	10	16	1.9E-04	0.101	0.131	2.0E-01	7.5E-01	8.2E-01
PCPG	GSTK1_AD_4.2:4.3_4.1_5	GSTK1	AD	4.2:4.3	4.1	5	8.0E-05	0.109	0.165	2.8E-01	2.6E-01	7.0E-01
PCPG	GTF2H2C_ES_2_1.2_3	GTF2H2C	ES	2	1.2	3	2.8E-04	0.123	-0.250	5.4E-01	9.7E-01	1.0E+00
PCPG	GUSB_ES_6_5.1_7	GUSB	ES	6	5.1	7	4.9E-06	0.139	-0.006	2.0E-01	5.9E-01	9.4E-01
PCPG	HEMK1_AD_1.4_1.3_2.1	HEMK1	AD	1.4	1.3	2.1	2.4E-04	0.114	0.102	6.7E-01	4.7E-02	1.0E+00
PCPG	HERC4_ES_19_18_20	HERC4	ES	19	18	20	1.8E-08	0.213	-0.058	1.7E-01	7.2E-02	2.8E-01
PCPG	HHLA3_ES_2.1:2.2_1.1_3	HHLA3	ES	2.1:2.2	1.1	3	7.7E-05	0.113	-0.423	2.3E-01	2.9E-01	9.5E-02
PCPG	HMBS_AD_1.2_1.1_4	HMBS	AD	1.2	1.1	4	3.1E-04	0.100	0.171	1.7E-01	5.8E-02	1.5E-01
PCPG	HMGB1_RI_7.2_7.1_7.3	HMGB1	RI	7.2	7.1	7.3	1.1E-06	0.156	0.035	6.4E-01	1.6E-01	3.9E-01
PCPG	HNRNPA2B1_AA_12.1:12.2:12.3_11_12.	HNRNPA2B1	AA	12.1:12.2:12.3	11	12.4	5.2E-04	0.109	0.310	4.4E-01	9.7E-01	3.4E-01
PCPG	HNRNPC_ES_2.2:2.3:2.4_1_3.2	HNRNPC	ES	2.2:2.3:2.4	1	3.2	8.4E-07	0.161	-0.091	6.4E-01	4.7E-01	9.4E-01
PCPG	HOOK2_ES_18_17_19	HOOK2	ES	18	17	19	1.4E-10	0.261	0.035	6.5E-01	9.7E-01	1.0E+00
PCPG	HSD11B1L_ES_5.2:5.3_4.2_8	HSD11B1L	ES	5.2:5.3	4.2	8	5.6E-04	0.108	-0.042	9.2E-01	9.8E-01	8.1E-01
PCPG	HSD17B14_ES_8_7_9	HSD17B14	ES	8	7	9	4.7E-09	0.209	-0.111	5.6E-01	4.4E-01	2.7E-01
PCPG	HUS1_RI_9.2_9.1_9.3	HUS1	RI	9.2	9.1	9.3	8.9E-06	0.133	-0.404	1.4E-01	5.0E-02	3.9E-01
PCPG	ICA1_ES_15_14_16	ICA1	ES	15	14	16	2.3E-09	0.216	0.260	2.7E-01	1.7E-01	1.0E+00
PCPG	IDI1_ES_2_1_3	IDI1	ES	2	1	3	6.6E-09	0.220	0.486	6.7E-01	9.3E-02	2.3E-01
PCPG	IL18BP_AD_1.2:1.3:1.4:1.5:1.6_1.1_1.8	IL18BP	AD	1.2:1.3:1.4:1.5:1.6	1.1	1.8	1.8E-06	0.168	0.013	5.9E-01	8.9E-01	1.0E+00
PCPG	IL18BP_RI_1.2:1.3:1.4:1.5:1.6:1.7_1.1_1.	IL18BP	RI	2:1.3:1.4:1.5:1.6:1	1.1	1.8	9.1E-04	0.104	-0.091	3.5E-01	1.0E+00	1.0E+00
PCPG	IL18BP_RI_1.7_1.6_1.8	IL18BP	RI	1.7	1.6	1.8	4.5E-05	0.120	-0.491	4.8E-01	2.4E-01	1.0E+00
PCPG	INADL_ES_46_45_47.1	INADL	ES	46	45	47.1	7.5E-07	0.173	-0.057	5.3E-01	3.4E-01	3.9E-01

cancer type	id	Gene Symbol	splice_type	Exon	From.Exon	To.Exon	anova.p	adj.r2	r	p.50	p.25	p.10
PCPG	INO80C_ES_3:4.2_1_5.1	INO80C	ES	03:04.2	1	5.1	5.6E-05	0.113	-0.053	4.8E-01	7.6E-01	2.6E-01
PCPG	INPP4A_AD_18.2_18.1_19	INPP4A	AD	18.2	18.1	19	1.6E-10	0.298	-0.256	9.5E-01	9.8E-01	3.5E-01
PCPG	INTS12_ES_4_1_5	INTS12	ES	4	1	5	5.8E-06	0.152	-0.246	7.0E-01	4.7E-01	8.0E-01
PCPG	IP6K2_AD_11.6:11.7_11.5_11.9	IP6K2	AD	11.6:11.7	11.5	11.9	6.6E-05	0.121	0.086	6.5E-01	4.7E-01	4.6E-01
PCPG	IPO11_ES_30_29_33	IPO11	ES	30	29	33	9.3E-06	0.155	0.299	5.8E-01	5.3E-01	1.0E+00
PCPG	ITGB3BP_ES_9_8_10	ITGB3BP	ES	9	8	10	7.8E-05	0.110	-0.208	7.9E-01	6.7E-01	1.0E+00
PCPG	JPH4_ES_2_1_3	JPH4	ES	2	1	3	1.4E-05	0.142	0.242	6.1E-01	2.6E-01	3.2E-01
PCPG	KARS_ES_2_1_3	KARS	ES	2	1	3	3.1E-05	0.124	-0.166	5.1E-01	1.9E-01	1.0E+00
PCPG	KBTBD3_ES_2_1_3.2	KBTBD3	ES	2	1	3.2	1.4E-08	0.198	-0.269	9.1E-01	5.4E-01	5.6E-01
PCPG	KIAA0513_ES_11_10_12	KIAA0513	ES	11	10	12	7.7E-05	0.123	0.240	1.7E-01	3.5E-01	1.7E-01
PCPG	KIAA1217_ES_23:24_22_25.1	KIAA1217	ES	23:24	22	25.1	7.8E-05	0.112	-0.046	4.9E-01	3.2E-01	1.0E+00
PCPG	KIAA1217_RI_25.2_25.1_25.3	KIAA1217	RI	25.2	25.1	25.3	6.9E-05	0.136	0.195	7.5E-01	1.2E-01	3.9E-01
PCPG	KIF1A_ES_27_26_28	KIF1A	ES	27	26	28	9.6E-06	0.138	0.235	9.0E-01	5.3E-01	9.9E-01
PCPG	KIF3A_ES_11_9_12	KIF3A	ES	11	9	12	1.0E-05	0.146	0.183	6.4E-01	2.6E-01	1.0E+00
PCPG	KLC1_RI_14.2_14.1_14.3	KLC1	RI	14.2	14.1	14.3	2.6E-06	0.146	-0.217	1.0E-01	4.7E-02	3.3E-01
PCPG	LETMD1_ES_2:3.2_1.2_7	LETMD1	ES	02:03.2	1.2	7	9.5E-07	0.167	-0.023	2.4E-02	2.1E-01	3.4E-01
PCPG	LETMD1_ES_2:3.2:4:5:6_1.2_7	LETMD1	ES	2:3.2:4:5:6	1.2	7	1.1E-05	0.133	-0.072	1.7E-01	5.5E-02	4.1E-01
PCPG	LETMD1_ES_2:4:5:6_1.2_7	LETMD1	ES	2:4:5:6	1.2	7	2.1E-04	0.103	-0.019	4.3E-01	3.7E-01	1.0E+00
PCPG	LETMD1_ES_4_2_5	LETMD1	ES	4	2	5	1.7E-05	0.158	0.089	6.1E-02	1.6E-01	3.0E-01
PCPG	LMAN2L_ES_6_5_7	LMAN2L	ES	6	5	7	1.7E-04	0.112	0.406	1.7E-02	9.3E-02	1.4E-01
PCPG	LRIF1_ES_2.2:2.3_1_3	LRIF1	ES	2.2:2.3	1	3	9.5E-08	0.186	-0.144	6.1E-01	5.9E-01	1.0E+00
PCPG	LRP8_ES_17_16_18	LRP8	ES	17	16	18	3.0E-04	0.109	-0.136	6.6E-01	9.3E-01	4.5E-01
PCPG	LRRC23_ES_5_4_6	LRRC23	ES	5	4	6	1.3E-04	0.105	0.144	1.4E-01	5.5E-01	3.3E-01
PCPG	LTA4H_ES_18.1:18.2_17_19	LTA4H	ES	18.1:18.2	17	19	1.8E-06	0.150	-0.135	7.1E-02	5.0E-01	1.0E+00
PCPG	LYRM1_ES_5_3_7	LYRM1	ES	5	3	7	3.2E-05	0.128	-0.166	1.4E-01	4.4E-01	9.8E-01
PCPG	MACF1_ES_103_102_104	MACF1	ES	103	102	104	1.3E-09	0.221	-0.125	3.4E-01	9.7E-01	3.2E-01
PCPG	MADD_ES_18_17_19	MADD	ES	18	17	19	3.4E-10	0.243	0.160	2.5E-02	5.2E-02	8.7E-02
PCPG	MAGI2_ES_24_23_25	MAGI2	ES	24	23	25	1.9E-09	0.274	-0.011	5.1E-01	2.2E-01	3.2E-01
PCPG	MANBAL_ES_3_1_4.2	MANBAL	ES	3	1	4.2	1.4E-04	0.103	-0.131	1.5E-01	6.0E-02	2.6E-01
PCPG	MAP3K12_RI_3.3_3.2_3.4	MAP3K12	RI	3.3	3.2	3.4	1.2E-04	0.110	0.085	9.9E-01	9.5E-01	3.6E-01
PCPG	MAP3K4_ES_18_17_19	MAP3K4	ES	18	17	19	2.3E-04	0.100	-0.066	6.3E-02	5.3E-02	8.9E-02
PCPG	MAP3K4_ES_24_23_25	MAP3K4	ES	24	23	25	1.5E-04	0.106	-0.081	8.8E-01	6.9E-01	3.2E-01
PCPG	MAP3K8_ES_2_1_3.2	MAP3K8	ES	2	1	3.2	8.3E-05	0.129	-0.017	4.7E-01	6.8E-01	5.8E-01
PCPG	MAPK10_ES_9_8.2_10	MAPK10	ES	9	8.2	10	2.1E-06	0.162	0.274	1.5E-01	2.7E-01	3.3E-01
PCPG	MAPK10_ME_12|13_11_14	MAPK10	ME	12|13	11	14	9.1E-05	0.110	0.307	3.0E-01	9.8E-01	3.8E-01
PCPG	MAPK9_ES_3_2_4	MAPK9	ES	3	2	4	1.2E-04	0.107	0.145	5.4E-01	9.9E-01	9.1E-01
PCPG	MAPT_ES_13_12_14	MAPT	ES	13	12	14	9.9E-08	0.181	0.110	3.9E-01	2.4E-02	3.7E-01
PCPG	MARK2_ES_18_17_19	MARK2	ES	18	17	19	3.8E-04	0.102	-0.017	5.8E-01	4.9E-01	1.0E+00
PCPG	MARK3_ES_17_16_18	MARK3	ES	17	16	18	1.8E-08	0.199	-0.085	4.9E-01	3.0E-01	1.0E+00
PCPG	MARK3_ES_17_16_19	MARK3	ES	17	16	19	7.1E-09	0.205	-0.223	1.2E-01	3.3E-01	1.0E+00

cancer type	id	Gene Symbol	splice_type	Exon	From.Exon	To.Exon	anova.p	adj.r2	r	p.50	p.25	p.10
PCPG	MARK3_ES_17:18_16_19	MARK3	ES	17:18	16	19	3.5E-09	0.211	-0.127	1.2E-01	1.8E-01	1.0E+00
PCPG	MBD1_ES_18.1_17_18.4	MBD1	ES	18.1	17	18.4	1.1E-04	0.107	0.138	3.7E-01	3.1E-01	1.0E+00
PCPG	MBD1_ES_18.1_17_18.5	MBD1	ES	18.1	17	18.5	4.3E-04	0.111	-0.187	4.8E-01	1.9E-01	6.6E-01
PCPG	MBD1_ES_18.1:18.4_17_18.5	MBD1	ES	18.1:18.4	17	18.5	5.6E-05	0.115	-0.046	6.2E-01	3.2E-01	1.0E+00
PCPG	MBD1_RI_18.2:18.3_18.1_18.4	MBD1	RI	18.2:18.3	18.1	18.4	1.4E-07	0.175	-0.074	1.0E-01	9.5E-01	1.0E+00
PCPG	MBNL1_ES_10_9_11	MBNL1	ES	10	9	11	1.8E-07	0.213	-0.241	7.3E-02	1.8E-01	3.8E-01
PCPG	MBNL2_ES_9_8_10	MBNL2	ES	9	8	10	1.6E-04	0.118	0.115	1.0E-01	3.6E-01	3.4E-01
PCPG	MEAF6_ES_6_5_8.1	MEAF6	ES	6	5	8.1	1.7E-10	0.239	0.282	8.0E-01	6.7E-01	2.5E-01
PCPG	MEAF6_ES_6:7_5_8.1	MEAF6	ES	6:07	5	8.1	6.6E-08	0.190	0.356	7.2E-01	5.2E-01	2.7E-01
PCPG	MED12_AD_39.2_39.1_40.1	MED12	AD	39.2	39.1	40.1	1.4E-04	0.104	0.042	4.2E-01	6.8E-01	3.2E-01
PCPG	MED24_ES_7_6_8	MED24	ES	7	6	8	2.7E-04	0.107	0.054	7.8E-02	1.4E-01	1.0E+00
PCPG	MEF2D_ES_10_9_11	MEF2D	ES	10	9	11	2.4E-12	0.284	-0.086	4.7E-02	1.6E-01	2.4E-01
PCPG	MEGF8_ES_41_40_42	MEGF8	ES	41	40	42	2.8E-05	0.122	-0.185	1.1E-01	2.9E-01	2.2E-01
PCPG	MEIS3_AA_7.1_6_7.2	MEIS3	AA	7.1	6	7.2	1.2E-06	0.171	0.813	3.3E-01	3.3E-01	1.2E-01
PCPG	MFF_ES_3_1_5	MFF	ES	3	1	5	3.1E-10	0.235	-0.305	5.5E-01	3.3E-01	1.0E+00
PCPG	MFF_ES_3:4_1_5	MFF	ES	3:04	1	5	2.2E-05	0.129	-0.309	8.4E-01	3.5E-01	1.0E+00
PCPG	MFF_ES_3:5_1_6	MFF	ES	3:05	1	6	2.8E-07	0.168	-0.224	1.4E-01	3.2E-01	3.5E-01
PCPG	MORF4L2_ES_4:5.1:5.2:5.3_3.2_6.2	MORF4L2	ES	4:5.1:5.2:5.3	3.2	6.2	6.5E-05	0.119	-0.251	8.0E-01	1.5E-01	4.1E-01
PCPG	MPI_ES_7_6.1_8	MPI	ES	7	6.1	8	2.1E-05	0.123	0.187	5.3E-01	8.3E-01	3.5E-01
PCPG	MPP5_AA_3.1_2_3.2	MPP5	AA	3.1	2	3.2	2.0E-04	0.130	-0.090	5.2E-01	2.7E-01	1.0E+00
PCPG	MPPE1_ES_9.1:9.2_8_10	MPPE1	ES	9.1:9.2	8	10	2.5E-05	0.132	-0.116	5.8E-01	2.8E-01	1.0E+00
PCPG	MPRIP_ES_24_23_25	MPRIP	ES	24	23	25	2.3E-07	0.171	0.198	1.8E-02	1.2E-01	1.0E+00
PCPG	MPST_ES_4_3_5	MPST	ES	4	3	5	2.0E-13	0.298	-0.768	2.1E-01	3.1E-01	2.9E-01
PCPG	MRPL35_RI_4.2:4.3_4.1_4.4	MRPL35	RI	4.2:4.3	4.1	4.4	1.4E-07	0.175	0.240	8.8E-02	1.3E-01	1.5E-01
PCPG	MTA1_ES_19_18_20	MTA1	ES	19	18	20	5.5E-10	0.229	0.197	8.0E-02	5.1E-02	1.0E+00
PCPG	MTERFD2_ES_2_1_3.1	MTERFD2	ES	2	1	3.1	4.7E-09	0.213	0.527	7.0E-01	9.4E-01	9.8E-01
PCPG	MTERFD2_ES_3.1:4_1_5	MTERFD2	ES	3.1:4	1	5	4.4E-05	0.138	0.294	1.7E-01	9.7E-01	3.9E-01
PCPG	MTHFD2L_ES_7_6_8	MTHFD2L	ES	7	6	8	9.9E-05	0.125	0.158	8.2E-01	9.3E-01	1.0E+00
PCPG	MTRR_ES_4.1:4.2_2_5	MTRR	ES	4.1:4.2	2	5	2.3E-04	0.118	-0.063	1.7E-01	5.9E-01	9.1E-01
PCPG	MTSS1_ES_13:14.1_11_14.2	MTSS1	ES	13:14.1	11	14.2	1.2E-04	0.115	0.046	1.9E-01	1.0E+00	1.0E+00
PCPG	MXRA7_RI_3.2_3.1_3.3	MXRA7	RI	3.2	3.1	3.3	7.8E-04	0.101	0.024	4.9E-01	1.0E+00	1.0E+00
PCPG	MYH11_ES_42_41_43	MYH11	ES	42	41	43	1.3E-11	0.268	0.390	6.9E-01	9.5E-01	5.6E-01
PCPG	MYO18A_ES_41_40_42	MYO18A	ES	41	40	42	4.6E-06	0.143	-0.125	4.9E-01	1.0E+00	1.0E+00
PCPG	MYO1B_ES_23_22_24	MYO1B	ES	23	22	24	3.4E-13	0.310	0.373	4.6E-01	5.6E-01	1.1E-01
PCPG	MYO1B_ES_23:24_22_25	MYO1B	ES	23:24	22	25	1.0E-05	0.133	0.348	8.9E-01	5.9E-01	9.4E-01
PCPG	MYO1B_ES_24_22_25	MYO1B	ES	24	22	25	2.4E-08	0.202	-0.135	4.7E-02	5.4E-02	4.0E-02
PCPG	MYO5A_ES_33_32_34	MYO5A	ES	33	32	34	1.8E-04	0.121	-0.317	8.1E-01	2.9E-01	1.0E+00
PCPG	MYO5A_ES_35_34_36	MYO5A	ES	35	34	36	1.8E-08	0.222	-0.123	9.7E-01	6.1E-01	3.5E-01
PCPG	MZT2B_ES_2:3_1_4	MZT2B	ES	2:03	1	4	8.4E-05	0.108	-0.404	7.4E-01	6.0E-01	1.7E-01
PCPG	N4BP2L2_ES_2.1:2.2_1.3_3.1	N4BP2L2	ES	2.1:2.2	1.3	3.1	4.8E-06	0.140	-0.046	7.7E-01	5.6E-01	9.4E-01

cancer type	id	Gene Symbol	splice_type	Exon	From.Exon	To.Exon	anova.p	adj.r2	r	p.50	p.25	p.10
PCPG	NAALADL1_ES_13_11_14	NAALADL1	ES	13	11	14	1.9E-05	0.142	-0.337	7.3E-01	9.9E-01	2.8E-01
PCPG	NABP2_RI_1.2:1.3_1.1_1.4	NABP2	RI	1.2:1.3	1.1	1.4	6.8E-06	0.136	0.009	4.5E-01	6.4E-01	1.0E+00
PCPG	NAP1L1_RI_14.2_14.1_14.3	NAP1L1	RI	14.2	14.1	14.3	4.4E-05	0.115	-0.161	1.4E-01	5.1E-02	1.8E-01
PCPG	NCAM1_ES_15_14_16.2	NCAM1	ES	15	14	16.2	3.2E-06	0.147	-0.387	7.9E-01	1.0E+00	1.0E+00
PCPG	NCKAP1_ES_2_1_3	NCKAP1	ES	2	1	3	7.2E-10	0.253	0.445	9.7E-01	2.6E-01	1.0E+00
PCPG	NDEL1_ES_11_10_12.1	NDEL1	ES	11	10	12.1	6.3E-05	0.114	-0.081	1.3E-01	3.7E-02	3.2E-01
PCPG	NDRG2_RI_4.3:4.4_4.2_4.5	NDRG2	RI	4.3:4.4	4.2	4.5	7.1E-08	0.206	-0.333	9.4E-01	2.1E-01	3.2E-01
PCPG	NDRG3_ES_16_15_17	NDRG3	ES	16	15	17	3.9E-08	0.189	0.112	2.6E-02	3.3E-01	1.0E+00
PCPG	NDRG4_ES_22_21_23	NDRG4	ES	22	21	23	1.8E-04	0.100	0.128	1.4E-02	3.9E-02	1.6E-01
PCPG	NDUFAF5_ES_9_8_10	NDUFAF5	ES	9	8	10	4.3E-07	0.164	-0.293	8.9E-01	6.7E-01	1.0E+00
PCPG	NDUFS7_RI_9.2_9.1_9.3	NDUFS7	RI	9.2	9.1	9.3	4.1E-05	0.116	0.055	7.8E-02	4.9E-01	3.4E-01
PCPG	NECAB3_AD_7.2:7.3_7.1_8	NECAB3	AD	7.2:7.3	7.1	8	1.5E-04	0.113	0.118	5.8E-01	1.0E+00	9.0E-01
PCPG	NEIL2_ES_1.3_1.1_2.1	NEIL2	ES	1.3	1.1	2.1	4.8E-04	0.107	-0.126	4.7E-01	2.6E-01	9.5E-01
PCPG	NEO1_ES_22_21_23	NEO1	ES	22	21	23	5.2E-05	0.146	0.196	1.8E-01	3.2E-01	1.0E+00
PCPG	NEO1_ES_27_26_28	NEO1	ES	27	26	28	1.8E-06	0.153	-0.216	4.0E-01	1.0E+00	1.0E+00
PCPG	NIN_ES_18_17_19	NIN	ES	18	17	19	4.1E-06	0.144	-0.143	8.5E-01	8.7E-02	1.6E-01
PCPG	NME4_ES_4_2_5	NME4	ES	4	2	5	6.0E-06	0.137	-0.039	9.3E-01	5.1E-01	3.5E-01
PCPG	NMRAL1_AD_2.3_2.2_2.6	NMRAL1	AD	2.3	2.2	2.6	1.4E-05	0.132	-0.067	8.5E-01	6.8E-01	2.9E-01
PCPG	NMRAL1_AD_2.4_2.3_2.6	NMRAL1	AD	2.4	2.3	2.6	1.6E-05	0.136	0.166	6.6E-01	9.3E-01	2.9E-01
PCPG	NPM2_AD_2.2_2.1_2.4	NPM2	AD	2.2	2.1	2.4	6.5E-07	0.169	-0.056	9.4E-01	1.7E-01	1.0E+00
PCPG	NPM2_RI_2.2:2.3_2.1_2.4	NPM2	RI	2.2:2.3	2.1	2.4	1.7E-06	0.158	-0.221	8.5E-01	2.2E-01	1.0E+00
PCPG	NSFL1C_ES_5.2_4_7.2	NSFL1C	ES	5.2	4	7.2	4.0E-07	0.165	-0.241	2.6E-01	1.5E-01	2.6E-01
PCPG	NSG1_RI_5.2_5.1_5.3	NSG1	RI	5.2	5.1	5.3	7.5E-05	0.117	0.021	7.5E-01	9.7E-01	3.7E-01
PCPG	NUDT22_ES_3_1.4_4	NUDT22	ES	3	1.4	4	6.2E-05	0.111	0.132	1.5E-01	6.0E-02	5.4E-02
PCPG	NUMB_ES_7_6_8.2	NUMB	ES	7	6	8.2	4.6E-09	0.232	-0.079	5.6E-02	2.9E-01	1.4E-01
PCPG	NVL_ES_2_1_3.1	NVL	ES	2	1	3.1	6.1E-05	0.144	-0.191	8.8E-01	5.9E-01	3.2E-01
PCPG	OCIAD1_ES_2.2:3:4_2.1_6	OCIAD1	ES	2.2:3:4	2.1	6	3.9E-04	0.101	-0.072	7.1E-01	6.1E-01	1.0E+00
PCPG	OFD1_ES_11_10_13	OFD1	ES	11	10	13	4.0E-07	0.183	-0.048	7.0E-01	7.9E-01	3.5E-01
PCPG	OPA1_ES_7_6_8	OPA1	ES	7	6	8	3.0E-05	0.148	-0.153	1.7E-01	4.0E-01	1.0E+00
PCPG	PACS2_ES_20_19_21	PACS2	ES	20	19	21	6.4E-12	0.271	0.077	9.2E-02	8.6E-02	1.6E-01
PCPG	PACSIN2_ES_12_11_13	PACSIN2	ES	12	11	13	7.8E-05	0.109	0.176	9.7E-01	3.5E-01	1.0E+00
PCPG	PCBP3_ES_11.1_10_12	PCBP3	ES	11.1	10	12	9.9E-08	0.180	-0.073	1.4E-01	1.7E-01	1.0E+00
PCPG	PCBP3_ES_11.1:11.2_10_12	PCBP3	ES	11.1:11.2	10	12	1.5E-08	0.202	-0.031	1.8E-01	3.3E-01	3.2E-01
PCPG	PCNP_AA_2.1_1_2.2	PCNP	AA	2.1	1	2.2	1.2E-04	0.106	0.155	1.6E-01	2.6E-01	1.0E+00
PCPG	PCNP_AA_2.2_1_2.3	PCNP	AA	2.2	1	2.3	3.3E-05	0.119	-0.297	8.0E-01	1.6E-01	1.0E+00
PCPG	PCNXL4_ES_2.1_1_3	PCNXL4	ES	2.1	1	3	8.7E-08	0.198	-0.302	2.9E-01	7.5E-01	1.6E-01
PCPG	PDCD2_RI_3.2_3.1_3.3	PDCD2	RI	3.2	3.1	3.3	1.4E-04	0.102	-0.005	5.1E-01	6.4E-01	3.3E-01
PCPG	PDLIM2_ES_10_9_11.1	PDLIM2	ES	10	9	11.1	6.9E-10	0.226	0.075	4.7E-01	9.6E-01	1.0E+00
PCPG	PDLIM5_AA_8.1_6.1_8.2	PDLIM5	AA	8.1	6.1	8.2	1.5E-05	0.133	-0.301	1.8E-02	1.5E-01	1.0E+00
PCPG	PEX19_ES_3_1_4	PEX19	ES	3	1	4	3.1E-08	0.193	-0.174	5.3E-01	2.4E-01	3.2E-01

cancer type	id	Gene Symbol	splice_type	Exon	From.Exon	To.Exon	anova.p	adj.r2	r	p.50	p.25	p.10
PCPG	PFDN1_ES_2_1_3.1	PFDN1	ES	2	1	3.1	8.0E-06	0.134	-0.086	6.5E-01	9.8E-01	6.1E-01
PCPG	PFDN5_ES_2_1_5	PFDN5	ES	2	1	5	5.2E-07	0.162	0.138	4.8E-01	4.9E-01	3.3E-01
PCPG	PFDN5_ES_2:3:5:6.1_1_6.2	PFDN5	ES	2:3:5:6.1	1	6.2	2.2E-05	0.135	-0.051	7.1E-01	9.1E-01	4.1E-01
PCPG	PFDN5_ES_2:4.1:4.2:5_1_6.2	PFDN5	ES	2:4.1:4.2:5	1	6.2	4.9E-04	0.104	-0.117	6.9E-01	3.9E-01	1.0E+00
PCPG	PFDN5_ES_2:4.2:5_1_6.2	PFDN5	ES	04:02.2	1	6.2	4.5E-05	0.137	-0.104	1.8E-01	6.5E-01	2.2E-01
PCPG	PFDN5_ES_2:5_1_6.2	PFDN5	ES	2:05	1	6.2	3.2E-08	0.194	0.009	2.8E-01	9.1E-01	4.6E-01
PCPG	PFDN5_ES_4.2_2_5	PFDN5	ES	4.2	2	5	8.0E-06	0.158	-0.215	1.6E-01	1.0E+00	2.4E-01
PCPG	PFN2_AA_6.1_5.4_6.2	PFN2	AA	6.1	5.4	6.2	9.7E-10	0.223	0.294	6.5E-01	8.8E-01	3.2E-01
PCPG	PHF21A_ME_16|17_15_18	PHF21A	ME	16|17	15	18	1.4E-16	0.361	0.242	5.2E-02	6.1E-02	2.5E-01
PCPG	PI4KB_ES_5_4_6	PI4KB	ES	5	4	6	1.2E-04	0.109	-0.010	1.9E-01	1.0E-01	1.8E-01
PCPG	PICALM_ES_19_18_20	PICALM	ES	19	18	20	1.4E-06	0.156	0.031	1.6E-02	2.0E-02	5.4E-02
PCPG	PIGQ_ES_12_11_13	PIGQ	ES	12	11	13	3.8E-06	0.142	-0.072	2.3E-01	3.6E-02	2.1E-01
PCPG	PINX1_ES_6_5_8	PINX1	ES	6	5	8	7.6E-06	0.135	-0.009	8.3E-01	3.3E-01	1.0E+00
PCPG	PLA2G6_ES_14_13_15	PLA2G6	ES	14	13	15	3.1E-05	0.120	0.003	6.2E-01	3.0E-01	2.7E-01
PCPG	POFUT2_AD_8.2:8.3_8.1_8.5	POFUT2	AD	8.2:8.3	8.1	8.5	8.4E-05	0.109	0.122	4.4E-01	9.5E-02	3.5E-01
PCPG	POLR2J3_ES_4.5_4.3_8	POLR2J3	ES	4.5	4.3	8	2.2E-06	0.147	0.255	5.1E-01	9.4E-01	9.2E-01
PCPG	POLR2J3_RI_4.4_4.3_4.5	POLR2J3	RI	4.4	4.3	4.5	1.0E-06	0.155	-0.231	9.5E-01	7.6E-02	1.0E+00
PCPG	PORCN_ES_7:8_6_9	PORCN	ES	7:08	6	9	1.3E-23	0.473	-0.372	5.9E-02	1.3E-01	1.0E+00
PCPG	PORCN_ES_8_6_9	PORCN	ES	8	6	9	1.2E-05	0.158	-0.204	9.2E-01	3.2E-01	1.0E+00
PCPG	PORCN_ES_8_7_9	PORCN	ES	8	7	9	7.1E-20	0.441	-0.568	5.3E-01	2.4E-01	5.2E-01
PCPG	PPCDC_ES_2_1_3.2	PPCDC	ES	2	1	3.2	1.3E-04	0.112	0.015	7.4E-01	4.5E-01	3.3E-01
PCPG	PPCS_AD_2.2_2.1_3.1	PPCS	AD	2.2	2.1	3.1	1.3E-05	0.128	-0.249	1.8E-02	1.5E-01	3.2E-01
PCPG	PPFIA1_ES_17_16_18	PPFIA1	ES	17	16	18	7.8E-08	0.215	0.305	1.3E-01	5.1E-01	3.2E-01
PCPG	PPIE_ES_11_9.1_12	PPIE	ES	11	9.1	12	2.9E-10	0.234	0.413	5.2E-01	8.2E-01	6.8E-01
PCPG	PPIP5K2_ES_26_25_27	PPIP5K2	ES	26	25	27	4.9E-04	0.106	0.031	3.0E-01	4.0E-01	3.4E-01
PCPG	PPP2R2B_ES_9_8.4_10	PPP2R2B	ES	9	8.4	10	1.1E-05	0.142	-0.076	7.8E-01	4.0E-01	3.5E-01
PCPG	PPP2R5C_ES_21_20.1_22	PPP2R5C	ES	21	20.1	22	2.1E-08	0.198	-0.275	6.1E-01	3.0E-01	1.0E+00
PCPG	PPP3CA_ES_13_12_14	PPP3CA	ES	13	12	14	1.0E-11	0.281	0.059	1.9E-01	3.6E-01	3.5E-01
PCPG	PPP3CC_ES_14_13_15	PPP3CC	ES	14	13	15	2.8E-05	0.120	-0.070	3.0E-01	5.2E-01	1.8E-01
PCPG	PPP4R1_ES_15_14_16	PPP4R1	ES	15	14	16	3.5E-05	0.123	-0.223	8.1E-01	8.9E-01	1.0E+00
PCPG	PPP6R3_ES_25_23_26.2	PPP6R3	ES	25	23	26.2	3.0E-05	0.128	-0.130	4.6E-01	5.2E-01	1.0E+00
PCPG	PRDX5_ES_3_1_4	PRDX5	ES	3	1	4	1.4E-12	0.282	-0.060	3.9E-01	3.4E-01	2.9E-01
PCPG	PRKACB_ES_5:6_3_8	PRKACB	ES	5:06	3	8	5.1E-06	0.181	-0.062	3.6E-01	3.5E-01	1.0E+00
PCPG	PRKACB_ES_7_6_8	PRKACB	ES	7	6	8	3.0E-06	0.184	0.123	3.3E-01	9.5E-01	2.9E-01
PCPG	PRKAG1_ES_3:4.1_2_4.2	PRKAG1	ES	03:04.1	2	4.2	1.9E-05	0.149	0.049	7.0E-01	1.9E-01	3.4E-01
PCPG	PRMT1_ES_4.2:5_4.1_6	PRMT1	ES	4.2:5	4.1	6	2.3E-06	0.148	0.073	6.6E-01	4.8E-01	3.5E-01
PCPG	PRMT2_ES_1.3_1.1_2	PRMT2	ES	1.3	1.1	2	5.4E-05	0.114	-0.081	9.8E-01	6.1E-01	9.0E-01
PCPG	PRPF39_ES_4.1:4.2:4.3_3_5	PRPF39	ES	4.1:4.2:4.3	3	5	2.0E-05	0.130	0.427	6.7E-01	1.0E+00	1.0E+00
PCPG	PRPF40A_ES_8_7_9	PRPF40A	ES	8	7	9	3.1E-05	0.137	-0.067	1.3E-01	3.2E-01	2.8E-01
PCPG	PSAP_AA_8.1_7_8.2	PSAP	AA	8.1	7	8.2	4.5E-09	0.210	0.257	8.2E-01	3.2E-01	3.7E-01

cancer type	id	Gene Symbol	splice_type	Exon	From.Exon	To.Exon	anova.p	adj.r2	r	p.50	p.25	p.10
PCPG	PSMD11_RI_13.2_13.1_13.3	PSMD11	RI	13.2	13.1	13.3	1.7E-07	0.173	0.025	6.5E-01	1.7E-01	1.0E+00
PCPG	PSME2_ES_2:3.1_1_3.2	PSME2	ES	02:03.1	1	3.2	4.5E-06	0.140	-0.229	9.2E-01	8.9E-01	4.3E-01
PCPG	PTK2_ES_20_19_21.2	PTK2	ES	20	19	21.2	4.2E-06	0.161	-0.167	3.4E-01	5.2E-01	1.0E+00
PCPG	PTK2_ES_22_21.2_23	PTK2	ES	22	21.2	23	3.0E-05	0.134	-0.259	1.7E-01	7.2E-02	1.0E+00
PCPG	PTK2_ES_39.2_37_39.5	PTK2	ES	39.2	37	39.5	3.2E-11	0.259	-0.220	3.1E-01	4.9E-01	2.6E-01
PCPG	PTPRA_ES_8_7_10	PTPRA	ES	8	7	10	5.6E-07	0.178	-0.314	1.4E-01	8.3E-02	1.9E-01
PCPG	PTPRF_ES_14_13_15	PTPRF	ES	14	13	15	5.4E-04	0.111	0.074	3.2E-02	7.4E-02	4.0E-01
PCPG	PTPRK_ES_19:20_18_21	PTPRK	ES	19:20	18	21	1.7E-06	0.183	0.508	3.5E-01	2.5E-01	3.8E-01
PCPG	PTPRS_ES_15:16:17:18:19_14_20	PTPRS	ES	15:16:17:18:19	14	20	3.3E-06	0.150	-0.077	4.9E-01	5.7E-01	2.8E-01
PCPG	PTPRS_ES_15:16:18:19_14_20	PTPRS	ES	15:16:18:19	14	20	2.1E-04	0.104	-0.181	3.4E-01	7.0E-01	7.1E-01
PCPG	PTPRS_ES_17_16_18	PTPRS	ES	17	16	18	4.5E-05	0.147	0.152	6.0E-01	7.0E-01	3.7E-01
PCPG	PUM2_ES_5_4.2_6	PUM2	ES	5	4.2	6	5.0E-04	0.114	-0.130	5.9E-01	3.4E-01	3.6E-01
PCPG	RAB11FIP3_ES_7_6_8	RAB11FIP3	ES	7	6	8	3.0E-05	0.119	-0.081	3.1E-01	1.6E-01	5.3E-02
PCPG	RABGGTA_RI_1.2:1.3_1.1_1.4	RABGGTA	RI	1.2:1.3	1.1	1.4	1.6E-04	0.101	0.217	9.8E-01	1.6E-01	1.0E+00
PCPG	RAP1GAP_ES_22.1_21_22.4	RAP1GAP	ES	22.1	21	22.4	7.6E-06	0.157	-0.069	8.2E-01	5.6E-01	9.1E-01
PCPG	RAP1GDS1_ES_6_5_7	RAP1GDS1	ES	6	5	7	3.2E-06	0.152	-0.370	6.0E-01	5.8E-01	1.0E+00
PCPG	RASA4_ES_17_16_18	RASA4	ES	17	16	18	1.0E-04	0.106	0.078	9.0E-01	9.0E-01	2.9E-01
PCPG	RBM42_ES_5:6.1_4_6.2	RBM42	ES	05:06.1	4	6.2	2.1E-07	0.172	-0.147	4.2E-01	9.9E-01	1.0E+00
PCPG	RBM6_AA_3.1_2_3.2	RBM6	AA	3.1	2	3.2	1.1E-05	0.137	0.278	7.8E-01	6.1E-01	3.7E-01
PCPG	RBM6_ES_3.1:3.2_2_4	RBM6	ES	3.1:3.2	2	4	1.5E-04	0.106	0.145	2.1E-02	3.2E-02	2.6E-01
PCPG	RBM6_ES_3.1:3.2:4:5:6_2_7	RBM6	ES	3.1:3.2:4:5:6	2	7	1.6E-04	0.106	0.127	7.9E-01	4.9E-01	3.5E-01
PCPG	RBMS1_ES_13_12_14	RBMS1	ES	13	12	14	7.1E-05	0.114	-0.173	5.7E-01	6.8E-01	4.7E-01
PCPG	RBPJ_ES_4.2_2.3_5	RBPJ	ES	4.2	2.3	5	3.7E-04	0.109	-0.005	9.1E-01	1.0E+00	1.0E+00
PCPG	RECQL5_ES_1.2:2.1_1.1_2.2	RECQL5	ES	1.2:2.1	1.1	2.2	1.9E-04	0.115	-0.166	2.9E-01	8.9E-01	3.7E-01
PCPG	REPS1_ES_9.3_9.1_10	REPS1	ES	9.3	9.1	10	2.5E-05	0.124	0.281	3.8E-01	7.3E-01	3.3E-01
PCPG	RGS7_ES_21:22_20_23	RGS7	ES	21:22	20	23	4.4E-10	0.242	0.223	3.5E-01	5.0E-01	1.9E-01
PCPG	RGS7_ES_22_20_23	RGS7	ES	22	20	23	1.0E-07	0.185	0.237	2.9E-01	3.4E-01	1.0E+00
PCPG	RIC8B_ES_13_11_17	RIC8B	ES	13	11	17	2.9E-05	0.132	0.066	6.5E-01	1.6E-01	3.5E-01
PCPG	RIT1_ES_6_5_7	RIT1	ES	6	5	7	3.0E-04	0.108	-0.228	6.8E-03	1.8E-01	1.0E+00
PCPG	RNF14_ES_2.2:3_2.1_4	RNF14	ES	2.2:3	2.1	4	2.8E-04	0.114	0.260	4.6E-01	9.9E-01	9.1E-01
PCPG	RNF146_ES_3:5.1_2_6	RNF146	ES	03:05.1	2	6	4.2E-05	0.122	0.236	3.7E-02	9.5E-02	7.4E-02
PCPG	RNF146_ES_4_2_5.1	RNF146	ES	4	2	5.1	2.2E-04	0.105	0.140	6.5E-01	5.4E-01	1.5E-01
PCPG	RNF181_ES_2:3.2_1_4.1	RNF181	ES	02:03.2	1	4.1	5.3E-05	0.120	-0.318	3.4E-01	1.5E-01	1.4E-01
PCPG	RNF216_AA_5.1_4_5.2	RNF216	AA	5.1	4	5.2	1.2E-07	0.204	-0.089	7.1E-01	9.0E-01	2.9E-01
PCPG	RNF7_ES_1.2:2_1.1_3	RNF7	ES	1.2:2	1.1	3	1.3E-04	0.104	0.024	4.6E-01	3.3E-01	1.0E+00
PCPG	ROBO2_ES_27_26_28	ROBO2	ES	27	26	28	4.4E-06	0.160	-0.185	7.9E-01	5.1E-01	9.5E-01
PCPG	RPGR_ES_14.1:14.3_13_16	RPGR	ES	14.1:14.3	13	16	7.5E-06	0.137	0.067	3.3E-01	1.6E-01	2.5E-01
PCPG	RPL32_RI_1.2_1.1_1.3	RPL32	RI	1.2	1.1	1.3	7.7E-06	0.134	-0.152	7.4E-01	9.5E-01	5.8E-01
PCPG	RPS24_ES_5.1:5.2_4_6	RPS24	ES	5.1:5.2	4	6	3.2E-05	0.119	0.032	7.4E-02	7.8E-02	3.2E-01
PCPG	RPS25_ES_2.2:3.1_2.1_3.2	RPS25	ES	2.2:3.1	2.1	3.2	1.1E-07	0.178	-0.094	5.3E-01	9.4E-01	1.0E+00

cancer type	id	Gene Symbol	splice_type	Exon	From.Exon	To.Exon	anova.p	adj.r2	r	p.50	p.25	p.10
PCPG	RTN2_ES_5_4.1_6	RTN2	ES	5	4.1	6	2.2E-05	0.125	-0.288	7.1E-01	5.5E-01	1.0E+00
PCPG	RUVBL2_AD_1.2:1.3:1.4:1.5:1.6_1.1_2	RUVBL2	AD	1.2:1.3:1.4:1.5:1.[illegible]	1.1	2	1.7E-04	0.101	-0.063	6.2E-02	1.3E-01	3.2E-01
PCPG	RWDD1_ES_2:3_1_4	RWDD1	ES	2:03	1	4	1.3E-06	0.153	-0.033	8.0E-01	6.9E-01	1.0E+00
PCPG	RWDD1_ES_3_1_4	RWDD1	ES	3	1	4	1.0E-09	0.223	0.101	2.5E-01	4.3E-01	1.3E-01
PCPG	RWDD2B_ES_3_2_4	RWDD2B	ES	3	2	4	1.7E-04	0.110	0.189	2.1E-01	1.7E-01	1.0E+00
PCPG	SAT2_AD_1.2_1.1_2	SAT2	AD	1.2	1.1	2	1.8E-05	0.125	-0.175	5.1E-01	9.3E-01	2.9E-01
PCPG	SBF1_ES_29_28_30	SBF1	ES	29	28	30	4.5E-07	0.165	-0.348	7.4E-01	6.6E-01	9.9E-01
PCPG	SCARB1_ES_14_13.2_15	SCARB1	ES	14	13.2	15	1.0E-11	0.264	0.382	4.6E-01	5.2E-01	1.0E+00
PCPG	SCYL1_AA_17.1:17.2_16.2_17.3	SCYL1	AA	17.1:17.2	16.2	17.3	1.5E-04	0.101	-0.155	3.9E-01	3.6E-01	2.4E-01
PCPG	SEC16A_ES_24_23.12_26	SEC16A	ES	24	23.12	26	5.1E-05	0.115	0.106	1.5E-02	7.4E-02	1.5E-01
PCPG	SEC24C_ES_8_7_9	SEC24C	ES	8	7	9	2.5E-04	0.108	-0.001	6.8E-01	5.3E-01	1.0E+00
PCPG	SEC31A_ES_26.1_25.1_28	SEC31A	ES	26.1	25.1	28	1.0E-07	0.179	0.124	3.9E-01	9.4E-02	2.9E-01
PCPG	SEC31A_ES_26.1:26.2_25.1_28	SEC31A	ES	26.1:26.2	25.1	28	5.1E-10	0.229	0.317	3.1E-01	6.6E-01	2.9E-01
PCPG	SEC31A_ES_26.2:27_26.1_28	SEC31A	ES	26.2:27	26.1	28	1.7E-11	0.262	-0.069	7.7E-02	2.7E-02	2.0E-01
PCPG	SELENBP1_ES_6_5_7	SELENBP1	ES	6	5	7	5.8E-09	0.245	-0.599	2.1E-01	9.3E-02	1.3E-01
PCPG	SEPT4_ES_7.1_3_8	SEPT4	ES	7.1	3	8	7.2E-08	0.207	-0.114	6.5E-01	4.6E-01	3.7E-01
PCPG	SEPT4_ES_7.1:7.2_3_8	SEPT4	ES	7.1:7.2	3	8	1.3E-04	0.104	0.030	9.4E-01	9.4E-01	8.5E-01
PCPG	SEPT6_ES_12_11.1_13.1	SEPT6	ES	12	11.1	13.1	8.3E-05	0.109	-0.083	9.5E-02	1.5E-01	1.2E-01
PCPG	SEPT6_ES_12:13.1_11.1_13.2	SEPT6	ES	12:13.1	11.1	13.2	2.1E-06	0.149	0.015	1.1E-01	1.8E-01	9.8E-01
PCPG	SEPT8_AA_12.1_11_12.2	SEPT8	AA	12.1	11	12.2	8.6E-07	0.159	0.368	1.2E-01	4.3E-01	3.9E-01
PCPG	SERP2_ES_4_3_6	SERP2	ES	4	3	6	6.0E-05	0.112	-0.147	7.7E-01	1.3E-01	1.7E-01
PCPG	SETX_ES_26_25_27	SETX	ES	26	25	27	2.3E-05	0.127	-0.184	7.6E-01	9.0E-01	3.7E-01
PCPG	SEZ6L2_ES_15_14_16	SEZ6L2	ES	15	14	16	4.2E-05	0.116	0.120	8.4E-01	4.1E-01	4.6E-01
PCPG	SGCE_ES_14_13.1_15	SGCE	ES	14	13.1	15	6.2E-05	0.111	0.219	8.2E-01	8.1E-01	9.6E-01
PCPG	SH3GL3_ES_2:3:4_1_5	SH3GL3	ES	2:03:04	1	5	5.2E-08	0.185	-0.880	5.8E-01	9.7E-01	9.8E-01
PCPG	SH3GLB1_ES_6:7_5_8	SH3GLB1	ES	6:07	5	8	4.7E-09	0.209	-0.317	4.3E-01	9.8E-01	3.4E-01
PCPG	SH3GLB2_ES_12_11_13	SH3GLB2	ES	12	11	13	9.0E-07	0.157	0.128	1.4E-01	4.7E-01	3.0E-01
PCPG	SH3GLB2_ES_7:8_6_9	SH3GLB2	ES	7:08	6	9	2.6E-06	0.160	0.096	8.0E-01	9.5E-01	3.2E-01
PCPG	SHANK3_ES_19_18.1_21	SHANK3	ES	19	18.1	21	1.1E-04	0.122	-0.448	1.8E-01	3.6E-01	4.5E-01
PCPG	SIDT2_ES_11_10_12	SIDT2	ES	11	10	12	1.6E-10	0.268	0.187	2.0E-01	9.9E-01	3.2E-01
PCPG	SIK3_ES_10_9_11	SIK3	ES	10	9	11	1.8E-04	0.118	-0.185	8.4E-01	5.5E-01	3.9E-01
PCPG	SIPA1L2_ES_20_19_21	SIPA1L2	ES	20	19	21	4.0E-05	0.129	0.178	1.0E-01	4.7E-01	2.6E-01
PCPG	SLAIN2_ES_8_6_9	SLAIN2	ES	8	6	9	1.0E-04	0.107	-0.038	4.9E-02	1.9E-02	1.7E-01
PCPG	SLC15A4_ES_3.2_2_3.4	SLC15A4	ES	3.2	2	3.4	4.9E-04	0.108	-0.054	1.2E-01	5.9E-01	1.0E+00
PCPG	SLC25A36_ES_6.1:6.2_4.1_7	SLC25A36	ES	6.1:6.2	4.1	7	6.2E-07	0.197	0.057	8.4E-02	3.1E-01	1.0E+00
PCPG	SLC26A10_AD_15.2_15.1_16	SLC26A10	AD	15.2	15.1	16	1.7E-05	0.156	-0.073	1.8E-01	7.1E-02	1.0E+00
PCPG	SLC2A8_ES_2:3_1_4	SLC2A8	ES	2:03	1	4	1.1E-04	0.107	0.205	4.4E-01	1.0E+00	1.0E+00
PCPG	SLCO2B1_RI_13.2_13.1_13.3	SLCO2B1	RI	13.2	13.1	13.3	1.0E-07	0.190	-0.557	2.4E-01	4.5E-01	2.3E-01
PCPG	SMARCA1_ES_13_12_14	SMARCA1	ES	13	12	14	2.2E-04	0.103	0.038	7.0E-01	7.4E-01	4.6E-01
PCPG	SMARCD3_RI_11.2_11.1_11.3	SMARCD3	RI	11.2	11.1	11.3	7.9E-06	0.134	-0.458	4.8E-01	8.6E-01	1.0E+00

cancer type	id	Gene Symbol	splice_type	Exon	From.Exon	To.Exon	anova.p	adj.r2	r	p.50	p.25	p.10
PCPG	SNAP91_ES_17_16_19	SNAP91	ES	17	16	19	7.0E-05	0.126	-0.186	3.4E-01	7.4E-01	1.0E+00
PCPG	SNX21_ES_3_2_4.1	SNX21	ES	3	2	4.1	1.9E-13	0.310	-0.171	4.6E-01	9.7E-01	3.2E-01
PCPG	SOGA2_ES_13_11_14	SOGA2	ES	13	11	14	3.4E-07	0.212	0.116	6.7E-01	5.6E-01	4.4E-01
PCPG	SORBS1_ES_9.1:9.2_8_10	SORBS1	ES	9.1:9.2	8	10	4.9E-18	0.471	-0.410	7.9E-01	4.5E-01	5.5E-01
PCPG	SORBS2_ES_20_19_22	SORBS2	ES	20	19	22	4.1E-06	0.180	-0.009	5.0E-01	6.5E-01	2.7E-01
PCPG	SORBS2_ES_20:22_19_23	SORBS2	ES	20:22	19	23	3.8E-07	0.175	-0.038	2.9E-01	5.6E-01	1.0E+00
PCPG	SPAG9_ES_30_29_31	SPAG9	ES	30	29	31	4.2E-07	0.169	0.014	1.2E-02	3.5E-02	2.5E-01
PCPG	SPATS2L_ES_10_9_11	SPATS2L	ES	10	9	11	2.5E-07	0.172	0.317	9.9E-02	6.1E-01	2.7E-01
PCPG	SPRTN_RI_4.2_4.1_4.3	SPRTN	RI	4.2	4.1	4.3	2.5E-09	0.214	-0.095	4.9E-01	4.4E-01	3.0E-01
PCPG	SPTAN1_ES_23_22_24	SPTAN1	ES	23	22	24	1.2E-07	0.207	0.285	2.7E-01	1.0E+00	1.0E+00
PCPG	SPTAN1_ES_38_36.1_39	SPTAN1	ES	38	36.1	39	3.3E-11	0.255	0.272	1.6E-02	2.6E-01	3.2E-01
PCPG	SS18_ES_15_14_16	SS18	ES	15	14	16	7.3E-10	0.233	0.039	2.7E-01	1.0E+00	1.0E+00
PCPG	SSR4_AD_1.2_1.1_2.2	SSR4	AD	1.2	1.1	2.2	3.5E-08	0.190	-0.506	5.7E-01	4.3E-01	8.5E-01
PCPG	STAG2_ES_35_34_36	STAG2	ES	35	34	36	2.5E-05	0.125	0.000	8.6E-01	5.7E-01	3.2E-01
PCPG	STAU2_ES_19_18.1_20	STAU2	ES	19	18.1	20	1.3E-04	0.107	0.062	1.7E-01	3.6E-01	8.0E-01
PCPG	STEAP3_ES_2_1.1_5.3	STEAP3	ES	2	1.1	5.3	1.4E-05	0.146	-0.053	6.6E-02	1.1E-01	1.0E+00
PCPG	STMN4_ES_7_6.1_8	STMN4	ES	7	6.1	8	1.9E-04	0.101	-0.129	1.0E-02	2.4E-02	1.0E+00
PCPG	STRA6_AD_18.2_18.1_19	STRA6	AD	18.2	18.1	19	1.2E-08	0.200	-0.849	7.1E-01	7.2E-01	3.5E-01
PCPG	STRADA_ES_3:4_2.2_6	STRADA	ES	3:04	2.2	6	6.1E-05	0.118	0.076	1.4E-02	1.5E-01	2.9E-01
PCPG	STRADA_RI_12.4_12.3_12.5	STRADA	RI	12.4	12.3	12.5	6.3E-05	0.111	0.193	8.6E-01	2.1E-01	2.4E-01
PCPG	STX16_AD_1.5_1.4_3	STX16	AD	1.5	1.4	3	4.1E-14	0.317	-0.485	3.6E-02	1.0E+00	1.0E+00
PCPG	STX16_ES_3_1.4_5.1	STX16	ES	3	1.4	5.1	4.9E-08	0.223	0.459	3.5E-02	1.1E-01	1.0E+00
PCPG	STX2_ES_10_9_11	STX2	ES	10	9	11	1.2E-04	0.109	0.281	4.5E-01	5.7E-01	2.7E-01
PCPG	STXBP5_ES_22:23_21_24	STXBP5	ES	22:23	21	24	2.6E-05	0.154	-0.398	9.2E-01	8.9E-01	1.0E+00
PCPG	SUMF2_ES_5.1:5.2:6_3_7	SUMF2	ES	5.1:5.2:6	3	7	5.9E-05	0.121	0.125	5.2E-01	6.9E-01	1.0E+00
PCPG	SVIL_ES_10:11:12_9_13	SVIL	ES	10:11:12	9	13	1.5E-09	0.251	-0.194	8.6E-01	5.1E-01	2.7E-01
PCPG	SYN1_AA_13.1_12_13.2	SYN1	AA	13.1	12	13.2	1.4E-04	0.103	0.027	5.0E-01	6.5E-01	7.0E-01
PCPG	SYNE1_ES_143_142_144	SYNE1	ES	143	142	144	2.0E-09	0.224	-0.374	7.1E-01	9.3E-01	2.3E-01
PCPG	SYNRG_ES_7_6_8	SYNRG	ES	7	6	8	5.3E-05	0.146	-0.409	6.1E-01	9.9E-01	3.4E-01
PCPG	TAGLN3_AA_2.2:2.3_1_2.4	TAGLN3	AA	2.2:2.3	1	2.4	2.1E-06	0.149	-0.385	7.7E-01	8.4E-01	1.5E-01
PCPG	TANK_ES_2.2:3_2.1_4	TANK	ES	2.2:3	2.1	4	3.2E-05	0.129	-0.187	5.4E-01	2.5E-01	1.8E-01
PCPG	TBC1D15_ES_10_9_11	TBC1D15	ES	10	9	11	1.5E-28	0.599	0.065	1.4E-01	4.1E-01	3.3E-01
PCPG	TBC1D23_ES_15_14_16	TBC1D23	ES	15	14	16	6.0E-06	0.154	0.007	9.3E-01	5.9E-01	1.0E+00
PCPG	TBRG1_ES_4_3_6	TBRG1	ES	4	3	6	5.3E-06	0.142	-0.244	4.2E-01	1.6E-01	1.6E-01
PCPG	TCEB1_AD_1.2:1.3_1.1_6	TCEB1	AD	1.2:1.3	1.1	6	8.3E-08	0.182	0.025	6.7E-01	9.1E-01	2.8E-01
PCPG	TCEB1_ES_1.2:5_1.1_6	TCEB1	ES	1.2:5	1.1	6	3.1E-05	0.119	-0.141	8.6E-02	4.0E-01	2.6E-01
PCPG	TCEB1_ES_4:6_1.2_7	TCEB1	ES	4:06	1.2	7	2.0E-05	0.129	-0.333	3.8E-01	9.5E-01	9.6E-01
PCPG	TERF1_ES_7_6_8	TERF1	ES	7	6	8	3.6E-05	0.118	0.224	5.6E-01	3.7E-01	3.8E-01
PCPG	TIA1_ES_5_4_7	TIA1	ES	5	4	7	9.2E-05	0.126	0.078	1.8E-01	3.0E-01	1.0E+00
PCPG	TJP1_AD_30.2_30.1_31	TJP1	AD	30.2	30.1	31	1.7E-05	0.126	-0.071	7.8E-01	4.9E-01	1.8E-01

cancer type	id	Gene Symbol	splice_type	Exon	From.Exon	To.Exon	anova.p	adj.r2	r	p.50	p.25	p.10
PCPG	TMEM106C_AD_5.2_5.1_6	TMEM106C	AD	5.2	5.1	6	1.1E-05	0.132	0.158	8.2E-01	5.8E-01	3.4E-01
PCPG	TMEM126B_ES_3_1_4	TMEM126B	ES	3	1	4	8.1E-05	0.109	-0.165	7.7E-01	3.1E-01	1.0E+00
PCPG	TMEM127_AA_2.1_1_2.2	TMEM127	AA	2.1	1	2.2	1.0E-04	0.126	-0.122	9.1E-01	7.7E-01	9.8E-01
PCPG	TMEM134_AD_2.2_2.1_3.1	TMEM134	AD	2.2	2.1	3.1	7.5E-05	0.109	-0.241	3.6E-01	3.4E-01	1.0E+00
PCPG	TMEM14B_ES_10:11_8_12	TMEM14B	ES	10:11	8	12	1.9E-06	0.149	-0.014	8.9E-02	7.7E-02	1.2E-01
PCPG	TMEM14B_ES_3_2_5.1	TMEM14B	ES	3	2	5.1	5.0E-05	0.129	0.093	9.3E-01	7.5E-01	3.3E-01
PCPG	TMEM159_AA_2.1:2.2_1_2.3	TMEM159	AA	2.1:2.2	1	2.3	1.4E-04	0.125	-0.277	6.2E-01	5.5E-01	2.5E-01
PCPG	TMEM205_AD_2.2:2.3_2.1_2.6	TMEM205	AD	2.2:2.3	2.1	2.6	1.4E-07	0.176	-0.186	1.1E-01	1.2E-01	3.5E-01
PCPG	TMEM230_AD_1.3_1.2_4	TMEM230	AD	1.3	1.2	4	5.4E-05	0.119	-0.003	4.9E-01	5.7E-01	3.5E-01
PCPG	TMEM234_ES_4_3_5.1	TMEM234	ES	4	3	5.1	2.8E-05	0.122	-0.211	4.4E-01	2.4E-01	3.5E-01
PCPG	TMEM234_RI_5.5_5.4_5.6	TMEM234	RI	5.5	5.4	5.6	6.1E-07	0.160	0.235	8.6E-01	9.8E-01	3.2E-01
PCPG	TMEM234_RI_5.7_5.6_5.8	TMEM234	RI	5.7	5.6	5.8	1.1E-04	0.105	0.336	7.4E-01	5.2E-01	3.2E-01
PCPG	TMEM5_ES_3_2_4	TMEM5	ES	3	2	4	2.8E-05	0.128	-0.310	1.3E-01	5.8E-01	3.2E-01
PCPG	TMEM91_RI_7.2:7.3_7.1_7.4	TMEM91	RI	7.2:7.3	7.1	7.4	3.6E-05	0.117	-0.511	4.0E-01	3.1E-01	6.8E-01
PCPG	TMPO_ES_6:7:8_5.1_9	TMPO	ES	6:07:08	5.1	9	3.3E-12	0.293	-0.196	1.5E-01	1.1E-01	6.2E-02
PCPG	TMUB2_ES_4.4:4.5:4.7:4.8_4.3_5	TMUB2	ES	4.4:4.5:4.7:4.8	4.3	5	3.5E-04	0.121	0.006	5.4E-01	7.9E-02	8.2E-03
PCPG	TMX2_ES_3.2:3.3:4:5.1:5.3_2_6	TMX2	ES	3.2:3.3:4:5.1:5.3	2	6	5.4E-06	0.141	-0.115	7.1E-02	9.9E-01	1.0E+00
PCPG	TMX2_ES_3.3:4:5.1:5.2:5.3_2_6	TMX2	ES	3.3:4:5.1:5.2:5.3	2	6	1.0E-04	0.115	-0.328	2.0E-01	9.9E-01	1.0E+00
PCPG	TNC_ES_12:13:14:15:16:18:19_11_20	TNC	ES	:13:14:15:16:18:	11	20	2.9E-04	0.121	0.103	8.2E-01	3.3E-01	3.8E-01
PCPG	TNC_ES_12:13:14:15:16:19_11_20	TNC	ES	12:13:14:15:16:1	11	20	4.3E-06	0.169	0.163	6.3E-01	9.2E-01	3.4E-01
PCPG	TNRC18_ES_15_14_16	TNRC18	ES	15	14	16	6.6E-04	0.102	0.072	8.2E-01	3.3E-01	3.3E-01
PCPG	TPD52_ES_9_8_10	TPD52	ES	9	8	10	2.2E-05	0.137	0.037	2.5E-01	1.7E-01	1.7E-01
PCPG	TPD52L2_ES_4_3_5	TPD52L2	ES	4	3	5	1.3E-04	0.104	0.085	1.1E-01	3.3E-02	3.9E-02
PCPG	TPM1_AD_12.2_12.1_13.1	TPM1	AD	12.2	12.1	13.1	7.6E-05	0.120	0.025	3.9E-01	3.4E-01	1.0E+00
PCPG	TPM1_ES_12.1_11.1_13.1	TPM1	ES	12.1	11.1	13.1	4.2E-06	0.161	-0.112	6.6E-01	1.0E+00	1.0E+00
PCPG	TPM1_ES_8_7_9	TPM1	ES	8	7	9	8.9E-08	0.180	-0.032	4.7E-01	5.9E-01	3.4E-01
PCPG	TPM2_ES_6_5_7	TPM2	ES	6	5	7	1.4E-06	0.174	-0.476	1.6E-01	3.8E-01	1.0E+00
PCPG	TPT1_AD_1.2:1.3:1.4_1.1_2	TPT1	AD	1.2:1.3:1.4	1.1	2	7.5E-08	0.182	-0.130	4.5E-01	1.5E-01	1.0E+00
PCPG	TRAF3IP2_ES_2_1_3	TRAF3IP2	ES	2	1	3	4.6E-04	0.104	-0.273	8.2E-01	5.0E-01	1.0E+00
PCPG	TRERF1_ES_8.1_7_9	TRERF1	ES	8.1	7	9	2.3E-07	0.214	-0.068	4.9E-01	9.3E-01	2.6E-01
PCPG	TRPT1_ES_2.2_1_3	TRPT1	ES	2.2	1	3	9.0E-05	0.107	0.044	1.7E-01	7.6E-02	1.0E+00
PCPG	TSEN15_ES_5.1:5.2_3_6	TSEN15	ES	5.1:5.2	3	6	4.3E-07	0.171	-0.004	6.5E-01	3.1E-01	1.0E+00
PCPG	TSPAN19_ES_5_4_6	TSPAN19	ES	5	4	6	1.5E-06	0.155	-0.811	8.4E-01	5.3E-01	3.6E-01
PCPG	TSTD1_ES_2.3_1_3.1	TSTD1	ES	2.3	1	3.1	2.1E-04	0.106	0.064	2.2E-02	1.1E-01	3.5E-01
PCPG	TUSC3_ES_11_10_12	TUSC3	ES	11	10	12	2.1E-09	0.216	0.065	3.8E-01	5.2E-01	1.4E-01
PCPG	TXNDC11_ES_4_3_5	TXNDC11	ES	4	3	5	2.1E-04	0.117	0.337	6.5E-01	5.0E-01	3.4E-01
PCPG	UBE2D4_ES_2_1_3	UBE2D4	ES	2	1	3	3.2E-05	0.119	-0.161	6.4E-01	5.6E-01	9.6E-01
PCPG	UBP1_ES_8_7_9	UBP1	ES	8	7	9	2.7E-05	0.126	0.075	4.3E-01	7.9E-01	4.8E-01
PCPG	UBR2_ME_12|13_10_15	UBR2	ME	12|13	10	15	1.6E-04	0.131	-0.065	7.4E-01	1.0E+00	1.0E+00
PCPG	UQCRQ_AD_1.2:1.3:1.4_1.1_2	UQCRQ	AD	1.2:1.3:1.4	1.1	2	2.6E-04	0.103	-0.141	9.7E-01	1.9E-01	3.5E-01

cancer type	id	Gene Symbol	splice_type	Exon	From.Exon	To.Exon	anova.p	adj.r2	r	p.50	p.25	p.10
PCPG	VAV2_ES_16_15_17	VAV2	ES	16	15	17	8.3E-06	0.139	0.153	2.4E-02	6.4E-02	1.0E+00
PCPG	VDAC3_ES_5.2_4.1_6	VDAC3	ES	5.2	4.1	6	5.2E-13	0.291	0.167	2.6E-01	3.3E-01	1.0E+00
PCPG	VEGFA_ES_7.1:7.2_6_8.1	VEGFA	ES	7.1:7.2	6	8.1	1.6E-06	0.154	0.205	7.3E-01	1.7E-01	3.7E-01
PCPG	VEGFA_ES_7.1:7.2:8.1:8.2_6_9.1	VEGFA	ES	7.1:7.2:8.1:8.2	6	9.1	9.5E-07	0.159	0.317	5.9E-01	1.6E-01	3.2E-01
PCPG	VPS29_AD_3.2_3.1_5	VPS29	AD	3.2	3.1	5	4.6E-07	0.165	-0.051	3.6E-01	1.7E-01	3.5E-01
PCPG	VPS29_ES_3.1_1_5	VPS29	ES	3.1	1	5	1.6E-04	0.101	0.205	6.0E-01	9.4E-01	7.2E-01
PCPG	VRK2_ES_7_6.2_8	VRK2	ES	7	6.2	8	2.2E-07	0.199	-0.111	6.8E-01	6.4E-01	3.8E-01
PCPG	VSTM2A_AA_6.1_4.1_6.2	VSTM2A	AA	6.1	4.1	6.2	2.6E-06	0.153	-0.112	6.6E-01	3.4E-01	6.7E-01
PCPG	WIPI2_ES_2_1_3	WIPI2	ES	2	1	3	2.5E-04	0.108	-0.143	2.8E-01	5.5E-01	4.8E-01
PCPG	ZC3H18_ES_4_3_5	ZC3H18	ES	4	3	5	5.6E-05	0.124	-0.166	8.1E-01	2.7E-01	1.0E+00
PCPG	ZDHHC14_AA_9.1_8_9.2	ZDHHC14	AA	9.1	8	9.2	7.3E-06	0.142	-0.016	4.8E-01	6.1E-02	2.2E-01
PCPG	ZDHHC20_ES_12_11_13.1	ZDHHC20	ES	12	11	13.1	1.1E-05	0.168	-0.199	6.1E-01	7.4E-01	1.0E+00
PCPG	ZNF229_AD_6.3_6.2_7	ZNF229	AD	6.3	6.2	7	2.9E-06	0.183	-0.111	6.0E-01	4.0E-01	4.5E-01
PCPG	ZNF384_AD_1.2_1.1_2.2	ZNF384	AD	1.2	1.1	2.2	3.7E-05	0.135	-0.067	4.8E-01	9.3E-01	3.2E-01
PCPG	ZNF655_ES_4:5.3_3.2_7	ZNF655	ES	04:05.3	3.2	7	4.1E-04	0.101	-0.118	3.3E-01	5.0E-01	9.7E-01
PCPG	ZNF655_ES_6.1:6.2_5.3_7	ZNF655	ES	6.1:6.2	5.3	7	2.9E-05	0.153	0.199	8.8E-01	9.3E-01	1.0E+00
PCPG	ZNF707_AD_7.2_7.1_8.2	ZNF707	AD	7.2	7.1	8.2	1.7E-04	0.131	-0.142	5.5E-01	2.5E-01	3.7E-01
PCPG	ZSWIM7_ES_2:3_1.2_4	ZSWIM7	ES	2:03	1.2	4	1.1E-05	0.158	-0.323	4.8E-01	1.2E-01	3.9E-01
PRAD	ABCE1_ES_14_13_15	ABCE1	ES	14	13	15	4.8E-07	0.102	-0.656	8.5E-01	7.2E-01	1.0E+00
PRAD	ABI1_ES_12_11.2_13	ABI1	ES	12	11.2	13	2.7E-07	0.105	-0.140	9.4E-01	3.0E-01	3.8E-01
PRAD	ABLIM1_ES_16_15_17	ABLIM1	ES	16	15	17	4.8E-09	0.143	0.244	2.9E-01	5.1E-01	2.9E-01
PRAD	ACLY_ES_14_13_15	ACLY	ES	14	13	15	3.4E-10	0.145	0.106	3.7E-01	8.7E-01	2.0E-01
PRAD	ADAM15_ES_21.1:21.2_20_22.1	ADAM15	ES	21.1:21.2	20	22.1	7.7E-10	0.141	-0.187	9.8E-01	1.9E-01	3.2E-01
PRAD	AFMID_ES_7:8:9:10:11.1:12_6_13	AFMID	ES	7:8:9:10:11.1:12	6	13	3.6E-08	0.122	0.246	9.0E-01	4.9E-01	6.3E-01
PRAD	APLP2_ES_9_8_10	APLP2	ES	9	8	10	1.8E-15	0.210	0.325	2.0E-01	8.0E-01	3.7E-01
PRAD	APOD_ES_3_2_4	APOD	ES	3	2	4	9.3E-13	0.178	-0.756	2.6E-01	5.6E-01	9.7E-01
PRAD	ARAP1_ES_32_31_33	ARAP1	ES	32	31	33	3.6E-07	0.104	0.059	4.2E-01	1.3E-01	4.3E-01
PRAD	BAIAP2_ES_17.1_16.1_18.1	BAIAP2	ES	17.1	16.1	18.1	2.7E-11	0.161	0.320	4.2E-01	7.2E-01	2.3E-01
PRAD	BAIAP2_ES_17.1:17.2_16.1_18.1	BAIAP2	ES	17.1:17.2	16.1	18.1	3.8E-08	0.117	0.289	1.0E-01	3.2E-01	7.7E-01
PRAD	BCAS3_ES_28:29_27_30	BCAS3	ES	28:29:00	27	30	5.5E-09	0.129	-0.253	2.1E-01	8.5E-01	9.4E-01
PRAD	BCKDHB_RI_11.2_11.1_11.3	BCKDHB	RI	11.2	11.1	11.3	2.5E-08	0.120	0.056	4.2E-01	3.4E-01	9.8E-01
PRAD	CACNA1D_ME_8|9_7_10	CACNA1D	ME	8|9	7	10	2.7E-07	0.114	0.198	1.5E-01	3.5E-01	3.8E-01
PRAD	CD44_ES_10:11_5_12.1	CD44	ES	10:11	5	12.1	5.1E-17	0.239	0.009	8.1E-01	8.4E-01	2.1E-01
PRAD	CD44_ES_6_5_7	CD44	ES	6	5	7	3.0E-14	0.213	0.014	2.3E-01	2.5E-01	6.5E-01
PRAD	CD44_ES_6:7:8:9.1:9.2_5_10	CD44	ES	6:7:8:9.1:9.2	5	10	1.7E-10	0.161	0.066	2.6E-01	2.9E-01	5.0E-01
PRAD	CD44_ES_6:7:8:9.1:9.2:10:11_5_12.1	CD44	ES	:7:8:9.1:9.2:10:1	5	12.1	1.2E-15	0.220	0.097	3.5E-01	2.6E-01	9.1E-01
PRAD	CD44_ES_6:7:8:9.1:9.2:10:11:12.1:13:14	CD44	ES	.1:9.2:10:11:12.1	5	15	7.4E-10	0.141	0.356	2.5E-01	4.2E-01	7.9E-01
PRAD	CD44_ES_6:7:8:9.2_5_10	CD44	ES	6:7:8:9.2	5	10	1.9E-09	0.147	-0.003	7.8E-02	3.2E-01	4.7E-01
PRAD	CD44_ES_6:7:8:9.2:10:11_5_12.1	CD44	ES	6:7:8:9.2:10:11	5	12.1	2.1E-13	0.192	0.042	5.8E-01	2.8E-01	2.9E-01
PRAD	CD44_ES_6:7:8:9.2:10:11:12.1:13:14_5_	CD44	ES	:9.2:10:11:12.1:1	5	15	9.2E-10	0.140	0.315	2.2E-01	7.1E-01	8.5E-01

cancer type	id	Gene Symbol	splice_type	Exon	From.Exon	To.Exon	anova.p	adj.r2	r	p.50	p.25	p.10
PRAD	CD44_ES_7:8:9.1:9.2:10:11_5_12.1	CD44	ES	7:8:9.1:9.2:10:11	5	12.1	1.7E-09	0.138	0.062	8.4E-01	7.7E-01	3.2E-01
PRAD	CD44_ES_7:8:9.2:10:11_5_12.1	CD44	ES	7:8:9.2:10:11	5	12.1	4.1E-08	0.119	0.055	7.2E-01	1.3E-01	2.9E-01
PRAD	CDC42SE2_RI_5.2_5.1_5.3	CDC42SE2	RI	5.2	5.1	5.3	2.4E-09	0.134	-0.462	1.6E-01	1.7E-01	8.0E-01
PRAD	CEP57_RI_10.2_10.1_10.3	CEP57	RI	10.2	10.1	10.3	1.0E-08	0.126	-0.484	7.7E-01	8.3E-01	4.8E-01
PRAD	CSDE1_ES_2:3_1_4	CSDE1	ES	2:03	1	4	2.1E-07	0.111	0.046	9.1E-01	7.2E-01	9.6E-01
PRAD	CYB561A3_AA_6.6_6.4_6.7	CYB561A3	AA	6.6	6.4	6.7	2.0E-08	0.121	-0.246	2.3E-01	8.3E-01	2.4E-01
PRAD	EEF1B2_AA_1.3_1.1_1.4	EEF1B2	AA	1.3	1.1	1.4	6.9E-09	0.129	0.500	8.1E-01	6.8E-01	1.0E+00
PRAD	EFCAB14_ES_7_6_8	EFCAB14	ES	7	6	8	1.4E-12	0.176	-0.183	3.3E-02	4.3E-01	7.9E-02
PRAD	ESRP1_AD_12.2_12.1_13	ESRP1	AD	12.2	12.1	13	3.0E-09	0.132	-0.337	7.0E-02	5.6E-01	1.0E-01
PRAD	FAM13C_AA_8.1_7_8.2	FAM13C	AA	8.1	7	8.2	2.3E-13	0.189	0.561	4.2E-01	1.6E-01	2.5E-01
PRAD	FAM160A2_AD_8.2_8.1_9.1	FAM160A2	AD	8.2	8.1	9.1	1.4E-07	0.111	-0.084	2.2E-01	2.3E-01	2.0E-01
PRAD	FLAD1_ES_2.1:2.2:3:4.1:4.3_1.3_6.1	FLAD1	ES	2.1:2.2:3:4.1:4.3	1.3	6.1	1.4E-05	0.105	-0.382	1.9E-01	6.8E-01	3.3E-01
PRAD	FLNB_ES_27_26_28	FLNB	ES	27	26	28	4.2E-09	0.130	0.047	4.5E-01	4.6E-01	6.9E-01
PRAD	FLNB_ES_32.1_31_33	FLNB	ES	32.1	31	33	1.6E-10	0.150	-0.208	5.4E-01	8.8E-01	6.6E-01
PRAD	GK_ES_23_22_24	GK	ES	23	22	24	4.6E-07	0.104	-0.197	6.9E-01	4.9E-01	2.6E-01
PRAD	GPR116_ES_22_21_23	GPR116	ES	22	21	23	1.4E-24	0.316	-0.664	3.9E-01	8.9E-01	1.0E+00
PRAD	GSTT1_ES_3_1_4.2	GSTT1	ES	3	1	4.2	5.5E-07	0.130	-0.247	9.8E-01	9.7E-01	2.5E-01
PRAD	HNRNPC_ES_2.4:2.5:2.6_1_3.2	HNRNPC	ES	2.4:2.5:2.6	1	3.2	1.6E-08	0.123	-0.370	7.4E-01	5.5E-01	8.9E-01
PRAD	KCTD7_ES_13_12_14	KCTD7	ES	13	12	14	3.4E-07	0.105	0.382	9.9E-01	9.5E-01	8.3E-02
PRAD	KLC1_AD_13.3_13.2_18	KLC1	AD	13.3	13.2	18	1.9E-08	0.122	-0.130	2.6E-01	4.7E-01	1.4E-01
PRAD	KLK4_ES_5.2_4_6	KLK4	ES	5.2	4	6	1.5E-09	0.137	0.078	5.1E-02	2.5E-01	1.1E-01
PRAD	LGALS8_ES_11_10.1_12	LGALS8	ES	11	10.1	12	9.5E-08	0.112	-0.059	3.2E-01	9.4E-02	1.7E-01
PRAD	LIMCH1_ES_20_12.1_21	LIMCH1	ES	20	12.1	21	1.3E-07	0.114	-0.188	4.2E-01	8.4E-01	2.8E-01
PRAD	LPHN2_ES_15_14_16	LPHN2	ES	15	14	16	3.3E-09	0.136	-0.272	5.3E-01	9.3E-02	1.4E-01
PRAD	LPHN2_ES_28_27_31	LPHN2	ES	28	27	31	2.7E-13	0.191	0.444	5.7E-01	6.4E-01	4.4E-01
PRAD	LRRFIP2_ES_7:8_5_18	LRRFIP2	ES	7:08	5	18	5.5E-09	0.134	0.208	9.0E-01	9.1E-01	9.4E-01
PRAD	LTBP3_ES_25_24_26	LTBP3	ES	25	24	26	7.8E-07	0.100	0.340	7.6E-01	9.6E-01	1.4E-01
PRAD	MAP2_ES_16_15_17	MAP2	ES	16	15	17	1.0E-08	0.127	0.036	9.3E-01	9.6E-01	4.2E-01
PRAD	MAP7_ES_9_8_10	MAP7	ES	9	8	10	3.1E-07	0.105	-0.133	1.1E-01	8.3E-01	5.4E-01
PRAD	MAPRE3_AD_5.2_5.1_6	MAPRE3	AD	5.2	5.1	6	1.7E-07	0.109	-0.115	4.1E-01	5.8E-02	1.3E-01
PRAD	MARK2_ES_18:19_17_20	MARK2	ES	18:19	17	20	2.1E-07	0.110	0.050	6.1E-02	5.8E-02	1.1E-01
PRAD	MBNL2_ES_7_6.3_8	MBNL2	ES	7	6.3	8	6.1E-08	0.121	-0.014	1.8E-01	3.7E-01	4.8E-01
PRAD	MFF_ES_3:4_1_5	MFF	ES	3:04	1	5	3.7E-07	0.104	-0.014	4.4E-01	2.6E-01	6.4E-01
PRAD	NF2_ES_16.1_15_17	NF2	ES	16.1	15	17	2.0E-09	0.135	0.014	1.0E+00	3.4E-01	4.8E-01
PRAD	ORMDL1_ES_2.2_1_2.4	ORMDL1	ES	2.2	1	2.4	2.6E-09	0.134	0.110	8.0E-02	1.3E-01	9.0E-01
PRAD	PARP8_ES_9_8_10	PARP8	ES	9	8	10	1.9E-07	0.110	0.072	8.5E-01	9.3E-01	9.0E-01
PRAD	PDE9A_ES_6_5_7	PDE9A	ES	6	5	7	1.0E-10	0.152	0.408	4.9E-02	3.0E-01	4.2E-01
PRAD	PEX10_ES_2_1_3	PEX10	ES	2	1	3	5.2E-39	0.443	0.793	7.2E-01	6.8E-01	9.2E-01
PRAD	PEX10_ES_4.2:5.1_4.1_5.2	PEX10	ES	4.2:5.1	4.1	5.2	1.5E-18	0.262	-0.562	9.5E-01	2.7E-01	5.4E-01
PRAD	PIGF_ES_6_5_7	PIGF	ES	6	5	7	4.6E-07	0.102	-0.071	7.7E-01	9.5E-02	2.6E-01

cancer type	id	Gene Symbol	splice_type	Exon	From.Exon	To.Exon	anova.p	adj.r2	r	p.50	p.25	p.10
PRAD	PLA2G2A_ES_2_1_3	PLA2G2A	ES	2	1	3	5.2E-14	0.193	0.657	1.9E-01	1.2E-01	3.2E-01
PRAD	PRICKLE4_AA_8.1_7_8.2	PRICKLE4	AA	8.1	7	8.2	9.6E-11	0.154	-0.136	3.8E-01	2.7E-01	2.4E-01
PRAD	RABGGTA_RI_1.2:1.3_1.1_1.4	RABGGTA	RI	1.2:1.3	1.1	1.4	4.3E-07	0.103	0.279	2.2E-01	7.4E-01	4.8E-01
PRAD	REPS1_ES_9.3_9.1_10	REPS1	ES	9.3	9.1	10	1.6E-08	0.122	0.149	5.7E-02	1.9E-01	1.0E-01
PRAD	RHBDD2_ES_2_1_3	RHBDD2	ES	2	1	3	5.4E-10	0.142	-0.089	1.6E-01	7.0E-01	8.9E-01
PRAD	RLN2_ES_2_1_3	RLN2	ES	2	1	3	6.4E-14	0.237	-0.722	2.0E-01	6.9E-01	6.9E-01
PRAD	SEC31A_ES_26.1:26.2_25.1_28	SEC31A	ES	26.1:26.2	25.1	28	7.8E-08	0.113	0.034	7.8E-01	6.1E-01	1.8E-01
PRAD	SLC25A27_AA_10.1_9_10.2	SLC25A27	AA	10.1	9	10.2	4.1E-08	0.120	-0.101	7.1E-01	3.5E-01	1.0E+00
PRAD	SLC7A2_ME_9|10_8_11	SLC7A2	ME	9|10	8	11	1.8E-08	0.123	-0.076	8.2E-01	8.1E-01	3.6E-01
PRAD	SREBF1_ES_2_1_3.2	SREBF1	ES	2	1	3.2	4.6E-11	0.156	0.650	7.6E-01	9.5E-01	5.5E-01
PRAD	SUGP2_ES_12.5_12.3_14	SUGP2	ES	12.5	12.3	14	3.9E-16	0.220	-0.364	2.9E-01	8.6E-01	7.3E-01
PRAD	SYNE4_ES_2_1_3	SYNE4	ES	2	1	3	7.9E-09	0.129	-0.119	1.5E-01	2.5E-01	1.0E+00
PRAD	TBC1D13_ES_3_2_4	TBC1D13	ES	3	2	4	1.2E-08	0.132	-0.054	2.4E-01	6.6E-01	3.2E-01
PRAD	TBX3_ES_3_2_4	TBX3	ES	3	2	4	4.8E-09	0.130	-0.033	2.1E-01	1.1E-01	1.9E-01
PRAD	TBXAS1_ES_8_7.1_9	TBXAS1	ES	8	7.1	9	3.8E-08	0.118	-0.431	6.8E-01	4.8E-01	1.0E+00
PRAD	TC2N_AD_2.2_2.1_3	TC2N	AD	2.2	2.1	3	1.5E-08	0.124	-0.031	3.9E-01	4.3E-01	2.2E-01
PRAD	TCEB1_AD_1.2_1.1_6	TCEB1	AD	1.2	1.1	6	8.7E-08	0.112	-0.058	9.6E-01	6.7E-01	8.4E-02
PRAD	TCEB1_ES_5_1.1_6	TCEB1	ES	5	1.1	6	2.1E-07	0.117	-0.092	3.6E-01	5.0E-01	8.1E-01
PRAD	THYN1_AD_1.2_1.1_1.4	THYN1	AD	1.2	1.1	1.4	1.9E-15	0.211	0.645	6.7E-01	3.2E-01	4.7E-01
PRAD	THYN1_RI_1.2:1.3_1.1_1.4	THYN1	RI	1.2:1.3	1.1	1.4	7.7E-11	0.153	0.381	4.7E-01	3.9E-01	3.9E-01
PRAD	TMEM106C_AD_5.2_5.1_6	TMEM106C	AD	5.2	5.1	6	1.2E-08	0.124	0.085	4.9E-01	4.0E-02	1.1E-01
PRAD	TMEM125_AA_2.1_1_2.2	TMEM125	AA	2.1	1	2.2	3.7E-07	0.105	-0.219	9.3E-01	3.2E-01	1.0E+00
PRAD	TRAPPC9_ES_6_5_7	TRAPPC9	ES	6	5	7	1.3E-09	0.137	-0.140	9.9E-01	7.2E-01	9.2E-01
PRAD	TRDMT1_ES_3_2_4	TRDMT1	ES	3	2	4	2.2E-06	0.102	0.079	9.7E-02	1.7E-01	8.7E-01
PRAD	TSC2_ES_27_26_28.1	TSC2	ES	27	26	28.1	1.6E-09	0.136	-0.124	5.8E-01	8.9E-02	1.0E+00
PRAD	TSC2_ES_27:28.1_26_28.2	TSC2	ES	27:28.1	26	28.2	3.1E-08	0.119	-0.109	9.1E-01	4.1E-01	9.5E-01
PRAD	TSPAN19_ES_5_4_6	TSPAN19	ES	5	4	6	2.2E-11	0.162	-0.810	9.5E-01	6.0E-01	1.8E-01
PRAD	USO1_ES_14_13_15	USO1	ES	14	13	15	1.5E-28	0.348	0.181	7.9E-01	8.9E-01	6.2E-01
PRAD	UTRN_ES_67_66_68	UTRN	ES	67	66	68	4.8E-09	0.130	0.083	8.1E-01	5.6E-01	3.8E-01
PRAD	VLDLR_ES_16_15_17	VLDLR	ES	16	15	17	9.6E-09	0.127	-0.367	9.8E-01	6.9E-01	1.0E+00
PRAD	VPS39_ES_3_2_4	VPS39	ES	3	2	4	2.6E-25	0.323	0.092	4.8E-01	6.5E-02	3.4E-01
PRAD	WDR24_ES_1.6:2:3_1.5_4	WDR24	ES	1.6:2:3	1.5	4	9.6E-08	0.113	0.317	4.7E-01	9.2E-01	6.8E-01
PRAD	WNK2_ES_26:27_25.3_28	WNK2	ES	26:27:00	25.3	28	2.6E-07	0.108	-0.470	6.2E-01	6.1E-02	2.5E-01
PRAD	ZBTB45_AD_1.2_1.1_3	ZBTB45	AD	1.2	1.1	3	4.7E-09	0.132	0.215	6.7E-02	4.3E-01	9.8E-01
PRAD	ZNF692_RI_6.3_6.2_6.4	ZNF692	RI	6.3	6.2	6.4	1.6E-07	0.109	0.553	7.3E-01	3.3E-01	1.0E+00
READ	ARID1B_ES_12_11_13	ARID1B	ES	12	11	13	1.1E-04	0.148	-0.168	3.2E-01	2.2E-03	3.0E-02
READ	ATP2C1_AA_3.2_2_3.3	ATP2C1	AA	3.2	2	3.3	4.7E-04	0.140	0.065	7.1E-01	2.1E-01	9.0E-02
READ	BCL11A_AD_5.2:5.3:5.4_5.1_6	BCL11A	AD	5.2:5.3:5.4	5.1	6	1.9E-04	0.147	-0.495	9.3E-01	6.5E-01	5.3E-01
READ	C20orf24_ES_3:4.1_2_5	C20orf24	ES	03:04.1	2	5	8.3E-05	0.149	0.006	7.5E-01	7.4E-01	9.4E-01
READ	CCDC112_ES_10_9_11	CCDC112	ES	10	9	11	5.8E-06	0.211	-0.026	4.5E-01	1.3E-01	3.8E-01

cancer type	id	Gene Symbol	splice_type	Exon	From.Exon	To.Exon	anova.p	adj.r2	r	p.50	p.25	p.10
READ	CELF2_RI_16.2_16.1_16.3	CELF2	RI	16.2	16.1	16.3	2.5E-04	0.138	-0.466	2.8E-02	4.4E-01	6.3E-01
READ	CEP63_ES_12_11_13	CEP63	ES	12	11	13	1.8E-04	0.154	0.080	7.1E-01	5.3E-01	8.9E-01
READ	CPSF3L_ES_4:5.2:6.2_1_7.1	CPSF3L	ES	4:5.2:6.2	1	7.1	4.7E-06	0.220	0.043	4.7E-01	3.5E-01	3.2E-01
READ	CPSF4_AA_9.1_8_9.2	CPSF4	AA	9.1	8	9.2	2.7E-04	0.132	-0.157	2.1E-01	8.0E-01	8.3E-01
READ	CRAT_ES_2_1_4.1	CRAT	ES	2	1	4.1	2.1E-04	0.141	-0.163	4.6E-01	1.8E-01	8.5E-01
READ	CYP2R1_ES_2.2:3_2.1_4.2	CYP2R1	ES	2.2:3	2.1	4.2	6.3E-04	0.139	-0.037	7.1E-01	2.7E-01	8.1E-01
READ	DNM1L_ES_17:18_16_19	DNM1L	ES	17:18	16	19	8.7E-04	0.118	-0.254	2.4E-01	4.8E-01	9.7E-01
READ	E2F5_AA_8.1_7_8.2	E2F5	AA	8.1	7	8.2	5.6E-04	0.126	-0.122	8.1E-01	5.4E-01	8.4E-01
READ	EFHC1_RI_5.2_5.1_5.3	EFHC1	RI	5.2	5.1	5.3	5.3E-04	0.136	0.256	9.3E-01	9.7E-01	7.5E-01
READ	EPS15L1_ES_22_21_23.1	EPS15L1	ES	22	21	23.1	1.4E-05	0.187	-0.045	5.6E-01	2.6E-01	1.2E-01
READ	FAM219B_ES_3.1:3.2_2_4	FAM219B	ES	3.1:3.2	2	4	9.9E-04	0.115	0.101	4.1E-01	2.8E-01	1.6E-01
READ	GMPR2_AD_1.2_1.1_2.3	GMPR2	AD	1.2	1.1	2.3	3.5E-04	0.129	0.093	1.0E+00	7.8E-01	6.3E-01
READ	GNPDA1_ES_2.4_1_2.6	GNPDA1	ES	2.4	1	2.6	1.9E-04	0.154	-0.029	4.1E-01	9.0E-01	2.6E-01
READ	GTF2IRD1_AA_20.1_19_20.2	GTF2IRD1	AA	20.1	19	20.2	4.5E-06	0.198	-0.226	1.0E-01	1.9E-01	2.0E-01
READ	INO80C_ES_3:4.1:4.2:5.1:5.2:6:7_1_8	INO80C	ES	:4.1:4.2:5.1:5.2:6	1	8	1.3E-04	0.165	-0.159	8.1E-01	9.3E-01	6.2E-01
READ	KDM6A_ES_16_15_17	KDM6A	ES	16	15	17	5.4E-04	0.137	0.126	6.5E-01	2.5E-01	8.1E-01
READ	LRR1_ES_4_3_5	LRR1	ES	4	3	5	4.4E-04	0.153	-0.248	7.2E-01	1.8E-01	4.8E-01
READ	LYSMD1_AD_1.2_1.1_2	LYSMD1	AD	1.2	1.1	2	1.3E-07	0.251	0.170	2.8E-01	4.0E-01	7.9E-01
READ	MACF1_ES_107_106_108	MACF1	ES	107	106	108	9.0E-04	0.112	-0.075	7.3E-01	7.8E-01	7.8E-01
READ	MELK_ES_3_2_4	MELK	ES	3	2	4	6.7E-04	0.130	-0.198	9.2E-01	4.3E-01	9.9E-02
READ	METTL10_ES_6_5_7	METTL10	ES	6	5	7	1.0E-04	0.152	-0.221	8.2E-01	7.2E-01	4.9E-01
READ	MORF4L2_ES_4_3.2_5.1	MORF4L2	ES	4	3.2	5.1	8.1E-04	0.114	-0.180	6.5E-02	2.6E-01	4.3E-01
READ	MRPL55_AA_2.2:2.3:2.4:2.5:2.6:2.7:2.8_	MRPL55	AA	2.3:2.4:2.5:2.6:2.7	1.1	2.9	9.9E-06	0.214	0.003	9.3E-01	7.5E-01	4.9E-01
READ	MSI2_AA_18.1_17_18.2	MSI2	AA	18.1	17	18.2	6.5E-06	0.189	0.238	5.1E-01	8.4E-01	4.5E-01
READ	OFD1_ES_11_10_13	OFD1	ES	11	10	13	2.0E-04	0.150	-0.061	9.6E-01	8.8E-01	9.9E-01
READ	PRAP1_AD_4.3_4.2_5	PRAP1	AD	4.3	4.2	5	1.3E-04	0.137	0.733	4.0E-01	8.8E-03	1.0E-01
READ	PTK2_ES_39.2_37_39.5	PTK2	ES	39.2	37	39.5	4.4E-04	0.124	-0.210	3.8E-01	2.7E-01	5.4E-01
READ	RHOA_ES_4_2_6	RHOA	ES	4	2	6	6.1E-05	0.166	0.006	2.6E-01	3.2E-01	7.5E-01
READ	RPL21_AD_1.3:1.4_1.2_2	RPL21	AD	1.3:1.4	1.2	2	5.3E-04	0.120	-0.060	9.6E-01	4.9E-01	6.6E-01
READ	RPL22L1_AA_3.1_2.2_3.2	RPL22L1	AA	3.1	2.2	3.2	9.1E-04	0.115	-0.760	8.6E-01	9.8E-01	1.5E-01
READ	SNUPN_AD_1.2_1.1_4	SNUPN	AD	1.2	1.1	4	7.4E-08	0.278	0.241	6.3E-01	8.3E-01	9.4E-01
READ	TMC5_AD_1.2_1.1_3	TMC5	AD	1.2	1.1	3	2.3E-04	0.158	-0.160	1.4E-01	9.0E-02	7.2E-02
READ	TMEM205_ES_2.2:2.3:2.5_2.1_2.6	TMEM205	ES	2.2:2.3:2.5	2.1	2.6	5.0E-06	0.232	0.271	5.3E-01	8.0E-01	2.2E-01
READ	TMUB2_ES_2.3:2.4:2.5:3_2.2_4.3	TMUB2	ES	2.3:2.4:2.5:3	2.2	4.3	8.3E-06	0.209	-0.293	2.7E-01	3.0E-01	3.2E-01
READ	TPT1_AD_1.2:1.3:1.4_1.1_2	TPT1	AD	1.2:1.3:1.4	1.1	2	1.7E-04	0.140	-0.248	7.5E-01	2.5E-01	1.6E-01
READ	TRAPPC6B_ES_4_3_5	TRAPPC6B	ES	4	3	5	8.7E-04	0.124	-0.018	1.5E-01	2.8E-02	1.4E-01
READ	YY1AP1_ES_3.2_2_4.1	YY1AP1	ES	3.2	2	4.1	1.8E-05	0.181	0.130	2.0E-01	2.6E-02	4.4E-01
READ	ZNF646_RI_2.3_2.2_2.4	ZNF646	RI	2.3	2.2	2.4	9.1E-05	0.148	-0.018	6.6E-02	8.1E-01	5.1E-02
SKCM	ANKS3_ES_5.1:5.2_3_6	ANKS3	ES	5.1:5.2	3	6	1.1E-04	0.333	-0.104	6.1E-01	9.9E-01	9.9E-01
SKCM	BANP_AA_14.1_13_14.2	BANP	AA	14.1	13	14.2	8.0E-04	0.219	-0.053	9.2E-01	3.4E-01	4.0E-01

cancer type	id	Gene Symbol	splice_type	Exon	From.Exon	To.Exon	anova.p	adj.r2	r	p.50	p.25	p.10
SKCM	C8orf59_ES_2.1:2.2_1_3.2	C8orf59	ES	2.1:2.2	1	3.2	1.7E-05	0.307	0.232	7.3E-01	6.8E-01	3.4E-01
SKCM	CADM1_ES_10_9_12	CADM1	ES	10	9	12	1.6E-04	0.266	-0.277	2.6E-01	4.9E-02	4.1E-01
SKCM	CREB1_ES_5_4_6	CREB1	ES	5	4	6	4.2E-04	0.247	-0.143	6.9E-01	8.4E-01	4.6E-01
SKCM	CSDE1_ES_2:3_1_4	CSDE1	ES	2:03	1	4	2.6E-04	0.291	0.164	8.3E-01	3.1E-01	3.6E-02
SKCM	EDEM2_ES_10:11:12_9_13	EDEM2	ES	10:11:12	9	13	3.3E-04	0.258	-0.065	7.0E-01	9.0E-01	7.6E-01
SKCM	ESRP1_ES_15_13_16	ESRP1	ES	15	13	16	4.7E-05	0.311	0.061	7.3E-01	3.1E-01	3.4E-01
SKCM	FAM214B_RI_4.2_4.1_4.3	FAM214B	RI	4.2	4.1	4.3	2.2E-04	0.317	-0.335	6.4E-01	6.2E-01	3.2E-01
SKCM	FCRLA_ES_4:5_1_7	FCRLA	ES	4:05	1	7	3.7E-04	0.271	-0.058	9.6E-01	8.7E-01	3.9E-01
SKCM	FIP1L1_ES_9_8_10	FIP1L1	ES	9	8	10	8.3E-04	0.208	0.239	3.9E-01	4.1E-02	3.6E-01
SKCM	HAUS8_AA_5.1_4_5.2	HAUS8	AA	5.1	4	5.2	4.8E-04	0.236	0.154	1.3E-03	4.6E-02	2.1E-01
SKCM	OPA1_ES_5_4_6	OPA1	ES	5	4	6	3.8E-05	0.321	0.186	2.4E-01	7.8E-01	1.0E-01
SKCM	P4HA1_ME_10|11_9_12.1	P4HA1	ME	10|11	9	12.1	5.0E-04	0.222	-0.003	4.6E-01	4.5E-01	7.5E-01
SKCM	PAM_ES_23.1:23.2_22_25.1	PAM	ES	23.1:23.2	22	25.1	3.7E-04	0.237	0.352	3.2E-01	8.3E-01	6.9E-01
SKCM	PPIA_AD_2.2_2.1_3	PPIA	AD	2.2	2.1	3	5.5E-04	0.219	-0.061	4.8E-01	5.8E-01	9.2E-01
SKCM	PPIA_ES_5.1:6_4_7	PPIA	ES	5.1:6	4	7	9.4E-04	0.205	-0.099	7.8E-01	4.6E-01	5.8E-01
SKCM	SEC31A_ES_26.2:27_26.1_28	SEC31A	ES	26.2:27	26.1	28	3.0E-04	0.236	-0.008	8.6E-01	8.1E-01	1.0E+00
SKCM	SEMA4F_ES_5:6:7:8_4_9	SEMA4F	ES	5:6:7:8	4	9	3.3E-04	0.254	0.172	7.5E-01	7.0E-01	4.0E-01
SKCM	SMG7_AD_16.2_16.1_17	SMG7	AD	16.2	16.1	17	2.4E-04	0.244	-0.068	1.9E-01	2.6E-01	5.6E-01
SKCM	SREBF1_ES_2_1_3.2	SREBF1	ES	2	1	3.2	8.3E-04	0.214	0.324	9.9E-01	9.4E-01	6.5E-01
SKCM	ST3GAL3_ES_16.1:16.2:19.1:19.2:20_15.	ST3GAL3	ES	.1:16.2:19.1:19.2	15.1	21	3.7E-04	0.262	-0.088	5.4E-01	5.4E-01	5.3E-01
SKCM	TREX2_ES_9_8.2_10	TREX2	ES	9	8.2	10	7.8E-05	0.307	0.096	2.2E-01	6.8E-02	9.7E-01
SKCM	ZC3H14_ES_13:14.1:14.2_10_15	ZC3H14	ES	13:14.1:14.2	10	15	1.5E-04	0.264	-0.212	4.1E-01	9.6E-01	7.6E-01
STAD	ACOT9_ES_6_5.1_7.1	ACOT9	ES	6	5.1	7.1	2.9E-18	0.202	-0.044	4.0E-03	6.3E-03	6.0E-01
STAD	ACSM3_ES_5_4_6	ACSM3	ES	5	4	6	1.9E-12	0.150	-0.060	1.7E-01	9.8E-02	3.0E-02
STAD	ADD3_ES_15_14_16	ADD3	ES	15	14	16	2.3E-12	0.140	0.382	1.6E-02	2.2E-02	1.2E-02
STAD	ADNP_ES_2_1_4.2	ADNP	ES	2	1	4.2	8.9E-11	0.122	0.330	2.6E-01	2.8E-01	2.9E-01
STAD	AKAP9_ES_20_19_21	AKAP9	ES	20	19	21	2.6E-15	0.178	-0.086	2.6E-01	4.9E-02	4.8E-01
STAD	ALG2_ES_2_1_3	ALG2	ES	2	1	3	1.5E-15	0.173	-0.248	5.5E-02	4.8E-01	9.7E-01
STAD	ALG8_ES_14_13_15	ALG8	ES	14	13	15	5.1E-19	0.209	0.154	5.4E-02	3.8E-01	6.4E-01
STAD	ANK3_ME_26|27_25_29.1	ANK3	ME	26|27	25	29.1	1.0E-07	0.108	-0.048	1.9E-03	2.6E-02	1.5E-02
STAD	ANKS6_ES_3_2_4	ANKS6	ES	3	2	4	3.6E-09	0.109	0.028	8.7E-01	4.7E-01	8.8E-01
STAD	ANXA6_ES_22_21_23	ANXA6	ES	22	21	23	4.1E-09	0.103	0.011	2.6E-01	7.4E-01	4.8E-01
STAD	AP1B1_ES_24_23_25	AP1B1	ES	24	23	25	2.9E-13	0.149	-0.126	4.8E-02	6.5E-02	2.5E-01
STAD	APP_ES_10_9_11	APP	ES	10	9	11	1.7E-09	0.107	-0.099	7.4E-02	1.5E-02	7.8E-02
STAD	ARHGEF1_ES_15_14_16	ARHGEF1	ES	15	14	16	2.7E-09	0.105	0.178	7.5E-01	4.7E-01	7.5E-01
STAD	ARHGEF11_ES_39_38_40	ARHGEF11	ES	39	38	40	3.0E-19	0.211	-0.203	1.5E-01	4.4E-02	1.6E-04
STAD	ARL6IP4_AD_2.2_2.1_3.1	ARL6IP4	AD	2.2	2.1	3.1	1.2E-15	0.175	0.053	8.4E-01	6.4E-01	2.7E-01
STAD	ARNT_ES_7_6_8	ARNT	ES	7	6	8	7.5E-09	0.100	0.066	1.9E-01	2.2E-01	6.8E-01
STAD	ATG16L1_ES_8_7_10	ATG16L1	ES	8	7	10	5.7E-12	0.137	-0.325	1.4E-01	5.2E-01	6.8E-01
STAD	ATP11C_ES_32_29_33	ATP11C	ES	32	29	33	5.3E-09	0.103	0.106	3.5E-01	2.3E-01	2.5E-01

cancer type	id	Gene Symbol	splice_type	Exon	From.Exon	To.Exon	anova.p	adj.r2	r	p.50	p.25	p.10
STAD	ATP2C1_AA_3.2_2_3.3	ATP2C1	AA	3.2	2	3.3	2.6E-20	0.223	-0.282	3.9E-01	2.9E-02	9.4E-02
STAD	ATP2C1_ES_3.2:3.3_2_4	ATP2C1	ES	3.2:3.3	2	4	2.4E-10	0.118	-0.123	8.1E-01	5.1E-01	7.4E-01
STAD	BAIAP2_ES_17.1:17.2_16.1_18.1	BAIAP2	ES	17.1:17.2	16.1	18.1	5.4E-12	0.137	0.326	5.9E-01	6.2E-01	9.5E-01
STAD	BICD2_RI_7.2_7.1_7.3	BICD2	RI	7.2	7.1	7.3	5.6E-12	0.135	-0.313	3.4E-03	2.4E-03	2.2E-01
STAD	BOD1L1_ES_2_1_3	BOD1L1	ES	2	1	3	1.9E-31	0.322	-0.304	9.3E-01	7.4E-01	2.5E-01
STAD	C16orf13_ES_2_1_3	C16orf13	ES	2	1	3	2.5E-10	0.117	-0.089	5.6E-02	3.9E-01	4.5E-01
STAD	C1orf122_AD_1.2_1.1_1.4	C1orf122	AD	1.2	1.1	1.4	2.2E-10	0.118	0.015	3.1E-01	7.8E-01	1.6E-01
STAD	CA5B_ES_4_3_5	CA5B	ES	4	3	5	2.3E-11	0.129	0.636	2.1E-02	9.5E-02	5.8E-01
STAD	CAMK2D_ES_17_14.2_18	CAMK2D	ES	17	14.2	18	9.9E-12	0.133	0.043	8.6E-01	5.6E-01	9.9E-01
STAD	CAMK2G_ES_13_12_14	CAMK2G	ES	13	12	14	1.5E-13	0.152	0.476	1.2E-03	7.0E-03	1.0E-01
STAD	CAMK2G_ES_15_14_17	CAMK2G	ES	15	14	17	5.0E-09	0.126	0.416	3.4E-01	3.3E-01	2.7E-01
STAD	CAMKK2_ES_17_16_19.1	CAMKK2	ES	17	16	19.1	3.5E-21	0.230	0.137	2.5E-03	1.4E-02	5.3E-01
STAD	CAST_ES_7.1:8.2_5.2_9	CAST	ES	7.1:8.2	5.2	9	5.1E-09	0.117	-0.015	9.8E-02	1.0E-01	3.7E-02
STAD	CAST_ES_7.1:8.2:9_5.2_10	CAST	ES	7.1:8.2:9	5.2	10	1.3E-11	0.158	0.015	2.2E-02	8.0E-04	2.6E-02
STAD	CAST_ES_8.2_7.1_9	CAST	ES	8.2	7.1	9	2.3E-15	0.172	0.083	6.3E-03	7.7E-02	1.2E-01
STAD	CAST_ES_8.2:9_7.1_10	CAST	ES	8.2:9	7.1	10	2.6E-10	0.120	-0.077	3.3E-02	4.7E-03	8.6E-03
STAD	CBFA2T2_ES_3_1_6	CBFA2T2	ES	3	1	6	5.8E-09	0.105	0.234	5.9E-01	8.0E-01	9.6E-01
STAD	CC2D2A_ES_5_4_6	CC2D2A	ES	5	4	6	6.6E-09	0.111	-0.051	3.8E-01	3.2E-02	2.2E-03
STAD	CCDC112_ES_10_9_11	CCDC112	ES	10	9	11	2.2E-29	0.306	-0.090	5.1E-01	8.9E-02	3.8E-01
STAD	CCDC138_ES_2_1_3	CCDC138	ES	2	1	3	1.1E-11	0.162	0.020	1.5E-01	1.7E-01	4.8E-01
STAD	CCDC50_ES_6_5_7	CCDC50	ES	6	5	7	1.5E-14	0.163	-0.386	1.3E-01	4.1E-03	4.0E-03
STAD	CCNDBP1_ES_10.1_9_10.3	CCNDBP1	ES	10.1	9	10.3	8.7E-63	0.540	0.144	6.6E-01	9.1E-01	1.5E-01
STAD	CCNDBP1_RI_10.2_10.1_10.3	CCNDBP1	RI	10.2	10.1	10.3	9.6E-10	0.112	-0.081	5.6E-01	8.9E-01	3.7E-01
STAD	CCSER2_ES_11_10_12	CCSER2	ES	11	10	12	2.3E-14	0.161	0.515	1.2E-01	2.3E-02	1.8E-02
STAD	CD46_ES_13_12_14	CD46	ES	13	12	14	2.4E-10	0.117	-0.344	8.8E-02	2.8E-02	1.1E-01
STAD	CD47_ES_9:10_8_11	CD47	ES	9:10	8	11	2.9E-10	0.116	0.236	1.7E-01	9.6E-03	2.9E-02
STAD	CD99_ES_10_9_11	CD99	ES	10	9	11	3.2E-13	0.167	0.212	1.0E-01	5.0E-01	2.4E-01
STAD	CDC16_ES_2_1_3.1	CDC16	ES	2	1	3.1	1.0E-40	0.422	0.154	8.0E-01	4.3E-01	2.6E-01
STAD	CDK12_AA_14.1_13_14.2	CDK12	AA	14.1	13	14.2	3.5E-10	0.115	-0.085	1.3E-01	2.5E-01	2.0E-01
STAD	CEACAM5_ES_5.1:5.2:6:7_3_8	CEACAM5	ES	5.1:5.2:6:7	3	8	9.6E-08	0.109	-0.193	3.9E-01	1.6E-01	8.2E-01
STAD	CIZ1_ES_6_5_7	CIZ1	ES	6	5	7	2.4E-12	0.139	-0.175	8.9E-01	9.3E-01	8.6E-01
STAD	CLEC16A_ES_24_23_25	CLEC16A	ES	24	23	25	2.8E-10	0.120	-0.092	1.8E-01	1.9E-01	2.4E-01
STAD	CLIP1_ES_9:10_8_11.1	CLIP1	ES	9:10	8	11.1	2.4E-09	0.106	0.431	6.9E-02	2.2E-01	4.0E-01
STAD	CLSTN1_ES_11_10_12	CLSTN1	ES	11	10	12	1.4E-19	0.214	-0.161	4.2E-05	4.9E-03	9.1E-04
STAD	CLSTN1_ES_3_2_4	CLSTN1	ES	3	2	4	1.5E-16	0.184	-0.180	5.9E-04	2.0E-03	3.9E-03
STAD	COL12A1_ES_3:4:5:6:7:8:9:10:11:12:13:[illegible]	COL12A1	ES	[illegible]:9:10:11:12:13:[illegible]	2	18	1.8E-19	0.213	-0.095	6.6E-01	3.9E-01	3.5E-01
STAD	COL6A3_ES_6_5_7	COL6A3	ES	6	5	7	9.4E-17	0.186	-0.137	9.1E-02	1.9E-01	8.2E-01
STAD	CPNE1_AD_1.2_1.1_5	CPNE1	AD	1.2	1.1	5	2.9E-11	0.127	0.099	8.1E-01	6.5E-01	9.7E-01
STAD	CPNE1_ES_2.1:2.2:3_1.2_5	CPNE1	ES	2.1:2.2:3	1.2	5	4.1E-11	0.126	-0.615	4.4E-01	8.9E-01	6.4E-01
STAD	CSDE1_ES_3:4_2_5	CSDE1	ES	3:04	2	5	1.8E-08	0.101	0.262	6.8E-01	4.0E-01	7.3E-01

cancer type	id	Gene Symbol	splice_type	Exon	From.Exon	To.Exon	anova.p	adj.r2	r	p.50	p.25	p.10
STAD	CSPP1_ES_7:8_6_9	CSPP1	ES	7:08	6	9	2.1E-12	0.163	-0.002	1.9E-01	3.4E-01	2.4E-01
STAD	CTNNB1_AA_18.3_18.1_18.4	CTNNB1	AA	18.3	18.1	18.4	8.8E-20	0.216	-0.009	5.6E-01	8.6E-02	3.9E-01
STAD	CTNNB1_RI_18.2:18.3_18.1_18.4	CTNNB1	RI	18.2:18.3	18.1	18.4	1.6E-16	0.183	-0.088	2.0E-01	3.1E-01	9.9E-01
STAD	CTNND1_ES_2.2:2.3:3:4.1:4.2:4.3:5_2.1_	CTNND1	ES	2:2.3:3:4.1:4.2:4.3	2.1	6	2.2E-10	0.121	-0.301	4.1E-01	4.1E-01	5.7E-01
STAD	CTNND1_ES_21_20_22.1	CTNND1	ES	21	20	22.1	4.4E-09	0.103	0.250	1.3E-01	7.2E-03	3.1E-02
STAD	CTNND1_ES_3:4.1:4.2:4.3_2.1_5	CTNND1	ES	3:4.1:4.2:4.3	2.1	5	2.8E-19	0.211	-0.414	7.1E-03	3.7E-02	5.6E-01
STAD	CTNND1_ES_3:4.1:4.2:4.3_2.1_6	CTNND1	ES	3:4.1:4.2:4.3	2.1	6	3.7E-17	0.230	-0.434	2.5E-01	2.9E-02	9.8E-01
STAD	CTNND1_ES_3:4.1:4.2:4.3_2.3_5	CTNND1	ES	3:4.1:4.2:4.3	2.3	5	2.2E-09	0.109	-0.328	2.1E-01	6.5E-01	7.4E-01
STAD	CTNND1_ES_3:4.1:4.2:4.3:5_2.1_6	CTNND1	ES	3:4.1:4.2:4.3:5	2.1	6	8.1E-15	0.166	-0.322	5.5E-02	5.1E-03	1.1E-01
STAD	CTNND1_ES_3:4.2:4.3:5_2.1_6	CTNND1	ES	3:4.2:4.3:5	2.1	6	8.4E-17	0.189	-0.340	2.8E-01	1.5E-02	4.5E-01
STAD	CTNND1_ES_4.1:4.2:4.3:5_2.1_6	CTNND1	ES	4.1:4.2:4.3:5	2.1	6	2.2E-09	0.107	-0.078	9.0E-02	1.6E-01	1.2E-02
STAD	CYB561A3_AA_6.6_6.4_6.7	CYB561A3	AA	6.6	6.4	6.7	3.7E-10	0.115	0.305	2.1E-02	2.7E-01	7.8E-01
STAD	CYB561A3_RI_6.5:6.6_6.4_6.7	CYB561A3	RI	6.5:6.6	6.4	6.7	4.2E-09	0.103	-0.014	7.1E-01	6.6E-01	8.2E-01
STAD	DAP3_AD_2.3_2.2_3	DAP3	AD	2.3	2.2	3	5.2E-09	0.102	0.170	1.1E-01	4.3E-01	8.9E-01
STAD	DCAF6_ES_11_10_13.1	DCAF6	ES	11	10	13.1	7.6E-10	0.114	0.049	4.8E-02	9.4E-02	6.1E-01
STAD	DCAF6_ES_11:13.1_10_14	DCAF6	ES	11:13.1	10	14	1.6E-09	0.108	0.097	1.4E-02	1.8E-02	5.0E-01
STAD	DENND1A_ES_23_21_24	DENND1A	ES	23	21	24	8.3E-09	0.103	-0.080	1.5E-01	9.6E-01	9.5E-01
STAD	DENND5B_ES_5_1_6	DENND5B	ES	5	1	6	2.7E-09	0.109	-0.006	9.7E-02	4.5E-01	4.9E-01
STAD	DGUOK_ES_5:6_4_7	DGUOK	ES	5:06	4	7	5.0E-11	0.125	-0.087	3.4E-01	2.4E-01	1.5E-01
STAD	DHX30_ES_6_5_10	DHX30	ES	6	5	10	3.9E-09	0.105	0.060	9.9E-01	7.7E-01	1.3E-01
STAD	DMD_ES_82_81_83	DMD	ES	82	81	83	1.7E-08	0.126	0.584	1.7E-01	6.1E-01	2.5E-01
STAD	DMWD_ES_4_3_5	DMWD	ES	4	3	5	8.3E-11	0.122	-0.082	3.8E-01	1.8E-01	5.4E-02
STAD	DNM1L_ES_17_16_18	DNM1L	ES	17	16	18	5.9E-11	0.125	-0.055	9.7E-02	2.4E-01	6.9E-01
STAD	DOCK6_ES_23_22_24	DOCK6	ES	23	22	24	2.8E-09	0.105	-0.130	8.1E-03	1.1E-03	9.0E-03
STAD	DST_ES_43:44:45:46.1:48_42_49	DST	ES	43:44:45:46.1:48	42	49	2.2E-13	0.152	-0.096	9.2E-01	6.8E-01	6.3E-01
STAD	DVL1_AD_11.2_11.1_12	DVL1	AD	11.2	11.1	12	9.2E-16	0.176	0.209	1.2E-03	2.8E-04	9.9E-03
STAD	DYRK1B_AA_10.1:10.2_9_10.3	DYRK1B	AA	10.1:10.2	9	10.3	7.2E-11	0.124	0.158	2.2E-01	8.4E-01	1.8E-01
STAD	DYSF_ES_19_18_20	DYSF	ES	19	18	20	6.3E-11	0.147	0.308	2.2E-01	3.5E-02	1.0E+00
STAD	DYSF_ES_7_6_8	DYSF	ES	7	6	8	3.4E-09	0.113	-0.290	1.3E-02	6.7E-02	5.1E-01
STAD	EBPL_ES_2_1_6	EBPL	ES	2	1	6	2.1E-10	0.120	0.153	9.3E-01	9.9E-01	2.2E-01
STAD	ECHDC2_ES_5.1:6.1_2.1_6.2	ECHDC2	ES	5.1:6.1	2.1	6.2	4.4E-10	0.117	-0.041	2.8E-01	3.2E-01	1.2E-01
STAD	ECT2_ES_2_1_3.2	ECT2	ES	2	1	3.2	1.1E-19	0.215	-0.352	3.7E-01	9.9E-02	3.2E-01
STAD	EHBP1_ES_18_17_19	EHBP1	ES	18	17	19	4.0E-09	0.103	-0.067	6.3E-02	1.0E-01	1.1E-01
STAD	EIF4H_ES_5_4_6	EIF4H	ES	5	4	6	1.2E-10	0.120	0.160	3.8E-01	5.2E-01	1.1E-01
STAD	ENAH_ES_13_12_14	ENAH	ES	13	12	14	1.4E-10	0.120	0.006	2.9E-01	4.7E-02	1.6E-02
STAD	ENTHD2_AD_1.2_1.1_2	ENTHD2	AD	1.2	1.1	2	1.3E-09	0.109	0.125	7.1E-03	3.8E-02	8.9E-01
STAD	EPB41_ES_16:18_15_19.1	EPB41	ES	16:18	15	19.1	9.4E-09	0.103	0.105	2.7E-01	4.1E-02	1.0E-01
STAD	EPB41L1_ES_20_19_21	EPB41L1	ES	20	19	21	3.5E-12	0.137	-0.155	3.6E-02	4.2E-02	2.5E-01
STAD	EPB41L2_ES_17:18_14_20.1	EPB41L2	ES	17:18	14	20.1	2.1E-09	0.107	-0.205	1.2E-02	4.4E-02	4.5E-01
STAD	ESRP1_ES_15_13_16	ESRP1	ES	15	13	16	3.6E-10	0.117	-0.119	4.5E-01	4.6E-01	6.8E-01

cancer type	id	Gene Symbol	splice_type	Exon	From.Exon	To.Exon	anova.p	adj.r2	r	p.50	p.25	p.10
STAD	EVI5_ES_12_11_13	EVI5	ES	12	11	13	2.0E-12	0.150	-0.080	3.3E-02	4.9E-01	9.7E-01
STAD	EVI5L_ES_12_11_13	EVI5L	ES	12	11	13	3.9E-14	0.171	0.115	2.5E-02	2.2E-03	2.6E-02
STAD	EXOC1_ES_11_10_12	EXOC1	ES	11	10	12	7.6E-17	0.187	0.258	6.6E-03	1.5E-02	1.5E-01
STAD	EXOC7_ES_8.2_6_9	EXOC7	ES	8.2	6	9	4.5E-15	0.168	-0.110	7.4E-02	2.1E-02	5.1E-02
STAD	FAM129C_AA_14.1_13_14.2	FAM129C	AA	14.1	13	14.2	6.0E-12	0.135	-0.753	4.7E-01	9.7E-01	3.6E-01
STAD	FAM168A_ES_4:5_3_6	FAM168A	ES	4:05	3	6	1.2E-08	0.122	0.388	4.3E-02	7.9E-02	8.6E-01
STAD	FBLN2_ES_11_10_12	FBLN2	ES	11	10	12	1.3E-11	0.137	0.390	4.5E-02	2.1E-01	1.1E-01
STAD	FIP1L1_ES_11_10_12	FIP1L1	ES	11	10	12	1.3E-13	0.153	-0.163	4.6E-01	6.3E-01	8.4E-01
STAD	FLAD1_ES_2.2:4.1:4.2:4.3_1.3_6.1	FLAD1	ES	2.2:4.1:4.2:4.3	1.3	6.1	2.6E-08	0.115	0.066	6.2E-01	5.3E-01	1.8E-01
STAD	FLNA_ES_30_29_31	FLNA	ES	30	29	31	3.8E-09	0.104	-0.626	8.1E-03	6.6E-02	9.1E-01
STAD	FMNL3_ES_6_5_7	FMNL3	ES	6	5	7	2.1E-09	0.108	-0.340	1.0E-01	3.0E-01	3.2E-01
STAD	FN1_ES_40.2_39_40.4	FN1	ES	40.2	39	40.4	2.1E-11	0.129	0.025	5.5E-02	9.1E-01	3.0E-01
STAD	FNBP1_ES_10.1:10.2:10.3_9_14.2	FNBP1	ES	10.1:10.2:10.3	9	14.2	3.7E-09	0.116	-0.005	1.4E-02	3.9E-02	2.0E-01
STAD	FNBP1_ES_10.2:10.3_9_14.2	FNBP1	ES	10.2:10.3	9	14.2	3.0E-09	0.120	0.224	1.4E-01	9.0E-02	5.1E-02
STAD	FNIP1_ES_7_6_8	FNIP1	ES	7	6	8	2.3E-11	0.132	-0.002	1.1E-02	4.3E-02	3.8E-02
STAD	FYN_ME_11|12_10_13	FYN	ME	11|12	10	13	3.4E-17	0.190	0.045	8.3E-02	4.7E-02	1.8E-01
STAD	GAK_ES_3:4:5.1:5.2_2_6	GAK	ES	3:4:5.1:5.2	2	6	4.3E-09	0.113	0.148	8.8E-01	4.7E-01	9.3E-01
STAD	GEMIN2_ES_7_6_8	GEMIN2	ES	7	6	8	5.3E-15	0.169	0.006	3.8E-01	2.2E-02	3.0E-01
STAD	GIT2_ES_19_18.2_20	GIT2	ES	19	18.2	20	1.6E-11	0.131	0.423	7.6E-03	1.1E-02	5.7E-02
STAD	GK_ES_12_11_13	GK	ES	12	11	13	1.1E-08	0.105	-0.326	1.2E-01	1.0E-01	1.1E-01
STAD	GOLGA4_ES_24_23_25	GOLGA4	ES	24	23	25	1.2E-13	0.153	0.316	9.8E-02	2.9E-02	6.0E-02
STAD	GPR107_ES_15_14_17	GPR107	ES	15	14	17	1.4E-12	0.143	0.062	8.3E-02	2.1E-02	9.0E-02
STAD	H2AFY_ME_7|8_6.3_9	H2AFY	ME	7|8	6.3	9	4.8E-09	0.102	0.458	6.8E-04	3.5E-03	2.7E-01
STAD	HNRNPA2B1_ES_2:3:4:5:6_1_10	HNRNPA2B1	ES	2:3:4:5:6	1	10	3.7E-08	0.110	0.094	9.5E-01	4.8E-01	5.8E-02
STAD	HTATIP2_AD_1.2_1.1_1.5	HTATIP2	AD	1.2	1.1	1.5	2.1E-09	0.109	0.131	6.4E-01	9.3E-01	4.3E-01
STAD	IL32_AA_1.3:1.4:1.5:1.6:1.7_1.1_1.8	IL32	AA	1.3:1.4:1.5:1.6:1.[illegible]	1.1	1.8	3.9E-13	0.150	0.299	1.6E-01	2.7E-01	4.5E-01
STAD	IL32_AD_1.2:1.3:1.4:1.5_1.1_1.8	IL32	AD	1.2:1.3:1.4:1.5	1.1	1.8	6.1E-15	0.168	0.478	5.8E-01	5.5E-01	4.0E-01
STAD	IL32_AD_1.2:1.3:1.4:1.5_1.1_1.9	IL32	AD	1.2:1.3:1.4:1.5	1.1	1.9	7.9E-18	0.197	0.503	2.5E-02	2.7E-02	8.5E-01
STAD	IL32_ES_1.3:1.4:1.5_1.1_1.9	IL32	ES	1.3:1.4:1.5	1.1	1.9	3.3E-17	0.191	0.364	1.6E-01	1.9E-02	4.0E-01
STAD	IL32_ES_1.3:1.4:1.5:1.8:1.9_1.1_2.1	IL32	ES	1.3:1.4:1.5:1.8:1.[illegible]	1.1	2.1	7.8E-09	0.123	0.338	1.9E-01	6.1E-02	4.9E-02
STAD	ILK_AA_1.3_1.1_1.4	ILK	AA	1.3	1.1	1.4	1.8E-10	0.119	-0.266	4.5E-01	2.1E-01	2.7E-01
STAD	INPP4A_AD_18.2_18.1_19	INPP4A	AD	18.2	18.1	19	1.7E-13	0.153	-0.295	1.7E-01	2.6E-01	5.4E-02
STAD	INSR_ES_11_10_12	INSR	ES	11	10	12	2.2E-14	0.163	0.021	2.1E-01	4.4E-02	3.1E-02
STAD	ITGAE_ES_28_27_29	ITGAE	ES	28	27	29	3.1E-17	0.191	0.540	4.3E-01	6.0E-01	6.6E-01
STAD	ITGB4_ES_35_34_36	ITGB4	ES	35	34	36	3.0E-19	0.211	-0.644	1.3E-04	1.9E-03	1.7E-02
STAD	KANK1_ES_12_11_13	KANK1	ES	12	11	13	2.0E-07	0.108	-0.077	4.3E-01	5.1E-01	4.4E-01
STAD	KIAA0226_ES_14_13_15	KIAA0226	ES	14	13	15	2.1E-18	0.209	0.148	6.9E-01	4.5E-01	5.3E-01
STAD	KIAA1432_ES_14_13_15	KIAA1432	ES	14	13	15	5.2E-12	0.138	0.029	5.2E-01	4.6E-01	3.8E-01
STAD	KIAA1468_ME_24|25_23_26	KIAA1468	ME	24|25	23	26	4.1E-09	0.103	-0.329	3.0E-02	4.2E-02	6.5E-01
STAD	KIF12_ES_5_4.1_6	KIF12	ES	5	4.1	6	1.8E-11	0.138	0.507	3.9E-01	2.8E-01	1.8E-01

cancer type	id	Gene Symbol	splice_type	Exon	From.Exon	To.Exon	anova.p	adj.r2	r	p.50	p.25	p.10
STAD	KIF13A_ES_40_39_41.1	KIF13A	ES	40	39	41.1	4.3E-17	0.190	-0.069	1.1E-02	1.1E-01	3.6E-02
STAD	KIF21A_ES_24_23_25.2	KIF21A	ES	24	23	25.2	1.2E-12	0.144	-0.296	3.2E-01	3.1E-01	3.2E-01
STAD	KIF21A_ES_29:30:32_28_33.2	KIF21A	ES	29:30:32	28	33.2	1.8E-10	0.147	-0.213	NA	NA	NA
STAD	KIFC2_RI_16.5_16.4_16.6	KIFC2	RI	16.5	16.4	16.6	5.8E-11	0.126	0.060	2.3E-01	4.4E-01	2.4E-01
STAD	KTN1_ES_35_34.2_36	KTN1	ES	35	34.2	36	5.2E-09	0.102	0.073	1.3E-02	4.4E-01	3.1E-01
STAD	LAS1L_AD_11.2_11.1_12	LAS1L	AD	11.2	11.1	12	2.2E-12	0.140	-0.474	1.6E-01	3.6E-01	5.4E-01
STAD	LAS1L_ES_9_8_10	LAS1L	ES	9	8	10	7.2E-21	0.228	-0.323	3.4E-02	2.3E-02	1.8E-01
STAD	LPHN2_ES_15_14_16	LPHN2	ES	15	14	16	2.2E-10	0.127	0.230	1.0E-01	3.9E-01	6.0E-01
STAD	LRP8_ES_20_19_21	LRP8	ES	20	19	21	9.7E-09	0.113	-0.157	1.4E-02	5.7E-03	9.6E-01
STAD	LRR1_ES_4_3_5	LRR1	ES	4	3	5	4.8E-16	0.183	-0.069	9.2E-01	3.3E-01	6.6E-01
STAD	LRR1_ES_4:5_3_7	LRR1	ES	4:05	3	7	2.0E-12	0.141	-0.175	4.8E-01	8.1E-01	9.8E-01
STAD	LRRFIP1_ES_15_14_16	LRRFIP1	ES	15	14	16	2.6E-15	0.171	0.111	1.9E-01	4.4E-02	7.7E-02
STAD	LRRFIP1_ES_4:5_3_6	LRRFIP1	ES	4:05	3	6	2.2E-09	0.115	0.058	1.9E-02	1.2E-01	3.5E-01
STAD	LRRFIP2_ES_20_19_21	LRRFIP2	ES	20	19	21	4.6E-13	0.147	-0.162	1.1E-03	2.5E-03	1.8E-01
STAD	LTBP3_ES_25_24_26	LTBP3	ES	25	24	26	2.5E-13	0.150	0.485	2.2E-01	4.3E-01	5.8E-01
STAD	LTBP4_ES_29_28_31	LTBP4	ES	29	28	31	7.5E-09	0.104	-0.218	2.6E-01	3.0E-01	1.6E-01
STAD	MAGI1_ES_7_6_8	MAGI1	ES	7	6	8	3.3E-14	0.166	-0.174	4.6E-01	3.5E-01	4.6E-01
STAD	MAP2K7_ES_2_1_3	MAP2K7	ES	2	1	3	3.5E-14	0.172	0.035	4.2E-03	1.5E-02	1.1E-02
STAD	MAP3K7_ES_11_10_12	MAP3K7	ES	11	10	12	5.6E-19	0.208	0.033	2.5E-02	5.7E-03	2.2E-04
STAD	MARCH8_ES_7_6_8	MARCH8	ES	7	6	8	5.3E-11	0.125	-0.281	5.2E-02	8.3E-03	6.2E-01
STAD	MARK3_ES_17_16_18	MARK3	ES	17	16	18	3.6E-11	0.140	-0.252	4.3E-01	1.6E-01	2.5E-01
STAD	MARK3_ES_17_16_19	MARK3	ES	17	16	19	2.3E-13	0.150	-0.187	1.7E-01	1.4E-01	4.6E-01
STAD	MARK3_ES_17:18_16_19	MARK3	ES	17:18	16	19	1.2E-17	0.195	-0.219	2.7E-02	1.2E-01	2.5E-01
STAD	MBNL1_ES_10:11_9_13	MBNL1	ES	10:11	9	13	2.6E-09	0.105	-0.459	7.3E-02	1.6E-01	2.8E-01
STAD	MBNL1_ES_11_9_13	MBNL1	ES	11	9	13	8.8E-13	0.144	-0.536	1.1E-01	2.8E-01	4.6E-01
STAD	MBNL1_ES_8_7_9	MBNL1	ES	8	7	9	1.4E-17	0.194	-0.652	9.8E-03	3.4E-01	3.4E-01
STAD	MBNL2_ES_10_8_11	MBNL2	ES	10	8	11	2.5E-11	0.129	-0.131	9.5E-01	8.9E-01	2.1E-01
STAD	MBNL2_ES_7_6.3_8	MBNL2	ES	7	6.3	8	7.6E-11	0.123	-0.190	1.3E-02	6.7E-02	8.3E-01
STAD	MDM4_ES_8_5_9	MDM4	ES	8	5	9	2.1E-27	0.290	0.068	6.7E-01	4.8E-01	6.8E-01
STAD	METTL8_ES_2_1_3	METTL8	ES	2	1	3	3.0E-33	0.378	-0.012	2.1E-01	7.0E-03	1.0E-01
STAD	MFF_ES_8:9:10_7_11	MFF	ES	8:09:10	7	11	4.4E-09	0.103	-0.090	8.0E-01	5.0E-01	1.0E+00
STAD	MICAL3_ES_19:20:21:22_17_23	MICAL3	ES	19:20:21:22	17	23	8.9E-11	0.130	0.387	2.6E-01	4.9E-01	2.2E-01
STAD	MICAL3_ES_29_28_30	MICAL3	ES	29	28	30	6.5E-10	0.123	0.349	1.3E-02	1.4E-01	3.3E-01
STAD	MKI67_ES_7_6_8	MKI67	ES	7	6	8	6.0E-12	0.135	-0.228	1.4E-01	2.5E-01	3.6E-01
STAD	MORF4L2_ES_4:5.1:5.2_3.2_5.3	MORF4L2	ES	4:5.1:5.2	3.2	5.3	1.9E-10	0.118	0.118	1.7E-01	1.1E-01	5.0E-01
STAD	MPDU1_AD_5.3_5.2_7	MPDU1	AD	5.3	5.2	7	9.6E-10	0.116	-0.128	4.8E-01	7.6E-01	3.9E-01
STAD	MPRIP_ES_24_23_25	MPRIP	ES	24	23	25	2.8E-16	0.181	0.205	3.5E-03	6.9E-03	1.3E-02
STAD	MRPL33_ES_3_2_4	MRPL33	ES	3	2	4	2.0E-12	0.140	0.003	6.9E-01	5.3E-01	4.0E-02
STAD	MRPL52_ES_3:4.1_2_5	MRPL52	ES	03:04.1	2	5	1.1E-11	0.132	-0.076	8.1E-01	5.3E-01	1.6E-01
STAD	MRRF_ES_5_4_6	MRRF	ES	5	4	6	3.2E-10	0.118	-0.057	9.7E-01	9.3E-01	7.1E-01

cancer type	id	Gene Symbol	splice_type	Exon	From.Exon	To.Exon	anova.p	adj.r2	r	p.50	p.25	p.10
STAD	MSI2_ES_19_18.2_20	MSI2	ES	19	18.2	20	1.2E-07	0.106	0.175	7.9E-01	5.1E-01	5.9E-03
STAD	MST1_RI_8.2_8.1_8.3	MST1	RI	8.2	8.1	8.3	5.5E-11	0.142	-0.359	1.1E-01	2.8E-01	9.3E-01
STAD	MTMR1_ES_4_3_5	MTMR1	ES	4	3	5	9.6E-15	0.169	-0.185	4.2E-02	2.3E-01	7.8E-01
STAD	MTMR3_ES_20_19_21	MTMR3	ES	20	19	21	2.4E-16	0.183	0.110	2.8E-02	3.9E-02	2.5E-02
STAD	MTSS1L_ES_12_11_13	MTSS1L	ES	12	11	13	5.6E-09	0.121	-0.144	2.5E-01	8.0E-02	1.7E-01
STAD	MUM1_ES_5.1:5.2_4_6	MUM1	ES	5.1:5.2	4	6	7.8E-09	0.100	-0.157	3.6E-01	7.6E-01	5.6E-01
STAD	MYL12A_ES_1.2:2_1.1_4	MYL12A	ES	1.2:2	1.1	4	3.1E-11	0.127	0.191	9.1E-01	2.1E-01	7.2E-01
STAD	MYO18A_ES_41_40_42	MYO18A	ES	41	40	42	2.9E-17	0.191	-0.259	1.9E-02	2.2E-03	1.4E-02
STAD	MYOF_ES_17_16_18	MYOF	ES	17	16	18	2.7E-10	0.118	-0.258	1.1E-01	9.8E-05	2.3E-02
STAD	NAA60_RI_10.4:10.5_10.3_10.6	NAA60	RI	10.4:10.5	10.3	10.6	3.2E-13	0.149	-0.150	7.4E-01	9.2E-01	8.7E-01
STAD	NAA60_RI_10.5_10.4_10.6	NAA60	RI	10.5	10.4	10.6	3.9E-10	0.115	-0.156	4.2E-01	7.5E-01	4.8E-01
STAD	NASP_ES_9_8_10	NASP	ES	9	8	10	1.2E-09	0.109	0.181	2.1E-01	2.6E-01	1.2E-01
STAD	NAV2_ES_23:24_22_25	NAV2	ES	23:24	22	25	4.7E-08	0.104	0.281	5.6E-02	7.8E-01	6.6E-01
STAD	NCOR2_AD_46.2_46.1_47	NCOR2	AD	46.2	46.1	47	2.7E-24	0.259	-0.121	6.0E-02	2.5E-02	3.1E-01
STAD	NCOR2_ES_46.1:46.2_45_47	NCOR2	ES	46.1:46.2	45	47	3.0E-23	0.259	-0.153	3.6E-02	1.5E-02	5.5E-01
STAD	NDEL1_ES_11_10_12.1	NDEL1	ES	11	10	12.1	7.1E-11	0.123	0.079	2.6E-04	3.2E-04	3.6E-01
STAD	NEDD1_ES_3:4.2_2.3_5	NEDD1	ES	03:04.2	2.3	5	6.0E-10	0.113	0.105	3.7E-01	3.2E-01	3.5E-01
STAD	NFIB_ES_12.1:12.2:13:14_11_15	NFIB	ES	12.1:12.2:13:14	11	15	2.8E-13	0.151	-0.075	5.6E-01	8.1E-01	3.9E-01
STAD	NFIB_ES_12.1:13:14_11_15	NFIB	ES	12.1:13:14	11	15	2.8E-12	0.141	-0.085	9.5E-01	6.9E-01	8.2E-01
STAD	NFRKB_ES_2_1_3.2	NFRKB	ES	2	1	3.2	3.2E-09	0.111	0.085	6.7E-01	6.3E-01	4.6E-01
STAD	NFYA_ES_3_2_4	NFYA	ES	3	2	4	1.9E-23	0.252	-0.432	5.4E-03	7.9E-03	4.4E-02
STAD	NIN_ES_18_17_19	NIN	ES	18	17	19	1.9E-09	0.107	-0.110	2.5E-02	1.4E-02	1.4E-01
STAD	NPRL3_ES_2:3_1.4_4	NPRL3	ES	2:03	1.4	4	4.2E-20	0.225	-0.115	8.5E-01	3.0E-01	2.6E-01
STAD	NPRL3_ES_3_2_4	NPRL3	ES	3	2	4	6.3E-14	0.156	-0.215	8.8E-01	4.4E-01	2.0E-01
STAD	NPRL3_ES_3:4:5_2_6	NPRL3	ES	3:04:05	2	6	9.8E-09	0.100	-0.066	7.0E-01	2.5E-01	4.2E-01
STAD	NSFL1C_ES_5.2_4_7.2	NSFL1C	ES	5.2	4	7.2	4.2E-21	0.229	-0.270	1.1E-02	7.7E-03	3.3E-02
STAD	NUMB_ES_13_12_14	NUMB	ES	13	12	14	4.3E-14	0.158	0.233	7.1E-01	4.3E-01	7.3E-03
STAD	NUP153_ES_8_7_9	NUP153	ES	8	7	9	2.7E-35	0.387	0.174	8.8E-01	9.4E-02	1.6E-01
STAD	NVL_ES_12_10_13	NVL	ES	12	10	13	1.9E-09	0.109	-0.034	6.5E-01	5.2E-01	9.0E-01
STAD	OGG1_RI_6.2:6.3_6.1_6.4	OGG1	RI	6.2:6.3	6.1	6.4	1.6E-10	0.119	0.052	6.3E-01	4.9E-01	9.6E-01
STAD	OPA1_ES_7_6_8	OPA1	ES	7	6	8	2.8E-09	0.105	-0.010	1.4E-01	2.9E-02	5.9E-01
STAD	OSBPL8_ES_6_5_7.1	OSBPL8	ES	6	5	7.1	2.1E-10	0.120	-0.036	4.2E-02	3.2E-03	4.2E-02
STAD	OSBPL8_ES_6:7.1_5_7.2	OSBPL8	ES	06:07.1	5	7.2	7.4E-09	0.103	-0.059	2.2E-01	2.7E-01	1.2E-02
STAD	OSBPL9_ES_17_15_18	OSBPL9	ES	17	15	18	1.1E-09	0.110	-0.104	5.6E-01	6.9E-01	8.7E-01
STAD	OSR2_ES_2_1_3	OSR2	ES	2	1	3	2.5E-09	0.115	-0.121	2.2E-01	7.6E-02	6.7E-01
STAD	PACRGL_ES_11_9_12	PACRGL	ES	11	9	12	1.3E-08	0.102	-0.190	3.4E-02	3.3E-03	3.0E-02
STAD	PACSIN2_ES_12_11_13	PACSIN2	ES	12	11	13	4.0E-09	0.103	0.116	5.2E-02	3.2E-03	3.9E-02
STAD	PAIP1_AD_2.2_2.1_3	PAIP1	AD	2.2	2.1	3	1.0E-14	0.165	0.051	7.0E-01	4.6E-01	3.8E-01
STAD	PAM_ES_14_13_15	PAM	ES	14	13	15	1.8E-09	0.107	0.327	2.9E-01	1.7E-01	4.0E-01
STAD	PAPOLA_ES_20_18_21	PAPOLA	ES	20	18	21	1.4E-12	0.142	-0.035	2.6E-01	6.4E-01	6.0E-01

cancer type	id	Gene Symbol	splice_type	Exon	From.Exon	To.Exon	anova.p	adj.r2	r	p.50	p.25	p.10
STAD	PARD3_AD_19.2:19.3_19.1_20	PARD3	AD	19.2:19.3	19.1	20	6.6E-09	0.101	0.035	8.8E-01	6.4E-01	7.9E-01
STAD	PBRM1_ES_28:29_27_30	PBRM1	ES	28:29:00	27	30	1.5E-11	0.131	0.006	2.6E-03	1.2E-01	1.8E-01
STAD	PBRM1_ES_29_27_30	PBRM1	ES	29	27	30	1.4E-12	0.142	0.030	9.9E-02	2.4E-02	1.7E-02
STAD	PBRM1_ES_29_28_30	PBRM1	ES	29	28	30	8.1E-14	0.159	-0.051	2.5E-03	9.5E-02	2.3E-01
STAD	PDLIM5_AA_8.1_6.1_8.2	PDLIM5	AA	8.1	6.1	8.2	4.4E-17	0.189	-0.217	1.4E-02	1.6E-02	3.7E-01
STAD	PDPR_AA_13.1_12_13.2	PDPR	AA	13.1	12	13.2	1.3E-08	0.100	-0.147	6.3E-02	2.1E-01	6.5E-01
STAD	PFN2_AA_6.1_5.4_6.2	PFN2	AA	6.1	5.4	6.2	5.5E-12	0.135	0.493	9.0E-01	5.6E-01	9.6E-01
STAD	PHKB_ES_2_1_3	PHKB	ES	2	1	3	1.1E-09	0.110	0.123	3.8E-01	7.3E-02	3.4E-01
STAD	PITPNM2_ES_21_20_22	PITPNM2	ES	21	20	22	2.3E-12	0.152	-0.035	2.2E-01	5.3E-01	4.6E-01
STAD	PLEKHA1_ES_15.1_14_16	PLEKHA1	ES	15.1	14	16	1.2E-17	0.195	-0.279	1.8E-02	2.9E-03	6.3E-02
STAD	PLEKHA6_AA_7.1_6_7.2	PLEKHA6	AA	7.1	6	7.2	1.2E-13	0.155	0.275	7.9E-01	4.1E-02	9.5E-01
STAD	PLEKHG3_AD_15.2_15.1_16	PLEKHG3	AD	15.2	15.1	16	1.2E-08	0.108	-0.295	2.5E-01	9.7E-02	3.7E-02
STAD	PLEKHM2_ES_7_6_8	PLEKHM2	ES	7	6	8	2.9E-11	0.127	0.146	4.9E-02	1.4E-01	2.9E-01
STAD	PLOD2_ES_15_14_16	PLOD2	ES	15	14	16	1.0E-13	0.155	-0.256	5.1E-01	3.3E-01	3.9E-01
STAD	POLD4_AD_1.2:1.3_1.1_2	POLD4	AD	1.2:1.3	1.1	2	2.6E-14	0.161	0.066	5.3E-02	7.5E-01	9.9E-01
STAD	POR_RI_16.3_16.2_16.4	POR	RI	16.3	16.2	16.4	8.6E-17	0.209	-0.051	7.3E-01	4.9E-01	2.7E-01
STAD	PPFIBP1_ES_11_10_12	PPFIBP1	ES	11	10	12	3.0E-17	0.194	-0.022	9.9E-04	1.2E-01	9.6E-01
STAD	PPIA_ES_3:4:5.1:6_1_7	PPIA	ES	3:4:5.1:6	1	7	1.7E-14	0.201	0.080	2.1E-01	4.8E-01	7.4E-01
STAD	PPIP5K2_ES_26_25_27	PPIP5K2	ES	26	25	27	2.3E-11	0.128	-0.236	8.2E-02	2.9E-02	1.0E-02
STAD	PRKCSH_AA_13.1_12.2_13.2	PRKCSH	AA	13.1	12.2	13.2	9.4E-16	0.176	0.000	5.1E-01	6.4E-01	6.4E-01
STAD	PSIP1_AA_11.1_10_11.2	PSIP1	AA	11.1	10	11.2	4.2E-16	0.180	0.015	3.1E-01	2.0E-02	1.0E-01
STAD	PSMD3_ES_10:11_9_12	PSMD3	ES	10:11	9	12	3.8E-09	0.122	-0.344	2.7E-02	1.3E-02	1.1E-01
STAD	PTK2B_ES_28_27_29	PTK2B	ES	28	27	29	4.4E-09	0.103	-0.063	7.3E-01	3.6E-01	1.3E-01
STAD	PTS_ES_4_3_5	PTS	ES	4	3	5	9.2E-16	0.176	0.342	8.7E-02	1.3E-01	6.1E-02
STAD	PUM2_ES_16_15_17	PUM2	ES	16	15	17	1.0E-14	0.165	-0.046	5.5E-01	8.0E-01	9.4E-01
STAD	RAC1_ES_4_3_5	RAC1	ES	4	3	5	1.3E-10	0.120	-0.004	6.9E-01	7.1E-01	6.0E-01
STAD	RAI14_ES_18_17_19	RAI14	ES	18	17	19	1.1E-11	0.132	0.004	6.2E-01	3.2E-01	3.0E-01
STAD	RALGAPA1_ES_42_41_43	RALGAPA1	ES	42	41	43	4.6E-13	0.147	-0.078	2.4E-02	2.3E-02	7.5E-02
STAD	RALGPS2_ES_15_14_16	RALGPS2	ES	15	14	16	3.1E-14	0.161	0.062	7.2E-01	3.0E-01	5.3E-01
STAD	RASAL2_AA_14.1_13_14.2	RASAL2	AA	14.1	13	14.2	3.9E-09	0.107	0.182	6.7E-01	6.6E-01	5.6E-01
STAD	RASSF7_AA_6.3:6.4_6.1_6.5	RASSF7	AA	6.3:6.4	6.1	6.5	5.9E-09	0.103	0.299	2.2E-01	1.3E-01	9.9E-01
STAD	RASSF7_RI_6.2:6.3:6.4_6.1_6.5	RASSF7	RI	6.2:6.3:6.4	6.1	6.5	2.8E-25	0.268	0.267	4.8E-01	2.7E-01	1.1E-01
STAD	RBM26_ES_14_13.2_15	RBM26	ES	14	13.2	15	1.1E-19	0.215	-0.130	1.2E-02	8.5E-02	4.1E-01
STAD	REPIN1_ES_3.2:4.2:5.2_2.1_5.3	REPIN1	ES	3.2:4.2:5.2	2.1	5.3	5.8E-08	0.103	0.058	3.4E-01	3.6E-01	1.6E-01
STAD	RFC5_ES_2.1:2.2_1_3	RFC5	ES	2.1:2.2	1	3	6.3E-15	0.167	-0.194	7.4E-01	8.3E-01	1.9E-01
STAD	RFWD2_ES_9_8_10	RFWD2	ES	9	8	10	3.8E-20	0.220	-0.251	8.1E-01	4.6E-01	4.3E-01
STAD	RHNO1_ES_2.1:2.2:3_1_4	RHNO1	ES	2.1:2.2:3	1	4	2.2E-09	0.127	-0.094	9.6E-01	5.0E-01	1.1E-01
STAD	RHOT1_ES_19.3:20_19.1_22	RHOT1	ES	19.3:20	19.1	22	3.9E-12	0.139	-0.166	1.1E-04	8.1E-03	4.4E-01
STAD	RPGR_ES_14.1:14.3_13_16	RPGR	ES	14.1:14.3	13	16	2.8E-09	0.107	-0.020	7.5E-02	1.8E-01	8.7E-01
STAD	RPL15_AD_1.3_1.2_3.2	RPL15	AD	1.3	1.2	3.2	4.5E-09	0.103	-0.183	2.8E-01	9.8E-01	5.3E-01

cancer type	id	Gene Symbol	splice_type	Exon	From.Exon	To.Exon	anova.p	adj.r2	r	p.50	p.25	p.10
STAD	RPL18A_ES_2.2:2.4_1_3	RPL18A	ES	2.2:2.4	1	3	2.1E-09	0.111	-0.073	2.9E-01	3.9E-01	4.6E-01
STAD	RPL22L1_AA_3.1_2.2_3.2	RPL22L1	AA	3.1	2.2	3.2	2.9E-44	0.422	-0.668	8.0E-01	7.6E-01	5.3E-01
STAD	RPL32_RI_1.2_1.1_1.3	RPL32	RI	1.2	1.1	1.3	6.0E-10	0.113	-0.295	7.6E-01	4.7E-01	2.8E-01
STAD	RPL6_ES_1.2:2:3:4_1.1_5	RPL6	ES	1.2:2:3:4	1.1	5	7.3E-09	0.101	-0.055	6.5E-01	5.5E-01	8.4E-01
STAD	RPLP0_ES_5.3_4.2_6.2	RPLP0	ES	5.3	4.2	6.2	1.4E-10	0.121	0.098	1.4E-01	1.1E-01	6.6E-02
STAD	RPP38_ES_2_1.2_3.1	RPP38	ES	2	1.2	3.1	1.4E-08	0.105	0.047	3.7E-01	6.3E-01	3.5E-01
STAD	RPS15A_ES_3.3_2.2_5.1	RPS15A	ES	3.3	2.2	5.1	4.4E-08	0.107	0.083	6.9E-01	9.3E-01	2.3E-01
STAD	RPS21_AA_3.3:3.4_3.1_3.5	RPS21	AA	3.3:3.4	3.1	3.5	3.7E-09	0.104	-0.078	4.5E-02	8.0E-03	5.7E-02
STAD	RPS24_AA_5.1_4_5.2	RPS24	AA	5.1	4	5.2	1.6E-10	0.119	0.151	2.2E-02	1.3E-03	5.3E-02
STAD	RPS24_ES_5.2_4_6	RPS24	ES	5.2	4	6	4.8E-11	0.125	-0.137	7.0E-01	5.7E-01	2.9E-01
STAD	RTN2_ES_5_4.1_6	RTN2	ES	5	4.1	6	1.3E-11	0.133	-0.120	2.3E-01	2.6E-01	7.1E-01
STAD	S100A1_AA_4.1_3_4.2	S100A1	AA	4.1	3	4.2	7.5E-14	0.160	-0.651	3.5E-01	4.9E-01	4.4E-01
STAD	SBF1_ES_29_28_30	SBF1	ES	29	28	30	4.3E-13	0.147	-0.114	7.4E-03	7.3E-03	7.9E-03
STAD	SCOC_ES_4_3.2_6	SCOC	ES	4	3.2	6	1.3E-08	0.101	0.180	5.2E-01	2.2E-01	7.0E-02
STAD	SDCCAG3_ES_2_1_4	SDCCAG3	ES	2	1	4	2.2E-10	0.118	0.347	6.6E-01	5.5E-01	8.4E-01
STAD	SEC16A_ES_24:25_23.12_26	SEC16A	ES	24:25:00	23.12	26	8.7E-10	0.112	-0.218	6.1E-03	1.6E-01	1.6E-01
STAD	SEC31A_ES_26.1:26.2_25.1_28	SEC31A	ES	26.1:26.2	25.1	28	1.6E-10	0.119	0.028	6.5E-02	8.2E-02	8.8E-04
STAD	SENP1_AD_2.2_2.1_3	SENP1	AD	2.2	2.1	3	5.2E-09	0.109	-0.099	5.4E-03	2.9E-03	4.7E-01
STAD	SEPSECS_ES_2:3_1_4	SEPSECS	ES	2:03	1	4	5.0E-19	0.226	-0.135	1.1E-01	4.1E-02	5.5E-01
STAD	SEPT6_ES_12_11.1_13.2	SEPT6	ES	12	11.1	13.2	3.4E-10	0.129	-0.107	6.9E-03	6.6E-02	7.5E-01
STAD	SEPT6_ES_12:13.1_11.1_13.2	SEPT6	ES	12:13.1	11.1	13.2	2.3E-10	0.117	-0.267	8.1E-05	7.5E-02	4.0E-01
STAD	SERGEF_AD_1.2_1.1_3	SERGEF	AD	1.2	1.1	3	7.8E-08	0.108	0.021	4.0E-01	4.8E-02	9.7E-02
STAD	SERPINA1_AA_2.1:2.2:2.3_1.1_2.4	SERPINA1	AA	2.1:2.2:2.3	1.1	2.4	5.0E-10	0.120	0.557	7.3E-01	5.4E-01	5.5E-01
STAD	SERPINA1_RI_2.2:2.3_2.1_2.4	SERPINA1	RI	2.2:2.3	2.1	2.4	1.2E-10	0.128	0.441	2.1E-01	2.0E-01	9.7E-01
STAD	SERPINA1_RI_2.3_2.2_2.4	SERPINA1	RI	2.3	2.2	2.4	3.8E-09	0.110	0.422	3.3E-02	1.0E-01	6.6E-01
STAD	SETD4_ES_4_3.4_5	SETD4	ES	4	3.4	5	8.1E-13	0.147	-0.002	2.9E-01	6.3E-01	8.2E-01
STAD	SH2D4A_ES_6_5_7	SH2D4A	ES	6	5	7	6.9E-09	0.101	0.307	5.4E-01	7.3E-01	6.6E-01
STAD	SLAIN2_ES_8_6_9	SLAIN2	ES	8	6	9	3.3E-12	0.138	0.338	1.8E-01	9.5E-02	2.5E-01
STAD	SLC25A16_ES_2.1:2.2_1_3	SLC25A16	ES	2.1:2.2	1	3	2.5E-10	0.120	0.244	2.5E-01	6.1E-01	8.2E-01
STAD	SLK_ES_13_12_14	SLK	ES	13	12	14	1.5E-20	0.224	0.280	1.6E-01	2.7E-02	8.7E-02
STAD	SLMAP_ES_25_24_26	SLMAP	ES	25	24	26	3.4E-10	0.115	0.265	1.6E-01	3.2E-02	7.7E-04
STAD	SMAGP_RI_3.2_3.1_3.3	SMAGP	RI	3.2	3.1	3.3	1.2E-09	0.109	-0.201	8.9E-01	5.0E-01	2.9E-01
STAD	SMARCB1_AD_2.2_2.1_3	SMARCB1	AD	2.2	2.1	3	1.9E-12	0.140	-0.113	1.0E-01	7.1E-01	2.8E-01
STAD	SMARCC2_AD_28.2:28.3_28.1_29	SMARCC2	AD	28.2:28.3	28.1	29	1.6E-22	0.243	0.046	1.9E-04	8.5E-03	4.0E-02
STAD	SMARCC2_ES_28.3_28.1_29	SMARCC2	ES	28.3	28.1	29	1.2E-11	0.143	0.204	6.6E-01	3.7E-03	3.3E-03
STAD	SMG7_ES_2:3_1_4	SMG7	ES	2:03	1	4	9.4E-18	0.198	-0.099	4.9E-01	2.9E-01	1.6E-01
STAD	SMTN_ES_21.1:21.2_20_22	SMTN	ES	21.1:21.2	20	22	6.9E-09	0.101	-0.755	2.1E-01	1.4E-01	4.9E-01
STAD	SNX14_ES_16_15_17	SNX14	ES	16	15	17	5.6E-09	0.102	-0.210	4.7E-01	1.8E-01	2.6E-01
STAD	SNX27_AA_12.1_11_12.2	SNX27	AA	12.1	11	12.2	9.7E-28	0.290	-0.009	2.5E-01	6.8E-01	7.1E-01
STAD	SOGA2_ES_13_11_14	SOGA2	ES	13	11	14	1.5E-10	0.149	0.226	1.5E-01	7.3E-02	6.7E-01

cancer type	id	Gene Symbol	splice_type	Exon	From.Exon	To.Exon	anova.p	adj.r2	r	p.50	p.25	p.10
STAD	SPAG9_ES_30_29_31	SPAG9	ES	30	29	31	1.6E-20	0.223	0.135	3.9E-01	4.1E-02	1.5E-02
STAD	SPAG9_ES_7_6_8	SPAG9	ES	7	6	8	6.3E-10	0.113	-0.110	5.7E-02	7.9E-02	5.4E-01
STAD	SPTAN1_ES_23_22_24	SPTAN1	ES	23	22	24	7.2E-20	0.221	0.014	4.5E-03	1.2E-02	6.3E-02
STAD	SREBF1_ES_2_1_3.2	SREBF1	ES	2	1	3.2	1.2E-11	0.132	0.618	5.5E-02	6.0E-02	6.4E-01
STAD	SRP9_ES_3_2_5	SRP9	ES	3	2	5	9.3E-36	0.357	0.078	9.7E-02	1.3E-01	2.6E-01
STAD	SRP9_ME_3|4_2_5	SRP9	ME	3|4	2	5	2.7E-12	0.139	-0.077	7.4E-01	9.9E-01	5.8E-01
STAD	SRSF6_ES_3_2_4	SRSF6	ES	3	2	4	5.3E-10	0.113	0.448	1.3E-01	5.0E-02	4.4E-01
STAD	SSBP3_ES_7_6_8	SSBP3	ES	7	6	8	3.6E-12	0.137	0.108	6.1E-01	7.4E-01	5.1E-01
STAD	ST7_ES_10_9_11.1	ST7	ES	10	9	11.1	6.0E-10	0.113	-0.198	6.3E-02	3.1E-02	1.8E-01
STAD	STAG3_ES_13_12_14	STAG3	ES	13	12	14	3.6E-10	0.135	0.547	2.6E-03	1.6E-02	3.6E-01
STAD	SULF2_ES_20_19.2_21	SULF2	ES	20	19.2	21	9.7E-12	0.133	-0.362	2.6E-02	2.1E-02	3.0E-01
STAD	SUN1_ES_12_11_13	SUN1	ES	12	11	13	1.3E-09	0.115	0.013	8.3E-01	9.5E-01	3.2E-01
STAD	SVIL_ES_10:11:12_9_13	SVIL	ES	10:11:12	9	13	1.1E-09	0.110	0.534	1.2E-01	7.8E-03	4.5E-01
STAD	SVIL_ES_16_15_17	SVIL	ES	16	15	17	1.0E-09	0.111	0.741	1.0E+00	1.9E-02	8.0E-01
STAD	SYNE2_ES_118_117.2_119.2	SYNE2	ES	118	117.2	119.2	9.3E-13	0.144	-0.132	4.8E-04	1.9E-02	2.4E-01
STAD	TANGO2_ES_7.2_6_8	TANGO2	ES	7.2	6	8	2.9E-09	0.105	0.257	5.8E-01	1.4E-01	2.6E-01
STAD	TAZ_ES_7_6_8.1	TAZ	ES	7	6	8.1	2.3E-14	0.161	-0.171	1.1E-01	1.9E-02	4.1E-02
STAD	TBC1D1_ES_13:14_12_15	TBC1D1	ES	13:14	12	15	9.4E-10	0.111	0.505	1.9E-01	2.0E-02	7.3E-02
STAD	TBC1D23_ES_15_14_16	TBC1D23	ES	15	14	16	5.9E-12	0.135	-0.246	4.5E-02	8.8E-02	4.8E-01
STAD	TCF3_AA_19.3_19.1_19.4	TCF3	AA	19.3	19.1	19.4	1.0E-09	0.110	-0.167	4.5E-01	7.5E-01	1.6E-01
STAD	TCF3_RI_19.2_19.1_19.3	TCF3	RI	19.2	19.1	19.3	3.1E-27	0.286	0.063	6.4E-01	9.8E-01	3.4E-01
STAD	TCF7_ES_6_5_7	TCF7	ES	6	5	7	1.2E-09	0.111	-0.014	4.6E-01	3.8E-01	3.4E-01
STAD	TCF7L2_ES_17_14_19	TCF7L2	ES	17	14	19	2.0E-10	0.118	0.182	2.7E-01	2.2E-01	2.6E-01
STAD	TCF7L2_ES_4_3_5	TCF7L2	ES	4	3	5	1.5E-11	0.131	0.360	8.2E-02	6.5E-02	4.0E-02
STAD	TEAD1_ES_6_5_7.2	TEAD1	ES	6	5	7.2	2.5E-10	0.130	0.453	6.0E-02	2.8E-02	2.4E-01
STAD	THUMPD1_RI_5.2_5.1_5.3	THUMPD1	RI	5.2	5.1	5.3	2.4E-17	0.192	0.148	8.4E-01	6.5E-01	5.6E-01
STAD	TJP1_AD_30.2_30.1_31	TJP1	AD	30.2	30.1	31	4.5E-14	0.158	0.030	3.4E-01	3.3E-01	1.2E-01
STAD	TMC6_AD_14.2:14.3_14.1_15	TMC6	AD	14.2:14.3	14.1	15	4.2E-09	0.106	-0.094	3.5E-01	3.8E-01	5.8E-01
STAD	TMC6_ES_14.3_14.1_15	TMC6	ES	14.3	14.1	15	5.0E-18	0.199	0.094	8.9E-01	6.7E-01	5.6E-01
STAD	TMEM164_ES_3_1_4	TMEM164	ES	3	1	4	6.4E-11	0.145	0.126	2.3E-01	8.2E-01	5.8E-01
STAD	TMUB1_RI_4.2_4.1_4.3	TMUB1	RI	4.2	4.1	4.3	1.9E-16	0.187	-0.136	2.8E-01	2.3E-01	3.5E-01
STAD	TMUB2_AA_4.2_2.5_4.3	TMUB2	AA	4.2	2.5	4.3	7.5E-19	0.209	-0.005	6.3E-01	3.0E-01	6.2E-01
STAD	TMUB2_AA_4.2_3_4.3	TMUB2	AA	4.2	3	4.3	5.2E-41	0.405	0.109	6.6E-01	1.7E-01	1.2E-02
STAD	TMUB2_AD_2.2:2.3:2.4:2.5_2.1_4.3	TMUB2	AD	2.2:2.3:2.4:2.5	2.1	4.3	5.5E-09	0.102	-0.085	8.9E-01	7.9E-01	8.0E-01
STAD	TMUB2_AD_2.3:2.4:2.5_2.2_4.3	TMUB2	AD	2.3:2.4:2.5	2.2	4.3	4.4E-18	0.200	-0.127	4.4E-01	7.9E-01	7.4E-01
STAD	TMUB2_ES_2.2:2.3:2.4:2.5:3_2.1_4.3	TMUB2	ES	2.2:2.3:2.4:2.5:3	2.1	4.3	2.6E-11	0.143	-0.110	5.3E-01	2.0E-01	1.5E-01
STAD	TMUB2_ES_2.3:2.4:2.5:3_2.2_4.3	TMUB2	ES	2.3:2.4:2.5:3	2.2	4.3	2.0E-25	0.277	-0.232	1.1E-01	1.4E-01	5.6E-01
STAD	TMUB2_ES_3:4.2_2.5_4.3	TMUB2	ES	03:04.2	2.5	4.3	3.2E-24	0.258	-0.022	7.7E-01	6.5E-01	9.5E-01
STAD	TMUB2_ES_4.7:4.8_4.5_5	TMUB2	ES	4.7:4.8	4.5	5	3.3E-10	0.123	-0.149	4.1E-01	1.5E-01	3.6E-01
STAD	TNFRSF10B_RI_5.2_5.1_5.3	TNFRSF10B	RI	5.2	5.1	5.3	1.7E-13	0.152	-0.118	6.8E-01	2.4E-01	3.7E-01

cancer type	id	Gene Symbol	splice_type	Exon	From.Exon	To.Exon	anova.p	adj.r2	r	p.50	p.25	p.10
STAD	TNFRSF12A_ES_3.1:3.2_2.1_4	TNFRSF12A	ES	3.1:3.2	2.1	4	6.5E-08	0.102	-0.106	3.3E-01	7.7E-01	2.8E-01
STAD	TNS1_ES_23:24_22_25	TNS1	ES	23:24	22	25	6.6E-10	0.114	0.756	2.8E-02	3.2E-01	7.9E-01
STAD	TOM1L2_ES_14_13_15	TOM1L2	ES	14	13	15	1.0E-12	0.144	0.390	2.5E-01	3.9E-02	4.8E-01
STAD	TRERF1_ES_8.1_7_9	TRERF1	ES	8.1	7	9	1.7E-08	0.101	0.014	2.8E-01	1.1E-01	1.6E-01
STAD	TRPM2_ES_29_28_30	TRPM2	ES	29	28	30	1.6E-20	0.237	-0.361	1.9E-01	9.6E-01	5.2E-01
STAD	TSC2_ES_27:28.1_26_28.2	TSC2	ES	27:28.1	26	28.2	4.2E-09	0.104	-0.118	3.6E-01	2.9E-01	1.8E-01
STAD	TTC7B_ES_18_17_19	TTC7B	ES	18	17	19	6.1E-12	0.146	0.352	1.9E-01	1.6E-01	4.9E-01
STAD	TTI1_ES_2_1_3.2	TTI1	ES	2	1	3.2	7.3E-09	0.102	0.299	9.2E-01	4.2E-01	8.5E-01
STAD	VCL_ES_19_18.2_20	VCL	ES	19	18.2	20	1.2E-14	0.164	0.595	1.5E-01	6.8E-02	1.7E-02
STAD	VPS54_AA_3.1_2_3.2	VPS54	AA	3.1	2	3.2	2.3E-09	0.106	0.005	9.9E-01	4.2E-01	6.3E-01
STAD	WASF3_ME_7|8_6_9	WASF3	ME	7|8	6	9	2.9E-14	0.181	0.522	8.6E-01	9.7E-01	3.3E-01
STAD	WDFY3_ES_15_14_16	WDFY3	ES	15	14	16	4.4E-08	0.104	0.039	4.8E-02	1.6E-02	1.0E-01
STAD	WDFY3_ES_45_44_46	WDFY3	ES	45	44	46	6.2E-10	0.123	0.008	3.0E-02	1.7E-02	2.9E-01
STAD	WDR62_AA_25.7_25.5_25.8	WDR62	AA	25.7	25.5	25.8	1.7E-09	0.121	-0.113	1.2E-01	9.9E-01	2.7E-01
STAD	WDR62_RI_25.6:25.7_25.5_25.8	WDR62	RI	25.6:25.7	25.5	25.8	3.3E-10	0.119	-0.274	3.3E-01	7.2E-01	4.6E-01
STAD	WHSC1_RI_13.2_13.1_13.3	WHSC1	RI	13.2	13.1	13.3	2.3E-09	0.106	-0.134	9.7E-01	4.0E-01	3.2E-01
STAD	WHSC1L1_AD_21.2_21.1_22	WHSC1L1	AD	21.2	21.1	22	1.0E-08	0.104	-0.109	9.2E-01	1.0E+00	1.2E-01
STAD	WIPI2_ES_2_1_3	WIPI2	ES	2	1	3	7.0E-10	0.112	0.014	5.5E-01	3.9E-01	9.6E-03
STAD	WNK1_ES_15_13.2_16	WNK1	ES	15	13.2	16	8.4E-12	0.133	-0.084	2.5E-02	1.0E-01	4.3E-01
STAD	YBX3_ES_6_5_7	YBX3	ES	6	5	7	1.5E-11	0.131	0.010	3.3E-01	2.4E-02	1.6E-02
STAD	YIPF1_ES_12_11_13	YIPF1	ES	12	11	13	6.9E-09	0.101	-0.073	1.5E-01	3.9E-01	8.0E-01
STAD	YIPF2_AD_1.2_1.1_2	YIPF2	AD	1.2	1.1	2	1.5E-10	0.120	0.252	2.0E-01	1.1E-02	1.3E-02
STAD	ZDHHC20_ES_14_13.2_15	ZDHHC20	ES	14	13.2	15	2.4E-19	0.212	-0.080	6.6E-01	1.2E-01	3.0E-02
STAD	ZDHHC7_ES_4_3_5	ZDHHC7	ES	4	3	5	4.1E-11	0.126	0.006	1.5E-01	4.7E-02	2.1E-01
STAD	ZFAND1_ES_3_2_4	ZFAND1	ES	3	2	4	1.1E-09	0.110	-0.118	2.7E-01	3.8E-01	8.1E-01
STAD	ZNF384_AD_1.2_1.1_2.2	ZNF384	AD	1.2	1.1	2.2	4.5E-12	0.136	-0.215	1.8E-01	6.0E-02	3.5E-02
STAD	ZNF384_ES_8_7_9	ZNF384	ES	8	7	9	4.3E-14	0.158	-0.015	9.0E-01	2.0E-01	6.9E-01
STAD	ZNF438_ES_9_8_11.1	ZNF438	ES	9	8	11.1	1.7E-10	0.126	0.292	1.3E-01	1.7E-01	4.5E-01
STAD	ZNF791_ES_2.1:2.2_1_3	ZNF791	ES	2.1:2.2	1	3	2.9E-09	0.108	-0.408	4.2E-02	4.1E-02	1.3E-01
THCA	ABCA2_ES_4.1_3_4.6	ABCA2	ES	4.1	3	4.6	8.8E-15	0.162	-0.135	2.8E-01	4.9E-01	5.0E-02
THCA	ABCC3_RI_16.2_16.1_16.3	ABCC3	RI	16.2	16.1	16.3	2.0E-11	0.109	-0.493	4.8E-01	5.5E-01	2.1E-01
THCA	ABHD11_ES_5_3_6	ABHD11	ES	5	3	6	6.7E-15	0.136	0.172	1.0E+00	8.1E-01	6.5E-01
THCA	ABI1_ES_11.2_9_13	ABI1	ES	11.2	9	13	4.1E-11	0.105	-0.175	8.2E-01	9.6E-01	7.9E-01
THCA	ABI1_ES_11.2:12_9_13	ABI1	ES	11.2:12	9	13	1.3E-24	0.217	-0.368	7.2E-01	8.9E-01	9.5E-01
THCA	ABI1_ES_12_11.2_13	ABI1	ES	12	11.2	13	1.6E-27	0.236	-0.409	3.9E-01	6.0E-01	4.7E-01
THCA	ABLIM1_ES_16_15_17	ABLIM1	ES	16	15	17	2.1E-27	0.250	-0.190	6.1E-01	9.3E-01	5.7E-01
THCA	ACLY_ES_14_13_15	ACLY	ES	14	13	15	2.6E-27	0.238	0.153	5.9E-01	1.3E-01	1.1E-01
THCA	ACPL2_ES_6_5_9	ACPL2	ES	6	5	9	5.3E-10	0.110	0.135	8.1E-02	2.2E-01	2.1E-01
THCA	ACSL1_ME_16|17_15_18.1	ACSL1	ME	16|17	15	18.1	8.2E-23	0.204	-0.464	3.6E-01	5.3E-01	1.0E+00
THCA	ACTG1_RI_1.3:1.4_1.2_1.5	ACTG1	RI	1.3:1.4	1.2	1.5	2.7E-19	0.173	-0.171	1.1E-01	9.1E-01	9.6E-01

cancer type	id	Gene Symbol	splice_type	Exon	From.Exon	To.Exon	anova.p	adj.r2	r	p.50	p.25	p.10
THCA	ADAM15_ES_21.1:21.2_20_22.1	ADAM15	ES	21.1:21.2	20	22.1	4.0E-12	0.111	-0.151	4.5E-02	1.4E-02	5.6E-02
THCA	ADAM15_ES_21.2_20_22.1	ADAM15	ES	21.2	20	22.1	4.3E-11	0.102	-0.185	6.1E-02	1.3E-02	1.7E-01
THCA	ADAMTS9_ES_5_4_6	ADAMTS9	ES	5	4	6	2.1E-10	0.121	-0.113	7.7E-01	9.6E-01	3.8E-01
THCA	ADORA1_ES_4_3.2_5	ADORA1	ES	4	3.2	5	1.5E-15	0.151	-0.376	5.1E-01	2.5E-02	5.5E-02
THCA	ANKLE2_RI_1.2_1.1_1.3	ANKLE2	RI	1.2	1.1	1.3	2.5E-14	0.130	-0.290	7.3E-01	9.1E-01	5.1E-01
THCA	ANKRD13D_ES_2_1_3.1	ANKRD13D	ES	2	1	3.1	2.5E-12	0.118	-0.008	4.2E-01	4.9E-01	3.3E-01
THCA	AP1B1_ES_24_23_25	AP1B1	ES	24	23	25	1.2E-14	0.133	-0.092	4.2E-01	8.7E-01	4.4E-01
THCA	AP1G1_ES_12_10.1_13.2	AP1G1	ES	12	10.1	13.2	1.5E-12	0.136	0.012	1.9E-02	1.1E-01	1.7E-01
THCA	AP1G2_ES_5_4_6	AP1G2	ES	5	4	6	2.5E-14	0.134	-0.392	2.0E-01	1.3E-02	1.4E-01
THCA	AP1G2_RI_1.2:1.3_1.1_1.4	AP1G2	RI	1.2:1.3	1.1	1.4	6.5E-14	0.127	0.228	5.3E-01	9.3E-01	3.7E-01
THCA	AP1G2_RI_1.3_1.2_1.4	AP1G2	RI	1.3	1.2	1.4	1.3E-14	0.135	0.107	1.3E-01	1.1E-01	7.5E-01
THCA	APBB2_ES_8_7.2_9	APBB2	ES	8	7.2	9	3.3E-13	0.125	-0.007	2.5E-01	4.6E-02	9.8E-01
THCA	APEH_ES_2_1_4	APEH	ES	2	1	4	9.0E-13	0.125	0.130	2.9E-01	2.6E-01	8.2E-01
THCA	APOD_ES_3_2_4	APOD	ES	3	2	4	4.0E-25	0.219	-0.644	5.2E-01	2.5E-02	9.8E-04
THCA	ARAP1_ES_32_31_33	ARAP1	ES	32	31	33	1.4E-27	0.235	0.331	8.7E-01	2.1E-01	9.7E-01
THCA	ARHGAP17_ES_18_17_19	ARHGAP17	ES	18	17	19	1.5E-37	0.306	0.025	9.9E-01	2.9E-01	7.5E-01
THCA	ARHGEF1_ES_15_14_16	ARHGEF1	ES	15	14	16	1.3E-15	0.142	0.721	2.4E-01	6.0E-01	5.7E-01
THCA	ARHGEF10_ES_10_9_11	ARHGEF10	ES	10	9	11	1.3E-10	0.107	0.019	9.3E-01	4.1E-01	7.0E-01
THCA	ARHGEF11_ES_39_38_40	ARHGEF11	ES	39	38	40	4.7E-19	0.171	-0.026	7.0E-01	9.9E-01	6.0E-01
THCA	ARID1B_ES_12_11_13	ARID1B	ES	12	11	13	1.0E-13	0.129	-0.227	3.1E-02	9.7E-01	7.4E-01
THCA	ARMCX3_AA_3.1_1_3.2	ARMCX3	AA	3.1	1	3.2	5.5E-22	0.198	0.423	3.2E-02	1.3E-02	6.7E-01
THCA	ARRDC1_RI_2.2_2.1_2.3	ARRDC1	RI	2.2	2.1	2.3	6.4E-12	0.109	0.158	1.5E-01	7.2E-01	4.5E-01
THCA	ARSA_RI_1.3:1.4_1.2_1.5	ARSA	RI	1.3:1.4	1.2	1.5	1.6E-18	0.166	-0.193	7.4E-01	3.5E-01	3.9E-02
THCA	ASAP2_ES_23_22_24	ASAP2	ES	23	22	24	3.2E-36	0.299	0.522	6.9E-02	1.4E-01	1.4E-01
THCA	ATL2_AA_17.1_15_17.2	ATL2	AA	17.1	15	17.2	2.4E-12	0.113	0.360	8.7E-01	3.1E-01	7.4E-01
THCA	ATP9B_ES_30_29_31.1	ATP9B	ES	30	29	31.1	1.2E-12	0.116	0.027	6.8E-01	7.8E-01	5.1E-01
THCA	ATXN2L_ES_22.2:22.4:22.5_22.1_22.6	ATXN2L	ES	22.2:22.4:22.5	22.1	22.6	2.8E-15	0.138	0.037	7.3E-01	9.5E-01	4.9E-01
THCA	ATXN2L_ES_22.2:22.4:22.5:22.6_22.1_2	ATXN2L	ES	2.2:22.4:22.5:22.	22.1	22.7	5.5E-11	0.101	-0.008	9.3E-01	6.9E-01	6.2E-01
THCA	B3GALNT1_ES_6:7_5_9.1	B3GALNT1	ES	6:07	5	9.1	2.6E-10	0.109	-0.034	9.3E-01	7.0E-01	8.3E-01
THCA	B4GALT3_AA_3.1_2.2_3.2	B4GALT3	AA	3.1	2.2	3.2	1.2E-26	0.231	-0.017	2.4E-01	1.4E-01	1.8E-01
THCA	BAIAP2_ES_17.1_16.1_18.1	BAIAP2	ES	17.1	16.1	18.1	3.2E-23	0.202	0.438	9.0E-01	3.0E-01	4.7E-01
THCA	BAIAP2_ES_17.1:17.2_16.1_18.1	BAIAP2	ES	17.1:17.2	16.1	18.1	2.4E-31	0.263	0.418	5.4E-01	4.6E-02	1.0E+00
THCA	BBIP1_ES_3:4_1_5.1	BBIP1	ES	3:04	1	5.1	8.3E-22	0.192	0.189	3.7E-01	6.4E-01	4.8E-01
THCA	BCKDHB_RI_11.2_11.1_11.3	BCKDHB	RI	11.2	11.1	11.3	5.6E-29	0.246	0.198	7.4E-01	4.7E-01	3.6E-01
THCA	BEX2_AD_1.2:1.3_1.1_2	BEX2	AD	1.2:1.3	1.1	2	3.0E-12	0.113	-0.179	9.6E-01	4.2E-01	8.3E-01
THCA	BIN1_ES_13_12_17	BIN1	ES	13	12	17	1.8E-14	0.131	-0.324	9.2E-02	5.7E-01	7.2E-01
THCA	BIN1_ES_13:16:17_12_18	BIN1	ES	13:16:17	12	18	2.8E-12	0.126	0.044	7.1E-01	2.2E-01	2.5E-02
THCA	BIN1_ES_16_13_17	BIN1	ES	16	13	17	9.6E-11	0.100	0.145	2.8E-01	8.9E-01	5.8E-02
THCA	BIN1_ES_16:17_12_18	BIN1	ES	16:17	12	18	2.5E-18	0.195	0.110	5.7E-01	6.4E-01	4.2E-02
THCA	BMP1_ES_17.1:17.2_16_18.1	BMP1	ES	17.1:17.2	16	18.1	3.5E-18	0.163	-0.240	3.7E-01	8.7E-01	8.8E-02

cancer type	id	Gene Symbol	splice_type	Exon	From.Exon	To.Exon	anova.p	adj.r2	r	p.50	p.25	p.10
THCA	C11orf49_ES_3:4_1_7	C11orf49	ES	3:04	1	7	3.3E-10	0.105	0.229	2.0E-01	7.9E-02	7.5E-02
THCA	C11orf49_RI_14.2_14.1_14.3	C11orf49	RI	14.2	14.1	14.3	6.0E-12	0.109	0.059	2.0E-03	1.1E-02	2.2E-01
THCA	C11orf74_AD_1.2_1.1_4	C11orf74	AD	1.2	1.1	4	2.3E-24	0.212	0.477	9.9E-01	6.0E-01	8.8E-01
THCA	C11orf80_ES_2:3_1_4	C11orf80	ES	2:03	1	4	3.4E-14	0.130	-0.260	5.9E-01	5.7E-01	8.2E-01
THCA	C14orf159_ES_6_5.2_7	C14orf159	ES	6	5.2	7	6.0E-17	0.155	-0.310	3.3E-01	1.6E-01	8.3E-01
THCA	C16orf91_RI_4.2_4.1_4.3	C16orf91	RI	4.2	4.1	4.3	9.0E-20	0.176	0.148	7.1E-01	4.2E-01	9.2E-01
THCA	C1orf50_ES_1.3_1.1_2.2	C1orf50	ES	1.3	1.1	2.2	6.0E-12	0.125	-0.099	5.3E-01	4.3E-01	7.8E-01
THCA	C22orf29_AD_1.2_1.1_1.4	C22orf29	AD	1.2	1.1	1.4	3.0E-10	0.107	0.201	7.7E-01	4.0E-01	9.8E-01
THCA	C4orf22_ES_6_4_8	C4orf22	ES	6	4	8	3.0E-10	0.113	-0.490	8.1E-01	5.7E-01	1.8E-01
THCA	C6orf203_AD_1.2_1.1_3	C6orf203	AD	1.2	1.1	3	2.2E-12	0.114	-0.086	5.5E-01	2.1E-01	3.0E-01
THCA	C6orf203_ES_1.2:2_1.1_3	C6orf203	ES	1.2:2	1.1	3	4.6E-14	0.128	-0.045	3.5E-01	2.8E-01	7.6E-01
THCA	CACNB3_ES_4_3_5	CACNB3	ES	4	3	5	7.9E-13	0.140	0.184	4.1E-01	4.2E-01	4.2E-01
THCA	CACNB3_ES_9_8_10	CACNB3	ES	9	8	10	2.5E-56	0.430	0.078	1.9E-04	7.5E-02	3.5E-01
THCA	CADM1_ES_10_9_12	CADM1	ES	10	9	12	3.9E-48	0.372	-0.209	7.9E-01	8.7E-01	8.1E-02
THCA	CAMK2B_ES_18:19:20_17_21.1	CAMK2B	ES	18:19:20	17	21.1	1.2E-12	0.116	-0.133	6.1E-01	8.0E-01	9.5E-01
THCA	CAMTA2_AA_7.1_6_7.2	CAMTA2	AA	7.1	6	7.2	2.8E-10	0.107	-0.042	7.1E-01	2.9E-01	2.9E-01
THCA	CAMTA2_ES_7.1:7.2:7.3_6_8	CAMTA2	ES	7.1:7.2:7.3	6	8	2.9E-10	0.100	0.040	7.5E-01	4.6E-01	2.4E-01
THCA	CAPN3_ES_11_10_12	CAPN3	ES	11	10	12	1.3E-10	0.117	-0.068	7.2E-02	2.2E-01	1.5E-02
THCA	CAPN3_ES_23:24_21_25	CAPN3	ES	23:24	21	25	2.1E-19	0.184	-0.263	4.3E-01	1.2E-01	9.6E-02
THCA	CARD8_ES_7.2_5_8	CARD8	ES	7.2	5	8	5.9E-13	0.129	0.048	2.2E-01	1.7E-01	4.7E-01
THCA	CASC4_ES_10_9_11	CASC4	ES	10	9	11	1.4E-22	0.198	-0.247	4.0E-01	4.5E-01	5.5E-01
THCA	CASK_ES_20_19.1_22	CASK	ES	20	19.1	22	8.2E-21	0.212	0.343	6.2E-01	2.8E-01	8.5E-01
THCA	CAST_ES_7.1:8.2_5.2_9	CAST	ES	7.1:8.2	5.2	9	1.5E-13	0.138	-0.458	7.9E-01	6.2E-01	1.8E-01
THCA	CCDC50_ES_6_5_7	CCDC50	ES	6	5	7	2.1E-20	0.181	-0.098	2.5E-01	2.0E-01	6.4E-01
THCA	CCDC84_AD_6.2_6.1_7	CCDC84	AD	6.2	6.1	7	1.2E-16	0.150	0.333	7.3E-02	1.7E-01	7.2E-02
THCA	CCDC90B_AD_1.2:1.3_1.1_2	CCDC90B	AD	1.2:1.3	1.1	2	2.5E-12	0.113	0.043	2.8E-01	4.2E-01	1.0E+00
THCA	CCND3_AD_3.2_3.1_4	CCND3	AD	3.2	3.1	4	5.7E-15	0.137	0.337	9.7E-01	6.6E-01	7.4E-01
THCA	CCNDBP1_ES_10.1_9_10.3	CCNDBP1	ES	10.1	9	10.3	1.5E-34	0.285	-0.112	3.2E-01	9.1E-02	8.9E-01
THCA	CD44_ES_10:11_5_12.1	CD44	ES	10:11	5	12.1	1.8E-11	0.106	-0.396	1.8E-01	7.1E-01	3.5E-01
THCA	CD44_ES_10:11:12.1:13:14_5_15	CD44	ES	10:11:12.1:13:14	5	15	9.0E-16	0.147	0.165	9.5E-01	1.8E-01	3.9E-01
THCA	CD44_ES_12.1:13:14_5_15	CD44	ES	12.1:13:14	5	15	4.3E-14	0.128	0.404	8.0E-01	7.4E-01	5.3E-01
THCA	CD44_ES_3.1:3.2:4:5:6:7:8:9.1:9.2:10:11	CD44	ES	9.2:10:11:12.1:13	2.1	17.2	1.3E-20	0.191	-0.439	9.6E-01	3.6E-01	3.1E-01
THCA	CD44_ES_3.1:3.2:4:5:6:7:8:9.2:10:11:12.	CD44	ES	2:10:11:12.1:13:1	2.1	17.2	6.7E-19	0.176	-0.435	7.1E-01	1.0E-01	5.1E-01
THCA	CD44_ES_3.1:3.2:4:5:7:8:9.1:9.2:10:11:1	CD44	ES	.2:10:11:12.1:13:	2.1	17.2	2.2E-12	0.114	-0.286	8.2E-01	7.9E-01	4.3E-01
THCA	CD44_ES_3.1:3.2:4:5:7:8:9.2:10:11:12.1:	CD44	ES	:10:11:12.1:13:14	2.1	17.2	1.1E-12	0.117	-0.315	8.7E-01	3.6E-01	6.9E-01
THCA	CD44_ES_6:7:8:9.1:9.2_5_10	CD44	ES	6:7:8:9.1:9.2	5	10	7.2E-15	0.139	-0.370	8.7E-01	2.1E-01	9.4E-01
THCA	CD44_ES_6:7:8:9.1:9.2:10:11:12.1:13_5_	CD44	ES	:9.1:9.2:10:11:12	5	14	5.8E-15	0.144	-0.209	1.9E-01	7.7E-02	4.2E-01
THCA	CD44_ES_6:7:8:9.1:9.2:10:11:12.1:13:14	CD44	ES	9.1:9.2:10:11:12.1	5	15	2.6E-31	0.264	-0.117	3.9E-01	6.7E-01	7.8E-01
THCA	CD44_ES_6:7:8:9.2:10:11:12.1:13_5_14	CD44	ES	:8:9.2:10:11:12.1	5	14	1.4E-16	0.162	-0.241	2.7E-01	4.7E-01	7.6E-01
THCA	CD44_ES_6:7:8:9.2:10:11:12.1:13:14_5_	CD44	ES	8:9.2:10:11:12.1:1	5	15	9.3E-30	0.253	-0.117	9.6E-01	1.1E-01	9.5E-01

cancer type	id	Gene Symbol	splice_type	Exon	From.Exon	To.Exon	anova.p	adj.r2	r	p.50	p.25	p.10
THCA	CD44_ES_7:12.1:13:14_5_15	CD44	ES	7:12.1:13:14	5	15	1.2E-37	0.320	0.314	8.2E-01	8.2E-01	8.1E-01
THCA	CD44_ES_7:8:9.1:9.2_5_10	CD44	ES	7:8:9.1:9.2	5	10	5.2E-13	0.121	-0.401	8.8E-01	7.3E-01	3.1E-01
THCA	CD44_ES_7:8:9.1:9.2:10:11_5_12.1	CD44	ES	7:8:9.1:9.2:10:11	5	12.1	2.7E-15	0.139	-0.431	7.7E-01	5.8E-01	8.8E-01
THCA	CD44_ES_7:8:9.1:9.2:10:11:12.1:13:14_5	CD44	ES	1:9.2:10:11:12.1:	5	15	1.5E-33	0.279	-0.104	9.3E-01	9.5E-01	5.7E-01
THCA	CD44_ES_7:8:9.2_5_10	CD44	ES	08:09.2	5	10	8.6E-15	0.137	-0.424	2.7E-01	1.7E-01	1.1E-01
THCA	CD44_ES_7:8:9.2:10:11_5_12.1	CD44	ES	7:8:9.2:10:11	5	12.1	1.5E-15	0.141	-0.429	4.6E-01	1.0E-01	9.3E-02
THCA	CD44_ES_7:8:9.2:10:11:12.1:13:14_5_15	CD44	ES	9.2:10:11:12.1:1	5	15	3.1E-34	0.284	-0.112	7.1E-01	1.8E-01	2.4E-01
THCA	CD44_ES_8:9.1:9.2:10:11_7_12.1	CD44	ES	8:9.1:9.2:10:11	7	12.1	2.0E-12	0.125	-0.388	6.0E-01	5.8E-01	3.1E-01
THCA	CD44_ES_8:9.2:10:11_7_12.1	CD44	ES	8:9.2:10:11	7	12.1	9.4E-13	0.126	-0.404	2.6E-01	2.7E-01	1.0E+00
THCA	CDC42SE1_ES_5_4_6	CDC42SE1	ES	5	4	6	5.5E-20	0.178	-0.057	6.3E-02	5.3E-02	3.1E-01
THCA	CDKL1_AA_9.1_8_9.2	CDKL1	AA	9.1	8	9.2	1.7E-10	0.104	-0.506	8.6E-01	4.6E-01	7.9E-01
THCA	CDKN2AIP_AD_2.2_2.1_3	CDKN2AIP	AD	2.2	2.1	3	9.4E-13	0.122	-0.116	2.3E-01	1.9E-02	1.0E-01
THCA	CEP170_AD_16.2_16.1_17	CEP170	AD	16.2	16.1	17	3.1E-12	0.118	0.095	4.9E-01	1.5E-01	1.5E-01
THCA	CEP170_AD_16.3_16.2_17	CEP170	AD	16.3	16.2	17	1.0E-12	0.126	-0.091	3.6E-01	4.5E-01	9.9E-02
THCA	CEP57_AD_10.2:10.3_10.1_11	CEP57	AD	10.2:10.3	10.1	11	2.1E-20	0.181	-0.134	1.9E-01	3.4E-01	5.1E-01
THCA	CFLAR_ES_10_8.1_11	CFLAR	ES	10	8.1	11	3.9E-26	0.225	-0.690	3.5E-01	9.5E-01	3.9E-01
THCA	CHCHD4_ES_2_1_4	CHCHD4	ES	2	1	4	7.4E-20	0.178	-0.131	4.9E-01	4.8E-01	5.7E-01
THCA	CHEK2_ES_2_1_3	CHEK2	ES	2	1	3	6.4E-14	0.144	-0.352	2.1E-01	1.2E-01	2.4E-01
THCA	CHEK2_ME_6|7.1:7.2_5_9	CHEK2	ME	6|7.1:7.2	5	9	1.3E-37	0.306	0.528	7.3E-01	7.4E-01	9.7E-01
THCA	CHTF8_AA_4.1:4.2_3_4.3	CHTF8	AA	4.1:4.2	3	4.3	1.0E-10	0.103	-0.207	8.0E-02	1.7E-01	3.7E-02
THCA	CHTF8_ES_4.3:4.4_4.1_4.6	CHTF8	ES	4.3:4.4	4.1	4.6	4.3E-13	0.120	0.071	3.0E-01	5.9E-01	9.5E-01
THCA	CKB_ES_5:6_3_7	CKB	ES	5:06	3	7	8.8E-09	0.104	-0.191	7.2E-02	2.4E-01	3.2E-01
THCA	CKMT2_ES_2_1.1_3	CKMT2	ES	2	1.1	3	2.1E-10	0.112	-0.719	5.0E-02	1.6E-01	4.6E-01
THCA	CLASP1_ES_22_21_24	CLASP1	ES	22	21	24	6.8E-16	0.167	-0.035	3.2E-01	4.4E-01	1.0E+00
THCA	CLEC16A_AD_11.2_11.1_12	CLEC16A	AD	11.2	11.1	12	4.9E-13	0.125	-0.165	6.4E-01	6.2E-01	1.4E-01
THCA	CLSTN1_ES_11_10_12	CLSTN1	ES	11	10	12	1.9E-19	0.174	-0.190	6.1E-01	4.7E-01	3.7E-01
THCA	CLU_RI_3.2_3.1_3.3	CLU	RI	3.2	3.1	3.3	3.3E-14	0.142	0.450	3.2E-01	4.9E-01	5.0E-01
THCA	CLYBL_RI_9.2_9.1_9.3	CLYBL	RI	9.2	9.1	9.3	1.2E-11	0.107	-0.019	9.9E-01	9.0E-01	1.3E-01
THCA	COASY_RI_1.2:1.3:1.4_1.1_1.5	COASY	RI	1.2:1.3:1.4	1.1	1.5	4.8E-14	0.129	-0.246	9.6E-01	6.6E-01	4.0E-01
THCA	COL16A1_ES_44_43_45.1	COL16A1	ES	44	43	45.1	5.5E-23	0.202	0.053	1.1E-03	6.4E-02	4.8E-01
THCA	COL6A3_ES_3:4_2_5	COL6A3	ES	3:04	2	5	1.6E-10	0.121	0.183	7.8E-01	6.0E-01	5.6E-01
THCA	COL6A3_ES_6_5_7	COL6A3	ES	6	5	7	7.6E-25	0.242	0.364	7.6E-01	6.0E-02	3.0E-01
THCA	COPS5_ES_2.3_2.1_5	COPS5	ES	2.3	2.1	5	2.0E-11	0.126	-0.169	7.4E-01	8.0E-01	2.3E-01
THCA	COQ4_RI_2.2_2.1_2.3	COQ4	RI	2.2	2.1	2.3	3.9E-12	0.111	0.245	3.8E-01	8.1E-01	2.3E-01
THCA	COX11_RI_3.2_3.1_3.3	COX11	RI	3.2	3.1	3.3	2.9E-15	0.138	0.009	2.0E-01	1.8E-01	7.6E-01
THCA	COX4I1_AA_5.1:5.2:5.3_4.1_5.4	COX4I1	AA	5.1:5.2:5.3	4.1	5.4	1.2E-17	0.158	-0.336	8.3E-02	1.2E-01	4.2E-01
THCA	CRB3_RI_5.2_5.1_5.3	CRB3	RI	5.2	5.1	5.3	2.0E-24	0.212	-0.490	1.2E-01	1.2E-01	8.0E-01
THCA	CRELD1_AD_1.2_1.1_1.4	CRELD1	AD	1.2	1.1	1.4	1.3E-11	0.107	0.506	3.8E-01	2.5E-01	9.8E-01
THCA	CRELD1_RI_1.2:1.3_1.1_1.4	CRELD1	RI	1.2:1.3	1.1	1.4	4.0E-17	0.154	0.372	8.2E-02	2.3E-01	1.2E-01
THCA	CRYGN_AD_2.2_2.1_3	CRYGN	AD	2.2	2.1	3	1.2E-21	0.196	-0.599	3.7E-01	2.2E-02	1.1E-01

cancer type	id	Gene Symbol	splice_type	Exon	From.Exon	To.Exon	anova.p	adj.r2	r	p.50	p.25	p.10
THCA	CSF2RA_ES_13_12_14	CSF2RA	ES	13	12	14	5.9E-39	0.315	-0.780	7.2E-01	9.0E-01	8.8E-01
THCA	CYB561A3_AA_6.6_6.4_6.7	CYB561A3	AA	6.6	6.4	6.7	2.0E-16	0.148	0.356	4.0E-01	3.9E-01	7.2E-01
THCA	CYTH1_ES_11.2:13.1_11.1_13.2	CYTH1	ES	11.2:13.1	11.1	13.2	3.6E-13	0.123	0.237	5.2E-01	1.4E-01	3.5E-01
THCA	CYTH1_ES_12_11.1_13.2	CYTH1	ES	12	11.1	13.2	2.4E-13	0.125	0.235	9.3E-01	1.2E-01	2.0E-01
THCA	D2HGDH_ES_7.1_6_8	D2HGDH	ES	7.1	6	8	1.6E-15	0.153	0.013	9.9E-01	5.1E-01	5.0E-01
THCA	D2HGDH_ES_7.1:7.2:7.3_6_8	D2HGDH	ES	7.1:7.2:7.3	6	8	5.1E-11	0.102	0.259	5.2E-01	4.0E-01	9.6E-01
THCA	DAP3_AD_2.3_2.2_3	DAP3	AD	2.3	2.2	3	5.2E-15	0.136	-0.228	3.7E-01	7.7E-01	9.6E-01
THCA	DAW1_ES_12_11_13	DAW1	ES	12	11	13	1.1E-13	0.157	-0.666	7.9E-01	4.3E-01	7.6E-01
THCA	DBI_AD_1.2:1.3_1.1_3.1	DBI	AD	1.2:1.3	1.1	3.1	2.2E-13	0.124	-0.054	7.6E-01	6.7E-01	7.2E-01
THCA	DCAF11_ES_1.2:2.2_1.1_2.4	DCAF11	ES	1.2:2.2	1.1	2.4	4.6E-15	0.141	-0.011	2.8E-01	4.1E-02	3.7E-02
THCA	DCAF6_ES_11:13.1_10_14	DCAF6	ES	11:13.1	10	14	6.0E-14	0.131	0.107	6.4E-01	2.5E-01	9.7E-01
THCA	DCAF6_ES_13.1_10_14	DCAF6	ES	13.1	10	14	1.2E-11	0.109	0.090	8.9E-01	3.1E-01	5.3E-01
THCA	DDX52_ES_3_2_5	DDX52	ES	3	2	5	5.6E-18	0.166	-0.583	3.3E-02	1.4E-01	5.1E-01
THCA	DERL3_AA_5.4_5.2_5.5	DERL3	AA	5.4	5.2	5.5	2.2E-13	0.122	0.283	8.7E-01	9.1E-01	5.3E-01
THCA	DERL3_RI_5.3_5.2_5.4	DERL3	RI	5.3	5.2	5.4	3.4E-14	0.134	-0.405	2.1E-01	3.9E-01	1.1E-01
THCA	DGUOK_ES_4:5_1_7	DGUOK	ES	4:05	1	7	2.0E-13	0.123	-0.019	9.4E-01	9.0E-01	5.8E-01
THCA	DLG1_ES_9_7_10	DLG1	ES	9	7	10	9.4E-19	0.178	-0.228	8.9E-01	3.4E-01	4.4E-01
THCA	DMD_ES_82_81_83	DMD	ES	82	81	83	2.8E-13	0.133	-0.408	3.0E-01	4.7E-02	4.0E-01
THCA	DMKN_ES_11:12_6.4_13	DMKN	ES	11:12	6.4	13	6.7E-13	0.124	-0.260	4.2E-01	8.7E-01	7.7E-01
THCA	DMKN_ES_11:12_7_13	DMKN	ES	11:12	7	13	5.1E-15	0.136	-0.204	6.9E-01	9.7E-02	2.2E-01
THCA	DMKN_ES_7:11:12_6.4_13	DMKN	ES	7:11:12	6.4	13	6.4E-15	0.135	-0.224	4.2E-01	6.2E-01	4.9E-01
THCA	DNASE1L2_RI_1.2:1.3_1.1_1.4	DNASE1L2	RI	1.2:1.3	1.1	1.4	4.0E-10	0.101	-0.543	6.6E-01	9.6E-01	8.0E-01
THCA	DNM1L_ES_17:18_16_19	DNM1L	ES	17:18	16	19	3.4E-14	0.130	-0.255	6.8E-01	6.7E-01	1.9E-01
THCA	DNM1L_ES_18_16_19	DNM1L	ES	18	16	19	2.0E-16	0.152	-0.200	3.9E-01	8.4E-01	6.3E-01
THCA	DOCK7_ES_24_23_25	DOCK7	ES	24	23	25	5.2E-10	0.110	0.109	5.7E-01	9.2E-02	3.8E-01
THCA	DOCK9_ES_48_47_49	DOCK9	ES	48	47	49	5.9E-21	0.187	0.446	4.8E-01	9.6E-01	1.0E+00
THCA	DST_ES_106_105_107	DST	ES	106	105	107	1.3E-25	0.221	-0.435	1.4E-01	7.4E-01	6.5E-01
THCA	DST_ES_43:44:45:46.1:48_42_49	DST	ES	43:44:45:46.1:48	42	49	1.1E-22	0.249	-0.634	3.5E-01	2.6E-01	5.2E-01
THCA	DTD2_RI_3.2_3.1_3.3	DTD2	RI	3.2	3.1	3.3	4.9E-12	0.110	0.367	7.4E-02	8.8E-02	5.2E-01
THCA	DUOXA2_AA_2.1_1_2.2	DUOXA2	AA	2.1	1	2.2	3.5E-19	0.171	-0.563	8.5E-01	8.1E-01	4.6E-02
THCA	DYSF_ES_19_18_20	DYSF	ES	19	18	20	3.8E-49	0.428	-0.132	1.9E-01	3.2E-02	8.8E-01
THCA	DYSF_ES_43_42_44	DYSF	ES	43	42	44	1.2E-28	0.248	-0.034	3.2E-01	3.5E-01	4.6E-01
THCA	DYSF_ES_7_6_8	DYSF	ES	7	6	8	1.8E-24	0.236	0.130	8.2E-01	8.5E-01	9.7E-01
THCA	EEF1B2_AA_1.3_1.1_1.4	EEF1B2	AA	1.3	1.1	1.4	6.2E-25	0.217	0.490	5.5E-01	7.6E-01	9.0E-01
THCA	EFCAB2_ES_2:3_1.3_4	EFCAB2	ES	2:03	1.3	4	3.3E-10	0.103	0.214	1.4E-01	7.9E-01	5.3E-01
THCA	EHBP1_ES_18_17_19	EHBP1	ES	18	17	19	1.3E-15	0.143	0.350	4.4E-01	9.6E-01	7.2E-01
THCA	EIF4A2_ES_9.1:9.2_8_10	EIF4A2	ES	9.1:9.2	8	10	1.0E-12	0.119	-0.245	2.3E-02	1.7E-02	1.1E-01
THCA	EIF4E2_ES_8_6.1_9	EIF4E2	ES	8	6.1	9	2.7E-12	0.113	0.075	9.8E-01	6.0E-01	1.7E-01
THCA	ELF2_AA_10.1_9_10.2	ELF2	AA	10.1	9	10.2	4.0E-13	0.125	-0.048	2.5E-01	3.4E-01	4.6E-01
THCA	ELMO1_AD_1.2_1.1_3	ELMO1	AD	1.2	1.1	3	4.7E-35	0.323	-0.673	1.7E-01	1.4E-01	3.8E-01

cancer type	id	Gene Symbol	splice_type	Exon	From.Exon	To.Exon	anova.p	adj.r2	r	p.50	p.25	p.10
THCA	ELN_AA_24.1_23_24.2	ELN	AA	24.1	23	24.2	5.2E-22	0.221	-0.702	7.4E-01	6.7E-01	9.8E-01
THCA	ELN_ES_22:23:24.1_21_24.2	ELN	ES	23:24.1	21	24.2	6.6E-25	0.270	-0.567	3.0E-01	7.3E-01	5.1E-01
THCA	ELN_ES_23:24.1_21_24.2	ELN	ES	23:24.1	21	24.2	2.0E-28	0.298	-0.570	3.5E-01	6.6E-01	7.5E-01
THCA	EMC10_ES_7_6_8.1	EMC10	ES	7	6	8.1	6.7E-18	0.160	0.104	5.0E-01	8.9E-01	7.6E-01
THCA	EMID1_ME_14|15_13_16	EMID1	ME	14|15	13	16	2.8E-14	0.131	0.204	4.4E-01	3.5E-01	5.4E-02
THCA	EPB41L1_ES_20_19_21	EPB41L1	ES	20	19	21	5.1E-22	0.194	-0.228	5.8E-01	7.4E-01	9.5E-01
THCA	EPB41L3_ES_25_24_26	EPB41L3	ES	25	24	26	8.2E-25	0.219	-0.367	4.3E-01	7.4E-01	2.3E-01
THCA	EPOR_ES_4.1:4.2_3_5	EPOR	ES	4.1:4.2	3	5	3.1E-13	0.121	0.653	1.4E-01	1.4E-01	4.1E-01
THCA	EPOR_ES_4.2_3_5	EPOR	ES	4.2	3	5	1.9E-13	0.126	0.698	9.3E-02	1.9E-02	1.8E-01
THCA	EPS15L1_ES_22:23.1_21_24	EPS15L1	ES	22:23.1	21	24	2.0E-18	0.164	0.171	9.1E-01	3.3E-01	3.2E-01
THCA	ERLIN2_RI_7.2_7.1_7.3	ERLIN2	RI	7.2	7.1	7.3	9.7E-13	0.119	0.290	2.1E-01	9.5E-02	9.6E-02
THCA	ERMAP_RI_3.3_3.2_3.4	ERMAP	RI	3.3	3.2	3.4	1.4E-13	0.128	-0.398	8.2E-02	2.7E-01	6.7E-01
THCA	ERRFI1_AA_3.1:3.2:3.3_2_3.4	ERRFI1	AA	3.1:3.2:3.3	2	3.4	6.6E-13	0.129	-0.077	8.5E-02	7.0E-01	3.8E-01
THCA	EXOC7_ES_7_6_8.2	EXOC7	ES	7	6	8.2	4.2E-37	0.303	0.221	7.2E-02	1.6E-01	6.7E-01
THCA	EXOC7_ES_7_6_9	EXOC7	ES	7	6	9	3.8E-16	0.149	0.199	9.5E-01	3.6E-01	2.9E-01
THCA	EXOC7_ES_7:8.1_6_8.2	EXOC7	ES	07:08.1	6	8.2	4.3E-12	0.112	0.481	8.2E-01	6.7E-01	8.1E-01
THCA	EXOC7_ES_7:8.1:8.2_6_9	EXOC7	ES	7:8.1:8.2	6	9	1.3E-11	0.107	0.489	7.9E-01	9.0E-01	6.3E-01
THCA	EXOC7_ES_7:8.2_6_9	EXOC7	ES	07:08.2	6	9	1.0E-28	0.245	0.195	2.2E-01	7.0E-01	7.2E-01
THCA	FAM13A_AA_16.1_15_16.2	FAM13A	AA	16.1	15	16.2	3.1E-15	0.146	0.657	3.1E-01	8.2E-01	7.2E-01
THCA	FAM195A_ES_3_2_4	FAM195A	ES	3	2	4	2.2E-20	0.180	0.016	1.5E-01	4.1E-01	2.2E-01
THCA	FAM214A_AD_12.2_12.1_13	FAM214A	AD	12.2	12.1	13	7.1E-11	0.101	0.017	2.5E-01	3.2E-01	4.3E-01
THCA	FAM47E_AD_6.2_6.1_7	FAM47E	AD	6.2	6.1	7	3.4E-14	0.130	0.168	4.7E-01	2.8E-01	5.0E-01
THCA	FAM47E_ES_4_3_5	FAM47E	ES	4	3	5	3.8E-27	0.233	-0.338	4.7E-01	7.7E-01	1.3E-01
THCA	FAM63A_AA_4.1_2.2_4.2	FAM63A	AA	4.1	2.2	4.2	1.8E-22	0.202	0.331	8.4E-01	6.6E-01	8.9E-01
THCA	FAM63A_AA_4.2:4.3_2.2_4.4	FAM63A	AA	4.2:4.3	2.2	4.4	1.3E-14	0.144	-0.220	6.4E-01	6.2E-01	5.9E-01
THCA	FAM86B1_ES_4:5:6:7.1_3.2_8.1	FAM86B1	ES	4:5:6:7.1	3.2	8.1	8.2E-18	0.164	0.413	6.4E-01	8.3E-01	4.0E-01
THCA	FAM86B1_ES_5_4_6	FAM86B1	ES	5	4	6	2.0E-15	0.144	0.298	4.9E-01	6.3E-01	7.2E-01
THCA	FAM86B1_ES_5:6:7.1_4_8.1	FAM86B1	ES	06:07.1	4	8.1	3.2E-43	0.344	0.424	2.4E-01	2.8E-01	4.4E-01
THCA	FAM86B1_ES_5:6:7.1:7.2:7.3_4_8.1	FAM86B1	ES	5:6:7.1:7.2:7.3	4	8.1	7.8E-31	0.264	0.253	1.2E-01	1.3E-01	1.0E-02
THCA	FAM86B1_ES_5:6:7.1:7.3_4_8.1	FAM86B1	ES	5:6:7.1:7.3	4	8.1	1.9E-19	0.184	0.298	2.4E-01	2.8E-01	2.3E-01
THCA	FAM86B1_ES_6:7.1_4_8.1	FAM86B1	ES	06:07.1	4	8.1	4.5E-16	0.152	0.146	9.8E-01	7.4E-01	4.6E-01
THCA	FAM86B1_ES_6:7.1:7.2:7.3_4_8.1	FAM86B1	ES	6:7.1:7.2:7.3	4	8.1	3.9E-12	0.118	-0.004	3.8E-01	1.5E-01	1.7E-01
THCA	FAM86B1_ES_7.1_4_8.1	FAM86B1	ES	7.1	4	8.1	1.7E-19	0.183	0.294	2.7E-01	2.8E-01	8.0E-02
THCA	FAM86B1_ES_7.1:7.2:7.3_4_8.1	FAM86B1	ES	7.1:7.2:7.3	4	8.1	2.1E-21	0.199	0.164	3.0E-02	8.6E-02	2.5E-02
THCA	FAM86C1_ES_3.1:3.2:4_2_5.1	FAM86C1	ES	3.1:3.2:4	2	5.1	5.7E-10	0.106	0.181	4.8E-01	1.5E-02	4.0E-02
THCA	FAM86C1_ES_3.2_2_5.1	FAM86C1	ES	3.2	2	5.1	1.8E-12	0.114	-0.046	9.3E-01	4.7E-01	8.0E-01
THCA	FAM86C1_ES_4_3.2_5.1	FAM86C1	ES	4	3.2	5.1	9.8E-19	0.168	0.169	1.4E-01	8.3E-02	2.6E-03
THCA	FBLN2_ES_11_10_12	FBLN2	ES	11	10	12	1.3E-14	0.139	0.358	7.6E-01	5.1E-01	2.6E-01
THCA	FBLN5_ES_7_5_8	FBLN5	ES	7	5	8	4.8E-12	0.110	-0.444	9.8E-01	7.6E-01	7.6E-01
THCA	FCHSD1_ES_19_18_20	FCHSD1	ES	19	18	20	2.8E-13	0.124	0.108	4.8E-01	6.8E-01	8.6E-01

cancer type	id	Gene Symbol	splice_type	Exon	From.Exon	To.Exon	anova.p	adj.r2	r	p.50	p.25	p.10
THCA	FGFR1_ES_6_4_8.2	FGFR1	ES	6	4	8.2	1.0E-15	0.150	0.344	3.5E-01	3.7E-02	4.5E-02
THCA	FGFR2_ES_4_3_5	FGFR2	ES	4	3	5	1.3E-14	0.135	0.125	3.1E-01	4.1E-01	3.4E-01
THCA	FMNL3_ES_6_5_7	FMNL3	ES	6	5	7	4.4E-30	0.262	0.092	8.0E-01	4.7E-01	1.2E-01
THCA	FN1_ES_25_24_26	FN1	ES	25	24	26	5.7E-25	0.217	0.124	5.5E-02	1.3E-01	2.9E-02
THCA	FN1_ES_33_32_34	FN1	ES	33	32	34	2.7E-11	0.104	-0.193	1.7E-02	2.3E-02	1.7E-01
THCA	GBA2_RI_14.3_14.2_14.4	GBA2	RI	14.3	14.2	14.4	4.5E-19	0.170	-0.453	8.2E-01	8.0E-01	9.1E-01
THCA	GBA2_RI_14.5_14.4_14.6	GBA2	RI	14.5	14.4	14.6	4.0E-21	0.186	-0.463	7.5E-01	6.1E-01	4.2E-01
THCA	GBA2_RI_15.2:15.3_15.1_15.4	GBA2	RI	15.2:15.3	15.1	15.4	3.2E-16	0.146	-0.361	4.3E-01	7.3E-02	1.6E-01
THCA	GBP3_AD_5.2_5.1_6	GBP3	AD	5.2	5.1	6	5.0E-14	0.129	-0.626	8.3E-01	3.5E-01	6.5E-01
THCA	GEMIN2_ES_5_4_6	GEMIN2	ES	5	4	6	2.8E-21	0.187	-0.336	5.0E-01	2.7E-01	9.3E-01
THCA	GGT1_AA_15.3_14_15.4	GGT1	AA	15.3	14	15.4	8.7E-19	0.167	-0.293	1.3E-02	5.7E-02	3.7E-01
THCA	GGT1_AA_7.1:7.2_6.2_7.3	GGT1	AA	7.1:7.2	6.2	7.3	6.4E-14	0.128	-0.086	3.9E-01	2.2E-01	1.6E-01
THCA	GGT1_ES_6.2_4_7.3	GGT1	ES	6.2	4	7.3	2.2E-12	0.124	0.157	9.6E-02	2.6E-01	6.2E-01
THCA	GGT1_RI_17.3:17.4_17.2_17.5	GGT1	RI	17.3:17.4	17.2	17.5	2.6E-14	0.130	-0.395	3.8E-01	9.9E-01	1.4E-01
THCA	GIT2_ES_19_18.2_20	GIT2	ES	19	18.2	20	1.1E-17	0.161	-0.178	6.9E-01	9.0E-01	7.5E-01
THCA	GK_ES_23_22_24	GK	ES	23	22	24	1.4E-13	0.127	-0.220	2.1E-02	8.3E-01	5.0E-01
THCA	GLIPR1_RI_5.2_5.1_5.3	GLIPR1	RI	5.2	5.1	5.3	2.2E-21	0.188	-0.524	9.7E-01	7.9E-01	3.3E-01
THCA	GLS2_AA_9.1_8.3_9.2	GLS2	AA	9.1	8.3	9.2	8.9E-17	0.158	-0.530	1.7E-01	3.2E-02	1.5E-01
THCA	GLS2_RI_8.2_8.1_8.3	GLS2	RI	8.2	8.1	8.3	2.3E-14	0.136	-0.455	5.0E-01	3.3E-01	4.1E-01
THCA	GNAS_ES_6:8.1_5_8.2	GNAS	ES	06:08.1	5	8.2	1.5E-20	0.182	0.125	5.2E-02	1.0E-01	6.7E-01
THCA	GNB1L_RI_1.2_1.1_1.3	GNB1L	RI	1.2	1.1	1.3	1.8E-12	0.117	0.067	3.9E-01	1.4E-01	8.4E-02
THCA	GNPDA1_RI_2.5_2.4_2.6	GNPDA1	RI	2.5	2.4	2.6	5.4E-12	0.115	-0.142	3.5E-01	9.8E-01	2.1E-01
THCA	GPM6B_AA_11.1_10_11.2	GPM6B	AA	11.1	10	11.2	4.3E-13	0.119	-0.276	3.2E-01	5.8E-01	5.6E-01
THCA	GPR133_ES_4_3_5	GPR133	ES	4	3	5	4.5E-09	0.108	0.077	1.5E-01	2.4E-01	6.9E-01
THCA	GRK6_AA_17.1_15_17.2	GRK6	AA	17.1	15	17.2	3.4E-16	0.146	0.330	1.4E-01	1.5E-01	3.9E-01
THCA	GSE1_ES_3_2_4	GSE1	ES	3	2	4	1.6E-21	0.206	0.033	9.8E-01	5.5E-01	9.7E-01
THCA	HAUS1_ES_3_2.1_4	HAUS1	ES	3	2.1	4	1.9E-13	0.122	-0.111	5.9E-01	9.4E-02	1.8E-01
THCA	HCFC1R1_AA_3.1_1.5_3.2	HCFC1R1	AA	3.1	1.5	3.2	4.8E-38	0.309	0.173	6.7E-02	1.9E-01	1.4E-02
THCA	HHLA3_ES_4_3_5	HHLA3	ES	4	3	5	2.0E-17	0.156	-0.149	3.0E-01	4.2E-01	9.1E-01
THCA	HIPK3_ES_14_13_15	HIPK3	ES	14	13	15	1.7E-13	0.138	-0.058	5.4E-01	8.8E-01	1.3E-01
THCA	HNRNPC_ES_2.2:2.3:2.4_1_3.2	HNRNPC	ES	2.2:2.3:2.4	1	3.2	4.9E-40	0.323	-0.545	5.2E-01	5.5E-01	6.9E-01
THCA	HNRNPC_ES_2.4:2.5:2.6_1_3.2	HNRNPC	ES	2.4:2.5:2.6	1	3.2	1.9E-44	0.352	-0.425	8.0E-01	5.9E-01	9.0E-01
THCA	HYAL2_RI_1.4_1.3_1.5	HYAL2	RI	1.4	1.3	1.5	1.0E-11	0.109	0.062	1.4E-01	1.4E-01	1.2E-01
THCA	ICAM3_RI_3.2_3.1_3.3	ICAM3	RI	3.2	3.1	3.3	1.9E-56	0.421	-0.560	8.8E-01	2.7E-01	1.6E-01
THCA	IGBP1_RI_1.2_1.1_1.3	IGBP1	RI	1.2	1.1	1.3	2.7E-17	0.164	0.083	4.3E-02	6.5E-01	8.4E-01
THCA	IGFLR1_ES_3:4.1:4.2:4.3_2.2_5	IGFLR1	ES	3:4.1:4.2:4.3	2.2	5	2.1E-12	0.115	-0.433	6.5E-01	3.6E-01	2.0E-01
THCA	IGSF3_ES_6_5_7	IGSF3	ES	6	5	7	2.9E-26	0.261	-0.343	6.5E-01	2.5E-01	6.9E-01
THCA	IL18BP_RI_1.7_1.6_1.8	IL18BP	RI	1.7	1.6	1.8	7.7E-17	0.157	-0.339	4.0E-01	5.5E-01	6.7E-01
THCA	IL32_AD_1.2:1.3:1.4:1.5_1.1_1.9	IL32	AD	1.2:1.3:1.4:1.5	1.1	1.9	1.7E-12	0.116	0.277	4.7E-02	6.6E-02	1.3E-01
THCA	IMMP1L_ES_4:5:6_1_7	IMMP1L	ES	4:05:06	1	7	1.5E-18	0.165	-0.287	7.9E-01	3.3E-01	9.5E-01

cancer type	id	Gene Symbol	splice_type	Exon	From.Exon	To.Exon	anova.p	adj.r2	r	p.50	p.25	p.10
THCA	INCENP_ES_11_10_12	INCENP	ES	11	10	12	8.6E-28	0.254	-0.192	5.7E-01	3.0E-01	3.3E-01
THCA	INO80C_AA_4.1_3_4.2	INO80C	AA	4.1	3	4.2	1.2E-11	0.107	-0.168	9.7E-01	6.5E-01	6.8E-01
THCA	INPP5B_AD_7.2_7.1_8	INPP5B	AD	7.2	7.1	8	3.2E-10	0.118	0.089	7.9E-01	7.7E-01	4.6E-01
THCA	INPP5J_ES_2_1_3.1	INPP5J	ES	2	1	3.1	1.7E-28	0.245	0.399	9.5E-01	4.8E-01	5.6E-01
THCA	INSR_ES_11_10_12	INSR	ES	11	10	12	1.2E-12	0.125	-0.168	2.0E-01	8.5E-01	6.0E-01
THCA	IP6K2_ES_11.4:11.5_11.2_11.9	IP6K2	ES	11.4:11.5	11.2	11.9	2.3E-12	0.113	-0.360	4.3E-02	5.2E-01	5.9E-01
THCA	IPO11_ES_30_29_33	IPO11	ES	30	29	33	9.5E-23	0.206	-0.056	1.1E-01	2.6E-01	3.0E-01
THCA	IRF5_ES_5_4_6.1	IRF5	ES	5	4	6.1	2.1E-26	0.237	-0.510	6.6E-01	3.5E-01	1.1E-01
THCA	ISOC2_ES_3:4.1_2_4.2	ISOC2	ES	03:04.1	2	4.2	1.2E-17	0.159	0.153	3.3E-02	2.7E-02	4.0E-01
THCA	ITGA7_ES_29_28_30	ITGA7	ES	29	28	30	1.5E-25	0.221	-0.175	7.8E-01	5.3E-01	1.9E-01
THCA	ITGAE_ES_28_27_29	ITGAE	ES	28	27	29	8.8E-21	0.184	0.218	3.5E-01	1.8E-01	9.8E-01
THCA	ITGB1BP1_AA_8.1:8.2_7_8.3	ITGB1BP1	AA	8.1:8.2	7	8.3	1.3E-20	0.182	-0.149	7.8E-01	8.6E-01	5.7E-01
THCA	ITGB4_ES_35_34_36	ITGB4	ES	35	34	36	1.3E-40	0.325	-0.620	3.2E-01	5.0E-01	1.9E-01
THCA	IVNS1ABP_ES_9_8_10	IVNS1ABP	ES	9	8	10	8.8E-19	0.168	0.095	8.7E-02	7.4E-01	1.9E-01
THCA	KCTD7_ES_13_12_14	KCTD7	ES	13	12	14	8.8E-18	0.159	-0.050	6.4E-01	5.4E-01	1.1E-01
THCA	KIAA1217_ES_12_11_13	KIAA1217	ES	12	11	13	1.2E-45	0.360	-0.465	8.4E-01	9.1E-01	8.1E-01
THCA	KIAA1468_ES_24_23_26	KIAA1468	ES	24	23	26	1.1E-10	0.122	0.093	1.0E-02	2.7E-01	8.2E-01
THCA	KIAA1468_ME_24|25_23_26	KIAA1468	ME	24|25	23	26	8.5E-36	0.296	-0.109	7.5E-01	2.3E-01	4.1E-01
THCA	KIF12_ES_5_4.1_6	KIF12	ES	5	4.1	6	2.9E-11	0.121	0.602	4.4E-01	6.6E-01	3.0E-01
THCA	KIF13A_ES_28_27_29	KIF13A	ES	28	27	29	4.2E-26	0.237	-0.138	3.8E-01	9.1E-02	5.6E-01
THCA	KRT15_RI_7.2_7.1_7.3	KRT15	RI	7.2	7.1	7.3	1.8E-73	0.507	-0.867	7.6E-01	7.8E-02	4.6E-01
THCA	LCN10_ES_3_2.2_4.2	LCN10	ES	3	2.2	4.2	2.6E-15	0.161	0.459	5.5E-01	4.2E-01	6.7E-01
THCA	LCN10_ES_3:4.2_2.2_5	LCN10	ES	03:04.2	2.2	5	3.5E-16	0.155	0.181	8.7E-01	9.2E-01	2.2E-01
THCA	LCN10_ES_4.2_2.2_5	LCN10	ES	4.2	2.2	5	1.3E-18	0.193	0.188	5.9E-01	6.0E-01	6.2E-01
THCA	LCN12_ES_4_3_5	LCN12	ES	4	3	5	2.5E-29	0.249	0.000	9.5E-01	9.8E-01	8.1E-01
THCA	LEF1_ES_7_6.1_8	LEF1	ES	7	6.1	8	2.7E-10	0.101	0.223	9.2E-01	8.9E-01	3.6E-01
THCA	LETMD1_ES_2:3.2_1.2_7	LETMD1	ES	02:03.2	1.2	7	8.9E-12	0.112	-0.126	5.4E-01	3.5E-01	6.7E-01
THCA	LGALS9_ES_5_4_6	LGALS9	ES	5	4	6	9.0E-24	0.208	0.374	9.5E-01	2.0E-01	5.0E-01
THCA	LGMN_ES_2_1_3	LGMN	ES	2	1	3	3.5E-16	0.146	-0.633	6.8E-01	3.1E-01	6.4E-01
THCA	LMO3_AD_4.2:4.3_4.1_6.3	LMO3	AD	4.2:4.3	4.1	6.3	9.6E-17	0.154	-0.278	5.4E-03	1.7E-02	3.3E-01
THCA	LMO7_AA_19.1_18_19.2	LMO7	AA	19.1	18	19.2	1.1E-18	0.169	0.167	1.1E-02	6.8E-02	7.3E-01
THCA	LMO7_ES_12_9_13	LMO7	ES	12	9	13	5.5E-12	0.116	0.080	9.6E-01	7.5E-01	8.2E-01
THCA	LPIN1_ES_10_9_12	LPIN1	ES	10	9	12	9.9E-11	0.112	-0.146	2.1E-01	9.2E-01	3.9E-01
THCA	LRRC23_ES_7.1:8_6_10	LRRC23	ES	7.1:8	6	10	2.1E-12	0.115	0.044	8.4E-01	7.3E-01	7.1E-01
THCA	LRRC23_ES_8_7.1_10	LRRC23	ES	8	7.1	10	3.0E-22	0.196	0.115	7.6E-01	4.1E-01	1.7E-01
THCA	LRRC23_ES_8:9_7.1_10	LRRC23	ES	8:09	7.1	10	3.5E-25	0.217	0.007	5.7E-02	1.8E-02	1.4E-02
THCA	LRRFIP2_ES_20_19_21	LRRFIP2	ES	20	19	21	5.9E-22	0.194	-0.320	5.3E-01	3.7E-01	4.2E-01
THCA	LSR_ES_4:5_3_6	LSR	ES	4:05	3	6	1.5E-44	0.350	-0.452	7.4E-01	8.7E-01	7.2E-01
THCA	LSR_ES_5_3_6	LSR	ES	5	3	6	8.0E-62	0.450	-0.520	7.7E-01	9.9E-01	8.8E-01
THCA	LUC7L_AD_1.2:1.3_1.1_2.2	LUC7L	AD	1.2:1.3	1.1	2.2	4.3E-13	0.121	0.211	1.7E-01	1.6E-02	8.4E-02

cancer type	id	Gene Symbol	splice_type	Exon	From.Exon	To.Exon	anova.p	adj.r2	r	p.50	p.25	p.10
THCA	LUC7L_ES_1.3_1.1_2.2	LUC7L	ES	1.3	1.1	2.2	1.7E-28	0.251	0.122	3.4E-02	1.5E-02	1.5E-01
THCA	LUC7L_ES_1.3:1.4_1.1_2.2	LUC7L	ES	1.3:1.4	1.1	2.2	2.2E-30	0.257	0.407	4.6E-02	5.2E-02	8.6E-01
THCA	LUC7L_ES_4_3_5	LUC7L	ES	4	3	5	7.5E-31	0.260	0.518	3.6E-01	6.7E-01	5.1E-01
THCA	LUC7L_RI_1.2_1.1_1.3	LUC7L	RI	1.2	1.1	1.3	9.3E-12	0.109	0.057	3.9E-01	3.1E-01	2.0E-01
THCA	LYRM1_ES_5:7_3_8.1	LYRM1	ES	5:07	3	8.1	1.3E-10	0.105	-0.011	4.2E-01	3.3E-01	7.0E-01
THCA	MAD2L2_ES_2:3_1_4	MAD2L2	ES	2:03	1	4	9.7E-13	0.116	-0.567	2.9E-01	4.8E-01	6.3E-01
THCA	MAF_RI_1.2_1.1_1.3	MAF	RI	1.2	1.1	1.3	6.7E-31	0.259	0.147	6.2E-01	4.1E-01	7.6E-01
THCA	MAP2_ES_13_12_14	MAP2	ES	13	12	14	2.2E-10	0.100	-0.297	2.0E-01	3.4E-02	1.6E-01
THCA	MAP3K4_ES_18_17_19	MAP3K4	ES	18	17	19	1.5E-26	0.231	0.224	9.6E-01	2.9E-01	3.1E-01
THCA	MAP4K1_ES_31_30_32	MAP4K1	ES	31	30	32	5.9E-24	0.210	-0.618	7.9E-01	5.0E-01	7.4E-02
THCA	MAP4K4_ES_17_16.2_19	MAP4K4	ES	17	16.2	19	1.2E-13	0.126	0.187	4.9E-01	2.9E-01	7.4E-01
THCA	MAPK10_ES_9_8.2_10	MAPK10	ES	9	8.2	10	1.0E-26	0.244	0.290	1.2E-01	5.1E-01	7.4E-01
THCA	MAPK10_ME_12|13_11_14	MAPK10	ME	12|13	11	14	5.3E-15	0.138	0.217	2.9E-01	1.9E-01	3.0E-01
THCA	MAPK9_ES_3_2_4	MAPK9	ES	3	2	4	2.7E-23	0.209	-0.187	3.5E-01	7.8E-01	2.4E-01
THCA	MAPRE3_AD_5.2_5.1_6	MAPRE3	AD	5.2	5.1	6	1.0E-30	0.260	-0.307	9.4E-01	9.0E-01	1.7E-01
THCA	MAX_RI_5.6:5.7_5.5_5.8	MAX	RI	5.6:5.7	5.5	5.8	5.3E-12	0.110	-0.037	2.3E-01	2.7E-01	9.4E-01
THCA	MBNL1_ES_10_9_11	MBNL1	ES	10	9	11	1.1E-16	0.163	0.389	7.4E-01	3.4E-01	9.7E-01
THCA	MBNL1_ES_8_7_9	MBNL1	ES	8	7	9	2.5E-19	0.185	-0.603	6.1E-01	6.9E-01	5.4E-01
THCA	MEIS3_AA_7.1_6_7.2	MEIS3	AA	7.1	6	7.2	5.8E-18	0.166	0.731	2.1E-01	1.8E-01	8.5E-02
THCA	METTL15_AA_10.1_9_10.2	METTL15	AA	10.1	9	10.2	1.2E-15	0.149	0.005	9.9E-01	9.6E-01	5.0E-01
THCA	MFF_ES_8:9:10_7_11	MFF	ES	8:09:10	7	11	5.4E-40	0.321	0.070	2.8E-01	1.8E-02	5.8E-02
THCA	MFF_ES_9:10_7_11	MFF	ES	9:10	7	11	7.7E-17	0.151	0.061	3.7E-01	2.4E-02	2.1E-01
THCA	MFSD11_AD_2.2_2.1_2.6	MFSD11	AD	2.2	2.1	2.6	4.6E-14	0.145	0.026	1.2E-01	1.6E-01	1.7E-01
THCA	MFSD11_AD_2.2:2.3_2.1_2.6	MFSD11	AD	2.2:2.3	2.1	2.6	2.5E-10	0.109	0.006	1.4E-01	6.9E-02	2.4E-01
THCA	MLH1_AD_1.2_1.1_2.1	MLH1	AD	1.2	1.1	2.1	1.4E-10	0.103	0.199	1.1E-01	1.4E-01	5.4E-02
THCA	MON2_ES_31_30_32	MON2	ES	31	30	32	1.3E-10	0.101	-0.177	2.3E-01	4.2E-01	6.8E-01
THCA	MORF4L2_ES_4:5.1:5.2:5.3_3.2_6.2	MORF4L2	ES	4:5.1:5.2:5.3	3.2	6.2	6.6E-18	0.163	0.007	4.4E-01	2.1E-01	1.7E-02
THCA	MORF4L2_ES_4:5.3_3.2_6.2	MORF4L2	ES	04:05.3	3.2	6.2	5.7E-29	0.252	0.029	2.5E-01	3.0E-02	4.4E-02
THCA	MROH1_ES_26_25.2_27	MROH1	ES	26	25.2	27	2.3E-36	0.300	0.123	5.4E-01	1.3E-01	7.5E-01
THCA	MRPL52_ES_4.1:4.2_2_5	MRPL52	ES	4.1:4.2	2	5	4.2E-18	0.162	-0.107	1.3E-01	4.0E-01	1.7E-01
THCA	MRPL55_ES_1.2:2.2:2.3:2.4:2.5:2.6_1.1_	MRPL55	ES	2:2.2:2.3:2.4:2.5:2	1.1	2.9	3.7E-17	0.154	-0.244	9.3E-01	4.2E-01	8.2E-01
THCA	MRPL55_ES_1.2:2.2:2.3:2.4:2.5:2.6:2.7:2	MRPL55	ES	2:2.3:2.4:2.5:2.6:2	1.1	2.9	6.2E-11	0.101	-0.149	8.3E-01	7.4E-01	5.5E-01
THCA	MRPL55_ES_2.2:2.3:2.4:2.5:2.6_1.2_2.9	MRPL55	ES	2.2:2.3:2.4:2.5:2.6	1.2	2.9	2.1E-11	0.105	-0.167	2.6E-01	5.8E-01	2.0E-01
THCA	MTMR1_ES_4_3_5	MTMR1	ES	4	3	5	6.2E-16	0.177	-0.066	5.4E-01	8.8E-01	1.8E-01
THCA	MVD_ES_3:4_1_5	MVD	ES	3:04	1	5	3.2E-12	0.112	0.306	5.5E-01	2.1E-01	2.0E-01
THCA	MYL6_ES_4.3_4.1_5	MYL6	ES	4.3	4.1	5	2.0E-24	0.212	-0.056	5.6E-01	3.4E-01	5.1E-01
THCA	MYO1B_ES_23_22_24	MYO1B	ES	23	22	24	1.4E-10	0.104	-0.132	9.2E-01	9.6E-01	5.1E-01
THCA	MYO1B_ES_23:24_22_25	MYO1B	ES	23:24	22	25	8.2E-56	0.427	-0.185	9.8E-01	6.2E-01	3.9E-01
THCA	MYO1B_ES_24_22_25	MYO1B	ES	24	22	25	4.4E-32	0.274	-0.129	9.3E-01	5.5E-01	5.1E-01
THCA	MYO5A_ES_35_34_36	MYO5A	ES	35	34	36	3.7E-26	0.253	0.144	6.2E-01	8.6E-01	1.7E-01

cancer type	id	Gene Symbol	splice_type	Exon	From.Exon	To.Exon	anova.p	adj.r2	r	p.50	p.25	p.10
THCA	MYO9B_ES_37_36_38.1	MYO9B	ES	37	36	38.1	8.1E-22	0.217	-0.548	3.6E-01	7.6E-01	3.8E-01
THCA	NAA60_RI_10.4:10.5_10.3_10.6	NAA60	RI	10.4:10.5	10.3	10.6	4.7E-19	0.170	0.059	3.3E-01	5.1E-01	2.0E-01
THCA	NAGA_RI_1.2_1.1_1.3	NAGA	RI	1.2	1.1	1.3	1.1E-11	0.108	0.142	4.3E-01	5.1E-01	8.0E-01
THCA	NASP_ES_9_8_10	NASP	ES	9	8	10	6.4E-18	0.160	0.115	1.5E-01	3.8E-01	3.9E-01
THCA	NDRG2_RI_4.3:4.4_4.2_4.5	NDRG2	RI	4.3:4.4	4.2	4.5	8.5E-11	0.110	-0.391	2.2E-02	3.0E-03	2.3E-02
THCA	NDUFAF6_ES_10_9.2_11	NDUFAF6	ES	10	9.2	11	2.7E-13	0.123	-0.164	9.6E-01	5.7E-01	8.5E-01
THCA	NDUFAF6_ES_9.2:10_9.1_11	NDUFAF6	ES	9.2:10	9.1	11	9.3E-16	0.144	-0.123	2.5E-02	3.6E-02	5.8E-01
THCA	NDUFS7_RI_9.2_9.1_9.3	NDUFS7	RI	9.2	9.1	9.3	4.4E-14	0.128	-0.080	6.8E-01	2.2E-01	3.0E-01
THCA	NDUFV3_ES_3_2_4	NDUFV3	ES	3	2	4	2.3E-16	0.147	-0.582	1.5E-01	2.6E-01	1.8E-01
THCA	NEK1_ES_19_18_20	NEK1	ES	19	18	20	1.2E-12	0.152	0.011	2.9E-01	2.0E-01	3.9E-01
THCA	NF2_ES_16.1_15_17	NF2	ES	16.1	15	17	1.7E-14	0.132	0.231	8.1E-01	1.7E-01	1.4E-01
THCA	NFKBIB_RI_5.2_5.1_5.3	NFKBIB	RI	5.2	5.1	5.3	2.0E-16	0.148	-0.089	5.1E-01	9.1E-01	4.9E-01
THCA	NME4_ES_4_2_5	NME4	ES	4	2	5	3.9E-22	0.194	-0.223	1.4E-02	1.7E-01	4.1E-01
THCA	NMRK1_ES_3_2_4	NMRK1	ES	3	2	4	4.5E-14	0.128	0.076	1.3E-01	1.4E-01	6.4E-01
THCA	NONO_ES_2:3_1_4	NONO	ES	2:03	1	4	1.4E-15	0.145	0.116	7.5E-01	5.5E-01	3.8E-01
THCA	NPIPB4_ES_3.1_2.5_4	NPIPB4	ES	3.1	2.5	4	9.9E-11	0.110	0.324	9.2E-01	4.7E-01	1.7E-01
THCA	NPIPB4_ES_3.3_2.5_4	NPIPB4	ES	3.3	2.5	4	8.4E-23	0.221	0.351	3.6E-01	6.6E-01	4.7E-01
THCA	NPIPB5_ES_3.1_2.5_5	NPIPB5	ES	3.1	2.5	5	5.9E-12	0.116	0.261	5.2E-01	7.6E-01	3.5E-01
THCA	NPIPB5_ES_3.3_2.5_5	NPIPB5	ES	3.3	2.5	5	2.4E-27	0.248	-0.013	6.8E-01	7.2E-01	5.2E-01
THCA	NRCAM_ES_12_11_13	NRCAM	ES	12	11	13	1.1E-22	0.242	0.108	1.7E-01	7.4E-02	3.2E-01
THCA	NSRP1_ES_3_1_8	NSRP1	ES	3	1	8	5.2E-17	0.185	0.069	2.4E-01	3.8E-01	3.5E-01
THCA	NT5C2_ES_5_4_6	NT5C2	ES	5	4	6	1.7E-13	0.126	0.363	7.0E-01	5.0E-01	7.9E-01
THCA	NUBPL_ES_3_2_4	NUBPL	ES	3	2	4	2.2E-20	0.193	-0.394	4.4E-01	1.1E-01	6.2E-01
THCA	NUMA1_ES_18_17_19	NUMA1	ES	18	17	19	3.4E-52	0.396	0.018	2.2E-01	2.6E-01	1.0E-01
THCA	NUMA1_ES_4_3_5	NUMA1	ES	4	3	5	1.1E-12	0.116	-0.160	7.2E-01	5.5E-01	6.2E-01
THCA	NUMB_ES_13_12_14	NUMB	ES	13	12	14	7.3E-19	0.170	0.010	2.2E-01	6.6E-01	3.2E-01
THCA	NVL_ME_11|12_10_13	NVL	ME	11|12	10	13	8.4E-10	0.107	-0.057	1.3E-01	1.5E-01	3.0E-02
THCA	NWD1_AD_7.2_7.1_8	NWD1	AD	7.2	7.1	8	1.4E-18	0.169	-0.566	5.8E-02	2.3E-02	1.6E-01
THCA	NXF1_RI_11.2_11.1_11.3	NXF1	RI	11.2	11.1	11.3	1.2E-13	0.124	0.774	1.8E-02	4.1E-02	2.4E-01
THCA	OAZ1_ES_3.2:3.3:3.4:3.5_1_4	OAZ1	ES	3.2:3.3:3.4:3.5	1	4	1.8E-11	0.106	0.120	8.7E-01	3.7E-01	2.4E-01
THCA	OBSL1_ES_13_12_14.1	OBSL1	ES	13	12	14.1	5.4E-12	0.110	0.297	9.7E-01	7.8E-01	7.6E-01
THCA	OPA1_ES_7_6_8	OPA1	ES	7	6	8	4.4E-22	0.209	-0.023	7.5E-01	6.8E-01	4.9E-01
THCA	ORAOV1_RI_5.2_5.1_5.3	ORAOV1	RI	5.2	5.1	5.3	3.3E-11	0.103	0.350	5.4E-01	4.7E-01	7.2E-01
THCA	OSBPL3_ES_9_8_10	OSBPL3	ES	9	8	10	1.4E-45	0.371	-0.265	7.7E-01	2.9E-01	4.6E-02
THCA	P4HA2_ES_2.1:2.2_1_3	P4HA2	ES	2.1:2.2	1	3	1.6E-31	0.269	0.246	1.3E-01	1.2E-01	2.8E-01
THCA	PALLD_ES_14_12_15	PALLD	ES	14	12	15	2.9E-11	0.111	0.120	6.7E-01	7.1E-01	3.9E-01
THCA	PAM_ES_14_13_15	PAM	ES	14	13	15	2.1E-17	0.157	0.197	5.3E-01	9.1E-01	5.9E-01
THCA	PAM_ES_23.2:25.1:25.2_22_26.1	PAM	ES	23.2:25.1:25.2	22	26.1	6.7E-19	0.170	0.496	2.8E-01	3.8E-01	7.1E-01
THCA	PAM_ES_23.2:25.2_22_26.1	PAM	ES	23.2:25.2	22	26.1	3.7E-19	0.172	0.518	3.5E-01	1.4E-01	7.7E-01
THCA	PARP3_ES_2_1_3.1	PARP3	ES	2	1	3.1	2.4E-23	0.215	-0.079	1.8E-01	6.0E-02	4.1E-01

cancer type	id	Gene Symbol	splice_type	Exon	From.Exon	To.Exon	anova.p	adj.r2	r	p.50	p.25	p.10
THCA	PARP3_ES_2:3.1_1_3.2	PARP3	ES	02:03.1	1	3.2	4.2E-29	0.255	-0.038	5.8E-01	3.9E-01	7.1E-01
THCA	PBX1_ES_11_10_12	PBX1	ES	11	10	12	8.9E-12	0.108	0.199	6.2E-01	7.2E-01	5.9E-01
THCA	PCGF3_RI_5.2_5.1_5.3	PCGF3	RI	5.2	5.1	5.3	2.7E-14	0.131	-0.506	3.5E-01	4.4E-01	7.9E-01
THCA	PCYT2_ES_7_6_8	PCYT2	ES	7	6	8	4.0E-41	0.330	0.037	9.7E-01	6.0E-01	9.7E-01
THCA	PDDC1_ES_4.1:4.2_3_5	PDDC1	ES	4.1:4.2	3	5	4.5E-14	0.155	-0.051	5.5E-01	6.9E-01	8.7E-01
THCA	PDE4DIP_ES_5.2:5.3_4_6.1	PDE4DIP	ES	5.2:5.3	4	6.1	1.5E-12	0.128	-0.366	4.8E-01	4.4E-01	6.2E-01
THCA	PDGFA_ES_6_5_7	PDGFA	ES	6	5	7	9.3E-14	0.126	-0.050	2.7E-01	9.7E-01	9.1E-01
THCA	PFDN5_ES_2_1_4.1	PFDN5	ES	2	1	4.1	7.6E-23	0.202	-0.040	3.2E-01	2.4E-01	2.3E-01
THCA	PFDN5_ES_2_1_4.2	PFDN5	ES	2	1	4.2	6.1E-12	0.112	-0.127	5.9E-01	4.4E-01	9.0E-01
THCA	PFDN5_ES_2:4.1_1_4.2	PFDN5	ES	02:04.1	1	4.2	9.8E-18	0.162	-0.043	9.7E-01	4.7E-01	3.8E-01
THCA	PFDN5_ES_2:4.1:4.2_1_5	PFDN5	ES	2:4.1:4.2	1	5	1.3E-15	0.142	-0.060	7.0E-01	5.7E-01	4.0E-01
THCA	PFKM_AD_16.2:16.3_16.1_17	PFKM	AD	16.2:16.3	16.1	17	1.1E-13	0.127	-0.204	2.5E-01	8.8E-02	4.0E-01
THCA	PGAP2_ES_7_6_10	PGAP2	ES	7	6	10	9.0E-22	0.198	0.178	5.4E-01	9.7E-01	8.0E-01
THCA	PHLDB2_ES_14:15_13_16	PHLDB2	ES	14:15	13	16	1.7E-18	0.173	0.348	4.1E-01	8.0E-01	2.4E-01
THCA	PHYHIP_ES_2_1_3	PHYHIP	ES	2	1	3	2.3E-12	0.130	-0.599	2.8E-01	5.4E-01	4.6E-01
THCA	PI4KA_ES_37:38:39:40:41:42:43_36_44	PI4KA	ES	':38:39:40:41:42:·	36	44	2.7E-15	0.167	0.445	9.6E-01	8.4E-01	9.2E-01
THCA	PICALM_ES_14.1:14.2_13_15	PICALM	ES	14.1:14.2	13	15	1.7E-12	0.115	0.294	4.1E-01	6.7E-01	4.4E-01
THCA	PILRA_ES_3_2_4	PILRA	ES	3	2	4	5.5E-15	0.136	-0.304	8.1E-01	2.4E-01	7.8E-01
THCA	PKP2_ES_6_5_7	PKP2	ES	6	5	7	1.9E-16	0.168	-0.554	9.3E-01	7.0E-01	9.5E-01
THCA	PLA2G6_ES_14_13_15	PLA2G6	ES	14	13	15	1.0E-11	0.109	0.335	8.3E-01	8.9E-01	2.1E-01
THCA	PLCD4_ES_13_12_14	PLCD4	ES	13	12	14	1.9E-26	0.282	-0.659	4.9E-03	2.8E-04	5.7E-02
THCA	PLD3_ES_1.2:4:5.1_1.1_5.2	PLD3	ES	1.2:4:5.1	1.1	5.2	2.3E-11	0.115	0.223	3.9E-01	8.0E-01	8.3E-01
THCA	PLD3_ES_3_1.2_5.2	PLD3	ES	3	1.2	5.2	1.6E-14	0.135	-0.498	8.2E-01	4.4E-02	5.7E-02
THCA	PLD3_ES_4:5.1_1.2_5.2	PLD3	ES	04:05.1	1.2	5.2	6.6E-12	0.112	0.175	9.9E-01	6.5E-01	3.3E-01
THCA	POLL_ES_1.2:1.3:1.5_1.1_3	POLL	ES	1.2:1.3:1.5	1.1	3	1.5E-11	0.126	-0.060	8.3E-01	7.2E-01	3.4E-01
THCA	POLR2J3_ES_4.1_2_4.3	POLR2J3	ES	4.1	2	4.3	6.1E-13	0.118	0.306	9.0E-01	7.7E-01	8.6E-01
THCA	POLR2J3_ES_6:7_4.3_8	POLR2J3	ES	6:07	4.3	8	3.2E-58	0.430	0.661	8.7E-01	9.3E-01	1.6E-01
THCA	PPFIBP1_ES_11_10_12	PPFIBP1	ES	11	10	12	2.6E-26	0.252	-0.219	4.8E-02	3.2E-01	7.8E-01
THCA	PPIL3_ES_1.2:2.2:3:4.1_1.1_4.2	PPIL3	ES	1.2:2.2:3:4.1	1.1	4.2	1.2E-10	0.108	0.172	7.2E-01	6.3E-01	9.9E-01
THCA	PPIL3_ES_2.2:3:4.1_1.1_4.2	PPIL3	ES	2.2:3:4.1	1.1	4.2	1.5E-12	0.128	0.174	5.9E-02	5.5E-01	6.6E-01
THCA	PPIP5K1_ES_31_30_32	PPIP5K1	ES	31	30	32	2.8E-14	0.130	-0.505	2.7E-01	4.1E-01	7.9E-01
THCA	PPIP5K2_ES_28:29_27_30	PPIP5K2	ES	28:29:00	27	30	1.2E-12	0.120	-0.018	6.0E-01	4.2E-01	4.4E-01
THCA	PPP1CA_ES_2.1:2.2_1_3	PPP1CA	ES	2.1:2.2	1	3	2.1E-11	0.105	0.135	3.5E-01	1.9E-01	6.3E-02
THCA	PPP1R7_AD_1.2:1.3_1.1_3	PPP1R7	AD	1.2:1.3	1.1	3	1.9E-15	0.152	-0.497	8.6E-01	8.9E-01	8.7E-01
THCA	PPP2R4_ES_4:5:6:7:8:9:10_3.1_15.1	PPP2R4	ES	4:5:6:7:8:9:10	3.1	15.1	4.2E-13	0.121	-0.289	4.8E-01	3.3E-01	6.8E-01
THCA	PPP2R4_ES_6:7:8:9:10_3.1_15.1	PPP2R4	ES	6:7:8:9:10	3.1	15.1	7.7E-11	0.113	-0.174	6.1E-01	8.1E-01	5.0E-01
THCA	PPRC1_ES_8:9_7_10	PPRC1	ES	8:09	7	10	1.7E-31	0.264	0.267	4.4E-01	9.5E-01	2.4E-01
THCA	PRDM16_ES_17_16_18.1	PRDM16	ES	17	16	18.1	1.9E-10	0.102	0.226	2.9E-01	2.1E-01	9.1E-01
THCA	PRKAG1_ES_3:4.1_2_4.2	PRKAG1	ES	03:04.1	2	4.2	2.4E-11	0.111	-0.130	3.8E-01	2.3E-01	3.8E-01
THCA	PRKDC_ES_81_80_82	PRKDC	ES	81	80	82	5.9E-15	0.137	-0.176	2.0E-01	1.2E-01	6.8E-01

cancer type	id	Gene Symbol	splice_type	Exon	From.Exon	To.Exon	anova.p	adj.r2	r	p.50	p.25	p.10
THCA	PRMT2_ES_1.3_1.1_2	PRMT2	ES	1.3	1.1	2	2.2E-15	0.139	-0.321	7.0E-01	3.5E-01	6.3E-01
THCA	PSMC3IP_RI_1.2_1.1_1.3	PSMC3IP	RI	1.2	1.1	1.3	1.1E-10	0.105	-0.170	1.8E-01	6.3E-01	1.3E-01
THCA	PSTPIP1_ES_3_2.2_5	PSTPIP1	ES	3	2.2	5	8.0E-35	0.290	-0.763	3.9E-01	4.6E-01	1.8E-01
THCA	PTBP2_ES_12_11.2_13	PTBP2	ES	12	11.2	13	4.2E-18	0.182	0.216	2.1E-02	1.5E-01	1.2E-01
THCA	PTK2_ES_39.2_37_39.5	PTK2	ES	39.2	37	39.5	2.3E-26	0.228	-0.088	1.8E-01	4.7E-01	2.4E-01
THCA	PXN_AA_14.1_9_14.2	PXN	AA	14.1	9	14.2	5.2E-15	0.138	0.163	7.5E-01	8.8E-01	6.0E-01
THCA	RAB3GAP1_ES_4:5_3.1_6	RAB3GAP1	ES	4:05	3.1	6	2.6E-11	0.104	-0.789	3.5E-01	6.6E-01	8.1E-01
THCA	RABEP2_ES_3_2.2_4	RABEP2	ES	3	2.2	4	6.2E-16	0.144	0.245	5.7E-01	4.8E-01	6.1E-01
THCA	RABGGTA_RI_1.2:1.3_1.1_1.4	RABGGTA	RI	1.2:1.3	1.1	1.4	3.3E-27	0.233	-0.009	5.7E-01	9.7E-01	9.9E-01
THCA	RAI14_ES_18_17_19	RAI14	ES	18	17	19	3.0E-14	0.139	0.394	5.6E-01	4.4E-01	1.0E+00
THCA	RALGPS2_ES_15_14_16	RALGPS2	ES	15	14	16	1.2E-20	0.209	-0.138	2.6E-01	9.1E-01	9.9E-01
THCA	RANGRF_AD_3.2:3.3_3.1_3.5	RANGRF	AD	3.2:3.3	3.1	3.5	1.3E-15	0.141	-0.048	4.8E-01	6.2E-01	8.8E-01
THCA	RANGRF_RI_3.4_3.3_3.5	RANGRF	RI	3.4	3.3	3.5	3.1E-35	0.290	-0.048	7.1E-01	9.0E-01	6.6E-02
THCA	RAPGEF2_ES_17_16_18	RAPGEF2	ES	17	16	18	8.6E-19	0.186	0.421	6.1E-01	9.1E-02	1.0E+00
THCA	REPIN1_ES_4.1:4.2:5.2_3.2_5.3	REPIN1	ES	4.1:4.2:5.2	3.2	5.3	2.9E-10	0.101	0.039	5.4E-01	8.5E-01	5.9E-01
THCA	REPS1_ES_9.3_9.1_10	REPS1	ES	9.3	9.1	10	3.2E-13	0.121	0.039	8.6E-01	9.5E-01	4.7E-01
THCA	RGS12_ES_19_18_20.1	RGS12	ES	19	18	20.1	8.3E-15	0.134	-0.072	7.1E-01	8.3E-01	9.2E-01
THCA	RNF146_ES_3:5.1_2_6	RNF146	ES	03:05.1	2	6	1.6E-14	0.134	0.170	8.6E-01	4.9E-01	5.6E-01
THCA	RNF216_ES_4:5.1:5.2:6.2_2_7	RNF216	ES	4:5.1:5.2:6.2	2	7	9.6E-11	0.102	0.262	7.4E-01	9.5E-01	3.4E-01
THCA	RNH1_AA_4.2_3_4.3	RNH1	AA	4.2	3	4.3	1.6E-17	0.157	0.341	3.6E-02	5.8E-02	3.5E-01
THCA	RNH1_ES_3_1_4.2	RNH1	ES	3	1	4.2	8.9E-18	0.160	0.265	6.5E-01	6.5E-01	6.4E-01
THCA	RNH1_ES_3_2_4.3	RNH1	ES	3	2	4.3	2.0E-13	0.123	0.115	9.0E-01	6.4E-01	3.3E-01
THCA	RNH1_ES_3:4.2_1_4.3	RNH1	ES	03:04.2	1	4.3	6.0E-11	0.101	0.207	8.7E-01	9.5E-01	5.2E-01
THCA	RNH1_ES_3:4.2_2_4.3	RNH1	ES	03:04.2	2	4.3	6.5E-18	0.160	0.240	6.8E-01	9.7E-01	4.1E-01
THCA	RPL19_ES_1.2:1.3:2.1_1.1_2.2	RPL19	ES	1.2:1.3:2.1	1.1	2.2	2.3E-15	0.139	-0.204	8.2E-01	6.2E-01	3.0E-01
THCA	RPL21_AD_1.3:1.4_1.2_2	RPL21	AD	1.3:1.4	1.2	2	3.9E-11	0.102	0.016	7.6E-01	6.5E-01	7.7E-01
THCA	RPL21_AD_1.4_1.3_2	RPL21	AD	1.4	1.3	2	1.5E-11	0.107	-0.017	9.9E-01	9.0E-01	6.6E-01
THCA	RPRD2_ES_4_3.1_5	RPRD2	ES	4	3.1	5	3.0E-16	0.155	-0.025	9.7E-01	2.6E-01	6.6E-01
THCA	RPS25_ES_2.1:2.2_1_3.1	RPS25	ES	2.1:2.2	1	3.1	1.5E-12	0.118	-0.161	8.8E-01	7.8E-01	3.2E-01
THCA	RREB1_ES_12_11_13	RREB1	ES	12	11	13	5.7E-12	0.118	-0.261	3.6E-01	1.3E-01	8.3E-01
THCA	RRN3_ES_12:13_11.1_14	RRN3	ES	12:13	11.1	14	1.7E-12	0.119	0.498	9.1E-01	8.6E-01	3.2E-01
THCA	S100A6_ES_2_1.1_3.2	S100A6	ES	2	1.1	3.2	1.9E-52	0.483	0.683	8.7E-01	6.2E-01	3.0E-01
THCA	SAE1_AD_1.2:1.3_1.1_2	SAE1	AD	1.2:1.3	1.1	2	4.9E-11	0.113	0.129	7.5E-01	9.2E-01	8.2E-01
THCA	SBF1_ES_29_28_30	SBF1	ES	29	28	30	4.5E-19	0.171	0.059	5.7E-01	3.9E-01	1.0E-01
THCA	SCNN1A_ES_10_9_11	SCNN1A	ES	10	9	11	5.5E-33	0.276	-0.640	6.9E-01	7.2E-01	5.9E-01
THCA	SCRIB_ES_17_16_18	SCRIB	ES	17	16	18	4.1E-17	0.156	-0.240	3.1E-01	4.1E-01	1.6E-01
THCA	SCRIB_ES_36_35_37	SCRIB	ES	36	35	37	5.8E-15	0.136	-0.042	8.1E-01	3.2E-01	6.4E-01
THCA	SCRN2_RI_7.2_7.1_7.3	SCRN2	RI	7.2	7.1	7.3	4.7E-11	0.101	-0.063	2.7E-01	4.2E-02	1.7E-02
THCA	SEC31A_ES_16:17_15_18	SEC31A	ES	16:17	15	18	7.5E-15	0.136	-0.061	7.1E-01	7.6E-01	9.2E-01
THCA	SEC31A_ES_26.1:26.2_25.1_28	SEC31A	ES	26.1:26.2	25.1	28	2.5E-14	0.131	-0.032	7.2E-03	5.0E-03	5.0E-01

cancer type	id	Gene Symbol	splice_type	Exon	From.Exon	To.Exon	anova.p	adj.r2	r	p.50	p.25	p.10
THCA	SEC31A_ES_26.1:26.2:27_25.1_28	SEC31A	ES	26.1:26.2:27	25.1	28	1.5E-21	0.190	0.020	9.2E-01	8.2E-01	9.4E-01
THCA	SEC31A_ES_26.1:27_25.1_28	SEC31A	ES	26.1:27	25.1	28	4.7E-35	0.294	0.054	8.8E-01	9.7E-01	2.1E-01
THCA	SEC31A_ES_26.2:27_26.1_28	SEC31A	ES	26.2:27	26.1	28	1.8E-28	0.243	0.046	3.7E-01	1.0E-01	1.5E-01
THCA	SEC31A_ES_27_26.1_28	SEC31A	ES	27	26.1	28	5.5E-49	0.382	0.069	2.3E-01	5.6E-02	7.2E-02
THCA	SEC31A_ES_27_26.2_28	SEC31A	ES	27	26.2	28	7.2E-46	0.361	0.079	1.1E-01	1.5E-01	3.1E-01
THCA	SEPT8_AA_12.1_11_12.2	SEPT8	AA	12.1	11	12.2	9.6E-16	0.143	-0.224	5.0E-01	5.8E-01	3.4E-01
THCA	SERPINA1_AA_2.1:2.2:2.3_1.1_2.4	SERPINA1	AA	2.1:2.2:2.3	1.1	2.4	1.8E-38	0.333	0.683	3.2E-02	2.6E-03	7.9E-02
THCA	SERPINA1_ES_2.1_1.1_2.4	SERPINA1	ES	2.1	1.1	2.4	4.0E-14	0.153	0.416	4.1E-02	2.0E-01	2.3E-01
THCA	SERPINA1_ES_2.1:2.2:2.3:2.4:2.5_1.1_3.	SERPINA1	ES	.1:2.2:2.3:2.4:2.	1.1	3.2	4.2E-110	0.654	0.884	3.9E-01	3.4E-01	2.3E-01
THCA	SERPINA1_ES_2.4:2.5_1.1_3.2	SERPINA1	ES	2.4:2.5	1.1	3.2	4.9E-21	0.188	0.426	6.5E-01	9.5E-01	6.4E-01
THCA	SERPINA1_ES_2.5_1.1_3.2	SERPINA1	ES	2.5	1.1	3.2	7.7E-16	0.145	0.366	1.8E-01	4.3E-01	4.9E-01
THCA	SH3BP1_ES_16_15_17.2	SH3BP1	ES	16	15	17.2	6.1E-49	0.377	-0.748	3.5E-02	9.9E-01	3.4E-01
THCA	SKA2_ES_2_1.1_5	SKA2	ES	2	1.1	5	1.5E-20	0.182	-0.019	5.9E-01	9.4E-01	8.1E-02
THCA	SKA2_ES_2:4.1_1.1_5	SKA2	ES	02:04.1	1.1	5	5.4E-16	0.145	-0.002	6.2E-01	8.2E-01	1.3E-01
THCA	SLA_ES_4_3.2_5	SLA	ES	4	3.2	5	8.1E-55	0.498	-0.137	5.8E-01	2.4E-01	4.1E-02
THCA	SLAIN2_ES_8_6_9	SLAIN2	ES	8	6	9	1.8E-30	0.258	0.263	3.1E-01	2.5E-01	2.6E-01
THCA	SLC25A36_ES_6.1:6.2_4.1_7	SLC25A36	ES	6.1:6.2	4.1	7	1.7E-23	0.223	0.059	2.0E-01	3.8E-01	9.9E-01
THCA	SLC25A37_AA_3.1_2.1_3.2	SLC25A37	AA	3.1	2.1	3.2	1.5E-16	0.151	0.659	5.3E-01	6.4E-01	7.4E-01
THCA	SLC25A37_AD_2.2_2.1_3.2	SLC25A37	AD	2.2	2.1	3.2	1.1E-29	0.251	0.598	9.1E-01	9.8E-01	9.5E-01
THCA	SLK_ES_13_12_14	SLK	ES	13	12	14	3.2E-15	0.139	0.160	1.3E-01	3.3E-01	6.0E-01
THCA	SMAGP_ES_5_3.3_6.1	SMAGP	ES	5	3.3	6.1	2.8E-11	0.104	-0.444	4.6E-01	8.8E-01	5.8E-01
THCA	SMURF2_ES_4_3_5	SMURF2	ES	4	3	5	9.8E-12	0.109	-0.628	4.9E-01	9.8E-01	9.2E-01
THCA	SNAPC3_RI_9.2_9.1_9.3	SNAPC3	RI	9.2	9.1	9.3	4.1E-28	0.239	0.082	7.0E-01	3.1E-01	4.4E-03
THCA	SNAPC5_ES_1.2:2.1_1.1_3.1	SNAPC5	ES	1.2:2.1	1.1	3.1	5.9E-17	0.155	-0.195	6.4E-01	1.0E+00	7.2E-01
THCA	SNCA_AD_2.3_2.2_4.2	SNCA	AD	2.3	2.2	4.2	1.2E-11	0.127	0.224	4.5E-03	1.5E-01	2.6E-02
THCA	SNUPN_AD_1.2_1.1_4	SNUPN	AD	1.2	1.1	4	1.4E-12	0.116	-0.068	6.4E-01	5.3E-01	6.5E-01
THCA	SNX14_ES_16_15_17	SNX14	ES	16	15	17	9.2E-20	0.177	-0.148	6.7E-01	3.8E-01	8.7E-01
THCA	SPAG9_ES_30_29_31	SPAG9	ES	30	29	31	1.4E-15	0.142	-0.366	9.1E-01	9.6E-01	3.4E-01
THCA	SPTAN1_ES_38_36.1_39	SPTAN1	ES	38	36.1	39	8.7E-41	0.328	0.272	2.4E-02	1.6E-01	1.3E-01
THCA	SRSF4_ES_2:3:4_1_5	SRSF4	ES	2:03:04	1	5	1.9E-11	0.106	0.175	9.5E-02	2.0E-01	5.5E-01
THCA	SRSF4_ES_6_5_7	SRSF4	ES	6	5	7	1.5E-18	0.167	0.254	4.4E-01	5.8E-01	7.4E-02
THCA	SSBP4_ES_7_6_8	SSBP4	ES	7	6	8	1.2E-46	0.363	0.016	7.2E-01	8.6E-01	7.3E-01
THCA	ST7_ES_10_9_11.1	ST7	ES	10	9	11.1	1.3E-15	0.143	-0.354	2.5E-01	1.0E-01	6.1E-01
THCA	STAG3_ES_13_12_14	STAG3	ES	13	12	14	1.3E-13	0.132	-0.023	6.9E-01	7.0E-01	1.9E-01
THCA	STRA6_AD_18.2_18.1_19	STRA6	AD	18.2	18.1	19	1.5E-81	0.544	-0.909	7.8E-01	8.6E-01	1.9E-01
THCA	STRADA_ES_3:4_2.2_6	STRADA	ES	3:04	2.2	6	2.0E-09	0.104	-0.092	2.7E-02	5.1E-02	4.6E-02
THCA	STRADA_ES_4_3_6	STRADA	ES	4	3	6	1.1E-10	0.113	-0.066	6.4E-01	7.9E-01	1.2E-01
THCA	STRADA_RI_12.4_12.3_12.5	STRADA	RI	12.4	12.3	12.5	7.1E-12	0.109	0.255	7.6E-01	8.9E-01	5.0E-01
THCA	STRADA_RI_12.6_12.5_12.7	STRADA	RI	12.6	12.5	12.7	1.0E-12	0.116	0.332	6.1E-01	9.1E-01	8.2E-01
THCA	STRADB_AA_12.1_11_12.2	STRADB	AA	12.1	11	12.2	2.0E-11	0.106	0.242	9.8E-01	8.1E-01	6.4E-01

cancer type	id	Gene Symbol	splice_type	Exon	From.Exon	To.Exon	anova.p	adj.r2	r	p.50	p.25	p.10
THCA	SUGT1_ES_7_6_8	SUGT1	ES	7	6	8	3.6E-13	0.120	-0.060	1.8E-01	3.0E-01	3.5E-01
THCA	SULF2_ES_20_19.2_21	SULF2	ES	20	19.2	21	1.6E-29	0.250	-0.413	9.4E-03	5.4E-02	8.5E-01
THCA	SULT1A2_RI_1.2:1.3_1.1_1.4	SULT1A2	RI	1.2:1.3	1.1	1.4	1.5E-13	0.124	-0.416	7.4E-01	7.2E-01	1.4E-01
THCA	SUPT20H_AA_21.1_20_21.2	SUPT20H	AA	21.1	20	21.2	4.3E-13	0.119	0.104	6.0E-01	9.9E-01	8.6E-01
THCA	SUPT20H_AA_21.2_20_21.3	SUPT20H	AA	21.2	20	21.3	7.1E-11	0.101	0.036	4.7E-01	6.5E-01	6.1E-01
THCA	SYK_ES_9_8_10	SYK	ES	9	8	10	2.5E-17	0.157	-0.218	8.1E-01	4.8E-01	6.1E-01
THCA	SYNE1_ES_143_142_144	SYNE1	ES	143	142	144	9.6E-22	0.199	-0.493	4.3E-01	2.1E-01	2.1E-01
THCA	SYNE1_ES_151_150_152	SYNE1	ES	151	150	152	1.9E-26	0.229	-0.407	8.9E-01	5.7E-01	4.3E-01
THCA	SYNE4_ES_2_1_3	SYNE4	ES	2	1	3	1.4E-13	0.124	-0.220	8.0E-01	7.0E-01	7.1E-01
THCA	SYNJ2_AD_19.3_19.2_20	SYNJ2	AD	19.3	19.2	20	1.8E-13	0.123	-0.770	4.9E-01	2.0E-01	1.8E-01
THCA	SYTL2_AD_8.3_8.2_9	SYTL2	AD	8.3	8.2	9	7.7E-21	0.191	0.225	4.8E-01	9.5E-01	1.9E-01
THCA	SYTL2_ES_11.2_10.3_12.2	SYTL2	ES	11.2	10.3	12.2	6.9E-35	0.333	-0.556	7.9E-01	6.8E-01	2.3E-01
THCA	SYTL2_ES_13_12.2_14	SYTL2	ES	13	12.2	14	7.7E-27	0.249	0.398	6.0E-01	9.7E-01	8.2E-01
THCA	TACC2_ES_12_11_13	TACC2	ES	12	11	13	8.6E-15	0.134	-0.632	9.1E-01	5.5E-01	1.6E-01
THCA	TBC1D10C_AA_6.1_5_6.2	TBC1D10C	AA	6.1	5	6.2	1.7E-10	0.100	0.051	3.0E-01	4.5E-01	5.9E-02
THCA	TBC1D15_ES_10_9_11	TBC1D15	ES	10	9	11	1.9E-31	0.272	0.221	3.2E-01	2.0E-01	4.3E-01
THCA	TBC1D23_ES_15_14_16	TBC1D23	ES	15	14	16	5.2E-14	0.132	-0.092	1.0E-01	9.0E-01	4.0E-01
THCA	TCF20_AA_4.1_3_4.2	TCF20	AA	4.1	3	4.2	5.7E-12	0.110	0.102	9.4E-01	3.6E-01	1.5E-01
THCA	TCF20_ES_4.1:4.2_3_5	TCF20	ES	4.1:4.2	3	5	3.7E-38	0.310	0.171	5.4E-01	5.5E-01	3.2E-01
THCA	TCF20_ES_4.2_3_5	TCF20	ES	4.2	3	5	3.5E-14	0.129	0.115	8.6E-01	5.4E-01	2.2E-01
THCA	TCF7_ES_6_5_7	TCF7	ES	6	5	7	4.6E-14	0.142	0.008	6.8E-01	8.6E-01	8.4E-01
THCA	TERF1_ES_7_6_8	TERF1	ES	7	6	8	1.9E-11	0.106	0.132	5.4E-01	8.4E-01	2.8E-01
THCA	TFIP11_ES_3_2.2_4	TFIP11	ES	3	2.2	4	8.6E-13	0.122	-0.200	1.9E-01	2.3E-01	9.0E-01
THCA	THEMIS_ES_6_5_7	THEMIS	ES	6	5	7	5.1E-19	0.219	-0.692	7.6E-01	4.6E-01	6.4E-01
THCA	THEMIS2_ES_3_2_5	THEMIS2	ES	3	2	5	2.7E-10	0.101	0.024	6.8E-01	9.5E-01	9.6E-01
THCA	THEMIS2_ES_3:4.1:4.2:4.3_2_5	THEMIS2	ES	3:4.1:4.2:4.3	2	5	7.0E-14	0.127	0.164	5.3E-01	5.4E-02	5.7E-01
THCA	THUMPD2_ES_4_3_5	THUMPD2	ES	4	3	5	5.3E-12	0.116	0.252	9.1E-01	6.2E-01	1.8E-01
THCA	TIA1_ES_6_5_7	TIA1	ES	6	5	7	5.3E-13	0.120	0.415	2.0E-01	4.3E-01	3.0E-01
THCA	TIMM17B_AA_4.1:4.2_3_4.3	TIMM17B	AA	4.1:4.2	3	4.3	5.0E-13	0.119	-0.305	8.0E-01	6.5E-01	4.8E-01
THCA	TIMM8B_ES_2_1_3	TIMM8B	ES	2	1	3	3.8E-19	0.170	-0.478	9.7E-01	9.1E-01	6.1E-01
THCA	TMC6_AD_14.2:14.3_14.1_15	TMC6	AD	14.2:14.3	14.1	15	4.7E-23	0.208	-0.396	2.5E-01	9.0E-01	9.4E-01
THCA	TMEM107_ES_2:3.1:3.2:3.4:3.5_1_3.7	TMEM107	ES	2:3.1:3.2:3.4:3.5	1	3.7	7.6E-11	0.100	0.059	5.7E-01	8.4E-01	8.7E-01
THCA	TMEM107_ES_2:3.2:3.4_1_3.7	TMEM107	ES	2:3.2:3.4	1	3.7	6.2E-20	0.178	0.106	1.7E-01	2.8E-01	1.0E+00
THCA	TMEM107_ES_2:3.2:3.4:3.5_1_3.7	TMEM107	ES	2:3.2:3.4:3.5	1	3.7	7.2E-35	0.287	0.077	9.3E-01	9.9E-01	1.8E-01
THCA	TMEM116_ES_3_2_4	TMEM116	ES	3	2	4	7.1E-12	0.117	0.251	2.9E-02	4.1E-02	8.9E-03
THCA	TMEM126B_ES_3_1_4	TMEM126B	ES	3	1	4	3.9E-26	0.225	-0.151	3.5E-01	8.6E-02	1.4E-01
THCA	TMEM168_ES_2:3_1_4	TMEM168	ES	2:03	1	4	6.2E-10	0.116	0.063	2.1E-01	4.5E-02	1.5E-01
THCA	TMEM180_ES_6_5_7	TMEM180	ES	6	5	7	1.1E-35	0.293	0.139	3.1E-01	9.2E-01	9.7E-01
THCA	TMEM205_AD_2.2:2.3_2.1_2.6	TMEM205	AD	2.2:2.3	2.1	2.6	2.9E-11	0.104	-0.290	8.0E-01	7.2E-01	2.4E-01
THCA	TMEM33_RI_8.2_8.1_8.3	TMEM33	RI	8.2	8.1	8.3	1.8E-12	0.114	0.654	3.1E-01	2.8E-01	4.2E-01

cancer type	id	Gene Symbol	splice_type	Exon	From.Exon	To.Exon	anova.p	adj.r2	r	p.50	p.25	p.10
THCA	TMEM62_ES_5:6_4_7	TMEM62	ES	5:06	4	7	1.3E-10	0.123	0.321	4.9E-01	8.1E-01	5.5E-01
THCA	TMUB2_AA_4.2_3_4.3	TMUB2	AA	4.2	3	4.3	1.3E-10	0.101	-0.115	9.1E-02	6.3E-01	5.1E-01
THCA	TMUB2_AD_2.3:2.4:2.5_2.2_4.3	TMUB2	AD	2.3:2.4:2.5	2.2	4.3	7.8E-11	0.101	0.225	5.8E-01	5.8E-01	4.2E-02
THCA	TMUB2_ES_2.3:2.4:2.5:3_2.2_4.3	TMUB2	ES	2.3:2.4:2.5:3	2.2	4.3	2.1E-18	0.170	0.237	7.5E-01	4.5E-01	2.2E-01
THCA	TNC_ES_12:13:14:15_11_16	TNC	ES	12:13:14:15	11	16	1.1E-19	0.199	-0.338	1.3E-01	6.9E-01	2.9E-01
THCA	TNC_ES_19_11_20	TNC	ES	19	11	20	6.2E-10	0.112	0.219	7.7E-01	1.3E-01	7.4E-01
THCA	TNIK_ES_22_21.1_23	TNIK	ES	22	21.1	23	1.1E-09	0.105	-0.171	8.5E-01	9.8E-01	5.2E-01
THCA	TNRC6A_ES_7_6_8	TNRC6A	ES	7	6	8	4.0E-10	0.103	0.274	7.3E-01	8.6E-01	5.1E-01
THCA	TOP3B_ES_6_5_7	TOP3B	ES	6	5	7	2.2E-11	0.111	0.315	3.6E-01	7.9E-01	6.3E-01
THCA	TP53I3_RI_1.2_1.1_1.3	TP53I3	RI	1.2	1.1	1.3	3.4E-18	0.162	-0.105	7.0E-01	7.2E-01	9.3E-01
THCA	TPD52L1_ES_8:9.1_6_10	TPD52L1	ES	08:09.1	6	10	7.7E-15	0.134	0.348	1.6E-02	2.5E-01	9.7E-01
THCA	TPM2_ES_6_5_7	TPM2	ES	6	5	7	2.7E-23	0.244	-0.276	9.7E-01	4.6E-01	2.6E-01
THCA	TSTD1_ES_2.2:2.3_1_3.1	TSTD1	ES	2.2:2.3	1	3.1	5.1E-41	0.328	0.181	2.7E-01	2.3E-01	7.9E-02
THCA	TUBB3_ES_6.1:6.2_5.4_7.1	TUBB3	ES	6.1:6.2	5.4	7.1	3.5E-62	0.488	-0.770	3.8E-01	9.5E-01	1.3E-01
THCA	TUSC3_ES_11_10_12	TUSC3	ES	11	10	12	5.5E-21	0.186	0.038	2.9E-01	1.4E-01	7.0E-02
THCA	UAP1_ES_9.2_8_10	UAP1	ES	9.2	8	10	8.7E-15	0.136	0.092	8.1E-03	3.2E-02	2.9E-01
THCA	ULK3_RI_6.2_6.1_6.3	ULK3	RI	6.2	6.1	6.3	8.0E-12	0.109	0.707	9.2E-01	3.6E-01	2.4E-01
THCA	UNC5B_ES_8_7_9	UNC5B	ES	8	7	9	1.4E-40	0.375	0.383	4.6E-01	6.3E-01	1.6E-01
THCA	UPF3B_ES_8_7_9	UPF3B	ES	8	7	9	1.2E-12	0.116	0.052	2.8E-01	1.4E-01	9.3E-01
THCA	WSB1_RI_5.2_5.1_5.3	WSB1	RI	5.2	5.1	5.3	1.1E-31	0.270	-0.342	4.0E-01	3.7E-02	8.5E-01
THCA	XRRA1_AA_18.1:18.2_17_18.3	XRRA1	AA	18.1:18.2	17	18.3	8.8E-14	0.127	-0.097	7.3E-01	5.1E-01	5.9E-02
THCA	XRRA1_RI_18.2_18.1_18.3	XRRA1	RI	18.2	18.1	18.3	8.9E-14	0.126	-0.045	1.6E-01	7.4E-01	1.8E-01
THCA	YBX3_ES_6_5_7	YBX3	ES	6	5	7	3.5E-18	0.162	-0.106	1.4E-01	4.4E-01	9.3E-01
THCA	YIPF1_ES_3_2_4	YIPF1	ES	3	2	4	1.1E-13	0.126	0.360	1.1E-01	3.4E-01	9.7E-01
THCA	YPEL4_RI_1.4:1.5_1.3_1.6	YPEL4	RI	1.4:1.5	1.3	1.6	1.0E-12	0.149	-0.466	1.2E-02	1.7E-03	7.0E-02
THCA	ZBTB8OS_ES_2:3:4_1_6	ZBTB8OS	ES	2:03:04	1	6	3.6E-14	0.139	-0.147	3.6E-01	1.4E-01	5.2E-01
THCA	ZC2HC1C_AD_2.2:2.3_2.1_3	ZC2HC1C	AD	2.2:2.3	2.1	3	7.9E-14	0.126	-0.356	9.4E-01	7.5E-01	2.3E-01
THCA	ZFAND1_ES_3_2_4	ZFAND1	ES	3	2	4	1.5E-25	0.221	-0.262	5.0E-01	3.9E-01	5.5E-02
THCA	ZFAND4_ES_11_10_12	ZFAND4	ES	11	10	12	2.2E-09	0.108	0.429	1.4E-01	3.7E-01	8.3E-01
THCA	ZGLP1_RI_1.4_1.3_1.5	ZGLP1	RI	1.4	1.3	1.5	2.9E-15	0.146	-0.609	7.5E-01	8.0E-01	1.5E-01
THCA	ZNF185_ES_15_14_16	ZNF185	ES	15	14	16	1.7E-12	0.124	0.006	2.5E-01	9.6E-01	5.0E-01
THCA	ZNF580_RI_2.2_2.1_2.3	ZNF580	RI	2.2	2.1	2.3	6.2E-11	0.101	0.118	3.6E-01	2.2E-01	9.6E-01
THCA	ZNF664_ES_2.2:4_1.4_5	ZNF664	ES	2.2:4	1.4	5	9.6E-10	0.110	0.169	8.1E-01	6.1E-01	6.3E-01
THCA	ZNF667_ES_2:3.2_1_6	ZNF667	ES	02:03.2	1	6	3.1E-11	0.104	-0.382	3.5E-02	1.3E-01	9.9E-01
THCA	ZNF667_ES_9_8_12	ZNF667	ES	9	8	12	7.7E-11	0.101	-0.623	3.3E-01	6.1E-01	5.6E-02
THCA	ZNF814_ES_2:3.1_1_9	ZNF814	ES	02:03.1	1	9	2.8E-12	0.114	-0.286	8.0E-01	3.5E-01	5.9E-01
UCEC	AGBL5_ES_14_13_15	AGBL5	ES	14	13	15	1.6E-13	0.135	0.110	5.8E-02	1.7E-02	4.3E-01
UCEC	ALAS1_ES_2_1_3.1	ALAS1	ES	2	1	3.1	7.2E-18	0.166	-0.024	5.1E-01	9.9E-02	6.5E-01
UCEC	ALG8_ES_14_13_15	ALG8	ES	14	13	15	6.0E-13	0.107	-0.059	5.2E-02	2.2E-01	1.2E-01
UCEC	ARFIP2_AA_2.1_1_2.2	ARFIP2	AA	2.1	1	2.2	1.1E-14	0.130	-0.106	8.8E-02	5.8E-01	1.7E-01

cancer type	id	Gene Symbol	splice_type	Exon	From.Exon	To.Exon	anova.p	adj.r2	r	p.50	p.25	p.10
UCEC	ARHGEF1_ES_29_28_30	ARHGEF1	ES	29	28	30	3.3E-21	0.169	0.131	1.2E-02	7.2E-04	4.3E-03
UCEC	ATF2_ES_7_6_8	ATF2	ES	7	6	8	8.0E-12	0.120	-0.073	8.9E-01	9.2E-02	9.6E-02
UCEC	ATP5G1_AD_1.2_1.1_2	ATP5G1	AD	1.2	1.1	2	2.2E-12	0.101	0.100	3.6E-02	7.2E-02	8.8E-02
UCEC	BCL2L12_ES_3.1:3.2_2_4	BCL2L12	ES	3.1:3.2	2	4	7.3E-14	0.113	0.093	2.5E-02	1.6E-02	2.7E-03
UCEC	C16orf13_ES_2_1_3	C16orf13	ES	2	1	3	1.2E-35	0.268	-0.105	8.9E-05	1.8E-03	3.4E-02
UCEC	C16orf13_ES_2_1_5	C16orf13	ES	2	1	5	1.7E-26	0.211	-0.065	6.1E-03	4.9E-04	2.0E-03
UCEC	C16orf13_ES_2:3_1_5	C16orf13	ES	2:03	1	5	4.8E-20	0.160	-0.116	1.0E-03	8.4E-03	1.0E-02
UCEC	C16orf13_ES_2:3:4_1_5	C16orf13	ES	2:03:04	1	5	3.1E-25	0.198	-0.081	2.4E-04	1.8E-04	4.6E-03
UCEC	C21orf58_RI_9.2_9.1_9.3	C21orf58	RI	9.2	9.1	9.3	4.1E-13	0.111	-0.419	1.2E-01	1.1E-02	1.3E-02
UCEC	C4orf48_AD_1.2_1.1_2	C4orf48	AD	1.2	1.1	2	3.7E-12	0.121	-0.215	6.9E-03	2.2E-02	5.2E-01
UCEC	CCDC107_RI_3.4_3.3_3.5	CCDC107	RI	3.4	3.3	3.5	3.4E-32	0.246	-0.449	8.4E-06	3.5E-05	2.6E-03
UCEC	CCDC51_AA_3.1_2.2_3.2	CCDC51	AA	3.1	2.2	3.2	1.2E-10	0.107	0.482	3.0E-02	3.6E-02	5.6E-03
UCEC	CCNDBP1_ES_10.1_9_10.3	CCNDBP1	ES	10.1	9	10.3	1.6E-23	0.195	0.247	1.9E-01	4.9E-01	8.6E-01
UCEC	CD47_ES_8_7_11	CD47	ES	8	7	11	3.6E-19	0.182	-0.324	6.3E-02	2.5E-01	4.8E-01
UCEC	CD47_ES_8:9:10_7_11	CD47	ES	8:09:10	7	11	2.0E-15	0.152	-0.140	4.9E-01	1.2E-01	7.6E-02
UCEC	CDC42SE1_ES_5_4_6	CDC42SE1	ES	5	4	6	1.4E-17	0.147	-0.223	2.1E-03	9.0E-04	8.6E-02
UCEC	CROCC_ES_36_35_37	CROCC	ES	36	35	37	2.9E-12	0.124	0.190	4.1E-02	2.2E-03	5.6E-02
UCEC	CTNNB1_AA_18.3_18.1_18.4	CTNNB1	AA	18.3	18.1	18.4	8.7E-17	0.135	-0.150	1.3E-02	2.1E-03	2.3E-02
UCEC	DCTD_ES_2.1:2.2:3_1.2_5	DCTD	ES	2.1:2.2:3	1.2	5	4.5E-10	0.106	0.070	6.6E-01	2.7E-01	8.9E-02
UCEC	DMKN_AA_18.3:18.4_18.1_18.5	DMKN	AA	18.3:18.4	18.1	18.5	4.0E-12	0.110	-0.082	1.7E-02	1.3E-01	4.4E-02
UCEC	DNAJB12_RI_9.3_9.2_9.4	DNAJB12	RI	9.3	9.2	9.4	5.9E-21	0.167	0.035	1.9E-02	4.4E-02	6.5E-01
UCEC	DYRK1B_AA_10.1:10.2_9_10.3	DYRK1B	AA	10.1:10.2	9	10.3	1.1E-32	0.259	0.148	5.8E-03	7.6E-03	1.3E-03
UCEC	EFCAB4A_ES_8_7_9	EFCAB4A	ES	8	7	9	7.1E-28	0.218	0.356	4.0E-04	2.9E-05	3.3E-02
UCEC	FDPS_AD_1.2_1.1_3.2	FDPS	AD	1.2	1.1	3.2	2.0E-10	0.107	-0.101	3.0E-02	2.4E-02	7.2E-02
UCEC	FYN_ME_11|12_10_13	FYN	ME	11|12	10	13	9.3E-14	0.117	0.298	1.7E-01	2.2E-03	2.3E-01
UCEC	HAUS4_AD_1.3_1.2_2	HAUS4	AD	1.3	1.2	2	2.4E-12	0.117	-0.242	8.7E-02	1.2E-01	2.2E-01
UCEC	HNRNPC_ES_2.2:2.3:2.4_1_3.2	HNRNPC	ES	2.2:2.3:2.4	1	3.2	1.6E-12	0.104	-0.112	1.8E-02	6.5E-02	5.5E-02
UCEC	HNRNPC_ES_2.4_1_3.2	HNRNPC	ES	2.4	1	3.2	1.0E-15	0.128	-0.134	2.0E-02	2.4E-04	1.1E-01
UCEC	HNRNPC_ES_2.4:2.5:2.6_1_3.2	HNRNPC	ES	2.4:2.5:2.6	1	3.2	3.1E-16	0.133	-0.135	4.7E-03	5.4E-03	5.4E-02
UCEC	IFT122_ES_5:7_3_8.2	IFT122	ES	5:07	3	8.2	1.7E-12	0.129	0.065	2.8E-04	6.4E-03	1.3E-02
UCEC	ITGB3BP_ES_9_8_10	ITGB3BP	ES	9	8	10	1.3E-18	0.153	-0.022	4.0E-02	4.4E-02	7.4E-02
UCEC	KARS_ES_2_1_3	KARS	ES	2	1	3	1.0E-14	0.124	-0.154	9.1E-02	1.1E-01	2.9E-01
UCEC	KIAA1217_ES_12_11_13	KIAA1217	ES	12	11	13	1.7E-12	0.124	-0.310	6.1E-02	1.6E-01	8.4E-02
UCEC	LRRC23_ES_5_4_6	LRRC23	ES	5	4	6	2.6E-19	0.162	0.344	6.2E-04	6.8E-02	8.0E-02
UCEC	LSR_ES_4_3_5	LSR	ES	4	3	5	6.9E-23	0.184	0.115	1.2E-02	1.2E-01	9.0E-03
UCEC	MAP4K4_ES_17:18_16.2_19	MAP4K4	ES	17:18	16.2	19	3.2E-10	0.106	0.148	2.2E-02	2.0E-01	2.9E-01
UCEC	MAP4K4_ES_18_16.2_19	MAP4K4	ES	18	16.2	19	1.8E-15	0.158	0.080	6.6E-02	3.9E-01	6.8E-01
UCEC	MED24_ES_7_6_8	MED24	ES	7	6	8	1.7E-14	0.142	0.019	9.9E-04	6.8E-04	4.2E-02
UCEC	MRPS12_AD_1.2_1.1_1.4	MRPS12	AD	1.2	1.1	1.4	5.0E-34	0.261	0.192	1.5E-02	1.0E-04	1.4E-02
UCEC	MTCH1_AD_8.2_8.1_9	MTCH1	AD	8.2	8.1	9	7.4E-18	0.144	0.191	3.0E-02	5.8E-02	3.7E-01

cancer type	id	Gene Symbol	splice_type	Exon	From.Exon	To.Exon	anova.p	adj.r2	r	p.50	p.25	p.10
UCEC	MVD_ES_3:4_1_5	MVD	ES	3:04	1	5	1.8E-12	0.110	0.024	4.2E-02	3.8E-02	7.0E-02
UCEC	MYL6_ES_2.1_1.4_3.1	MYL6	ES	2.1	1.4	3.1	4.2E-13	0.108	-0.223	2.0E-01	7.2E-03	1.2E-01
UCEC	MYL6_ES_4.3_4.1_5	MYL6	ES	4.3	4.1	5	4.8E-17	0.137	-0.134	3.1E-03	3.7E-03	5.4E-02
UCEC	NLRX1_AA_10.1_9_10.2	NLRX1	AA	10.1	9	10.2	8.0E-18	0.143	-0.343	9.6E-02	3.3E-02	3.0E-01
UCEC	NT5C_AD_3.3_3.2_4.1	NT5C	AD	3.3	3.2	4.1	8.3E-13	0.104	-0.182	8.2E-02	8.5E-02	1.5E-01
UCEC	ODF2L_AA_20.1_19_20.2	ODF2L	AA	20.1	19	20.2	1.9E-13	0.109	-0.261	3.8E-04	4.7E-05	1.0E-03
UCEC	ODF3B_AA_4.1_3.2_4.2	ODF3B	AA	4.1	3.2	4.2	2.6E-12	0.116	-0.365	5.7E-02	7.0E-03	4.5E-02
UCEC	OSBPL9_ES_17_15_18	OSBPL9	ES	17	15	18	7.3E-19	0.172	-0.175	2.4E-02	5.1E-02	3.0E-01
UCEC	PACRGL_ES_12_9_13.1	PACRGL	ES	12	9	13.1	2.0E-12	0.118	0.080	2.8E-01	3.9E-02	1.7E-01
UCEC	PALM_ES_8_7_9	PALM	ES	8	7	9	9.1E-11	0.112	-0.534	8.2E-02	3.3E-02	2.2E-03
UCEC	PAPOLA_ES_19_18_20	PAPOLA	ES	19	18	20	1.4E-14	0.121	-0.167	3.3E-01	1.5E-01	1.6E-01
UCEC	PCM1_ES_25_24_27	PCM1	ES	25	24	27	2.4E-13	0.123	0.166	7.3E-02	4.9E-01	3.5E-01
UCEC	PDLIM7_AA_10.1_8_10.2	PDLIM7	AA	10.1	8	10.2	6.1E-15	0.122	0.047	6.0E-02	2.2E-01	3.3E-01
UCEC	PKP4_ES_25_24_26	PKP4	ES	25	24	26	2.0E-18	0.149	-0.065	4.6E-01	4.9E-01	8.7E-01
UCEC	PLSCR1_ES_4_1_5	PLSCR1	ES	4	1	5	4.6E-16	0.137	-0.034	4.4E-01	2.6E-01	9.0E-01
UCEC	PORCN_AA_3.1_2.2_3.2	PORCN	AA	3.1	2.2	3.2	3.4E-13	0.120	-0.130	6.0E-01	2.2E-02	1.2E-02
UCEC	PPP3CC_ES_14_13_15	PPP3CC	ES	14	13	15	2.6E-11	0.104	-0.013	9.3E-01	2.1E-01	6.3E-02
UCEC	PRDX5_ES_3_1_4	PRDX5	ES	3	1	4	4.0E-20	0.161	-0.166	4.5E-03	2.4E-03	4.2E-03
UCEC	PROM1_ES_27:28_25_30	PROM1	ES	27:28:00	25	30	3.6E-20	0.186	0.178	1.3E-01	6.3E-02	6.5E-02
UCEC	PUF60_ES_6_5_7	PUF60	ES	6	5	7	1.5E-14	0.118	0.368	3.5E-02	3.9E-03	1.4E-01
UCEC	RAB34_ES_4.1:4.2_3_5	RAB34	ES	4.1:4.2	3	5	8.1E-11	0.102	-0.069	9.7E-01	4.6E-01	4.7E-01
UCEC	RBM42_ES_3.2:4:6.1:6.2:6.3:7:8:9.1_3.1	RBM42	ES	:4:6.1:6.2:6.3:7:8	3.1	9.2	4.1E-15	0.161	-0.181	1.6E-03	5.8E-03	1.4E-02
UCEC	RNPS1_ES_3_1.1_4	RNPS1	ES	3	1.1	4	6.1E-13	0.106	-0.044	1.8E-03	4.3E-04	1.3E-01
UCEC	RPGR_ES_14.1:14.3_13_16	RPGR	ES	14.1:14.3	13	16	4.6E-13	0.124	0.272	1.4E-03	1.9E-04	6.6E-06
UCEC	RPL22L1_AA_3.1_2.2_3.2	RPL22L1	AA	3.1	2.2	3.2	1.4E-53	0.379	-0.612	9.3E-04	1.6E-03	1.6E-05
UCEC	RPS24_AA_5.1_4_5.2	RPS24	AA	5.1	4	5.2	6.2E-17	0.137	-0.130	6.4E-03	4.0E-04	1.5E-04
UCEC	RPS24_ES_5.1:5.2_4_6	RPS24	ES	5.1:5.2	4	6	7.2E-20	0.159	-0.049	9.9E-04	1.0E-02	5.6E-01
UCEC	RWDD1_ES_2_1_3	RWDD1	ES	2	1	3	1.9E-11	0.103	-0.135	1.6E-04	1.9E-03	3.5E-02
UCEC	S100A1_AA_4.1_3_4.2	S100A1	AA	4.1	3	4.2	2.9E-19	0.161	-0.764	1.3E-01	1.1E-01	2.7E-02
UCEC	SCP2_ES_12_11_13	SCP2	ES	12	11	13	1.7E-12	0.102	-0.592	9.1E-01	9.6E-01	1.3E-01
UCEC	SCRIB_ES_36_35_37	SCRIB	ES	36	35	37	1.0E-17	0.143	0.060	1.5E-03	5.1E-06	1.4E-03
UCEC	SCRN2_RI_7.2_7.1_7.3	SCRN2	RI	7.2	7.1	7.3	6.3E-17	0.137	-0.426	1.4E-02	2.2E-02	1.1E-01
UCEC	SPINT1_AD_5.2_5.1_6	SPINT1	AD	5.2	5.1	6	2.5E-19	0.158	-0.099	5.9E-02	2.4E-03	5.8E-03
UCEC	SSBP3_ES_7_6_8	SSBP3	ES	7	6	8	3.8E-15	0.125	0.179	3.0E-02	7.5E-02	9.9E-01
UCEC	SUPT20H_ES_26_25_27	SUPT20H	ES	26	25	27	7.6E-15	0.120	0.271	1.2E-01	2.4E-02	4.6E-04
UCEC	TANK_ES_3_2.2_4	TANK	ES	3	2.2	4	2.4E-11	0.103	-0.583	1.6E-02	1.9E-02	3.8E-01
UCEC	TCF20_ES_4.1:4.2_3_5	TCF20	ES	4.1:4.2	3	5	5.7E-23	0.184	0.058	2.8E-02	2.9E-03	3.1E-01
UCEC	TMC6_ES_14.3_14.1_15	TMC6	ES	14.3	14.1	15	7.2E-16	0.129	0.106	3.9E-02	2.3E-01	1.6E-01
UCEC	TMEM107_ES_2:3.2:3.3:3.4:3.5_1_3.7	TMEM107	ES	2:3.2:3.3:3.4:3.5	1	3.7	2.2E-19	0.183	-0.424	2.4E-03	2.0E-03	3.6E-03
UCEC	TMEM107_RI_3.3_3.2_3.4	TMEM107	RI	3.3	3.2	3.4	3.6E-15	0.127	-0.573	1.3E-02	2.9E-02	9.9E-02

cancer type	id	Gene Symbol	splice_type	Exon	From.Exon	To.Exon	anova.p	adj.r2	r	p.50	p.25	p.10
UCEC	TPM2_ME_6|7_5_8	TPM2	ME	6|7	5	8	1.1E-17	0.142	-0.004	2.7E-04	3.6E-02	7.6E-02
UCEC	TRAPPC6A_AD_1.2_1.1_2	TRAPPC6A	AD	1.2	1.1	2	2.5E-12	0.106	-0.052	4.2E-02	1.1E-03	3.0E-01
UCEC	TRAPPC6A_ES_1.2:2_1.1_3	TRAPPC6A	ES	1.2:2	1.1	3	7.6E-17	0.140	-0.128	6.8E-05	1.1E-05	6.2E-03
UCEC	TRO_AD_12.2:12.3_12.1_13	TRO	AD	12.2:12.3	12.1	13	1.0E-21	0.175	-0.610	5.7E-03	1.6E-03	1.1E-02
UCEC	UBQLN1_ES_8_7_9	UBQLN1	ES	8	7	9	7.6E-17	0.138	-0.216	1.5E-03	1.0E-03	1.7E-03
UCEC	UBXN11_ES_4:5:6:7:8:9_2_10	UBXN11	ES	4:5:6:7:8:9	2	10	1.3E-13	0.123	0.256	1.6E-02	2.7E-02	1.4E-02
UCEC	USF2_ES_3_2.3_4	USF2	ES	3	2.3	4	1.2E-15	0.127	-0.285	1.2E-01	3.5E-02	1.1E-01
UCEC	USP21_ES_2_1_3	USP21	ES	2	1	3	1.7E-12	0.116	-0.067	4.3E-02	1.0E-01	7.5E-02
UCEC	WDR41_ES_3_2_4	WDR41	ES	3	2	4	1.2E-12	0.107	-0.534	3.1E-01	4.6E-01	7.1E-01
UCEC	WFDC2_ES_2:4.2_1_4.3	WFDC2	ES	02:04.2	1	4.3	1.5E-15	0.147	0.093	8.4E-05	2.6E-04	8.6E-02
UCEC	YIPF1_ES_12_11_13	YIPF1	ES	12	11	13	1.5E-12	0.102	-0.104	8.9E-02	3.1E-02	9.1E-02
UCEC	YWHAE_ES_2:3:4:5:6_1_7	YWHAE	ES	2:3:4:5:6	1	7	3.8E-12	0.101	-0.141	8.3E-02	1.7E-01	8.8E-01
UCEC	ZFAND1_ES_3_2_4	ZFAND1	ES	3	2	4	2.0E-13	0.125	0.064	5.5E-01	8.8E-01	6.7E-01
UCEC	ZFYVE19_ES_8_7_9	ZFYVE19	ES	8	7	9	7.2E-15	0.121	0.242	1.4E-01	1.1E-01	4.3E-01
UCEC	ZMIZ2_AA_5.1:5.2_4_5.3	ZMIZ2	AA	5.1:5.2	4	5.3	5.9E-13	0.113	-0.095	4.7E-03	1.0E-01	4.7E-01
UCEC	ZSWIM7_RI_7.2_7.1_7.3	ZSWIM7	RI	7.2	7.1	7.3	1.8E-18	0.148	-0.040	1.0E-01	2.9E-02	2.1E-01
UCS	AAGAB_AD_2.2_2.1_3	AAGAB	AD	2.2	2.1	3	9.1E-04	0.198	-0.070	9.2E-01	6.6E-01	2.4E-01
UCS	ABI2_ES_13.1:13.2_12_14	ABI2	ES	13.1:13.2	12	14	5.5E-12	0.581	-0.040	5.8E-01	2.8E-01	7.4E-01
UCS	ACHE_AA_6.1_5_6.2	ACHE	AA	6.1	5	6.2	8.1E-04	0.177	-0.445	3.7E-01	5.2E-01	5.7E-01
UCS	ACSS2_ES_9_8_10	ACSS2	ES	9	8	10	4.7E-10	0.549	0.701	NA	NA	NA
UCS	ADAM15_ES_22.1:22.2_21.2_23	ADAM15	ES	22.1:22.2	21.2	23	5.0E-05	0.247	-0.452	5.3E-01	7.2E-01	3.0E-01
UCS	AFMID_ES_5:6_2_13	AFMID	ES	5:06	2	13	5.8E-05	0.255	0.206	2.7E-01	6.5E-01	8.6E-01
UCS	AFMID_ES_5:6:7:8:12_2_13	AFMID	ES	5:6:7:8:12	2	13	3.0E-05	0.287	0.148	7.3E-01	9.3E-01	1.1E-01
UCS	AFMID_ES_5:6:8:9:10:11.1:12_2_13	AFMID	ES	5:6:8:9:10:11.1:12	2	13	2.5E-04	0.254	0.158	2.9E-01	4.6E-01	3.5E-01
UCS	AFMID_ES_7:8:9:10:12_6_13	AFMID	ES	7:8:9:10:12	6	13	7.1E-04	0.206	-0.082	3.9E-01	4.3E-01	8.9E-01
UCS	AFMID_ES_9:10_8_12	AFMID	ES	9:10	8	12	8.4E-04	0.189	-0.071	8.0E-01	6.6E-01	3.2E-01
UCS	ALDOA_AD_6.3_6.2_9	ALDOA	AD	6.3	6.2	9	5.5E-10	0.497	0.367	4.8E-02	4.0E-01	9.4E-02
UCS	AMPD2_ES_2.2_1_4	AMPD2	ES	2.2	1	4	5.3E-04	0.196	0.246	6.7E-01	4.1E-01	8.3E-01
UCS	ANKRD10_ME_4|5_3_6.1	ANKRD10	ME	4|5	3	6.1	1.8E-05	0.274	0.028	7.3E-01	8.3E-01	3.0E-01
UCS	ANXA6_ES_22_21_23	ANXA6	ES	22	21	23	3.3E-04	0.197	0.698	8.6E-01	8.1E-01	1.2E-01
UCS	ANXA7_ES_6_5_7	ANXA7	ES	6	5	7	7.7E-09	0.448	-0.173	8.1E-01	8.2E-01	9.0E-01
UCS	AP2A1_ES_16_15_17	AP2A1	ES	16	15	17	9.3E-05	0.231	-0.117	6.3E-01	4.0E-01	3.0E-01
UCS	AP3D1_AA_24.1_23_24.2	AP3D1	AA	24.1	23	24.2	7.4E-05	0.241	-0.157	6.3E-01	2.7E-01	4.2E-01
UCS	APH1A_RI_6.2_6.1_6.3	APH1A	RI	6.2	6.1	6.3	7.3E-05	0.237	0.116	5.8E-01	1.8E-01	5.3E-01
UCS	APTX_ES_6.1:6.2:7.1:7.2:7.3_5.2_7.4	APTX	ES	6.1:6.2:7.1:7.2:7.3	5.2	7.4	3.5E-04	0.201	-0.173	8.6E-01	2.4E-01	4.3E-01
UCS	APTX_ES_6.2:7.1:7.2:7.3_5.2_7.4	APTX	ES	6.2:7.1:7.2:7.3	5.2	7.4	7.7E-04	0.185	-0.163	5.9E-01	7.5E-01	1.6E-01
UCS	ARAP1_ES_16_15_17	ARAP1	ES	16	15	17	1.3E-05	0.281	-0.019	7.2E-01	7.5E-01	4.6E-01
UCS	ARFGAP2_ES_4.2:5:6.1:6.2:7_3_8	ARFGAP2	ES	4.2:5:6.1:6.2:7	3	8	5.7E-04	0.191	0.315	2.8E-01	4.9E-01	9.5E-01
UCS	ARHGAP23_ES_24_23_25	ARHGAP23	ES	24	23	25	2.9E-12	0.619	0.574	NA	NA	NA
UCS	ARHGEF40_ES_21_20_22	ARHGEF40	ES	21	20	22	3.9E-11	0.543	-0.168	6.2E-01	7.1E-01	1.3E-01

cancer type	id	Gene Symbol	splice_type	Exon	From.Exon	To.Exon	anova.p	adj.r2	r	p.50	p.25	p.10
UCS	ARMC10_ES_2_1_5.1	ARMC10	ES	2	1	5.1	5.4E-05	0.245	-0.158	5.9E-01	4.0E-01	4.8E-01
UCS	ASB8_ES_5.2_4.1_5.4	ASB8	ES	5.2	4.1	5.4	1.1E-05	0.344	0.197	7.6E-01	5.9E-01	8.3E-01
UCS	ATG13_ES_13:14_12_15	ATG13	ES	13:14	12	15	1.4E-10	0.535	0.053	6.7E-01	6.7E-01	1.0E+00
UCS	ATG13_ES_14_12_15	ATG13	ES	14	12	15	2.1E-07	0.390	0.123	3.4E-01	2.9E-01	1.6E-01
UCS	ATP2A2_RI_21.2:21.3_21.1_21.4	ATP2A2	RI	21.2:21.3	21.1	21.4	2.7E-17	0.726	-0.762	7.3E-01	4.4E-01	3.0E-01
UCS	ATP2B1_ES_21_20_22	ATP2B1	ES	21	20	22	2.0E-12	0.603	0.455	1.8E-01	7.4E-01	3.0E-01
UCS	ATP5C1_ES_9_8.1_10	ATP5C1	ES	9	8.1	10	1.4E-10	0.521	0.141	1.1E-01	3.1E-01	3.1E-01
UCS	ATP5H_ES_5_3_6	ATP5H	ES	5	3	6	4.0E-05	0.261	-0.224	6.1E-01	5.0E-01	5.0E-01
UCS	ATP6V1H_ES_8_7_9	ATP6V1H	ES	8	7	9	6.7E-11	0.541	0.052	9.3E-01	9.5E-01	3.2E-01
UCS	BAIAP2_ES_17.1:17.2_16.1_18.1	BAIAP2	ES	17.1:17.2	16.1	18.1	8.9E-04	0.169	0.092	3.7E-01	3.7E-01	1.7E-01
UCS	BCAS3_ES_20_19_21	BCAS3	ES	20	19	21	9.8E-04	0.184	-0.119	7.2E-01	6.2E-01	5.0E-01
UCS	BCL2L1_AA_2.2_1_2.3	BCL2L1	AA	2.2	1	2.3	5.8E-05	0.260	-0.224	6.5E-01	7.3E-02	2.8E-01
UCS	BCL7A_AD_5.2_5.1_6	BCL7A	AD	5.2	5.1	6	1.6E-04	0.244	0.174	6.9E-01	3.7E-01	2.5E-01
UCS	BIN1_ES_11_10_12	BIN1	ES	11	10	12	8.6E-08	0.398	0.698	8.9E-01	4.7E-01	7.7E-01
UCS	BIN1_ES_13_12_18	BIN1	ES	13	12	18	5.4E-04	0.195	-0.358	4.1E-02	1.1E-01	5.5E-01
UCS	BMP1_AA_5.1_4_5.2	BMP1	AA	5.1	4	5.2	7.7E-04	0.185	0.007	3.1E-01	4.5E-01	4.6E-01
UCS	C16orf13_ES_2_1_5	C16orf13	ES	2	1	5	8.6E-04	0.169	-0.046	8.6E-01	7.5E-01	7.4E-01
UCS	C16orf13_ES_2:3_1_5	C16orf13	ES	2:03	1	5	4.7E-04	0.186	-0.071	5.4E-01	9.7E-01	5.0E-01
UCS	C16orf13_ES_2:3:4_1_5	C16orf13	ES	2:03:04	1	5	1.4E-04	0.220	-0.204	1.7E-01	3.8E-01	6.1E-01
UCS	C21orf59_AA_5.3:5.4_5.1_5.5	C21orf59	AA	5.3:5.4	5.1	5.5	8.8E-07	0.347	-0.096	3.5E-02	9.6E-01	5.7E-01
UCS	C21orf59_AD_5.2:5.3_5.1_5.5	C21orf59	AD	5.2:5.3	5.1	5.5	1.9E-04	0.211	-0.142	4.0E-01	1.7E-01	9.4E-01
UCS	C9orf89_AA_3.3:3.4_3.1_3.5	C9orf89	AA	3.3:3.4	3.1	3.5	8.4E-05	0.233	0.127	6.9E-01	8.5E-01	3.7E-01
UCS	C9orf89_AD_3.2:3.3_3.1_3.5	C9orf89	AD	3.2:3.3	3.1	3.5	4.0E-04	0.191	-0.066	8.1E-01	6.3E-01	5.8E-01
UCS	C9orf89_ES_3.3_3.1_3.5	C9orf89	ES	3.3	3.1	3.5	6.5E-06	0.299	0.264	3.1E-01	4.8E-01	2.8E-01
UCS	CADM1_ES_11_10_12	CADM1	ES	11	10	12	9.0E-04	0.194	-0.279	9.9E-01	7.1E-01	6.1E-01
UCS	CAMK2D_ES_17_14.2_18	CAMK2D	ES	17	14.2	18	8.6E-09	0.539	0.401	3.2E-01	4.0E-01	3.8E-01
UCS	CAMK2D_ES_22_21.1_23	CAMK2D	ES	22	21.1	23	1.9E-04	0.238	0.356	5.7E-01	3.3E-01	7.5E-01
UCS	CAMK2G_ES_13_12_14	CAMK2G	ES	13	12	14	6.8E-05	0.279	0.030	5.3E-02	6.6E-02	3.6E-01
UCS	CAMKK2_ES_17_16_19.1	CAMKK2	ES	17	16	19.1	6.8E-04	0.196	-0.016	7.9E-01	5.2E-01	5.6E-01
UCS	CAPZB_ES_11_10_12	CAPZB	ES	11	10	12	2.7E-05	0.263	-0.028	8.0E-01	7.2E-02	6.6E-01
UCS	CARD9_AA_8.1_7_8.2	CARD9	AA	8.1	7	8.2	6.0E-05	0.305	-0.401	5.9E-01	8.9E-01	2.6E-01
UCS	CARM1_ES_16.1_15_16.3	CARM1	ES	16.1	15	16.3	4.3E-10	0.502	0.290	8.8E-01	1.0E-01	4.8E-01
UCS	CBR1_AA_3.1_2.1_3.2	CBR1	AA	3.1	2.1	3.2	5.3E-04	0.229	0.336	4.0E-01	5.3E-01	7.3E-01
UCS	CCDC64_ES_7_6_8	CCDC64	ES	7	6	8	1.8E-05	0.317	0.030	7.7E-01	4.1E-01	9.8E-01
UCS	CCSER2_ES_11_10_12	CCSER2	ES	11	10	12	2.0E-04	0.237	0.163	2.8E-02	1.0E-01	9.9E-01
UCS	CD44_ES_12.1:13:14_5_15	CD44	ES	12.1:13:14	5	15	2.2E-04	0.208	0.274	3.7E-01	6.6E-01	2.5E-01
UCS	CD46_ES_8_6_9	CD46	ES	8	6	9	3.2E-04	0.203	0.243	4.4E-01	8.5E-02	5.0E-01
UCS	CDC42BPA_ES_36_35_37	CDC42BPA	ES	36	35	37	5.0E-08	0.461	-0.116	7.9E-01	8.9E-02	7.0E-01
UCS	CDK10_ES_5_2.2_6	CDK10	ES	5	2.2	6	3.8E-04	0.203	0.176	7.4E-01	2.7E-01	6.9E-01
UCS	CDKN1C_AD_1.4_1.3_2	CDKN1C	AD	1.4	1.3	2	7.8E-04	0.172	-0.074	8.7E-01	1.5E-01	7.4E-01

cancer type	id	Gene Symbol	splice_type	Exon	From.Exon	To.Exon	anova.p	adj.r2	r	p.50	p.25	p.10
UCS	CENPW_AD_1.2_1.1_2.1	CENPW	AD	1.2	1.1	2.1	4.4E-05	0.250	-0.114	4.8E-01	2.8E-01	8.4E-01
UCS	CERS5_AA_17.1_16_17.2	CERS5	AA	17.1	16	17.2	7.4E-07	0.350	-0.008	1.9E-02	3.8E-02	3.2E-03
UCS	CERS6_ES_10_9_11	CERS6	ES	10	9	11	6.2E-09	0.478	0.187	5.1E-01	8.6E-01	1.0E+00
UCS	CHD4_AA_4.1_3.2_4.2	CHD4	AA	4.1	3.2	4.2	4.7E-05	0.285	-0.003	1.4E-01	2.2E-01	4.9E-01
UCS	CLIP2_ES_9_8_10	CLIP2	ES	9	8	10	4.7E-05	0.275	-0.164	1.9E-02	2.7E-01	5.0E-01
UCS	CLSTN1_ES_11_10_12	CLSTN1	ES	11	10	12	1.2E-04	0.228	-0.133	4.2E-01	5.6E-01	3.9E-01
UCS	CLUAP1_RI_12.4_12.3_12.5	CLUAP1	RI	12.4	12.3	12.5	5.9E-09	0.472	0.115	1.0E+00	7.3E-01	5.2E-01
UCS	CMTM7_ES_3_2_4.2	CMTM7	ES	3	2	4.2	1.4E-05	0.279	0.293	4.9E-01	4.4E-01	8.3E-02
UCS	CMTM7_ES_3:4.1_2_4.2	CMTM7	ES	03:04.1	2	4.2	1.0E-04	0.228	0.081	5.4E-01	7.0E-01	3.7E-01
UCS	COA1_ES_3.1_1_4.2	COA1	ES	3.1	1	4.2	1.2E-04	0.225	-0.367	4.2E-01	7.9E-01	2.4E-01
UCS	COA1_ES_3.1:3.2_1_4.2	COA1	ES	3.1:3.2	1	4.2	2.4E-04	0.205	-0.372	6.9E-01	1.9E-01	4.1E-01
UCS	COA1_ME_2|3.1:3.2_1_4.2	COA1	ME	2|3.1:3.2	1	4.2	1.9E-04	0.215	0.223	2.5E-01	6.0E-01	6.4E-01
UCS	COL23A1_ES_6_5_7	COL23A1	ES	6	5	7	1.1E-04	0.259	-0.171	3.6E-01	7.9E-01	4.2E-01
UCS	CPSF4_AA_9.1_8_9.2	CPSF4	AA	9.1	8	9.2	2.6E-05	0.264	-0.107	4.2E-01	1.8E-01	3.1E-01
UCS	CSDE1_ES_3_2_4	CSDE1	ES	3	2	4	4.4E-12	0.598	0.366	4.1E-01	2.2E-01	5.7E-01
UCS	CSDE1_ES_3:4_2_5	CSDE1	ES	3:04	2	5	6.4E-09	0.537	0.073	6.4E-01	7.5E-01	6.4E-01
UCS	CSNK1D_ES_11_10_12	CSNK1D	ES	11	10	12	6.0E-10	0.496	0.020	9.5E-01	5.9E-01	2.7E-01
UCS	CSNK1G3_ES_11_10_12	CSNK1G3	ES	11	10	12	6.9E-10	0.527	-0.070	2.4E-02	3.1E-01	1.6E-01
UCS	CTNNB1_ES_5_4_6	CTNNB1	ES	5	4	6	1.3E-08	0.456	0.121	8.2E-01	1.8E-01	3.4E-03
UCS	CTNND1_ES_12_11_13	CTNND1	ES	12	11	13	9.4E-13	0.658	-0.012	7.2E-01	3.4E-01	3.9E-01
UCS	CTNND1_ES_3:4.1:4.2:4.3_2.1_5	CTNND1	ES	3:4.1:4.2:4.3	2.1	5	2.0E-04	0.256	-0.280	7.5E-01	2.3E-01	4.0E-01
UCS	CTTN_ES_11_10_13	CTTN	ES	11	10	13	2.6E-05	0.264	0.332	6.7E-01	7.3E-01	5.6E-01
UCS	CYTH2_AA_9.1_8.1_9.2	CYTH2	AA	9.1	8.1	9.2	2.8E-05	0.262	-0.261	5.0E-01	7.7E-01	1.2E-01
UCS	DCAF6_ME_11|12_10_13.1	DCAF6	ME	11|12	10	13.1	5.1E-04	0.208	0.319	9.7E-01	6.2E-01	4.3E-01
UCS	DCLK2_ES_17_16_18	DCLK2	ES	17	16	18	9.2E-07	0.391	-0.341	7.8E-01	8.9E-01	9.7E-01
UCS	DCTN1_ES_7.2:8_5_9	DCTN1	ES	7.2:8	5	9	2.3E-05	0.290	-0.007	8.1E-01	6.7E-01	2.7E-01
UCS	DDIT3_RI_2.2:2.3_2.1_2.4	DDIT3	RI	2.2:2.3	2.1	2.4	9.2E-05	0.231	-0.280	4.0E-01	1.1E-01	5.6E-01
UCS	DDIT3_RI_2.2:2.3:2.4_2.1_2.5	DDIT3	RI	2.2:2.3:2.4	2.1	2.5	1.7E-05	0.274	-0.109	9.5E-01	5.9E-01	4.5E-01
UCS	DENND1A_ES_23_21_24	DENND1A	ES	23	21	24	3.4E-05	0.294	0.011	3.6E-01	5.6E-01	7.0E-01
UCS	DEPDC5_ES_36_35_37	DEPDC5	ES	36	35	37	3.2E-09	0.569	-0.053	3.3E-01	4.7E-01	8.4E-02
UCS	DGUOK_ES_5_4_7	DGUOK	ES	5	4	7	2.4E-04	0.204	0.122	1.3E-01	4.9E-01	5.8E-01
UCS	DGUOK_ES_5:6_4_7	DGUOK	ES	5:06	4	7	6.3E-04	0.178	-0.001	7.3E-01	9.9E-01	8.4E-01
UCS	DHRS12_ES_3_2_4	DHRS12	ES	3	2	4	3.5E-05	0.260	0.313	9.7E-01	7.0E-01	5.3E-01
UCS	DNM2_ES_15_14_17	DNM2	ES	15	14	17	3.9E-09	0.461	-0.006	1.5E-01	4.7E-01	1.6E-01
UCS	DNMT3B_ES_22:23_21_24	DNMT3B	ES	22:23	21	24	2.5E-04	0.218	-0.361	8.8E-01	3.2E-01	5.7E-01
UCS	DNMT3B_ES_6_5_7	DNMT3B	ES	6	5	7	9.9E-05	0.273	-0.111	8.0E-01	7.7E-01	5.1E-01
UCS	DOLPP1_ES_6_5_7	DOLPP1	ES	6	5	7	3.5E-04	0.198	0.304	3.2E-01	7.9E-01	8.8E-01
UCS	DPF2_ES_7_6_8	DPF2	ES	7	6	8	1.6E-09	0.478	0.228	8.9E-01	2.5E-01	9.0E-01
UCS	DST_ES_104_103_105	DST	ES	104	103	105	1.2E-12	0.611	0.113	6.7E-01	3.1E-01	4.6E-01
UCS	DTNB_ES_17_16_19	DTNB	ES	17	16	19	5.7E-10	0.497	-0.279	1.6E-01	8.6E-01	3.3E-01

cancer type	id	Gene Symbol	splice_type	Exon	From.Exon	To.Exon	anova.p	adj.r2	r	p.50	p.25	p.10
UCS	DTNB_ES_20_19_21	DTNB	ES	20	19	21	1.7E-04	0.226	0.084	6.4E-01	2.7E-01	2.9E-01
UCS	DYRK1B_AA_10.1:10.2_9_10.3	DYRK1B	AA	10.1:10.2	9	10.3	4.6E-10	0.501	0.217	9.5E-01	7.1E-01	2.9E-01
UCS	DYRK1B_ES_10.1_9_10.3	DYRK1B	ES	10.1	9	10.3	2.2E-04	0.207	-0.007	9.9E-01	5.0E-01	7.2E-01
UCS	EEF1D_ME_5|6_1_7.2	EEF1D	ME	5|6	1	7.2	2.4E-04	0.205	-0.039	4.2E-01	9.5E-01	9.6E-01
UCS	EMC10_ES_7_6_8.1	EMC10	ES	7	6	8.1	8.4E-04	0.170	0.287	9.0E-01	3.1E-01	9.2E-01
UCS	EMID1_ES_15_13_16	EMID1	ES	15	13	16	4.8E-04	0.195	0.544	6.6E-01	5.8E-01	2.5E-01
UCS	EPB41L1_ES_20_19_21	EPB41L1	ES	20	19	21	7.2E-04	0.174	-0.024	2.3E-01	3.3E-02	4.5E-01
UCS	EPB41L2_AD_20.2_20.1_21	EPB41L2	AD	20.2	20.1	21	7.0E-05	0.238	-0.355	7.7E-01	7.2E-01	7.6E-01
UCS	ERBB2IP_ES_24.1:24.2:24.3_21_25	ERBB2IP	ES	24.1:24.2:24.3	21	25	2.7E-04	0.209	0.195	3.3E-02	2.2E-01	4.3E-01
UCS	EVI5L_ES_12_11_13	EVI5L	ES	12	11	13	1.6E-06	0.372	0.130	3.8E-01	4.0E-01	9.6E-01
UCS	EXOC1_ES_11_10_12	EXOC1	ES	11	10	12	2.2E-05	0.291	-0.086	4.5E-01	5.2E-01	9.0E-01
UCS	EXOC7_ES_7_6_9	EXOC7	ES	7	6	9	9.7E-06	0.293	-0.103	3.4E-01	6.5E-01	2.3E-02
UCS	EXOC7_ES_8.1:8.2_7_9	EXOC7	ES	8.1:8.2	7	9	1.6E-04	0.243	0.168	5.9E-01	6.6E-01	2.8E-01
UCS	EXOC7_ES_8.2_7_9	EXOC7	ES	8.2	7	9	8.2E-06	0.334	0.248	4.3E-01	6.5E-01	3.1E-01
UCS	FAM13A_ES_23_22_24	FAM13A	ES	23	22	24	4.2E-04	0.218	0.115	8.9E-01	9.1E-01	9.6E-01
UCS	FAM21A_ES_28_27_29	FAM21A	ES	28	27	29	1.8E-04	0.213	-0.116	5.0E-01	4.6E-01	4.8E-01
UCS	FAU_AA_2.4:2.5_2.2_2.6	FAU	AA	2.4:2.5	2.2	2.6	4.5E-04	0.187	0.003	6.5E-01	1.9E-01	8.8E-03
UCS	FAU_AD_2.3:2.4_2.2_2.6	FAU	AD	2.3:2.4	2.2	2.6	1.9E-04	0.211	-0.158	3.3E-01	1.6E-01	1.3E-01
UCS	FBXO16_ES_9_8_10	FBXO16	ES	9	8	10	2.0E-07	0.458	-0.260	4.2E-01	9.9E-01	8.5E-01
UCS	FBXO25_ES_10_9_11	FBXO25	ES	10	9	11	9.9E-10	0.493	-0.015	5.3E-01	2.6E-01	3.5E-01
UCS	FBXO8_ES_4_3_5	FBXO8	ES	4	3	5	2.6E-06	0.353	-0.337	NA	NA	NA
UCS	FDPS_ES_2_1.2_3.1	FDPS	ES	2	1.2	3.1	8.7E-04	0.188	0.071	5.0E-01	8.6E-01	8.6E-01
UCS	FERMT2_ES_14_13_15	FERMT2	ES	14	13	15	1.0E-07	0.400	0.264	3.3E-01	4.4E-01	3.5E-01
UCS	FGFR1OP2_ES_5.1_4_6	FGFR1OP2	ES	5.1	4	6	5.1E-05	0.272	-0.029	7.5E-01	6.4E-01	5.0E-01
UCS	FHOD3_ES_27_26_28	FHOD3	ES	27	26	28	2.5E-10	0.552	0.373	NA	NA	NA
UCS	FIP1L1_ES_14_13.1_15	FIP1L1	ES	14	13.1	15	7.8E-04	0.172	-0.101	9.7E-01	6.9E-01	8.1E-01
UCS	FLNB_ES_32.1:32.2_31_33	FLNB	ES	32.1:32.2	31	33	2.4E-05	0.284	0.391	3.9E-01	2.5E-01	9.2E-01
UCS	FLNC_ES_31_30_32	FLNC	ES	31	30	32	2.4E-11	0.593	-0.718	9.8E-01	6.7E-01	4.6E-01
UCS	FMNL1_ES_26_25_27	FMNL1	ES	26	25	27	5.0E-09	0.456	-0.250	1.8E-01	5.8E-01	9.4E-01
UCS	FMNL3_ES_6_5_7	FMNL3	ES	6	5	7	9.7E-04	0.196	0.270	7.5E-01	4.1E-01	2.7E-02
UCS	FNBP1L_RI_16.2_16.1_16.3	FNBP1L	RI	16.2	16.1	16.3	4.8E-06	0.306	0.472	1.1E-01	1.3E-01	2.8E-01
UCS	FOXM1_ES_6_5_7	FOXM1	ES	6	5	7	1.5E-04	0.218	0.157	6.9E-01	5.1E-01	6.0E-01
UCS	FUZ_AD_2.4_2.3_3	FUZ	AD	2.4	2.3	3	8.8E-04	0.169	-0.302	4.8E-01	3.0E-01	4.4E-01
UCS	FXR1_ES_19_18_20	FXR1	ES	19	18	20	7.3E-07	0.351	0.519	6.2E-01	6.2E-01	7.3E-01
UCS	GFPT1_ES_9_8_10	GFPT1	ES	9	8	10	9.2E-09	0.497	0.473	NA	NA	NA
UCS	GLRX3_ES_11.1_10_12.1	GLRX3	ES	11.1	10	12.1	1.1E-05	0.299	0.034	7.9E-01	5.7E-01	9.0E-01
UCS	GMPPA_RI_9.2_9.1_9.3	GMPPA	RI	9.2	9.1	9.3	8.3E-05	0.234	-0.108	1.9E-02	9.6E-02	1.0E-01
UCS	GMPR2_ES_4_3.2_5	GMPR2	ES	4	3.2	5	1.7E-04	0.215	-0.494	2.3E-01	5.0E-01	4.9E-03
UCS	GPC2_ES_3_2_4	GPC2	ES	3	2	4	1.1E-04	0.279	0.603	1.3E-01	2.2E-01	3.0E-01
UCS	H2AFY_ME_7|8_6.3_9	H2AFY	ME	7|8	6.3	9	4.2E-06	0.309	0.605	6.5E-01	1.7E-01	4.7E-01

cancer type	id	Gene Symbol	splice_type	Exon	From.Exon	To.Exon	anova.p	adj.r2	r	p.50	p.25	p.10
UCS	HCFC1R1_ES_2_1.5_3.2	HCFC1R1	ES	2	1.5	3.2	5.7E-05	0.244	0.058	7.6E-01	4.6E-01	8.8E-01
UCS	HIBCH_ES_14_13_15	HIBCH	ES	14	13	15	2.3E-05	0.267	0.187	4.0E-01	9.3E-01	6.7E-01
UCS	HLTF_RI_25.2_25.1_25.3	HLTF	RI	25.2	25.1	25.3	8.1E-04	0.171	0.092	8.3E-02	5.4E-02	6.8E-01
UCS	HM13_ES_12.1_10_13	HM13	ES	12.1	10	13	8.4E-04	0.217	-0.423	6.5E-02	4.1E-01	2.9E-01
UCS	HM13_ES_12.1:12.2_11_13	HM13	ES	12.1:12.2	11	13	6.7E-07	0.353	-0.183	7.6E-02	2.8E-01	1.1E-01
UCS	HNRNPA1_ES_6.2:7.1:7.2:8:9.1_6.1_9.2	HNRNPA1	ES	6.2:7.1:7.2:8:9.1	6.1	9.2	2.1E-04	0.208	-0.091	6.4E-01	2.0E-02	1.8E-02
UCS	HNRNPA1_ES_7.1:7.2:8:9.1:9.2_6.2_10	HNRNPA1	ES	7.1:7.2:8:9.1:9.2	6.2	10	1.1E-04	0.225	-0.041	5.9E-01	1.7E-01	2.9E-01
UCS	HNRNPA3_AD_1.2_1.1_2	HNRNPA3	AD	1.2	1.1	2	1.9E-04	0.211	-0.040	9.1E-01	6.3E-01	6.7E-01
UCS	HOOK2_ES_18_17_19	HOOK2	ES	18	17	19	2.2E-04	0.207	-0.303	6.2E-01	8.9E-01	6.6E-01
UCS	IDH3B_AA_12.1_11_12.2	IDH3B	AA	12.1	11	12.2	1.1E-08	0.440	0.252	6.4E-01	7.4E-01	7.5E-01
UCS	IFI27L1_ES_5:6:7.1_4.3_8	IFI27L1	ES	06:07.1	4.3	8	8.7E-06	0.311	-0.130	2.0E-01	3.4E-01	7.0E-01
UCS	IFI27L1_ES_5:6:7.1:7.2_4.3_8	IFI27L1	ES	5:6:7.1:7.2	4.3	8	2.3E-05	0.300	-0.198	2.1E-01	6.6E-01	5.5E-01
UCS	IFI27L1_ES_6:7.1_4.3_8	IFI27L1	ES	06:07.1	4.3	8	4.9E-07	0.360	0.162	9.7E-01	8.9E-01	5.5E-01
UCS	IFI27L1_ES_6:7.1:7.2_4.3_8	IFI27L1	ES	6:7.1:7.2	4.3	8	8.6E-11	0.530	0.125	8.0E-01	9.8E-01	5.5E-01
UCS	IFI27L1_ES_7.1_6_8	IFI27L1	ES	7.1	6	8	1.6E-04	0.215	0.123	9.3E-01	8.9E-01	9.1E-01
UCS	IFI27L1_ES_7.1:7.2_6_8	IFI27L1	ES	7.1:7.2	6	8	1.4E-07	0.394	-0.032	7.6E-01	8.8E-01	3.0E-01
UCS	IL32_RI_2.2:2.3_2.1_2.4	IL32	RI	2.2:2.3	2.1	2.4	6.1E-05	0.246	0.524	7.5E-01	2.1E-01	3.9E-01
UCS	INCENP_ES_11_10_12	INCENP	ES	11	10	12	5.9E-09	0.453	0.043	2.6E-01	6.8E-01	3.0E-01
UCS	INF2_ES_22_21_23	INF2	ES	22	21	23	1.0E-07	0.395	0.200	8.4E-01	7.4E-01	9.4E-01
UCS	INO80C_ES_3:4.2_1_5.1	INO80C	ES	03:04.2	1	5.1	1.9E-04	0.212	-0.185	9.2E-01	6.9E-01	4.7E-01
UCS	ISOC2_ES_3_2_4.2	ISOC2	ES	3	2	4.2	8.5E-06	0.292	-0.097	1.1E-01	5.8E-01	2.1E-01
UCS	ISOC2_ES_3:4.1_2_4.2	ISOC2	ES	03:04.1	2	4.2	3.8E-04	0.192	-0.122	8.6E-01	5.2E-01	1.1E-01
UCS	ITGA6_ES_27_26_28	ITGA6	ES	27	26	28	6.1E-05	0.267	0.058	3.3E-01	9.4E-01	7.2E-01
UCS	ITGA7_ES_29_28_30	ITGA7	ES	29	28	30	6.2E-11	0.542	0.461	3.7E-01	5.3E-01	1.2E-02
UCS	ITGB1_ME_17|18_16_19	ITGB1	ME	17|18	16	19	6.5E-13	0.688	-0.394	5.0E-01	6.8E-01	2.3E-01
UCS	ITGB4_ES_33_32_34	ITGB4	ES	33	32	34	1.3E-07	0.461	-0.505	3.4E-01	6.4E-01	4.7E-01
UCS	KANSL1_ES_11_10_12	KANSL1	ES	11	10	12	2.6E-04	0.210	0.218	4.1E-01	2.6E-01	4.4E-01
UCS	KANSL3_ES_18_17_19.1	KANSL3	ES	18	17	19.1	9.7E-08	0.426	-0.017	2.1E-01	2.2E-02	2.8E-01
UCS	KIDINS220_ES_28_27_29	KIDINS220	ES	28	27	29	5.5E-07	0.448	-0.068	NA	NA	NA
UCS	KIF13A_ES_40_39_41.1	KIF13A	ES	40	39	41.1	1.9E-05	0.286	-0.522	3.0E-01	2.6E-01	7.0E-01
UCS	KIF1A_ES_27_26_28	KIF1A	ES	27	26	28	7.0E-05	0.283	-0.028	2.7E-01	9.8E-01	5.5E-01
UCS	KIFC3_ES_28_27_29	KIFC3	ES	28	27	29	3.1E-08	0.420	-0.080	6.0E-01	3.5E-01	5.4E-01
UCS	KLC1_AD_13.3_13.2_14.1	KLC1	AD	13.3	13.2	14.1	2.9E-04	0.200	-0.397	5.7E-02	3.4E-02	8.1E-01
UCS	KLC1_ES_13.3:14.1_13.2_14.3	KLC1	ES	13.3:14.1	13.2	14.3	8.5E-05	0.249	-0.234	7.5E-02	4.1E-01	9.3E-01
UCS	KLC1_ES_13.3:14.1:14.2_13.2_14.3	KLC1	ES	13.3:14.1:14.2	13.2	14.3	4.3E-06	0.340	-0.483	2.8E-01	1.4E-01	9.1E-01
UCS	KLC1_ES_15_13.2_18	KLC1	ES	15	13.2	18	2.3E-04	0.207	0.261	5.7E-01	7.0E-02	8.2E-02
UCS	KLC1_ES_15_13.3_18	KLC1	ES	15	13.3	18	7.1E-04	0.175	0.354	7.4E-01	9.7E-01	1.6E-01
UCS	KLC1_ES_16_15_18	KLC1	ES	16	15	18	4.9E-05	0.248	-0.292	5.4E-01	2.9E-02	3.7E-01
UCS	KREMEN2_AA_7.3:7.4_7.1_7.5	KREMEN2	AA	7.3:7.4	7.1	7.5	5.3E-04	0.219	-0.365	1.6E-01	1.3E-01	3.5E-01
UCS	KRT15_RI_7.2_7.1_7.3	KRT15	RI	7.2	7.1	7.3	5.9E-04	0.180	-0.873	2.2E-01	5.6E-01	9.4E-01

cancer type	id	Gene Symbol	splice_type	Exon	From.Exon	To.Exon	anova.p	adj.r2	r	p.50	p.25	p.10
UCS	KTN1_ES_42_41_43	KTN1	ES	42	41	43	5.1E-05	0.247	0.091	9.4E-01	9.9E-01	6.9E-02
UCS	LAMTOR3_ES_2_1_3	LAMTOR3	ES	2	1	3	1.3E-04	0.249	0.205	2.8E-01	1.8E-01	5.5E-02
UCS	LAS1L_ES_9_8_10	LAS1L	ES	9	8	10	4.9E-05	0.248	-0.027	9.9E-01	7.3E-01	2.7E-01
UCS	LIMCH1_ES_36_35_37	LIMCH1	ES	36	35	37	1.0E-12	0.649	0.312	5.8E-01	2.5E-01	2.8E-01
UCS	LMO7_ES_10:11_9_12	LMO7	ES	10:11	9	12	9.5E-12	0.674	0.369	2.8E-01	5.3E-01	4.7E-01
UCS	LRRFIP1_ES_15_14_16	LRRFIP1	ES	15	14	16	8.2E-10	0.517	0.431	1.2E-01	2.1E-01	2.9E-02
UCS	LRRFIP1_ES_18:19_17_20	LRRFIP1	ES	18:19	17	20	1.2E-11	0.575	0.488	3.8E-01	9.6E-01	8.3E-01
UCS	LRRFIP1_ES_4:5:6:7_3_13	LRRFIP1	ES	4:5:6:7	3	13	9.9E-04	0.199	0.085	3.4E-01	6.3E-01	9.0E-01
UCS	LRRFIP1_ES_4:5:6:7:8:9:10:11:12_3_13	LRRFIP1	ES	:5:6:7:8:9:10:11:1	3	13	4.9E-06	0.374	0.474	6.8E-01	5.5E-01	4.1E-01
UCS	LRRFIP2_ES_23:24_22_25	LRRFIP2	ES	23:24	22	25	8.1E-08	0.399	0.149	5.9E-01	1.6E-01	3.4E-01
UCS	LTBP1_ES_26_25_27	LTBP1	ES	26	25	27	1.1E-05	0.291	-0.124	8.4E-01	9.1E-01	7.6E-01
UCS	MACF1_ES_105_104_106	MACF1	ES	105	104	106	1.5E-10	0.520	0.477	8.2E-01	9.5E-01	3.7E-01
UCS	MACF1_ES_107_106_108	MACF1	ES	107	106	108	5.7E-06	0.302	0.044	2.3E-02	7.3E-01	8.6E-01
UCS	MACF1_ES_77_76.1_79	MACF1	ES	77	76.1	79	1.2E-07	0.488	-0.408	7.1E-01	9.1E-01	6.3E-01
UCS	MADD_ES_23.2_22_24	MADD	ES	23.2	22	24	2.6E-05	0.313	-0.147	4.7E-01	3.1E-01	2.6E-02
UCS	MADD_ES_28_27_29	MADD	ES	28	27	29	3.0E-09	0.472	-0.237	7.3E-01	4.7E-01	5.1E-01
UCS	MAGI1_ES_24_23.1_25	MAGI1	ES	24	23.1	25	3.7E-06	0.396	0.257	6.9E-01	5.7E-01	7.4E-01
UCS	MAP3K7_ES_11_10_12	MAP3K7	ES	11	10	12	1.9E-04	0.212	-0.240	4.3E-02	3.8E-01	3.4E-01
UCS	MAP4_AA_24.1_23_24.2	MAP4	AA	24.1	23	24.2	4.0E-05	0.257	-0.277	2.3E-01	2.6E-01	9.3E-01
UCS	MAP4_ES_12.1:12.2_11_14	MAP4	ES	12.1:12.2	11	14	1.2E-09	0.483	0.252	5.6E-01	2.4E-01	3.7E-01
UCS	MAP4_ES_19:20_18_21	MAP4	ES	19:20	18	21	1.0E-07	0.394	-0.444	3.2E-01	1.1E-01	2.4E-01
UCS	MAP4K4_ES_17:18_16.2_19	MAP4K4	ES	17:18	16.2	19	4.4E-05	0.254	0.441	1.9E-01	2.1E-02	1.5E-01
UCS	MAP4K4_ES_18_16.2_19	MAP4K4	ES	18	16.2	19	2.3E-06	0.334	0.496	8.7E-02	3.1E-01	3.7E-01
UCS	MAP4K4_ES_18_17_19	MAP4K4	ES	18	17	19	1.7E-06	0.370	0.511	4.5E-03	9.8E-02	1.1E-01
UCS	MAPK10_ME_12|13_11_14	MAPK10	ME	12|13	11	14	1.9E-04	0.257	0.166	4.6E-01	4.4E-02	2.2E-01
UCS	MAPK8IP3_ES_10_9_11	MAPK8IP3	ES	10	9	11	1.5E-05	0.317	-0.006	4.4E-01	7.8E-01	2.4E-01
UCS	MARK2_ES_18_17_19	MARK2	ES	18	17	19	6.4E-04	0.187	0.640	1.5E-01	1.3E-01	2.9E-02
UCS	MARK2_ES_18_17_20	MARK2	ES	18	17	20	9.5E-04	0.193	0.335	5.6E-01	9.5E-01	2.1E-01
UCS	MARK2_ES_18:19_17_20	MARK2	ES	18:19	17	20	2.9E-07	0.413	0.513	8.1E-01	9.0E-01	6.3E-01
UCS	MARK2_ES_19_17_20	MARK2	ES	19	17	20	1.5E-07	0.386	0.241	1.8E-01	5.0E-01	8.7E-01
UCS	MARK3_ES_17:18_16_19	MARK3	ES	17:18	16	19	1.1E-04	0.226	0.030	6.4E-01	9.2E-02	6.3E-01
UCS	MARK3_ES_18_16_19	MARK3	ES	18	16	19	1.6E-06	0.332	0.040	3.6E-02	1.1E-02	3.7E-01
UCS	MARK3_ES_18_17_19	MARK3	ES	18	17	19	1.9E-04	0.219	0.267	1.0E-01	3.7E-01	3.4E-01
UCS	MARK4_ES_16_15_17	MARK4	ES	16	15	17	1.4E-11	0.559	0.029	5.5E-01	6.1E-01	2.9E-01
UCS	MBD1_ES_10_9_11	MBD1	ES	10	9	11	5.0E-10	0.499	-0.193	8.1E-01	3.4E-01	8.4E-01
UCS	MBD1_ES_13.1:13.2_12_14	MBD1	ES	13.1:13.2	12	14	5.1E-06	0.305	0.257	6.4E-01	8.4E-01	2.5E-01
UCS	MED22_RI_5.2_5.1_5.3	MED22	RI	5.2	5.1	5.3	4.9E-12	0.576	-0.068	5.8E-01	5.8E-01	3.2E-01
UCS	MEF2D_ES_10_9_11	MEF2D	ES	10	9	11	4.6E-13	0.617	0.728	9.5E-01	6.1E-01	4.2E-01
UCS	MEF2D_ME_5|6_4_7	MEF2D	ME	5|6	4	7	2.5E-09	0.476	-0.710	9.6E-01	8.8E-01	8.5E-01
UCS	MEGF6_ES_26:27_25_28	MEGF6	ES	26:27:00	25	28	9.9E-05	0.241	-0.149	8.5E-01	7.6E-01	8.0E-01

cancer type	id	Gene Symbol	splice_type	Exon	From.Exon	To.Exon	anova.p	adj.r2	r	p.50	p.25	p.10
UCS	MEIS1_ES_12.3_12.1_12.5	MEIS1	ES	12.3	12.1	12.5	6.8E-04	0.189	0.097	6.9E-01	7.8E-01	1.6E-01
UCS	MFF_ES_10_7_11	MFF	ES	10	7	11	1.1E-05	0.286	0.160	4.4E-01	1.5E-01	3.8E-01
UCS	MFF_ES_8:9_7_10	MFF	ES	8:09	7	10	1.3E-04	0.222	-0.051	1.2E-01	1.6E-01	3.9E-02
UCS	MFF_ES_9_7_10	MFF	ES	9	7	10	8.0E-06	0.294	-0.159	3.2E-01	1.2E-01	3.4E-02
UCS	MINK1_ES_19_18_20	MINK1	ES	19	18	20	3.5E-04	0.194	0.082	8.4E-01	5.5E-01	2.0E-01
UCS	MKNK1_ES_4_3_5	MKNK1	ES	4	3	5	1.7E-07	0.389	-0.089	9.7E-01	4.6E-01	7.9E-01
UCS	MPND_ES_11.1:11.2_10_12	MPND	ES	11.1:11.2	10	12	9.3E-04	0.176	-0.011	1.9E-01	5.0E-01	7.2E-01
UCS	MPND_ES_11.2_10_12	MPND	ES	11.2	10	12	1.8E-08	0.430	0.297	4.2E-01	3.7E-01	2.5E-01
UCS	MPRIP_ES_24_23_25	MPRIP	ES	24	23	25	2.4E-04	0.205	0.479	6.8E-01	8.8E-01	1.1E-01
UCS	MRPL55_AA_2.2:2.3:2.4_1.1_2.5	MRPL55	AA	2.2:2.3:2.4	1.1	2.5	9.9E-05	0.229	0.100	9.3E-01	2.7E-01	9.9E-01
UCS	MTA1_ES_18:19_17_20	MTA1	ES	18:19	17	20	8.4E-04	0.189	0.051	4.9E-01	8.4E-01	9.0E-01
UCS	MTCH1_AD_8.2_8.1_9	MTCH1	AD	8.2	8.1	9	3.9E-07	0.365	0.242	9.2E-01	3.9E-01	6.1E-01
UCS	MTMR3_ES_20_19_21	MTMR3	ES	20	19	21	2.1E-09	0.479	-0.114	2.8E-01	3.4E-03	4.0E-01
UCS	MTSS1L_ES_7_6_8	MTSS1L	ES	7	6	8	2.5E-06	0.366	0.512	6.1E-01	3.1E-01	2.6E-01
UCS	MXRA7_ES_5_4_6	MXRA7	ES	5	4	6	5.2E-09	0.462	0.184	7.9E-01	3.8E-01	6.1E-01
UCS	MYL6_ES_2.1_1.4_3.1	MYL6	ES	2.1	1.4	3.1	3.5E-05	0.256	0.079	1.9E-01	6.4E-01	3.1E-01
UCS	MYL6_RI_1.2_1.1_1.3	MYL6	RI	1.2	1.1	1.3	1.0E-03	0.165	0.056	8.0E-01	7.6E-01	8.9E-01
UCS	MYO18A_ES_41_40_42	MYO18A	ES	41	40	42	1.4E-06	0.336	0.197	2.7E-01	3.4E-01	9.9E-01
UCS	MYO19_ES_23_22_24	MYO19	ES	23	22	24	1.1E-05	0.291	-0.053	9.5E-01	1.3E-02	3.6E-01
UCS	MYO1B_ES_23:24_22_25	MYO1B	ES	23:24	22	25	6.9E-04	0.179	-0.043	9.9E-01	4.5E-02	5.0E-01
UCS	MZT2B_ES_2:3_1_4	MZT2B	ES	2:03	1	4	1.8E-08	0.430	-0.405	7.1E-01	9.2E-01	2.9E-01
UCS	N4BP2L1_ME_5|6_4_7.1	N4BP2L1	ME	5|6	4	7.1	4.9E-06	0.374	0.099	4.7E-01	1.3E-01	8.4E-01
UCS	NAP1L1_ES_7.2:7.4_6_7.5	NAP1L1	ES	7.2:7.4	6	7.5	8.7E-09	0.451	0.268	3.0E-02	3.0E-02	1.0E-01
UCS	NAP1L1_RI_7.3_7.2_7.4	NAP1L1	RI	7.3	7.2	7.4	2.2E-07	0.383	-0.500	4.8E-01	5.4E-02	1.6E-01
UCS	NDEL1_ES_11_10_12.1	NDEL1	ES	11	10	12.1	2.1E-04	0.212	-0.009	1.0E-01	6.9E-01	1.1E-01
UCS	NDUFAF6_ME_12|13_11_14	NDUFAF6	ME	12|13	11	14	1.2E-04	0.232	-0.215	4.1E-01	2.3E-01	5.4E-01
UCS	NDUFS8_ES_3:4.1:4.2_1_5	NDUFS8	ES	3:4.1:4.2	1	5	4.9E-05	0.269	-0.110	9.0E-01	6.2E-01	2.9E-01
UCS	NDUFV3_ES_3_2_4	NDUFV3	ES	3	2	4	3.1E-04	0.198	-0.440	6.1E-01	4.8E-01	3.5E-01
UCS	NEXN_RI_14.2_14.1_14.3	NEXN	RI	14.2	14.1	14.3	4.3E-14	0.732	-0.760	4.1E-01	1.0E+00	3.5E-01
UCS	NFATC3_ES_10_9.1_13	NFATC3	ES	10	9.1	13	1.8E-05	0.273	0.000	9.0E-02	3.6E-01	9.1E-01
UCS	NFYA_ES_3_2_4	NFYA	ES	3	2	4	7.0E-05	0.263	0.250	7.5E-02	1.9E-01	8.4E-01
UCS	NKAIN4_ES_8_7_9	NKAIN4	ES	8	7	9	3.9E-04	0.229	-0.331	1.6E-01	2.6E-01	9.9E-01
UCS	NPTN_ES_2:3:4_1_5	NPTN	ES	2:03:04	1	5	2.6E-04	0.252	0.004	5.4E-01	6.4E-01	3.9E-01
UCS	NSFL1C_ES_5.2_4_7.2	NSFL1C	ES	5.2	4	7.2	9.6E-08	0.396	-0.170	2.7E-01	3.8E-01	6.2E-01
UCS	NUMB_ES_13_12_14	NUMB	ES	13	12	14	1.5E-05	0.296	0.402	2.9E-01	4.5E-01	9.7E-01
UCS	OGG1_ES_5:6.1_4_8	OGG1	ES	05:06.1	4	8	2.7E-04	0.209	0.066	2.5E-01	3.3E-01	2.0E-01
UCS	OSBPL9_ES_17_15_18	OSBPL9	ES	17	15	18	4.3E-06	0.324	-0.012	4.9E-01	3.2E-01	6.9E-01
UCS	OXR1_ES_16_14_17	OXR1	ES	16	14	17	3.4E-04	0.225	-0.048	6.2E-01	7.1E-03	2.2E-01
UCS	PAAF1_ES_4.2_3.2_5	PAAF1	ES	4.2	3.2	5	2.6E-04	0.243	0.095	3.3E-01	2.3E-02	3.4E-02
UCS	PALLD_ES_17_16_18	PALLD	ES	17	16	18	2.5E-10	0.592	0.249	4.8E-01	3.9E-01	7.7E-01

cancer type	id	Gene Symbol	splice_type	Exon	From.Exon	To.Exon	anova.p	adj.r2	r	p.50	p.25	p.10
UCS	PALLD_ES_25_24_26	PALLD	ES	25	24	26	4.4E-06	0.309	0.169	3.1E-01	4.0E-01	6.0E-01
UCS	PAM_ES_14_13_15	PAM	ES	14	13	15	4.6E-05	0.249	-0.316	7.9E-01	9.4E-01	3.0E-01
UCS	PARD3_ES_20_19.3_21	PARD3	ES	20	19.3	21	1.9E-07	0.381	-0.214	3.3E-02	1.1E-02	4.1E-01
UCS	PBRM1_ES_28:29_27_30	PBRM1	ES	28:29:00	27	30	8.4E-05	0.258	-0.112	7.7E-02	1.7E-01	7.3E-01
UCS	PBRM1_ES_29_27_30	PBRM1	ES	29	27	30	1.7E-06	0.370	-0.161	1.6E-01	3.0E-01	3.6E-01
UCS	PCBP2_ES_15_14.1_16.1	PCBP2	ES	15	14.1	16.1	7.6E-04	0.173	-0.195	4.6E-01	1.1E-01	2.1E-01
UCS	PCBP2_ES_15_14.1_16.2	PCBP2	ES	15	14.1	16.2	9.6E-05	0.230	-0.188	9.3E-01	3.9E-01	7.8E-01
UCS	PCMT1_AD_9.2_9.1_10	PCMT1	AD	9.2	9.1	10	1.2E-04	0.225	0.161	7.4E-01	4.9E-01	3.3E-02
UCS	PDCD5_AA_5.1:5.2_4.2_5.3	PDCD5	AA	5.1:5.2	4.2	5.3	1.8E-05	0.273	-0.311	6.8E-01	3.3E-01	2.7E-01
UCS	PDCD6IP_AA_8.1_7_8.2	PDCD6IP	AA	8.1	7	8.2	1.5E-05	0.323	-0.121	3.3E-01	7.3E-01	8.1E-01
UCS	PDE4DIP_AA_52.1_51_52.2	PDE4DIP	AA	52.1	51	52.2	3.1E-08	0.426	-0.642	4.5E-01	4.9E-01	3.0E-01
UCS	PDE4DIP_AD_32.2_32.1_33	PDE4DIP	AD	32.2	32.1	33	2.1E-04	0.254	-0.718	1.9E-01	2.3E-01	4.6E-01
UCS	PDE4DIP_RI_30.2_30.1_30.3	PDE4DIP	RI	30.2	30.1	30.3	3.4E-07	0.409	-0.672	4.2E-01	6.8E-01	6.1E-01
UCS	PDLIM5_AA_8.1_6.1_8.2	PDLIM5	AA	8.1	6.1	8.2	3.2E-14	0.660	-0.110	9.2E-01	8.6E-01	3.5E-01
UCS	PDLIM5_ES_13_8.2_14	PDLIM5	ES	13	8.2	14	6.4E-15	0.739	0.150	8.5E-01	7.5E-01	2.5E-01
UCS	PDLIM7_ES_6_5_7	PDLIM7	ES	6	5	7	1.8E-08	0.431	0.121	8.1E-01	5.9E-01	8.3E-01
UCS	PFKFB3_ES_18.1_17_20	PFKFB3	ES	18.1	17	20	6.2E-05	0.276	-0.148	6.4E-01	6.6E-01	4.0E-01
UCS	PI4KB_ES_2_1_4	PI4KB	ES	2	1	4	2.6E-04	0.221	0.000	6.6E-01	6.2E-01	3.6E-01
UCS	PI4KB_ES_3_2_4	PI4KB	ES	3	2	4	1.4E-04	0.247	0.100	4.1E-01	1.9E-01	1.9E-01
UCS	PICALM_ES_19_18_20	PICALM	ES	19	18	20	2.6E-10	0.511	-0.106	6.3E-01	7.7E-01	4.3E-01
UCS	PICALM_ES_19:20_18_21	PICALM	ES	19:20	18	21	1.7E-08	0.432	0.048	6.9E-01	8.1E-01	1.8E-01
UCS	PKP4_ES_25_24_26	PKP4	ES	25	24	26	3.3E-05	0.257	-0.197	5.8E-01	7.1E-01	5.9E-01
UCS	PLEKHA1_ES_15.1_14_16	PLEKHA1	ES	15.1	14	16	4.2E-06	0.371	-0.315	3.6E-01	1.2E-01	9.8E-01
UCS	PLEKHA1_ES_15.1:15.2_14_16	PLEKHA1	ES	15.1:15.2	14	16	2.6E-04	0.264	-0.174	7.4E-02	5.0E-02	2.2E-01
UCS	PLEKHG3_AD_15.2_15.1_16	PLEKHG3	AD	15.2	15.1	16	8.6E-05	0.249	-0.564	4.5E-01	7.6E-01	1.0E+00
UCS	PLSCR4_ES_7:8_6_9	PLSCR4	ES	7:08	6	9	7.8E-04	0.211	0.040	4.0E-01	3.8E-01	3.7E-01
UCS	POMGNT2_ES_2_1_3	POMGNT2	ES	2	1	3	1.1E-06	0.407	0.183	4.9E-02	3.8E-02	3.6E-01
UCS	PORCN_ES_7:8_6_9	PORCN	ES	7:08	6	9	1.1E-05	0.326	-0.181	7.8E-01	6.9E-01	5.4E-01
UCS	PPHLN1_ES_10_9_11	PPHLN1	ES	10	9	11	2.9E-08	0.421	-0.354	2.1E-01	2.3E-01	6.0E-03
UCS	PPHLN1_ES_6_5_7	PPHLN1	ES	6	5	7	1.5E-04	0.217	-0.389	3.7E-01	5.5E-01	9.9E-01
UCS	PPP1CA_ES_2.1:2.2_1_3	PPP1CA	ES	2.1:2.2	1	3	7.1E-04	0.175	-0.087	3.4E-01	4.0E-01	8.9E-01
UCS	PPP3CA_ES_13_12_14	PPP3CA	ES	13	12	14	1.9E-08	0.475	-0.018	7.3E-01	8.5E-01	6.0E-01
UCS	PPP3CC_ES_11_10_12	PPP3CC	ES	11	10	12	4.6E-04	0.193	-0.011	NA	NA	NA
UCS	PQBP1_AD_1.2_1.1_2.3	PQBP1	AD	1.2	1.1	2.3	7.9E-06	0.308	-0.096	1.7E-01	1.1E-01	2.5E-01
UCS	PQBP1_AD_1.2:1.3_1.1_2.3	PQBP1	AD	1.2:1.3	1.1	2.3	6.8E-04	0.176	-0.218	2.5E-01	6.1E-01	5.1E-01
UCS	PQLC1_ES_6:7_5_9	PQLC1	ES	6:07	5	9	1.5E-05	0.277	-0.209	1.3E-01	3.6E-01	2.0E-01
UCS	PQLC1_ES_6:7:9_5_10	PQLC1	ES	6:07:09	5	10	1.3E-05	0.281	-0.155	1.3E-01	1.7E-01	2.0E-01
UCS	PQLC1_ES_7_6_9	PQLC1	ES	7	6	9	2.0E-04	0.210	-0.102	3.1E-01	1.4E-01	1.6E-02
UCS	PRKAR1A_RI_3.3_3.2_3.4	PRKAR1A	RI	3.3	3.2	3.4	2.2E-04	0.269	-0.301	9.9E-01	6.9E-01	6.4E-01
UCS	PTK2_ES_22_21.2_23	PTK2	ES	22	21.2	23	1.6E-08	0.479	-0.178	8.4E-01	5.5E-01	4.8E-01

cancer type	id	Gene Symbol	splice_type	Exon	From.Exon	To.Exon	anova.p	adj.r2	r	p.50	p.25	p.10
UCS	PTS_ES_4_3_5	PTS	ES	4	3	5	9.7E-11	0.528	0.208	5.3E-01	6.1E-01	2.5E-01
UCS	PTTG1_RI_2.2_2.1_2.3	PTTG1	RI	2.2	2.1	2.3	2.3E-04	0.206	0.340	8.1E-01	5.4E-01	5.8E-02
UCS	PXMP4_ES_3.2_2_4	PXMP4	ES	3.2	2	4	1.8E-04	0.217	-0.095	9.2E-01	9.3E-01	9.5E-01
UCS	QKI_AA_8.1_7_8.2	QKI	AA	8.1	7	8.2	4.8E-08	0.441	-0.656	2.3E-01	2.9E-02	1.2E-01
UCS	R3HDM2_ES_14_13_15	R3HDM2	ES	14	13	15	2.9E-09	0.529	0.030	NA	NA	NA
UCS	RAB11FIP3_ES_7_6_8	RAB11FIP3	ES	7	6	8	9.9E-04	0.166	-0.182	7.1E-02	3.5E-02	1.1E-01
UCS	RAI14_ES_18_17_19	RAI14	ES	18	17	19	6.6E-06	0.334	0.118	6.1E-02	3.7E-01	7.1E-01
UCS	RBFOX2_ES_12_11.2_14	RBFOX2	ES	12	11.2	14	8.7E-05	0.232	-0.611	9.9E-01	3.8E-01	8.3E-02
UCS	RBFOX2_ES_12:13_11.2_14	RBFOX2	ES	12:13	11.2	14	6.0E-05	0.288	0.464	9.0E-01	4.5E-01	4.6E-01
UCS	RBM26_ES_14_13.2_15	RBM26	ES	14	13.2	15	3.9E-07	0.387	0.115	6.0E-02	1.3E-01	2.1E-01
UCS	RIMKLB_ES_3_2_4	RIMKLB	ES	3	2	4	1.6E-06	0.377	-0.089	7.1E-01	1.5E-02	3.6E-01
UCS	RNF115_ES_3_2_4	RNF115	ES	3	2	4	1.0E-05	0.297	0.252	8.0E-01	2.2E-01	6.1E-01
UCS	RNF32_AA_12.1_11_12.2	RNF32	AA	12.1	11	12.2	2.1E-05	0.297	-0.523	9.0E-01	3.5E-01	8.9E-01
UCS	RNF34_ES_2_1_3	RNF34	ES	2	1	3	5.0E-04	0.205	0.140	4.8E-01	2.5E-01	5.2E-01
UCS	RNMT_AA_12.1:12.2_11_12.3	RNMT	AA	12.1:12.2	11	12.3	2.0E-04	0.210	-0.376	7.3E-01	3.9E-01	5.5E-01
UCS	ROGDI_ES_4.1:4.2_3_5	ROGDI	ES	4.1:4.2	3	5	3.6E-05	0.287	0.515	3.1E-01	6.4E-01	8.0E-01
UCS	RPS15_ES_2:3.2_1.5_3.3	RPS15	ES	02:03.2	1.5	3.3	6.9E-05	0.239	-0.050	6.8E-01	4.9E-01	6.4E-01
UCS	RPS20_AA_2.1:2.2_1.4_2.3	RPS20	AA	2.1:2.2	1.4	2.3	9.3E-09	0.444	-0.387	6.6E-01	5.6E-01	6.4E-01
UCS	RPS21_AA_3.3:3.4_3.1_3.5	RPS21	AA	3.3:3.4	3.1	3.5	7.8E-06	0.294	-0.160	1.2E-01	5.7E-01	8.8E-01
UCS	RPS21_AD_3.2:3.3_3.1_3.5	RPS21	AD	3.2:3.3	3.1	3.5	1.3E-05	0.282	-0.243	2.9E-01	3.0E-01	6.9E-02
UCS	RPS25_ES_2.1:2.2_1_3.1	RPS25	ES	2.1:2.2	1	3.1	2.2E-04	0.214	-0.357	3.2E-01	3.5E-01	8.8E-01
UCS	RRBP1_ES_2_1_3.1	RRBP1	ES	2	1	3.1	5.6E-04	0.188	0.169	8.4E-01	9.9E-01	8.2E-01
UCS	RTN2_ES_5_4.1_6	RTN2	ES	5	4.1	6	9.8E-04	0.171	0.377	6.6E-01	3.4E-01	6.4E-01
UCS	RTN4_ES_5:6.1:6.2_2.5_8	RTN4	ES	5:6.1:6.2	2.5	8	3.2E-12	0.589	0.626	3.3E-02	5.8E-01	3.6E-01
UCS	RTN4_ES_6.1:6.2_5_8	RTN4	ES	6.1:6.2	5	8	2.5E-04	0.204	0.262	3.0E-01	3.2E-01	4.6E-01
UCS	SAMD4A_ES_4_3_5	SAMD4A	ES	4	3	5	9.0E-07	0.399	0.426	6.2E-01	9.9E-01	2.8E-01
UCS	SCAMP5_ES_7_6_8.2	SCAMP5	ES	7	6	8.2	8.2E-04	0.214	-0.128	2.5E-01	5.3E-01	5.3E-01
UCS	SCARB1_ES_14_13.2_15	SCARB1	ES	14	13.2	15	2.8E-07	0.372	0.129	5.2E-01	1.1E-01	7.0E-03
UCS	SCRIB_ES_17_16_18	SCRIB	ES	17	16	18	9.8E-05	0.237	0.119	1.3E-01	5.5E-01	2.0E-01
UCS	SCRIB_ES_36_35_37	SCRIB	ES	36	35	37	2.1E-04	0.209	0.088	1.2E-02	6.4E-02	9.2E-01
UCS	SEC14L1_ES_22.2:22.4_22.1_22.5	SEC14L1	ES	22.2:22.4	22.1	22.5	3.1E-07	0.370	0.099	5.7E-01	5.5E-01	6.9E-02
UCS	SEC14L1_RI_22.2:22.3:22.4_22.1_22.5	SEC14L1	RI	22.2:22.3:22.4	22.1	22.5	3.1E-04	0.198	0.099	3.9E-01	3.8E-01	2.7E-01
UCS	SEC24C_ES_8_7_9	SEC24C	ES	8	7	9	5.2E-06	0.365	0.106	4.8E-01	2.9E-01	1.7E-01
UCS	SEC31A_AD_26.2_26.1_28	SEC31A	AD	26.2	26.1	28	9.2E-08	0.397	-0.286	1.0E-01	1.7E-01	3.1E-01
UCS	SEH1L_AA_9.1_8_9.2	SEH1L	AA	9.1	8	9.2	5.0E-08	0.410	0.096	2.3E-01	4.5E-01	5.1E-01
UCS	SENP6_ES_8_6_9	SENP6	ES	8	6	9	8.2E-05	0.290	-0.014	5.5E-01	4.9E-02	7.6E-02
UCS	SEPT2_ES_3:4:7_2_9	SEPT2	ES	3:04:07	2	9	8.3E-04	0.193	-0.336	3.8E-01	1.1E-01	3.0E-01
UCS	SEPT2_ES_4:7_2_9	SEPT2	ES	4:07	2	9	9.5E-05	0.285	-0.045	6.8E-01	3.1E-01	3.4E-01
UCS	SEPT4_ES_7.1:7.2_3_8	SEPT4	ES	7.1:7.2	3	8	3.8E-05	0.326	0.397	1.7E-01	2.8E-01	8.5E-01
UCS	SEPT6_ES_12_11.1_13.1	SEPT6	ES	12	11.1	13.1	3.8E-05	0.254	-0.279	1.1E-01	4.8E-01	7.5E-01

cancer type	id	Gene Symbol	splice_type	Exon	From.Exon	To.Exon	anova.p	adj.r2	r	p.50	p.25	p.10
UCS	SEPT6_ES_12:13.1_11.1_13.2	SEPT6	ES	12:13.1	11.1	13.2	2.8E-04	0.201	-0.248	3.3E-01	6.3E-01	9.5E-01
UCS	SERP2_ES_4_3_6	SERP2	ES	4	3	6	4.7E-06	0.307	-0.295	5.6E-01	9.5E-03	4.7E-03
UCS	SHF_ES_7_6_8.1	SHF	ES	7	6	8.1	6.1E-07	0.355	-0.075	4.6E-01	1.3E-01	9.2E-01
UCS	SHF_ES_7:8.1:8.2:9.1_6_10	SHF	ES	7:8.1:8.2:9.1	6	10	8.8E-08	0.409	-0.005	9.5E-01	1.3E-01	3.1E-01
UCS	SHF_ES_7:8.1:9.1_6_10	SHF	ES	7:8.1:9.1	6	10	1.1E-05	0.295	0.055	4.4E-01	1.5E-01	9.7E-01
UCS	SIDT2_ES_11_10_12	SIDT2	ES	11	10	12	5.2E-04	0.229	0.022	8.6E-01	9.1E-01	3.4E-01
UCS	SIPA1L2_ES_20_19_21	SIPA1L2	ES	20	19	21	3.8E-07	0.382	0.214	5.6E-01	4.6E-01	2.3E-02
UCS	SLC25A14_ES_2.2:2.3_1_3	SLC25A14	ES	2.2:2.3	1	3	7.5E-06	0.348	-0.087	7.9E-01	7.3E-01	6.9E-01
UCS	SLC25A3_AD_2.2_2.1_4.1	SLC25A3	AD	2.2	2.1	4.1	1.0E-07	0.400	-0.366	7.8E-01	6.0E-01	5.9E-01
UCS	SLC29A1_ES_4.2_3.1_5.3	SLC29A1	ES	4.2	3.1	5.3	5.5E-04	0.214	-0.196	3.8E-01	8.4E-01	8.1E-01
UCS	SLC37A4_ES_11_10_12	SLC37A4	ES	11	10	12	7.0E-08	0.402	-0.545	3.4E-01	3.6E-01	3.8E-01
UCS	SLC38A6_ES_4_3_5	SLC38A6	ES	4	3	5	2.4E-04	0.265	-0.122	8.7E-04	7.1E-05	2.3E-02
UCS	SLC50A1_ES_5_3_6	SLC50A1	ES	5	3	6	1.5E-04	0.222	0.047	4.9E-01	2.3E-01	2.0E-01
UCS	SLK_ES_13_12_14	SLK	ES	13	12	14	1.8E-04	0.225	-0.101	5.7E-01	6.3E-01	6.6E-01
UCS	SMAP1_ES_6_5.2_7	SMAP1	ES	6	5.2	7	6.4E-09	0.477	0.217	4.6E-01	5.5E-01	6.4E-01
UCS	SMARCC2_AD_28.2:28.3_28.1_29	SMARCC2	AD	28.2:28.3	28.1	29	1.6E-06	0.333	0.203	1.1E-01	4.5E-01	1.5E-01
UCS	SMIM19_AD_2.2_2.1_3.2	SMIM19	AD	2.2	2.1	3.2	4.8E-04	0.186	-0.134	8.2E-01	5.7E-01	9.8E-01
UCS	SMPD4_ES_11_10_14	SMPD4	ES	11	10	14	6.9E-04	0.179	0.027	6.8E-01	3.0E-01	7.0E-01
UCS	SMPD4_ES_11:12:14_10_15.1	SMPD4	ES	11:12:14	10	15.1	5.2E-04	0.197	0.021	4.3E-01	9.7E-02	2.7E-01
UCS	SMPD4_ES_11:13:14_10_15.1	SMPD4	ES	11:13:14	10	15.1	1.1E-05	0.321	-0.041	9.0E-02	6.5E-02	5.0E-01
UCS	SOS1_ES_24_23_25	SOS1	ES	24	23	25	7.3E-05	0.262	-0.119	2.0E-01	1.8E-01	2.9E-03
UCS	SPOP_ME_2|3_1_6	SPOP	ME	2|3	1	6	3.4E-04	0.249	-0.087	7.5E-02	1.7E-01	9.7E-01
UCS	SPTAN1_ES_23_22_24	SPTAN1	ES	23	22	24	1.1E-05	0.327	-0.214	8.8E-01	6.6E-01	3.0E-01
UCS	SRSF5_AD_2.2:2.3_2.1_4	SRSF5	AD	2.2:2.3	2.1	4	8.5E-05	0.233	-0.100	8.3E-01	6.9E-01	3.5E-01
UCS	SRSF5_AD_2.3_2.2_4	SRSF5	AD	2.3	2.2	4	4.4E-05	0.250	-0.122	1.1E-01	6.6E-01	4.3E-01
UCS	SSFA2_AD_13.2_13.1_14	SSFA2	AD	13.2	13.1	14	1.6E-08	0.464	-0.025	4.6E-01	3.1E-01	4.1E-01
UCS	ST7_ES_10_9_11.1	ST7	ES	10	9	11.1	2.8E-04	0.204	0.186	2.5E-01	3.0E-01	4.5E-01
UCS	STAU1_ES_4_3_5	STAU1	ES	4	3	5	3.4E-06	0.324	-0.057	8.6E-01	3.1E-01	1.6E-01
UCS	STRA13_ES_3.1_2_4.1	STRA13	ES	3.1	2	4.1	1.4E-04	0.219	-0.137	9.8E-01	4.8E-01	7.8E-01
UCS	STRN4_AA_10.1_9.2_10.2	STRN4	AA	10.1	9.2	10.2	5.8E-05	0.247	-0.076	8.0E-01	6.2E-01	1.9E-01
UCS	SVIL_ES_10:11:12_9_13	SVIL	ES	10:11:12	9	13	5.0E-04	0.209	0.298	7.2E-01	4.6E-01	9.4E-01
UCS	SYNE2_ES_118_117.2_119.2	SYNE2	ES	118	117.2	119.2	4.9E-05	0.264	-0.441	2.9E-01	5.4E-01	8.2E-01
UCS	SYNM_RI_6.2_6.1_6.3	SYNM	RI	6.2	6.1	6.3	1.2E-05	0.292	-0.708	3.9E-01	8.7E-01	7.7E-01
UCS	TACC2_ES_20_19_22	TACC2	ES	20	19	22	1.3E-08	0.438	0.511	8.7E-01	3.0E-01	2.3E-01
UCS	TACC2_ES_20:21_19_22	TACC2	ES	20:21	19	22	4.3E-12	0.578	0.634	9.8E-01	7.3E-01	4.9E-01
UCS	TAF1D_AA_12.3:12.4_12.1_12.5	TAF1D	AA	12.3:12.4	12.1	12.5	9.0E-04	0.174	-0.167	2.7E-01	7.6E-01	6.0E-01
UCS	TAF1D_AD_12.2:12.3_12.1_12.5	TAF1D	AD	12.2:12.3	12.1	12.5	3.5E-04	0.201	-0.283	8.3E-02	3.8E-01	9.1E-01
UCS	TAF1D_RI_12.2:12.3:12.4_12.1_12.5	TAF1D	RI	12.2:12.3:12.4	12.1	12.5	8.5E-04	0.176	-0.254	6.0E-01	8.5E-01	5.6E-01
UCS	TAZ_ES_5_4_6	TAZ	ES	5	4	6	1.7E-04	0.215	0.001	5.2E-01	5.3E-01	4.7E-01
UCS	TAZ_ES_5:6:7_4_8.1	TAZ	ES	5:06:07	4	8.1	7.0E-04	0.181	0.046	6.3E-01	1.0E-01	9.2E-02

cancer type	id	Gene Symbol	splice_type	Exon	From.Exon	To.Exon	anova.p	adj.r2	r	p.50	p.25	p.10
UCS	TBC1D1_ES_13:14_12_15	TBC1D1	ES	13:14	12	15	2.1E-11	0.581	0.057	6.1E-01	3.6E-01	5.6E-02
UCS	TBC1D3_ES_17_16_18.1	TBC1D3	ES	17	16	18.1	5.7E-04	0.181	0.031	6.0E-01	1.7E-01	7.8E-01
UCS	TBC1D9B_ES_20_19_21	TBC1D9B	ES	20	19	21	2.3E-04	0.206	-0.145	1.3E-01	2.1E-01	8.5E-01
UCS	TBRG4_ES_4:5_3_6	TBRG4	ES	4:05	3	6	8.6E-06	0.292	-0.194	3.0E-01	7.9E-02	2.5E-02
UCS	TCF20_ES_4.1:4.2_3_5	TCF20	ES	4.1:4.2	3	5	8.7E-06	0.292	-0.115	2.6E-02	1.1E-02	1.5E-01
UCS	TCF7_ES_11_10_14.2	TCF7	ES	11	10	14.2	1.0E-05	0.353	-0.411	8.6E-01	1.8E-01	2.4E-01
UCS	TCF7_ES_11:14.1_10_14.2	TCF7	ES	11:14.1	10	14.2	7.3E-04	0.227	-0.338	1.6E-01	2.3E-01	6.2E-02
UCS	TEAD2_ES_6_5_7.1	TEAD2	ES	6	5	7.1	9.4E-04	0.167	0.281	5.7E-01	7.0E-01	7.9E-01
UCS	TEAD2_ES_6:7.1_5_8	TEAD2	ES	06:07.1	5	8	8.3E-05	0.258	0.368	6.7E-01	7.5E-01	2.2E-01
UCS	THNSL2_ES_9_8_11	THNSL2	ES	9	8	11	1.4E-04	0.232	-0.199	8.4E-02	2.1E-01	2.5E-01
UCS	THNSL2_ME_9|10_8_11	THNSL2	ME	9|10	8	11	2.3E-04	0.213	-0.014	4.8E-01	4.9E-01	9.3E-02
UCS	THUMPD3_AD_1.2:1.3_1.1_2	THUMPD3	AD	1.2:1.3	1.1	2	4.8E-04	0.203	-0.038	9.9E-01	6.7E-01	7.6E-01
UCS	TMCC1_AA_4.1_3_4.2	TMCC1	AA	4.1	3	4.2	9.4E-07	0.355	-0.045	7.9E-01	8.6E-01	2.2E-01
UCS	TMEM205_AD_2.2:2.3_2.1_2.6	TMEM205	AD	2.2:2.3	2.1	2.6	1.5E-05	0.283	-0.053	8.9E-01	1.0E-01	1.9E-01
UCS	TMEM205_AD_2.3_2.2_2.6	TMEM205	AD	2.3	2.2	2.6	2.4E-05	0.266	-0.096	2.1E-01	3.4E-01	3.9E-02
UCS	TMEM205_ES_2.2:2.3:2.5_2.1_2.6	TMEM205	ES	2.2:2.3:2.5	2.1	2.6	8.2E-04	0.174	-0.385	1.7E-01	2.4E-01	7.9E-02
UCS	TMEM206_ES_2_1_3	TMEM206	ES	2	1	3	7.6E-07	0.383	-0.046	4.5E-01	4.8E-01	9.1E-01
UCS	TMPO_ES_6:7:8_5.1_9	TMPO	ES	6:07:08	5.1	9	1.7E-05	0.274	0.003	3.3E-01	8.7E-01	5.7E-01
UCS	TMX2_ES_3.1:3.2:3.3:4:5.1:5.3_2_6	TMX2	ES	.1:3.2:3.3:4:5.1:5.	2	6	4.7E-04	0.219	0.053	2.6E-01	5.1E-01	5.6E-01
UCS	TMX2_ES_3.3:4:5.1:5.2:5.3_2_6	TMX2	ES	3.3:4:5.1:5.2:5.3	2	6	7.7E-04	0.185	0.156	4.3E-01	3.3E-01	4.2E-01
UCS	TMX2_ES_4:5.1:5.3_2_6	TMX2	ES	4:5.1:5.3	2	6	9.0E-04	0.177	0.460	9.6E-01	2.4E-01	8.9E-01
UCS	TNC_ES_12:13:14:15:16:19_11_20	TNC	ES	12:13:14:15:16:19	11	20	1.7E-04	0.225	0.116	9.0E-02	7.9E-02	7.2E-01
UCS	TNC_ES_18_16_19	TNC	ES	18	16	19	9.4E-09	0.456	-0.162	2.2E-01	9.7E-01	3.7E-01
UCS	TNK2_ES_13:14:15.2:16:17_12_18	TNK2	ES	13:14:15.2:16:17	12	18	7.0E-04	0.210	0.219	9.8E-01	6.5E-01	4.7E-01
UCS	TNK2_ES_16_15.2_17	TNK2	ES	16	15.2	17	2.0E-08	0.440	0.536	2.1E-01	8.1E-01	9.5E-01
UCS	TPD52L2_ES_4_3_5	TPD52L2	ES	4	3	5	9.3E-05	0.231	-0.137	6.3E-01	5.9E-01	2.3E-01
UCS	TPM1_AD_12.2_12.1_13.1	TPM1	AD	12.2	12.1	13.1	2.6E-08	0.423	-0.375	5.6E-01	9.7E-01	4.0E-01
UCS	TPM1_ES_3.2:5.2:6:7:8:10:11.1:12.1:12.	TPM1	ES	7:8:10:11.1:12.1:	3.1	13.2	4.7E-10	0.500	-0.630	3.9E-01	3.9E-01	4.3E-01
UCS	TPM2_AA_11.1_9_11.2	TPM2	AA	11.1	9	11.2	6.8E-06	0.298	0.398	1.2E-01	1.5E-02	1.5E-01
UCS	TPM2_ES_6_5_7	TPM2	ES	6	5	7	4.4E-04	0.188	-0.576	6.6E-01	8.3E-01	8.3E-01
UCS	TPM2_ME_6|7_5_8	TPM2	ME	6|7	5	8	1.5E-10	0.520	-0.638	4.9E-01	8.6E-01	3.8E-01
UCS	TRAPPC9_ES_6_5_7	TRAPPC9	ES	6	5	7	2.6E-04	0.257	-0.160	6.2E-01	2.7E-01	5.5E-01
UCS	TTC7B_ES_18_17_19	TTC7B	ES	18	17	19	8.8E-05	0.240	0.305	7.5E-01	1.4E-01	3.9E-01
UCS	UBE2G2_ES_7_6_8	UBE2G2	ES	7	6	8	4.4E-04	0.191	0.017	6.9E-01	8.9E-01	1.3E-01
UCS	UBE2V1_ES_3:4_2_5	UBE2V1	ES	3:04	2	5	8.3E-05	0.241	0.036	2.0E-01	3.2E-01	1.5E-01
UCS	UBE4B_ES_8_7_9	UBE4B	ES	8	7	9	5.2E-08	0.507	-0.044	2.3E-01	2.0E-01	2.1E-01
UCS	UCKL1_ES_9_8_11.2	UCKL1	ES	9	8	11.2	4.9E-05	0.248	-0.122	8.8E-01	8.9E-01	2.7E-01
UCS	UPF3B_ES_8_7_9	UPF3B	ES	8	7	9	5.7E-05	0.247	-0.147	4.5E-01	1.6E-01	9.4E-02
UCS	USO1_ES_14_13_15	USO1	ES	14	13	15	2.5E-05	0.293	0.234	3.8E-01	9.7E-01	1.4E-01
UCS	USP28_ES_21_20_22	USP28	ES	21	20	22	4.8E-07	0.421	0.554	3.6E-01	6.5E-01	2.8E-01

cancer type	id	Gene Symbol	splice_type	Exon	From.Exon	To.Exon	anova.p	adj.r2	r	p.50	p.25	p.10
UCS	USP5_AD_15.2_15.1_16	USP5	AD	15.2	15.1	16	2.7E-10	0.510	0.061	1.4E-01	1.6E-01	1.4E-01
UCS	VCL_ES_19_18.2_20	VCL	ES	19	18.2	20	1.7E-04	0.226	-0.001	7.1E-01	5.4E-01	8.3E-01
UCS	VDAC3_ES_5.2_4.1_6	VDAC3	ES	5.2	4.1	6	2.5E-11	0.550	0.338	9.4E-01	9.1E-01	8.3E-01
UCS	VEGFA_ES_8.1:8.2_6_9.1	VEGFA	ES	8.1:8.2	6	9.1	7.9E-06	0.294	0.016	5.7E-02	7.8E-01	1.8E-01
UCS	VIPAS39_ES_1.3:1.4_1.1_2	VIPAS39	ES	1.3:1.4	1.1	2	2.1E-04	0.264	0.268	1.3E-01	2.8E-02	2.6E-02
UCS	VPS29_ES_3.1_1_5	VPS29	ES	3.1	1	5	6.2E-04	0.178	-0.100	9.6E-01	9.8E-01	9.2E-01
UCS	VPS29_ES_3.1:3.2_1_5	VPS29	ES	3.1:3.2	1	5	6.0E-04	0.180	-0.247	9.7E-01	3.4E-01	7.5E-01
UCS	VRK2_ES_18_17_19	VRK2	ES	18	17	19	4.9E-05	0.248	-0.006	8.8E-01	5.8E-01	2.4E-01
UCS	WDR13_RI_1.2_1.1_1.3	WDR13	RI	1.2	1.1	1.3	2.2E-04	0.207	-0.062	7.5E-01	3.4E-01	4.3E-01
UCS	WIZ_ES_9_8_10	WIZ	ES	9	8	10	1.6E-04	0.215	-0.046	2.1E-01	5.3E-01	3.2E-01
UCS	ZCCHC9_RI_1.4_1.3_1.5	ZCCHC9	RI	1.4	1.3	1.5	5.7E-04	0.181	-0.320	1.1E-01	3.1E-02	7.5E-01
UCS	ZDHHC16_ES_9_8.2_10	ZDHHC16	ES	9	8.2	10	4.5E-07	0.362	-0.241	8.7E-01	3.3E-01	5.5E-02
UCS	ZFYVE21_ES_6.1_5_7	ZFYVE21	ES	6.1	5	7	1.1E-07	0.393	-0.281	2.7E-01	9.5E-01	2.0E-01
UCS	ZNF530_ES_4.1_3_5	ZNF530	ES	4.1	3	5	2.1E-05	0.274	-0.183	2.9E-01	9.9E-01	6.6E-01
UCS	ZNHIT3_ES_2_1_3.1	ZNHIT3	ES	2	1	3.1	2.7E-05	0.263	-0.142	5.3E-01	1.5E-01	7.8E-02
